# Cerebral cortical structural alteration patterns across four major psychiatric disorders in 5549 individuals

**DOI:** 10.1038/s41380-023-02224-7

**Published:** 2023-08-18

**Authors:** Junya Matsumoto, Masaki Fukunaga, Kenichiro Miura, Kiyotaka Nemoto, Naohiro Okada, Naoki Hashimoto, Kentaro Morita, Daisuke Koshiyama, Kazutaka Ohi, Tsutomu Takahashi, Michihiko Koeda, Hidenaga Yamamori, Michiko Fujimoto, Yuka Yasuda, Satsuki Ito, Ryuichi Yamazaki, Naomi Hasegawa, Hisashi Narita, Satoshi Yokoyama, Ryo Mishima, Jun Miyata, Yuko Kobayashi, Daiki Sasabayashi, Kenichiro Harada, Maeri Yamamoto, Yoji Hirano, Takashi Itahashi, Masahito Nakataki, Ryu-ichiro Hashimoto, Khin K. Tha, Shinsuke Koike, Toshio Matsubara, Go Okada, Reiji Yoshimura, Osamu Abe, Theo G. M. van Erp, Jessica A. Turner, Neda Jahanshad, Paul M. Thompson, Toshiaki Onitsuka, Yoshiyuki Watanabe, Koji Matsuo, Hidenori Yamasue, Yasumasa Okamoto, Michio Suzuki, Norio Ozaki, Kiyoto Kasai, Ryota Hashimoto

**Affiliations:** 1 Department of Pathology of Mental Diseases, National Institute of Mental Health, National Center of Neurology and Psychiatry, Kodaira, 187-8553 Japan; 2 Section of Brain Function Information, National Institute for Physiological Sciences, Okazaki, 444-8585 Japan; 3 Department of Psychiatry, Institute of Medicine, University of Tsukuba, Tsukuba, 305-8575 Japan; 4 Department of Neuropsychiatry, Graduate School of Medicine, The University of Tokyo, Tokyo, 113-8655 Japan; 5 The International Research Center for Neurointelligence (WPI-IRCN), The University of Tokyo Institutes for Advanced Study (UTIAS), Tokyo, 113-0033 Japan; 6 Department of Psychiatry, Hokkaido University Graduate School of Medicine, Sapporo, 060‐8638 Japan; 7 Department of Rehabilitation, University of Tokyo Hospital, Tokyo, 113-8655 Japan; 8 Department of Psychiatry, Gifu University Graduate School of Medicine, Gifu, 501-1194 Japan; 9 Department of General Internal Medicine, Kanazawa Medical University, Ishikawa, 920-0293 Japan; 10 Department of Neuropsychiatry, University of Toyama Graduate School of Medicine and Pharmaceutical Sciences, Toyama, 930-0194 Japan; 11 Research Center for Idling Brain Science, University of Toyama, Toyama, 930-0194 Japan; 12 Department of Neuropsychiatry, Graduate School of Medicine, Nippon Medical School, Tokyo, 113-8602 Japan; 13 Department of Psychiatry, Osaka University Graduate School of Medicine, Suita, 565-0871 Japan; 14 Japan Community Health Care Organization Osaka Hospital, Osaka, 553-0003 Japan; 15 Life Grow Brilliant Mental Clinic, Medical Corporation Foster, Osaka, 530-0013 Japan; 16 Department of Developmental and Clinical Psychology, The Division of Human Developmental Sciences, Graduate School of Humanity and Sciences, Ochanomizu University, Tokyo, 112-8610 Japan; 17 Department of Psychiatry, The Jikei University School of Medicine, Tokyo, 105-8461 Japan; 18 Department of Psychiatry and Neurosciences, Hiroshima University, Hiroshima, 734-8551 Japan; 19 Department of Psychiatry, Graduate School of Medicine, Kyoto University, Kyoto, 606-8507 Japan; 20 Division of Neuropsychiatry, Department of Neuroscience, Yamaguchi University Graduate School of Medicine, Ube, 755-8505 Japan; 21 Department of Psychiatry, Nagoya University Graduate School of Medicine, Nagoya, 466-8550 Japan; 22 Department of Psychiatry, Division of Clinical Neuroscience, Faculty of Medicine, University of Miyazaki, Miyazaki, 889-1692 Japan; 23 Department of Neuropsychiatry, Graduate School of Medical Sciences, Kyushu University, Fukuoka, 812-8582 Japan; 24 Institute of Industrial Science, The University of Tokyo, Tokyo, 153-8505 Japan; 25 Medical Institute of Developmental Disabilities Research, Showa University, Tokyo, 157-8577 Japan; 26 Department of Psychiatry, Tokushima University Hospital, Tokushima, 770-8503 Japan; 27 Department of Language Sciences, Graduate School of Humanities, Tokyo Metropolitan University, Hachioji, 192-0397 Japan; 28 Global Center for Biomedical Science and Engineering, Hokkaido University Faculty of Medicine, Sapporo, 060-8638 Japan; 29 University of Tokyo Institute for Diversity & Adaptation of Human Mind (UTIDAHM), Tokyo, 153-8902 Japan; 30 Center for Evolutionary Cognitive Sciences, Graduate School of Arts and Sciences, The University of Tokyo, Tokyo, 153-8902 Japan; 31 Department of Psychiatry, University of Occupational and Environmental Health, Kitakyushu, 807-8555 Japan; 32 Department of Radiology, Graduate School of Medicine, The University of Tokyo, Tokyo, 113-8655 Japan; 33 Clinical Translatational Neuroscience Laboratory, Department of Psychiatry and Human Behavior, University of California Irvine, Irvine, CA 92697 USA; 34 Center for the Neurobiology of Learning and Memory, University of California Irvine, Irvine, CA 92697 USA; 35 Department of Psychiatry and Behavioral Health, Wexner Medical Center, the Ohio State University, Columbus, OH 43210 USA; 36 Imaging Genetics Center, Mark & Mary Stevens Neuroimaging & Informatics Institute, Keck School of Medicine, University of Southern California, Los Angeles, CA 90292 USA; 37 National Hospital Organization Sakakibara Hospital, Tsu, 514-1292 Japan; 38 Department of Radiology, Shiga University of Medical Science, Otsu, 520-2192 Japan; 39 Department of Psychiatry, Faculty of Medicine, Saitama Medical University, Saitama, 350-0495 Japan; 40 Department of Psychiatry, Hamamatsu University School of Medicine, Hamamatsu, 431-3192 Japan; 41 Pathophysiology of Mental Disorders, Nagoya University Graduate School of Medicine, Nagoya, 466-8550 Japan

**Keywords:** Schizophrenia, Bipolar disorder, Depression, Autism spectrum disorders

## Abstract

According to the operational diagnostic criteria, psychiatric disorders such as schizophrenia (SZ), bipolar disorder (BD), major depressive disorder (MDD), and autism spectrum disorder (ASD) are classified based on symptoms. While its cluster of symptoms defines each of these psychiatric disorders, there is also an overlap in symptoms between the disorders. We hypothesized that there are also similarities and differences in cortical structural neuroimaging features among these psychiatric disorders. T1-weighted magnetic resonance imaging scans were performed for 5,549 subjects recruited from 14 sites. Effect sizes were determined using a linear regression model within each protocol, and these effect sizes were meta-analyzed. The similarity of the differences in cortical thickness and surface area of each disorder group was calculated using cosine similarity, which was calculated from the effect sizes of each cortical regions. The thinnest cortex was found in SZ, followed by BD and MDD. The cosine similarity values between disorders were 0.943 for SZ and BD, 0.959 for SZ and MDD, and 0.943 for BD and MDD, which indicated that a common pattern of cortical thickness alterations was found among SZ, BD, and MDD. Additionally, a generally smaller cortical surface area was found in SZ and MDD than in BD, and the effect was larger in SZ. The cosine similarity values between disorders were 0.945 for SZ and MDD, 0.867 for SZ and ASD, and 0.811 for MDD and ASD, which indicated a common pattern of cortical surface area alterations among SZ, MDD, and ASD. Patterns of alterations in cortical thickness and surface area were revealed in the four major psychiatric disorders. To our knowledge, this is the first report of a cross-disorder analysis conducted on four major psychiatric disorders. Cross-disorder brain imaging research can help to advance our understanding of the pathogenesis of psychiatric disorders and common symptoms.

## Introduction

According to the operational diagnostic criteria set out in the Diagnostic and Statistical Manual of Mental Disorders and the International Classification of Diseases, psychiatric disorders are classified based on clusters of symptoms. Specific psychiatric disorders have distinct, characteristic symptoms that define each disorder, although they also have some symptoms in common. It is therefore be expected that biological similarities and differences can be found among psychiatric disorders, and identifying these profiles could support the development of biologically based treatment methods. It is also expected that psychiatric disorders show similarities and differences in their structural brain imaging characteristics. Many efforts are under way by researchers worldwide to elucidate relevant pathophysiological features using brain imaging [1, 2]; such features include cortical and subcortical structures and white matter microstructure [2–5]. Specifically, it is important to investigate whether there are similarities and differences in gray matter structure across psychiatric disorders.

Brain-wide studies, which use an exploratory approach without requiring a specific hypothesis, may require thousands of samples to define robust effects; as such, brain-wide studies with small samples may lack statistical power and have poor reproducibility due to MRI scanner differences and differences in analysis methods, as well as the possibly small effects of the disorder relative to the normal range of brain variation [6]. To address this issue, the Enhancing Neuroimaging Genetics through Meta-Analysis (ENIGMA) consortium has been adopting an approach that unifies protocols and uses the same processing pipelines, quality control (QC), and statistical methods from study sites around the world to perform meta-analysis of large, multicenter samples and thereby validate the brain structural and functional profiles of disorder effects in thousands of cases [2, 7–11]. The results have thus far revealed disorder-related structural abnormalities in the cerebral cortex in major psychiatric disorders. In groups of individuals with SZ, significantly thinner cortex and smaller regional cortical surface areas have been demonstrated compared to healthy comparison subjects (HC) [7]. In BD, a significantly thinner cortex was shown, but with a more spatially restrictive pattern than SZ and generally weaker effects [8]. In MDD, a thinner cortex was reported in several regions [9]. In ASD, cortical thickness abnormalities were detected, including thickening in frontal lobe regions and thinning in temporal lobe regions [10]. The findings from these groups have been compared through several efforts, such as Boedhoe et al. [12]. and Cheon et al. [3], highlighting the similarities in effects across various disorders. Recent cross-disorder comparisons have compared ASD with attention deficit hyperactivity disorder and obsessive-compulsive disorder in an analysis of 151 worldwide cohorts [12], but thus far, there has been no such cross-disorder analysis of SZ, BD, MDD, and ASD.

A previous cross-disorder diffusion tensor imaging (DTI) study of SZ, BD, MDD, and ASD revealed white matter microstructural abnormalities common to SZ, BD, and ASD, with the greatest degree of impairment observed in SZ [4]. It is important to investigate whether the similar cross-disorder pattern of alterations seen in white matter microstructure has an equivalent in gray matter structures. Is there a pattern of cortical thickness and surface area in gray matter, similar to white matter microstructural abnormalities, where BD, ASD, and SZ show a pattern of thinner cortical thickness or small cortical surface area, with the greatest abnormalities appearing in SZ?

In the current study, we hypothesized that similarities and differences in cortical structure among SZ, BD, MDD, and ASD could be investigated by structural neuroimaging. To investigate our hypothesis, we conducted a cross-disorder analysis of cortical structure, including regional cortical thickness and surface area in four major psychiatric disorders, SZ, BD, MDD, and ASD, in a large-scale coordinated multicenter analysis by the Cognitive Genetics Collaborative Research Organization (COCORO), using a meta-analysis as a method for harmonizing the protocols despite their differences. We quantified the similarities and differences in the patterns of cortical thickness and surface area across disorders.

## Materials and methods

### Subjects

A total of 5549 subjects from 14 COCORO sites participated in the study: 3068 HC, 1426 individuals with SZ, 237 individuals with BD, 612 individuals with MDD, and 206 individuals with ASD (Table 1, and Supplementary Table S1). Details of the inclusion and exclusion criteria at each site are described in Supplementary Methods 1. Age at onset, duration of illness (DoI), information on antipsychotics, and severity in individuals with SZ (Supplementary Table S2); DoI, diagnosis type, and psychotropic medication in individuals with BD (Supplementary Table S3); and DoI, age at onset, number of depressive episodes, psychotropic medications, and severity for individuals with MDD (Supplementary Table S4) are summarized in Supplementary Tables. Some of these subjects also participated in prior neuroimaging studies [4, 11, 13–22]. This study was approved by the institutional review board of Osaka University (approval number: 706-12), the National Center of Neurology and Psychiatry (approval number: A2018-09), and each local institutional review board. Written informed consent was obtained from each subject before participation.

**Table 1:** Number of individuals per diagnosis by institute and scanner.

Protocol name	Total	HC	SZ	BD	MDD	ASD
Total	5549	3068	1426	237	612	206
Osaka1	607	410	142		15	40
Osaka2	336	236	74		9	17
Osaka3	687	549	91		19	28
Tokyo1	318	225	93			
Tokyo2	151	83	41			27
Tokyo3	64	39	15			10
Tokyo4	55	45	10			
Tokyo5	142	43	27	23	43	6
NipponMedical2	408	193	215			
Hiroshima1	126	57			69	
Hiroshima2^a^	42				42	
Hiroshima3	78	29			49	
Hiroshima4	42	34			8	
Hiroshima5	137	52		26	59	
Kyoto1	183	111	72			
Kyoto2	172	127	45			
Toyama1	221	117	104			
Toyama2	127	56	71			
Kanazawa1	288	109	103	34	42	
Nagoya1	203	118	54	19		12
Nagoya3	30	13	17			
Hokkaido1	386	31	108	78	169	
Hokkaido2	58	25	33			
Kyushu1	147	78	41	18	10	
Kyushu2	67	27	31	9		
Kyushu3^b^	7			7		
Yamaguchi1	114	67	21	8	18	
Yamaguchi2	182	112		15	55	
Showa1	132	66				66
Tokushima2	17	5	12			
UOEH1	22	11	6		5	

*ASD* autism spectrum disorder, *BD* bipolar disorder, *HC* healthy comparison subjects, *MDD* major depressive disorder, *SZ* schizophrenia.

^a^Hiroshima2 is not used in the case‒control analysis because of the absence of healthy comparison subjects and is used only for analysis of associations with severity, the number of episodes, and duration of illness in up to 42 subjects. Forty-two individuals were included in analyses related to the Beck Depression Inventory, 28 individuals were included in analyses related to the Hamilton Depression Rating Scale, 25 individuals were included in analyses related to the number of episodes in recurrent individuals, and 41 individuals were included in analyses related to the duration of illness.

^b^Kyushu3 is not used in the case‒control analysis because of the absence of healthy comparison subjects and is used only for analysis of associations with duration of illness.

### Image processing

Structural T1-weighted magnetic resonance imaging scans of the brain were performed at each site. Details of the scanner and imaging parameters for each site are described in Supplementary Methods 2. Visual QC of the T1-weighted images was also performed. Poor-quality images with incomplete brain coverage, a low signal-to-noise ratio, or significant visible artifacts such as ringing or blurring throughout the entire brain were excluded. Images with significant organic abnormalities were excluded. The cortical thickness was calculated for 68 Desikan-Killiany (DK) atlas regions [23] (34 left and 34 right), the overall left and right hemispheres, and the global mean cortical thickness using FreeSurfer [24] version 5.3. The cortical surface area was calculated for 68 DK atlas regions, the overall left and right hemispheres, and the total cortical surface area. The cortical volume was calculated as the product of the cortical thickness and surface area of each area. QC of the segmentations and parcellations by FreeSurfer was performed by visual inspection. Cases that failed FreeSurfer processing or had poor parcellations were excluded. The subjects were divided into groups by site, scanner, and protocol. On site with multiple protocols, a separate group was defined for each scanner. Those groups with fewer than five cases in each diagnostic group were excluded. No groups with different imaging parameters on the same scanner at the same site remained. Five thousand and five hundred forty-nine subjects were used from the original dataset of 6,772 subjects; 748 subjects were excluded by QC of raw images due to significant motion or other incidental findings, 273 subjects were excluded by QC at the FreeSurfer stage for reasons such as poor segmentation, and 202 subjects were excluded because of the sample size of each protocol. Details of the numbers of each disorder are described in Supplementary Methods 3.

### Statistical analysis

R version 4.2.1 (R Foundation for Statistical Computing, Vienna, Austria) was used for statistical analysis. Effect size (unbiased *d*) [25] for differences in cortical thickness, surface area, and volume between HC and each disorder group (SZ, BD, MDD, and ASD groups) were determined using a linear regression model, within the same scanner and protocols at each site group. This was done for 71 regions, adjusting for age and sex as covariates, and referred to as Analysis A. In Analysis B, global mean cortical thickness, total cortical surface area, or intracranial volume (ICV) were added as additional covariates to calculate effect size for each of the DK atlas regions to evaluate regional specificity. Other analyses included the following: effect size was obtained for the interaction between sex and diagnosis and between age and diagnosis using a linear regression model with age and sex as covariates within the same group of scanners and protocols at each site. For between-group comparisons of cortical thickness and surface area stratified by clinical variables such as sub-diagnosis, age, and psychotropic drugs, effect size was calculated using a linear regression model with age and sex as covariates within the same group of scanners and protocols at each site. If additional or different covariates were used, they are indicated in the title of the supplementary tables for each analysis. In the analysis of the association between clinical measures and cortical thickness and surface area, partial *R* was obtained by partial correlation analysis using age and sex as covariates within the same group of scanners and protocols at each site. In the analysis of the association between age and cortical thickness and surface area in ASD, partial *R* was obtained with sex as a covariate. For protocols for which intelligence quotient (IQ) [26] data were available in ASD, we added a supplemental analysis in which IQ was added as a covariate to account for the influence of IQ in ASD in the analyses of differences in cortical thickness, surface area, and volume between HC and ASD. A meta-analysis was performed on effect size for group comparison and interaction analysis, and partial *R* was computed for the partial correlation analysis (metafor package; version 3.4-0). The significance level was set at a false discovery rate (FDR) *q* value < 0.05. Analysis A and the other analyses consisted of 71 multiple comparisons in 71 regions: 68 DK atlas regions, the left and right hemispheres, and the global or total brain (global mean thickness, total cortical surface area, or total cortical volume). Analysis B consisted of 68 multiple comparisons in the 68 DK atlas regions. The Benjamini‒Hochberg FDR procedure was used to perform 71 multiple-comparison corrections for Analysis A and other analyses and 68 multiple-comparison corrections for Analysis B. Power analysis was described in Supplementary Methods 4.

### Similarity of disorders

We analyzed the similarity of the differences in regional cortical thickness and surface area of each disorder group from the HC using the effect size of each of the 68 regions calculated in the between-group comparison in Analysis A for cortical thickness and surface area. The 68 effect size values for each of the 68 regions for each disorder were made into a 68-dimensional vector (***a*** for disease a and ***b*** for disease b), and the inner product of the two disorder vectors (***a*** ∙ ***b***) was adjusted from −1 to 1 for the cosine similarity $( {\cos \theta = \frac{{{{{{{{{\boldsymbol{a}}}}}}}} \cdot {{{{{{{\boldsymbol{b}}}}}}}}}}{{\left| {{{{{{{\boldsymbol{a}}}}}}}} \right|\left| {{{{{{{\boldsymbol{b}}}}}}}} \right|}}} )$ for both cortical thickness and surface area. If the cosine similarity was close to 1, the patterns of cortical thickness and surface area were considered similar. If the cosine similarity was close to −1, the patterns were judged to be different. A similar measure has been used in a prior study [27].

## Results

### Cross-disorder comparison of overall hemispheric cortical thickness, surface area, and volume

**Fig. 1:**
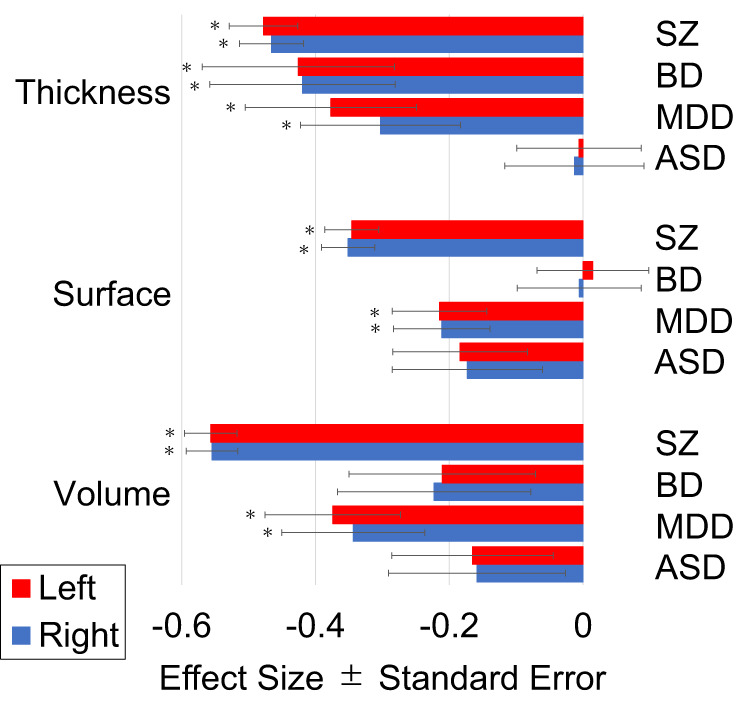
Effect sizes for global mean cortical thickness, total cortical surface area, and total cortical volume in a group comparison (Analysis A) between healthy comparison subjects and schizophrenia, bipolar disorder, major depressive disorder, and autism spectrum disorder are shown. Error bars are standard errors. The red bar indicates the left hemisphere, and the blue bar indicates the right hemisphere. FDR *q* values less than 0.05 are marked with an asterisk. ASD autism spectrum disorder, BD bipolar disorder, MDD major depressive disorder, Surface total cortical surface area, SZ schizophrenia, Thickness global mean cortical thickness, Volume total cortical volume.

The results of the meta-analysis of effect size for the group comparisons between HC and groups of individuals with SZ, BD, MDD, and ASD in Analysis A are shown in Fig. 1. The cortical thickness was significantly lower in the group of individuals with SZ (left, *d* = −0.478; right, *d* = −0.466), BD (left, *d* = −0.426; right, *d* = −0.420) and MDD (left, *d* = −0.377; right, *d* = −0.303). The cortical surface area and volume were significantly smaller in individuals with SZ (surface area of left hemisphere, *d* = −0.346; surface area of right hemisphere, *d* = −0.351; volume of left hemisphere, *d* = −0.557; volume of right hemisphere, *d* = −0.555) and MDD (surface area of left hemisphere, *d* = −0.215; surface area of right hemisphere, *d* = −0.211; volume of left hemisphere, *d* = −0.374; volume of right hemisphere, *d* = −0.344).

### Regional comparison of cortical thickness

**Fig. 2:**
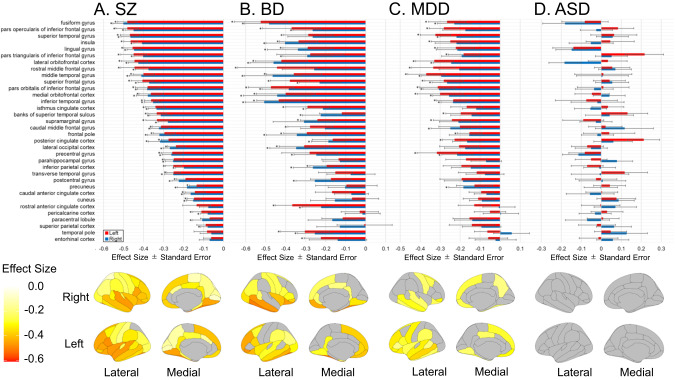
Effect sizes for cortical thickness are shown for each region of interest in the comparison between healthy comparison subjects and individuals with each disorder group (Analysis A). Error bars indicate the standard error. The red bar indicates the left hemisphere, and the blue bar indicates the right hemisphere; **A** schizophrenia; **B** bipolar disorder; **C** major depressive disorder; and **D** autism spectrum disorder. FDR *q*-values less than 0.05 are marked with an asterisk. ASD autism spectrum disorder, BD bipolar disorder, HC healthy comparison subjects, MDD major depressive disorder, SZ schizophrenia.

In Analysis A (Fig. 2), the group of individuals with SZ showed a thinner cortex in all regions relative to HC, with 60 regions significant, and the region with the largest effect size was the right fusiform gyrus with *d* = −0.500 (Supplementary Table S5). The group of individuals with BD showed a thinner cortex in all regions relative to HC, significant in 42 regions, with the largest effect size in the left fusiform gyrus, *d* = −0.524, with similar results for the subset of individuals aged 25 years and older (Supplementary Tables S6 and S7). The group of individuals with MDD showed thinner gray matter in 67 regions relative to HC—significant in 29 regions—with the largest effect size in the left middle temporal gyrus at *d* = −0.369, and similar results for the subset of individuals over age 21 (Supplementary Tables S8 and S9). There were no detectable differences in each region in the group of individuals with ASD (Supplementary Table S10).

In Analysis B, 27 regions were significantly thinner than average, and the largest effect size in the negative direction was observed in the left lingual gyrus in SZ (*d* = −0.250, Supplementary Table S11). In BD, four regions had a significantly thinner cortex, and the largest effect size was in the right inferior temporal gyrus with *d* = −0.307. In individuals aged 25 years and older, two regions were significantly thinner (the larger effect size was in the left *pars orbitalis* of the inferior frontal gyrus, *d* = −0.306; Supplementary Tables S12, S13). There were no differences between the HC group and the group of individuals with MDD (at all ages or over 21 years old) or individuals with ASD (Supplementary Tables S14–S16).

### Regional comparison of cortical surface area

**Fig. 3:**
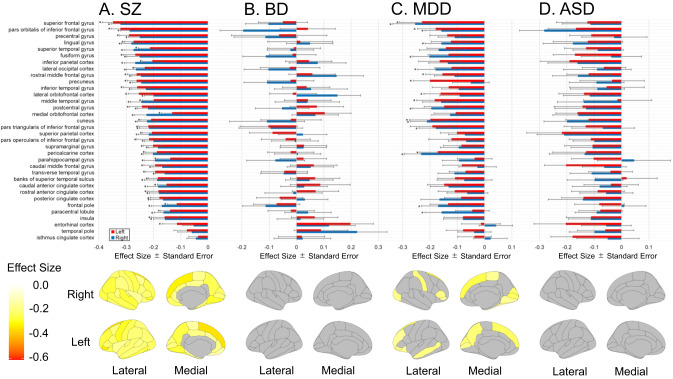
Effect sizes for the cortical surface area are shown for each region of interest in the comparison between healthy comparison subjects and individuals with each disorder group (Analysis A). Error bars indicate the standard error. The red bar indicates the left hemisphere, and the blue bar indicates the right hemisphere; **A** schizophrenia; **B** bipolar disorder; **C** major depressive disorder; and **D** autism spectrum disorder. FDR *q* values less than 0.05 are marked with an asterisk. ASD autism spectrum disorder, BD bipolar disorder, HC healthy comparison subjects, MDD major depressive disorder, SZ schizophrenia.

In Analysis A (Fig. 3), all areas were smaller in individuals with SZ: 63 areas were significant, and the largest effect size was found in the left superior frontal gyrus (*d* = −0.347, Supplementary Table S17). There was no detectable difference in groups of individuals with BD of all ages or over 25 (Supplementary Tables S18 and S19). In groups of individuals with MDD, 66 areas were smaller, both for all ages and in the analysis of those above 21 years of age, with 21 significant areas for all ages. For all ages, the largest effect size was found in the right superior frontal gyrus (*d* = −0.254, Supplementary Table S20), and 2 significant areas were found above 21 years of age, with the largest effect size in the right pericalcarine cortex (*d* = −0.227, Supplementary Table S21). There was no detectable difference in each region in the group of individuals with ASD (Supplementary Table S22).

In Analysis B, the group of individuals with SZ showed disproportionately smaller areas in two regions, with the largest effect size being in the left *pars orbitalis* of the inferior frontal gyrus with *d* = −0.134 (Supplementary Table S23). There were no detectable differences for groups of individuals with BD (all ages or older than 25), MDD (all ages or above 21 years of age), or ASD (Supplementary Tables S24–S28).

The cortical surface area analysis of groups of individuals with BD aged 25 years and older was also conducted with a combination of covariates in line with the ENIGMA study [8] and showed no group differences (Supplementary Tables S29 and S30).

### Regional comparison of cortical volume

In Analysis A, in the group of individuals with SZ, all regions showed smaller cortical volume, 66 regions were significant, and the largest effect size was *d* = −0.544 in the left superior frontal gyrus (Supplementary Table S31). In the groups of individuals with BD, there were significantly smaller cortical volumes in two regions, and the largest effect size was in the right *pars orbitalis* of the inferior frontal gyrus, with *d* = −0.382. In those aged 25 years and older, there were significantly smaller cortical volumes in three regions (with the largest effect sizes in the right *pars orbitalis* of the inferior frontal gyrus, *d* = −0.417; Supplementary Tables S32 and S33). In the groups of individuals with MDD, cortical volume was smaller in 66 regions and significant in 50 regions, with a maximum effect size of −0.381 in the right superior frontal gyrus, which was similar above age 21 (Supplementary Tables S34 and S35). In groups of individuals with ASD, there was one region with a significantly smaller area (the right *pars orbitalis* of the inferior frontal gyrus, *d* = −0.276; Supplementary Table S36).

In Analysis B, ICV was also included as a covariate. In the groups of individuals with SZ, 65 regions showed significantly smaller cortical volume (maximum effect size was left superior frontal gyrus, *d* = −0.575; Supplementary Table S37). In the group with BD, there were significantly smaller cortical volumes for two regions (the largest effect size was in the right *pars orbitalis* of the inferior frontal gyrus, *d* = −0.403) and—in those aged 25 years and older—in three regions with significantly smaller cortical volume (the largest effect size was in the right *pars orbitalis* of the inferior frontal gyrus, *d* = −0.445; Supplementary Tables S38 and S39). The group with MDD showed significantly smaller cortical volume in 34 regions, with a maximum effect size in the right superior frontal gyrus (*d* = −0.378), and above 21 years of age showed significantly smaller cortical volume in 20 regions, with a maximum effect size of *d* = −0.341 in the right superior frontal gyrus (Supplementary Tables S40 and S41). In the group of individuals with ASD, there were no detectable differences in each region (Supplementary Table S42).

### Similarity of patterns

**Fig. 4:**
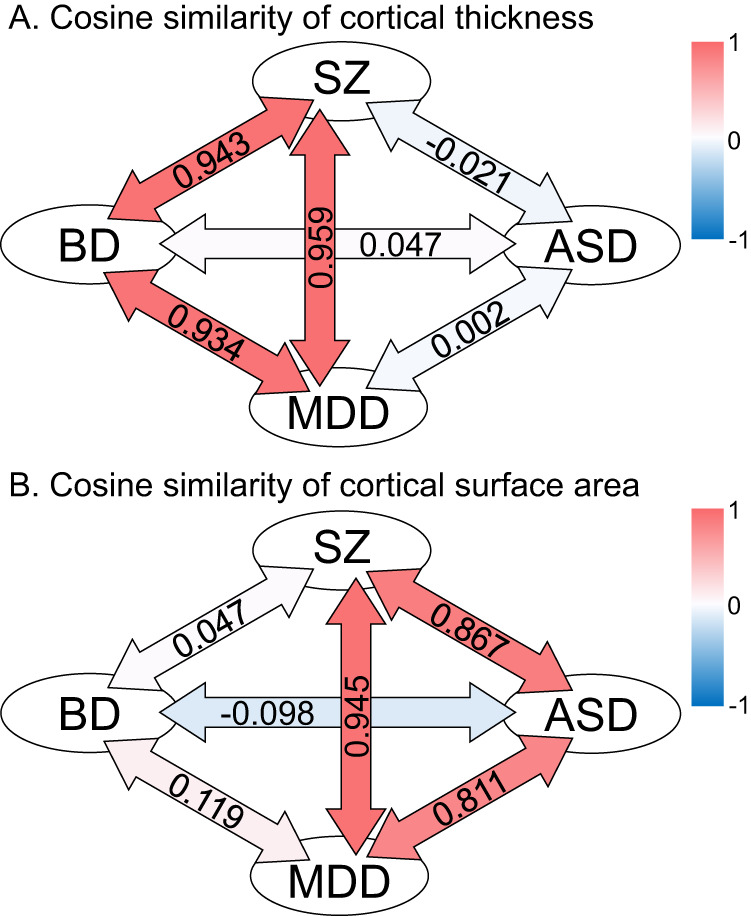
The similarity of the pattern of cortical thickness thinning and the similarity of the pattern of smaller cortical surface area, using effect sizes of 68 areas calculated by group comparison between healthy comparison subjects and each disorder group, is shown using cosine similarity. Cosine similarity takes values from −1 to 1, with values closer to 1 indicating similarity, values close to 0 indicating no similarity, and values near −1 indicating an opposite pattern. Red arrows indicate more similar combinations, and blue arrows indicate the opposite combinations. Cosine similarity values of cortical thickness patterns (**A**) and cortical surface area patterns (**B**) are noted in each arrow. ASD autism spectrum disorder; BD bipolar disorder, MDD major depressive disorder; Surface total cortical surface area, SZ schizophrenia, Thickness global mean cortical thickness.

For 68 cortical regions, we analyzed the similarity of the patterns of cortical thickness and surface area using effect sizes for group comparisons between HC and individuals with SZ, BD, MDD, and ASD using cosine similarity (Fig. 4). Cosine similarity takes values from −1 to 1, with values closer to 1 indicating greater similarity. The groups of individuals with SZ and MDD were similar in both the pattern of cortical thickness (0.959) and the pattern of cortical surface area (0.945). The similarity of the pattern of cortical thickness in the group of individuals with BD was 0.943 with SZ and 0.934 with MDD, indicating that the pattern of cortical thickness was also similar between the group of individuals with SZ and BD and between the group of individuals with BD and MDD. The similarity of the pattern of cortical surface area in individuals with ASD was 0.867 with SZ and 0.811 with MDD, indicating that the pattern of cortical surface area was also similar between the group of individuals with ASD and SZ and the group of individuals with ASD and MDD. The other combinations, namely, cortical thickness in the ASD group versus the other three disorder groups and cortical surface area in the group of individuals with BD versus the other three disorders, all had cosine similarity values less than 0.2, indicating that the patterns were not similar.

### Relationship with clinical variables

There was no interaction between diagnosis and age or diagnosis and sex for either cortical thickness or surface area when comparing any disease group to HC (Supplementary Tables S43–S68). There was no association of cortical thickness and surface area in the left and right hemispheres with age at onset in individuals with SZ and MDD, DoI in individuals with SZ, BD, and MDD, or age in individuals with ASD, except for a negative correlation of cortical thickness in the left and right hemispheres with age for individuals with ASD (Supplementary Tables S69–S89). There were no detectable differences between groups of individuals with BD types I and II, between the group of individuals with recurrent-episode MDD and the HC group, or between the groups of individuals with first-episode and recurrent-episode MDD for bilateral hemisphere cortical thickness and surface area. There were also no significant correlations between the number of recurrent episodes in the individuals with MDD and bilateral hemisphere cortical thickness and surface area, except for thinning in the group comparison between the group of individuals with first-episode MDD and the HC group for the left and right hemisphere overall cortical thickness (Supplementary Tables S90–S110). With respect to antipsychotics in SZ, the group of individuals with SZ on second-generation antipsychotic medications showed bilateral thinning relative to the HC group. The group of individuals with SZ on first-generation antipsychotic medications showed left hemispheric thinning relative to the HC group; the group of individuals with SZ on both first-generation and second-generation antipsychotic medications showed bilateral thinning relative to the HC group and the group of individuals with SZ on second-generation antipsychotic medications, and they also showed right hemispheric thinning relative to the group of individuals with SZ who were not medicated with antipsychotics. The partial correlations of the chlorpromazine equivalents and cortical thickness in the left and right hemispheres were significant (Supplementary Tables S111–S121). The cortical thickness related to medications in the left and right hemispheres generally did not differ significantly in individuals with BD and individuals with MDD, except for thinner cortex in the individuals with BD with valproate use versus without valproate use in the bilateral hemispheres, individuals with MDD without antidepressant use in the left hemisphere versus HC, and individuals with MDD without antipsychotic use versus HC at all ages. Individuals with MDD on second-generation antipsychotic medication showed a thinner cortex in the left hemisphere relative to HC (Supplementary Tables S122–S147). The cortical surface area in the left and right hemispheres was smaller in the individuals with SZ who were on second-generation antipsychotics, first-generation antipsychotics, and both first and second-generation antipsychotic groups relative to HC (Supplementary Tables S148–S158). The cortical surface area related to medications in the left and right hemispheres was generally not significantly different for individuals with BD and MDD, except for individuals with MDD on antidepressants, and all age groups of individuals with MDD who were unmedicated with antipsychotics showed significantly smaller cortical surface areas in the left and right hemispheres compared to HC (Supplementary Tables S159–S189). The Positive and Negative Syndrome Scale (PANSS) [28] scores for individuals with SZ and the Hamilton Depression Rating Scale [29] and the Beck Depression Inventory scores [30] for individuals with MDD were not correlated with cortical thickness and surface area in either brain hemisphere (Supplementary Tables S190–S205). In the case‒control analyses of ASD with IQ added as a covariate, the trend was not different from that of the analyses without IQ as a covariate (Supplementary Tables S206–S212).

## Discussion

**Fig. 5:**
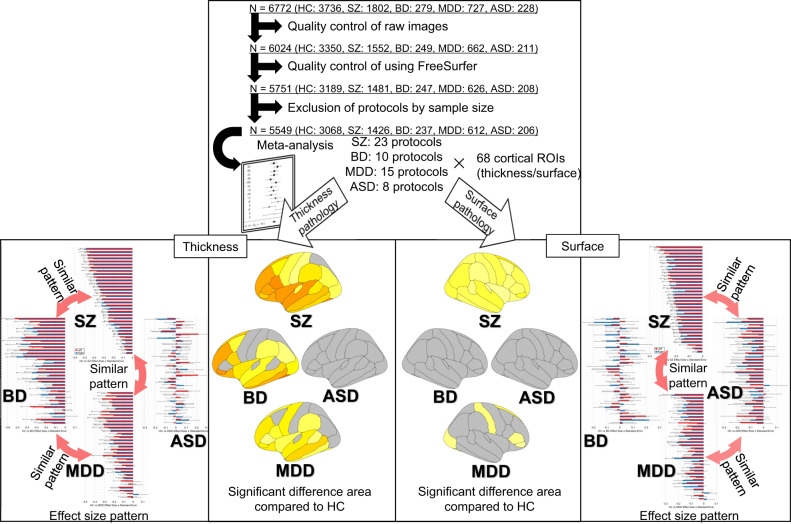
An infographic of the study design and a summary of the results. Large-scale multicenter analyses were conducted by COCORO for SZ, BD, MDD, and ASD. Harmonization was performed by meta-analysis. The effect sizes were calculated in the group comparison of Analysis A between each disorder group and the healthy control group obtained in each of 68 DK atlas ROIs for cortical thickness and surface area. To investigate the patterns of thickness and surface area, cosine similarity was calculated between SZ and BD, between SZ and MDD, between SZ and ASD, between BD and MDD, between BD and ASD, and between MDD and ASD using the effect sizes obtained in the meta-analysis of each of the 68 ROIs. ASD autism spectrum disorder, BD bipolar disorder, HC healthy comparison subjects, MDD major depressive disorder, ROI region of interest, SZ schizophrenia.

In this multicenter cross-disorder study of 5549 individuals with four major psychiatric disorders, we found a similar pattern of cortical thickness by region among SZ, BD, and MDD, with generally thinner cortical thickness in SZ, BD, and MDD (Fig. 5). We also found a similar pattern of cortical surface area by region among SZ, MDD, and ASD, in addition to generally smaller cortical surface area in SZ and MDD. The effect sizes for both cortical thickness and surface area compared to HC were the largest in SZ. To the best of our knowledge, this is the first report of a cross-disorder comparison of these disorders in a single study.

The similar patterns among SZ, BD, and MDD and different pattern in ASD in cortical thickness, as well as the similar patterns among SZ, MDD, and ASD and different pattern in BD in cortical surface area, were novel findings from the present study. In comparison with disorder-specific ENIGMA studies, the case‒control results were approximately the same in SZ [7] and BD [8]. In MDD [9] and ASD [10], the case‒control studies were different results; the differences may be due to the differences in the age range. Genetic correlation studies show that SZ, BD, and MDD correlate strongly with each other, while ASD is not strongly correlated with MDD or BD [31, 32]. Therefore, the similarity pattern of cortical thickness shows a parallel trend to the genetic correlation between SZ, BD, and MDD, but the similarity pattern of the surface area is inconsistent with the genetic correlation pattern. These facts may suggest that the genome has a greater effect on cortical thickness than on cortical surface area. Studies examining biological associations with effect size maps from previous ENIGMA studies have been attempted for cortical thickness [33] as well as connectivity [34]. On the other hand, approximately 50 loci have been found to affect cortical thickness, and approximately 200 have been found to affect cortical surface area [35]. In addition, cortical thickness and surface area show a negative genetic correlation [35]. According to the radial unit hypothesis [36], cortical surface area is expanded by the proliferation of neural progenitor cells through a neurobiological mechanism distinct from the mechanisms that govern cortical thickness. Cortical thickness is influenced by myelination, branching, pruning, and other processes that occur after mid-fetal development [37] and it may be related to common pathologies shared among SZ, BD, and MDD. The similarity of the cortical surface area pattern in SZ, MDD, and ASD may indicate that these disorders share a common pathology originating in the fetal neural progenitor cell stage.

The effect sizes for both cortical thickness and surface area compared to HC were the largest in SZ. The global mean thickness of SZ, BD, and MDD patients was thinner than that of HC. The effect sizes were the largest for SZ, the second largest for BD, and the third largest for MDD in the negative direction. The cortical surface area of SZ and MDD patients was smaller than that of HC. Their effect sizes were the largest for SZ and the second largest for MDD. The effect size of ASD was also negative, which means the same direction for SZ and MDD, although it was not statistically significant. The largest effect size on cortical thickness and surface area in SZ is consistent with each disorder-specific ENIGMA study of individual disorders, which discussed their reviews [3, 7–10]. The strength of the present study is the comparison within the same study, rather than a comparison of the effect sizes of multiple previous studies. A previous cross-disorder DTI study [4] showed that white matter microstructural abnormalities in SZ had the largest effect sizes of the four major psychiatric disorders examined here. This is similar to the present gray matter study; abnormalities of cortical thickness and surface area in SZ had the largest effect sizes of the four major psychiatric disorders. White matter, cortical thickness, and surface area were larger in effect size in SZ than in other major psychiatric disorders, suggesting that SZ may have the greatest impairment in white matter, cortical thickness, and surface area. Except for SZ, the order of magnitude of effect sizes differed for white matter, cortical thickness, and surface area. The findings suggest that the pathogenesis of BD, MDD, and ASD may have different effects on white matter, cortical thickness, and surface area. Differences in the effect size of white matter, cortical thickness, and surface area may explain the differences in the symptoms of each disorder.

Bedford et al. [38]. reported that rigorous QC (the final dataset was 35% of the initial dataset for ASD and 48% of the initial dataset for HC) to exclude body motion artifacts would result in smaller estimates of cortical thickening, especially in ASD. However, in the present study, we performed QC at the general level. Therefore, the thinning of cortical thickness in SZ, BD, and MDD reported in this study might have been more intense if QC had been performed at the strict level of Bedford et al. In addition, cortical thickness thickening may have been smaller in ASD, while cortical thickness thinning may have been more intense.

This study has several limitations. First, although there are many previous studies of older adults with MDD and of children to adolescents with ASD, our study focuses mainly on adults, making it difficult to make direct comparisons with many prior studies, which also included many older individuals with MDD or with children to adolescents with ASD. In the future, it may be possible to detect cross-disorder cortical changes according to the stage of brain development by analyzing a larger number of patients with a more evenly balanced age distribution - from adolescents to older adults - for all disorders. Second, the current study included many medicated patients, so it is not clear to what extent the effects are due to the disease, drug treatment, or both. Addressing this issue requires studies focusing on drug-free patients or, more ideally, drug-naïve patients. To reveal the effects of psychotropic drug use on cortical structure, interventional studies comparing pre- and post-use of specific medications are needed. Large sample, cross-disorder, prospective, and longitudinal studies are optimal. In addition, alcohol dependence [39, 40], smoking [41, 42], and abuse of substances such as cocaine [43] may affect cortical structures, and were not examined here. Third, not only cortical structures but also the association between structures and symptoms should be examined in a cross-disorder fashion. Unfortunately, in the present study, our data on symptoms are limited to diagnosis-specific rating scales; for example, we have PANSS scores for SZ, and we have HRSD and BDI scores for MDD. In the future, it will be necessary to conduct evaluations using cross-disorder rating scales.

In conclusion, a common cortical thickness pattern was found in SZ, BD, and MDD. It was also found that SZ, MDD, and ASD share a common pattern of cortical surface area abnormalities. Cross-disorder brain imaging research based on multicenter studies can help to advance the understanding of the pathogenesis of psychiatric disorders.

### Supplementary information

Supplementary Methods

Supplementary Tables
