# Supplementary material for: Cerebral cortical structural alteration patterns across four major psychiatric disorders in 5549 individuals
Source: Mol Psychiatry. 2023 Aug 18;28(11):4915–23. doi: 10.1038/s41380-023-02224-7 (PMC10914601; doi:10.1038/s41380-023-02224-7)
Supplement: Supplementary file 1 — Supplementary Methods [file 41380_2023_2224_MOESM1_ESM.docx]

**Supplementary Methods 1** *Subject inclusion and exclusion criteria by site*

Participants recruited from the Osaka site had no biological relations, and all of them were of Japanese descent.^1-6^ Patients with schizophrenia, bipolar disorder, autism spectrum disorder, and major depressive disorder were recruited from the Osaka University Hospital. Each patient had been diagnosed by at least two trained psychiatrists according to the criteria from the diagnostic and statistical manual of mental disorders, fourth edition (DSM-IV) based on the structured clinical interview for DSM-IV (SCID).^7^ Healthy comparison subjects were recruited through local advertisements at Osaka University. They were evaluated using the non-patient version of the SCID ^8^ to exclude individuals who had current or past contact with psychiatric services or who had received psychiatric medications. They were excluded if they had neurological or medical conditions that could potentially affect the central nervous system, such as atypical headaches, head trauma with loss of consciousness, chronic lung disease, kidney disease, chronic hepatic disease, thyroid disease, active cancer, cerebrovascular disease, epilepsy, seizures, substance-related disorders, or mental retardation.

Inclusion and exclusion criteria for participants with schizophrenia at the Tokyo1, Tokyo2, and Tokyo3 site have been described elsewhere.^9-11^ Briefly, the diagnosis of patients with schizophrenia was determined according to the SCID-I clinical version. Healthy comparison subjects were screened for neuropsychiatric disorders through the SCID-I non-patient edition. The exclusion criteria for these groups were current or past neurological illness, previous traumatic brain injury with any known cognitive consequences or loss of consciousness for more than 5 min, history of electroconvulsive therapy, autism spectrum disorder (ASD) that met DSM-IV criteria, and previous substance abuse or dependence based on clinical histories. Additional exclusion criteria for the healthy comparison subjects were a history of psychiatric disease in the subjects themselves or of axis I disorders amongst their first-degree relatives. For participants with ASD, high-functioning ASD males, who satisfied the eligibility criteria (firm ASD diagnosis, aged between 20 and 55 years, full-scale intelligence quotient [IQ] > 80, verbal-IQ > 85), were participated.^12-14^ Briefly, participants with ASD were diagnosed according to the strict criteria included in the Diagnostic and Statistical Manual-Revision IV-Text Revision (DSM-IV TR).^15^ The diagnosis was further confirmed using the validated Japanese version of the Autism Diagnostic Interview-Revised (ADI-R).^16^ For the participants not reaching the threshold in the ADI-R social domain, an evaluation with the Autism Diagnostic Observation Schedule^17^ was employed and confirmed the diagnosis of ASD. A group of age- and IQ- matched, typically developed (TD) adult males were included as controls. All of the ASD and TD participants were interviewed by a trained psychiatrist (H.Y.) to screen for the presence of neuropsychiatric disorders using the Structured Clinical Interview for DSM-IV Axis I Disorder.^18^ The exclusion criteria for both groups were: current or past neurological comorbidity, traumatic brain injury with any known cognitive consequences or loss of consciousness for more than 5 minutes, a history of electroconvulsive therapy, and substance abuse or addiction. An additional exclusion criterion for the control group was a history of psychiatric disease in the subjects themselves or a family history of an axis I disorder in their first-degree relatives.

Participants at the Tokyo4 were excluded if they had physical conditions that might potentially influence the central nervous system. Patients with schizophrenia were recruited from the University of Tokyo Hospital. Healthy comparison subjects were sampled from the Japanese study of stratification, health, income, and neighborhood (J-SHINE) survey.^19^ The diagnostic procedure was the same as that at Yaesu site described in our previous study.^20^

Participants at the Tokyo5 were excluded if they had current or past neurological illness, history of electroconvulsive therapy, or previous substance abuse or dependence based on clinical histories. Individuals with schizophrenia, bipolar disorder, major depressive disorder, and autism spectrum disorder as well as healthy comparison subjects were recruited at the University of Tokyo Hospital. The diagnostic procedure was the same as that at the University of Tokyo described in our previous study.^21^

Participants recruited at the Nippon Medical site were excluded if they had a history of head injury, neurological illness, or a diagnosis of substance abuse or dependence. Patients with SZ were recruited from the Asai Hospital, and all patients were diagnosed by trained psychiatrists according to the DSM-IV or DSM5 criteria. Healthy comparison subjects were excluded if they had a history of DSM-IV axis I or axis II disorder. These comparison subjects were recruited through local advertisements and from hospital staff at Asai hospital.

Participants recruited from the Hiroshima site were all right-handed, native speakers of Japanese. Patients with bipolar disorder and major depressive disorder were recruited from Hiroshima University Hospital or local clinics in Hiroshima city. The patients were diagnosed by a senior psychiatrist according to the DSM-IV or DSM-5 criteria beforehand, and the M.I.N.I. was performed at the time of participation in the study to confirm the diagnosis. Healthy comparison subjects were recruited through a newspaper advertisement. These participants were interviewed prior to enrollment by a trained psychiatrist using the M.I.N.I. Participants were excluded from all groups if they had diagnosis of schizophrenia, alcohol and substance abuse/dependence, dementia, developmental disorders, eating disorders, personality disorder, or severe physical illness, or if they had high-level suicide risk, or if they were currently breast-feeding during pregnancy or in the postpartum period. Individuals who demonstrated a history of psychiatric illness or contact with psychiatric services were excluded from the healthy comparison subject group.

Participants recruited at the Kyoto site were physically healthy when they underwent scanning. Exclusion criteria for all individuals included a history of head trauma, neurological illness, serious medical or surgical illness, and substance abuse. Patients with schizophrenia recruited at Kyoto University included diagnoses of schizophrenia, schizoaffective disorder, and schizophreniform disorder. were diagnosed with the patient edition of the Structured Clinical Interview for DSM-IV (SCID) Axis I Disorders (SCID-I). They were not comorbid with any other DSM-IV axis I psychiatric disorders. Healthy comparison subjects were recruited by local advertisements and word of mouth. They had no history of psychiatric illness, as screened with the non-patient edition of the SCID, and it was confirmed that their first-degree relatives had no history of psychotic disorders.

All subjects recruited from the Toyama1 site were Japanese and physically healthy at the time of the study. None had a lifetime history of serious head trauma, neurological illness, serious medical or surgical illness, or substance abuse disorders. Patients with schizophrenia were recruited from the in- and outpatient clinics of the Department of Neuropsychiatry of Toyama University Hospital. The patients were diagnosed by experienced psychiatrists according to the ICD-10 research criteria.^13^ They were asked to complete a questionnaire consisting of 15 items concerning their personal (13 items; including a history of obstetric complications, substantial head injury, seizures, neurological or psychiatric disease, impaired thyroid function, hypertension, diabetes, and substance abuse) and family (2 items) histories of illness. Controls with any personal or family history of psychiatric illness among their first-degree relatives were excluded.

All subjects recruited from the Toyama2 site were Japanese and physically healthy at the time of the study. None had a lifetime history of serious head trauma, neurological illness, serious medical or surgical illness, or substance abuse disorders. Patients with schizophrenia fulfilling the DSM-IV-TR criteria were recruited from the in- and outpatient clinics of the department of neuropsychiatry of Toyama University Hospital. They were diagnosed based on information obtained from a clinical assessment using the SCID-I, a detailed chart review, as well as the clinical symptoms rated at the time of scanning. Healthy comparison subjects, who were screened for psychiatric illness using the SCID-I non-patient edition, were recruited from members of the local community, hospital staff, and university students. They were asked to complete a questionnaire consisting of 19 items concerning their personal (17 items; including a history of obstetric complications, substantial head injury, seizures, neurological illness, impaired thyroid function, hypertension, diabetes, and substance abuse) and family (2 items) histories of illness. Subjects with family history of psychiatric illness among their first-degree relatives were excluded.

All subjects recruited in Kanazawa site were of Japanese descent, and all were biologically unrelated to at least the second degree. Patients were recruited from both the outpatient and inpatient populations at Kanazawa Medical University Hospital.^22-26^ Each patient with schizophrenia, bipolar disorder, or major depressive disorder had been diagnosed by at least two trained psychiatrists on the basis of unstructured clinical interviews, medical records and clinical conferences. Diagnoses were made according to criteria in the DSM-5. Healthy comparison subjects were recruited through local advertisements and from among hospital staff at Kanazawa Medical University. Healthy comparison subjects were evaluated using Structured Clinical Interview for DSM-IV-Non-Patient version (SCID-NP) to exclude individuals who had had current or past contact with psychiatric services or who had received psychiatric medication. Subjects were excluded from analysis if they had neurological or medical conditions that could affect the central nervous system, including head trauma with loss of consciousness, chronic lung disease, chronic hepatic disease, kidney disease, active cancer, cerebrovascular disease, seizures, epilepsy, substance-related disorders or intellectual disorder.^22-26^ Written informed consent was obtained from all subjects after the procedures were fully explained. This study was performed according to the world medical association’s declaration of Helsinki and was approved by the research ethical committees of Kanazawa Medical University and Gifu University.

Participants at the Nagoya site were excluded if they had physical conditions that might potentially influence the central nervous system. All patients (schizophrenia, bipolar disorder and autism spectrum disorder) were recruited from Nagoya University Hospital and affiliated psychiatric hospitals. All patients were diagnosed by trained psychiatrists according to the DSM-IV criteria. Healthy comparison subjects were recruited from the local community, hospital staff, and university students at Nagoya University and affiliated psychiatric hospitals. Healthy comparison subjects were evaluated using the non-patient version of the SCID to exclude individuals who had current or past contact with psychiatric services or who had received psychiatric medications.

Participants at the Hokkaido site were excluded if they had physical conditions that could potentially influence the central nervous system. Patients whose diagnosis fell under any of the following were recruited from the Hokkaido University Hospital; schizophrenia, bipolar disorder, and major depressive disorder. All patients were diagnosed by psychiatrists with at least 6 years of clinical experience according to the DSM-IV or DSM-5 criteria. Healthy comparison subjects were recruited through local advertisements at Hokkaido University. All healthy comparison subjects were screened to exclude any psychiatric disorders using the Japanese version of the M.I.N.I.^27^ by trained psychiatrists.

All subjects recruited from the Kyushu site were Japanese and physically healthy at the time of the study. The exclusion criteria were: 1) neurological illness or major head trauma; 2) electroconvulsive therapy; 3) alcohol or drug dependence; 4) alcohol or drug abuse within the past 5 years. Healthy comparison subjects were screened using the SCID non-patient edition. No healthy comparison subjects had an Axis-I psychiatric disorder themselves or amongst their first-degree relatives. All patients were recruited from Kyushu University Hospital and Hoaki Hospital and were diagnosed by at least two trained psychiatrists based on the SCID-DSM IV and medical records.

Participants at the Yamaguchi site were recruited from Yamaguchi University Hospital and healthy subjects were recruited from the local area. Patients met the DSM-IV-text revision (TR) criteria by M.I.N.I., clinical interviews, and case conferences by senior psychiatrists. Healthy participants were recruited by advertisements and word-of-mouth in the surrounding community. Patients with current or history of substance abuse or dependence and other psychotic illnesses were excluded. Healthy comparison subjects were screened during clinical interviews using the M.I.N.I. Healthy comparison subjects with immediate family members having any psychiatric disorder were excluded. Based on interviews, blood tests and physical examinations, subjects with an endocrinological disease, head trauma, neurological disease, family history of any hereditary neurological disorder, or other medical conditions (e.g., hypertension, diabetes, active liver disease, kidney problems, or respiratory problems) were also excluded. Subjects also participated in interviews to obtain clinical demographics. This study protocol was approved by the institutional review board of Yamaguchi University Hospital.

Participants recruited from the Showa site had no biological relations, and all of them were of Japanese descent. The subjects were excluded if they had neurological or medical conditions that could potentially affect the central nervous system. Individuals with autism spectrum disorder were recruited from the Showa University Karasuyama Hospital. The diagnostic procedure for individuals with autism spectrum disorder was the same as our previous studies.^28-31^ Briefly, experienced psychiatrists carefully diagnosed the patients as autism spectrum disorder if there was a consensus between the psychiatrist and clinical psychologist who interviewed the patients independently based on the DSM-IV-TR. Healthy comparison subjects were recruited by advertisements and acquaintances. None of the healthy comparison subjects reported any severe medical problem or any neurological or psychiatric history. Moreover, the M.I.N.I. was used to confirm that none of the healthy comparison subjects met the diagnostic criteria for any psychiatric disorder.

Participants recruited from Tokushima University Hospital had no biological relations, and all of them were of Japanese descent. The patients had been diagnosed with schizophrenia based on the criteria in the DSM-IV. At the time enrolment, all patients were clinically stable, as judged by a therapeutic psychiatrist. The criteria for determining clinical stability were no schedule to change treatment contents; essentially no psychopathological changes; judgment of clinically stable by a therapeutic psychiatrist and patients themselves. Each patient’s clinical stability was assessed from medical records, self-reports, and the observations of psychiatric staff and relatives. The exclusion criteria were a past history or presence of any serious disorders affecting the brain or cognitive functioning, such as epilepsy, serious head injury, or brain tumor; alcohol abuse; active drug use in the past year; or pregnancy or intention to become pregnant during the study period. Healthy comparison subjects were evaluated with structured clinical interviews to confirm the absence of schizophrenia and had no history of neurological or psychiatric disorders or any first-degree relatives with psychotic episodes.

Participants at the UOEH site were excluded if they had physical conditions that might influence the central nervous system or if they had been previously diagnosed with epilepsy, seizures, substance-related disorders, or mental retardation. Patients with schizophrenia were recruited from the in- and outpatient units at University of Occupational and Environmental Health. All patients were diagnosed by trained psychiatrists according to DSM-IV criteria based on the SCID. Controls were recruited through local advertisements and acquaintances. Healthy subjects were evaluated using the M.I.N.I. to confirm not meeting the diagnostic criteria for any psychiatric disorders.

**References of Supplementary Methods 1**

1. Hashimoto R, Ohi K, Yasuda Y, Fukumoto M, Yamamori H, Takahashi H *et al.* Variants of the RELA gene are associated with schizophrenia and their startle responses. *Neuropsychopharmacology : official publication of the American College of Neuropsychopharmacology* 2011; **36**(9)**:** 1921-1931.

2. Ohi K, Hashimoto R, Yasuda Y, Nemoto K, Ohnishi T, Fukumoto M *et al.* Impact of the genome wide supported NRGN gene on anterior cingulate morphology in schizophrenia. *PloS one* 2012; **7**(1)**:** e29780.

3. Hashimoto R, Ohi K, Yasuda Y, Fukumoto M, Yamamori H, Kamino K *et al.* The KCNH2 gene is associated with neurocognition and the risk of schizophrenia. *The world journal of biological psychiatry : the official journal of the World Federation of Societies of Biological Psychiatry* 2013; **14**(2)**:** 114-120.

4. Ohi K, Hashimoto R, Yasuda Y, Kiribayashi M, Iike N, Yoshida T *et al.* TATA box-binding protein gene is associated with risk for schizophrenia, age at onset and prefrontal function. *Genes, brain, and behavior* 2009; **8**(4)**:** 473-480.

5. Hashimoto R, Ohi K, Yasuda Y, Fukumoto M, Iwase M, Iike N *et al.* The impact of a genome-wide supported psychosis variant in the ZNF804A gene on memory function in schizophrenia. *American journal of medical genetics Part B, Neuropsychiatric genetics : the official publication of the International Society of Psychiatric Genetics* 2010; **153b**(8)**:** 1459-1464.

6. Hashimoto R, Ikeda M, Yamashita F, Ohi K, Yamamori H, Yasuda Y *et al.* Common variants at 1p36 are associated with superior frontal gyrus volume. *Translational psychiatry* 2014; **4:** e472.

7. First M, Spitzer R, Gibbon M, Williams J. *Structured Clinical Interview for DSM-IV Axis I Disorders. Clinical Version.* American Psychiatric Press: Washington, 1997.

8. First M, Spitzer R, Gibbon M, Williams J. *Structured Clinical Interview for DSM-IV axis I disorders, Non-patient Edition.* Biometrics Research Department, New York State Psychiatric Institute: New York, 1997.

9. Iwashiro N, Suga M, Takano Y, Inoue H, Natsubori T, Satomura Y *et al.* Localized gray matter volume reductions in the pars triangularis of the inferior frontal gyrus in individuals at clinical high-risk for psychosis and first episode for schizophrenia. *Schizophrenia research* 2012; **137**(1-3)**:** 124-131.

10. Natsubori T, Inoue H, Abe O, Takano Y, Iwashiro N, Aoki Y *et al.* Reduced frontal glutamate + glutamine and N-acetylaspartate levels in patients with chronic schizophrenia but not in those at clinical high risk for psychosis or with first-episode schizophrenia. *Schizophrenia bulletin* 2014; **40**(5)**:** 1128-1139.

11. Natsubori T, Hashimoto R, Yahata N, Inoue H, Takano Y, Iwashiro N *et al.* An fMRI study of visual lexical decision in patients with schizophrenia and clinical high-risk individuals. *Schizophrenia research* 2014; **157**(1-3)**:** 218-224.

12. Aoki Y, Yahata N, Watanabe T, Takano Y, Kawakubo Y, Kuwabara H *et al.* Oxytocin improves behavioural and neural deficits in inferring others' social emotions in autism. *Brain* 2014; **137**(Pt 11)**:** 3073-3086.

13. Aoki Y, Watanabe T, Abe O, Kuwabara H, Yahata N, Takano Y *et al.* Oxytocin's neurochemical effects in the medial prefrontal cortex underlie recovery of task-specific brain activity in autism: a randomized controlled trial. *Mol Psychiatry* 2015; **20**(4)**:** 447-453.

14. Watanabe T, Abe O, Kuwabara H, Yahata N, Takano Y, Iwashiro N *et al.* Mitigation of sociocommunicational deficits of autism through oxytocin-induced recovery of medial prefrontal activity: a randomized trial. *JAMA psychiatry* 2014; **71**(2)**:** 166-175.

15. American Psychiatric Association. *Diagnostic and Statistical Manual of Mental Disorders, 4th edition text revision (DSM-Ⅳ-TR)***:** American Psychiatric Publishing, Inc.: Washington (DC), 2000.

16. Lord C, Rutter M, Le Couteur A. Autism Diagnostic Interview-Revised: a revised version of a diagnostic interview for caregivers of individuals with possible pervasive developmental disorders. *J Autism Dev Disord* 1994; **24**(5)**:** 659-685.

17. Lord C, Rutter M, Goode S, Heemsbergen J, Jordan H, Mawhood L *et al.* Autism diagnostic observation schedule: a standardized observation of communicative and social behavior. *J Autism Dev Disord* 1989; **19**(2)**:** 185-212.

18. First M, Spitzer R, Gibbon M, Williams J. *Structured Clinical Interview for DSM-IV Axis I disorders – Clinician Version (SCID-CV)*. American Psychiatric Publishing, Inc.: Washington (DC), 1997.

19. Takada M, Kondo N, Hashimoto H. Japanese study on stratification, health, income, and neighborhood: study protocol and profiles of participants. *Journal of epidemiology* 2014; **24**(4)**:** 334-344.

20. Okada N, Fukunaga M, Yamashita F, Koshiyama D, Yamamori H, Ohi K *et al.* Abnormal asymmetries in subcortical brain volume in schizophrenia. *Mol Psychiatry* 2016; **21**(10)**:** 1460-1466.

21. Tanaka SC, Yamashita A, Yahata N, Itahashi T, Lisi G, Yamada T *et al.* A multi-site, multi-disorder resting-state magnetic resonance image database. *Sci Data* 2021; **8**(1)**:** 227.

22. Ohi K, Shimada T, Kihara H, Yasuyama T, Sawai K, Matsuda Y *et al.* Impact of Familial Loading on Prefrontal Activation in Major Psychiatric Disorders: A Near-Infrared Spectroscopy (NIRS) Study. *Scientific reports* 2017; **7:** 44268.

23. Ohi K, Shimada T, Nemoto K, Kataoka Y, Yasuyama T, Kimura K *et al.* Cognitive clustering in schizophrenia patients, their first-degree relatives and healthy subjects is associated with anterior cingulate cortex volume. *NeuroImage Clinical* 2017; **16:** 248-256.

24. Yasuyama T, Ohi K, Shimada T, Uehara T, Kawasaki Y. Differences in social functioning among patients with major psychiatric disorders: Interpersonal communication is impaired in patients with schizophrenia and correlates with an increase in schizotypal traits. *Psychiatry research* 2017; **249:** 30-34.

25. Ohi K, Kataoka Y, Shimada T, Kuwata A, Okubo H, Kimura K *et al.* Meta-analysis of physical activity and effects of social function and quality of life on the physical activity in patients with schizophrenia. *European archives of psychiatry and clinical neuroscience* 2018.

26. Ohi K, Matsuda Y, Shimada T, Yasuyama T, Oshima K, Sawai K *et al.* Structural alterations of the superior temporal gyrus in schizophrenia: Detailed subregional differences. *European psychiatry : the journal of the Association of European Psychiatrists* 2016; **35:** 25-31.

27. Otsubo T, Tanaka K, Koda R, Shinoda J, Sano N, Tanaka S *et al.* Reliability and validity of Japanese version of the Mini-International Neuropsychiatric Interview. *Psychiatry and clinical neurosciences* 2005; **59**(5)**:** 517-526.

28. Itahashi T, Yamada T, Watanabe H, Nakamura M, Jimbo D, Shioda S *et al.* Altered network topologies and hub organization in adults with autism: a resting-state fMRI study. *PloS one* 2014; **9**(4)**:** e94115.

29. Lin IF, Kashino M, Ohta H, Yamada T, Tani M, Watanabe H *et al.* The effect of intranasal oxytocin versus placebo treatment on the autonomic responses to human sounds in autism: a single-blind, randomized, placebo-controlled, crossover design study. *Molecular autism* 2014; **5**(1)**:** 20.

30. Ohta H, Yamada T, Watanabe H, Kanai C, Tanaka E, Ohno T *et al.* An fMRI study of reduced perceptual load-dependent modulation of task-irrelevant activity in adults with autism spectrum conditions. *NeuroImage* 2012; **61**(4)**:** 1176-1187.

31. Watanabe H, Nakamura M, Ohno T, Itahashi T, Tanaka E, Ohta H *et al.* Altered orbitofrontal sulcogyral patterns in adult males with high-functioning autism spectrum disorders. *Social cognitive and affective neuroscience* 2014; **9**(4)**:** 520-528.

**Supplementary Methods 2** *Detailed imaging parameters for each protocol*

In the Osaka1 group, the acquisitions of T1-weighted images were performed on a 1.5 Tesla GE Signa EXCITE scanner using a 3D IR-fast spoiled gradient echo (SPGR) sequence and a HEAD (QD) coil with the following parameters: repetition time (TR) = 12.6 ms, echo time (TE) = 4.2 ms, repetition time (TR) = 12.6 ms, inversion time (TI) = 400 ms, flip angle = 15 degrees, matrix = 256 × 256 × 124, field of view (FOV) = 240 × 240 × 172 mm, voxel size = 0.9375 × 0.9375 × 1.4 mm. The slice direction was in the sagittal plane.

In the Osaka2 group, the acquisitions of T1-weighted images were performed on a 3.0 Tesla GE Signa HDxt 3.0T scanner using a fast SPGR sequence and 8HRBRAIN coil with the following parameters; TR = 7.2 ms, TE = 2.9 ms, TI = 400 ms, flip angle = 11 degrees, matrix = 256 × 256 × 172, FOV = 240 × 240 × 172 mm, voxel size = 0.9375 × 0.9375 × 1 mm. The slice direction was in the sagittal plane.

In the Osaka3 group, the acquisitions of T1-weighted images were performed on a 3.0 Tesla GE DISCOVERY 750 scanner using a fast SPGR sequence and HNS Head coil with the following parameters; TR = 8.152 ms, TE = 3.172 ms, TI = 400 ms, flip angle = 11 degrees, matrix = 256 × 256 × 156, FOV = 260 × 260 × 188 mm, voxel size = 1.0156 × 1.0156 × 1.2 mm. The slice direction was in the sagittal plane.

In the Tokyo1 group, the acquisitions of T1-weighted images were performed on a 1.5 Tesla GE Signa Horizon scanner using a SPGR sequence and Circularly polarized head coil coil with the following parameters; TR = 35 ms, TE = 7 ms, flip angle = 30 degrees, matrix = 256 × 256 × 124, FOV = 186 × 240 × 240 mm, voxel size = 1.5 × 0.9375 × 0.9375 mm. The slice direction was in the Axial plane.

In the Tokyo2 group, the acquisitions of T1-weighted images were performed on a 3T Tesla GE Signa scanner using a fast SPGR sequence and 8 ch coil with the following parameters; TR = 6.8 ms, TE = 1.94 ms, TI was unknown, flip angle = 20 degrees, matrix = 256 × 256 × 176, FOV = 176 × 256 × 256 mm, voxel size = 1 × 1 × 1 mm. The slice direction was in the axial plane.

In the Tokyo3 group, the acquisitions of T1-weighted images were performed on a 3T Tesla GE Discovery MR750W scanner using a fast SPGR sequence and 32 ch coil with the following parameters; TR = 8.5 ms, TE = Min Full ms, TI = 450 ms, flip angle = 20 degrees, matrix = 256 × 256 × 176, FOV = 176 × 256 × 256 mm, voxel size = 1 × 1 × 1 mm. The slice direction was in the axial plane.

In the Tokyo4 group, the acquisitions of T1-weighted images were performed on a 3.0 Tesla Philips Achieva scanner using a MPRAGE sequence and SENSE-Head-8 coil with the following parameters; TR = 6.8 (shortest) ms, TE = 3.1 ms, TI = 845.9 (minimum delay) ms, flip angle = 9 degrees, matrix = 256 × 256 (ACQ: 256 × 240 × 170), FOV = 256 × 240 × 204 mm, voxel size = 1 × 1 × 1.2 mm. The slice direction was in the sagittal plane.

In the Tokyo5 group, the acquisitions of T1-weighted images were performed on a 3.0 Tesla GE Discovery MR750w scanner using a SPGR sequence and Head 24 coil with the following parameters; TR = 7.7 ms, TE = 3.1 ms, TI = 400 ms, flip angle = 11 degrees, matrix = 256 × 256 × 200, FOV = 260 × 260 × 240 mm, voxel size = 1 × 1 × 1.2 mm. The slice direction was in the sagittal plane.

In the NipponMedical2 group, the acquisitions of T1-weighted images were performed on a 1.5 Tesla GE Signa HDxp scanner using a SPGR sequence and phased-array coil with the following parameters; TR = 7.4 ms, TE = 3.4 ms, TI = 20 ms, flip angle = 20 degrees, matrix = 256 × 256 × 50, FOV = 256 × 256 × 200 mm, voxel size = 1 × 1 × 1 mm. The slice direction was in the axial plane.

In the Hiroshima1 group, the acquisitions of T1-weighted images were performed on a 3.0 Tesla GE Signa HDxt scanner using a SPGR sequence and 8-channel head coil with the following parameters; TR = 6.9 ms, TE = 1.9 ms, TI = 450 ms, flip angle = 20 degrees, matrix = 256 × 256 × 184, FOV = 256 × 256 × 184 mm, voxel size = 1 × 1 × 1 mm. The slice direction was in the sagittal plane.

In the Hiroshima2 group, the acquisitions of T1-weighted images were performed on a 1.5 Tesla SIEMENS Symphony scanner using a gradient echo pulse sequence and head coil with the following parameters; TR = 2160 ms, TE = 3.93 ms, TI = 1100 ms, flip angle = 15 degrees, matrix = 256 × 256 × 256, FOV = 256 × 256 × 256 mm, voxel size = 1 × 1 × 1 mm. The slice direction was in the sagittal plane.

In the Hiroshima3 group, the acquisitions of T1-weighted images were performed on a 3.0 Tesla SIEMENS MAGNETOM Spectra scanner using a MPRAGE sequence and 12-channel head coil with the following parameters; TR = 1900 ms, TE = 2.38 ms, TI = 900 ms, flip angle = 10 degrees, matrix = 256 × 256 × 192, FOV = 256 × 256 × 192 mm, voxel size = 0.8 × 0.8 × 0.8 mm. The slice direction was in the sagittal plane.

In the Hiroshima4 group, the acquisitions of T1-weighted images were performed on a 3.0 Tesla GE Signa HDxt scanner using a SPGR sequence and 8-channel head coil with the following parameters; TR = 6.9 ms, TE = 1.9 ms, TI = 450 ms, flip angle = 20 degrees, matrix = 256 × 256 × 184, FOV = 256 × 256 × 184 mm, voxel size = 1 × 1 × 1 mm. The slice direction was in the sagittal plane.

In the Hiroshima5 group, the acquisitions of T1-weighted images were performed on a 3.0 Tesla SIEMENS MAGNETOM Verio.Dot scanner using a MPRAGE sequence and 12-channel head coil with the following parameters; TR = 2300 ms, TE = 2.98 ms, TI = 900 ms, flip angle = 9 degrees, matrix = 256 × 256 × 192, FOV = 256 × 256 × 192 mm, voxel size = 1 × 1 × 1 mm. The slice direction was in the sagittal plane.

In the Kyoto1 group, the acquisitions of T1-weighted images were performed on a 3.0 Tesla Siemens Trio scanner using a MPRAGE sequence and 8ch coil with the following parameters; TR = 2000 ms, TE = 4.38 ms, TI = 990 ms, flip angle = 8 degrees, matrix = 240 × 256 × 208, FOV = 208 × 240 × 225 mm, voxel size = 1 × 0.9375 × 0.9375 mm. The slice direction was in the transversal plane.

In the Kyoto2 group, the acquisitions of T1-weighted images were performed on a 3.0 Tesla Siemens Trio-Tim scanner using a MPRAGE sequence and 32ch coil with the following parameters; TR = 2000 ms, TE = 3.4 ms, TI = 990 ms, flip angle = 8 degrees, matrix = 240 × 256 × 208, FOV = 208 × 240 × 225 mm, voxel size = 1 × 0.9375 × 0.9375 mm. The slice direction was in the transversal plane.

In the Toyama1 group, the acquisitions of T1-weighted images were performed on a 1.5 Tesla Siemens Magnetom Vision scanner using a 3D gradient echo sequence FLASH sequence and CP Head coil coil with the following parameters; TR = 24 ms, TE = 10 ms, flip angle = 40 degrees, matrix = 256 × 256 × 160-180, FOV = 256 × 256 × 179 mm, voxel size = 1 × 1 × 1 mm. The slice direction was in the sagittal plane.

In the Toyama2 group, the acquisitions of T1-weighted images were performed on a 3T Tesla Siemens Verio scanner using a MPRAGE sequence and 12ch head coil coil with the following parameters; TR = 2300 ms, TE = 2.9 ms, TI = 900 ms, flip angle = 9 degrees, matrix = 256 × 256 × 176, FOV = 256 × 240 × 212 mm, voxel size = 1 × 1 × 1.2 mm. The slice direction was in the sagittal plane.

In the Kanazawa1 group, the acquisitions of T1-weighted images were performed on a 3.0 Tesla SIEMENS MAGNETOM Trio A Tim System scanner using a MPRAGE sequence and 32ch coil with the following parameters; TR = 1420 ms, TE = 2.08 ms, TI = 800 ms, flip angle = 9 degrees, matrix = 256 × 256 × 192, FOV = 230 × 230 × 173 mm, voxel size = 1 × 1 × 1 mm. The slice direction was in the Sagittal plane.

In the Nagoya1 group, the acquisitions of T1-weighted images were performed on a 3.0 Tesla Siemens Verio scanner using a MPRAGE sequence and Head-32 coil with the following parameters; TR = 2500 ms, TE = 2.48 ms, TI = 900 ms, flip angle = 8 degrees, matrix = 256 × 256 × 192, FOV = 256 × 256 × 192 mm, voxel size = 1 × 1 × 1 mm. The slice direction was in the sagittal plane.

In the Nagoya3 group, the acquisitions of T1-weighted images were performed on a 3.0 Tesla Siemens Trio scanner using a MPRAGE sequence and Head-32 coil with the following parameters; TR = 1570 ms, TE = 2.19 ms, TI = 800 ms, flip angle = 15 degrees, matrix = 256 × 256 × 224, FOV = 256 × 256 × 224 mm, voxel size = 1 × 1 × 1 mm. The slice direction was in the sagittal plane.

In the Hokkaido1 group, the acquisitions of T1-weighted images were performed on a 1.5 Tesla SIEMENS Magnetom Symphony scanner using a MPRAGE sequence and Quadrature Coil coil with the following parameters; TR = 1900 ms, TE = 3.93 ms, TI = 1100 ms, flip angle = 15 degrees, matrix = 256 × 256 × 240, FOV = 250 × 240 × 250 mm, voxel size = 0.977 × 1 × 0.977 mm. The slice direction was in the Cor plane.

In the Hokkaido2 group, the acquisitions of T1-weighted images were performed on a 3.0 Tesla Philips Achieva scanner using a MPRAGE sequence and SENSE-Head-32 coil with the following parameters; TR = 6.8 ms, TE = 3.1 ms, TI = 2500 ms, flip angle = 8 degrees, matrix = 256 × 256 × 170, FOV = 256 × 240 × 204 mm, voxel size = 1 × 1 × 1.2 mm. The slice direction was in the sagittal plane.

In the Kyushu1 group, the acquisitions of T1-weighted images were performed on a 3T Tesla Philips Achieva 3.0T TX/Achieva 3.0T scanner using a 3D T1-TFE sequence and 8ch coil with the following parameters; TR = 8.2 ms, TE = 3.8 ms, TI = 1025.9 ms, flip angle = 8 degrees, matrix = 240 × 240 × 190, FOV = 240 × 240 × 190 mm, voxel size = 1 × 1 × 1 mm. The slice direction was in the sagittal plane.

In the Kyushu2 group, the acquisitions of T1-weighted images were performed on a 3T Tesla Philips Achieva 3.0T TX/Achieva 3.0T scanner using a 3D T1-TFE sequence and 8ch coil with the following parameters; TR = 8.6 ms, TE = 4 ms, TI = 1072.5 ms, flip angle = 8 degrees, matrix = 240 × 240 × 190, FOV = 240 × 240 × 190 mm, voxel size = 1 × 1 × 1 mm. The slice direction was in the sagittal plane.

In the Kyushu3 group, the acquisitions of T1-weighted images were performed on a 3T Tesla Philips Achieva 3.0T TX/Achieva 3.0T scanner using a 3D T1-TFE sequence and 8ch coil with the following parameters; TR = 8.2 ms, TE = 3.8 ms, TI = 1025.8 ms, flip angle = 8 degrees, matrix = 240 × 240 × 190, FOV = 240 × 240 × 190 mm, voxel size = 1 × 1 × 1 mm. The slice direction was in the sagittal plane.

In the Yamaguchi1 group, the acquisitions of T1-weighted images were performed on a 1.5 Tesla SIEMENS MAGNETOM VISION scanner using a 3D FLASH sequence and CD Head coil with the following parameters; TR = 24 ms, TE = 5 ms, flip angle = 40 degrees, matrix = 256 × 256 × 170, FOV = 256 × 256 × 256 mm, voxel size = 1 × 1 × 1 mm. The slice direction was in the Sagittal plane.

In the Yamaguchi2 group, the acquisitions of T1-weighted images were performed on a 3.0 Tesla SIEMENS Skyra scanner using a MPRAGE sequence and Head-Neck Coil 20ch coil with the following parameters; TR = 2300 ms, TE = 2.95 ms, TI = 900 ms, flip angle = 9 degrees, matrix = 256 × 256 × 176, FOV = 270 × 254 × - mm, voxel size = 1.1 × 1.1 × 1.2 mm. The slice direction was in the Sagittal plane.

In the Showa1 group, the acquisitions of T1-weighted images were performed on a 3.0 Tesla Siemens Verio scanner using a MPRAGE sequence and Head-12 coil with the following parameters; TR = 2300 ms, TE = 2.98 ms, TI = 900 ms, flip angle = 9 degrees, matrix = 256 × 256 × 240, FOV = 256 × 256 × 240 mm, voxel size = 1 × 1 × 1 mm. The slice direction was in the sagittal plane.

In the Tokushima2 group, the acquisitions of T1-weighted images were performed on a 3.0 Tesla GE DISCOVERY MR750 scanner using a Fast SPGR sequence and 32ch coil with the following parameters; TR = 6.9 ms, TE = 3 ms, TI = 11 ms, flip angle = 11 degrees, matrix = 512 × 512 × 392, FOV = 236 × 240 × 240 mm, voxel size = 0.6 ×0.4688 × 0.4688 mm. The slice direction was in the sagittal plane.

In the UOEH1 group, the acquisitions of T1-weighted images were performed on a 3.0 Tesla GE Signa EXCITE scanner using a FSPGR sequence and Head-8 coil with the following parameters; TR = 10 ms, TE = 4.1 ms, TI = 700 ms, flip angle = 10 degrees, matrix = 256 × 256 × 128, FOV = 240 × 240 × 240 mm, voxel size = 0.9 × 0.9 × 1.2 mm. The slice direction was in the sagittal plane.

**Supplementary Methods 3** *Number of cases eliminated at the QC steps, by diagnosis*

Initially, 6772 participants’ data were included across the HC, SZ, BD, MDD, and ASD groups; the number of included participants was reduced to 6,024 by raw image QC, to 5,751 by FreeSurfer QC, and to 5549 based on the sample size of each protocol. If there were fewer than 5 cases in each diagnostic group, that group was excluded from the protocol. Furthermore, if fewer than 15 cases remained for each protocol as a whole or if only the HC group remained, that protocol was excluded. The MDD group in Hiroshima2 and the BD group in Kyushu3 were excluded from the case‒control comparison because there was no HC group, but 42 cases from the former and 7 cases from the latter remained because they could be used for partial correlation analysis with clinical indicators. Regarding the HC group, 3068 individuals remained as a final dataset which means that 18% were excluded from the initial dataset of 3736 individuals (386 individuals, according for 10% of the initial dataset, were excluded by raw image QC). In the SZ group, 1426 individuals remained as a final dataset, which means that 21% were excluded from the initial dataset of 1802 individuals (250 individuals, accounting for 14% of the initial dataset, were excluded by raw image QC). In the BD group, 237 individuals remained as a final dataset, which means that 15% were excluded from the initial dataset of 279 individuals (30 individuals, accounting for 11% of the initial dataset, were excluded by raw image QC). In the MDD group, 612 individuals remained as a final dataset, which means that 16% were excluded from the initial dataset of 727 (65 individuals, accounting for 9% of the initial dataset, were excluded by raw image QC). In the ASD group, 206 individuals remained as a final dataset, which means that 10% were excluded from the initial dataset of 228 individuals (17 individuals, accounting for 7% of the initial dataset, were excluded by raw image QC).

**Supplementary Methods 4** *Power analysis*

Power analyses were conducted at a power of 0.80 and a one-tailed significance level of 0.05 with G*Power version 3.1.9.7 to estimate the sample sizes required to detect the effect sizes. Number of subjects needed in each group for the given effect sizes in Analysis A of left/right hemisphere mean cortical thickness for the group comparisons between HC and SZ are 55 (left, d = -0.478) and 58 (right, d = -0.466), HC and BD are 69 (left, d = -0.426) and 71 (right, d = -0.420), HC and MDD are 88 (left, d = -0.377) and 136 (right, d = -0.303), HC and ASD are 343477 (left, d = -0.006) and 73168 (right, d = -0.013). Number of subjects needed in each group for the given effect sizes in Analysis A of cortical surface area of left/right hemisphere for the group comparisons between HC and SZ are 104 (left, d = -0.346) and 102 (right, d = -0.351), HC and BD are 54957 (left, d = 0.015) and 343477 (right, d = -0.006), HC and MDD are 269 (left, d = -0.215) and 279 (right, d = -0.211), HC and ASD are 366 (left, d = -0.184) and 414 (right, d = -0.173). Number of subjects needed in each group for the given effect sizes in Analysis A of cortical volume of left/right hemisphere for the group comparisons between HC and SZ are 41 (left, d = -0.557) and 41 (right, d = -0.555), HC and BD are 279 (left, d = -0.211) and 250 (right, d = -0.223), HC and MDD are 90 (left, d = -0.374) and 106 (right, d = -0.344), HC and ASD are 455 (left, d = -0.165) and 490 (right, d = -0.159).
