# Supplementary material for: Cerebral cortical structural alteration patterns across four major psychiatric disorders in 5549 individuals
Source: Mol Psychiatry. 2023 Aug 18;28(11):4915–23. doi: 10.1038/s41380-023-02224-7 (PMC10914601; doi:10.1038/s41380-023-02224-7)
Supplement: Supplementary file 2 — Supplementary Tables [file 41380_2023_2224_MOESM2_ESM.pdf]

Supplementary Tables

Supplementary Table S1. Demographic characteristics of the included individuals
Supplementary Table S2. Clinical characteristics of individuals with schizophrenia
Supplementary Table S3. Clinical characteristics of individuals with bipolar disorder
Supplementary Table S4. Clinical characteristics of individuals with major depressive disorder
Supplementary Table S5. Cortical thickness differences between individuals with schizophrenia and healthy comparison subjects controlling for age and sex
Supplementary Table S6. Cortical thickness differences between individuals with bipolar disorder and healthy comparison subjects controlling for age and sex
Supplementary Table S7. Cortical thickness differences between individuals with bipolar disorder and healthy comparison subjects controlling for age and sex at 25 years of age or older
Supplementary Table S8. Cortical thickness differences between individuals with major depressive disorder and healthy comparison subjects controlling for age and sex
Supplementary Table S9. Cortical thickness differences between individuals with major depressive disorder and healthy comparison subjects controlling for age and sex over 21 years old
Supplementary Table S10. Cortical thickness differences between individuals with autism spectrum disorder and healthy comparison subjects controlling for age and sex
Supplementary Table S11. Cortical thickness differences between individuals with schizophrenia and healthy comparison subjects controlling for age, sex, and global mean cortical thickness
Supplementary Table S12. Cortical thickness differences between individuals with bipolar disorder and healthy comparison subjects controlling for age, sex, and global mean cortical thickness
Supplementary Table S13. Cortical thickness differences between individuals with bipolar disorder and healthy comparison subjects controlling for age, sex, and global mean cortical thickness at 25 years of age or older
Supplementary Table S14. Cortical thickness differences between individuals with major depressive disorder and healthy comparison subjects controlling for age, sex, and global mean cortical thickness
Supplementary Table S15. Cortical thickness differences between individuals with major depressive disorder and healthy comparison subjects controlling for age, sex, and global mean cortical thickness over 21 years old
Supplementary Table S16. Cortical thickness differences between individuals with autism spectrum disorder and healthy comparison subjects controlling for age, sex, and global mean cortical thickness
Supplementary Table S17. Cortical surface area differences between individuals with schizophrenia and healthy comparison subjects controlling for age and sex
Supplementary Table S18. Cortical surface area differences between individuals with bipolar disorder and healthy comparison subjects controlling for age and sex
Supplementary Table S19. Cortical surface area differences between individuals with bipolar disorder and healthy comparison subjects controlling for age and sex at 25 years of age or older
Supplementary Table S20. Cortical surface area differences between individuals with major depressive disorder and healthy comparison subjects controlling for age and sex
Supplementary Table S21. Cortical surface area differences between individuals with major depressive disorder and healthy comparison subjects controlling for age and sex over 21 years of old
Supplementary Table S22. Cortical surface area differences between individuals with autism spectrum disorder and healthy comparison subjects controlling for age and sex
Supplementary Table S23. Cortical surface area differences between individuals with schizophrenia and healthy comparison subjects controlling for age, sex, and total cortical surface area
Supplementary Table S24. Cortical surface area differences between individuals with bipolar disorder and healthy comparison subjects controlling for age, sex, and total cortical surface area
Supplementary Table S25. Cortical surface area differences between individuals with bipolar disorder and healthy comparison subjects controlling for age, sex, and total cortical surface area at 25 years of age or older
Supplementary Table S26. Cortical surface area differences between individuals with major depressive disorder and healthy comparison subjects controlling for age, sex, and total cortical surface area
Supplementary Table S27. Cortical surface area differences between individuals with major depressive disorder and healthy comparison subjects controlling for age, sex, and total cortical surface area over 21 years of old
Supplementary Table S28. Cortical surface area differences between individuals with autism spectrum disorder and healthy comparison subjects controlling for age, sex, and total cortical surface area
Supplementary Table S29. Cortical surface area differences between individuals with bipolar disorder and healthy comparison subjects controlling for age, sex, age × sex, age^2, and age^2 × sex at 25 years of age or older
Supplementary Table S30. Cortical surface area differences between individuals with bipolar disorder and healthy comparison subjects controlling for age, sex, age × sex, age^2, age^2 × sex, and ICV at 25 years of age or older
Supplementary Table S31. Cortical volume differences between individuals with schizophrenia and healthy comparison subjects controlling for age and sex
Supplementary Table S32. Cortical volume differences between individuals with bipolar disorder and healthy comparison subjects controlling for age and sex
Supplementary Table S33. Cortical volume differences between individuals with bipolar disorder and healthy comparison subjects controlling for age and sex at 25 years of age or older
Supplementary Table S34. Cortical volume differences between individuals with major depressive disorder and healthy comparison subjects controlling for age and sex
Supplementary Table S35. Cortical volume differences between individuals with major depressive disorder and healthy comparison subjects controlling for age and sex over 21 years old
Supplementary Table S36. Cortical volume differences between individuals with autism spectrum disorder and healthy comparison subjects controlling for age and sex
Supplementary Table S37. Cortical volume differences between individuals with schizophrenia and healthy comparison subjects controlling for age, sex, and ICV
Supplementary Table S38. Cortical volume differences between individuals with bipolar disorder and healthy comparison subjects controlling for age, sex, and ICV
Supplementary Table S39. Cortical volume differences between individuals with bipolar disorder and healthy comparison subjects controlling for age, sex, and ICV at 25 years of age or older
Supplementary Table S40. Cortical volume differences between individuals with major depressive disorder and healthy comparison subjects controlling for age, sex, and ICV
Supplementary Table S41. Cortical volume differences between individuals with major depressive disorder and healthy comparison subjects controlling for age, sex, and ICV over 21 years old
Supplementary Table S42. Cortical volume differences between individuals with autism spectrum disorder and healthy comparison subjects controlling for age, sex, and ICV
Supplementary Table S43. Sex-by-Diagnosis interaction (SZ/HC) on cortical thickness differences controlling for age and sex
Supplementary Table S44. Age-by-Diagnosis interaction (SZ/HC) on cortical thickness differences controlling for age and sex
Supplementary Table S45. Sex-by-Diagnosis interaction (BD/HC) on cortical thickness differences controlling for age and sex
Supplementary Table S46. Sex-by-Diagnosis interaction (BD/HC) on cortical thickness differences controlling for age and sex at 25 years of age or older
Supplementary Table S47. Age-by-Diagnosis interaction (BD/HC) on cortical thickness differences controlling for age and sex
Supplementary Table S48. Age-by-Diagnosis interaction (BD/HC) on cortical thickness differences controlling for age and sex at 25 years of age or older
Supplementary Table S49. Sex-by-Diagnosis interaction (MDD/HC) on cortical thickness differences controlling for age and sex
Supplementary Table S50. Sex-by-Diagnosis interaction (MDD/HC) on cortical thickness differences controlling for age and sex over 21 years of old
Supplementary Table S51. Age-by-Diagnosis interaction (MDD/HC) on cortical thickness differences controlling for age and sex
Supplementary Table S52. Age-by-Diagnosis interaction (MDD/HC) on cortical thickness differences controlling for age and sex over 21 years of old
Supplementary Table S53. Sex-by-Diagnosis interaction (ASD/HC) on cortical thickness differences controlling for age and sex
Supplementary Table S54. Age-by-Diagnosis interaction (ASD/HC) on cortical thickness differences controlling for age and sex
Supplementary Table S55. Sex-by-Diagnosis interaction (SZ/HC) on cortical surface area differences controlling for age and sex
Supplementary Table S56. Age-by-Diagnosis interaction (SZ/HC) on cortical surface area differences controlling for age and sex
Supplementary Table S57. Sex-by-Diagnosis interaction (BD/HC) on cortical surface area differences controlling for age and sex
Supplementary Table S58. Sex-by-Diagnosis interaction (BD/HC) on cortical surface area differences controlling for age, sex, age × sex, age^2, age^2 × sex, and ICV at 25 years of age or older
Supplementary Table S59. Sex-by-Diagnosis interaction (BD/HC) on cortical surface area differences controlling for age and sex at 25 years of age or older
Supplementary Table S60. Age-by-Diagnosis interaction (BD/HC) on cortical surface area differences controlling for age and sex
Supplementary Table S61. Age-by-Diagnosis interaction (BD/HC) on cortical surface area differences controlling for age, sex, age × sex, age^2, age^2 × sex, and ICV at 25 years of age or older
Supplementary Table S62. Age-by-Diagnosis interaction (BD/HC) on cortical surface area differences controlling for age and sex at 25 years of age or older
Supplementary Table S63. Sex-by-Diagnosis interaction (MDD/HC) on cortical surface area differences controlling for age and sex
Supplementary Table S64. Sex-by-Diagnosis interaction (MDD/HC) on cortical surface area differences controlling for age and sex over 21 years of old
Supplementary Table S65. Age-by-Diagnosis interaction (MDD/HC) on cortical surface area differences controlling for age and sex

Supplementary Table S66. Age-by-Diagnosis interaction (MDD/HC) on cortical surface area differences controlling for age and sex over 21 years of old
Supplementary Table S67. Sex-by-Diagnosis interaction (ASD/HC) on cortical surface area differences controlling for age and sex
Supplementary Table S68. Age-by-Diagnosis interaction (ASD/HC) on cortical surface area differences controlling for age and sex
Supplementary Table S69. Partial correlations between cortical thickness and age of onset controlling for age and sex in individuals with schizophrenia
Supplementary Table S70. Partial correlations between cortical thickness and duration of illness controlling for age and sex in individuals with schizophrenia
Supplementary Table S71. Partial correlations between cortical thickness and duration of illness controlling for age and sex in individuals with bipolar disorder
Supplementary Table S72. Partial correlations between cortical thickness and duration of illness controlling for age and sex in individuals with bipolar disorder aged 25 years and older
Supplementary Table S73. Cortical thickness differences between individuals with major depressive disorder with a late age of onset (over 21 years old; LAO) versus healthy comparison subjects controlling for age and sex
Supplementary Table S74. Cortical thickness differences between individuals with major depressive disorder with an early age of onset (21 years of old or younger; EAO) versus healthy comparison subjects controlling for age and sex
Supplementary Table S75. Cortical thickness differences between individuals with major depressive disorder with an early age of onset (21 years of old or younger; EAO) versus individuals with major depressive disorder with a late age of onset (over 21 years old; LAO) controlling for age and sex
Supplementary Table S76. Partial correlations between cortical thickness and duration of illness controlling for age and sex in individuals with major depressive disorder
Supplementary Table S77. Partial correlations between cortical thickness and duration of illness controlling for age and sex in individuals with major depressive disorder over 21 years of old
Supplementary Table S78. Partial correlations between cortical thickness and age controlling for sex in individuals with autism spectrum disorder
Supplementary Table S79. Partial correlations between cortical surface area and age of onset controlling for age and sex in individuals with schizophrenia
Supplementary Table S80. Partial correlations between cortical surface area and duration of illness controlling for age and sex in individuals with schizophrenia
Supplementary Table S81. Partial correlations between cortical surface area and duration of illness controlling for age and sex in individuals with bipolar disorder
Supplementary Table S82. Partial correlations between cortical surface area and duration of illness controlling for age, sex, age × sex, age^2, age^2 × sex, and ICV in individuals with bipolar disorder aged 25 years and older
Supplementary Table S83. Partial correlations between cortical surface area and duration of illness controlling for age and sex in individuals with bipolar disorder aged 25 years and older
Supplementary Table S84. Cortical surface area differences between individuals with major depressive disorder with a late age of onset (over 21 years old; LAO) versus healthy comparison subjects controlling for age and sex
Supplementary Table S85. Cortical surface area differences between individuals with major depressive disorder with an early age of onset (21 years of old or younger; EAO) versus healthy comparison subjects controlling for age and sex
Supplementary Table S86. Cortical surface area differences between individuals with major depressive disorder with an early age of onset (21 years of old or younger; EAO) versus individuals with major depressive disorder with a late age of onset (over 21 years old; LAO) controlling for age and sex
Supplementary Table S87. Partial correlations between cortical surface area and duration of illness controlling for age and sex in individuals with major depressive disorder
Supplementary Table S88. Partial correlations between cortical surface area and duration of illness controlling for age and sex in individuals with major depressive disorder over 21 years of old
Supplementary Table S89. Partial correlations between cortical surface area and age controlling for sex in individuals with autism spectrum disorder
Supplementary Table S90. Cortical thickness differences between individuals with bipolar disorder type-1 compared to individuals with bipolar disorder type-2 controlling for age and sex
Supplementary Table S91. Cortical thickness differences between individuals with bipolar disorder type-1 compared to individuals with bipolar disorder type-2 controlling for age and sex at 25 years of age or older
Supplementary Table S92. Cortical thickness differences between individuals with first episode major depressive disorder versus healthy comparison subjects controlling for age and sex
Supplementary Table S93. Cortical thickness differences between individuals with first episode major depressive disorder versus healthy comparison subjects controlling for age and sex over 21 years of old
Supplementary Table S94. Cortical thickness differences between individuals with recurrent episode major depressive disorder versus healthy comparison subjects controlling for age and sex
Supplementary Table S95. Cortical thickness differences between individuals with recurrent episode major depressive disorder versus healthy comparison subjects controlling for age and sex over 21 years of old
Supplementary Table S96. Cortical thickness differences between individuals with first episode major depressive disorder versus individuals with recurrent episode major depressive disorder controlling for age and sex
Supplementary Table S97. Cortical thickness differences between individuals with first episode major depressive disorder versus individuals with recurrent episode major depressive disorder controlling for age and sex over 21 years of old
Supplementary Table S98. Partial correlations between cortical thickness and number of episodes controlling for age and sex in individuals with major depressive disorder
Supplementary Table S99. Partial correlations between cortical thickness and number of episodes controlling for age and sex in individuals with major depressive disorder over 21 years of old
Supplementary Table S100. Cortical surface area differences between individuals with bipolar disorder type-1 compared to individuals with bipolar disorder type-2 controlling for age and sex
Supplementary Table S101. Cortical surface area differences between individuals with bipolar disorder type-1 compared to individuals with bipolar disorder type-2 controlling for age, sex, age × sex, age^2, age^2 × sex, and ICV in individuals with bipolar disorder at 25 years of age or older
Supplementary Table S102. Cortical surface area differences between individuals with bipolar disorder type-1 compared to individuals with bipolar disorder type-2 controlling for age and sex at 25 years of age or older
Supplementary Table S103. Cortical surface area differences between individuals with first episode major depressive disorder versus healthy comparison subjects controlling for age and sex
Supplementary Table S104. Cortical surface area differences between individuals with first episode major depressive disorder versus healthy comparison subjects controlling for age and sex over 21 years of old
Supplementary Table S105. Cortical surface area differences between individuals with recurrent episode major depressive disorder versus healthy comparison subjects controlling for age and sex
Supplementary Table S106. Cortical surface area differences between individuals with recurrent episode major depressive disorder versus healthy comparison subjects controlling for age and sex over 21 years of old
Supplementary Table S107. Cortical surface area differences between individuals with first episode major depressive disorder versus individuals with recurrent episode major depressive disorder controlling for age and sex
Supplementary Table S108. Cortical surface area differences between individuals with first episode major depressive disorder versus individuals with recurrent episode major depressive disorder controlling for age and sex over 21 years of old
Supplementary Table S109. Partial correlations between cortical surface area and number of episodes controlling for age and sex in individuals with major depressive disorder
Supplementary Table S110. Partial correlations between cortical surface area and number of episodes controlling for age and sex in individuals with major depressive disorder over 21 years of old
Supplementary Table S111. Cortical thickness differences between individuals with schizophrenia who are unmedicated with antipsychotics and healthy comparison subjects controlling for age and sex
Supplementary Table S112. Cortical thickness differences between individuals with schizophrenia on second-generation antipsychotic medications and healthy comparison subjects controlling for age and sex
Supplementary Table S113. Cortical thickness differences between individuals with schizophrenia on second-generation antipsychotic medications and individuals with schizophrenia who are unmedicated with antipsychotics controlling for age and sex
Supplementary Table S114. Cortical thickness differences between individuals with schizophrenia on first-generation antipsychotic medications and healthy comparison subjects controlling for age and sex
Supplementary Table S115. Cortical thickness differences between individuals with schizophrenia on first-generation antipsychotic medications and individuals with schizophrenia who are unmedicated with antipsychotics controlling for age and sex
Supplementary Table S116. Cortical thickness differences between individuals with schizophrenia on first-generation antipsychotic medications and individuals with schizophrenia on second-generation antipsychotic medications controlling for age and sex
Supplementary Table S117. Cortical thickness differences between individuals with schizophrenia on both first-generation and second-generation antipsychotic medications and healthy comparison subjects controlling for age and sex
Supplementary Table S118. Cortical thickness differences between individuals with schizophrenia on both first-generation and second-generation antipsychotic medications and individuals with schizophrenia who are unmedicated with antipsychotics controlling for age and sex
Supplementary Table S119. Cortical thickness differences between individuals with schizophrenia on both first-generation and second-generation antipsychotic medications and

individuals with schizophrenia on second-generation antipsychotic medications controlling for age and sex

Supplementary Table S120. Cortical thickness differences between individuals with schizophrenia on both first-generation and second-generation antipsychotic medications and individuals with schizophrenia on first-generation antipsychotic medications controlling for age and sex

Supplementary Table S121. Partial correlations between cortical thickness and chlorpromazine equivalents controlling for age and sex in individuals with schizophrenia who are medicated with antipsychotics

Supplementary Table S122. Cortical thickness differences between individuals with bipolar disorder taking lithium carbonate and individuals with bipolar disorder not taking lithium carbonate controlling for age, sex, and other medications

Supplementary Table S123. Cortical thickness differences between individuals with bipolar disorder taking lithium carbonate and individuals with bipolar disorder not taking lithium carbonate controlling for age, sex, and other medications at 25 years of age or older

Supplementary Table S124. Cortical thickness differences between individuals with bipolar disorder taking sodium valproate and individuals with bipolar disorder not taking sodium valproate controlling for age, sex, and other medications

Supplementary Table S125. Cortical thickness differences between individuals with bipolar disorder taking sodium valproate and individuals with bipolar disorder not taking sodium valproate controlling for age, sex, and other medications at 25 years of age or older

Supplementary Table S126. Cortical thickness differences between individuals with bipolar disorder taking second-generation antipsychotics and individuals with bipolar disorder not taking second-generation antipsychotics controlling for age, sex, and other medications

Supplementary Table S127. Cortical thickness differences between individuals with bipolar disorder taking second-generation antipsychotics and individuals with bipolar disorder not taking second-generation antipsychotics controlling for age, sex, and other medications at 25 years of age or older

Supplementary Table S128. Cortical thickness differences between individuals with bipolar disorder taking first-generation antipsychotics and individuals with bipolar disorder not taking first-generation antipsychotics controlling for age, sex, and other medications

Supplementary Table S129. Cortical thickness differences between individuals with bipolar disorder taking first-generation antipsychotics and individuals with bipolar disorder not taking first-generation antipsychotics controlling for age, sex, and other medications at 25 years of age or older

Supplementary Table S130. Cortical thickness differences between individuals with bipolar disorder taking antidepressants and individuals with bipolar disorder not taking antidepressants controlling for age, sex, and other medications

Supplementary Table S131. Cortical thickness differences between individuals with bipolar disorder taking antidepressants and individuals with bipolar disorder not taking antidepressants controlling for age, sex, and other medications at 25 years of age or older

Supplementary Table S132. Cortical thickness differences between individuals with major depressive disorder not taking antidepressants and healthy comparison subjects controlling for age and sex

Supplementary Table S133. Cortical thickness differences between individuals with major depressive disorder not taking antidepressants and healthy comparison subjects controlling for age and sex over 21 years of old

Supplementary Table S134. Cortical thickness differences between individuals with major depressive disorder taking antidepressants and healthy comparison subjects controlling for age and sex

Supplementary Table S135. Cortical thickness differences between individuals with major depressive disorder taking antidepressants and healthy comparison subjects controlling for age and sex over 21 years of old

Supplementary Table S136. Cortical thickness differences between individuals with major depressive disorder taking antidepressants and individuals with major depressive disorder not taking antidepressants controlling for age and sex

Supplementary Table S137. Cortical thickness differences between individuals with major depressive disorder taking antidepressants and individuals with major depressive disorder not taking antidepressants controlling for age and sex over 21 years of old

Supplementary Table S138. Cortical thickness differences between individuals with major depressive disorder not taking antipsychotics and healthy comparison subjects controlling for age and sex

Supplementary Table S139. Cortical thickness differences between individuals with major depressive disorder not taking antipsychotics and healthy comparison subjects controlling for age and sex over 21 years of old

Supplementary Table S140. Cortical thickness differences between individuals with major depressive disorder taking second-generation antipsychotics and healthy comparison subjects controlling for age and sex

Supplementary Table S141. Cortical thickness differences between individuals with major depressive disorder taking second-generation antipsychotics and healthy comparison subjects controlling for age and sex over 21 years of old

Supplementary Table S142. Cortical thickness differences between individuals with major depressive disorder taking second-generation antipsychotics and individuals with major depressive disorder not taking antipsychotics controlling for age and sex

Supplementary Table S143. Cortical thickness differences between individuals with major depressive disorder taking second-generation antipsychotics and individuals with major depressive disorder not taking antipsychotics controlling for age and sex over 21 years of old

Supplementary Table S144. Cortical thickness differences between individuals with major depressive disorder taking first-generation antipsychotics and healthy comparison subjects controlling for age and sex

Supplementary Table S145. Cortical thickness differences between individuals with major depressive disorder taking first-generation antipsychotics and healthy comparison subjects controlling for age and sex over 21 years of old

Supplementary Table S146. Cortical thickness differences between individuals with major depressive disorder taking first-generation antipsychotics and individuals with major depressive disorder not taking antipsychotics controlling for age and sex

Supplementary Table S147. Cortical thickness differences between individuals with major depressive disorder taking first-generation antipsychotics and individuals with major depressive disorder not taking antipsychotics controlling for age and sex over 21 years of old

Supplementary Table S148. Cortical surface area differences between individuals with schizophrenia who are unmedicated with antipsychotics and healthy comparison subjects controlling for age and sex

Supplementary Table S149. Cortical surface area differences between individuals with schizophrenia on second-generation antipsychotic medications and healthy comparison subjects controlling for age and sex

Supplementary Table S150. Cortical surface area differences between individuals with schizophrenia on second-generation antipsychotic medications and individuals with schizophrenia who are unmedicated with antipsychotics controlling for age and sex

Supplementary Table S151. Cortical surface area differences between individuals with schizophrenia on first-generation antipsychotic medications and healthy comparison subjects controlling for age and sex

Supplementary Table S152. Cortical surface area differences between individuals with schizophrenia on first-generation antipsychotic medications and individuals with schizophrenia who are unmedicated with antipsychotics controlling for age and sex

Supplementary Table S153. Cortical surface area differences between individuals with schizophrenia on first-generation antipsychotic medications and individuals with schizophrenia on second-generation antipsychotic medications controlling for age and sex

Supplementary Table S154. Cortical surface area differences between individuals with schizophrenia on both first-generation and second-generation antipsychotic medications and healthy comparison subjects controlling for age and sex

Supplementary Table S155. Cortical surface area differences between individuals with schizophrenia on both first-generation and second-generation antipsychotic medications and individuals with schizophrenia who are unmedicated with antipsychotics controlling for age and sex

Supplementary Table S156. Cortical surface area differences between individuals with schizophrenia on both first-generation and second-generation antipsychotic medications and individuals with schizophrenia on second-generation antipsychotic medications controlling for age and sex

Supplementary Table S157. Cortical surface area differences between individuals with schizophrenia on both first-generation and second-generation antipsychotic medications and individuals with schizophrenia on first-generation antipsychotic medications controlling for age and sex

Supplementary Table S158. Partial correlations between cortical surface area and chlorpromazine equivalents controlling for age and sex in individuals with schizophrenia who are medicated with antipsychotics

Supplementary Table S159. Cortical surface area differences between individuals with bipolar disorder taking lithium carbonate and individuals with bipolar disorder not taking lithium carbonate controlling for age, sex, and other medications

Supplementary Table S160. Cortical surface area differences between individuals with bipolar disorder taking lithium carbonate and individuals with bipolar disorder not taking lithium carbonate controlling for age, sex, age × sex, age^2, age^2 × sex, ICV, and other medications at 25 years of age or older

Supplementary Table S161. Cortical surface area differences between individuals with bipolar disorder taking lithium carbonate and individuals with bipolar disorder not taking lithium carbonate controlling for age, sex, and other medications at 25 years of age or older

Supplementary Table S162. Cortical surface area differences between individuals with bipolar disorder taking sodium valproate and individuals with bipolar disorder not taking sodium valproate controlling for age, sex, and other medications

Supplementary Table S163. Cortical surface area differences between individuals with bipolar disorder taking sodium valproate and individuals with bipolar disorder not taking sodium valproate controlling for age, sex, age × sex, age^2, age^2 × sex, ICV, and other medications at 25 years of age or older

Supplementary Table S164. Cortical surface area differences between individuals with bipolar disorder taking sodium valproate and individuals with bipolar disorder not taking sodium valproate controlling for age, sex, and other medications at 25 years of age or older

Supplementary Table S165. Cortical surface area differences between individuals with bipolar disorder taking second-generation antipsychotics and individuals with bipolar disorder

not taking second-generation antipsychotics controlling for age, sex, and other medications
Supplementary Table S166. Cortical surface area differences between individuals with bipolar disorder taking second-generation antipsychotics and individuals with bipolar disorder not taking second-generation antipsychotics controlling for age, sex, age × sex, age^2, age^2 × sex, ICV, and other medications at 25 years of age or older
Supplementary Table S167. Cortical surface area differences between individuals with bipolar disorder taking second-generation antipsychotics and individuals with bipolar disorder not taking second-generation antipsychotics controlling for age, sex, and other medications at 25 years of age or older
Supplementary Table S168. Cortical surface area differences between individuals with bipolar disorder taking first-generation antipsychotics and individuals with bipolar disorder not taking first-generation antipsychotics controlling for age, sex, and other medications
Supplementary Table S169. Cortical surface area differences between individuals with bipolar disorder taking first-generation antipsychotics and individuals with bipolar disorder not taking first-generation antipsychotics controlling for age, sex, age × sex, age^2, age^2 × sex, ICV, and other medications at 25 years of age or older
Supplementary Table S170. Cortical surface area differences between individuals with bipolar disorder taking first-generation antipsychotics and individuals with bipolar disorder not taking first-generation antipsychotics controlling for age, sex, and other medications at 25 years of age or older
Supplementary Table S171. Cortical surface area differences between individuals with bipolar disorder taking antidepressants and individuals with bipolar disorder not taking antidepressants controlling for age, sex, and other medications
Supplementary Table S172. Cortical surface area differences between individuals with bipolar disorder taking antidepressants and individuals with bipolar disorder not taking antidepressants controlling for age, sex, age × sex, age^2, age^2 × sex, ICV, and other medications at 25 years of age or older
Supplementary Table S173. Cortical surface area differences between individuals with bipolar disorder taking antidepressants and individuals with bipolar disorder not taking antidepressants controlling for age, sex, and other medications at 25 years of age or older
Supplementary Table S174. Cortical surface area differences between individuals with major depressive disorder not taking antidepressants and healthy comparison subjects controlling for age and sex
Supplementary Table S175. Cortical surface area differences between individuals with major depressive disorder not taking antidepressants and healthy comparison subjects controlling for age and sex over 21 years of old
Supplementary Table S176. Cortical surface area differences between individuals with major depressive disorder taking antidepressants and healthy comparison subjects controlling for age and sex
Supplementary Table S177. Cortical surface area differences between individuals with major depressive disorder taking antidepressants and healthy comparison subjects controlling for age and sex over 21 years of old
Supplementary Table S178. Cortical surface area differences between individuals with major depressive disorder taking antidepressants and individuals with major depressive disorder not taking antidepressants controlling for age and sex
Supplementary Table S179. Cortical surface area differences between individuals with major depressive disorder taking antidepressants and individuals with major depressive disorder not taking antidepressants controlling for age and sex over 21 years of old
Supplementary Table S180. Cortical surface area differences between individuals with major depressive disorder not taking antipsychotics and healthy comparison subjects controlling for age and sex
Supplementary Table S181. Cortical surface area differences between individuals with major depressive disorder not taking antipsychotics and healthy comparison subjects controlling for age and sex over 21 years of old
Supplementary Table S182. Cortical surface area differences between individuals with major depressive disorder taking second-generation antipsychotics and healthy comparison subjects controlling for age and sex
Supplementary Table S183. Cortical surface area differences between individuals with major depressive disorder taking second-generation antipsychotics and healthy comparison subjects controlling for age and sex over 21 years of old
Supplementary Table S184. Cortical surface area differences between individuals with major depressive disorder taking second-generation antipsychotics and individuals with major depressive disorder not taking antipsychotics controlling for age and sex
Supplementary Table S185. Cortical surface area differences between individuals with major depressive disorder taking second-generation antipsychotics and individuals with major depressive disorder not taking antipsychotics controlling for age and sex over 21 years of old
Supplementary Table S186. Cortical surface area differences between individuals with major depressive disorder taking first-generation antipsychotics and healthy comparison subjects controlling for age and sex
Supplementary Table S187. Cortical surface area differences between individuals with major depressive disorder taking first-generation antipsychotics and healthy comparison subjects controlling for age and sex over 21 years of old
Supplementary Table S188. Cortical surface area differences between individuals with major depressive disorder taking first-generation antipsychotics and individuals with major depressive disorder not taking antipsychotics controlling for age and sex
Supplementary Table S189. Cortical surface differences between individuals with major depressive disorder taking first-generation antipsychotics and individuals with major depressive disorder not taking antipsychotics controlling for age and sex over 21 years of old
Supplementary Table S190. Partial correlations between cortical thickness and PANSS total controlling for age and sex in individuals with schizophrenia
Supplementary Table S191. Partial correlations between cortical thickness and PANSS positive controlling for age and sex in individuals with schizophrenia
Supplementary Table S192. Partial correlations between cortical thickness and PANSS negative controlling for age and sex in individuals with schizophrenia
Supplementary Table S193. Partial correlations between cortical thickness and PANSS general controlling for age and sex in individuals with schizophrenia
Supplementary Table S194. Partial correlations between cortical thickness and severity of symptoms measured by the HDRS-17 controlling for age and sex in individuals with major depressive disorder
Supplementary Table S195. Partial correlations between cortical thickness and severity of symptoms measured by the HDRS-17 controlling for age and sex in individuals with major depressive disorder over 21 years of old
Supplementary Table S196. Partial correlations between cortical thickness and severity of symptoms measured by the BDI-II controlling for age and sex in individuals with major depressive disorder
Supplementary Table S197. Partial correlations between cortical thickness and severity of symptoms measured by the BDI-II controlling for age and sex in individuals with major depressive disorder over 21 years of old
Supplementary Table S198. Partial correlations between cortical surface area and PANSS total controlling for age and sex in individuals with schizophrenia
Supplementary Table S199. Partial correlations between cortical surface area and PANSS positive controlling for age and sex in individuals with schizophrenia
Supplementary Table S200. Partial correlations between cortical surface area and PANSS negative controlling for age and sex in individuals with schizophrenia
Supplementary Table S201. Partial correlations between cortical surface area and PANSS general controlling for age and sex in individuals with schizophrenia
Supplementary Table S202. Partial correlations between cortical surface area and severity of symptoms measured by the HDRS-17 controlling for age and sex in individuals with major depressive disorder
Supplementary Table S203. Partial correlations between cortical surface area and severity of symptoms measured by the HDRS-17 controlling for age and sex in individuals with major depressive disorder over 21 years of old
Supplementary Table S204. Partial correlations between cortical surface area and severity of symptoms measured by the BDI-II controlling for age and sex in individuals with major depressive disorder
Supplementary Table S205. Partial correlations between cortical surface area and severity of symptoms measured by the BDI-II controlling for age and sex in individuals with major depressive disorder over 21 years of old
Supplementary Table S206. Demographic characteristics used in the analyses of individuals with autism spectrum disorder and healthy comparison subjects controlling for IQ as an additional covariate
Supplementary Table S207. Cortical thickness differences between individuals with autism spectrum disorder and healthy comparison subjects controlling for age, sex, and IQ
Supplementary Table S208. Cortical thickness differences between individuals with autism spectrum disorder and healthy comparison subjects controlling for age, sex, global mean cortical thickness, and IQ
Supplementary Table S209. Cortical surface area differences between individuals with autism spectrum disorder and healthy comparison subjects controlling for age, sex, and IQ
Supplementary Table S210. Cortical surface area differences between individuals with autism spectrum disorder and healthy comparison subjects controlling for age, sex, total cortical surface area, and IQ
Supplementary Table S211. Cortical volume differences between individuals with autism spectrum disorder and healthy comparison subjects controlling for age, sex, and IQ
Supplementary Table S212. Cortical volume differences between individuals with autism spectrum disorder and healthy comparison subjects controlling for age, sex, ICV, and IQ
Abbreviations: ASD, autism spectrum disorder; BD, bipolar disorder; BDI-II, Beck Depression Inventory-II; FDR, false-discovery rate; HC, healthy comparison subjects; HDRS-17, 17-item Hamilton Depression Rating Scale; ICV, intracranial volume; IQ, intelligence quotient as determined by the Wechsler Adult Intelligence Scale; MDD, major depressive disorder; PANSS, Positive and Negative Syndrome Scale; SZ, schizophrenia.

Supplementary Table S1. Demographic characteristics of the included individuals

		Healthy comparison subjects					Individuals with schizophrenia				
					Age (range: 15-77)					Age (range: 12-78)	
Protocol name	Total *N*	*N*	Male	Female	Mean	s.d.	*N*	Male	Female	Mean	s.d.
Total	4,144	2,718	1387	1331	35.4	13.6	1,426	717	709	36.7	12.6
Osaka1	552	410	191	219	35.5	12.5	142	84	58	37.0	12.9
Osaka2	310	236	130	106	31.3	13.2	74	34	40	33.0	12.3
Osaka3	640	549	283	266	34.4	16.5	91	46	45	34.1	12.4
Tokyo1	318	225	136	89	34.1	11.2	93	53	40	33.4	9.6
Tokyo2	124	83	57	26	28.7	5.5	41	26	15	31.5	9.1
Tokyo3	54	39	25	14	27.5	4.8	15	8	7	30.5	12.7
Tokyo4	55	45	17	28	38.8	9.2	10	6	4	38.4	4.4
Tokyo5	70	43	17	26	37.6	7.6	27	19	8	30.6	10.5
NipponMedical2	408	193	41	152	48.2	8.9	215	122	93	44.3	13.6
Kyoto1	183	111	63	48	31.9	10.6	72	38	34	36.1	8.9
Kyoto2	172	127	83	44	37.2	11.8	45	25	20	40.7	9.4
Toyama1	221	117	63	54	25.9	6.3	104	53	51	26.6	6.3
Toyama2	127	56	31	25	25.9	3.4	71	33	38	28.2	9.0
Kanazawa1	212	109	67	42	35.4	11.7	103	39	64	41.0	12.6
Nagoya1	172	118	72	46	36.7	9.9	54	30	24	43.3	10.0
Nagoya3	30	13	8	5	66.8	4.3	17	8	9	41.2	11.3
Hokkaido1	139	31	13	18	48.7	13.4	108	44	64	34.8	12.3
Hokkaido2	58	25	15	10	34.8	8.1	33	11	22	37.9	9.5
Kyushu1	119	78	36	42	33.2	11.9	41	11	30	38.2	9.6
Kyushu2	58	27	11	16	34.6	13.8	31	15	16	35.5	11.1
Yamaguchi1	88	67	14	53	47.8	16.3	21	4	17	55.7	7.5
Tokushima2	17	5	5	0	39.0	9.2	12	7	5	45.3	9.5
UOEH1	17	11	9	2	35.0	13.3	6	1	5	22.3	9.7

		Healthy comparison subjects					Individuals with bipolar disorder				
					Age (range: 19-80)					Age (range: 14-84)	
Protocol name	Total *N*	*N*	Male	Female	Mean	s.d.	*N*	Male	Female	Mean	s.d.
Total	874	637	304	333	40.7	15.2	237	118	119	45.9	15.0
Yamaguchi1	75	67	14	53	47.8	16.3	8	0	8	50.3	11.2
Yamaguchi2	127	112	46	66	44.3	19.0	15	9	6	40.9	13.6
Kanazawa1	143	109	67	42	35.4	11.7	34	18	16	45.6	15.0
Kyushu1	96	78	36	42	33.2	11.9	18	7	11	48.4	14.7
Kyushu2	36	27	11	16	34.6	13.8	9	5	4	48.8	7.9
[*]Kyushu3	7	0	0	0	n/a	n/a	7	5	2	48.7	10.5
Nagoya1	137	118	72	46	36.7	9.9	19	8	11	49.0	14.0
Hokkaido1	109	31	13	18	48.7	13.4	78	41	37	46.0	16.3
Hiroshima5	78	52	28	24	55.8	14.3	26	10	16	52.1	16.1
Tokyo5	66	43	17	26	37.6	7.6	23	15	8	34.4	9.5

		Healthy comparison subjects					Individuals with major depressive disorder				
					Age (range: 15-80)					Age (range: 14-84)	
Protocol name	Total *N*	*N*	Male	Female	Mean	s.d.	*N*	Male	Female	Mean	s.d.
Total	2,430	1,818	882	936	36.6	15.3	612	314	298	45.2	14.6
Osaka1	425	410	191	219	35.5	12.5	15	9	6	30.7	15.5
Osaka2	245	236	130	106	31.3	13.2	9	4	5	58.3	19.4
Osaka3	568	549	283	266	34.4	16.5	19	9	10	50.4	15.2
Hiroshima1	126	57	28	29	33.8	13.0	69	35	34	42.3	11.6
[†]Hiroshima2	42	0	0	0	n/a	n/a	42	31	11	38.9	8.2
Hiroshima3	78	29	12	17	44.0	8.9	49	27	22	44.3	10.6
Hiroshima4	42	34	8	26	42.4	12.4	8	1	7	37.5	9.4
Hiroshima5	111	52	28	24	55.8	14.3	59	23	36	49.5	14.3
Yamaguchi1	85	67	14	53	47.8	16.3	18	9	9	47.8	11.3
Yamaguchi2	167	112	46	66	44.3	19.0	55	24	31	51.6	12.7
Hokkaido1	200	31	13	18	48.7	13.4	169	84	85	47.2	16.9
Kanazawa1	151	109	67	42	35.4	11.7	42	26	16	42.9	13.8
Kyushu1	88	78	36	42	33.2	11.9	10	6	4	49.2	11.0
Tokyo5	86	43	17	26	37.6	7.6	43	24	19	37.7	11.3
UOEH1	16	11	9	2	35.0	13.3	5	2	3	36.0	12.8

		Healthy comparison subjects					Individuals with autism spectrum disorder				
					Age (range: 16-76)					Age (range: 11-55)	
Protocol name	Total *N*	*N*	Male	Female	Mean	s.d.	*N*	Male	Female	Mean	s.d.
Total	1,750	1,544	828	716	33.7	13.6	206	167	39	28.6	8.7
Osaka1	450	410	191	219	35.5	12.5	40	27	13	26.1	10.3
Osaka2	253	236	130	106	31.3	13.2	17	12	5	24.6	9.0
Osaka3	577	549	283	266	34.4	16.5	28	16	12	26.1	10.2
Tokyo2	110	83	57	26	28.7	5.5	27	27	0	29.9	6.7
Tokyo3	49	39	25	14	27.5	4.8	10	10	0	32.0	8.4
Tokyo5	49	43	17	26	37.6	7.6	6	6	0	36.8	8.4
Nagoya1	130	118	72	46	36.7	9.9	12	12	0	30.7	9.5
Showa1	132	66	53	13	27.0	6.0	66	57	9	30.1	6.5

[*]Kyushu3 is not used for case–control analysis because of the absence of healthy subjects, and is used only for analysis of associations with duration of illness.

[†]Hiroshima2 is not used for case–control analysis because of the absence of healthy subjects, and is used only for analysis of associations with severity, the number of episodes, and duration of illness in up to 42 subjects. 42 patients are included in analyses related to the Beck Depression Inventory (BDI-II), 28 patients are included in analyses related to the Hamilton Depression Rating Scale (HDRS-17), 25 patients are included in analyses related to the number of episodes in recurrent patients, and 41 patients are included in analyses related to the duration of illness.

Supplementary Table S2. Clinical characteristics of individuals with schizophrenia

	Onset Age			Duration of Illness			Antipsychotics								PANSS								
							HC	Non	Second	First	Both	Chlorpromazine Equivalent				Total		Possitive		Negative		General	
Protocol name	*N*	Mean	s.d.	*N*	Mean	s.d.	*N*					*N*	Mean	s.d.	*N*	Mean	s.d.	Mean	s.d.	Mean	s.d.	Mean	s.d.
Total	1,145	24.5	9.0	1,151	10.7	9.7	2,496	84	625	121	226	1,039	649.2	553.5	650	72.8	23.2	16.9	6.3	18.7	6.7	37.3	12.5
Hokkaido1	108	27.2	10.7	108	7.5	9.5	31	22	59		23	86	773.9	629.2									
Hokkaido2	33	24.3	8.0	33	13.5	8.7	25		16			29	870.6	1000.9	31	84.2	19.9	19.1	6.1	21.1	6.0	44.0	9.8
Kanazawa1	103	26.5	9.1	103	14.4	11.9	109	6	71		25	97	572.5	535.1	103	61.6	17.4	15.1	6.2	17.3	6.7	29.3	8.2
Kyoto1	70	24.1	6.9	70	12.3	8.2	111		53	6	13	72	561.1	371.5	69	59.1	16.6	13.7	4.5	15.2	5.3	30.2	9.7
Kyoto2	45	25.4	7.4	45	15.5	9.4	127		20		6	27	665.8	458.1	44	53.3	16.2	12.5	4.8	14.3	5.9	26.5	7.3
Kyushu1	26	25.1	8.0	26	13.5	7.6	78		13		11	25	673.5	410.2									
Kyushu2	25	26.5	9.2	25	9.0	7.6	27		19			24	576.9	491.5									
Nagoya1	54	24.1	6.7	54	19.2	11.0	118		28	5	10	54	610.5	422.7									
Osaka1	142	24.9	9.8	142	11.5	9.2	410	14	84	7	37	128	597.7	503.7	138	79.0	20.6	18.4	5.8	19.1	6.0	41.4	11.2
Osaka2	74	22.8	10.7	74	10.1	8.4	236	8	55		9	66	812.6	621.5	71	86.8	23.7	19.7	6.2	21.0	6.1	46.1	12.3
Osaka3	91	22.1	9.3	91	12.0	10.1	549	17	58		15	74	566.6	495.2	89	91.9	16.0	21.1	5.2	23.1	4.2	47.8	8.1
Tokushima2	9	28.6	5.7	9	17.8	11.0						9	379.2	348.2									
Tokyo1	91	23.0	7.2	91	10.5	8.0	225		14	52	25	91	810.7	602.7									
Tokyo2	41	22.1	8.1	41	9.5	6.9	83		20	6	14	40	889.3	750.7									
Tokyo3	12	23.4	11.8	12	7.1	8.6	39		11			12	482.3	446.5									
Tokyo4	10	28.7	9.3	10	9.7	5.9	45		5		5	10	754.1	503.7									
Tokyo5	16	21.6	4.9	16	10.3	10.0	43		13			19	715.4	577.4	9	73.4	10.4	17.1	2.8	20.9	5.8	35.4	6.5
Toyama1	104	22.3	4.7	104	4.0	5.4	117	6	36	39	23	98	563.6	454.5									
Toyama2	71	23.2	7.9	71	4.7	6.0	56	11	50		10	60	529.7	511.0	69	67.9	20.8	15.1	6.0	18.3	7.7	34.5	11.2
UOEH1				6	0.06	0.14						5	347.4	197.0	6	71.8	21.5	15.0	2.5	20.7	7.9	36.2	12.2
Yamaguchi1	20	39.4	15.3	20	17.0	13.4	67			6		13	358.7	387.4	21	43.8	15.4	9.6	3.2	12.3	7.1	21.9	6.0

Supplementary Table S3. Clinical characteristics of individuals with bipolar disorder

	Duration of Illness						Diagnosis Type				Lithium carbonate				Valproate				Antipsychotics								Antidepressants			
	All age			Over 25			All age		Over 25		All age		Over 25		All age		Over 25		All age		Over 25		All age		Over 25		All age		Over 25	
Protocol name	*N*	Mean	s.d.	*N*	Mean	s.d.	Type-1	Type-2	Type-1	Type-2	Lithium	Not taking Lithium	Lithium	Not taking Lithium	Valproate	Not taking Valproate	Valproate	Not taking Valproate	Second	Not taking Second	Second	Not taking Second	First	Not taking First	First	Not taking First	Antidepre ssants	Not taking Antidressa nts	Antidepre ssants	Not taking Antidressa nts
Total	232	13.0	10.8	214	13.8	10.9	50	56	44	46	90	116	84	105	43	104	38	96	106	100	97	92	14	77	14	66	81	106	76	94
Hiroshima5	26	18.0	11.2	25	18.6	11.1	17	9	16	9	13	13	13	12					12	14	11	14					12	14	12	13
Hokkaido1	76	11.1	11.1	67	12.3	11.3					30	43	28	36	26	47	21	43	39	34	34	30	7	66	7	57	32	41	29	35
Kanazawa1	34	11.6	10.4	32	12.2	10.4	9	25	9	23	13	21	12	20	6	28	6	26	19	15	18	14					13	21	12	20
Kyushu1	18	15.6	15.3	16	17.1	15.6					7	11	6	10					8	10	7	9	7	11	7	9	6	12	6	10
Kyushu2	9	14.0	8.6	9	14.0	8.6																								
Kyushu3	7	12.3	4.8	7	12.3	4.8																								
Nagoya1	19	14.6	9.0	19	14.6	9.0	14	5	14	5	10	9	10	9	5	14	5	14	14	5	14	5								
Tokyo5	20	8.9	7.6	17	9.9	7.8	5	7			10	11	9	10	6	15	6	13	8	13	7	12					12	9	11	8
Yamaguchi1	8	16.1	11.0	8	16.1	11.0																								
Yamaguchi2	15	14.5	10.5	14	15.3	10.5	5	10	5	9	7	8	6	8					6	9	6	8					6	9	6	8

Supplementary Table S4. Clinical characteristics of individuals with major depressive disorder

	Duration of Illness						Onset age			First or Reccurent					
	All age			Over 21			All age			All age			Over 21		
Protocol name	*N*	Mean	s.d.	*N*	Mean	s.d.	Late onset	Early onset	HC	First	Recurrent	HC	First	Recurrent	HC
Total	577	6.7	7.6	558	6.8	7.7	470	31	1,571	207	312	578	197	308	548
Hiroshima1	68	4.5	6.7	68	4.5	6.7	61	7	52	36	33	57	36	33	52
Hiroshima2	41	4.3	4.3	41	4.3	4.3				16	25		16	25	
Hiroshima3	23	5.4	6.9	23	5.4	6.9	20		29	9	14	29	9	14	29
Hiroshima4	8	6.5	9.7	8	6.5	9.7	8		34						
Hiroshima5	59	8.8	9.7	59	8.8	9.7	56		51	25	34	52	25	34	51
Hokkaido1	169	5.5	6.5	160	5.7	6.6	151	9	31	76	91	31	70	88	31
Kanazawa1	42	8.0	8.8	40	8.1	9.0	35	5	108	16	26	109	15	25	108
Kyushu1	10	8.9	3.8	10	8.9	3.8	10		74		10	78		10	74
Osaka1	15	2.4	4.3	10	3.0	5.2	8		374						
Osaka2	9	13.6	10.8	9	13.6	10.8	7		188						
Osaka3	19	7.1	6.7	19	7.1	6.7	19		427						
Tokyo5	41	8.4	7.0	38	8.9	7.0	28	10	43	11	28	43	8	28	43
UOEH1															
Yamaguchi1	18	6.5	7.5	18	6.5	7.5	16		64		14	67		14	64
Yamaguchi2	55	10.0	9.1	55	10.0	9.1	51		96	18	37	112	18	37	96

	Number of Episodes						Antidepressants					
	All age			Over 21			All age			Over 21		
Protocol name	*N*	Mean	s.d.	*N*	Mean	s.d.	HC	Not taking anti-depressants	Anti-depressants	HC	Not taking antidepressants	Anti-depressants
Total	249	3.3	3.1	246	3.3	3.1	1,807	72	481	1,571	62	471
Hiroshima1	32	2.1	0.5	32	2.1	0.5	57	6	63	52	6	63
Hiroshima2	25	2.4	0.7	25	2.4	0.7						
Hiroshima3	14	3.1	2.1	14	3.1	2.1	29	7	42	29	7	42
Hiroshima4							34		7	34		7
Hiroshima5	33	5.2	4.8	33	5.2	4.8	52		55	51		55
Hokkaido1	68	2.5	0.8	65	2.6	0.8	31	36	133	31	33	127
Kanazawa1							109	5	37	108	5	35
Kyushu1	7	3.4	1.4	7	3.4	1.4	78		9	74		9
Osaka1							410	5	10	374		9
Osaka2							236		9	188		9
Osaka3							549		16	427		16
Tokyo5	20	3.3	1.2	20	3.3	1.2	43	13	30	43	11	29
UOEH1												
Yamaguchi1	14	3.8	3.6	14	3.8	3.6	67		18	64		18
Yamaguchi2	36	4.4	5.6	36	4.4	5.6	112		52	96		52

	Antipsychotics							
	All age				Over 21			
Protocol name	HC	Not taking anti-psychotics	Second	First	HC	Not taking anti-psychotics	Second	First
Total	1,818	447	78	27	1,571	428	74	27
Hiroshima1	57	64			52	64		
Hiroshima2		23		5		23		5
Hiroshima3	29	39	7		29	39	7	
Hiroshima4	34	5			34	5		
Hiroshima5	52	53			51	53		
Hokkaido1	31	107	41	17	31	100	39	17
Kanazawa1	109	28	13		108	27	12	
Kyushu1	78	5			74	5		
Osaka1	410	12			374	8		
Osaka2	236	8			188	8		
Osaka3	549	13			427	13		
Tokyo5	43	33	7		43	31	6	
UOEH1	11	5						
Yamaguchi1	67	13			64	13		
Yamaguchi2	112	39	10	5	96	39	10	5

	17-item Hamilton Depression Rating Scale						Beck Depression Inventory II					
	All age			Over 21			All age			Over 21		
Protocol name	*N*	Mean	s.d.	*N*	Mean	s.d.	*N*	Mean	s.d.	*N*	Mean	s.d.
Total	425	15.6	7.2	417	15.6	7.2	319	26.5	11.5	317	26.5	11.4
Hiroshima1	69	19.9	5.2	69	19.9	5.2	69	30.2	9.2	69	30.2	9.2
Hiroshima2	28	13.4	4.6	28	13.4	4.6	42	22.0	8.4	42	22.0	8.4
Hiroshima3	49	14.5	4.3	49	14.5	4.3	49	28.9	9.8	49	28.9	9.8
Hiroshima4	8	22.9	6.0	8	22.9	6.0	8	37.4	9.0	8	37.4	9.0
Hiroshima5	59	11.7	8.0	59	11.7	8.0	59	19.3	12.3	59	19.3	12.3
Hokkaido1	14	17.4	11.2	13	18.0	11.4						
Kanazawa1	42	13.1	6.9	40	13.3	6.9						
Kyushu1	7	10.4	5.5	7	10.4	5.5						
Osaka1	9	13.4	6.6	7	14.0	6.5						
Osaka2	9	12.1	11.3	9	12.1	11.3						
Osaka3	15	13.7	6.7	15	13.7	6.7						
Tokyo5	43	11.5	6.4	40	11.3	6.1	20	23.9	11.7	18	23.4	10.7
UOEH1												
Yamaguchi1	18	20.2	4.0	18	20.2	4.0	18	27.4	13.6	18	27.4	13.6
Yamaguchi2	55	20.2	5.0	55	20.2	5.0	54	30.2	11.2	54	30.2	11.2

Supplementary Table S5. Cortical thickness differences between individuals with schizophrenia and healthy comparison subjects controlling for age and sex

	d	Std. Err.	95% CI	*p*-value	FDR *q*-value
Global mean cortical thickness	-0.479	0.048	[-0.573 - -0.384]	3.270E-23	3.317E-22
Left hemisphere	-0.478	0.050	[-0.575 - -0.380]	7.965E-22	5.141E-21
Right hemisphere	-0.466	0.047	[-0.558 - -0.373]	5.983E-23	5.310E-22
Left fusiform gyrus	-0.481	0.048	[-0.576 - -0.387]	2.022E-23	2.393E-22
Right fusiform gyrus	-0.500	0.053	[-0.604 - -0.395]	7.834E-21	4.278E-20
Left pars opercularis of inferior frontal gyrus	-0.481	0.057	[-0.593 - -0.369]	3.245E-17	1.536E-16
Right pars opercularis of inferior frontal gyrus	-0.449	0.043	[-0.534 - -0.364]	3.839E-25	6.192E-24
Left superior temporal gyrus	-0.468	0.049	[-0.564 - -0.372]	9.014E-22	5.333E-21
Right superior temporal gyrus	-0.465	0.048	[-0.559 - -0.371]	4.821E-22	3.804E-21
Left insula	-0.464	0.035	[-0.533 - -0.396]	4.350E-40	3.088E-38
Right insula	-0.407	0.042	[-0.489 - -0.324]	7.584E-22	5.141E-21
Left lingual gyrus	-0.455	0.044	[-0.541 - -0.369]	3.723E-25	6.192E-24
Right lingual gyrus	-0.447	0.035	[-0.516 - -0.379]	2.430E-37	8.626E-36
Left pars triangularis of inferior frontal gyrus	-0.449	0.065	[-0.576 - -0.323]	3.757E-12	9.527E-12
Right pars triangularis of inferior frontal gyrus	-0.401	0.048	[-0.495 - -0.307]	5.092E-17	2.259E-16
Left lateral orbitofrontal cortex	-0.445	0.065	[-0.573 - -0.317]	1.012E-11	2.394E-11
Right lateral orbitofrontal cortex	-0.426	0.050	[-0.524 - -0.327]	2.702E-17	1.370E-16
Left rostral middle frontal gyrus	-0.445	0.070	[-0.581 - -0.308]	1.745E-10	3.441E-10
Right rostral middle frontal gyrus	-0.375	0.067	[-0.506 - -0.243]	2.199E-08	3.632E-08
Left middle temporal gyrus	-0.399	0.053	[-0.503 - -0.295]	5.588E-14	1.803E-13
Right middle temporal gyrus	-0.410	0.062	[-0.532 - -0.288]	4.898E-11	1.122E-10
Left superior frontal gyrus	-0.404	0.057	[-0.517 - -0.292]	1.996E-12	5.249E-12
Right superior frontal gyrus	-0.371	0.057	[-0.483 - -0.259]	8.601E-11	1.908E-10
Left pars orbitalis of inferior frontal gyrus	-0.379	0.062	[-0.501 - -0.257]	1.230E-09	2.298E-09
Right pars orbitalis of inferior frontal gyrus	-0.381	0.050	[-0.479 - -0.283]	2.514E-14	8.499E-14
Left medial orbitofrontal cortex	-0.362	0.061	[-0.481 - -0.242]	3.219E-09	5.860E-09
Right medial orbitofrontal cortex	-0.378	0.059	[-0.493 - -0.262]	1.377E-10	2.875E-10
Left inferior temporal gyrus	-0.361	0.035	[-0.430 - -0.293]	4.361E-25	6.192E-24
Right inferior temporal gyrus	-0.352	0.043	[-0.436 - -0.267]	2.974E-16	1.242E-15
Left isthmus cingulate cortex	-0.340	0.050	[-0.437 - -0.243]	6.782E-12	1.661E-11
Right isthmus cingulate cortex	-0.334	0.042	[-0.416 - -0.252]	1.178E-15	4.645E-15
Left banks of superior temporal sulcus	-0.332	0.046	[-0.423 - -0.242]	5.113E-13	1.452E-12
Right banks of superior temporal sulcus	-0.314	0.055	[-0.422 - -0.205]	1.533E-08	2.655E-08
Left supramarginal gyrus	-0.332	0.057	[-0.443 - -0.221]	4.099E-09	7.277E-09
Right supramarginal gyrus	-0.277	0.050	[-0.375 - -0.179]	3.459E-08	5.457E-08
Left caudal middle frontal gyrus	-0.312	0.043	[-0.396 - -0.229]	2.277E-13	6.735E-13
Right caudal middle frontal gyrus	-0.322	0.042	[-0.404 - -0.240]	1.188E-14	4.440E-14
Left frontal pole	-0.295	0.056	[-0.404 - -0.185]	1.332E-07	1.970E-07
Right frontal pole	-0.320	0.061	[-0.440 - -0.200]	1.625E-07	2.307E-07
Left posterior cingulate cortex	-0.274	0.037	[-0.346 - -0.202]	8.702E-14	2.686E-13
Right posterior cingulate cortex	-0.316	0.060	[-0.434 - -0.199]	1.359E-07	1.970E-07
Left lateral occipital cortex	-0.237	0.043	[-0.320 - -0.153]	2.791E-08	4.504E-08
Right lateral occipital cortex	-0.268	0.035	[-0.336 - -0.199]	1.479E-14	5.249E-14
Left precentral gyrus	-0.256	0.056	[-0.367 - -0.145]	5.888E-06	7.888E-06
Right precentral gyrus	-0.260	0.047	[-0.352 - -0.168]	3.545E-08	5.471E-08
Left parahippocampal gyrus	-0.248	0.047	[-0.340 - -0.156]	1.230E-07	1.858E-07
Right parahippocampal gyrus	-0.251	0.041	[-0.331 - -0.170]	1.073E-09	2.060E-09
Left inferior parietal cortex	-0.251	0.039	[-0.327 - -0.174]	1.542E-10	3.128E-10
Right inferior parietal cortex	-0.197	0.043	[-0.282 - -0.112]	5.343E-06	7.295E-06
Left transverse temporal gyrus	-0.249	0.035	[-0.318 - -0.181]	7.256E-13	1.981E-12
Right transverse temporal gyrus	-0.250	0.044	[-0.337 - -0.163]	1.932E-08	3.266E-08
Left postcentral gyrus	-0.189	0.046	[-0.279 - -0.099]	3.756E-05	4.763E-05
Right postcentral gyrus	-0.223	0.035	[-0.292 - -0.155]	1.316E-10	2.831E-10
Left precuneus	-0.134	0.036	[-0.206 - -0.063]	2.255E-04	2.761E-04
Right precuneus	-0.182	0.049	[-0.279 - -0.086]	2.189E-04	2.727E-04
Left caudal anterior cingulate cortex	-0.142	0.050	[-0.241 - -0.044]	4.589E-03	5.523E-03
Right caudal anterior cingulate cortex	-0.163	0.035	[-0.232 - -0.095]	2.502E-06	3.483E-06
Left cuneus	-0.147	0.035	[-0.215 - -0.079]	2.306E-05	2.976E-05
Right cuneus	-0.151	0.035	[-0.219 - -0.083]	1.364E-05	1.793E-05
Left rostral anterior cingulate cortex	-0.134	0.070	[-0.272 - 0.004]	5.655E-02	6.177E-02
Right rostral anterior cingulate cortex	-0.075	0.046	[-0.165 - 0.014]	9.998E-02	1.076E-01
Left pericalcarine cortex	-0.110	0.044	[-0.197 - -0.023]	1.338E-02	1.584E-02
Right pericalcarine cortex	-0.078	0.036	[-0.149 - -0.008]	2.879E-02	3.291E-02
Left paracentral lobule	-0.068	0.047	[-0.160 - 0.023]	1.433E-01	1.497E-01
Right paracentral lobule	-0.106	0.049	[-0.201 - -0.011]	2.920E-02	3.291E-02
Left superior parietal cortex	-0.088	0.045	[-0.177 - 0.001]	5.233E-02	5.805E-02
Right superior parietal cortex	-0.079	0.036	[-0.149 - -0.009]	2.739E-02	3.188E-02
Left temporal pole	-0.081	0.066	[-0.211 - 0.048]	2.167E-01	2.198E-01
Right temporal pole	-0.060	0.058	[-0.174 - 0.054]	3.047E-01	3.047E-01
Left entorhinal cortex	-0.061	0.039	[-0.138 - 0.016]	1.202E-01	1.274E-01
Right entorhinal cortex	-0.068	0.054	[-0.174 - 0.038]	2.090E-01	2.150E-01

Supplementary Table S6. Cortical thickness differences between individuals with bipolar disorder and healthy comparison subjects controlling for age and sex

	d	Std. Err.	95% CI	*p*-value	FDR *q*-value
Global mean cortical thickness	-0.430	0.147	[-0.719 - -0.141]	3.508E-03	1.756E-02
Left hemisphere	-0.426	0.150	[-0.719 - -0.132]	4.491E-03	1.876E-02
Right hemisphere	-0.420	0.143	[-0.700 - -0.139]	3.397E-03	1.756E-02
Left fusiform gyrus	-0.524	0.133	[-0.785 - -0.262]	8.668E-05	7.943E-04
Right fusiform gyrus	-0.483	0.123	[-0.723 - -0.242]	8.362E-05	7.943E-04
Left pars opercularis of inferior frontal gyrus	-0.370	0.145	[-0.654 - -0.087]	1.051E-02	2.665E-02
Right pars opercularis of inferior frontal gyrus	-0.265	0.099	[-0.459 - -0.071]	7.455E-03	2.493E-02
Left superior temporal gyrus	-0.286	0.124	[-0.529 - -0.044]	2.063E-02	3.958E-02
Right superior temporal gyrus	-0.265	0.125	[-0.510 - -0.020]	3.431E-02	5.296E-02
Left insula	-0.335	0.095	[-0.521 - -0.149]	4.078E-04	2.895E-03
Right insula	-0.403	0.096	[-0.591 - -0.215]	2.639E-05	6.141E-04
Left lingual gyrus	-0.289	0.125	[-0.534 - -0.045]	2.025E-02	3.958E-02
Right lingual gyrus	-0.338	0.154	[-0.639 - -0.036]	2.806E-02	4.577E-02
Left pars triangularis of inferior frontal gyrus	-0.276	0.168	[-0.605 - 0.052]	9.935E-02	1.331E-01
Right pars triangularis of inferior frontal gyrus	-0.292	0.123	[-0.532 - -0.052]	1.730E-02	3.509E-02
Left lateral orbitofrontal cortex	-0.421	0.129	[-0.675 - -0.167]	1.152E-03	6.979E-03
Right lateral orbitofrontal cortex	-0.460	0.118	[-0.691 - -0.230]	8.950E-05	7.943E-04
Left rostral middle frontal gyrus	-0.443	0.197	[-0.830 - -0.056]	2.497E-02	4.341E-02
Right rostral middle frontal gyrus	-0.256	0.162	[-0.573 - 0.060]	1.126E-01	1.437E-01
Left middle temporal gyrus	-0.358	0.138	[-0.629 - -0.088]	9.481E-03	2.493E-02
Right middle temporal gyrus	-0.465	0.143	[-0.745 - -0.184]	1.180E-03	6.979E-03
Left superior frontal gyrus	-0.378	0.098	[-0.571 - -0.186]	1.174E-04	9.265E-04
Right superior frontal gyrus	-0.229	0.103	[-0.431 - -0.028]	2.591E-02	4.380E-02
Left pars orbitalis of inferior frontal gyrus	-0.473	0.114	[-0.697 - -0.249]	3.460E-05	6.141E-04
Right pars orbitalis of inferior frontal gyrus	-0.384	0.135	[-0.648 - -0.119]	4.486E-03	1.876E-02
Left medial orbitofrontal cortex	-0.400	0.147	[-0.688 - -0.111]	6.639E-03	2.481E-02
Right medial orbitofrontal cortex	-0.442	0.169	[-0.773 - -0.110]	9.051E-03	2.493E-02
Left inferior temporal gyrus	-0.434	0.111	[-0.651 - -0.217]	8.680E-05	7.943E-04
Right inferior temporal gyrus	-0.507	0.096	[-0.695 - -0.319]	1.214E-07	8.617E-06
Left isthmus cingulate cortex	-0.291	0.109	[-0.505 - -0.076]	7.887E-03	2.493E-02
Right isthmus cingulate cortex	-0.214	0.085	[-0.381 - -0.046]	1.233E-02	2.892E-02
Left banks of superior temporal sulcus	-0.119	0.113	[-0.339 - 0.102]	2.922E-01	3.293E-01
Right banks of superior temporal sulcus	-0.224	0.092	[-0.405 - -0.044]	1.479E-02	3.182E-02
Left supramarginal gyrus	-0.240	0.085	[-0.408 - -0.073]	4.875E-03	1.923E-02
Right supramarginal gyrus	-0.307	0.156	[-0.613 - -0.002]	4.863E-02	7.193E-02
Left caudal middle frontal gyrus	-0.277	0.112	[-0.497 - -0.056]	1.383E-02	3.068E-02
Right caudal middle frontal gyrus	-0.203	0.122	[-0.442 - 0.035]	9.453E-02	1.291E-01
Left frontal pole	-0.294	0.116	[-0.521 - -0.067]	1.125E-02	2.753E-02
Right frontal pole	-0.343	0.153	[-0.644 - -0.043]	2.507E-02	4.341E-02
Left posterior cingulate cortex	-0.164	0.104	[-0.367 - 0.039]	1.133E-01	1.437E-01
Right posterior cingulate cortex	-0.186	0.085	[-0.353 - -0.019]	2.901E-02	4.577E-02
Left lateral occipital cortex	-0.306	0.127	[-0.555 - -0.058]	1.568E-02	3.274E-02
Right lateral occipital cortex	-0.347	0.133	[-0.607 - -0.086]	9.173E-03	2.493E-02
Left precentral gyrus	-0.280	0.140	[-0.554 - -0.006]	4.495E-02	6.790E-02
Right precentral gyrus	-0.246	0.113	[-0.466 - -0.025]	2.898E-02	4.577E-02
Left parahippocampal gyrus	-0.135	0.085	[-0.302 - 0.032]	1.134E-01	1.437E-01
Right parahippocampal gyrus	-0.129	0.085	[-0.296 - 0.038]	1.307E-01	1.628E-01
Left inferior parietal cortex	-0.195	0.085	[-0.362 - -0.028]	2.235E-02	4.080E-02
Right inferior parietal cortex	-0.263	0.099	[-0.457 - -0.069]	7.761E-03	2.493E-02
Left transverse temporal gyrus	-0.151	0.085	[-0.318 - 0.016]	7.625E-02	1.083E-01
Right transverse temporal gyrus	-0.075	0.121	[-0.311 - 0.162]	5.370E-01	5.690E-01
Left postcentral gyrus	-0.173	0.103	[-0.375 - 0.029]	9.366E-02	1.291E-01
Right postcentral gyrus	-0.253	0.096	[-0.442 - -0.065]	8.447E-03	2.493E-02
Left precuneus	-0.096	0.154	[-0.397 - 0.205]	5.317E-01	5.690E-01
Right precuneus	-0.103	0.085	[-0.270 - 0.064]	2.272E-01	2.688E-01
Left caudal anterior cingulate cortex	-0.167	0.085	[-0.335 - 0.000]	4.967E-02	7.197E-02
Right caudal anterior cingulate cortex	-0.071	0.085	[-0.238 - 0.096]	4.022E-01	4.393E-01
Left cuneus	-0.067	0.132	[-0.326 - 0.192]	6.118E-01	6.388E-01
Right cuneus	-0.154	0.123	[-0.394 - 0.087]	2.110E-01	2.539E-01
Left rostral anterior cingulate cortex	-0.366	0.086	[-0.534 - -0.198]	1.914E-05	6.141E-04
Right rostral anterior cingulate cortex	-0.222	0.097	[-0.412 - -0.031]	2.241E-02	4.080E-02
Left pericalcarine cortex	-0.030	0.094	[-0.214 - 0.155]	7.528E-01	7.747E-01
Right pericalcarine cortex	-0.015	0.088	[-0.187 - 0.157]	8.607E-01	8.730E-01
Left paracentral lobule	-0.114	0.106	[-0.321 - 0.094]	2.822E-01	3.232E-01
Right paracentral lobule	-0.167	0.121	[-0.405 - 0.071]	1.693E-01	2.073E-01
Left superior parietal cortex	-0.006	0.138	[-0.277 - 0.265]	9.672E-01	9.672E-01
Right superior parietal cortex	-0.128	0.116	[-0.356 - 0.099]	2.693E-01	3.134E-01
Left temporal pole	-0.303	0.122	[-0.542 - -0.065]	1.263E-02	2.892E-02
Right temporal pole	-0.253	0.087	[-0.424 - -0.082]	3.710E-03	1.756E-02
Left entorhinal cortex	-0.223	0.085	[-0.390 - -0.055]	9.094E-03	2.493E-02
Right entorhinal cortex	-0.139	0.144	[-0.420 - 0.143]	3.338E-01	3.703E-01

Supplementary Table S7. Cortical thickness differences between individuals with bipolar disorder and healthy comparison subjects controlling for age and sex at 25 years of age or older

	d	Std. Err.	95% CI	*p*-value	FDR *q*-value
Global mean cortical thickness	-0.445	0.152	[-0.744 - -0.147]	3.478E-03	1.300E-02
Left hemisphere	-0.437	0.155	[-0.740 - -0.134]	4.712E-03	1.605E-02
Right hemisphere	-0.439	0.149	[-0.731 - -0.147]	3.231E-03	1.300E-02
Left fusiform gyrus	-0.517	0.135	[-0.782 - -0.252]	1.329E-04	1.180E-03
Right fusiform gyrus	-0.474	0.123	[-0.715 - -0.234]	1.090E-04	1.106E-03
Left pars opercularis of inferior frontal gyrus	-0.389	0.156	[-0.694 - -0.083]	1.256E-02	2.714E-02
Right pars opercularis of inferior frontal gyrus	-0.291	0.099	[-0.486 - -0.096]	3.385E-03	1.300E-02
Left superior temporal gyrus	-0.281	0.147	[-0.570 - 0.008]	5.675E-02	8.222E-02
Right superior temporal gyrus	-0.284	0.143	[-0.565 - -0.003]	4.785E-02	7.385E-02
Left insula	-0.349	0.104	[-0.552 - -0.145]	7.813E-04	5.043E-03
Right insula	-0.404	0.103	[-0.605 - -0.203]	8.264E-05	1.057E-03
Left lingual gyrus	-0.235	0.114	[-0.457 - -0.012]	3.872E-02	6.109E-02
Right lingual gyrus	-0.278	0.133	[-0.539 - -0.017]	3.668E-02	5.919E-02
Left pars triangularis of inferior frontal gyrus	-0.281	0.182	[-0.637 - 0.075]	1.223E-01	1.578E-01
Right pars triangularis of inferior frontal gyrus	-0.320	0.151	[-0.616 - -0.025]	3.380E-02	5.580E-02
Left lateral orbitofrontal cortex	-0.434	0.141	[-0.709 - -0.159]	2.010E-03	9.515E-03
Right lateral orbitofrontal cortex	-0.449	0.115	[-0.673 - -0.224]	8.936E-05	1.057E-03
Left rostral middle frontal gyrus	-0.477	0.211	[-0.890 - -0.064]	2.355E-02	4.239E-02
Right rostral middle frontal gyrus	-0.313	0.189	[-0.683 - 0.058]	9.846E-02	1.301E-01
Left middle temporal gyrus	-0.351	0.140	[-0.626 - -0.076]	1.236E-02	2.714E-02
Right middle temporal gyrus	-0.479	0.155	[-0.783 - -0.176]	1.969E-03	9.515E-03
Left superior frontal gyrus	-0.430	0.123	[-0.670 - -0.189]	4.581E-04	3.614E-03
Right superior frontal gyrus	-0.261	0.113	[-0.483 - -0.039]	2.109E-02	4.048E-02
Left pars orbitalis of inferior frontal gyrus	-0.485	0.108	[-0.697 - -0.273]	7.373E-06	1.745E-04
Right pars orbitalis of inferior frontal gyrus	-0.435	0.137	[-0.704 - -0.166]	1.530E-03	9.053E-03
Left medial orbitofrontal cortex	-0.392	0.165	[-0.714 - -0.069]	1.726E-02	3.502E-02
Right medial orbitofrontal cortex	-0.423	0.170	[-0.755 - -0.091]	1.261E-02	2.714E-02
Left inferior temporal gyrus	-0.463	0.115	[-0.689 - -0.237]	6.023E-05	1.057E-03
Right inferior temporal gyrus	-0.509	0.113	[-0.730 - -0.288]	6.445E-06	1.745E-04
Left isthmus cingulate cortex	-0.284	0.112	[-0.502 - -0.065]	1.110E-02	2.714E-02
Right isthmus cingulate cortex	-0.229	0.102	[-0.428 - -0.030]	2.388E-02	4.239E-02
Left banks of superior temporal sulcus	-0.129	0.120	[-0.364 - 0.106]	2.833E-01	3.094E-01
Right banks of superior temporal sulcus	-0.206	0.110	[-0.422 - 0.009]	6.083E-02	8.638E-02
Left supramarginal gyrus	-0.252	0.100	[-0.449 - -0.056]	1.168E-02	2.714E-02
Right supramarginal gyrus	-0.329	0.154	[-0.631 - -0.028]	3.225E-02	5.452E-02
Left caudal middle frontal gyrus	-0.279	0.110	[-0.495 - -0.063]	1.118E-02	2.714E-02
Right caudal middle frontal gyrus	-0.215	0.128	[-0.466 - 0.036]	9.384E-02	1.281E-01
Left frontal pole	-0.332	0.128	[-0.582 - -0.081]	9.432E-03	2.668E-02
Right frontal pole	-0.407	0.159	[-0.720 - -0.095]	1.061E-02	2.714E-02
Left posterior cingulate cortex	-0.172	0.118	[-0.403 - 0.059]	1.450E-01	1.775E-01
Right posterior cingulate cortex	-0.245	0.088	[-0.417 - -0.072]	5.581E-03	1.801E-02
Left lateral occipital cortex	-0.350	0.132	[-0.608 - -0.092]	7.916E-03	2.342E-02
Right lateral occipital cortex	-0.350	0.129	[-0.603 - -0.096]	6.828E-03	2.108E-02
Left precentral gyrus	-0.280	0.144	[-0.562 - 0.001]	5.080E-02	7.675E-02
Right precentral gyrus	-0.286	0.122	[-0.525 - -0.048]	1.851E-02	3.651E-02
Left parahippocampal gyrus	-0.145	0.088	[-0.318 - 0.027]	9.892E-02	1.301E-01
Right parahippocampal gyrus	-0.127	0.088	[-0.300 - 0.046]	1.503E-01	1.809E-01
Left inferior parietal cortex	-0.210	0.088	[-0.383 - -0.038]	1.701E-02	3.502E-02
Right inferior parietal cortex	-0.292	0.103	[-0.495 - -0.089]	4.748E-03	1.605E-02
Left transverse temporal gyrus	-0.169	0.088	[-0.342 - 0.003]	5.456E-02	8.070E-02
Right transverse temporal gyrus	-0.095	0.131	[-0.351 - 0.162]	4.705E-01	5.061E-01
Left postcentral gyrus	-0.180	0.107	[-0.390 - 0.030]	9.225E-02	1.281E-01
Right postcentral gyrus	-0.260	0.100	[-0.457 - -0.063]	9.769E-03	2.668E-02
Left precuneus	-0.095	0.151	[-0.391 - 0.201]	5.290E-01	5.606E-01
Right precuneus	-0.098	0.088	[-0.270 - 0.075]	2.668E-01	3.007E-01
Left caudal anterior cingulate cortex	-0.196	0.088	[-0.369 - -0.023]	2.628E-02	4.551E-02
Right caudal anterior cingulate cortex	-0.095	0.088	[-0.268 - 0.078]	2.810E-01	3.094E-01
Left cuneus	-0.065	0.131	[-0.321 - 0.191]	6.182E-01	6.455E-01
Right cuneus	-0.162	0.126	[-0.408 - 0.085]	1.994E-01	2.360E-01
Left rostral anterior cingulate cortex	-0.416	0.089	[-0.590 - -0.242]	2.876E-06	1.745E-04
Right rostral anterior cingulate cortex	-0.226	0.099	[-0.421 - -0.032]	2.275E-02	4.239E-02
Left pericalcarine cortex	-0.006	0.088	[-0.178 - 0.167]	9.474E-01	9.474E-01
Right pericalcarine cortex	0.008	0.088	[-0.166 - 0.181]	9.322E-01	9.455E-01
Left paracentral lobule	-0.123	0.107	[-0.334 - 0.087]	2.516E-01	2.881E-01
Right paracentral lobule	-0.183	0.126	[-0.429 - 0.063]	1.446E-01	1.775E-01
Left superior parietal cortex	-0.015	0.136	[-0.280 - 0.251]	9.137E-01	9.402E-01
Right superior parietal cortex	-0.132	0.107	[-0.342 - 0.078]	2.184E-01	2.542E-01
Left temporal pole	-0.321	0.108	[-0.532 - -0.110]	2.923E-03	1.297E-02
Right temporal pole	-0.274	0.088	[-0.447 - -0.101]	1.926E-03	9.515E-03
Left entorhinal cortex	-0.304	0.088	[-0.477 - -0.131]	5.894E-04	4.185E-03
Right entorhinal cortex	-0.213	0.143	[-0.492 - 0.067]	1.366E-01	1.732E-01

Supplementary Table S8. Cortical thickness differences between individuals with major depressive disorder and healthy comparison subjects controlling for age and sex

	d	Std. Err.	95% CI	*p*-value	FDR *q*-value
Global mean cortical thickness	-0.350	0.122	[-0.590 - -0.110]	4.267E-03	1.894E-02
Left hemisphere	-0.377	0.125	[-0.622 - -0.132]	2.555E-03	1.395E-02
Right hemisphere	-0.303	0.116	[-0.530 - -0.075]	9.076E-03	2.784E-02
Left fusiform gyrus	-0.264	0.094	[-0.448 - -0.081]	4.826E-03	2.002E-02
Right fusiform gyrus	-0.229	0.085	[-0.394 - -0.063]	6.833E-03	2.426E-02
Left pars opercularis of inferior frontal gyrus	-0.281	0.074	[-0.426 - -0.135]	1.523E-04	2.596E-03
Right pars opercularis of inferior frontal gyrus	-0.173	0.114	[-0.396 - 0.051]	1.305E-01	1.742E-01
Left superior temporal gyrus	-0.323	0.076	[-0.472 - -0.174]	2.173E-05	1.543E-03
Right superior temporal gyrus	-0.217	0.084	[-0.382 - -0.053]	9.506E-03	2.784E-02
Left insula	-0.248	0.097	[-0.438 - -0.057]	1.079E-02	2.837E-02
Right insula	-0.217	0.108	[-0.428 - -0.006]	4.389E-02	8.200E-02
Left lingual gyrus	-0.221	0.094	[-0.404 - -0.037]	1.863E-02	4.268E-02
Right lingual gyrus	-0.213	0.107	[-0.423 - -0.003]	4.632E-02	8.294E-02
Left pars triangularis of inferior frontal gyrus	-0.181	0.078	[-0.333 - -0.029]	1.927E-02	4.275E-02
Right pars triangularis of inferior frontal gyrus	-0.191	0.101	[-0.389 - 0.006]	5.786E-02	9.781E-02
Left lateral orbitofrontal cortex	-0.329	0.095	[-0.514 - -0.143]	5.173E-04	4.819E-03
Right lateral orbitofrontal cortex	-0.243	0.092	[-0.423 - -0.064]	7.844E-03	2.652E-02
Left rostral middle frontal gyrus	-0.334	0.109	[-0.547 - -0.121]	2.095E-03	1.239E-02
Right rostral middle frontal gyrus	-0.203	0.102	[-0.404 - -0.003]	4.673E-02	8.294E-02
Left middle temporal gyrus	-0.369	0.093	[-0.552 - -0.186]	7.875E-05	2.596E-03
Right middle temporal gyrus	-0.293	0.099	[-0.488 - -0.099]	3.065E-03	1.554E-02
Left superior frontal gyrus	-0.281	0.097	[-0.470 - -0.091]	3.670E-03	1.737E-02
Right superior frontal gyrus	-0.264	0.077	[-0.414 - -0.113]	6.006E-04	4.819E-03
Left pars orbitalis of inferior frontal gyrus	-0.311	0.091	[-0.489 - -0.133]	6.109E-04	4.819E-03
Right pars orbitalis of inferior frontal gyrus	-0.305	0.082	[-0.465 - -0.145]	1.881E-04	2.596E-03
Left medial orbitofrontal cortex	-0.300	0.115	[-0.525 - -0.074]	9.151E-03	2.784E-02
Right medial orbitofrontal cortex	-0.255	0.083	[-0.417 - -0.093]	2.013E-03	1.239E-02
Left inferior temporal gyrus	-0.234	0.083	[-0.397 - -0.070]	5.075E-03	2.002E-02
Right inferior temporal gyrus	-0.235	0.061	[-0.355 - -0.114]	1.360E-04	2.596E-03
Left isthmus cingulate cortex	-0.167	0.080	[-0.324 - -0.010]	3.651E-02	7.241E-02
Right isthmus cingulate cortex	-0.118	0.071	[-0.258 - 0.021]	9.676E-02	1.431E-01
Left banks of superior temporal sulcus	-0.196	0.076	[-0.345 - -0.047]	9.898E-03	2.784E-02
Right banks of superior temporal sulcus	-0.142	0.106	[-0.349 - 0.065]	1.799E-01	2.202E-01
Left supramarginal gyrus	-0.241	0.094	[-0.424 - -0.057]	1.020E-02	2.784E-02
Right supramarginal gyrus	-0.208	0.095	[-0.395 - -0.021]	2.924E-02	6.107E-02
Left caudal middle frontal gyrus	-0.200	0.106	[-0.409 - 0.008]	5.965E-02	9.849E-02
Right caudal middle frontal gyrus	-0.248	0.098	[-0.441 - -0.055]	1.184E-02	3.002E-02
Left frontal pole	-0.152	0.061	[-0.273 - -0.032]	1.315E-02	3.219E-02
Right frontal pole	-0.154	0.102	[-0.353 - 0.045]	1.303E-01	1.742E-01
Left posterior cingulate cortex	-0.227	0.061	[-0.348 - -0.107]	2.194E-04	2.596E-03
Right posterior cingulate cortex	-0.164	0.074	[-0.309 - -0.019]	2.659E-02	5.721E-02
Left lateral occipital cortex	-0.205	0.098	[-0.397 - -0.012]	3.740E-02	7.241E-02
Right lateral occipital cortex	-0.174	0.104	[-0.378 - 0.029]	9.350E-02	1.416E-01
Left precentral gyrus	-0.315	0.100	[-0.511 - -0.119]	1.607E-03	1.141E-02
Right precentral gyrus	-0.214	0.078	[-0.366 - -0.062]	5.836E-03	2.181E-02
Left parahippocampal gyrus	-0.170	0.102	[-0.369 - 0.029]	9.371E-02	1.416E-01
Right parahippocampal gyrus	-0.069	0.077	[-0.220 - 0.082]	3.680E-01	4.083E-01
Left inferior parietal cortex	-0.192	0.093	[-0.374 - -0.011]	3.773E-02	7.241E-02
Right inferior parietal cortex	-0.146	0.097	[-0.337 - 0.044]	1.325E-01	1.742E-01
Left transverse temporal gyrus	-0.117	0.092	[-0.296 - 0.063]	2.021E-01	2.432E-01
Right transverse temporal gyrus	-0.076	0.095	[-0.261 - 0.110]	4.247E-01	4.639E-01
Left postcentral gyrus	-0.191	0.110	[-0.408 - 0.025]	8.311E-02	1.311E-01
Right postcentral gyrus	-0.185	0.095	[-0.370 - 0.001]	5.106E-02	8.842E-02
Left precuneus	-0.128	0.091	[-0.306 - 0.051]	1.603E-01	1.997E-01
Right precuneus	-0.183	0.074	[-0.329 - -0.037]	1.377E-02	3.259E-02
Left caudal anterior cingulate cortex	-0.094	0.061	[-0.214 - 0.026]	1.249E-01	1.742E-01
Right caudal anterior cingulate cortex	-0.068	0.061	[-0.188 - 0.052]	2.673E-01	3.112E-01
Left cuneus	-0.109	0.076	[-0.258 - 0.040]	1.521E-01	1.946E-01
Right cuneus	-0.090	0.063	[-0.213 - 0.034]	1.535E-01	1.946E-01
Left rostral anterior cingulate cortex	-0.128	0.082	[-0.288 - 0.032]	1.167E-01	1.691E-01
Right rostral anterior cingulate cortex	-0.006	0.080	[-0.164 - 0.152]	9.404E-01	9.539E-01
Left pericalcarine cortex	-0.051	0.078	[-0.204 - 0.103]	5.193E-01	5.557E-01
Right pericalcarine cortex	0.004	0.089	[-0.170 - 0.177]	9.679E-01	9.679E-01
Left paracentral lobule	-0.153	0.084	[-0.317 - 0.012]	6.907E-02	1.114E-01
Right paracentral lobule	-0.138	0.091	[-0.316 - 0.041]	1.308E-01	1.742E-01
Left superior parietal cortex	-0.140	0.114	[-0.364 - 0.084]	2.197E-01	2.600E-01
Right superior parietal cortex	-0.092	0.097	[-0.282 - 0.097]	3.398E-01	3.830E-01
Left temporal pole	-0.063	0.065	[-0.191 - 0.065]	3.373E-01	3.830E-01
Right temporal pole	0.057	0.090	[-0.119 - 0.233]	5.244E-01	5.557E-01
Left entorhinal cortex	-0.059	0.097	[-0.248 - 0.131]	5.453E-01	5.694E-01
Right entorhinal cortex	-0.022	0.103	[-0.223 - 0.179]	8.283E-01	8.523E-01

Supplementary Table S9. Cortical thickness differences between individuals with major depressive disorder and healthy comparison subjects controlling for age and sex over 21 years old

	d	Std. Err.	95% CI	*p*-value	FDR *q*-value
Global mean cortical thickness	-0.315	0.124	[-0.557 - -0.073]	1.080E-02	3.836E-02
Left hemisphere	-0.341	0.126	[-0.589 - -0.094]	6.843E-03	3.276E-02
Right hemisphere	-0.274	0.118	[-0.506 - -0.042]	2.041E-02	5.176E-02
Left fusiform gyrus	-0.241	0.091	[-0.419 - -0.063]	7.845E-03	3.276E-02
Right fusiform gyrus	-0.218	0.087	[-0.390 - -0.047]	1.238E-02	3.940E-02
Left pars opercularis of inferior frontal gyrus	-0.288	0.078	[-0.440 - -0.136]	2.069E-04	3.412E-03
Right pars opercularis of inferior frontal gyrus	-0.177	0.118	[-0.408 - 0.055]	1.347E-01	1.974E-01
Left superior temporal gyrus	-0.312	0.081	[-0.471 - -0.153]	1.184E-04	3.412E-03
Right superior temporal gyrus	-0.212	0.089	[-0.386 - -0.037]	1.772E-02	4.748E-02
Left insula	-0.224	0.095	[-0.410 - -0.038]	1.805E-02	4.748E-02
Right insula	-0.201	0.109	[-0.415 - 0.012]	6.498E-02	1.153E-01
Left lingual gyrus	-0.175	0.090	[-0.351 - 0.002]	5.207E-02	1.057E-01
Right lingual gyrus	-0.156	0.101	[-0.354 - 0.042]	1.220E-01	1.883E-01
Left pars triangularis of inferior frontal gyrus	-0.187	0.083	[-0.349 - -0.024]	2.429E-02	5.948E-02
Right pars triangularis of inferior frontal gyrus	-0.208	0.109	[-0.422 - 0.006]	5.659E-02	1.057E-01
Left lateral orbitofrontal cortex	-0.329	0.106	[-0.536 - -0.121]	1.906E-03	1.603E-02
Right lateral orbitofrontal cortex	-0.268	0.100	[-0.465 - -0.071]	7.560E-03	3.276E-02
Left rostral middle frontal gyrus	-0.361	0.122	[-0.601 - -0.121]	3.196E-03	1.976E-02
Right rostral middle frontal gyrus	-0.192	0.111	[-0.411 - 0.026]	8.466E-02	1.398E-01
Left middle temporal gyrus	-0.400	0.105	[-0.606 - -0.194]	1.454E-04	3.412E-03
Right middle temporal gyrus	-0.308	0.105	[-0.513 - -0.102]	3.340E-03	1.976E-02
Left superior frontal gyrus	-0.276	0.107	[-0.487 - -0.065]	1.023E-02	3.836E-02
Right superior frontal gyrus	-0.264	0.085	[-0.431 - -0.096]	2.032E-03	1.603E-02
Left pars orbitalis of inferior frontal gyrus	-0.303	0.097	[-0.494 - -0.112]	1.851E-03	1.603E-02
Right pars orbitalis of inferior frontal gyrus	-0.325	0.089	[-0.500 - -0.150]	2.795E-04	3.412E-03
Left medial orbitofrontal cortex	-0.258	0.118	[-0.489 - -0.028]	2.813E-02	6.241E-02
Right medial orbitofrontal cortex	-0.246	0.091	[-0.425 - -0.067]	7.099E-03	3.276E-02
Left inferior temporal gyrus	-0.211	0.078	[-0.363 - -0.058]	6.771E-03	3.276E-02
Right inferior temporal gyrus	-0.231	0.063	[-0.355 - -0.106]	2.767E-04	3.412E-03
Left isthmus cingulate cortex	-0.156	0.080	[-0.314 - 0.002]	5.271E-02	1.057E-01
Right isthmus cingulate cortex	-0.118	0.078	[-0.270 - 0.034]	1.274E-01	1.924E-01
Left banks of superior temporal sulcus	-0.206	0.086	[-0.375 - -0.037]	1.684E-02	4.748E-02
Right banks of superior temporal sulcus	-0.123	0.100	[-0.320 - 0.073]	2.192E-01	2.829E-01
Left supramarginal gyrus	-0.248	0.102	[-0.448 - -0.049]	1.482E-02	4.385E-02
Right supramarginal gyrus	-0.188	0.091	[-0.365 - -0.011]	3.787E-02	8.147E-02
Left caudal middle frontal gyrus	-0.196	0.112	[-0.415 - 0.023]	8.005E-02	1.386E-01
Right caudal middle frontal gyrus	-0.236	0.093	[-0.418 - -0.055]	1.080E-02	3.836E-02
Left frontal pole	-0.170	0.068	[-0.303 - -0.036]	1.276E-02	3.940E-02
Right frontal pole	-0.166	0.112	[-0.385 - 0.053]	1.371E-01	1.974E-01
Left posterior cingulate cortex	-0.229	0.063	[-0.353 - -0.105]	2.883E-04	3.412E-03
Right posterior cingulate cortex	-0.161	0.073	[-0.304 - -0.018]	2.771E-02	6.241E-02
Left lateral occipital cortex	-0.169	0.088	[-0.342 - 0.004]	5.547E-02	1.057E-01
Right lateral occipital cortex	-0.135	0.100	[-0.331 - 0.060]	1.748E-01	2.342E-01
Left precentral gyrus	-0.279	0.093	[-0.462 - -0.097]	2.698E-03	1.915E-02
Right precentral gyrus	-0.203	0.081	[-0.361 - -0.045]	1.184E-02	3.940E-02
Left parahippocampal gyrus	-0.137	0.095	[-0.324 - 0.050]	1.515E-01	2.069E-01
Right parahippocampal gyrus	-0.054	0.076	[-0.203 - 0.096]	4.821E-01	5.433E-01
Left inferior parietal cortex	-0.172	0.090	[-0.349 - 0.005]	5.652E-02	1.057E-01
Right inferior parietal cortex	-0.156	0.098	[-0.349 - 0.036]	1.115E-01	1.759E-01
Left transverse temporal gyrus	-0.092	0.092	[-0.273 - 0.088]	3.163E-01	3.743E-01
Right transverse temporal gyrus	-0.054	0.098	[-0.246 - 0.139]	5.850E-01	6.390E-01
Left postcentral gyrus	-0.153	0.105	[-0.359 - 0.054]	1.469E-01	2.045E-01
Right postcentral gyrus	-0.167	0.096	[-0.355 - 0.021]	8.236E-02	1.392E-01
Left precuneus	-0.119	0.094	[-0.303 - 0.065]	2.048E-01	2.692E-01
Right precuneus	-0.168	0.075	[-0.315 - -0.020]	2.596E-02	6.144E-02
Left caudal anterior cingulate cortex	-0.107	0.063	[-0.230 - 0.017]	9.158E-02	1.478E-01
Right caudal anterior cingulate cortex	-0.079	0.066	[-0.208 - 0.049]	2.262E-01	2.868E-01
Left cuneus	-0.086	0.076	[-0.236 - 0.064]	2.602E-01	3.185E-01
Right cuneus	-0.065	0.067	[-0.197 - 0.066]	3.283E-01	3.821E-01
Left rostral anterior cingulate cortex	-0.154	0.082	[-0.314 - 0.006]	5.888E-02	1.072E-01
Right rostral anterior cingulate cortex	-0.005	0.080	[-0.163 - 0.152]	9.467E-01	9.467E-01
Left pericalcarine cortex	-0.022	0.065	[-0.151 - 0.106]	7.322E-01	7.759E-01
Right pericalcarine cortex	0.023	0.091	[-0.156 - 0.203]	7.975E-01	8.327E-01
Left paracentral lobule	-0.120	0.081	[-0.278 - 0.039]	1.390E-01	1.974E-01
Right paracentral lobule	-0.109	0.094	[-0.294 - 0.075]	2.451E-01	3.053E-01
Left superior parietal cortex	-0.083	0.100	[-0.280 - 0.114]	4.101E-01	4.696E-01
Right superior parietal cortex	-0.064	0.093	[-0.247 - 0.119]	4.901E-01	5.437E-01
Left temporal pole	-0.084	0.079	[-0.240 - 0.071]	2.887E-01	3.474E-01
Right temporal pole	0.050	0.096	[-0.139 - 0.239]	6.072E-01	6.532E-01
Left entorhinal cortex	-0.013	0.093	[-0.195 - 0.170]	8.910E-01	9.168E-01
Right entorhinal cortex	-0.008	0.107	[-0.217 - 0.202]	9.430E-01	9.467E-01

Supplementary Table S10. Cortical thickness differences between individuals with autism spectrum disorder and healthy comparison subjects controlling for age and sex

	d	Std. Err.	95% CI	*p*-value	FDR *q*-value
Global mean cortical thickness	-0.010	0.096	[-0.198 - 0.178]	9.148E-01	9.764E-01
Left hemisphere	-0.006	0.094	[-0.191 - 0.178]	9.473E-01	9.764E-01
Right hemisphere	-0.013	0.096	[-0.202 - 0.176]	8.927E-01	9.764E-01
Left fusiform gyrus	-0.081	0.116	[-0.309 - 0.146]	4.843E-01	9.764E-01
Right fusiform gyrus	-0.180	0.112	[-0.400 - 0.040]	1.085E-01	9.764E-01
Left pars opercularis of inferior frontal gyrus	0.083	0.080	[-0.074 - 0.240]	3.019E-01	9.764E-01
Right pars opercularis of inferior frontal gyrus	-0.024	0.080	[-0.181 - 0.133]	7.624E-01	9.764E-01
Left superior temporal gyrus	0.063	0.115	[-0.162 - 0.289]	5.830E-01	9.764E-01
Right superior temporal gyrus	0.056	0.080	[-0.101 - 0.213]	4.847E-01	9.764E-01
Left insula	0.045	0.121	[-0.192 - 0.282]	7.121E-01	9.764E-01
Right insula	-0.051	0.110	[-0.267 - 0.165]	6.439E-01	9.764E-01
Left lingual gyrus	-0.138	0.105	[-0.343 - 0.067]	1.882E-01	9.764E-01
Right lingual gyrus	-0.147	0.080	[-0.304 - 0.010]	6.587E-02	9.764E-01
Left pars triangularis of inferior frontal gyrus	0.216	0.096	[0.027 - 0.404]	2.496E-02	5.906E-01
Right pars triangularis of inferior frontal gyrus	0.051	0.080	[-0.106 - 0.208]	5.262E-01	9.764E-01
Left lateral orbitofrontal cortex	0.032	0.097	[-0.158 - 0.223]	7.403E-01	9.764E-01
Right lateral orbitofrontal cortex	-0.182	0.080	[-0.339 - -0.025]	2.279E-02	5.906E-01
Left rostral middle frontal gyrus	0.055	0.088	[-0.118 - 0.228]	5.367E-01	9.764E-01
Right rostral middle frontal gyrus	0.069	0.082	[-0.092 - 0.230]	3.992E-01	9.764E-01
Left middle temporal gyrus	0.010	0.143	[-0.269 - 0.290]	9.433E-01	9.764E-01
Right middle temporal gyrus	0.006	0.130	[-0.249 - 0.260]	9.657E-01	9.764E-01
Left superior frontal gyrus	0.041	0.080	[-0.116 - 0.198]	6.088E-01	9.764E-01
Right superior frontal gyrus	0.054	0.083	[-0.108 - 0.217]	5.117E-01	9.764E-01
Left pars orbitalis of inferior frontal gyrus	0.010	0.129	[-0.243 - 0.263]	9.400E-01	9.764E-01
Right pars orbitalis of inferior frontal gyrus	-0.035	0.094	[-0.219 - 0.149]	7.102E-01	9.764E-01
Left medial orbitofrontal cortex	-0.046	0.086	[-0.215 - 0.123]	5.926E-01	9.764E-01
Right medial orbitofrontal cortex	0.040	0.080	[-0.117 - 0.197]	6.189E-01	9.764E-01
Left inferior temporal gyrus	-0.075	0.155	[-0.378 - 0.229]	6.306E-01	9.764E-01
Right inferior temporal gyrus	-0.019	0.133	[-0.279 - 0.241]	8.867E-01	9.764E-01
Left isthmus cingulate cortex	0.033	0.080	[-0.124 - 0.190]	6.814E-01	9.764E-01
Right isthmus cingulate cortex	-0.053	0.080	[-0.210 - 0.104]	5.056E-01	9.764E-01
Left banks of superior temporal sulcus	0.130	0.103	[-0.073 - 0.332]	2.087E-01	9.764E-01
Right banks of superior temporal sulcus	0.041	0.120	[-0.195 - 0.277]	7.330E-01	9.764E-01
Left supramarginal gyrus	-0.090	0.103	[-0.291 - 0.112]	3.825E-01	9.764E-01
Right supramarginal gyrus	-0.026	0.080	[-0.183 - 0.131]	7.428E-01	9.764E-01
Left caudal middle frontal gyrus	0.020	0.084	[-0.144 - 0.185]	8.082E-01	9.764E-01
Right caudal middle frontal gyrus	0.116	0.145	[-0.169 - 0.401]	4.241E-01	9.764E-01
Left frontal pole	0.039	0.129	[-0.214 - 0.292]	7.604E-01	9.764E-01
Right frontal pole	-0.019	0.080	[-0.176 - 0.138]	8.148E-01	9.764E-01
Left posterior cingulate cortex	0.212	0.080	[0.054 - 0.369]	8.336E-03	5.906E-01
Right posterior cingulate cortex	0.059	0.096	[-0.129 - 0.247]	5.371E-01	9.764E-01
Left lateral occipital cortex	-0.069	0.080	[-0.226 - 0.088]	3.866E-01	9.764E-01
Right lateral occipital cortex	-0.050	0.080	[-0.207 - 0.107]	5.306E-01	9.764E-01
Left precentral gyrus	-0.082	0.080	[-0.239 - 0.075]	3.062E-01	9.764E-01
Right precentral gyrus	-0.114	0.080	[-0.271 - 0.043]	1.564E-01	9.764E-01
Left parahippocampal gyrus	-0.051	0.080	[-0.208 - 0.106]	5.264E-01	9.764E-01
Right parahippocampal gyrus	0.077	0.080	[-0.080 - 0.234]	3.350E-01	9.764E-01
Left inferior parietal cortex	-0.070	0.080	[-0.227 - 0.087]	3.813E-01	9.764E-01
Right inferior parietal cortex	-0.008	0.080	[-0.165 - 0.148]	9.166E-01	9.764E-01
Left transverse temporal gyrus	0.116	0.115	[-0.110 - 0.341]	3.138E-01	9.764E-01
Right transverse temporal gyrus	-0.006	0.144	[-0.289 - 0.277]	9.657E-01	9.764E-01
Left postcentral gyrus	-0.025	0.103	[-0.228 - 0.177]	8.059E-01	9.764E-01
Right postcentral gyrus	0.006	0.116	[-0.222 - 0.234]	9.598E-01	9.764E-01
Left precuneus	0.042	0.080	[-0.115 - 0.199]	6.014E-01	9.764E-01
Right precuneus	-0.002	0.083	[-0.166 - 0.161]	9.764E-01	9.764E-01
Left caudal anterior cingulate cortex	0.003	0.080	[-0.154 - 0.160]	9.708E-01	9.764E-01
Right caudal anterior cingulate cortex	-0.056	0.120	[-0.291 - 0.179]	6.408E-01	9.764E-01
Left cuneus	0.075	0.097	[-0.115 - 0.265]	4.376E-01	9.764E-01
Right cuneus	0.085	0.080	[-0.071 - 0.242]	2.857E-01	9.764E-01
Left rostral anterior cingulate cortex	-0.003	0.096	[-0.192 - 0.186]	9.757E-01	9.764E-01
Right rostral anterior cingulate cortex	0.070	0.106	[-0.139 - 0.278]	5.110E-01	9.764E-01
Left pericalcarine cortex	0.028	0.080	[-0.128 - 0.185]	7.228E-01	9.764E-01
Right pericalcarine cortex	-0.031	0.080	[-0.187 - 0.126]	7.013E-01	9.764E-01
Left paracentral lobule	0.009	0.080	[-0.148 - 0.166]	9.109E-01	9.764E-01
Right paracentral lobule	-0.069	0.111	[-0.286 - 0.147]	5.306E-01	9.764E-01
Left superior parietal cortex	-0.021	0.092	[-0.201 - 0.159]	8.195E-01	9.764E-01
Right superior parietal cortex	0.063	0.088	[-0.109 - 0.235]	4.706E-01	9.764E-01
Left temporal pole	0.047	0.080	[-0.110 - 0.203]	5.607E-01	9.764E-01
Right temporal pole	0.126	0.104	[-0.077 - 0.330]	2.230E-01	9.764E-01
Left entorhinal cortex	-0.013	0.097	[-0.202 - 0.177]	8.955E-01	9.764E-01
Right entorhinal cortex	0.057	0.080	[-0.099 - 0.214]	4.730E-01	9.764E-01

Supplementary Table S11. Cortical thickness differences between individuals with schizophrenia and healthy comparison subjects controlling for age, sex, and global mean cortical thickness

	d	Std. Err.	95% CI	*p*-value	FDR *q*-value
Left fusiform gyrus	-0.218	0.045	[-0.306 - -0.130]	1.196E-06	7.392E-06
Right fusiform gyrus	-0.245	0.048	[-0.340 - -0.150]	4.098E-07	3.484E-06
Left pars opercularis of inferior frontal gyrus	-0.228	0.063	[-0.351 - -0.105]	2.686E-04	8.696E-04
Right pars opercularis of inferior frontal gyrus	-0.198	0.052	[-0.300 - -0.096]	1.510E-04	5.135E-04
Left superior temporal gyrus	-0.188	0.040	[-0.265 - -0.110]	2.060E-06	1.167E-05
Right superior temporal gyrus	-0.181	0.042	[-0.264 - -0.097]	2.069E-05	9.378E-05
Left insula	-0.244	0.035	[-0.312 - -0.176]	2.280E-12	7.750E-11
Right insula	-0.191	0.035	[-0.259 - -0.122]	4.841E-08	4.702E-07
Left lingual gyrus	-0.250	0.053	[-0.353 - -0.146]	2.407E-06	1.259E-05
Right lingual gyrus	-0.236	0.035	[-0.304 - -0.168]	1.068E-11	2.421E-10
Left pars triangularis of inferior frontal gyrus	-0.185	0.060	[-0.303 - -0.067]	2.170E-03	5.675E-03
Right pars triangularis of inferior frontal gyrus	-0.126	0.049	[-0.223 - -0.030]	1.045E-02	2.089E-02
Left lateral orbitofrontal cortex	-0.200	0.062	[-0.322 - -0.078]	1.281E-03	3.486E-03
Right lateral orbitofrontal cortex	-0.176	0.045	[-0.264 - -0.088]	8.962E-05	3.585E-04
Left rostral middle frontal gyrus	-0.137	0.070	[-0.273 - 0.000]	4.918E-02	7.452E-02
Right rostral middle frontal gyrus	-0.053	0.069	[-0.188 - 0.082]	4.439E-01	4.716E-01
Left middle temporal gyrus	-0.107	0.044	[-0.193 - -0.020]	1.580E-02	2.844E-02
Right middle temporal gyrus	-0.086	0.058	[-0.199 - 0.028]	1.381E-01	1.772E-01
Left superior frontal gyrus	-0.050	0.041	[-0.131 - 0.030]	2.193E-01	2.617E-01
Right superior frontal gyrus	-0.009	0.049	[-0.105 - 0.087]	8.546E-01	8.546E-01
Left pars orbitalis of inferior frontal gyrus	-0.172	0.050	[-0.270 - -0.074]	5.598E-04	1.655E-03
Right pars orbitalis of inferior frontal gyrus	-0.173	0.045	[-0.260 - -0.085]	1.118E-04	4.224E-04
Left medial orbitofrontal cortex	-0.155	0.058	[-0.268 - -0.041]	7.530E-03	1.600E-02
Right medial orbitofrontal cortex	-0.162	0.054	[-0.268 - -0.056]	2.755E-03	6.691E-03
Left inferior temporal gyrus	-0.079	0.035	[-0.147 - -0.011]	2.298E-02	3.907E-02
Right inferior temporal gyrus	-0.059	0.040	[-0.137 - 0.019]	1.354E-01	1.770E-01
Left isthmus cingulate cortex	-0.169	0.049	[-0.264 - -0.074]	5.039E-04	1.558E-03
Right isthmus cingulate cortex	-0.187	0.041	[-0.267 - -0.106]	5.158E-06	2.505E-05
Left banks of superior temporal sulcus	-0.097	0.040	[-0.176 - -0.018]	1.589E-02	2.844E-02
Right banks of superior temporal sulcus	-0.086	0.045	[-0.174 - 0.002]	5.682E-02	8.221E-02
Left supramarginal gyrus	0.045	0.057	[-0.067 - 0.158]	4.276E-01	4.668E-01
Right supramarginal gyrus	0.126	0.059	[0.010 - 0.242]	3.279E-02	5.438E-02
Left caudal middle frontal gyrus	0.035	0.044	[-0.052 - 0.121]	4.324E-01	4.668E-01
Right caudal middle frontal gyrus	0.007	0.035	[-0.060 - 0.075]	8.289E-01	8.413E-01
Left frontal pole	-0.121	0.043	[-0.206 - -0.036]	5.300E-03	1.243E-02
Right frontal pole	-0.140	0.057	[-0.251 - -0.029]	1.335E-02	2.522E-02
Left posterior cingulate cortex	-0.067	0.035	[-0.135 - 0.001]	5.310E-02	7.849E-02
Right posterior cingulate cortex	-0.101	0.051	[-0.202 - 0.000]	4.931E-02	7.452E-02
Left lateral occipital cortex	0.101	0.059	[-0.016 - 0.218]	8.926E-02	1.239E-01
Right lateral occipital cortex	0.078	0.051	[-0.022 - 0.177]	1.247E-01	1.663E-01
Left precentral gyrus	0.134	0.054	[0.029 - 0.240]	1.262E-02	2.452E-02
Right precentral gyrus	0.091	0.046	[0.000 - 0.181]	4.917E-02	7.452E-02
Left parahippocampal gyrus	-0.134	0.039	[-0.211 - -0.056]	7.160E-04	2.029E-03
Right parahippocampal gyrus	-0.137	0.035	[-0.205 - -0.069]	7.494E-05	3.185E-04
Left inferior parietal cortex	0.155	0.058	[0.042 - 0.268]	7.323E-03	1.600E-02
Right inferior parietal cortex	0.241	0.049	[0.144 - 0.338]	1.092E-06	7.392E-06
Left transverse temporal gyrus	-0.036	0.035	[-0.104 - 0.032]	3.000E-01	3.518E-01
Right transverse temporal gyrus	-0.039	0.044	[-0.126 - 0.047]	3.718E-01	4.213E-01
Left postcentral gyrus	0.152	0.055	[0.045 - 0.259]	5.548E-03	1.258E-02
Right postcentral gyrus	0.072	0.051	[-0.028 - 0.173]	1.561E-01	1.930E-01
Left precuneus	0.295	0.036	[0.225 - 0.366]	3.162E-16	2.150E-14
Right precuneus	0.229	0.060	[0.112 - 0.347]	1.337E-04	4.785E-04
Left caudal anterior cingulate cortex	0.010	0.041	[-0.070 - 0.091]	8.011E-01	8.254E-01
Right caudal anterior cingulate cortex	-0.024	0.035	[-0.092 - 0.044]	4.846E-01	5.069E-01
Left cuneus	0.091	0.035	[0.023 - 0.159]	8.626E-03	1.778E-02
Right cuneus	0.081	0.035	[0.013 - 0.149]	1.970E-02	3.436E-02
Left rostral anterior cingulate cortex	0.056	0.062	[-0.065 - 0.177]	3.649E-01	4.206E-01
Right rostral anterior cingulate cortex	0.107	0.035	[0.038 - 0.177]	2.443E-03	6.153E-03
Left pericalcarine cortex	0.045	0.052	[-0.057 - 0.147]	3.857E-01	4.300E-01
Right pericalcarine cortex	0.069	0.041	[-0.011 - 0.148]	9.252E-02	1.258E-01
Left paracentral lobule	0.307	0.048	[0.214 - 0.401]	1.106E-10	1.881E-09
Right paracentral lobule	0.265	0.055	[0.158 - 0.372]	1.193E-06	7.392E-06
Left superior parietal cortex	0.402	0.067	[0.270 - 0.533]	2.173E-09	2.463E-08
Right superior parietal cortex	0.440	0.070	[0.303 - 0.576]	2.562E-10	3.484E-09
Left temporal pole	0.085	0.058	[-0.028 - 0.198]	1.414E-01	1.781E-01
Right temporal pole	0.110	0.055	[0.002 - 0.217]	4.552E-02	7.369E-02
Left entorhinal cortex	0.064	0.037	[-0.009 - 0.136]	8.375E-02	1.186E-01
Right entorhinal cortex	0.058	0.046	[-0.032 - 0.147]	2.049E-01	2.488E-01

Supplementary Table S12. Cortical thickness differences between individuals with bipolar disorder and healthy comparison subjects controlling for age, sex, and global mean cortical thickness

	d	Std. Err.	95% CI	*p*-value	FDR *q*-value
Left fusiform gyrus	-0.290	0.094	[-0.475 - -0.106]	2.009E-03	3.415E-02
Right fusiform gyrus	-0.241	0.085	[-0.409 - -0.074]	4.677E-03	4.543E-02
Left pars opercularis of inferior frontal gyrus	-0.084	0.115	[-0.310 - 0.142]	4.663E-01	6.342E-01
Right pars opercularis of inferior frontal gyrus	-0.014	0.098	[-0.206 - 0.178]	8.865E-01	9.375E-01
Left superior temporal gyrus	-0.012	0.085	[-0.179 - 0.155]	8.847E-01	9.375E-01
Right superior temporal gyrus	0.060	0.107	[-0.149 - 0.269]	5.725E-01	7.209E-01
Left insula	-0.104	0.099	[-0.298 - 0.089]	2.915E-01	5.283E-01
Right insula	-0.223	0.085	[-0.390 - -0.056]	8.921E-03	7.583E-02
Left lingual gyrus	-0.072	0.089	[-0.246 - 0.101]	4.144E-01	6.180E-01
Right lingual gyrus	-0.107	0.105	[-0.313 - 0.099]	3.075E-01	5.283E-01
Left pars triangularis of inferior frontal gyrus	0.021	0.124	[-0.223 - 0.264]	8.678E-01	9.375E-01
Right pars triangularis of inferior frontal gyrus	-0.010	0.085	[-0.177 - 0.156]	9.027E-01	9.375E-01
Left lateral orbitofrontal cortex	-0.176	0.085	[-0.343 - -0.009]	3.912E-02	1.900E-01
Right lateral orbitofrontal cortex	-0.231	0.101	[-0.430 - -0.033]	2.210E-02	1.200E-01
Left rostral middle frontal gyrus	-0.141	0.146	[-0.427 - 0.145]	3.330E-01	5.392E-01
Right rostral middle frontal gyrus	0.076	0.110	[-0.140 - 0.292]	4.881E-01	6.382E-01
Left middle temporal gyrus	-0.067	0.095	[-0.254 - 0.119]	4.793E-01	6.382E-01
Right middle temporal gyrus	-0.214	0.144	[-0.497 - 0.068]	1.372E-01	3.751E-01
Left superior frontal gyrus	-0.076	0.086	[-0.245 - 0.092]	3.751E-01	5.932E-01
Right superior frontal gyrus	0.156	0.085	[-0.011 - 0.323]	6.701E-02	2.681E-01
Left pars orbitalis of inferior frontal gyrus	-0.294	0.090	[-0.470 - -0.117]	1.101E-03	2.533E-02
Right pars orbitalis of inferior frontal gyrus	-0.193	0.116	[-0.420 - 0.034]	9.531E-02	3.601E-01
Left medial orbitofrontal cortex	-0.201	0.133	[-0.461 - 0.060]	1.311E-01	3.751E-01
Right medial orbitofrontal cortex	-0.224	0.119	[-0.458 - 0.010]	6.112E-02	2.597E-01
Left inferior temporal gyrus	-0.194	0.085	[-0.361 - -0.027]	2.295E-02	1.200E-01
Right inferior temporal gyrus	-0.307	0.105	[-0.512 - -0.101]	3.442E-03	3.901E-02
Left isthmus cingulate cortex	-0.119	0.085	[-0.286 - 0.047]	1.608E-01	3.904E-01
Right isthmus cingulate cortex	-0.086	0.085	[-0.253 - 0.080]	3.108E-01	5.283E-01
Left banks of superior temporal sulcus	0.109	0.085	[-0.058 - 0.276]	2.002E-01	4.179E-01
Right banks of superior temporal sulcus	-0.044	0.085	[-0.210 - 0.123]	6.073E-01	7.509E-01
Left supramarginal gyrus	0.094	0.085	[-0.073 - 0.261]	2.693E-01	5.283E-01
Right supramarginal gyrus	-0.011	0.095	[-0.198 - 0.176]	9.099E-01	9.375E-01
Left caudal middle frontal gyrus	0.033	0.085	[-0.134 - 0.200]	6.960E-01	8.452E-01
Right caudal middle frontal gyrus	0.109	0.085	[-0.058 - 0.276]	2.003E-01	4.179E-01
Left frontal pole	-0.151	0.094	[-0.335 - 0.034]	1.095E-01	3.751E-01
Right frontal pole	-0.193	0.132	[-0.453 - 0.066]	1.434E-01	3.751E-01
Left posterior cingulate cortex	0.067	0.085	[-0.099 - 0.234]	4.277E-01	6.188E-01
Right posterior cingulate cortex	0.033	0.102	[-0.166 - 0.232]	7.431E-01	8.865E-01
Left lateral occipital cortex	-0.070	0.085	[-0.237 - 0.097]	4.145E-01	6.180E-01
Right lateral occipital cortex	-0.110	0.085	[-0.277 - 0.057]	1.976E-01	4.179E-01
Left precentral gyrus	0.022	0.085	[-0.145 - 0.189]	7.974E-01	9.190E-01
Right precentral gyrus	0.019	0.085	[-0.148 - 0.185]	8.259E-01	9.207E-01
Left parahippocampal gyrus	-0.020	0.085	[-0.186 - 0.147]	8.174E-01	9.207E-01
Right parahippocampal gyrus	0.006	0.091	[-0.172 - 0.183]	9.506E-01	9.648E-01
Left inferior parietal cortex	0.173	0.085	[0.006 - 0.340]	4.239E-02	1.922E-01
Right inferior parietal cortex	0.052	0.085	[-0.115 - 0.218]	5.444E-01	6.985E-01
Left transverse temporal gyrus	0.086	0.086	[-0.083 - 0.256]	3.193E-01	5.295E-01
Right transverse temporal gyrus	0.187	0.119	[-0.047 - 0.421]	1.168E-01	3.751E-01
Left postcentral gyrus	0.134	0.085	[-0.033 - 0.301]	1.168E-01	3.751E-01
Right postcentral gyrus	0.000	0.085	[-0.167 - 0.167]	9.974E-01	9.974E-01
Left precuneus	0.290	0.114	[0.066 - 0.515]	1.111E-02	8.395E-02
Right precuneus	0.299	0.092	[0.119 - 0.479]	1.118E-03	2.533E-02
Left caudal anterior cingulate cortex	-0.073	0.090	[-0.249 - 0.103]	4.181E-01	6.180E-01
Right caudal anterior cingulate cortex	0.076	0.104	[-0.127 - 0.279]	4.631E-01	6.342E-01
Left cuneus	0.138	0.094	[-0.045 - 0.321]	1.405E-01	3.751E-01
Right cuneus	0.087	0.085	[-0.080 - 0.254]	3.081E-01	5.283E-01
Left rostral anterior cingulate cortex	-0.205	0.085	[-0.372 - -0.037]	1.645E-02	1.119E-01
Right rostral anterior cingulate cortex	-0.062	0.085	[-0.229 - 0.105]	4.659E-01	6.342E-01
Left pericalcarine cortex	0.108	0.085	[-0.058 - 0.275]	2.028E-01	4.179E-01
Right pericalcarine cortex	0.121	0.085	[-0.046 - 0.289]	1.551E-01	3.904E-01
Left paracentral lobule	0.200	0.085	[0.033 - 0.367]	1.866E-02	1.154E-01
Right paracentral lobule	0.128	0.085	[-0.039 - 0.295]	1.320E-01	3.751E-01
Left superior parietal cortex	0.454	0.112	[0.234 - 0.674]	5.195E-05	3.533E-03
Right superior parietal cortex	0.306	0.103	[0.104 - 0.507]	2.931E-03	3.901E-02
Left temporal pole	-0.110	0.106	[-0.317 - 0.098]	2.991E-01	5.283E-01
Right temporal pole	-0.088	0.085	[-0.255 - 0.079]	3.031E-01	5.283E-01
Left entorhinal cortex	-0.108	0.085	[-0.275 - 0.058]	2.026E-01	4.179E-01
Right entorhinal cortex	-0.037	0.125	[-0.282 - 0.209]	7.683E-01	9.008E-01

Supplementary Table S13. Cortical thickness differences between individuals with bipolar disorder and healthy comparison subjects controlling for age, sex, and global mean cortical thickness at 25 years of age or older

	d	Std. Err.	95% CI	*p*-value	FDR *q*-value
Left fusiform gyrus	-0.257	0.093	[-0.439 - -0.074]	5.900E-03	6.686E-02
Right fusiform gyrus	-0.215	0.090	[-0.391 - -0.039]	1.678E-02	1.082E-01
Left pars opercularis of inferior frontal gyrus	-0.093	0.121	[-0.330 - 0.145]	4.448E-01	6.435E-01
Right pars opercularis of inferior frontal gyrus	-0.031	0.088	[-0.204 - 0.141]	7.219E-01	8.446E-01
Left superior temporal gyrus	0.024	0.103	[-0.179 - 0.226]	8.183E-01	8.920E-01
Right superior temporal gyrus	0.056	0.114	[-0.168 - 0.280]	6.215E-01	7.974E-01
Left insula	-0.101	0.088	[-0.273 - 0.072]	2.533E-01	4.533E-01
Right insula	-0.200	0.088	[-0.373 - -0.027]	2.312E-02	1.310E-01
Left lingual gyrus	0.021	0.088	[-0.152 - 0.193]	8.152E-01	8.920E-01
Right lingual gyrus	-0.019	0.088	[-0.191 - 0.154]	8.336E-01	8.920E-01
Left pars triangularis of inferior frontal gyrus	0.045	0.132	[-0.214 - 0.305]	7.328E-01	8.446E-01
Right pars triangularis of inferior frontal gyrus	-0.020	0.088	[-0.192 - 0.153]	8.226E-01	8.920E-01
Left lateral orbitofrontal cortex	-0.175	0.088	[-0.348 - -0.002]	4.748E-02	1.958E-01
Right lateral orbitofrontal cortex	-0.202	0.092	[-0.382 - -0.023]	2.717E-02	1.320E-01
Left rostral middle frontal gyrus	-0.166	0.159	[-0.477 - 0.146]	2.968E-01	4.806E-01
Right rostral middle frontal gyrus	0.023	0.127	[-0.226 - 0.272]	8.556E-01	8.920E-01
Left middle temporal gyrus	-0.035	0.088	[-0.207 - 0.138]	6.935E-01	8.446E-01
Right middle temporal gyrus	-0.212	0.150	[-0.506 - 0.081]	1.558E-01	3.794E-01
Left superior frontal gyrus	-0.131	0.088	[-0.303 - 0.042]	1.378E-01	3.605E-01
Right superior frontal gyrus	0.125	0.088	[-0.048 - 0.297]	1.562E-01	3.794E-01
Left pars orbitalis of inferior frontal gyrus	-0.306	0.088	[-0.480 - -0.133]	5.280E-04	1.197E-02
Right pars orbitalis of inferior frontal gyrus	-0.242	0.102	[-0.442 - -0.042]	1.751E-02	1.082E-01
Left medial orbitofrontal cortex	-0.181	0.132	[-0.440 - 0.078]	1.711E-01	3.878E-01
Right medial orbitofrontal cortex	-0.198	0.112	[-0.418 - 0.023]	7.847E-02	2.346E-01
Left inferior temporal gyrus	-0.220	0.088	[-0.393 - -0.047]	1.276E-02	9.644E-02
Right inferior temporal gyrus	-0.291	0.113	[-0.512 - -0.069]	1.006E-02	8.551E-02
Left isthmus cingulate cortex	-0.103	0.088	[-0.276 - 0.069]	2.416E-01	4.441E-01
Right isthmus cingulate cortex	-0.076	0.088	[-0.249 - 0.096]	3.856E-01	5.848E-01
Left banks of superior temporal sulcus	0.093	0.088	[-0.079 - 0.266]	2.906E-01	4.806E-01
Right banks of superior temporal sulcus	-0.031	0.088	[-0.203 - 0.142]	7.256E-01	8.446E-01
Left supramarginal gyrus	0.099	0.088	[-0.075 - 0.272]	2.644E-01	4.610E-01
Right supramarginal gyrus	-0.036	0.093	[-0.218 - 0.147]	7.007E-01	8.446E-01
Left caudal middle frontal gyrus	0.062	0.088	[-0.111 - 0.234]	4.816E-01	6.822E-01
Right caudal middle frontal gyrus	0.116	0.088	[-0.057 - 0.288]	1.887E-01	4.010E-01
Left frontal pole	-0.168	0.089	[-0.342 - 0.006]	5.835E-02	1.958E-01
Right frontal pole	-0.245	0.123	[-0.487 - -0.004]	4.663E-02	1.958E-01
Left posterior cingulate cortex	0.075	0.088	[-0.098 - 0.247]	3.956E-01	5.848E-01
Right posterior cingulate cortex	0.010	0.101	[-0.188 - 0.208]	9.214E-01	9.214E-01
Left lateral occipital cortex	-0.110	0.088	[-0.283 - 0.063]	2.120E-01	4.184E-01
Right lateral occipital cortex	-0.112	0.088	[-0.285 - 0.061]	2.031E-01	4.184E-01
Left precentral gyrus	0.045	0.088	[-0.127 - 0.218]	6.073E-01	7.974E-01
Right precentral gyrus	-0.010	0.088	[-0.182 - 0.163]	9.120E-01	9.214E-01
Left parahippocampal gyrus	-0.031	0.088	[-0.203 - 0.142]	7.283E-01	8.446E-01
Right parahippocampal gyrus	0.017	0.098	[-0.176 - 0.210]	8.658E-01	8.920E-01
Left inferior parietal cortex	0.169	0.088	[-0.004 - 0.342]	5.535E-02	1.958E-01
Right inferior parietal cortex	0.044	0.088	[-0.129 - 0.216]	6.195E-01	7.974E-01
Left transverse temporal gyrus	0.082	0.088	[-0.091 - 0.254]	3.532E-01	5.586E-01
Right transverse temporal gyrus	0.197	0.126	[-0.050 - 0.443]	1.174E-01	3.194E-01
Left postcentral gyrus	0.152	0.088	[-0.021 - 0.325]	8.435E-02	2.390E-01
Right postcentral gyrus	0.016	0.088	[-0.157 - 0.188]	8.573E-01	8.920E-01
Left precuneus	0.300	0.111	[0.082 - 0.518]	7.042E-03	6.841E-02
Right precuneus	0.343	0.097	[0.154 - 0.533]	3.776E-04	1.197E-02
Left caudal anterior cingulate cortex	-0.111	0.091	[-0.289 - 0.067]	2.206E-01	4.184E-01
Right caudal anterior cingulate cortex	0.059	0.104	[-0.144 - 0.263]	5.661E-01	7.856E-01
Left cuneus	0.165	0.088	[-0.007 - 0.338]	6.047E-02	1.958E-01
Right cuneus	0.094	0.088	[-0.078 - 0.267]	2.839E-01	4.806E-01
Left rostral anterior cingulate cortex	-0.258	0.088	[-0.431 - -0.084]	3.551E-03	4.829E-02
Right rostral anterior cingulate cortex	-0.044	0.088	[-0.216 - 0.128]	6.172E-01	7.974E-01
Left pericalcarine cortex	0.167	0.088	[-0.006 - 0.339]	5.821E-02	1.958E-01
Right pericalcarine cortex	0.155	0.088	[-0.018 - 0.327]	7.934E-02	2.346E-01
Left paracentral lobule	0.196	0.088	[0.023 - 0.369]	2.606E-02	1.320E-01
Right paracentral lobule	0.121	0.088	[-0.052 - 0.294]	1.692E-01	3.878E-01
Left superior parietal cortex	0.489	0.132	[0.230 - 0.748]	2.118E-04	1.197E-02
Right superior parietal cortex	0.326	0.096	[0.137 - 0.515]	7.144E-04	1.215E-02
Left temporal pole	-0.124	0.092	[-0.303 - 0.056]	1.773E-01	3.889E-01
Right temporal pole	-0.108	0.088	[-0.280 - 0.065]	2.215E-01	4.184E-01
Left entorhinal cortex	-0.166	0.088	[-0.339 - 0.007]	5.985E-02	1.958E-01
Right entorhinal cortex	-0.099	0.116	[-0.326 - 0.127]	3.896E-01	5.848E-01

Supplementary Table S14. Cortical thickness differences between individuals with major depressive disorder and healthy comparison subjects controlling for age, sex, and global mean cortical thickness

	d	Std. Err.	95% CI	*p*-value	FDR *q*-value
Left fusiform gyrus	-0.086	0.061	[-0.206 - 0.035]	1.627E-01	6.507E-01
Right fusiform gyrus	-0.033	0.061	[-0.153 - 0.088]	5.943E-01	8.785E-01
Left pars opercularis of inferior frontal gyrus	-0.066	0.088	[-0.237 - 0.106]	4.521E-01	8.308E-01
Right pars opercularis of inferior frontal gyrus	0.022	0.115	[-0.202 - 0.247]	8.454E-01	9.888E-01
Left superior temporal gyrus	-0.137	0.061	[-0.257 - -0.017]	2.536E-02	2.155E-01
Right superior temporal gyrus	0.000	0.061	[-0.120 - 0.121]	9.942E-01	9.942E-01
Left insula	-0.067	0.064	[-0.192 - 0.058]	2.904E-01	7.478E-01
Right insula	-0.086	0.074	[-0.231 - 0.058]	2.409E-01	7.478E-01
Left lingual gyrus	-0.074	0.063	[-0.197 - 0.049]	2.405E-01	7.478E-01
Right lingual gyrus	-0.025	0.068	[-0.159 - 0.108]	7.114E-01	9.051E-01
Left pars triangularis of inferior frontal gyrus	0.003	0.061	[-0.118 - 0.123]	9.635E-01	9.942E-01
Right pars triangularis of inferior frontal gyrus	-0.013	0.081	[-0.172 - 0.145]	8.691E-01	9.888E-01
Left lateral orbitofrontal cortex	-0.185	0.076	[-0.334 - -0.036]	1.489E-02	1.687E-01
Right lateral orbitofrontal cortex	-0.073	0.069	[-0.208 - 0.062]	2.903E-01	7.478E-01
Left rostral middle frontal gyrus	-0.138	0.068	[-0.271 - -0.004]	4.364E-02	2.967E-01
Right rostral middle frontal gyrus	0.057	0.061	[-0.063 - 0.177]	3.525E-01	7.901E-01
Left middle temporal gyrus	-0.209	0.077	[-0.360 - -0.057]	7.022E-03	1.217E-01
Right middle temporal gyrus	-0.116	0.079	[-0.271 - 0.039]	1.428E-01	6.507E-01
Left superior frontal gyrus	-0.039	0.061	[-0.159 - 0.081]	5.229E-01	8.330E-01
Right superior frontal gyrus	-0.001	0.063	[-0.124 - 0.121]	9.842E-01	9.942E-01
Left pars orbitalis of inferior frontal gyrus	-0.178	0.061	[-0.298 - -0.058]	3.756E-03	1.217E-01
Right pars orbitalis of inferior frontal gyrus	-0.170	0.065	[-0.298 - -0.043]	8.948E-03	1.217E-01
Left medial orbitofrontal cortex	-0.150	0.070	[-0.287 - -0.014]	3.071E-02	2.320E-01
Right medial orbitofrontal cortex	-0.110	0.076	[-0.259 - 0.038]	1.454E-01	6.507E-01
Left inferior temporal gyrus	-0.043	0.061	[-0.163 - 0.077]	4.830E-01	8.330E-01
Right inferior temporal gyrus	-0.038	0.064	[-0.164 - 0.088]	5.506E-01	8.330E-01
Left isthmus cingulate cortex	-0.038	0.061	[-0.159 - 0.082]	5.322E-01	8.330E-01
Right isthmus cingulate cortex	-0.022	0.061	[-0.142 - 0.099]	7.256E-01	9.051E-01
Left banks of superior temporal sulcus	-0.030	0.061	[-0.150 - 0.090]	6.270E-01	8.805E-01
Right banks of superior temporal sulcus	0.007	0.075	[-0.140 - 0.153]	9.286E-01	9.942E-01
Left supramarginal gyrus	0.010	0.069	[-0.125 - 0.145]	8.870E-01	9.888E-01
Right supramarginal gyrus	0.024	0.061	[-0.096 - 0.145]	6.913E-01	9.040E-01
Left caudal middle frontal gyrus	0.047	0.074	[-0.099 - 0.193]	5.273E-01	8.330E-01
Right caudal middle frontal gyrus	-0.029	0.073	[-0.172 - 0.114]	6.904E-01	9.040E-01
Left frontal pole	-0.029	0.061	[-0.150 - 0.091]	6.345E-01	8.805E-01
Right frontal pole	-0.051	0.084	[-0.215 - 0.114]	5.458E-01	8.330E-01
Left posterior cingulate cortex	-0.069	0.063	[-0.193 - 0.056]	2.798E-01	7.478E-01
Right posterior cingulate cortex	0.010	0.067	[-0.122 - 0.142]	8.808E-01	9.888E-01
Left lateral occipital cortex	0.037	0.062	[-0.084 - 0.158]	5.513E-01	8.330E-01
Right lateral occipital cortex	0.061	0.067	[-0.070 - 0.193]	3.593E-01	7.901E-01
Left precentral gyrus	-0.097	0.074	[-0.241 - 0.047]	1.880E-01	7.102E-01
Right precentral gyrus	-0.005	0.061	[-0.125 - 0.115]	9.325E-01	9.942E-01
Left parahippocampal gyrus	-0.078	0.083	[-0.241 - 0.085]	3.453E-01	7.901E-01
Right parahippocampal gyrus	0.044	0.061	[-0.076 - 0.164]	4.741E-01	8.330E-01
Left inferior parietal cortex	0.087	0.061	[-0.033 - 0.208]	1.546E-01	6.507E-01
Right inferior parietal cortex	0.102	0.088	[-0.070 - 0.274]	2.442E-01	7.478E-01
Left transverse temporal gyrus	0.024	0.071	[-0.115 - 0.164]	7.321E-01	9.051E-01
Right transverse temporal gyrus	0.060	0.077	[-0.090 - 0.210]	4.341E-01	8.308E-01
Left postcentral gyrus	0.034	0.077	[-0.118 - 0.185]	6.636E-01	9.025E-01
Right postcentral gyrus	-0.001	0.061	[-0.121 - 0.119]	9.840E-01	9.942E-01
Left precuneus	0.164	0.062	[0.043 - 0.284]	7.833E-03	1.217E-01
Right precuneus	0.076	0.073	[-0.067 - 0.219]	2.969E-01	7.478E-01
Left caudal anterior cingulate cortex	0.013	0.061	[-0.107 - 0.134]	8.266E-01	9.888E-01
Right caudal anterior cingulate cortex	0.029	0.061	[-0.091 - 0.149]	6.335E-01	8.805E-01
Left cuneus	0.053	0.061	[-0.067 - 0.174]	3.834E-01	7.901E-01
Right cuneus	0.077	0.061	[-0.043 - 0.197]	2.109E-01	7.478E-01
Left rostral anterior cingulate cortex	0.003	0.077	[-0.148 - 0.154]	9.708E-01	9.942E-01
Right rostral anterior cingulate cortex	0.108	0.061	[-0.013 - 0.228]	7.968E-02	4.926E-01
Left pericalcarine cortex	0.054	0.061	[-0.067 - 0.174]	3.811E-01	7.901E-01
Right pericalcarine cortex	0.108	0.070	[-0.028 - 0.245]	1.200E-01	6.275E-01
Left paracentral lobule	0.054	0.061	[-0.066 - 0.174]	3.797E-01	7.901E-01
Right paracentral lobule	0.087	0.077	[-0.064 - 0.238]	2.611E-01	7.478E-01
Left superior parietal cortex	0.166	0.098	[-0.026 - 0.358]	8.958E-02	5.076E-01
Right superior parietal cortex	0.235	0.073	[0.092 - 0.378]	1.304E-03	8.870E-02
Left temporal pole	0.051	0.061	[-0.069 - 0.172]	4.037E-01	8.074E-01
Right temporal pole	0.189	0.084	[0.025 - 0.353]	2.380E-02	2.155E-01
Left entorhinal cortex	0.018	0.086	[-0.151 - 0.187]	8.383E-01	9.888E-01
Right entorhinal cortex	0.067	0.088	[-0.106 - 0.240]	4.463E-01	8.308E-01

Supplementary Table S15. Cortical thickness differences between individuals with major depressive disorder and healthy comparison subjects controlling for age, sex, and global mean cortical thickness over 21 years old

	d	Std. Err.	95% CI	*p*-value	FDR *q*-value
Left fusiform gyrus	-0.079	0.063	[-0.203 - 0.045]	2.107E-01	6.433E-01
Right fusiform gyrus	-0.036	0.063	[-0.160 - 0.088]	5.682E-01	7.885E-01
Left pars opercularis of inferior frontal gyrus	-0.095	0.081	[-0.254 - 0.064]	2.423E-01	6.447E-01
Right pars opercularis of inferior frontal gyrus	-0.006	0.114	[-0.229 - 0.216]	9.545E-01	9.794E-01
Left superior temporal gyrus	-0.135	0.063	[-0.259 - -0.012]	3.211E-02	2.426E-01
Right superior temporal gyrus	0.006	0.063	[-0.118 - 0.129]	9.276E-01	9.794E-01
Left insula	-0.057	0.066	[-0.186 - 0.072]	3.861E-01	7.524E-01
Right insula	-0.076	0.068	[-0.210 - 0.058]	2.644E-01	6.447E-01
Left lingual gyrus	-0.036	0.066	[-0.166 - 0.093]	5.807E-01	7.898E-01
Right lingual gyrus	0.012	0.068	[-0.121 - 0.144]	8.633E-01	9.694E-01
Left pars triangularis of inferior frontal gyrus	-0.004	0.063	[-0.128 - 0.119]	9.471E-01	9.794E-01
Right pars triangularis of inferior frontal gyrus	-0.035	0.078	[-0.188 - 0.119]	6.590E-01	8.584E-01
Left lateral orbitofrontal cortex	-0.200	0.081	[-0.359 - -0.042]	1.313E-02	1.325E-01
Right lateral orbitofrontal cortex	-0.125	0.063	[-0.249 - -0.001]	4.803E-02	2.969E-01
Left rostral middle frontal gyrus	-0.183	0.069	[-0.318 - -0.048]	7.849E-03	1.183E-01
Right rostral middle frontal gyrus	0.048	0.065	[-0.080 - 0.176]	4.621E-01	7.524E-01
Left middle temporal gyrus	-0.246	0.082	[-0.407 - -0.084]	2.873E-03	6.512E-02
Right middle temporal gyrus	-0.137	0.077	[-0.287 - 0.013]	7.395E-02	4.191E-01
Left superior frontal gyrus	-0.051	0.063	[-0.175 - 0.073]	4.180E-01	7.524E-01
Right superior frontal gyrus	-0.001	0.063	[-0.125 - 0.122]	9.843E-01	9.843E-01
Left pars orbitalis of inferior frontal gyrus	-0.166	0.063	[-0.289 - -0.042]	8.700E-03	1.183E-01
Right pars orbitalis of inferior frontal gyrus	-0.191	0.063	[-0.315 - -0.068]	2.442E-03	6.512E-02
Left medial orbitofrontal cortex	-0.129	0.074	[-0.275 - 0.016]	8.201E-02	4.290E-01
Right medial orbitofrontal cortex	-0.114	0.084	[-0.278 - 0.051]	1.767E-01	6.158E-01
Left inferior temporal gyrus	-0.027	0.063	[-0.150 - 0.097]	6.727E-01	8.584E-01
Right inferior temporal gyrus	-0.045	0.067	[-0.175 - 0.086]	5.024E-01	7.524E-01
Left isthmus cingulate cortex	-0.042	0.063	[-0.165 - 0.082]	5.090E-01	7.524E-01
Right isthmus cingulate cortex	-0.030	0.063	[-0.154 - 0.093]	6.311E-01	8.415E-01
Left banks of superior temporal sulcus	-0.047	0.063	[-0.171 - 0.077]	4.561E-01	7.524E-01
Right banks of superior temporal sulcus	0.003	0.063	[-0.121 - 0.127]	9.650E-01	9.794E-01
Left supramarginal gyrus	-0.015	0.074	[-0.161 - 0.130]	8.348E-01	9.694E-01
Right supramarginal gyrus	0.039	0.063	[-0.084 - 0.163]	5.345E-01	7.677E-01
Left caudal middle frontal gyrus	0.024	0.072	[-0.117 - 0.164]	7.422E-01	9.088E-01
Right caudal middle frontal gyrus	-0.048	0.071	[-0.188 - 0.092]	5.012E-01	7.524E-01
Left frontal pole	-0.053	0.063	[-0.177 - 0.071]	4.018E-01	7.524E-01
Right frontal pole	-0.077	0.089	[-0.251 - 0.097]	3.874E-01	7.524E-01
Left posterior cingulate cortex	-0.081	0.063	[-0.205 - 0.042]	1.977E-01	6.402E-01
Right posterior cingulate cortex	-0.004	0.074	[-0.149 - 0.141]	9.559E-01	9.794E-01
Left lateral occipital cortex	0.062	0.063	[-0.061 - 0.186]	3.237E-01	6.878E-01
Right lateral occipital cortex	0.089	0.066	[-0.041 - 0.219]	1.811E-01	6.158E-01
Left precentral gyrus	-0.083	0.079	[-0.237 - 0.071]	2.927E-01	6.635E-01
Right precentral gyrus	-0.004	0.063	[-0.127 - 0.120]	9.545E-01	9.794E-01
Left parahippocampal gyrus	-0.060	0.077	[-0.212 - 0.091]	4.366E-01	7.524E-01
Right parahippocampal gyrus	0.051	0.063	[-0.073 - 0.174]	4.217E-01	7.524E-01
Left inferior parietal cortex	0.088	0.063	[-0.036 - 0.212]	1.625E-01	6.158E-01
Right inferior parietal cortex	0.104	0.085	[-0.063 - 0.270]	2.226E-01	6.433E-01
Left transverse temporal gyrus	0.030	0.073	[-0.114 - 0.174]	6.816E-01	8.584E-01
Right transverse temporal gyrus	0.082	0.080	[-0.075 - 0.239]	3.056E-01	6.704E-01
Left postcentral gyrus	0.055	0.074	[-0.091 - 0.200]	4.620E-01	7.524E-01
Right postcentral gyrus	0.012	0.063	[-0.112 - 0.135]	8.543E-01	9.694E-01
Left precuneus	0.153	0.063	[0.029 - 0.277]	1.559E-02	1.325E-01
Right precuneus	0.084	0.075	[-0.063 - 0.231]	2.634E-01	6.447E-01
Left caudal anterior cingulate cortex	-0.013	0.063	[-0.137 - 0.110]	8.345E-01	9.694E-01
Right caudal anterior cingulate cortex	0.010	0.063	[-0.113 - 0.134]	8.696E-01	9.694E-01
Left cuneus	0.070	0.063	[-0.053 - 0.194]	2.655E-01	6.447E-01
Right cuneus	0.095	0.063	[-0.028 - 0.219]	1.314E-01	5.583E-01
Left rostral anterior cingulate cortex	-0.025	0.077	[-0.175 - 0.126]	7.484E-01	9.088E-01
Right rostral anterior cingulate cortex	0.099	0.063	[-0.025 - 0.222]	1.179E-01	5.346E-01
Left pericalcarine cortex	0.067	0.063	[-0.057 - 0.190]	2.899E-01	6.635E-01
Right pericalcarine cortex	0.122	0.072	[-0.019 - 0.264]	9.072E-02	4.406E-01
Left paracentral lobule	0.076	0.063	[-0.047 - 0.200]	2.271E-01	6.433E-01
Right paracentral lobule	0.114	0.084	[-0.051 - 0.278]	1.758E-01	6.158E-01
Left superior parietal cortex	0.216	0.088	[0.042 - 0.389]	1.469E-02	1.325E-01
Right superior parietal cortex	0.255	0.076	[0.106 - 0.403]	7.864E-04	5.347E-02
Left temporal pole	0.039	0.063	[-0.085 - 0.162]	5.419E-01	7.677E-01
Right temporal pole	0.171	0.086	[0.002 - 0.340]	4.791E-02	2.969E-01
Left entorhinal cortex	0.055	0.083	[-0.107 - 0.218]	5.038E-01	7.524E-01
Right entorhinal cortex	0.065	0.090	[-0.111 - 0.240]	4.724E-01	7.524E-01

Supplementary Table S16. Cortical thickness differences between individuals with autism spectrum disorder and healthy comparison subjects controlling for age, sex, and global mean cortical thickness

	d	Std. Err.	95% CI	*p*-value	FDR *q*-value
Left fusiform gyrus	-0.108	0.080	[-0.265 - 0.049]	1.790E-01	8.969E-01
Right fusiform gyrus	-0.261	0.120	[-0.496 - -0.027]	2.909E-02	3.608E-01
Left pars opercularis of inferior frontal gyrus	0.115	0.080	[-0.042 - 0.272]	1.498E-01	8.969E-01
Right pars opercularis of inferior frontal gyrus	-0.017	0.080	[-0.174 - 0.140]	8.279E-01	9.880E-01
Left superior temporal gyrus	0.087	0.120	[-0.148 - 0.323]	4.662E-01	9.004E-01
Right superior temporal gyrus	0.098	0.080	[-0.059 - 0.255]	2.203E-01	8.969E-01
Left insula	0.026	0.090	[-0.150 - 0.202]	7.750E-01	9.880E-01
Right insula	-0.057	0.080	[-0.214 - 0.100]	4.757E-01	9.004E-01
Left lingual gyrus	-0.182	0.083	[-0.346 - -0.019]	2.904E-02	3.608E-01
Right lingual gyrus	-0.172	0.080	[-0.329 - -0.015]	3.184E-02	3.608E-01
Left pars triangularis of inferior frontal gyrus	0.253	0.109	[0.039 - 0.467]	2.032E-02	3.608E-01
Right pars triangularis of inferior frontal gyrus	0.065	0.080	[-0.091 - 0.222]	4.145E-01	9.004E-01
Left lateral orbitofrontal cortex	0.049	0.082	[-0.112 - 0.209]	5.520E-01	9.004E-01
Right lateral orbitofrontal cortex	-0.210	0.080	[-0.367 - -0.053]	8.692E-03	2.955E-01
Left rostral middle frontal gyrus	0.076	0.080	[-0.080 - 0.233]	3.397E-01	9.004E-01
Right rostral middle frontal gyrus	0.095	0.092	[-0.086 - 0.276]	3.025E-01	8.969E-01
Left middle temporal gyrus	0.002	0.127	[-0.246 - 0.250]	9.880E-01	9.880E-01
Right middle temporal gyrus	-0.006	0.114	[-0.230 - 0.218]	9.563E-01	9.880E-01
Left superior frontal gyrus	0.089	0.080	[-0.068 - 0.246]	2.663E-01	8.969E-01
Right superior frontal gyrus	0.097	0.091	[-0.080 - 0.275]	2.827E-01	8.969E-01
Left pars orbitalis of inferior frontal gyrus	-0.012	0.136	[-0.278 - 0.254]	9.314E-01	9.880E-01
Right pars orbitalis of inferior frontal gyrus	-0.069	0.109	[-0.284 - 0.145]	5.257E-01	9.004E-01
Left medial orbitofrontal cortex	-0.046	0.096	[-0.234 - 0.142]	6.308E-01	9.127E-01
Right medial orbitofrontal cortex	0.043	0.083	[-0.119 - 0.206]	6.008E-01	9.004E-01
Left inferior temporal gyrus	-0.118	0.143	[-0.399 - 0.162]	4.080E-01	9.004E-01
Right inferior temporal gyrus	-0.035	0.115	[-0.260 - 0.190]	7.627E-01	9.880E-01
Left isthmus cingulate cortex	0.047	0.080	[-0.110 - 0.204]	5.566E-01	9.004E-01
Right isthmus cingulate cortex	-0.060	0.090	[-0.236 - 0.117]	5.067E-01	9.004E-01
Left banks of superior temporal sulcus	0.156	0.122	[-0.084 - 0.396]	2.017E-01	8.969E-01
Right banks of superior temporal sulcus	0.026	0.092	[-0.155 - 0.207]	7.792E-01	9.880E-01
Left supramarginal gyrus	-0.127	0.099	[-0.321 - 0.067]	1.988E-01	8.969E-01
Right supramarginal gyrus	-0.013	0.080	[-0.170 - 0.143]	8.670E-01	9.880E-01
Left caudal middle frontal gyrus	0.026	0.097	[-0.164 - 0.216]	7.899E-01	9.880E-01
Right caudal middle frontal gyrus	0.113	0.151	[-0.184 - 0.410]	4.552E-01	9.004E-01
Left frontal pole	0.035	0.130	[-0.220 - 0.291]	7.861E-01	9.880E-01
Right frontal pole	-0.015	0.080	[-0.172 - 0.142]	8.514E-01	9.880E-01
Left posterior cingulate cortex	0.241	0.080	[0.084 - 0.398]	2.686E-03	1.827E-01
Right posterior cingulate cortex	0.072	0.117	[-0.157 - 0.301]	5.368E-01	9.004E-01
Left lateral occipital cortex	-0.083	0.080	[-0.240 - 0.074]	2.983E-01	8.969E-01
Right lateral occipital cortex	-0.059	0.080	[-0.216 - 0.098]	4.639E-01	9.004E-01
Left precentral gyrus	-0.094	0.080	[-0.251 - 0.063]	2.402E-01	8.969E-01
Right precentral gyrus	-0.135	0.084	[-0.300 - 0.030]	1.094E-01	8.969E-01
Left parahippocampal gyrus	-0.041	0.080	[-0.198 - 0.116]	6.091E-01	9.004E-01
Right parahippocampal gyrus	0.084	0.080	[-0.073 - 0.241]	2.946E-01	8.969E-01
Left inferior parietal cortex	-0.082	0.080	[-0.239 - 0.074]	3.034E-01	8.969E-01
Right inferior parietal cortex	0.003	0.080	[-0.153 - 0.160]	9.653E-01	9.880E-01
Left transverse temporal gyrus	0.094	0.080	[-0.063 - 0.251]	2.393E-01	8.969E-01
Right transverse temporal gyrus	-0.032	0.128	[-0.282 - 0.219]	8.054E-01	9.880E-01
Left postcentral gyrus	-0.047	0.086	[-0.216 - 0.121]	5.806E-01	9.004E-01
Right postcentral gyrus	-0.008	0.117	[-0.237 - 0.221]	9.462E-01	9.880E-01
Left precuneus	0.091	0.104	[-0.114 - 0.295]	3.854E-01	9.004E-01
Right precuneus	0.005	0.080	[-0.152 - 0.162]	9.497E-01	9.880E-01
Left caudal anterior cingulate cortex	-0.002	0.080	[-0.158 - 0.155]	9.845E-01	9.880E-01
Right caudal anterior cingulate cortex	-0.065	0.101	[-0.263 - 0.134]	5.216E-01	9.004E-01
Left cuneus	0.091	0.108	[-0.120 - 0.303]	3.966E-01	9.004E-01
Right cuneus	0.106	0.080	[-0.051 - 0.263]	1.843E-01	8.969E-01
Left rostral anterior cingulate cortex	-0.002	0.084	[-0.167 - 0.164]	9.839E-01	9.880E-01
Right rostral anterior cingulate cortex	0.068	0.083	[-0.095 - 0.231]	4.133E-01	9.004E-01
Left pericalcarine cortex	0.046	0.080	[-0.111 - 0.202]	5.688E-01	9.004E-01
Right pericalcarine cortex	-0.020	0.080	[-0.177 - 0.136]	7.986E-01	9.880E-01
Left paracentral lobule	0.029	0.080	[-0.128 - 0.186]	7.195E-01	9.880E-01
Right paracentral lobule	-0.109	0.082	[-0.270 - 0.052]	1.835E-01	8.969E-01
Left superior parietal cortex	0.005	0.125	[-0.240 - 0.249]	9.704E-01	9.880E-01
Right superior parietal cortex	0.071	0.132	[-0.187 - 0.329]	5.902E-01	9.004E-01
Left temporal pole	0.056	0.080	[-0.101 - 0.213]	4.857E-01	9.004E-01
Right temporal pole	0.131	0.104	[-0.072 - 0.335]	2.064E-01	8.969E-01
Left entorhinal cortex	0.005	0.085	[-0.161 - 0.172]	9.494E-01	9.880E-01
Right entorhinal cortex	0.066	0.080	[-0.091 - 0.223]	4.071E-01	9.004E-01

Supplementary Table S17. Cortical surface area differences between individuals with schizophrenia and healthy comparison subjects controlling for age and sex

	d	Std. Err.	95% CI	*p*-value	FDR *q*-value
Total cortical surface area	-0.350	0.040	[-0.429 - -0.271]	3.769E-18	6.690E-17
Left hemisphere	-0.346	0.041	[-0.426 - -0.266]	1.999E-17	2.365E-16
Right hemisphere	-0.351	0.040	[-0.430 - -0.273]	1.283E-18	3.037E-17
Left superior frontal gyrus	-0.347	0.041	[-0.427 - -0.267]	1.471E-17	2.088E-16
Right superior frontal gyrus	-0.321	0.035	[-0.389 - -0.252]	3.706E-20	1.389E-18
Left pars orbitalis of inferior frontal gyrus	-0.320	0.035	[-0.389 - -0.252]	3.914E-20	1.389E-18
Right pars orbitalis of inferior frontal gyrus	-0.286	0.035	[-0.354 - -0.218]	2.191E-16	1.945E-15
Left precentral gyrus	-0.289	0.035	[-0.357 - -0.221]	1.053E-16	1.068E-15
Right precentral gyrus	-0.250	0.042	[-0.331 - -0.168]	2.394E-09	7.081E-09
Left lingual gyrus	-0.272	0.035	[-0.340 - -0.203]	5.956E-15	4.699E-14
Right lingual gyrus	-0.280	0.037	[-0.354 - -0.207]	6.716E-14	3.406E-13
Left superior temporal gyrus	-0.212	0.044	[-0.298 - -0.127]	1.154E-06	1.820E-06
Right superior temporal gyrus	-0.272	0.036	[-0.342 - -0.201]	3.902E-14	2.771E-13
Left fusiform gyrus	-0.266	0.045	[-0.354 - -0.178]	2.845E-09	8.079E-09
Right fusiform gyrus	-0.250	0.047	[-0.343 - -0.157]	1.322E-07	2.536E-07
Left inferior parietal cortex	-0.203	0.036	[-0.274 - -0.132]	2.151E-08	4.628E-08
Right inferior parietal cortex	-0.266	0.042	[-0.347 - -0.184]	1.855E-10	7.315E-10
Left lateral occipital cortex	-0.231	0.037	[-0.303 - -0.158]	4.365E-10	1.550E-09
Right lateral occipital cortex	-0.263	0.035	[-0.332 - -0.195]	6.082E-14	3.406E-13
Left rostral middle frontal gyrus	-0.261	0.035	[-0.329 - -0.193]	6.412E-14	3.406E-13
Right rostral middle frontal gyrus	-0.244	0.035	[-0.312 - -0.176]	2.159E-12	1.022E-11
Left precuneus	-0.261	0.035	[-0.329 - -0.193]	6.633E-14	3.406E-13
Right precuneus	-0.244	0.035	[-0.312 - -0.175]	2.502E-12	1.110E-11
Left inferior temporal gyrus	-0.259	0.051	[-0.359 - -0.159]	3.589E-07	6.067E-07
Right inferior temporal gyrus	-0.227	0.052	[-0.329 - -0.126]	1.194E-05	1.570E-05
Left lateral orbitofrontal cortex	-0.252	0.041	[-0.332 - -0.172]	7.306E-10	2.470E-09
Right lateral orbitofrontal cortex	-0.199	0.035	[-0.267 - -0.131]	1.081E-08	2.554E-08
Left middle temporal gyrus	-0.198	0.044	[-0.283 - -0.112]	5.859E-06	8.157E-06
Right middle temporal gyrus	-0.244	0.041	[-0.323 - -0.164]	1.919E-09	6.127E-09
Left postcentral gyrus	-0.241	0.046	[-0.331 - -0.151]	1.372E-07	2.564E-07
Right postcentral gyrus	-0.219	0.038	[-0.294 - -0.144]	1.014E-08	2.554E-08
Left medial orbitofrontal cortex	-0.131	0.040	[-0.208 - -0.053]	9.270E-04	1.062E-03
Right medial orbitofrontal cortex	-0.224	0.044	[-0.311 - -0.138]	4.240E-07	7.000E-07
Left cuneus	-0.208	0.037	[-0.282 - -0.135]	2.461E-08	5.140E-08
Right cuneus	-0.223	0.035	[-0.291 - -0.155]	1.417E-10	5.920E-10
Left pars triangularis of inferior frontal gyrus	-0.218	0.035	[-0.286 - -0.150]	3.471E-10	1.297E-09
Right pars triangularis of inferior frontal gyrus	-0.215	0.036	[-0.286 - -0.145]	1.985E-09	6.127E-09
Left superior parietal cortex	-0.205	0.035	[-0.273 - -0.137]	3.795E-09	1.036E-08
Right superior parietal cortex	-0.217	0.038	[-0.291 - -0.142]	1.115E-08	2.554E-08
Left pars opercularis of inferior frontal gyrus	-0.215	0.046	[-0.305 - -0.125]	2.576E-06	3.976E-06
Right pars opercularis of inferior frontal gyrus	-0.199	0.035	[-0.267 - -0.130]	1.096E-08	2.554E-08
Left supramarginal gyrus	-0.201	0.035	[-0.269 - -0.133]	6.991E-09	1.838E-08
Right supramarginal gyrus	-0.183	0.035	[-0.251 - -0.115]	1.413E-07	2.573E-07
Left pericalcarine cortex	-0.187	0.042	[-0.269 - -0.105]	7.907E-06	1.059E-05
Right pericalcarine cortex	-0.201	0.036	[-0.270 - -0.131]	1.683E-08	3.735E-08
Left parahippocampal gyrus	-0.138	0.048	[-0.231 - -0.044]	3.925E-03	4.288E-03
Right parahippocampal gyrus	-0.194	0.038	[-0.268 - -0.120]	3.164E-07	5.615E-07
Left caudal middle frontal gyrus	-0.193	0.042	[-0.275 - -0.111]	3.747E-06	5.542E-06
Right caudal middle frontal gyrus	-0.167	0.039	[-0.243 - -0.091]	1.744E-05	2.211E-05
Left transverse temporal gyrus	-0.193	0.035	[-0.261 - -0.125]	2.861E-08	5.803E-08
Right transverse temporal gyrus	-0.158	0.035	[-0.226 - -0.090]	5.352E-06	7.600E-06
Left banks of superior temporal sulcus	-0.184	0.035	[-0.252 - -0.116]	1.214E-07	2.395E-07
Right banks of superior temporal sulcus	-0.186	0.047	[-0.279 - -0.093]	8.582E-05	1.033E-04
Left caudal anterior cingulate cortex	-0.151	0.035	[-0.219 - -0.083]	1.296E-05	1.673E-05
Right caudal anterior cingulate cortex	-0.182	0.040	[-0.261 - -0.103]	6.475E-06	8.841E-06
Left rostral anterior cingulate cortex	-0.181	0.039	[-0.258 - -0.104]	3.989E-06	5.780E-06
Right rostral anterior cingulate cortex	-0.179	0.036	[-0.250 - -0.108]	8.162E-07	1.317E-06
Left posterior cingulate cortex	-0.177	0.035	[-0.245 - -0.109]	3.522E-07	6.067E-07
Right posterior cingulate cortex	-0.165	0.047	[-0.256 - -0.073]	4.352E-04	5.066E-04
Left frontal pole	-0.114	0.035	[-0.182 - -0.046]	9.813E-04	1.106E-03
Right frontal pole	-0.168	0.040	[-0.247 - -0.090]	2.717E-05	3.385E-05
Left paracentral lobule	-0.138	0.035	[-0.206 - -0.070]	6.648E-05	8.138E-05
Right paracentral lobule	-0.162	0.035	[-0.231 - -0.094]	2.854E-06	4.311E-06
Left insula	-0.158	0.049	[-0.254 - -0.061]	1.324E-03	1.468E-03
Right insula	-0.157	0.042	[-0.240 - -0.074]	2.113E-04	2.501E-04
Left entorhinal cortex	-0.113	0.055	[-0.220 - -0.006]	3.814E-02	4.103E-02
Right entorhinal cortex	-0.052	0.041	[-0.132 - 0.028]	2.062E-01	2.153E-01
Left temporal pole	-0.077	0.049	[-0.172 - 0.019]	1.161E-01	1.230E-01
Right temporal pole	-0.059	0.052	[-0.161 - 0.043]	2.578E-01	2.615E-01
Left isthmus cingulate cortex	-0.035	0.035	[-0.104 - 0.033]	3.125E-01	3.125E-01
Right isthmus cingulate cortex	-0.043	0.035	[-0.111 - 0.025]	2.124E-01	2.185E-01

Supplementary Table S18. Cortical surface area differences between individuals with bipolar disorder and healthy comparison subjects controlling for age and sex

	d	Std. Err.	95% CI	*p*-value	FDR *q*-value
Total cortical surface area	0.005	0.095	[-0.181 - 0.191]	9.581E-01	9.597E-01
Left hemisphere	0.015	0.090	[-0.162 - 0.191]	8.709E-01	9.597E-01
Right hemisphere	-0.006	0.100	[-0.202 - 0.191]	9.540E-01	9.597E-01
Left superior frontal gyrus	-0.049	0.090	[-0.225 - 0.126]	5.807E-01	9.597E-01
Right superior frontal gyrus	-0.102	0.085	[-0.269 - 0.065]	2.299E-01	9.597E-01
Left pars orbitalis of inferior frontal gyrus	0.041	0.101	[-0.157 - 0.239]	6.856E-01	9.597E-01
Right pars orbitalis of inferior frontal gyrus	-0.195	0.085	[-0.362 - -0.028]	2.222E-02	7.887E-01
Left precentral gyrus	-0.064	0.175	[-0.406 - 0.279]	7.158E-01	9.597E-01
Right precentral gyrus	-0.113	0.116	[-0.340 - 0.114]	3.299E-01	9.597E-01
Left lingual gyrus	0.013	0.119	[-0.220 - 0.246]	9.112E-01	9.597E-01
Right lingual gyrus	0.049	0.085	[-0.118 - 0.215]	5.676E-01	9.597E-01
Left superior temporal gyrus	-0.009	0.097	[-0.199 - 0.181]	9.262E-01	9.597E-01
Right superior temporal gyrus	-0.022	0.085	[-0.189 - 0.145]	7.977E-01	9.597E-01
Left fusiform gyrus	-0.014	0.085	[-0.180 - 0.153]	8.735E-01	9.597E-01
Right fusiform gyrus	-0.111	0.085	[-0.278 - 0.056]	1.911E-01	9.597E-01
Left inferior parietal cortex	0.045	0.085	[-0.122 - 0.211]	6.004E-01	9.597E-01
Right inferior parietal cortex	0.077	0.104	[-0.127 - 0.282]	4.586E-01	9.597E-01
Left lateral occipital cortex	-0.024	0.110	[-0.238 - 0.191]	8.289E-01	9.597E-01
Right lateral occipital cortex	-0.102	0.089	[-0.276 - 0.072]	2.515E-01	9.597E-01
Left rostral middle frontal gyrus	0.057	0.085	[-0.110 - 0.224]	5.013E-01	9.597E-01
Right rostral middle frontal gyrus	0.146	0.099	[-0.048 - 0.340]	1.409E-01	9.597E-01
Left precuneus	-0.021	0.085	[-0.188 - 0.146]	8.059E-01	9.597E-01
Right precuneus	-0.107	0.085	[-0.274 - 0.060]	2.099E-01	9.597E-01
Left inferior temporal gyrus	0.036	0.087	[-0.135 - 0.206]	6.800E-01	9.597E-01
Right inferior temporal gyrus	0.053	0.134	[-0.209 - 0.315]	6.932E-01	9.597E-01
Left lateral orbitofrontal cortex	-0.013	0.085	[-0.180 - 0.154]	8.800E-01	9.597E-01
Right lateral orbitofrontal cortex	0.149	0.085	[-0.017 - 0.316]	7.932E-02	9.597E-01
Left middle temporal gyrus	0.041	0.085	[-0.126 - 0.209]	6.305E-01	9.597E-01
Right middle temporal gyrus	0.040	0.118	[-0.192 - 0.272]	7.374E-01	9.597E-01
Left postcentral gyrus	0.074	0.098	[-0.118 - 0.267]	4.499E-01	9.597E-01
Right postcentral gyrus	-0.052	0.098	[-0.245 - 0.140]	5.935E-01	9.597E-01
Left medial orbitofrontal cortex	0.103	0.097	[-0.088 - 0.293]	2.904E-01	9.597E-01
Right medial orbitofrontal cortex	0.068	0.085	[-0.098 - 0.235]	4.210E-01	9.597E-01
Left cuneus	-0.020	0.111	[-0.237 - 0.197]	8.570E-01	9.597E-01
Right cuneus	-0.108	0.137	[-0.377 - 0.161]	4.317E-01	9.597E-01
Left pars triangularis of inferior frontal gyrus	-0.102	0.105	[-0.307 - 0.103]	3.306E-01	9.597E-01
Right pars triangularis of inferior frontal gyrus	-0.094	0.117	[-0.324 - 0.135]	4.210E-01	9.597E-01
Left superior parietal cortex	-0.087	0.085	[-0.254 - 0.080]	3.073E-01	9.597E-01
Right superior parietal cortex	0.023	0.088	[-0.150 - 0.196]	7.955E-01	9.597E-01
Left pars opercularis of inferior frontal gyrus	-0.039	0.090	[-0.215 - 0.137]	6.635E-01	9.597E-01
Right pars opercularis of inferior frontal gyrus	-0.006	0.085	[-0.173 - 0.161]	9.462E-01	9.597E-01
Left supramarginal gyrus	0.027	0.085	[-0.140 - 0.193]	7.547E-01	9.597E-01
Right supramarginal gyrus	0.025	0.085	[-0.142 - 0.192]	7.700E-01	9.597E-01
Left pericalcarine cortex	-0.021	0.085	[-0.188 - 0.146]	8.062E-01	9.597E-01
Right pericalcarine cortex	0.004	0.085	[-0.163 - 0.171]	9.597E-01	9.597E-01
Left parahippocampal gyrus	0.025	0.085	[-0.142 - 0.192]	7.665E-01	9.597E-01
Right parahippocampal gyrus	-0.077	0.106	[-0.285 - 0.132]	4.707E-01	9.597E-01
Left caudal middle frontal gyrus	0.063	0.085	[-0.104 - 0.230]	4.584E-01	9.597E-01
Right caudal middle frontal gyrus	0.051	0.085	[-0.116 - 0.218]	5.484E-01	9.597E-01
Left transverse temporal gyrus	-0.044	0.085	[-0.211 - 0.123]	6.052E-01	9.597E-01
Right transverse temporal gyrus	-0.046	0.085	[-0.213 - 0.121]	5.899E-01	9.597E-01
Left banks of superior temporal sulcus	0.068	0.090	[-0.109 - 0.245]	4.526E-01	9.597E-01
Right banks of superior temporal sulcus	0.048	0.087	[-0.123 - 0.218]	5.841E-01	9.597E-01
Left caudal anterior cingulate cortex	0.087	0.110	[-0.128 - 0.302]	4.292E-01	9.597E-01
Right caudal anterior cingulate cortex	0.028	0.085	[-0.139 - 0.194]	7.463E-01	9.597E-01
Left rostral anterior cingulate cortex	0.069	0.085	[-0.098 - 0.236]	4.152E-01	9.597E-01
Right rostral anterior cingulate cortex	-0.011	0.105	[-0.216 - 0.194]	9.154E-01	9.597E-01
Left posterior cingulate cortex	-0.061	0.085	[-0.228 - 0.106]	4.727E-01	9.597E-01
Right posterior cingulate cortex	0.030	0.086	[-0.139 - 0.198]	7.298E-01	9.597E-01
Left frontal pole	-0.072	0.085	[-0.239 - 0.094]	3.960E-01	9.597E-01
Right frontal pole	-0.112	0.085	[-0.279 - 0.055]	1.884E-01	9.597E-01
Left paracentral lobule	-0.020	0.085	[-0.186 - 0.147]	8.177E-01	9.597E-01
Right paracentral lobule	0.042	0.085	[-0.125 - 0.209]	6.205E-01	9.597E-01
Left insula	0.067	0.085	[-0.100 - 0.233]	4.339E-01	9.597E-01
Right insula	0.014	0.085	[-0.153 - 0.181]	8.666E-01	9.597E-01
Left entorhinal cortex	0.197	0.085	[0.030 - 0.364]	2.083E-02	7.887E-01
Right entorhinal cortex	0.118	0.096	[-0.070 - 0.307]	2.177E-01	9.597E-01
Left temporal pole	0.090	0.098	[-0.103 - 0.282]	3.627E-01	9.597E-01
Right temporal pole	0.221	0.110	[0.005 - 0.438]	4.511E-02	9.597E-01
Left isthmus cingulate cortex	0.019	0.085	[-0.148 - 0.185]	8.271E-01	9.597E-01
Right isthmus cingulate cortex	0.022	0.104	[-0.182 - 0.226]	8.350E-01	9.597E-01

Supplementary Table S19. Cortical surface area differences between individuals with bipolar disorder and healthy comparison subjects controlling for age and sex at 25 years of age or older

	d	Std. Err.	95% CI	*p*-value	FDR *q*-value
Total cortical surface area	0.027	0.094	[-0.157 - 0.210]	7.757E-01	9.831E-01
Left hemisphere	0.035	0.088	[-0.138 - 0.208]	6.940E-01	9.831E-01
Right hemisphere	0.017	0.099	[-0.177 - 0.212]	8.613E-01	9.831E-01
Left superior frontal gyrus	-0.070	0.101	[-0.267 - 0.128]	4.887E-01	9.831E-01
Right superior frontal gyrus	-0.109	0.088	[-0.282 - 0.063]	2.147E-01	9.831E-01
Left pars orbitalis of inferior frontal gyrus	0.004	0.088	[-0.169 - 0.177]	9.653E-01	9.831E-01
Right pars orbitalis of inferior frontal gyrus	-0.198	0.088	[-0.371 - -0.026]	2.433E-02	8.314E-01
Left precentral gyrus	-0.068	0.163	[-0.388 - 0.252]	6.786E-01	9.831E-01
Right precentral gyrus	-0.099	0.111	[-0.317 - 0.118]	3.699E-01	9.831E-01
Left lingual gyrus	0.029	0.112	[-0.190 - 0.249]	7.936E-01	9.831E-01
Right lingual gyrus	0.069	0.088	[-0.103 - 0.242]	4.298E-01	9.831E-01
Left superior temporal gyrus	-0.024	0.108	[-0.235 - 0.188]	8.262E-01	9.831E-01
Right superior temporal gyrus	-0.031	0.088	[-0.203 - 0.142]	7.282E-01	9.831E-01
Left fusiform gyrus	0.004	0.088	[-0.169 - 0.176]	9.646E-01	9.831E-01
Right fusiform gyrus	-0.105	0.088	[-0.278 - 0.067]	2.323E-01	9.831E-01
Left inferior parietal cortex	0.063	0.088	[-0.110 - 0.235]	4.772E-01	9.831E-01
Right inferior parietal cortex	0.094	0.115	[-0.131 - 0.319]	4.114E-01	9.831E-01
Left lateral occipital cortex	-0.014	0.126	[-0.261 - 0.233]	9.117E-01	9.831E-01
Right lateral occipital cortex	-0.101	0.103	[-0.302 - 0.100]	3.257E-01	9.831E-01
Left rostral middle frontal gyrus	0.087	0.088	[-0.086 - 0.260]	3.231E-01	9.831E-01
Right rostral middle frontal gyrus	0.166	0.104	[-0.037 - 0.370]	1.089E-01	9.831E-01
Left precuneus	0.010	0.088	[-0.163 - 0.182]	9.110E-01	9.831E-01
Right precuneus	-0.062	0.093	[-0.245 - 0.121]	5.050E-01	9.831E-01
Left inferior temporal gyrus	0.061	0.097	[-0.130 - 0.252]	5.313E-01	9.831E-01
Right inferior temporal gyrus	0.037	0.139	[-0.236 - 0.310]	7.927E-01	9.831E-01
Left lateral orbitofrontal cortex	0.005	0.088	[-0.168 - 0.177]	9.576E-01	9.831E-01
Right lateral orbitofrontal cortex	0.175	0.088	[0.002 - 0.348]	4.684E-02	8.314E-01
Left middle temporal gyrus	0.104	0.088	[-0.068 - 0.277]	2.359E-01	9.831E-01
Right middle temporal gyrus	0.036	0.123	[-0.205 - 0.278]	7.677E-01	9.831E-01
Left postcentral gyrus	0.073	0.114	[-0.150 - 0.296]	5.189E-01	9.831E-01
Right postcentral gyrus	-0.037	0.103	[-0.239 - 0.166]	7.218E-01	9.831E-01
Left medial orbitofrontal cortex	0.062	0.088	[-0.110 - 0.235]	4.789E-01	9.831E-01
Right medial orbitofrontal cortex	0.059	0.088	[-0.114 - 0.231]	5.046E-01	9.831E-01
Left cuneus	-0.019	0.111	[-0.237 - 0.199]	8.632E-01	9.831E-01
Right cuneus	-0.112	0.125	[-0.357 - 0.133]	3.699E-01	9.831E-01
Left pars triangularis of inferior frontal gyrus	-0.134	0.118	[-0.367 - 0.098]	2.562E-01	9.831E-01
Right pars triangularis of inferior frontal gyrus	-0.070	0.121	[-0.306 - 0.166]	5.615E-01	9.831E-01
Left superior parietal cortex	-0.069	0.088	[-0.242 - 0.103]	4.308E-01	9.831E-01
Right superior parietal cortex	0.034	0.089	[-0.141 - 0.208]	7.060E-01	9.831E-01
Left pars opercularis of inferior frontal gyrus	-0.020	0.088	[-0.193 - 0.152]	8.171E-01	9.831E-01
Right pars opercularis of inferior frontal gyrus	0.009	0.088	[-0.164 - 0.182]	9.154E-01	9.831E-01
Left supramarginal gyrus	0.022	0.088	[-0.150 - 0.195]	8.001E-01	9.831E-01
Right supramarginal gyrus	0.035	0.088	[-0.138 - 0.208]	6.920E-01	9.831E-01
Left pericalcarine cortex	-0.003	0.088	[-0.175 - 0.169]	9.735E-01	9.831E-01
Right pericalcarine cortex	0.002	0.088	[-0.171 - 0.174]	9.831E-01	9.831E-01
Left parahippocampal gyrus	0.007	0.088	[-0.166 - 0.179]	9.391E-01	9.831E-01
Right parahippocampal gyrus	-0.077	0.095	[-0.263 - 0.109]	4.185E-01	9.831E-01
Left caudal middle frontal gyrus	0.041	0.088	[-0.131 - 0.214]	6.383E-01	9.831E-01
Right caudal middle frontal gyrus	0.059	0.095	[-0.126 - 0.245]	5.308E-01	9.831E-01
Left transverse temporal gyrus	-0.049	0.088	[-0.222 - 0.123]	5.750E-01	9.831E-01
Right transverse temporal gyrus	-0.033	0.088	[-0.205 - 0.140]	7.113E-01	9.831E-01
Left banks of superior temporal sulcus	0.069	0.100	[-0.128 - 0.266]	4.928E-01	9.831E-01
Right banks of superior temporal sulcus	0.074	0.088	[-0.098 - 0.247]	3.985E-01	9.831E-01
Left caudal anterior cingulate cortex	0.115	0.110	[-0.099 - 0.330]	2.922E-01	9.831E-01
Right caudal anterior cingulate cortex	0.017	0.088	[-0.155 - 0.190]	8.445E-01	9.831E-01
Left rostral anterior cingulate cortex	0.120	0.090	[-0.055 - 0.296]	1.793E-01	9.831E-01
Right rostral anterior cingulate cortex	-0.009	0.110	[-0.226 - 0.207]	9.323E-01	9.831E-01
Left posterior cingulate cortex	-0.060	0.088	[-0.232 - 0.113]	4.971E-01	9.831E-01
Right posterior cingulate cortex	0.069	0.088	[-0.103 - 0.242]	4.316E-01	9.831E-01
Left frontal pole	-0.068	0.088	[-0.241 - 0.104]	4.377E-01	9.831E-01
Right frontal pole	-0.135	0.088	[-0.307 - 0.038]	1.260E-01	9.831E-01
Left paracentral lobule	-0.015	0.088	[-0.188 - 0.157]	8.612E-01	9.831E-01
Right paracentral lobule	0.060	0.088	[-0.113 - 0.233]	4.955E-01	9.831E-01
Left insula	0.055	0.088	[-0.117 - 0.228]	5.285E-01	9.831E-01
Right insula	0.017	0.090	[-0.160 - 0.194]	8.489E-01	9.831E-01
Left entorhinal cortex	0.215	0.088	[0.042 - 0.388]	1.475E-02	8.314E-01
Right entorhinal cortex	0.166	0.107	[-0.044 - 0.377]	1.214E-01	9.831E-01
Left temporal pole	0.092	0.125	[-0.153 - 0.338]	4.611E-01	9.831E-01
Right temporal pole	0.227	0.112	[0.008 - 0.446]	4.237E-02	8.314E-01
Left isthmus cingulate cortex	0.015	0.088	[-0.157 - 0.187]	8.646E-01	9.831E-01
Right isthmus cingulate cortex	0.064	0.088	[-0.108 - 0.237]	4.656E-01	9.831E-01

Supplementary Table S20. Cortical surface area differences between individuals with major depressive disorder and healthy comparison subjects controlling for age and sex

	d	Std. Err.	95% CI	*p*-value	FDR *q*-value
Total cortical surface area	-0.214	0.071	[-0.354 - -0.074]	2.745E-03	2.784E-02
Left hemisphere	-0.215	0.070	[-0.352 - -0.077]	2.232E-03	2.728E-02
Right hemisphere	-0.211	0.072	[-0.353 - -0.070]	3.467E-03	2.967E-02
Left superior frontal gyrus	-0.229	0.083	[-0.391 - -0.066]	5.760E-03	3.403E-02
Right superior frontal gyrus	-0.254	0.083	[-0.417 - -0.091]	2.306E-03	2.728E-02
Left pars orbitalis of inferior frontal gyrus	-0.178	0.068	[-0.311 - -0.045]	8.608E-03	3.660E-02
Right pars orbitalis of inferior frontal gyrus	-0.157	0.061	[-0.278 - -0.037]	1.041E-02	3.660E-02
Left precentral gyrus	-0.136	0.065	[-0.263 - -0.009]	3.542E-02	8.548E-02
Right precentral gyrus	-0.108	0.061	[-0.229 - 0.012]	7.724E-02	1.373E-01
Left lingual gyrus	-0.123	0.071	[-0.263 - 0.017]	8.522E-02	1.441E-01
Right lingual gyrus	-0.157	0.061	[-0.278 - -0.037]	1.048E-02	3.660E-02
Left superior temporal gyrus	-0.141	0.071	[-0.279 - -0.002]	4.711E-02	1.018E-01
Right superior temporal gyrus	-0.168	0.071	[-0.307 - -0.029]	1.796E-02	5.099E-02
Left fusiform gyrus	-0.131	0.076	[-0.280 - 0.017]	8.279E-02	1.434E-01
Right fusiform gyrus	-0.201	0.062	[-0.322 - -0.081]	1.059E-03	2.069E-02
Left inferior parietal cortex	-0.164	0.078	[-0.317 - -0.011]	3.612E-02	8.548E-02
Right inferior parietal cortex	-0.134	0.071	[-0.274 - 0.006]	6.141E-02	1.183E-01
Left lateral occipital cortex	-0.121	0.061	[-0.241 - 0.000]	4.949E-02	1.033E-01
Right lateral occipital cortex	-0.179	0.067	[-0.309 - -0.048]	7.443E-03	3.660E-02
Left rostral middle frontal gyrus	-0.154	0.062	[-0.276 - -0.032]	1.343E-02	4.335E-02
Right rostral middle frontal gyrus	-0.109	0.075	[-0.255 - 0.037]	1.431E-01	2.162E-01
Left precuneus	-0.200	0.061	[-0.320 - -0.079]	1.166E-03	2.069E-02
Right precuneus	-0.050	0.067	[-0.182 - 0.082]	4.563E-01	5.211E-01
Left inferior temporal gyrus	-0.131	0.061	[-0.251 - -0.011]	3.287E-02	8.336E-02
Right inferior temporal gyrus	-0.094	0.063	[-0.218 - 0.029]	1.336E-01	2.062E-01
Left lateral orbitofrontal cortex	-0.161	0.061	[-0.281 - -0.040]	8.852E-03	3.660E-02
Right lateral orbitofrontal cortex	-0.091	0.073	[-0.233 - 0.052]	2.125E-01	2.847E-01
Left middle temporal gyrus	-0.182	0.066	[-0.312 - -0.052]	6.231E-03	3.403E-02
Right middle temporal gyrus	-0.159	0.069	[-0.293 - -0.024]	2.081E-02	5.472E-02
Left postcentral gyrus	-0.148	0.074	[-0.293 - -0.003]	4.607E-02	1.018E-01
Right postcentral gyrus	-0.194	0.069	[-0.330 - -0.059]	5.007E-03	3.403E-02
Left medial orbitofrontal cortex	-0.105	0.080	[-0.262 - 0.052]	1.911E-01	2.661E-01
Right medial orbitofrontal cortex	-0.126	0.068	[-0.259 - 0.006]	6.166E-02	1.183E-01
Left cuneus	-0.198	0.078	[-0.350 - -0.046]	1.083E-02	3.660E-02
Right cuneus	-0.210	0.062	[-0.331 - -0.090]	6.284E-04	2.069E-02
Left pars triangularis of inferior frontal gyrus	-0.191	0.082	[-0.351 - -0.031]	1.929E-02	5.268E-02
Right pars triangularis of inferior frontal gyrus	-0.178	0.061	[-0.298 - -0.058]	3.761E-03	2.967E-02
Left superior parietal cortex	-0.101	0.061	[-0.221 - 0.020]	1.012E-01	1.632E-01
Right superior parietal cortex	-0.071	0.061	[-0.191 - 0.049]	2.473E-01	3.135E-01
Left pars opercularis of inferior frontal gyrus	-0.121	0.084	[-0.285 - 0.044]	1.519E-01	2.246E-01
Right pars opercularis of inferior frontal gyrus	-0.067	0.066	[-0.197 - 0.063]	3.156E-01	3.734E-01
Left supramarginal gyrus	-0.084	0.067	[-0.215 - 0.047]	2.082E-01	2.842E-01
Right supramarginal gyrus	-0.094	0.068	[-0.227 - 0.039]	1.644E-01	2.357E-01
Left pericalcarine cortex	-0.171	0.066	[-0.301 - -0.042]	9.519E-03	3.660E-02
Right pericalcarine cortex	-0.232	0.062	[-0.353 - -0.112]	1.607E-04	1.141E-02
Left parahippocampal gyrus	-0.036	0.064	[-0.160 - 0.089]	5.720E-01	5.972E-01
Right parahippocampal gyrus	-0.093	0.061	[-0.214 - 0.027]	1.293E-01	2.040E-01
Left caudal middle frontal gyrus	-0.082	0.071	[-0.220 - 0.056]	2.451E-01	3.135E-01
Right caudal middle frontal gyrus	-0.025	0.069	[-0.160 - 0.110]	7.148E-01	7.337E-01
Left transverse temporal gyrus	-0.069	0.061	[-0.189 - 0.052]	2.636E-01	3.268E-01
Right transverse temporal gyrus	-0.110	0.061	[-0.230 - 0.010]	7.346E-02	1.373E-01
Left banks of superior temporal sulcus	-0.108	0.063	[-0.232 - 0.016]	8.872E-02	1.465E-01
Right banks of superior temporal sulcus	-0.073	0.101	[-0.271 - 0.124]	4.655E-01	5.211E-01
Left caudal anterior cingulate cortex	-0.147	0.061	[-0.268 - -0.027]	1.644E-02	4.868E-02
Right caudal anterior cingulate cortex	-0.131	0.068	[-0.264 - 0.002]	5.331E-02	1.081E-01
Left rostral anterior cingulate cortex	-0.108	0.061	[-0.229 - 0.012]	7.736E-02	1.373E-01
Right rostral anterior cingulate cortex	-0.063	0.075	[-0.209 - 0.084]	4.027E-01	4.687E-01
Left posterior cingulate cortex	-0.085	0.061	[-0.205 - 0.035]	1.660E-01	2.357E-01
Right posterior cingulate cortex	-0.165	0.069	[-0.299 - -0.030]	1.645E-02	4.868E-02
Left frontal pole	-0.133	0.067	[-0.265 - -0.002]	4.733E-02	1.018E-01
Right frontal pole	-0.170	0.061	[-0.290 - -0.049]	5.779E-03	3.403E-02
Left paracentral lobule	-0.044	0.061	[-0.165 - 0.076]	4.697E-01	5.211E-01
Right paracentral lobule	-0.157	0.061	[-0.277 - -0.036]	1.069E-02	3.660E-02
Left insula	-0.078	0.071	[-0.217 - 0.060]	2.669E-01	3.268E-01
Right insula	-0.078	0.073	[-0.220 - 0.064]	2.828E-01	3.403E-01
Left entorhinal cortex	-0.013	0.071	[-0.153 - 0.127]	8.561E-01	8.561E-01
Right entorhinal cortex	0.042	0.061	[-0.079 - 0.162]	4.974E-01	5.433E-01
Left temporal pole	-0.078	0.064	[-0.202 - 0.047]	2.226E-01	2.926E-01
Right temporal pole	-0.037	0.061	[-0.157 - 0.083]	5.456E-01	5.842E-01
Left isthmus cingulate cortex	-0.037	0.061	[-0.157 - 0.084]	5.513E-01	5.842E-01
Right isthmus cingulate cortex	0.022	0.061	[-0.099 - 0.142]	7.233E-01	7.337E-01

Supplementary Table S21. Cortical surface area differences between individuals with major depressive disorder and healthy comparison subjects controlling for age and sex over 21 years of old

	d	Std. Err.	95% CI	*p*-value	FDR *q*-value
Total cortical surface area	-0.190	0.076	[-0.339 - -0.040]	1.288E-02	7.386E-02
Left hemisphere	-0.190	0.076	[-0.338 - -0.041]	1.233E-02	7.386E-02
Right hemisphere	-0.188	0.076	[-0.338 - -0.038]	1.394E-02	7.386E-02
Left superior frontal gyrus	-0.185	0.079	[-0.339 - -0.031]	1.873E-02	7.386E-02
Right superior frontal gyrus	-0.231	0.086	[-0.399 - -0.063]	7.066E-03	7.386E-02
Left pars orbitalis of inferior frontal gyrus	-0.157	0.078	[-0.309 - -0.004]	4.467E-02	1.220E-01
Right pars orbitalis of inferior frontal gyrus	-0.164	0.063	[-0.287 - -0.040]	9.634E-03	7.386E-02
Left precentral gyrus	-0.116	0.072	[-0.258 - 0.025]	1.072E-01	1.931E-01
Right precentral gyrus	-0.097	0.063	[-0.220 - 0.027]	1.252E-01	2.168E-01
Left lingual gyrus	-0.119	0.079	[-0.274 - 0.036]	1.332E-01	2.251E-01
Right lingual gyrus	-0.152	0.063	[-0.276 - -0.028]	1.652E-02	7.386E-02
Left superior temporal gyrus	-0.126	0.074	[-0.271 - 0.018]	8.672E-02	1.866E-01
Right superior temporal gyrus	-0.157	0.079	[-0.311 - -0.002]	4.711E-02	1.239E-01
Left fusiform gyrus	-0.134	0.077	[-0.285 - 0.018]	8.364E-02	1.856E-01
Right fusiform gyrus	-0.177	0.063	[-0.301 - -0.053]	5.048E-03	7.386E-02
Left inferior parietal cortex	-0.154	0.088	[-0.326 - 0.019]	8.035E-02	1.840E-01
Right inferior parietal cortex	-0.130	0.079	[-0.285 - 0.025]	1.000E-01	1.919E-01
Left lateral occipital cortex	-0.107	0.063	[-0.231 - 0.017]	8.982E-02	1.876E-01
Right lateral occipital cortex	-0.172	0.071	[-0.311 - -0.034]	1.458E-02	7.386E-02
Left rostral middle frontal gyrus	-0.133	0.065	[-0.260 - -0.007]	3.895E-02	1.106E-01
Right rostral middle frontal gyrus	-0.089	0.076	[-0.238 - 0.060]	2.430E-01	3.450E-01
Left precuneus	-0.185	0.063	[-0.309 - -0.061]	3.454E-03	7.386E-02
Right precuneus	-0.031	0.075	[-0.178 - 0.116]	6.776E-01	6.973E-01
Left inferior temporal gyrus	-0.118	0.063	[-0.241 - 0.006]	6.258E-02	1.532E-01
Right inferior temporal gyrus	-0.086	0.067	[-0.217 - 0.044]	1.959E-01	2.960E-01
Left lateral orbitofrontal cortex	-0.136	0.063	[-0.260 - -0.012]	3.140E-02	9.692E-02
Right lateral orbitofrontal cortex	-0.050	0.070	[-0.187 - 0.087]	4.753E-01	5.273E-01
Left middle temporal gyrus	-0.162	0.063	[-0.286 - -0.038]	1.037E-02	7.386E-02
Right middle temporal gyrus	-0.174	0.072	[-0.315 - -0.032]	1.612E-02	7.386E-02
Left postcentral gyrus	-0.149	0.077	[-0.299 - 0.002]	5.280E-02	1.339E-01
Right postcentral gyrus	-0.193	0.077	[-0.345 - -0.041]	1.254E-02	7.386E-02
Left medial orbitofrontal cortex	-0.078	0.086	[-0.247 - 0.091]	3.679E-01	4.849E-01
Right medial orbitofrontal cortex	-0.109	0.074	[-0.255 - 0.037]	1.426E-01	2.354E-01
Left cuneus	-0.193	0.082	[-0.353 - -0.033]	1.826E-02	7.386E-02
Right cuneus	-0.205	0.063	[-0.329 - -0.081]	1.214E-03	4.309E-02
Left pars triangularis of inferior frontal gyrus	-0.157	0.070	[-0.295 - -0.019]	2.604E-02	8.828E-02
Right pars triangularis of inferior frontal gyrus	-0.135	0.065	[-0.264 - -0.007]	3.890E-02	1.106E-01
Left superior parietal cortex	-0.089	0.063	[-0.212 - 0.035]	1.611E-01	2.542E-01
Right superior parietal cortex	-0.056	0.063	[-0.179 - 0.068]	3.788E-01	4.890E-01
Left pars opercularis of inferior frontal gyrus	-0.089	0.090	[-0.265 - 0.087]	3.224E-01	4.402E-01
Right pars opercularis of inferior frontal gyrus	-0.063	0.080	[-0.220 - 0.094]	4.286E-01	4.939E-01
Left supramarginal gyrus	-0.070	0.070	[-0.208 - 0.067]	3.166E-01	4.402E-01
Right supramarginal gyrus	-0.088	0.066	[-0.217 - 0.042]	1.833E-01	2.829E-01
Left pericalcarine cortex	-0.158	0.071	[-0.297 - -0.019]	2.611E-02	8.828E-02
Right pericalcarine cortex	-0.227	0.066	[-0.355 - -0.098]	5.440E-04	3.863E-02
Left parahippocampal gyrus	-0.051	0.063	[-0.175 - 0.072]	4.162E-01	4.939E-01
Right parahippocampal gyrus	-0.103	0.063	[-0.227 - 0.021]	1.037E-01	1.931E-01
Left caudal middle frontal gyrus	-0.067	0.074	[-0.213 - 0.079]	3.688E-01	4.849E-01
Right caudal middle frontal gyrus	-0.010	0.080	[-0.168 - 0.148]	9.011E-01	9.011E-01
Left transverse temporal gyrus	-0.076	0.063	[-0.199 - 0.048]	2.302E-01	3.335E-01
Right transverse temporal gyrus	-0.101	0.063	[-0.225 - 0.022]	1.088E-01	1.931E-01
Left banks of superior temporal sulcus	-0.117	0.070	[-0.254 - 0.020]	9.525E-02	1.878E-01
Right banks of superior temporal sulcus	-0.086	0.109	[-0.299 - 0.128]	4.313E-01	4.939E-01
Left caudal anterior cingulate cortex	-0.150	0.063	[-0.274 - -0.026]	1.744E-02	7.386E-02
Right caudal anterior cingulate cortex	-0.105	0.074	[-0.250 - 0.039]	1.530E-01	2.469E-01
Left rostral anterior cingulate cortex	-0.106	0.063	[-0.230 - 0.018]	9.252E-02	1.877E-01
Right rostral anterior cingulate cortex	-0.051	0.085	[-0.217 - 0.116]	5.507E-01	6.015E-01
Left posterior cingulate cortex	-0.078	0.063	[-0.202 - 0.046]	2.167E-01	3.206E-01
Right posterior cingulate cortex	-0.168	0.074	[-0.314 - -0.022]	2.452E-02	8.828E-02
Left frontal pole	-0.125	0.070	[-0.262 - 0.011]	7.250E-02	1.716E-01
Right frontal pole	-0.175	0.063	[-0.299 - -0.052]	5.494E-03	7.386E-02
Left paracentral lobule	-0.030	0.063	[-0.153 - 0.094]	6.390E-01	6.672E-01
Right paracentral lobule	-0.136	0.063	[-0.260 - -0.012]	3.133E-02	9.692E-02
Left insula	-0.059	0.074	[-0.204 - 0.086]	4.234E-01	4.939E-01
Right insula	-0.064	0.079	[-0.220 - 0.091]	4.166E-01	4.939E-01
Left entorhinal cortex	-0.026	0.072	[-0.167 - 0.115]	7.178E-01	7.281E-01
Right entorhinal cortex	0.049	0.063	[-0.075 - 0.172]	4.424E-01	4.986E-01
Left temporal pole	-0.058	0.072	[-0.200 - 0.084]	4.248E-01	4.939E-01
Right temporal pole	-0.051	0.063	[-0.175 - 0.072]	4.149E-01	4.939E-01
Left isthmus cingulate cortex	-0.032	0.063	[-0.155 - 0.092]	6.153E-01	6.521E-01
Right isthmus cingulate cortex	0.039	0.071	[-0.100 - 0.178]	5.794E-01	6.233E-01

Supplementary Table S22. Cortical surface area differences between individuals with autism spectrum disorder and healthy comparison subjects controlling for age and sex

	d	Std. Err.	95% CI	*p*-value	FDR *q*-value
Total cortical surface area	-0.179	0.108	[-0.391 - 0.032]	9.670E-02	4.034E-01
Left hemisphere	-0.184	0.102	[-0.384 - 0.016]	7.093E-02	4.034E-01
Right hemisphere	-0.173	0.113	[-0.395 - 0.049]	1.259E-01	4.243E-01
Left superior frontal gyrus	-0.124	0.115	[-0.349 - 0.101]	2.803E-01	4.905E-01
Right superior frontal gyrus	-0.113	0.103	[-0.316 - 0.089]	2.711E-01	4.905E-01
Left pars orbitalis of inferior frontal gyrus	-0.166	0.103	[-0.368 - 0.036]	1.077E-01	4.034E-01
Right pars orbitalis of inferior frontal gyrus	-0.282	0.091	[-0.460 - -0.104]	1.922E-03	1.364E-01
Left precentral gyrus	-0.110	0.080	[-0.267 - 0.047]	1.689E-01	4.243E-01
Right precentral gyrus	-0.026	0.119	[-0.260 - 0.208]	8.261E-01	8.626E-01
Left lingual gyrus	-0.123	0.127	[-0.372 - 0.127]	3.346E-01	5.139E-01
Right lingual gyrus	-0.177	0.110	[-0.392 - 0.037]	1.056E-01	4.034E-01
Left superior temporal gyrus	-0.129	0.080	[-0.286 - 0.028]	1.079E-01	4.034E-01
Right superior temporal gyrus	-0.086	0.092	[-0.266 - 0.094]	3.490E-01	5.139E-01
Left fusiform gyrus	-0.170	0.080	[-0.327 - -0.013]	3.373E-02	4.034E-01
Right fusiform gyrus	-0.037	0.111	[-0.254 - 0.179]	7.346E-01	7.902E-01
Left inferior parietal cortex	-0.190	0.105	[-0.397 - 0.016]	7.121E-02	4.034E-01
Right inferior parietal cortex	-0.161	0.109	[-0.374 - 0.053]	1.402E-01	4.243E-01
Left lateral occipital cortex	-0.064	0.080	[-0.221 - 0.093]	4.241E-01	5.580E-01
Right lateral occipital cortex	-0.088	0.124	[-0.332 - 0.155]	4.766E-01	6.042E-01
Left rostral middle frontal gyrus	-0.120	0.080	[-0.277 - 0.037]	1.343E-01	4.243E-01
Right rostral middle frontal gyrus	-0.159	0.093	[-0.342 - 0.024]	8.875E-02	4.034E-01
Left precuneus	-0.034	0.118	[-0.266 - 0.197]	7.713E-01	8.174E-01
Right precuneus	-0.091	0.100	[-0.288 - 0.106]	3.640E-01	5.139E-01
Left inferior temporal gyrus	-0.054	0.136	[-0.321 - 0.214]	6.937E-01	7.790E-01
Right inferior temporal gyrus	-0.088	0.099	[-0.281 - 0.106]	3.741E-01	5.139E-01
Left lateral orbitofrontal cortex	-0.137	0.093	[-0.318 - 0.045]	1.413E-01	4.243E-01
Right lateral orbitofrontal cortex	-0.115	0.098	[-0.307 - 0.076]	2.376E-01	4.821E-01
Left middle temporal gyrus	-0.014	0.125	[-0.260 - 0.232]	9.111E-01	9.111E-01
Right middle temporal gyrus	-0.138	0.097	[-0.328 - 0.052]	1.551E-01	4.243E-01
Left postcentral gyrus	-0.143	0.104	[-0.347 - 0.062]	1.713E-01	4.243E-01
Right postcentral gyrus	-0.130	0.114	[-0.353 - 0.093]	2.522E-01	4.905E-01
Left medial orbitofrontal cortex	-0.161	0.080	[-0.318 - -0.004]	4.472E-02	4.034E-01
Right medial orbitofrontal cortex	-0.158	0.094	[-0.341 - 0.026]	9.275E-02	4.034E-01
Left cuneus	-0.120	0.084	[-0.284 - 0.044]	1.520E-01	4.243E-01
Right cuneus	-0.198	0.086	[-0.366 - -0.030]	2.066E-02	4.034E-01
Left pars triangularis of inferior frontal gyrus	-0.131	0.080	[-0.287 - 0.026]	1.029E-01	4.034E-01
Right pars triangularis of inferior frontal gyrus	-0.067	0.115	[-0.293 - 0.158]	5.589E-01	6.788E-01
Left superior parietal cortex	-0.217	0.130	[-0.472 - 0.038]	9.511E-02	4.034E-01
Right superior parietal cortex	-0.210	0.099	[-0.404 - -0.016]	3.413E-02	4.034E-01
Left pars opercularis of inferior frontal gyrus	-0.111	0.080	[-0.268 - 0.046]	1.646E-01	4.243E-01
Right pars opercularis of inferior frontal gyrus	-0.098	0.080	[-0.255 - 0.059]	2.211E-01	4.670E-01
Left supramarginal gyrus	-0.105	0.103	[-0.307 - 0.096]	3.057E-01	4.933E-01
Right supramarginal gyrus	-0.105	0.080	[-0.262 - 0.052]	1.898E-01	4.347E-01
Left pericalcarine cortex	-0.129	0.123	[-0.370 - 0.111]	2.916E-01	4.905E-01
Right pericalcarine cortex	-0.135	0.129	[-0.388 - 0.119]	2.971E-01	4.905E-01
Left parahippocampal gyrus	-0.101	0.081	[-0.261 - 0.058]	2.133E-01	4.670E-01
Right parahippocampal gyrus	0.045	0.134	[-0.217 - 0.308]	7.342E-01	7.902E-01
Left caudal middle frontal gyrus	-0.202	0.080	[-0.359 - -0.045]	1.150E-02	4.034E-01
Right caudal middle frontal gyrus	-0.063	0.080	[-0.220 - 0.094]	4.323E-01	5.580E-01
Left transverse temporal gyrus	-0.040	0.080	[-0.197 - 0.117]	6.174E-01	7.186E-01
Right transverse temporal gyrus	-0.109	0.080	[-0.265 - 0.048]	1.750E-01	4.243E-01
Left banks of superior temporal sulcus	0.018	0.113	[-0.203 - 0.240]	8.719E-01	8.972E-01
Right banks of superior temporal sulcus	-0.097	0.102	[-0.298 - 0.104]	3.428E-01	5.139E-01
Left caudal anterior cingulate cortex	-0.038	0.100	[-0.233 - 0.157]	7.022E-01	7.790E-01
Right caudal anterior cingulate cortex	-0.079	0.100	[-0.275 - 0.117]	4.276E-01	5.580E-01
Left rostral anterior cingulate cortex	-0.086	0.096	[-0.275 - 0.103]	3.709E-01	5.139E-01
Right rostral anterior cingulate cortex	-0.056	0.097	[-0.247 - 0.136]	5.688E-01	6.788E-01
Left posterior cingulate cortex	-0.139	0.080	[-0.296 - 0.018]	8.326E-02	4.034E-01
Right posterior cingulate cortex	-0.141	0.128	[-0.391 - 0.110]	2.713E-01	4.905E-01
Left frontal pole	-0.080	0.091	[-0.259 - 0.098]	3.764E-01	5.139E-01
Right frontal pole	0.010	0.080	[-0.147 - 0.166]	9.047E-01	9.111E-01
Left paracentral lobule	-0.077	0.080	[-0.233 - 0.080]	3.376E-01	5.139E-01
Right paracentral lobule	-0.090	0.080	[-0.247 - 0.067]	2.598E-01	4.905E-01
Left insula	-0.111	0.091	[-0.289 - 0.068]	2.236E-01	4.670E-01
Right insula	-0.111	0.083	[-0.274 - 0.051]	1.793E-01	4.243E-01
Left entorhinal cortex	-0.203	0.117	[-0.433 - 0.027]	8.312E-02	4.034E-01
Right entorhinal cortex	-0.055	0.091	[-0.234 - 0.123]	5.431E-01	6.765E-01
Left temporal pole	-0.058	0.104	[-0.261 - 0.145]	5.736E-01	6.788E-01
Right temporal pole	-0.098	0.093	[-0.281 - 0.084]	2.915E-01	4.905E-01
Left isthmus cingulate cortex	-0.178	0.080	[-0.335 - -0.021]	2.588E-02	4.034E-01
Right isthmus cingulate cortex	-0.050	0.114	[-0.273 - 0.173]	6.596E-01	7.553E-01

Supplementary Table S23. Cortical surface area differences between individuals with schizophrenia and healthy comparison subjects controlling for age, sex, and total cortical surface area

	d	Std. Err.	95% CI	*p*-value	FDR *q*-value
Left superior frontal gyrus	-0.105	0.041	[-0.186 - -0.024]	1.126E-02	9.573E-02
Right superior frontal gyrus	-0.067	0.035	[-0.135 - 0.001]	5.174E-02	3.198E-01
Left pars orbitalis of inferior frontal gyrus	-0.134	0.036	[-0.204 - -0.064]	1.628E-04	3.691E-03
Right pars orbitalis of inferior frontal gyrus	-0.103	0.035	[-0.171 - -0.035]	2.997E-03	4.076E-02
Left precentral gyrus	-0.051	0.039	[-0.127 - 0.024]	1.845E-01	5.083E-01
Right precentral gyrus	-0.014	0.042	[-0.097 - 0.069]	7.343E-01	8.917E-01
Left lingual gyrus	-0.094	0.035	[-0.162 - -0.026]	6.983E-03	6.914E-02
Right lingual gyrus	-0.093	0.035	[-0.161 - -0.025]	7.117E-03	6.914E-02
Left superior temporal gyrus	0.057	0.041	[-0.023 - 0.138]	1.628E-01	5.083E-01
Right superior temporal gyrus	-0.018	0.035	[-0.086 - 0.050]	6.078E-01	8.104E-01
Left fusiform gyrus	-0.052	0.035	[-0.120 - 0.016]	1.311E-01	5.083E-01
Right fusiform gyrus	-0.022	0.043	[-0.106 - 0.062]	6.045E-01	8.104E-01
Left inferior parietal cortex	0.045	0.038	[-0.030 - 0.120]	2.424E-01	5.683E-01
Right inferior parietal cortex	-0.041	0.035	[-0.109 - 0.027]	2.377E-01	5.683E-01
Left lateral occipital cortex	-0.005	0.035	[-0.073 - 0.063]	8.851E-01	9.554E-01
Right lateral occipital cortex	-0.046	0.044	[-0.132 - 0.040]	2.977E-01	5.784E-01
Left rostral middle frontal gyrus	0.019	0.035	[-0.049 - 0.087]	5.909E-01	8.104E-01
Right rostral middle frontal gyrus	0.061	0.047	[-0.030 - 0.153]	1.891E-01	5.083E-01
Left precuneus	-0.013	0.044	[-0.099 - 0.073]	7.665E-01	9.144E-01
Right precuneus	0.020	0.035	[-0.048 - 0.088]	5.557E-01	8.104E-01
Left inferior temporal gyrus	-0.042	0.038	[-0.117 - 0.032]	2.672E-01	5.784E-01
Right inferior temporal gyrus	-0.003	0.043	[-0.088 - 0.082]	9.435E-01	9.829E-01
Left lateral orbitofrontal cortex	0.003	0.049	[-0.093 - 0.099]	9.558E-01	9.829E-01
Right lateral orbitofrontal cortex	0.065	0.047	[-0.026 - 0.157]	1.618E-01	5.083E-01
Left middle temporal gyrus	0.060	0.038	[-0.015 - 0.135]	1.178E-01	5.083E-01
Right middle temporal gyrus	0.017	0.035	[-0.051 - 0.085]	6.321E-01	8.110E-01
Left postcentral gyrus	0.001	0.035	[-0.068 - 0.070]	9.829E-01	9.829E-01
Right postcentral gyrus	0.039	0.055	[-0.069 - 0.148]	4.782E-01	7.562E-01
Left medial orbitofrontal cortex	0.112	0.037	[0.039 - 0.184]	2.635E-03	4.076E-02
Right medial orbitofrontal cortex	0.013	0.035	[-0.055 - 0.081]	7.057E-01	8.800E-01
Left cuneus	-0.053	0.035	[-0.121 - 0.015]	1.271E-01	5.083E-01
Right cuneus	-0.020	0.042	[-0.103 - 0.062]	6.292E-01	8.110E-01
Left pars triangularis of inferior frontal gyrus	-0.051	0.035	[-0.119 - 0.017]	1.445E-01	5.083E-01
Right pars triangularis of inferior frontal gyrus	-0.060	0.037	[-0.134 - 0.013]	1.056E-01	5.083E-01
Left superior parietal cortex	0.037	0.035	[-0.031 - 0.105]	2.859E-01	5.784E-01
Right superior parietal cortex	0.015	0.042	[-0.066 - 0.097]	7.118E-01	8.800E-01
Left pars opercularis of inferior frontal gyrus	-0.037	0.047	[-0.130 - 0.055]	4.277E-01	7.093E-01
Right pars opercularis of inferior frontal gyrus	-0.050	0.037	[-0.123 - 0.023]	1.798E-01	5.083E-01
Left supramarginal gyrus	0.046	0.035	[-0.022 - 0.114]	1.864E-01	5.083E-01
Right supramarginal gyrus	0.052	0.035	[-0.016 - 0.120]	1.324E-01	5.083E-01
Left pericalcarine cortex	-0.042	0.035	[-0.110 - 0.026]	2.279E-01	5.683E-01
Right pericalcarine cortex	-0.040	0.038	[-0.115 - 0.035]	2.931E-01	5.784E-01
Left parahippocampal gyrus	0.026	0.047	[-0.067 - 0.118]	5.849E-01	8.104E-01
Right parahippocampal gyrus	-0.003	0.043	[-0.087 - 0.082]	9.520E-01	9.829E-01
Left caudal middle frontal gyrus	0.007	0.045	[-0.081 - 0.096]	8.693E-01	9.535E-01
Right caudal middle frontal gyrus	0.028	0.035	[-0.039 - 0.096]	4.111E-01	6.989E-01
Left transverse temporal gyrus	-0.022	0.035	[-0.090 - 0.046]	5.283E-01	8.104E-01
Right transverse temporal gyrus	0.025	0.035	[-0.043 - 0.093]	4.772E-01	7.562E-01
Left banks of superior temporal sulcus	-0.018	0.035	[-0.086 - 0.050]	6.022E-01	8.104E-01
Right banks of superior temporal sulcus	-0.009	0.040	[-0.088 - 0.070]	8.224E-01	9.321E-01
Left caudal anterior cingulate cortex	0.006	0.036	[-0.064 - 0.077]	8.601E-01	9.535E-01
Right caudal anterior cingulate cortex	-0.033	0.035	[-0.101 - 0.035]	3.398E-01	6.080E-01
Left rostral anterior cingulate cortex	0.039	0.040	[-0.039 - 0.117]	3.255E-01	5.982E-01
Right rostral anterior cingulate cortex	-0.011	0.040	[-0.090 - 0.068]	7.811E-01	9.148E-01
Left posterior cingulate cortex	0.018	0.035	[-0.049 - 0.086]	5.943E-01	8.104E-01
Right posterior cingulate cortex	0.050	0.045	[-0.038 - 0.138]	2.648E-01	5.784E-01
Left frontal pole	-0.001	0.035	[-0.069 - 0.067]	9.728E-01	9.829E-01
Right frontal pole	-0.066	0.040	[-0.145 - 0.013]	1.031E-01	5.083E-01
Left paracentral lobule	0.054	0.038	[-0.019 - 0.128]	1.489E-01	5.083E-01
Right paracentral lobule	0.030	0.035	[-0.038 - 0.098]	3.910E-01	6.817E-01
Left insula	0.111	0.047	[0.018 - 0.203]	1.876E-02	1.304E-01
Right insula	0.097	0.041	[0.016 - 0.177]	1.917E-02	1.304E-01
Left entorhinal cortex	0.012	0.047	[-0.080 - 0.104]	7.937E-01	9.148E-01
Right entorhinal cortex	0.039	0.037	[-0.034 - 0.112]	2.952E-01	5.784E-01
Left temporal pole	0.053	0.041	[-0.027 - 0.134]	1.944E-01	5.083E-01
Right temporal pole	0.047	0.047	[-0.045 - 0.140]	3.150E-01	5.950E-01
Left isthmus cingulate cortex	0.220	0.035	[0.151 - 0.290]	4.296E-10	2.921E-08
Right isthmus cingulate cortex	0.198	0.035	[0.130 - 0.266]	1.183E-08	4.021E-07

Supplementary Table S24. Cortical surface area differences between individuals with bipolar disorder and healthy comparison subjects controlling for age, sex, and total cortical surface area

	d	Std. Err.	95% CI	*p*-value	FDR *q*-value
Left superior frontal gyrus	-0.084	0.085	[-0.251 - 0.083]	3.247E-01	9.453E-01
Right superior frontal gyrus	-0.167	0.085	[-0.334 - 0.000]	4.963E-02	7.404E-01
Left pars orbitalis of inferior frontal gyrus	0.037	0.113	[-0.183 - 0.258]	7.406E-01	9.580E-01
Right pars orbitalis of inferior frontal gyrus	-0.237	0.085	[-0.404 - -0.070]	5.518E-03	3.752E-01
Left precentral gyrus	-0.070	0.169	[-0.400 - 0.261]	6.787E-01	9.580E-01
Right precentral gyrus	-0.136	0.105	[-0.342 - 0.069]	1.927E-01	9.453E-01
Left lingual gyrus	0.027	0.098	[-0.165 - 0.219]	7.838E-01	9.580E-01
Right lingual gyrus	0.063	0.085	[-0.104 - 0.230]	4.591E-01	9.453E-01
Left superior temporal gyrus	-0.005	0.085	[-0.172 - 0.161]	9.502E-01	9.789E-01
Right superior temporal gyrus	-0.034	0.085	[-0.200 - 0.133]	6.933E-01	9.580E-01
Left fusiform gyrus	-0.021	0.085	[-0.188 - 0.145]	8.012E-01	9.580E-01
Right fusiform gyrus	-0.157	0.085	[-0.324 - 0.010]	6.533E-02	7.404E-01
Left inferior parietal cortex	0.047	0.085	[-0.119 - 0.214]	5.767E-01	9.477E-01
Right inferior parietal cortex	0.111	0.085	[-0.056 - 0.278]	1.921E-01	9.453E-01
Left lateral occipital cortex	-0.023	0.090	[-0.200 - 0.155]	8.031E-01	9.580E-01
Right lateral occipital cortex	-0.135	0.085	[-0.302 - 0.032]	1.126E-01	9.453E-01
Left rostral middle frontal gyrus	0.070	0.085	[-0.097 - 0.236]	4.134E-01	9.453E-01
Right rostral middle frontal gyrus	0.182	0.126	[-0.064 - 0.429]	1.476E-01	9.453E-01
Left precuneus	-0.048	0.085	[-0.215 - 0.119]	5.722E-01	9.477E-01
Right precuneus	-0.159	0.085	[-0.327 - 0.008]	6.144E-02	7.404E-01
Left inferior temporal gyrus	0.046	0.104	[-0.157 - 0.250]	6.546E-01	9.580E-01
Right inferior temporal gyrus	0.077	0.133	[-0.185 - 0.338]	5.655E-01	9.477E-01
Left lateral orbitofrontal cortex	-0.011	0.137	[-0.279 - 0.257]	9.354E-01	9.786E-01
Right lateral orbitofrontal cortex	0.199	0.126	[-0.047 - 0.445]	1.123E-01	9.453E-01
Left middle temporal gyrus	0.048	0.091	[-0.130 - 0.227]	5.966E-01	9.477E-01
Right middle temporal gyrus	0.062	0.087	[-0.109 - 0.234]	4.744E-01	9.453E-01
Left postcentral gyrus	0.093	0.112	[-0.128 - 0.313]	4.106E-01	9.453E-01
Right postcentral gyrus	-0.075	0.085	[-0.242 - 0.092]	3.765E-01	9.453E-01
Left medial orbitofrontal cortex	0.168	0.152	[-0.129 - 0.465]	2.669E-01	9.453E-01
Right medial orbitofrontal cortex	0.104	0.092	[-0.075 - 0.284]	2.551E-01	9.453E-01
Left cuneus	-0.029	0.092	[-0.210 - 0.152]	7.554E-01	9.580E-01
Right cuneus	-0.125	0.122	[-0.365 - 0.115]	3.060E-01	9.453E-01
Left pars triangularis of inferior frontal gyrus	-0.126	0.118	[-0.358 - 0.106]	2.866E-01	9.453E-01
Right pars triangularis of inferior frontal gyrus	-0.101	0.119	[-0.335 - 0.133]	3.988E-01	9.453E-01
Left superior parietal cortex	-0.130	0.085	[-0.297 - 0.037]	1.277E-01	9.453E-01
Right superior parietal cortex	0.025	0.085	[-0.142 - 0.192]	7.661E-01	9.580E-01
Left pars opercularis of inferior frontal gyrus	-0.026	0.125	[-0.270 - 0.219]	8.375E-01	9.716E-01
Right pars opercularis of inferior frontal gyrus	-0.024	0.085	[-0.191 - 0.142]	7.739E-01	9.580E-01
Left supramarginal gyrus	0.024	0.085	[-0.143 - 0.191]	7.762E-01	9.580E-01
Right supramarginal gyrus	0.018	0.100	[-0.178 - 0.215]	8.537E-01	9.716E-01
Left pericalcarine cortex	-0.026	0.085	[-0.193 - 0.141]	7.590E-01	9.580E-01
Right pericalcarine cortex	-0.001	0.085	[-0.168 - 0.166]	9.928E-01	9.928E-01
Left parahippocampal gyrus	0.036	0.085	[-0.131 - 0.203]	6.717E-01	9.580E-01
Right parahippocampal gyrus	-0.095	0.112	[-0.315 - 0.125]	3.983E-01	9.453E-01
Left caudal middle frontal gyrus	0.069	0.085	[-0.098 - 0.236]	4.176E-01	9.453E-01
Right caudal middle frontal gyrus	0.045	0.085	[-0.122 - 0.212]	5.985E-01	9.477E-01
Left transverse temporal gyrus	-0.066	0.085	[-0.233 - 0.101]	4.366E-01	9.453E-01
Right transverse temporal gyrus	-0.059	0.085	[-0.226 - 0.108]	4.865E-01	9.453E-01
Left banks of superior temporal sulcus	0.068	0.096	[-0.120 - 0.256]	4.785E-01	9.453E-01
Right banks of superior temporal sulcus	0.049	0.099	[-0.145 - 0.242]	6.212E-01	9.580E-01
Left caudal anterior cingulate cortex	0.096	0.115	[-0.128 - 0.321]	4.005E-01	9.453E-01
Right caudal anterior cingulate cortex	0.014	0.085	[-0.153 - 0.180]	8.719E-01	9.716E-01
Left rostral anterior cingulate cortex	0.079	0.085	[-0.088 - 0.246]	3.521E-01	9.453E-01
Right rostral anterior cingulate cortex	-0.015	0.102	[-0.215 - 0.186]	8.859E-01	9.716E-01
Left posterior cingulate cortex	-0.094	0.094	[-0.279 - 0.091]	3.209E-01	9.453E-01
Right posterior cingulate cortex	0.051	0.085	[-0.116 - 0.218]	5.458E-01	9.477E-01
Left frontal pole	-0.088	0.085	[-0.255 - 0.079]	3.028E-01	9.453E-01
Right frontal pole	-0.116	0.100	[-0.313 - 0.080]	2.446E-01	9.453E-01
Left paracentral lobule	-0.009	0.085	[-0.175 - 0.158]	9.195E-01	9.786E-01
Right paracentral lobule	0.052	0.098	[-0.141 - 0.244]	5.993E-01	9.477E-01
Left insula	0.059	0.094	[-0.124 - 0.243]	5.271E-01	9.477E-01
Right insula	-0.003	0.085	[-0.170 - 0.164]	9.713E-01	9.858E-01
Left entorhinal cortex	0.224	0.092	[0.043 - 0.405]	1.505E-02	5.117E-01
Right entorhinal cortex	0.127	0.100	[-0.070 - 0.323]	2.063E-01	9.453E-01
Left temporal pole	0.074	0.086	[-0.094 - 0.243]	3.872E-01	9.453E-01
Right temporal pole	0.223	0.106	[0.015 - 0.432]	3.594E-02	7.404E-01
Left isthmus cingulate cortex	-0.007	0.085	[-0.174 - 0.160]	9.351E-01	9.786E-01
Right isthmus cingulate cortex	0.016	0.095	[-0.171 - 0.203]	8.641E-01	9.716E-01

Supplementary Table S25. Cortical surface area differences between individuals with bipolar disorder and healthy comparison subjects controlling for age, sex, and total cortical surface area at 25 years of age or older

	d	Std. Err.	95% CI	*p*-value	FDR *q*-value
Left superior frontal gyrus	-0.130	0.088	[-0.303 - 0.042]	1.387E-01	7.139E-01
Right superior frontal gyrus	-0.201	0.088	[-0.374 - -0.028]	2.267E-02	5.139E-01
Left pars orbitalis of inferior frontal gyrus	-0.015	0.110	[-0.229 - 0.200]	8.944E-01	9.726E-01
Right pars orbitalis of inferior frontal gyrus	-0.248	0.088	[-0.421 - -0.076]	4.837E-03	3.289E-01
Left precentral gyrus	-0.103	0.164	[-0.426 - 0.219]	5.302E-01	9.245E-01
Right precentral gyrus	-0.142	0.107	[-0.352 - 0.069]	1.876E-01	7.139E-01
Left lingual gyrus	0.037	0.097	[-0.153 - 0.228]	7.008E-01	9.726E-01
Right lingual gyrus	0.080	0.088	[-0.092 - 0.253]	3.610E-01	9.163E-01
Left superior temporal gyrus	-0.034	0.088	[-0.206 - 0.139]	7.019E-01	9.726E-01
Right superior temporal gyrus	-0.065	0.088	[-0.238 - 0.107]	4.595E-01	9.245E-01
Left fusiform gyrus	-0.012	0.088	[-0.185 - 0.160]	8.871E-01	9.726E-01
Right fusiform gyrus	-0.164	0.088	[-0.337 - 0.008]	6.197E-02	7.139E-01
Left inferior parietal cortex	0.057	0.088	[-0.116 - 0.229]	5.180E-01	9.245E-01
Right inferior parietal cortex	0.114	0.088	[-0.058 - 0.287]	1.946E-01	7.139E-01
Left lateral occipital cortex	-0.013	0.104	[-0.216 - 0.191]	9.037E-01	9.726E-01
Right lateral occipital cortex	-0.128	0.088	[-0.301 - 0.044]	1.451E-01	7.139E-01
Left rostral middle frontal gyrus	0.096	0.088	[-0.077 - 0.268]	2.763E-01	9.163E-01
Right rostral middle frontal gyrus	0.198	0.126	[-0.048 - 0.445]	1.145E-01	7.139E-01
Left precuneus	-0.020	0.088	[-0.192 - 0.153]	8.220E-01	9.726E-01
Right precuneus	-0.118	0.090	[-0.294 - 0.057]	1.863E-01	7.139E-01
Left inferior temporal gyrus	0.065	0.116	[-0.162 - 0.293]	5.751E-01	9.641E-01
Right inferior temporal gyrus	0.049	0.137	[-0.219 - 0.317]	7.211E-01	9.726E-01
Left lateral orbitofrontal cortex	-0.007	0.137	[-0.276 - 0.261]	9.569E-01	9.914E-01
Right lateral orbitofrontal cortex	0.217	0.125	[-0.027 - 0.461]	8.126E-02	7.139E-01
Left middle temporal gyrus	0.113	0.088	[-0.060 - 0.286]	1.995E-01	7.139E-01
Right middle temporal gyrus	0.039	0.090	[-0.139 - 0.216]	6.694E-01	9.726E-01
Left postcentral gyrus	0.079	0.123	[-0.162 - 0.320]	5.230E-01	9.245E-01
Right postcentral gyrus	-0.071	0.088	[-0.244 - 0.101]	4.192E-01	9.245E-01
Left medial orbitofrontal cortex	0.126	0.153	[-0.175 - 0.426]	4.130E-01	9.245E-01
Right medial orbitofrontal cortex	0.068	0.089	[-0.106 - 0.242]	4.435E-01	9.245E-01
Left cuneus	-0.037	0.094	[-0.221 - 0.147]	6.947E-01	9.726E-01
Right cuneus	-0.145	0.111	[-0.363 - 0.073]	1.927E-01	7.139E-01
Left pars triangularis of inferior frontal gyrus	-0.170	0.127	[-0.418 - 0.079]	1.807E-01	7.139E-01
Right pars triangularis of inferior frontal gyrus	-0.078	0.120	[-0.313 - 0.156]	5.129E-01	9.245E-01
Left superior parietal cortex	-0.122	0.088	[-0.294 - 0.051]	1.659E-01	7.139E-01
Right superior parietal cortex	0.020	0.088	[-0.153 - 0.193]	8.197E-01	9.726E-01
Left pars opercularis of inferior frontal gyrus	-0.021	0.106	[-0.229 - 0.187]	8.438E-01	9.726E-01
Right pars opercularis of inferior frontal gyrus	-0.015	0.088	[-0.187 - 0.158]	8.659E-01	9.726E-01
Left supramarginal gyrus	0.002	0.088	[-0.171 - 0.175]	9.816E-01	9.914E-01
Right supramarginal gyrus	0.022	0.107	[-0.187 - 0.232]	8.332E-01	9.726E-01
Left pericalcarine cortex	-0.014	0.088	[-0.187 - 0.158]	8.709E-01	9.726E-01
Right pericalcarine cortex	-0.013	0.088	[-0.186 - 0.159]	8.807E-01	9.726E-01
Left parahippocampal gyrus	0.001	0.088	[-0.172 - 0.173]	9.914E-01	9.914E-01
Right parahippocampal gyrus	-0.106	0.109	[-0.319 - 0.107]	3.299E-01	9.163E-01
Left caudal middle frontal gyrus	0.032	0.088	[-0.140 - 0.205]	7.129E-01	9.726E-01
Right caudal middle frontal gyrus	0.038	0.095	[-0.149 - 0.225]	6.917E-01	9.726E-01
Left transverse temporal gyrus	-0.081	0.088	[-0.254 - 0.091]	3.569E-01	9.163E-01
Right transverse temporal gyrus	-0.045	0.088	[-0.218 - 0.127]	6.060E-01	9.726E-01
Left banks of superior temporal sulcus	0.060	0.109	[-0.153 - 0.273]	5.813E-01	9.641E-01
Right banks of superior temporal sulcus	0.062	0.088	[-0.111 - 0.234]	4.849E-01	9.245E-01
Left caudal anterior cingulate cortex	0.125	0.122	[-0.115 - 0.365]	3.069E-01	9.163E-01
Right caudal anterior cingulate cortex	-0.003	0.088	[-0.175 - 0.169]	9.736E-01	9.914E-01
Left rostral anterior cingulate cortex	0.129	0.093	[-0.053 - 0.310]	1.641E-01	7.139E-01
Right rostral anterior cingulate cortex	-0.011	0.101	[-0.209 - 0.188]	9.153E-01	9.726E-01
Left posterior cingulate cortex	-0.105	0.100	[-0.301 - 0.090]	2.913E-01	9.163E-01
Right posterior cingulate cortex	0.080	0.088	[-0.093 - 0.252]	3.638E-01	9.163E-01
Left frontal pole	-0.085	0.088	[-0.258 - 0.087]	3.334E-01	9.163E-01
Right frontal pole	-0.133	0.093	[-0.315 - 0.048]	1.506E-01	7.139E-01
Left paracentral lobule	-0.011	0.088	[-0.184 - 0.161]	8.961E-01	9.726E-01
Right paracentral lobule	0.069	0.088	[-0.104 - 0.241]	4.357E-01	9.245E-01
Left insula	0.039	0.095	[-0.146 - 0.225]	6.799E-01	9.726E-01
Right insula	-0.010	0.088	[-0.182 - 0.163]	9.119E-01	9.726E-01
Left entorhinal cortex	0.229	0.091	[0.050 - 0.407]	1.198E-02	4.075E-01
Right entorhinal cortex	0.170	0.107	[-0.039 - 0.379]	1.104E-01	7.139E-01
Left temporal pole	0.088	0.116	[-0.139 - 0.315]	4.469E-01	9.245E-01
Right temporal pole	0.225	0.105	[0.020 - 0.430]	3.151E-02	5.357E-01
Left isthmus cingulate cortex	-0.015	0.090	[-0.191 - 0.161]	8.661E-01	9.726E-01
Right isthmus cingulate cortex	0.061	0.088	[-0.111 - 0.233]	4.874E-01	9.245E-01

Supplementary Table S26. Cortical surface area differences between individuals with major depressive disorder and healthy comparison subjects controlling for age, sex, and total cortical surface area

	d	Std. Err.	95% CI	*p*-value	FDR *q*-value
Left superior frontal gyrus	-0.088	0.064	[-0.213 - 0.037]	1.684E-01	7.134E-01
Right superior frontal gyrus	-0.148	0.092	[-0.329 - 0.032]	1.072E-01	7.134E-01
Left pars orbitalis of inferior frontal gyrus	-0.075	0.061	[-0.195 - 0.045]	2.216E-01	7.134E-01
Right pars orbitalis of inferior frontal gyrus	-0.047	0.061	[-0.167 - 0.074]	4.470E-01	8.005E-01
Left precentral gyrus	-0.003	0.061	[-0.123 - 0.117]	9.587E-01	9.725E-01
Right precentral gyrus	0.059	0.061	[-0.061 - 0.179]	3.372E-01	7.738E-01
Left lingual gyrus	-0.028	0.068	[-0.161 - 0.106]	6.871E-01	9.430E-01
Right lingual gyrus	-0.042	0.061	[-0.162 - 0.079]	4.982E-01	8.067E-01
Left superior temporal gyrus	0.012	0.061	[-0.108 - 0.133]	8.389E-01	9.725E-01
Right superior temporal gyrus	-0.005	0.061	[-0.125 - 0.115]	9.330E-01	9.725E-01
Left fusiform gyrus	-0.005	0.086	[-0.174 - 0.164]	9.518E-01	9.725E-01
Right fusiform gyrus	-0.091	0.061	[-0.212 - 0.029]	1.359E-01	7.134E-01
Left inferior parietal cortex	-0.028	0.083	[-0.191 - 0.135]	7.348E-01	9.609E-01
Right inferior parietal cortex	-0.007	0.077	[-0.157 - 0.143]	9.290E-01	9.725E-01
Left lateral occipital cortex	0.008	0.061	[-0.112 - 0.128]	8.984E-01	9.725E-01
Right lateral occipital cortex	-0.056	0.061	[-0.177 - 0.064]	3.603E-01	7.738E-01
Left rostral middle frontal gyrus	0.009	0.061	[-0.111 - 0.130]	8.787E-01	9.725E-01
Right rostral middle frontal gyrus	0.070	0.071	[-0.069 - 0.210]	3.221E-01	7.738E-01
Left precuneus	-0.072	0.061	[-0.193 - 0.048]	2.378E-01	7.134E-01
Right precuneus	0.129	0.061	[0.009 - 0.250]	3.495E-02	7.134E-01
Left inferior temporal gyrus	0.017	0.061	[-0.103 - 0.137]	7.819E-01	9.725E-01
Right inferior temporal gyrus	0.057	0.074	[-0.088 - 0.203]	4.409E-01	8.005E-01
Left lateral orbitofrontal cortex	-0.009	0.061	[-0.129 - 0.111]	8.808E-01	9.725E-01
Right lateral orbitofrontal cortex	0.077	0.075	[-0.070 - 0.224]	3.052E-01	7.738E-01
Left middle temporal gyrus	-0.050	0.065	[-0.178 - 0.078]	4.473E-01	8.005E-01
Right middle temporal gyrus	-0.027	0.061	[-0.147 - 0.094]	6.650E-01	9.430E-01
Left postcentral gyrus	-0.008	0.061	[-0.128 - 0.112]	8.967E-01	9.725E-01
Right postcentral gyrus	-0.065	0.079	[-0.219 - 0.089]	4.088E-01	7.942E-01
Left medial orbitofrontal cortex	0.028	0.077	[-0.123 - 0.178]	7.172E-01	9.563E-01
Right medial orbitofrontal cortex	0.024	0.061	[-0.096 - 0.144]	6.934E-01	9.430E-01
Left cuneus	-0.124	0.075	[-0.271 - 0.023]	9.758E-02	7.134E-01
Right cuneus	-0.098	0.070	[-0.235 - 0.038]	1.586E-01	7.134E-01
Left pars triangularis of inferior frontal gyrus	-0.109	0.084	[-0.274 - 0.056]	1.967E-01	7.134E-01
Right pars triangularis of inferior frontal gyrus	-0.097	0.061	[-0.217 - 0.023]	1.126E-01	7.134E-01
Left superior parietal cortex	0.055	0.061	[-0.065 - 0.175]	3.703E-01	7.738E-01
Right superior parietal cortex	0.085	0.061	[-0.035 - 0.205]	1.668E-01	7.134E-01
Left pars opercularis of inferior frontal gyrus	-0.003	0.077	[-0.153 - 0.148]	9.725E-01	9.725E-01
Right pars opercularis of inferior frontal gyrus	0.044	0.061	[-0.076 - 0.164]	4.722E-01	8.067E-01
Left supramarginal gyrus	0.074	0.061	[-0.046 - 0.195]	2.260E-01	7.134E-01
Right supramarginal gyrus	0.035	0.061	[-0.085 - 0.155]	5.691E-01	8.600E-01
Left pericalcarine cortex	-0.089	0.061	[-0.209 - 0.031]	1.476E-01	7.134E-01
Right pericalcarine cortex	-0.140	0.065	[-0.267 - -0.013]	3.054E-02	7.134E-01
Left parahippocampal gyrus	0.060	0.061	[-0.061 - 0.180]	3.309E-01	7.738E-01
Right parahippocampal gyrus	-0.003	0.076	[-0.152 - 0.145]	9.674E-01	9.725E-01
Left caudal middle frontal gyrus	0.043	0.061	[-0.077 - 0.164]	4.789E-01	8.067E-01
Right caudal middle frontal gyrus	0.106	0.061	[-0.014 - 0.226]	8.413E-02	7.134E-01
Left transverse temporal gyrus	0.040	0.061	[-0.081 - 0.160]	5.194E-01	8.213E-01
Right transverse temporal gyrus	0.007	0.061	[-0.113 - 0.127]	9.064E-01	9.725E-01
Left banks of superior temporal sulcus	-0.022	0.073	[-0.165 - 0.121]	7.637E-01	9.725E-01
Right banks of superior temporal sulcus	0.006	0.089	[-0.167 - 0.180]	9.446E-01	9.725E-01
Left caudal anterior cingulate cortex	-0.052	0.061	[-0.172 - 0.069]	4.012E-01	7.942E-01
Right caudal anterior cingulate cortex	-0.048	0.069	[-0.183 - 0.088]	4.924E-01	8.067E-01
Left rostral anterior cingulate cortex	0.025	0.061	[-0.095 - 0.146]	6.813E-01	9.430E-01
Right rostral anterior cingulate cortex	0.039	0.064	[-0.086 - 0.164]	5.405E-01	8.354E-01
Left posterior cingulate cortex	0.028	0.061	[-0.093 - 0.148]	6.526E-01	9.430E-01
Right posterior cingulate cortex	-0.072	0.066	[-0.202 - 0.057]	2.745E-01	7.738E-01
Left frontal pole	-0.081	0.066	[-0.210 - 0.048]	2.207E-01	7.134E-01
Right frontal pole	-0.120	0.061	[-0.240 - 0.001]	5.112E-02	7.134E-01
Left paracentral lobule	0.056	0.061	[-0.064 - 0.177]	3.592E-01	7.738E-01
Right paracentral lobule	-0.075	0.075	[-0.222 - 0.072]	3.153E-01	7.738E-01
Left insula	0.089	0.061	[-0.031 - 0.210]	1.457E-01	7.134E-01
Right insula	0.073	0.062	[-0.049 - 0.195]	2.413E-01	7.134E-01
Left entorhinal cortex	0.081	0.061	[-0.040 - 0.201]	1.882E-01	7.134E-01
Right entorhinal cortex	0.113	0.061	[-0.008 - 0.233]	6.703E-02	7.134E-01
Left temporal pole	0.004	0.061	[-0.117 - 0.124]	9.545E-01	9.725E-01
Right temporal pole	0.066	0.074	[-0.080 - 0.211]	3.755E-01	7.738E-01
Left isthmus cingulate cortex	0.105	0.067	[-0.026 - 0.235]	1.170E-01	7.134E-01
Right isthmus cingulate cortex	0.158	0.061	[0.038 - 0.279]	9.956E-03	6.770E-01

Supplementary Table S27. Cortical surface area differences between individuals with major depressive disorder and healthy comparison subjects controlling for age, sex, and total cortical surface area over 21 years of old

	d	Std. Err.	95% CI	*p*-value	FDR *q*-value
Left superior frontal gyrus	-0.054	0.063	[-0.178 - 0.069]	3.881E-01	8.066E-01
Right superior frontal gyrus	-0.144	0.095	[-0.330 - 0.042]	1.302E-01	7.631E-01
Left pars orbitalis of inferior frontal gyrus	-0.067	0.063	[-0.191 - 0.056]	2.857E-01	7.631E-01
Right pars orbitalis of inferior frontal gyrus	-0.065	0.063	[-0.189 - 0.059]	3.018E-01	7.631E-01
Left precentral gyrus	-0.003	0.068	[-0.135 - 0.130]	9.689E-01	9.689E-01
Right precentral gyrus	0.056	0.063	[-0.067 - 0.180]	3.725E-01	8.066E-01
Left lingual gyrus	-0.028	0.078	[-0.180 - 0.124]	7.173E-01	9.062E-01
Right lingual gyrus	-0.045	0.063	[-0.169 - 0.078]	4.714E-01	8.580E-01
Left superior temporal gyrus	0.007	0.063	[-0.117 - 0.130]	9.177E-01	9.689E-01
Right superior temporal gyrus	-0.011	0.063	[-0.134 - 0.113]	8.659E-01	9.497E-01
Left fusiform gyrus	-0.034	0.079	[-0.188 - 0.121]	6.704E-01	9.062E-01
Right fusiform gyrus	-0.074	0.063	[-0.198 - 0.049]	2.393E-01	7.631E-01
Left inferior parietal cortex	-0.043	0.089	[-0.217 - 0.131]	6.284E-01	9.062E-01
Right inferior parietal cortex	-0.016	0.085	[-0.182 - 0.150]	8.457E-01	9.497E-01
Left lateral occipital cortex	0.008	0.063	[-0.116 - 0.132]	8.977E-01	9.689E-01
Right lateral occipital cortex	-0.062	0.063	[-0.186 - 0.061]	3.228E-01	7.631E-01
Left rostral middle frontal gyrus	0.014	0.065	[-0.114 - 0.142]	8.292E-01	9.497E-01
Right rostral middle frontal gyrus	0.075	0.078	[-0.078 - 0.228]	3.367E-01	7.631E-01
Left precuneus	-0.073	0.063	[-0.197 - 0.050]	2.455E-01	7.631E-01
Right precuneus	0.137	0.063	[0.013 - 0.260]	3.048E-02	7.505E-01
Left inferior temporal gyrus	0.027	0.063	[-0.097 - 0.150]	6.729E-01	9.062E-01
Right inferior temporal gyrus	0.049	0.079	[-0.105 - 0.204]	5.308E-01	9.056E-01
Left lateral orbitofrontal cortex	0.006	0.063	[-0.118 - 0.129]	9.287E-01	9.689E-01
Right lateral orbitofrontal cortex	0.117	0.070	[-0.020 - 0.254]	9.342E-02	7.631E-01
Left middle temporal gyrus	-0.039	0.063	[-0.163 - 0.084]	5.327E-01	9.056E-01
Right middle temporal gyrus	-0.065	0.063	[-0.189 - 0.059]	3.028E-01	7.631E-01
Left postcentral gyrus	-0.030	0.063	[-0.154 - 0.093]	6.305E-01	9.062E-01
Right postcentral gyrus	-0.097	0.083	[-0.259 - 0.064]	2.384E-01	7.631E-01
Left medial orbitofrontal cortex	0.042	0.081	[-0.118 - 0.201]	6.079E-01	9.062E-01
Right medial orbitofrontal cortex	0.022	0.063	[-0.102 - 0.145]	7.302E-01	9.062E-01
Left cuneus	-0.120	0.078	[-0.273 - 0.033]	1.240E-01	7.631E-01
Right cuneus	-0.105	0.074	[-0.250 - 0.041]	1.600E-01	7.631E-01
Left pars triangularis of inferior frontal gyrus	-0.076	0.068	[-0.209 - 0.057]	2.643E-01	7.631E-01
Right pars triangularis of inferior frontal gyrus	-0.061	0.063	[-0.185 - 0.063]	3.332E-01	7.631E-01
Left superior parietal cortex	0.049	0.063	[-0.075 - 0.173]	4.358E-01	8.420E-01
Right superior parietal cortex	0.085	0.063	[-0.039 - 0.209]	1.781E-01	7.631E-01
Left pars opercularis of inferior frontal gyrus	0.024	0.084	[-0.141 - 0.189]	7.776E-01	9.277E-01
Right pars opercularis of inferior frontal gyrus	0.048	0.063	[-0.076 - 0.172]	4.458E-01	8.420E-01
Left supramarginal gyrus	0.074	0.063	[-0.050 - 0.198]	2.422E-01	7.631E-01
Right supramarginal gyrus	0.029	0.063	[-0.094 - 0.153]	6.402E-01	9.062E-01
Left pericalcarine cortex	-0.079	0.063	[-0.203 - 0.045]	2.096E-01	7.631E-01
Right pericalcarine cortex	-0.141	0.069	[-0.276 - -0.006]	4.119E-02	7.505E-01
Left parahippocampal gyrus	0.033	0.063	[-0.091 - 0.157]	6.031E-01	9.062E-01
Right parahippocampal gyrus	-0.024	0.077	[-0.175 - 0.127]	7.573E-01	9.196E-01
Left caudal middle frontal gyrus	0.054	0.063	[-0.070 - 0.178]	3.928E-01	8.066E-01
Right caudal middle frontal gyrus	0.107	0.063	[-0.017 - 0.231]	9.048E-02	7.631E-01
Left transverse temporal gyrus	0.022	0.063	[-0.102 - 0.145]	7.289E-01	9.062E-01
Right transverse temporal gyrus	0.004	0.063	[-0.120 - 0.127]	9.529E-01	9.689E-01
Left banks of superior temporal sulcus	-0.036	0.080	[-0.194 - 0.122]	6.544E-01	9.062E-01
Right banks of superior temporal sulcus	-0.005	0.097	[-0.194 - 0.184]	9.578E-01	9.689E-01
Left caudal anterior cingulate cortex	-0.062	0.063	[-0.186 - 0.061]	3.229E-01	7.631E-01
Right caudal anterior cingulate cortex	-0.027	0.078	[-0.180 - 0.127]	7.330E-01	9.062E-01
Left rostral anterior cingulate cortex	0.011	0.063	[-0.113 - 0.135]	8.607E-01	9.497E-01
Right rostral anterior cingulate cortex	0.041	0.075	[-0.105 - 0.188]	5.796E-01	9.062E-01
Left posterior cingulate cortex	0.030	0.063	[-0.094 - 0.153]	6.362E-01	9.062E-01
Right posterior cingulate cortex	-0.091	0.075	[-0.238 - 0.056]	2.269E-01	7.631E-01
Left frontal pole	-0.079	0.069	[-0.215 - 0.056]	2.494E-01	7.631E-01
Right frontal pole	-0.133	0.066	[-0.262 - -0.003]	4.415E-02	7.505E-01
Left paracentral lobule	0.065	0.063	[-0.059 - 0.189]	3.021E-01	7.631E-01
Right paracentral lobule	-0.047	0.067	[-0.178 - 0.084]	4.795E-01	8.580E-01
Left insula	0.091	0.063	[-0.032 - 0.215]	1.481E-01	7.631E-01
Right insula	0.072	0.069	[-0.063 - 0.207]	2.975E-01	7.631E-01
Left entorhinal cortex	0.053	0.063	[-0.071 - 0.176]	4.033E-01	8.066E-01
Right entorhinal cortex	0.111	0.063	[-0.013 - 0.234]	7.976E-02	7.631E-01
Left temporal pole	0.011	0.063	[-0.112 - 0.135]	8.568E-01	9.497E-01
Right temporal pole	0.023	0.063	[-0.100 - 0.147]	7.117E-01	9.062E-01
Left isthmus cingulate cortex	0.100	0.065	[-0.028 - 0.228]	1.261E-01	7.631E-01
Right isthmus cingulate cortex	0.172	0.063	[0.048 - 0.296]	6.510E-03	4.427E-01

Supplementary Table S28. Cortical surface area differences between individuals with autism spectrum disorder and healthy comparison subjects controlling for age, sex, and total cortical surface area

	d	Std. Err.	95% CI	*p*-value	FDR *q*-value
Left superior frontal gyrus	0.041	0.080	[-0.116 - 0.198]	6.098E-01	9.448E-01
Right superior frontal gyrus	0.043	0.091	[-0.136 - 0.222]	6.365E-01	9.448E-01
Left pars orbitalis of inferior frontal gyrus	-0.078	0.086	[-0.246 - 0.091]	3.678E-01	9.448E-01
Right pars orbitalis of inferior frontal gyrus	-0.202	0.080	[-0.359 - -0.045]	1.167E-02	7.938E-01
Left precentral gyrus	0.046	0.124	[-0.197 - 0.288]	7.114E-01	9.448E-01
Right precentral gyrus	0.099	0.133	[-0.161 - 0.359]	4.553E-01	9.448E-01
Left lingual gyrus	-0.028	0.121	[-0.265 - 0.208]	8.135E-01	9.448E-01
Right lingual gyrus	-0.088	0.114	[-0.311 - 0.135]	4.404E-01	9.448E-01
Left superior temporal gyrus	0.021	0.080	[-0.135 - 0.178]	7.883E-01	9.448E-01
Right superior temporal gyrus	0.074	0.118	[-0.158 - 0.305]	5.330E-01	9.448E-01
Left fusiform gyrus	-0.066	0.080	[-0.223 - 0.091]	4.128E-01	9.448E-01
Right fusiform gyrus	0.107	0.083	[-0.056 - 0.270]	1.985E-01	9.448E-01
Left inferior parietal cortex	-0.071	0.080	[-0.228 - 0.086]	3.729E-01	9.448E-01
Right inferior parietal cortex	-0.085	0.080	[-0.242 - 0.072]	2.890E-01	9.448E-01
Left lateral occipital cortex	0.037	0.110	[-0.178 - 0.252]	7.346E-01	9.448E-01
Right lateral occipital cortex	0.019	0.101	[-0.179 - 0.218]	8.500E-01	9.448E-01
Left rostral middle frontal gyrus	0.057	0.144	[-0.226 - 0.339]	6.939E-01	9.448E-01
Right rostral middle frontal gyrus	-0.021	0.080	[-0.178 - 0.136]	7.905E-01	9.448E-01
Left precuneus	0.124	0.080	[-0.033 - 0.281]	1.214E-01	9.448E-01
Right precuneus	0.061	0.080	[-0.096 - 0.218]	4.470E-01	9.448E-01
Left inferior temporal gyrus	0.072	0.135	[-0.192 - 0.337]	5.909E-01	9.448E-01
Right inferior temporal gyrus	0.056	0.089	[-0.118 - 0.231]	5.275E-01	9.448E-01
Left lateral orbitofrontal cortex	-0.008	0.080	[-0.165 - 0.149]	9.199E-01	9.452E-01
Right lateral orbitofrontal cortex	0.044	0.119	[-0.190 - 0.277]	7.131E-01	9.448E-01
Left middle temporal gyrus	0.110	0.087	[-0.061 - 0.281]	2.091E-01	9.448E-01
Right middle temporal gyrus	-0.006	0.112	[-0.226 - 0.214]	9.540E-01	9.540E-01
Left postcentral gyrus	-0.040	0.080	[-0.197 - 0.117]	6.146E-01	9.448E-01
Right postcentral gyrus	-0.055	0.080	[-0.212 - 0.102]	4.908E-01	9.448E-01
Left medial orbitofrontal cortex	-0.039	0.080	[-0.196 - 0.118]	6.287E-01	9.448E-01
Right medial orbitofrontal cortex	-0.034	0.080	[-0.191 - 0.122]	6.667E-01	9.448E-01
Left cuneus	-0.042	0.094	[-0.227 - 0.143]	6.573E-01	9.448E-01
Right cuneus	-0.117	0.086	[-0.286 - 0.053]	1.766E-01	9.448E-01
Left pars triangularis of inferior frontal gyrus	-0.035	0.080	[-0.192 - 0.122]	6.597E-01	9.448E-01
Right pars triangularis of inferior frontal gyrus	0.020	0.095	[-0.166 - 0.206]	8.343E-01	9.448E-01
Left superior parietal cortex	-0.122	0.101	[-0.319 - 0.076]	2.270E-01	9.448E-01
Right superior parietal cortex	-0.103	0.080	[-0.260 - 0.054]	1.968E-01	9.448E-01
Left pars opercularis of inferior frontal gyrus	-0.020	0.080	[-0.177 - 0.136]	7.995E-01	9.448E-01
Right pars opercularis of inferior frontal gyrus	-0.022	0.089	[-0.196 - 0.152]	8.041E-01	9.448E-01
Left supramarginal gyrus	0.014	0.114	[-0.210 - 0.237]	9.056E-01	9.452E-01
Right supramarginal gyrus	0.032	0.090	[-0.145 - 0.210]	7.217E-01	9.448E-01
Left pericalcarine cortex	-0.055	0.121	[-0.292 - 0.183]	6.531E-01	9.448E-01
Right pericalcarine cortex	-0.066	0.130	[-0.320 - 0.189]	6.128E-01	9.448E-01
Left parahippocampal gyrus	-0.026	0.080	[-0.183 - 0.131]	7.421E-01	9.448E-01
Right parahippocampal gyrus	0.110	0.120	[-0.126 - 0.346]	3.621E-01	9.448E-01
Left caudal middle frontal gyrus	-0.127	0.080	[-0.284 - 0.030]	1.132E-01	9.448E-01
Right caudal middle frontal gyrus	0.034	0.097	[-0.157 - 0.224]	7.294E-01	9.448E-01
Left transverse temporal gyrus	0.077	0.085	[-0.090 - 0.245]	3.652E-01	9.448E-01
Right transverse temporal gyrus	0.007	0.080	[-0.150 - 0.164]	9.313E-01	9.452E-01
Left banks of superior temporal sulcus	0.098	0.086	[-0.071 - 0.267]	2.541E-01	9.448E-01
Right banks of superior temporal sulcus	-0.019	0.085	[-0.185 - 0.147]	8.205E-01	9.448E-01
Left caudal anterior cingulate cortex	0.044	0.107	[-0.166 - 0.254]	6.828E-01	9.448E-01
Right caudal anterior cingulate cortex	-0.017	0.091	[-0.194 - 0.161]	8.526E-01	9.448E-01
Left rostral anterior cingulate cortex	0.020	0.080	[-0.136 - 0.177]	7.978E-01	9.448E-01
Right rostral anterior cingulate cortex	0.031	0.080	[-0.126 - 0.188]	6.964E-01	9.448E-01
Left posterior cingulate cortex	-0.039	0.080	[-0.196 - 0.118]	6.252E-01	9.448E-01
Right posterior cingulate cortex	-0.041	0.123	[-0.283 - 0.201]	7.406E-01	9.448E-01
Left frontal pole	-0.028	0.099	[-0.223 - 0.167]	7.781E-01	9.448E-01
Right frontal pole	0.082	0.080	[-0.075 - 0.239]	3.045E-01	9.448E-01
Left paracentral lobule	0.038	0.080	[-0.119 - 0.195]	6.380E-01	9.448E-01
Right paracentral lobule	0.017	0.080	[-0.140 - 0.174]	8.287E-01	9.448E-01
Left insula	0.014	0.080	[-0.143 - 0.171]	8.615E-01	9.448E-01
Right insula	0.014	0.080	[-0.143 - 0.171]	8.597E-01	9.448E-01
Left entorhinal cortex	-0.159	0.097	[-0.349 - 0.032]	1.024E-01	9.448E-01
Right entorhinal cortex	-0.008	0.080	[-0.165 - 0.149]	9.232E-01	9.452E-01
Left temporal pole	0.010	0.080	[-0.146 - 0.167]	8.963E-01	9.452E-01
Right temporal pole	-0.039	0.090	[-0.217 - 0.138]	6.624E-01	9.448E-01
Left isthmus cingulate cortex	-0.081	0.080	[-0.237 - 0.076]	3.140E-01	9.448E-01
Right isthmus cingulate cortex	0.061	0.104	[-0.143 - 0.264]	5.587E-01	9.448E-01

Supplementary Table S29. Cortical surface area differences between individuals with bipolar disorder and healthy comparison subjects controlling for age, sex, age × sex, age^2, and age^2 × sex at 25 years of age or older

	d	Std. Err.	95% CI	*p*-value	FDR *q*-value
Total cortical surface area	0.020	0.096	[-0.168 - 0.207]	8.373E-01	9.860E-01
Left hemisphere	0.029	0.092	[-0.152 - 0.209]	7.562E-01	9.860E-01
Right hemisphere	0.010	0.100	[-0.186 - 0.206]	9.229E-01	9.860E-01
Left superior frontal gyrus	-0.070	0.105	[-0.275 - 0.136]	5.069E-01	9.860E-01
Right superior frontal gyrus	-0.099	0.088	[-0.272 - 0.073]	2.590E-01	9.860E-01
Left pars orbitalis of inferior frontal gyrus	0.012	0.088	[-0.161 - 0.184]	8.942E-01	9.860E-01
Right pars orbitalis of inferior frontal gyrus	-0.186	0.088	[-0.358 - -0.013]	3.512E-02	7.476E-01
Left precentral gyrus	-0.098	0.174	[-0.439 - 0.244]	5.759E-01	9.860E-01
Right precentral gyrus	-0.134	0.127	[-0.383 - 0.115]	2.900E-01	9.860E-01
Left lingual gyrus	0.038	0.124	[-0.206 - 0.281]	7.620E-01	9.860E-01
Right lingual gyrus	0.070	0.088	[-0.102 - 0.243]	4.234E-01	9.860E-01
Left superior temporal gyrus	-0.050	0.116	[-0.277 - 0.177]	6.647E-01	9.860E-01
Right superior temporal gyrus	-0.031	0.088	[-0.204 - 0.141]	7.240E-01	9.860E-01
Left fusiform gyrus	0.015	0.088	[-0.157 - 0.188]	8.620E-01	9.860E-01
Right fusiform gyrus	-0.096	0.088	[-0.268 - 0.077]	2.769E-01	9.860E-01
Left inferior parietal cortex	0.009	0.106	[-0.198 - 0.216]	9.304E-01	9.860E-01
Right inferior parietal cortex	0.068	0.119	[-0.165 - 0.301]	5.659E-01	9.860E-01
Left lateral occipital cortex	-0.019	0.129	[-0.272 - 0.234]	8.848E-01	9.860E-01
Right lateral occipital cortex	-0.099	0.112	[-0.317 - 0.120]	3.767E-01	9.860E-01
Left rostral middle frontal gyrus	0.087	0.088	[-0.086 - 0.260]	3.237E-01	9.860E-01
Right rostral middle frontal gyrus	0.159	0.107	[-0.050 - 0.368]	1.362E-01	9.860E-01
Left precuneus	0.008	0.088	[-0.165 - 0.180]	9.319E-01	9.860E-01
Right precuneus	-0.070	0.100	[-0.267 - 0.126]	4.848E-01	9.860E-01
Left inferior temporal gyrus	0.065	0.090	[-0.111 - 0.242]	4.685E-01	9.860E-01
Right inferior temporal gyrus	0.024	0.132	[-0.235 - 0.283]	8.565E-01	9.860E-01
Left lateral orbitofrontal cortex	-0.008	0.088	[-0.181 - 0.164]	9.258E-01	9.860E-01
Right lateral orbitofrontal cortex	0.179	0.088	[0.006 - 0.352]	4.212E-02	7.476E-01
Left middle temporal gyrus	0.067	0.112	[-0.152 - 0.286]	5.501E-01	9.860E-01
Right middle temporal gyrus	0.008	0.144	[-0.275 - 0.290]	9.583E-01	9.860E-01
Left postcentral gyrus	0.074	0.117	[-0.156 - 0.304]	5.274E-01	9.860E-01
Right postcentral gyrus	-0.028	0.105	[-0.235 - 0.178]	7.867E-01	9.860E-01
Left medial orbitofrontal cortex	0.061	0.088	[-0.113 - 0.234]	4.918E-01	9.860E-01
Right medial orbitofrontal cortex	0.054	0.088	[-0.118 - 0.226]	5.383E-01	9.860E-01
Left cuneus	-0.021	0.114	[-0.245 - 0.203]	8.548E-01	9.860E-01
Right cuneus	-0.161	0.141	[-0.438 - 0.116]	2.544E-01	9.860E-01
Left pars triangularis of inferior frontal gyrus	-0.130	0.115	[-0.354 - 0.095]	2.589E-01	9.860E-01
Right pars triangularis of inferior frontal gyrus	-0.051	0.129	[-0.304 - 0.202]	6.928E-01	9.860E-01
Left superior parietal cortex	-0.063	0.088	[-0.236 - 0.109]	4.726E-01	9.860E-01
Right superior parietal cortex	0.038	0.088	[-0.134 - 0.211]	6.647E-01	9.860E-01
Left pars opercularis of inferior frontal gyrus	-0.005	0.088	[-0.177 - 0.168]	9.576E-01	9.860E-01
Right pars opercularis of inferior frontal gyrus	0.020	0.088	[-0.153 - 0.192]	8.204E-01	9.860E-01
Left supramarginal gyrus	0.045	0.088	[-0.128 - 0.218]	6.089E-01	9.860E-01
Right supramarginal gyrus	0.026	0.088	[-0.147 - 0.198]	7.720E-01	9.860E-01
Left pericalcarine cortex	0.005	0.088	[-0.167 - 0.177]	9.542E-01	9.860E-01
Right pericalcarine cortex	-0.002	0.088	[-0.174 - 0.170]	9.819E-01	9.959E-01
Left parahippocampal gyrus	0.000	0.089	[-0.174 - 0.175]	9.962E-01	9.962E-01
Right parahippocampal gyrus	-0.077	0.111	[-0.294 - 0.140]	4.886E-01	9.860E-01
Left caudal middle frontal gyrus	0.035	0.088	[-0.137 - 0.208]	6.894E-01	9.860E-01
Right caudal middle frontal gyrus	0.041	0.097	[-0.149 - 0.231]	6.698E-01	9.860E-01
Left transverse temporal gyrus	-0.045	0.088	[-0.218 - 0.127]	6.082E-01	9.860E-01
Right transverse temporal gyrus	-0.039	0.088	[-0.212 - 0.133]	6.548E-01	9.860E-01
Left banks of superior temporal sulcus	0.058	0.097	[-0.132 - 0.249]	5.494E-01	9.860E-01
Right banks of superior temporal sulcus	0.069	0.090	[-0.108 - 0.245]	4.457E-01	9.860E-01
Left caudal anterior cingulate cortex	0.104	0.114	[-0.120 - 0.328]	3.624E-01	9.860E-01
Right caudal anterior cingulate cortex	-0.017	0.088	[-0.190 - 0.155]	8.423E-01	9.860E-01
Left rostral anterior cingulate cortex	0.100	0.094	[-0.083 - 0.284]	2.839E-01	9.860E-01
Right rostral anterior cingulate cortex	-0.039	0.111	[-0.256 - 0.179]	7.264E-01	9.860E-01
Left posterior cingulate cortex	-0.064	0.088	[-0.236 - 0.108]	4.677E-01	9.860E-01
Right posterior cingulate cortex	0.066	0.088	[-0.106 - 0.239]	4.502E-01	9.860E-01
Left frontal pole	-0.086	0.088	[-0.259 - 0.086]	3.275E-01	9.860E-01
Right frontal pole	-0.119	0.088	[-0.292 - 0.054]	1.766E-01	9.860E-01
Left paracentral lobule	-0.031	0.088	[-0.204 - 0.141]	7.240E-01	9.860E-01
Right paracentral lobule	0.062	0.088	[-0.111 - 0.234]	4.840E-01	9.860E-01
Left insula	0.064	0.088	[-0.108 - 0.237]	4.640E-01	9.860E-01
Right insula	0.013	0.088	[-0.160 - 0.185]	8.848E-01	9.860E-01
Left entorhinal cortex	0.199	0.088	[0.026 - 0.372]	2.402E-02	7.476E-01
Right entorhinal cortex	0.170	0.107	[-0.039 - 0.379]	1.107E-01	9.860E-01
Left temporal pole	0.087	0.128	[-0.165 - 0.338]	4.988E-01	9.860E-01
Right temporal pole	0.237	0.110	[0.022 - 0.452]	3.109E-02	7.476E-01
Left isthmus cingulate cortex	0.011	0.088	[-0.161 - 0.184]	8.981E-01	9.860E-01
Right isthmus cingulate cortex	0.046	0.095	[-0.140 - 0.231]	6.297E-01	9.860E-01

Supplementary Table S30. Cortical surface area differences between individuals with bipolar disorder and healthy comparison subjects controlling for age, sex, age × sex, age^2, age^2 × sex, and ICV at 25 years of age or older

	d	Std. Err.	95% CI	*p*-value	FDR *q*-value
Total cortical surface area	-0.031	0.134	[-0.293 - 0.231]	8.162E-01	9.988E-01
Left hemisphere	-0.020	0.130	[-0.276 - 0.235]	8.755E-01	9.988E-01
Right hemisphere	-0.044	0.137	[-0.313 - 0.226]	7.508E-01	9.988E-01
Left superior frontal gyrus	-0.135	0.120	[-0.369 - 0.100]	2.598E-01	9.988E-01
Right superior frontal gyrus	-0.152	0.100	[-0.347 - 0.044]	1.286E-01	9.988E-01
Left pars orbitalis of inferior frontal gyrus	-0.005	0.092	[-0.185 - 0.175]	9.560E-01	9.988E-01
Right pars orbitalis of inferior frontal gyrus	-0.212	0.088	[-0.384 - -0.039]	1.625E-02	7.708E-01
Left precentral gyrus	-0.157	0.196	[-0.540 - 0.227]	4.237E-01	9.988E-01
Right precentral gyrus	-0.193	0.148	[-0.482 - 0.096]	1.905E-01	9.988E-01
Left lingual gyrus	0.032	0.122	[-0.207 - 0.272]	7.906E-01	9.988E-01
Right lingual gyrus	0.055	0.088	[-0.117 - 0.227]	5.323E-01	9.988E-01
Left superior temporal gyrus	-0.084	0.123	[-0.325 - 0.157]	4.927E-01	9.988E-01
Right superior temporal gyrus	-0.054	0.088	[-0.227 - 0.118]	5.362E-01	9.988E-01
Left fusiform gyrus	0.000	0.093	[-0.182 - 0.182]	9.988E-01	9.988E-01
Right fusiform gyrus	-0.128	0.088	[-0.301 - 0.044]	1.443E-01	9.988E-01
Left inferior parietal cortex	-0.012	0.119	[-0.246 - 0.222]	9.187E-01	9.988E-01
Right inferior parietal cortex	0.064	0.121	[-0.174 - 0.302]	5.969E-01	9.988E-01
Left lateral occipital cortex	-0.076	0.156	[-0.381 - 0.229]	6.234E-01	9.988E-01
Right lateral occipital cortex	-0.132	0.115	[-0.358 - 0.093]	2.501E-01	9.988E-01
Left rostral middle frontal gyrus	0.073	0.091	[-0.105 - 0.251]	4.198E-01	9.988E-01
Right rostral middle frontal gyrus	0.135	0.120	[-0.100 - 0.370]	2.601E-01	9.988E-01
Left precuneus	-0.005	0.088	[-0.178 - 0.167]	9.533E-01	9.988E-01
Right precuneus	-0.108	0.106	[-0.316 - 0.100]	3.074E-01	9.988E-01
Left inferior temporal gyrus	0.069	0.108	[-0.142 - 0.280]	5.209E-01	9.988E-01
Right inferior temporal gyrus	0.010	0.149	[-0.283 - 0.302]	9.472E-01	9.988E-01
Left lateral orbitofrontal cortex	-0.036	0.102	[-0.235 - 0.163]	7.213E-01	9.988E-01
Right lateral orbitofrontal cortex	0.187	0.088	[0.014 - 0.360]	3.401E-02	7.708E-01
Left middle temporal gyrus	0.064	0.107	[-0.146 - 0.274]	5.501E-01	9.988E-01
Right middle temporal gyrus	-0.021	0.161	[-0.336 - 0.293]	8.942E-01	9.988E-01
Left postcentral gyrus	0.073	0.117	[-0.157 - 0.303]	5.353E-01	9.988E-01
Right postcentral gyrus	-0.045	0.116	[-0.273 - 0.182]	6.966E-01	9.988E-01
Left medial orbitofrontal cortex	0.063	0.115	[-0.163 - 0.289]	5.858E-01	9.988E-01
Right medial orbitofrontal cortex	0.053	0.088	[-0.119 - 0.225]	5.468E-01	9.988E-01
Left cuneus	-0.033	0.119	[-0.267 - 0.201]	7.822E-01	9.988E-01
Right cuneus	-0.215	0.160	[-0.529 - 0.098]	1.786E-01	9.988E-01
Left pars triangularis of inferior frontal gyrus	-0.145	0.114	[-0.368 - 0.079]	2.041E-01	9.988E-01
Right pars triangularis of inferior frontal gyrus	-0.053	0.129	[-0.306 - 0.200]	6.828E-01	9.988E-01
Left superior parietal cortex	-0.096	0.088	[-0.268 - 0.077]	2.777E-01	9.988E-01
Right superior parietal cortex	0.022	0.088	[-0.150 - 0.195]	8.000E-01	9.988E-01
Left pars opercularis of inferior frontal gyrus	-0.001	0.096	[-0.189 - 0.188]	9.947E-01	9.988E-01
Right pars opercularis of inferior frontal gyrus	0.011	0.088	[-0.162 - 0.183]	9.018E-01	9.988E-01
Left supramarginal gyrus	0.028	0.093	[-0.154 - 0.210]	7.629E-01	9.988E-01
Right supramarginal gyrus	0.007	0.096	[-0.181 - 0.195]	9.418E-01	9.988E-01
Left pericalcarine cortex	0.001	0.088	[-0.172 - 0.173]	9.949E-01	9.988E-01
Right pericalcarine cortex	-0.009	0.088	[-0.182 - 0.163]	9.150E-01	9.988E-01
Left parahippocampal gyrus	-0.015	0.092	[-0.194 - 0.165]	8.738E-01	9.988E-01
Right parahippocampal gyrus	-0.106	0.119	[-0.339 - 0.128]	3.739E-01	9.988E-01
Left caudal middle frontal gyrus	0.022	0.094	[-0.163 - 0.206]	8.193E-01	9.988E-01
Right caudal middle frontal gyrus	0.030	0.103	[-0.173 - 0.232]	7.738E-01	9.988E-01
Left transverse temporal gyrus	-0.060	0.088	[-0.233 - 0.112]	4.937E-01	9.988E-01
Right transverse temporal gyrus	-0.052	0.088	[-0.225 - 0.120]	5.534E-01	9.988E-01
Left banks of superior temporal sulcus	0.052	0.101	[-0.146 - 0.251]	6.072E-01	9.988E-01
Right banks of superior temporal sulcus	0.063	0.095	[-0.123 - 0.248]	5.081E-01	9.988E-01
Left caudal anterior cingulate cortex	0.109	0.127	[-0.140 - 0.359]	3.903E-01	9.988E-01
Right caudal anterior cingulate cortex	-0.030	0.088	[-0.203 - 0.142]	7.316E-01	9.988E-01
Left rostral anterior cingulate cortex	0.087	0.109	[-0.127 - 0.300]	4.256E-01	9.988E-01
Right rostral anterior cingulate cortex	-0.056	0.112	[-0.276 - 0.164]	6.166E-01	9.988E-01
Left posterior cingulate cortex	-0.087	0.088	[-0.259 - 0.085]	3.230E-01	9.988E-01
Right posterior cingulate cortex	0.058	0.088	[-0.114 - 0.231]	5.083E-01	9.988E-01
Left frontal pole	-0.099	0.088	[-0.271 - 0.074]	2.615E-01	9.988E-01
Right frontal pole	-0.124	0.088	[-0.297 - 0.048]	1.579E-01	9.988E-01
Left paracentral lobule	-0.052	0.088	[-0.225 - 0.120]	5.526E-01	9.988E-01
Right paracentral lobule	0.061	0.090	[-0.115 - 0.237]	4.986E-01	9.988E-01
Left insula	0.059	0.088	[-0.113 - 0.232]	5.019E-01	9.988E-01
Right insula	-0.006	0.104	[-0.210 - 0.199]	9.560E-01	9.988E-01
Left entorhinal cortex	0.191	0.088	[0.018 - 0.363]	3.057E-02	7.708E-01
Right entorhinal cortex	0.181	0.110	[-0.035 - 0.397]	9.968E-02	9.988E-01
Left temporal pole	0.079	0.133	[-0.183 - 0.340]	5.549E-01	9.988E-01
Right temporal pole	0.247	0.122	[0.007 - 0.486]	4.342E-02	7.708E-01
Left isthmus cingulate cortex	-0.004	0.088	[-0.176 - 0.168]	9.634E-01	9.988E-01
Right isthmus cingulate cortex	0.030	0.093	[-0.151 - 0.212]	7.426E-01	9.988E-01

Supplementary Table S31. Cortical volume differences between individuals with schizophrenia and healthy comparison subjects controlling for age and sex

	d	Std. Err.	95% CI	*p*-value	FDR *q*-value
Total cortical volume	-0.559	0.038	[-0.634 - -0.483]	5.359E-48	3.805E-46
Left hemisphere	-0.557	0.039	[-0.632 - -0.481]	2.251E-47	5.327E-46
Right hemisphere	-0.555	0.038	[-0.630 - -0.480]	1.512E-47	5.327E-46
Left superior frontal gyrus	-0.544	0.043	[-0.629 - -0.460]	2.108E-36	3.741E-35
Right superior frontal gyrus	-0.510	0.044	[-0.596 - -0.424]	5.072E-31	4.501E-30
Left lateral orbitofrontal cortex	-0.495	0.058	[-0.609 - -0.382]	1.407E-17	2.855E-17
Right lateral orbitofrontal cortex	-0.424	0.047	[-0.516 - -0.333]	1.131E-19	2.769E-19
Left pars orbitalis of inferior frontal gyrus	-0.473	0.046	[-0.564 - -0.383]	1.711E-24	6.749E-24
Right pars orbitalis of inferior frontal gyrus	-0.453	0.037	[-0.525 - -0.380]	1.712E-34	2.432E-33
Left rostral middle frontal gyrus	-0.456	0.039	[-0.532 - -0.379]	1.245E-31	1.262E-30
Right rostral middle frontal gyrus	-0.397	0.043	[-0.482 - -0.312]	5.093E-20	1.339E-19
Left superior temporal gyrus	-0.404	0.035	[-0.472 - -0.335]	8.000E-31	6.311E-30
Right superior temporal gyrus	-0.453	0.041	[-0.533 - -0.374]	6.849E-29	4.743E-28
Left lingual gyrus	-0.436	0.041	[-0.516 - -0.356]	1.314E-26	6.662E-26
Right lingual gyrus	-0.450	0.040	[-0.530 - -0.371]	7.749E-29	4.743E-28
Left fusiform gyrus	-0.445	0.042	[-0.528 - -0.362]	9.131E-26	4.322E-25
Right fusiform gyrus	-0.428	0.036	[-0.498 - -0.358]	4.765E-33	5.639E-32
Left medial orbitofrontal cortex	-0.304	0.045	[-0.393 - -0.215]	2.288E-11	3.185E-11
Right medial orbitofrontal cortex	-0.442	0.054	[-0.548 - -0.337]	1.934E-16	3.520E-16
Left inferior temporal gyrus	-0.398	0.046	[-0.488 - -0.309]	2.207E-18	4.748E-18
Right inferior temporal gyrus	-0.358	0.039	[-0.433 - -0.282]	2.526E-20	6.898E-20
Left precentral gyrus	-0.390	0.035	[-0.458 - -0.321]	8.016E-29	4.743E-28
Right precentral gyrus	-0.370	0.045	[-0.458 - -0.281]	2.812E-16	4.991E-16
Left middle temporal gyrus	-0.357	0.038	[-0.431 - -0.284]	1.671E-21	5.392E-21
Right middle temporal gyrus	-0.389	0.041	[-0.469 - -0.310]	1.240E-21	4.191E-21
Left pars triangularis of inferior frontal gyrus	-0.387	0.035	[-0.456 - -0.319]	1.493E-28	8.157E-28
Right pars triangularis of inferior frontal gyrus	-0.364	0.035	[-0.433 - -0.296]	1.660E-25	7.366E-25
Left insula	-0.368	0.044	[-0.455 - -0.281]	1.251E-16	2.401E-16
Right insula	-0.353	0.045	[-0.441 - -0.265]	3.227E-15	5.091E-15
Left pars opercularis of inferior frontal gyrus	-0.368	0.051	[-0.468 - -0.267]	8.276E-13	1.175E-12
Right pars opercularis of inferior frontal gyrus	-0.362	0.036	[-0.432 - -0.292]	7.062E-24	2.639E-23
Left lateral occipital cortex	-0.323	0.035	[-0.391 - -0.254]	2.185E-20	6.206E-20
Right lateral occipital cortex	-0.361	0.035	[-0.429 - -0.293]	4.438E-25	1.854E-24
Left parahippocampal gyrus	-0.326	0.038	[-0.400 - -0.251]	9.407E-18	1.964E-17
Right parahippocampal gyrus	-0.355	0.044	[-0.442 - -0.268]	1.027E-15	1.696E-15
Left inferior parietal cortex	-0.308	0.035	[-0.376 - -0.239]	1.046E-18	2.320E-18
Right inferior parietal cortex	-0.350	0.036	[-0.420 - -0.279]	2.242E-22	7.958E-22
Left frontal pole	-0.251	0.035	[-0.319 - -0.183]	5.317E-13	7.704E-13
Right frontal pole	-0.330	0.035	[-0.399 - -0.262]	2.517E-21	7.771E-21
Left supramarginal gyrus	-0.326	0.035	[-0.395 - -0.258]	7.563E-21	2.237E-20
Right supramarginal gyrus	-0.282	0.035	[-0.350 - -0.214]	5.268E-16	9.123E-16
Left transverse temporal gyrus	-0.323	0.036	[-0.394 - -0.253]	3.482E-19	7.975E-19
Right transverse temporal gyrus	-0.280	0.035	[-0.348 - -0.212]	7.914E-16	1.338E-15
Left precuneus	-0.312	0.035	[-0.380 - -0.244]	3.443E-19	7.975E-19
Right precuneus	-0.319	0.035	[-0.387 - -0.251]	5.491E-20	1.392E-19
Left caudal middle frontal gyrus	-0.296	0.038	[-0.371 - -0.221]	1.356E-14	2.093E-14
Right caudal middle frontal gyrus	-0.261	0.039	[-0.338 - -0.184]	3.516E-11	4.710E-11
Left banks of superior temporal sulcus	-0.293	0.035	[-0.361 - -0.225]	4.134E-17	8.154E-17
Right banks of superior temporal sulcus	-0.295	0.050	[-0.394 - -0.196]	4.738E-09	5.515E-09
Left posterior cingulate cortex	-0.287	0.035	[-0.355 - -0.219]	1.690E-16	3.158E-16
Right posterior cingulate cortex	-0.294	0.048	[-0.387 - -0.200]	6.937E-10	8.492E-10
Left postcentral gyrus	-0.288	0.045	[-0.376 - -0.200]	1.235E-10	1.566E-10
Right postcentral gyrus	-0.290	0.036	[-0.361 - -0.219]	1.096E-15	1.769E-15
Left cuneus	-0.260	0.036	[-0.330 - -0.191]	2.206E-13	3.262E-13
Right cuneus	-0.267	0.035	[-0.335 - -0.199]	1.690E-14	2.553E-14
Left caudal anterior cingulate cortex	-0.192	0.035	[-0.260 - -0.124]	3.495E-08	4.002E-08
Right caudal anterior cingulate cortex	-0.241	0.037	[-0.314 - -0.169]	7.134E-11	9.209E-11
Left rostral anterior cingulate cortex	-0.237	0.043	[-0.321 - -0.153]	3.665E-08	4.131E-08
Right rostral anterior cingulate cortex	-0.211	0.035	[-0.279 - -0.143]	1.247E-09	1.501E-09
Left superior parietal cortex	-0.230	0.035	[-0.298 - -0.162]	3.709E-11	4.877E-11
Right superior parietal cortex	-0.230	0.035	[-0.299 - -0.162]	3.440E-11	4.696E-11
Left isthmus cingulate cortex	-0.203	0.043	[-0.286 - -0.119]	1.879E-06	2.022E-06
Right isthmus cingulate cortex	-0.220	0.035	[-0.288 - -0.151]	2.646E-10	3.296E-10
Left pericalcarine cortex	-0.208	0.040	[-0.286 - -0.131]	1.491E-07	1.629E-07
Right pericalcarine cortex	-0.211	0.035	[-0.279 - -0.143]	1.276E-09	1.510E-09
Left paracentral lobule	-0.154	0.035	[-0.222 - -0.086]	8.669E-06	9.187E-06
Right paracentral lobule	-0.188	0.035	[-0.256 - -0.120]	6.571E-08	7.289E-08
Left entorhinal cortex	-0.145	0.044	[-0.231 - -0.060]	8.697E-04	9.080E-04
Right entorhinal cortex	-0.076	0.041	[-0.157 - 0.005]	6.468E-02	6.561E-02
Left temporal pole	-0.105	0.047	[-0.197 - -0.013]	2.562E-02	2.636E-02
Right temporal pole	-0.075	0.050	[-0.172 - 0.022]	1.313E-01	1.313E-01

Supplementary Table S32. Cortical volume differences between individuals with bipolar disorder and healthy comparison subjects controlling for age and sex

	d	Std. Err.	95% CI	*p*-value	FDR *q*-value
Total cortical volume	-0.218	0.151	[-0.515 - 0.078]	1.490E-01	4.461E-01
Left hemisphere	-0.211	0.148	[-0.502 - 0.080]	1.561E-01	4.461E-01
Right hemisphere	-0.223	0.153	[-0.523 - 0.077]	1.456E-01	4.461E-01
Left superior frontal gyrus	-0.265	0.131	[-0.522 - -0.009]	4.271E-02	4.461E-01
Right superior frontal gyrus	-0.223	0.129	[-0.475 - 0.030]	8.348E-02	4.461E-01
Left lateral orbitofrontal cortex	-0.271	0.136	[-0.537 - -0.005]	4.548E-02	4.461E-01
Right lateral orbitofrontal cortex	-0.075	0.107	[-0.284 - 0.134]	4.828E-01	6.456E-01
Left pars orbitalis of inferior frontal gyrus	-0.251	0.129	[-0.505 - 0.002]	5.215E-02	4.461E-01
Right pars orbitalis of inferior frontal gyrus	-0.382	0.108	[-0.593 - -0.170]	4.012E-04	2.848E-02
Left rostral middle frontal gyrus	-0.160	0.139	[-0.433 - 0.113]	2.504E-01	5.387E-01
Right rostral middle frontal gyrus	-0.001	0.137	[-0.270 - 0.267]	9.928E-01	9.928E-01
Left superior temporal gyrus	-0.164	0.121	[-0.401 - 0.074]	1.767E-01	4.461E-01
Right superior temporal gyrus	-0.138	0.102	[-0.338 - 0.062]	1.772E-01	4.461E-01
Left lingual gyrus	-0.120	0.142	[-0.399 - 0.159]	3.990E-01	6.083E-01
Right lingual gyrus	-0.075	0.090	[-0.251 - 0.101]	4.027E-01	6.083E-01
Left fusiform gyrus	-0.194	0.108	[-0.405 - 0.017]	7.082E-02	4.461E-01
Right fusiform gyrus	-0.291	0.087	[-0.462 - -0.120]	8.525E-04	3.026E-02
Left medial orbitofrontal cortex	-0.085	0.104	[-0.288 - 0.118]	4.132E-01	6.112E-01
Right medial orbitofrontal cortex	-0.144	0.136	[-0.410 - 0.122]	2.889E-01	5.585E-01
Left inferior temporal gyrus	-0.127	0.095	[-0.315 - 0.060]	1.822E-01	4.461E-01
Right inferior temporal gyrus	-0.151	0.131	[-0.408 - 0.105]	2.475E-01	5.387E-01
Left precentral gyrus	-0.201	0.196	[-0.584 - 0.183]	3.054E-01	5.585E-01
Right precentral gyrus	-0.235	0.128	[-0.485 - 0.015]	6.512E-02	4.461E-01
Left middle temporal gyrus	-0.116	0.117	[-0.345 - 0.114]	3.228E-01	5.590E-01
Right middle temporal gyrus	-0.164	0.151	[-0.459 - 0.131]	2.755E-01	5.585E-01
Left pars triangularis of inferior frontal gyrus	-0.199	0.122	[-0.438 - 0.040]	1.034E-01	4.461E-01
Right pars triangularis of inferior frontal gyrus	-0.207	0.142	[-0.485 - 0.071]	1.442E-01	4.461E-01
Left insula	-0.126	0.085	[-0.292 - 0.041]	1.402E-01	4.461E-01
Right insula	-0.190	0.108	[-0.402 - 0.022]	7.948E-02	4.461E-01
Left pars opercularis of inferior frontal gyrus	-0.155	0.091	[-0.334 - 0.024]	8.917E-02	4.461E-01
Right pars opercularis of inferior frontal gyrus	-0.094	0.090	[-0.271 - 0.082]	2.952E-01	5.585E-01
Left lateral occipital cortex	-0.159	0.141	[-0.435 - 0.117]	2.592E-01	5.414E-01
Right lateral occipital cortex	-0.272	0.120	[-0.507 - -0.038]	2.281E-02	3.239E-01
Left parahippocampal gyrus	-0.063	0.101	[-0.260 - 0.134]	5.304E-01	6.725E-01
Right parahippocampal gyrus	-0.154	0.106	[-0.362 - 0.053]	1.438E-01	4.461E-01
Left inferior parietal cortex	-0.070	0.109	[-0.284 - 0.143]	5.182E-01	6.690E-01
Right inferior parietal cortex	-0.077	0.142	[-0.355 - 0.201]	5.883E-01	6.962E-01
Left frontal pole	-0.208	0.085	[-0.375 - -0.040]	1.492E-02	2.648E-01
Right frontal pole	-0.313	0.107	[-0.523 - -0.103]	3.446E-03	8.155E-02
Left supramarginal gyrus	-0.086	0.100	[-0.281 - 0.110]	3.896E-01	6.083E-01
Right supramarginal gyrus	-0.108	0.086	[-0.278 - 0.061]	2.093E-01	4.955E-01
Left transverse temporal gyrus	-0.115	0.085	[-0.282 - 0.052]	1.755E-01	4.461E-01
Right transverse temporal gyrus	-0.116	0.085	[-0.282 - 0.051]	1.745E-01	4.461E-01
Left precuneus	-0.065	0.120	[-0.301 - 0.170]	5.876E-01	6.962E-01
Right precuneus	-0.164	0.106	[-0.373 - 0.044]	1.227E-01	4.461E-01
Left caudal middle frontal gyrus	-0.027	0.085	[-0.193 - 0.140]	7.552E-01	8.511E-01
Right caudal middle frontal gyrus	-0.009	0.085	[-0.176 - 0.158]	9.162E-01	9.428E-01
Left banks of superior temporal sulcus	0.029	0.086	[-0.138 - 0.197]	7.318E-01	8.460E-01
Right banks of superior temporal sulcus	-0.063	0.088	[-0.235 - 0.108]	4.688E-01	6.456E-01
Left posterior cingulate cortex	-0.145	0.085	[-0.312 - 0.022]	8.889E-02	4.461E-01
Right posterior cingulate cortex	-0.052	0.087	[-0.222 - 0.117]	5.450E-01	6.788E-01
Left postcentral gyrus	-0.029	0.117	[-0.259 - 0.200]	8.012E-01	8.751E-01
Right postcentral gyrus	-0.165	0.122	[-0.404 - 0.074]	1.758E-01	4.461E-01
Left cuneus	-0.048	0.143	[-0.327 - 0.232]	7.388E-01	8.460E-01
Right cuneus	-0.161	0.161	[-0.476 - 0.154]	3.169E-01	5.590E-01
Left caudal anterior cingulate cortex	0.022	0.105	[-0.185 - 0.229]	8.336E-01	8.968E-01
Right caudal anterior cingulate cortex	-0.003	0.092	[-0.184 - 0.178]	9.766E-01	9.905E-01
Left rostral anterior cingulate cortex	-0.103	0.107	[-0.313 - 0.106]	3.341E-01	5.647E-01
Right rostral anterior cingulate cortex	-0.112	0.124	[-0.355 - 0.132]	3.694E-01	5.960E-01
Left superior parietal cortex	-0.096	0.102	[-0.295 - 0.104]	3.467E-01	5.724E-01
Right superior parietal cortex	-0.032	0.111	[-0.250 - 0.186]	7.720E-01	8.565E-01
Left isthmus cingulate cortex	-0.122	0.085	[-0.289 - 0.045]	1.518E-01	4.461E-01
Right isthmus cingulate cortex	-0.095	0.138	[-0.364 - 0.175]	4.910E-01	6.456E-01
Left pericalcarine cortex	-0.012	0.085	[-0.179 - 0.154]	8.839E-01	9.229E-01
Right pericalcarine cortex	0.016	0.085	[-0.150 - 0.183]	8.478E-01	8.984E-01
Left paracentral lobule	-0.087	0.085	[-0.254 - 0.080]	3.068E-01	5.585E-01
Right paracentral lobule	-0.060	0.085	[-0.227 - 0.107]	4.792E-01	6.456E-01
Left entorhinal cortex	0.105	0.085	[-0.062 - 0.271]	2.190E-01	5.015E-01
Right entorhinal cortex	0.066	0.094	[-0.118 - 0.250]	4.832E-01	6.456E-01
Left temporal pole	-0.092	0.128	[-0.343 - 0.159]	4.724E-01	6.456E-01
Right temporal pole	0.052	0.091	[-0.127 - 0.231]	5.714E-01	6.962E-01

Supplementary Table S33. Cortical volume differences between individuals with bipolar disorder and healthy comparison subjects controlling for age and sex at 25 years of age or older

	d	Std. Err.	95% CI	*p*-value	FDR *q*-value
Total cortical volume	-0.200	0.155	[-0.504 - 0.103]	1.963E-01	5.703E-01
Left hemisphere	-0.194	0.153	[-0.494 - 0.105]	2.036E-01	5.703E-01
Right hemisphere	-0.204	0.156	[-0.510 - 0.102]	1.922E-01	5.703E-01
Left superior frontal gyrus	-0.321	0.152	[-0.620 - -0.023]	3.479E-02	3.660E-01
Right superior frontal gyrus	-0.257	0.141	[-0.534 - 0.020]	6.924E-02	4.916E-01
Left lateral orbitofrontal cortex	-0.262	0.135	[-0.528 - 0.003]	5.270E-02	4.158E-01
Right lateral orbitofrontal cortex	-0.052	0.104	[-0.257 - 0.152]	6.157E-01	7.948E-01
Left pars orbitalis of inferior frontal gyrus	-0.291	0.139	[-0.564 - -0.019]	3.608E-02	3.660E-01
Right pars orbitalis of inferior frontal gyrus	-0.417	0.110	[-0.632 - -0.203]	1.404E-04	9.970E-03
Left rostral middle frontal gyrus	-0.138	0.150	[-0.432 - 0.156]	3.581E-01	6.059E-01
Right rostral middle frontal gyrus	-0.009	0.152	[-0.308 - 0.290]	9.549E-01	9.560E-01
Left superior temporal gyrus	-0.173	0.127	[-0.422 - 0.077]	1.752E-01	5.703E-01
Right superior temporal gyrus	-0.151	0.101	[-0.349 - 0.048]	1.365E-01	5.384E-01
Left lingual gyrus	-0.092	0.142	[-0.371 - 0.187]	5.196E-01	7.686E-01
Right lingual gyrus	-0.036	0.089	[-0.209 - 0.138]	6.869E-01	8.275E-01
Left fusiform gyrus	-0.175	0.113	[-0.397 - 0.047]	1.227E-01	5.384E-01
Right fusiform gyrus	-0.279	0.088	[-0.453 - -0.106]	1.582E-03	3.743E-02
Left medial orbitofrontal cortex	-0.112	0.098	[-0.304 - 0.080]	2.526E-01	5.785E-01
Right medial orbitofrontal cortex	-0.152	0.131	[-0.409 - 0.104]	2.442E-01	5.785E-01
Left inferior temporal gyrus	-0.113	0.106	[-0.321 - 0.095]	2.872E-01	5.785E-01
Right inferior temporal gyrus	-0.161	0.132	[-0.420 - 0.099]	2.254E-01	5.785E-01
Left precentral gyrus	-0.202	0.192	[-0.578 - 0.175]	2.933E-01	5.785E-01
Right precentral gyrus	-0.231	0.115	[-0.456 - -0.005]	4.491E-02	3.986E-01
Left middle temporal gyrus	-0.051	0.118	[-0.282 - 0.180]	6.659E-01	8.275E-01
Right middle temporal gyrus	-0.180	0.155	[-0.484 - 0.124]	2.467E-01	5.785E-01
Left pars triangularis of inferior frontal gyrus	-0.245	0.144	[-0.527 - 0.038]	8.936E-02	5.384E-01
Right pars triangularis of inferior frontal gyrus	-0.192	0.153	[-0.492 - 0.108]	2.088E-01	5.703E-01
Left insula	-0.139	0.088	[-0.312 - 0.033]	1.142E-01	5.384E-01
Right insula	-0.189	0.119	[-0.422 - 0.044]	1.122E-01	5.384E-01
Left pars opercularis of inferior frontal gyrus	-0.137	0.088	[-0.310 - 0.035]	1.183E-01	5.384E-01
Right pars opercularis of inferior frontal gyrus	-0.091	0.101	[-0.288 - 0.106]	3.670E-01	6.059E-01
Left lateral occipital cortex	-0.166	0.152	[-0.465 - 0.132]	2.739E-01	5.785E-01
Right lateral occipital cortex	-0.272	0.125	[-0.518 - -0.026]	2.998E-02	3.660E-01
Left parahippocampal gyrus	-0.092	0.106	[-0.300 - 0.115]	3.829E-01	6.179E-01
Right parahippocampal gyrus	-0.141	0.104	[-0.345 - 0.064]	1.780E-01	5.703E-01
Left inferior parietal cortex	-0.066	0.119	[-0.300 - 0.168]	5.814E-01	7.948E-01
Right inferior parietal cortex	-0.059	0.148	[-0.349 - 0.230]	6.876E-01	8.275E-01
Left frontal pole	-0.217	0.088	[-0.390 - -0.044]	1.374E-02	2.439E-01
Right frontal pole	-0.363	0.101	[-0.561 - -0.164]	3.461E-04	1.229E-02
Left supramarginal gyrus	-0.091	0.110	[-0.306 - 0.124]	4.064E-01	6.401E-01
Right supramarginal gyrus	-0.106	0.098	[-0.298 - 0.086]	2.805E-01	5.785E-01
Left transverse temporal gyrus	-0.131	0.088	[-0.304 - 0.041]	1.357E-01	5.384E-01
Right transverse temporal gyrus	-0.122	0.088	[-0.295 - 0.050]	1.641E-01	5.703E-01
Left precuneus	-0.031	0.136	[-0.297 - 0.235]	8.176E-01	8.796E-01
Right precuneus	-0.111	0.115	[-0.336 - 0.115]	3.365E-01	6.059E-01
Left caudal middle frontal gyrus	-0.054	0.088	[-0.227 - 0.118]	5.362E-01	7.769E-01
Right caudal middle frontal gyrus	-0.005	0.088	[-0.178 - 0.168]	9.560E-01	9.560E-01
Left banks of superior temporal sulcus	0.024	0.092	[-0.156 - 0.204]	7.947E-01	8.746E-01
Right banks of superior temporal sulcus	-0.045	0.088	[-0.217 - 0.128]	6.114E-01	7.948E-01
Left posterior cingulate cortex	-0.144	0.088	[-0.317 - 0.028]	1.013E-01	5.384E-01
Right posterior cingulate cortex	-0.031	0.088	[-0.203 - 0.142]	7.273E-01	8.398E-01
Left postcentral gyrus	-0.018	0.126	[-0.266 - 0.229]	8.851E-01	9.108E-01
Right postcentral gyrus	-0.159	0.136	[-0.424 - 0.107]	2.426E-01	5.785E-01
Left cuneus	-0.050	0.148	[-0.340 - 0.240]	7.352E-01	8.398E-01
Right cuneus	-0.172	0.154	[-0.475 - 0.131]	2.653E-01	5.785E-01
Left caudal anterior cingulate cortex	0.036	0.108	[-0.176 - 0.248]	7.371E-01	8.398E-01
Right caudal anterior cingulate cortex	-0.024	0.094	[-0.208 - 0.160]	8.007E-01	8.746E-01
Left rostral anterior cingulate cortex	-0.063	0.110	[-0.279 - 0.154]	5.712E-01	7.948E-01
Right rostral anterior cingulate cortex	-0.120	0.133	[-0.380 - 0.140]	3.643E-01	6.059E-01
Left superior parietal cortex	-0.082	0.103	[-0.284 - 0.119]	4.237E-01	6.401E-01
Right superior parietal cortex	-0.023	0.115	[-0.248 - 0.203]	8.447E-01	8.951E-01
Left isthmus cingulate cortex	-0.116	0.088	[-0.288 - 0.057]	1.889E-01	5.703E-01
Right isthmus cingulate cortex	-0.068	0.134	[-0.330 - 0.195]	6.120E-01	7.948E-01
Left pericalcarine cortex	0.013	0.088	[-0.159 - 0.186]	8.808E-01	9.108E-01
Right pericalcarine cortex	0.029	0.088	[-0.144 - 0.201]	7.452E-01	8.398E-01
Left paracentral lobule	-0.089	0.093	[-0.272 - 0.094]	3.409E-01	6.059E-01
Right paracentral lobule	-0.048	0.088	[-0.221 - 0.124]	5.826E-01	7.948E-01
Left entorhinal cortex	0.085	0.088	[-0.087 - 0.257]	3.333E-01	6.059E-01
Right entorhinal cortex	0.097	0.103	[-0.105 - 0.299]	3.466E-01	6.059E-01
Left temporal pole	-0.118	0.147	[-0.406 - 0.169]	4.201E-01	6.401E-01
Right temporal pole	0.042	0.088	[-0.131 - 0.215]	6.337E-01	8.034E-01

Supplementary Table S34. Cortical volume differences between individuals with major depressive disorder and healthy comparison subjects controlling for age and sex

	d	Std. Err.	95% CI	*p*-value	FDR *q*-value
Total cortical volume	-0.361	0.105	[-0.566 - -0.156]	5.617E-04	3.036E-03
Left hemisphere	-0.374	0.101	[-0.573 - -0.175]	2.246E-04	1.450E-03
Right hemisphere	-0.344	0.107	[-0.554 - -0.134]	1.325E-03	4.480E-03
Left superior frontal gyrus	-0.367	0.107	[-0.577 - -0.157]	6.036E-04	3.036E-03
Right superior frontal gyrus	-0.381	0.095	[-0.567 - -0.195]	5.980E-05	6.065E-04
Left lateral orbitofrontal cortex	-0.362	0.075	[-0.509 - -0.215]	1.320E-06	9.369E-05
Right lateral orbitofrontal cortex	-0.233	0.084	[-0.398 - -0.068]	5.574E-03	1.131E-02
Left pars orbitalis of inferior frontal gyrus	-0.328	0.087	[-0.498 - -0.158]	1.566E-04	1.235E-03
Right pars orbitalis of inferior frontal gyrus	-0.311	0.077	[-0.462 - -0.161]	4.967E-05	6.065E-04
Left rostral middle frontal gyrus	-0.305	0.077	[-0.456 - -0.154]	7.688E-05	6.823E-04
Right rostral middle frontal gyrus	-0.206	0.084	[-0.371 - -0.042]	1.400E-02	2.263E-02
Left superior temporal gyrus	-0.279	0.082	[-0.439 - -0.118]	6.842E-04	3.036E-03
Right superior temporal gyrus	-0.249	0.087	[-0.419 - -0.079]	4.102E-03	9.708E-03
Left lingual gyrus	-0.204	0.072	[-0.346 - -0.063]	4.744E-03	1.070E-02
Right lingual gyrus	-0.234	0.080	[-0.390 - -0.077]	3.371E-03	8.253E-03
Left fusiform gyrus	-0.224	0.089	[-0.398 - -0.049]	1.213E-02	2.050E-02
Right fusiform gyrus	-0.292	0.073	[-0.435 - -0.150]	5.815E-05	6.065E-04
Left medial orbitofrontal cortex	-0.247	0.112	[-0.466 - -0.028]	2.685E-02	3.891E-02
Right medial orbitofrontal cortex	-0.278	0.083	[-0.439 - -0.116]	7.772E-04	3.246E-03
Left inferior temporal gyrus	-0.215	0.067	[-0.345 - -0.085]	1.229E-03	4.365E-03
Right inferior temporal gyrus	-0.184	0.073	[-0.327 - -0.041]	1.175E-02	2.036E-02
Left precentral gyrus	-0.261	0.062	[-0.381 - -0.140]	2.320E-05	4.118E-04
Right precentral gyrus	-0.222	0.063	[-0.346 - -0.098]	4.586E-04	2.713E-03
Left middle temporal gyrus	-0.331	0.076	[-0.480 - -0.182]	1.300E-05	3.076E-04
Right middle temporal gyrus	-0.282	0.095	[-0.468 - -0.096]	2.916E-03	7.963E-03
Left pars triangularis of inferior frontal gyrus	-0.248	0.083	[-0.410 - -0.086]	2.647E-03	7.517E-03
Right pars triangularis of inferior frontal gyrus	-0.262	0.082	[-0.423 - -0.101]	1.448E-03	4.672E-03
Left insula	-0.196	0.082	[-0.358 - -0.035]	1.731E-02	2.731E-02
Right insula	-0.203	0.096	[-0.392 - -0.014]	3.538E-02	4.831E-02
Left pars opercularis of inferior frontal gyrus	-0.210	0.086	[-0.378 - -0.042]	1.403E-02	2.263E-02
Right pars opercularis of inferior frontal gyrus	-0.145	0.083	[-0.308 - 0.017]	7.917E-02	9.691E-02
Left lateral occipital cortex	-0.200	0.071	[-0.339 - -0.060]	4.971E-03	1.070E-02
Right lateral occipital cortex	-0.241	0.088	[-0.414 - -0.069]	6.120E-03	1.207E-02
Left parahippocampal gyrus	-0.153	0.070	[-0.291 - -0.015]	2.974E-02	4.223E-02
Right parahippocampal gyrus	-0.135	0.076	[-0.284 - 0.015]	7.719E-02	9.614E-02
Left inferior parietal cortex	-0.250	0.095	[-0.436 - -0.065]	8.247E-03	1.501E-02
Right inferior parietal cortex	-0.203	0.092	[-0.383 - -0.023]	2.678E-02	3.891E-02
Left frontal pole	-0.185	0.072	[-0.325 - -0.044]	1.008E-02	1.789E-02
Right frontal pole	-0.268	0.062	[-0.389 - -0.148]	1.284E-05	3.076E-04
Left supramarginal gyrus	-0.195	0.091	[-0.373 - -0.018]	3.132E-02	4.360E-02
Right supramarginal gyrus	-0.180	0.096	[-0.368 - 0.007]	5.944E-02	7.673E-02
Left transverse temporal gyrus	-0.113	0.074	[-0.258 - 0.031]	1.242E-01	1.494E-01
Right transverse temporal gyrus	-0.144	0.079	[-0.299 - 0.011]	6.794E-02	8.613E-02
Left precuneus	-0.245	0.075	[-0.392 - -0.098]	1.077E-03	4.023E-03
Right precuneus	-0.126	0.083	[-0.288 - 0.037]	1.293E-01	1.530E-01
Left caudal middle frontal gyrus	-0.125	0.090	[-0.301 - 0.051]	1.640E-01	1.896E-01
Right caudal middle frontal gyrus	-0.104	0.077	[-0.254 - 0.047]	1.783E-01	2.010E-01
Left banks of superior temporal sulcus	-0.166	0.061	[-0.287 - -0.046]	6.780E-03	1.301E-02
Right banks of superior temporal sulcus	-0.137	0.108	[-0.350 - 0.075]	2.048E-01	2.237E-01
Left posterior cingulate cortex	-0.182	0.061	[-0.302 - -0.062]	3.036E-03	7.984E-03
Right posterior cingulate cortex	-0.234	0.071	[-0.374 - -0.094]	1.020E-03	4.023E-03
Left postcentral gyrus	-0.219	0.095	[-0.406 - -0.032]	2.150E-02	3.249E-02
Right postcentral gyrus	-0.254	0.082	[-0.414 - -0.093]	1.918E-03	5.674E-03
Left cuneus	-0.227	0.067	[-0.357 - -0.096]	6.694E-04	3.036E-03
Right cuneus	-0.228	0.061	[-0.348 - -0.107]	2.120E-04	1.450E-03
Left caudal anterior cingulate cortex	-0.172	0.061	[-0.292 - -0.051]	5.154E-03	1.076E-02
Right caudal anterior cingulate cortex	-0.150	0.072	[-0.291 - -0.009]	3.644E-02	4.881E-02
Left rostral anterior cingulate cortex	-0.173	0.061	[-0.293 - -0.053]	4.872E-03	1.070E-02
Right rostral anterior cingulate cortex	-0.078	0.079	[-0.232 - 0.076]	3.220E-01	3.412E-01
Left superior parietal cortex	-0.141	0.061	[-0.262 - -0.021]	2.148E-02	3.249E-02
Right superior parietal cortex	-0.089	0.080	[-0.247 - 0.068]	2.648E-01	2.849E-01
Left isthmus cingulate cortex	-0.125	0.061	[-0.245 - -0.004]	4.248E-02	5.586E-02
Right isthmus cingulate cortex	-0.046	0.064	[-0.172 - 0.079]	4.678E-01	4.839E-01
Left pericalcarine cortex	-0.169	0.063	[-0.294 - -0.045]	7.638E-03	1.427E-02
Right pericalcarine cortex	-0.199	0.067	[-0.331 - -0.066]	3.228E-03	8.186E-03
Left paracentral lobule	-0.105	0.076	[-0.254 - 0.043]	1.655E-01	1.896E-01
Right paracentral lobule	-0.211	0.068	[-0.345 - -0.078]	1.899E-03	5.674E-03
Left entorhinal cortex	-0.036	0.082	[-0.197 - 0.125]	6.599E-01	6.694E-01
Right entorhinal cortex	0.044	0.061	[-0.076 - 0.165]	4.702E-01	4.839E-01
Left temporal pole	-0.101	0.077	[-0.251 - 0.049]	1.873E-01	2.078E-01
Right temporal pole	0.003	0.061	[-0.117 - 0.124]	9.558E-01	9.558E-01

Supplementary Table S35. Cortical volume differences between individuals with major depressive disorder and healthy comparison subjects controlling for age and sex over 21 years old

	d	Std. Err.	95% CI	*p*-value	FDR *q*-value
Total cortical volume	-0.323	0.104	[-0.526 - -0.119]	1.864E-03	8.010E-03
Left hemisphere	-0.334	0.100	[-0.530 - -0.139]	7.828E-04	5.053E-03
Right hemisphere	-0.307	0.107	[-0.517 - -0.098]	3.998E-03	1.183E-02
Left superior frontal gyrus	-0.318	0.102	[-0.518 - -0.117]	1.902E-03	8.010E-03
Right superior frontal gyrus	-0.354	0.094	[-0.539 - -0.169]	1.748E-04	1.551E-03
Left lateral orbitofrontal cortex	-0.337	0.070	[-0.474 - -0.200]	1.471E-06	1.045E-04
Right lateral orbitofrontal cortex	-0.202	0.080	[-0.359 - -0.045]	1.171E-02	2.519E-02
Left pars orbitalis of inferior frontal gyrus	-0.309	0.099	[-0.503 - -0.114]	1.848E-03	8.010E-03
Right pars orbitalis of inferior frontal gyrus	-0.329	0.079	[-0.484 - -0.175]	2.856E-05	5.069E-04
Left rostral middle frontal gyrus	-0.294	0.076	[-0.442 - -0.145]	1.098E-04	1.300E-03
Right rostral middle frontal gyrus	-0.186	0.084	[-0.351 - -0.021]	2.703E-02	4.597E-02
Left superior temporal gyrus	-0.259	0.077	[-0.410 - -0.108]	7.683E-04	5.053E-03
Right superior temporal gyrus	-0.230	0.090	[-0.406 - -0.053]	1.069E-02	2.371E-02
Left lingual gyrus	-0.174	0.079	[-0.328 - -0.019]	2.760E-02	4.597E-02
Right lingual gyrus	-0.201	0.076	[-0.350 - -0.051]	8.475E-03	1.951E-02
Left fusiform gyrus	-0.214	0.093	[-0.397 - -0.032]	2.122E-02	3.964E-02
Right fusiform gyrus	-0.266	0.065	[-0.394 - -0.139]	4.459E-05	6.332E-04
Left medial orbitofrontal cortex	-0.190	0.108	[-0.400 - 0.021]	7.800E-02	1.026E-01
Right medial orbitofrontal cortex	-0.250	0.081	[-0.410 - -0.091]	2.124E-03	8.378E-03
Left inferior temporal gyrus	-0.197	0.066	[-0.326 - -0.067]	2.868E-03	1.072E-02
Right inferior temporal gyrus	-0.175	0.075	[-0.323 - -0.027]	2.013E-02	3.862E-02
Left precentral gyrus	-0.241	0.063	[-0.365 - -0.117]	1.411E-04	1.431E-03
Right precentral gyrus	-0.209	0.063	[-0.332 - -0.085]	9.582E-04	5.669E-03
Left middle temporal gyrus	-0.322	0.071	[-0.461 - -0.182]	6.395E-06	1.513E-04
Right middle temporal gyrus	-0.301	0.097	[-0.491 - -0.111]	1.918E-03	8.010E-03
Left pars triangularis of inferior frontal gyrus	-0.219	0.080	[-0.375 - -0.063]	5.955E-03	1.458E-02
Right pars triangularis of inferior frontal gyrus	-0.210	0.092	[-0.391 - -0.029]	2.274E-02	4.140E-02
Left insula	-0.166	0.075	[-0.314 - -0.018]	2.784E-02	4.597E-02
Right insula	-0.175	0.098	[-0.367 - 0.017]	7.333E-02	9.824E-02
Left pars opercularis of inferior frontal gyrus	-0.183	0.091	[-0.362 - -0.004]	4.540E-02	6.578E-02
Right pars opercularis of inferior frontal gyrus	-0.143	0.093	[-0.326 - 0.039]	1.229E-01	1.531E-01
Left lateral occipital cortex	-0.173	0.072	[-0.314 - -0.031]	1.666E-02	3.285E-02
Right lateral occipital cortex	-0.222	0.092	[-0.402 - -0.043]	1.531E-02	3.105E-02
Left parahippocampal gyrus	-0.157	0.073	[-0.300 - -0.013]	3.198E-02	5.046E-02
Right parahippocampal gyrus	-0.136	0.080	[-0.292 - 0.020]	8.737E-02	1.128E-01
Left inferior parietal cortex	-0.225	0.100	[-0.421 - -0.029]	2.437E-02	4.325E-02
Right inferior parietal cortex	-0.202	0.098	[-0.394 - -0.010]	3.894E-02	5.883E-02
Left frontal pole	-0.188	0.077	[-0.339 - -0.038]	1.434E-02	2.995E-02
Right frontal pole	-0.287	0.063	[-0.411 - -0.163]	5.865E-06	1.513E-04
Left supramarginal gyrus	-0.178	0.088	[-0.350 - -0.005]	4.371E-02	6.465E-02
Right supramarginal gyrus	-0.159	0.089	[-0.333 - 0.014]	7.192E-02	9.820E-02
Left transverse temporal gyrus	-0.116	0.079	[-0.271 - 0.039]	1.409E-01	1.696E-01
Right transverse temporal gyrus	-0.126	0.082	[-0.288 - 0.036]	1.266E-01	1.549E-01
Left precuneus	-0.228	0.077	[-0.379 - -0.076]	3.204E-03	1.113E-02
Right precuneus	-0.101	0.088	[-0.273 - 0.072]	2.533E-01	2.901E-01
Left caudal middle frontal gyrus	-0.113	0.094	[-0.296 - 0.071]	2.304E-01	2.727E-01
Right caudal middle frontal gyrus	-0.086	0.085	[-0.253 - 0.081]	3.138E-01	3.427E-01
Left banks of superior temporal sulcus	-0.180	0.064	[-0.306 - -0.054]	5.232E-03	1.327E-02
Right banks of superior temporal sulcus	-0.132	0.114	[-0.356 - 0.092]	2.472E-01	2.878E-01
Left posterior cingulate cortex	-0.178	0.063	[-0.302 - -0.055]	4.733E-03	1.244E-02
Right posterior cingulate cortex	-0.238	0.076	[-0.386 - -0.089]	1.736E-03	8.010E-03
Left postcentral gyrus	-0.205	0.093	[-0.388 - -0.021]	2.854E-02	4.606E-02
Right postcentral gyrus	-0.249	0.086	[-0.417 - -0.081]	3.669E-03	1.134E-02
Left cuneus	-0.212	0.073	[-0.355 - -0.069]	3.672E-03	1.134E-02
Right cuneus	-0.213	0.063	[-0.337 - -0.089]	7.442E-04	5.053E-03
Left caudal anterior cingulate cortex	-0.181	0.063	[-0.305 - -0.057]	4.180E-03	1.187E-02
Right caudal anterior cingulate cortex	-0.132	0.078	[-0.283 - 0.020]	8.968E-02	1.137E-01
Left rostral anterior cingulate cortex	-0.186	0.063	[-0.309 - -0.062]	3.292E-03	1.113E-02
Right rostral anterior cingulate cortex	-0.066	0.088	[-0.239 - 0.107]	4.556E-01	4.757E-01
Left superior parietal cortex	-0.120	0.063	[-0.244 - 0.003]	5.674E-02	8.058E-02
Right superior parietal cortex	-0.067	0.083	[-0.230 - 0.096]	4.180E-01	4.429E-01
Left isthmus cingulate cortex	-0.118	0.063	[-0.242 - 0.005]	6.049E-02	8.421E-02
Right isthmus cingulate cortex	-0.026	0.071	[-0.165 - 0.112]	7.109E-01	7.316E-01
Left pericalcarine cortex	-0.147	0.070	[-0.284 - -0.009]	3.701E-02	5.713E-02
Right pericalcarine cortex	-0.187	0.071	[-0.326 - -0.048]	8.517E-03	1.951E-02
Left paracentral lobule	-0.076	0.070	[-0.213 - 0.061]	2.752E-01	3.053E-01
Right paracentral lobule	-0.179	0.063	[-0.303 - -0.055]	4.532E-03	1.238E-02
Left entorhinal cortex	-0.026	0.074	[-0.170 - 0.118]	7.242E-01	7.345E-01
Right entorhinal cortex	0.054	0.063	[-0.070 - 0.178]	3.929E-01	4.227E-01
Left temporal pole	-0.095	0.086	[-0.262 - 0.073]	2.695E-01	3.037E-01
Right temporal pole	-0.010	0.064	[-0.136 - 0.115]	8.720E-01	8.720E-01

Supplementary Table S36. Cortical volume differences between individuals with autism spectrum disorder and healthy comparison subjects controlling for age and sex

	d	Std. Err.	95% CI	*p*-value	FDR *q*-value
Total cortical volume	-0.161	0.123	[-0.402 - 0.079]	1.880E-01	5.133E-01
Left hemisphere	-0.165	0.116	[-0.393 - 0.063]	1.552E-01	4.940E-01
Right hemisphere	-0.159	0.127	[-0.408 - 0.090]	2.118E-01	5.185E-01
Left superior frontal gyrus	-0.091	0.114	[-0.314 - 0.132]	4.253E-01	7.057E-01
Right superior frontal gyrus	-0.085	0.115	[-0.310 - 0.140]	4.580E-01	7.069E-01
Left lateral orbitofrontal cortex	-0.139	0.089	[-0.314 - 0.036]	1.198E-01	4.940E-01
Right lateral orbitofrontal cortex	-0.209	0.105	[-0.414 - -0.004]	4.554E-02	3.592E-01
Left pars orbitalis of inferior frontal gyrus	-0.155	0.097	[-0.345 - 0.034]	1.085E-01	4.940E-01
Right pars orbitalis of inferior frontal gyrus	-0.276	0.080	[-0.433 - -0.118]	5.896E-04	4.186E-02
Left rostral middle frontal gyrus	-0.091	0.098	[-0.283 - 0.100]	3.501E-01	6.215E-01
Right rostral middle frontal gyrus	-0.128	0.092	[-0.309 - 0.053]	1.670E-01	4.940E-01
Left superior temporal gyrus	-0.095	0.080	[-0.252 - 0.062]	2.352E-01	5.313E-01
Right superior temporal gyrus	-0.041	0.104	[-0.244 - 0.163]	6.960E-01	8.179E-01
Left lingual gyrus	-0.181	0.125	[-0.426 - 0.065]	1.497E-01	4.940E-01
Right lingual gyrus	-0.250	0.091	[-0.429 - -0.071]	6.228E-03	2.211E-01
Left fusiform gyrus	-0.200	0.086	[-0.369 - -0.031]	2.038E-02	3.022E-01
Right fusiform gyrus	-0.111	0.099	[-0.304 - 0.083]	2.621E-01	5.438E-01
Left medial orbitofrontal cortex	-0.184	0.080	[-0.341 - -0.027]	2.128E-02	3.022E-01
Right medial orbitofrontal cortex	-0.131	0.080	[-0.288 - 0.026]	1.017E-01	4.940E-01
Left inferior temporal gyrus	-0.072	0.135	[-0.337 - 0.193]	5.937E-01	7.703E-01
Right inferior temporal gyrus	-0.089	0.138	[-0.359 - 0.181]	5.180E-01	7.356E-01
Left precentral gyrus	-0.150	0.080	[-0.307 - 0.007]	6.122E-02	3.951E-01
Right precentral gyrus	-0.081	0.105	[-0.288 - 0.125]	4.402E-01	7.069E-01
Left middle temporal gyrus	-0.002	0.129	[-0.255 - 0.251]	9.882E-01	9.882E-01
Right middle temporal gyrus	-0.126	0.119	[-0.359 - 0.107]	2.883E-01	5.686E-01
Left pars triangularis of inferior frontal gyrus	-0.052	0.080	[-0.209 - 0.104]	5.122E-01	7.356E-01
Right pars triangularis of inferior frontal gyrus	-0.040	0.123	[-0.281 - 0.201]	7.451E-01	8.532E-01
Left insula	-0.046	0.121	[-0.283 - 0.191]	7.027E-01	8.179E-01
Right insula	-0.121	0.099	[-0.315 - 0.073]	2.220E-01	5.254E-01
Left pars opercularis of inferior frontal gyrus	-0.089	0.080	[-0.245 - 0.068]	2.681E-01	5.438E-01
Right pars opercularis of inferior frontal gyrus	-0.123	0.080	[-0.280 - 0.034]	1.255E-01	4.940E-01
Left lateral occipital cortex	-0.091	0.080	[-0.248 - 0.066]	2.562E-01	5.438E-01
Right lateral occipital cortex	-0.103	0.103	[-0.305 - 0.099]	3.174E-01	5.868E-01
Left parahippocampal gyrus	-0.128	0.080	[-0.285 - 0.029]	1.100E-01	4.940E-01
Right parahippocampal gyrus	0.047	0.102	[-0.154 - 0.247]	6.467E-01	7.917E-01
Left inferior parietal cortex	-0.204	0.107	[-0.415 - 0.006]	5.747E-02	3.951E-01
Right inferior parietal cortex	-0.160	0.108	[-0.371 - 0.051]	1.361E-01	4.940E-01
Left frontal pole	-0.064	0.081	[-0.222 - 0.094]	4.274E-01	7.057E-01
Right frontal pole	-0.009	0.080	[-0.166 - 0.148]	9.103E-01	9.369E-01
Left supramarginal gyrus	-0.138	0.109	[-0.351 - 0.075]	2.050E-01	5.185E-01
Right supramarginal gyrus	-0.113	0.080	[-0.270 - 0.044]	1.579E-01	4.940E-01
Left transverse temporal gyrus	-0.009	0.080	[-0.166 - 0.148]	9.105E-01	9.369E-01
Right transverse temporal gyrus	-0.125	0.106	[-0.332 - 0.083]	2.395E-01	5.313E-01
Left precuneus	0.014	0.124	[-0.229 - 0.257]	9.085E-01	9.369E-01
Right precuneus	-0.070	0.118	[-0.302 - 0.162]	5.541E-01	7.584E-01
Left caudal middle frontal gyrus	-0.202	0.080	[-0.359 - -0.045]	1.163E-02	2.751E-01
Right caudal middle frontal gyrus	-0.018	0.080	[-0.175 - 0.139]	8.225E-01	9.125E-01
Left banks of superior temporal sulcus	0.051	0.105	[-0.155 - 0.256]	6.288E-01	7.833E-01
Right banks of superior temporal sulcus	-0.085	0.104	[-0.289 - 0.119]	4.143E-01	7.057E-01
Left posterior cingulate cortex	-0.039	0.097	[-0.230 - 0.151]	6.869E-01	8.179E-01
Right posterior cingulate cortex	-0.083	0.149	[-0.375 - 0.210]	5.799E-01	7.703E-01
Left postcentral gyrus	-0.117	0.118	[-0.348 - 0.115]	3.223E-01	5.868E-01
Right postcentral gyrus	-0.093	0.125	[-0.339 - 0.152]	4.566E-01	7.069E-01
Left cuneus	-0.059	0.114	[-0.282 - 0.165]	6.075E-01	7.703E-01
Right cuneus	-0.130	0.094	[-0.314 - 0.053]	1.633E-01	4.940E-01
Left caudal anterior cingulate cortex	-0.047	0.080	[-0.204 - 0.110]	5.554E-01	7.584E-01
Right caudal anterior cingulate cortex	-0.089	0.129	[-0.343 - 0.164]	4.892E-01	7.236E-01
Left rostral anterior cingulate cortex	-0.113	0.080	[-0.270 - 0.044]	1.584E-01	4.940E-01
Right rostral anterior cingulate cortex	-0.014	0.113	[-0.235 - 0.207]	9.015E-01	9.369E-01
Left superior parietal cortex	-0.217	0.105	[-0.423 - -0.011]	3.918E-02	3.585E-01
Right superior parietal cortex	-0.179	0.080	[-0.336 - -0.022]	2.575E-02	3.047E-01
Left isthmus cingulate cortex	-0.169	0.083	[-0.331 - -0.007]	4.039E-02	3.585E-01
Right isthmus cingulate cortex	-0.061	0.115	[-0.286 - 0.164]	5.967E-01	7.703E-01
Left pericalcarine cortex	-0.110	0.108	[-0.321 - 0.102]	3.092E-01	5.868E-01
Right pericalcarine cortex	-0.141	0.112	[-0.361 - 0.079]	2.091E-01	5.185E-01
Left paracentral lobule	-0.055	0.080	[-0.212 - 0.101]	4.888E-01	7.236E-01
Right paracentral lobule	-0.123	0.080	[-0.280 - 0.033]	1.232E-01	4.940E-01
Left entorhinal cortex	-0.181	0.136	[-0.448 - 0.085]	1.827E-01	5.133E-01
Right entorhinal cortex	-0.017	0.108	[-0.228 - 0.194]	8.751E-01	9.369E-01
Left temporal pole	-0.026	0.097	[-0.217 - 0.165]	7.877E-01	8.877E-01
Right temporal pole	-0.009	0.107	[-0.219 - 0.201]	9.322E-01	9.455E-01

Supplementary Table S37. Cortical volume differences between individuals with schizophrenia and healthy comparison subjects controlling for age, sex, and ICV

	d	Std. Err.	95% CI	*p*-value	FDR *q*-value
Left superior frontal gyrus	-0.575	0.050	[-0.673 - -0.477]	1.616E-30	3.663E-29
Right superior frontal gyrus	-0.523	0.053	[-0.626 - -0.419]	5.617E-23	3.183E-22
Left lateral orbitofrontal cortex	-0.490	0.061	[-0.609 - -0.371]	7.988E-16	1.873E-15
Right lateral orbitofrontal cortex	-0.398	0.058	[-0.511 - -0.286]	4.304E-12	7.505E-12
Left pars orbitalis of inferior frontal gyrus	-0.451	0.050	[-0.549 - -0.354]	1.068E-19	4.036E-19
Right pars orbitalis of inferior frontal gyrus	-0.430	0.039	[-0.507 - -0.353]	8.460E-28	1.151E-26
Left rostral middle frontal gyrus	-0.464	0.044	[-0.550 - -0.379]	2.588E-26	2.693E-25
Right rostral middle frontal gyrus	-0.369	0.051	[-0.469 - -0.269]	4.922E-13	9.046E-13
Left superior temporal gyrus	-0.386	0.036	[-0.457 - -0.315]	2.772E-26	2.693E-25
Right superior temporal gyrus	-0.446	0.035	[-0.514 - -0.377]	4.230E-37	2.876E-35
Left lingual gyrus	-0.417	0.041	[-0.498 - -0.337]	3.968E-24	2.698E-23
Right lingual gyrus	-0.430	0.038	[-0.505 - -0.355]	1.976E-29	3.359E-28
Left fusiform gyrus	-0.433	0.042	[-0.514 - -0.351]	2.262E-25	1.922E-24
Right fusiform gyrus	-0.415	0.035	[-0.483 - -0.346]	2.162E-32	7.352E-31
Left medial orbitofrontal cortex	-0.262	0.051	[-0.363 - -0.161]	3.409E-07	4.140E-07
Right medial orbitofrontal cortex	-0.420	0.050	[-0.517 - -0.322]	3.349E-17	9.488E-17
Left inferior temporal gyrus	-0.379	0.038	[-0.452 - -0.305]	6.648E-24	4.110E-23
Right inferior temporal gyrus	-0.332	0.039	[-0.408 - -0.256]	8.397E-18	2.596E-17
Left precentral gyrus	-0.376	0.043	[-0.461 - -0.291]	4.133E-18	1.405E-17
Right precentral gyrus	-0.353	0.056	[-0.463 - -0.242]	4.123E-10	5.965E-10
Left middle temporal gyrus	-0.334	0.035	[-0.403 - -0.266]	9.172E-22	4.158E-21
Right middle temporal gyrus	-0.370	0.036	[-0.441 - -0.299]	1.440E-24	1.088E-23
Left pars triangularis of inferior frontal gyrus	-0.369	0.038	[-0.444 - -0.294]	6.076E-22	2.951E-21
Right pars triangularis of inferior frontal gyrus	-0.337	0.035	[-0.405 - -0.269]	4.238E-22	2.217E-21
Left insula	-0.355	0.044	[-0.441 - -0.270]	3.972E-16	1.000E-15
Right insula	-0.336	0.046	[-0.427 - -0.245]	4.294E-13	8.111E-13
Left pars opercularis of inferior frontal gyrus	-0.335	0.051	[-0.435 - -0.235]	4.919E-11	7.433E-11
Right pars opercularis of inferior frontal gyrus	-0.332	0.035	[-0.400 - -0.263]	2.589E-21	1.035E-20
Left lateral occipital cortex	-0.295	0.035	[-0.363 - -0.227]	2.450E-17	7.243E-17
Right lateral occipital cortex	-0.328	0.041	[-0.409 - -0.247]	1.626E-15	3.686E-15
Left parahippocampal gyrus	-0.287	0.041	[-0.367 - -0.207]	2.001E-12	3.581E-12
Right parahippocampal gyrus	-0.333	0.042	[-0.415 - -0.251]	2.113E-15	4.634E-15
Left inferior parietal cortex	-0.283	0.035	[-0.351 - -0.214]	4.687E-16	1.138E-15
Right inferior parietal cortex	-0.334	0.035	[-0.402 - -0.265]	1.109E-21	4.713E-21
Left frontal pole	-0.232	0.035	[-0.300 - -0.164]	2.520E-11	3.984E-11
Right frontal pole	-0.314	0.035	[-0.382 - -0.246]	1.975E-19	7.067E-19
Left supramarginal gyrus	-0.300	0.035	[-0.368 - -0.232]	6.747E-18	2.185E-17
Right supramarginal gyrus	-0.244	0.038	[-0.318 - -0.171]	8.089E-11	1.196E-10
Left transverse temporal gyrus	-0.292	0.043	[-0.375 - -0.208]	7.271E-12	1.236E-11
Right transverse temporal gyrus	-0.254	0.035	[-0.322 - -0.186]	2.634E-13	5.118E-13
Left precuneus	-0.286	0.035	[-0.354 - -0.218]	2.062E-16	5.392E-16
Right precuneus	-0.291	0.035	[-0.359 - -0.223]	6.182E-17	1.682E-16
Left caudal middle frontal gyrus	-0.272	0.035	[-0.340 - -0.204]	5.802E-15	1.233E-14
Right caudal middle frontal gyrus	-0.230	0.035	[-0.299 - -0.162]	3.427E-11	5.297E-11
Left banks of superior temporal sulcus	-0.266	0.035	[-0.334 - -0.198]	2.192E-14	4.518E-14
Right banks of superior temporal sulcus	-0.259	0.045	[-0.346 - -0.172]	6.084E-09	8.274E-09
Left posterior cingulate cortex	-0.257	0.035	[-0.325 - -0.189]	1.560E-13	3.120E-13
Right posterior cingulate cortex	-0.264	0.050	[-0.362 - -0.165]	1.607E-07	2.062E-07
Left postcentral gyrus	-0.255	0.042	[-0.337 - -0.174]	9.177E-10	1.300E-09
Right postcentral gyrus	-0.249	0.052	[-0.351 - -0.147]	1.746E-06	2.047E-06
Left cuneus	-0.235	0.035	[-0.303 - -0.167]	1.387E-11	2.301E-11
Right cuneus	-0.234	0.035	[-0.302 - -0.166]	1.714E-11	2.775E-11
Left caudal anterior cingulate cortex	-0.164	0.036	[-0.235 - -0.093]	5.757E-06	6.418E-06
Right caudal anterior cingulate cortex	-0.218	0.037	[-0.291 - -0.145]	4.028E-09	5.590E-09
Left rostral anterior cingulate cortex	-0.201	0.042	[-0.283 - -0.118]	1.974E-06	2.275E-06
Right rostral anterior cingulate cortex	-0.182	0.037	[-0.255 - -0.109]	1.009E-06	1.204E-06
Left superior parietal cortex	-0.184	0.039	[-0.262 - -0.107]	2.877E-06	3.260E-06
Right superior parietal cortex	-0.191	0.037	[-0.263 - -0.119]	1.804E-07	2.271E-07
Left isthmus cingulate cortex	-0.160	0.039	[-0.236 - -0.085]	3.240E-05	3.497E-05
Right isthmus cingulate cortex	-0.181	0.035	[-0.249 - -0.113]	1.887E-07	2.334E-07
Left pericalcarine cortex	-0.185	0.035	[-0.253 - -0.117]	9.628E-08	1.284E-07
Right pericalcarine cortex	-0.182	0.035	[-0.250 - -0.114]	1.542E-07	2.017E-07
Left paracentral lobule	-0.110	0.035	[-0.178 - -0.042]	1.570E-03	1.669E-03
Right paracentral lobule	-0.148	0.035	[-0.216 - -0.080]	2.019E-05	2.215E-05
Left entorhinal cortex	-0.111	0.039	[-0.187 - -0.035]	4.079E-03	4.267E-03
Right entorhinal cortex	-0.046	0.042	[-0.129 - 0.037]	2.793E-01	2.835E-01
Left temporal pole	-0.074	0.047	[-0.167 - 0.019]	1.167E-01	1.202E-01
Right temporal pole	-0.044	0.050	[-0.141 - 0.054]	3.817E-01	3.817E-01

Supplementary Table S38. Cortical volume differences between individuals with bipolar disorder and healthy comparison subjects controlling for age, sex, and ICV

	d	Std. Err.	95% CI	*p*-value	FDR *q*-value
Left superior frontal gyrus	-0.350	0.148	[-0.641 - -0.059]	1.827E-02	2.071E-01
Right superior frontal gyrus	-0.292	0.149	[-0.584 - -0.001]	4.948E-02	2.971E-01
Left lateral orbitofrontal cortex	-0.322	0.149	[-0.614 - -0.030]	3.070E-02	2.971E-01
Right lateral orbitofrontal cortex	-0.096	0.107	[-0.306 - 0.113]	3.679E-01	5.559E-01
Left pars orbitalis of inferior frontal gyrus	-0.268	0.136	[-0.534 - -0.002]	4.858E-02	2.971E-01
Right pars orbitalis of inferior frontal gyrus	-0.403	0.109	[-0.616 - -0.190]	2.087E-04	7.095E-03
Left rostral middle frontal gyrus	-0.222	0.155	[-0.525 - 0.081]	1.512E-01	3.961E-01
Right rostral middle frontal gyrus	-0.028	0.145	[-0.312 - 0.255]	8.442E-01	8.888E-01
Left superior temporal gyrus	-0.212	0.128	[-0.463 - 0.039]	9.850E-02	3.349E-01
Right superior temporal gyrus	-0.177	0.102	[-0.377 - 0.022]	8.189E-02	3.338E-01
Left lingual gyrus	-0.131	0.141	[-0.408 - 0.146]	3.550E-01	5.487E-01
Right lingual gyrus	-0.090	0.088	[-0.262 - 0.082]	3.038E-01	4.918E-01
Left fusiform gyrus	-0.231	0.118	[-0.461 - 0.000]	4.978E-02	2.971E-01
Right fusiform gyrus	-0.347	0.091	[-0.526 - -0.168]	1.436E-04	7.095E-03
Left medial orbitofrontal cortex	-0.096	0.127	[-0.345 - 0.152]	4.460E-01	5.723E-01
Right medial orbitofrontal cortex	-0.176	0.150	[-0.470 - 0.117]	2.382E-01	4.481E-01
Left inferior temporal gyrus	-0.143	0.113	[-0.364 - 0.079]	2.081E-01	4.481E-01
Right inferior temporal gyrus	-0.189	0.147	[-0.477 - 0.099]	1.983E-01	4.481E-01
Left precentral gyrus	-0.260	0.211	[-0.673 - 0.153]	2.170E-01	4.481E-01
Right precentral gyrus	-0.305	0.145	[-0.590 - -0.021]	3.528E-02	2.971E-01
Left middle temporal gyrus	-0.152	0.127	[-0.402 - 0.097]	2.320E-01	4.481E-01
Right middle temporal gyrus	-0.226	0.170	[-0.559 - 0.107]	1.841E-01	4.481E-01
Left pars triangularis of inferior frontal gyrus	-0.208	0.124	[-0.450 - 0.034]	9.272E-02	3.338E-01
Right pars triangularis of inferior frontal gyrus	-0.211	0.145	[-0.494 - 0.072]	1.442E-01	3.961E-01
Left insula	-0.160	0.085	[-0.326 - 0.007]	6.113E-02	2.971E-01
Right insula	-0.230	0.126	[-0.478 - 0.017]	6.815E-02	3.089E-01
Left pars opercularis of inferior frontal gyrus	-0.162	0.103	[-0.363 - 0.039]	1.140E-01	3.595E-01
Right pars opercularis of inferior frontal gyrus	-0.106	0.092	[-0.287 - 0.075]	2.504E-01	4.481E-01
Left lateral occipital cortex	-0.194	0.153	[-0.495 - 0.107]	2.065E-01	4.481E-01
Right lateral occipital cortex	-0.318	0.127	[-0.567 - -0.069]	1.226E-02	1.863E-01
Left parahippocampal gyrus	-0.081	0.097	[-0.271 - 0.109]	4.022E-01	5.634E-01
Right parahippocampal gyrus	-0.180	0.107	[-0.389 - 0.030]	9.327E-02	3.338E-01
Left inferior parietal cortex	-0.101	0.116	[-0.328 - 0.126]	3.824E-01	5.634E-01
Right inferior parietal cortex	-0.108	0.150	[-0.403 - 0.186]	4.699E-01	5.917E-01
Left frontal pole	-0.210	0.085	[-0.377 - -0.043]	1.370E-02	1.863E-01
Right frontal pole	-0.314	0.114	[-0.538 - -0.091]	5.894E-03	1.336E-01
Left supramarginal gyrus	-0.105	0.100	[-0.302 - 0.092]	2.969E-01	4.918E-01
Right supramarginal gyrus	-0.131	0.106	[-0.339 - 0.078]	2.195E-01	4.481E-01
Left transverse temporal gyrus	-0.127	0.085	[-0.294 - 0.040]	1.352E-01	3.961E-01
Right transverse temporal gyrus	-0.134	0.085	[-0.301 - 0.033]	1.163E-01	3.595E-01
Left precuneus	-0.085	0.142	[-0.363 - 0.192]	5.463E-01	6.541E-01
Right precuneus	-0.214	0.115	[-0.439 - 0.010]	6.117E-02	2.971E-01
Left caudal middle frontal gyrus	-0.039	0.093	[-0.220 - 0.143]	6.745E-01	7.892E-01
Right caudal middle frontal gyrus	-0.017	0.085	[-0.184 - 0.150]	8.455E-01	8.888E-01
Left banks of superior temporal sulcus	0.023	0.085	[-0.144 - 0.190]	7.876E-01	8.638E-01
Right banks of superior temporal sulcus	-0.078	0.094	[-0.261 - 0.106]	4.064E-01	5.634E-01
Left posterior cingulate cortex	-0.163	0.085	[-0.329 - 0.004]	5.622E-02	2.971E-01
Right posterior cingulate cortex	-0.061	0.087	[-0.230 - 0.109]	4.841E-01	5.986E-01
Left postcentral gyrus	-0.048	0.127	[-0.296 - 0.201]	7.071E-01	8.014E-01
Right postcentral gyrus	-0.186	0.130	[-0.440 - 0.068]	1.514E-01	3.961E-01
Left cuneus	-0.051	0.145	[-0.335 - 0.233]	7.242E-01	8.073E-01
Right cuneus	-0.193	0.172	[-0.530 - 0.144]	2.611E-01	4.521E-01
Left caudal anterior cingulate cortex	0.016	0.105	[-0.190 - 0.222]	8.763E-01	8.894E-01
Right caudal anterior cingulate cortex	-0.010	0.093	[-0.193 - 0.174]	9.186E-01	9.186E-01
Left rostral anterior cingulate cortex	-0.114	0.102	[-0.314 - 0.087]	2.659E-01	4.521E-01
Right rostral anterior cingulate cortex	-0.119	0.122	[-0.359 - 0.120]	3.292E-01	5.207E-01
Left superior parietal cortex	-0.121	0.103	[-0.323 - 0.081]	2.398E-01	4.481E-01
Right superior parietal cortex	-0.047	0.116	[-0.274 - 0.180]	6.848E-01	7.892E-01
Left isthmus cingulate cortex	-0.147	0.085	[-0.314 - 0.020]	8.344E-02	3.338E-01
Right isthmus cingulate cortex	-0.110	0.143	[-0.391 - 0.170]	4.401E-01	5.723E-01
Left pericalcarine cortex	-0.016	0.085	[-0.183 - 0.151]	8.495E-01	8.888E-01
Right pericalcarine cortex	0.014	0.085	[-0.153 - 0.180]	8.731E-01	8.894E-01
Left paracentral lobule	-0.101	0.085	[-0.268 - 0.066]	2.342E-01	4.481E-01
Right paracentral lobule	-0.072	0.085	[-0.239 - 0.095]	3.981E-01	5.634E-01
Left entorhinal cortex	0.099	0.085	[-0.068 - 0.266]	2.439E-01	4.481E-01
Right entorhinal cortex	0.080	0.104	[-0.124 - 0.284]	4.446E-01	5.723E-01
Left temporal pole	-0.105	0.129	[-0.357 - 0.147]	4.142E-01	5.634E-01
Right temporal pole	0.060	0.100	[-0.136 - 0.256]	5.483E-01	6.541E-01

Supplementary Table S39. Cortical volume differences between individuals with bipolar disorder and healthy comparison subjects controlling for age, sex, and ICV at 25 years of age or older

	d	Std. Err.	95% CI	*p*-value	FDR *q*-value
Left superior frontal gyrus	-0.428	0.175	[-0.771 - -0.085]	1.449E-02	1.696E-01
Right superior frontal gyrus	-0.345	0.169	[-0.676 - -0.013]	4.159E-02	2.571E-01
Left lateral orbitofrontal cortex	-0.320	0.153	[-0.619 - -0.020]	3.642E-02	2.571E-01
Right lateral orbitofrontal cortex	-0.078	0.108	[-0.290 - 0.134]	4.699E-01	6.688E-01
Left pars orbitalis of inferior frontal gyrus	-0.317	0.147	[-0.606 - -0.029]	3.122E-02	2.571E-01
Right pars orbitalis of inferior frontal gyrus	-0.445	0.112	[-0.664 - -0.226]	6.873E-05	4.674E-03
Left rostral middle frontal gyrus	-0.208	0.168	[-0.538 - 0.122]	2.163E-01	4.745E-01
Right rostral middle frontal gyrus	-0.044	0.163	[-0.364 - 0.276]	7.888E-01	8.416E-01
Left superior temporal gyrus	-0.229	0.136	[-0.497 - 0.038]	9.256E-02	3.703E-01
Right superior temporal gyrus	-0.198	0.102	[-0.399 - 0.002]	5.260E-02	2.980E-01
Left lingual gyrus	-0.097	0.140	[-0.372 - 0.179]	4.911E-01	6.780E-01
Right lingual gyrus	-0.050	0.088	[-0.223 - 0.123]	5.693E-01	7.099E-01
Left fusiform gyrus	-0.214	0.121	[-0.452 - 0.023]	7.730E-02	3.504E-01
Right fusiform gyrus	-0.335	0.090	[-0.511 - -0.159]	1.877E-04	6.381E-03
Left medial orbitofrontal cortex	-0.122	0.123	[-0.363 - 0.119]	3.214E-01	5.344E-01
Right medial orbitofrontal cortex	-0.193	0.147	[-0.482 - 0.095]	1.896E-01	4.745E-01
Left inferior temporal gyrus	-0.125	0.123	[-0.366 - 0.115]	3.065E-01	5.344E-01
Right inferior temporal gyrus	-0.194	0.147	[-0.482 - 0.094]	1.867E-01	4.745E-01
Left precentral gyrus	-0.280	0.217	[-0.705 - 0.144]	1.954E-01	4.745E-01
Right precentral gyrus	-0.308	0.136	[-0.576 - -0.041]	2.382E-02	2.314E-01
Left middle temporal gyrus	-0.083	0.122	[-0.322 - 0.157]	4.986E-01	6.780E-01
Right middle temporal gyrus	-0.238	0.172	[-0.575 - 0.098]	1.650E-01	4.745E-01
Left pars triangularis of inferior frontal gyrus	-0.254	0.143	[-0.533 - 0.026]	7.526E-02	3.504E-01
Right pars triangularis of inferior frontal gyrus	-0.196	0.154	[-0.498 - 0.106]	2.036E-01	4.745E-01
Left insula	-0.181	0.088	[-0.354 - -0.008]	3.987E-02	2.571E-01
Right insula	-0.234	0.138	[-0.504 - 0.037]	9.065E-02	3.703E-01
Left pars opercularis of inferior frontal gyrus	-0.143	0.088	[-0.315 - 0.030]	1.046E-01	3.707E-01
Right pars opercularis of inferior frontal gyrus	-0.106	0.102	[-0.306 - 0.095]	3.029E-01	5.344E-01
Left lateral occipital cortex	-0.212	0.168	[-0.542 - 0.117]	2.069E-01	4.745E-01
Right lateral occipital cortex	-0.315	0.129	[-0.569 - -0.061]	1.496E-02	1.696E-01
Left parahippocampal gyrus	-0.108	0.101	[-0.306 - 0.091]	2.868E-01	5.344E-01
Right parahippocampal gyrus	-0.166	0.107	[-0.376 - 0.044]	1.207E-01	3.735E-01
Left inferior parietal cortex	-0.093	0.128	[-0.345 - 0.158]	4.676E-01	6.688E-01
Right inferior parietal cortex	-0.086	0.154	[-0.387 - 0.215]	5.742E-01	7.099E-01
Left frontal pole	-0.222	0.088	[-0.394 - -0.049]	1.186E-02	1.696E-01
Right frontal pole	-0.371	0.114	[-0.595 - -0.147]	1.153E-03	2.613E-02
Left supramarginal gyrus	-0.111	0.112	[-0.330 - 0.108]	3.184E-01	5.344E-01
Right supramarginal gyrus	-0.131	0.117	[-0.361 - 0.100]	2.659E-01	5.319E-01
Left transverse temporal gyrus	-0.144	0.088	[-0.316 - 0.029]	1.028E-01	3.707E-01
Right transverse temporal gyrus	-0.137	0.088	[-0.309 - 0.036]	1.208E-01	3.735E-01
Left precuneus	-0.044	0.166	[-0.368 - 0.281]	7.921E-01	8.416E-01
Right precuneus	-0.154	0.128	[-0.405 - 0.096]	2.264E-01	4.811E-01
Left caudal middle frontal gyrus	-0.073	0.097	[-0.263 - 0.117]	4.488E-01	6.688E-01
Right caudal middle frontal gyrus	-0.014	0.091	[-0.194 - 0.165]	8.743E-01	8.874E-01
Left banks of superior temporal sulcus	0.021	0.092	[-0.160 - 0.201]	8.235E-01	8.485E-01
Right banks of superior temporal sulcus	-0.063	0.088	[-0.236 - 0.109]	4.721E-01	6.688E-01
Left posterior cingulate cortex	-0.164	0.088	[-0.337 - 0.008]	6.182E-02	3.234E-01
Right posterior cingulate cortex	-0.040	0.088	[-0.213 - 0.133]	6.499E-01	7.753E-01
Left postcentral gyrus	-0.032	0.131	[-0.289 - 0.225]	8.094E-01	8.468E-01
Right postcentral gyrus	-0.184	0.144	[-0.467 - 0.098]	2.005E-01	4.745E-01
Left cuneus	-0.053	0.150	[-0.348 - 0.242]	7.236E-01	8.336E-01
Right cuneus	-0.207	0.167	[-0.534 - 0.120]	2.143E-01	4.745E-01
Left caudal anterior cingulate cortex	0.036	0.114	[-0.186 - 0.259]	7.491E-01	8.336E-01
Right caudal anterior cingulate cortex	-0.031	0.094	[-0.215 - 0.154]	7.437E-01	8.336E-01
Left rostral anterior cingulate cortex	-0.070	0.112	[-0.290 - 0.149]	5.307E-01	6.809E-01
Right rostral anterior cingulate cortex	-0.129	0.131	[-0.385 - 0.127]	3.222E-01	5.344E-01
Left superior parietal cortex	-0.113	0.109	[-0.328 - 0.101]	2.986E-01	5.344E-01
Right superior parietal cortex	-0.039	0.123	[-0.279 - 0.202]	7.538E-01	8.336E-01
Left isthmus cingulate cortex	-0.141	0.088	[-0.314 - 0.032]	1.090E-01	3.707E-01
Right isthmus cingulate cortex	-0.084	0.134	[-0.348 - 0.179]	5.305E-01	6.809E-01
Left pericalcarine cortex	0.011	0.088	[-0.162 - 0.183]	9.042E-01	9.042E-01
Right pericalcarine cortex	0.027	0.088	[-0.146 - 0.199]	7.600E-01	8.336E-01
Left paracentral lobule	-0.107	0.093	[-0.289 - 0.074]	2.471E-01	5.091E-01
Right paracentral lobule	-0.057	0.088	[-0.229 - 0.115]	5.175E-01	6.809E-01
Left entorhinal cortex	0.076	0.088	[-0.096 - 0.249]	3.863E-01	5.970E-01
Right entorhinal cortex	0.103	0.110	[-0.112 - 0.318]	3.460E-01	5.601E-01
Left temporal pole	-0.131	0.147	[-0.420 - 0.158]	3.739E-01	5.913E-01
Right temporal pole	0.046	0.095	[-0.140 - 0.232]	6.259E-01	7.600E-01

Supplementary Table S40. Cortical volume differences between individuals with major depressive disorder and healthy comparison subjects controlling for age, sex, and ICV

	d	Std. Err.	95% CI	*p*-value	FDR *q*-value
Left superior frontal gyrus	-0.371	0.112	[-0.590 - -0.152]	8.965E-04	6.096E-03
Right superior frontal gyrus	-0.378	0.100	[-0.574 - -0.182]	1.542E-04	2.097E-03
Left lateral orbitofrontal cortex	-0.349	0.090	[-0.526 - -0.173]	1.049E-04	2.097E-03
Right lateral orbitofrontal cortex	-0.207	0.098	[-0.399 - -0.015]	3.427E-02	6.132E-02
Left pars orbitalis of inferior frontal gyrus	-0.309	0.088	[-0.482 - -0.136]	4.607E-04	5.221E-03
Right pars orbitalis of inferior frontal gyrus	-0.279	0.082	[-0.440 - -0.117]	7.103E-04	5.367E-03
Left rostral middle frontal gyrus	-0.289	0.085	[-0.456 - -0.123]	6.732E-04	5.367E-03
Right rostral middle frontal gyrus	-0.174	0.087	[-0.345 - -0.003]	4.643E-02	7.894E-02
Left superior temporal gyrus	-0.257	0.086	[-0.424 - -0.089]	2.701E-03	1.225E-02
Right superior temporal gyrus	-0.214	0.088	[-0.386 - -0.043]	1.447E-02	3.628E-02
Left lingual gyrus	-0.177	0.075	[-0.324 - -0.029]	1.872E-02	3.858E-02
Right lingual gyrus	-0.209	0.081	[-0.368 - -0.050]	1.002E-02	2.844E-02
Left fusiform gyrus	-0.193	0.095	[-0.380 - -0.006]	4.356E-02	7.594E-02
Right fusiform gyrus	-0.265	0.077	[-0.416 - -0.115]	5.451E-04	5.295E-03
Left medial orbitofrontal cortex	-0.219	0.120	[-0.453 - 0.016]	6.761E-02	9.995E-02
Right medial orbitofrontal cortex	-0.249	0.088	[-0.421 - -0.078]	4.455E-03	1.893E-02
Left inferior temporal gyrus	-0.183	0.067	[-0.315 - -0.051]	6.411E-03	2.295E-02
Right inferior temporal gyrus	-0.160	0.085	[-0.327 - 0.007]	6.040E-02	9.374E-02
Left precentral gyrus	-0.242	0.063	[-0.365 - -0.118]	1.257E-04	2.097E-03
Right precentral gyrus	-0.192	0.069	[-0.327 - -0.057]	5.193E-03	2.077E-02
Left middle temporal gyrus	-0.310	0.072	[-0.450 - -0.169]	1.493E-05	1.015E-03
Right middle temporal gyrus	-0.263	0.100	[-0.459 - -0.068]	8.309E-03	2.568E-02
Left pars triangularis of inferior frontal gyrus	-0.224	0.083	[-0.386 - -0.062]	6.751E-03	2.295E-02
Right pars triangularis of inferior frontal gyrus	-0.242	0.081	[-0.400 - -0.084]	2.684E-03	1.225E-02
Left insula	-0.178	0.094	[-0.362 - 0.006]	5.829E-02	9.374E-02
Right insula	-0.172	0.099	[-0.365 - 0.022]	8.240E-02	1.192E-01
Left pars opercularis of inferior frontal gyrus	-0.174	0.082	[-0.334 - -0.013]	3.373E-02	6.132E-02
Right pars opercularis of inferior frontal gyrus	-0.113	0.077	[-0.265 - 0.038]	1.431E-01	1.909E-01
Left lateral occipital cortex	-0.172	0.071	[-0.311 - -0.033]	1.505E-02	3.628E-02
Right lateral occipital cortex	-0.208	0.087	[-0.378 - -0.038]	1.654E-02	3.628E-02
Left parahippocampal gyrus	-0.115	0.070	[-0.252 - 0.022]	1.001E-01	1.418E-01
Right parahippocampal gyrus	-0.103	0.079	[-0.258 - 0.051]	1.902E-01	2.395E-01
Left inferior parietal cortex	-0.223	0.102	[-0.423 - -0.022]	2.930E-02	5.534E-02
Right inferior parietal cortex	-0.172	0.091	[-0.351 - 0.008]	6.065E-02	9.374E-02
Left frontal pole	-0.173	0.074	[-0.318 - -0.029]	1.837E-02	3.858E-02
Right frontal pole	-0.254	0.062	[-0.375 - -0.133]	3.629E-05	1.234E-03
Left supramarginal gyrus	-0.151	0.097	[-0.342 - 0.039]	1.186E-01	1.613E-01
Right supramarginal gyrus	-0.145	0.102	[-0.346 - 0.055]	1.557E-01	2.036E-01
Left transverse temporal gyrus	-0.073	0.076	[-0.222 - 0.076]	3.391E-01	3.719E-01
Right transverse temporal gyrus	-0.106	0.085	[-0.272 - 0.061]	2.144E-01	2.603E-01
Left precuneus	-0.220	0.080	[-0.378 - -0.063]	6.103E-03	2.295E-02
Right precuneus	-0.078	0.079	[-0.234 - 0.077]	3.228E-01	3.599E-01
Left caudal middle frontal gyrus	-0.087	0.087	[-0.257 - 0.083]	3.140E-01	3.559E-01
Right caudal middle frontal gyrus	-0.073	0.069	[-0.209 - 0.062]	2.887E-01	3.394E-01
Left banks of superior temporal sulcus	-0.135	0.061	[-0.255 - -0.014]	2.863E-02	5.534E-02
Right banks of superior temporal sulcus	-0.113	0.107	[-0.322 - 0.096]	2.895E-01	3.394E-01
Left posterior cingulate cortex	-0.148	0.061	[-0.268 - -0.027]	1.613E-02	3.628E-02
Right posterior cingulate cortex	-0.208	0.067	[-0.340 - -0.077]	1.888E-03	9.874E-03
Left postcentral gyrus	-0.184	0.094	[-0.367 - 0.000]	5.011E-02	8.311E-02
Right postcentral gyrus	-0.228	0.091	[-0.407 - -0.049]	1.248E-02	3.395E-02
Left cuneus	-0.204	0.062	[-0.326 - -0.082]	1.013E-03	6.264E-03
Right cuneus	-0.200	0.061	[-0.321 - -0.080]	1.119E-03	6.340E-03
Left caudal anterior cingulate cortex	-0.148	0.061	[-0.268 - -0.027]	1.626E-02	3.628E-02
Right caudal anterior cingulate cortex	-0.133	0.072	[-0.274 - 0.007]	6.255E-02	9.452E-02
Left rostral anterior cingulate cortex	-0.139	0.061	[-0.259 - -0.019]	2.368E-02	4.736E-02
Right rostral anterior cingulate cortex	-0.048	0.075	[-0.196 - 0.099]	5.190E-01	5.514E-01
Left superior parietal cortex	-0.099	0.061	[-0.219 - 0.021]	1.073E-01	1.489E-01
Right superior parietal cortex	-0.037	0.084	[-0.202 - 0.128]	6.606E-01	6.806E-01
Left isthmus cingulate cortex	-0.085	0.061	[-0.205 - 0.036]	1.676E-01	2.151E-01
Right isthmus cingulate cortex	-0.023	0.061	[-0.143 - 0.097]	7.063E-01	7.168E-01
Left pericalcarine cortex	-0.151	0.061	[-0.271 - -0.031]	1.402E-02	3.628E-02
Right pericalcarine cortex	-0.167	0.065	[-0.295 - -0.040]	1.004E-02	2.844E-02
Left paracentral lobule	-0.071	0.077	[-0.222 - 0.080]	3.543E-01	3.825E-01
Right paracentral lobule	-0.186	0.070	[-0.323 - -0.048]	8.209E-03	2.568E-02
Left entorhinal cortex	0.001	0.080	[-0.156 - 0.158]	9.911E-01	9.911E-01
Right entorhinal cortex	0.077	0.061	[-0.044 - 0.197]	2.110E-01	2.603E-01
Left temporal pole	-0.078	0.075	[-0.225 - 0.069]	2.970E-01	3.423E-01
Right temporal pole	0.034	0.061	[-0.086 - 0.155]	5.758E-01	6.023E-01

Supplementary Table S41. Cortical volume differences between individuals with major depressive disorder and healthy comparison subjects controlling for age, sex, and ICV over 21 years old

	d	Std. Err.	95% CI	*p*-value	FDR *q*-value
Left superior frontal gyrus	-0.310	0.102	[-0.510 - -0.110]	2.400E-03	1.632E-02
Right superior frontal gyrus	-0.341	0.095	[-0.526 - -0.155]	3.155E-04	4.572E-03
Left lateral orbitofrontal cortex	-0.306	0.077	[-0.457 - -0.155]	6.905E-05	1.565E-03
Right lateral orbitofrontal cortex	-0.160	0.082	[-0.320 - 0.000]	4.941E-02	9.708E-02
Left pars orbitalis of inferior frontal gyrus	-0.282	0.102	[-0.482 - -0.083]	5.581E-03	2.635E-02
Right pars orbitalis of inferior frontal gyrus	-0.298	0.084	[-0.462 - -0.133]	4.034E-04	4.572E-03
Left rostral middle frontal gyrus	-0.272	0.076	[-0.422 - -0.123]	3.577E-04	4.572E-03
Right rostral middle frontal gyrus	-0.146	0.082	[-0.307 - 0.015]	7.473E-02	1.303E-01
Left superior temporal gyrus	-0.233	0.075	[-0.380 - -0.085]	1.960E-03	1.481E-02
Right superior temporal gyrus	-0.191	0.089	[-0.365 - -0.017]	3.147E-02	7.379E-02
Left lingual gyrus	-0.140	0.082	[-0.302 - 0.021]	8.783E-02	1.389E-01
Right lingual gyrus	-0.174	0.078	[-0.326 - -0.022]	2.528E-02	6.824E-02
Left fusiform gyrus	-0.180	0.098	[-0.373 - 0.013]	6.697E-02	1.198E-01
Right fusiform gyrus	-0.231	0.068	[-0.364 - -0.099]	6.196E-04	5.267E-03
Left medial orbitofrontal cortex	-0.147	0.116	[-0.374 - 0.080]	2.046E-01	2.714E-01
Right medial orbitofrontal cortex	-0.213	0.087	[-0.383 - -0.043]	1.411E-02	4.820E-02
Left inferior temporal gyrus	-0.163	0.064	[-0.289 - -0.037]	1.106E-02	4.425E-02
Right inferior temporal gyrus	-0.141	0.080	[-0.297 - 0.015]	7.678E-02	1.305E-01
Left precentral gyrus	-0.216	0.063	[-0.340 - -0.093]	6.196E-04	5.267E-03
Right precentral gyrus	-0.171	0.063	[-0.295 - -0.048]	6.676E-03	2.837E-02
Left middle temporal gyrus	-0.302	0.063	[-0.426 - -0.177]	1.933E-06	1.314E-04
Right middle temporal gyrus	-0.284	0.099	[-0.479 - -0.089]	4.307E-03	2.253E-02
Left pars triangularis of inferior frontal gyrus	-0.191	0.077	[-0.342 - -0.039]	1.353E-02	4.820E-02
Right pars triangularis of inferior frontal gyrus	-0.182	0.093	[-0.365 - 0.000]	4.997E-02	9.708E-02
Left insula	-0.132	0.066	[-0.261 - -0.003]	4.498E-02	9.268E-02
Right insula	-0.134	0.099	[-0.328 - 0.060]	1.770E-01	2.456E-01
Left pars opercularis of inferior frontal gyrus	-0.139	0.089	[-0.313 - 0.034]	1.155E-01	1.745E-01
Right pars opercularis of inferior frontal gyrus	-0.107	0.085	[-0.274 - 0.061]	2.115E-01	2.714E-01
Left lateral occipital cortex	-0.142	0.069	[-0.279 - -0.006]	4.024E-02	8.551E-02
Right lateral occipital cortex	-0.183	0.089	[-0.358 - -0.009]	3.882E-02	8.515E-02
Left parahippocampal gyrus	-0.119	0.072	[-0.260 - 0.023]	1.009E-01	1.560E-01
Right parahippocampal gyrus	-0.104	0.081	[-0.263 - 0.055]	1.991E-01	2.708E-01
Left inferior parietal cortex	-0.185	0.107	[-0.394 - 0.024]	8.221E-02	1.346E-01
Right inferior parietal cortex	-0.166	0.096	[-0.355 - 0.022]	8.314E-02	1.346E-01
Left frontal pole	-0.176	0.078	[-0.330 - -0.023]	2.444E-02	6.824E-02
Right frontal pole	-0.273	0.063	[-0.397 - -0.149]	1.598E-05	5.435E-04
Left supramarginal gyrus	-0.127	0.093	[-0.310 - 0.056]	1.723E-01	2.456E-01
Right supramarginal gyrus	-0.116	0.092	[-0.296 - 0.064]	2.078E-01	2.714E-01
Left transverse temporal gyrus	-0.072	0.081	[-0.231 - 0.087]	3.746E-01	4.391E-01
Right transverse temporal gyrus	-0.084	0.088	[-0.257 - 0.089]	3.400E-01	4.096E-01
Left precuneus	-0.196	0.081	[-0.356 - -0.037]	1.602E-02	5.188E-02
Right precuneus	-0.045	0.082	[-0.206 - 0.116]	5.840E-01	6.304E-01
Left caudal middle frontal gyrus	-0.075	0.089	[-0.249 - 0.100]	4.028E-01	4.642E-01
Right caudal middle frontal gyrus	-0.054	0.075	[-0.201 - 0.093]	4.720E-01	5.262E-01
Left banks of superior temporal sulcus	-0.144	0.068	[-0.278 - -0.011]	3.443E-02	7.804E-02
Right banks of superior temporal sulcus	-0.105	0.111	[-0.323 - 0.112]	3.434E-01	4.096E-01
Left posterior cingulate cortex	-0.140	0.063	[-0.264 - -0.017]	2.609E-02	6.824E-02
Right posterior cingulate cortex	-0.212	0.072	[-0.354 - -0.070]	3.383E-03	2.091E-02
Left postcentral gyrus	-0.167	0.091	[-0.345 - 0.011]	6.515E-02	1.197E-01
Right postcentral gyrus	-0.213	0.097	[-0.403 - -0.024]	2.711E-02	6.828E-02
Left cuneus	-0.186	0.068	[-0.319 - -0.054]	5.813E-03	2.635E-02
Right cuneus	-0.183	0.063	[-0.307 - -0.059]	3.746E-03	2.123E-02
Left caudal anterior cingulate cortex	-0.155	0.063	[-0.279 - -0.031]	1.418E-02	4.820E-02
Right caudal anterior cingulate cortex	-0.114	0.078	[-0.266 - 0.039]	1.434E-01	2.120E-01
Left rostral anterior cingulate cortex	-0.151	0.063	[-0.275 - -0.027]	1.685E-02	5.208E-02
Right rostral anterior cingulate cortex	-0.034	0.084	[-0.199 - 0.130]	6.826E-01	7.253E-01
Left superior parietal cortex	-0.069	0.063	[-0.193 - 0.054]	2.716E-01	3.358E-01
Right superior parietal cortex	-0.010	0.086	[-0.178 - 0.157]	9.032E-01	9.166E-01
Left isthmus cingulate cortex	-0.077	0.063	[-0.201 - 0.047]	2.227E-01	2.804E-01
Right isthmus cingulate cortex	-0.004	0.064	[-0.130 - 0.122]	9.506E-01	9.506E-01
Left pericalcarine cortex	-0.127	0.068	[-0.259 - 0.006]	6.128E-02	1.157E-01
Right pericalcarine cortex	-0.151	0.069	[-0.286 - -0.016]	2.823E-02	6.856E-02
Left paracentral lobule	-0.040	0.071	[-0.178 - 0.099]	5.743E-01	6.298E-01
Right paracentral lobule	-0.149	0.063	[-0.272 - -0.025]	1.867E-02	5.521E-02
Left entorhinal cortex	0.011	0.070	[-0.127 - 0.149]	8.755E-01	9.020E-01
Right entorhinal cortex	0.085	0.063	[-0.038 - 0.209]	1.769E-01	2.456E-01
Left temporal pole	-0.067	0.083	[-0.230 - 0.095]	4.160E-01	4.715E-01
Right temporal pole	0.021	0.064	[-0.105 - 0.147]	7.397E-01	7.739E-01

Supplementary Table S42. Cortical volume differences between individuals with autism spectrum disorder and healthy comparison subjects controlling for age, sex, and ICV

	d	Std. Err.	95% CI	*p*-value	FDR *q*-value
Left superior frontal gyrus	-0.077	0.086	[-0.247 - 0.092]	3.700E-01	7.189E-01
Right superior frontal gyrus	-0.077	0.092	[-0.258 - 0.104]	4.025E-01	7.196E-01
Left lateral orbitofrontal cortex	-0.118	0.080	[-0.275 - 0.039]	1.407E-01	6.276E-01
Right lateral orbitofrontal cortex	-0.196	0.099	[-0.390 - -0.003]	4.710E-02	4.003E-01
Left pars orbitalis of inferior frontal gyrus	-0.150	0.084	[-0.315 - 0.015]	7.520E-02	4.261E-01
Right pars orbitalis of inferior frontal gyrus	-0.253	0.080	[-0.410 - -0.095]	1.629E-03	1.108E-01
Left rostral middle frontal gyrus	-0.066	0.082	[-0.227 - 0.094]	4.179E-01	7.196E-01
Right rostral middle frontal gyrus	-0.107	0.080	[-0.264 - 0.049]	1.793E-01	6.276E-01
Left superior temporal gyrus	-0.051	0.080	[-0.208 - 0.106]	5.213E-01	7.638E-01
Right superior temporal gyrus	-0.006	0.101	[-0.205 - 0.193]	9.519E-01	9.661E-01
Left lingual gyrus	-0.163	0.121	[-0.400 - 0.073]	1.762E-01	6.276E-01
Right lingual gyrus	-0.239	0.088	[-0.411 - -0.067]	6.334E-03	1.879E-01
Left fusiform gyrus	-0.186	0.086	[-0.354 - -0.018]	2.957E-02	3.351E-01
Right fusiform gyrus	-0.086	0.080	[-0.243 - 0.071]	2.839E-01	6.624E-01
Left medial orbitofrontal cortex	-0.167	0.080	[-0.324 - -0.010]	3.702E-02	3.596E-01
Right medial orbitofrontal cortex	-0.095	0.080	[-0.252 - 0.062]	2.351E-01	6.624E-01
Left inferior temporal gyrus	-0.057	0.124	[-0.301 - 0.186]	6.438E-01	8.419E-01
Right inferior temporal gyrus	-0.065	0.136	[-0.331 - 0.201]	6.319E-01	8.419E-01
Left precentral gyrus	-0.115	0.080	[-0.271 - 0.042]	1.521E-01	6.276E-01
Right precentral gyrus	-0.062	0.080	[-0.219 - 0.095]	4.361E-01	7.196E-01
Left middle temporal gyrus	0.006	0.110	[-0.209 - 0.221]	9.579E-01	9.661E-01
Right middle temporal gyrus	-0.116	0.110	[-0.332 - 0.099]	2.892E-01	6.624E-01
Left pars triangularis of inferior frontal gyrus	-0.016	0.080	[-0.173 - 0.141]	8.445E-01	9.571E-01
Right pars triangularis of inferior frontal gyrus	-0.017	0.111	[-0.236 - 0.201]	8.771E-01	9.661E-01
Left insula	-0.034	0.109	[-0.248 - 0.180]	7.576E-01	9.200E-01
Right insula	-0.126	0.080	[-0.283 - 0.031]	1.154E-01	6.035E-01
Left pars opercularis of inferior frontal gyrus	-0.060	0.080	[-0.217 - 0.096]	4.502E-01	7.196E-01
Right pars opercularis of inferior frontal gyrus	-0.107	0.095	[-0.292 - 0.079]	2.591E-01	6.624E-01
Left lateral occipital cortex	-0.063	0.080	[-0.220 - 0.093]	4.282E-01	7.196E-01
Right lateral occipital cortex	-0.085	0.094	[-0.269 - 0.098]	3.624E-01	7.189E-01
Left parahippocampal gyrus	-0.106	0.080	[-0.263 - 0.051]	1.846E-01	6.276E-01
Right parahippocampal gyrus	0.065	0.097	[-0.125 - 0.255]	5.026E-01	7.595E-01
Left inferior parietal cortex	-0.186	0.084	[-0.351 - -0.020]	2.785E-02	3.351E-01
Right inferior parietal cortex	-0.170	0.090	[-0.346 - 0.007]	5.985E-02	4.087E-01
Left frontal pole	-0.051	0.081	[-0.210 - 0.107]	5.279E-01	7.638E-01
Right frontal pole	0.005	0.080	[-0.152 - 0.162]	9.514E-01	9.661E-01
Left supramarginal gyrus	-0.120	0.101	[-0.318 - 0.078]	2.344E-01	6.624E-01
Right supramarginal gyrus	-0.075	0.080	[-0.232 - 0.082]	3.496E-01	7.189E-01
Left transverse temporal gyrus	0.040	0.080	[-0.117 - 0.197]	6.183E-01	8.419E-01
Right transverse temporal gyrus	-0.122	0.083	[-0.286 - 0.041]	1.434E-01	6.276E-01
Left precuneus	0.033	0.100	[-0.163 - 0.230]	7.392E-01	9.139E-01
Right precuneus	-0.071	0.083	[-0.233 - 0.091]	3.923E-01	7.196E-01
Left caudal middle frontal gyrus	-0.193	0.080	[-0.350 - -0.036]	1.584E-02	2.693E-01
Right caudal middle frontal gyrus	0.020	0.080	[-0.137 - 0.177]	7.999E-01	9.438E-01
Left banks of superior temporal sulcus	0.070	0.093	[-0.113 - 0.252]	4.551E-01	7.196E-01
Right banks of superior temporal sulcus	-0.082	0.091	[-0.260 - 0.096]	3.646E-01	7.189E-01
Left posterior cingulate cortex	-0.018	0.080	[-0.175 - 0.139]	8.181E-01	9.438E-01
Right posterior cingulate cortex	-0.068	0.137	[-0.337 - 0.200]	6.173E-01	8.419E-01
Left postcentral gyrus	-0.108	0.103	[-0.309 - 0.093]	2.922E-01	6.624E-01
Right postcentral gyrus	-0.103	0.107	[-0.312 - 0.107]	3.373E-01	7.189E-01
Left cuneus	-0.050	0.118	[-0.282 - 0.182]	6.725E-01	8.628E-01
Right cuneus	-0.116	0.089	[-0.291 - 0.059]	1.941E-01	6.285E-01
Left caudal anterior cingulate cortex	-0.040	0.080	[-0.197 - 0.117]	6.196E-01	8.419E-01
Right caudal anterior cingulate cortex	-0.085	0.126	[-0.331 - 0.162]	5.018E-01	7.595E-01
Left rostral anterior cingulate cortex	-0.093	0.080	[-0.250 - 0.064]	2.471E-01	6.624E-01
Right rostral anterior cingulate cortex	-0.010	0.098	[-0.203 - 0.183]	9.221E-01	9.661E-01
Left superior parietal cortex	-0.212	0.080	[-0.369 - -0.055]	8.289E-03	1.879E-01
Right superior parietal cortex	-0.147	0.080	[-0.304 - 0.010]	6.611E-02	4.087E-01
Left isthmus cingulate cortex	-0.151	0.080	[-0.307 - 0.006]	6.020E-02	4.087E-01
Right isthmus cingulate cortex	-0.039	0.108	[-0.252 - 0.174]	7.198E-01	9.065E-01
Left pericalcarine cortex	-0.091	0.107	[-0.300 - 0.118]	3.927E-01	7.196E-01
Right pericalcarine cortex	-0.127	0.113	[-0.348 - 0.094]	2.588E-01	6.624E-01
Left paracentral lobule	-0.018	0.080	[-0.175 - 0.138]	8.189E-01	9.438E-01
Right paracentral lobule	-0.087	0.080	[-0.244 - 0.069]	2.750E-01	6.624E-01
Left entorhinal cortex	-0.173	0.131	[-0.429 - 0.083]	1.846E-01	6.276E-01
Right entorhinal cortex	-0.006	0.101	[-0.204 - 0.193]	9.549E-01	9.661E-01
Left temporal pole	-0.005	0.100	[-0.202 - 0.191]	9.564E-01	9.661E-01
Right temporal pole	0.005	0.109	[-0.209 - 0.218]	9.661E-01	9.661E-01

Supplementary Table S43. Sex-by-Diagnosis interaction (SZ/HC) on cortical thickness differences controlling for age and sex

	d	Std. Err.	95% CI	*p*-value	FDR *q*-value
Global mean cortical thickness	0.046	0.047	[-0.047 - 0.138]	3.303E-01	8.422E-01
Left hemisphere	0.051	0.046	[-0.039 - 0.142]	2.651E-01	8.422E-01
Right hemisphere	0.038	0.047	[-0.054 - 0.129]	4.184E-01	8.422E-01
Left fusiform gyrus	0.000	0.048	[-0.093 - 0.094]	9.927E-01	9.927E-01
Right fusiform gyrus	-0.005	0.041	[-0.086 - 0.077]	9.108E-01	9.748E-01
Left pars opercularis of inferior frontal gyrus	-0.014	0.039	[-0.090 - 0.062]	7.198E-01	8.617E-01
Right pars opercularis of inferior frontal gyrus	0.002	0.050	[-0.096 - 0.101]	9.611E-01	9.748E-01
Left superior temporal gyrus	0.029	0.035	[-0.039 - 0.097]	3.982E-01	8.422E-01
Right superior temporal gyrus	0.021	0.037	[-0.052 - 0.095]	5.649E-01	8.422E-01
Left insula	0.015	0.046	[-0.074 - 0.105]	7.403E-01	8.617E-01
Right insula	0.050	0.048	[-0.045 - 0.144]	3.064E-01	8.422E-01
Left lingual gyrus	0.058	0.035	[-0.010 - 0.126]	9.265E-02	8.359E-01
Right lingual gyrus	0.017	0.035	[-0.052 - 0.085]	6.287E-01	8.422E-01
Left pars triangularis of inferior frontal gyrus	0.029	0.042	[-0.053 - 0.110]	4.921E-01	8.422E-01
Right pars triangularis of inferior frontal gyrus	0.026	0.039	[-0.050 - 0.102]	5.039E-01	8.422E-01
Left lateral orbitofrontal cortex	-0.025	0.053	[-0.129 - 0.079]	6.407E-01	8.424E-01
Right lateral orbitofrontal cortex	-0.035	0.049	[-0.131 - 0.060]	4.708E-01	8.422E-01
Left rostral middle frontal gyrus	0.021	0.039	[-0.055 - 0.098]	5.862E-01	8.422E-01
Right rostral middle frontal gyrus	0.041	0.044	[-0.045 - 0.127]	3.533E-01	8.422E-01
Left middle temporal gyrus	0.048	0.041	[-0.033 - 0.129]	2.471E-01	8.422E-01
Right middle temporal gyrus	0.075	0.044	[-0.011 - 0.160]	8.831E-02	8.359E-01
Left superior frontal gyrus	0.021	0.047	[-0.072 - 0.114]	6.561E-01	8.470E-01
Right superior frontal gyrus	0.044	0.047	[-0.048 - 0.136]	3.522E-01	8.422E-01
Left pars orbitalis of inferior frontal gyrus	0.014	0.049	[-0.081 - 0.109]	7.750E-01	8.734E-01
Right pars orbitalis of inferior frontal gyrus	0.032	0.045	[-0.056 - 0.120]	4.783E-01	8.422E-01
Left medial orbitofrontal cortex	-0.027	0.044	[-0.113 - 0.059]	5.357E-01	8.422E-01
Right medial orbitofrontal cortex	-0.017	0.035	[-0.085 - 0.051]	6.194E-01	8.422E-01
Left inferior temporal gyrus	0.032	0.040	[-0.046 - 0.109]	4.240E-01	8.422E-01
Right inferior temporal gyrus	0.044	0.042	[-0.039 - 0.126]	3.004E-01	8.422E-01
Left isthmus cingulate cortex	0.083	0.051	[-0.018 - 0.183]	1.060E-01	8.359E-01
Right isthmus cingulate cortex	0.074	0.044	[-0.013 - 0.161]	9.769E-02	8.359E-01
Left banks of superior temporal sulcus	0.018	0.035	[-0.050 - 0.086]	6.118E-01	8.422E-01
Right banks of superior temporal sulcus	0.064	0.037	[-0.009 - 0.137]	8.463E-02	8.359E-01
Left supramarginal gyrus	0.023	0.036	[-0.046 - 0.093]	5.134E-01	8.422E-01
Right supramarginal gyrus	-0.002	0.041	[-0.084 - 0.079]	9.577E-01	9.748E-01
Left caudal middle frontal gyrus	0.029	0.051	[-0.071 - 0.130]	5.688E-01	8.422E-01
Right caudal middle frontal gyrus	0.010	0.054	[-0.095 - 0.115]	8.570E-01	9.361E-01
Left frontal pole	0.026	0.039	[-0.051 - 0.103]	5.054E-01	8.422E-01
Right frontal pole	0.007	0.035	[-0.061 - 0.075]	8.376E-01	9.292E-01
Left posterior cingulate cortex	0.022	0.037	[-0.050 - 0.094]	5.436E-01	8.422E-01
Right posterior cingulate cortex	0.026	0.035	[-0.042 - 0.094]	4.490E-01	8.422E-01
Left lateral occipital cortex	0.036	0.043	[-0.048 - 0.120]	3.985E-01	8.422E-01
Right lateral occipital cortex	0.026	0.047	[-0.067 - 0.119]	5.781E-01	8.422E-01
Left precentral gyrus	0.026	0.051	[-0.074 - 0.127]	6.063E-01	8.422E-01
Right precentral gyrus	-0.004	0.055	[-0.112 - 0.103]	9.368E-01	9.748E-01
Left parahippocampal gyrus	-0.027	0.035	[-0.095 - 0.041]	4.292E-01	8.422E-01
Right parahippocampal gyrus	-0.075	0.035	[-0.143 - -0.007]	3.158E-02	8.359E-01
Left inferior parietal cortex	0.135	0.048	[0.040 - 0.229]	5.307E-03	3.768E-01
Right inferior parietal cortex	0.062	0.045	[-0.027 - 0.150]	1.703E-01	8.422E-01
Left transverse temporal gyrus	0.063	0.053	[-0.040 - 0.166]	2.290E-01	8.422E-01
Right transverse temporal gyrus	0.032	0.035	[-0.037 - 0.101]	3.675E-01	8.422E-01
Left postcentral gyrus	0.074	0.039	[-0.002 - 0.150]	5.477E-02	8.359E-01
Right postcentral gyrus	0.043	0.045	[-0.044 - 0.130]	3.361E-01	8.422E-01
Left precuneus	0.054	0.042	[-0.029 - 0.137]	2.017E-01	8.422E-01
Right precuneus	0.040	0.038	[-0.034 - 0.114]	2.870E-01	8.422E-01
Left caudal anterior cingulate cortex	0.010	0.035	[-0.058 - 0.078]	7.660E-01	8.734E-01
Right caudal anterior cingulate cortex	-0.051	0.039	[-0.127 - 0.025]	1.896E-01	8.422E-01
Left cuneus	0.070	0.035	[0.002 - 0.138]	4.358E-02	8.359E-01
Right cuneus	0.056	0.036	[-0.016 - 0.127]	1.260E-01	8.422E-01
Left rostral anterior cingulate cortex	-0.012	0.035	[-0.080 - 0.056]	7.303E-01	8.617E-01
Right rostral anterior cingulate cortex	0.012	0.035	[-0.055 - 0.080]	7.187E-01	8.617E-01
Left pericalcarine cortex	0.022	0.042	[-0.061 - 0.105]	5.994E-01	8.422E-01
Right pericalcarine cortex	0.026	0.035	[-0.042 - 0.094]	4.506E-01	8.422E-01
Left paracentral lobule	0.030	0.040	[-0.050 - 0.109]	4.647E-01	8.422E-01
Right paracentral lobule	0.049	0.049	[-0.047 - 0.145]	3.149E-01	8.422E-01
Left superior parietal cortex	0.052	0.041	[-0.029 - 0.133]	2.084E-01	8.422E-01
Right superior parietal cortex	0.038	0.052	[-0.064 - 0.140]	4.649E-01	8.422E-01
Left temporal pole	-0.018	0.044	[-0.103 - 0.068]	6.883E-01	8.617E-01
Right temporal pole	-0.015	0.043	[-0.099 - 0.070]	7.372E-01	8.617E-01
Left entorhinal cortex	0.030	0.035	[-0.038 - 0.098]	3.862E-01	8.422E-01
Right entorhinal cortex	0.002	0.035	[-0.066 - 0.070]	9.529E-01	9.748E-01

Supplementary Table S44. Age-by-Diagnosis interaction (SZ/HC) on cortical thickness differences controlling for age and sex

	d	Std. Err.	95% CI	*p*-value	FDR *q*-value
Global mean cortical thickness	-0.009	0.063	[-0.133 - 0.114]	8.823E-01	9.575E-01
Left hemisphere	-0.012	0.062	[-0.134 - 0.110]	8.416E-01	9.575E-01
Right hemisphere	-0.005	0.062	[-0.126 - 0.117]	9.416E-01	9.689E-01
Left fusiform gyrus	-0.046	0.046	[-0.136 - 0.045]	3.255E-01	8.559E-01
Right fusiform gyrus	-0.057	0.054	[-0.163 - 0.049]	2.927E-01	8.559E-01
Left pars opercularis of inferior frontal gyrus	0.056	0.050	[-0.042 - 0.153]	2.613E-01	8.559E-01
Right pars opercularis of inferior frontal gyrus	0.059	0.050	[-0.039 - 0.157]	2.406E-01	8.559E-01
Left superior temporal gyrus	-0.010	0.055	[-0.119 - 0.099]	8.553E-01	9.575E-01
Right superior temporal gyrus	-0.030	0.059	[-0.146 - 0.087]	6.176E-01	9.575E-01
Left insula	-0.060	0.055	[-0.168 - 0.049]	2.798E-01	8.559E-01
Right insula	-0.046	0.052	[-0.149 - 0.056]	3.759E-01	8.712E-01
Left lingual gyrus	0.009	0.044	[-0.077 - 0.095]	8.341E-01	9.575E-01
Right lingual gyrus	-0.014	0.045	[-0.102 - 0.074]	7.553E-01	9.575E-01
Left pars triangularis of inferior frontal gyrus	-0.015	0.046	[-0.106 - 0.076]	7.455E-01	9.575E-01
Right pars triangularis of inferior frontal gyrus	0.068	0.050	[-0.030 - 0.167]	1.738E-01	8.559E-01
Left lateral orbitofrontal cortex	-0.022	0.051	[-0.121 - 0.077]	6.574E-01	9.575E-01
Right lateral orbitofrontal cortex	0.021	0.041	[-0.060 - 0.101]	6.158E-01	9.575E-01
Left rostral middle frontal gyrus	0.006	0.047	[-0.087 - 0.098]	9.054E-01	9.575E-01
Right rostral middle frontal gyrus	0.031	0.049	[-0.065 - 0.127]	5.229E-01	9.575E-01
Left middle temporal gyrus	-0.020	0.054	[-0.126 - 0.085]	7.024E-01	9.575E-01
Right middle temporal gyrus	-0.040	0.050	[-0.138 - 0.058]	4.267E-01	8.911E-01
Left superior frontal gyrus	0.051	0.046	[-0.039 - 0.141]	2.664E-01	8.559E-01
Right superior frontal gyrus	0.065	0.046	[-0.026 - 0.155]	1.603E-01	8.559E-01
Left pars orbitalis of inferior frontal gyrus	-0.039	0.037	[-0.111 - 0.033]	2.858E-01	8.559E-01
Right pars orbitalis of inferior frontal gyrus	-0.022	0.044	[-0.108 - 0.064]	6.130E-01	9.575E-01
Left medial orbitofrontal cortex	-0.026	0.051	[-0.125 - 0.074]	6.155E-01	9.575E-01
Right medial orbitofrontal cortex	-0.041	0.046	[-0.130 - 0.048]	3.673E-01	8.712E-01
Left inferior temporal gyrus	-0.093	0.049	[-0.188 - 0.002]	5.611E-02	8.559E-01
Right inferior temporal gyrus	-0.086	0.050	[-0.184 - 0.012]	8.515E-02	8.559E-01
Left isthmus cingulate cortex	-0.019	0.050	[-0.118 - 0.080]	7.067E-01	9.575E-01
Right isthmus cingulate cortex	-0.047	0.057	[-0.159 - 0.064]	4.049E-01	8.712E-01
Left banks of superior temporal sulcus	0.029	0.035	[-0.039 - 0.097]	4.037E-01	8.712E-01
Right banks of superior temporal sulcus	-0.018	0.045	[-0.107 - 0.071]	6.903E-01	9.575E-01
Left supramarginal gyrus	-0.033	0.054	[-0.139 - 0.073]	5.414E-01	9.575E-01
Right supramarginal gyrus	-0.006	0.059	[-0.122 - 0.109]	9.136E-01	9.575E-01
Left caudal middle frontal gyrus	0.055	0.054	[-0.050 - 0.160]	3.031E-01	8.559E-01
Right caudal middle frontal gyrus	0.051	0.050	[-0.046 - 0.149]	3.035E-01	8.559E-01
Left frontal pole	-0.066	0.035	[-0.134 - 0.002]	5.612E-02	8.559E-01
Right frontal pole	-0.098	0.057	[-0.210 - 0.014]	8.704E-02	8.559E-01
Left posterior cingulate cortex	-0.011	0.056	[-0.121 - 0.099]	8.451E-01	9.575E-01
Right posterior cingulate cortex	-0.047	0.040	[-0.125 - 0.030]	2.302E-01	8.559E-01
Left lateral occipital cortex	-0.008	0.059	[-0.124 - 0.108]	8.951E-01	9.575E-01
Right lateral occipital cortex	-0.018	0.057	[-0.130 - 0.095]	7.588E-01	9.575E-01
Left precentral gyrus	0.028	0.053	[-0.076 - 0.133]	5.962E-01	9.575E-01
Right precentral gyrus	0.048	0.048	[-0.046 - 0.143]	3.177E-01	8.559E-01
Left parahippocampal gyrus	0.007	0.050	[-0.092 - 0.106]	8.916E-01	9.575E-01
Right parahippocampal gyrus	0.014	0.043	[-0.071 - 0.099]	7.483E-01	9.575E-01
Left inferior parietal cortex	0.019	0.053	[-0.086 - 0.123]	7.266E-01	9.575E-01
Right inferior parietal cortex	0.026	0.055	[-0.081 - 0.133]	6.388E-01	9.575E-01
Left transverse temporal gyrus	-0.027	0.052	[-0.128 - 0.074]	6.012E-01	9.575E-01
Right transverse temporal gyrus	-0.008	0.044	[-0.094 - 0.077]	8.459E-01	9.575E-01
Left postcentral gyrus	-0.034	0.049	[-0.129 - 0.061]	4.817E-01	9.575E-01
Right postcentral gyrus	0.000	0.049	[-0.096 - 0.097]	9.939E-01	9.939E-01
Left precuneus	0.019	0.057	[-0.092 - 0.131]	7.347E-01	9.575E-01
Right precuneus	0.046	0.054	[-0.059 - 0.151]	3.922E-01	8.712E-01
Left caudal anterior cingulate cortex	-0.013	0.047	[-0.105 - 0.078]	7.763E-01	9.575E-01
Right caudal anterior cingulate cortex	0.022	0.035	[-0.046 - 0.090]	5.307E-01	9.575E-01
Left cuneus	0.020	0.044	[-0.066 - 0.106]	6.481E-01	9.575E-01
Right cuneus	0.001	0.056	[-0.109 - 0.112]	9.796E-01	9.936E-01
Left rostral anterior cingulate cortex	-0.058	0.041	[-0.139 - 0.022]	1.552E-01	8.559E-01
Right rostral anterior cingulate cortex	-0.089	0.048	[-0.184 - 0.005]	6.282E-02	8.559E-01
Left pericalcarine cortex	0.077	0.040	[-0.002 - 0.155]	5.688E-02	8.559E-01
Right pericalcarine cortex	0.053	0.040	[-0.025 - 0.131]	1.839E-01	8.559E-01
Left paracentral lobule	0.050	0.049	[-0.047 - 0.147]	3.103E-01	8.559E-01
Right paracentral lobule	0.071	0.043	[-0.013 - 0.154]	9.886E-02	8.559E-01
Left superior parietal cortex	0.062	0.055	[-0.045 - 0.169]	2.540E-01	8.559E-01
Right superior parietal cortex	0.055	0.051	[-0.044 - 0.154]	2.780E-01	8.559E-01
Left temporal pole	-0.074	0.041	[-0.154 - 0.007]	7.309E-02	8.559E-01
Right temporal pole	-0.073	0.046	[-0.164 - 0.017]	1.097E-01	8.559E-01
Left entorhinal cortex	-0.006	0.053	[-0.109 - 0.098]	9.171E-01	9.575E-01
Right entorhinal cortex	-0.034	0.039	[-0.111 - 0.042]	3.795E-01	8.712E-01

Supplementary Table S45. Sex-by-Diagnosis interaction (BD/HC) on cortical thickness differences controlling for age and sex

	d	Std. Err.	95% CI	*p*-value	FDR *q*-value
Global mean cortical thickness	0.041	0.093	[-0.141 - 0.223]	6.586E-01	9.624E-01
Left hemisphere	0.052	0.100	[-0.144 - 0.249]	6.009E-01	9.624E-01
Right hemisphere	0.028	0.087	[-0.143 - 0.199]	7.479E-01	9.624E-01
Left fusiform gyrus	-0.014	0.087	[-0.185 - 0.157]	8.739E-01	9.624E-01
Right fusiform gyrus	0.026	0.093	[-0.157 - 0.209]	7.792E-01	9.624E-01
Left pars opercularis of inferior frontal gyrus	0.015	0.110	[-0.200 - 0.230]	8.912E-01	9.624E-01
Right pars opercularis of inferior frontal gyrus	-0.030	0.087	[-0.201 - 0.142]	7.336E-01	9.624E-01
Left superior temporal gyrus	-0.110	0.087	[-0.281 - 0.061]	2.080E-01	9.624E-01
Right superior temporal gyrus	0.002	0.087	[-0.170 - 0.173]	9.841E-01	9.841E-01
Left insula	-0.040	0.114	[-0.263 - 0.182]	7.242E-01	9.624E-01
Right insula	-0.017	0.087	[-0.188 - 0.154]	8.433E-01	9.624E-01
Left lingual gyrus	0.012	0.087	[-0.159 - 0.184]	8.867E-01	9.624E-01
Right lingual gyrus	-0.029	0.094	[-0.215 - 0.156]	7.548E-01	9.624E-01
Left pars triangularis of inferior frontal gyrus	-0.060	0.087	[-0.232 - 0.111]	4.901E-01	9.624E-01
Right pars triangularis of inferior frontal gyrus	0.009	0.090	[-0.168 - 0.185]	9.223E-01	9.624E-01
Left lateral orbitofrontal cortex	-0.052	0.090	[-0.228 - 0.124]	5.640E-01	9.624E-01
Right lateral orbitofrontal cortex	0.021	0.087	[-0.150 - 0.192]	8.093E-01	9.624E-01
Left rostral middle frontal gyrus	0.040	0.099	[-0.154 - 0.233]	6.881E-01	9.624E-01
Right rostral middle frontal gyrus	0.080	0.098	[-0.112 - 0.272]	4.142E-01	9.624E-01
Left middle temporal gyrus	0.042	0.099	[-0.152 - 0.236]	6.709E-01	9.624E-01
Right middle temporal gyrus	0.013	0.087	[-0.159 - 0.184]	8.857E-01	9.624E-01
Left superior frontal gyrus	0.054	0.142	[-0.223 - 0.332]	7.013E-01	9.624E-01
Right superior frontal gyrus	0.028	0.109	[-0.187 - 0.242]	7.996E-01	9.624E-01
Left pars orbitalis of inferior frontal gyrus	0.038	0.125	[-0.207 - 0.283]	7.617E-01	9.624E-01
Right pars orbitalis of inferior frontal gyrus	-0.037	0.087	[-0.208 - 0.134]	6.720E-01	9.624E-01
Left medial orbitofrontal cortex	0.023	0.091	[-0.156 - 0.201]	8.031E-01	9.624E-01
Right medial orbitofrontal cortex	-0.008	0.087	[-0.179 - 0.163]	9.251E-01	9.624E-01
Left inferior temporal gyrus	0.075	0.098	[-0.118 - 0.267]	4.476E-01	9.624E-01
Right inferior temporal gyrus	0.044	0.087	[-0.127 - 0.215]	6.127E-01	9.624E-01
Left isthmus cingulate cortex	0.071	0.087	[-0.100 - 0.243]	4.139E-01	9.624E-01
Right isthmus cingulate cortex	0.005	0.087	[-0.166 - 0.176]	9.566E-01	9.703E-01
Left banks of superior temporal sulcus	-0.129	0.103	[-0.330 - 0.072]	2.084E-01	9.624E-01
Right banks of superior temporal sulcus	0.056	0.087	[-0.115 - 0.228]	5.189E-01	9.624E-01
Left supramarginal gyrus	0.102	0.092	[-0.078 - 0.281]	2.671E-01	9.624E-01
Right supramarginal gyrus	-0.036	0.101	[-0.235 - 0.162]	7.188E-01	9.624E-01
Left caudal middle frontal gyrus	0.197	0.088	[0.026 - 0.369]	2.433E-02	9.624E-01
Right caudal middle frontal gyrus	0.065	0.090	[-0.112 - 0.241]	4.737E-01	9.624E-01
Left frontal pole	0.036	0.087	[-0.136 - 0.207]	6.837E-01	9.624E-01
Right frontal pole	0.101	0.146	[-0.186 - 0.388]	4.911E-01	9.624E-01
Left posterior cingulate cortex	0.095	0.130	[-0.159 - 0.349]	4.628E-01	9.624E-01
Right posterior cingulate cortex	-0.073	0.087	[-0.244 - 0.099]	4.061E-01	9.624E-01
Left lateral occipital cortex	-0.037	0.087	[-0.208 - 0.134]	6.727E-01	9.624E-01
Right lateral occipital cortex	-0.035	0.087	[-0.207 - 0.136]	6.856E-01	9.624E-01
Left precentral gyrus	0.091	0.087	[-0.080 - 0.263]	2.972E-01	9.624E-01
Right precentral gyrus	0.027	0.096	[-0.162 - 0.216]	7.768E-01	9.624E-01
Left parahippocampal gyrus	-0.175	0.088	[-0.346 - -0.003]	4.576E-02	9.624E-01
Right parahippocampal gyrus	-0.080	0.087	[-0.251 - 0.092]	3.626E-01	9.624E-01
Left inferior parietal cortex	0.071	0.087	[-0.100 - 0.243]	4.144E-01	9.624E-01
Right inferior parietal cortex	0.111	0.087	[-0.060 - 0.283]	2.025E-01	9.624E-01
Left transverse temporal gyrus	-0.007	0.087	[-0.178 - 0.164]	9.353E-01	9.624E-01
Right transverse temporal gyrus	0.008	0.087	[-0.163 - 0.179]	9.275E-01	9.624E-01
Left postcentral gyrus	0.086	0.087	[-0.085 - 0.258]	3.229E-01	9.624E-01
Right postcentral gyrus	-0.025	0.111	[-0.243 - 0.193]	8.210E-01	9.624E-01
Left precuneus	0.095	0.087	[-0.076 - 0.266]	2.766E-01	9.624E-01
Right precuneus	0.084	0.087	[-0.088 - 0.255]	3.380E-01	9.624E-01
Left caudal anterior cingulate cortex	-0.125	0.122	[-0.364 - 0.114]	3.060E-01	9.624E-01
Right caudal anterior cingulate cortex	-0.026	0.090	[-0.202 - 0.150]	7.723E-01	9.624E-01
Left cuneus	0.115	0.099	[-0.080 - 0.310]	2.466E-01	9.624E-01
Right cuneus	0.048	0.136	[-0.220 - 0.315]	7.258E-01	9.624E-01
Left rostral anterior cingulate cortex	0.029	0.106	[-0.178 - 0.237]	7.834E-01	9.624E-01
Right rostral anterior cingulate cortex	-0.038	0.106	[-0.246 - 0.170]	7.212E-01	9.624E-01
Left pericalcarine cortex	0.081	0.104	[-0.122 - 0.284]	4.361E-01	9.624E-01
Right pericalcarine cortex	-0.018	0.087	[-0.189 - 0.153]	8.387E-01	9.624E-01
Left paracentral lobule	0.081	0.087	[-0.090 - 0.253]	3.514E-01	9.624E-01
Right paracentral lobule	0.114	0.116	[-0.115 - 0.342]	3.287E-01	9.624E-01
Left superior parietal cortex	0.066	0.093	[-0.116 - 0.248]	4.784E-01	9.624E-01
Right superior parietal cortex	0.057	0.087	[-0.115 - 0.228]	5.171E-01	9.624E-01
Left temporal pole	-0.096	0.087	[-0.267 - 0.076]	2.743E-01	9.624E-01
Right temporal pole	-0.056	0.090	[-0.233 - 0.120]	5.309E-01	9.624E-01
Left entorhinal cortex	0.044	0.087	[-0.127 - 0.216]	6.128E-01	9.624E-01
Right entorhinal cortex	0.110	0.087	[-0.062 - 0.281]	2.091E-01	9.624E-01

Supplementary Table S46. Sex-by-Diagnosis interaction (BD/HC) on cortical thickness differences controlling for age and sex at 25 years of age or older

	d	Std. Err.	95% CI	*p*-value	FDR *q*-value
Global mean cortical thickness	0.019	0.090	[-0.158 - 0.197]	8.300E-01	9.681E-01
Left hemisphere	0.029	0.091	[-0.148 - 0.207]	7.460E-01	9.681E-01
Right hemisphere	0.008	0.090	[-0.170 - 0.185]	9.330E-01	9.681E-01
Left fusiform gyrus	-0.030	0.090	[-0.207 - 0.147]	7.394E-01	9.681E-01
Right fusiform gyrus	-0.005	0.091	[-0.183 - 0.172]	9.550E-01	9.681E-01
Left pars opercularis of inferior frontal gyrus	-0.012	0.100	[-0.209 - 0.185]	9.026E-01	9.681E-01
Right pars opercularis of inferior frontal gyrus	-0.063	0.090	[-0.240 - 0.114]	4.866E-01	9.681E-01
Left superior temporal gyrus	-0.150	0.091	[-0.328 - 0.028]	9.775E-02	9.681E-01
Right superior temporal gyrus	-0.014	0.095	[-0.201 - 0.172]	8.795E-01	9.681E-01
Left insula	-0.007	0.130	[-0.262 - 0.248]	9.564E-01	9.681E-01
Right insula	-0.022	0.090	[-0.200 - 0.155]	8.046E-01	9.681E-01
Left lingual gyrus	-0.016	0.091	[-0.194 - 0.161]	8.564E-01	9.681E-01
Right lingual gyrus	-0.058	0.091	[-0.235 - 0.120]	5.232E-01	9.681E-01
Left pars triangularis of inferior frontal gyrus	-0.047	0.091	[-0.225 - 0.130]	6.005E-01	9.681E-01
Right pars triangularis of inferior frontal gyrus	-0.033	0.102	[-0.232 - 0.166]	7.461E-01	9.681E-01
Left lateral orbitofrontal cortex	-0.053	0.100	[-0.248 - 0.142]	5.952E-01	9.681E-01
Right lateral orbitofrontal cortex	0.007	0.090	[-0.171 - 0.184]	9.415E-01	9.681E-01
Left rostral middle frontal gyrus	0.040	0.101	[-0.158 - 0.238]	6.915E-01	9.681E-01
Right rostral middle frontal gyrus	0.098	0.091	[-0.082 - 0.277]	2.858E-01	9.681E-01
Left middle temporal gyrus	0.016	0.110	[-0.199 - 0.231]	8.832E-01	9.681E-01
Right middle temporal gyrus	-0.012	0.090	[-0.189 - 0.166]	8.965E-01	9.681E-01
Left superior frontal gyrus	0.056	0.143	[-0.224 - 0.336]	6.932E-01	9.681E-01
Right superior frontal gyrus	-0.004	0.096	[-0.193 - 0.185]	9.681E-01	9.681E-01
Left pars orbitalis of inferior frontal gyrus	0.049	0.121	[-0.188 - 0.286]	6.843E-01	9.681E-01
Right pars orbitalis of inferior frontal gyrus	-0.032	0.091	[-0.209 - 0.146]	7.271E-01	9.681E-01
Left medial orbitofrontal cortex	0.007	0.091	[-0.170 - 0.185]	9.357E-01	9.681E-01
Right medial orbitofrontal cortex	-0.025	0.090	[-0.202 - 0.153]	7.858E-01	9.681E-01
Left inferior temporal gyrus	0.041	0.096	[-0.146 - 0.229]	6.646E-01	9.681E-01
Right inferior temporal gyrus	0.031	0.091	[-0.147 - 0.208]	7.355E-01	9.681E-01
Left isthmus cingulate cortex	0.040	0.090	[-0.137 - 0.218]	6.559E-01	9.681E-01
Right isthmus cingulate cortex	-0.024	0.090	[-0.202 - 0.153]	7.889E-01	9.681E-01
Left banks of superior temporal sulcus	-0.145	0.101	[-0.343 - 0.053]	1.522E-01	9.681E-01
Right banks of superior temporal sulcus	0.061	0.091	[-0.117 - 0.239]	5.005E-01	9.681E-01
Left supramarginal gyrus	0.091	0.091	[-0.086 - 0.269]	3.134E-01	9.681E-01
Right supramarginal gyrus	-0.052	0.104	[-0.256 - 0.151]	6.146E-01	9.681E-01
Left caudal middle frontal gyrus	0.184	0.091	[0.006 - 0.361]	4.275E-02	9.681E-01
Right caudal middle frontal gyrus	0.063	0.091	[-0.115 - 0.240]	4.881E-01	9.681E-01
Left frontal pole	0.030	0.091	[-0.147 - 0.207]	7.394E-01	9.681E-01
Right frontal pole	0.074	0.143	[-0.206 - 0.354]	6.032E-01	9.681E-01
Left posterior cingulate cortex	0.066	0.117	[-0.163 - 0.296]	5.704E-01	9.681E-01
Right posterior cingulate cortex	-0.070	0.090	[-0.248 - 0.107]	4.366E-01	9.681E-01
Left lateral occipital cortex	-0.047	0.091	[-0.224 - 0.131]	6.045E-01	9.681E-01
Right lateral occipital cortex	-0.071	0.093	[-0.254 - 0.112]	4.471E-01	9.681E-01
Left precentral gyrus	0.089	0.091	[-0.089 - 0.266]	3.270E-01	9.681E-01
Right precentral gyrus	0.018	0.098	[-0.173 - 0.210]	8.519E-01	9.681E-01
Left parahippocampal gyrus	-0.170	0.091	[-0.347 - 0.008]	6.117E-02	9.681E-01
Right parahippocampal gyrus	-0.098	0.091	[-0.276 - 0.079]	2.773E-01	9.681E-01
Left inferior parietal cortex	0.065	0.090	[-0.113 - 0.242]	4.747E-01	9.681E-01
Right inferior parietal cortex	0.104	0.091	[-0.073 - 0.282]	2.488E-01	9.681E-01
Left transverse temporal gyrus	-0.040	0.090	[-0.217 - 0.138]	6.613E-01	9.681E-01
Right transverse temporal gyrus	-0.010	0.091	[-0.188 - 0.167]	9.099E-01	9.681E-01
Left postcentral gyrus	0.092	0.096	[-0.095 - 0.280]	3.344E-01	9.681E-01
Right postcentral gyrus	-0.018	0.119	[-0.251 - 0.216]	8.829E-01	9.681E-01
Left precuneus	0.085	0.090	[-0.093 - 0.262]	3.484E-01	9.681E-01
Right precuneus	0.097	0.091	[-0.080 - 0.275]	2.817E-01	9.681E-01
Left caudal anterior cingulate cortex	-0.158	0.122	[-0.397 - 0.080]	1.934E-01	9.681E-01
Right caudal anterior cingulate cortex	-0.028	0.091	[-0.205 - 0.149]	7.574E-01	9.681E-01
Left cuneus	0.113	0.092	[-0.068 - 0.294]	2.204E-01	9.681E-01
Right cuneus	0.034	0.123	[-0.207 - 0.275]	7.811E-01	9.681E-01
Left rostral anterior cingulate cortex	-0.014	0.091	[-0.192 - 0.163]	8.760E-01	9.681E-01
Right rostral anterior cingulate cortex	-0.015	0.109	[-0.229 - 0.200]	8.937E-01	9.681E-01
Left pericalcarine cortex	0.064	0.115	[-0.160 - 0.289]	5.739E-01	9.681E-01
Right pericalcarine cortex	-0.043	0.090	[-0.220 - 0.134]	6.356E-01	9.681E-01
Left paracentral lobule	0.064	0.091	[-0.113 - 0.242]	4.769E-01	9.681E-01
Right paracentral lobule	0.100	0.106	[-0.107 - 0.308]	3.435E-01	9.681E-01
Left superior parietal cortex	0.060	0.091	[-0.117 - 0.238]	5.043E-01	9.681E-01
Right superior parietal cortex	0.036	0.091	[-0.142 - 0.213]	6.947E-01	9.681E-01
Left temporal pole	-0.098	0.091	[-0.276 - 0.079]	2.786E-01	9.681E-01
Right temporal pole	-0.028	0.102	[-0.229 - 0.172]	7.812E-01	9.681E-01
Left entorhinal cortex	0.059	0.091	[-0.118 - 0.237]	5.133E-01	9.681E-01
Right entorhinal cortex	0.119	0.091	[-0.058 - 0.297]	1.875E-01	9.681E-01

Supplementary Table S47. Age-by-Diagnosis interaction (BD/HC) on cortical thickness differences controlling for age and sex

	d	Std. Err.	95% CI	*p*-value	FDR *q*-value
Global mean cortical thickness	-0.069	0.171	[-0.403 - 0.266]	6.884E-01	9.975E-01
Left hemisphere	-0.088	0.167	[-0.416 - 0.240]	5.982E-01	9.975E-01
Right hemisphere	-0.045	0.173	[-0.384 - 0.293]	7.932E-01	9.975E-01
Left fusiform gyrus	-0.195	0.115	[-0.421 - 0.030]	8.879E-02	9.975E-01
Right fusiform gyrus	-0.168	0.148	[-0.458 - 0.122]	2.555E-01	9.975E-01
Left pars opercularis of inferior frontal gyrus	0.034	0.165	[-0.289 - 0.358]	8.351E-01	9.975E-01
Right pars opercularis of inferior frontal gyrus	0.065	0.176	[-0.279 - 0.410]	7.096E-01	9.975E-01
Left superior temporal gyrus	-0.153	0.157	[-0.460 - 0.155]	3.308E-01	9.975E-01
Right superior temporal gyrus	-0.099	0.163	[-0.419 - 0.221]	5.453E-01	9.975E-01
Left insula	-0.116	0.132	[-0.374 - 0.143]	3.796E-01	9.975E-01
Right insula	-0.019	0.110	[-0.235 - 0.196]	8.614E-01	9.975E-01
Left lingual gyrus	0.019	0.129	[-0.234 - 0.272]	8.823E-01	9.975E-01
Right lingual gyrus	0.055	0.131	[-0.201 - 0.311]	6.752E-01	9.975E-01
Left pars triangularis of inferior frontal gyrus	-0.039	0.127	[-0.288 - 0.209]	7.578E-01	9.975E-01
Right pars triangularis of inferior frontal gyrus	0.092	0.107	[-0.118 - 0.302]	3.915E-01	9.975E-01
Left lateral orbitofrontal cortex	-0.221	0.106	[-0.429 - -0.012]	3.811E-02	9.975E-01
Right lateral orbitofrontal cortex	-0.114	0.144	[-0.396 - 0.168]	4.290E-01	9.975E-01
Left rostral middle frontal gyrus	-0.195	0.108	[-0.406 - 0.016]	6.984E-02	9.975E-01
Right rostral middle frontal gyrus	-0.144	0.121	[-0.381 - 0.094]	2.353E-01	9.975E-01
Left middle temporal gyrus	-0.112	0.161	[-0.427 - 0.204]	4.889E-01	9.975E-01
Right middle temporal gyrus	-0.080	0.138	[-0.350 - 0.189]	5.589E-01	9.975E-01
Left superior frontal gyrus	-0.016	0.144	[-0.299 - 0.266]	9.089E-01	9.975E-01
Right superior frontal gyrus	-0.028	0.134	[-0.290 - 0.234]	8.344E-01	9.975E-01
Left pars orbitalis of inferior frontal gyrus	-0.180	0.137	[-0.449 - 0.089]	1.890E-01	9.975E-01
Right pars orbitalis of inferior frontal gyrus	-0.176	0.121	[-0.414 - 0.062]	1.471E-01	9.975E-01
Left medial orbitofrontal cortex	-0.083	0.138	[-0.354 - 0.188]	5.476E-01	9.975E-01
Right medial orbitofrontal cortex	-0.129	0.147	[-0.418 - 0.160]	3.816E-01	9.975E-01
Left inferior temporal gyrus	-0.025	0.162	[-0.343 - 0.293]	8.773E-01	9.975E-01
Right inferior temporal gyrus	-0.064	0.156	[-0.370 - 0.241]	6.794E-01	9.975E-01
Left isthmus cingulate cortex	-0.066	0.085	[-0.233 - 0.101]	4.367E-01	9.975E-01
Right isthmus cingulate cortex	-0.002	0.139	[-0.274 - 0.270]	9.894E-01	9.975E-01
Left banks of superior temporal sulcus	0.019	0.124	[-0.225 - 0.263]	8.792E-01	9.975E-01
Right banks of superior temporal sulcus	-0.005	0.157	[-0.313 - 0.303]	9.736E-01	9.975E-01
Left supramarginal gyrus	-0.057	0.172	[-0.395 - 0.281]	7.397E-01	9.975E-01
Right supramarginal gyrus	0.023	0.184	[-0.337 - 0.383]	8.995E-01	9.975E-01
Left caudal middle frontal gyrus	-0.051	0.126	[-0.297 - 0.195]	6.821E-01	9.975E-01
Right caudal middle frontal gyrus	0.031	0.110	[-0.184 - 0.245]	7.796E-01	9.975E-01
Left frontal pole	-0.168	0.085	[-0.335 - -0.001]	4.907E-02	9.975E-01
Right frontal pole	-0.179	0.096	[-0.366 - 0.008]	6.073E-02	9.975E-01
Left posterior cingulate cortex	0.019	0.148	[-0.270 - 0.309]	8.953E-01	9.975E-01
Right posterior cingulate cortex	-0.003	0.144	[-0.286 - 0.279]	9.818E-01	9.975E-01
Left lateral occipital cortex	-0.140	0.122	[-0.379 - 0.099]	2.524E-01	9.975E-01
Right lateral occipital cortex	-0.083	0.123	[-0.324 - 0.159]	5.023E-01	9.975E-01
Left precentral gyrus	-0.077	0.107	[-0.288 - 0.133]	4.712E-01	9.975E-01
Right precentral gyrus	-0.026	0.117	[-0.255 - 0.203]	8.255E-01	9.975E-01
Left parahippocampal gyrus	-0.002	0.085	[-0.168 - 0.165]	9.846E-01	9.975E-01
Right parahippocampal gyrus	0.001	0.093	[-0.182 - 0.184]	9.927E-01	9.975E-01
Left inferior parietal cortex	0.030	0.147	[-0.258 - 0.319]	8.366E-01	9.975E-01
Right inferior parietal cortex	-0.016	0.144	[-0.298 - 0.265]	9.097E-01	9.975E-01
Left transverse temporal gyrus	-0.085	0.123	[-0.327 - 0.156]	4.889E-01	9.975E-01
Right transverse temporal gyrus	0.028	0.116	[-0.200 - 0.256]	8.122E-01	9.975E-01
Left postcentral gyrus	-0.111	0.163	[-0.431 - 0.208]	4.950E-01	9.975E-01
Right postcentral gyrus	-0.041	0.133	[-0.302 - 0.220]	7.583E-01	9.975E-01
Left precuneus	0.060	0.168	[-0.270 - 0.390]	7.225E-01	9.975E-01
Right precuneus	0.021	0.166	[-0.304 - 0.345]	9.014E-01	9.975E-01
Left caudal anterior cingulate cortex	-0.014	0.098	[-0.206 - 0.177]	8.836E-01	9.975E-01
Right caudal anterior cingulate cortex	0.049	0.101	[-0.149 - 0.248]	6.277E-01	9.975E-01
Left cuneus	0.011	0.101	[-0.186 - 0.208]	9.101E-01	9.975E-01
Right cuneus	-0.023	0.113	[-0.244 - 0.198]	8.406E-01	9.975E-01
Left rostral anterior cingulate cortex	-0.028	0.114	[-0.252 - 0.196]	8.080E-01	9.975E-01
Right rostral anterior cingulate cortex	-0.135	0.085	[-0.302 - 0.032]	1.141E-01	9.975E-01
Left pericalcarine cortex	0.101	0.085	[-0.066 - 0.267]	2.373E-01	9.975E-01
Right pericalcarine cortex	0.082	0.090	[-0.095 - 0.259]	3.644E-01	9.975E-01
Left paracentral lobule	0.044	0.118	[-0.188 - 0.275]	7.100E-01	9.975E-01
Right paracentral lobule	0.014	0.124	[-0.230 - 0.258]	9.114E-01	9.975E-01
Left superior parietal cortex	-0.002	0.115	[-0.228 - 0.224]	9.849E-01	9.975E-01
Right superior parietal cortex	0.000	0.130	[-0.255 - 0.255]	9.975E-01	9.975E-01
Left temporal pole	-0.068	0.117	[-0.297 - 0.161]	5.606E-01	9.975E-01
Right temporal pole	-0.198	0.149	[-0.489 - 0.094]	1.848E-01	9.975E-01
Left entorhinal cortex	-0.215	0.132	[-0.475 - 0.044]	1.038E-01	9.975E-01
Right entorhinal cortex	-0.116	0.123	[-0.356 - 0.124]	3.427E-01	9.975E-01

Supplementary Table S48. Age-by-Diagnosis interaction (BD/HC) on cortical thickness differences controlling for age and sex at 25 years of age or older

	d	Std. Err.	95% CI	*p*-value	FDR *q*-value
Global mean cortical thickness	-0.059	0.161	[-0.374 - 0.256]	7.146E-01	9.922E-01
Left hemisphere	-0.085	0.157	[-0.392 - 0.222]	5.875E-01	9.922E-01
Right hemisphere	-0.029	0.164	[-0.350 - 0.291]	8.572E-01	9.922E-01
Left fusiform gyrus	-0.203	0.107	[-0.413 - 0.007]	5.770E-02	9.922E-01
Right fusiform gyrus	-0.182	0.150	[-0.475 - 0.112]	2.247E-01	9.922E-01
Left pars opercularis of inferior frontal gyrus	0.017	0.174	[-0.324 - 0.359]	9.202E-01	9.922E-01
Right pars opercularis of inferior frontal gyrus	0.075	0.167	[-0.252 - 0.402]	6.528E-01	9.922E-01
Left superior temporal gyrus	-0.215	0.141	[-0.492 - 0.063]	1.292E-01	9.922E-01
Right superior temporal gyrus	-0.100	0.153	[-0.400 - 0.199]	5.109E-01	9.922E-01
Left insula	-0.121	0.137	[-0.389 - 0.147]	3.754E-01	9.922E-01
Right insula	-0.019	0.122	[-0.257 - 0.219]	8.748E-01	9.922E-01
Left lingual gyrus	-0.025	0.116	[-0.252 - 0.202]	8.289E-01	9.922E-01
Right lingual gyrus	-0.011	0.109	[-0.225 - 0.203]	9.196E-01	9.922E-01
Left pars triangularis of inferior frontal gyrus	-0.054	0.119	[-0.287 - 0.178]	6.476E-01	9.922E-01
Right pars triangularis of inferior frontal gyrus	0.095	0.088	[-0.077 - 0.268]	2.793E-01	9.922E-01
Left lateral orbitofrontal cortex	-0.243	0.109	[-0.456 - -0.030]	2.510E-02	9.922E-01
Right lateral orbitofrontal cortex	-0.120	0.153	[-0.420 - 0.180]	4.321E-01	9.922E-01
Left rostral middle frontal gyrus	-0.194	0.103	[-0.396 - 0.009]	6.076E-02	9.922E-01
Right rostral middle frontal gyrus	-0.094	0.123	[-0.335 - 0.147]	4.445E-01	9.922E-01
Left middle temporal gyrus	-0.119	0.158	[-0.428 - 0.191]	4.519E-01	9.922E-01
Right middle temporal gyrus	-0.042	0.151	[-0.338 - 0.255]	7.832E-01	9.922E-01
Left superior frontal gyrus	0.035	0.135	[-0.230 - 0.299]	7.973E-01	9.922E-01
Right superior frontal gyrus	-0.009	0.132	[-0.268 - 0.250]	9.468E-01	9.922E-01
Left pars orbitalis of inferior frontal gyrus	-0.189	0.146	[-0.475 - 0.097]	1.961E-01	9.922E-01
Right pars orbitalis of inferior frontal gyrus	-0.151	0.129	[-0.403 - 0.101]	2.404E-01	9.922E-01
Left medial orbitofrontal cortex	-0.105	0.152	[-0.403 - 0.193]	4.891E-01	9.922E-01
Right medial orbitofrontal cortex	-0.150	0.156	[-0.456 - 0.156]	3.375E-01	9.922E-01
Left inferior temporal gyrus	-0.046	0.148	[-0.336 - 0.244]	7.568E-01	9.922E-01
Right inferior temporal gyrus	-0.092	0.150	[-0.386 - 0.202]	5.384E-01	9.922E-01
Left isthmus cingulate cortex	-0.101	0.088	[-0.274 - 0.071]	2.487E-01	9.922E-01
Right isthmus cingulate cortex	-0.005	0.129	[-0.258 - 0.248]	9.698E-01	9.922E-01
Left banks of superior temporal sulcus	0.000	0.107	[-0.209 - 0.209]	9.971E-01	9.971E-01
Right banks of superior temporal sulcus	-0.033	0.125	[-0.278 - 0.212]	7.920E-01	9.922E-01
Left supramarginal gyrus	-0.080	0.171	[-0.415 - 0.255]	6.410E-01	9.922E-01
Right supramarginal gyrus	0.055	0.187	[-0.311 - 0.421]	7.691E-01	9.922E-01
Left caudal middle frontal gyrus	-0.041	0.124	[-0.285 - 0.203]	7.412E-01	9.922E-01
Right caudal middle frontal gyrus	0.074	0.106	[-0.134 - 0.281]	4.876E-01	9.922E-01
Left frontal pole	-0.138	0.088	[-0.311 - 0.034]	1.159E-01	9.922E-01
Right frontal pole	-0.149	0.103	[-0.350 - 0.052]	1.457E-01	9.922E-01
Left posterior cingulate cortex	0.046	0.132	[-0.213 - 0.304]	7.304E-01	9.922E-01
Right posterior cingulate cortex	0.055	0.114	[-0.169 - 0.278]	6.313E-01	9.922E-01
Left lateral occipital cortex	-0.099	0.114	[-0.322 - 0.124]	3.853E-01	9.922E-01
Right lateral occipital cortex	-0.106	0.099	[-0.299 - 0.087]	2.822E-01	9.922E-01
Left precentral gyrus	-0.097	0.104	[-0.301 - 0.106]	3.487E-01	9.922E-01
Right precentral gyrus	0.025	0.111	[-0.192 - 0.242]	8.215E-01	9.922E-01
Left parahippocampal gyrus	0.004	0.088	[-0.168 - 0.176]	9.621E-01	9.922E-01
Right parahippocampal gyrus	-0.018	0.088	[-0.191 - 0.154]	8.342E-01	9.922E-01
Left inferior parietal cortex	0.064	0.153	[-0.235 - 0.364]	6.736E-01	9.922E-01
Right inferior parietal cortex	0.027	0.148	[-0.263 - 0.316]	8.566E-01	9.922E-01
Left transverse temporal gyrus	-0.065	0.112	[-0.283 - 0.154]	5.623E-01	9.922E-01
Right transverse temporal gyrus	0.062	0.118	[-0.169 - 0.294]	5.987E-01	9.922E-01
Left postcentral gyrus	-0.144	0.139	[-0.417 - 0.130]	3.027E-01	9.922E-01
Right postcentral gyrus	-0.085	0.111	[-0.303 - 0.133]	4.448E-01	9.922E-01
Left precuneus	0.069	0.151	[-0.228 - 0.365]	6.504E-01	9.922E-01
Right precuneus	0.004	0.157	[-0.303 - 0.312]	9.775E-01	9.922E-01
Left caudal anterior cingulate cortex	0.035	0.105	[-0.172 - 0.242]	7.392E-01	9.922E-01
Right caudal anterior cingulate cortex	0.087	0.092	[-0.093 - 0.267]	3.444E-01	9.922E-01
Left cuneus	0.020	0.107	[-0.189 - 0.230]	8.479E-01	9.922E-01
Right cuneus	-0.010	0.120	[-0.245 - 0.225]	9.339E-01	9.922E-01
Left rostral anterior cingulate cortex	-0.006	0.111	[-0.223 - 0.211]	9.542E-01	9.922E-01
Right rostral anterior cingulate cortex	-0.144	0.088	[-0.316 - 0.029]	1.033E-01	9.922E-01
Left pericalcarine cortex	0.060	0.088	[-0.112 - 0.233]	4.920E-01	9.922E-01
Right pericalcarine cortex	0.038	0.088	[-0.135 - 0.211]	6.678E-01	9.922E-01
Left paracentral lobule	0.056	0.114	[-0.167 - 0.280]	6.210E-01	9.922E-01
Right paracentral lobule	0.026	0.123	[-0.215 - 0.267]	8.316E-01	9.922E-01
Left superior parietal cortex	-0.003	0.113	[-0.225 - 0.219]	9.782E-01	9.922E-01
Right superior parietal cortex	-0.016	0.117	[-0.245 - 0.213]	8.920E-01	9.922E-01
Left temporal pole	-0.060	0.139	[-0.332 - 0.211]	6.635E-01	9.922E-01
Right temporal pole	-0.220	0.157	[-0.527 - 0.087]	1.595E-01	9.922E-01
Left entorhinal cortex	-0.117	0.119	[-0.351 - 0.117]	3.264E-01	9.922E-01
Right entorhinal cortex	-0.058	0.145	[-0.342 - 0.227]	6.914E-01	9.922E-01

Supplementary Table S49. Sex-by-Diagnosis interaction (MD/HC) on cortical thickness differences controlling for age and sex

	d	Std. Err.	95% CI	*p*-value	FDR *q*-value
Global mean cortical thickness	0.018	0.061	[-0.102 - 0.139]	7.634E-01	9.873E-01
Left hemisphere	0.021	0.061	[-0.099 - 0.141]	7.309E-01	9.873E-01
Right hemisphere	0.015	0.061	[-0.105 - 0.136]	8.026E-01	9.873E-01
Left fusiform gyrus	-0.055	0.082	[-0.216 - 0.106]	5.014E-01	9.873E-01
Right fusiform gyrus	0.047	0.100	[-0.148 - 0.242]	6.380E-01	9.873E-01
Left pars opercularis of inferior frontal gyrus	0.051	0.061	[-0.070 - 0.171]	4.094E-01	9.873E-01
Right pars opercularis of inferior frontal gyrus	0.011	0.061	[-0.110 - 0.131]	8.636E-01	9.983E-01
Left superior temporal gyrus	-0.119	0.078	[-0.272 - 0.034]	1.276E-01	9.873E-01
Right superior temporal gyrus	-0.061	0.065	[-0.189 - 0.067]	3.522E-01	9.873E-01
Left insula	-0.074	0.061	[-0.194 - 0.046]	2.277E-01	9.873E-01
Right insula	-0.058	0.064	[-0.185 - 0.068]	3.648E-01	9.873E-01
Left lingual gyrus	-0.013	0.061	[-0.133 - 0.107]	8.297E-01	9.873E-01
Right lingual gyrus	-0.025	0.061	[-0.145 - 0.095]	6.840E-01	9.873E-01
Left pars triangularis of inferior frontal gyrus	0.003	0.061	[-0.117 - 0.123]	9.642E-01	9.983E-01
Right pars triangularis of inferior frontal gyrus	0.004	0.061	[-0.116 - 0.125]	9.434E-01	9.983E-01
Left lateral orbitofrontal cortex	-0.035	0.087	[-0.206 - 0.136]	6.858E-01	9.873E-01
Right lateral orbitofrontal cortex	0.014	0.067	[-0.117 - 0.144]	8.344E-01	9.873E-01
Left rostral middle frontal gyrus	0.065	0.068	[-0.069 - 0.199]	3.429E-01	9.873E-01
Right rostral middle frontal gyrus	0.099	0.061	[-0.022 - 0.219]	1.082E-01	9.873E-01
Left middle temporal gyrus	0.017	0.073	[-0.126 - 0.159]	8.155E-01	9.873E-01
Right middle temporal gyrus	0.073	0.076	[-0.075 - 0.221]	3.348E-01	9.873E-01
Left superior frontal gyrus	0.063	0.061	[-0.057 - 0.183]	3.032E-01	9.873E-01
Right superior frontal gyrus	0.026	0.061	[-0.094 - 0.146]	6.729E-01	9.873E-01
Left pars orbitalis of inferior frontal gyrus	0.057	0.103	[-0.144 - 0.259]	5.765E-01	9.873E-01
Right pars orbitalis of inferior frontal gyrus	-0.016	0.061	[-0.136 - 0.105]	7.974E-01	9.873E-01
Left medial orbitofrontal cortex	-0.010	0.085	[-0.177 - 0.157]	9.091E-01	9.983E-01
Right medial orbitofrontal cortex	-0.029	0.064	[-0.154 - 0.096]	6.485E-01	9.873E-01
Left inferior temporal gyrus	-0.028	0.068	[-0.160 - 0.105]	6.834E-01	9.873E-01
Right inferior temporal gyrus	-0.001	0.079	[-0.156 - 0.154]	9.898E-01	9.983E-01
Left isthmus cingulate cortex	0.003	0.061	[-0.117 - 0.123]	9.638E-01	9.983E-01
Right isthmus cingulate cortex	0.029	0.078	[-0.124 - 0.182]	7.115E-01	9.873E-01
Left banks of superior temporal sulcus	-0.027	0.071	[-0.166 - 0.111]	6.993E-01	9.873E-01
Right banks of superior temporal sulcus	0.051	0.061	[-0.069 - 0.172]	4.024E-01	9.873E-01
Left supramarginal gyrus	0.074	0.061	[-0.046 - 0.194]	2.273E-01	9.873E-01
Right supramarginal gyrus	0.044	0.064	[-0.082 - 0.171]	4.910E-01	9.873E-01
Left caudal middle frontal gyrus	0.059	0.063	[-0.065 - 0.182]	3.512E-01	9.873E-01
Right caudal middle frontal gyrus	0.023	0.061	[-0.097 - 0.143]	7.075E-01	9.873E-01
Left frontal pole	0.027	0.061	[-0.093 - 0.148]	6.565E-01	9.873E-01
Right frontal pole	0.084	0.061	[-0.036 - 0.204]	1.714E-01	9.873E-01
Left posterior cingulate cortex	-0.056	0.064	[-0.182 - 0.070]	3.828E-01	9.873E-01
Right posterior cingulate cortex	0.000	0.061	[-0.120 - 0.120]	9.983E-01	9.983E-01
Left lateral occipital cortex	-0.041	0.071	[-0.180 - 0.098]	5.622E-01	9.873E-01
Right lateral occipital cortex	-0.052	0.064	[-0.178 - 0.074]	4.170E-01	9.873E-01
Left precentral gyrus	0.002	0.061	[-0.118 - 0.122]	9.764E-01	9.983E-01
Right precentral gyrus	-0.005	0.061	[-0.125 - 0.115]	9.336E-01	9.983E-01
Left parahippocampal gyrus	-0.120	0.075	[-0.267 - 0.027]	1.098E-01	9.873E-01
Right parahippocampal gyrus	-0.042	0.064	[-0.167 - 0.084]	5.164E-01	9.873E-01
Left inferior parietal cortex	0.119	0.088	[-0.054 - 0.291]	1.778E-01	9.873E-01
Right inferior parietal cortex	0.139	0.061	[0.018 - 0.259]	2.389E-02	9.323E-01
Left transverse temporal gyrus	-0.072	0.061	[-0.192 - 0.048]	2.399E-01	9.873E-01
Right transverse temporal gyrus	-0.020	0.071	[-0.160 - 0.120]	7.815E-01	9.873E-01
Left postcentral gyrus	0.002	0.061	[-0.118 - 0.122]	9.754E-01	9.983E-01
Right postcentral gyrus	-0.047	0.093	[-0.229 - 0.136]	6.183E-01	9.873E-01
Left precuneus	0.005	0.063	[-0.118 - 0.127]	9.406E-01	9.983E-01
Right precuneus	0.048	0.068	[-0.086 - 0.183]	4.785E-01	9.873E-01
Left caudal anterior cingulate cortex	0.054	0.063	[-0.069 - 0.177]	3.914E-01	9.873E-01
Right caudal anterior cingulate cortex	-0.076	0.066	[-0.205 - 0.053]	2.465E-01	9.873E-01
Left cuneus	0.129	0.061	[0.009 - 0.249]	3.543E-02	9.323E-01
Right cuneus	0.106	0.076	[-0.043 - 0.255]	1.623E-01	9.873E-01
Left rostral anterior cingulate cortex	-0.056	0.072	[-0.197 - 0.085]	4.363E-01	9.873E-01
Right rostral anterior cingulate cortex	0.031	0.061	[-0.090 - 0.151]	6.165E-01	9.873E-01
Left pericalcarine cortex	0.077	0.079	[-0.077 - 0.231]	3.292E-01	9.873E-01
Right pericalcarine cortex	0.100	0.086	[-0.068 - 0.267]	2.449E-01	9.873E-01
Left paracentral lobule	0.089	0.061	[-0.031 - 0.210]	1.452E-01	9.873E-01
Right paracentral lobule	0.039	0.061	[-0.081 - 0.159]	5.254E-01	9.873E-01
Left superior parietal cortex	0.093	0.061	[-0.028 - 0.213]	1.310E-01	9.873E-01
Right superior parietal cortex	0.034	0.061	[-0.086 - 0.155]	5.764E-01	9.873E-01
Left temporal pole	-0.035	0.061	[-0.155 - 0.086]	5.717E-01	9.873E-01
Right temporal pole	-0.126	0.061	[-0.247 - -0.006]	3.939E-02	9.323E-01
Left entorhinal cortex	-0.084	0.080	[-0.240 - 0.072]	2.918E-01	9.873E-01
Right entorhinal cortex	-0.024	0.081	[-0.182 - 0.134]	7.671E-01	9.873E-01

Supplementary Table S50. Sex-by-Diagnosis interaction (MD/HC) on cortical thickness differences controlling for age and sex over 21 years of old

	d	Std. Err.	95% CI	*p*-value	FDR *q*-value
Global mean cortical thickness	0.013	0.063	[-0.110 - 0.137]	8.313E-01	9.985E-01
Left hemisphere	0.010	0.063	[-0.113 - 0.134]	8.702E-01	9.985E-01
Right hemisphere	0.017	0.063	[-0.107 - 0.141]	7.872E-01	9.985E-01
Left fusiform gyrus	-0.068	0.081	[-0.227 - 0.090]	3.981E-01	9.640E-01
Right fusiform gyrus	0.011	0.092	[-0.169 - 0.190]	9.068E-01	9.985E-01
Left pars opercularis of inferior frontal gyrus	0.039	0.063	[-0.084 - 0.163]	5.333E-01	9.640E-01
Right pars opercularis of inferior frontal gyrus	0.004	0.063	[-0.120 - 0.128]	9.491E-01	9.985E-01
Left superior temporal gyrus	-0.095	0.074	[-0.241 - 0.050]	1.996E-01	9.640E-01
Right superior temporal gyrus	-0.044	0.063	[-0.168 - 0.080]	4.859E-01	9.640E-01
Left insula	-0.065	0.063	[-0.189 - 0.059]	3.044E-01	9.640E-01
Right insula	-0.057	0.063	[-0.181 - 0.066]	3.639E-01	9.640E-01
Left lingual gyrus	-0.031	0.063	[-0.155 - 0.092]	6.210E-01	9.799E-01
Right lingual gyrus	-0.034	0.063	[-0.158 - 0.089]	5.872E-01	9.792E-01
Left pars triangularis of inferior frontal gyrus	0.000	0.063	[-0.124 - 0.123]	9.985E-01	9.985E-01
Right pars triangularis of inferior frontal gyrus	0.032	0.063	[-0.092 - 0.155]	6.155E-01	9.799E-01
Left lateral orbitofrontal cortex	-0.062	0.086	[-0.230 - 0.107]	4.753E-01	9.640E-01
Right lateral orbitofrontal cortex	-0.001	0.063	[-0.124 - 0.123]	9.923E-01	9.985E-01
Left rostral middle frontal gyrus	0.041	0.068	[-0.093 - 0.175]	5.459E-01	9.640E-01
Right rostral middle frontal gyrus	0.090	0.063	[-0.033 - 0.214]	1.522E-01	9.640E-01
Left middle temporal gyrus	-0.015	0.075	[-0.162 - 0.132]	8.412E-01	9.985E-01
Right middle temporal gyrus	0.076	0.077	[-0.075 - 0.227]	3.218E-01	9.640E-01
Left superior frontal gyrus	0.040	0.063	[-0.084 - 0.163]	5.292E-01	9.640E-01
Right superior frontal gyrus	0.026	0.063	[-0.097 - 0.150]	6.776E-01	9.818E-01
Left pars orbitalis of inferior frontal gyrus	0.047	0.105	[-0.158 - 0.253]	6.508E-01	9.813E-01
Right pars orbitalis of inferior frontal gyrus	-0.019	0.063	[-0.142 - 0.105]	7.690E-01	9.985E-01
Left medial orbitofrontal cortex	-0.011	0.082	[-0.172 - 0.151]	8.975E-01	9.985E-01
Right medial orbitofrontal cortex	-0.029	0.063	[-0.153 - 0.095]	6.463E-01	9.813E-01
Left inferior temporal gyrus	-0.051	0.077	[-0.201 - 0.099]	5.091E-01	9.640E-01
Right inferior temporal gyrus	-0.008	0.078	[-0.161 - 0.145]	9.222E-01	9.985E-01
Left isthmus cingulate cortex	0.008	0.063	[-0.115 - 0.132]	8.955E-01	9.985E-01
Right isthmus cingulate cortex	0.043	0.081	[-0.115 - 0.201]	5.930E-01	9.792E-01
Left banks of superior temporal sulcus	-0.057	0.066	[-0.187 - 0.073]	3.884E-01	9.640E-01
Right banks of superior temporal sulcus	0.046	0.063	[-0.078 - 0.169]	4.686E-01	9.640E-01
Left supramarginal gyrus	0.080	0.063	[-0.043 - 0.204]	2.034E-01	9.640E-01
Right supramarginal gyrus	0.051	0.069	[-0.084 - 0.186]	4.572E-01	9.640E-01
Left caudal middle frontal gyrus	0.057	0.067	[-0.074 - 0.188]	3.948E-01	9.640E-01
Right caudal middle frontal gyrus	0.027	0.063	[-0.096 - 0.151]	6.634E-01	9.813E-01
Left frontal pole	0.013	0.063	[-0.111 - 0.136]	8.417E-01	9.985E-01
Right frontal pole	0.071	0.063	[-0.053 - 0.195]	2.627E-01	9.640E-01
Left posterior cingulate cortex	-0.056	0.066	[-0.185 - 0.072]	3.899E-01	9.640E-01
Right posterior cingulate cortex	0.002	0.063	[-0.122 - 0.125]	9.795E-01	9.985E-01
Left lateral occipital cortex	-0.015	0.077	[-0.165 - 0.135]	8.422E-01	9.985E-01
Right lateral occipital cortex	-0.037	0.064	[-0.162 - 0.087]	5.567E-01	9.640E-01
Left precentral gyrus	0.004	0.063	[-0.120 - 0.127]	9.533E-01	9.985E-01
Right precentral gyrus	0.009	0.063	[-0.115 - 0.132]	8.908E-01	9.985E-01
Left parahippocampal gyrus	-0.126	0.069	[-0.260 - 0.009]	6.803E-02	9.640E-01
Right parahippocampal gyrus	-0.042	0.063	[-0.166 - 0.081]	5.031E-01	9.640E-01
Left inferior parietal cortex	0.101	0.090	[-0.075 - 0.278]	2.615E-01	9.640E-01
Right inferior parietal cortex	0.149	0.063	[0.026 - 0.273]	1.803E-02	9.321E-01
Left transverse temporal gyrus	-0.056	0.063	[-0.180 - 0.067]	3.714E-01	9.640E-01
Right transverse temporal gyrus	-0.011	0.079	[-0.165 - 0.143]	8.873E-01	9.985E-01
Left postcentral gyrus	0.002	0.063	[-0.121 - 0.126]	9.716E-01	9.985E-01
Right postcentral gyrus	-0.026	0.093	[-0.208 - 0.156]	7.760E-01	9.985E-01
Left precuneus	-0.020	0.065	[-0.148 - 0.107]	7.530E-01	9.985E-01
Right precuneus	0.052	0.075	[-0.095 - 0.199]	4.874E-01	9.640E-01
Left caudal anterior cingulate cortex	0.057	0.066	[-0.072 - 0.187]	3.856E-01	9.640E-01
Right caudal anterior cingulate cortex	-0.047	0.071	[-0.186 - 0.092]	5.101E-01	9.640E-01
Left cuneus	0.130	0.063	[0.006 - 0.254]	3.939E-02	9.321E-01
Right cuneus	0.112	0.078	[-0.041 - 0.265]	1.527E-01	9.640E-01
Left rostral anterior cingulate cortex	-0.051	0.074	[-0.195 - 0.094]	4.913E-01	9.640E-01
Right rostral anterior cingulate cortex	0.053	0.063	[-0.071 - 0.177]	4.002E-01	9.640E-01
Left pericalcarine cortex	0.096	0.076	[-0.053 - 0.246]	2.068E-01	9.640E-01
Right pericalcarine cortex	0.112	0.088	[-0.059 - 0.284]	2.004E-01	9.640E-01
Left paracentral lobule	0.087	0.063	[-0.036 - 0.211]	1.667E-01	9.640E-01
Right paracentral lobule	0.042	0.063	[-0.082 - 0.166]	5.065E-01	9.640E-01
Left superior parietal cortex	0.094	0.063	[-0.029 - 0.218]	1.345E-01	9.640E-01
Right superior parietal cortex	0.043	0.068	[-0.091 - 0.176]	5.307E-01	9.640E-01
Left temporal pole	-0.066	0.063	[-0.190 - 0.057]	2.931E-01	9.640E-01
Right temporal pole	-0.138	0.063	[-0.262 - -0.015]	2.831E-02	9.321E-01
Left entorhinal cortex	-0.064	0.078	[-0.217 - 0.090]	4.165E-01	9.640E-01
Right entorhinal cortex	-0.030	0.077	[-0.182 - 0.122]	6.991E-01	9.928E-01

Supplementary Table S51. Age-by-Diagnosis interaction (MD/HC) on cortical thickness differences controlling for age and sex

	d	Std. Err.	95% CI	*p*-value	FDR *q*-value
Global mean cortical thickness	0.129	0.109	[-0.085 - 0.343]	2.384E-01	6.510E-01
Left hemisphere	0.147	0.115	[-0.078 - 0.372]	2.007E-01	6.296E-01
Right hemisphere	0.100	0.101	[-0.098 - 0.298]	3.229E-01	7.165E-01
Left fusiform gyrus	0.002	0.093	[-0.181 - 0.184]	9.839E-01	9.839E-01
Right fusiform gyrus	-0.095	0.072	[-0.236 - 0.047]	1.895E-01	6.296E-01
Left pars opercularis of inferior frontal gyrus	0.248	0.111	[0.031 - 0.466]	2.542E-02	3.609E-01
Right pars opercularis of inferior frontal gyrus	0.096	0.094	[-0.089 - 0.281]	3.104E-01	7.152E-01
Left superior temporal gyrus	-0.004	0.096	[-0.193 - 0.184]	9.637E-01	9.775E-01
Right superior temporal gyrus	-0.102	0.079	[-0.256 - 0.053]	1.965E-01	6.296E-01
Left insula	-0.014	0.076	[-0.163 - 0.135]	8.541E-01	9.461E-01
Right insula	0.046	0.082	[-0.116 - 0.207]	5.787E-01	8.503E-01
Left lingual gyrus	0.159	0.061	[0.039 - 0.280]	9.526E-03	2.829E-01
Right lingual gyrus	0.035	0.061	[-0.085 - 0.156]	5.630E-01	8.503E-01
Left pars triangularis of inferior frontal gyrus	0.110	0.109	[-0.103 - 0.322]	3.123E-01	7.152E-01
Right pars triangularis of inferior frontal gyrus	0.101	0.064	[-0.025 - 0.226]	1.159E-01	6.296E-01
Left lateral orbitofrontal cortex	0.054	0.107	[-0.156 - 0.263]	6.141E-01	8.503E-01
Right lateral orbitofrontal cortex	-0.031	0.094	[-0.216 - 0.153]	7.376E-01	8.585E-01
Left rostral middle frontal gyrus	0.103	0.119	[-0.131 - 0.336]	3.883E-01	7.755E-01
Right rostral middle frontal gyrus	-0.027	0.103	[-0.228 - 0.174]	7.932E-01	8.939E-01
Left middle temporal gyrus	0.037	0.104	[-0.167 - 0.242]	7.205E-01	8.525E-01
Right middle temporal gyrus	0.007	0.082	[-0.154 - 0.168]	9.363E-01	9.695E-01
Left superior frontal gyrus	0.127	0.102	[-0.073 - 0.328]	2.128E-01	6.296E-01
Right superior frontal gyrus	0.113	0.093	[-0.070 - 0.296]	2.274E-01	6.459E-01
Left pars orbitalis of inferior frontal gyrus	-0.047	0.093	[-0.228 - 0.135]	6.155E-01	8.503E-01
Right pars orbitalis of inferior frontal gyrus	0.007	0.092	[-0.173 - 0.186]	9.422E-01	9.695E-01
Left medial orbitofrontal cortex	0.043	0.102	[-0.157 - 0.243]	6.734E-01	8.503E-01
Right medial orbitofrontal cortex	0.072	0.097	[-0.117 - 0.262]	4.540E-01	8.266E-01
Left inferior temporal gyrus	-0.045	0.092	[-0.225 - 0.134]	6.204E-01	8.503E-01
Right inferior temporal gyrus	0.015	0.100	[-0.182 - 0.211]	8.824E-01	9.492E-01
Left isthmus cingulate cortex	0.007	0.072	[-0.135 - 0.149]	9.233E-01	9.695E-01
Right isthmus cingulate cortex	-0.016	0.097	[-0.207 - 0.174]	8.662E-01	9.461E-01
Left banks of superior temporal sulcus	-0.045	0.074	[-0.190 - 0.100]	5.450E-01	8.503E-01
Right banks of superior temporal sulcus	0.038	0.097	[-0.152 - 0.228]	6.945E-01	8.503E-01
Left supramarginal gyrus	0.061	0.095	[-0.124 - 0.247]	5.175E-01	8.503E-01
Right supramarginal gyrus	0.038	0.100	[-0.159 - 0.234]	7.054E-01	8.503E-01
Left caudal middle frontal gyrus	0.067	0.105	[-0.139 - 0.273]	5.217E-01	8.503E-01
Right caudal middle frontal gyrus	0.136	0.080	[-0.020 - 0.292]	8.688E-02	5.755E-01
Left frontal pole	-0.028	0.092	[-0.209 - 0.153]	7.627E-01	8.735E-01
Right frontal pole	-0.036	0.088	[-0.208 - 0.136]	6.829E-01	8.503E-01
Left posterior cingulate cortex	0.082	0.086	[-0.086 - 0.251]	3.378E-01	7.268E-01
Right posterior cingulate cortex	0.164	0.097	[-0.027 - 0.355]	9.191E-02	5.755E-01
Left lateral occipital cortex	0.037	0.083	[-0.127 - 0.200]	6.605E-01	8.503E-01
Right lateral occipital cortex	0.092	0.088	[-0.081 - 0.265]	2.975E-01	7.152E-01
Left precentral gyrus	0.135	0.101	[-0.062 - 0.333]	1.791E-01	6.296E-01
Right precentral gyrus	0.090	0.080	[-0.067 - 0.246]	2.610E-01	6.864E-01
Left parahippocampal gyrus	-0.063	0.061	[-0.184 - 0.057]	3.015E-01	7.152E-01
Right parahippocampal gyrus	-0.051	0.070	[-0.188 - 0.086]	4.671E-01	8.290E-01
Left inferior parietal cortex	0.049	0.090	[-0.128 - 0.226]	5.896E-01	8.503E-01
Right inferior parietal cortex	0.030	0.080	[-0.127 - 0.187]	7.066E-01	8.503E-01
Left transverse temporal gyrus	0.092	0.072	[-0.050 - 0.234]	2.024E-01	6.296E-01
Right transverse temporal gyrus	-0.039	0.084	[-0.204 - 0.125]	6.382E-01	8.503E-01
Left postcentral gyrus	0.053	0.098	[-0.140 - 0.246]	5.881E-01	8.503E-01
Right postcentral gyrus	0.049	0.073	[-0.095 - 0.193]	5.019E-01	8.503E-01
Left precuneus	0.065	0.078	[-0.088 - 0.218]	4.024E-01	7.755E-01
Right precuneus	0.131	0.094	[-0.053 - 0.316]	1.630E-01	6.296E-01
Left caudal anterior cingulate cortex	0.082	0.061	[-0.038 - 0.202]	1.799E-01	6.296E-01
Right caudal anterior cingulate cortex	0.137	0.071	[-0.001 - 0.275]	5.234E-02	4.675E-01
Left cuneus	0.148	0.061	[0.028 - 0.268]	1.594E-02	2.829E-01
Right cuneus	0.119	0.061	[-0.001 - 0.239]	5.268E-02	4.675E-01
Left rostral anterior cingulate cortex	0.113	0.090	[-0.063 - 0.289]	2.086E-01	6.296E-01
Right rostral anterior cingulate cortex	-0.026	0.068	[-0.159 - 0.106]	6.956E-01	8.503E-01
Left pericalcarine cortex	0.173	0.061	[0.053 - 0.294]	4.791E-03	2.829E-01
Right pericalcarine cortex	0.122	0.071	[-0.018 - 0.262]	8.695E-02	5.755E-01
Left paracentral lobule	0.219	0.090	[0.042 - 0.396]	1.533E-02	2.829E-01
Right paracentral lobule	0.138	0.090	[-0.038 - 0.313]	1.242E-01	6.296E-01
Left superior parietal cortex	0.183	0.087	[0.012 - 0.353]	3.540E-02	4.189E-01
Right superior parietal cortex	0.151	0.091	[-0.027 - 0.330]	9.726E-02	5.755E-01
Left temporal pole	-0.051	0.061	[-0.171 - 0.069]	4.074E-01	7.755E-01
Right temporal pole	-0.116	0.087	[-0.286 - 0.054]	1.811E-01	6.296E-01
Left entorhinal cortex	-0.079	0.097	[-0.268 - 0.111]	4.148E-01	7.755E-01
Right entorhinal cortex	-0.069	0.084	[-0.234 - 0.096]	4.150E-01	7.755E-01

Supplementary Table S52. Age-by-Diagnosis interaction (MD/HC) on cortical thickness differences controlling for age and sex over 21 years of old

	d	Std. Err.	95% CI	*p*-value	FDR *q*-value
Global mean cortical thickness	0.127	0.114	[-0.096 - 0.350]	2.653E-01	6.542E-01
Left hemisphere	0.139	0.117	[-0.091 - 0.369]	2.354E-01	6.388E-01
Right hemisphere	0.104	0.108	[-0.107 - 0.316]	3.339E-01	7.409E-01
Left fusiform gyrus	-0.031	0.086	[-0.199 - 0.137]	7.169E-01	8.615E-01
Right fusiform gyrus	-0.104	0.070	[-0.241 - 0.034]	1.391E-01	5.738E-01
Left pars opercularis of inferior frontal gyrus	0.272	0.111	[0.055 - 0.489]	1.402E-02	3.764E-01
Right pars opercularis of inferior frontal gyrus	0.119	0.087	[-0.051 - 0.290]	1.707E-01	5.738E-01
Left superior temporal gyrus	0.004	0.106	[-0.203 - 0.211]	9.702E-01	9.702E-01
Right superior temporal gyrus	-0.110	0.082	[-0.270 - 0.050]	1.778E-01	5.738E-01
Left insula	-0.014	0.074	[-0.160 - 0.132]	8.508E-01	9.153E-01
Right insula	0.049	0.093	[-0.134 - 0.232]	6.017E-01	8.534E-01
Left lingual gyrus	0.140	0.063	[0.016 - 0.264]	2.650E-02	3.764E-01
Right lingual gyrus	0.028	0.063	[-0.096 - 0.151]	6.610E-01	8.534E-01
Left pars triangularis of inferior frontal gyrus	0.110	0.111	[-0.108 - 0.328]	3.222E-01	7.380E-01
Right pars triangularis of inferior frontal gyrus	0.091	0.063	[-0.033 - 0.215]	1.486E-01	5.738E-01
Left lateral orbitofrontal cortex	0.043	0.110	[-0.172 - 0.259]	6.934E-01	8.615E-01
Right lateral orbitofrontal cortex	-0.004	0.105	[-0.211 - 0.202]	9.661E-01	9.702E-01
Left rostral middle frontal gyrus	0.082	0.106	[-0.126 - 0.291]	4.372E-01	7.892E-01
Right rostral middle frontal gyrus	-0.052	0.100	[-0.248 - 0.144]	6.044E-01	8.534E-01
Left middle temporal gyrus	0.074	0.106	[-0.133 - 0.281]	4.812E-01	7.946E-01
Right middle temporal gyrus	0.030	0.085	[-0.138 - 0.197]	7.281E-01	8.615E-01
Left superior frontal gyrus	0.121	0.104	[-0.082 - 0.325]	2.429E-01	6.388E-01
Right superior frontal gyrus	0.098	0.097	[-0.092 - 0.288]	3.133E-01	7.380E-01
Left pars orbitalis of inferior frontal gyrus	-0.066	0.101	[-0.264 - 0.133]	5.174E-01	8.163E-01
Right pars orbitalis of inferior frontal gyrus	0.030	0.100	[-0.167 - 0.226]	7.673E-01	8.787E-01
Left medial orbitofrontal cortex	0.005	0.099	[-0.190 - 0.199]	9.614E-01	9.702E-01
Right medial orbitofrontal cortex	0.049	0.096	[-0.139 - 0.236]	6.118E-01	8.534E-01
Left inferior temporal gyrus	-0.036	0.095	[-0.222 - 0.150]	7.026E-01	8.615E-01
Right inferior temporal gyrus	0.022	0.105	[-0.183 - 0.227]	8.355E-01	9.153E-01
Left isthmus cingulate cortex	0.006	0.078	[-0.147 - 0.159]	9.403E-01	9.702E-01
Right isthmus cingulate cortex	-0.026	0.102	[-0.227 - 0.175]	8.001E-01	9.017E-01
Left banks of superior temporal sulcus	-0.026	0.074	[-0.170 - 0.119]	7.267E-01	8.615E-01
Right banks of superior temporal sulcus	0.006	0.092	[-0.174 - 0.187]	9.445E-01	9.702E-01
Left supramarginal gyrus	0.071	0.094	[-0.113 - 0.256]	4.491E-01	7.892E-01
Right supramarginal gyrus	0.073	0.097	[-0.117 - 0.263]	4.522E-01	7.892E-01
Left caudal middle frontal gyrus	0.077	0.113	[-0.145 - 0.299]	4.964E-01	8.011E-01
Right caudal middle frontal gyrus	0.165	0.085	[-0.001 - 0.332]	5.207E-02	5.282E-01
Left frontal pole	-0.046	0.089	[-0.221 - 0.129]	6.068E-01	8.534E-01
Right frontal pole	-0.018	0.089	[-0.192 - 0.156]	8.405E-01	9.153E-01
Left posterior cingulate cortex	0.082	0.092	[-0.097 - 0.262]	3.697E-01	7.892E-01
Right posterior cingulate cortex	0.155	0.102	[-0.045 - 0.355]	1.278E-01	5.738E-01
Left lateral occipital cortex	0.028	0.086	[-0.140 - 0.197]	7.406E-01	8.620E-01
Right lateral occipital cortex	0.061	0.082	[-0.099 - 0.221]	4.558E-01	7.892E-01
Left precentral gyrus	0.150	0.110	[-0.065 - 0.364]	1.724E-01	5.738E-01
Right precentral gyrus	0.103	0.087	[-0.068 - 0.273]	2.395E-01	6.388E-01
Left parahippocampal gyrus	-0.078	0.063	[-0.201 - 0.046]	2.185E-01	6.388E-01
Right parahippocampal gyrus	-0.058	0.075	[-0.204 - 0.088]	4.341E-01	7.892E-01
Left inferior parietal cortex	0.045	0.092	[-0.136 - 0.226]	6.276E-01	8.534E-01
Right inferior parietal cortex	0.044	0.089	[-0.130 - 0.218]	6.199E-01	8.534E-01
Left transverse temporal gyrus	0.082	0.074	[-0.063 - 0.227]	2.672E-01	6.542E-01
Right transverse temporal gyrus	-0.067	0.095	[-0.252 - 0.119]	4.805E-01	7.946E-01
Left postcentral gyrus	0.047	0.101	[-0.151 - 0.246]	6.403E-01	8.534E-01
Right postcentral gyrus	0.062	0.080	[-0.095 - 0.220]	4.375E-01	7.892E-01
Left precuneus	0.068	0.082	[-0.093 - 0.229]	4.080E-01	7.892E-01
Right precuneus	0.145	0.098	[-0.047 - 0.336]	1.391E-01	5.738E-01
Left caudal anterior cingulate cortex	0.095	0.063	[-0.029 - 0.218]	1.325E-01	5.738E-01
Right caudal anterior cingulate cortex	0.132	0.065	[0.003 - 0.260]	4.426E-02	5.237E-01
Left cuneus	0.150	0.063	[0.026 - 0.274]	1.763E-02	3.764E-01
Right cuneus	0.110	0.063	[-0.014 - 0.233]	8.254E-02	5.738E-01
Left rostral anterior cingulate cortex	0.134	0.085	[-0.032 - 0.301]	1.140E-01	5.738E-01
Right rostral anterior cingulate cortex	-0.039	0.064	[-0.165 - 0.088]	5.481E-01	8.459E-01
Left pericalcarine cortex	0.166	0.063	[0.043 - 0.290]	8.445E-03	3.764E-01
Right pericalcarine cortex	0.119	0.085	[-0.047 - 0.285]	1.596E-01	5.738E-01
Left paracentral lobule	0.222	0.100	[0.026 - 0.418]	2.624E-02	3.764E-01
Right paracentral lobule	0.121	0.095	[-0.065 - 0.308]	2.015E-01	6.221E-01
Left superior parietal cortex	0.167	0.091	[-0.011 - 0.346]	6.638E-02	5.738E-01
Right superior parietal cortex	0.157	0.100	[-0.040 - 0.353]	1.176E-01	5.738E-01
Left temporal pole	-0.028	0.063	[-0.152 - 0.095]	6.529E-01	8.534E-01
Right temporal pole	-0.127	0.085	[-0.294 - 0.040]	1.374E-01	5.738E-01
Left entorhinal cortex	-0.132	0.094	[-0.317 - 0.053]	1.610E-01	5.738E-01
Right entorhinal cortex	-0.080	0.091	[-0.259 - 0.099]	3.797E-01	7.892E-01

Supplementary Table S53. Sex-by-Diagnosis interaction (ASD/HC) on cortical thickness differences controlling for age and sex

	d	Std. Err.	95% CI	*p*-value	FDR *q*-value
Global mean cortical thickness	-0.057	0.095	[-0.242 - 0.129]	5.485E-01	9.820E-01
Left hemisphere	-0.040	0.105	[-0.246 - 0.165]	7.021E-01	9.820E-01
Right hemisphere	-0.068	0.095	[-0.253 - 0.117]	4.709E-01	9.820E-01
Left fusiform gyrus	0.071	0.095	[-0.115 - 0.257]	4.533E-01	9.820E-01
Right fusiform gyrus	0.052	0.095	[-0.133 - 0.238]	5.814E-01	9.820E-01
Left pars opercularis of inferior frontal gyrus	-0.020	0.095	[-0.206 - 0.165]	8.298E-01	9.820E-01
Right pars opercularis of inferior frontal gyrus	0.050	0.095	[-0.135 - 0.235]	5.976E-01	9.820E-01
Left superior temporal gyrus	-0.031	0.179	[-0.382 - 0.319]	8.613E-01	9.820E-01
Right superior temporal gyrus	-0.023	0.234	[-0.483 - 0.436]	9.202E-01	9.820E-01
Left insula	0.024	0.114	[-0.200 - 0.247]	8.366E-01	9.820E-01
Right insula	-0.092	0.177	[-0.438 - 0.254]	6.015E-01	9.820E-01
Left lingual gyrus	-0.023	0.168	[-0.352 - 0.307]	8.932E-01	9.820E-01
Right lingual gyrus	-0.047	0.127	[-0.296 - 0.201]	7.083E-01	9.820E-01
Left pars triangularis of inferior frontal gyrus	0.046	0.095	[-0.139 - 0.232]	6.265E-01	9.820E-01
Right pars triangularis of inferior frontal gyrus	0.205	0.095	[0.019 - 0.390]	3.042E-02	5.053E-01
Left lateral orbitofrontal cortex	-0.059	0.095	[-0.244 - 0.126]	5.320E-01	9.820E-01
Right lateral orbitofrontal cortex	-0.094	0.095	[-0.279 - 0.092]	3.218E-01	9.820E-01
Left rostral middle frontal gyrus	0.020	0.100	[-0.175 - 0.215]	8.412E-01	9.820E-01
Right rostral middle frontal gyrus	-0.061	0.103	[-0.263 - 0.141]	5.523E-01	9.820E-01
Left middle temporal gyrus	0.054	0.095	[-0.132 - 0.239]	5.704E-01	9.820E-01
Right middle temporal gyrus	0.175	0.095	[-0.011 - 0.360]	6.515E-02	7.097E-01
Left superior frontal gyrus	-0.101	0.095	[-0.286 - 0.085]	2.866E-01	9.820E-01
Right superior frontal gyrus	-0.061	0.095	[-0.247 - 0.124]	5.158E-01	9.820E-01
Left pars orbitalis of inferior frontal gyrus	0.114	0.095	[-0.072 - 0.299]	2.292E-01	9.820E-01
Right pars orbitalis of inferior frontal gyrus	-0.030	0.095	[-0.216 - 0.155]	7.476E-01	9.820E-01
Left medial orbitofrontal cortex	-0.051	0.095	[-0.236 - 0.135]	5.919E-01	9.820E-01
Right medial orbitofrontal cortex	-0.054	0.095	[-0.239 - 0.132]	5.710E-01	9.820E-01
Left inferior temporal gyrus	0.022	0.172	[-0.316 - 0.360]	8.977E-01	9.820E-01
Right inferior temporal gyrus	0.105	0.095	[-0.080 - 0.291]	2.664E-01	9.820E-01
Left isthmus cingulate cortex	0.067	0.095	[-0.118 - 0.253]	4.757E-01	9.820E-01
Right isthmus cingulate cortex	-0.004	0.161	[-0.319 - 0.311]	9.794E-01	9.854E-01
Left banks of superior temporal sulcus	0.132	0.095	[-0.053 - 0.318]	1.624E-01	9.820E-01
Right banks of superior temporal sulcus	-0.022	0.095	[-0.207 - 0.163]	8.146E-01	9.820E-01
Left supramarginal gyrus	0.066	0.095	[-0.119 - 0.251]	4.847E-01	9.820E-01
Right supramarginal gyrus	0.018	0.095	[-0.168 - 0.203]	8.507E-01	9.820E-01
Left caudal middle frontal gyrus	0.050	0.108	[-0.162 - 0.262]	6.455E-01	9.820E-01
Right caudal middle frontal gyrus	-0.151	0.111	[-0.368 - 0.066]	1.735E-01	9.820E-01
Left frontal pole	0.038	0.135	[-0.226 - 0.303]	7.769E-01	9.820E-01
Right frontal pole	-0.087	0.095	[-0.272 - 0.099]	3.587E-01	9.820E-01
Left posterior cingulate cortex	0.005	0.095	[-0.180 - 0.190]	9.576E-01	9.854E-01
Right posterior cingulate cortex	0.091	0.095	[-0.094 - 0.277]	3.350E-01	9.820E-01
Left lateral occipital cortex	-0.074	0.095	[-0.259 - 0.111]	4.335E-01	9.820E-01
Right lateral occipital cortex	-0.031	0.143	[-0.310 - 0.249]	8.284E-01	9.820E-01
Left precentral gyrus	-0.171	0.095	[-0.357 - 0.014]	6.997E-02	7.097E-01
Right precentral gyrus	-0.237	0.097	[-0.426 - -0.048]	1.417E-02	5.053E-01
Left parahippocampal gyrus	-0.037	0.160	[-0.351 - 0.277]	8.155E-01	9.820E-01
Right parahippocampal gyrus	0.029	0.131	[-0.228 - 0.286]	8.271E-01	9.820E-01
Left inferior parietal cortex	0.200	0.095	[0.014 - 0.386]	3.460E-02	5.053E-01
Right inferior parietal cortex	0.149	0.095	[-0.036 - 0.334]	1.151E-01	9.240E-01
Left transverse temporal gyrus	-0.084	0.095	[-0.269 - 0.101]	3.744E-01	9.820E-01
Right transverse temporal gyrus	-0.042	0.123	[-0.282 - 0.199]	7.341E-01	9.820E-01
Left postcentral gyrus	-0.112	0.095	[-0.298 - 0.073]	2.353E-01	9.820E-01
Right postcentral gyrus	-0.219	0.095	[-0.405 - -0.034]	2.064E-02	5.053E-01
Left precuneus	-0.110	0.156	[-0.415 - 0.195]	4.800E-01	9.820E-01
Right precuneus	-0.030	0.139	[-0.302 - 0.241]	8.275E-01	9.820E-01
Left caudal anterior cingulate cortex	-0.003	0.168	[-0.332 - 0.326]	9.854E-01	9.854E-01
Right caudal anterior cingulate cortex	0.090	0.097	[-0.101 - 0.282]	3.533E-01	9.820E-01
Left cuneus	-0.056	0.136	[-0.322 - 0.210]	6.806E-01	9.820E-01
Right cuneus	-0.071	0.095	[-0.257 - 0.114]	4.507E-01	9.820E-01
Left rostral anterior cingulate cortex	0.086	0.117	[-0.143 - 0.316]	4.614E-01	9.820E-01
Right rostral anterior cingulate cortex	0.012	0.095	[-0.173 - 0.198]	8.948E-01	9.820E-01
Left pericalcarine cortex	0.003	0.103	[-0.198 - 0.205]	9.729E-01	9.854E-01
Right pericalcarine cortex	0.009	0.095	[-0.177 - 0.194]	9.267E-01	9.820E-01
Left paracentral lobule	-0.145	0.095	[-0.330 - 0.041]	1.257E-01	9.240E-01
Right paracentral lobule	-0.199	0.095	[-0.385 - -0.013]	3.559E-02	5.053E-01
Left superior parietal cortex	-0.042	0.095	[-0.229 - 0.144]	6.571E-01	9.820E-01
Right superior parietal cortex	-0.162	0.107	[-0.373 - 0.048]	1.301E-01	9.240E-01
Left temporal pole	-0.029	0.124	[-0.273 - 0.214]	8.137E-01	9.820E-01
Right temporal pole	-0.126	0.130	[-0.381 - 0.129]	3.320E-01	9.820E-01
Left entorhinal cortex	0.152	0.114	[-0.073 - 0.376]	1.851E-01	9.820E-01
Right entorhinal cortex	0.012	0.119	[-0.221 - 0.246]	9.184E-01	9.820E-01

Supplementary Table S54. Age-by-Diagnosis interaction (ASD/HC) on cortical thickness differences controlling for age and sex

	d	Std. Err.	95% CI	*p*-value	FDR *q*-value
Global mean cortical thickness	-0.093	0.140	[-0.367 - 0.181]	5.047E-01	8.917E-01
Left hemisphere	-0.079	0.150	[-0.373 - 0.214]	5.959E-01	8.917E-01
Right hemisphere	-0.108	0.124	[-0.352 - 0.136]	3.868E-01	8.917E-01
Left fusiform gyrus	-0.010	0.136	[-0.277 - 0.256]	9.386E-01	9.386E-01
Right fusiform gyrus	-0.035	0.144	[-0.317 - 0.247]	8.088E-01	9.244E-01
Left pars opercularis of inferior frontal gyrus	-0.161	0.162	[-0.477 - 0.156]	3.195E-01	8.917E-01
Right pars opercularis of inferior frontal gyrus	-0.123	0.136	[-0.390 - 0.144]	3.668E-01	8.917E-01
Left superior temporal gyrus	-0.040	0.112	[-0.260 - 0.180]	7.186E-01	9.244E-01
Right superior temporal gyrus	-0.060	0.083	[-0.222 - 0.102]	4.666E-01	8.917E-01
Left insula	0.197	0.126	[-0.050 - 0.444]	1.174E-01	8.144E-01
Right insula	0.135	0.129	[-0.118 - 0.388]	2.940E-01	8.917E-01
Left lingual gyrus	-0.007	0.080	[-0.164 - 0.149]	9.267E-01	9.386E-01
Right lingual gyrus	0.022	0.080	[-0.135 - 0.179]	7.836E-01	9.244E-01
Left pars triangularis of inferior frontal gyrus	-0.070	0.110	[-0.285 - 0.146]	5.274E-01	8.917E-01
Right pars triangularis of inferior frontal gyrus	-0.051	0.151	[-0.348 - 0.245]	7.335E-01	9.244E-01
Left lateral orbitofrontal cortex	-0.092	0.150	[-0.386 - 0.202]	5.410E-01	8.917E-01
Right lateral orbitofrontal cortex	-0.024	0.106	[-0.232 - 0.184]	8.231E-01	9.244E-01
Left rostral middle frontal gyrus	-0.194	0.157	[-0.502 - 0.115]	2.188E-01	8.917E-01
Right rostral middle frontal gyrus	-0.224	0.138	[-0.494 - 0.046]	1.042E-01	8.144E-01
Left middle temporal gyrus	-0.105	0.119	[-0.339 - 0.128]	3.770E-01	8.917E-01
Right middle temporal gyrus	-0.151	0.125	[-0.395 - 0.094]	2.278E-01	8.917E-01
Left superior frontal gyrus	-0.156	0.142	[-0.434 - 0.122]	2.714E-01	8.917E-01
Right superior frontal gyrus	-0.175	0.121	[-0.413 - 0.062]	1.473E-01	8.144E-01
Left pars orbitalis of inferior frontal gyrus	-0.093	0.114	[-0.316 - 0.131]	4.170E-01	8.917E-01
Right pars orbitalis of inferior frontal gyrus	-0.041	0.107	[-0.250 - 0.168]	6.988E-01	9.244E-01
Left medial orbitofrontal cortex	-0.047	0.120	[-0.282 - 0.188]	6.941E-01	9.244E-01
Right medial orbitofrontal cortex	0.015	0.098	[-0.178 - 0.208]	8.792E-01	9.271E-01
Left inferior temporal gyrus	-0.067	0.123	[-0.307 - 0.173]	5.828E-01	8.917E-01
Right inferior temporal gyrus	-0.125	0.082	[-0.285 - 0.035]	1.244E-01	8.144E-01
Left isthmus cingulate cortex	-0.170	0.080	[-0.327 - -0.013]	3.338E-02	6.798E-01
Right isthmus cingulate cortex	-0.137	0.080	[-0.294 - 0.020]	8.728E-02	8.144E-01
Left banks of superior temporal sulcus	-0.020	0.105	[-0.226 - 0.186]	8.519E-01	9.244E-01
Right banks of superior temporal sulcus	-0.177	0.104	[-0.380 - 0.026]	8.807E-02	8.144E-01
Left supramarginal gyrus	-0.094	0.123	[-0.335 - 0.146]	4.426E-01	8.917E-01
Right supramarginal gyrus	-0.091	0.114	[-0.314 - 0.132]	4.255E-01	8.917E-01
Left caudal middle frontal gyrus	-0.087	0.162	[-0.404 - 0.230]	5.900E-01	8.917E-01
Right caudal middle frontal gyrus	-0.069	0.085	[-0.236 - 0.097]	4.151E-01	8.917E-01
Left frontal pole	-0.058	0.080	[-0.215 - 0.099]	4.659E-01	8.917E-01
Right frontal pole	-0.019	0.080	[-0.176 - 0.138]	8.130E-01	9.244E-01
Left posterior cingulate cortex	-0.140	0.130	[-0.394 - 0.114]	2.807E-01	8.917E-01
Right posterior cingulate cortex	-0.166	0.080	[-0.323 - -0.009]	3.830E-02	6.798E-01
Left lateral occipital cortex	-0.053	0.080	[-0.209 - 0.104]	5.107E-01	8.917E-01
Right lateral occipital cortex	-0.069	0.081	[-0.228 - 0.089]	3.931E-01	8.917E-01
Left precentral gyrus	-0.047	0.080	[-0.204 - 0.110]	5.538E-01	8.917E-01
Right precentral gyrus	-0.014	0.080	[-0.171 - 0.143]	8.581E-01	9.244E-01
Left parahippocampal gyrus	-0.155	0.110	[-0.371 - 0.061]	1.598E-01	8.144E-01
Right parahippocampal gyrus	-0.049	0.080	[-0.206 - 0.107]	5.363E-01	8.917E-01
Left inferior parietal cortex	-0.044	0.101	[-0.241 - 0.154]	6.637E-01	9.244E-01
Right inferior parietal cortex	-0.085	0.128	[-0.336 - 0.167]	5.087E-01	8.917E-01
Left transverse temporal gyrus	-0.013	0.089	[-0.187 - 0.162]	8.880E-01	9.271E-01
Right transverse temporal gyrus	-0.112	0.080	[-0.269 - 0.045]	1.606E-01	8.144E-01
Left postcentral gyrus	-0.030	0.080	[-0.187 - 0.126]	7.042E-01	9.244E-01
Right postcentral gyrus	-0.022	0.090	[-0.199 - 0.154]	8.039E-01	9.244E-01
Left precuneus	-0.069	0.103	[-0.270 - 0.133]	5.047E-01	8.917E-01
Right precuneus	-0.117	0.100	[-0.313 - 0.078]	2.379E-01	8.917E-01
Left caudal anterior cingulate cortex	-0.162	0.096	[-0.351 - 0.026]	9.134E-02	8.144E-01
Right caudal anterior cingulate cortex	-0.174	0.080	[-0.331 - -0.017]	2.976E-02	6.798E-01
Left cuneus	-0.016	0.092	[-0.197 - 0.164]	8.585E-01	9.244E-01
Right cuneus	-0.073	0.080	[-0.230 - 0.084]	3.596E-01	8.917E-01
Left rostral anterior cingulate cortex	-0.133	0.117	[-0.363 - 0.096]	2.543E-01	8.917E-01
Right rostral anterior cingulate cortex	-0.232	0.105	[-0.438 - -0.025]	2.788E-02	6.798E-01
Left pericalcarine cortex	-0.042	0.080	[-0.198 - 0.115]	6.028E-01	8.917E-01
Right pericalcarine cortex	-0.088	0.080	[-0.245 - 0.069]	2.704E-01	8.917E-01
Left paracentral lobule	0.033	0.089	[-0.141 - 0.207]	7.106E-01	9.244E-01
Right paracentral lobule	-0.015	0.084	[-0.179 - 0.149]	8.593E-01	9.244E-01
Left superior parietal cortex	0.064	0.142	[-0.214 - 0.342]	6.539E-01	9.244E-01
Right superior parietal cortex	-0.010	0.119	[-0.244 - 0.223]	9.305E-01	9.386E-01
Left temporal pole	-0.118	0.080	[-0.275 - 0.039]	1.409E-01	8.144E-01
Right temporal pole	0.022	0.080	[-0.135 - 0.179]	7.847E-01	9.244E-01
Left entorhinal cortex	-0.126	0.103	[-0.328 - 0.077]	2.228E-01	8.917E-01
Right entorhinal cortex	-0.070	0.088	[-0.242 - 0.103]	4.299E-01	8.917E-01

Supplementary Table S55. Sex-by-Diagnosis interaction (SZ/HC) on cortical surface area differences controlling for age and sex

	d	Std. Err.	95% CI	*p*-value	FDR *q*-value
Total cortical surface area	-0.080	0.057	[-0.191 - 0.031]	1.587E-01	6.358E-01
Left hemisphere	-0.086	0.058	[-0.199 - 0.028]	1.388E-01	6.358E-01
Right hemisphere	-0.072	0.055	[-0.180 - 0.036]	1.928E-01	6.358E-01
Left superior frontal gyrus	-0.086	0.055	[-0.194 - 0.022]	1.185E-01	6.358E-01
Right superior frontal gyrus	-0.086	0.053	[-0.191 - 0.018]	1.058E-01	6.358E-01
Left pars orbitalis of inferior frontal gyrus	-0.055	0.038	[-0.128 - 0.019]	1.471E-01	6.358E-01
Right pars orbitalis of inferior frontal gyrus	0.004	0.046	[-0.087 - 0.095]	9.304E-01	9.700E-01
Left precentral gyrus	-0.041	0.045	[-0.129 - 0.048]	3.648E-01	8.080E-01
Right precentral gyrus	-0.057	0.051	[-0.157 - 0.043]	2.656E-01	7.539E-01
Left lingual gyrus	-0.008	0.044	[-0.095 - 0.078]	8.510E-01	9.700E-01
Right lingual gyrus	-0.006	0.043	[-0.089 - 0.078]	8.962E-01	9.700E-01
Left superior temporal gyrus	-0.055	0.057	[-0.167 - 0.057]	3.394E-01	7.781E-01
Right superior temporal gyrus	-0.024	0.046	[-0.114 - 0.066]	6.024E-01	9.000E-01
Left fusiform gyrus	-0.070	0.051	[-0.169 - 0.030]	1.708E-01	6.358E-01
Right fusiform gyrus	-0.050	0.047	[-0.142 - 0.043]	2.910E-01	7.539E-01
Left inferior parietal cortex	-0.095	0.056	[-0.206 - 0.016]	9.282E-02	6.358E-01
Right inferior parietal cortex	-0.035	0.059	[-0.151 - 0.080]	5.489E-01	9.000E-01
Left lateral occipital cortex	-0.048	0.056	[-0.158 - 0.063]	3.977E-01	8.080E-01
Right lateral occipital cortex	-0.028	0.053	[-0.132 - 0.077]	6.049E-01	9.000E-01
Left rostral middle frontal gyrus	-0.040	0.049	[-0.137 - 0.057]	4.170E-01	8.225E-01
Right rostral middle frontal gyrus	-0.043	0.049	[-0.139 - 0.053]	3.786E-01	8.080E-01
Left precuneus	-0.059	0.046	[-0.150 - 0.032]	2.046E-01	6.358E-01
Right precuneus	-0.025	0.039	[-0.101 - 0.051]	5.202E-01	9.000E-01
Left inferior temporal gyrus	-0.064	0.040	[-0.143 - 0.015]	1.126E-01	6.358E-01
Right inferior temporal gyrus	-0.049	0.049	[-0.146 - 0.047]	3.161E-01	7.740E-01
Left lateral orbitofrontal cortex	-0.111	0.048	[-0.205 - -0.017]	2.123E-02	6.358E-01
Right lateral orbitofrontal cortex	-0.078	0.050	[-0.175 - 0.019]	1.160E-01	6.358E-01
Left middle temporal gyrus	-0.031	0.054	[-0.137 - 0.076]	5.693E-01	9.000E-01
Right middle temporal gyrus	-0.063	0.048	[-0.157 - 0.031]	1.915E-01	6.358E-01
Left postcentral gyrus	-0.030	0.047	[-0.122 - 0.063]	5.322E-01	9.000E-01
Right postcentral gyrus	-0.009	0.048	[-0.103 - 0.086]	8.564E-01	9.700E-01
Left medial orbitofrontal cortex	-0.074	0.038	[-0.149 - 0.001]	5.398E-02	6.358E-01
Right medial orbitofrontal cortex	-0.039	0.046	[-0.130 - 0.052]	3.983E-01	8.080E-01
Left cuneus	-0.020	0.046	[-0.110 - 0.070]	6.671E-01	9.261E-01
Right cuneus	-0.009	0.035	[-0.077 - 0.059]	7.966E-01	9.700E-01
Left pars triangularis of inferior frontal gyrus	-0.008	0.035	[-0.076 - 0.060]	8.220E-01	9.700E-01
Right pars triangularis of inferior frontal gyrus	0.004	0.035	[-0.064 - 0.072]	9.124E-01	9.700E-01
Left superior parietal cortex	-0.032	0.050	[-0.130 - 0.066]	5.218E-01	9.000E-01
Right superior parietal cortex	-0.006	0.038	[-0.081 - 0.068]	8.653E-01	9.700E-01
Left pars opercularis of inferior frontal gyrus	0.013	0.035	[-0.056 - 0.083]	7.048E-01	9.267E-01
Right pars opercularis of inferior frontal gyrus	-0.019	0.035	[-0.087 - 0.049]	5.783E-01	9.000E-01
Left supramarginal gyrus	0.030	0.043	[-0.054 - 0.114]	4.871E-01	9.000E-01
Right supramarginal gyrus	-0.019	0.041	[-0.098 - 0.061]	6.416E-01	9.110E-01
Left pericalcarine cortex	-0.004	0.037	[-0.077 - 0.069]	9.175E-01	9.700E-01
Right pericalcarine cortex	0.020	0.042	[-0.062 - 0.101]	6.327E-01	9.110E-01
Left parahippocampal gyrus	0.000	0.041	[-0.081 - 0.081]	9.972E-01	9.972E-01
Right parahippocampal gyrus	0.004	0.040	[-0.075 - 0.083]	9.207E-01	9.700E-01
Left caudal middle frontal gyrus	-0.007	0.047	[-0.099 - 0.085]	8.749E-01	9.700E-01
Right caudal middle frontal gyrus	-0.021	0.041	[-0.101 - 0.059]	6.069E-01	9.000E-01
Left transverse temporal gyrus	-0.042	0.044	[-0.127 - 0.044]	3.397E-01	7.781E-01
Right transverse temporal gyrus	-0.068	0.035	[-0.136 - 0.000]	4.906E-02	6.358E-01
Left banks of superior temporal sulcus	-0.019	0.049	[-0.115 - 0.077]	6.969E-01	9.267E-01
Right banks of superior temporal sulcus	-0.062	0.050	[-0.160 - 0.036]	2.149E-01	6.358E-01
Left caudal anterior cingulate cortex	-0.054	0.043	[-0.138 - 0.031]	2.138E-01	6.358E-01
Right caudal anterior cingulate cortex	-0.016	0.038	[-0.091 - 0.059]	6.782E-01	9.261E-01
Left rostral anterior cingulate cortex	-0.068	0.052	[-0.170 - 0.034]	1.900E-01	6.358E-01
Right rostral anterior cingulate cortex	-0.012	0.036	[-0.083 - 0.060]	7.453E-01	9.622E-01
Left posterior cingulate cortex	-0.007	0.048	[-0.101 - 0.087]	8.853E-01	9.700E-01
Right posterior cingulate cortex	0.006	0.041	[-0.075 - 0.086]	8.926E-01	9.700E-01
Left frontal pole	0.059	0.039	[-0.018 - 0.135]	1.320E-01	6.358E-01
Right frontal pole	-0.004	0.056	[-0.113 - 0.105]	9.427E-01	9.700E-01
Left paracentral lobule	-0.020	0.040	[-0.098 - 0.057]	6.084E-01	9.000E-01
Right paracentral lobule	-0.053	0.051	[-0.152 - 0.046]	2.973E-01	7.539E-01
Left insula	-0.029	0.053	[-0.133 - 0.074]	5.792E-01	9.000E-01
Right insula	-0.002	0.039	[-0.079 - 0.075]	9.639E-01	9.777E-01
Left entorhinal cortex	-0.047	0.043	[-0.133 - 0.038]	2.762E-01	7.539E-01
Right entorhinal cortex	-0.071	0.052	[-0.174 - 0.031]	1.713E-01	6.358E-01
Left temporal pole	-0.068	0.053	[-0.173 - 0.036]	2.011E-01	6.358E-01
Right temporal pole	-0.066	0.035	[-0.135 - 0.002]	5.873E-02	6.358E-01
Left isthmus cingulate cortex	-0.087	0.050	[-0.185 - 0.011]	8.296E-02	6.358E-01
Right isthmus cingulate cortex	-0.056	0.040	[-0.134 - 0.023]	1.659E-01	6.358E-01

Supplementary Table S56. Age-by-Diagnosis interaction (SZ/HC) on cortical surface area differences controlling for age and sex

	d	Std. Err.	95% CI	*p*-value	FDR *q*-value
Total cortical surface area	0.031	0.044	[-0.055 - 0.117]	4.786E-01	7.925E-01
Left hemisphere	0.028	0.044	[-0.058 - 0.114]	5.251E-01	7.925E-01
Right hemisphere	0.034	0.044	[-0.052 - 0.120]	4.335E-01	7.925E-01
Left superior frontal gyrus	0.019	0.048	[-0.074 - 0.113]	6.862E-01	7.925E-01
Right superior frontal gyrus	0.024	0.047	[-0.068 - 0.116]	6.057E-01	7.925E-01
Left pars orbitalis of inferior frontal gyrus	-0.016	0.043	[-0.100 - 0.067]	6.998E-01	7.925E-01
Right pars orbitalis of inferior frontal gyrus	-0.073	0.043	[-0.157 - 0.010]	8.441E-02	7.925E-01
Left precentral gyrus	0.018	0.042	[-0.065 - 0.100]	6.777E-01	7.925E-01
Right precentral gyrus	0.016	0.035	[-0.052 - 0.084]	6.409E-01	7.925E-01
Left lingual gyrus	0.047	0.043	[-0.037 - 0.131]	2.700E-01	7.925E-01
Right lingual gyrus	0.035	0.041	[-0.045 - 0.116]	3.913E-01	7.925E-01
Left superior temporal gyrus	0.029	0.048	[-0.066 - 0.124]	5.439E-01	7.925E-01
Right superior temporal gyrus	0.036	0.046	[-0.055 - 0.126]	4.391E-01	7.925E-01
Left fusiform gyrus	0.025	0.036	[-0.045 - 0.095]	4.781E-01	7.925E-01
Right fusiform gyrus	0.003	0.042	[-0.079 - 0.085]	9.437E-01	9.455E-01
Left inferior parietal cortex	-0.015	0.035	[-0.083 - 0.053]	6.652E-01	7.925E-01
Right inferior parietal cortex	0.026	0.045	[-0.062 - 0.115]	5.622E-01	7.925E-01
Left lateral occipital cortex	0.013	0.040	[-0.065 - 0.092]	7.401E-01	8.211E-01
Right lateral occipital cortex	0.028	0.035	[-0.040 - 0.096]	4.257E-01	7.925E-01
Left rostral middle frontal gyrus	0.031	0.053	[-0.074 - 0.135]	5.640E-01	7.925E-01
Right rostral middle frontal gyrus	0.023	0.042	[-0.059 - 0.104]	5.878E-01	7.925E-01
Left precuneus	0.020	0.039	[-0.057 - 0.097]	6.129E-01	7.925E-01
Right precuneus	0.027	0.045	[-0.060 - 0.115]	5.377E-01	7.925E-01
Left inferior temporal gyrus	-0.019	0.035	[-0.087 - 0.049]	5.919E-01	7.925E-01
Right inferior temporal gyrus	0.027	0.035	[-0.041 - 0.095]	4.309E-01	7.925E-01
Left lateral orbitofrontal cortex	0.011	0.037	[-0.062 - 0.084]	7.699E-01	8.261E-01
Right lateral orbitofrontal cortex	0.018	0.042	[-0.064 - 0.100]	6.643E-01	7.925E-01
Left middle temporal gyrus	-0.020	0.041	[-0.101 - 0.060]	6.215E-01	7.925E-01
Right middle temporal gyrus	0.020	0.035	[-0.048 - 0.088]	5.599E-01	7.925E-01
Left postcentral gyrus	0.024	0.049	[-0.072 - 0.119]	6.296E-01	7.925E-01
Right postcentral gyrus	0.045	0.047	[-0.047 - 0.138]	3.397E-01	7.925E-01
Left medial orbitofrontal cortex	0.052	0.045	[-0.036 - 0.141]	2.473E-01	7.925E-01
Right medial orbitofrontal cortex	0.046	0.043	[-0.038 - 0.130]	2.820E-01	7.925E-01
Left cuneus	0.032	0.048	[-0.062 - 0.126]	5.048E-01	7.925E-01
Right cuneus	-0.048	0.051	[-0.147 - 0.051]	3.454E-01	7.925E-01
Left pars triangularis of inferior frontal gyrus	-0.031	0.038	[-0.105 - 0.043]	4.085E-01	7.925E-01
Right pars triangularis of inferior frontal gyrus	0.013	0.043	[-0.071 - 0.097]	7.613E-01	8.261E-01
Left superior parietal cortex	0.010	0.035	[-0.058 - 0.078]	7.795E-01	8.261E-01
Right superior parietal cortex	-0.028	0.035	[-0.096 - 0.040]	4.194E-01	7.925E-01
Left pars opercularis of inferior frontal gyrus	0.014	0.035	[-0.054 - 0.082]	6.934E-01	7.925E-01
Right pars opercularis of inferior frontal gyrus	-0.028	0.035	[-0.096 - 0.040]	4.151E-01	7.925E-01
Left supramarginal gyrus	0.056	0.042	[-0.027 - 0.139]	1.860E-01	7.925E-01
Right supramarginal gyrus	0.039	0.036	[-0.031 - 0.110]	2.764E-01	7.925E-01
Left pericalcarine cortex	0.035	0.049	[-0.061 - 0.131]	4.785E-01	7.925E-01
Right pericalcarine cortex	0.038	0.047	[-0.053 - 0.129]	4.160E-01	7.925E-01
Left parahippocampal gyrus	0.031	0.054	[-0.074 - 0.136]	5.590E-01	7.925E-01
Right parahippocampal gyrus	-0.015	0.039	[-0.092 - 0.062]	7.032E-01	7.925E-01
Left caudal middle frontal gyrus	-0.014	0.035	[-0.082 - 0.054]	6.924E-01	7.925E-01
Right caudal middle frontal gyrus	0.032	0.044	[-0.054 - 0.118]	4.620E-01	7.925E-01
Left transverse temporal gyrus	0.045	0.036	[-0.026 - 0.116]	2.107E-01	7.925E-01
Right transverse temporal gyrus	-0.004	0.040	[-0.082 - 0.074]	9.206E-01	9.455E-01
Left banks of superior temporal sulcus	-0.020	0.042	[-0.102 - 0.062]	6.335E-01	7.925E-01
Right banks of superior temporal sulcus	0.024	0.035	[-0.044 - 0.092]	4.907E-01	7.925E-01
Left caudal anterior cingulate cortex	0.038	0.035	[-0.030 - 0.106]	2.738E-01	7.925E-01
Right caudal anterior cingulate cortex	0.037	0.039	[-0.040 - 0.113]	3.482E-01	7.925E-01
Left rostral anterior cingulate cortex	0.075	0.035	[0.007 - 0.143]	3.065E-02	6.863E-01
Right rostral anterior cingulate cortex	0.098	0.047	[0.006 - 0.190]	3.772E-02	6.863E-01
Left posterior cingulate cortex	0.045	0.041	[-0.035 - 0.126]	2.693E-01	7.925E-01
Right posterior cingulate cortex	0.003	0.045	[-0.085 - 0.091]	9.455E-01	9.455E-01
Left frontal pole	-0.029	0.047	[-0.122 - 0.064]	5.430E-01	7.925E-01
Right frontal pole	-0.074	0.035	[-0.142 - -0.006]	3.346E-02	6.863E-01
Left paracentral lobule	0.042	0.035	[-0.026 - 0.110]	2.287E-01	7.925E-01
Right paracentral lobule	0.072	0.035	[0.004 - 0.140]	3.867E-02	6.863E-01
Left insula	0.056	0.049	[-0.039 - 0.151]	2.508E-01	7.925E-01
Right insula	-0.010	0.042	[-0.092 - 0.072]	8.043E-01	8.398E-01
Left entorhinal cortex	0.045	0.045	[-0.042 - 0.133]	3.111E-01	7.925E-01
Right entorhinal cortex	0.083	0.049	[-0.014 - 0.179]	9.278E-02	7.925E-01
Left temporal pole	0.017	0.035	[-0.051 - 0.085]	6.202E-01	7.925E-01
Right temporal pole	0.025	0.035	[-0.043 - 0.093]	4.671E-01	7.925E-01
Left isthmus cingulate cortex	0.020	0.035	[-0.048 - 0.088]	5.687E-01	7.925E-01
Right isthmus cingulate cortex	0.020	0.046	[-0.070 - 0.110]	6.649E-01	7.925E-01

Supplementary Table S57. Sex-by-Diagnosis interaction (BD/HC) on cortical surface area differences controlling for age and sex

	d	Std. Err.	95% CI	*p*-value	FDR *q*-value
Total cortical surface area	0.020	0.124	[-0.223 - 0.263]	8.728E-01	9.626E-01
Left hemisphere	0.020	0.125	[-0.224 - 0.264]	8.728E-01	9.626E-01
Right hemisphere	0.019	0.123	[-0.222 - 0.260]	8.767E-01	9.626E-01
Left superior frontal gyrus	0.025	0.123	[-0.216 - 0.266]	8.380E-01	9.626E-01
Right superior frontal gyrus	-0.108	0.087	[-0.279 - 0.064]	2.175E-01	9.626E-01
Left pars orbitalis of inferior frontal gyrus	0.063	0.087	[-0.108 - 0.235]	4.681E-01	9.626E-01
Right pars orbitalis of inferior frontal gyrus	0.117	0.087	[-0.055 - 0.288]	1.829E-01	9.626E-01
Left precentral gyrus	0.018	0.124	[-0.224 - 0.261]	8.813E-01	9.626E-01
Right precentral gyrus	0.101	0.128	[-0.150 - 0.352]	4.296E-01	9.626E-01
Left lingual gyrus	0.045	0.087	[-0.127 - 0.216]	6.095E-01	9.626E-01
Right lingual gyrus	0.038	0.103	[-0.164 - 0.240]	7.146E-01	9.626E-01
Left superior temporal gyrus	-0.030	0.110	[-0.245 - 0.185]	7.843E-01	9.626E-01
Right superior temporal gyrus	-0.024	0.100	[-0.220 - 0.171]	8.078E-01	9.626E-01
Left fusiform gyrus	0.069	0.161	[-0.246 - 0.384]	6.693E-01	9.626E-01
Right fusiform gyrus	-0.019	0.115	[-0.244 - 0.206]	8.665E-01	9.626E-01
Left inferior parietal cortex	0.011	0.108	[-0.201 - 0.223]	9.182E-01	9.815E-01
Right inferior parietal cortex	-0.152	0.117	[-0.382 - 0.077]	1.928E-01	9.626E-01
Left lateral occipital cortex	-0.058	0.114	[-0.282 - 0.167]	6.144E-01	9.626E-01
Right lateral occipital cortex	0.005	0.105	[-0.201 - 0.211]	9.642E-01	9.892E-01
Left rostral middle frontal gyrus	-0.002	0.120	[-0.237 - 0.233]	9.869E-01	9.892E-01
Right rostral middle frontal gyrus	-0.058	0.087	[-0.230 - 0.113]	5.059E-01	9.626E-01
Left precuneus	0.093	0.112	[-0.126 - 0.312]	4.046E-01	9.626E-01
Right precuneus	0.185	0.123	[-0.055 - 0.425]	1.314E-01	9.626E-01
Left inferior temporal gyrus	-0.035	0.093	[-0.217 - 0.147]	7.090E-01	9.626E-01
Right inferior temporal gyrus	-0.037	0.100	[-0.233 - 0.160]	7.134E-01	9.626E-01
Left lateral orbitofrontal cortex	0.081	0.113	[-0.140 - 0.302]	4.722E-01	9.626E-01
Right lateral orbitofrontal cortex	0.110	0.134	[-0.152 - 0.372]	4.111E-01	9.626E-01
Left middle temporal gyrus	-0.106	0.107	[-0.315 - 0.104]	3.219E-01	9.626E-01
Right middle temporal gyrus	-0.061	0.112	[-0.281 - 0.159]	5.877E-01	9.626E-01
Left postcentral gyrus	-0.033	0.125	[-0.278 - 0.212]	7.914E-01	9.626E-01
Right postcentral gyrus	-0.073	0.118	[-0.305 - 0.158]	5.347E-01	9.626E-01
Left medial orbitofrontal cortex	-0.048	0.087	[-0.219 - 0.124]	5.845E-01	9.626E-01
Right medial orbitofrontal cortex	-0.004	0.091	[-0.183 - 0.175]	9.655E-01	9.892E-01
Left cuneus	-0.128	0.094	[-0.312 - 0.056]	1.731E-01	9.626E-01
Right cuneus	-0.043	0.133	[-0.304 - 0.217]	7.436E-01	9.626E-01
Left pars triangularis of inferior frontal gyrus	0.065	0.087	[-0.106 - 0.236]	4.587E-01	9.626E-01
Right pars triangularis of inferior frontal gyrus	0.077	0.117	[-0.152 - 0.306]	5.089E-01	9.626E-01
Left superior parietal cortex	0.028	0.095	[-0.158 - 0.214]	7.676E-01	9.626E-01
Right superior parietal cortex	0.058	0.089	[-0.118 - 0.233]	5.192E-01	9.626E-01
Left pars opercularis of inferior frontal gyrus	-0.060	0.087	[-0.232 - 0.111]	4.909E-01	9.626E-01
Right pars opercularis of inferior frontal gyrus	0.089	0.129	[-0.163 - 0.341]	4.897E-01	9.626E-01
Left supramarginal gyrus	0.029	0.089	[-0.146 - 0.205]	7.427E-01	9.626E-01
Right supramarginal gyrus	0.072	0.087	[-0.099 - 0.243]	4.114E-01	9.626E-01
Left pericalcarine cortex	-0.032	0.087	[-0.204 - 0.139]	7.115E-01	9.626E-01
Right pericalcarine cortex	0.035	0.109	[-0.179 - 0.249]	7.473E-01	9.626E-01
Left parahippocampal gyrus	-0.008	0.087	[-0.179 - 0.163]	9.262E-01	9.815E-01
Right parahippocampal gyrus	-0.093	0.087	[-0.264 - 0.078]	2.882E-01	9.626E-01
Left caudal middle frontal gyrus	-0.019	0.087	[-0.191 - 0.152]	8.262E-01	9.626E-01
Right caudal middle frontal gyrus	0.026	0.087	[-0.145 - 0.198]	7.622E-01	9.626E-01
Left transverse temporal gyrus	0.063	0.087	[-0.108 - 0.234]	4.707E-01	9.626E-01
Right transverse temporal gyrus	0.023	0.102	[-0.177 - 0.224]	8.190E-01	9.626E-01
Left banks of superior temporal sulcus	-0.168	0.088	[-0.340 - 0.003]	5.456E-02	9.626E-01
Right banks of superior temporal sulcus	-0.144	0.088	[-0.315 - 0.028]	1.009E-01	9.626E-01
Left caudal anterior cingulate cortex	-0.016	0.099	[-0.209 - 0.177]	8.686E-01	9.626E-01
Right caudal anterior cingulate cortex	0.048	0.087	[-0.123 - 0.219]	5.817E-01	9.626E-01
Left rostral anterior cingulate cortex	0.001	0.087	[-0.170 - 0.173]	9.892E-01	9.892E-01
Right rostral anterior cingulate cortex	-0.054	0.087	[-0.226 - 0.117]	5.351E-01	9.626E-01
Left posterior cingulate cortex	0.063	0.087	[-0.108 - 0.235]	4.678E-01	9.626E-01
Right posterior cingulate cortex	0.075	0.087	[-0.096 - 0.247]	3.883E-01	9.626E-01
Left frontal pole	-0.189	0.087	[-0.361 - -0.018]	3.062E-02	9.626E-01
Right frontal pole	-0.118	0.107	[-0.328 - 0.092]	2.702E-01	9.626E-01
Left paracentral lobule	-0.048	0.087	[-0.220 - 0.123]	5.791E-01	9.626E-01
Right paracentral lobule	-0.183	0.087	[-0.354 - -0.011]	3.681E-02	9.626E-01
Left insula	0.118	0.088	[-0.053 - 0.290]	1.760E-01	9.626E-01
Right insula	0.053	0.115	[-0.172 - 0.279]	6.428E-01	9.626E-01
Left entorhinal cortex	-0.066	0.106	[-0.273 - 0.141]	5.343E-01	9.626E-01
Right entorhinal cortex	-0.086	0.168	[-0.415 - 0.243]	6.092E-01	9.626E-01
Left temporal pole	-0.043	0.116	[-0.270 - 0.184]	7.093E-01	9.626E-01
Right temporal pole	-0.028	0.096	[-0.215 - 0.160]	7.709E-01	9.626E-01
Left isthmus cingulate cortex	0.033	0.168	[-0.295 - 0.362]	8.419E-01	9.626E-01
Right isthmus cingulate cortex	0.100	0.126	[-0.147 - 0.348]	4.262E-01	9.626E-01

Supplementary Table S58. Sex-by-Diagnosis interaction (BD/HC) on cortical surface area differences controlling for age, sex, age × sex, age^2, age^2 × sex, and ICV at 25 years of age or older

	d	Std. Err.	95% CI	*p*-value	FDR *q*-value
Total cortical surface area	-0.126	0.099	[-0.320 - 0.069]	2.059E-01	9.030E-01
Left hemisphere	-0.140	0.098	[-0.332 - 0.051]	1.510E-01	8.935E-01
Right hemisphere	-0.109	0.101	[-0.306 - 0.089]	2.800E-01	9.030E-01
Left superior frontal gyrus	-0.017	0.112	[-0.236 - 0.202]	8.761E-01	9.450E-01
Right superior frontal gyrus	-0.190	0.093	[-0.373 - -0.007]	4.171E-02	8.935E-01
Left pars orbitalis of inferior frontal gyrus	0.041	0.091	[-0.136 - 0.219]	6.470E-01	9.450E-01
Right pars orbitalis of inferior frontal gyrus	0.024	0.102	[-0.177 - 0.225]	8.163E-01	9.450E-01
Left precentral gyrus	-0.095	0.091	[-0.273 - 0.082]	2.928E-01	9.030E-01
Right precentral gyrus	0.059	0.091	[-0.118 - 0.236]	5.146E-01	9.030E-01
Left lingual gyrus	0.029	0.091	[-0.148 - 0.207]	7.458E-01	9.450E-01
Right lingual gyrus	0.021	0.091	[-0.156 - 0.199]	8.136E-01	9.450E-01
Left superior temporal gyrus	-0.139	0.091	[-0.316 - 0.039]	1.263E-01	8.935E-01
Right superior temporal gyrus	-0.080	0.094	[-0.264 - 0.105]	3.966E-01	9.030E-01
Left fusiform gyrus	-0.081	0.141	[-0.357 - 0.195]	5.638E-01	9.030E-01
Right fusiform gyrus	-0.106	0.121	[-0.342 - 0.131]	3.797E-01	9.030E-01
Left inferior parietal cortex	-0.026	0.091	[-0.204 - 0.151]	7.721E-01	9.450E-01
Right inferior parietal cortex	-0.159	0.098	[-0.351 - 0.032]	1.033E-01	8.935E-01
Left lateral occipital cortex	-0.086	0.107	[-0.296 - 0.124]	4.215E-01	9.030E-01
Right lateral occipital cortex	0.044	0.123	[-0.197 - 0.284]	7.228E-01	9.450E-01
Left rostral middle frontal gyrus	-0.103	0.091	[-0.281 - 0.074]	2.546E-01	9.030E-01
Right rostral middle frontal gyrus	-0.162	0.111	[-0.380 - 0.056]	1.447E-01	8.935E-01
Left precuneus	-0.007	0.111	[-0.224 - 0.210]	9.478E-01	9.753E-01
Right precuneus	0.121	0.091	[-0.056 - 0.299]	1.813E-01	9.030E-01
Left inferior temporal gyrus	-0.053	0.091	[-0.231 - 0.125]	5.588E-01	9.030E-01
Right inferior temporal gyrus	-0.072	0.091	[-0.249 - 0.105]	4.262E-01	9.030E-01
Left lateral orbitofrontal cortex	-0.027	0.091	[-0.204 - 0.151]	7.672E-01	9.450E-01
Right lateral orbitofrontal cortex	0.016	0.108	[-0.195 - 0.227]	8.785E-01	9.450E-01
Left middle temporal gyrus	-0.167	0.108	[-0.378 - 0.045]	1.219E-01	8.935E-01
Right middle temporal gyrus	-0.096	0.091	[-0.274 - 0.081]	2.886E-01	9.030E-01
Left postcentral gyrus	-0.089	0.124	[-0.331 - 0.153]	4.720E-01	9.030E-01
Right postcentral gyrus	-0.156	0.134	[-0.419 - 0.107]	2.449E-01	9.030E-01
Left medial orbitofrontal cortex	-0.062	0.090	[-0.240 - 0.115]	4.906E-01	9.030E-01
Right medial orbitofrontal cortex	-0.081	0.114	[-0.304 - 0.141]	4.747E-01	9.030E-01
Left cuneus	-0.098	0.091	[-0.278 - 0.081]	2.822E-01	9.030E-01
Right cuneus	-0.035	0.093	[-0.218 - 0.148]	7.084E-01	9.450E-01
Left pars triangularis of inferior frontal gyrus	-0.051	0.091	[-0.229 - 0.126]	5.723E-01	9.030E-01
Right pars triangularis of inferior frontal gyrus	-0.053	0.091	[-0.230 - 0.125]	5.598E-01	9.030E-01
Left superior parietal cortex	-0.017	0.090	[-0.194 - 0.161]	8.551E-01	9.450E-01
Right superior parietal cortex	-0.018	0.091	[-0.196 - 0.159]	8.408E-01	9.450E-01
Left pars opercularis of inferior frontal gyrus	-0.130	0.091	[-0.308 - 0.047]	1.506E-01	8.935E-01
Right pars opercularis of inferior frontal gyrus	0.031	0.107	[-0.179 - 0.242]	7.711E-01	9.450E-01
Left supramarginal gyrus	-0.025	0.111	[-0.242 - 0.193]	8.226E-01	9.450E-01
Right supramarginal gyrus	0.032	0.090	[-0.146 - 0.209]	7.273E-01	9.450E-01
Left pericalcarine cortex	-0.004	0.090	[-0.181 - 0.173]	9.646E-01	9.784E-01
Right pericalcarine cortex	0.063	0.091	[-0.115 - 0.240]	4.894E-01	9.030E-01
Left parahippocampal gyrus	-0.002	0.090	[-0.179 - 0.176]	9.849E-01	9.849E-01
Right parahippocampal gyrus	-0.087	0.091	[-0.265 - 0.090]	3.359E-01	9.030E-01
Left caudal middle frontal gyrus	-0.011	0.091	[-0.188 - 0.167]	9.058E-01	9.500E-01
Right caudal middle frontal gyrus	-0.048	0.090	[-0.225 - 0.129]	5.969E-01	9.213E-01
Left transverse temporal gyrus	0.056	0.090	[-0.121 - 0.233]	5.353E-01	9.030E-01
Right transverse temporal gyrus	0.020	0.106	[-0.188 - 0.228]	8.507E-01	9.450E-01
Left banks of superior temporal sulcus	-0.196	0.111	[-0.414 - 0.022]	7.849E-02	8.935E-01
Right banks of superior temporal sulcus	-0.179	0.096	[-0.368 - 0.010]	6.296E-02	8.935E-01
Left caudal anterior cingulate cortex	-0.061	0.091	[-0.239 - 0.116]	4.988E-01	9.030E-01
Right caudal anterior cingulate cortex	0.056	0.091	[-0.121 - 0.233]	5.366E-01	9.030E-01
Left rostral anterior cingulate cortex	-0.058	0.091	[-0.235 - 0.120]	5.242E-01	9.030E-01
Right rostral anterior cingulate cortex	-0.062	0.107	[-0.271 - 0.147]	5.601E-01	9.030E-01
Left posterior cingulate cortex	0.068	0.090	[-0.109 - 0.245]	4.538E-01	9.030E-01
Right posterior cingulate cortex	0.098	0.091	[-0.080 - 0.275]	2.811E-01	9.030E-01
Left frontal pole	-0.245	0.091	[-0.423 - -0.068]	6.841E-03	4.857E-01
Right frontal pole	-0.196	0.113	[-0.417 - 0.024]	8.135E-02	8.935E-01
Left paracentral lobule	-0.113	0.117	[-0.342 - 0.117]	3.351E-01	9.030E-01
Right paracentral lobule	-0.166	0.091	[-0.343 - 0.012]	6.766E-02	8.935E-01
Left insula	0.036	0.091	[-0.142 - 0.213]	6.938E-01	9.450E-01
Right insula	0.018	0.156	[-0.289 - 0.324]	9.099E-01	9.500E-01
Left entorhinal cortex	-0.088	0.099	[-0.283 - 0.107]	3.754E-01	9.030E-01
Right entorhinal cortex	-0.156	0.159	[-0.467 - 0.155]	3.262E-01	9.030E-01
Left temporal pole	-0.082	0.097	[-0.272 - 0.108]	3.993E-01	9.030E-01
Right temporal pole	-0.037	0.091	[-0.214 - 0.140]	6.830E-01	9.450E-01
Left isthmus cingulate cortex	-0.041	0.153	[-0.341 - 0.259]	7.896E-01	9.450E-01
Right isthmus cingulate cortex	0.040	0.090	[-0.137 - 0.217]	6.576E-01	9.450E-01

Supplementary Table S59. Sex-by-Diagnosis interaction (BD/HC) on cortical surface area differences controlling for age and sex at 25 years of age or older

	d	Std. Err.	95% CI	*p*-value	FDR *q*-value
Total cortical surface area	0.030	0.132	[-0.228 - 0.289]	8.186E-01	9.863E-01
Left hemisphere	0.029	0.133	[-0.232 - 0.291]	8.261E-01	9.863E-01
Right hemisphere	0.030	0.130	[-0.225 - 0.286]	8.151E-01	9.863E-01
Left superior frontal gyrus	0.042	0.135	[-0.222 - 0.307]	7.533E-01	9.863E-01
Right superior frontal gyrus	-0.103	0.091	[-0.281 - 0.074]	2.543E-01	9.863E-01
Left pars orbitalis of inferior frontal gyrus	0.058	0.091	[-0.119 - 0.236]	5.206E-01	9.863E-01
Right pars orbitalis of inferior frontal gyrus	0.086	0.091	[-0.091 - 0.264]	3.408E-01	9.863E-01
Left precentral gyrus	0.041	0.140	[-0.233 - 0.315]	7.700E-01	9.863E-01
Right precentral gyrus	0.136	0.146	[-0.151 - 0.423]	3.529E-01	9.863E-01
Left lingual gyrus	0.044	0.091	[-0.133 - 0.222]	6.265E-01	9.863E-01
Right lingual gyrus	0.070	0.116	[-0.157 - 0.298]	5.450E-01	9.863E-01
Left superior temporal gyrus	-0.049	0.111	[-0.267 - 0.168]	6.580E-01	9.863E-01
Right superior temporal gyrus	-0.048	0.091	[-0.226 - 0.129]	5.935E-01	9.863E-01
Left fusiform gyrus	0.032	0.156	[-0.273 - 0.337]	8.366E-01	9.863E-01
Right fusiform gyrus	-0.047	0.119	[-0.280 - 0.186]	6.933E-01	9.863E-01
Left inferior parietal cortex	0.052	0.121	[-0.186 - 0.290]	6.678E-01	9.863E-01
Right inferior parietal cortex	-0.119	0.132	[-0.379 - 0.140]	3.677E-01	9.863E-01
Left lateral occipital cortex	-0.040	0.120	[-0.275 - 0.196]	7.420E-01	9.863E-01
Right lateral occipital cortex	0.029	0.113	[-0.192 - 0.250]	7.973E-01	9.863E-01
Left rostral middle frontal gyrus	-0.009	0.112	[-0.229 - 0.211]	9.364E-01	9.863E-01
Right rostral middle frontal gyrus	-0.071	0.091	[-0.249 - 0.107]	4.331E-01	9.863E-01
Left precuneus	0.063	0.111	[-0.154 - 0.281]	5.670E-01	9.863E-01
Right precuneus	0.177	0.132	[-0.082 - 0.437]	1.804E-01	9.863E-01
Left inferior temporal gyrus	-0.015	0.098	[-0.207 - 0.176]	8.748E-01	9.863E-01
Right inferior temporal gyrus	-0.008	0.118	[-0.239 - 0.224]	9.487E-01	9.863E-01
Left lateral orbitofrontal cortex	0.083	0.129	[-0.171 - 0.336]	5.227E-01	9.863E-01
Right lateral orbitofrontal cortex	0.114	0.149	[-0.179 - 0.407]	4.447E-01	9.863E-01
Left middle temporal gyrus	-0.055	0.127	[-0.303 - 0.193]	6.642E-01	9.863E-01
Right middle temporal gyrus	-0.007	0.118	[-0.239 - 0.225]	9.530E-01	9.863E-01
Left postcentral gyrus	-0.004	0.142	[-0.283 - 0.275]	9.778E-01	9.863E-01
Right postcentral gyrus	-0.065	0.131	[-0.321 - 0.192]	6.221E-01	9.863E-01
Left medial orbitofrontal cortex	-0.076	0.091	[-0.253 - 0.102]	4.022E-01	9.863E-01
Right medial orbitofrontal cortex	-0.037	0.091	[-0.214 - 0.141]	6.864E-01	9.863E-01
Left cuneus	-0.114	0.105	[-0.320 - 0.091]	2.746E-01	9.863E-01
Right cuneus	-0.015	0.133	[-0.276 - 0.246]	9.082E-01	9.863E-01
Left pars triangularis of inferior frontal gyrus	0.048	0.090	[-0.130 - 0.225]	5.991E-01	9.863E-01
Right pars triangularis of inferior frontal gyrus	0.024	0.107	[-0.185 - 0.234]	8.220E-01	9.863E-01
Left superior parietal cortex	-0.002	0.106	[-0.209 - 0.206]	9.863E-01	9.863E-01
Right superior parietal cortex	0.033	0.091	[-0.144 - 0.210]	7.152E-01	9.863E-01
Left pars opercularis of inferior frontal gyrus	-0.109	0.091	[-0.287 - 0.068]	2.273E-01	9.863E-01
Right pars opercularis of inferior frontal gyrus	0.071	0.128	[-0.180 - 0.321]	5.808E-01	9.863E-01
Left supramarginal gyrus	0.008	0.107	[-0.202 - 0.218]	9.430E-01	9.863E-01
Right supramarginal gyrus	0.074	0.090	[-0.104 - 0.251]	4.153E-01	9.863E-01
Left pericalcarine cortex	-0.028	0.091	[-0.205 - 0.149]	7.566E-01	9.863E-01
Right pericalcarine cortex	0.047	0.106	[-0.160 - 0.254]	6.543E-01	9.863E-01
Left parahippocampal gyrus	-0.009	0.091	[-0.187 - 0.168]	9.188E-01	9.863E-01
Right parahippocampal gyrus	-0.095	0.091	[-0.273 - 0.082]	2.922E-01	9.863E-01
Left caudal middle frontal gyrus	0.015	0.091	[-0.163 - 0.192]	8.720E-01	9.863E-01
Right caudal middle frontal gyrus	0.036	0.090	[-0.141 - 0.213]	6.919E-01	9.863E-01
Left transverse temporal gyrus	0.038	0.090	[-0.140 - 0.215]	6.763E-01	9.863E-01
Right transverse temporal gyrus	-0.005	0.100	[-0.200 - 0.191]	9.632E-01	9.863E-01
Left banks of superior temporal sulcus	-0.120	0.091	[-0.298 - 0.057]	1.847E-01	9.863E-01
Right banks of superior temporal sulcus	-0.137	0.091	[-0.314 - 0.041]	1.322E-01	9.863E-01
Left caudal anterior cingulate cortex	-0.027	0.101	[-0.226 - 0.171]	7.862E-01	9.863E-01
Right caudal anterior cingulate cortex	0.051	0.091	[-0.127 - 0.228]	5.740E-01	9.863E-01
Left rostral anterior cingulate cortex	0.010	0.091	[-0.168 - 0.187]	9.160E-01	9.863E-01
Right rostral anterior cingulate cortex	-0.049	0.091	[-0.226 - 0.128]	5.882E-01	9.863E-01
Left posterior cingulate cortex	0.111	0.090	[-0.066 - 0.288]	2.195E-01	9.863E-01
Right posterior cingulate cortex	0.081	0.092	[-0.098 - 0.261]	3.742E-01	9.863E-01
Left frontal pole	-0.212	0.091	[-0.390 - -0.034]	1.936E-02	9.863E-01
Right frontal pole	-0.137	0.116	[-0.364 - 0.090]	2.355E-01	9.863E-01
Left paracentral lobule	-0.057	0.090	[-0.235 - 0.120]	5.258E-01	9.863E-01
Right paracentral lobule	-0.178	0.091	[-0.356 - -0.001]	4.920E-02	9.863E-01
Left insula	0.092	0.094	[-0.093 - 0.277]	3.287E-01	9.863E-01
Right insula	0.033	0.122	[-0.206 - 0.271]	7.888E-01	9.863E-01
Left entorhinal cortex	-0.100	0.113	[-0.321 - 0.120]	3.721E-01	9.863E-01
Right entorhinal cortex	-0.068	0.176	[-0.413 - 0.277]	6.996E-01	9.863E-01
Left temporal pole	-0.067	0.132	[-0.326 - 0.193]	6.150E-01	9.863E-01
Right temporal pole	-0.029	0.098	[-0.222 - 0.164]	7.682E-01	9.863E-01
Left isthmus cingulate cortex	0.039	0.178	[-0.309 - 0.387]	8.246E-01	9.863E-01
Right isthmus cingulate cortex	0.083	0.121	[-0.154 - 0.321]	4.919E-01	9.863E-01

Supplementary Table S60. Age-by-Diagnosis interaction (BD/HC) on cortical surface area differences controlling for age and sex

	d	Std. Err.	95% CI	*p*-value	FDR *q*-value
Total cortical surface area	-0.029	0.090	[-0.205 - 0.147]	7.467E-01	9.446E-01
Left hemisphere	-0.019	0.093	[-0.201 - 0.163]	8.386E-01	9.534E-01
Right hemisphere	-0.041	0.086	[-0.210 - 0.128]	6.349E-01	9.446E-01
Left superior frontal gyrus	-0.085	0.085	[-0.252 - 0.082]	3.190E-01	9.446E-01
Right superior frontal gyrus	-0.095	0.089	[-0.270 - 0.080]	2.862E-01	9.446E-01
Left pars orbitalis of inferior frontal gyrus	0.040	0.111	[-0.178 - 0.259]	7.183E-01	9.446E-01
Right pars orbitalis of inferior frontal gyrus	-0.087	0.085	[-0.254 - 0.080]	3.085E-01	9.446E-01
Left precentral gyrus	-0.176	0.085	[-0.343 - -0.009]	3.852E-02	9.446E-01
Right precentral gyrus	-0.069	0.085	[-0.236 - 0.098]	4.167E-01	9.446E-01
Left lingual gyrus	-0.032	0.112	[-0.251 - 0.187]	7.753E-01	9.490E-01
Right lingual gyrus	-0.034	0.085	[-0.201 - 0.133]	6.923E-01	9.446E-01
Left superior temporal gyrus	-0.029	0.121	[-0.266 - 0.207]	8.090E-01	9.534E-01
Right superior temporal gyrus	-0.051	0.099	[-0.245 - 0.143]	6.072E-01	9.446E-01
Left fusiform gyrus	0.041	0.096	[-0.147 - 0.230]	6.673E-01	9.446E-01
Right fusiform gyrus	-0.041	0.085	[-0.208 - 0.126]	6.305E-01	9.446E-01
Left inferior parietal cortex	-0.048	0.085	[-0.215 - 0.119]	5.727E-01	9.446E-01
Right inferior parietal cortex	-0.005	0.108	[-0.216 - 0.206]	9.624E-01	9.903E-01
Left lateral occipital cortex	-0.028	0.088	[-0.200 - 0.145]	7.544E-01	9.446E-01
Right lateral occipital cortex	-0.022	0.109	[-0.236 - 0.191]	8.377E-01	9.534E-01
Left rostral middle frontal gyrus	0.040	0.115	[-0.187 - 0.266]	7.302E-01	9.446E-01
Right rostral middle frontal gyrus	0.065	0.114	[-0.157 - 0.288]	5.643E-01	9.446E-01
Left precuneus	-0.006	0.085	[-0.173 - 0.161]	9.415E-01	9.903E-01
Right precuneus	0.049	0.085	[-0.118 - 0.216]	5.680E-01	9.446E-01
Left inferior temporal gyrus	-0.051	0.085	[-0.218 - 0.116]	5.491E-01	9.446E-01
Right inferior temporal gyrus	-0.019	0.085	[-0.186 - 0.148]	8.213E-01	9.534E-01
Left lateral orbitofrontal cortex	0.138	0.085	[-0.029 - 0.305]	1.060E-01	9.446E-01
Right lateral orbitofrontal cortex	0.047	0.085	[-0.119 - 0.214]	5.769E-01	9.446E-01
Left middle temporal gyrus	-0.086	0.101	[-0.284 - 0.112]	3.959E-01	9.446E-01
Right middle temporal gyrus	-0.146	0.091	[-0.325 - 0.033]	1.099E-01	9.446E-01
Left postcentral gyrus	-0.055	0.085	[-0.222 - 0.112]	5.183E-01	9.446E-01
Right postcentral gyrus	-0.093	0.085	[-0.260 - 0.074]	2.759E-01	9.446E-01
Left medial orbitofrontal cortex	-0.095	0.087	[-0.265 - 0.075]	2.742E-01	9.446E-01
Right medial orbitofrontal cortex	0.000	0.085	[-0.167 - 0.166]	9.972E-01	9.972E-01
Left cuneus	-0.027	0.089	[-0.202 - 0.147]	7.575E-01	9.446E-01
Right cuneus	-0.047	0.085	[-0.214 - 0.120]	5.823E-01	9.446E-01
Left pars triangularis of inferior frontal gyrus	-0.054	0.088	[-0.226 - 0.119]	5.417E-01	9.446E-01
Right pars triangularis of inferior frontal gyrus	-0.080	0.101	[-0.278 - 0.117]	4.243E-01	9.446E-01
Left superior parietal cortex	-0.016	0.099	[-0.210 - 0.179]	8.732E-01	9.534E-01
Right superior parietal cortex	-0.058	0.085	[-0.225 - 0.109]	4.943E-01	9.446E-01
Left pars opercularis of inferior frontal gyrus	0.098	0.138	[-0.173 - 0.369]	4.787E-01	9.446E-01
Right pars opercularis of inferior frontal gyrus	-0.002	0.085	[-0.169 - 0.165]	9.785E-01	9.925E-01
Left supramarginal gyrus	0.097	0.112	[-0.123 - 0.316]	3.882E-01	9.446E-01
Right supramarginal gyrus	0.059	0.085	[-0.108 - 0.226]	4.902E-01	9.446E-01
Left pericalcarine cortex	-0.036	0.085	[-0.203 - 0.131]	6.734E-01	9.446E-01
Right pericalcarine cortex	-0.039	0.085	[-0.206 - 0.128]	6.485E-01	9.446E-01
Left parahippocampal gyrus	-0.074	0.141	[-0.350 - 0.202]	5.996E-01	9.446E-01
Right parahippocampal gyrus	-0.131	0.105	[-0.337 - 0.075]	2.115E-01	9.446E-01
Left caudal middle frontal gyrus	-0.058	0.085	[-0.225 - 0.109]	4.968E-01	9.446E-01
Right caudal middle frontal gyrus	-0.133	0.123	[-0.374 - 0.107]	2.772E-01	9.446E-01
Left transverse temporal gyrus	0.093	0.126	[-0.154 - 0.341]	4.603E-01	9.446E-01
Right transverse temporal gyrus	0.095	0.085	[-0.072 - 0.262]	2.671E-01	9.446E-01
Left banks of superior temporal sulcus	-0.014	0.085	[-0.180 - 0.153]	8.733E-01	9.534E-01
Right banks of superior temporal sulcus	-0.077	0.093	[-0.260 - 0.106]	4.100E-01	9.446E-01
Left caudal anterior cingulate cortex	0.012	0.085	[-0.155 - 0.179]	8.863E-01	9.534E-01
Right caudal anterior cingulate cortex	0.067	0.085	[-0.100 - 0.234]	4.294E-01	9.446E-01
Left rostral anterior cingulate cortex	0.092	0.115	[-0.133 - 0.317]	4.216E-01	9.446E-01
Right rostral anterior cingulate cortex	0.054	0.085	[-0.113 - 0.221]	5.282E-01	9.446E-01
Left posterior cingulate cortex	-0.013	0.085	[-0.179 - 0.154]	8.813E-01	9.534E-01
Right posterior cingulate cortex	0.005	0.085	[-0.162 - 0.172]	9.548E-01	9.903E-01
Left frontal pole	-0.052	0.085	[-0.218 - 0.115]	5.447E-01	9.446E-01
Right frontal pole	-0.071	0.121	[-0.309 - 0.166]	5.550E-01	9.446E-01
Left paracentral lobule	-0.026	0.085	[-0.193 - 0.141]	7.583E-01	9.446E-01
Right paracentral lobule	0.053	0.085	[-0.113 - 0.220]	5.302E-01	9.446E-01
Left insula	0.053	0.085	[-0.114 - 0.220]	5.327E-01	9.446E-01
Right insula	-0.033	0.095	[-0.220 - 0.154]	7.267E-01	9.446E-01
Left entorhinal cortex	0.117	0.085	[-0.050 - 0.284]	1.691E-01	9.446E-01
Right entorhinal cortex	0.184	0.141	[-0.093 - 0.462]	1.927E-01	9.446E-01
Left temporal pole	0.049	0.114	[-0.174 - 0.273]	6.648E-01	9.446E-01
Right temporal pole	-0.062	0.127	[-0.312 - 0.188]	6.258E-01	9.446E-01
Left isthmus cingulate cortex	0.058	0.085	[-0.109 - 0.225]	4.983E-01	9.446E-01
Right isthmus cingulate cortex	-0.057	0.106	[-0.264 - 0.149]	5.863E-01	9.446E-01

Supplementary Table S61. Age-by-Diagnosis interaction (BD/HC) on cortical surface area differences controlling for age, sex, age × sex, age^2, age^2 × sex, and ICV at 25 years of age or older

	d	Std. Err.	95% CI	*p*-value	FDR *q*-value
Total cortical surface area	-0.052	0.088	[-0.224 - 0.121]	5.576E-01	9.566E-01
Left hemisphere	-0.042	0.088	[-0.215 - 0.130]	6.311E-01	9.566E-01
Right hemisphere	-0.062	0.088	[-0.234 - 0.110]	4.812E-01	9.566E-01
Left superior frontal gyrus	-0.149	0.088	[-0.321 - 0.024]	9.136E-02	9.566E-01
Right superior frontal gyrus	-0.108	0.088	[-0.280 - 0.064]	2.197E-01	9.566E-01
Left pars orbitalis of inferior frontal gyrus	0.103	0.126	[-0.144 - 0.350]	4.158E-01	9.566E-01
Right pars orbitalis of inferior frontal gyrus	-0.096	0.088	[-0.268 - 0.076]	2.749E-01	9.566E-01
Left precentral gyrus	-0.131	0.088	[-0.303 - 0.042]	1.376E-01	9.566E-01
Right precentral gyrus	-0.032	0.088	[-0.204 - 0.140]	7.153E-01	9.566E-01
Left lingual gyrus	-0.065	0.091	[-0.243 - 0.113]	4.765E-01	9.566E-01
Right lingual gyrus	-0.050	0.098	[-0.242 - 0.141]	6.052E-01	9.566E-01
Left superior temporal gyrus	-0.033	0.105	[-0.238 - 0.172]	7.545E-01	9.566E-01
Right superior temporal gyrus	-0.134	0.088	[-0.307 - 0.039]	1.279E-01	9.566E-01
Left fusiform gyrus	0.024	0.088	[-0.148 - 0.196]	7.851E-01	9.567E-01
Right fusiform gyrus	-0.122	0.127	[-0.371 - 0.126]	3.347E-01	9.566E-01
Left inferior parietal cortex	-0.074	0.088	[-0.246 - 0.099]	4.016E-01	9.566E-01
Right inferior parietal cortex	-0.032	0.088	[-0.204 - 0.140]	7.161E-01	9.566E-01
Left lateral occipital cortex	0.032	0.100	[-0.164 - 0.228]	7.490E-01	9.566E-01
Right lateral occipital cortex	0.039	0.112	[-0.180 - 0.258]	7.293E-01	9.566E-01
Left rostral middle frontal gyrus	-0.056	0.105	[-0.262 - 0.150]	5.942E-01	9.566E-01
Right rostral middle frontal gyrus	-0.034	0.098	[-0.226 - 0.157]	7.249E-01	9.566E-01
Left precuneus	0.033	0.088	[-0.140 - 0.205]	7.113E-01	9.566E-01
Right precuneus	0.022	0.088	[-0.150 - 0.194]	8.019E-01	9.567E-01
Left inferior temporal gyrus	-0.157	0.088	[-0.330 - 0.016]	7.457E-02	9.566E-01
Right inferior temporal gyrus	0.005	0.094	[-0.179 - 0.190]	9.541E-01	9.797E-01
Left lateral orbitofrontal cortex	0.152	0.088	[-0.020 - 0.325]	8.349E-02	9.566E-01
Right lateral orbitofrontal cortex	-0.006	0.088	[-0.178 - 0.167]	9.488E-01	9.797E-01
Left middle temporal gyrus	-0.120	0.107	[-0.330 - 0.091]	2.650E-01	9.566E-01
Right middle temporal gyrus	-0.087	0.088	[-0.259 - 0.085]	3.229E-01	9.566E-01
Left postcentral gyrus	-0.059	0.088	[-0.231 - 0.114]	5.035E-01	9.566E-01
Right postcentral gyrus	-0.111	0.088	[-0.284 - 0.061]	2.064E-01	9.566E-01
Left medial orbitofrontal cortex	-0.021	0.119	[-0.254 - 0.212]	8.624E-01	9.567E-01
Right medial orbitofrontal cortex	-0.039	0.088	[-0.212 - 0.133]	6.549E-01	9.566E-01
Left cuneus	-0.023	0.092	[-0.203 - 0.156]	7.981E-01	9.567E-01
Right cuneus	0.044	0.099	[-0.149 - 0.238]	6.532E-01	9.566E-01
Left pars triangularis of inferior frontal gyrus	-0.020	0.103	[-0.221 - 0.182]	8.488E-01	9.567E-01
Right pars triangularis of inferior frontal gyrus	-0.116	0.088	[-0.288 - 0.057]	1.895E-01	9.566E-01
Left superior parietal cortex	-0.045	0.088	[-0.217 - 0.128]	6.106E-01	9.566E-01
Right superior parietal cortex	-0.007	0.088	[-0.179 - 0.166]	9.400E-01	9.797E-01
Left pars opercularis of inferior frontal gyrus	0.041	0.112	[-0.179 - 0.261]	7.142E-01	9.566E-01
Right pars opercularis of inferior frontal gyrus	0.003	0.102	[-0.196 - 0.202]	9.797E-01	9.797E-01
Left supramarginal gyrus	0.045	0.102	[-0.154 - 0.244]	6.561E-01	9.566E-01
Right supramarginal gyrus	0.007	0.088	[-0.165 - 0.180]	9.337E-01	9.797E-01
Left pericalcarine cortex	-0.020	0.088	[-0.192 - 0.153]	8.234E-01	9.567E-01
Right pericalcarine cortex	0.030	0.097	[-0.159 - 0.220]	7.524E-01	9.566E-01
Left parahippocampal gyrus	-0.050	0.088	[-0.223 - 0.122]	5.682E-01	9.566E-01
Right parahippocampal gyrus	-0.143	0.088	[-0.316 - 0.029]	1.037E-01	9.566E-01
Left caudal middle frontal gyrus	0.003	0.088	[-0.170 - 0.175]	9.751E-01	9.797E-01
Right caudal middle frontal gyrus	-0.162	0.110	[-0.377 - 0.053]	1.390E-01	9.566E-01
Left transverse temporal gyrus	0.087	0.125	[-0.157 - 0.331]	4.839E-01	9.566E-01
Right transverse temporal gyrus	0.059	0.100	[-0.136 - 0.255]	5.529E-01	9.566E-01
Left banks of superior temporal sulcus	-0.035	0.088	[-0.207 - 0.138]	6.934E-01	9.566E-01
Right banks of superior temporal sulcus	-0.067	0.090	[-0.244 - 0.110]	4.569E-01	9.566E-01
Left caudal anterior cingulate cortex	-0.041	0.088	[-0.213 - 0.132]	6.430E-01	9.566E-01
Right caudal anterior cingulate cortex	0.078	0.100	[-0.118 - 0.275]	4.358E-01	9.566E-01
Left rostral anterior cingulate cortex	0.079	0.101	[-0.118 - 0.277]	4.318E-01	9.566E-01
Right rostral anterior cingulate cortex	0.107	0.114	[-0.116 - 0.330]	3.478E-01	9.566E-01
Left posterior cingulate cortex	-0.019	0.088	[-0.192 - 0.153]	8.250E-01	9.567E-01
Right posterior cingulate cortex	-0.050	0.092	[-0.231 - 0.131]	5.895E-01	9.566E-01
Left frontal pole	-0.127	0.090	[-0.304 - 0.050]	1.601E-01	9.566E-01
Right frontal pole	-0.074	0.145	[-0.358 - 0.210]	6.089E-01	9.566E-01
Left paracentral lobule	0.012	0.088	[-0.161 - 0.184]	8.922E-01	9.746E-01
Right paracentral lobule	0.036	0.101	[-0.162 - 0.234]	7.241E-01	9.566E-01
Left insula	0.086	0.088	[-0.087 - 0.258]	3.309E-01	9.566E-01
Right insula	0.050	0.096	[-0.138 - 0.239]	5.995E-01	9.566E-01
Left entorhinal cortex	0.114	0.088	[-0.059 - 0.286]	1.962E-01	9.566E-01
Right entorhinal cortex	0.113	0.147	[-0.176 - 0.402]	4.425E-01	9.566E-01
Left temporal pole	0.019	0.104	[-0.184 - 0.223]	8.513E-01	9.567E-01
Right temporal pole	-0.055	0.147	[-0.344 - 0.233]	7.061E-01	9.566E-01
Left isthmus cingulate cortex	0.171	0.095	[-0.016 - 0.357]	7.303E-02	9.566E-01
Right isthmus cingulate cortex	-0.097	0.109	[-0.311 - 0.118]	3.759E-01	9.566E-01

Supplementary Table S62. Age-by-Diagnosis interaction (BD/HC) on cortical surface area differences controlling for age and sex at 25 years of age or older

	d	Std. Err.	95% CI	*p*-value	FDR *q*-value
Total cortical surface area	-0.038	0.114	[-0.261 - 0.186]	7.417E-01	9.693E-01
Left hemisphere	-0.016	0.120	[-0.252 - 0.219]	8.909E-01	9.781E-01
Right hemisphere	-0.061	0.107	[-0.270 - 0.148]	5.657E-01	9.436E-01
Left superior frontal gyrus	-0.052	0.094	[-0.237 - 0.132]	5.775E-01	9.436E-01
Right superior frontal gyrus	-0.109	0.089	[-0.283 - 0.065]	2.208E-01	9.436E-01
Left pars orbitalis of inferior frontal gyrus	0.097	0.120	[-0.138 - 0.332]	4.192E-01	9.436E-01
Right pars orbitalis of inferior frontal gyrus	-0.115	0.096	[-0.304 - 0.073]	2.295E-01	9.436E-01
Left precentral gyrus	-0.175	0.088	[-0.348 - -0.002]	4.749E-02	9.436E-01
Right precentral gyrus	-0.064	0.107	[-0.273 - 0.146]	5.524E-01	9.436E-01
Left lingual gyrus	-0.062	0.100	[-0.257 - 0.134]	5.373E-01	9.436E-01
Right lingual gyrus	-0.081	0.101	[-0.279 - 0.117]	4.219E-01	9.436E-01
Left superior temporal gyrus	-0.053	0.111	[-0.271 - 0.165]	6.339E-01	9.576E-01
Right superior temporal gyrus	-0.072	0.088	[-0.245 - 0.101]	4.143E-01	9.436E-01
Left fusiform gyrus	0.024	0.101	[-0.174 - 0.222]	8.115E-01	9.781E-01
Right fusiform gyrus	-0.005	0.114	[-0.230 - 0.219]	9.638E-01	9.830E-01
Left inferior parietal cortex	-0.086	0.095	[-0.274 - 0.101]	3.653E-01	9.436E-01
Right inferior parietal cortex	-0.076	0.093	[-0.258 - 0.106]	4.132E-01	9.436E-01
Left lateral occipital cortex	-0.039	0.119	[-0.272 - 0.195]	7.450E-01	9.693E-01
Right lateral occipital cortex	-0.018	0.134	[-0.281 - 0.246]	8.952E-01	9.781E-01
Left rostral middle frontal gyrus	0.007	0.133	[-0.254 - 0.267]	9.609E-01	9.830E-01
Right rostral middle frontal gyrus	0.003	0.119	[-0.231 - 0.236]	9.830E-01	9.830E-01
Left precuneus	-0.055	0.088	[-0.227 - 0.118]	5.330E-01	9.436E-01
Right precuneus	-0.012	0.088	[-0.184 - 0.161]	8.955E-01	9.781E-01
Left inferior temporal gyrus	-0.098	0.088	[-0.270 - 0.075]	2.679E-01	9.436E-01
Right inferior temporal gyrus	-0.029	0.088	[-0.202 - 0.143]	7.385E-01	9.693E-01
Left lateral orbitofrontal cortex	0.190	0.127	[-0.059 - 0.439]	1.347E-01	9.436E-01
Right lateral orbitofrontal cortex	0.013	0.096	[-0.174 - 0.201]	8.904E-01	9.781E-01
Left middle temporal gyrus	-0.150	0.089	[-0.324 - 0.024]	9.064E-02	9.436E-01
Right middle temporal gyrus	-0.180	0.095	[-0.367 - 0.006]	5.841E-02	9.436E-01
Left postcentral gyrus	-0.069	0.088	[-0.241 - 0.104]	4.363E-01	9.436E-01
Right postcentral gyrus	-0.137	0.088	[-0.309 - 0.036]	1.210E-01	9.436E-01
Left medial orbitofrontal cortex	-0.084	0.091	[-0.261 - 0.094]	3.555E-01	9.436E-01
Right medial orbitofrontal cortex	-0.008	0.088	[-0.180 - 0.164]	9.277E-01	9.830E-01
Left cuneus	-0.033	0.091	[-0.212 - 0.145]	7.143E-01	9.693E-01
Right cuneus	-0.022	0.101	[-0.220 - 0.175]	8.245E-01	9.781E-01
Left pars triangularis of inferior frontal gyrus	0.039	0.131	[-0.217 - 0.295]	7.649E-01	9.698E-01
Right pars triangularis of inferior frontal gyrus	-0.153	0.088	[-0.326 - 0.020]	8.207E-02	9.436E-01
Left superior parietal cortex	-0.076	0.100	[-0.271 - 0.120]	4.478E-01	9.436E-01
Right superior parietal cortex	-0.076	0.088	[-0.248 - 0.097]	3.908E-01	9.436E-01
Left pars opercularis of inferior frontal gyrus	0.101	0.151	[-0.195 - 0.398]	5.028E-01	9.436E-01
Right pars opercularis of inferior frontal gyrus	0.003	0.089	[-0.171 - 0.177]	9.725E-01	9.830E-01
Left supramarginal gyrus	0.058	0.088	[-0.114 - 0.231]	5.079E-01	9.436E-01
Right supramarginal gyrus	0.048	0.088	[-0.125 - 0.220]	5.868E-01	9.436E-01
Left pericalcarine cortex	-0.060	0.088	[-0.232 - 0.113]	4.985E-01	9.436E-01
Right pericalcarine cortex	-0.042	0.102	[-0.241 - 0.158]	6.813E-01	9.693E-01
Left parahippocampal gyrus	-0.022	0.152	[-0.321 - 0.276]	8.827E-01	9.781E-01
Right parahippocampal gyrus	-0.174	0.099	[-0.369 - 0.021]	8.036E-02	9.436E-01
Left caudal middle frontal gyrus	-0.028	0.088	[-0.201 - 0.145]	7.509E-01	9.693E-01
Right caudal middle frontal gyrus	-0.124	0.133	[-0.384 - 0.135]	3.482E-01	9.436E-01
Left transverse temporal gyrus	0.059	0.113	[-0.163 - 0.282]	6.016E-01	9.436E-01
Right transverse temporal gyrus	0.077	0.092	[-0.104 - 0.258]	4.054E-01	9.436E-01
Left banks of superior temporal sulcus	-0.035	0.088	[-0.208 - 0.137]	6.878E-01	9.693E-01
Right banks of superior temporal sulcus	-0.123	0.092	[-0.303 - 0.058]	1.826E-01	9.436E-01
Left caudal anterior cingulate cortex	-0.054	0.088	[-0.226 - 0.119]	5.414E-01	9.436E-01
Right caudal anterior cingulate cortex	0.082	0.088	[-0.091 - 0.254]	3.548E-01	9.436E-01
Left rostral anterior cingulate cortex	0.052	0.122	[-0.186 - 0.291]	6.684E-01	9.693E-01
Right rostral anterior cingulate cortex	0.064	0.088	[-0.109 - 0.236]	4.703E-01	9.436E-01
Left posterior cingulate cortex	-0.045	0.088	[-0.217 - 0.128]	6.113E-01	9.436E-01
Right posterior cingulate cortex	-0.047	0.088	[-0.220 - 0.125]	5.893E-01	9.436E-01
Left frontal pole	-0.126	0.088	[-0.299 - 0.046]	1.506E-01	9.436E-01
Right frontal pole	-0.082	0.126	[-0.328 - 0.165]	5.167E-01	9.436E-01
Left paracentral lobule	-0.014	0.088	[-0.186 - 0.158]	8.735E-01	9.781E-01
Right paracentral lobule	0.015	0.088	[-0.157 - 0.187]	8.642E-01	9.781E-01
Left insula	0.090	0.110	[-0.125 - 0.306]	4.108E-01	9.436E-01
Right insula	-0.007	0.120	[-0.242 - 0.228]	9.528E-01	9.830E-01
Left entorhinal cortex	0.084	0.088	[-0.089 - 0.256]	3.425E-01	9.436E-01
Right entorhinal cortex	0.146	0.151	[-0.151 - 0.442]	3.360E-01	9.436E-01
Left temporal pole	0.098	0.151	[-0.199 - 0.395]	5.193E-01	9.436E-01
Right temporal pole	-0.085	0.140	[-0.360 - 0.189]	5.427E-01	9.436E-01
Left isthmus cingulate cortex	0.081	0.088	[-0.092 - 0.254]	3.581E-01	9.436E-01
Right isthmus cingulate cortex	-0.100	0.097	[-0.290 - 0.090]	3.035E-01	9.436E-01

Supplementary Table S63. Sex-by-Diagnosis interaction (MD/HC) on cortical surface area differences controlling for age and sex

	d	Std. Err.	95% CI	*p*-value	FDR *q*-value
Total cortical surface area	-0.073	0.120	[-0.308 - 0.161]	5.409E-01	9.928E-01
Left hemisphere	-0.060	0.120	[-0.295 - 0.174]	6.138E-01	9.928E-01
Right hemisphere	-0.086	0.118	[-0.318 - 0.146]	4.654E-01	9.928E-01
Left superior frontal gyrus	-0.089	0.115	[-0.314 - 0.135]	4.354E-01	9.928E-01
Right superior frontal gyrus	-0.034	0.097	[-0.225 - 0.156]	7.232E-01	9.928E-01
Left pars orbitalis of inferior frontal gyrus	-0.034	0.119	[-0.268 - 0.200]	7.745E-01	9.928E-01
Right pars orbitalis of inferior frontal gyrus	-0.036	0.079	[-0.191 - 0.118]	6.453E-01	9.928E-01
Left precentral gyrus	0.005	0.112	[-0.215 - 0.225]	9.670E-01	9.928E-01
Right precentral gyrus	0.003	0.104	[-0.200 - 0.206]	9.769E-01	9.928E-01
Left lingual gyrus	-0.047	0.074	[-0.191 - 0.097]	5.222E-01	9.928E-01
Right lingual gyrus	-0.025	0.099	[-0.218 - 0.168]	8.017E-01	9.928E-01
Left superior temporal gyrus	-0.051	0.078	[-0.205 - 0.102]	5.118E-01	9.928E-01
Right superior temporal gyrus	-0.039	0.093	[-0.222 - 0.144]	6.787E-01	9.928E-01
Left fusiform gyrus	-0.077	0.106	[-0.284 - 0.129]	4.632E-01	9.928E-01
Right fusiform gyrus	-0.128	0.096	[-0.317 - 0.060]	1.822E-01	9.928E-01
Left inferior parietal cortex	-0.101	0.102	[-0.302 - 0.099]	3.219E-01	9.928E-01
Right inferior parietal cortex	-0.150	0.107	[-0.359 - 0.059]	1.604E-01	9.928E-01
Left lateral occipital cortex	-0.026	0.100	[-0.222 - 0.170]	7.938E-01	9.928E-01
Right lateral occipital cortex	0.040	0.091	[-0.138 - 0.217]	6.627E-01	9.928E-01
Left rostral middle frontal gyrus	-0.034	0.089	[-0.208 - 0.140]	7.011E-01	9.928E-01
Right rostral middle frontal gyrus	-0.034	0.087	[-0.205 - 0.137]	6.986E-01	9.928E-01
Left precuneus	-0.038	0.095	[-0.224 - 0.148]	6.881E-01	9.928E-01
Right precuneus	-0.068	0.090	[-0.245 - 0.109]	4.525E-01	9.928E-01
Left inferior temporal gyrus	-0.080	0.102	[-0.280 - 0.120]	4.343E-01	9.928E-01
Right inferior temporal gyrus	-0.190	0.077	[-0.341 - -0.040]	1.320E-02	9.370E-01
Left lateral orbitofrontal cortex	-0.019	0.096	[-0.208 - 0.170]	8.432E-01	9.928E-01
Right lateral orbitofrontal cortex	-0.085	0.087	[-0.255 - 0.085]	3.285E-01	9.928E-01
Left middle temporal gyrus	-0.014	0.102	[-0.213 - 0.185]	8.879E-01	9.928E-01
Right middle temporal gyrus	-0.071	0.108	[-0.283 - 0.141]	5.096E-01	9.928E-01
Left postcentral gyrus	-0.099	0.101	[-0.297 - 0.099]	3.259E-01	9.928E-01
Right postcentral gyrus	-0.064	0.096	[-0.253 - 0.124]	5.040E-01	9.928E-01
Left medial orbitofrontal cortex	-0.032	0.098	[-0.224 - 0.161]	7.483E-01	9.928E-01
Right medial orbitofrontal cortex	-0.047	0.093	[-0.229 - 0.134]	6.094E-01	9.928E-01
Left cuneus	0.002	0.079	[-0.152 - 0.156]	9.788E-01	9.928E-01
Right cuneus	0.007	0.081	[-0.152 - 0.166]	9.284E-01	9.928E-01
Left pars triangularis of inferior frontal gyrus	0.099	0.080	[-0.058 - 0.256]	2.172E-01	9.928E-01
Right pars triangularis of inferior frontal gyrus	-0.065	0.071	[-0.205 - 0.075]	3.608E-01	9.928E-01
Left superior parietal cortex	0.024	0.083	[-0.138 - 0.186]	7.685E-01	9.928E-01
Right superior parietal cortex	-0.015	0.094	[-0.200 - 0.169]	8.713E-01	9.928E-01
Left pars opercularis of inferior frontal gyrus	0.070	0.086	[-0.098 - 0.238]	4.152E-01	9.928E-01
Right pars opercularis of inferior frontal gyrus	0.019	0.103	[-0.182 - 0.221]	8.508E-01	9.928E-01
Left supramarginal gyrus	0.018	0.080	[-0.139 - 0.175]	8.228E-01	9.928E-01
Right supramarginal gyrus	-0.102	0.080	[-0.259 - 0.054]	2.008E-01	9.928E-01
Left pericalcarine cortex	-0.072	0.065	[-0.199 - 0.055]	2.695E-01	9.928E-01
Right pericalcarine cortex	-0.010	0.103	[-0.211 - 0.191]	9.243E-01	9.928E-01
Left parahippocampal gyrus	-0.044	0.073	[-0.186 - 0.098]	5.443E-01	9.928E-01
Right parahippocampal gyrus	-0.014	0.061	[-0.135 - 0.106]	8.148E-01	9.928E-01
Left caudal middle frontal gyrus	-0.038	0.085	[-0.204 - 0.128]	6.541E-01	9.928E-01
Right caudal middle frontal gyrus	-0.007	0.090	[-0.182 - 0.169]	9.418E-01	9.928E-01
Left transverse temporal gyrus	-0.017	0.075	[-0.164 - 0.129]	8.160E-01	9.928E-01
Right transverse temporal gyrus	-0.050	0.086	[-0.218 - 0.118]	5.590E-01	9.928E-01
Left banks of superior temporal sulcus	-0.113	0.088	[-0.285 - 0.059]	1.977E-01	9.928E-01
Right banks of superior temporal sulcus	-0.126	0.118	[-0.357 - 0.105]	2.864E-01	9.928E-01
Left caudal anterior cingulate cortex	-0.046	0.096	[-0.234 - 0.143]	6.341E-01	9.928E-01
Right caudal anterior cingulate cortex	-0.086	0.084	[-0.250 - 0.078]	3.064E-01	9.928E-01
Left rostral anterior cingulate cortex	-0.004	0.089	[-0.179 - 0.170]	9.613E-01	9.928E-01
Right rostral anterior cingulate cortex	-0.049	0.065	[-0.177 - 0.078]	4.467E-01	9.928E-01
Left posterior cingulate cortex	-0.076	0.078	[-0.229 - 0.077]	3.295E-01	9.928E-01
Right posterior cingulate cortex	-0.034	0.082	[-0.196 - 0.127]	6.789E-01	9.928E-01
Left frontal pole	0.027	0.093	[-0.156 - 0.209]	7.761E-01	9.928E-01
Right frontal pole	-0.119	0.072	[-0.262 - 0.023]	9.930E-02	9.928E-01
Left paracentral lobule	-0.077	0.064	[-0.203 - 0.050]	2.335E-01	9.928E-01
Right paracentral lobule	-0.056	0.075	[-0.203 - 0.091]	4.540E-01	9.928E-01
Left insula	0.000	0.092	[-0.181 - 0.181]	9.964E-01	9.964E-01
Right insula	-0.056	0.071	[-0.195 - 0.083]	4.314E-01	9.928E-01
Left entorhinal cortex	-0.041	0.100	[-0.236 - 0.154]	6.813E-01	9.928E-01
Right entorhinal cortex	-0.035	0.071	[-0.174 - 0.105]	6.284E-01	9.928E-01
Left temporal pole	-0.101	0.091	[-0.280 - 0.078]	2.677E-01	9.928E-01
Right temporal pole	-0.121	0.092	[-0.301 - 0.058]	1.857E-01	9.928E-01
Left isthmus cingulate cortex	0.010	0.086	[-0.158 - 0.179]	9.049E-01	9.928E-01
Right isthmus cingulate cortex	-0.054	0.081	[-0.213 - 0.106]	5.096E-01	9.928E-01

Supplementary Table S64. Sex-by-Diagnosis interaction (MD/HC) on cortical surface area differences controlling for age and sex over 21 years of old

	d	Std. Err.	95% CI	*p*-value	FDR *q*-value
Total cortical surface area	-0.064	0.126	[-0.312 - 0.183]	6.098E-01	9.967E-01
Left hemisphere	-0.051	0.126	[-0.299 - 0.196]	6.845E-01	9.967E-01
Right hemisphere	-0.079	0.125	[-0.324 - 0.166]	5.282E-01	9.967E-01
Left superior frontal gyrus	-0.063	0.117	[-0.291 - 0.166]	5.910E-01	9.967E-01
Right superior frontal gyrus	-0.059	0.103	[-0.261 - 0.143]	5.666E-01	9.967E-01
Left pars orbitalis of inferior frontal gyrus	-0.010	0.117	[-0.240 - 0.219]	9.295E-01	9.967E-01
Right pars orbitalis of inferior frontal gyrus	-0.032	0.077	[-0.183 - 0.119]	6.786E-01	9.967E-01
Left precentral gyrus	-0.016	0.116	[-0.244 - 0.211]	8.886E-01	9.967E-01
Right precentral gyrus	-0.033	0.102	[-0.233 - 0.167]	7.458E-01	9.967E-01
Left lingual gyrus	-0.052	0.080	[-0.208 - 0.104]	5.155E-01	9.967E-01
Right lingual gyrus	-0.024	0.101	[-0.223 - 0.174]	8.090E-01	9.967E-01
Left superior temporal gyrus	-0.059	0.083	[-0.221 - 0.103]	4.756E-01	9.967E-01
Right superior temporal gyrus	-0.062	0.098	[-0.254 - 0.131]	5.296E-01	9.967E-01
Left fusiform gyrus	-0.076	0.110	[-0.292 - 0.140]	4.917E-01	9.967E-01
Right fusiform gyrus	-0.116	0.094	[-0.300 - 0.068]	2.154E-01	9.967E-01
Left inferior parietal cortex	-0.089	0.108	[-0.302 - 0.123]	4.091E-01	9.967E-01
Right inferior parietal cortex	-0.129	0.113	[-0.351 - 0.093]	2.544E-01	9.967E-01
Left lateral occipital cortex	-0.009	0.106	[-0.216 - 0.198]	9.349E-01	9.967E-01
Right lateral occipital cortex	0.044	0.100	[-0.152 - 0.240]	6.580E-01	9.967E-01
Left rostral middle frontal gyrus	-0.050	0.097	[-0.240 - 0.140]	6.059E-01	9.967E-01
Right rostral middle frontal gyrus	-0.042	0.094	[-0.227 - 0.142]	6.538E-01	9.967E-01
Left precuneus	-0.004	0.093	[-0.186 - 0.178]	9.663E-01	9.967E-01
Right precuneus	-0.068	0.099	[-0.263 - 0.126]	4.919E-01	9.967E-01
Left inferior temporal gyrus	-0.080	0.112	[-0.300 - 0.140]	4.748E-01	9.967E-01
Right inferior temporal gyrus	-0.188	0.080	[-0.345 - -0.031]	1.925E-02	9.967E-01
Left lateral orbitofrontal cortex	-0.021	0.098	[-0.214 - 0.172]	8.304E-01	9.967E-01
Right lateral orbitofrontal cortex	-0.076	0.086	[-0.245 - 0.094]	3.822E-01	9.967E-01
Left middle temporal gyrus	-0.015	0.109	[-0.229 - 0.199]	8.914E-01	9.967E-01
Right middle temporal gyrus	-0.073	0.114	[-0.296 - 0.151]	5.237E-01	9.967E-01
Left postcentral gyrus	-0.081	0.108	[-0.293 - 0.131]	4.561E-01	9.967E-01
Right postcentral gyrus	-0.051	0.102	[-0.252 - 0.150]	6.197E-01	9.967E-01
Left medial orbitofrontal cortex	-0.018	0.090	[-0.195 - 0.159]	8.413E-01	9.967E-01
Right medial orbitofrontal cortex	-0.046	0.096	[-0.234 - 0.143]	6.361E-01	9.967E-01
Left cuneus	0.003	0.088	[-0.170 - 0.175]	9.763E-01	9.967E-01
Right cuneus	0.035	0.080	[-0.121 - 0.191]	6.565E-01	9.967E-01
Left pars triangularis of inferior frontal gyrus	0.101	0.063	[-0.023 - 0.225]	1.094E-01	9.967E-01
Right pars triangularis of inferior frontal gyrus	-0.066	0.073	[-0.208 - 0.076]	3.633E-01	9.967E-01
Left superior parietal cortex	0.031	0.090	[-0.146 - 0.207]	7.327E-01	9.967E-01
Right superior parietal cortex	0.010	0.098	[-0.182 - 0.201]	9.225E-01	9.967E-01
Left pars opercularis of inferior frontal gyrus	0.084	0.086	[-0.086 - 0.253]	3.331E-01	9.967E-01
Right pars opercularis of inferior frontal gyrus	0.003	0.114	[-0.220 - 0.226]	9.794E-01	9.967E-01
Left supramarginal gyrus	-0.012	0.089	[-0.187 - 0.163]	8.935E-01	9.967E-01
Right supramarginal gyrus	-0.123	0.082	[-0.284 - 0.037]	1.317E-01	9.967E-01
Left pericalcarine cortex	-0.077	0.069	[-0.212 - 0.058]	2.651E-01	9.967E-01
Right pericalcarine cortex	0.021	0.088	[-0.152 - 0.193]	8.125E-01	9.967E-01
Left parahippocampal gyrus	-0.056	0.070	[-0.194 - 0.082]	4.249E-01	9.967E-01
Right parahippocampal gyrus	-0.018	0.063	[-0.141 - 0.106]	7.795E-01	9.967E-01
Left caudal middle frontal gyrus	-0.081	0.089	[-0.256 - 0.094]	3.659E-01	9.967E-01
Right caudal middle frontal gyrus	-0.012	0.090	[-0.189 - 0.165]	8.922E-01	9.967E-01
Left transverse temporal gyrus	-0.021	0.079	[-0.177 - 0.135]	7.904E-01	9.967E-01
Right transverse temporal gyrus	-0.082	0.087	[-0.252 - 0.088]	3.459E-01	9.967E-01
Left banks of superior temporal sulcus	-0.155	0.085	[-0.321 - 0.011]	6.781E-02	9.967E-01
Right banks of superior temporal sulcus	-0.150	0.125	[-0.396 - 0.096]	2.313E-01	9.967E-01
Left caudal anterior cingulate cortex	-0.028	0.104	[-0.233 - 0.177]	7.888E-01	9.967E-01
Right caudal anterior cingulate cortex	-0.101	0.088	[-0.273 - 0.072]	2.530E-01	9.967E-01
Left rostral anterior cingulate cortex	-0.001	0.094	[-0.185 - 0.182]	9.890E-01	9.967E-01
Right rostral anterior cingulate cortex	-0.055	0.068	[-0.188 - 0.077]	4.122E-01	9.967E-01
Left posterior cingulate cortex	-0.083	0.080	[-0.239 - 0.074]	3.018E-01	9.967E-01
Right posterior cingulate cortex	-0.036	0.091	[-0.215 - 0.144]	6.975E-01	9.967E-01
Left frontal pole	0.020	0.104	[-0.183 - 0.223]	8.472E-01	9.967E-01
Right frontal pole	-0.117	0.063	[-0.240 - 0.007]	6.461E-02	9.967E-01
Left paracentral lobule	-0.090	0.070	[-0.227 - 0.046]	1.946E-01	9.967E-01
Right paracentral lobule	-0.053	0.074	[-0.199 - 0.092]	4.730E-01	9.967E-01
Left insula	0.000	0.100	[-0.195 - 0.195]	9.967E-01	9.967E-01
Right insula	-0.060	0.074	[-0.206 - 0.086]	4.191E-01	9.967E-01
Left entorhinal cortex	-0.061	0.104	[-0.266 - 0.143]	5.563E-01	9.967E-01
Right entorhinal cortex	-0.050	0.076	[-0.199 - 0.099]	5.107E-01	9.967E-01
Left temporal pole	-0.099	0.093	[-0.281 - 0.083]	2.853E-01	9.967E-01
Right temporal pole	-0.097	0.087	[-0.267 - 0.073]	2.646E-01	9.967E-01
Left isthmus cingulate cortex	0.009	0.093	[-0.174 - 0.191]	9.244E-01	9.967E-01
Right isthmus cingulate cortex	-0.059	0.091	[-0.236 - 0.119]	5.176E-01	9.967E-01

Supplementary Table S65. Age-by-Diagnosis interaction (MD/HC) on cortical surface area differences controlling for age and sex

	d	Std. Err.	95% CI	*p*-value	FDR *q*-value
Total cortical surface area	0.043	0.072	[-0.098 - 0.184]	5.531E-01	9.087E-01
Left hemisphere	0.036	0.073	[-0.107 - 0.179]	6.194E-01	9.087E-01
Right hemisphere	0.049	0.071	[-0.090 - 0.188]	4.893E-01	9.087E-01
Left superior frontal gyrus	0.041	0.081	[-0.119 - 0.200]	6.186E-01	9.087E-01
Right superior frontal gyrus	0.065	0.092	[-0.115 - 0.245]	4.800E-01	9.087E-01
Left pars orbitalis of inferior frontal gyrus	-0.020	0.086	[-0.188 - 0.148]	8.171E-01	9.522E-01
Right pars orbitalis of inferior frontal gyrus	-0.032	0.064	[-0.157 - 0.092]	6.107E-01	9.087E-01
Left precentral gyrus	-0.050	0.092	[-0.231 - 0.131]	5.877E-01	9.087E-01
Right precentral gyrus	0.028	0.061	[-0.092 - 0.148]	6.460E-01	9.124E-01
Left lingual gyrus	-0.087	0.069	[-0.222 - 0.047]	2.046E-01	9.087E-01
Right lingual gyrus	-0.070	0.062	[-0.191 - 0.051]	2.569E-01	9.087E-01
Left superior temporal gyrus	0.057	0.091	[-0.121 - 0.234]	5.317E-01	9.087E-01
Right superior temporal gyrus	0.023	0.080	[-0.133 - 0.179]	7.735E-01	9.522E-01
Left fusiform gyrus	0.004	0.061	[-0.116 - 0.125]	9.444E-01	9.578E-01
Right fusiform gyrus	0.012	0.061	[-0.108 - 0.132]	8.449E-01	9.522E-01
Left inferior parietal cortex	0.055	0.061	[-0.065 - 0.175]	3.673E-01	9.087E-01
Right inferior parietal cortex	0.045	0.066	[-0.084 - 0.174]	4.966E-01	9.087E-01
Left lateral occipital cortex	0.020	0.085	[-0.147 - 0.187]	8.131E-01	9.522E-01
Right lateral occipital cortex	0.047	0.081	[-0.111 - 0.205]	5.594E-01	9.087E-01
Left rostral middle frontal gyrus	0.081	0.069	[-0.054 - 0.216]	2.379E-01	9.087E-01
Right rostral middle frontal gyrus	0.082	0.081	[-0.077 - 0.242]	3.119E-01	9.087E-01
Left precuneus	0.050	0.065	[-0.078 - 0.178]	4.452E-01	9.087E-01
Right precuneus	-0.011	0.077	[-0.163 - 0.140]	8.834E-01	9.578E-01
Left inferior temporal gyrus	-0.008	0.061	[-0.128 - 0.113]	9.010E-01	9.578E-01
Right inferior temporal gyrus	-0.002	0.061	[-0.122 - 0.119]	9.772E-01	9.772E-01
Left lateral orbitofrontal cortex	0.026	0.085	[-0.141 - 0.194]	7.587E-01	9.522E-01
Right lateral orbitofrontal cortex	0.055	0.066	[-0.074 - 0.184]	4.015E-01	9.087E-01
Left middle temporal gyrus	0.061	0.071	[-0.078 - 0.200]	3.919E-01	9.087E-01
Right middle temporal gyrus	-0.017	0.081	[-0.176 - 0.142]	8.320E-01	9.522E-01
Left postcentral gyrus	0.057	0.061	[-0.063 - 0.177]	3.532E-01	9.087E-01
Right postcentral gyrus	-0.044	0.061	[-0.165 - 0.076]	4.693E-01	9.087E-01
Left medial orbitofrontal cortex	-0.026	0.061	[-0.146 - 0.095]	6.763E-01	9.124E-01
Right medial orbitofrontal cortex	0.046	0.061	[-0.075 - 0.166]	4.575E-01	9.087E-01
Left cuneus	-0.102	0.064	[-0.227 - 0.024]	1.115E-01	9.087E-01
Right cuneus	0.034	0.082	[-0.126 - 0.193]	6.811E-01	9.124E-01
Left pars triangularis of inferior frontal gyrus	0.072	0.070	[-0.066 - 0.210]	3.055E-01	9.087E-01
Right pars triangularis of inferior frontal gyrus	0.082	0.082	[-0.080 - 0.243]	3.229E-01	9.087E-01
Left superior parietal cortex	0.008	0.061	[-0.112 - 0.128]	8.941E-01	9.578E-01
Right superior parietal cortex	0.005	0.061	[-0.115 - 0.125]	9.342E-01	9.578E-01
Left pars opercularis of inferior frontal gyrus	0.083	0.083	[-0.079 - 0.245]	3.147E-01	9.087E-01
Right pars opercularis of inferior frontal gyrus	0.044	0.061	[-0.077 - 0.164]	4.774E-01	9.087E-01
Left supramarginal gyrus	0.017	0.083	[-0.146 - 0.179]	8.409E-01	9.522E-01
Right supramarginal gyrus	0.086	0.061	[-0.034 - 0.206]	1.617E-01	9.087E-01
Left pericalcarine cortex	-0.077	0.061	[-0.198 - 0.043]	2.077E-01	9.087E-01
Right pericalcarine cortex	0.025	0.064	[-0.100 - 0.150]	6.941E-01	9.126E-01
Left parahippocampal gyrus	-0.006	0.073	[-0.149 - 0.137]	9.316E-01	9.578E-01
Right parahippocampal gyrus	-0.067	0.061	[-0.187 - 0.053]	2.763E-01	9.087E-01
Left caudal middle frontal gyrus	0.049	0.061	[-0.071 - 0.170]	4.203E-01	9.087E-01
Right caudal middle frontal gyrus	0.068	0.065	[-0.060 - 0.195]	2.971E-01	9.087E-01
Left transverse temporal gyrus	0.005	0.061	[-0.115 - 0.126]	9.292E-01	9.578E-01
Right transverse temporal gyrus	0.054	0.078	[-0.099 - 0.208]	4.880E-01	9.087E-01
Left banks of superior temporal sulcus	-0.037	0.063	[-0.161 - 0.087]	5.595E-01	9.087E-01
Right banks of superior temporal sulcus	0.036	0.061	[-0.084 - 0.157]	5.522E-01	9.087E-01
Left caudal anterior cingulate cortex	0.042	0.068	[-0.092 - 0.176]	5.416E-01	9.087E-01
Right caudal anterior cingulate cortex	0.108	0.061	[-0.012 - 0.229]	7.755E-02	9.087E-01
Left rostral anterior cingulate cortex	0.036	0.062	[-0.086 - 0.159]	5.587E-01	9.087E-01
Right rostral anterior cingulate cortex	0.077	0.061	[-0.043 - 0.198]	2.074E-01	9.087E-01
Left posterior cingulate cortex	-0.041	0.066	[-0.170 - 0.089]	5.365E-01	9.087E-01
Right posterior cingulate cortex	-0.032	0.061	[-0.152 - 0.088]	6.005E-01	9.087E-01
Left frontal pole	0.072	0.061	[-0.048 - 0.192]	2.396E-01	9.087E-01
Right frontal pole	-0.030	0.061	[-0.150 - 0.090]	6.215E-01	9.087E-01
Left paracentral lobule	-0.051	0.065	[-0.179 - 0.077]	4.350E-01	9.087E-01
Right paracentral lobule	0.060	0.061	[-0.060 - 0.180]	3.282E-01	9.087E-01
Left insula	0.103	0.063	[-0.021 - 0.226]	1.027E-01	9.087E-01
Right insula	0.039	0.080	[-0.117 - 0.195]	6.271E-01	9.087E-01
Left entorhinal cortex	0.013	0.061	[-0.107 - 0.134]	8.285E-01	9.522E-01
Right entorhinal cortex	0.070	0.061	[-0.050 - 0.190]	2.537E-01	9.087E-01
Left temporal pole	0.028	0.082	[-0.132 - 0.189]	7.315E-01	9.443E-01
Right temporal pole	0.065	0.073	[-0.078 - 0.207]	3.726E-01	9.087E-01
Left isthmus cingulate cortex	0.054	0.077	[-0.096 - 0.205]	4.788E-01	9.087E-01
Right isthmus cingulate cortex	-0.030	0.071	[-0.170 - 0.110]	6.734E-01	9.124E-01

Supplementary Table S66. Age-by-Diagnosis interaction (MD/HC) on cortical surface area differences controlling for age and sex over 21 years of old

	d	Std. Err.	95% CI	*p*-value	FDR *q*-value
Total cortical surface area	0.035	0.074	[-0.109 - 0.179]	6.360E-01	8.856E-01
Left hemisphere	0.028	0.073	[-0.115 - 0.171]	6.974E-01	8.856E-01
Right hemisphere	0.042	0.074	[-0.104 - 0.187]	5.753E-01	8.819E-01
Left superior frontal gyrus	0.033	0.082	[-0.128 - 0.194]	6.907E-01	8.856E-01
Right superior frontal gyrus	0.077	0.094	[-0.106 - 0.261]	4.084E-01	8.819E-01
Left pars orbitalis of inferior frontal gyrus	-0.031	0.083	[-0.193 - 0.132]	7.110E-01	8.856E-01
Right pars orbitalis of inferior frontal gyrus	-0.036	0.066	[-0.166 - 0.093]	5.838E-01	8.819E-01
Left precentral gyrus	-0.034	0.087	[-0.204 - 0.137]	6.997E-01	8.856E-01
Right precentral gyrus	0.036	0.063	[-0.088 - 0.160]	5.684E-01	8.819E-01
Left lingual gyrus	-0.112	0.073	[-0.255 - 0.031]	1.255E-01	8.819E-01
Right lingual gyrus	-0.080	0.065	[-0.208 - 0.048]	2.214E-01	8.819E-01
Left superior temporal gyrus	0.058	0.092	[-0.122 - 0.238]	5.297E-01	8.819E-01
Right superior temporal gyrus	0.021	0.079	[-0.134 - 0.176]	7.876E-01	8.938E-01
Left fusiform gyrus	0.003	0.065	[-0.124 - 0.129]	9.673E-01	9.738E-01
Right fusiform gyrus	-0.017	0.063	[-0.141 - 0.106]	7.832E-01	8.938E-01
Left inferior parietal cortex	0.041	0.063	[-0.082 - 0.165]	5.131E-01	8.819E-01
Right inferior parietal cortex	0.039	0.069	[-0.096 - 0.174]	5.704E-01	8.819E-01
Left lateral occipital cortex	0.014	0.081	[-0.144 - 0.171]	8.663E-01	9.180E-01
Right lateral occipital cortex	0.053	0.073	[-0.090 - 0.197]	4.644E-01	8.819E-01
Left rostral middle frontal gyrus	0.081	0.073	[-0.062 - 0.225]	2.677E-01	8.819E-01
Right rostral middle frontal gyrus	0.101	0.079	[-0.054 - 0.257]	2.002E-01	8.819E-01
Left precuneus	0.039	0.071	[-0.099 - 0.178]	5.785E-01	8.819E-01
Right precuneus	-0.056	0.075	[-0.202 - 0.091]	4.572E-01	8.819E-01
Left inferior temporal gyrus	-0.040	0.063	[-0.164 - 0.083]	5.228E-01	8.819E-01
Right inferior temporal gyrus	-0.017	0.063	[-0.140 - 0.107]	7.931E-01	8.938E-01
Left lateral orbitofrontal cortex	0.031	0.077	[-0.120 - 0.182]	6.884E-01	8.856E-01
Right lateral orbitofrontal cortex	0.039	0.065	[-0.088 - 0.167]	5.470E-01	8.819E-01
Left middle temporal gyrus	0.041	0.067	[-0.090 - 0.172]	5.405E-01	8.819E-01
Right middle temporal gyrus	-0.003	0.085	[-0.169 - 0.164]	9.738E-01	9.738E-01
Left postcentral gyrus	0.069	0.063	[-0.055 - 0.193]	2.737E-01	8.819E-01
Right postcentral gyrus	-0.037	0.063	[-0.160 - 0.087]	5.620E-01	8.819E-01
Left medial orbitofrontal cortex	-0.037	0.063	[-0.161 - 0.086]	5.532E-01	8.819E-01
Right medial orbitofrontal cortex	0.050	0.063	[-0.073 - 0.174]	4.248E-01	8.819E-01
Left cuneus	-0.143	0.063	[-0.267 - -0.019]	2.346E-02	8.819E-01
Right cuneus	0.049	0.073	[-0.094 - 0.191]	5.036E-01	8.819E-01
Left pars triangularis of inferior frontal gyrus	0.068	0.073	[-0.075 - 0.210]	3.535E-01	8.819E-01
Right pars triangularis of inferior frontal gyrus	0.023	0.074	[-0.122 - 0.168]	7.562E-01	8.938E-01
Left superior parietal cortex	-0.002	0.063	[-0.126 - 0.121]	9.691E-01	9.738E-01
Right superior parietal cortex	-0.029	0.063	[-0.153 - 0.094]	6.421E-01	8.856E-01
Left pars opercularis of inferior frontal gyrus	0.053	0.088	[-0.120 - 0.226]	5.449E-01	8.819E-01
Right pars opercularis of inferior frontal gyrus	0.021	0.063	[-0.103 - 0.145]	7.389E-01	8.938E-01
Left supramarginal gyrus	0.031	0.085	[-0.135 - 0.198]	7.108E-01	8.856E-01
Right supramarginal gyrus	0.087	0.063	[-0.036 - 0.211]	1.657E-01	8.819E-01
Left pericalcarine cortex	-0.100	0.063	[-0.224 - 0.024]	1.136E-01	8.819E-01
Right pericalcarine cortex	0.012	0.063	[-0.111 - 0.136]	8.432E-01	9.071E-01
Left parahippocampal gyrus	0.008	0.080	[-0.148 - 0.164]	9.199E-01	9.605E-01
Right parahippocampal gyrus	-0.068	0.063	[-0.192 - 0.056]	2.801E-01	8.819E-01
Left caudal middle frontal gyrus	0.040	0.063	[-0.084 - 0.164]	5.280E-01	8.819E-01
Right caudal middle frontal gyrus	0.075	0.073	[-0.068 - 0.219]	3.036E-01	8.819E-01
Left transverse temporal gyrus	0.018	0.063	[-0.105 - 0.142]	7.713E-01	8.938E-01
Right transverse temporal gyrus	0.065	0.079	[-0.090 - 0.219]	4.117E-01	8.819E-01
Left banks of superior temporal sulcus	-0.027	0.070	[-0.164 - 0.110]	6.987E-01	8.856E-01
Right banks of superior temporal sulcus	0.042	0.063	[-0.081 - 0.166]	5.008E-01	8.819E-01
Left caudal anterior cingulate cortex	0.053	0.063	[-0.071 - 0.177]	4.014E-01	8.819E-01
Right caudal anterior cingulate cortex	0.092	0.063	[-0.032 - 0.215]	1.468E-01	8.819E-01
Left rostral anterior cingulate cortex	0.040	0.063	[-0.084 - 0.163]	5.300E-01	8.819E-01
Right rostral anterior cingulate cortex	0.074	0.063	[-0.050 - 0.197]	2.439E-01	8.819E-01
Left posterior cingulate cortex	-0.047	0.066	[-0.177 - 0.082]	4.732E-01	8.819E-01
Right posterior cingulate cortex	-0.033	0.063	[-0.157 - 0.090]	5.975E-01	8.837E-01
Left frontal pole	0.070	0.063	[-0.053 - 0.194]	2.654E-01	8.819E-01
Right frontal pole	-0.013	0.063	[-0.137 - 0.111]	8.356E-01	9.071E-01
Left paracentral lobule	-0.047	0.070	[-0.184 - 0.090]	5.003E-01	8.819E-01
Right paracentral lobule	0.069	0.063	[-0.055 - 0.193]	2.733E-01	8.819E-01
Left insula	0.100	0.065	[-0.028 - 0.227]	1.270E-01	8.819E-01
Right insula	0.044	0.076	[-0.106 - 0.194]	5.668E-01	8.819E-01
Left entorhinal cortex	0.050	0.063	[-0.074 - 0.173]	4.323E-01	8.819E-01
Right entorhinal cortex	0.066	0.063	[-0.058 - 0.190]	2.969E-01	8.819E-01
Left temporal pole	0.016	0.081	[-0.142 - 0.175]	8.385E-01	9.071E-01
Right temporal pole	0.073	0.075	[-0.075 - 0.221]	3.315E-01	8.819E-01
Left isthmus cingulate cortex	0.090	0.073	[-0.052 - 0.233]	2.125E-01	8.819E-01
Right isthmus cingulate cortex	-0.068	0.069	[-0.202 - 0.067]	3.246E-01	8.819E-01

Supplementary Table S67. Sex-by-Diagnosis interaction (ASD/HC) on cortical surface area differences controlling for age and sex

	d	Std. Err.	95% CI	*p*-value	FDR *q*-value
Total cortical surface area	-0.077	0.095	[-0.263 - 0.108]	4.156E-01	9.824E-01
Left hemisphere	-0.075	0.095	[-0.261 - 0.111]	4.281E-01	9.824E-01
Right hemisphere	-0.079	0.095	[-0.264 - 0.107]	4.056E-01	9.824E-01
Left superior frontal gyrus	-0.094	0.095	[-0.279 - 0.092]	3.233E-01	9.824E-01
Right superior frontal gyrus	-0.063	0.095	[-0.249 - 0.122]	5.023E-01	9.824E-01
Left pars orbitalis of inferior frontal gyrus	0.157	0.179	[-0.194 - 0.509]	3.810E-01	9.824E-01
Right pars orbitalis of inferior frontal gyrus	0.060	0.095	[-0.125 - 0.245]	5.249E-01	9.824E-01
Left precentral gyrus	-0.007	0.095	[-0.192 - 0.178]	9.392E-01	9.824E-01
Right precentral gyrus	-0.025	0.095	[-0.211 - 0.160]	7.895E-01	9.824E-01
Left lingual gyrus	0.028	0.095	[-0.157 - 0.213]	7.670E-01	9.824E-01
Right lingual gyrus	0.063	0.152	[-0.234 - 0.360]	6.796E-01	9.824E-01
Left superior temporal gyrus	-0.148	0.095	[-0.334 - 0.038]	1.180E-01	9.824E-01
Right superior temporal gyrus	-0.203	0.095	[-0.388 - -0.017]	3.229E-02	9.824E-01
Left fusiform gyrus	-0.070	0.095	[-0.255 - 0.116]	4.612E-01	9.824E-01
Right fusiform gyrus	-0.072	0.137	[-0.340 - 0.197]	6.013E-01	9.824E-01
Left inferior parietal cortex	-0.188	0.095	[-0.373 - -0.002]	4.732E-02	9.824E-01
Right inferior parietal cortex	-0.071	0.095	[-0.256 - 0.114]	4.535E-01	9.824E-01
Left lateral occipital cortex	-0.040	0.108	[-0.251 - 0.171]	7.108E-01	9.824E-01
Right lateral occipital cortex	-0.097	0.149	[-0.389 - 0.196]	5.174E-01	9.824E-01
Left rostral middle frontal gyrus	-0.032	0.095	[-0.217 - 0.154]	7.368E-01	9.824E-01
Right rostral middle frontal gyrus	-0.021	0.095	[-0.207 - 0.165]	8.246E-01	9.824E-01
Left precuneus	0.004	0.100	[-0.192 - 0.200]	9.674E-01	9.824E-01
Right precuneus	-0.042	0.102	[-0.242 - 0.159]	6.843E-01	9.824E-01
Left inferior temporal gyrus	-0.160	0.095	[-0.346 - 0.025]	9.071E-02	9.824E-01
Right inferior temporal gyrus	-0.141	0.095	[-0.327 - 0.045]	1.364E-01	9.824E-01
Left lateral orbitofrontal cortex	-0.038	0.095	[-0.223 - 0.147]	6.885E-01	9.824E-01
Right lateral orbitofrontal cortex	-0.032	0.095	[-0.217 - 0.154]	7.367E-01	9.824E-01
Left middle temporal gyrus	-0.056	0.095	[-0.241 - 0.130]	5.566E-01	9.824E-01
Right middle temporal gyrus	-0.171	0.124	[-0.414 - 0.073]	1.702E-01	9.824E-01
Left postcentral gyrus	-0.058	0.116	[-0.285 - 0.168]	6.147E-01	9.824E-01
Right postcentral gyrus	-0.102	0.095	[-0.287 - 0.084]	2.829E-01	9.824E-01
Left medial orbitofrontal cortex	0.070	0.095	[-0.115 - 0.256]	4.563E-01	9.824E-01
Right medial orbitofrontal cortex	0.140	0.095	[-0.045 - 0.325]	1.390E-01	9.824E-01
Left cuneus	0.104	0.095	[-0.082 - 0.289]	2.724E-01	9.824E-01
Right cuneus	-0.017	0.095	[-0.203 - 0.168]	8.551E-01	9.824E-01
Left pars triangularis of inferior frontal gyrus	-0.062	0.101	[-0.260 - 0.137]	5.432E-01	9.824E-01
Right pars triangularis of inferior frontal gyrus	-0.088	0.130	[-0.343 - 0.167]	4.979E-01	9.824E-01
Left superior parietal cortex	0.009	0.095	[-0.176 - 0.194]	9.245E-01	9.824E-01
Right superior parietal cortex	0.028	0.095	[-0.157 - 0.213]	7.665E-01	9.824E-01
Left pars opercularis of inferior frontal gyrus	0.006	0.158	[-0.303 - 0.316]	9.686E-01	9.824E-01
Right pars opercularis of inferior frontal gyrus	-0.086	0.115	[-0.311 - 0.139]	4.551E-01	9.824E-01
Left supramarginal gyrus	-0.115	0.095	[-0.300 - 0.070]	2.244E-01	9.824E-01
Right supramarginal gyrus	-0.078	0.095	[-0.264 - 0.107]	4.067E-01	9.824E-01
Left pericalcarine cortex	0.049	0.140	[-0.226 - 0.324]	7.282E-01	9.824E-01
Right pericalcarine cortex	-0.007	0.121	[-0.245 - 0.230]	9.511E-01	9.824E-01
Left parahippocampal gyrus	0.023	0.095	[-0.162 - 0.208]	8.073E-01	9.824E-01
Right parahippocampal gyrus	-0.012	0.131	[-0.269 - 0.245]	9.263E-01	9.824E-01
Left caudal middle frontal gyrus	-0.086	0.095	[-0.272 - 0.099]	3.620E-01	9.824E-01
Right caudal middle frontal gyrus	-0.091	0.117	[-0.320 - 0.138]	4.359E-01	9.824E-01
Left transverse temporal gyrus	-0.070	0.095	[-0.255 - 0.116]	4.602E-01	9.824E-01
Right transverse temporal gyrus	-0.061	0.095	[-0.246 - 0.124]	5.176E-01	9.824E-01
Left banks of superior temporal sulcus	-0.139	0.095	[-0.324 - 0.046]	1.414E-01	9.824E-01
Right banks of superior temporal sulcus	-0.118	0.095	[-0.304 - 0.067]	2.114E-01	9.824E-01
Left caudal anterior cingulate cortex	-0.002	0.101	[-0.200 - 0.196]	9.862E-01	9.862E-01
Right caudal anterior cingulate cortex	-0.009	0.095	[-0.194 - 0.176]	9.241E-01	9.824E-01
Left rostral anterior cingulate cortex	-0.004	0.095	[-0.189 - 0.181]	9.674E-01	9.824E-01
Right rostral anterior cingulate cortex	-0.107	0.138	[-0.377 - 0.164]	4.392E-01	9.824E-01
Left posterior cingulate cortex	0.026	0.095	[-0.159 - 0.212]	7.807E-01	9.824E-01
Right posterior cingulate cortex	0.063	0.142	[-0.215 - 0.341]	6.572E-01	9.824E-01
Left frontal pole	0.024	0.163	[-0.294 - 0.343]	8.812E-01	9.824E-01
Right frontal pole	0.095	0.095	[-0.090 - 0.280]	3.142E-01	9.824E-01
Left paracentral lobule	0.029	0.137	[-0.239 - 0.297]	8.301E-01	9.824E-01
Right paracentral lobule	0.033	0.133	[-0.228 - 0.294]	8.053E-01	9.824E-01
Left insula	-0.007	0.107	[-0.217 - 0.202]	9.460E-01	9.824E-01
Right insula	0.030	0.095	[-0.155 - 0.216]	7.485E-01	9.824E-01
Left entorhinal cortex	-0.052	0.128	[-0.304 - 0.199]	6.823E-01	9.824E-01
Right entorhinal cortex	-0.110	0.095	[-0.295 - 0.075]	2.449E-01	9.824E-01
Left temporal pole	0.081	0.121	[-0.157 - 0.318]	5.059E-01	9.824E-01
Right temporal pole	-0.119	0.127	[-0.369 - 0.131]	3.521E-01	9.824E-01
Left isthmus cingulate cortex	-0.030	0.095	[-0.215 - 0.155]	7.503E-01	9.824E-01
Right isthmus cingulate cortex	-0.028	0.095	[-0.213 - 0.158]	7.694E-01	9.824E-01

Supplementary Table S68. Age-by-Diagnosis interaction (ASD/HC) on cortical surface area differences controlling for age and sex

	d	Std. Err.	95% CI	*p*-value	FDR *q*-value
Total cortical surface area	0.106	0.087	[-0.065 - 0.277]	2.257E-01	7.632E-01
Left hemisphere	0.094	0.091	[-0.084 - 0.271]	2.999E-01	7.932E-01
Right hemisphere	0.117	0.085	[-0.049 - 0.283]	1.684E-01	7.632E-01
Left superior frontal gyrus	-0.002	0.092	[-0.181 - 0.178]	9.848E-01	9.981E-01
Right superior frontal gyrus	0.065	0.080	[-0.091 - 0.222]	4.137E-01	8.805E-01
Left pars orbitalis of inferior frontal gyrus	0.029	0.080	[-0.128 - 0.186]	7.170E-01	9.007E-01
Right pars orbitalis of inferior frontal gyrus	0.141	0.126	[-0.106 - 0.387]	2.627E-01	7.772E-01
Left precentral gyrus	0.076	0.109	[-0.137 - 0.289]	4.828E-01	9.007E-01
Right precentral gyrus	-0.029	0.080	[-0.186 - 0.128]	7.159E-01	9.007E-01
Left lingual gyrus	0.131	0.124	[-0.112 - 0.375]	2.901E-01	7.932E-01
Right lingual gyrus	0.112	0.118	[-0.118 - 0.343]	3.397E-01	8.334E-01
Left superior temporal gyrus	0.098	0.080	[-0.059 - 0.255]	2.203E-01	7.632E-01
Right superior temporal gyrus	0.186	0.080	[0.029 - 0.343]	2.016E-02	6.420E-01
Left fusiform gyrus	0.136	0.080	[-0.021 - 0.293]	8.984E-02	7.196E-01
Right fusiform gyrus	0.082	0.144	[-0.200 - 0.363]	5.688E-01	9.007E-01
Left inferior parietal cortex	0.037	0.092	[-0.144 - 0.218]	6.874E-01	9.007E-01
Right inferior parietal cortex	0.041	0.092	[-0.138 - 0.221]	6.528E-01	9.007E-01
Left lateral occipital cortex	0.027	0.080	[-0.129 - 0.184]	7.318E-01	9.007E-01
Right lateral occipital cortex	0.126	0.080	[-0.031 - 0.283]	1.159E-01	7.196E-01
Left rostral middle frontal gyrus	0.022	0.125	[-0.223 - 0.267]	8.599E-01	9.393E-01
Right rostral middle frontal gyrus	0.094	0.098	[-0.099 - 0.286]	3.404E-01	8.334E-01
Left precuneus	0.029	0.080	[-0.128 - 0.185]	7.201E-01	9.007E-01
Right precuneus	0.066	0.080	[-0.091 - 0.222]	4.127E-01	8.805E-01
Left inferior temporal gyrus	0.027	0.099	[-0.167 - 0.220]	7.869E-01	9.050E-01
Right inferior temporal gyrus	0.000	0.081	[-0.158 - 0.158]	9.981E-01	9.981E-01
Left lateral orbitofrontal cortex	0.039	0.092	[-0.142 - 0.220]	6.730E-01	9.007E-01
Right lateral orbitofrontal cortex	0.111	0.080	[-0.046 - 0.268]	1.656E-01	7.632E-01
Left middle temporal gyrus	0.142	0.094	[-0.043 - 0.327]	1.318E-01	7.196E-01
Right middle temporal gyrus	0.165	0.080	[0.008 - 0.322]	3.931E-02	6.420E-01
Left postcentral gyrus	0.042	0.080	[-0.115 - 0.199]	5.982E-01	9.007E-01
Right postcentral gyrus	0.032	0.080	[-0.125 - 0.189]	6.916E-01	9.007E-01
Left medial orbitofrontal cortex	-0.010	0.086	[-0.178 - 0.158]	9.114E-01	9.658E-01
Right medial orbitofrontal cortex	-0.090	0.080	[-0.247 - 0.067]	2.590E-01	7.772E-01
Left cuneus	0.002	0.080	[-0.155 - 0.159]	9.813E-01	9.981E-01
Right cuneus	0.066	0.084	[-0.098 - 0.231]	4.303E-01	8.805E-01
Left pars triangularis of inferior frontal gyrus	0.163	0.106	[-0.045 - 0.370]	1.238E-01	7.196E-01
Right pars triangularis of inferior frontal gyrus	0.070	0.115	[-0.156 - 0.296]	5.450E-01	9.007E-01
Left superior parietal cortex	0.030	0.094	[-0.154 - 0.214]	7.485E-01	9.007E-01
Right superior parietal cortex	0.083	0.080	[-0.074 - 0.240]	3.017E-01	7.932E-01
Left pars opercularis of inferior frontal gyrus	-0.031	0.083	[-0.193 - 0.132]	7.107E-01	9.007E-01
Right pars opercularis of inferior frontal gyrus	0.001	0.119	[-0.233 - 0.236]	9.900E-01	9.981E-01
Left supramarginal gyrus	0.150	0.080	[-0.007 - 0.306]	6.157E-02	7.196E-01
Right supramarginal gyrus	0.188	0.080	[0.031 - 0.345]	1.899E-02	6.420E-01
Left pericalcarine cortex	-0.062	0.112	[-0.281 - 0.158]	5.803E-01	9.007E-01
Right pericalcarine cortex	0.104	0.081	[-0.055 - 0.264]	2.008E-01	7.632E-01
Left parahippocampal gyrus	0.124	0.080	[-0.033 - 0.281]	1.206E-01	7.196E-01
Right parahippocampal gyrus	0.020	0.097	[-0.171 - 0.211]	8.368E-01	9.283E-01
Left caudal middle frontal gyrus	0.135	0.080	[-0.022 - 0.292]	9.221E-02	7.196E-01
Right caudal middle frontal gyrus	0.048	0.088	[-0.125 - 0.221]	5.886E-01	9.007E-01
Left transverse temporal gyrus	0.109	0.080	[-0.048 - 0.266]	1.723E-01	7.632E-01
Right transverse temporal gyrus	0.108	0.088	[-0.064 - 0.281]	2.171E-01	7.632E-01
Left banks of superior temporal sulcus	0.065	0.083	[-0.097 - 0.227]	4.340E-01	8.805E-01
Right banks of superior temporal sulcus	0.090	0.102	[-0.111 - 0.290]	3.808E-01	8.721E-01
Left caudal anterior cingulate cortex	0.029	0.098	[-0.163 - 0.222]	7.642E-01	9.043E-01
Right caudal anterior cingulate cortex	-0.014	0.104	[-0.218 - 0.190]	8.925E-01	9.601E-01
Left rostral anterior cingulate cortex	0.122	0.080	[-0.035 - 0.279]	1.289E-01	7.196E-01
Right rostral anterior cingulate cortex	0.071	0.080	[-0.086 - 0.228]	3.767E-01	8.721E-01
Left posterior cingulate cortex	0.045	0.080	[-0.111 - 0.202]	5.711E-01	9.007E-01
Right posterior cingulate cortex	0.034	0.080	[-0.123 - 0.191]	6.705E-01	9.007E-01
Left frontal pole	-0.051	0.086	[-0.219 - 0.116]	5.476E-01	9.007E-01
Right frontal pole	-0.105	0.080	[-0.262 - 0.052]	1.912E-01	7.632E-01
Left paracentral lobule	-0.090	0.080	[-0.247 - 0.067]	2.599E-01	7.772E-01
Right paracentral lobule	-0.024	0.098	[-0.216 - 0.168]	8.043E-01	9.065E-01
Left insula	0.046	0.080	[-0.111 - 0.203]	5.638E-01	9.007E-01
Right insula	0.051	0.080	[-0.105 - 0.208]	5.198E-01	9.007E-01
Left entorhinal cortex	0.032	0.098	[-0.161 - 0.225]	7.435E-01	9.007E-01
Right entorhinal cortex	0.050	0.121	[-0.187 - 0.287]	6.798E-01	9.007E-01
Left temporal pole	0.032	0.122	[-0.207 - 0.272]	7.903E-01	9.050E-01
Right temporal pole	0.045	0.132	[-0.213 - 0.303]	7.324E-01	9.007E-01
Left isthmus cingulate cortex	0.160	0.080	[0.003 - 0.317]	4.521E-02	6.420E-01
Right isthmus cingulate cortex	0.171	0.080	[0.014 - 0.328]	3.309E-02	6.420E-01

Supplementary Table S69. Partial correlations between cortical thickness and age of onset controlling for age and sex in individuals with schizophrenia

	Partial *R*	Std. Err.	95% CI	*p*-value	FDR *q*-value
Global mean cortical thickness	0.010	0.034	[-0.056 - 0.076]	7.617E-01	9.293E-01
Left hemisphere	0.007	0.035	[-0.061 - 0.076]	8.351E-01	9.293E-01
Right hemisphere	0.014	0.033	[-0.051 - 0.079]	6.791E-01	9.293E-01
Left fusiform gyrus	0.020	0.030	[-0.039 - 0.080]	5.031E-01	9.293E-01
Right fusiform gyrus	0.007	0.030	[-0.052 - 0.067]	8.163E-01	9.293E-01
Left pars opercularis of inferior frontal gyrus	0.050	0.031	[-0.011 - 0.111]	1.076E-01	9.293E-01
Right pars opercularis of inferior frontal gyrus	0.011	0.030	[-0.048 - 0.071]	7.136E-01	9.293E-01
Left superior temporal gyrus	-0.024	0.043	[-0.109 - 0.060]	5.690E-01	9.293E-01
Right superior temporal gyrus	-0.026	0.046	[-0.117 - 0.064]	5.725E-01	9.293E-01
Left insula	0.061	0.030	[0.002 - 0.121]	4.314E-02	7.658E-01
Right insula	0.069	0.030	[0.010 - 0.129]	2.252E-02	7.658E-01
Left lingual gyrus	0.014	0.036	[-0.056 - 0.085]	6.959E-01	9.293E-01
Right lingual gyrus	0.011	0.032	[-0.053 - 0.075]	7.352E-01	9.293E-01
Left pars triangularis of inferior frontal gyrus	0.040	0.030	[-0.020 - 0.099]	1.922E-01	9.293E-01
Right pars triangularis of inferior frontal gyrus	0.038	0.030	[-0.021 - 0.098]	2.069E-01	9.293E-01
Left lateral orbitofrontal cortex	0.025	0.030	[-0.035 - 0.084]	4.128E-01	9.293E-01
Right lateral orbitofrontal cortex	0.037	0.030	[-0.022 - 0.097]	2.204E-01	9.293E-01
Left rostral middle frontal gyrus	0.015	0.030	[-0.044 - 0.075]	6.150E-01	9.293E-01
Right rostral middle frontal gyrus	0.013	0.030	[-0.047 - 0.072]	6.688E-01	9.293E-01
Left middle temporal gyrus	-0.005	0.035	[-0.075 - 0.064]	8.765E-01	9.293E-01
Right middle temporal gyrus	-0.030	0.030	[-0.090 - 0.029]	3.200E-01	9.293E-01
Left superior frontal gyrus	-0.033	0.034	[-0.099 - 0.033]	3.272E-01	9.293E-01
Right superior frontal gyrus	-0.010	0.031	[-0.071 - 0.051]	7.498E-01	9.293E-01
Left pars orbitalis of inferior frontal gyrus	0.098	0.030	[0.038 - 0.157]	1.260E-03	8.945E-02
Right pars orbitalis of inferior frontal gyrus	0.040	0.030	[-0.019 - 0.100]	1.874E-01	9.293E-01
Left medial orbitofrontal cortex	0.012	0.034	[-0.054 - 0.079]	7.169E-01	9.293E-01
Right medial orbitofrontal cortex	0.001	0.031	[-0.060 - 0.063]	9.658E-01	9.658E-01
Left inferior temporal gyrus	0.027	0.038	[-0.046 - 0.101]	4.685E-01	9.293E-01
Right inferior temporal gyrus	-0.010	0.040	[-0.088 - 0.068]	8.015E-01	9.293E-01
Left isthmus cingulate cortex	0.056	0.035	[-0.014 - 0.125]	1.156E-01	9.293E-01
Right isthmus cingulate cortex	0.058	0.031	[-0.002 - 0.118]	5.760E-02	8.179E-01
Left banks of superior temporal sulcus	0.045	0.033	[-0.020 - 0.110]	1.757E-01	9.293E-01
Right banks of superior temporal sulcus	-0.004	0.030	[-0.064 - 0.055]	8.900E-01	9.293E-01
Left supramarginal gyrus	-0.015	0.035	[-0.083 - 0.054]	6.748E-01	9.293E-01
Right supramarginal gyrus	-0.019	0.039	[-0.096 - 0.057]	6.213E-01	9.293E-01
Left caudal middle frontal gyrus	-0.032	0.032	[-0.094 - 0.031]	3.202E-01	9.293E-01
Right caudal middle frontal gyrus	0.021	0.033	[-0.043 - 0.086]	5.165E-01	9.293E-01
Left frontal pole	-0.031	0.041	[-0.111 - 0.049]	4.503E-01	9.293E-01
Right frontal pole	0.031	0.044	[-0.055 - 0.118]	4.764E-01	9.293E-01
Left posterior cingulate cortex	0.025	0.030	[-0.034 - 0.085]	4.015E-01	9.293E-01
Right posterior cingulate cortex	0.062	0.030	[0.002 - 0.121]	4.272E-02	7.658E-01
Left lateral occipital cortex	0.023	0.031	[-0.039 - 0.085]	4.626E-01	9.293E-01
Right lateral occipital cortex	0.039	0.030	[-0.020 - 0.099]	1.978E-01	9.293E-01
Left precentral gyrus	-0.020	0.034	[-0.087 - 0.048]	5.677E-01	9.293E-01
Right precentral gyrus	0.011	0.030	[-0.048 - 0.071]	7.117E-01	9.293E-01
Left parahippocampal gyrus	0.039	0.035	[-0.030 - 0.107]	2.667E-01	9.293E-01
Right parahippocampal gyrus	0.028	0.044	[-0.058 - 0.114]	5.244E-01	9.293E-01
Left inferior parietal cortex	-0.009	0.037	[-0.082 - 0.064]	8.155E-01	9.293E-01
Right inferior parietal cortex	0.003	0.031	[-0.058 - 0.065]	9.152E-01	9.417E-01
Left transverse temporal gyrus	0.009	0.045	[-0.079 - 0.098]	8.354E-01	9.293E-01
Right transverse temporal gyrus	0.046	0.053	[-0.058 - 0.150]	3.833E-01	9.293E-01
Left postcentral gyrus	-0.017	0.040	[-0.096 - 0.061]	6.659E-01	9.293E-01
Right postcentral gyrus	-0.005	0.030	[-0.064 - 0.055]	8.791E-01	9.293E-01
Left precuneus	-0.020	0.035	[-0.088 - 0.048]	5.690E-01	9.293E-01
Right precuneus	-0.004	0.030	[-0.064 - 0.055]	8.834E-01	9.293E-01
Left caudal anterior cingulate cortex	0.039	0.036	[-0.033 - 0.110]	2.880E-01	9.293E-01
Right caudal anterior cingulate cortex	0.038	0.033	[-0.026 - 0.102]	2.461E-01	9.293E-01
Left cuneus	0.016	0.039	[-0.061 - 0.094]	6.783E-01	9.293E-01
Right cuneus	0.050	0.038	[-0.024 - 0.123]	1.882E-01	9.293E-01
Left rostral anterior cingulate cortex	0.021	0.033	[-0.044 - 0.086]	5.296E-01	9.293E-01
Right rostral anterior cingulate cortex	0.039	0.030	[-0.021 - 0.098]	2.026E-01	9.293E-01
Left pericalcarine cortex	0.028	0.046	[-0.062 - 0.118]	5.357E-01	9.293E-01
Right pericalcarine cortex	-0.006	0.045	[-0.095 - 0.082]	8.870E-01	9.293E-01
Left paracentral lobule	-0.011	0.030	[-0.071 - 0.048]	7.114E-01	9.293E-01
Right paracentral lobule	0.002	0.030	[-0.057 - 0.062]	9.445E-01	9.580E-01
Left superior parietal cortex	-0.020	0.033	[-0.085 - 0.045]	5.422E-01	9.293E-01
Right superior parietal cortex	-0.011	0.035	[-0.080 - 0.057]	7.422E-01	9.293E-01
Left temporal pole	0.051	0.041	[-0.030 - 0.132]	2.177E-01	9.293E-01
Right temporal pole	0.025	0.046	[-0.066 - 0.116]	5.906E-01	9.293E-01
Left entorhinal cortex	0.037	0.033	[-0.028 - 0.102]	2.636E-01	9.293E-01
Right entorhinal cortex	-0.007	0.030	[-0.067 - 0.052]	8.055E-01	9.293E-01

Supplementary Table S70. Partial correlations between cortical thickness and duration of illness controlling for age and sex in individuals with schizophrenia

	Partial *R*	Std. Err.	95% CI	*p*-value	FDR *q*-value
Global mean cortical thickness	-0.008	0.035	[-0.077 - 0.061]	8.293E-01	9.422E-01
Left hemisphere	-0.003	0.038	[-0.076 - 0.071]	9.455E-01	9.789E-01
Right hemisphere	-0.012	0.034	[-0.079 - 0.054]	7.171E-01	9.246E-01
Left fusiform gyrus	-0.019	0.030	[-0.079 - 0.040]	5.208E-01	9.246E-01
Right fusiform gyrus	-0.010	0.030	[-0.069 - 0.050]	7.488E-01	9.246E-01
Left pars opercularis of inferior frontal gyrus	-0.047	0.034	[-0.112 - 0.019]	1.648E-01	9.246E-01
Right pars opercularis of inferior frontal gyrus	-0.014	0.030	[-0.073 - 0.046]	6.552E-01	9.246E-01
Left superior temporal gyrus	0.023	0.042	[-0.058 - 0.105]	5.761E-01	9.246E-01
Right superior temporal gyrus	0.030	0.046	[-0.061 - 0.121]	5.233E-01	9.246E-01
Left insula	-0.062	0.030	[-0.122 - -0.002]	4.117E-02	8.456E-01
Right insula	-0.070	0.030	[-0.129 - -0.011]	2.106E-02	7.477E-01
Left lingual gyrus	-0.015	0.035	[-0.085 - 0.054]	6.667E-01	9.246E-01
Right lingual gyrus	-0.007	0.035	[-0.075 - 0.062]	8.513E-01	9.444E-01
Left pars triangularis of inferior frontal gyrus	-0.038	0.030	[-0.097 - 0.022]	2.122E-01	9.246E-01
Right pars triangularis of inferior frontal gyrus	-0.040	0.030	[-0.099 - 0.019]	1.878E-01	9.246E-01
Left lateral orbitofrontal cortex	-0.020	0.036	[-0.092 - 0.051]	5.750E-01	9.246E-01
Right lateral orbitofrontal cortex	-0.039	0.030	[-0.099 - 0.020]	1.961E-01	9.246E-01
Left rostral middle frontal gyrus	-0.010	0.037	[-0.083 - 0.063]	7.814E-01	9.246E-01
Right rostral middle frontal gyrus	-0.014	0.033	[-0.079 - 0.052]	6.787E-01	9.246E-01
Left middle temporal gyrus	0.018	0.043	[-0.066 - 0.102]	6.798E-01	9.246E-01
Right middle temporal gyrus	0.030	0.030	[-0.029 - 0.090]	3.159E-01	9.246E-01
Left superior frontal gyrus	0.035	0.034	[-0.033 - 0.102]	3.110E-01	9.246E-01
Right superior frontal gyrus	0.011	0.031	[-0.050 - 0.071]	7.289E-01	9.246E-01
Left pars orbitalis of inferior frontal gyrus	-0.098	0.030	[-0.157 - -0.038]	1.257E-03	8.925E-02
Right pars orbitalis of inferior frontal gyrus	-0.040	0.031	[-0.101 - 0.021]	2.020E-01	9.246E-01
Left medial orbitofrontal cortex	-0.016	0.033	[-0.082 - 0.049]	6.255E-01	9.246E-01
Right medial orbitofrontal cortex	0.001	0.033	[-0.063 - 0.065]	9.651E-01	9.789E-01
Left inferior temporal gyrus	-0.023	0.038	[-0.097 - 0.051]	5.384E-01	9.246E-01
Right inferior temporal gyrus	0.011	0.039	[-0.066 - 0.088]	7.756E-01	9.246E-01
Left isthmus cingulate cortex	-0.066	0.042	[-0.149 - 0.017]	1.213E-01	9.246E-01
Right isthmus cingulate cortex	-0.064	0.037	[-0.136 - 0.008]	8.336E-02	9.246E-01
Left banks of superior temporal sulcus	-0.047	0.032	[-0.111 - 0.016]	1.445E-01	9.246E-01
Right banks of superior temporal sulcus	0.005	0.030	[-0.055 - 0.064]	8.784E-01	9.595E-01
Left supramarginal gyrus	0.014	0.034	[-0.052 - 0.081]	6.714E-01	9.246E-01
Right supramarginal gyrus	0.016	0.038	[-0.057 - 0.090]	6.652E-01	9.246E-01
Left caudal middle frontal gyrus	0.035	0.032	[-0.027 - 0.097]	2.704E-01	9.246E-01
Right caudal middle frontal gyrus	-0.023	0.033	[-0.088 - 0.043]	4.985E-01	9.246E-01
Left frontal pole	0.026	0.042	[-0.056 - 0.107]	5.388E-01	9.246E-01
Right frontal pole	-0.027	0.044	[-0.114 - 0.060]	5.447E-01	9.246E-01
Left posterior cingulate cortex	-0.025	0.030	[-0.084 - 0.034]	4.090E-01	9.246E-01
Right posterior cingulate cortex	-0.060	0.030	[-0.119 - -0.001]	4.764E-02	8.456E-01
Left lateral occipital cortex	-0.023	0.030	[-0.082 - 0.037]	4.579E-01	9.246E-01
Right lateral occipital cortex	-0.040	0.030	[-0.100 - 0.019]	1.850E-01	9.246E-01
Left precentral gyrus	0.026	0.037	[-0.046 - 0.098]	4.774E-01	9.246E-01
Right precentral gyrus	-0.014	0.030	[-0.073 - 0.046]	6.500E-01	9.246E-01
Left parahippocampal gyrus	-0.038	0.034	[-0.105 - 0.029]	2.674E-01	9.246E-01
Right parahippocampal gyrus	-0.026	0.043	[-0.110 - 0.057]	5.387E-01	9.246E-01
Left inferior parietal cortex	0.013	0.040	[-0.065 - 0.092]	7.376E-01	9.246E-01
Right inferior parietal cortex	-0.002	0.031	[-0.062 - 0.059]	9.520E-01	9.789E-01
Left transverse temporal gyrus	-0.010	0.044	[-0.097 - 0.076]	8.191E-01	9.422E-01
Right transverse temporal gyrus	-0.049	0.052	[-0.151 - 0.054]	3.525E-01	9.246E-01
Left postcentral gyrus	0.014	0.040	[-0.065 - 0.093]	7.322E-01	9.246E-01
Right postcentral gyrus	0.002	0.030	[-0.057 - 0.062]	9.451E-01	9.789E-01
Left precuneus	0.024	0.035	[-0.046 - 0.093]	5.011E-01	9.246E-01
Right precuneus	0.012	0.036	[-0.059 - 0.083]	7.388E-01	9.246E-01
Left caudal anterior cingulate cortex	-0.038	0.036	[-0.108 - 0.032]	2.889E-01	9.246E-01
Right caudal anterior cingulate cortex	-0.040	0.035	[-0.109 - 0.029]	2.581E-01	9.246E-01
Left cuneus	-0.013	0.042	[-0.095 - 0.070]	7.657E-01	9.246E-01
Right cuneus	-0.051	0.036	[-0.122 - 0.019]	1.556E-01	9.246E-01
Left rostral anterior cingulate cortex	-0.022	0.033	[-0.086 - 0.043]	5.087E-01	9.246E-01
Right rostral anterior cingulate cortex	-0.042	0.030	[-0.101 - 0.018]	1.682E-01	9.246E-01
Left pericalcarine cortex	-0.003	0.056	[-0.112 - 0.107]	9.606E-01	9.789E-01
Right pericalcarine cortex	0.009	0.046	[-0.080 - 0.099]	8.360E-01	9.422E-01
Left paracentral lobule	0.014	0.030	[-0.045 - 0.074]	6.332E-01	9.246E-01
Right paracentral lobule	0.000	0.030	[-0.060 - 0.059]	9.888E-01	9.888E-01
Left superior parietal cortex	0.024	0.037	[-0.048 - 0.096]	5.179E-01	9.246E-01
Right superior parietal cortex	0.016	0.036	[-0.055 - 0.087]	6.554E-01	9.246E-01
Left temporal pole	-0.045	0.044	[-0.132 - 0.041]	3.017E-01	9.246E-01
Right temporal pole	-0.023	0.047	[-0.114 - 0.069]	6.294E-01	9.246E-01
Left entorhinal cortex	-0.036	0.032	[-0.099 - 0.027]	2.628E-01	9.246E-01
Right entorhinal cortex	0.010	0.030	[-0.050 - 0.069]	7.462E-01	9.246E-01

Supplementary Table S71. Partial correlations between cortical thickness and duration of illness controlling for age and sex in individuals with bipolar disorder

	Partial *R*	Std. Err.	95% CI	*p*-value	FDR *q*-value
Global mean cortical thickness	-0.004	0.070	[-0.142 - 0.134]	9.580E-01	9.637E-01
Left hemisphere	-0.013	0.070	[-0.151 - 0.125]	8.572E-01	9.637E-01
Right hemisphere	0.005	0.070	[-0.132 - 0.143]	9.385E-01	9.637E-01
Left fusiform gyrus	0.045	0.070	[-0.093 - 0.182]	5.268E-01	9.350E-01
Right fusiform gyrus	0.019	0.085	[-0.147 - 0.185]	8.235E-01	9.637E-01
Left pars opercularis of inferior frontal gyrus	-0.155	0.070	[-0.293 - -0.017]	2.770E-02	6.364E-01
Right pars opercularis of inferior frontal gyrus	-0.008	0.070	[-0.146 - 0.130]	9.122E-01	9.637E-01
Left superior temporal gyrus	-0.111	0.070	[-0.249 - 0.027]	1.138E-01	6.364E-01
Right superior temporal gyrus	-0.121	0.070	[-0.259 - 0.017]	8.480E-02	6.364E-01
Left insula	-0.063	0.070	[-0.201 - 0.075]	3.719E-01	8.517E-01
Right insula	0.065	0.070	[-0.073 - 0.203]	3.543E-01	8.386E-01
Left lingual gyrus	0.014	0.100	[-0.182 - 0.211]	8.855E-01	9.637E-01
Right lingual gyrus	0.027	0.098	[-0.165 - 0.219]	7.799E-01	9.637E-01
Left pars triangularis of inferior frontal gyrus	-0.108	0.070	[-0.246 - 0.030]	1.250E-01	6.364E-01
Right pars triangularis of inferior frontal gyrus	-0.125	0.070	[-0.262 - 0.013]	7.665E-02	6.364E-01
Left lateral orbitofrontal cortex	-0.076	0.070	[-0.214 - 0.062]	2.808E-01	7.362E-01
Right lateral orbitofrontal cortex	-0.032	0.081	[-0.191 - 0.126]	6.892E-01	9.637E-01
Left rostral middle frontal gyrus	-0.112	0.070	[-0.250 - 0.026]	1.109E-01	6.364E-01
Right rostral middle frontal gyrus	-0.071	0.083	[-0.234 - 0.092]	3.939E-01	8.568E-01
Left middle temporal gyrus	-0.108	0.070	[-0.246 - 0.030]	1.255E-01	6.364E-01
Right middle temporal gyrus	-0.018	0.070	[-0.155 - 0.120]	8.028E-01	9.637E-01
Left superior frontal gyrus	-0.048	0.070	[-0.186 - 0.090]	4.964E-01	9.207E-01
Right superior frontal gyrus	-0.076	0.070	[-0.214 - 0.062]	2.791E-01	7.362E-01
Left pars orbitalis of inferior frontal gyrus	-0.016	0.070	[-0.154 - 0.121]	8.151E-01	9.637E-01
Right pars orbitalis of inferior frontal gyrus	-0.093	0.081	[-0.253 - 0.067]	2.528E-01	7.362E-01
Left medial orbitofrontal cortex	-0.035	0.070	[-0.173 - 0.103]	6.213E-01	9.637E-01
Right medial orbitofrontal cortex	-0.095	0.133	[-0.356 - 0.166]	4.748E-01	9.207E-01
Left inferior temporal gyrus	0.056	0.070	[-0.082 - 0.194]	4.252E-01	8.879E-01
Right inferior temporal gyrus	0.027	0.070	[-0.111 - 0.165]	7.008E-01	9.637E-01
Left isthmus cingulate cortex	0.139	0.082	[-0.021 - 0.298]	8.895E-02	6.364E-01
Right isthmus cingulate cortex	0.108	0.083	[-0.054 - 0.270]	1.928E-01	7.362E-01
Left banks of superior temporal sulcus	-0.084	0.070	[-0.222 - 0.054]	2.312E-01	7.362E-01
Right banks of superior temporal sulcus	-0.101	0.098	[-0.292 - 0.090]	3.007E-01	7.362E-01
Left supramarginal gyrus	0.013	0.070	[-0.125 - 0.151]	8.575E-01	9.637E-01
Right supramarginal gyrus	0.035	0.094	[-0.149 - 0.218]	7.110E-01	9.637E-01
Left caudal middle frontal gyrus	-0.048	0.070	[-0.186 - 0.090]	4.968E-01	9.207E-01
Right caudal middle frontal gyrus	-0.005	0.100	[-0.201 - 0.192]	9.637E-01	9.637E-01
Left frontal pole	-0.026	0.100	[-0.222 - 0.170]	7.979E-01	9.637E-01
Right frontal pole	-0.108	0.070	[-0.246 - 0.030]	1.248E-01	6.364E-01
Left posterior cingulate cortex	0.165	0.083	[0.003 - 0.327]	4.570E-02	6.364E-01
Right posterior cingulate cortex	0.095	0.075	[-0.051 - 0.242]	2.004E-01	7.362E-01
Left lateral occipital cortex	0.147	0.070	[0.009 - 0.285]	3.658E-02	6.364E-01
Right lateral occipital cortex	0.193	0.070	[0.055 - 0.331]	6.140E-03	4.359E-01
Left precentral gyrus	-0.041	0.070	[-0.179 - 0.097]	5.583E-01	9.637E-01
Right precentral gyrus	0.019	0.070	[-0.119 - 0.157]	7.831E-01	9.637E-01
Left parahippocampal gyrus	0.041	0.080	[-0.115 - 0.197]	6.068E-01	9.637E-01
Right parahippocampal gyrus	-0.078	0.070	[-0.216 - 0.060]	2.691E-01	7.362E-01
Left inferior parietal cortex	0.015	0.070	[-0.123 - 0.153]	8.347E-01	9.637E-01
Right inferior parietal cortex	0.028	0.070	[-0.110 - 0.165]	6.955E-01	9.637E-01
Left transverse temporal gyrus	-0.093	0.110	[-0.308 - 0.122]	3.982E-01	8.568E-01
Right transverse temporal gyrus	-0.096	0.077	[-0.247 - 0.056]	2.176E-01	7.362E-01
Left postcentral gyrus	0.061	0.087	[-0.109 - 0.231]	4.814E-01	9.207E-01
Right postcentral gyrus	-0.011	0.108	[-0.223 - 0.201]	9.206E-01	9.637E-01
Left precuneus	-0.034	0.105	[-0.241 - 0.172]	7.445E-01	9.637E-01
Right precuneus	-0.033	0.085	[-0.200 - 0.135]	7.013E-01	9.637E-01
Left caudal anterior cingulate cortex	-0.050	0.122	[-0.290 - 0.190]	6.816E-01	9.637E-01
Right caudal anterior cingulate cortex	0.122	0.070	[-0.016 - 0.260]	8.335E-02	6.364E-01
Left cuneus	0.108	0.086	[-0.060 - 0.276]	2.068E-01	7.362E-01
Right cuneus	0.045	0.081	[-0.113 - 0.203]	5.748E-01	9.637E-01
Left rostral anterior cingulate cortex	-0.073	0.070	[-0.211 - 0.065]	2.977E-01	7.362E-01
Right rostral anterior cingulate cortex	0.078	0.070	[-0.060 - 0.216]	2.681E-01	7.362E-01
Left pericalcarine cortex	0.099	0.081	[-0.059 - 0.256]	2.202E-01	7.362E-01
Right pericalcarine cortex	-0.138	0.119	[-0.372 - 0.096]	2.473E-01	7.362E-01
Left paracentral lobule	-0.018	0.113	[-0.239 - 0.203]	8.741E-01	9.637E-01
Right paracentral lobule	-0.026	0.084	[-0.190 - 0.139]	7.596E-01	9.637E-01
Left superior parietal cortex	-0.012	0.073	[-0.155 - 0.131]	8.708E-01	9.637E-01
Right superior parietal cortex	-0.078	0.117	[-0.308 - 0.152]	5.058E-01	9.207E-01
Left temporal pole	-0.114	0.070	[-0.252 - 0.024]	1.047E-01	6.364E-01
Right temporal pole	0.006	0.085	[-0.161 - 0.172]	9.451E-01	9.637E-01
Left entorhinal cortex	0.078	0.070	[-0.060 - 0.216]	2.682E-01	7.362E-01
Right entorhinal cortex	-0.005	0.070	[-0.143 - 0.133]	9.449E-01	9.637E-01

Supplementary Table S72. Partial correlations between cortical thickness and duration of illness controlling for age and sex in individuals with bipolar disorder aged 25 years and older

	Partial *R*	Std. Err.	95% CI	*p*-value	FDR *q*-value
Global mean cortical thickness	-0.005	0.074	[-0.150 - 0.139]	9.442E-01	9.671E-01
Left hemisphere	-0.015	0.074	[-0.159 - 0.130]	8.418E-01	9.671E-01
Right hemisphere	0.004	0.074	[-0.140 - 0.149]	9.535E-01	9.671E-01
Left fusiform gyrus	0.051	0.074	[-0.093 - 0.196]	4.861E-01	9.383E-01
Right fusiform gyrus	0.026	0.087	[-0.145 - 0.198]	7.657E-01	9.671E-01
Left pars opercularis of inferior frontal gyrus	-0.149	0.074	[-0.294 - -0.005]	4.309E-02	7.398E-01
Right pars opercularis of inferior frontal gyrus	-0.014	0.074	[-0.159 - 0.130]	8.444E-01	9.671E-01
Left superior temporal gyrus	-0.100	0.086	[-0.269 - 0.069]	2.481E-01	7.398E-01
Right superior temporal gyrus	-0.130	0.074	[-0.275 - 0.014]	7.709E-02	7.398E-01
Left insula	-0.060	0.074	[-0.205 - 0.084]	4.142E-01	8.911E-01
Right insula	0.074	0.074	[-0.070 - 0.219]	3.154E-01	7.712E-01
Left lingual gyrus	0.009	0.102	[-0.190 - 0.209]	9.270E-01	9.671E-01
Right lingual gyrus	0.031	0.101	[-0.168 - 0.230]	7.581E-01	9.671E-01
Left pars triangularis of inferior frontal gyrus	-0.104	0.074	[-0.248 - 0.041]	1.591E-01	7.398E-01
Right pars triangularis of inferior frontal gyrus	-0.123	0.074	[-0.267 - 0.021]	9.520E-02	7.398E-01
Left lateral orbitofrontal cortex	-0.073	0.074	[-0.218 - 0.071]	3.196E-01	7.712E-01
Right lateral orbitofrontal cortex	-0.027	0.082	[-0.187 - 0.133]	7.372E-01	9.671E-01
Left rostral middle frontal gyrus	-0.111	0.074	[-0.256 - 0.033]	1.312E-01	7.398E-01
Right rostral middle frontal gyrus	-0.081	0.083	[-0.243 - 0.081]	3.259E-01	7.712E-01
Left middle temporal gyrus	-0.110	0.074	[-0.254 - 0.035]	1.372E-01	7.398E-01
Right middle temporal gyrus	-0.024	0.074	[-0.168 - 0.121]	7.495E-01	9.671E-01
Left superior frontal gyrus	-0.056	0.074	[-0.200 - 0.089]	4.481E-01	9.091E-01
Right superior frontal gyrus	-0.079	0.074	[-0.223 - 0.066]	2.869E-01	7.712E-01
Left pars orbitalis of inferior frontal gyrus	0.000	0.074	[-0.144 - 0.145]	9.990E-01	9.990E-01
Right pars orbitalis of inferior frontal gyrus	-0.097	0.083	[-0.260 - 0.066]	2.432E-01	7.398E-01
Left medial orbitofrontal cortex	-0.039	0.074	[-0.183 - 0.106]	5.991E-01	9.614E-01
Right medial orbitofrontal cortex	-0.086	0.136	[-0.352 - 0.180]	5.258E-01	9.383E-01
Left inferior temporal gyrus	0.056	0.074	[-0.089 - 0.200]	4.476E-01	9.091E-01
Right inferior temporal gyrus	0.036	0.074	[-0.109 - 0.180]	6.272E-01	9.671E-01
Left isthmus cingulate cortex	0.145	0.087	[-0.025 - 0.315]	9.445E-02	7.398E-01
Right isthmus cingulate cortex	0.124	0.095	[-0.062 - 0.309]	1.922E-01	7.398E-01
Left banks of superior temporal sulcus	-0.097	0.074	[-0.241 - 0.048]	1.895E-01	7.398E-01
Right banks of superior temporal sulcus	-0.098	0.103	[-0.300 - 0.104]	3.421E-01	7.835E-01
Left supramarginal gyrus	0.015	0.074	[-0.130 - 0.159]	8.394E-01	9.671E-01
Right supramarginal gyrus	0.041	0.095	[-0.146 - 0.228]	6.686E-01	9.671E-01
Left caudal middle frontal gyrus	-0.051	0.074	[-0.195 - 0.094]	4.893E-01	9.383E-01
Right caudal middle frontal gyrus	-0.012	0.106	[-0.219 - 0.195]	9.105E-01	9.671E-01
Left frontal pole	-0.011	0.107	[-0.221 - 0.199]	9.194E-01	9.671E-01
Right frontal pole	-0.114	0.074	[-0.259 - 0.030]	1.214E-01	7.398E-01
Left posterior cingulate cortex	0.186	0.093	[0.003 - 0.369]	4.654E-02	7.398E-01
Right posterior cingulate cortex	0.097	0.078	[-0.057 - 0.250]	2.173E-01	7.398E-01
Left lateral occipital cortex	0.151	0.074	[0.006 - 0.295]	4.115E-02	7.398E-01
Right lateral occipital cortex	0.196	0.074	[0.050 - 0.342]	8.422E-03	5.980E-01
Left precentral gyrus	-0.042	0.074	[-0.187 - 0.102]	5.683E-01	9.383E-01
Right precentral gyrus	0.010	0.074	[-0.135 - 0.154]	8.952E-01	9.671E-01
Left parahippocampal gyrus	0.049	0.083	[-0.113 - 0.211]	5.505E-01	9.383E-01
Right parahippocampal gyrus	-0.088	0.074	[-0.232 - 0.057]	2.343E-01	7.398E-01
Left inferior parietal cortex	0.015	0.074	[-0.129 - 0.160]	8.366E-01	9.671E-01
Right inferior parietal cortex	0.038	0.074	[-0.107 - 0.182]	6.094E-01	9.614E-01
Left transverse temporal gyrus	-0.094	0.114	[-0.318 - 0.129]	4.088E-01	8.911E-01
Right transverse temporal gyrus	-0.095	0.078	[-0.248 - 0.058]	2.224E-01	7.398E-01
Left postcentral gyrus	0.055	0.090	[-0.121 - 0.231]	5.392E-01	9.383E-01
Right postcentral gyrus	-0.025	0.112	[-0.244 - 0.195]	8.264E-01	9.671E-01
Left precuneus	-0.052	0.111	[-0.269 - 0.166]	6.415E-01	9.671E-01
Right precuneus	-0.040	0.090	[-0.215 - 0.136]	6.567E-01	9.671E-01
Left caudal anterior cingulate cortex	-0.044	0.125	[-0.289 - 0.201]	7.243E-01	9.671E-01
Right caudal anterior cingulate cortex	0.134	0.074	[-0.011 - 0.278]	6.944E-02	7.398E-01
Left cuneus	0.103	0.090	[-0.072 - 0.278]	2.501E-01	7.398E-01
Right cuneus	0.047	0.080	[-0.111 - 0.204]	5.598E-01	9.383E-01
Left rostral anterior cingulate cortex	-0.076	0.074	[-0.221 - 0.068]	3.011E-01	7.712E-01
Right rostral anterior cingulate cortex	0.091	0.074	[-0.053 - 0.236]	2.168E-01	7.398E-01
Left pericalcarine cortex	0.089	0.084	[-0.076 - 0.255]	2.893E-01	7.712E-01
Right pericalcarine cortex	-0.148	0.120	[-0.383 - 0.088]	2.186E-01	7.398E-01
Left paracentral lobule	-0.016	0.115	[-0.242 - 0.210]	8.892E-01	9.671E-01
Right paracentral lobule	-0.028	0.087	[-0.199 - 0.143]	7.458E-01	9.671E-01
Left superior parietal cortex	-0.012	0.076	[-0.162 - 0.137]	8.706E-01	9.671E-01
Right superior parietal cortex	-0.079	0.122	[-0.318 - 0.160]	5.185E-01	9.383E-01
Left temporal pole	-0.111	0.074	[-0.256 - 0.033]	1.305E-01	7.398E-01
Right temporal pole	0.009	0.084	[-0.156 - 0.174]	9.147E-01	9.671E-01
Left entorhinal cortex	0.095	0.074	[-0.050 - 0.239]	1.985E-01	7.398E-01
Right entorhinal cortex	0.005	0.074	[-0.140 - 0.149]	9.502E-01	9.671E-01

Supplementary Table S73. Cortical thickness differences between individuals with major depressive disorder with a late age of onset (over 21 years old; LAO) versus healthy comparison subjects controlling for age and sex

	d	Std. Err.	95% CI	*p*-value	FDR *q*-value
Global mean cortical thickness	-0.253	0.141	[-0.530 - 0.024]	7.390E-02	2.087E-01
Left hemisphere	-0.268	0.146	[-0.553 - 0.018]	6.587E-02	2.033E-01
Right hemisphere	-0.226	0.134	[-0.489 - 0.036]	9.133E-02	2.162E-01
Left fusiform gyrus	-0.174	0.112	[-0.393 - 0.046]	1.210E-01	2.481E-01
Right fusiform gyrus	-0.204	0.093	[-0.386 - -0.021]	2.856E-02	1.480E-01
Left pars opercularis of inferior frontal gyrus	-0.234	0.107	[-0.444 - -0.024]	2.919E-02	1.480E-01
Right pars opercularis of inferior frontal gyrus	-0.161	0.124	[-0.403 - 0.082]	1.940E-01	3.060E-01
Left superior temporal gyrus	-0.243	0.100	[-0.440 - -0.046]	1.542E-02	1.217E-01
Right superior temporal gyrus	-0.183	0.105	[-0.389 - 0.023]	8.231E-02	2.087E-01
Left insula	-0.185	0.108	[-0.397 - 0.027]	8.726E-02	2.136E-01
Right insula	-0.178	0.126	[-0.425 - 0.068]	1.566E-01	2.712E-01
Left lingual gyrus	-0.163	0.099	[-0.356 - 0.031]	9.987E-02	2.287E-01
Right lingual gyrus	-0.149	0.104	[-0.353 - 0.054]	1.496E-01	2.712E-01
Left pars triangularis of inferior frontal gyrus	-0.169	0.109	[-0.382 - 0.045]	1.223E-01	2.481E-01
Right pars triangularis of inferior frontal gyrus	-0.231	0.110	[-0.447 - -0.014]	3.667E-02	1.735E-01
Left lateral orbitofrontal cortex	-0.311	0.115	[-0.538 - -0.085]	7.025E-03	8.699E-02
Right lateral orbitofrontal cortex	-0.251	0.112	[-0.470 - -0.032]	2.441E-02	1.444E-01
Left rostral middle frontal gyrus	-0.317	0.139	[-0.588 - -0.045]	2.237E-02	1.444E-01
Right rostral middle frontal gyrus	-0.208	0.115	[-0.433 - 0.018]	7.156E-02	2.087E-01
Left middle temporal gyrus	-0.353	0.118	[-0.583 - -0.122]	2.727E-03	6.455E-02
Right middle temporal gyrus	-0.271	0.115	[-0.496 - -0.045]	1.872E-02	1.329E-01
Left superior frontal gyrus	-0.223	0.120	[-0.458 - 0.011]	6.165E-02	1.990E-01
Right superior frontal gyrus	-0.241	0.093	[-0.422 - -0.059]	9.331E-03	8.699E-02
Left pars orbitalis of inferior frontal gyrus	-0.291	0.111	[-0.508 - -0.074]	8.685E-03	8.699E-02
Right pars orbitalis of inferior frontal gyrus	-0.302	0.098	[-0.493 - -0.111]	1.959E-03	6.455E-02
Left medial orbitofrontal cortex	-0.253	0.126	[-0.501 - -0.006]	4.452E-02	1.859E-01
Right medial orbitofrontal cortex	-0.261	0.094	[-0.445 - -0.076]	5.621E-03	8.699E-02
Left inferior temporal gyrus	-0.179	0.091	[-0.358 - -0.001]	4.844E-02	1.866E-01
Right inferior temporal gyrus	-0.203	0.079	[-0.358 - -0.049]	9.802E-03	8.699E-02
Left isthmus cingulate cortex	-0.118	0.091	[-0.297 - 0.060]	1.934E-01	3.060E-01
Right isthmus cingulate cortex	-0.110	0.094	[-0.295 - 0.074]	2.405E-01	3.553E-01
Left banks of superior temporal sulcus	-0.167	0.096	[-0.356 - 0.021]	8.196E-02	2.087E-01
Right banks of superior temporal sulcus	-0.093	0.118	[-0.323 - 0.137]	4.279E-01	5.426E-01
Left supramarginal gyrus	-0.187	0.107	[-0.396 - 0.022]	7.928E-02	2.087E-01
Right supramarginal gyrus	-0.145	0.109	[-0.358 - 0.068]	1.817E-01	3.001E-01
Left caudal middle frontal gyrus	-0.134	0.126	[-0.380 - 0.112]	2.866E-01	4.070E-01
Right caudal middle frontal gyrus	-0.193	0.102	[-0.393 - 0.006]	5.755E-02	1.946E-01
Left frontal pole	-0.159	0.081	[-0.319 - 0.000]	4.994E-02	1.866E-01
Right frontal pole	-0.182	0.120	[-0.417 - 0.054]	1.305E-01	2.574E-01
Left posterior cingulate cortex	-0.216	0.071	[-0.356 - -0.077]	2.385E-03	6.455E-02
Right posterior cingulate cortex	-0.167	0.087	[-0.338 - 0.004]	5.541E-02	1.946E-01
Left lateral occipital cortex	-0.144	0.093	[-0.328 - 0.039]	1.219E-01	2.481E-01
Right lateral occipital cortex	-0.123	0.105	[-0.329 - 0.084]	2.452E-01	3.553E-01
Left precentral gyrus	-0.208	0.102	[-0.408 - -0.008]	4.164E-02	1.848E-01
Right precentral gyrus	-0.150	0.095	[-0.336 - 0.036]	1.148E-01	2.481E-01
Left parahippocampal gyrus	-0.129	0.110	[-0.345 - 0.087]	2.403E-01	3.553E-01
Right parahippocampal gyrus	-0.033	0.089	[-0.207 - 0.142]	7.112E-01	7.890E-01
Left inferior parietal cortex	-0.124	0.103	[-0.326 - 0.079]	2.317E-01	3.553E-01
Right inferior parietal cortex	-0.141	0.105	[-0.347 - 0.065]	1.811E-01	3.001E-01
Left transverse temporal gyrus	-0.006	0.113	[-0.226 - 0.215]	9.603E-01	9.627E-01
Right transverse temporal gyrus	-0.033	0.110	[-0.249 - 0.182]	7.604E-01	8.180E-01
Left postcentral gyrus	-0.093	0.109	[-0.307 - 0.121]	3.949E-01	5.098E-01
Right postcentral gyrus	-0.109	0.108	[-0.322 - 0.103]	3.129E-01	4.272E-01
Left precuneus	-0.074	0.115	[-0.299 - 0.150]	5.164E-01	6.321E-01
Right precuneus	-0.122	0.083	[-0.285 - 0.041]	1.431E-01	2.674E-01
Left caudal anterior cingulate cortex	-0.098	0.066	[-0.228 - 0.031]	1.370E-01	2.628E-01
Right caudal anterior cingulate cortex	-0.070	0.070	[-0.208 - 0.068]	3.194E-01	4.278E-01
Left cuneus	-0.083	0.080	[-0.240 - 0.074]	2.999E-01	4.175E-01
Right cuneus	-0.063	0.066	[-0.193 - 0.067]	3.452E-01	4.538E-01
Left rostral anterior cingulate cortex	-0.136	0.096	[-0.325 - 0.052]	1.555E-01	2.712E-01
Right rostral anterior cingulate cortex	0.021	0.091	[-0.157 - 0.199]	8.184E-01	8.673E-01
Left pericalcarine cortex	-0.031	0.069	[-0.166 - 0.105]	6.574E-01	7.528E-01
Right pericalcarine cortex	0.014	0.093	[-0.170 - 0.197]	8.851E-01	9.241E-01
Left paracentral lobule	-0.066	0.089	[-0.241 - 0.109]	4.623E-01	5.758E-01
Right paracentral lobule	-0.052	0.109	[-0.266 - 0.162]	6.359E-01	7.401E-01
Left superior parietal cortex	-0.042	0.105	[-0.248 - 0.164]	6.892E-01	7.767E-01
Right superior parietal cortex	-0.013	0.101	[-0.212 - 0.186]	8.999E-01	9.260E-01
Left temporal pole	-0.054	0.086	[-0.224 - 0.115]	5.305E-01	6.384E-01
Right temporal pole	0.049	0.096	[-0.140 - 0.237]	6.112E-01	7.232E-01
Left entorhinal cortex	-0.005	0.097	[-0.195 - 0.186]	9.627E-01	9.627E-01
Right entorhinal cortex	-0.041	0.117	[-0.270 - 0.188]	7.251E-01	7.920E-01

Supplementary Table S74. Cortical thickness differences between individuals with major depressive disorder with an early age of onset (21 years of old or younger; EAO) versus healthy comparison subjects controlling for age and sex

	d	Std. Err.	95% CI	*p*-value	FDR *q*-value
Global mean cortical thickness	-0.398	0.211	[-0.812 - 0.016]	5.954E-02	3.523E-01
Left hemisphere	-0.433	0.219	[-0.862 - -0.003]	4.827E-02	3.115E-01
Right hemisphere	-0.346	0.197	[-0.733 - 0.040]	7.895E-02	3.737E-01
Left fusiform gyrus	-0.303	0.197	[-0.688 - 0.082]	1.235E-01	3.881E-01
Right fusiform gyrus	-0.279	0.196	[-0.664 - 0.106]	1.550E-01	3.881E-01
Left pars opercularis of inferior frontal gyrus	-0.549	0.270	[-1.077 - -0.020]	4.205E-02	2.986E-01
Right pars opercularis of inferior frontal gyrus	-0.281	0.197	[-0.666 - 0.105]	1.539E-01	3.881E-01
Left superior temporal gyrus	-0.524	0.317	[-1.145 - 0.098]	9.856E-02	3.881E-01
Right superior temporal gyrus	-0.235	0.197	[-0.621 - 0.150]	2.320E-01	4.334E-01
Left insula	-0.592	0.199	[-0.982 - -0.202]	2.935E-03	5.983E-02
Right insula	-0.339	0.198	[-0.727 - 0.048]	8.588E-02	3.810E-01
Left lingual gyrus	-0.200	0.197	[-0.586 - 0.187]	3.114E-01	5.142E-01
Right lingual gyrus	0.117	0.197	[-0.268 - 0.502]	5.514E-01	6.991E-01
Left pars triangularis of inferior frontal gyrus	0.036	0.201	[-0.358 - 0.430]	8.580E-01	9.014E-01
Right pars triangularis of inferior frontal gyrus	-0.190	0.420	[-1.014 - 0.634]	6.518E-01	7.713E-01
Left lateral orbitofrontal cortex	-0.233	0.310	[-0.839 - 0.374]	4.525E-01	6.178E-01
Right lateral orbitofrontal cortex	-0.259	0.294	[-0.836 - 0.317]	3.780E-01	5.725E-01
Left rostral middle frontal gyrus	-0.342	0.233	[-0.799 - 0.115]	1.427E-01	3.881E-01
Right rostral middle frontal gyrus	0.075	0.244	[-0.403 - 0.554]	7.574E-01	8.815E-01
Left middle temporal gyrus	-0.407	0.338	[-1.070 - 0.255]	2.283E-01	4.334E-01
Right middle temporal gyrus	-0.511	0.243	[-0.988 - -0.034]	3.558E-02	2.986E-01
Left superior frontal gyrus	-0.286	0.251	[-0.777 - 0.205]	2.541E-01	4.510E-01
Right superior frontal gyrus	-0.173	0.225	[-0.615 - 0.269]	4.420E-01	6.153E-01
Left pars orbitalis of inferior frontal gyrus	0.000	0.196	[-0.385 - 0.384]	9.981E-01	9.981E-01
Right pars orbitalis of inferior frontal gyrus	-0.057	0.259	[-0.565 - 0.451]	8.260E-01	8.942E-01
Left medial orbitofrontal cortex	-0.063	0.218	[-0.490 - 0.364]	7.712E-01	8.832E-01
Right medial orbitofrontal cortex	-0.176	0.217	[-0.601 - 0.249]	4.181E-01	6.058E-01
Left inferior temporal gyrus	-0.045	0.196	[-0.430 - 0.340]	8.185E-01	8.942E-01
Right inferior temporal gyrus	-0.221	0.251	[-0.714 - 0.272]	3.790E-01	5.725E-01
Left isthmus cingulate cortex	-0.104	0.197	[-0.490 - 0.281]	5.963E-01	7.428E-01
Right isthmus cingulate cortex	-0.216	0.224	[-0.656 - 0.223]	3.343E-01	5.395E-01
Left banks of superior temporal sulcus	-0.441	0.217	[-0.866 - -0.017]	4.173E-02	2.986E-01
Right banks of superior temporal sulcus	-0.300	0.197	[-0.686 - 0.086]	1.276E-01	3.881E-01
Left supramarginal gyrus	-0.647	0.206	[-1.051 - -0.243]	1.703E-03	5.983E-02
Right supramarginal gyrus	-0.301	0.197	[-0.687 - 0.085]	1.268E-01	3.881E-01
Left caudal middle frontal gyrus	-0.368	0.347	[-1.048 - 0.312]	2.886E-01	4.998E-01
Right caudal middle frontal gyrus	-0.334	0.198	[-0.721 - 0.054]	9.123E-02	3.810E-01
Left frontal pole	-0.258	0.196	[-0.643 - 0.127]	1.897E-01	4.154E-01
Right frontal pole	-0.354	0.197	[-0.740 - 0.032]	7.208E-02	3.688E-01
Left posterior cingulate cortex	-0.314	0.234	[-0.773 - 0.145]	1.794E-01	4.110E-01
Right posterior cingulate cortex	-0.098	0.196	[-0.482 - 0.287]	6.184E-01	7.489E-01
Left lateral occipital cortex	0.124	0.196	[-0.261 - 0.509]	5.273E-01	6.807E-01
Right lateral occipital cortex	-0.042	0.196	[-0.426 - 0.342]	8.312E-01	8.942E-01
Left precentral gyrus	-0.602	0.236	[-1.065 - -0.139]	1.085E-02	1.541E-01
Right precentral gyrus	-0.446	0.203	[-0.843 - -0.049]	2.779E-02	2.819E-01
Left parahippocampal gyrus	-0.278	0.197	[-0.665 - 0.108]	1.585E-01	3.881E-01
Right parahippocampal gyrus	0.025	0.199	[-0.365 - 0.415]	8.987E-01	9.176E-01
Left inferior parietal cortex	-0.272	0.224	[-0.711 - 0.167]	2.241E-01	4.334E-01
Right inferior parietal cortex	-0.199	0.197	[-0.585 - 0.186]	3.106E-01	5.142E-01
Left transverse temporal gyrus	-0.427	0.354	[-1.122 - 0.267]	2.277E-01	4.334E-01
Right transverse temporal gyrus	-0.198	0.276	[-0.739 - 0.343]	4.735E-01	6.344E-01
Left postcentral gyrus	-0.603	0.206	[-1.005 - -0.200]	3.371E-03	5.983E-02
Right postcentral gyrus	-0.706	0.199	[-1.095 - -0.316]	3.909E-04	2.776E-02
Left precuneus	-0.034	0.196	[-0.418 - 0.351]	8.633E-01	9.014E-01
Right precuneus	-0.240	0.310	[-0.847 - 0.366]	4.373E-01	6.153E-01
Left caudal anterior cingulate cortex	-0.292	0.197	[-0.679 - 0.094]	1.385E-01	3.881E-01
Right caudal anterior cingulate cortex	-0.300	0.203	[-0.699 - 0.098]	1.392E-01	3.881E-01
Left cuneus	0.067	0.302	[-0.525 - 0.658]	8.252E-01	8.942E-01
Right cuneus	0.170	0.257	[-0.333 - 0.673]	5.075E-01	6.673E-01
Left rostral anterior cingulate cortex	-0.143	0.290	[-0.712 - 0.426]	6.223E-01	7.489E-01
Right rostral anterior cingulate cortex	-0.353	0.197	[-0.739 - 0.033]	7.273E-02	3.688E-01
Left pericalcarine cortex	-0.039	0.325	[-0.677 - 0.599]	9.046E-01	9.176E-01
Right pericalcarine cortex	0.340	0.265	[-0.179 - 0.859]	1.989E-01	4.154E-01
Left paracentral lobule	-0.230	0.197	[-0.615 - 0.156]	2.431E-01	4.426E-01
Right paracentral lobule	-0.281	0.197	[-0.668 - 0.105]	1.534E-01	3.881E-01
Left superior parietal cortex	-0.211	0.257	[-0.715 - 0.293]	4.116E-01	6.058E-01
Right superior parietal cortex	-0.282	0.197	[-0.668 - 0.104]	1.518E-01	3.881E-01
Left temporal pole	-0.473	0.199	[-0.862 - -0.083]	1.733E-02	2.050E-01
Right temporal pole	-0.182	0.197	[-0.568 - 0.203]	3.542E-01	5.588E-01
Left entorhinal cortex	-0.255	0.197	[-0.641 - 0.131]	1.957E-01	4.154E-01
Right entorhinal cortex	-0.272	0.197	[-0.658 - 0.113]	1.663E-01	3.935E-01

Supplementary Table S75. Cortical thickness differences between individuals with major depressive disorder with an early age of onset (21 years of old or younger; EAO) versus individuals with major depressive disorder with a late age of onset (over 21 years old; LAO) controlling for age and sex

	d	Std. Err.	95% CI	*p*-value	FDR *q*-value
Global mean cortical thickness	-0.319	0.195	[-0.702 - 0.063]	1.019E-01	5.103E-01
Left hemisphere	-0.354	0.195	[-0.737 - 0.029]	6.999E-02	4.925E-01
Right hemisphere	-0.273	0.195	[-0.655 - 0.109]	1.609E-01	5.485E-01
Left fusiform gyrus	-0.122	0.194	[-0.504 - 0.259]	5.293E-01	8.169E-01
Right fusiform gyrus	-0.250	0.195	[-0.632 - 0.132]	2.001E-01	5.485E-01
Left pars opercularis of inferior frontal gyrus	-0.337	0.196	[-0.720 - 0.047]	8.520E-02	4.925E-01
Right pars opercularis of inferior frontal gyrus	-0.330	0.195	[-0.712 - 0.052]	9.018E-02	4.925E-01
Left superior temporal gyrus	-0.481	0.270	[-1.009 - 0.048]	7.465E-02	4.925E-01
Right superior temporal gyrus	-0.245	0.195	[-0.627 - 0.137]	2.089E-01	5.485E-01
Left insula	-0.274	0.195	[-0.656 - 0.107]	1.587E-01	5.485E-01
Right insula	-0.297	0.238	[-0.764 - 0.170]	2.119E-01	5.485E-01
Left lingual gyrus	-0.041	0.194	[-0.422 - 0.340]	8.345E-01	8.843E-01
Right lingual gyrus	0.111	0.195	[-0.271 - 0.493]	5.687E-01	8.227E-01
Left pars triangularis of inferior frontal gyrus	0.066	0.194	[-0.316 - 0.447]	7.361E-01	8.633E-01
Right pars triangularis of inferior frontal gyrus	-0.006	0.366	[-0.722 - 0.711]	9.880E-01	9.880E-01
Left lateral orbitofrontal cortex	0.097	0.195	[-0.285 - 0.479]	6.199E-01	8.227E-01
Right lateral orbitofrontal cortex	0.044	0.195	[-0.337 - 0.426]	8.193E-01	8.843E-01
Left rostral middle frontal gyrus	-0.200	0.195	[-0.581 - 0.181]	3.041E-01	6.351E-01
Right rostral middle frontal gyrus	0.185	0.195	[-0.197 - 0.567]	3.422E-01	6.749E-01
Left middle temporal gyrus	-0.392	0.270	[-0.922 - 0.138]	1.471E-01	5.485E-01
Right middle temporal gyrus	-0.500	0.196	[-0.885 - -0.115]	1.089E-02	1.932E-01
Left superior frontal gyrus	-0.218	0.204	[-0.617 - 0.181]	2.844E-01	6.118E-01
Right superior frontal gyrus	-0.042	0.195	[-0.424 - 0.341]	8.303E-01	8.843E-01
Left pars orbitalis of inferior frontal gyrus	0.098	0.194	[-0.283 - 0.479]	6.149E-01	8.227E-01
Right pars orbitalis of inferior frontal gyrus	0.213	0.195	[-0.169 - 0.594]	2.747E-01	6.095E-01
Left medial orbitofrontal cortex	0.119	0.226	[-0.324 - 0.563]	5.975E-01	8.227E-01
Right medial orbitofrontal cortex	-0.053	0.195	[-0.435 - 0.328]	7.837E-01	8.718E-01
Left inferior temporal gyrus	-0.066	0.194	[-0.447 - 0.315]	7.328E-01	8.633E-01
Right inferior temporal gyrus	-0.242	0.196	[-0.625 - 0.142]	2.163E-01	5.485E-01
Left isthmus cingulate cortex	-0.262	0.216	[-0.686 - 0.162]	2.257E-01	5.485E-01
Right isthmus cingulate cortex	-0.180	0.257	[-0.683 - 0.324]	4.840E-01	7.991E-01
Left banks of superior temporal sulcus	-0.479	0.214	[-0.898 - -0.061]	2.485E-02	2.940E-01
Right banks of superior temporal sulcus	-0.383	0.195	[-0.766 - -0.001]	4.956E-02	4.925E-01
Left supramarginal gyrus	-0.824	0.198	[-1.212 - -0.436]	3.114E-05	1.106E-03
Right supramarginal gyrus	-0.300	0.195	[-0.683 - 0.083]	1.243E-01	5.485E-01
Left caudal middle frontal gyrus	-0.290	0.226	[-0.734 - 0.154]	2.003E-01	5.485E-01
Right caudal middle frontal gyrus	-0.015	0.194	[-0.396 - 0.366]	9.397E-01	9.669E-01
Left frontal pole	-0.156	0.194	[-0.537 - 0.225]	4.211E-01	7.475E-01
Right frontal pole	-0.229	0.195	[-0.610 - 0.152]	2.395E-01	5.485E-01
Left posterior cingulate cortex	-0.278	0.195	[-0.661 - 0.105]	1.544E-01	5.485E-01
Right posterior cingulate cortex	0.067	0.195	[-0.315 - 0.448]	7.322E-01	8.633E-01
Left lateral occipital cortex	0.106	0.194	[-0.275 - 0.487]	5.843E-01	8.227E-01
Right lateral occipital cortex	-0.004	0.194	[-0.385 - 0.376]	9.817E-01	9.880E-01
Left precentral gyrus	-0.341	0.195	[-0.723 - 0.042]	8.094E-02	4.925E-01
Right precentral gyrus	-0.167	0.194	[-0.548 - 0.214]	3.902E-01	7.475E-01
Left parahippocampal gyrus	-0.019	0.194	[-0.399 - 0.362]	9.237E-01	9.645E-01
Right parahippocampal gyrus	-0.130	0.195	[-0.511 - 0.252]	5.050E-01	8.149E-01
Left inferior parietal cortex	-0.360	0.224	[-0.799 - 0.079]	1.078E-01	5.103E-01
Right inferior parietal cortex	-0.252	0.195	[-0.633 - 0.130]	1.963E-01	5.485E-01
Left transverse temporal gyrus	-0.328	0.407	[-1.127 - 0.470]	4.207E-01	7.475E-01
Right transverse temporal gyrus	-0.183	0.220	[-0.613 - 0.247]	4.040E-01	7.475E-01
Left postcentral gyrus	-0.688	0.197	[-1.074 - -0.302]	4.766E-04	1.128E-02
Right postcentral gyrus	-0.843	0.198	[-1.232 - -0.455]	2.077E-05	1.106E-03
Left precuneus	-0.143	0.195	[-0.525 - 0.239]	4.619E-01	7.808E-01
Right precuneus	-0.161	0.254	[-0.659 - 0.337]	5.266E-01	8.169E-01
Left caudal anterior cingulate cortex	-0.195	0.194	[-0.576 - 0.186]	3.156E-01	6.403E-01
Right caudal anterior cingulate cortex	-0.098	0.217	[-0.523 - 0.327]	6.516E-01	8.227E-01
Left cuneus	0.104	0.195	[-0.278 - 0.485]	5.941E-01	8.227E-01
Right cuneus	0.109	0.195	[-0.272 - 0.491]	5.738E-01	8.227E-01
Left rostral anterior cingulate cortex	0.065	0.239	[-0.403 - 0.533]	7.859E-01	8.718E-01
Right rostral anterior cingulate cortex	-0.479	0.196	[-0.863 - -0.096]	1.426E-02	2.025E-01
Left pericalcarine cortex	-0.085	0.270	[-0.613 - 0.444]	7.538E-01	8.633E-01
Right pericalcarine cortex	0.120	0.263	[-0.395 - 0.635]	6.479E-01	8.227E-01
Left paracentral lobule	-0.085	0.194	[-0.467 - 0.296]	6.604E-01	8.227E-01
Right paracentral lobule	-0.232	0.195	[-0.614 - 0.150]	2.333E-01	5.485E-01
Left superior parietal cortex	-0.263	0.195	[-0.645 - 0.120]	1.781E-01	5.485E-01
Right superior parietal cortex	-0.337	0.196	[-0.720 - 0.047]	8.523E-02	4.925E-01
Left temporal pole	-0.290	0.246	[-0.772 - 0.193]	2.395E-01	5.485E-01
Right temporal pole	-0.151	0.195	[-0.533 - 0.230]	4.376E-01	7.577E-01
Left entorhinal cortex	-0.092	0.194	[-0.473 - 0.289]	6.350E-01	8.227E-01
Right entorhinal cortex	-0.062	0.194	[-0.443 - 0.319]	7.493E-01	8.633E-01

Supplementary Table S76. Partial correlations between cortical thickness and duration of illness controlling for age and sex in individuals with major depressive disorder

	Partial *R*	Std. Err.	95% CI	*p*-value	FDR *q*-value
Global mean cortical thickness	0.001	0.043	[-0.083 - 0.086]	9.747E-01	9.979E-01
Left hemisphere	-0.013	0.043	[-0.098 - 0.071]	7.568E-01	9.846E-01
Right hemisphere	0.017	0.043	[-0.068 - 0.101]	6.988E-01	9.846E-01
Left fusiform gyrus	0.021	0.043	[-0.064 - 0.106]	6.251E-01	9.846E-01
Right fusiform gyrus	0.069	0.043	[-0.016 - 0.154]	1.097E-01	9.846E-01
Left pars opercularis of inferior frontal gyrus	-0.044	0.043	[-0.129 - 0.040]	3.050E-01	9.846E-01
Right pars opercularis of inferior frontal gyrus	-0.013	0.043	[-0.098 - 0.072]	7.616E-01	9.846E-01
Left superior temporal gyrus	-0.033	0.045	[-0.122 - 0.055]	4.606E-01	9.846E-01
Right superior temporal gyrus	-0.037	0.043	[-0.122 - 0.048]	3.906E-01	9.846E-01
Left insula	-0.046	0.043	[-0.131 - 0.039]	2.897E-01	9.846E-01
Right insula	-0.059	0.045	[-0.148 - 0.030]	1.922E-01	9.846E-01
Left lingual gyrus	-0.041	0.043	[-0.126 - 0.044]	3.451E-01	9.846E-01
Right lingual gyrus	0.064	0.054	[-0.041 - 0.169]	2.346E-01	9.846E-01
Left pars triangularis of inferior frontal gyrus	-0.013	0.061	[-0.133 - 0.107]	8.320E-01	9.846E-01
Right pars triangularis of inferior frontal gyrus	0.022	0.043	[-0.062 - 0.107]	6.060E-01	9.846E-01
Left lateral orbitofrontal cortex	-0.047	0.043	[-0.131 - 0.038]	2.803E-01	9.846E-01
Right lateral orbitofrontal cortex	-0.021	0.043	[-0.105 - 0.064]	6.330E-01	9.846E-01
Left rostral middle frontal gyrus	0.009	0.054	[-0.097 - 0.115]	8.640E-01	9.968E-01
Right rostral middle frontal gyrus	0.085	0.043	[0.001 - 0.170]	4.825E-02	9.846E-01
Left middle temporal gyrus	0.030	0.065	[-0.098 - 0.158]	6.464E-01	9.846E-01
Right middle temporal gyrus	-0.046	0.059	[-0.161 - 0.069]	4.316E-01	9.846E-01
Left superior frontal gyrus	0.034	0.045	[-0.053 - 0.122]	4.423E-01	9.846E-01
Right superior frontal gyrus	0.037	0.043	[-0.048 - 0.122]	3.917E-01	9.846E-01
Left pars orbitalis of inferior frontal gyrus	0.021	0.043	[-0.063 - 0.106]	6.221E-01	9.846E-01
Right pars orbitalis of inferior frontal gyrus	0.056	0.043	[-0.029 - 0.141]	1.963E-01	9.846E-01
Left medial orbitofrontal cortex	0.010	0.043	[-0.075 - 0.094]	8.239E-01	9.846E-01
Right medial orbitofrontal cortex	0.031	0.043	[-0.054 - 0.115]	4.800E-01	9.846E-01
Left inferior temporal gyrus	0.059	0.057	[-0.054 - 0.171]	3.071E-01	9.846E-01
Right inferior temporal gyrus	0.008	0.057	[-0.103 - 0.119]	8.898E-01	9.968E-01
Left isthmus cingulate cortex	-0.062	0.043	[-0.146 - 0.023]	1.545E-01	9.846E-01
Right isthmus cingulate cortex	0.000	0.080	[-0.157 - 0.156]	9.979E-01	9.979E-01
Left banks of superior temporal sulcus	-0.065	0.043	[-0.150 - 0.020]	1.335E-01	9.846E-01
Right banks of superior temporal sulcus	-0.038	0.043	[-0.123 - 0.046]	3.735E-01	9.846E-01
Left supramarginal gyrus	-0.073	0.060	[-0.191 - 0.045]	2.234E-01	9.846E-01
Right supramarginal gyrus	-0.025	0.043	[-0.110 - 0.060]	5.649E-01	9.846E-01
Left caudal middle frontal gyrus	0.007	0.047	[-0.085 - 0.098]	8.894E-01	9.968E-01
Right caudal middle frontal gyrus	-0.002	0.058	[-0.116 - 0.111]	9.678E-01	9.979E-01
Left frontal pole	0.017	0.043	[-0.067 - 0.102]	6.901E-01	9.846E-01
Right frontal pole	0.027	0.043	[-0.057 - 0.112]	5.254E-01	9.846E-01
Left posterior cingulate cortex	-0.036	0.046	[-0.126 - 0.054]	4.338E-01	9.846E-01
Right posterior cingulate cortex	-0.024	0.043	[-0.109 - 0.060]	5.715E-01	9.846E-01
Left lateral occipital cortex	-0.021	0.043	[-0.106 - 0.064]	6.288E-01	9.846E-01
Right lateral occipital cortex	0.040	0.056	[-0.070 - 0.149]	4.777E-01	9.846E-01
Left precentral gyrus	0.010	0.043	[-0.075 - 0.095]	8.196E-01	9.846E-01
Right precentral gyrus	0.017	0.043	[-0.068 - 0.102]	6.932E-01	9.846E-01
Left parahippocampal gyrus	0.062	0.064	[-0.063 - 0.188]	3.294E-01	9.846E-01
Right parahippocampal gyrus	0.017	0.043	[-0.068 - 0.102]	6.950E-01	9.846E-01
Left inferior parietal cortex	-0.039	0.050	[-0.138 - 0.060]	4.421E-01	9.846E-01
Right inferior parietal cortex	0.021	0.043	[-0.064 - 0.105]	6.318E-01	9.846E-01
Left transverse temporal gyrus	-0.071	0.052	[-0.172 - 0.031]	1.744E-01	9.846E-01
Right transverse temporal gyrus	-0.046	0.043	[-0.131 - 0.039]	2.869E-01	9.846E-01
Left postcentral gyrus	-0.072	0.043	[-0.157 - 0.013]	9.622E-02	9.846E-01
Right postcentral gyrus	-0.038	0.045	[-0.127 - 0.051]	4.006E-01	9.846E-01
Left precuneus	-0.037	0.058	[-0.150 - 0.075]	5.152E-01	9.846E-01
Right precuneus	0.006	0.046	[-0.085 - 0.097]	8.985E-01	9.968E-01
Left caudal anterior cingulate cortex	-0.061	0.058	[-0.175 - 0.053]	2.970E-01	9.846E-01
Right caudal anterior cingulate cortex	-0.089	0.061	[-0.208 - 0.031]	1.444E-01	9.846E-01
Left cuneus	0.013	0.057	[-0.098 - 0.125]	8.148E-01	9.846E-01
Right cuneus	0.018	0.071	[-0.122 - 0.157]	8.051E-01	9.846E-01
Left rostral anterior cingulate cortex	0.002	0.060	[-0.115 - 0.120]	9.688E-01	9.979E-01
Right rostral anterior cingulate cortex	-0.002	0.043	[-0.086 - 0.083]	9.701E-01	9.979E-01
Left pericalcarine cortex	-0.001	0.059	[-0.116 - 0.115]	9.900E-01	9.979E-01
Right pericalcarine cortex	0.023	0.061	[-0.097 - 0.142]	7.106E-01	9.846E-01
Left paracentral lobule	-0.003	0.045	[-0.091 - 0.085]	9.503E-01	9.979E-01
Right paracentral lobule	0.046	0.043	[-0.039 - 0.131]	2.882E-01	9.846E-01
Left superior parietal cortex	0.013	0.046	[-0.077 - 0.102]	7.813E-01	9.846E-01
Right superior parietal cortex	0.044	0.045	[-0.045 - 0.132]	3.340E-01	9.846E-01
Left temporal pole	0.019	0.043	[-0.065 - 0.104]	6.560E-01	9.846E-01
Right temporal pole	0.016	0.043	[-0.069 - 0.101]	7.123E-01	9.846E-01
Left entorhinal cortex	0.011	0.043	[-0.073 - 0.096]	7.933E-01	9.846E-01
Right entorhinal cortex	-0.057	0.051	[-0.158 - 0.044]	2.674E-01	9.846E-01

Supplementary Table S77. Partial correlations between cortical thickness and duration of illness controlling for age and sex in individuals with major depressive disorder over 21 years of old

	Partial *R*	Std. Err.	95% CI	*p*-value	FDR *q*-value
Global mean cortical thickness	0.006	0.044	[-0.080 - 0.092]	8.886E-01	9.559E-01
Left hemisphere	-0.010	0.044	[-0.096 - 0.076]	8.236E-01	9.396E-01
Right hemisphere	0.023	0.044	[-0.064 - 0.109]	6.058E-01	9.396E-01
Left fusiform gyrus	0.022	0.044	[-0.064 - 0.109]	6.132E-01	9.396E-01
Right fusiform gyrus	0.071	0.044	[-0.015 - 0.157]	1.063E-01	9.396E-01
Left pars opercularis of inferior frontal gyrus	-0.044	0.044	[-0.130 - 0.043]	3.209E-01	9.396E-01
Right pars opercularis of inferior frontal gyrus	-0.012	0.044	[-0.098 - 0.075]	7.938E-01	9.396E-01
Left superior temporal gyrus	-0.028	0.047	[-0.120 - 0.065]	5.564E-01	9.396E-01
Right superior temporal gyrus	-0.037	0.044	[-0.124 - 0.049]	3.971E-01	9.396E-01
Left insula	-0.044	0.044	[-0.130 - 0.042]	3.191E-01	9.396E-01
Right insula	-0.057	0.047	[-0.148 - 0.035]	2.254E-01	9.396E-01
Left lingual gyrus	-0.045	0.044	[-0.132 - 0.041]	3.040E-01	9.396E-01
Right lingual gyrus	0.064	0.054	[-0.042 - 0.170]	2.359E-01	9.396E-01
Left pars triangularis of inferior frontal gyrus	-0.012	0.061	[-0.131 - 0.107]	8.428E-01	9.396E-01
Right pars triangularis of inferior frontal gyrus	0.031	0.047	[-0.061 - 0.123]	5.069E-01	9.396E-01
Left lateral orbitofrontal cortex	-0.042	0.044	[-0.128 - 0.044]	3.420E-01	9.396E-01
Right lateral orbitofrontal cortex	-0.014	0.044	[-0.100 - 0.073]	7.558E-01	9.396E-01
Left rostral middle frontal gyrus	0.015	0.055	[-0.093 - 0.123]	7.827E-01	9.396E-01
Right rostral middle frontal gyrus	0.091	0.044	[0.004 - 0.177]	3.929E-02	9.396E-01
Left middle temporal gyrus	0.039	0.064	[-0.086 - 0.164]	5.392E-01	9.396E-01
Right middle temporal gyrus	-0.040	0.057	[-0.153 - 0.072]	4.814E-01	9.396E-01
Left superior frontal gyrus	0.038	0.044	[-0.049 - 0.124]	3.913E-01	9.396E-01
Right superior frontal gyrus	0.043	0.044	[-0.044 - 0.129]	3.313E-01	9.396E-01
Left pars orbitalis of inferior frontal gyrus	0.019	0.044	[-0.067 - 0.106]	6.591E-01	9.396E-01
Right pars orbitalis of inferior frontal gyrus	0.061	0.044	[-0.025 - 0.148]	1.632E-01	9.396E-01
Left medial orbitofrontal cortex	0.012	0.044	[-0.074 - 0.098]	7.854E-01	9.396E-01
Right medial orbitofrontal cortex	0.033	0.044	[-0.053 - 0.119]	4.509E-01	9.396E-01
Left inferior temporal gyrus	0.061	0.059	[-0.054 - 0.176]	2.969E-01	9.396E-01
Right inferior temporal gyrus	0.014	0.055	[-0.093 - 0.122]	7.930E-01	9.396E-01
Left isthmus cingulate cortex	-0.060	0.044	[-0.146 - 0.026]	1.727E-01	9.396E-01
Right isthmus cingulate cortex	0.004	0.081	[-0.156 - 0.163]	9.645E-01	9.983E-01
Left banks of superior temporal sulcus	-0.065	0.044	[-0.151 - 0.022]	1.414E-01	9.396E-01
Right banks of superior temporal sulcus	-0.037	0.044	[-0.123 - 0.049]	4.033E-01	9.396E-01
Left supramarginal gyrus	-0.072	0.061	[-0.191 - 0.048]	2.387E-01	9.396E-01
Right supramarginal gyrus	-0.021	0.044	[-0.107 - 0.065]	6.359E-01	9.396E-01
Left caudal middle frontal gyrus	0.009	0.048	[-0.085 - 0.103]	8.470E-01	9.396E-01
Right caudal middle frontal gyrus	0.001	0.058	[-0.113 - 0.116]	9.800E-01	9.983E-01
Left frontal pole	0.017	0.044	[-0.069 - 0.103]	6.973E-01	9.396E-01
Right frontal pole	0.026	0.044	[-0.060 - 0.113]	5.508E-01	9.396E-01
Left posterior cingulate cortex	-0.033	0.046	[-0.123 - 0.057]	4.716E-01	9.396E-01
Right posterior cingulate cortex	-0.020	0.044	[-0.107 - 0.066]	6.435E-01	9.396E-01
Left lateral occipital cortex	-0.017	0.044	[-0.103 - 0.069]	6.993E-01	9.396E-01
Right lateral occipital cortex	0.042	0.057	[-0.070 - 0.153]	4.634E-01	9.396E-01
Left precentral gyrus	0.012	0.044	[-0.074 - 0.098]	7.836E-01	9.396E-01
Right precentral gyrus	0.021	0.044	[-0.065 - 0.107]	6.316E-01	9.396E-01
Left parahippocampal gyrus	0.058	0.067	[-0.073 - 0.188]	3.879E-01	9.396E-01
Right parahippocampal gyrus	0.016	0.044	[-0.070 - 0.102]	7.165E-01	9.396E-01
Left inferior parietal cortex	-0.038	0.051	[-0.138 - 0.062]	4.533E-01	9.396E-01
Right inferior parietal cortex	0.026	0.044	[-0.060 - 0.112]	5.552E-01	9.396E-01
Left transverse temporal gyrus	-0.065	0.052	[-0.167 - 0.037]	2.129E-01	9.396E-01
Right transverse temporal gyrus	-0.050	0.044	[-0.137 - 0.036]	2.528E-01	9.396E-01
Left postcentral gyrus	-0.074	0.044	[-0.160 - 0.012]	9.258E-02	9.396E-01
Right postcentral gyrus	-0.037	0.044	[-0.124 - 0.049]	3.977E-01	9.396E-01
Left precuneus	-0.039	0.059	[-0.154 - 0.076]	5.017E-01	9.396E-01
Right precuneus	0.008	0.047	[-0.084 - 0.100]	8.677E-01	9.478E-01
Left caudal anterior cingulate cortex	-0.057	0.058	[-0.171 - 0.058]	3.311E-01	9.396E-01
Right caudal anterior cingulate cortex	-0.087	0.060	[-0.204 - 0.031]	1.478E-01	9.396E-01
Left cuneus	0.012	0.058	[-0.102 - 0.126]	8.365E-01	9.396E-01
Right cuneus	0.019	0.073	[-0.124 - 0.161]	7.976E-01	9.396E-01
Left rostral anterior cingulate cortex	0.015	0.057	[-0.098 - 0.128]	7.940E-01	9.396E-01
Right rostral anterior cingulate cortex	0.001	0.044	[-0.085 - 0.088]	9.752E-01	9.983E-01
Left pericalcarine cortex	0.000	0.062	[-0.122 - 0.121]	9.983E-01	9.983E-01
Right pericalcarine cortex	0.026	0.065	[-0.102 - 0.155]	6.861E-01	9.396E-01
Left paracentral lobule	0.000	0.045	[-0.088 - 0.089]	9.952E-01	9.983E-01
Right paracentral lobule	0.047	0.044	[-0.040 - 0.133]	2.891E-01	9.396E-01
Left superior parietal cortex	0.014	0.045	[-0.075 - 0.102]	7.625E-01	9.396E-01
Right superior parietal cortex	0.048	0.044	[-0.039 - 0.134]	2.805E-01	9.396E-01
Left temporal pole	0.046	0.052	[-0.055 - 0.147]	3.698E-01	9.396E-01
Right temporal pole	0.022	0.044	[-0.065 - 0.108]	6.218E-01	9.396E-01
Left entorhinal cortex	0.009	0.044	[-0.077 - 0.095]	8.397E-01	9.396E-01
Right entorhinal cortex	-0.058	0.052	[-0.160 - 0.045]	2.683E-01	9.396E-01

Supplementary Table S78. Partial correlations between cortical thickness and age controlling for sex in individuals with autism spectrum disorder

	Partial *R*	Std. Err.	95% CI	*p*-value	FDR *q*-value
Global mean cortical thickness	-0.544	0.103	[-0.746 - -0.343]	1.157E-07	8.807E-07
Left hemisphere	-0.547	0.105	[-0.753 - -0.341]	1.836E-07	1.185E-06
Right hemisphere	-0.520	0.095	[-0.707 - -0.332]	5.238E-08	6.605E-07
Left fusiform gyrus	-0.304	0.121	[-0.542 - -0.067]	1.203E-02	1.424E-02
Right fusiform gyrus	-0.294	0.191	[-0.668 - 0.079]	1.227E-01	1.300E-01
Left pars opercularis of inferior frontal gyrus	-0.554	0.103	[-0.756 - -0.351]	8.459E-08	8.579E-07
Right pars opercularis of inferior frontal gyrus	-0.444	0.134	[-0.707 - -0.181]	9.457E-04	1.679E-03
Left superior temporal gyrus	-0.420	0.104	[-0.624 - -0.216]	5.330E-05	1.514E-04
Right superior temporal gyrus	-0.403	0.074	[-0.548 - -0.257]	5.582E-08	6.605E-07
Left insula	-0.237	0.075	[-0.383 - -0.091]	1.495E-03	2.308E-03
Right insula	-0.293	0.138	[-0.564 - -0.022]	3.388E-02	3.701E-02
Left lingual gyrus	-0.353	0.088	[-0.524 - -0.181]	5.696E-05	1.531E-04
Right lingual gyrus	-0.359	0.074	[-0.504 - -0.214]	1.265E-06	7.220E-06
Left pars triangularis of inferior frontal gyrus	-0.392	0.074	[-0.537 - -0.247]	1.240E-07	8.807E-07
Right pars triangularis of inferior frontal gyrus	-0.366	0.102	[-0.566 - -0.167]	3.134E-04	6.954E-04
Left lateral orbitofrontal cortex	-0.426	0.108	[-0.636 - -0.215]	7.612E-05	1.930E-04
Right lateral orbitofrontal cortex	-0.383	0.117	[-0.613 - -0.153]	1.082E-03	1.829E-03
Left rostral middle frontal gyrus	-0.472	0.133	[-0.733 - -0.211]	3.988E-04	8.089E-04
Right rostral middle frontal gyrus	-0.428	0.133	[-0.690 - -0.167]	1.311E-03	2.069E-03
Left middle temporal gyrus	-0.476	0.087	[-0.647 - -0.305]	4.667E-08	6.605E-07
Right middle temporal gyrus	-0.420	0.127	[-0.669 - -0.172]	9.252E-04	1.679E-03
Left superior frontal gyrus	-0.526	0.126	[-0.772 - -0.279]	2.965E-05	9.570E-05
Right superior frontal gyrus	-0.466	0.111	[-0.684 - -0.248]	2.763E-05	9.340E-05
Left pars orbitalis of inferior frontal gyrus	-0.309	0.137	[-0.578 - -0.040]	2.458E-02	2.726E-02
Right pars orbitalis of inferior frontal gyrus	-0.325	0.108	[-0.537 - -0.113]	2.704E-03	3.765E-03
Left medial orbitofrontal cortex	-0.306	0.106	[-0.515 - -0.097]	4.044E-03	5.165E-03
Right medial orbitofrontal cortex	-0.241	0.084	[-0.405 - -0.077]	4.074E-03	5.165E-03
Left inferior temporal gyrus	-0.289	0.093	[-0.471 - -0.107]	1.895E-03	2.690E-03
Right inferior temporal gyrus	-0.287	0.092	[-0.467 - -0.107]	1.807E-03	2.619E-03
Left isthmus cingulate cortex	-0.458	0.105	[-0.664 - -0.252]	1.297E-05	5.755E-05
Right isthmus cingulate cortex	-0.352	0.098	[-0.544 - -0.159]	3.445E-04	7.412E-04
Left banks of superior temporal sulcus	-0.363	0.080	[-0.520 - -0.207]	5.118E-06	2.422E-05
Right banks of superior temporal sulcus	-0.501	0.130	[-0.757 - -0.246]	1.180E-04	2.890E-04
Left supramarginal gyrus	-0.458	0.114	[-0.681 - -0.235]	5.822E-05	1.531E-04
Right supramarginal gyrus	-0.410	0.077	[-0.562 - -0.259]	1.148E-07	8.807E-07
Left caudal middle frontal gyrus	-0.390	0.138	[-0.661 - -0.119]	4.842E-03	6.031E-03
Right caudal middle frontal gyrus	-0.239	0.081	[-0.398 - -0.080]	3.261E-03	4.382E-03
Left frontal pole	-0.292	0.119	[-0.526 - -0.059]	1.409E-02	1.639E-02
Right frontal pole	-0.142	0.074	[-0.287 - 0.004]	5.615E-02	6.041E-02
Left posterior cingulate cortex	-0.474	0.074	[-0.619 - -0.329]	1.641E-10	1.165E-08
Right posterior cingulate cortex	-0.407	0.074	[-0.553 - -0.262]	3.900E-08	6.605E-07
Left lateral occipital cortex	-0.305	0.074	[-0.450 - -0.160]	3.908E-05	1.156E-04
Right lateral occipital cortex	-0.317	0.074	[-0.462 - -0.171]	1.954E-05	7.708E-05
Left precentral gyrus	-0.319	0.083	[-0.482 - -0.156]	1.262E-04	2.988E-04
Right precentral gyrus	-0.278	0.109	[-0.492 - -0.063]	1.118E-02	1.346E-02
Left parahippocampal gyrus	-0.325	0.103	[-0.526 - -0.124]	1.568E-03	2.342E-03
Right parahippocampal gyrus	-0.215	0.074	[-0.361 - -0.070]	3.661E-03	4.814E-03
Left inferior parietal cortex	-0.336	0.079	[-0.491 - -0.180]	2.349E-05	8.750E-05
Right inferior parietal cortex	-0.391	0.111	[-0.608 - -0.174]	4.121E-04	8.127E-04
Left transverse temporal gyrus	-0.251	0.078	[-0.403 - -0.098]	1.244E-03	2.008E-03
Right transverse temporal gyrus	-0.377	0.088	[-0.549 - -0.205]	1.717E-05	7.173E-05
Left postcentral gyrus	-0.234	0.074	[-0.379 - -0.089]	1.583E-03	2.342E-03
Right postcentral gyrus	-0.249	0.074	[-0.394 - -0.103]	7.922E-04	1.480E-03
Left precuneus	-0.390	0.081	[-0.548 - -0.232]	1.322E-06	7.220E-06
Right precuneus	-0.420	0.089	[-0.594 - -0.246]	2.151E-06	1.091E-05
Left caudal anterior cingulate cortex	-0.313	0.074	[-0.458 - -0.167]	2.465E-05	8.750E-05
Right caudal anterior cingulate cortex	-0.197	0.077	[-0.348 - -0.046]	1.042E-02	1.275E-02
Left cuneus	-0.294	0.090	[-0.471 - -0.117]	1.128E-03	1.863E-03
Right cuneus	-0.306	0.074	[-0.451 - -0.160]	3.724E-05	1.150E-04
Left rostral anterior cingulate cortex	-0.420	0.074	[-0.565 - -0.274]	1.513E-08	5.372E-07
Right rostral anterior cingulate cortex	-0.373	0.111	[-0.590 - -0.156]	7.453E-04	1.430E-03
Left pericalcarine cortex	-0.272	0.075	[-0.418 - -0.126]	2.667E-04	6.108E-04
Right pericalcarine cortex	-0.262	0.074	[-0.408 - -0.117]	3.985E-04	8.089E-04
Left paracentral lobule	-0.234	0.080	[-0.390 - -0.078]	3.271E-03	4.382E-03
Right paracentral lobule	-0.229	0.096	[-0.416 - -0.041]	1.676E-02	1.889E-02
Left superior parietal cortex	-0.280	0.115	[-0.504 - -0.055]	1.462E-02	1.674E-02
Right superior parietal cortex	-0.299	0.092	[-0.479 - -0.120]	1.073E-03	1.829E-03
Left temporal pole	-0.100	0.074	[-0.245 - 0.046]	1.794E-01	1.873E-01
Right temporal pole	-0.002	0.074	[-0.148 - 0.143]	9.760E-01	9.760E-01
Left entorhinal cortex	-0.055	0.089	[-0.229 - 0.119]	5.324E-01	5.478E-01
Right entorhinal cortex	-0.016	0.074	[-0.161 - 0.129]	8.289E-01	8.407E-01

Supplementary Table S79. Partial correlations between cortical surface area and age of onset controlling for age and sex in individuals with schizophrenia

	Partial *R*	Std. Err.	95% CI	*p*-value	FDR *q*-value
Total cortical surface area	-0.033	0.041	[-0.113 - 0.048]	4.241E-01	7.177E-01
Left hemisphere	-0.032	0.041	[-0.113 - 0.048]	4.285E-01	7.177E-01
Right hemisphere	-0.035	0.040	[-0.114 - 0.044]	3.886E-01	7.177E-01
Left superior frontal gyrus	-0.030	0.030	[-0.090 - 0.029]	3.164E-01	7.177E-01
Right superior frontal gyrus	-0.028	0.031	[-0.089 - 0.034]	3.810E-01	7.177E-01
Left pars orbitalis of inferior frontal gyrus	-0.020	0.030	[-0.079 - 0.040]	5.187E-01	7.590E-01
Right pars orbitalis of inferior frontal gyrus	-0.072	0.030	[-0.131 - -0.012]	1.847E-02	6.556E-01
Left precentral gyrus	-0.048	0.031	[-0.108 - 0.012]	1.157E-01	7.177E-01
Right precentral gyrus	-0.007	0.035	[-0.075 - 0.062]	8.496E-01	9.512E-01
Left lingual gyrus	-0.041	0.036	[-0.112 - 0.029]	2.487E-01	7.177E-01
Right lingual gyrus	-0.051	0.033	[-0.117 - 0.014]	1.226E-01	7.177E-01
Left superior temporal gyrus	0.003	0.037	[-0.069 - 0.076]	9.328E-01	9.599E-01
Right superior temporal gyrus	-0.036	0.037	[-0.109 - 0.036]	3.277E-01	7.177E-01
Left fusiform gyrus	-0.068	0.039	[-0.145 - 0.009]	8.405E-02	7.177E-01
Right fusiform gyrus	-0.032	0.035	[-0.102 - 0.037]	3.630E-01	7.177E-01
Left inferior parietal cortex	-0.030	0.037	[-0.103 - 0.042]	4.098E-01	7.177E-01
Right inferior parietal cortex	-0.029	0.030	[-0.088 - 0.031]	3.399E-01	7.177E-01
Left lateral occipital cortex	-0.041	0.039	[-0.117 - 0.034]	2.817E-01	7.177E-01
Right lateral occipital cortex	-0.003	0.046	[-0.093 - 0.087]	9.557E-01	9.694E-01
Left rostral middle frontal gyrus	-0.025	0.030	[-0.085 - 0.034]	4.067E-01	7.177E-01
Right rostral middle frontal gyrus	-0.041	0.039	[-0.118 - 0.035]	2.862E-01	7.177E-01
Left precuneus	-0.012	0.048	[-0.105 - 0.082]	8.085E-01	9.512E-01
Right precuneus	0.005	0.043	[-0.080 - 0.089]	9.151E-01	9.599E-01
Left inferior temporal gyrus	-0.023	0.033	[-0.087 - 0.042]	4.916E-01	7.426E-01
Right inferior temporal gyrus	-0.036	0.031	[-0.096 - 0.024]	2.358E-01	7.177E-01
Left lateral orbitofrontal cortex	-0.030	0.030	[-0.089 - 0.030]	3.264E-01	7.177E-01
Right lateral orbitofrontal cortex	-0.063	0.030	[-0.123 - -0.004]	3.699E-02	7.177E-01
Left middle temporal gyrus	-0.063	0.032	[-0.126 - 0.001]	5.358E-02	7.177E-01
Right middle temporal gyrus	-0.058	0.038	[-0.133 - 0.016]	1.234E-01	7.177E-01
Left postcentral gyrus	-0.031	0.041	[-0.112 - 0.050]	4.549E-01	7.177E-01
Right postcentral gyrus	-0.047	0.036	[-0.117 - 0.023]	1.866E-01	7.177E-01
Left medial orbitofrontal cortex	-0.053	0.043	[-0.137 - 0.030]	2.118E-01	7.177E-01
Right medial orbitofrontal cortex	-0.052	0.041	[-0.132 - 0.028]	1.997E-01	7.177E-01
Left cuneus	-0.040	0.030	[-0.099 - 0.020]	1.898E-01	7.177E-01
Right cuneus	-0.004	0.041	[-0.084 - 0.075]	9.208E-01	9.599E-01
Left pars triangularis of inferior frontal gyrus	-0.001	0.032	[-0.063 - 0.062]	9.850E-01	9.850E-01
Right pars triangularis of inferior frontal gyrus	-0.004	0.030	[-0.064 - 0.055]	8.842E-01	9.512E-01
Left superior parietal cortex	-0.010	0.046	[-0.099 - 0.080]	8.288E-01	9.512E-01
Right superior parietal cortex	-0.020	0.043	[-0.104 - 0.063]	6.321E-01	8.311E-01
Left pars opercularis of inferior frontal gyrus	0.010	0.030	[-0.049 - 0.070]	7.417E-01	9.238E-01
Right pars opercularis of inferior frontal gyrus	-0.028	0.030	[-0.088 - 0.031]	3.505E-01	7.177E-01
Left supramarginal gyrus	-0.031	0.034	[-0.098 - 0.037]	3.716E-01	7.177E-01
Right supramarginal gyrus	-0.029	0.035	[-0.098 - 0.041]	4.217E-01	7.177E-01
Left pericalcarine cortex	-0.018	0.030	[-0.078 - 0.041]	5.441E-01	7.726E-01
Right pericalcarine cortex	-0.028	0.031	[-0.089 - 0.034]	3.764E-01	7.177E-01
Left parahippocampal gyrus	-0.049	0.030	[-0.109 - 0.010]	1.058E-01	7.177E-01
Right parahippocampal gyrus	-0.025	0.039	[-0.100 - 0.051]	5.238E-01	7.590E-01
Left caudal middle frontal gyrus	0.007	0.030	[-0.053 - 0.066]	8.221E-01	9.512E-01
Right caudal middle frontal gyrus	0.023	0.030	[-0.036 - 0.083]	4.468E-01	7.177E-01
Left transverse temporal gyrus	0.015	0.030	[-0.044 - 0.075]	6.176E-01	8.311E-01
Right transverse temporal gyrus	-0.007	0.034	[-0.074 - 0.060]	8.397E-01	9.512E-01
Left banks of superior temporal sulcus	-0.051	0.042	[-0.133 - 0.031]	2.263E-01	7.177E-01
Right banks of superior temporal sulcus	-0.096	0.035	[-0.165 - -0.027]	6.350E-03	4.508E-01
Left caudal anterior cingulate cortex	-0.008	0.042	[-0.091 - 0.075]	8.474E-01	9.512E-01
Right caudal anterior cingulate cortex	-0.049	0.030	[-0.108 - 0.011]	1.091E-01	7.177E-01
Left rostral anterior cingulate cortex	0.018	0.041	[-0.062 - 0.098]	6.544E-01	8.447E-01
Right rostral anterior cingulate cortex	-0.039	0.030	[-0.098 - 0.021]	2.038E-01	7.177E-01
Left posterior cingulate cortex	-0.023	0.046	[-0.114 - 0.067]	6.132E-01	8.311E-01
Right posterior cingulate cortex	-0.051	0.030	[-0.111 - 0.008]	9.208E-02	7.177E-01
Left frontal pole	-0.029	0.030	[-0.088 - 0.031]	3.471E-01	7.177E-01
Right frontal pole	-0.046	0.038	[-0.120 - 0.029]	2.304E-01	7.177E-01
Left paracentral lobule	-0.005	0.030	[-0.064 - 0.055]	8.776E-01	9.512E-01
Right paracentral lobule	-0.016	0.039	[-0.092 - 0.059]	6.708E-01	8.505E-01
Left insula	-0.023	0.030	[-0.083 - 0.036]	4.471E-01	7.177E-01
Right insula	-0.017	0.035	[-0.086 - 0.052]	6.308E-01	8.311E-01
Left entorhinal cortex	-0.037	0.046	[-0.128 - 0.054]	4.213E-01	7.177E-01
Right entorhinal cortex	0.031	0.043	[-0.054 - 0.115]	4.781E-01	7.380E-01
Left temporal pole	-0.056	0.030	[-0.116 - 0.003]	6.319E-02	7.177E-01
Right temporal pole	0.005	0.033	[-0.060 - 0.070]	8.796E-01	9.512E-01
Left isthmus cingulate cortex	-0.029	0.035	[-0.098 - 0.040]	4.073E-01	7.177E-01
Right isthmus cingulate cortex	-0.037	0.036	[-0.107 - 0.033]	3.031E-01	7.177E-01

Supplementary Table S80. Partial correlations between cortical surface area and duration of illness controlling for age and sex in individuals with schizophrenia

	Partial *R*	Std. Err.	95% CI	*p*-value	FDR *q*-value
Total cortical surface area	0.034	0.041	[-0.046 - 0.113]	4.078E-01	7.216E-01
Left hemisphere	0.033	0.041	[-0.046 - 0.113]	4.099E-01	7.216E-01
Right hemisphere	0.035	0.040	[-0.043 - 0.114]	3.758E-01	7.216E-01
Left superior frontal gyrus	0.032	0.030	[-0.028 - 0.091]	2.968E-01	7.216E-01
Right superior frontal gyrus	0.031	0.031	[-0.030 - 0.091]	3.244E-01	7.216E-01
Left pars orbitalis of inferior frontal gyrus	0.020	0.030	[-0.040 - 0.079]	5.167E-01	7.487E-01
Right pars orbitalis of inferior frontal gyrus	0.073	0.030	[0.014 - 0.132]	1.606E-02	5.702E-01
Left precentral gyrus	0.050	0.030	[-0.010 - 0.109]	1.001E-01	7.216E-01
Right precentral gyrus	0.010	0.034	[-0.057 - 0.078]	7.634E-01	9.509E-01
Left lingual gyrus	0.040	0.035	[-0.030 - 0.110]	2.601E-01	7.216E-01
Right lingual gyrus	0.047	0.034	[-0.020 - 0.113]	1.699E-01	7.216E-01
Left superior temporal gyrus	-0.006	0.038	[-0.080 - 0.068]	8.727E-01	9.520E-01
Right superior temporal gyrus	0.038	0.036	[-0.034 - 0.109]	3.022E-01	7.216E-01
Left fusiform gyrus	0.069	0.039	[-0.007 - 0.145]	7.566E-02	7.216E-01
Right fusiform gyrus	0.029	0.035	[-0.039 - 0.098]	4.044E-01	7.216E-01
Left inferior parietal cortex	0.033	0.037	[-0.039 - 0.105]	3.737E-01	7.216E-01
Right inferior parietal cortex	0.031	0.030	[-0.029 - 0.090]	3.138E-01	7.216E-01
Left lateral occipital cortex	0.043	0.039	[-0.033 - 0.120]	2.683E-01	7.216E-01
Right lateral occipital cortex	0.011	0.047	[-0.082 - 0.103]	8.203E-01	9.520E-01
Left rostral middle frontal gyrus	0.024	0.030	[-0.036 - 0.083]	4.360E-01	7.216E-01
Right rostral middle frontal gyrus	0.036	0.041	[-0.044 - 0.116]	3.786E-01	7.216E-01
Left precuneus	0.012	0.047	[-0.080 - 0.105]	7.951E-01	9.520E-01
Right precuneus	-0.003	0.043	[-0.087 - 0.081]	9.486E-01	9.677E-01
Left inferior temporal gyrus	0.025	0.032	[-0.038 - 0.088]	4.370E-01	7.216E-01
Right inferior temporal gyrus	0.040	0.030	[-0.019 - 0.100]	1.840E-01	7.216E-01
Left lateral orbitofrontal cortex	0.028	0.030	[-0.032 - 0.087]	3.573E-01	7.216E-01
Right lateral orbitofrontal cortex	0.064	0.030	[0.005 - 0.123]	3.493E-02	7.216E-01
Left middle temporal gyrus	0.054	0.036	[-0.017 - 0.125]	1.368E-01	7.216E-01
Right middle temporal gyrus	0.059	0.038	[-0.015 - 0.133]	1.205E-01	7.216E-01
Left postcentral gyrus	0.033	0.040	[-0.047 - 0.112]	4.185E-01	7.216E-01
Right postcentral gyrus	0.046	0.036	[-0.025 - 0.117]	2.004E-01	7.216E-01
Left medial orbitofrontal cortex	0.057	0.042	[-0.024 - 0.139]	1.670E-01	7.216E-01
Right medial orbitofrontal cortex	0.054	0.041	[-0.026 - 0.135]	1.858E-01	7.216E-01
Left cuneus	0.039	0.030	[-0.020 - 0.098]	1.983E-01	7.216E-01
Right cuneus	-0.008	0.045	[-0.096 - 0.080]	8.641E-01	9.520E-01
Left pars triangularis of inferior frontal gyrus	0.001	0.031	[-0.059 - 0.062]	9.677E-01	9.677E-01
Right pars triangularis of inferior frontal gyrus	0.007	0.030	[-0.053 - 0.066]	8.293E-01	9.520E-01
Left superior parietal cortex	0.012	0.045	[-0.077 - 0.100]	7.943E-01	9.520E-01
Right superior parietal cortex	0.024	0.043	[-0.059 - 0.107]	5.731E-01	7.979E-01
Left pars opercularis of inferior frontal gyrus	-0.006	0.031	[-0.067 - 0.056]	8.568E-01	9.520E-01
Right pars opercularis of inferior frontal gyrus	0.032	0.030	[-0.027 - 0.092]	2.868E-01	7.216E-01
Left supramarginal gyrus	0.031	0.034	[-0.037 - 0.098]	3.712E-01	7.216E-01
Right supramarginal gyrus	0.026	0.036	[-0.045 - 0.097]	4.667E-01	7.487E-01
Left pericalcarine cortex	0.016	0.030	[-0.043 - 0.076]	5.908E-01	8.067E-01
Right pericalcarine cortex	0.017	0.038	[-0.058 - 0.091]	6.595E-01	8.604E-01
Left parahippocampal gyrus	0.057	0.036	[-0.014 - 0.127]	1.138E-01	7.216E-01
Right parahippocampal gyrus	0.025	0.037	[-0.049 - 0.098]	5.067E-01	7.487E-01
Left caudal middle frontal gyrus	-0.004	0.030	[-0.064 - 0.055]	8.831E-01	9.520E-01
Right caudal middle frontal gyrus	-0.021	0.030	[-0.080 - 0.039]	4.946E-01	7.487E-01
Left transverse temporal gyrus	-0.015	0.030	[-0.074 - 0.045]	6.239E-01	8.358E-01
Right transverse temporal gyrus	0.012	0.035	[-0.058 - 0.081]	7.357E-01	9.327E-01
Left banks of superior temporal sulcus	0.052	0.041	[-0.030 - 0.133]	2.128E-01	7.216E-01
Right banks of superior temporal sulcus	0.093	0.036	[0.022 - 0.163]	9.743E-03	5.702E-01
Left caudal anterior cingulate cortex	0.006	0.042	[-0.076 - 0.089]	8.850E-01	9.520E-01
Right caudal anterior cingulate cortex	0.047	0.030	[-0.013 - 0.106]	1.224E-01	7.216E-01
Left rostral anterior cingulate cortex	-0.017	0.041	[-0.097 - 0.062]	6.665E-01	8.604E-01
Right rostral anterior cingulate cortex	0.036	0.030	[-0.023 - 0.096]	2.299E-01	7.216E-01
Left posterior cingulate cortex	0.029	0.046	[-0.062 - 0.120]	5.295E-01	7.519E-01
Right posterior cingulate cortex	0.047	0.030	[-0.013 - 0.106]	1.222E-01	7.216E-01
Left frontal pole	0.038	0.037	[-0.035 - 0.111]	3.131E-01	7.216E-01
Right frontal pole	0.040	0.037	[-0.033 - 0.113]	2.826E-01	7.216E-01
Left paracentral lobule	0.003	0.030	[-0.056 - 0.063]	9.168E-01	9.572E-01
Right paracentral lobule	-0.007	0.057	[-0.119 - 0.105]	8.994E-01	9.531E-01
Left insula	0.027	0.030	[-0.032 - 0.087]	3.673E-01	7.216E-01
Right insula	0.026	0.040	[-0.052 - 0.103]	5.154E-01	7.487E-01
Left entorhinal cortex	0.041	0.047	[-0.050 - 0.133]	3.769E-01	7.216E-01
Right entorhinal cortex	-0.029	0.043	[-0.114 - 0.057]	5.110E-01	7.487E-01
Left temporal pole	0.077	0.044	[-0.009 - 0.162]	7.923E-02	7.216E-01
Right temporal pole	-0.002	0.033	[-0.066 - 0.063]	9.578E-01	9.677E-01
Left isthmus cingulate cortex	0.034	0.036	[-0.036 - 0.104]	3.467E-01	7.216E-01
Right isthmus cingulate cortex	0.040	0.036	[-0.029 - 0.110]	2.556E-01	7.216E-01

Supplementary Table S81. Partial correlations between cortical surface area and duration of illness controlling for age and sex in individuals with bipolar disorder

	Partial *R*	Std. Err.	95% CI	*p*-value	FDR *q*-value
Total cortical surface area	-0.184	0.070	[-0.322 - -0.046]	9.007E-03	1.146E-01
Left hemisphere	-0.184	0.070	[-0.322 - -0.046]	8.831E-03	1.146E-01
Right hemisphere	-0.181	0.070	[-0.319 - -0.043]	1.013E-02	1.146E-01
Left superior frontal gyrus	-0.216	0.070	[-0.354 - -0.078]	2.125E-03	7.544E-02
Right superior frontal gyrus	-0.084	0.070	[-0.222 - 0.054]	2.324E-01	3.511E-01
Left pars orbitalis of inferior frontal gyrus	-0.210	0.119	[-0.443 - 0.023]	7.785E-02	2.047E-01
Right pars orbitalis of inferior frontal gyrus	-0.160	0.070	[-0.298 - -0.022]	2.264E-02	1.389E-01
Left precentral gyrus	-0.163	0.070	[-0.301 - -0.025]	2.031E-02	1.389E-01
Right precentral gyrus	-0.128	0.070	[-0.265 - 0.010]	6.993E-02	2.047E-01
Left lingual gyrus	-0.086	0.070	[-0.224 - 0.052]	2.198E-01	3.393E-01
Right lingual gyrus	-0.103	0.071	[-0.243 - 0.036]	1.473E-01	2.676E-01
Left superior temporal gyrus	-0.100	0.070	[-0.238 - 0.038]	1.557E-01	2.696E-01
Right superior temporal gyrus	-0.058	0.124	[-0.301 - 0.184]	6.369E-01	7.122E-01
Left fusiform gyrus	-0.055	0.070	[-0.192 - 0.083]	4.381E-01	5.555E-01
Right fusiform gyrus	-0.062	0.070	[-0.200 - 0.076]	3.754E-01	5.126E-01
Left inferior parietal cortex	-0.118	0.072	[-0.259 - 0.023]	1.014E-01	2.182E-01
Right inferior parietal cortex	-0.051	0.073	[-0.194 - 0.092]	4.885E-01	5.979E-01
Left lateral occipital cortex	-0.131	0.070	[-0.269 - 0.006]	6.179E-02	1.994E-01
Right lateral occipital cortex	-0.175	0.070	[-0.313 - -0.037]	1.292E-02	1.146E-01
Left rostral middle frontal gyrus	-0.163	0.070	[-0.301 - -0.025]	2.066E-02	1.389E-01
Right rostral middle frontal gyrus	-0.134	0.072	[-0.275 - 0.006]	6.053E-02	1.994E-01
Left precuneus	-0.222	0.105	[-0.428 - -0.015]	3.519E-02	1.784E-01
Right precuneus	-0.139	0.070	[-0.277 - -0.001]	4.824E-02	1.994E-01
Left inferior temporal gyrus	-0.088	0.102	[-0.289 - 0.113]	3.894E-01	5.216E-01
Right inferior temporal gyrus	-0.040	0.070	[-0.178 - 0.098]	5.662E-01	6.591E-01
Left lateral orbitofrontal cortex	-0.139	0.086	[-0.308 - 0.030]	1.074E-01	2.184E-01
Right lateral orbitofrontal cortex	-0.231	0.070	[-0.369 - -0.093]	1.010E-03	7.169E-02
Left middle temporal gyrus	-0.143	0.096	[-0.332 - 0.045]	1.370E-01	2.630E-01
Right middle temporal gyrus	-0.151	0.091	[-0.329 - 0.028]	9.803E-02	2.175E-01
Left postcentral gyrus	-0.151	0.070	[-0.289 - -0.013]	3.144E-02	1.717E-01
Right postcentral gyrus	-0.057	0.070	[-0.195 - 0.081]	4.164E-01	5.375E-01
Left medial orbitofrontal cortex	-0.132	0.070	[-0.270 - 0.005]	5.985E-02	1.994E-01
Right medial orbitofrontal cortex	-0.117	0.070	[-0.255 - 0.021]	9.729E-02	2.175E-01
Left cuneus	-0.186	0.099	[-0.381 - 0.008]	6.077E-02	1.994E-01
Right cuneus	-0.159	0.070	[-0.297 - -0.022]	2.347E-02	1.389E-01
Left pars triangularis of inferior frontal gyrus	-0.142	0.084	[-0.307 - 0.022]	8.934E-02	2.175E-01
Right pars triangularis of inferior frontal gyrus	-0.097	0.070	[-0.235 - 0.041]	1.685E-01	2.782E-01
Left superior parietal cortex	-0.059	0.070	[-0.197 - 0.079]	4.043E-01	5.315E-01
Right superior parietal cortex	-0.241	0.096	[-0.429 - -0.052]	1.227E-02	1.146E-01
Left pars opercularis of inferior frontal gyrus	-0.127	0.070	[-0.264 - 0.011]	7.214E-02	2.047E-01
Right pars opercularis of inferior frontal gyrus	-0.135	0.070	[-0.273 - 0.003]	5.529E-02	1.994E-01
Left supramarginal gyrus	-0.153	0.078	[-0.305 - 0.000]	4.981E-02	1.994E-01
Right supramarginal gyrus	-0.124	0.070	[-0.262 - 0.013]	7.690E-02	2.047E-01
Left pericalcarine cortex	-0.204	0.120	[-0.438 - 0.031]	8.881E-02	2.175E-01
Right pericalcarine cortex	-0.161	0.103	[-0.363 - 0.041]	1.181E-01	2.329E-01
Left parahippocampal gyrus	-0.098	0.070	[-0.236 - 0.040]	1.645E-01	2.781E-01
Right parahippocampal gyrus	-0.067	0.070	[-0.205 - 0.071]	3.391E-01	4.721E-01
Left caudal middle frontal gyrus	0.007	0.070	[-0.131 - 0.145]	9.224E-01	9.308E-01
Right caudal middle frontal gyrus	-0.052	0.085	[-0.218 - 0.115]	5.434E-01	6.430E-01
Left transverse temporal gyrus	0.006	0.070	[-0.132 - 0.144]	9.308E-01	9.308E-01
Right transverse temporal gyrus	-0.037	0.070	[-0.175 - 0.101]	5.995E-01	6.866E-01
Left banks of superior temporal sulcus	-0.015	0.070	[-0.153 - 0.123]	8.313E-01	8.680E-01
Right banks of superior temporal sulcus	-0.052	0.111	[-0.269 - 0.166]	6.419E-01	7.122E-01
Left caudal anterior cingulate cortex	-0.089	0.091	[-0.266 - 0.089]	3.273E-01	4.721E-01
Right caudal anterior cingulate cortex	-0.049	0.112	[-0.269 - 0.171]	6.645E-01	7.258E-01
Left rostral anterior cingulate cortex	0.018	0.070	[-0.120 - 0.156]	7.958E-01	8.433E-01
Right rostral anterior cingulate cortex	-0.108	0.074	[-0.253 - 0.037]	1.442E-01	2.676E-01
Left posterior cingulate cortex	-0.116	0.070	[-0.254 - 0.021]	9.782E-02	2.175E-01
Right posterior cingulate cortex	-0.113	0.070	[-0.251 - 0.025]	1.077E-01	2.184E-01
Left frontal pole	-0.105	0.073	[-0.248 - 0.038]	1.508E-01	2.676E-01
Right frontal pole	-0.125	0.070	[-0.263 - 0.013]	7.652E-02	2.047E-01
Left paracentral lobule	-0.093	0.070	[-0.230 - 0.045]	1.885E-01	2.974E-01
Right paracentral lobule	-0.189	0.070	[-0.327 - -0.051]	7.213E-03	1.146E-01
Left insula	-0.048	0.070	[-0.186 - 0.090]	4.974E-01	5.985E-01
Right insula	-0.133	0.070	[-0.271 - 0.005]	5.804E-02	1.994E-01
Left entorhinal cortex	-0.103	0.076	[-0.251 - 0.045]	1.732E-01	2.795E-01
Right entorhinal cortex	-0.049	0.070	[-0.187 - 0.088]	4.825E-01	5.979E-01
Left temporal pole	-0.013	0.074	[-0.158 - 0.133]	8.638E-01	8.889E-01
Right temporal pole	-0.106	0.101	[-0.303 - 0.092]	2.941E-01	4.350E-01
Left isthmus cingulate cortex	-0.068	0.070	[-0.206 - 0.070]	3.364E-01	4.721E-01
Right isthmus cingulate cortex	-0.023	0.070	[-0.160 - 0.115]	7.489E-01	8.056E-01

Supplementary Table S82. Partial correlations between cortical surface area and duration of illness controlling for age, sex, age × sex, age^2, age^2 × sex, and ICV in individuals with bipolar disorder aged 25 years and older

	Partial *R*	Std. Err.	95% CI	*p*-value	FDR *q*-value
Total cortical surface area	-0.177	0.109	[-0.391 - 0.037]	1.057E-01	6.344E-01
Left hemisphere	-0.186	0.108	[-0.397 - 0.025]	8.370E-02	6.344E-01
Right hemisphere	-0.162	0.110	[-0.376 - 0.053]	1.406E-01	6.344E-01
Left superior frontal gyrus	-0.174	0.077	[-0.325 - -0.024]	2.342E-02	6.344E-01
Right superior frontal gyrus	0.042	0.091	[-0.137 - 0.222]	6.423E-01	9.099E-01
Left pars orbitalis of inferior frontal gyrus	-0.067	0.099	[-0.260 - 0.127]	4.990E-01	8.435E-01
Right pars orbitalis of inferior frontal gyrus	-0.063	0.077	[-0.214 - 0.088]	4.139E-01	8.164E-01
Left precentral gyrus	-0.132	0.078	[-0.285 - 0.020]	8.833E-02	6.344E-01
Right precentral gyrus	-0.237	0.164	[-0.558 - 0.084]	1.477E-01	6.344E-01
Left lingual gyrus	-0.024	0.077	[-0.174 - 0.127]	7.590E-01	9.160E-01
Right lingual gyrus	-0.191	0.144	[-0.474 - 0.092]	1.869E-01	6.663E-01
Left superior temporal gyrus	0.031	0.077	[-0.119 - 0.182]	6.822E-01	9.099E-01
Right superior temporal gyrus	0.027	0.125	[-0.218 - 0.273]	8.292E-01	9.345E-01
Left fusiform gyrus	-0.002	0.084	[-0.168 - 0.163]	9.800E-01	9.897E-01
Right fusiform gyrus	-0.053	0.077	[-0.204 - 0.098]	4.914E-01	8.435E-01
Left inferior parietal cortex	-0.112	0.129	[-0.365 - 0.141]	3.874E-01	8.164E-01
Right inferior parietal cortex	0.003	0.121	[-0.235 - 0.241]	9.783E-01	9.897E-01
Left lateral occipital cortex	0.013	0.077	[-0.138 - 0.164]	8.677E-01	9.626E-01
Right lateral occipital cortex	-0.157	0.086	[-0.326 - 0.012]	6.876E-02	6.344E-01
Left rostral middle frontal gyrus	-0.140	0.096	[-0.327 - 0.048]	1.443E-01	6.344E-01
Right rostral middle frontal gyrus	-0.067	0.077	[-0.218 - 0.084]	3.829E-01	8.164E-01
Left precuneus	-0.194	0.077	[-0.345 - -0.043]	1.175E-02	6.344E-01
Right precuneus	-0.032	0.080	[-0.188 - 0.125]	6.921E-01	9.099E-01
Left inferior temporal gyrus	-0.144	0.171	[-0.479 - 0.191]	3.988E-01	8.164E-01
Right inferior temporal gyrus	-0.082	0.143	[-0.363 - 0.199]	5.664E-01	8.937E-01
Left lateral orbitofrontal cortex	-0.036	0.110	[-0.251 - 0.179]	7.424E-01	9.160E-01
Right lateral orbitofrontal cortex	-0.133	0.077	[-0.284 - 0.018]	8.374E-02	6.344E-01
Left middle temporal gyrus	-0.136	0.126	[-0.383 - 0.111]	2.800E-01	7.951E-01
Right middle temporal gyrus	-0.119	0.095	[-0.306 - 0.067]	2.099E-01	6.908E-01
Left postcentral gyrus	-0.104	0.092	[-0.284 - 0.077]	2.605E-01	7.706E-01
Right postcentral gyrus	0.038	0.082	[-0.122 - 0.199]	6.406E-01	9.099E-01
Left medial orbitofrontal cortex	-0.001	0.077	[-0.152 - 0.150]	9.882E-01	9.897E-01
Right medial orbitofrontal cortex	0.012	0.083	[-0.150 - 0.175]	8.813E-01	9.626E-01
Left cuneus	-0.184	0.133	[-0.445 - 0.077]	1.661E-01	6.551E-01
Right cuneus	-0.152	0.105	[-0.358 - 0.054]	1.474E-01	6.344E-01
Left pars triangularis of inferior frontal gyrus	-0.154	0.117	[-0.382 - 0.075]	1.877E-01	6.663E-01
Right pars triangularis of inferior frontal gyrus	0.032	0.077	[-0.119 - 0.183]	6.792E-01	9.099E-01
Left superior parietal cortex	-0.024	0.080	[-0.182 - 0.133]	7.612E-01	9.160E-01
Right superior parietal cortex	-0.058	0.077	[-0.209 - 0.093]	4.500E-01	8.243E-01
Left pars opercularis of inferior frontal gyrus	-0.036	0.077	[-0.187 - 0.114]	6.367E-01	9.099E-01
Right pars opercularis of inferior frontal gyrus	-0.087	0.125	[-0.331 - 0.157]	4.845E-01	8.435E-01
Left supramarginal gyrus	-0.137	0.110	[-0.353 - 0.079]	2.141E-01	6.908E-01
Right supramarginal gyrus	-0.136	0.090	[-0.313 - 0.040]	1.302E-01	6.344E-01
Left pericalcarine cortex	-0.293	0.147	[-0.581 - -0.005]	4.584E-02	6.344E-01
Right pericalcarine cortex	-0.253	0.161	[-0.569 - 0.062]	1.159E-01	6.344E-01
Left parahippocampal gyrus	-0.046	0.077	[-0.196 - 0.105]	5.535E-01	8.937E-01
Right parahippocampal gyrus	0.005	0.077	[-0.146 - 0.156]	9.469E-01	9.897E-01
Left caudal middle frontal gyrus	0.158	0.077	[0.007 - 0.309]	3.985E-02	6.344E-01
Right caudal middle frontal gyrus	0.094	0.083	[-0.068 - 0.256]	2.576E-01	7.706E-01
Left transverse temporal gyrus	0.074	0.077	[-0.076 - 0.225]	3.333E-01	8.164E-01
Right transverse temporal gyrus	0.024	0.077	[-0.127 - 0.174]	7.585E-01	9.160E-01
Left banks of superior temporal sulcus	-0.044	0.104	[-0.247 - 0.159]	6.709E-01	9.099E-01
Right banks of superior temporal sulcus	-0.004	0.114	[-0.227 - 0.220]	9.736E-01	9.897E-01
Left caudal anterior cingulate cortex	-0.144	0.138	[-0.414 - 0.126]	2.948E-01	8.049E-01
Right caudal anterior cingulate cortex	-0.042	0.103	[-0.243 - 0.160]	6.849E-01	9.099E-01
Left rostral anterior cingulate cortex	0.055	0.164	[-0.268 - 0.377]	7.394E-01	9.160E-01
Right rostral anterior cingulate cortex	-0.058	0.077	[-0.210 - 0.094]	4.528E-01	8.243E-01
Left posterior cingulate cortex	-0.066	0.077	[-0.217 - 0.085]	3.910E-01	8.164E-01
Right posterior cingulate cortex	-0.048	0.082	[-0.210 - 0.114]	5.601E-01	8.937E-01
Left frontal pole	-0.017	0.077	[-0.168 - 0.134]	8.272E-01	9.345E-01
Right frontal pole	-0.112	0.077	[-0.263 - 0.039]	1.452E-01	6.344E-01
Left paracentral lobule	0.099	0.127	[-0.151 - 0.349]	4.364E-01	8.243E-01
Right paracentral lobule	-0.081	0.086	[-0.249 - 0.088]	3.492E-01	8.164E-01
Left insula	0.024	0.087	[-0.148 - 0.195]	7.851E-01	9.291E-01
Right insula	-0.064	0.077	[-0.214 - 0.087]	4.079E-01	8.164E-01
Left entorhinal cortex	-0.073	0.087	[-0.242 - 0.097]	4.006E-01	8.164E-01
Right entorhinal cortex	0.043	0.093	[-0.139 - 0.224]	6.438E-01	9.099E-01
Left temporal pole	-0.001	0.102	[-0.202 - 0.199]	9.897E-01	9.897E-01
Right temporal pole	0.069	0.077	[-0.082 - 0.220]	3.710E-01	8.164E-01
Left isthmus cingulate cortex	-0.146	0.102	[-0.346 - 0.054]	1.519E-01	6.344E-01
Right isthmus cingulate cortex	0.033	0.137	[-0.236 - 0.302]	8.101E-01	9.345E-01

Supplementary Table S83. Partial correlations between cortical surface area and duration of illness controlling for age and sex in individuals with bipolar disorder aged 25 years and older

	Partial *R*	Std. Err.	95% CI	*p*-value	FDR *q*-value
Total cortical surface area	-0.200	0.074	[-0.344 - -0.055]	6.673E-03	9.102E-02
Left hemisphere	-0.201	0.074	[-0.346 - -0.057]	6.311E-03	9.102E-02
Right hemisphere	-0.196	0.074	[-0.341 - -0.052]	7.769E-03	9.102E-02
Left superior frontal gyrus	-0.232	0.074	[-0.377 - -0.088]	1.642E-03	5.829E-02
Right superior frontal gyrus	-0.087	0.074	[-0.232 - 0.057]	2.371E-01	3.582E-01
Left pars orbitalis of inferior frontal gyrus	-0.221	0.123	[-0.463 - 0.021]	7.298E-02	1.906E-01
Right pars orbitalis of inferior frontal gyrus	-0.174	0.074	[-0.319 - -0.030]	1.812E-02	1.169E-01
Left precentral gyrus	-0.183	0.074	[-0.327 - -0.038]	1.309E-02	9.299E-02
Right precentral gyrus	-0.145	0.074	[-0.290 - -0.001]	4.861E-02	1.808E-01
Left lingual gyrus	-0.090	0.074	[-0.235 - 0.054]	2.196E-01	3.430E-01
Right lingual gyrus	-0.102	0.075	[-0.249 - 0.044]	1.710E-01	2.824E-01
Left superior temporal gyrus	-0.116	0.076	[-0.264 - 0.032]	1.256E-01	2.371E-01
Right superior temporal gyrus	-0.076	0.130	[-0.332 - 0.179]	5.580E-01	6.384E-01
Left fusiform gyrus	-0.068	0.074	[-0.213 - 0.076]	3.553E-01	4.586E-01
Right fusiform gyrus	-0.074	0.074	[-0.219 - 0.070]	3.129E-01	4.273E-01
Left inferior parietal cortex	-0.157	0.088	[-0.328 - 0.015]	7.426E-02	1.906E-01
Right inferior parietal cortex	-0.072	0.082	[-0.234 - 0.089]	3.805E-01	4.825E-01
Left lateral occipital cortex	-0.139	0.074	[-0.284 - 0.005]	5.907E-02	1.906E-01
Right lateral occipital cortex	-0.196	0.074	[-0.341 - -0.052]	7.753E-03	9.102E-02
Left rostral middle frontal gyrus	-0.169	0.074	[-0.314 - -0.025]	2.186E-02	1.293E-01
Right rostral middle frontal gyrus	-0.146	0.075	[-0.292 - 0.001]	5.229E-02	1.808E-01
Left precuneus	-0.235	0.110	[-0.450 - -0.020]	3.197E-02	1.746E-01
Right precuneus	-0.147	0.074	[-0.292 - -0.003]	4.595E-02	1.808E-01
Left inferior temporal gyrus	-0.126	0.121	[-0.362 - 0.111]	2.983E-01	4.167E-01
Right inferior temporal gyrus	-0.049	0.074	[-0.193 - 0.096]	5.082E-01	5.915E-01
Left lateral orbitofrontal cortex	-0.144	0.087	[-0.314 - 0.026]	9.758E-02	2.165E-01
Right lateral orbitofrontal cortex	-0.250	0.074	[-0.394 - -0.105]	7.121E-04	5.056E-02
Left middle temporal gyrus	-0.163	0.104	[-0.367 - 0.040]	1.153E-01	2.360E-01
Right middle temporal gyrus	-0.178	0.100	[-0.373 - 0.018]	7.516E-02	1.906E-01
Left postcentral gyrus	-0.162	0.077	[-0.313 - -0.011]	3.505E-02	1.777E-01
Right postcentral gyrus	-0.063	0.074	[-0.207 - 0.082]	3.945E-01	4.914E-01
Left medial orbitofrontal cortex	-0.137	0.074	[-0.282 - 0.007]	6.277E-02	1.906E-01
Right medial orbitofrontal cortex	-0.105	0.074	[-0.250 - 0.039]	1.529E-01	2.616E-01
Left cuneus	-0.193	0.100	[-0.388 - 0.003]	5.346E-02	1.808E-01
Right cuneus	-0.188	0.074	[-0.332 - -0.043]	1.097E-02	9.299E-02
Left pars triangularis of inferior frontal gyrus	-0.166	0.090	[-0.342 - 0.011]	6.548E-02	1.906E-01
Right pars triangularis of inferior frontal gyrus	-0.090	0.074	[-0.234 - 0.055]	2.222E-01	3.430E-01
Left superior parietal cortex	-0.055	0.074	[-0.199 - 0.090]	4.582E-01	5.422E-01
Right superior parietal cortex	-0.246	0.099	[-0.441 - -0.052]	1.310E-02	9.299E-02
Left pars opercularis of inferior frontal gyrus	-0.128	0.074	[-0.272 - 0.017]	8.290E-02	1.947E-01
Right pars opercularis of inferior frontal gyrus	-0.148	0.074	[-0.293 - -0.004]	4.425E-02	1.808E-01
Left supramarginal gyrus	-0.165	0.092	[-0.346 - 0.016]	7.479E-02	1.906E-01
Right supramarginal gyrus	-0.122	0.079	[-0.276 - 0.032]	1.204E-01	2.371E-01
Left pericalcarine cortex	-0.222	0.127	[-0.472 - 0.027]	8.059E-02	1.947E-01
Right pericalcarine cortex	-0.203	0.118	[-0.433 - 0.028]	8.503E-02	1.947E-01
Left parahippocampal gyrus	-0.108	0.074	[-0.253 - 0.036]	1.425E-01	2.560E-01
Right parahippocampal gyrus	-0.086	0.074	[-0.230 - 0.059]	2.453E-01	3.629E-01
Left caudal middle frontal gyrus	0.004	0.074	[-0.141 - 0.148]	9.580E-01	9.580E-01
Right caudal middle frontal gyrus	-0.066	0.087	[-0.236 - 0.105]	4.491E-01	5.405E-01
Left transverse temporal gyrus	0.005	0.074	[-0.139 - 0.150]	9.411E-01	9.546E-01
Right transverse temporal gyrus	-0.031	0.074	[-0.176 - 0.113]	6.704E-01	7.323E-01
Left banks of superior temporal sulcus	-0.023	0.074	[-0.168 - 0.121]	7.531E-01	7.981E-01
Right banks of superior temporal sulcus	-0.068	0.119	[-0.300 - 0.164]	5.665E-01	6.384E-01
Left caudal anterior cingulate cortex	-0.102	0.098	[-0.294 - 0.090]	2.993E-01	4.167E-01
Right caudal anterior cingulate cortex	-0.059	0.116	[-0.287 - 0.168]	6.082E-01	6.747E-01
Left rostral anterior cingulate cortex	0.021	0.074	[-0.124 - 0.165]	7.805E-01	8.149E-01
Right rostral anterior cingulate cortex	-0.117	0.080	[-0.275 - 0.040]	1.442E-01	2.560E-01
Left posterior cingulate cortex	-0.119	0.074	[-0.263 - 0.026]	1.076E-01	2.315E-01
Right posterior cingulate cortex	-0.116	0.074	[-0.260 - 0.029]	1.163E-01	2.360E-01
Left frontal pole	-0.117	0.077	[-0.268 - 0.033]	1.269E-01	2.371E-01
Right frontal pole	-0.144	0.074	[-0.289 - 0.000]	5.025E-02	1.808E-01
Left paracentral lobule	-0.101	0.075	[-0.248 - 0.045]	1.759E-01	2.838E-01
Right paracentral lobule	-0.193	0.074	[-0.337 - -0.048]	8.973E-03	9.102E-02
Left insula	-0.060	0.074	[-0.205 - 0.084]	4.131E-01	5.056E-01
Right insula	-0.146	0.074	[-0.290 - -0.001]	4.789E-02	1.808E-01
Left entorhinal cortex	-0.121	0.085	[-0.287 - 0.046]	1.547E-01	2.616E-01
Right entorhinal cortex	-0.072	0.074	[-0.216 - 0.073]	3.290E-01	4.326E-01
Left temporal pole	-0.030	0.095	[-0.216 - 0.156]	7.506E-01	7.981E-01
Right temporal pole	-0.120	0.107	[-0.329 - 0.090]	2.623E-01	3.801E-01
Left isthmus cingulate cortex	-0.073	0.074	[-0.218 - 0.073]	3.271E-01	4.326E-01
Right isthmus cingulate cortex	-0.018	0.074	[-0.163 - 0.126]	8.044E-01	8.277E-01

Supplementary Table S84. Cortical surface area differences between individuals with major depressive disorder with a late age of onset (over 21 years old; LAO) versus healthy comparison subjects controlling for age and sex

	d	Std. Err.	95% CI	*p*-value	FDR *q*-value
Total cortical surface area	-0.146	0.077	[-0.298 - 0.006]	5.909E-02	2.715E-01
Left hemisphere	-0.149	0.077	[-0.300 - 0.001]	5.131E-02	2.715E-01
Right hemisphere	-0.141	0.078	[-0.294 - 0.011]	6.912E-02	2.715E-01
Left superior frontal gyrus	-0.148	0.080	[-0.305 - 0.009]	6.535E-02	2.715E-01
Right superior frontal gyrus	-0.176	0.085	[-0.343 - -0.010]	3.818E-02	2.516E-01
Left pars orbitalis of inferior frontal gyrus	-0.140	0.078	[-0.292 - 0.013]	7.248E-02	2.715E-01
Right pars orbitalis of inferior frontal gyrus	-0.111	0.078	[-0.264 - 0.042]	1.559E-01	3.757E-01
Left precentral gyrus	-0.063	0.080	[-0.220 - 0.094]	4.316E-01	6.139E-01
Right precentral gyrus	-0.047	0.066	[-0.176 - 0.083]	4.786E-01	6.411E-01
Left lingual gyrus	-0.107	0.090	[-0.284 - 0.070]	2.366E-01	4.667E-01
Right lingual gyrus	-0.145	0.067	[-0.276 - -0.014]	3.065E-02	2.516E-01
Left superior temporal gyrus	-0.100	0.073	[-0.243 - 0.044]	1.733E-01	3.844E-01
Right superior temporal gyrus	-0.134	0.080	[-0.290 - 0.022]	9.284E-02	2.866E-01
Left fusiform gyrus	-0.111	0.092	[-0.292 - 0.070]	2.306E-01	4.667E-01
Right fusiform gyrus	-0.139	0.066	[-0.268 - -0.009]	3.611E-02	2.516E-01
Left inferior parietal cortex	-0.114	0.095	[-0.300 - 0.071]	2.273E-01	4.667E-01
Right inferior parietal cortex	-0.089	0.081	[-0.249 - 0.070]	2.718E-01	4.824E-01
Left lateral occipital cortex	-0.065	0.067	[-0.197 - 0.067]	3.377E-01	5.182E-01
Right lateral occipital cortex	-0.140	0.069	[-0.275 - -0.005]	4.253E-02	2.516E-01
Left rostral middle frontal gyrus	-0.120	0.071	[-0.260 - 0.019]	9.121E-02	2.866E-01
Right rostral middle frontal gyrus	-0.041	0.073	[-0.184 - 0.102]	5.728E-01	7.012E-01
Left precuneus	-0.165	0.066	[-0.295 - -0.036]	1.244E-02	2.148E-01
Right precuneus	-0.013	0.067	[-0.145 - 0.119]	8.444E-01	8.574E-01
Left inferior temporal gyrus	-0.092	0.066	[-0.221 - 0.038]	1.640E-01	3.757E-01
Right inferior temporal gyrus	-0.069	0.066	[-0.199 - 0.061]	2.964E-01	5.011E-01
Left lateral orbitofrontal cortex	-0.102	0.070	[-0.238 - 0.034]	1.422E-01	3.757E-01
Right lateral orbitofrontal cortex	-0.016	0.083	[-0.179 - 0.147]	8.453E-01	8.574E-01
Left middle temporal gyrus	-0.149	0.067	[-0.281 - -0.017]	2.646E-02	2.516E-01
Right middle temporal gyrus	-0.144	0.076	[-0.293 - 0.004]	5.724E-02	2.715E-01
Left postcentral gyrus	-0.124	0.086	[-0.293 - 0.046]	1.519E-01	3.757E-01
Right postcentral gyrus	-0.157	0.088	[-0.329 - 0.014]	7.265E-02	2.715E-01
Left medial orbitofrontal cortex	-0.045	0.095	[-0.231 - 0.140]	6.317E-01	7.261E-01
Right medial orbitofrontal cortex	-0.104	0.075	[-0.252 - 0.043]	1.639E-01	3.757E-01
Left cuneus	-0.178	0.075	[-0.326 - -0.030]	1.815E-02	2.148E-01
Right cuneus	-0.177	0.066	[-0.307 - -0.048]	7.358E-03	2.148E-01
Left pars triangularis of inferior frontal gyrus	-0.091	0.081	[-0.249 - 0.067]	2.607E-01	4.824E-01
Right pars triangularis of inferior frontal gyrus	-0.120	0.070	[-0.256 - 0.017]	8.524E-02	2.866E-01
Left superior parietal cortex	-0.062	0.066	[-0.191 - 0.068]	3.503E-01	5.182E-01
Right superior parietal cortex	-0.050	0.066	[-0.180 - 0.079]	4.476E-01	6.231E-01
Left pars opercularis of inferior frontal gyrus	-0.045	0.076	[-0.193 - 0.103]	5.531E-01	6.889E-01
Right pars opercularis of inferior frontal gyrus	-0.041	0.086	[-0.209 - 0.128]	6.341E-01	7.261E-01
Left supramarginal gyrus	-0.068	0.066	[-0.198 - 0.063]	3.093E-01	5.107E-01
Right supramarginal gyrus	-0.049	0.071	[-0.189 - 0.090]	4.901E-01	6.444E-01
Left pericalcarine cortex	-0.158	0.066	[-0.288 - -0.029]	1.663E-02	2.148E-01
Right pericalcarine cortex	-0.217	0.075	[-0.364 - -0.069]	3.919E-03	2.148E-01
Left parahippocampal gyrus	-0.023	0.069	[-0.159 - 0.113]	7.396E-01	8.078E-01
Right parahippocampal gyrus	-0.063	0.066	[-0.193 - 0.066]	3.393E-01	5.182E-01
Left caudal middle frontal gyrus	-0.028	0.080	[-0.185 - 0.129]	7.279E-01	8.075E-01
Right caudal middle frontal gyrus	0.028	0.073	[-0.115 - 0.171]	7.057E-01	7.953E-01
Left transverse temporal gyrus	-0.062	0.066	[-0.192 - 0.067]	3.463E-01	5.182E-01
Right transverse temporal gyrus	-0.071	0.066	[-0.201 - 0.058]	2.796E-01	4.842E-01
Left banks of superior temporal sulcus	-0.107	0.083	[-0.271 - 0.056]	1.972E-01	4.243E-01
Right banks of superior temporal sulcus	-0.088	0.111	[-0.306 - 0.131]	4.324E-01	6.139E-01
Left caudal anterior cingulate cortex	-0.134	0.066	[-0.264 - -0.005]	4.235E-02	2.516E-01
Right caudal anterior cingulate cortex	-0.081	0.073	[-0.225 - 0.063]	2.690E-01	4.824E-01
Left rostral anterior cingulate cortex	-0.093	0.066	[-0.222 - 0.037]	1.603E-01	3.757E-01
Right rostral anterior cingulate cortex	-0.021	0.084	[-0.186 - 0.144]	8.028E-01	8.507E-01
Left posterior cingulate cortex	-0.063	0.066	[-0.192 - 0.067]	3.438E-01	5.182E-01
Right posterior cingulate cortex	-0.143	0.081	[-0.302 - 0.016]	7.707E-02	2.736E-01
Left frontal pole	-0.114	0.076	[-0.264 - 0.036]	1.351E-01	3.757E-01
Right frontal pole	-0.160	0.066	[-0.290 - -0.031]	1.533E-02	2.148E-01
Left paracentral lobule	0.012	0.066	[-0.118 - 0.141]	8.606E-01	8.606E-01
Right paracentral lobule	-0.096	0.066	[-0.225 - 0.034]	1.466E-01	3.757E-01
Left insula	-0.044	0.066	[-0.174 - 0.086]	5.059E-01	6.530E-01
Right insula	-0.037	0.077	[-0.188 - 0.114]	6.310E-01	7.261E-01
Left entorhinal cortex	-0.020	0.074	[-0.165 - 0.125]	7.831E-01	8.425E-01
Right entorhinal cortex	0.076	0.066	[-0.053 - 0.206]	2.495E-01	4.789E-01
Left temporal pole	-0.046	0.074	[-0.191 - 0.098]	5.285E-01	6.701E-01
Right temporal pole	-0.048	0.066	[-0.177 - 0.082]	4.693E-01	6.407E-01
Left isthmus cingulate cortex	-0.014	0.066	[-0.144 - 0.116]	8.322E-01	8.574E-01
Right isthmus cingulate cortex	0.032	0.066	[-0.097 - 0.162]	6.250E-01	7.261E-01

Supplementary Table S85. Cortical surface area differences between individuals with major depressive disorder with an early age of onset (21 years of old or younger; EAO) versus healthy comparison subjects controlling for age and sex

	d	Std. Err.	95% CI	*p*-value	FDR *q*-value
Total cortical surface area	-0.354	0.397	[-1.132 - 0.423]	3.719E-01	8.927E-01
Left hemisphere	-0.343	0.402	[-1.131 - 0.446]	3.942E-01	9.029E-01
Right hemisphere	-0.363	0.388	[-1.124 - 0.397]	3.493E-01	8.927E-01
Left superior frontal gyrus	-0.421	0.340	[-1.086 - 0.245]	2.155E-01	8.724E-01
Right superior frontal gyrus	-0.450	0.276	[-0.991 - 0.092]	1.039E-01	8.724E-01
Left pars orbitalis of inferior frontal gyrus	-0.312	0.262	[-0.825 - 0.201]	2.335E-01	8.724E-01
Right pars orbitalis of inferior frontal gyrus	-0.245	0.197	[-0.630 - 0.141]	2.135E-01	8.724E-01
Left precentral gyrus	-0.520	0.218	[-0.947 - -0.094]	1.686E-02	4.337E-01
Right precentral gyrus	-0.466	0.198	[-0.853 - -0.079]	1.833E-02	4.337E-01
Left lingual gyrus	-0.003	0.416	[-0.819 - 0.813]	9.947E-01	9.947E-01
Right lingual gyrus	-0.034	0.209	[-0.443 - 0.376]	8.720E-01	9.947E-01
Left superior temporal gyrus	-0.120	0.283	[-0.674 - 0.434]	6.721E-01	9.719E-01
Right superior temporal gyrus	-0.024	0.356	[-0.723 - 0.675]	9.467E-01	9.947E-01
Left fusiform gyrus	-0.244	0.196	[-0.629 - 0.141]	2.141E-01	8.724E-01
Right fusiform gyrus	-0.283	0.252	[-0.777 - 0.211]	2.619E-01	8.927E-01
Left inferior parietal cortex	-0.386	0.255	[-0.886 - 0.113]	1.297E-01	8.724E-01
Right inferior parietal cortex	-0.317	0.359	[-1.020 - 0.386]	3.772E-01	8.927E-01
Left lateral occipital cortex	0.059	0.249	[-0.428 - 0.546]	8.128E-01	9.947E-01
Right lateral occipital cortex	-0.215	0.288	[-0.779 - 0.349]	4.543E-01	9.719E-01
Left rostral middle frontal gyrus	-0.096	0.197	[-0.481 - 0.290]	6.265E-01	9.719E-01
Right rostral middle frontal gyrus	0.013	0.248	[-0.473 - 0.499]	9.582E-01	9.947E-01
Left precuneus	-0.190	0.377	[-0.928 - 0.548]	6.143E-01	9.719E-01
Right precuneus	-0.221	0.440	[-1.084 - 0.641]	6.148E-01	9.719E-01
Left inferior temporal gyrus	-0.400	0.319	[-1.026 - 0.226]	2.100E-01	8.724E-01
Right inferior temporal gyrus	-0.328	0.304	[-0.924 - 0.268]	2.812E-01	8.927E-01
Left lateral orbitofrontal cortex	-0.219	0.196	[-0.604 - 0.166]	2.646E-01	8.927E-01
Right lateral orbitofrontal cortex	-0.148	0.196	[-0.533 - 0.236]	4.494E-01	9.719E-01
Left middle temporal gyrus	-0.266	0.278	[-0.812 - 0.280]	3.396E-01	8.927E-01
Right middle temporal gyrus	-0.214	0.226	[-0.656 - 0.228]	3.433E-01	8.927E-01
Left postcentral gyrus	-0.541	0.439	[-1.401 - 0.318]	2.172E-01	8.724E-01
Right postcentral gyrus	-0.433	0.301	[-1.024 - 0.158]	1.511E-01	8.724E-01
Left medial orbitofrontal cortex	-0.297	0.245	[-0.778 - 0.185]	2.271E-01	8.724E-01
Right medial orbitofrontal cortex	-0.169	0.311	[-0.778 - 0.441]	5.876E-01	9.719E-01
Left cuneus	-0.161	0.227	[-0.606 - 0.285]	4.793E-01	9.719E-01
Right cuneus	-0.294	0.197	[-0.680 - 0.091]	1.347E-01	8.724E-01
Left pars triangularis of inferior frontal gyrus	-0.442	0.308	[-1.047 - 0.162]	1.516E-01	8.724E-01
Right pars triangularis of inferior frontal gyrus	-0.202	0.196	[-0.587 - 0.183]	3.040E-01	8.927E-01
Left superior parietal cortex	-0.179	0.430	[-1.021 - 0.664]	6.776E-01	9.719E-01
Right superior parietal cortex	-0.122	0.386	[-0.878 - 0.634]	7.517E-01	9.947E-01
Left pars opercularis of inferior frontal gyrus	0.240	0.528	[-0.795 - 1.275]	6.499E-01	9.719E-01
Right pars opercularis of inferior frontal gyrus	-0.163	0.308	[-0.766 - 0.441]	5.971E-01	9.719E-01
Left supramarginal gyrus	0.003	0.246	[-0.480 - 0.486]	9.892E-01	9.947E-01
Right supramarginal gyrus	-0.176	0.197	[-0.562 - 0.210]	3.724E-01	8.927E-01
Left pericalcarine cortex	-0.218	0.319	[-0.844 - 0.407]	4.938E-01	9.719E-01
Right pericalcarine cortex	-0.329	0.197	[-0.714 - 0.056]	9.439E-02	8.724E-01
Left parahippocampal gyrus	0.046	0.236	[-0.416 - 0.509]	8.440E-01	9.947E-01
Right parahippocampal gyrus	0.099	0.237	[-0.365 - 0.564]	6.759E-01	9.719E-01
Left caudal middle frontal gyrus	-0.076	0.196	[-0.461 - 0.309]	6.982E-01	9.719E-01
Right caudal middle frontal gyrus	-0.041	0.280	[-0.589 - 0.508]	8.847E-01	9.947E-01
Left transverse temporal gyrus	-0.159	0.266	[-0.680 - 0.362]	5.502E-01	9.719E-01
Right transverse temporal gyrus	-0.352	0.365	[-1.067 - 0.363]	3.343E-01	8.927E-01
Left banks of superior temporal sulcus	-0.004	0.196	[-0.388 - 0.381]	9.851E-01	9.947E-01
Right banks of superior temporal sulcus	-0.057	0.202	[-0.452 - 0.338]	7.782E-01	9.947E-01
Left caudal anterior cingulate cortex	0.019	0.251	[-0.473 - 0.512]	9.385E-01	9.947E-01
Right caudal anterior cingulate cortex	-0.076	0.207	[-0.482 - 0.330]	7.136E-01	9.743E-01
Left rostral anterior cingulate cortex	0.021	0.280	[-0.528 - 0.569]	9.414E-01	9.947E-01
Right rostral anterior cingulate cortex	0.115	0.197	[-0.270 - 0.500]	5.590E-01	9.719E-01
Left posterior cingulate cortex	-0.053	0.270	[-0.582 - 0.476]	8.432E-01	9.947E-01
Right posterior cingulate cortex	-0.108	0.197	[-0.494 - 0.278]	5.833E-01	9.719E-01
Left frontal pole	-0.430	0.234	[-0.890 - 0.029]	6.619E-02	8.724E-01
Right frontal pole	-0.236	0.197	[-0.622 - 0.149]	2.296E-01	8.724E-01
Left paracentral lobule	-0.508	0.198	[-0.895 - -0.120]	1.018E-02	4.337E-01
Right paracentral lobule	-0.366	0.197	[-0.752 - 0.020]	6.280E-02	8.724E-01
Left insula	0.061	0.447	[-0.815 - 0.937]	8.917E-01	9.947E-01
Right insula	-0.205	0.512	[-1.209 - 0.798]	6.886E-01	9.719E-01
Left entorhinal cortex	0.035	0.281	[-0.516 - 0.585]	9.020E-01	9.947E-01
Right entorhinal cortex	0.042	0.338	[-0.620 - 0.704]	9.019E-01	9.947E-01
Left temporal pole	0.109	0.196	[-0.276 - 0.494]	5.800E-01	9.719E-01
Right temporal pole	-0.076	0.196	[-0.461 - 0.308]	6.964E-01	9.719E-01
Left isthmus cingulate cortex	-0.042	0.196	[-0.427 - 0.343]	8.316E-01	9.947E-01
Right isthmus cingulate cortex	-0.022	0.209	[-0.431 - 0.388]	9.179E-01	9.947E-01

Supplementary Table S86. Cortical surface area differences between individuals with major depressive disorder with an early age of onset (21 years of old or younger; EAO) versus individuals with major depressive disorder with a late age of onset (over 21 years old; LAO) controlling for age and sex

	d	Std. Err.	95% CI	*p*-value	FDR *q*-value
Total cortical surface area	-0.324	0.286	[-0.886 - 0.238]	2.581E-01	8.228E-01
Left hemisphere	-0.312	0.301	[-0.902 - 0.277]	2.992E-01	8.228E-01
Right hemisphere	-0.332	0.272	[-0.864 - 0.201]	2.219E-01	8.228E-01
Left superior frontal gyrus	-0.357	0.265	[-0.877 - 0.162]	1.777E-01	8.227E-01
Right superior frontal gyrus	-0.444	0.321	[-1.073 - 0.186]	1.676E-01	8.227E-01
Left pars orbitalis of inferior frontal gyrus	-0.283	0.205	[-0.684 - 0.119]	1.680E-01	8.227E-01
Right pars orbitalis of inferior frontal gyrus	-0.332	0.195	[-0.713 - 0.050]	8.867E-02	7.894E-01
Left precentral gyrus	-0.580	0.196	[-0.965 - -0.196]	3.111E-03	1.619E-01
Right precentral gyrus	-0.556	0.196	[-0.940 - -0.172]	4.560E-03	1.619E-01
Left lingual gyrus	0.016	0.281	[-0.534 - 0.567]	9.533E-01	9.809E-01
Right lingual gyrus	-0.071	0.195	[-0.454 - 0.311]	7.141E-01	9.578E-01
Left superior temporal gyrus	-0.083	0.317	[-0.704 - 0.539]	7.946E-01	9.578E-01
Right superior temporal gyrus	-0.047	0.343	[-0.718 - 0.625]	8.919E-01	9.595E-01
Left fusiform gyrus	-0.218	0.195	[-0.600 - 0.165]	2.644E-01	8.228E-01
Right fusiform gyrus	-0.202	0.195	[-0.583 - 0.180]	2.996E-01	8.228E-01
Left inferior parietal cortex	-0.295	0.195	[-0.677 - 0.087]	1.301E-01	8.227E-01
Right inferior parietal cortex	-0.397	0.211	[-0.811 - 0.016]	5.985E-02	7.082E-01
Left lateral occipital cortex	-0.048	0.309	[-0.655 - 0.559]	8.768E-01	9.578E-01
Right lateral occipital cortex	-0.228	0.244	[-0.707 - 0.250]	3.496E-01	8.228E-01
Left rostral middle frontal gyrus	-0.034	0.195	[-0.416 - 0.348]	8.624E-01	9.578E-01
Right rostral middle frontal gyrus	-0.018	0.195	[-0.399 - 0.364]	9.279E-01	9.689E-01
Left precuneus	-0.175	0.354	[-0.869 - 0.520]	6.219E-01	9.578E-01
Right precuneus	-0.360	0.390	[-1.125 - 0.404]	3.557E-01	8.228E-01
Left inferior temporal gyrus	-0.435	0.196	[-0.818 - -0.051]	2.629E-02	4.666E-01
Right inferior temporal gyrus	-0.258	0.195	[-0.639 - 0.124]	1.854E-01	8.227E-01
Left lateral orbitofrontal cortex	-0.123	0.200	[-0.515 - 0.269]	5.397E-01	9.109E-01
Right lateral orbitofrontal cortex	-0.157	0.194	[-0.538 - 0.224]	4.203E-01	9.042E-01
Left middle temporal gyrus	-0.266	0.195	[-0.648 - 0.116]	1.722E-01	8.227E-01
Right middle temporal gyrus	-0.244	0.195	[-0.626 - 0.139]	2.117E-01	8.228E-01
Left postcentral gyrus	-0.461	0.228	[-0.908 - -0.014]	4.315E-02	6.127E-01
Right postcentral gyrus	-0.230	0.239	[-0.699 - 0.239]	3.367E-01	8.228E-01
Left medial orbitofrontal cortex	-0.296	0.283	[-0.850 - 0.258]	2.952E-01	8.228E-01
Right medial orbitofrontal cortex	-0.090	0.253	[-0.586 - 0.406]	7.209E-01	9.578E-01
Left cuneus	-0.125	0.201	[-0.520 - 0.270]	5.351E-01	9.109E-01
Right cuneus	-0.116	0.194	[-0.497 - 0.266]	5.525E-01	9.109E-01
Left pars triangularis of inferior frontal gyrus	-0.340	0.200	[-0.732 - 0.052]	8.894E-02	7.894E-01
Right pars triangularis of inferior frontal gyrus	-0.056	0.195	[-0.438 - 0.326]	7.729E-01	9.578E-01
Left superior parietal cortex	-0.134	0.380	[-0.880 - 0.611]	7.235E-01	9.578E-01
Right superior parietal cortex	-0.097	0.312	[-0.708 - 0.513]	7.544E-01	9.578E-01
Left pars opercularis of inferior frontal gyrus	-0.080	0.407	[-0.877 - 0.717]	8.436E-01	9.578E-01
Right pars opercularis of inferior frontal gyrus	-0.315	0.211	[-0.729 - 0.099]	1.357E-01	8.227E-01
Left supramarginal gyrus	-0.132	0.229	[-0.581 - 0.316]	5.632E-01	9.109E-01
Right supramarginal gyrus	-0.098	0.195	[-0.480 - 0.283]	6.135E-01	9.578E-01
Left pericalcarine cortex	-0.094	0.267	[-0.618 - 0.429]	7.242E-01	9.578E-01
Right pericalcarine cortex	-0.087	0.225	[-0.529 - 0.354]	6.975E-01	9.578E-01
Left parahippocampal gyrus	-0.262	0.195	[-0.644 - 0.120]	1.788E-01	8.227E-01
Right parahippocampal gyrus	0.022	0.195	[-0.360 - 0.404]	9.114E-01	9.658E-01
Left caudal middle frontal gyrus	-0.044	0.214	[-0.465 - 0.376]	8.364E-01	9.578E-01
Right caudal middle frontal gyrus	-0.239	0.267	[-0.762 - 0.284]	3.708E-01	8.228E-01
Left transverse temporal gyrus	-0.400	0.397	[-1.178 - 0.378]	3.140E-01	8.228E-01
Right transverse temporal gyrus	-0.169	0.267	[-0.692 - 0.354]	5.275E-01	9.109E-01
Left banks of superior temporal sulcus	0.001	0.194	[-0.379 - 0.382]	9.943E-01	9.943E-01
Right banks of superior temporal sulcus	-0.113	0.194	[-0.494 - 0.268]	5.613E-01	9.109E-01
Left caudal anterior cingulate cortex	0.231	0.214	[-0.189 - 0.651]	2.806E-01	8.228E-01
Right caudal anterior cingulate cortex	0.049	0.194	[-0.332 - 0.430]	7.993E-01	9.578E-01
Left rostral anterior cingulate cortex	0.140	0.242	[-0.335 - 0.614]	5.645E-01	9.109E-01
Right rostral anterior cingulate cortex	0.223	0.194	[-0.158 - 0.604]	2.520E-01	8.228E-01
Left posterior cingulate cortex	-0.087	0.197	[-0.472 - 0.299]	6.587E-01	9.578E-01
Right posterior cingulate cortex	-0.084	0.194	[-0.465 - 0.296]	6.642E-01	9.578E-01
Left frontal pole	-0.219	0.243	[-0.695 - 0.257]	3.667E-01	8.228E-01
Right frontal pole	-0.142	0.195	[-0.523 - 0.240]	4.668E-01	9.109E-01
Left paracentral lobule	-0.514	0.196	[-0.898 - -0.130]	8.642E-03	2.045E-01
Right paracentral lobule	-0.183	0.194	[-0.564 - 0.198]	3.466E-01	8.228E-01
Left insula	0.058	0.358	[-0.644 - 0.761]	8.707E-01	9.578E-01
Right insula	-0.112	0.447	[-0.988 - 0.764]	8.026E-01	9.578E-01
Left entorhinal cortex	-0.063	0.218	[-0.489 - 0.364]	7.738E-01	9.578E-01
Right entorhinal cortex	-0.128	0.194	[-0.509 - 0.253]	5.108E-01	9.109E-01
Left temporal pole	-0.041	0.195	[-0.423 - 0.340]	8.329E-01	9.578E-01
Right temporal pole	0.009	0.214	[-0.411 - 0.428]	9.682E-01	9.821E-01
Left isthmus cingulate cortex	0.156	0.248	[-0.329 - 0.642]	5.277E-01	9.109E-01
Right isthmus cingulate cortex	-0.125	0.195	[-0.506 - 0.257]	5.225E-01	9.109E-01

Supplementary Table S87. Partial correlations between cortical surface area and duration of illness controlling for age and sex in individuals with major depressive disorder

	Partial *R*	Std. Err.	95% CI	*p*-value	FDR *q*-value
Total cortical surface area	-0.071	0.067	[-0.202 - 0.060]	2.907E-01	7.559E-01
Left hemisphere	-0.061	0.066	[-0.190 - 0.068]	3.557E-01	7.767E-01
Right hemisphere	-0.080	0.068	[-0.213 - 0.053]	2.387E-01	7.559E-01
Left superior frontal gyrus	-0.060	0.065	[-0.188 - 0.068]	3.584E-01	7.767E-01
Right superior frontal gyrus	-0.074	0.056	[-0.184 - 0.036]	1.874E-01	7.559E-01
Left pars orbitalis of inferior frontal gyrus	-0.027	0.056	[-0.137 - 0.084]	6.366E-01	8.512E-01
Right pars orbitalis of inferior frontal gyrus	-0.009	0.058	[-0.123 - 0.105]	8.773E-01	9.583E-01
Left precentral gyrus	-0.096	0.043	[-0.181 - -0.011]	2.609E-02	3.014E-01
Right precentral gyrus	-0.101	0.043	[-0.186 - -0.016]	1.968E-02	2.794E-01
Left lingual gyrus	-0.008	0.077	[-0.159 - 0.144]	9.217E-01	9.651E-01
Right lingual gyrus	-0.023	0.071	[-0.162 - 0.116]	7.433E-01	8.512E-01
Left superior temporal gyrus	-0.031	0.058	[-0.144 - 0.083]	5.956E-01	8.512E-01
Right superior temporal gyrus	-0.065	0.056	[-0.174 - 0.044]	2.396E-01	7.559E-01
Left fusiform gyrus	-0.049	0.058	[-0.162 - 0.065]	4.012E-01	7.767E-01
Right fusiform gyrus	-0.062	0.060	[-0.179 - 0.054]	2.946E-01	7.559E-01
Left inferior parietal cortex	-0.112	0.043	[-0.197 - -0.027]	9.585E-03	2.312E-01
Right inferior parietal cortex	-0.099	0.059	[-0.214 - 0.017]	9.327E-02	5.881E-01
Left lateral occipital cortex	-0.016	0.043	[-0.101 - 0.068]	7.064E-01	8.512E-01
Right lateral occipital cortex	-0.070	0.043	[-0.155 - 0.015]	1.063E-01	5.881E-01
Left rostral middle frontal gyrus	-0.057	0.067	[-0.188 - 0.075]	3.966E-01	7.767E-01
Right rostral middle frontal gyrus	-0.019	0.071	[-0.159 - 0.121]	7.886E-01	8.749E-01
Left precuneus	-0.024	0.080	[-0.179 - 0.132]	7.666E-01	8.640E-01
Right precuneus	-0.055	0.070	[-0.193 - 0.083]	4.341E-01	7.767E-01
Left inferior temporal gyrus	-0.044	0.059	[-0.160 - 0.072]	4.601E-01	7.767E-01
Right inferior temporal gyrus	-0.023	0.054	[-0.129 - 0.084]	6.767E-01	8.512E-01
Left lateral orbitofrontal cortex	-0.036	0.051	[-0.135 - 0.064]	4.813E-01	7.767E-01
Right lateral orbitofrontal cortex	-0.025	0.050	[-0.122 - 0.072]	6.156E-01	8.512E-01
Left middle temporal gyrus	-0.063	0.052	[-0.164 - 0.039]	2.249E-01	7.559E-01
Right middle temporal gyrus	-0.062	0.049	[-0.158 - 0.034]	2.069E-01	7.559E-01
Left postcentral gyrus	-0.076	0.053	[-0.179 - 0.028]	1.512E-01	7.156E-01
Right postcentral gyrus	-0.110	0.043	[-0.195 - -0.025]	1.086E-02	2.312E-01
Left medial orbitofrontal cortex	-0.048	0.043	[-0.133 - 0.036]	2.633E-01	7.559E-01
Right medial orbitofrontal cortex	-0.049	0.068	[-0.181 - 0.084]	4.722E-01	7.767E-01
Left cuneus	-0.050	0.048	[-0.145 - 0.044]	2.978E-01	7.559E-01
Right cuneus	-0.130	0.043	[-0.215 - -0.045]	2.625E-03	1.864E-01
Left pars triangularis of inferior frontal gyrus	-0.033	0.048	[-0.127 - 0.061]	4.937E-01	7.790E-01
Right pars triangularis of inferior frontal gyrus	0.022	0.052	[-0.080 - 0.125]	6.687E-01	8.512E-01
Left superior parietal cortex	-0.042	0.052	[-0.143 - 0.059]	4.129E-01	7.767E-01
Right superior parietal cortex	-0.031	0.064	[-0.156 - 0.093]	6.227E-01	8.512E-01
Left pars opercularis of inferior frontal gyrus	0.007	0.056	[-0.104 - 0.117]	9.065E-01	9.651E-01
Right pars opercularis of inferior frontal gyrus	-0.080	0.043	[-0.165 - 0.005]	6.501E-02	5.129E-01
Left supramarginal gyrus	-0.039	0.043	[-0.124 - 0.046]	3.648E-01	7.767E-01
Right supramarginal gyrus	-0.078	0.057	[-0.189 - 0.034]	1.712E-01	7.559E-01
Left pericalcarine cortex	-0.029	0.048	[-0.123 - 0.066]	5.516E-01	8.486E-01
Right pericalcarine cortex	-0.023	0.062	[-0.145 - 0.099]	7.148E-01	8.512E-01
Left parahippocampal gyrus	-0.025	0.072	[-0.167 - 0.116]	7.242E-01	8.512E-01
Right parahippocampal gyrus	-0.062	0.080	[-0.220 - 0.095]	4.382E-01	7.767E-01
Left caudal middle frontal gyrus	-0.044	0.056	[-0.153 - 0.065]	4.318E-01	7.767E-01
Right caudal middle frontal gyrus	-0.087	0.055	[-0.194 - 0.021]	1.159E-01	5.881E-01
Left transverse temporal gyrus	-0.003	0.067	[-0.134 - 0.128]	9.651E-01	9.651E-01
Right transverse temporal gyrus	-0.046	0.044	[-0.132 - 0.041]	2.981E-01	7.559E-01
Left banks of superior temporal sulcus	-0.084	0.053	[-0.188 - 0.021]	1.160E-01	5.881E-01
Right banks of superior temporal sulcus	-0.107	0.043	[-0.192 - -0.023]	1.303E-02	2.312E-01
Left caudal anterior cingulate cortex	-0.004	0.043	[-0.088 - 0.081]	9.337E-01	9.651E-01
Right caudal anterior cingulate cortex	-0.046	0.043	[-0.131 - 0.039]	2.869E-01	7.559E-01
Left rostral anterior cingulate cortex	-0.018	0.051	[-0.119 - 0.082]	7.210E-01	8.512E-01
Right rostral anterior cingulate cortex	-0.048	0.050	[-0.146 - 0.049]	3.304E-01	7.767E-01
Left posterior cingulate cortex	0.003	0.051	[-0.097 - 0.104]	9.501E-01	9.651E-01
Right posterior cingulate cortex	0.045	0.054	[-0.061 - 0.151]	4.079E-01	7.767E-01
Left frontal pole	-0.040	0.043	[-0.125 - 0.044]	3.504E-01	7.767E-01
Right frontal pole	-0.076	0.047	[-0.169 - 0.017]	1.090E-01	5.881E-01
Left paracentral lobule	-0.094	0.043	[-0.179 - -0.009]	2.972E-02	3.014E-01
Right paracentral lobule	-0.054	0.043	[-0.138 - 0.031]	2.151E-01	7.559E-01
Left insula	0.035	0.060	[-0.083 - 0.152]	5.618E-01	8.486E-01
Right insula	-0.017	0.043	[-0.102 - 0.068]	6.975E-01	8.512E-01
Left entorhinal cortex	0.002	0.043	[-0.083 - 0.087]	9.614E-01	9.651E-01
Right entorhinal cortex	0.015	0.043	[-0.069 - 0.100]	7.209E-01	8.512E-01
Left temporal pole	-0.118	0.058	[-0.232 - -0.003]	4.410E-02	3.914E-01
Right temporal pole	-0.031	0.043	[-0.116 - 0.053]	4.691E-01	7.767E-01
Left isthmus cingulate cortex	0.019	0.058	[-0.094 - 0.132]	7.414E-01	8.512E-01
Right isthmus cingulate cortex	0.022	0.057	[-0.089 - 0.133]	6.967E-01	8.512E-01

Supplementary Table S88. Partial correlations between cortical surface area and duration of illness controlling for age and sex in individuals with major depressive disorder over 21 years of old

	Partial *R*	Std. Err.	95% CI	*p*-value	FDR *q*-value
Total cortical surface area	-0.074	0.065	[-0.200 - 0.053]	2.543E-01	6.688E-01
Left hemisphere	-0.065	0.063	[-0.189 - 0.059]	3.064E-01	6.830E-01
Right hemisphere	-0.082	0.066	[-0.211 - 0.047]	2.134E-01	6.573E-01
Left superior frontal gyrus	-0.061	0.064	[-0.187 - 0.065]	3.427E-01	7.157E-01
Right superior frontal gyrus	-0.074	0.054	[-0.179 - 0.031]	1.677E-01	6.573E-01
Left pars orbitalis of inferior frontal gyrus	-0.037	0.055	[-0.145 - 0.071]	4.991E-01	7.382E-01
Right pars orbitalis of inferior frontal gyrus	-0.010	0.057	[-0.122 - 0.102]	8.591E-01	9.102E-01
Left precentral gyrus	-0.097	0.044	[-0.183 - -0.010]	2.822E-02	3.339E-01
Right precentral gyrus	-0.097	0.044	[-0.183 - -0.010]	2.815E-02	3.339E-01
Left lingual gyrus	-0.013	0.078	[-0.165 - 0.140]	8.717E-01	9.102E-01
Right lingual gyrus	-0.028	0.071	[-0.168 - 0.111]	6.911E-01	8.707E-01
Left superior temporal gyrus	-0.030	0.054	[-0.137 - 0.076]	5.787E-01	8.218E-01
Right superior temporal gyrus	-0.069	0.053	[-0.174 - 0.035]	1.937E-01	6.573E-01
Left fusiform gyrus	-0.041	0.056	[-0.151 - 0.069]	4.641E-01	7.197E-01
Right fusiform gyrus	-0.064	0.060	[-0.181 - 0.053]	2.817E-01	6.830E-01
Left inferior parietal cortex	-0.117	0.044	[-0.203 - -0.031]	7.953E-03	1.993E-01
Right inferior parietal cortex	-0.101	0.059	[-0.216 - 0.014]	8.488E-02	6.026E-01
Left lateral occipital cortex	-0.017	0.044	[-0.103 - 0.069]	6.999E-01	8.707E-01
Right lateral occipital cortex	-0.072	0.044	[-0.158 - 0.015]	1.040E-01	6.155E-01
Left rostral middle frontal gyrus	-0.063	0.068	[-0.195 - 0.070]	3.533E-01	7.168E-01
Right rostral middle frontal gyrus	-0.020	0.071	[-0.160 - 0.119]	7.756E-01	8.707E-01
Left precuneus	-0.022	0.079	[-0.177 - 0.133]	7.824E-01	8.707E-01
Right precuneus	-0.058	0.070	[-0.195 - 0.079]	4.042E-01	7.175E-01
Left inferior temporal gyrus	-0.058	0.057	[-0.169 - 0.054]	3.103E-01	6.830E-01
Right inferior temporal gyrus	-0.021	0.053	[-0.126 - 0.084]	6.929E-01	8.707E-01
Left lateral orbitofrontal cortex	-0.042	0.048	[-0.135 - 0.052]	3.831E-01	7.175E-01
Right lateral orbitofrontal cortex	-0.035	0.047	[-0.127 - 0.057]	4.532E-01	7.197E-01
Left middle temporal gyrus	-0.074	0.048	[-0.169 - 0.020]	1.225E-01	6.573E-01
Right middle temporal gyrus	-0.060	0.048	[-0.154 - 0.033]	2.064E-01	6.573E-01
Left postcentral gyrus	-0.072	0.055	[-0.180 - 0.035]	1.863E-01	6.573E-01
Right postcentral gyrus	-0.112	0.044	[-0.199 - -0.026]	1.063E-02	1.993E-01
Left medial orbitofrontal cortex	-0.048	0.044	[-0.134 - 0.039]	2.791E-01	6.830E-01
Right medial orbitofrontal cortex	-0.048	0.068	[-0.181 - 0.084]	4.764E-01	7.197E-01
Left cuneus	-0.063	0.044	[-0.149 - 0.023]	1.511E-01	6.573E-01
Right cuneus	-0.122	0.048	[-0.216 - -0.029]	9.957E-03	1.993E-01
Left pars triangularis of inferior frontal gyrus	-0.045	0.044	[-0.132 - 0.041]	3.027E-01	6.830E-01
Right pars triangularis of inferior frontal gyrus	0.016	0.052	[-0.085 - 0.118]	7.518E-01	8.707E-01
Left superior parietal cortex	-0.042	0.053	[-0.147 - 0.062]	4.259E-01	7.197E-01
Right superior parietal cortex	-0.033	0.064	[-0.160 - 0.093]	6.055E-01	8.430E-01
Left pars opercularis of inferior frontal gyrus	0.002	0.053	[-0.102 - 0.106]	9.657E-01	9.657E-01
Right pars opercularis of inferior frontal gyrus	-0.085	0.044	[-0.172 - 0.001]	5.221E-02	4.119E-01
Left supramarginal gyrus	-0.044	0.044	[-0.130 - 0.042]	3.175E-01	6.830E-01
Right supramarginal gyrus	-0.080	0.058	[-0.194 - 0.035]	1.718E-01	6.573E-01
Left pericalcarine cortex	-0.042	0.046	[-0.132 - 0.049]	3.698E-01	7.175E-01
Right pericalcarine cortex	-0.026	0.064	[-0.152 - 0.100]	6.838E-01	8.707E-01
Left parahippocampal gyrus	-0.016	0.075	[-0.163 - 0.131]	8.353E-01	9.096E-01
Right parahippocampal gyrus	-0.059	0.081	[-0.218 - 0.100]	4.652E-01	7.197E-01
Left caudal middle frontal gyrus	-0.047	0.056	[-0.158 - 0.063]	4.007E-01	7.175E-01
Right caudal middle frontal gyrus	-0.093	0.056	[-0.202 - 0.017]	9.773E-02	6.155E-01
Left transverse temporal gyrus	-0.006	0.066	[-0.135 - 0.124]	9.298E-01	9.541E-01
Right transverse temporal gyrus	-0.051	0.044	[-0.137 - 0.036]	2.508E-01	6.688E-01
Left banks of superior temporal sulcus	-0.084	0.056	[-0.193 - 0.026]	1.335E-01	6.573E-01
Right banks of superior temporal sulcus	-0.112	0.044	[-0.198 - -0.025]	1.123E-02	1.993E-01
Left caudal anterior cingulate cortex	-0.003	0.044	[-0.090 - 0.083]	9.407E-01	9.541E-01
Right caudal anterior cingulate cortex	-0.054	0.044	[-0.140 - 0.033]	2.222E-01	6.573E-01
Left rostral anterior cingulate cortex	-0.017	0.048	[-0.111 - 0.076]	7.173E-01	8.707E-01
Right rostral anterior cingulate cortex	-0.057	0.045	[-0.146 - 0.032]	2.120E-01	6.573E-01
Left posterior cingulate cortex	-0.014	0.046	[-0.105 - 0.077]	7.628E-01	8.707E-01
Right posterior cingulate cortex	0.038	0.052	[-0.065 - 0.141]	4.693E-01	7.197E-01
Left frontal pole	-0.038	0.044	[-0.124 - 0.049]	3.935E-01	7.175E-01
Right frontal pole	-0.073	0.053	[-0.177 - 0.030]	1.653E-01	6.573E-01
Left paracentral lobule	-0.093	0.044	[-0.179 - -0.006]	3.516E-02	3.566E-01
Right paracentral lobule	-0.052	0.044	[-0.138 - 0.034]	2.389E-01	6.688E-01
Left insula	0.036	0.056	[-0.075 - 0.146]	5.278E-01	7.648E-01
Right insula	-0.013	0.044	[-0.099 - 0.073]	7.689E-01	8.707E-01
Left entorhinal cortex	0.009	0.044	[-0.078 - 0.095]	8.456E-01	9.096E-01
Right entorhinal cortex	0.015	0.044	[-0.071 - 0.102]	7.288E-01	8.707E-01
Left temporal pole	-0.120	0.060	[-0.236 - -0.003]	4.481E-02	3.976E-01
Right temporal pole	-0.035	0.044	[-0.121 - 0.052]	4.303E-01	7.197E-01
Left isthmus cingulate cortex	0.019	0.057	[-0.092 - 0.131]	7.334E-01	8.707E-01
Right isthmus cingulate cortex	0.015	0.055	[-0.093 - 0.123]	7.849E-01	8.707E-01

Supplementary Table S89. Partial correlations between cortical surface area and age controlling for sex in individuals with autism spectrum disorder

	Partial *R*	Std. Err.	95% CI	*p*-value	FDR *q*-value
Total cortical surface area	-0.157	0.100	[-0.353 - 0.039]	1.156E-01	3.956E-01
Left hemisphere	-0.159	0.097	[-0.350 - 0.032]	1.019E-01	3.956E-01
Right hemisphere	-0.153	0.103	[-0.354 - 0.048]	1.361E-01	3.956E-01
Left superior frontal gyrus	-0.228	0.074	[-0.373 - -0.082]	2.135E-03	7.581E-02
Right superior frontal gyrus	-0.194	0.074	[-0.339 - -0.048]	9.004E-03	1.677E-01
Left pars orbitalis of inferior frontal gyrus	-0.114	0.074	[-0.259 - 0.031]	1.234E-01	3.956E-01
Right pars orbitalis of inferior frontal gyrus	0.012	0.101	[-0.186 - 0.210]	9.044E-01	9.493E-01
Left precentral gyrus	0.006	0.074	[-0.139 - 0.151]	9.359E-01	9.493E-01
Right precentral gyrus	-0.190	0.077	[-0.341 - -0.038]	1.417E-02	1.677E-01
Left lingual gyrus	0.071	0.103	[-0.130 - 0.273]	4.881E-01	6.931E-01
Right lingual gyrus	0.014	0.118	[-0.217 - 0.245]	9.063E-01	9.493E-01
Left superior temporal gyrus	-0.103	0.074	[-0.248 - 0.043]	1.657E-01	3.956E-01
Right superior temporal gyrus	-0.090	0.084	[-0.256 - 0.076]	2.865E-01	5.467E-01
Left fusiform gyrus	-0.078	0.074	[-0.223 - 0.067]	2.926E-01	5.467E-01
Right fusiform gyrus	-0.060	0.107	[-0.269 - 0.150]	5.780E-01	7.753E-01
Left inferior parietal cortex	-0.147	0.103	[-0.350 - 0.055]	1.539E-01	3.956E-01
Right inferior parietal cortex	-0.114	0.074	[-0.259 - 0.032]	1.258E-01	3.956E-01
Left lateral occipital cortex	-0.172	0.088	[-0.345 - 0.001]	5.200E-02	3.827E-01
Right lateral occipital cortex	-0.099	0.074	[-0.244 - 0.047]	1.834E-01	4.070E-01
Left rostral middle frontal gyrus	-0.189	0.117	[-0.419 - 0.041]	1.067E-01	3.956E-01
Right rostral middle frontal gyrus	-0.188	0.136	[-0.454 - 0.077]	1.650E-01	3.956E-01
Left precuneus	-0.150	0.080	[-0.307 - 0.008]	6.221E-02	3.956E-01
Right precuneus	-0.168	0.074	[-0.313 - -0.022]	2.368E-02	2.401E-01
Left inferior temporal gyrus	-0.107	0.076	[-0.255 - 0.041]	1.558E-01	3.956E-01
Right inferior temporal gyrus	-0.130	0.074	[-0.275 - 0.015]	7.928E-02	3.956E-01
Left lateral orbitofrontal cortex	-0.163	0.101	[-0.361 - 0.035]	1.070E-01	3.956E-01
Right lateral orbitofrontal cortex	-0.128	0.119	[-0.360 - 0.105]	2.817E-01	5.467E-01
Left middle temporal gyrus	-0.044	0.084	[-0.208 - 0.120]	5.982E-01	7.866E-01
Right middle temporal gyrus	-0.054	0.074	[-0.199 - 0.091]	4.648E-01	6.914E-01
Left postcentral gyrus	-0.054	0.074	[-0.199 - 0.091]	4.674E-01	6.914E-01
Right postcentral gyrus	-0.101	0.074	[-0.246 - 0.044]	1.727E-01	3.956E-01
Left medial orbitofrontal cortex	-0.049	0.116	[-0.276 - 0.178]	6.714E-01	8.451E-01
Right medial orbitofrontal cortex	-0.143	0.074	[-0.288 - 0.002]	5.390E-02	3.827E-01
Left cuneus	-0.057	0.076	[-0.207 - 0.092]	4.543E-01	6.914E-01
Right cuneus	-0.079	0.134	[-0.341 - 0.183]	5.564E-01	7.747E-01
Left pars triangularis of inferior frontal gyrus	-0.041	0.121	[-0.279 - 0.196]	7.337E-01	8.733E-01
Right pars triangularis of inferior frontal gyrus	-0.160	0.101	[-0.358 - 0.037]	1.114E-01	3.956E-01
Left superior parietal cortex	-0.149	0.094	[-0.334 - 0.036]	1.139E-01	3.956E-01
Right superior parietal cortex	-0.084	0.074	[-0.229 - 0.061]	2.573E-01	5.288E-01
Left pars opercularis of inferior frontal gyrus	-0.137	0.075	[-0.284 - 0.010]	6.700E-02	3.956E-01
Right pars opercularis of inferior frontal gyrus	-0.136	0.096	[-0.323 - 0.052]	1.563E-01	3.956E-01
Left supramarginal gyrus	-0.094	0.101	[-0.293 - 0.105]	3.541E-01	5.986E-01
Right supramarginal gyrus	-0.004	0.074	[-0.150 - 0.141]	9.531E-01	9.531E-01
Left pericalcarine cortex	-0.020	0.114	[-0.244 - 0.204]	8.586E-01	9.493E-01
Right pericalcarine cortex	0.077	0.074	[-0.069 - 0.222]	3.006E-01	5.473E-01
Left parahippocampal gyrus	0.057	0.074	[-0.089 - 0.202]	4.447E-01	6.914E-01
Right parahippocampal gyrus	-0.077	0.080	[-0.234 - 0.080]	3.339E-01	5.927E-01
Left caudal middle frontal gyrus	-0.065	0.074	[-0.211 - 0.080]	3.775E-01	6.233E-01
Right caudal middle frontal gyrus	-0.185	0.074	[-0.330 - -0.040]	1.262E-02	1.677E-01
Left transverse temporal gyrus	0.052	0.074	[-0.093 - 0.197]	4.825E-01	6.931E-01
Right transverse temporal gyrus	-0.007	0.081	[-0.166 - 0.151]	9.276E-01	9.493E-01
Left banks of superior temporal sulcus	-0.118	0.082	[-0.280 - 0.043]	1.519E-01	3.956E-01
Right banks of superior temporal sulcus	-0.075	0.103	[-0.276 - 0.126]	4.621E-01	6.914E-01
Left caudal anterior cingulate cortex	-0.161	0.123	[-0.402 - 0.081]	1.919E-01	4.129E-01
Right caudal anterior cingulate cortex	-0.124	0.132	[-0.383 - 0.134]	3.446E-01	5.967E-01
Left rostral anterior cingulate cortex	-0.048	0.120	[-0.284 - 0.188]	6.903E-01	8.451E-01
Right rostral anterior cingulate cortex	-0.036	0.083	[-0.199 - 0.127]	6.652E-01	8.451E-01
Left posterior cingulate cortex	-0.126	0.075	[-0.273 - 0.021]	9.375E-02	3.956E-01
Right posterior cingulate cortex	-0.108	0.079	[-0.262 - 0.047]	1.717E-01	3.956E-01
Left frontal pole	-0.251	0.074	[-0.397 - -0.106]	6.960E-04	4.942E-02
Right frontal pole	-0.152	0.074	[-0.298 - -0.007]	3.975E-02	3.528E-01
Left paracentral lobule	-0.190	0.074	[-0.336 - -0.045]	1.019E-02	1.677E-01
Right paracentral lobule	-0.041	0.131	[-0.298 - 0.215]	7.508E-01	8.739E-01
Left insula	-0.083	0.074	[-0.229 - 0.062]	2.607E-01	5.288E-01
Right insula	-0.024	0.086	[-0.192 - 0.144]	7.781E-01	8.911E-01
Left entorhinal cortex	0.015	0.101	[-0.184 - 0.213]	8.841E-01	9.493E-01
Right entorhinal cortex	0.041	0.074	[-0.104 - 0.186]	5.788E-01	7.753E-01
Left temporal pole	0.015	0.116	[-0.212 - 0.241]	8.989E-01	9.493E-01
Right temporal pole	0.013	0.116	[-0.214 - 0.239]	9.130E-01	9.493E-01
Left isthmus cingulate cortex	-0.031	0.074	[-0.176 - 0.115]	6.791E-01	8.451E-01
Right isthmus cingulate cortex	0.030	0.089	[-0.144 - 0.204]	7.380E-01	8.733E-01

Supplementary Table S90. Cortical thickness differences between individuals with bipolar disorder type-1 compared to individuals with bipolar disorder type-2 controlling for age and sex

	d	Std. Err.	95% CI	*p*-value	FDR *q*-value
Global mean cortical thickness	0.455	0.215	[0.034 - 0.876]	3.420E-02	2.698E-01
Left hemisphere	0.371	0.214	[-0.048 - 0.789]	8.243E-02	4.008E-01
Right hemisphere	0.535	0.217	[0.111 - 0.959]	1.346E-02	1.982E-01
Left fusiform gyrus	0.315	0.350	[-0.371 - 1.000]	3.684E-01	5.968E-01
Right fusiform gyrus	0.625	0.217	[0.200 - 1.051]	3.974E-03	1.465E-01
Left pars opercularis of inferior frontal gyrus	0.291	0.213	[-0.127 - 0.709]	1.729E-01	4.484E-01
Right pars opercularis of inferior frontal gyrus	0.348	0.214	[-0.071 - 0.767]	1.033E-01	4.315E-01
Left superior temporal gyrus	0.411	0.238	[-0.056 - 0.877]	8.467E-02	4.008E-01
Right superior temporal gyrus	0.533	0.217	[0.108 - 0.957]	1.396E-02	1.982E-01
Left insula	0.301	0.238	[-0.166 - 0.768]	2.060E-01	4.484E-01
Right insula	0.411	0.215	[-0.010 - 0.833]	5.592E-02	3.312E-01
Left lingual gyrus	0.328	0.213	[-0.089 - 0.746]	1.232E-01	4.459E-01
Right lingual gyrus	0.500	0.215	[0.079 - 0.921]	1.995E-02	2.024E-01
Left pars triangularis of inferior frontal gyrus	0.173	0.213	[-0.244 - 0.591]	4.157E-01	6.023E-01
Right pars triangularis of inferior frontal gyrus	0.251	0.213	[-0.167 - 0.669]	2.391E-01	4.589E-01
Left lateral orbitofrontal cortex	0.173	0.293	[-0.401 - 0.748]	5.544E-01	7.718E-01
Right lateral orbitofrontal cortex	0.650	0.420	[-0.173 - 1.473]	1.216E-01	4.459E-01
Left rostral middle frontal gyrus	0.041	0.211	[-0.373 - 0.455]	8.451E-01	9.077E-01
Right rostral middle frontal gyrus	0.197	0.212	[-0.219 - 0.613]	3.533E-01	5.968E-01
Left middle temporal gyrus	0.409	0.214	[-0.010 - 0.829]	5.598E-02	3.312E-01
Right middle temporal gyrus	0.089	0.215	[-0.332 - 0.510]	6.796E-01	8.665E-01
Left superior frontal gyrus	0.297	0.214	[-0.123 - 0.717]	1.652E-01	4.484E-01
Right superior frontal gyrus	0.275	0.214	[-0.145 - 0.695]	1.988E-01	4.484E-01
Left pars orbitalis of inferior frontal gyrus	0.085	0.211	[-0.329 - 0.500]	6.861E-01	8.665E-01
Right pars orbitalis of inferior frontal gyrus	0.299	0.214	[-0.120 - 0.718]	1.622E-01	4.484E-01
Left medial orbitofrontal cortex	0.128	0.212	[-0.288 - 0.544]	5.474E-01	7.718E-01
Right medial orbitofrontal cortex	0.300	0.225	[-0.140 - 0.740]	1.819E-01	4.484E-01
Left inferior temporal gyrus	0.286	0.216	[-0.136 - 0.709]	1.841E-01	4.484E-01
Right inferior temporal gyrus	0.465	0.380	[-0.280 - 1.210]	2.212E-01	4.484E-01
Left isthmus cingulate cortex	0.344	0.257	[-0.161 - 0.848]	1.817E-01	4.484E-01
Right isthmus cingulate cortex	0.064	0.321	[-0.565 - 0.693]	8.409E-01	9.077E-01
Left banks of superior temporal sulcus	-0.003	0.212	[-0.418 - 0.412]	9.896E-01	9.896E-01
Right banks of superior temporal sulcus	-0.052	0.213	[-0.470 - 0.366]	8.078E-01	9.077E-01
Left supramarginal gyrus	0.276	0.218	[-0.152 - 0.704]	2.057E-01	4.484E-01
Right supramarginal gyrus	0.502	0.215	[0.080 - 0.924]	1.979E-02	2.024E-01
Left caudal middle frontal gyrus	0.377	0.310	[-0.230 - 0.985]	2.232E-01	4.484E-01
Right caudal middle frontal gyrus	0.327	0.214	[-0.092 - 0.746]	1.256E-01	4.459E-01
Left frontal pole	0.238	0.283	[-0.318 - 0.793]	4.015E-01	5.968E-01
Right frontal pole	-0.053	0.356	[-0.749 - 0.644]	8.822E-01	9.077E-01
Left posterior cingulate cortex	0.337	0.391	[-0.429 - 1.103]	3.881E-01	5.968E-01
Right posterior cingulate cortex	0.311	0.214	[-0.108 - 0.731]	1.454E-01	4.484E-01
Left lateral occipital cortex	0.106	0.212	[-0.310 - 0.522]	6.169E-01	8.281E-01
Right lateral occipital cortex	0.388	0.213	[-0.030 - 0.806]	6.891E-02	3.764E-01
Left precentral gyrus	0.356	0.213	[-0.062 - 0.774]	9.496E-02	4.214E-01
Right precentral gyrus	0.246	0.214	[-0.174 - 0.665]	2.513E-01	4.696E-01
Left parahippocampal gyrus	-0.073	0.212	[-0.489 - 0.343]	7.311E-01	8.916E-01
Right parahippocampal gyrus	-0.056	0.212	[-0.472 - 0.359]	7.911E-01	9.077E-01
Left inferior parietal cortex	0.263	0.214	[-0.156 - 0.683]	2.185E-01	4.484E-01
Right inferior parietal cortex	0.419	0.215	[-0.002 - 0.840]	5.115E-02	3.312E-01
Left transverse temporal gyrus	0.593	0.217	[0.168 - 1.017]	6.191E-03	1.465E-01
Right transverse temporal gyrus	0.047	0.271	[-0.484 - 0.578]	8.622E-01	9.077E-01
Left postcentral gyrus	0.178	0.213	[-0.239 - 0.594]	4.034E-01	5.968E-01
Right postcentral gyrus	-0.039	0.211	[-0.453 - 0.375]	8.525E-01	9.077E-01
Left precuneus	0.401	0.298	[-0.183 - 0.984]	1.784E-01	4.484E-01
Right precuneus	0.341	0.242	[-0.134 - 0.815]	1.594E-01	4.484E-01
Left caudal anterior cingulate cortex	0.257	0.213	[-0.160 - 0.674]	2.274E-01	4.484E-01
Right caudal anterior cingulate cortex	0.056	0.255	[-0.443 - 0.555]	8.259E-01	9.077E-01
Left cuneus	0.027	0.211	[-0.387 - 0.442]	8.968E-01	9.096E-01
Right cuneus	0.265	0.268	[-0.261 - 0.791]	3.240E-01	5.899E-01
Left rostral anterior cingulate cortex	0.184	0.213	[-0.233 - 0.601]	3.871E-01	5.968E-01
Right rostral anterior cingulate cortex	0.207	0.216	[-0.216 - 0.630]	3.377E-01	5.968E-01
Left pericalcarine cortex	0.110	0.221	[-0.323 - 0.544]	6.181E-01	8.281E-01
Right pericalcarine cortex	-0.083	0.213	[-0.501 - 0.335]	6.957E-01	8.665E-01
Left paracentral lobule	0.483	0.216	[0.060 - 0.906]	2.524E-02	2.240E-01
Right paracentral lobule	0.609	0.218	[0.181 - 1.037]	5.309E-03	1.465E-01
Left superior parietal cortex	0.033	0.212	[-0.383 - 0.449]	8.764E-01	9.077E-01
Right superior parietal cortex	0.190	0.213	[-0.228 - 0.608]	3.722E-01	5.968E-01
Left temporal pole	-0.192	0.226	[-0.635 - 0.250]	3.943E-01	5.968E-01
Right temporal pole	0.083	0.213	[-0.334 - 0.501]	6.955E-01	8.665E-01
Left entorhinal cortex	-0.049	0.218	[-0.475 - 0.378]	8.231E-01	9.077E-01
Right entorhinal cortex	-0.070	0.213	[-0.488 - 0.347]	7.409E-01	8.916E-01

Supplementary Table S91. Cortical thickness differences between individuals with bipolar disorder type-1 compared to individuals with bipolar disorder type-2 controlling for age and sex at 25 years of age or older

	d	Std. Err.	95% CI	*p*-value	FDR *q*-value
Global mean cortical thickness	0.354	0.231	[-0.098 - 0.807]	1.247E-01	7.507E-01
Left hemisphere	0.282	0.230	[-0.169 - 0.732]	2.206E-01	7.507E-01
Right hemisphere	0.422	0.232	[-0.032 - 0.877]	6.862E-02	6.960E-01
Left fusiform gyrus	0.067	0.294	[-0.510 - 0.643]	8.211E-01	9.631E-01
Right fusiform gyrus	0.639	0.235	[0.177 - 1.100]	6.651E-03	2.361E-01
Left pars opercularis of inferior frontal gyrus	0.188	0.231	[-0.264 - 0.641]	4.143E-01	8.825E-01
Right pars opercularis of inferior frontal gyrus	0.241	0.230	[-0.210 - 0.693]	2.945E-01	8.042E-01
Left superior temporal gyrus	0.358	0.293	[-0.217 - 0.932]	2.220E-01	7.507E-01
Right superior temporal gyrus	0.500	0.263	[-0.015 - 1.015]	5.697E-02	6.960E-01
Left insula	0.153	0.250	[-0.337 - 0.642]	5.409E-01	8.825E-01
Right insula	0.262	0.230	[-0.189 - 0.713]	2.551E-01	7.641E-01
Left lingual gyrus	0.326	0.231	[-0.126 - 0.778]	1.575E-01	7.507E-01
Right lingual gyrus	0.434	0.232	[-0.020 - 0.889]	6.074E-02	6.960E-01
Left pars triangularis of inferior frontal gyrus	0.106	0.256	[-0.395 - 0.608]	6.777E-01	9.535E-01
Right pars triangularis of inferior frontal gyrus	0.153	0.231	[-0.299 - 0.606]	5.060E-01	8.825E-01
Left lateral orbitofrontal cortex	-0.033	0.230	[-0.484 - 0.418]	8.851E-01	9.752E-01
Right lateral orbitofrontal cortex	0.288	0.313	[-0.327 - 0.902]	3.587E-01	8.489E-01
Left rostral middle frontal gyrus	0.003	0.229	[-0.446 - 0.451]	9.907E-01	9.936E-01
Right rostral middle frontal gyrus	0.152	0.230	[-0.299 - 0.603]	5.085E-01	8.825E-01
Left middle temporal gyrus	0.364	0.231	[-0.089 - 0.817]	1.154E-01	7.507E-01
Right middle temporal gyrus	-0.031	0.230	[-0.482 - 0.420]	8.928E-01	9.752E-01
Left superior frontal gyrus	0.165	0.229	[-0.284 - 0.615]	4.716E-01	8.825E-01
Right superior frontal gyrus	0.161	0.231	[-0.292 - 0.614]	4.865E-01	8.825E-01
Left pars orbitalis of inferior frontal gyrus	-0.004	0.229	[-0.452 - 0.444]	9.854E-01	9.936E-01
Right pars orbitalis of inferior frontal gyrus	0.192	0.258	[-0.314 - 0.698]	4.577E-01	8.825E-01
Left medial orbitofrontal cortex	0.042	0.229	[-0.407 - 0.490]	8.546E-01	9.631E-01
Right medial orbitofrontal cortex	0.169	0.231	[-0.284 - 0.622]	4.655E-01	8.825E-01
Left inferior temporal gyrus	0.314	0.244	[-0.165 - 0.793]	1.987E-01	7.507E-01
Right inferior temporal gyrus	0.199	0.327	[-0.441 - 0.839]	5.417E-01	8.825E-01
Left isthmus cingulate cortex	0.230	0.247	[-0.254 - 0.714]	3.521E-01	8.489E-01
Right isthmus cingulate cortex	-0.179	0.308	[-0.782 - 0.424]	5.610E-01	8.825E-01
Left banks of superior temporal sulcus	0.019	0.229	[-0.431 - 0.469]	9.343E-01	9.901E-01
Right banks of superior temporal sulcus	-0.082	0.240	[-0.553 - 0.389]	7.322E-01	9.535E-01
Left supramarginal gyrus	0.246	0.251	[-0.246 - 0.737]	3.276E-01	8.306E-01
Right supramarginal gyrus	0.437	0.232	[-0.017 - 0.892]	5.931E-02	6.960E-01
Left caudal middle frontal gyrus	0.454	0.401	[-0.333 - 1.240]	2.583E-01	7.641E-01
Right caudal middle frontal gyrus	0.329	0.232	[-0.125 - 0.783]	1.556E-01	7.507E-01
Left frontal pole	-0.002	0.230	[-0.453 - 0.449]	9.936E-01	9.936E-01
Right frontal pole	-0.010	0.422	[-0.838 - 0.818]	9.811E-01	9.936E-01
Left posterior cingulate cortex	0.065	0.353	[-0.627 - 0.756]	8.545E-01	9.631E-01
Right posterior cingulate cortex	0.230	0.230	[-0.222 - 0.681]	3.190E-01	8.306E-01
Left lateral occipital cortex	0.131	0.230	[-0.320 - 0.582]	5.699E-01	8.825E-01
Right lateral occipital cortex	0.365	0.231	[-0.088 - 0.817]	1.140E-01	7.507E-01
Left precentral gyrus	0.304	0.231	[-0.149 - 0.756]	1.884E-01	7.507E-01
Right precentral gyrus	0.130	0.230	[-0.322 - 0.582]	5.733E-01	8.825E-01
Left parahippocampal gyrus	-0.152	0.230	[-0.602 - 0.298]	5.071E-01	8.825E-01
Right parahippocampal gyrus	-0.126	0.229	[-0.575 - 0.323]	5.823E-01	8.825E-01
Left inferior parietal cortex	0.253	0.233	[-0.203 - 0.710]	2.769E-01	7.863E-01
Right inferior parietal cortex	0.320	0.230	[-0.131 - 0.772]	1.642E-01	7.507E-01
Left transverse temporal gyrus	0.689	0.235	[0.228 - 1.151]	3.406E-03	2.361E-01
Right transverse temporal gyrus	0.081	0.374	[-0.652 - 0.814]	8.290E-01	9.631E-01
Left postcentral gyrus	0.092	0.229	[-0.358 - 0.541]	6.894E-01	9.535E-01
Right postcentral gyrus	-0.072	0.229	[-0.520 - 0.376]	7.532E-01	9.535E-01
Left precuneus	0.126	0.231	[-0.326 - 0.578]	5.842E-01	8.825E-01
Right precuneus	0.165	0.230	[-0.285 - 0.615]	4.716E-01	8.825E-01
Left caudal anterior cingulate cortex	0.286	0.231	[-0.166 - 0.738]	2.150E-01	7.507E-01
Right caudal anterior cingulate cortex	-0.056	0.273	[-0.591 - 0.479]	8.378E-01	9.631E-01
Left cuneus	0.049	0.229	[-0.400 - 0.498]	8.309E-01	9.631E-01
Right cuneus	0.252	0.332	[-0.399 - 0.903]	4.489E-01	8.825E-01
Left rostral anterior cingulate cortex	0.074	0.229	[-0.375 - 0.523]	7.472E-01	9.535E-01
Right rostral anterior cingulate cortex	0.297	0.231	[-0.156 - 0.751]	1.987E-01	7.507E-01
Left pericalcarine cortex	0.276	0.231	[-0.178 - 0.729]	2.333E-01	7.529E-01
Right pericalcarine cortex	-0.022	0.241	[-0.494 - 0.450]	9.270E-01	9.901E-01
Left paracentral lobule	0.346	0.231	[-0.106 - 0.798]	1.332E-01	7.507E-01
Right paracentral lobule	0.471	0.232	[0.015 - 0.926]	4.282E-02	6.960E-01
Left superior parietal cortex	-0.068	0.229	[-0.517 - 0.380]	7.655E-01	9.535E-01
Right superior parietal cortex	0.096	0.229	[-0.354 - 0.545]	6.767E-01	9.535E-01
Left temporal pole	-0.337	0.231	[-0.789 - 0.115]	1.438E-01	7.507E-01
Right temporal pole	0.094	0.240	[-0.377 - 0.565]	6.955E-01	9.535E-01
Left entorhinal cortex	-0.075	0.247	[-0.560 - 0.410]	7.613E-01	9.535E-01
Right entorhinal cortex	-0.072	0.231	[-0.524 - 0.380]	7.554E-01	9.535E-01

Supplementary Table S92. Cortical thickness differences between individuals with first episode major depressive disorder versus healthy comparison subjects controlling for age and sex

	d	Std. Err.	95% CI	*p*-value	FDR *q*-value
Global mean cortical thickness	-0.367	0.098	[-0.559 - -0.175]	1.797E-04	2.126E-03
Left hemisphere	-0.382	0.098	[-0.574 - -0.190]	9.795E-05	1.946E-03
Right hemisphere	-0.333	0.108	[-0.544 - -0.121]	2.025E-03	9.585E-03
Left fusiform gyrus	-0.341	0.098	[-0.533 - -0.149]	4.948E-04	3.751E-03
Right fusiform gyrus	-0.330	0.118	[-0.560 - -0.099]	5.100E-03	1.811E-02
Left pars opercularis of inferior frontal gyrus	-0.379	0.098	[-0.570 - -0.187]	1.097E-04	1.946E-03
Right pars opercularis of inferior frontal gyrus	-0.258	0.103	[-0.461 - -0.056]	1.250E-02	3.617E-02
Left superior temporal gyrus	-0.338	0.098	[-0.530 - -0.147]	5.447E-04	3.751E-03
Right superior temporal gyrus	-0.130	0.121	[-0.368 - 0.107]	2.819E-01	3.707E-01
Left insula	-0.295	0.103	[-0.496 - -0.093]	4.143E-03	1.657E-02
Right insula	-0.192	0.149	[-0.484 - 0.100]	1.974E-01	2.860E-01
Left lingual gyrus	-0.275	0.098	[-0.466 - -0.084]	4.805E-03	1.796E-02
Right lingual gyrus	-0.155	0.106	[-0.363 - 0.053]	1.438E-01	2.220E-01
Left pars triangularis of inferior frontal gyrus	-0.178	0.097	[-0.368 - 0.013]	6.815E-02	1.308E-01
Right pars triangularis of inferior frontal gyrus	-0.344	0.098	[-0.535 - -0.152]	4.350E-04	3.751E-03
Left lateral orbitofrontal cortex	-0.433	0.098	[-0.625 - -0.241]	1.021E-05	7.073E-04
Right lateral orbitofrontal cortex	-0.272	0.131	[-0.527 - -0.016]	3.756E-02	7.844E-02
Left rostral middle frontal gyrus	-0.356	0.104	[-0.559 - -0.153]	5.811E-04	3.751E-03
Right rostral middle frontal gyrus	-0.281	0.128	[-0.531 - -0.030]	2.804E-02	6.032E-02
Left middle temporal gyrus	-0.418	0.098	[-0.610 - -0.226]	1.992E-05	7.073E-04
Right middle temporal gyrus	-0.257	0.144	[-0.539 - 0.024]	7.307E-02	1.365E-01
Left superior frontal gyrus	-0.369	0.098	[-0.560 - -0.177]	1.658E-04	2.126E-03
Right superior frontal gyrus	-0.320	0.098	[-0.512 - -0.129]	1.045E-03	5.298E-03
Left pars orbitalis of inferior frontal gyrus	-0.334	0.143	[-0.615 - -0.053]	1.980E-02	4.393E-02
Right pars orbitalis of inferior frontal gyrus	-0.332	0.125	[-0.577 - -0.087]	7.948E-03	2.565E-02
Left medial orbitofrontal cortex	-0.289	0.119	[-0.522 - -0.055]	1.526E-02	3.687E-02
Right medial orbitofrontal cortex	-0.343	0.120	[-0.577 - -0.108]	4.202E-03	1.657E-02
Left inferior temporal gyrus	-0.295	0.098	[-0.486 - -0.103]	2.540E-03	1.127E-02
Right inferior temporal gyrus	-0.237	0.098	[-0.428 - -0.046]	1.521E-02	3.687E-02
Left isthmus cingulate cortex	-0.174	0.126	[-0.420 - 0.072]	1.661E-01	2.457E-01
Right isthmus cingulate cortex	-0.235	0.098	[-0.426 - -0.044]	1.610E-02	3.687E-02
Left banks of superior temporal sulcus	-0.221	0.133	[-0.481 - 0.039]	9.547E-02	1.614E-01
Right banks of superior temporal sulcus	-0.152	0.159	[-0.463 - 0.160]	3.407E-01	4.319E-01
Left supramarginal gyrus	-0.118	0.097	[-0.309 - 0.073]	2.254E-01	3.078E-01
Right supramarginal gyrus	-0.266	0.098	[-0.457 - -0.074]	6.541E-03	2.212E-02
Left caudal middle frontal gyrus	-0.243	0.097	[-0.434 - -0.052]	1.273E-02	3.617E-02
Right caudal middle frontal gyrus	-0.350	0.098	[-0.542 - -0.159]	3.401E-04	3.449E-03
Left frontal pole	-0.249	0.098	[-0.440 - -0.057]	1.086E-02	3.352E-02
Right frontal pole	-0.408	0.167	[-0.736 - -0.081]	1.463E-02	3.687E-02
Left posterior cingulate cortex	-0.136	0.097	[-0.326 - 0.055]	1.638E-01	2.457E-01
Right posterior cingulate cortex	-0.235	0.097	[-0.426 - -0.044]	1.606E-02	3.687E-02
Left lateral occipital cortex	-0.240	0.098	[-0.431 - -0.048]	1.408E-02	3.687E-02
Right lateral occipital cortex	-0.215	0.126	[-0.463 - 0.032]	8.840E-02	1.569E-01
Left precentral gyrus	-0.324	0.098	[-0.516 - -0.132]	9.398E-04	5.133E-03
Right precentral gyrus	-0.345	0.102	[-0.545 - -0.144]	7.577E-04	4.483E-03
Left parahippocampal gyrus	-0.150	0.124	[-0.392 - 0.092]	2.242E-01	3.078E-01
Right parahippocampal gyrus	0.005	0.113	[-0.217 - 0.226]	9.668E-01	9.838E-01
Left inferior parietal cortex	-0.230	0.118	[-0.461 - 0.001]	5.137E-02	1.042E-01
Right inferior parietal cortex	-0.214	0.140	[-0.488 - 0.060]	1.257E-01	2.029E-01
Left transverse temporal gyrus	-0.154	0.097	[-0.345 - 0.037]	1.135E-01	1.875E-01
Right transverse temporal gyrus	-0.171	0.097	[-0.362 - 0.020]	7.915E-02	1.441E-01
Left postcentral gyrus	-0.104	0.128	[-0.354 - 0.146]	4.140E-01	5.068E-01
Right postcentral gyrus	-0.143	0.144	[-0.425 - 0.139]	3.194E-01	4.123E-01
Left precuneus	-0.098	0.142	[-0.377 - 0.181]	4.909E-01	5.809E-01
Right precuneus	-0.071	0.116	[-0.297 - 0.156]	5.394E-01	6.278E-01
Left caudal anterior cingulate cortex	-0.043	0.097	[-0.233 - 0.148]	6.613E-01	7.224E-01
Right caudal anterior cingulate cortex	-0.097	0.129	[-0.350 - 0.156]	4.503E-01	5.419E-01
Left cuneus	-0.107	0.097	[-0.298 - 0.084]	2.714E-01	3.636E-01
Right cuneus	-0.052	0.097	[-0.243 - 0.138]	5.910E-01	6.768E-01
Left rostral anterior cingulate cortex	-0.174	0.103	[-0.376 - 0.028]	9.058E-02	1.569E-01
Right rostral anterior cingulate cortex	-0.051	0.114	[-0.274 - 0.173]	6.561E-01	7.224E-01
Left pericalcarine cortex	-0.012	0.097	[-0.203 - 0.178]	8.996E-01	9.393E-01
Right pericalcarine cortex	0.016	0.097	[-0.174 - 0.207]	8.652E-01	9.169E-01
Left paracentral lobule	-0.162	0.111	[-0.380 - 0.055]	1.437E-01	2.220E-01
Right paracentral lobule	-0.113	0.129	[-0.365 - 0.139]	3.805E-01	4.740E-01
Left superior parietal cortex	-0.044	0.143	[-0.324 - 0.237]	7.595E-01	8.171E-01
Right superior parietal cortex	-0.074	0.151	[-0.370 - 0.223]	6.257E-01	7.052E-01
Left temporal pole	-0.180	0.097	[-0.371 - 0.011]	6.500E-02	1.282E-01
Right temporal pole	0.118	0.097	[-0.072 - 0.309]	2.240E-01	3.078E-01
Left entorhinal cortex	-0.002	0.126	[-0.249 - 0.245]	9.871E-01	9.871E-01
Right entorhinal cortex	-0.006	0.156	[-0.312 - 0.300]	9.700E-01	9.838E-01

Supplementary Table S93. Cortical thickness differences between individuals with first episode major depressive disorder versus healthy comparison subjects controlling for age and sex over 21 years of old

	d	Std. Err.	95% CI	*p*-value	FDR *q*-value
Global mean cortical thickness	-0.386	0.107	[-0.596 - -0.176]	3.126E-04	2.774E-03
Left hemisphere	-0.400	0.100	[-0.596 - -0.203]	6.770E-05	1.265E-03
Right hemisphere	-0.358	0.116	[-0.586 - -0.130]	2.052E-03	9.803E-03
Left fusiform gyrus	-0.355	0.102	[-0.555 - -0.155]	4.927E-04	3.790E-03
Right fusiform gyrus	-0.333	0.126	[-0.581 - -0.085]	8.383E-03	2.705E-02
Left pars opercularis of inferior frontal gyrus	-0.398	0.100	[-0.594 - -0.201]	7.125E-05	1.265E-03
Right pars opercularis of inferior frontal gyrus	-0.287	0.104	[-0.490 - -0.084]	5.564E-03	1.975E-02
Left superior temporal gyrus	-0.347	0.100	[-0.543 - -0.151]	5.338E-04	3.790E-03
Right superior temporal gyrus	-0.135	0.125	[-0.379 - 0.109]	2.790E-01	3.537E-01
Left insula	-0.304	0.111	[-0.521 - -0.087]	5.954E-03	2.013E-02
Right insula	-0.214	0.141	[-0.491 - 0.062]	1.288E-01	1.946E-01
Left lingual gyrus	-0.254	0.100	[-0.450 - -0.059]	1.084E-02	2.960E-02
Right lingual gyrus	-0.141	0.109	[-0.355 - 0.072]	1.951E-01	2.664E-01
Left pars triangularis of inferior frontal gyrus	-0.197	0.100	[-0.392 - -0.002]	4.814E-02	9.494E-02
Right pars triangularis of inferior frontal gyrus	-0.364	0.100	[-0.560 - -0.168]	2.773E-04	2.774E-03
Left lateral orbitofrontal cortex	-0.457	0.108	[-0.668 - -0.245]	2.367E-05	8.403E-04
Right lateral orbitofrontal cortex	-0.318	0.139	[-0.590 - -0.046]	2.211E-02	4.906E-02
Left rostral middle frontal gyrus	-0.381	0.124	[-0.623 - -0.138]	2.071E-03	9.803E-03
Right rostral middle frontal gyrus	-0.313	0.148	[-0.602 - -0.023]	3.446E-02	7.196E-02
Left middle temporal gyrus	-0.433	0.100	[-0.630 - -0.237]	1.556E-05	8.403E-04
Right middle temporal gyrus	-0.291	0.142	[-0.568 - -0.013]	4.026E-02	8.167E-02
Left superior frontal gyrus	-0.385	0.100	[-0.581 - -0.188]	1.232E-04	1.750E-03
Right superior frontal gyrus	-0.336	0.100	[-0.532 - -0.140]	7.684E-04	4.960E-03
Left pars orbitalis of inferior frontal gyrus	-0.335	0.149	[-0.627 - -0.043]	2.473E-02	5.321E-02
Right pars orbitalis of inferior frontal gyrus	-0.347	0.135	[-0.611 - -0.083]	9.899E-03	2.929E-02
Left medial orbitofrontal cortex	-0.320	0.139	[-0.593 - -0.046]	2.187E-02	4.906E-02
Right medial orbitofrontal cortex	-0.367	0.115	[-0.593 - -0.140]	1.493E-03	8.154E-03
Left inferior temporal gyrus	-0.293	0.100	[-0.489 - -0.097]	3.339E-03	1.317E-02
Right inferior temporal gyrus	-0.249	0.100	[-0.445 - -0.054]	1.252E-02	3.065E-02
Left isthmus cingulate cortex	-0.209	0.118	[-0.442 - 0.023]	7.712E-02	1.404E-01
Right isthmus cingulate cortex	-0.284	0.100	[-0.480 - -0.089]	4.383E-03	1.638E-02
Left banks of superior temporal sulcus	-0.246	0.144	[-0.528 - 0.036]	8.758E-02	1.555E-01
Right banks of superior temporal sulcus	-0.183	0.148	[-0.473 - 0.107]	2.159E-01	2.839E-01
Left supramarginal gyrus	-0.145	0.100	[-0.341 - 0.050]	1.444E-01	2.092E-01
Right supramarginal gyrus	-0.300	0.100	[-0.496 - -0.105]	2.627E-03	1.097E-02
Left caudal middle frontal gyrus	-0.250	0.100	[-0.446 - -0.055]	1.210E-02	3.065E-02
Right caudal middle frontal gyrus	-0.365	0.100	[-0.561 - -0.169]	2.636E-04	2.774E-03
Left frontal pole	-0.272	0.107	[-0.482 - -0.061]	1.141E-02	3.001E-02
Right frontal pole	-0.458	0.179	[-0.809 - -0.108]	1.038E-02	2.948E-02
Left posterior cingulate cortex	-0.146	0.100	[-0.341 - 0.049]	1.421E-01	2.092E-01
Right posterior cingulate cortex	-0.259	0.100	[-0.454 - -0.064]	9.360E-03	2.889E-02
Left lateral occipital cortex	-0.243	0.100	[-0.439 - -0.048]	1.479E-02	3.500E-02
Right lateral occipital cortex	-0.215	0.132	[-0.473 - 0.043]	1.022E-01	1.649E-01
Left precentral gyrus	-0.332	0.100	[-0.528 - -0.136]	9.068E-04	5.365E-03
Right precentral gyrus	-0.349	0.115	[-0.574 - -0.124]	2.359E-03	1.047E-02
Left parahippocampal gyrus	-0.137	0.122	[-0.376 - 0.101]	2.586E-01	3.339E-01
Right parahippocampal gyrus	0.024	0.118	[-0.208 - 0.255]	8.407E-01	8.728E-01
Left inferior parietal cortex	-0.226	0.137	[-0.495 - 0.043]	9.972E-02	1.647E-01
Right inferior parietal cortex	-0.249	0.129	[-0.503 - 0.004]	5.378E-02	1.032E-01
Left transverse temporal gyrus	-0.168	0.100	[-0.363 - 0.027]	9.206E-02	1.594E-01
Right transverse temporal gyrus	-0.159	0.100	[-0.354 - 0.036]	1.102E-01	1.738E-01
Left postcentral gyrus	-0.128	0.133	[-0.388 - 0.133]	3.360E-01	4.186E-01
Right postcentral gyrus	-0.199	0.140	[-0.473 - 0.075]	1.555E-01	2.209E-01
Left precuneus	-0.105	0.151	[-0.402 - 0.192]	4.879E-01	5.679E-01
Right precuneus	-0.073	0.122	[-0.313 - 0.166]	5.484E-01	6.273E-01
Left caudal anterior cingulate cortex	-0.039	0.099	[-0.234 - 0.156]	6.953E-01	7.595E-01
Right caudal anterior cingulate cortex	-0.154	0.100	[-0.349 - 0.041]	1.222E-01	1.886E-01
Left cuneus	-0.077	0.099	[-0.272 - 0.118]	4.401E-01	5.296E-01
Right cuneus	-0.037	0.099	[-0.232 - 0.158]	7.115E-01	7.654E-01
Left rostral anterior cingulate cortex	-0.251	0.132	[-0.509 - 0.007]	5.645E-02	1.055E-01
Right rostral anterior cingulate cortex	-0.076	0.110	[-0.291 - 0.139]	4.878E-01	5.679E-01
Left pericalcarine cortex	0.008	0.099	[-0.187 - 0.203]	9.365E-01	9.499E-01
Right pericalcarine cortex	0.058	0.099	[-0.136 - 0.253]	5.566E-01	6.273E-01
Left paracentral lobule	-0.162	0.121	[-0.400 - 0.076]	1.817E-01	2.530E-01
Right paracentral lobule	-0.109	0.137	[-0.377 - 0.159]	4.263E-01	5.219E-01
Left superior parietal cortex	-0.047	0.151	[-0.343 - 0.250]	7.583E-01	8.036E-01
Right superior parietal cortex	-0.090	0.156	[-0.396 - 0.217]	5.664E-01	6.284E-01
Left temporal pole	-0.164	0.100	[-0.359 - 0.031]	9.965E-02	1.647E-01
Right temporal pole	0.124	0.100	[-0.072 - 0.319]	2.145E-01	2.839E-01
Left entorhinal cortex	0.026	0.137	[-0.242 - 0.294]	8.483E-01	8.728E-01
Right entorhinal cortex	-0.006	0.155	[-0.310 - 0.298]	9.707E-01	9.707E-01

Supplementary Table S94. Cortical thickness differences between individuals with recurrent episode major depressive disorder versus healthy comparison subjects controlling for age and sex

	d	Std. Err.	95% CI	*p*-value	FDR *q*-value
Global mean cortical thickness	-0.325	0.150	[-0.620 - -0.031]	3.044E-02	9.274E-02
Left hemisphere	-0.352	0.155	[-0.655 - -0.048]	2.315E-02	8.826E-02
Right hemisphere	-0.286	0.143	[-0.567 - -0.005]	4.598E-02	1.126E-01
Left fusiform gyrus	-0.271	0.111	[-0.488 - -0.055]	1.417E-02	7.741E-02
Right fusiform gyrus	-0.203	0.102	[-0.403 - -0.004]	4.591E-02	1.126E-01
Left pars opercularis of inferior frontal gyrus	-0.312	0.080	[-0.469 - -0.155]	9.770E-05	3.468E-03
Right pars opercularis of inferior frontal gyrus	-0.203	0.147	[-0.491 - 0.084]	1.656E-01	2.380E-01
Left superior temporal gyrus	-0.339	0.082	[-0.500 - -0.177]	4.028E-05	2.860E-03
Right superior temporal gyrus	-0.299	0.116	[-0.526 - -0.071]	1.018E-02	6.456E-02
Left insula	-0.220	0.102	[-0.421 - -0.020]	3.135E-02	9.274E-02
Right insula	-0.240	0.128	[-0.490 - 0.010]	5.993E-02	1.289E-01
Left lingual gyrus	-0.210	0.087	[-0.381 - -0.040]	1.572E-02	7.973E-02
Right lingual gyrus	-0.145	0.130	[-0.399 - 0.109]	2.632E-01	3.340E-01
Left pars triangularis of inferior frontal gyrus	-0.215	0.096	[-0.403 - -0.027]	2.500E-02	8.875E-02
Right pars triangularis of inferior frontal gyrus	-0.273	0.152	[-0.572 - 0.026]	7.309E-02	1.468E-01
Left lateral orbitofrontal cortex	-0.343	0.126	[-0.589 - -0.097]	6.344E-03	6.388E-02
Right lateral orbitofrontal cortex	-0.280	0.107	[-0.490 - -0.070]	8.820E-03	6.388E-02
Left rostral middle frontal gyrus	-0.370	0.163	[-0.691 - -0.050]	2.342E-02	8.826E-02
Right rostral middle frontal gyrus	-0.201	0.146	[-0.487 - 0.085]	1.675E-01	2.380E-01
Left middle temporal gyrus	-0.335	0.123	[-0.576 - -0.095]	6.223E-03	6.388E-02
Right middle temporal gyrus	-0.359	0.135	[-0.624 - -0.094]	7.837E-03	6.388E-02
Left superior frontal gyrus	-0.228	0.144	[-0.511 - 0.055]	1.149E-01	1.942E-01
Right superior frontal gyrus	-0.220	0.114	[-0.443 - 0.003]	5.276E-02	1.171E-01
Left pars orbitalis of inferior frontal gyrus	-0.276	0.154	[-0.578 - 0.027]	7.444E-02	1.468E-01
Right pars orbitalis of inferior frontal gyrus	-0.305	0.117	[-0.533 - -0.076]	8.997E-03	6.388E-02
Left medial orbitofrontal cortex	-0.345	0.152	[-0.643 - -0.046]	2.362E-02	8.826E-02
Right medial orbitofrontal cortex	-0.170	0.136	[-0.438 - 0.097]	2.114E-01	2.832E-01
Left inferior temporal gyrus	-0.138	0.124	[-0.380 - 0.104]	2.635E-01	3.340E-01
Right inferior temporal gyrus	-0.183	0.085	[-0.349 - -0.017]	3.039E-02	9.274E-02
Left isthmus cingulate cortex	-0.139	0.093	[-0.321 - 0.043]	1.340E-01	2.037E-01
Right isthmus cingulate cortex	-0.128	0.086	[-0.296 - 0.040]	1.349E-01	2.037E-01
Left banks of superior temporal sulcus	-0.196	0.093	[-0.379 - -0.014]	3.528E-02	9.797E-02
Right banks of superior temporal sulcus	-0.237	0.088	[-0.409 - -0.065]	6.848E-03	6.388E-02
Left supramarginal gyrus	-0.290	0.107	[-0.499 - -0.081]	6.563E-03	6.388E-02
Right supramarginal gyrus	-0.210	0.122	[-0.449 - 0.029]	8.512E-02	1.590E-01
Left caudal middle frontal gyrus	-0.244	0.144	[-0.526 - 0.037]	8.912E-02	1.622E-01
Right caudal middle frontal gyrus	-0.279	0.118	[-0.511 - -0.047]	1.829E-02	8.115E-02
Left frontal pole	-0.155	0.093	[-0.338 - 0.028]	9.783E-02	1.694E-01
Right frontal pole	-0.234	0.141	[-0.511 - 0.042]	9.697E-02	1.694E-01
Left posterior cingulate cortex	-0.285	0.079	[-0.441 - -0.129]	3.335E-04	7.894E-03
Right posterior cingulate cortex	-0.135	0.099	[-0.329 - 0.058]	1.710E-01	2.380E-01
Left lateral occipital cortex	-0.143	0.116	[-0.370 - 0.085]	2.180E-01	2.867E-01
Right lateral occipital cortex	-0.118	0.111	[-0.335 - 0.099]	2.874E-01	3.580E-01
Left precentral gyrus	-0.257	0.129	[-0.510 - -0.005]	4.585E-02	1.126E-01
Right precentral gyrus	-0.230	0.090	[-0.407 - -0.053]	1.091E-02	6.456E-02
Left parahippocampal gyrus	-0.120	0.138	[-0.390 - 0.150]	3.839E-01	4.407E-01
Right parahippocampal gyrus	-0.031	0.119	[-0.263 - 0.202]	7.968E-01	8.199E-01
Left inferior parietal cortex	-0.159	0.105	[-0.364 - 0.046]	1.277E-01	2.014E-01
Right inferior parietal cortex	-0.202	0.117	[-0.431 - 0.027]	8.446E-02	1.590E-01
Left transverse temporal gyrus	-0.233	0.106	[-0.441 - -0.025]	2.800E-02	9.274E-02
Right transverse temporal gyrus	-0.171	0.082	[-0.331 - -0.011]	3.588E-02	9.797E-02
Left postcentral gyrus	-0.263	0.110	[-0.479 - -0.046]	1.731E-02	8.115E-02
Right postcentral gyrus	-0.239	0.122	[-0.477 - 0.000]	4.970E-02	1.138E-01
Left precuneus	-0.115	0.120	[-0.351 - 0.120]	3.368E-01	4.123E-01
Right precuneus	-0.156	0.079	[-0.311 - 0.000]	4.941E-02	1.138E-01
Left caudal anterior cingulate cortex	-0.142	0.079	[-0.297 - 0.013]	7.235E-02	1.468E-01
Right caudal anterior cingulate cortex	-0.088	0.101	[-0.286 - 0.110]	3.849E-01	4.407E-01
Left cuneus	-0.091	0.098	[-0.284 - 0.102]	3.546E-01	4.267E-01
Right cuneus	-0.123	0.091	[-0.301 - 0.055]	1.743E-01	2.380E-01
Left rostral anterior cingulate cortex	-0.144	0.105	[-0.350 - 0.062]	1.704E-01	2.380E-01
Right rostral anterior cingulate cortex	0.011	0.113	[-0.211 - 0.234]	9.202E-01	9.202E-01
Left pericalcarine cortex	-0.080	0.089	[-0.255 - 0.095]	3.689E-01	4.366E-01
Right pericalcarine cortex	-0.045	0.109	[-0.259 - 0.169]	6.806E-01	7.212E-01
Left paracentral lobule	-0.148	0.097	[-0.337 - 0.041]	1.248E-01	2.014E-01
Right paracentral lobule	-0.165	0.107	[-0.374 - 0.044]	1.222E-01	2.014E-01
Left superior parietal cortex	-0.110	0.135	[-0.375 - 0.154]	4.139E-01	4.665E-01
Right superior parietal cortex	-0.070	0.094	[-0.254 - 0.113]	4.531E-01	5.026E-01
Left temporal pole	-0.012	0.117	[-0.242 - 0.217]	9.165E-01	9.202E-01
Right temporal pole	0.036	0.131	[-0.221 - 0.293]	7.833E-01	8.179E-01
Left entorhinal cortex	-0.082	0.134	[-0.346 - 0.181]	5.408E-01	5.907E-01
Right entorhinal cortex	-0.081	0.167	[-0.409 - 0.247]	6.281E-01	6.756E-01

Supplementary Table S95. Cortical thickness differences between individuals with recurrent episode major depressive disorder versus healthy comparison subjects controlling for age and sex over 21 years of old

	d	Std. Err.	95% CI	*p*-value	FDR *q*-value
Global mean cortical thickness	-0.315	0.153	[-0.616 - -0.015]	3.936E-02	1.217E-01
Left hemisphere	-0.344	0.158	[-0.655 - -0.034]	2.972E-02	1.217E-01
Right hemisphere	-0.274	0.145	[-0.559 - 0.011]	5.912E-02	1.399E-01
Left fusiform gyrus	-0.264	0.110	[-0.480 - -0.048]	1.637E-02	8.894E-02
Right fusiform gyrus	-0.197	0.102	[-0.396 - 0.002]	5.243E-02	1.379E-01
Left pars opercularis of inferior frontal gyrus	-0.307	0.083	[-0.470 - -0.144]	2.167E-04	7.693E-03
Right pars opercularis of inferior frontal gyrus	-0.209	0.146	[-0.494 - 0.077]	1.515E-01	2.352E-01
Left superior temporal gyrus	-0.323	0.087	[-0.495 - -0.152]	2.129E-04	7.693E-03
Right superior temporal gyrus	-0.285	0.116	[-0.514 - -0.057]	1.431E-02	8.894E-02
Left insula	-0.220	0.106	[-0.427 - -0.013]	3.760E-02	1.217E-01
Right insula	-0.225	0.129	[-0.478 - 0.029]	8.275E-02	1.726E-01
Left lingual gyrus	-0.196	0.093	[-0.378 - -0.014]	3.518E-02	1.217E-01
Right lingual gyrus	-0.130	0.132	[-0.390 - 0.129]	3.254E-01	4.054E-01
Left pars triangularis of inferior frontal gyrus	-0.195	0.103	[-0.396 - 0.007]	5.791E-02	1.399E-01
Right pars triangularis of inferior frontal gyrus	-0.254	0.156	[-0.559 - 0.051]	1.026E-01	1.822E-01
Left lateral orbitofrontal cortex	-0.319	0.133	[-0.580 - -0.058]	1.647E-02	8.894E-02
Right lateral orbitofrontal cortex	-0.270	0.110	[-0.486 - -0.054]	1.437E-02	8.894E-02
Left rostral middle frontal gyrus	-0.366	0.172	[-0.702 - -0.030]	3.293E-02	1.217E-01
Right rostral middle frontal gyrus	-0.196	0.153	[-0.496 - 0.104]	1.997E-01	2.878E-01
Left middle temporal gyrus	-0.340	0.126	[-0.588 - -0.093]	7.012E-03	8.894E-02
Right middle temporal gyrus	-0.352	0.140	[-0.627 - -0.078]	1.189E-02	8.894E-02
Left superior frontal gyrus	-0.218	0.152	[-0.517 - 0.081]	1.524E-01	2.352E-01
Right superior frontal gyrus	-0.207	0.120	[-0.443 - 0.029]	8.536E-02	1.726E-01
Left pars orbitalis of inferior frontal gyrus	-0.262	0.155	[-0.565 - 0.042]	9.134E-02	1.726E-01
Right pars orbitalis of inferior frontal gyrus	-0.296	0.125	[-0.540 - -0.052]	1.754E-02	8.894E-02
Left medial orbitofrontal cortex	-0.329	0.161	[-0.643 - -0.014]	4.073E-02	1.217E-01
Right medial orbitofrontal cortex	-0.154	0.144	[-0.437 - 0.129]	2.852E-01	3.702E-01
Left inferior temporal gyrus	-0.143	0.126	[-0.390 - 0.104]	2.556E-01	3.424E-01
Right inferior temporal gyrus	-0.171	0.087	[-0.342 - 0.000]	4.939E-02	1.349E-01
Left isthmus cingulate cortex	-0.123	0.097	[-0.313 - 0.066]	2.027E-01	2.878E-01
Right isthmus cingulate cortex	-0.109	0.090	[-0.285 - 0.066]	2.225E-01	3.038E-01
Left banks of superior temporal sulcus	-0.185	0.097	[-0.376 - 0.006]	5.786E-02	1.399E-01
Right banks of superior temporal sulcus	-0.223	0.089	[-0.397 - -0.048]	1.225E-02	8.894E-02
Left supramarginal gyrus	-0.296	0.111	[-0.514 - -0.078]	7.907E-03	8.894E-02
Right supramarginal gyrus	-0.207	0.118	[-0.438 - 0.024]	7.917E-02	1.721E-01
Left caudal middle frontal gyrus	-0.246	0.147	[-0.534 - 0.041]	9.307E-02	1.726E-01
Right caudal middle frontal gyrus	-0.286	0.119	[-0.519 - -0.053]	1.615E-02	8.894E-02
Left frontal pole	-0.159	0.098	[-0.352 - 0.034]	1.064E-01	1.825E-01
Right frontal pole	-0.242	0.145	[-0.526 - 0.042]	9.481E-02	1.726E-01
Left posterior cingulate cortex	-0.269	0.080	[-0.426 - -0.112]	7.928E-04	1.876E-02
Right posterior cingulate cortex	-0.135	0.102	[-0.335 - 0.065]	1.859E-01	2.750E-01
Left lateral occipital cortex	-0.143	0.115	[-0.368 - 0.082]	2.139E-01	2.977E-01
Right lateral occipital cortex	-0.110	0.107	[-0.320 - 0.101]	3.064E-01	3.885E-01
Left precentral gyrus	-0.263	0.129	[-0.515 - -0.011]	4.113E-02	1.217E-01
Right precentral gyrus	-0.231	0.089	[-0.405 - -0.056]	9.439E-03	8.894E-02
Left parahippocampal gyrus	-0.113	0.137	[-0.380 - 0.155]	4.095E-01	4.690E-01
Right parahippocampal gyrus	-0.028	0.117	[-0.259 - 0.202]	8.090E-01	8.324E-01
Left inferior parietal cortex	-0.160	0.100	[-0.356 - 0.035]	1.080E-01	1.825E-01
Right inferior parietal cortex	-0.193	0.114	[-0.416 - 0.030]	8.960E-02	1.726E-01
Left transverse temporal gyrus	-0.222	0.107	[-0.432 - -0.012]	3.844E-02	1.217E-01
Right transverse temporal gyrus	-0.174	0.083	[-0.337 - -0.011]	3.644E-02	1.217E-01
Left postcentral gyrus	-0.257	0.115	[-0.483 - -0.032]	2.506E-02	1.186E-01
Right postcentral gyrus	-0.238	0.120	[-0.474 - -0.002]	4.804E-02	1.349E-01
Left precuneus	-0.110	0.119	[-0.344 - 0.124]	3.567E-01	4.221E-01
Right precuneus	-0.140	0.080	[-0.297 - 0.017]	8.000E-02	1.721E-01
Left caudal anterior cingulate cortex	-0.148	0.080	[-0.305 - 0.009]	6.399E-02	1.466E-01
Right caudal anterior cingulate cortex	-0.099	0.105	[-0.305 - 0.108]	3.485E-01	4.221E-01
Left cuneus	-0.091	0.102	[-0.291 - 0.109]	3.743E-01	4.356E-01
Right cuneus	-0.102	0.096	[-0.291 - 0.086]	2.867E-01	3.702E-01
Left rostral anterior cingulate cortex	-0.157	0.110	[-0.372 - 0.057]	1.508E-01	2.352E-01
Right rostral anterior cingulate cortex	0.004	0.114	[-0.220 - 0.229]	9.694E-01	9.694E-01
Left pericalcarine cortex	-0.083	0.090	[-0.258 - 0.093]	3.560E-01	4.221E-01
Right pericalcarine cortex	-0.039	0.104	[-0.243 - 0.164]	7.053E-01	7.474E-01
Left paracentral lobule	-0.131	0.093	[-0.313 - 0.051]	1.581E-01	2.388E-01
Right paracentral lobule	-0.157	0.106	[-0.365 - 0.050]	1.379E-01	2.277E-01
Left superior parietal cortex	-0.107	0.134	[-0.370 - 0.156]	4.270E-01	4.812E-01
Right superior parietal cortex	-0.064	0.094	[-0.249 - 0.120]	4.954E-01	5.495E-01
Left temporal pole	-0.019	0.122	[-0.257 - 0.220]	8.775E-01	8.900E-01
Right temporal pole	0.034	0.130	[-0.221 - 0.289]	7.938E-01	8.288E-01
Left entorhinal cortex	-0.090	0.136	[-0.358 - 0.177]	5.070E-01	5.538E-01
Right entorhinal cortex	-0.087	0.167	[-0.414 - 0.241]	6.044E-01	6.502E-01

Supplementary Table S96. Cortical thickness differences between individuals with first episode major depressive disorder versus individuals with recurrent episode major depressive disorder controlling for age and sex

	d	Std. Err.	95% CI	*p*-value	FDR *q*-value
Global mean cortical thickness	-0.103	0.107	[-0.312 - 0.106]	3.327E-01	8.384E-01
Left hemisphere	-0.116	0.118	[-0.347 - 0.115]	3.243E-01	8.384E-01
Right hemisphere	-0.078	0.092	[-0.259 - 0.103]	3.978E-01	8.384E-01
Left fusiform gyrus	-0.065	0.092	[-0.246 - 0.116]	4.818E-01	8.384E-01
Right fusiform gyrus	-0.114	0.092	[-0.294 - 0.067]	2.185E-01	8.384E-01
Left pars opercularis of inferior frontal gyrus	-0.110	0.131	[-0.366 - 0.146]	4.014E-01	8.384E-01
Right pars opercularis of inferior frontal gyrus	-0.096	0.152	[-0.394 - 0.202]	5.293E-01	8.384E-01
Left superior temporal gyrus	-0.009	0.092	[-0.190 - 0.172]	9.240E-01	9.562E-01
Right superior temporal gyrus	0.096	0.095	[-0.089 - 0.282]	3.083E-01	8.384E-01
Left insula	-0.075	0.092	[-0.256 - 0.106]	4.166E-01	8.384E-01
Right insula	0.119	0.092	[-0.062 - 0.300]	1.974E-01	8.384E-01
Left lingual gyrus	-0.087	0.092	[-0.268 - 0.093]	3.437E-01	8.384E-01
Right lingual gyrus	-0.085	0.092	[-0.266 - 0.096]	3.573E-01	8.384E-01
Left pars triangularis of inferior frontal gyrus	0.052	0.106	[-0.156 - 0.260]	6.221E-01	8.580E-01
Right pars triangularis of inferior frontal gyrus	-0.114	0.120	[-0.350 - 0.122]	3.429E-01	8.384E-01
Left lateral orbitofrontal cortex	-0.102	0.140	[-0.377 - 0.173]	4.681E-01	8.384E-01
Right lateral orbitofrontal cortex	0.051	0.092	[-0.130 - 0.232]	5.786E-01	8.384E-01
Left rostral middle frontal gyrus	-0.092	0.097	[-0.283 - 0.099]	3.467E-01	8.384E-01
Right rostral middle frontal gyrus	-0.107	0.092	[-0.288 - 0.074]	2.453E-01	8.384E-01
Left middle temporal gyrus	-0.098	0.092	[-0.279 - 0.083]	2.872E-01	8.384E-01
Right middle temporal gyrus	0.062	0.092	[-0.119 - 0.243]	5.014E-01	8.384E-01
Left superior frontal gyrus	-0.223	0.121	[-0.461 - 0.015]	6.679E-02	8.384E-01
Right superior frontal gyrus	-0.109	0.099	[-0.303 - 0.084]	2.685E-01	8.384E-01
Left pars orbitalis of inferior frontal gyrus	-0.021	0.112	[-0.241 - 0.198]	8.491E-01	9.286E-01
Right pars orbitalis of inferior frontal gyrus	-0.062	0.092	[-0.243 - 0.119]	4.992E-01	8.384E-01
Left medial orbitofrontal cortex	0.042	0.092	[-0.139 - 0.222]	6.526E-01	8.580E-01
Right medial orbitofrontal cortex	-0.152	0.124	[-0.395 - 0.091]	2.211E-01	8.384E-01
Left inferior temporal gyrus	-0.157	0.124	[-0.399 - 0.086]	2.051E-01	8.384E-01
Right inferior temporal gyrus	-0.017	0.092	[-0.198 - 0.163]	8.501E-01	9.286E-01
Left isthmus cingulate cortex	-0.047	0.092	[-0.228 - 0.134]	6.096E-01	8.580E-01
Right isthmus cingulate cortex	-0.094	0.092	[-0.275 - 0.087]	3.088E-01	8.384E-01
Left banks of superior temporal sulcus	0.010	0.142	[-0.268 - 0.288]	9.427E-01	9.562E-01
Right banks of superior temporal sulcus	0.054	0.092	[-0.127 - 0.235]	5.580E-01	8.384E-01
Left supramarginal gyrus	0.115	0.096	[-0.073 - 0.302]	2.298E-01	8.384E-01
Right supramarginal gyrus	-0.053	0.132	[-0.312 - 0.206]	6.892E-01	8.738E-01
Left caudal middle frontal gyrus	-0.059	0.106	[-0.267 - 0.148]	5.769E-01	8.384E-01
Right caudal middle frontal gyrus	-0.063	0.095	[-0.250 - 0.123]	5.053E-01	8.384E-01
Left frontal pole	-0.065	0.092	[-0.245 - 0.116]	4.827E-01	8.384E-01
Right frontal pole	-0.026	0.092	[-0.207 - 0.155]	7.794E-01	9.043E-01
Left posterior cingulate cortex	0.033	0.092	[-0.148 - 0.214]	7.218E-01	8.836E-01
Right posterior cingulate cortex	-0.110	0.092	[-0.291 - 0.070]	2.310E-01	8.384E-01
Left lateral occipital cortex	-0.114	0.092	[-0.295 - 0.067]	2.185E-01	8.384E-01
Right lateral occipital cortex	-0.128	0.092	[-0.309 - 0.052]	1.640E-01	8.384E-01
Left precentral gyrus	-0.096	0.150	[-0.390 - 0.198]	5.229E-01	8.384E-01
Right precentral gyrus	-0.095	0.104	[-0.298 - 0.108]	3.600E-01	8.384E-01
Left parahippocampal gyrus	-0.074	0.092	[-0.254 - 0.107]	4.225E-01	8.384E-01
Right parahippocampal gyrus	-0.001	0.092	[-0.182 - 0.180]	9.909E-01	9.909E-01
Left inferior parietal cortex	-0.067	0.146	[-0.353 - 0.219]	6.472E-01	8.580E-01
Right inferior parietal cortex	-0.040	0.092	[-0.220 - 0.141]	6.672E-01	8.613E-01
Left transverse temporal gyrus	0.079	0.105	[-0.127 - 0.286]	4.503E-01	8.384E-01
Right transverse temporal gyrus	0.019	0.092	[-0.161 - 0.200]	8.347E-01	9.286E-01
Left postcentral gyrus	0.042	0.092	[-0.139 - 0.223]	6.493E-01	8.580E-01
Right postcentral gyrus	-0.034	0.110	[-0.249 - 0.181]	7.546E-01	8.929E-01
Left precuneus	-0.093	0.092	[-0.274 - 0.087]	3.117E-01	8.384E-01
Right precuneus	-0.025	0.092	[-0.205 - 0.156]	7.897E-01	9.043E-01
Left caudal anterior cingulate cortex	0.057	0.092	[-0.123 - 0.238]	5.331E-01	8.384E-01
Right caudal anterior cingulate cortex	-0.012	0.128	[-0.263 - 0.240]	9.277E-01	9.562E-01
Left cuneus	-0.071	0.092	[-0.252 - 0.109]	4.394E-01	8.384E-01
Right cuneus	0.011	0.092	[-0.169 - 0.192]	9.033E-01	9.562E-01
Left rostral anterior cingulate cortex	-0.099	0.124	[-0.342 - 0.143]	4.224E-01	8.384E-01
Right rostral anterior cingulate cortex	-0.060	0.092	[-0.240 - 0.120]	5.149E-01	8.384E-01
Left pericalcarine cortex	0.052	0.092	[-0.129 - 0.233]	5.713E-01	8.384E-01
Right pericalcarine cortex	0.053	0.092	[-0.128 - 0.234]	5.675E-01	8.384E-01
Left paracentral lobule	-0.045	0.124	[-0.289 - 0.198]	7.148E-01	8.836E-01
Right paracentral lobule	-0.007	0.092	[-0.187 - 0.174]	9.427E-01	9.562E-01
Left superior parietal cortex	-0.048	0.151	[-0.344 - 0.248]	7.518E-01	8.929E-01
Right superior parietal cortex	-0.106	0.152	[-0.404 - 0.191]	4.844E-01	8.384E-01
Left temporal pole	-0.116	0.106	[-0.324 - 0.093]	2.759E-01	8.384E-01
Right temporal pole	0.131	0.092	[-0.050 - 0.312]	1.545E-01	8.384E-01
Left entorhinal cortex	0.116	0.107	[-0.094 - 0.327]	2.792E-01	8.384E-01
Right entorhinal cortex	0.198	0.110	[-0.018 - 0.415]	7.246E-02	8.384E-01

Supplementary Table S97. Cortical thickness differences between individuals with first episode major depressive disorder versus individuals with recurrent episode major depressive disorder controlling for age and sex over 21 years of old

	d	Std. Err.	95% CI	*p*-value	FDR *q*-value
Global mean cortical thickness	-0.131	0.100	[-0.328 - 0.065]	1.901E-01	7.502E-01
Left hemisphere	-0.144	0.113	[-0.364 - 0.077]	2.016E-01	7.502E-01
Right hemisphere	-0.117	0.094	[-0.302 - 0.067]	2.130E-01	7.502E-01
Left fusiform gyrus	-0.077	0.094	[-0.262 - 0.107]	4.120E-01	7.502E-01
Right fusiform gyrus	-0.115	0.094	[-0.299 - 0.070]	2.221E-01	7.502E-01
Left pars opercularis of inferior frontal gyrus	-0.140	0.130	[-0.394 - 0.115]	2.812E-01	7.502E-01
Right pars opercularis of inferior frontal gyrus	-0.123	0.151	[-0.419 - 0.173]	4.146E-01	7.502E-01
Left superior temporal gyrus	-0.044	0.094	[-0.228 - 0.141]	6.415E-01	8.134E-01
Right superior temporal gyrus	0.077	0.094	[-0.107 - 0.262]	4.118E-01	7.502E-01
Left insula	-0.089	0.094	[-0.273 - 0.096]	3.448E-01	7.502E-01
Right insula	0.103	0.094	[-0.082 - 0.288]	2.753E-01	7.502E-01
Left lingual gyrus	-0.087	0.094	[-0.271 - 0.098]	3.570E-01	7.502E-01
Right lingual gyrus	-0.081	0.094	[-0.265 - 0.104]	3.917E-01	7.502E-01
Left pars triangularis of inferior frontal gyrus	0.008	0.111	[-0.210 - 0.226]	9.417E-01	9.551E-01
Right pars triangularis of inferior frontal gyrus	-0.154	0.119	[-0.389 - 0.080]	1.961E-01	7.502E-01
Left lateral orbitofrontal cortex	-0.138	0.137	[-0.407 - 0.131]	3.155E-01	7.502E-01
Right lateral orbitofrontal cortex	0.012	0.094	[-0.173 - 0.196]	9.008E-01	9.435E-01
Left rostral middle frontal gyrus	-0.109	0.094	[-0.293 - 0.076]	2.486E-01	7.502E-01
Right rostral middle frontal gyrus	-0.145	0.094	[-0.330 - 0.040]	1.234E-01	7.502E-01
Left middle temporal gyrus	-0.133	0.094	[-0.318 - 0.051]	1.575E-01	7.502E-01
Right middle temporal gyrus	0.031	0.094	[-0.154 - 0.215]	7.437E-01	8.517E-01
Left superior frontal gyrus	-0.247	0.116	[-0.475 - -0.019]	3.342E-02	7.502E-01
Right superior frontal gyrus	-0.152	0.103	[-0.355 - 0.050]	1.411E-01	7.502E-01
Left pars orbitalis of inferior frontal gyrus	-0.031	0.110	[-0.246 - 0.184]	7.782E-01	8.770E-01
Right pars orbitalis of inferior frontal gyrus	-0.077	0.094	[-0.261 - 0.108]	4.143E-01	7.502E-01
Left medial orbitofrontal cortex	0.016	0.094	[-0.168 - 0.201]	8.628E-01	9.281E-01
Right medial orbitofrontal cortex	-0.176	0.128	[-0.427 - 0.076]	1.705E-01	7.502E-01
Left inferior temporal gyrus	-0.153	0.127	[-0.403 - 0.096]	2.287E-01	7.502E-01
Right inferior temporal gyrus	-0.040	0.094	[-0.225 - 0.144]	6.688E-01	8.220E-01
Left isthmus cingulate cortex	-0.074	0.094	[-0.258 - 0.111]	4.332E-01	7.502E-01
Right isthmus cingulate cortex	-0.129	0.094	[-0.314 - 0.055]	1.701E-01	7.502E-01
Left banks of superior temporal sulcus	-0.031	0.137	[-0.300 - 0.238]	8.204E-01	9.101E-01
Right banks of superior temporal sulcus	0.038	0.094	[-0.146 - 0.223]	6.831E-01	8.220E-01
Left supramarginal gyrus	0.085	0.094	[-0.099 - 0.270]	3.645E-01	7.502E-01
Right supramarginal gyrus	-0.084	0.135	[-0.349 - 0.181]	5.335E-01	7.795E-01
Left caudal middle frontal gyrus	-0.082	0.103	[-0.283 - 0.120]	4.260E-01	7.502E-01
Right caudal middle frontal gyrus	-0.076	0.096	[-0.264 - 0.111]	4.246E-01	7.502E-01
Left frontal pole	-0.068	0.094	[-0.252 - 0.116]	4.702E-01	7.587E-01
Right frontal pole	-0.036	0.094	[-0.221 - 0.148]	6.990E-01	8.271E-01
Left posterior cingulate cortex	0.000	0.094	[-0.185 - 0.184]	9.964E-01	9.964E-01
Right posterior cingulate cortex	-0.153	0.094	[-0.337 - 0.031]	1.034E-01	7.502E-01
Left lateral occipital cortex	-0.129	0.094	[-0.314 - 0.055]	1.700E-01	7.502E-01
Right lateral occipital cortex	-0.144	0.094	[-0.329 - 0.040]	1.253E-01	7.502E-01
Left precentral gyrus	-0.117	0.158	[-0.427 - 0.194]	4.610E-01	7.587E-01
Right precentral gyrus	-0.111	0.109	[-0.324 - 0.102]	3.055E-01	7.502E-01
Left parahippocampal gyrus	-0.070	0.094	[-0.255 - 0.114]	4.530E-01	7.587E-01
Right parahippocampal gyrus	0.011	0.094	[-0.173 - 0.196]	9.036E-01	9.435E-01
Left inferior parietal cortex	-0.089	0.149	[-0.380 - 0.203]	5.505E-01	7.795E-01
Right inferior parietal cortex	-0.078	0.094	[-0.263 - 0.106]	4.047E-01	7.502E-01
Left transverse temporal gyrus	0.064	0.099	[-0.130 - 0.258]	5.183E-01	7.795E-01
Right transverse temporal gyrus	0.045	0.094	[-0.139 - 0.229]	6.345E-01	8.134E-01
Left postcentral gyrus	0.018	0.094	[-0.166 - 0.202]	8.467E-01	9.248E-01
Right postcentral gyrus	-0.061	0.098	[-0.253 - 0.131]	5.330E-01	7.795E-01
Left precuneus	-0.118	0.094	[-0.302 - 0.067]	2.111E-01	7.502E-01
Right precuneus	-0.045	0.094	[-0.229 - 0.139]	6.330E-01	8.134E-01
Left caudal anterior cingulate cortex	0.054	0.094	[-0.130 - 0.238]	5.666E-01	7.795E-01
Right caudal anterior cingulate cortex	-0.039	0.112	[-0.260 - 0.181]	7.255E-01	8.444E-01
Left cuneus	-0.053	0.094	[-0.237 - 0.131]	5.709E-01	7.795E-01
Right cuneus	0.007	0.094	[-0.177 - 0.192]	9.387E-01	9.551E-01
Left rostral anterior cingulate cortex	-0.136	0.131	[-0.392 - 0.121]	3.008E-01	7.502E-01
Right rostral anterior cingulate cortex	-0.066	0.094	[-0.250 - 0.119]	4.854E-01	7.658E-01
Left pericalcarine cortex	0.074	0.094	[-0.110 - 0.259]	4.302E-01	7.502E-01
Right pericalcarine cortex	0.080	0.094	[-0.105 - 0.264]	3.975E-01	7.502E-01
Left paracentral lobule	-0.078	0.133	[-0.337 - 0.182]	5.580E-01	7.795E-01
Right paracentral lobule	-0.039	0.094	[-0.224 - 0.145]	6.745E-01	8.220E-01
Left superior parietal cortex	-0.074	0.149	[-0.367 - 0.218]	6.193E-01	8.134E-01
Right superior parietal cortex	-0.136	0.150	[-0.430 - 0.158]	3.644E-01	7.502E-01
Left temporal pole	-0.124	0.115	[-0.350 - 0.101]	2.799E-01	7.502E-01
Right temporal pole	0.116	0.094	[-0.069 - 0.300]	2.200E-01	7.502E-01
Left entorhinal cortex	0.136	0.114	[-0.087 - 0.360]	2.315E-01	7.502E-01
Right entorhinal cortex	0.195	0.105	[-0.011 - 0.400]	6.293E-02	7.502E-01

Supplementary Table S98. Partial correlations between cortical thickness and number of episodes controlling for age and sex in individuals with major depressive disorder

	Partial *R*	Std. Err.	95% CI	*p*-value	FDR *q*-value
Global mean cortical thickness	0.026	0.071	[-0.113 - 0.164]	7.188E-01	9.306E-01
Left hemisphere	0.004	0.067	[-0.128 - 0.135]	9.553E-01	9.955E-01
Right hemisphere	0.035	0.083	[-0.128 - 0.198]	6.714E-01	9.296E-01
Left fusiform gyrus	0.028	0.067	[-0.103 - 0.160]	6.725E-01	9.296E-01
Right fusiform gyrus	0.125	0.067	[-0.006 - 0.257]	6.216E-02	8.814E-01
Left pars opercularis of inferior frontal gyrus	-0.061	0.067	[-0.192 - 0.071]	3.639E-01	8.814E-01
Right pars opercularis of inferior frontal gyrus	-0.099	0.067	[-0.230 - 0.033]	1.405E-01	8.814E-01
Left superior temporal gyrus	-0.139	0.105	[-0.345 - 0.066]	1.833E-01	8.814E-01
Right superior temporal gyrus	-0.074	0.116	[-0.301 - 0.153]	5.214E-01	8.814E-01
Left insula	-0.032	0.071	[-0.172 - 0.107]	6.481E-01	9.296E-01
Right insula	-0.097	0.081	[-0.255 - 0.061]	2.290E-01	8.814E-01
Left lingual gyrus	0.064	0.067	[-0.067 - 0.196]	3.367E-01	8.814E-01
Right lingual gyrus	0.065	0.067	[-0.066 - 0.197]	3.297E-01	8.814E-01
Left pars triangularis of inferior frontal gyrus	0.037	0.067	[-0.094 - 0.169]	5.776E-01	9.123E-01
Right pars triangularis of inferior frontal gyrus	0.056	0.069	[-0.079 - 0.191]	4.131E-01	8.814E-01
Left lateral orbitofrontal cortex	-0.003	0.067	[-0.134 - 0.129]	9.694E-01	9.955E-01
Right lateral orbitofrontal cortex	0.092	0.067	[-0.040 - 0.223]	1.726E-01	8.814E-01
Left rostral middle frontal gyrus	0.069	0.067	[-0.063 - 0.200]	3.073E-01	8.814E-01
Right rostral middle frontal gyrus	0.071	0.110	[-0.146 - 0.287]	5.208E-01	8.814E-01
Left middle temporal gyrus	0.045	0.087	[-0.126 - 0.215]	6.071E-01	9.296E-01
Right middle temporal gyrus	0.001	0.067	[-0.131 - 0.132]	9.931E-01	9.955E-01
Left superior frontal gyrus	0.014	0.067	[-0.117 - 0.146]	8.316E-01	9.955E-01
Right superior frontal gyrus	0.065	0.067	[-0.066 - 0.197]	3.296E-01	8.814E-01
Left pars orbitalis of inferior frontal gyrus	0.071	0.067	[-0.061 - 0.203]	2.946E-01	8.814E-01
Right pars orbitalis of inferior frontal gyrus	0.146	0.073	[0.003 - 0.290]	4.610E-02	8.814E-01
Left medial orbitofrontal cortex	0.111	0.130	[-0.143 - 0.365]	3.929E-01	8.814E-01
Right medial orbitofrontal cortex	0.142	0.087	[-0.028 - 0.312]	1.014E-01	8.814E-01
Left inferior temporal gyrus	-0.001	0.067	[-0.133 - 0.130]	9.827E-01	9.955E-01
Right inferior temporal gyrus	-0.040	0.097	[-0.229 - 0.150]	6.809E-01	9.296E-01
Left isthmus cingulate cortex	-0.048	0.067	[-0.180 - 0.083]	4.722E-01	8.814E-01
Right isthmus cingulate cortex	0.046	0.067	[-0.086 - 0.177]	4.938E-01	8.814E-01
Left banks of superior temporal sulcus	-0.032	0.088	[-0.205 - 0.142]	7.209E-01	9.306E-01
Right banks of superior temporal sulcus	-0.081	0.097	[-0.271 - 0.108]	4.003E-01	8.814E-01
Left supramarginal gyrus	-0.099	0.073	[-0.241 - 0.043]	1.717E-01	8.814E-01
Right supramarginal gyrus	-0.061	0.124	[-0.304 - 0.183]	6.258E-01	9.296E-01
Left caudal middle frontal gyrus	-0.136	0.079	[-0.291 - 0.018]	8.299E-02	8.814E-01
Right caudal middle frontal gyrus	-0.058	0.067	[-0.189 - 0.074]	3.893E-01	8.814E-01
Left frontal pole	0.072	0.094	[-0.112 - 0.257]	4.417E-01	8.814E-01
Right frontal pole	0.100	0.082	[-0.061 - 0.260]	2.243E-01	8.814E-01
Left posterior cingulate cortex	-0.044	0.067	[-0.176 - 0.087]	5.073E-01	8.814E-01
Right posterior cingulate cortex	0.060	0.069	[-0.075 - 0.195]	3.830E-01	8.814E-01
Left lateral occipital cortex	0.135	0.067	[0.004 - 0.267]	4.382E-02	8.814E-01
Right lateral occipital cortex	0.134	0.086	[-0.035 - 0.303]	1.207E-01	8.814E-01
Left precentral gyrus	-0.085	0.086	[-0.255 - 0.084]	3.239E-01	8.814E-01
Right precentral gyrus	-0.079	0.120	[-0.315 - 0.157]	5.101E-01	8.814E-01
Left parahippocampal gyrus	0.090	0.067	[-0.041 - 0.222]	1.790E-01	8.814E-01
Right parahippocampal gyrus	0.008	0.084	[-0.157 - 0.173]	9.246E-01	9.955E-01
Left inferior parietal cortex	-0.079	0.142	[-0.357 - 0.199]	5.782E-01	9.123E-01
Right inferior parietal cortex	-0.066	0.094	[-0.249 - 0.118]	4.834E-01	8.814E-01
Left transverse temporal gyrus	-0.112	0.098	[-0.305 - 0.081]	2.554E-01	8.814E-01
Right transverse temporal gyrus	0.011	0.102	[-0.188 - 0.211]	9.136E-01	9.955E-01
Left postcentral gyrus	0.030	0.072	[-0.111 - 0.170]	6.784E-01	9.296E-01
Right postcentral gyrus	-0.001	0.092	[-0.181 - 0.180]	9.955E-01	9.955E-01
Left precuneus	-0.009	0.091	[-0.188 - 0.170]	9.223E-01	9.955E-01
Right precuneus	-0.003	0.090	[-0.179 - 0.173]	9.718E-01	9.955E-01
Left caudal anterior cingulate cortex	-0.025	0.067	[-0.157 - 0.107]	7.091E-01	9.306E-01
Right caudal anterior cingulate cortex	-0.067	0.099	[-0.262 - 0.128]	5.015E-01	8.814E-01
Left cuneus	-0.041	0.067	[-0.172 - 0.091]	5.453E-01	9.004E-01
Right cuneus	0.012	0.067	[-0.119 - 0.144]	8.543E-01	9.955E-01
Left rostral anterior cingulate cortex	0.007	0.093	[-0.175 - 0.189]	9.410E-01	9.955E-01
Right rostral anterior cingulate cortex	-0.022	0.083	[-0.185 - 0.141]	7.896E-01	9.845E-01
Left pericalcarine cortex	-0.020	0.077	[-0.172 - 0.131]	7.904E-01	9.845E-01
Right pericalcarine cortex	-0.066	0.068	[-0.199 - 0.066]	3.259E-01	8.814E-01
Left paracentral lobule	-0.127	0.094	[-0.312 - 0.058]	1.770E-01	8.814E-01
Right paracentral lobule	-0.084	0.102	[-0.284 - 0.116]	4.118E-01	8.814E-01
Left superior parietal cortex	-0.026	0.109	[-0.241 - 0.188]	8.099E-01	9.914E-01
Right superior parietal cortex	0.066	0.074	[-0.078 - 0.210]	3.679E-01	8.814E-01
Left temporal pole	0.082	0.068	[-0.052 - 0.215]	2.307E-01	8.814E-01
Right temporal pole	0.073	0.067	[-0.059 - 0.204]	2.797E-01	8.814E-01
Left entorhinal cortex	-0.002	0.074	[-0.147 - 0.143]	9.801E-01	9.955E-01
Right entorhinal cortex	-0.059	0.088	[-0.230 - 0.113]	5.026E-01	8.814E-01

Supplementary Table S99. Partial correlations between cortical thickness and number of episodes controlling for age and sex in individuals with major depressive disorder over 21 years of old

	Partial *R*	Std. Err.	95% CI	*p*-value	FDR *q*-value
Global mean cortical thickness	0.025	0.071	[-0.115 - 0.164]	7.291E-01	9.412E-01
Left hemisphere	0.003	0.068	[-0.129 - 0.135]	9.645E-01	9.911E-01
Right hemisphere	0.034	0.083	[-0.129 - 0.198]	6.802E-01	9.405E-01
Left fusiform gyrus	0.028	0.068	[-0.104 - 0.161]	6.753E-01	9.405E-01
Right fusiform gyrus	0.126	0.068	[-0.007 - 0.258]	6.283E-02	8.961E-01
Left pars opercularis of inferior frontal gyrus	-0.060	0.068	[-0.192 - 0.073]	3.767E-01	8.961E-01
Right pars opercularis of inferior frontal gyrus	-0.094	0.068	[-0.226 - 0.039]	1.663E-01	8.961E-01
Left superior temporal gyrus	-0.139	0.105	[-0.345 - 0.066]	1.834E-01	8.961E-01
Right superior temporal gyrus	-0.075	0.115	[-0.301 - 0.151]	5.160E-01	8.961E-01
Left insula	-0.029	0.072	[-0.169 - 0.111]	6.873E-01	9.405E-01
Right insula	-0.097	0.081	[-0.256 - 0.062]	2.318E-01	8.961E-01
Left lingual gyrus	0.063	0.068	[-0.070 - 0.195]	3.524E-01	8.961E-01
Right lingual gyrus	0.063	0.068	[-0.069 - 0.196]	3.484E-01	8.961E-01
Left pars triangularis of inferior frontal gyrus	0.041	0.068	[-0.092 - 0.173]	5.475E-01	9.040E-01
Right pars triangularis of inferior frontal gyrus	0.059	0.069	[-0.077 - 0.195]	3.921E-01	8.961E-01
Left lateral orbitofrontal cortex	0.004	0.068	[-0.129 - 0.136]	9.554E-01	9.911E-01
Right lateral orbitofrontal cortex	0.097	0.068	[-0.035 - 0.230]	1.496E-01	8.961E-01
Left rostral middle frontal gyrus	0.075	0.068	[-0.057 - 0.208]	2.649E-01	8.961E-01
Right rostral middle frontal gyrus	0.072	0.111	[-0.146 - 0.290]	5.175E-01	8.961E-01
Left middle temporal gyrus	0.044	0.087	[-0.127 - 0.215]	6.152E-01	9.377E-01
Right middle temporal gyrus	0.001	0.068	[-0.131 - 0.134]	9.846E-01	9.911E-01
Left superior frontal gyrus	0.017	0.068	[-0.116 - 0.149]	8.058E-01	9.864E-01
Right superior frontal gyrus	0.065	0.068	[-0.067 - 0.198]	3.344E-01	8.961E-01
Left pars orbitalis of inferior frontal gyrus	0.065	0.068	[-0.067 - 0.198]	3.345E-01	8.961E-01
Right pars orbitalis of inferior frontal gyrus	0.158	0.074	[0.013 - 0.304]	3.261E-02	8.961E-01
Left medial orbitofrontal cortex	0.111	0.130	[-0.143 - 0.366]	3.915E-01	8.961E-01
Right medial orbitofrontal cortex	0.147	0.087	[-0.023 - 0.317]	9.008E-02	8.961E-01
Left inferior temporal gyrus	0.001	0.068	[-0.131 - 0.134]	9.854E-01	9.911E-01
Right inferior temporal gyrus	-0.041	0.097	[-0.231 - 0.149]	6.744E-01	9.405E-01
Left isthmus cingulate cortex	-0.051	0.068	[-0.183 - 0.082]	4.511E-01	8.961E-01
Right isthmus cingulate cortex	0.042	0.068	[-0.091 - 0.174]	5.381E-01	9.040E-01
Left banks of superior temporal sulcus	-0.032	0.089	[-0.206 - 0.141]	7.153E-01	9.405E-01
Right banks of superior temporal sulcus	-0.082	0.096	[-0.271 - 0.106]	3.918E-01	8.961E-01
Left supramarginal gyrus	-0.101	0.073	[-0.243 - 0.042]	1.662E-01	8.961E-01
Right supramarginal gyrus	-0.062	0.124	[-0.305 - 0.182]	6.207E-01	9.377E-01
Left caudal middle frontal gyrus	-0.136	0.080	[-0.292 - 0.020]	8.787E-02	8.961E-01
Right caudal middle frontal gyrus	-0.049	0.068	[-0.182 - 0.083]	4.669E-01	8.961E-01
Left frontal pole	0.077	0.094	[-0.107 - 0.261]	4.113E-01	8.961E-01
Right frontal pole	0.102	0.082	[-0.060 - 0.263]	2.175E-01	8.961E-01
Left posterior cingulate cortex	-0.046	0.068	[-0.179 - 0.086]	4.917E-01	8.961E-01
Right posterior cingulate cortex	0.051	0.068	[-0.082 - 0.184]	4.506E-01	8.961E-01
Left lateral occipital cortex	0.127	0.068	[-0.005 - 0.260]	5.940E-02	8.961E-01
Right lateral occipital cortex	0.126	0.088	[-0.047 - 0.299]	1.532E-01	8.961E-01
Left precentral gyrus	-0.084	0.087	[-0.255 - 0.088]	3.375E-01	8.961E-01
Right precentral gyrus	-0.080	0.120	[-0.316 - 0.156]	5.082E-01	8.961E-01
Left parahippocampal gyrus	0.091	0.068	[-0.041 - 0.224]	1.773E-01	8.961E-01
Right parahippocampal gyrus	0.011	0.085	[-0.156 - 0.178]	8.978E-01	9.911E-01
Left inferior parietal cortex	-0.082	0.142	[-0.362 - 0.197]	5.626E-01	9.078E-01
Right inferior parietal cortex	-0.069	0.092	[-0.250 - 0.113]	4.580E-01	8.961E-01
Left transverse temporal gyrus	-0.113	0.098	[-0.305 - 0.080]	2.523E-01	8.961E-01
Right transverse temporal gyrus	0.013	0.104	[-0.190 - 0.216]	9.016E-01	9.911E-01
Left postcentral gyrus	0.028	0.072	[-0.112 - 0.169]	6.919E-01	9.405E-01
Right postcentral gyrus	-0.001	0.092	[-0.182 - 0.180]	9.911E-01	9.911E-01
Left precuneus	-0.012	0.091	[-0.189 - 0.166]	8.974E-01	9.911E-01
Right precuneus	-0.005	0.090	[-0.181 - 0.171]	9.562E-01	9.911E-01
Left caudal anterior cingulate cortex	-0.025	0.068	[-0.158 - 0.107]	7.079E-01	9.405E-01
Right caudal anterior cingulate cortex	-0.068	0.100	[-0.264 - 0.128]	4.968E-01	8.961E-01
Left cuneus	-0.036	0.068	[-0.169 - 0.096]	5.913E-01	9.330E-01
Right cuneus	0.010	0.068	[-0.123 - 0.142]	8.843E-01	9.911E-01
Left rostral anterior cingulate cortex	0.010	0.093	[-0.173 - 0.193]	9.125E-01	9.911E-01
Right rostral anterior cingulate cortex	-0.026	0.084	[-0.190 - 0.138]	7.539E-01	9.558E-01
Left pericalcarine cortex	-0.017	0.077	[-0.169 - 0.134]	8.206E-01	9.876E-01
Right pericalcarine cortex	-0.070	0.071	[-0.209 - 0.069]	3.238E-01	8.961E-01
Left paracentral lobule	-0.134	0.093	[-0.317 - 0.048]	1.482E-01	8.961E-01
Right paracentral lobule	-0.088	0.100	[-0.284 - 0.108]	3.785E-01	8.961E-01
Left superior parietal cortex	-0.028	0.109	[-0.241 - 0.186]	8.004E-01	9.864E-01
Right superior parietal cortex	0.063	0.073	[-0.080 - 0.206]	3.856E-01	8.961E-01
Left temporal pole	0.082	0.069	[-0.052 - 0.217]	2.303E-01	8.961E-01
Right temporal pole	0.068	0.068	[-0.065 - 0.200]	3.165E-01	8.961E-01
Left entorhinal cortex	0.006	0.079	[-0.148 - 0.160]	9.358E-01	9.911E-01
Right entorhinal cortex	-0.058	0.088	[-0.231 - 0.114]	5.070E-01	8.961E-01

Supplementary Table S100. Cortical surface area differences between individuals with bipolar disorder type-1 compared to individuals with bipolar disorder type-2 controlling for age and sex

	d	Std. Err.	95% CI	*p*-value	FDR *q*-value
Total cortical surface area	-0.225	0.287	[-0.788 - 0.337]	4.325E-01	9.242E-01
Left hemisphere	-0.249	0.292	[-0.821 - 0.324]	3.941E-01	9.242E-01
Right hemisphere	-0.201	0.281	[-0.753 - 0.350]	4.748E-01	9.242E-01
Left superior frontal gyrus	-0.079	0.213	[-0.496 - 0.337]	7.091E-01	9.606E-01
Right superior frontal gyrus	0.057	0.366	[-0.660 - 0.775]	8.754E-01	9.606E-01
Left pars orbitalis of inferior frontal gyrus	-0.336	0.213	[-0.754 - 0.082]	1.154E-01	7.125E-01
Right pars orbitalis of inferior frontal gyrus	-0.442	0.216	[-0.864 - -0.019]	4.041E-02	7.125E-01
Left precentral gyrus	0.106	0.213	[-0.311 - 0.523]	6.178E-01	9.535E-01
Right precentral gyrus	0.183	0.245	[-0.298 - 0.664]	4.560E-01	9.242E-01
Left lingual gyrus	-0.183	0.214	[-0.603 - 0.236]	3.917E-01	9.242E-01
Right lingual gyrus	-0.389	0.256	[-0.891 - 0.113]	1.286E-01	7.125E-01
Left superior temporal gyrus	-0.132	0.213	[-0.549 - 0.285]	5.359E-01	9.242E-01
Right superior temporal gyrus	-0.090	0.213	[-0.506 - 0.327]	6.732E-01	9.606E-01
Left fusiform gyrus	-0.232	0.302	[-0.823 - 0.359]	4.416E-01	9.242E-01
Right fusiform gyrus	-0.192	0.212	[-0.608 - 0.224]	3.653E-01	9.242E-01
Left inferior parietal cortex	-0.058	0.213	[-0.474 - 0.359]	7.868E-01	9.606E-01
Right inferior parietal cortex	0.214	0.298	[-0.370 - 0.798]	4.729E-01	9.242E-01
Left lateral occipital cortex	-0.355	0.215	[-0.777 - 0.067]	9.930E-02	7.125E-01
Right lateral occipital cortex	-0.090	0.212	[-0.506 - 0.325]	6.706E-01	9.606E-01
Left rostral middle frontal gyrus	-0.489	0.280	[-1.038 - 0.059]	8.051E-02	7.125E-01
Right rostral middle frontal gyrus	-0.587	0.265	[-1.106 - -0.067]	2.682E-02	7.125E-01
Left precuneus	-0.175	0.272	[-0.708 - 0.357]	5.186E-01	9.242E-01
Right precuneus	-0.281	0.274	[-0.818 - 0.255]	3.045E-01	9.242E-01
Left inferior temporal gyrus	0.334	0.213	[-0.084 - 0.752]	1.171E-01	7.125E-01
Right inferior temporal gyrus	0.054	0.330	[-0.594 - 0.701]	8.707E-01	9.606E-01
Left lateral orbitofrontal cortex	-0.323	0.214	[-0.743 - 0.097]	1.319E-01	7.125E-01
Right lateral orbitofrontal cortex	-0.389	0.271	[-0.919 - 0.142]	1.513E-01	7.125E-01
Left middle temporal gyrus	-0.232	0.423	[-1.060 - 0.596]	5.832E-01	9.410E-01
Right middle temporal gyrus	-0.131	0.212	[-0.547 - 0.285]	5.380E-01	9.242E-01
Left postcentral gyrus	-0.335	0.242	[-0.810 - 0.141]	1.677E-01	7.125E-01
Right postcentral gyrus	-0.062	0.292	[-0.633 - 0.510]	8.328E-01	9.606E-01
Left medial orbitofrontal cortex	-0.211	0.213	[-0.629 - 0.207]	3.228E-01	9.242E-01
Right medial orbitofrontal cortex	-0.014	0.218	[-0.441 - 0.412]	9.470E-01	9.606E-01
Left cuneus	-0.467	0.216	[-0.891 - -0.044]	3.062E-02	7.125E-01
Right cuneus	-0.303	0.214	[-0.723 - 0.117]	1.572E-01	7.125E-01
Left pars triangularis of inferior frontal gyrus	-0.291	0.213	[-0.708 - 0.125]	1.706E-01	7.125E-01
Right pars triangularis of inferior frontal gyrus	-0.167	0.213	[-0.584 - 0.250]	4.329E-01	9.242E-01
Left superior parietal cortex	0.206	0.214	[-0.213 - 0.625]	3.359E-01	9.242E-01
Right superior parietal cortex	-0.224	0.276	[-0.764 - 0.317]	4.172E-01	9.242E-01
Left pars opercularis of inferior frontal gyrus	0.064	0.233	[-0.392 - 0.520]	7.833E-01	9.606E-01
Right pars opercularis of inferior frontal gyrus	0.125	0.212	[-0.290 - 0.540]	5.540E-01	9.242E-01
Left supramarginal gyrus	0.011	0.213	[-0.407 - 0.428]	9.606E-01	9.606E-01
Right supramarginal gyrus	-0.172	0.283	[-0.727 - 0.382]	5.426E-01	9.242E-01
Left pericalcarine cortex	-0.420	0.240	[-0.891 - 0.051]	8.055E-02	7.125E-01
Right pericalcarine cortex	-0.443	0.227	[-0.887 - 0.002]	5.092E-02	7.125E-01
Left parahippocampal gyrus	0.052	0.212	[-0.365 - 0.468]	8.083E-01	9.606E-01
Right parahippocampal gyrus	-0.030	0.212	[-0.445 - 0.386]	8.892E-01	9.606E-01
Left caudal middle frontal gyrus	0.185	0.270	[-0.345 - 0.714]	4.937E-01	9.242E-01
Right caudal middle frontal gyrus	0.064	0.212	[-0.351 - 0.479]	7.618E-01	9.606E-01
Left transverse temporal gyrus	-0.211	0.361	[-0.919 - 0.497]	5.597E-01	9.242E-01
Right transverse temporal gyrus	-0.023	0.277	[-0.566 - 0.519]	9.332E-01	9.606E-01
Left banks of superior temporal sulcus	-0.284	0.382	[-1.032 - 0.465]	4.579E-01	9.242E-01
Right banks of superior temporal sulcus	-0.044	0.241	[-0.517 - 0.429]	8.544E-01	9.606E-01
Left caudal anterior cingulate cortex	-0.021	0.256	[-0.523 - 0.480]	9.337E-01	9.606E-01
Right caudal anterior cingulate cortex	-0.203	0.212	[-0.618 - 0.213]	3.390E-01	9.242E-01
Left rostral anterior cingulate cortex	0.017	0.269	[-0.511 - 0.545]	9.499E-01	9.606E-01
Right rostral anterior cingulate cortex	-0.108	0.212	[-0.523 - 0.307]	6.111E-01	9.535E-01
Left posterior cingulate cortex	-0.373	0.214	[-0.792 - 0.046]	8.085E-02	7.125E-01
Right posterior cingulate cortex	-0.152	0.212	[-0.569 - 0.264]	4.738E-01	9.242E-01
Left frontal pole	-0.307	0.342	[-0.976 - 0.363]	3.699E-01	9.242E-01
Right frontal pole	-0.050	0.222	[-0.485 - 0.385]	8.214E-01	9.606E-01
Left paracentral lobule	-0.132	0.402	[-0.919 - 0.655]	7.425E-01	9.606E-01
Right paracentral lobule	0.145	0.400	[-0.640 - 0.930]	7.175E-01	9.606E-01
Left insula	0.024	0.228	[-0.422 - 0.470]	9.148E-01	9.606E-01
Right insula	-0.049	0.323	[-0.683 - 0.584]	8.792E-01	9.606E-01
Left entorhinal cortex	-0.077	0.213	[-0.493 - 0.340]	7.189E-01	9.606E-01
Right entorhinal cortex	0.164	0.212	[-0.252 - 0.581]	4.391E-01	9.242E-01
Left temporal pole	-0.318	0.213	[-0.736 - 0.100]	1.361E-01	7.125E-01
Right temporal pole	0.066	0.228	[-0.381 - 0.513]	7.729E-01	9.606E-01
Left isthmus cingulate cortex	-0.296	0.503	[-1.282 - 0.691]	5.567E-01	9.242E-01
Right isthmus cingulate cortex	-0.045	0.301	[-0.635 - 0.544]	8.798E-01	9.606E-01

Supplementary Table S101. Cortical surface area differences between individuals with bipolar disorder type-1 compared to individuals with bipolar disorder type-2 controlling for age, sex, age × sex, age^2, age^2 × sex, and ICV in individuals with bipolar disorder at 25 years of age or older

	d	Std. Err.	95% CI	*p*-value	FDR *q*-value
Total cortical surface area	-0.054	0.250	[-0.544 - 0.436]	8.282E-01	9.731E-01
Left hemisphere	-0.120	0.275	[-0.658 - 0.418]	6.610E-01	9.731E-01
Right hemisphere	0.012	0.231	[-0.441 - 0.464]	9.589E-01	9.731E-01
Left superior frontal gyrus	0.076	0.229	[-0.373 - 0.526]	7.386E-01	9.731E-01
Right superior frontal gyrus	0.299	0.318	[-0.325 - 0.923]	3.476E-01	9.206E-01
Left pars orbitalis of inferior frontal gyrus	-0.305	0.326	[-0.945 - 0.335]	3.501E-01	9.206E-01
Right pars orbitalis of inferior frontal gyrus	-0.398	0.351	[-1.085 - 0.289]	2.565E-01	8.864E-01
Left precentral gyrus	0.199	0.230	[-0.251 - 0.649]	3.851E-01	9.429E-01
Right precentral gyrus	0.198	0.235	[-0.263 - 0.658]	4.001E-01	9.470E-01
Left lingual gyrus	0.015	0.229	[-0.434 - 0.465]	9.464E-01	9.731E-01
Right lingual gyrus	-0.317	0.269	[-0.845 - 0.211]	2.391E-01	8.864E-01
Left superior temporal gyrus	-0.157	0.230	[-0.607 - 0.294]	4.947E-01	9.731E-01
Right superior temporal gyrus	0.216	0.231	[-0.237 - 0.669]	3.493E-01	9.206E-01
Left fusiform gyrus	-0.013	0.247	[-0.498 - 0.472]	9.585E-01	9.731E-01
Right fusiform gyrus	0.053	0.229	[-0.395 - 0.502]	8.162E-01	9.731E-01
Left inferior parietal cortex	0.200	0.230	[-0.250 - 0.650]	3.838E-01	9.429E-01
Right inferior parietal cortex	0.518	0.413	[-0.292 - 1.327]	2.100E-01	8.864E-01
Left lateral occipital cortex	-0.257	0.231	[-0.709 - 0.196]	2.665E-01	8.864E-01
Right lateral occipital cortex	-0.315	0.438	[-1.172 - 0.543]	4.722E-01	9.731E-01
Left rostral middle frontal gyrus	-0.480	0.321	[-1.109 - 0.148]	1.344E-01	8.864E-01
Right rostral middle frontal gyrus	-0.540	0.329	[-1.184 - 0.105]	1.007E-01	8.864E-01
Left precuneus	-0.053	0.284	[-0.610 - 0.504]	8.520E-01	9.731E-01
Right precuneus	-0.092	0.229	[-0.541 - 0.357]	6.887E-01	9.731E-01
Left inferior temporal gyrus	0.605	0.235	[0.145 - 1.065]	9.974E-03	7.082E-01
Right inferior temporal gyrus	0.256	0.243	[-0.220 - 0.731]	2.923E-01	8.864E-01
Left lateral orbitofrontal cortex	-0.281	0.231	[-0.734 - 0.173]	2.251E-01	8.864E-01
Right lateral orbitofrontal cortex	-0.115	0.230	[-0.565 - 0.336]	6.181E-01	9.731E-01
Left middle temporal gyrus	0.020	0.500	[-0.959 - 1.000]	9.673E-01	9.731E-01
Right middle temporal gyrus	0.053	0.230	[-0.397 - 0.503]	8.183E-01	9.731E-01
Left postcentral gyrus	-0.182	0.230	[-0.632 - 0.268]	4.275E-01	9.731E-01
Right postcentral gyrus	0.285	0.275	[-0.254 - 0.824]	2.996E-01	8.864E-01
Left medial orbitofrontal cortex	-0.246	0.230	[-0.697 - 0.205]	2.853E-01	8.864E-01
Right medial orbitofrontal cortex	-0.139	0.365	[-0.855 - 0.577]	7.033E-01	9.731E-01
Left cuneus	-0.328	0.232	[-0.783 - 0.127]	1.577E-01	8.864E-01
Right cuneus	-0.331	0.231	[-0.784 - 0.122]	1.524E-01	8.864E-01
Left pars triangularis of inferior frontal gyrus	-0.368	0.232	[-0.823 - 0.087]	1.125E-01	8.864E-01
Right pars triangularis of inferior frontal gyrus	0.065	0.371	[-0.663 - 0.792]	8.620E-01	9.731E-01
Left superior parietal cortex	0.344	0.230	[-0.107 - 0.796]	1.346E-01	8.864E-01
Right superior parietal cortex	0.074	0.230	[-0.376 - 0.523]	7.486E-01	9.731E-01
Left pars opercularis of inferior frontal gyrus	0.008	0.247	[-0.477 - 0.493]	9.731E-01	9.731E-01
Right pars opercularis of inferior frontal gyrus	0.023	0.229	[-0.426 - 0.473]	9.192E-01	9.731E-01
Left supramarginal gyrus	0.099	0.230	[-0.353 - 0.550]	6.686E-01	9.731E-01
Right supramarginal gyrus	0.144	0.230	[-0.306 - 0.594]	5.305E-01	9.731E-01
Left pericalcarine cortex	-0.402	0.233	[-0.859 - 0.054]	8.394E-02	8.864E-01
Right pericalcarine cortex	-0.380	0.232	[-0.834 - 0.075]	1.016E-01	8.864E-01
Left parahippocampal gyrus	0.269	0.231	[-0.183 - 0.721]	2.434E-01	8.864E-01
Right parahippocampal gyrus	0.105	0.229	[-0.344 - 0.554]	6.458E-01	9.731E-01
Left caudal middle frontal gyrus	0.158	0.376	[-0.579 - 0.894]	6.750E-01	9.731E-01
Right caudal middle frontal gyrus	0.119	0.229	[-0.331 - 0.568]	6.051E-01	9.731E-01
Left transverse temporal gyrus	-0.230	0.461	[-1.133 - 0.673]	6.176E-01	9.731E-01
Right transverse temporal gyrus	-0.043	0.326	[-0.682 - 0.597]	8.962E-01	9.731E-01
Left banks of superior temporal sulcus	-0.406	0.640	[-1.661 - 0.849]	5.260E-01	9.731E-01
Right banks of superior temporal sulcus	0.091	0.270	[-0.439 - 0.621]	7.367E-01	9.731E-01
Left caudal anterior cingulate cortex	0.078	0.230	[-0.372 - 0.529]	7.340E-01	9.731E-01
Right caudal anterior cingulate cortex	-0.156	0.230	[-0.607 - 0.295]	4.981E-01	9.731E-01
Left rostral anterior cingulate cortex	0.012	0.230	[-0.440 - 0.463]	9.595E-01	9.731E-01
Right rostral anterior cingulate cortex	-0.056	0.230	[-0.507 - 0.396]	8.088E-01	9.731E-01
Left posterior cingulate cortex	-0.326	0.231	[-0.780 - 0.127]	1.579E-01	8.864E-01
Right posterior cingulate cortex	-0.158	0.230	[-0.609 - 0.294]	4.937E-01	9.731E-01
Left frontal pole	-0.399	0.374	[-1.133 - 0.334]	2.862E-01	8.864E-01
Right frontal pole	-0.111	0.406	[-0.906 - 0.685]	7.851E-01	9.731E-01
Left paracentral lobule	-0.330	0.268	[-0.856 - 0.196]	2.190E-01	8.864E-01
Right paracentral lobule	0.090	0.374	[-0.643 - 0.822]	8.107E-01	9.731E-01
Left insula	0.042	0.285	[-0.517 - 0.600]	8.837E-01	9.731E-01
Right insula	0.125	0.230	[-0.327 - 0.576]	5.880E-01	9.731E-01
Left entorhinal cortex	-0.242	0.231	[-0.695 - 0.211]	2.946E-01	8.864E-01
Right entorhinal cortex	0.282	0.230	[-0.169 - 0.733]	2.200E-01	8.864E-01
Left temporal pole	-0.448	0.233	[-0.904 - 0.009]	5.461E-02	8.864E-01
Right temporal pole	0.198	0.333	[-0.454 - 0.850]	5.518E-01	9.731E-01
Left isthmus cingulate cortex	-0.079	0.525	[-1.107 - 0.949]	8.805E-01	9.731E-01
Right isthmus cingulate cortex	0.073	0.354	[-0.621 - 0.767]	8.371E-01	9.731E-01

Supplementary Table S102. Cortical surface area differences between individuals with bipolar disorder type-1 compared to individuals with bipolar disorder type-2 controlling for age and sex at 25 years of age or older

	d	Std. Err.	95% CI	*p*-value	FDR *q*-value
Total cortical surface area	-0.082	0.299	[-0.669 - 0.505]	7.835E-01	9.671E-01
Left hemisphere	-0.103	0.303	[-0.697 - 0.491]	7.339E-01	9.671E-01
Right hemisphere	-0.061	0.295	[-0.640 - 0.518]	8.363E-01	9.671E-01
Left superior frontal gyrus	-0.072	0.231	[-0.524 - 0.380]	7.543E-01	9.671E-01
Right superior frontal gyrus	0.193	0.432	[-0.654 - 1.040]	6.555E-01	9.671E-01
Left pars orbitalis of inferior frontal gyrus	-0.351	0.232	[-0.805 - 0.104]	1.305E-01	9.671E-01
Right pars orbitalis of inferior frontal gyrus	-0.494	0.273	[-1.029 - 0.041]	7.032E-02	9.671E-01
Left precentral gyrus	0.062	0.230	[-0.389 - 0.513]	7.875E-01	9.671E-01
Right precentral gyrus	0.161	0.285	[-0.399 - 0.720]	5.735E-01	9.671E-01
Left lingual gyrus	-0.033	0.229	[-0.482 - 0.416]	8.854E-01	9.671E-01
Right lingual gyrus	-0.284	0.288	[-0.848 - 0.280]	3.234E-01	9.671E-01
Left superior temporal gyrus	-0.143	0.231	[-0.596 - 0.309]	5.354E-01	9.671E-01
Right superior temporal gyrus	-0.039	0.230	[-0.490 - 0.412]	8.655E-01	9.671E-01
Left fusiform gyrus	-0.015	0.255	[-0.514 - 0.485]	9.538E-01	9.674E-01
Right fusiform gyrus	-0.170	0.230	[-0.620 - 0.281]	4.602E-01	9.671E-01
Left inferior parietal cortex	-0.003	0.230	[-0.454 - 0.448]	9.899E-01	9.899E-01
Right inferior parietal cortex	0.293	0.352	[-0.396 - 0.982]	4.049E-01	9.671E-01
Left lateral occipital cortex	-0.201	0.230	[-0.652 - 0.249]	3.812E-01	9.671E-01
Right lateral occipital cortex	-0.084	0.230	[-0.534 - 0.366]	7.145E-01	9.671E-01
Left rostral middle frontal gyrus	-0.333	0.246	[-0.816 - 0.149]	1.754E-01	9.671E-01
Right rostral middle frontal gyrus	-0.402	0.233	[-0.859 - 0.055]	8.469E-02	9.671E-01
Left precuneus	-0.070	0.307	[-0.673 - 0.532]	8.189E-01	9.671E-01
Right precuneus	-0.073	0.230	[-0.523 - 0.377]	7.508E-01	9.671E-01
Left inferior temporal gyrus	0.387	0.232	[-0.067 - 0.841]	9.438E-02	9.671E-01
Right inferior temporal gyrus	0.265	0.295	[-0.314 - 0.843]	3.695E-01	9.671E-01
Left lateral orbitofrontal cortex	-0.237	0.230	[-0.687 - 0.213]	3.021E-01	9.671E-01
Right lateral orbitofrontal cortex	-0.190	0.229	[-0.639 - 0.260]	4.076E-01	9.671E-01
Left middle temporal gyrus	-0.116	0.485	[-1.067 - 0.834]	8.104E-01	9.671E-01
Right middle temporal gyrus	-0.052	0.229	[-0.501 - 0.398]	8.219E-01	9.671E-01
Left postcentral gyrus	-0.232	0.275	[-0.770 - 0.306]	3.984E-01	9.671E-01
Right postcentral gyrus	0.042	0.343	[-0.629 - 0.714]	9.014E-01	9.674E-01
Left medial orbitofrontal cortex	-0.295	0.231	[-0.747 - 0.157]	2.008E-01	9.671E-01
Right medial orbitofrontal cortex	-0.041	0.261	[-0.552 - 0.471]	8.759E-01	9.671E-01
Left cuneus	-0.390	0.242	[-0.864 - 0.083]	1.063E-01	9.671E-01
Right cuneus	-0.365	0.232	[-0.819 - 0.089]	1.148E-01	9.671E-01
Left pars triangularis of inferior frontal gyrus	-0.344	0.231	[-0.796 - 0.108]	1.353E-01	9.671E-01
Right pars triangularis of inferior frontal gyrus	-0.193	0.231	[-0.647 - 0.260]	4.028E-01	9.671E-01
Left superior parietal cortex	0.349	0.231	[-0.105 - 0.802]	1.317E-01	9.671E-01
Right superior parietal cortex	-0.058	0.230	[-0.509 - 0.392]	7.998E-01	9.671E-01
Left pars opercularis of inferior frontal gyrus	0.020	0.279	[-0.526 - 0.567]	9.423E-01	9.674E-01
Right pars opercularis of inferior frontal gyrus	0.054	0.229	[-0.395 - 0.503]	8.133E-01	9.671E-01
Left supramarginal gyrus	0.116	0.230	[-0.335 - 0.567]	6.137E-01	9.671E-01
Right supramarginal gyrus	0.083	0.229	[-0.365 - 0.532]	7.161E-01	9.671E-01
Left pericalcarine cortex	-0.385	0.286	[-0.947 - 0.176]	1.786E-01	9.671E-01
Right pericalcarine cortex	-0.355	0.261	[-0.867 - 0.156]	1.733E-01	9.671E-01
Left parahippocampal gyrus	0.065	0.230	[-0.386 - 0.516]	7.766E-01	9.671E-01
Right parahippocampal gyrus	0.071	0.229	[-0.378 - 0.519]	7.568E-01	9.671E-01
Left caudal middle frontal gyrus	0.158	0.331	[-0.490 - 0.806]	6.325E-01	9.671E-01
Right caudal middle frontal gyrus	-0.016	0.229	[-0.465 - 0.432]	9.427E-01	9.674E-01
Left transverse temporal gyrus	-0.331	0.425	[-1.164 - 0.502]	4.365E-01	9.671E-01
Right transverse temporal gyrus	-0.187	0.269	[-0.715 - 0.340]	4.858E-01	9.671E-01
Left banks of superior temporal sulcus	-0.197	0.481	[-1.140 - 0.745]	6.813E-01	9.671E-01
Right banks of superior temporal sulcus	0.071	0.244	[-0.406 - 0.549]	7.698E-01	9.671E-01
Left caudal anterior cingulate cortex	0.079	0.284	[-0.477 - 0.636]	7.804E-01	9.671E-01
Right caudal anterior cingulate cortex	-0.239	0.230	[-0.690 - 0.212]	2.987E-01	9.671E-01
Left rostral anterior cingulate cortex	-0.047	0.311	[-0.656 - 0.563]	8.806E-01	9.671E-01
Right rostral anterior cingulate cortex	-0.182	0.229	[-0.631 - 0.268]	4.283E-01	9.671E-01
Left posterior cingulate cortex	-0.440	0.232	[-0.895 - 0.016]	5.846E-02	9.671E-01
Right posterior cingulate cortex	-0.161	0.230	[-0.613 - 0.290]	4.842E-01	9.671E-01
Left frontal pole	-0.347	0.397	[-1.125 - 0.431]	3.818E-01	9.671E-01
Right frontal pole	-0.086	0.259	[-0.594 - 0.422]	7.398E-01	9.671E-01
Left paracentral lobule	-0.370	0.405	[-1.163 - 0.424]	3.613E-01	9.671E-01
Right paracentral lobule	-0.049	0.495	[-1.019 - 0.921]	9.211E-01	9.674E-01
Left insula	0.122	0.235	[-0.338 - 0.582]	6.037E-01	9.671E-01
Right insula	0.076	0.375	[-0.659 - 0.810]	8.401E-01	9.671E-01
Left entorhinal cortex	-0.107	0.230	[-0.558 - 0.344]	6.412E-01	9.671E-01
Right entorhinal cortex	0.207	0.230	[-0.245 - 0.659]	3.693E-01	9.671E-01
Left temporal pole	-0.261	0.231	[-0.713 - 0.191]	2.570E-01	9.671E-01
Right temporal pole	0.177	0.255	[-0.322 - 0.677]	4.862E-01	9.671E-01
Left isthmus cingulate cortex	0.087	0.466	[-0.825 - 1.000]	8.513E-01	9.671E-01
Right isthmus cingulate cortex	0.150	0.266	[-0.371 - 0.672]	5.720E-01	9.671E-01

Supplementary Table S103. Cortical surface area differences between individuals with first episode major depressive disorder versus healthy comparison subjects controlling for age and sex

	d	Std. Err.	95% CI	*p*-value	FDR *q*-value
Total cortical surface area	-0.113	0.129	[-0.366 - 0.140]	3.816E-01	8.770E-01
Left hemisphere	-0.107	0.134	[-0.370 - 0.156]	4.251E-01	8.770E-01
Right hemisphere	-0.118	0.123	[-0.359 - 0.124]	3.385E-01	8.770E-01
Left superior frontal gyrus	-0.091	0.110	[-0.307 - 0.125]	4.100E-01	8.770E-01
Right superior frontal gyrus	-0.139	0.137	[-0.407 - 0.130]	3.121E-01	8.770E-01
Left pars orbitalis of inferior frontal gyrus	-0.158	0.097	[-0.348 - 0.033]	1.047E-01	8.770E-01
Right pars orbitalis of inferior frontal gyrus	-0.102	0.112	[-0.322 - 0.118]	3.623E-01	8.770E-01
Left precentral gyrus	-0.019	0.097	[-0.210 - 0.172]	8.475E-01	9.618E-01
Right precentral gyrus	-0.018	0.097	[-0.209 - 0.172]	8.512E-01	9.618E-01
Left lingual gyrus	-0.019	0.115	[-0.244 - 0.205]	8.664E-01	9.618E-01
Right lingual gyrus	-0.023	0.115	[-0.248 - 0.203]	8.443E-01	9.618E-01
Left superior temporal gyrus	-0.071	0.097	[-0.261 - 0.120]	4.690E-01	8.900E-01
Right superior temporal gyrus	-0.098	0.112	[-0.318 - 0.122]	3.829E-01	8.770E-01
Left fusiform gyrus	-0.125	0.131	[-0.382 - 0.133]	3.419E-01	8.770E-01
Right fusiform gyrus	-0.157	0.097	[-0.348 - 0.034]	1.071E-01	8.770E-01
Left inferior parietal cortex	-0.089	0.127	[-0.337 - 0.160]	4.856E-01	8.900E-01
Right inferior parietal cortex	0.029	0.195	[-0.354 - 0.412]	8.826E-01	9.618E-01
Left lateral occipital cortex	-0.061	0.140	[-0.335 - 0.214]	6.643E-01	8.900E-01
Right lateral occipital cortex	-0.148	0.120	[-0.383 - 0.088]	2.186E-01	8.770E-01
Left rostral middle frontal gyrus	-0.087	0.168	[-0.416 - 0.242]	6.044E-01	8.900E-01
Right rostral middle frontal gyrus	-0.004	0.159	[-0.316 - 0.309]	9.820E-01	9.820E-01
Left precuneus	-0.206	0.098	[-0.397 - -0.015]	3.463E-02	8.196E-01
Right precuneus	-0.076	0.097	[-0.267 - 0.114]	4.323E-01	8.770E-01
Left inferior temporal gyrus	0.007	0.097	[-0.184 - 0.197]	9.459E-01	9.618E-01
Right inferior temporal gyrus	-0.128	0.097	[-0.319 - 0.063]	1.885E-01	8.770E-01
Left lateral orbitofrontal cortex	-0.131	0.097	[-0.322 - 0.059]	1.770E-01	8.770E-01
Right lateral orbitofrontal cortex	-0.062	0.101	[-0.260 - 0.136]	5.404E-01	8.900E-01
Left middle temporal gyrus	-0.051	0.139	[-0.324 - 0.221]	7.117E-01	9.040E-01
Right middle temporal gyrus	-0.128	0.120	[-0.363 - 0.107]	2.846E-01	8.770E-01
Left postcentral gyrus	-0.120	0.151	[-0.417 - 0.176]	4.270E-01	8.770E-01
Right postcentral gyrus	-0.125	0.127	[-0.374 - 0.124]	3.255E-01	8.770E-01
Left medial orbitofrontal cortex	-0.167	0.138	[-0.437 - 0.102]	2.240E-01	8.770E-01
Right medial orbitofrontal cortex	-0.164	0.098	[-0.355 - 0.027]	9.194E-02	8.770E-01
Left cuneus	-0.258	0.098	[-0.449 - -0.066]	8.294E-03	5.889E-01
Right cuneus	-0.137	0.097	[-0.328 - 0.054]	1.592E-01	8.770E-01
Left pars triangularis of inferior frontal gyrus	-0.165	0.103	[-0.368 - 0.038]	1.104E-01	8.770E-01
Right pars triangularis of inferior frontal gyrus	-0.152	0.097	[-0.342 - 0.039]	1.183E-01	8.770E-01
Left superior parietal cortex	-0.091	0.109	[-0.305 - 0.122]	4.034E-01	8.770E-01
Right superior parietal cortex	-0.093	0.097	[-0.284 - 0.098]	3.380E-01	8.770E-01
Left pars opercularis of inferior frontal gyrus	-0.016	0.097	[-0.206 - 0.175]	8.723E-01	9.618E-01
Right pars opercularis of inferior frontal gyrus	0.051	0.132	[-0.207 - 0.309]	6.992E-01	9.040E-01
Left supramarginal gyrus	-0.046	0.097	[-0.237 - 0.145]	6.355E-01	8.900E-01
Right supramarginal gyrus	-0.053	0.110	[-0.268 - 0.161]	6.268E-01	8.900E-01
Left pericalcarine cortex	-0.114	0.097	[-0.305 - 0.076]	2.399E-01	8.770E-01
Right pericalcarine cortex	-0.230	0.097	[-0.421 - -0.039]	1.836E-02	6.517E-01
Left parahippocampal gyrus	0.045	0.135	[-0.219 - 0.309]	7.385E-01	9.040E-01
Right parahippocampal gyrus	-0.131	0.097	[-0.322 - 0.060]	1.789E-01	8.770E-01
Left caudal middle frontal gyrus	-0.044	0.097	[-0.235 - 0.147]	6.523E-01	8.900E-01
Right caudal middle frontal gyrus	0.034	0.097	[-0.157 - 0.225]	7.284E-01	9.040E-01
Left transverse temporal gyrus	-0.137	0.097	[-0.328 - 0.054]	1.595E-01	8.770E-01
Right transverse temporal gyrus	-0.052	0.097	[-0.242 - 0.139]	5.950E-01	8.900E-01
Left banks of superior temporal sulcus	-0.016	0.117	[-0.245 - 0.213]	8.890E-01	9.618E-01
Right banks of superior temporal sulcus	-0.077	0.212	[-0.493 - 0.338]	7.158E-01	9.040E-01
Left caudal anterior cingulate cortex	-0.134	0.105	[-0.340 - 0.073]	2.040E-01	8.770E-01
Right caudal anterior cingulate cortex	-0.101	0.119	[-0.335 - 0.132]	3.944E-01	8.770E-01
Left rostral anterior cingulate cortex	-0.132	0.139	[-0.403 - 0.140]	3.425E-01	8.770E-01
Right rostral anterior cingulate cortex	-0.018	0.163	[-0.336 - 0.301]	9.137E-01	9.618E-01
Left posterior cingulate cortex	-0.048	0.109	[-0.261 - 0.165]	6.587E-01	8.900E-01
Right posterior cingulate cortex	-0.087	0.130	[-0.342 - 0.168]	5.024E-01	8.900E-01
Left frontal pole	-0.139	0.097	[-0.330 - 0.051]	1.522E-01	8.770E-01
Right frontal pole	0.008	0.125	[-0.236 - 0.252]	9.483E-01	9.618E-01
Left paracentral lobule	0.008	0.097	[-0.183 - 0.198]	9.368E-01	9.618E-01
Right paracentral lobule	-0.011	0.119	[-0.245 - 0.223]	9.272E-01	9.618E-01
Left insula	0.069	0.127	[-0.180 - 0.318]	5.877E-01	8.900E-01
Right insula	-0.069	0.111	[-0.285 - 0.148]	5.353E-01	8.900E-01
Left entorhinal cortex	-0.126	0.109	[-0.340 - 0.088]	2.473E-01	8.770E-01
Right entorhinal cortex	0.048	0.097	[-0.143 - 0.238]	6.240E-01	8.900E-01
Left temporal pole	-0.071	0.105	[-0.277 - 0.135]	4.988E-01	8.900E-01
Right temporal pole	-0.052	0.097	[-0.243 - 0.138]	5.893E-01	8.900E-01
Left isthmus cingulate cortex	0.059	0.116	[-0.169 - 0.287]	6.130E-01	8.900E-01
Right isthmus cingulate cortex	0.044	0.097	[-0.146 - 0.235]	6.480E-01	8.900E-01

Supplementary Table S104. Cortical surface area differences between individuals with first episode major depressive disorder versus healthy comparison subjects controlling for age and sex over 21 years of old

	d	Std. Err.	95% CI	*p*-value	FDR *q*-value
Total cortical surface area	-0.080	0.150	[-0.374 - 0.213]	5.923E-01	9.819E-01
Left hemisphere	-0.070	0.157	[-0.378 - 0.238]	6.562E-01	9.891E-01
Right hemisphere	-0.090	0.141	[-0.367 - 0.187]	5.244E-01	9.819E-01
Left superior frontal gyrus	-0.062	0.125	[-0.307 - 0.183]	6.178E-01	9.819E-01
Right superior frontal gyrus	-0.127	0.150	[-0.421 - 0.166]	3.950E-01	9.819E-01
Left pars orbitalis of inferior frontal gyrus	-0.136	0.100	[-0.331 - 0.059]	1.725E-01	9.819E-01
Right pars orbitalis of inferior frontal gyrus	-0.088	0.127	[-0.337 - 0.162]	4.916E-01	9.819E-01
Left precentral gyrus	0.011	0.117	[-0.219 - 0.240]	9.282E-01	9.933E-01
Right precentral gyrus	-0.032	0.099	[-0.227 - 0.163]	7.476E-01	9.933E-01
Left lingual gyrus	0.012	0.132	[-0.246 - 0.271]	9.251E-01	9.933E-01
Right lingual gyrus	-0.002	0.114	[-0.226 - 0.222]	9.842E-01	9.960E-01
Left superior temporal gyrus	-0.055	0.111	[-0.272 - 0.163]	6.223E-01	9.819E-01
Right superior temporal gyrus	-0.067	0.134	[-0.329 - 0.195]	6.162E-01	9.819E-01
Left fusiform gyrus	-0.122	0.138	[-0.392 - 0.148]	3.749E-01	9.819E-01
Right fusiform gyrus	-0.163	0.100	[-0.359 - 0.032]	1.015E-01	9.819E-01
Left inferior parietal cortex	-0.074	0.139	[-0.346 - 0.197]	5.920E-01	9.819E-01
Right inferior parietal cortex	0.076	0.215	[-0.346 - 0.498]	7.242E-01	9.891E-01
Left lateral occipital cortex	-0.032	0.154	[-0.334 - 0.269]	8.348E-01	9.933E-01
Right lateral occipital cortex	-0.132	0.127	[-0.380 - 0.116]	2.973E-01	9.819E-01
Left rostral middle frontal gyrus	-0.076	0.179	[-0.427 - 0.274]	6.693E-01	9.891E-01
Right rostral middle frontal gyrus	0.012	0.169	[-0.320 - 0.344]	9.442E-01	9.933E-01
Left precuneus	-0.203	0.100	[-0.398 - -0.007]	4.224E-02	9.819E-01
Right precuneus	-0.059	0.101	[-0.257 - 0.139]	5.594E-01	9.819E-01
Left inferior temporal gyrus	0.010	0.099	[-0.185 - 0.205]	9.230E-01	9.933E-01
Right inferior temporal gyrus	-0.133	0.100	[-0.328 - 0.062]	1.820E-01	9.819E-01
Left lateral orbitofrontal cortex	-0.115	0.100	[-0.310 - 0.080]	2.466E-01	9.819E-01
Right lateral orbitofrontal cortex	-0.022	0.100	[-0.217 - 0.173]	8.244E-01	9.933E-01
Left middle temporal gyrus	-0.023	0.156	[-0.329 - 0.283]	8.831E-01	9.933E-01
Right middle temporal gyrus	-0.131	0.130	[-0.385 - 0.123]	3.108E-01	9.819E-01
Left postcentral gyrus	-0.150	0.139	[-0.424 - 0.123]	2.815E-01	9.819E-01
Right postcentral gyrus	-0.156	0.129	[-0.408 - 0.097]	2.278E-01	9.819E-01
Left medial orbitofrontal cortex	-0.130	0.159	[-0.442 - 0.182]	4.133E-01	9.819E-01
Right medial orbitofrontal cortex	-0.155	0.110	[-0.371 - 0.061]	1.595E-01	9.819E-01
Left cuneus	-0.221	0.125	[-0.466 - 0.024]	7.704E-02	9.819E-01
Right cuneus	-0.123	0.100	[-0.318 - 0.072]	2.169E-01	9.819E-01
Left pars triangularis of inferior frontal gyrus	-0.147	0.111	[-0.366 - 0.071]	1.857E-01	9.819E-01
Right pars triangularis of inferior frontal gyrus	-0.134	0.099	[-0.328 - 0.061]	1.789E-01	9.819E-01
Left superior parietal cortex	-0.088	0.113	[-0.311 - 0.134]	4.363E-01	9.819E-01
Right superior parietal cortex	-0.070	0.099	[-0.265 - 0.125]	4.829E-01	9.819E-01
Left pars opercularis of inferior frontal gyrus	0.004	0.099	[-0.190 - 0.199]	9.653E-01	9.933E-01
Right pars opercularis of inferior frontal gyrus	0.125	0.156	[-0.181 - 0.431]	4.230E-01	9.819E-01
Left supramarginal gyrus	-0.035	0.101	[-0.233 - 0.162]	7.244E-01	9.891E-01
Right supramarginal gyrus	-0.051	0.113	[-0.272 - 0.171]	6.550E-01	9.891E-01
Left pericalcarine cortex	-0.079	0.102	[-0.280 - 0.121]	4.375E-01	9.819E-01
Right pericalcarine cortex	-0.201	0.100	[-0.396 - -0.006]	4.292E-02	9.819E-01
Left parahippocampal gyrus	0.011	0.127	[-0.238 - 0.260]	9.289E-01	9.933E-01
Right parahippocampal gyrus	-0.132	0.100	[-0.328 - 0.063]	1.830E-01	9.819E-01
Left caudal middle frontal gyrus	-0.005	0.111	[-0.222 - 0.212]	9.637E-01	9.933E-01
Right caudal middle frontal gyrus	0.059	0.106	[-0.148 - 0.266]	5.788E-01	9.819E-01
Left transverse temporal gyrus	-0.117	0.100	[-0.312 - 0.078]	2.394E-01	9.819E-01
Right transverse temporal gyrus	-0.036	0.099	[-0.231 - 0.159]	7.187E-01	9.891E-01
Left banks of superior temporal sulcus	-0.018	0.124	[-0.261 - 0.225]	8.875E-01	9.933E-01
Right banks of superior temporal sulcus	-0.041	0.234	[-0.499 - 0.418]	8.625E-01	9.933E-01
Left caudal anterior cingulate cortex	-0.119	0.100	[-0.314 - 0.076]	2.319E-01	9.819E-01
Right caudal anterior cingulate cortex	-0.072	0.140	[-0.346 - 0.201]	6.051E-01	9.819E-01
Left rostral anterior cingulate cortex	-0.109	0.158	[-0.418 - 0.199]	4.874E-01	9.819E-01
Right rostral anterior cingulate cortex	0.047	0.187	[-0.320 - 0.414]	8.028E-01	9.933E-01
Left posterior cingulate cortex	-0.036	0.127	[-0.284 - 0.213]	7.785E-01	9.933E-01
Right posterior cingulate cortex	-0.092	0.136	[-0.358 - 0.174]	4.997E-01	9.819E-01
Left frontal pole	-0.140	0.100	[-0.335 - 0.055]	1.583E-01	9.819E-01
Right frontal pole	-0.001	0.137	[-0.268 - 0.267]	9.960E-01	9.960E-01
Left paracentral lobule	0.008	0.100	[-0.187 - 0.203]	9.343E-01	9.933E-01
Right paracentral lobule	-0.024	0.121	[-0.261 - 0.213]	8.410E-01	9.933E-01
Left insula	0.087	0.151	[-0.210 - 0.384]	5.670E-01	9.819E-01
Right insula	-0.083	0.121	[-0.321 - 0.155]	4.946E-01	9.819E-01
Left entorhinal cortex	-0.177	0.100	[-0.373 - 0.018]	7.576E-02	9.819E-01
Right entorhinal cortex	0.050	0.099	[-0.145 - 0.245]	6.149E-01	9.819E-01
Left temporal pole	-0.047	0.117	[-0.277 - 0.182]	6.848E-01	9.891E-01
Right temporal pole	-0.056	0.099	[-0.251 - 0.138]	5.706E-01	9.819E-01
Left isthmus cingulate cortex	0.069	0.105	[-0.137 - 0.275]	5.108E-01	9.819E-01
Right isthmus cingulate cortex	0.071	0.099	[-0.124 - 0.266]	4.727E-01	9.819E-01

Supplementary Table S105. Cortical surface area differences between individuals with recurrent episode major depressive disorder versus healthy comparison subjects controlling for age and sex

	d	Std. Err.	95% CI	*p*-value	FDR *q*-value
Total cortical surface area	-0.241	0.099	[-0.435 - -0.047]	1.473E-02	6.352E-02
Left hemisphere	-0.243	0.096	[-0.432 - -0.054]	1.190E-02	6.352E-02
Right hemisphere	-0.238	0.101	[-0.437 - -0.039]	1.884E-02	7.017E-02
Left superior frontal gyrus	-0.278	0.098	[-0.469 - -0.086]	4.525E-03	5.731E-02
Right superior frontal gyrus	-0.238	0.096	[-0.427 - -0.049]	1.362E-02	6.352E-02
Left pars orbitalis of inferior frontal gyrus	-0.205	0.098	[-0.397 - -0.014]	3.568E-02	1.101E-01
Right pars orbitalis of inferior frontal gyrus	-0.163	0.079	[-0.318 - -0.008]	3.926E-02	1.115E-01
Left precentral gyrus	-0.169	0.091	[-0.347 - 0.009]	6.256E-02	1.388E-01
Right precentral gyrus	-0.087	0.079	[-0.242 - 0.068]	2.714E-01	4.015E-01
Left lingual gyrus	-0.170	0.102	[-0.370 - 0.029]	9.441E-02	1.915E-01
Right lingual gyrus	-0.254	0.079	[-0.410 - -0.098]	1.373E-03	3.249E-02
Left superior temporal gyrus	-0.175	0.109	[-0.388 - 0.037]	1.061E-01	2.035E-01
Right superior temporal gyrus	-0.193	0.109	[-0.407 - 0.021]	7.720E-02	1.661E-01
Left fusiform gyrus	-0.165	0.105	[-0.371 - 0.041]	1.157E-01	2.161E-01
Right fusiform gyrus	-0.258	0.079	[-0.414 - -0.102]	1.162E-03	3.249E-02
Left inferior parietal cortex	-0.234	0.090	[-0.410 - -0.059]	8.980E-03	6.352E-02
Right inferior parietal cortex	-0.042	0.093	[-0.223 - 0.140]	6.535E-01	6.925E-01
Left lateral occipital cortex	-0.106	0.079	[-0.261 - 0.049]	1.803E-01	3.123E-01
Right lateral occipital cortex	-0.224	0.089	[-0.399 - -0.050]	1.186E-02	6.352E-02
Left rostral middle frontal gyrus	-0.160	0.079	[-0.315 - -0.005]	4.336E-02	1.122E-01
Right rostral middle frontal gyrus	-0.083	0.081	[-0.242 - 0.075]	3.015E-01	4.299E-01
Left precuneus	-0.209	0.079	[-0.365 - -0.054]	8.355E-03	6.352E-02
Right precuneus	-0.083	0.096	[-0.272 - 0.106]	3.893E-01	4.849E-01
Left inferior temporal gyrus	-0.196	0.079	[-0.351 - -0.040]	1.366E-02	6.352E-02
Right inferior temporal gyrus	-0.093	0.093	[-0.276 - 0.090]	3.188E-01	4.434E-01
Left lateral orbitofrontal cortex	-0.157	0.079	[-0.313 - -0.002]	4.743E-02	1.122E-01
Right lateral orbitofrontal cortex	-0.051	0.079	[-0.206 - 0.104]	5.202E-01	5.957E-01
Left middle temporal gyrus	-0.192	0.079	[-0.348 - -0.037]	1.544E-02	6.352E-02
Right middle temporal gyrus	-0.191	0.093	[-0.373 - -0.010]	3.865E-02	1.115E-01
Left postcentral gyrus	-0.198	0.103	[-0.400 - 0.004]	5.532E-02	1.267E-01
Right postcentral gyrus	-0.273	0.101	[-0.470 - -0.076]	6.640E-03	5.893E-02
Left medial orbitofrontal cortex	-0.138	0.079	[-0.293 - 0.017]	8.128E-02	1.697E-01
Right medial orbitofrontal cortex	-0.198	0.079	[-0.354 - -0.043]	1.246E-02	6.352E-02
Left cuneus	-0.175	0.118	[-0.406 - 0.057]	1.394E-01	2.475E-01
Right cuneus	-0.276	0.097	[-0.465 - -0.086]	4.385E-03	5.731E-02
Left pars triangularis of inferior frontal gyrus	-0.207	0.086	[-0.375 - -0.038]	1.610E-02	6.352E-02
Right pars triangularis of inferior frontal gyrus	-0.208	0.090	[-0.385 - -0.032]	2.075E-02	7.017E-02
Left superior parietal cortex	-0.083	0.081	[-0.241 - 0.075]	3.027E-01	4.299E-01
Right superior parietal cortex	-0.130	0.111	[-0.347 - 0.086]	2.379E-01	3.929E-01
Left pars opercularis of inferior frontal gyrus	-0.035	0.132	[-0.295 - 0.224]	7.892E-01	8.114E-01
Right pars opercularis of inferior frontal gyrus	-0.058	0.093	[-0.240 - 0.124]	5.318E-01	5.994E-01
Left supramarginal gyrus	-0.086	0.115	[-0.311 - 0.139]	4.542E-01	5.400E-01
Right supramarginal gyrus	-0.185	0.093	[-0.368 - -0.003]	4.611E-02	1.122E-01
Left pericalcarine cortex	-0.265	0.096	[-0.453 - -0.076]	5.929E-03	5.893E-02
Right pericalcarine cortex	-0.319	0.089	[-0.494 - -0.144]	3.591E-04	2.550E-02
Left parahippocampal gyrus	-0.054	0.092	[-0.233 - 0.126]	5.570E-01	6.084E-01
Right parahippocampal gyrus	-0.107	0.093	[-0.289 - 0.076]	2.523E-01	3.981E-01
Left caudal middle frontal gyrus	-0.159	0.079	[-0.314 - -0.003]	4.513E-02	1.122E-01
Right caudal middle frontal gyrus	-0.067	0.092	[-0.248 - 0.114]	4.707E-01	5.478E-01
Left transverse temporal gyrus	-0.088	0.079	[-0.243 - 0.068]	2.689E-01	4.015E-01
Right transverse temporal gyrus	-0.184	0.079	[-0.339 - -0.028]	2.056E-02	7.017E-02
Left banks of superior temporal sulcus	-0.112	0.097	[-0.302 - 0.079]	2.508E-01	3.981E-01
Right banks of superior temporal sulcus	-0.076	0.130	[-0.331 - 0.178]	5.568E-01	6.084E-01
Left caudal anterior cingulate cortex	-0.129	0.079	[-0.284 - 0.027]	1.043E-01	2.035E-01
Right caudal anterior cingulate cortex	-0.072	0.079	[-0.227 - 0.083]	3.638E-01	4.697E-01
Left rostral anterior cingulate cortex	-0.039	0.079	[-0.194 - 0.116]	6.234E-01	6.706E-01
Right rostral anterior cingulate cortex	0.008	0.079	[-0.147 - 0.163]	9.215E-01	9.215E-01
Left posterior cingulate cortex	-0.027	0.079	[-0.182 - 0.128]	7.348E-01	7.673E-01
Right posterior cingulate cortex	-0.168	0.083	[-0.330 - -0.005]	4.310E-02	1.122E-01
Left frontal pole	-0.172	0.079	[-0.327 - -0.016]	3.031E-02	9.780E-02
Right frontal pole	-0.224	0.079	[-0.379 - -0.068]	4.843E-03	5.731E-02
Left paracentral lobule	-0.064	0.079	[-0.219 - 0.091]	4.201E-01	5.143E-01
Right paracentral lobule	-0.122	0.079	[-0.277 - 0.033]	1.229E-01	2.237E-01
Left insula	-0.083	0.086	[-0.253 - 0.086]	3.340E-01	4.474E-01
Right insula	-0.077	0.104	[-0.281 - 0.126]	4.563E-01	5.400E-01
Left entorhinal cortex	0.088	0.101	[-0.110 - 0.286]	3.825E-01	4.849E-01
Right entorhinal cortex	0.095	0.104	[-0.108 - 0.299]	3.578E-01	4.697E-01
Left temporal pole	-0.141	0.109	[-0.355 - 0.072]	1.943E-01	3.285E-01
Right temporal pole	-0.088	0.079	[-0.243 - 0.068]	2.690E-01	4.015E-01
Left isthmus cingulate cortex	-0.078	0.079	[-0.233 - 0.077]	3.247E-01	4.434E-01
Right isthmus cingulate cortex	-0.022	0.088	[-0.196 - 0.151]	8.000E-01	8.114E-01

Supplementary Table S106. Cortical surface area differences between individuals with recurrent episode major depressive disorder versus healthy comparison subjects controlling for age and sex over 21 years of old

	d	Std. Err.	95% CI	*p*-value	FDR *q*-value
Total cortical surface area	-0.230	0.100	[-0.426 - -0.035]	2.086E-02	9.258E-02
Left hemisphere	-0.232	0.098	[-0.423 - -0.041]	1.734E-02	8.962E-02
Right hemisphere	-0.227	0.102	[-0.427 - -0.027]	2.582E-02	9.650E-02
Left superior frontal gyrus	-0.270	0.099	[-0.464 - -0.076]	6.410E-03	8.124E-02
Right superior frontal gyrus	-0.230	0.098	[-0.422 - -0.039]	1.856E-02	8.962E-02
Left pars orbitalis of inferior frontal gyrus	-0.211	0.101	[-0.409 - -0.012]	3.732E-02	1.152E-01
Right pars orbitalis of inferior frontal gyrus	-0.173	0.080	[-0.330 - -0.016]	3.066E-02	9.941E-02
Left precentral gyrus	-0.152	0.096	[-0.340 - 0.036]	1.119E-01	2.235E-01
Right precentral gyrus	-0.081	0.080	[-0.238 - 0.076]	3.105E-01	4.491E-01
Left lingual gyrus	-0.169	0.101	[-0.366 - 0.028]	9.349E-02	1.952E-01
Right lingual gyrus	-0.248	0.080	[-0.405 - -0.091]	1.934E-03	4.577E-02
Left superior temporal gyrus	-0.175	0.111	[-0.393 - 0.042]	1.133E-01	2.235E-01
Right superior temporal gyrus	-0.189	0.111	[-0.407 - 0.029]	8.914E-02	1.918E-01
Left fusiform gyrus	-0.158	0.106	[-0.366 - 0.049]	1.353E-01	2.527E-01
Right fusiform gyrus	-0.250	0.080	[-0.407 - -0.093]	1.819E-03	4.577E-02
Left inferior parietal cortex	-0.229	0.094	[-0.414 - -0.044]	1.533E-02	8.962E-02
Right inferior parietal cortex	-0.037	0.095	[-0.223 - 0.148]	6.927E-01	7.340E-01
Left lateral occipital cortex	-0.090	0.080	[-0.247 - 0.066]	2.573E-01	4.226E-01
Right lateral occipital cortex	-0.211	0.089	[-0.385 - -0.037]	1.768E-02	8.962E-02
Left rostral middle frontal gyrus	-0.146	0.080	[-0.303 - 0.011]	6.765E-02	1.549E-01
Right rostral middle frontal gyrus	-0.068	0.085	[-0.235 - 0.099]	4.271E-01	5.593E-01
Left precuneus	-0.193	0.080	[-0.350 - -0.036]	1.592E-02	8.962E-02
Right precuneus	-0.068	0.100	[-0.264 - 0.128]	4.961E-01	5.970E-01
Left inferior temporal gyrus	-0.179	0.080	[-0.336 - -0.023]	2.489E-02	9.650E-02
Right inferior temporal gyrus	-0.092	0.092	[-0.271 - 0.088]	3.174E-01	4.491E-01
Left lateral orbitofrontal cortex	-0.159	0.080	[-0.316 - -0.002]	4.718E-02	1.241E-01
Right lateral orbitofrontal cortex	-0.048	0.080	[-0.205 - 0.109]	5.479E-01	6.201E-01
Left middle temporal gyrus	-0.182	0.080	[-0.339 - -0.025]	2.316E-02	9.650E-02
Right middle temporal gyrus	-0.191	0.094	[-0.376 - -0.006]	4.333E-02	1.231E-01
Left postcentral gyrus	-0.193	0.105	[-0.399 - 0.013]	6.646E-02	1.549E-01
Right postcentral gyrus	-0.273	0.106	[-0.480 - -0.066]	9.849E-03	8.124E-02
Left medial orbitofrontal cortex	-0.140	0.080	[-0.297 - 0.017]	8.056E-02	1.787E-01
Right medial orbitofrontal cortex	-0.206	0.080	[-0.363 - -0.049]	1.030E-02	8.124E-02
Left cuneus	-0.166	0.115	[-0.391 - 0.059]	1.487E-01	2.651E-01
Right cuneus	-0.262	0.097	[-0.452 - -0.072]	6.754E-03	8.124E-02
Left pars triangularis of inferior frontal gyrus	-0.206	0.088	[-0.378 - -0.034]	1.893E-02	8.962E-02
Right pars triangularis of inferior frontal gyrus	-0.198	0.092	[-0.378 - -0.018]	3.080E-02	9.941E-02
Left superior parietal cortex	-0.066	0.085	[-0.234 - 0.101]	4.374E-01	5.593E-01
Right superior parietal cortex	-0.115	0.107	[-0.326 - 0.095]	2.834E-01	4.375E-01
Left pars opercularis of inferior frontal gyrus	-0.028	0.134	[-0.292 - 0.235]	8.328E-01	8.569E-01
Right pars opercularis of inferior frontal gyrus	-0.048	0.093	[-0.229 - 0.134]	6.053E-01	6.612E-01
Left supramarginal gyrus	-0.092	0.114	[-0.315 - 0.132]	4.222E-01	5.593E-01
Right supramarginal gyrus	-0.185	0.090	[-0.361 - -0.009]	3.913E-02	1.158E-01
Left pericalcarine cortex	-0.256	0.098	[-0.448 - -0.065]	8.749E-03	8.124E-02
Right pericalcarine cortex	-0.316	0.090	[-0.493 - -0.139]	4.613E-04	3.275E-02
Left parahippocampal gyrus	-0.056	0.089	[-0.230 - 0.118]	5.274E-01	6.138E-01
Right parahippocampal gyrus	-0.105	0.092	[-0.284 - 0.075]	2.528E-01	4.226E-01
Left caudal middle frontal gyrus	-0.148	0.080	[-0.304 - 0.009]	6.493E-02	1.549E-01
Right caudal middle frontal gyrus	-0.052	0.094	[-0.236 - 0.132]	5.796E-01	6.430E-01
Left transverse temporal gyrus	-0.089	0.080	[-0.245 - 0.068]	2.678E-01	4.226E-01
Right transverse temporal gyrus	-0.174	0.080	[-0.331 - -0.017]	2.938E-02	9.941E-02
Left banks of superior temporal sulcus	-0.121	0.098	[-0.314 - 0.072]	2.185E-01	3.784E-01
Right banks of superior temporal sulcus	-0.079	0.132	[-0.336 - 0.179]	5.502E-01	6.201E-01
Left caudal anterior cingulate cortex	-0.125	0.080	[-0.281 - 0.032]	1.190E-01	2.283E-01
Right caudal anterior cingulate cortex	-0.061	0.080	[-0.217 - 0.096]	4.490E-01	5.593E-01
Left rostral anterior cingulate cortex	-0.032	0.080	[-0.189 - 0.124]	6.868E-01	7.340E-01
Right rostral anterior cingulate cortex	0.012	0.080	[-0.145 - 0.169]	8.816E-01	8.816E-01
Left posterior cingulate cortex	-0.029	0.080	[-0.186 - 0.127]	7.124E-01	7.438E-01
Right posterior cingulate cortex	-0.162	0.086	[-0.331 - 0.007]	5.991E-02	1.519E-01
Left frontal pole	-0.159	0.080	[-0.316 - -0.002]	4.688E-02	1.241E-01
Right frontal pole	-0.213	0.080	[-0.370 - -0.056]	7.725E-03	8.124E-02
Left paracentral lobule	-0.055	0.080	[-0.212 - 0.101]	4.884E-01	5.970E-01
Right paracentral lobule	-0.115	0.080	[-0.272 - 0.041]	1.494E-01	2.651E-01
Left insula	-0.085	0.090	[-0.260 - 0.091]	3.450E-01	4.711E-01
Right insula	-0.081	0.106	[-0.288 - 0.126]	4.434E-01	5.593E-01
Left entorhinal cortex	0.070	0.107	[-0.141 - 0.280]	5.164E-01	6.110E-01
Right entorhinal cortex	0.101	0.101	[-0.097 - 0.299]	3.164E-01	4.491E-01
Left temporal pole	-0.126	0.113	[-0.348 - 0.095]	2.645E-01	4.226E-01
Right temporal pole	-0.079	0.080	[-0.236 - 0.078]	3.226E-01	4.491E-01
Left isthmus cingulate cortex	-0.082	0.080	[-0.239 - 0.075]	3.051E-01	4.491E-01
Right isthmus cingulate cortex	-0.015	0.093	[-0.196 - 0.167]	8.728E-01	8.816E-01

Supplementary Table S107. Cortical surface area differences between individuals with first episode major depressive disorder versus individuals with recurrent episode major depressive disorder controlling for age and sex

	d	Std. Err.	95% CI	*p*-value	FDR *q*-value
Total cortical surface area	0.160	0.092	[-0.021 - 0.341]	8.280E-02	4.842E-01
Left hemisphere	0.163	0.092	[-0.019 - 0.344]	7.847E-02	4.842E-01
Right hemisphere	0.157	0.092	[-0.024 - 0.339]	8.865E-02	4.842E-01
Left superior frontal gyrus	0.154	0.092	[-0.027 - 0.335]	9.563E-02	4.850E-01
Right superior frontal gyrus	0.127	0.092	[-0.054 - 0.307]	1.701E-01	6.016E-01
Left pars orbitalis of inferior frontal gyrus	0.122	0.092	[-0.059 - 0.302]	1.873E-01	6.016E-01
Right pars orbitalis of inferior frontal gyrus	0.068	0.092	[-0.112 - 0.249]	4.589E-01	7.405E-01
Left precentral gyrus	0.220	0.092	[0.039 - 0.401]	1.734E-02	2.890E-01
Right precentral gyrus	0.075	0.092	[-0.105 - 0.256]	4.136E-01	6.830E-01
Left lingual gyrus	0.231	0.093	[0.049 - 0.412]	1.274E-02	2.890E-01
Right lingual gyrus	0.185	0.103	[-0.017 - 0.386]	7.241E-02	4.842E-01
Left superior temporal gyrus	0.059	0.092	[-0.121 - 0.240]	5.195E-01	7.848E-01
Right superior temporal gyrus	0.011	0.094	[-0.172 - 0.195]	9.047E-01	9.732E-01
Left fusiform gyrus	0.047	0.092	[-0.134 - 0.228]	6.091E-01	8.167E-01
Right fusiform gyrus	0.088	0.106	[-0.121 - 0.296]	4.096E-01	6.830E-01
Left inferior parietal cortex	0.198	0.092	[0.017 - 0.379]	3.248E-02	3.294E-01
Right inferior parietal cortex	0.065	0.141	[-0.211 - 0.341]	6.443E-01	8.421E-01
Left lateral occipital cortex	0.004	0.116	[-0.223 - 0.231]	9.739E-01	9.946E-01
Right lateral occipital cortex	0.103	0.092	[-0.078 - 0.284]	2.636E-01	6.016E-01
Left rostral middle frontal gyrus	0.134	0.118	[-0.097 - 0.365]	2.556E-01	6.016E-01
Right rostral middle frontal gyrus	0.127	0.122	[-0.112 - 0.367]	2.976E-01	6.016E-01
Left precuneus	0.008	0.101	[-0.189 - 0.205]	9.402E-01	9.946E-01
Right precuneus	0.090	0.092	[-0.091 - 0.271]	3.304E-01	6.016E-01
Left inferior temporal gyrus	0.213	0.092	[0.033 - 0.394]	2.068E-02	2.890E-01
Right inferior temporal gyrus	0.062	0.092	[-0.119 - 0.242]	5.036E-01	7.772E-01
Left lateral orbitofrontal cortex	0.084	0.092	[-0.097 - 0.264]	3.642E-01	6.465E-01
Right lateral orbitofrontal cortex	0.033	0.092	[-0.148 - 0.213]	7.235E-01	8.421E-01
Left middle temporal gyrus	0.105	0.092	[-0.076 - 0.286]	2.558E-01	6.016E-01
Right middle temporal gyrus	0.101	0.092	[-0.080 - 0.282]	2.739E-01	6.016E-01
Left postcentral gyrus	0.095	0.092	[-0.086 - 0.275]	3.051E-01	6.016E-01
Right postcentral gyrus	0.226	0.092	[0.045 - 0.407]	1.422E-02	2.890E-01
Left medial orbitofrontal cortex	0.033	0.092	[-0.148 - 0.214]	7.229E-01	8.421E-01
Right medial orbitofrontal cortex	0.119	0.092	[-0.062 - 0.299]	1.984E-01	6.016E-01
Left cuneus	0.014	0.092	[-0.166 - 0.195]	8.760E-01	9.568E-01
Right cuneus	0.130	0.128	[-0.121 - 0.380]	3.101E-01	6.016E-01
Left pars triangularis of inferior frontal gyrus	0.114	0.092	[-0.066 - 0.295]	2.148E-01	6.016E-01
Right pars triangularis of inferior frontal gyrus	0.056	0.092	[-0.125 - 0.236]	5.445E-01	8.052E-01
Left superior parietal cortex	0.040	0.092	[-0.140 - 0.221]	6.609E-01	8.421E-01
Right superior parietal cortex	0.050	0.129	[-0.204 - 0.303]	7.004E-01	8.421E-01
Left pars opercularis of inferior frontal gyrus	0.002	0.104	[-0.202 - 0.207]	9.825E-01	9.946E-01
Right pars opercularis of inferior frontal gyrus	0.041	0.109	[-0.173 - 0.256]	7.050E-01	8.421E-01
Left supramarginal gyrus	0.034	0.097	[-0.156 - 0.225]	7.233E-01	8.421E-01
Right supramarginal gyrus	0.110	0.092	[-0.071 - 0.290]	2.354E-01	6.016E-01
Left pericalcarine cortex	0.235	0.093	[0.054 - 0.417]	1.096E-02	2.890E-01
Right pericalcarine cortex	0.115	0.092	[-0.066 - 0.295]	2.145E-01	6.016E-01
Left parahippocampal gyrus	0.004	0.105	[-0.202 - 0.210]	9.677E-01	9.946E-01
Right parahippocampal gyrus	-0.025	0.104	[-0.229 - 0.179]	8.117E-01	9.208E-01
Left caudal middle frontal gyrus	0.133	0.092	[-0.047 - 0.314]	1.479E-01	6.016E-01
Right caudal middle frontal gyrus	0.122	0.092	[-0.059 - 0.303]	1.873E-01	6.016E-01
Left transverse temporal gyrus	-0.059	0.116	[-0.287 - 0.168]	6.097E-01	8.167E-01
Right transverse temporal gyrus	0.139	0.092	[-0.042 - 0.320]	1.324E-01	6.016E-01
Left banks of superior temporal sulcus	0.090	0.092	[-0.091 - 0.270]	3.296E-01	6.016E-01
Right banks of superior temporal sulcus	0.064	0.112	[-0.155 - 0.284]	5.671E-01	8.052E-01
Left caudal anterior cingulate cortex	0.071	0.132	[-0.188 - 0.331]	5.898E-01	8.167E-01
Right caudal anterior cingulate cortex	-0.107	0.092	[-0.287 - 0.074]	2.482E-01	6.016E-01
Left rostral anterior cingulate cortex	-0.056	0.147	[-0.343 - 0.232]	7.041E-01	8.421E-01
Right rostral anterior cingulate cortex	-0.032	0.150	[-0.326 - 0.262]	8.300E-01	9.208E-01
Left posterior cingulate cortex	0.053	0.092	[-0.127 - 0.234]	5.631E-01	8.052E-01
Right posterior cingulate cortex	-0.023	0.100	[-0.218 - 0.173]	8.206E-01	9.208E-01
Left frontal pole	0.093	0.092	[-0.088 - 0.274]	3.137E-01	6.016E-01
Right frontal pole	0.220	0.098	[0.028 - 0.412]	2.442E-02	2.890E-01
Left paracentral lobule	0.110	0.092	[-0.071 - 0.291]	2.331E-01	6.016E-01
Right paracentral lobule	0.126	0.092	[-0.056 - 0.307]	1.742E-01	6.016E-01
Left insula	0.098	0.092	[-0.083 - 0.279]	2.889E-01	6.016E-01
Right insula	-0.001	0.092	[-0.181 - 0.180]	9.946E-01	9.946E-01
Left entorhinal cortex	-0.202	0.102	[-0.402 - -0.002]	4.809E-02	4.268E-01
Right entorhinal cortex	-0.067	0.092	[-0.247 - 0.114]	4.705E-01	7.424E-01
Left temporal pole	0.128	0.146	[-0.158 - 0.413]	3.816E-01	6.608E-01
Right temporal pole	0.096	0.092	[-0.084 - 0.277]	2.959E-01	6.016E-01
Left isthmus cingulate cortex	0.162	0.092	[-0.019 - 0.343]	7.938E-02	4.842E-01
Right isthmus cingulate cortex	0.134	0.092	[-0.046 - 0.315]	1.449E-01	6.016E-01

Supplementary Table S108. Cortical surface area differences between individuals with first episode major depressive disorder versus individuals with recurrent episode major depressive disorder controlling for age and sex over 21 years of old

	d	Std. Err.	95% CI	*p*-value	FDR *q*-value
Total cortical surface area	0.159	0.094	[-0.026 - 0.343]	9.260E-02	5.154E-01
Left hemisphere	0.162	0.094	[-0.023 - 0.347]	8.525E-02	5.154E-01
Right hemisphere	0.154	0.094	[-0.030 - 0.339]	1.016E-01	5.154E-01
Left superior frontal gyrus	0.157	0.094	[-0.028 - 0.342]	9.578E-02	5.154E-01
Right superior frontal gyrus	0.104	0.094	[-0.080 - 0.289]	2.675E-01	6.453E-01
Left pars orbitalis of inferior frontal gyrus	0.141	0.094	[-0.043 - 0.326]	1.328E-01	5.650E-01
Right pars orbitalis of inferior frontal gyrus	0.072	0.094	[-0.112 - 0.257]	4.431E-01	7.452E-01
Left precentral gyrus	0.205	0.094	[0.021 - 0.390]	2.943E-02	3.345E-01
Right precentral gyrus	0.055	0.094	[-0.129 - 0.239]	5.581E-01	7.480E-01
Left lingual gyrus	0.244	0.098	[0.051 - 0.437]	1.320E-02	3.125E-01
Right lingual gyrus	0.218	0.102	[0.018 - 0.418]	3.297E-02	3.345E-01
Left superior temporal gyrus	0.067	0.094	[-0.117 - 0.252]	4.742E-01	7.452E-01
Right superior temporal gyrus	0.022	0.094	[-0.163 - 0.206]	8.179E-01	9.000E-01
Left fusiform gyrus	0.047	0.094	[-0.138 - 0.231]	6.195E-01	7.951E-01
Right fusiform gyrus	0.080	0.102	[-0.120 - 0.281]	4.308E-01	7.452E-01
Left inferior parietal cortex	0.219	0.094	[0.034 - 0.404]	2.053E-02	3.304E-01
Right inferior parietal cortex	0.073	0.133	[-0.188 - 0.334]	5.834E-01	7.671E-01
Left lateral occipital cortex	0.011	0.112	[-0.208 - 0.230]	9.219E-01	9.486E-01
Right lateral occipital cortex	0.094	0.094	[-0.090 - 0.279]	3.163E-01	6.453E-01
Left rostral middle frontal gyrus	0.125	0.111	[-0.093 - 0.344]	2.617E-01	6.453E-01
Right rostral middle frontal gyrus	0.118	0.118	[-0.113 - 0.348]	3.160E-01	6.453E-01
Left precuneus	0.004	0.097	[-0.186 - 0.194]	9.665E-01	9.665E-01
Right precuneus	0.085	0.094	[-0.100 - 0.269]	3.675E-01	6.524E-01
Left inferior temporal gyrus	0.214	0.094	[0.029 - 0.398]	2.327E-02	3.304E-01
Right inferior temporal gyrus	0.061	0.094	[-0.123 - 0.245]	5.181E-01	7.474E-01
Left lateral orbitofrontal cortex	0.091	0.094	[-0.093 - 0.276]	3.304E-01	6.453E-01
Right lateral orbitofrontal cortex	0.044	0.094	[-0.140 - 0.229]	6.371E-01	7.951E-01
Left middle temporal gyrus	0.096	0.094	[-0.088 - 0.281]	3.061E-01	6.453E-01
Right middle temporal gyrus	0.089	0.094	[-0.096 - 0.273]	3.454E-01	6.453E-01
Left postcentral gyrus	0.087	0.094	[-0.097 - 0.271]	3.551E-01	6.464E-01
Right postcentral gyrus	0.241	0.094	[0.056 - 0.425]	1.065E-02	3.125E-01
Left medial orbitofrontal cortex	0.042	0.096	[-0.147 - 0.231]	6.607E-01	7.951E-01
Right medial orbitofrontal cortex	0.125	0.094	[-0.059 - 0.309]	1.836E-01	5.924E-01
Left cuneus	0.021	0.094	[-0.164 - 0.205]	8.239E-01	9.000E-01
Right cuneus	0.128	0.125	[-0.116 - 0.372]	3.026E-01	6.453E-01
Left pars triangularis of inferior frontal gyrus	0.127	0.094	[-0.058 - 0.311]	1.788E-01	5.924E-01
Right pars triangularis of inferior frontal gyrus	0.068	0.094	[-0.116 - 0.252]	4.682E-01	7.452E-01
Left superior parietal cortex	0.043	0.094	[-0.142 - 0.227]	6.500E-01	7.951E-01
Right superior parietal cortex	0.072	0.122	[-0.168 - 0.311]	5.584E-01	7.480E-01
Left pars opercularis of inferior frontal gyrus	0.026	0.094	[-0.158 - 0.211]	7.793E-01	8.782E-01
Right pars opercularis of inferior frontal gyrus	0.072	0.099	[-0.122 - 0.267]	4.654E-01	7.452E-01
Left supramarginal gyrus	0.060	0.094	[-0.125 - 0.244]	5.263E-01	7.474E-01
Right supramarginal gyrus	0.130	0.094	[-0.055 - 0.315]	1.679E-01	5.924E-01
Left pericalcarine cortex	0.253	0.094	[0.068 - 0.438]	7.436E-03	3.125E-01
Right pericalcarine cortex	0.147	0.094	[-0.038 - 0.331]	1.198E-01	5.650E-01
Left parahippocampal gyrus	-0.012	0.103	[-0.214 - 0.189]	9.036E-01	9.434E-01
Right parahippocampal gyrus	-0.019	0.103	[-0.221 - 0.182]	8.495E-01	9.138E-01
Left caudal middle frontal gyrus	0.138	0.094	[-0.046 - 0.322]	1.413E-01	5.650E-01
Right caudal middle frontal gyrus	0.109	0.094	[-0.076 - 0.293]	2.471E-01	6.453E-01
Left transverse temporal gyrus	-0.043	0.115	[-0.269 - 0.183]	7.080E-01	8.339E-01
Right transverse temporal gyrus	0.131	0.094	[-0.054 - 0.316]	1.641E-01	5.924E-01
Left banks of superior temporal sulcus	0.091	0.094	[-0.093 - 0.275]	3.315E-01	6.453E-01
Right banks of superior temporal sulcus	0.086	0.125	[-0.159 - 0.331]	4.912E-01	7.452E-01
Left caudal anterior cingulate cortex	0.078	0.126	[-0.170 - 0.325]	5.380E-01	7.480E-01
Right caudal anterior cingulate cortex	-0.093	0.094	[-0.278 - 0.091]	3.213E-01	6.453E-01
Left rostral anterior cingulate cortex	-0.051	0.141	[-0.328 - 0.225]	7.164E-01	8.339E-01
Right rostral anterior cingulate cortex	-0.008	0.153	[-0.308 - 0.293]	9.606E-01	9.665E-01
Left posterior cingulate cortex	0.043	0.094	[-0.141 - 0.227]	6.472E-01	7.951E-01
Right posterior cingulate cortex	-0.032	0.100	[-0.229 - 0.164]	7.455E-01	8.537E-01
Left frontal pole	0.063	0.094	[-0.121 - 0.247]	5.038E-01	7.452E-01
Right frontal pole	0.212	0.102	[0.012 - 0.411]	3.775E-02	3.350E-01
Left paracentral lobule	0.097	0.095	[-0.089 - 0.283]	3.080E-01	6.453E-01
Right paracentral lobule	0.100	0.094	[-0.085 - 0.285]	2.883E-01	6.453E-01
Left insula	0.097	0.094	[-0.087 - 0.282]	3.025E-01	6.453E-01
Right insula	-0.012	0.094	[-0.196 - 0.172]	9.011E-01	9.434E-01
Left entorhinal cortex	-0.224	0.117	[-0.454 - 0.006]	5.616E-02	4.430E-01
Right entorhinal cortex	-0.063	0.094	[-0.247 - 0.121]	5.031E-01	7.452E-01
Left temporal pole	0.143	0.151	[-0.154 - 0.439]	3.454E-01	6.453E-01
Right temporal pole	0.099	0.094	[-0.086 - 0.283]	2.942E-01	6.453E-01
Left isthmus cingulate cortex	0.170	0.094	[-0.014 - 0.355]	7.071E-02	5.021E-01
Right isthmus cingulate cortex	0.138	0.094	[-0.047 - 0.322]	1.432E-01	5.650E-01

Supplementary Table S109. Partial correlations between cortical surface area and number of episodes controlling for age and sex in individuals with major depressive disorder

	Partial *R*	Std. Err.	95% CI	*p*-value	FDR *q*-value
Total cortical surface area	-0.085	0.067	[-0.217 - 0.046]	2.031E-01	8.129E-01
Left hemisphere	-0.080	0.067	[-0.212 - 0.051]	2.319E-01	8.129E-01
Right hemisphere	-0.089	0.067	[-0.221 - 0.042]	1.838E-01	8.129E-01
Left superior frontal gyrus	-0.072	0.067	[-0.204 - 0.059]	2.805E-01	8.129E-01
Right superior frontal gyrus	-0.083	0.067	[-0.214 - 0.049]	2.182E-01	8.129E-01
Left pars orbitalis of inferior frontal gyrus	-0.010	0.067	[-0.142 - 0.121]	8.785E-01	9.466E-01
Right pars orbitalis of inferior frontal gyrus	-0.058	0.095	[-0.245 - 0.128]	5.388E-01	9.319E-01
Left precentral gyrus	-0.020	0.067	[-0.151 - 0.112]	7.692E-01	9.466E-01
Right precentral gyrus	-0.077	0.072	[-0.217 - 0.064]	2.862E-01	8.129E-01
Left lingual gyrus	-0.005	0.067	[-0.137 - 0.126]	9.396E-01	9.621E-01
Right lingual gyrus	-0.043	0.072	[-0.185 - 0.098]	5.484E-01	9.319E-01
Left superior temporal gyrus	-0.085	0.067	[-0.216 - 0.047]	2.067E-01	8.129E-01
Right superior temporal gyrus	-0.128	0.067	[-0.260 - 0.003]	5.617E-02	8.129E-01
Left fusiform gyrus	-0.043	0.089	[-0.218 - 0.132]	6.279E-01	9.319E-01
Right fusiform gyrus	0.012	0.067	[-0.120 - 0.143]	8.613E-01	9.466E-01
Left inferior parietal cortex	-0.028	0.067	[-0.160 - 0.103]	6.735E-01	9.319E-01
Right inferior parietal cortex	-0.013	0.073	[-0.157 - 0.131]	8.572E-01	9.466E-01
Left lateral occipital cortex	-0.041	0.067	[-0.173 - 0.090]	5.405E-01	9.319E-01
Right lateral occipital cortex	-0.046	0.078	[-0.199 - 0.107]	5.538E-01	9.319E-01
Left rostral middle frontal gyrus	-0.080	0.067	[-0.211 - 0.052]	2.347E-01	8.129E-01
Right rostral middle frontal gyrus	-0.081	0.067	[-0.212 - 0.051]	2.282E-01	8.129E-01
Left precuneus	-0.043	0.068	[-0.176 - 0.090]	5.301E-01	9.319E-01
Right precuneus	-0.093	0.078	[-0.246 - 0.060]	2.348E-01	8.129E-01
Left inferior temporal gyrus	-0.037	0.067	[-0.168 - 0.095]	5.832E-01	9.319E-01
Right inferior temporal gyrus	0.036	0.067	[-0.096 - 0.167]	5.946E-01	9.319E-01
Left lateral orbitofrontal cortex	-0.108	0.067	[-0.240 - 0.023]	1.065E-01	8.129E-01
Right lateral orbitofrontal cortex	-0.116	0.067	[-0.247 - 0.016]	8.412E-02	8.129E-01
Left middle temporal gyrus	-0.005	0.067	[-0.136 - 0.127]	9.455E-01	9.621E-01
Right middle temporal gyrus	0.011	0.070	[-0.127 - 0.148]	8.799E-01	9.466E-01
Left postcentral gyrus	0.003	0.067	[-0.128 - 0.135]	9.621E-01	9.621E-01
Right postcentral gyrus	-0.029	0.067	[-0.161 - 0.102]	6.636E-01	9.319E-01
Left medial orbitofrontal cortex	-0.032	0.089	[-0.206 - 0.143]	7.221E-01	9.321E-01
Right medial orbitofrontal cortex	-0.056	0.077	[-0.208 - 0.095]	4.639E-01	9.319E-01
Left cuneus	-0.110	0.067	[-0.242 - 0.021]	1.002E-01	8.129E-01
Right cuneus	-0.087	0.070	[-0.224 - 0.051]	2.160E-01	8.129E-01
Left pars triangularis of inferior frontal gyrus	-0.057	0.067	[-0.189 - 0.074]	3.944E-01	8.888E-01
Right pars triangularis of inferior frontal gyrus	-0.125	0.067	[-0.257 - 0.006]	6.204E-02	8.129E-01
Left superior parietal cortex	-0.115	0.067	[-0.246 - 0.017]	8.681E-02	8.129E-01
Right superior parietal cortex	-0.105	0.068	[-0.239 - 0.029]	1.242E-01	8.129E-01
Left pars opercularis of inferior frontal gyrus	-0.015	0.067	[-0.147 - 0.117]	8.236E-01	9.466E-01
Right pars opercularis of inferior frontal gyrus	-0.057	0.067	[-0.188 - 0.075]	3.991E-01	8.888E-01
Left supramarginal gyrus	-0.080	0.082	[-0.241 - 0.081]	3.310E-01	8.704E-01
Right supramarginal gyrus	-0.049	0.067	[-0.180 - 0.083]	4.684E-01	9.319E-01
Left pericalcarine cortex	-0.050	0.067	[-0.181 - 0.082]	4.594E-01	9.319E-01
Right pericalcarine cortex	-0.061	0.067	[-0.193 - 0.070]	3.614E-01	8.888E-01
Left parahippocampal gyrus	-0.074	0.067	[-0.206 - 0.057]	2.680E-01	8.129E-01
Right parahippocampal gyrus	-0.012	0.067	[-0.143 - 0.120]	8.603E-01	9.466E-01
Left caudal middle frontal gyrus	-0.019	0.067	[-0.151 - 0.112]	7.739E-01	9.466E-01
Right caudal middle frontal gyrus	-0.076	0.067	[-0.207 - 0.056]	2.590E-01	8.129E-01
Left transverse temporal gyrus	-0.123	0.067	[-0.254 - 0.009]	6.713E-02	8.129E-01
Right transverse temporal gyrus	-0.033	0.067	[-0.164 - 0.099]	6.270E-01	9.319E-01
Left banks of superior temporal sulcus	-0.017	0.067	[-0.148 - 0.115]	8.015E-01	9.466E-01
Right banks of superior temporal sulcus	-0.066	0.067	[-0.198 - 0.065]	3.241E-01	8.704E-01
Left caudal anterior cingulate cortex	-0.025	0.067	[-0.156 - 0.107]	7.117E-01	9.321E-01
Right caudal anterior cingulate cortex	0.036	0.075	[-0.112 - 0.184]	6.345E-01	9.319E-01
Left rostral anterior cingulate cortex	-0.013	0.067	[-0.145 - 0.119]	8.469E-01	9.466E-01
Right rostral anterior cingulate cortex	0.006	0.089	[-0.169 - 0.180]	9.489E-01	9.621E-01
Left posterior cingulate cortex	0.025	0.067	[-0.107 - 0.156]	7.130E-01	9.321E-01
Right posterior cingulate cortex	-0.054	0.086	[-0.223 - 0.115]	5.301E-01	9.319E-01
Left frontal pole	0.027	0.067	[-0.104 - 0.159]	6.825E-01	9.319E-01
Right frontal pole	0.071	0.084	[-0.094 - 0.236]	4.006E-01	8.888E-01
Left paracentral lobule	-0.057	0.067	[-0.189 - 0.074]	3.939E-01	8.888E-01
Right paracentral lobule	-0.101	0.067	[-0.233 - 0.030]	1.309E-01	8.129E-01
Left insula	-0.086	0.067	[-0.217 - 0.046]	2.022E-01	8.129E-01
Right insula	-0.108	0.067	[-0.240 - 0.023]	1.070E-01	8.129E-01
Left entorhinal cortex	0.049	0.107	[-0.161 - 0.258]	6.495E-01	9.319E-01
Right entorhinal cortex	0.019	0.067	[-0.113 - 0.150]	7.778E-01	9.466E-01
Left temporal pole	-0.039	0.071	[-0.179 - 0.100]	5.799E-01	9.319E-01
Right temporal pole	-0.032	0.067	[-0.164 - 0.099]	6.308E-01	9.319E-01
Left isthmus cingulate cortex	0.005	0.067	[-0.126 - 0.137]	9.386E-01	9.621E-01
Right isthmus cingulate cortex	-0.072	0.067	[-0.204 - 0.060]	2.833E-01	8.129E-01

Supplementary Table S110. Partial correlations between cortical surface area and number of episodes controlling for age and sex in individuals with major depressive disorder over 21 years of old

	Partial *R*	Std. Err.	95% CI	*p*-value	FDR *q*-value
Total cortical surface area	-0.098	0.068	[-0.231 - 0.034]	1.467E-01	6.305E-01
Left hemisphere	-0.093	0.068	[-0.225 - 0.040]	1.697E-01	6.305E-01
Right hemisphere	-0.102	0.068	[-0.234 - 0.031]	1.319E-01	6.305E-01
Left superior frontal gyrus	-0.084	0.068	[-0.216 - 0.049]	2.163E-01	6.678E-01
Right superior frontal gyrus	-0.093	0.068	[-0.225 - 0.040]	1.692E-01	6.305E-01
Left pars orbitalis of inferior frontal gyrus	-0.012	0.068	[-0.145 - 0.120]	8.581E-01	9.561E-01
Right pars orbitalis of inferior frontal gyrus	-0.065	0.099	[-0.259 - 0.129]	5.097E-01	8.417E-01
Left precentral gyrus	-0.034	0.068	[-0.167 - 0.098]	6.120E-01	8.910E-01
Right precentral gyrus	-0.084	0.074	[-0.228 - 0.060]	2.541E-01	7.216E-01
Left lingual gyrus	-0.009	0.068	[-0.142 - 0.123]	8.887E-01	9.561E-01
Right lingual gyrus	-0.045	0.072	[-0.187 - 0.097]	5.318E-01	8.581E-01
Left superior temporal gyrus	-0.094	0.068	[-0.227 - 0.038]	1.634E-01	6.305E-01
Right superior temporal gyrus	-0.137	0.068	[-0.269 - -0.004]	4.303E-02	6.305E-01
Left fusiform gyrus	-0.044	0.090	[-0.220 - 0.131]	6.199E-01	8.910E-01
Right fusiform gyrus	-0.001	0.068	[-0.134 - 0.131]	9.848E-01	9.848E-01
Left inferior parietal cortex	-0.029	0.068	[-0.161 - 0.104]	6.700E-01	8.910E-01
Right inferior parietal cortex	-0.019	0.075	[-0.167 - 0.128]	7.981E-01	9.289E-01
Left lateral occipital cortex	-0.049	0.068	[-0.183 - 0.085]	4.719E-01	8.310E-01
Right lateral occipital cortex	-0.056	0.079	[-0.211 - 0.098]	4.742E-01	8.310E-01
Left rostral middle frontal gyrus	-0.099	0.068	[-0.231 - 0.034]	1.446E-01	6.305E-01
Right rostral middle frontal gyrus	-0.093	0.068	[-0.226 - 0.039]	1.670E-01	6.305E-01
Left precuneus	-0.057	0.068	[-0.189 - 0.076]	4.018E-01	8.310E-01
Right precuneus	-0.101	0.074	[-0.247 - 0.045]	1.753E-01	6.305E-01
Left inferior temporal gyrus	-0.047	0.068	[-0.179 - 0.085]	4.869E-01	8.310E-01
Right inferior temporal gyrus	0.027	0.068	[-0.106 - 0.159]	6.909E-01	8.910E-01
Left lateral orbitofrontal cortex	-0.118	0.068	[-0.250 - 0.015]	8.111E-02	6.305E-01
Right lateral orbitofrontal cortex	-0.128	0.068	[-0.261 - 0.004]	5.768E-02	6.305E-01
Left middle temporal gyrus	-0.010	0.068	[-0.142 - 0.123]	8.861E-01	9.561E-01
Right middle temporal gyrus	0.006	0.070	[-0.132 - 0.143]	9.371E-01	9.664E-01
Left postcentral gyrus	0.003	0.068	[-0.129 - 0.135]	9.640E-01	9.778E-01
Right postcentral gyrus	-0.033	0.068	[-0.165 - 0.100]	6.289E-01	8.910E-01
Left medial orbitofrontal cortex	-0.036	0.087	[-0.207 - 0.135]	6.821E-01	8.910E-01
Right medial orbitofrontal cortex	-0.064	0.072	[-0.206 - 0.078]	3.783E-01	8.310E-01
Left cuneus	-0.119	0.068	[-0.252 - 0.013]	7.724E-02	6.305E-01
Right cuneus	-0.098	0.072	[-0.240 - 0.044]	1.776E-01	6.305E-01
Left pars triangularis of inferior frontal gyrus	-0.059	0.068	[-0.191 - 0.074]	3.862E-01	8.310E-01
Right pars triangularis of inferior frontal gyrus	-0.125	0.068	[-0.258 - 0.007]	6.405E-02	6.305E-01
Left superior parietal cortex	-0.118	0.068	[-0.250 - 0.015]	8.139E-02	6.305E-01
Right superior parietal cortex	-0.106	0.069	[-0.241 - 0.029]	1.240E-01	6.305E-01
Left pars opercularis of inferior frontal gyrus	-0.017	0.068	[-0.150 - 0.115]	7.979E-01	9.289E-01
Right pars opercularis of inferior frontal gyrus	-0.062	0.068	[-0.195 - 0.070]	3.559E-01	8.310E-01
Left supramarginal gyrus	-0.086	0.081	[-0.246 - 0.073]	2.878E-01	7.860E-01
Right supramarginal gyrus	-0.048	0.068	[-0.181 - 0.084]	4.763E-01	8.310E-01
Left pericalcarine cortex	-0.054	0.068	[-0.186 - 0.079]	4.264E-01	8.310E-01
Right pericalcarine cortex	-0.064	0.068	[-0.196 - 0.069]	3.443E-01	8.310E-01
Left parahippocampal gyrus	-0.087	0.068	[-0.219 - 0.046]	1.988E-01	6.415E-01
Right parahippocampal gyrus	-0.022	0.068	[-0.154 - 0.111]	7.472E-01	9.146E-01
Left caudal middle frontal gyrus	-0.026	0.068	[-0.158 - 0.107]	7.028E-01	8.910E-01
Right caudal middle frontal gyrus	-0.088	0.068	[-0.221 - 0.044]	1.917E-01	6.415E-01
Left transverse temporal gyrus	-0.135	0.068	[-0.267 - -0.002]	4.600E-02	6.305E-01
Right transverse temporal gyrus	-0.052	0.068	[-0.185 - 0.080]	4.408E-01	8.310E-01
Left banks of superior temporal sulcus	-0.024	0.068	[-0.157 - 0.108]	7.177E-01	8.940E-01
Right banks of superior temporal sulcus	-0.069	0.068	[-0.202 - 0.063]	3.053E-01	8.029E-01
Left caudal anterior cingulate cortex	-0.034	0.068	[-0.166 - 0.099]	6.176E-01	8.910E-01
Right caudal anterior cingulate cortex	0.031	0.075	[-0.115 - 0.178]	6.733E-01	8.910E-01
Left rostral anterior cingulate cortex	-0.028	0.068	[-0.160 - 0.104]	6.786E-01	8.910E-01
Right rostral anterior cingulate cortex	0.007	0.089	[-0.168 - 0.182]	9.392E-01	9.664E-01
Left posterior cingulate cortex	0.011	0.068	[-0.122 - 0.143]	8.729E-01	9.561E-01
Right posterior cingulate cortex	-0.059	0.085	[-0.226 - 0.109]	4.916E-01	8.310E-01
Left frontal pole	0.018	0.068	[-0.115 - 0.150]	7.949E-01	9.289E-01
Right frontal pole	0.065	0.087	[-0.105 - 0.236]	4.511E-01	8.310E-01
Left paracentral lobule	-0.066	0.068	[-0.199 - 0.066]	3.265E-01	8.279E-01
Right paracentral lobule	-0.112	0.068	[-0.245 - 0.020]	9.617E-02	6.305E-01
Left insula	-0.092	0.068	[-0.225 - 0.040]	1.727E-01	6.305E-01
Right insula	-0.112	0.068	[-0.245 - 0.020]	9.684E-02	6.305E-01
Left entorhinal cortex	0.042	0.108	[-0.169 - 0.253]	6.949E-01	8.910E-01
Right entorhinal cortex	0.014	0.068	[-0.119 - 0.146]	8.388E-01	9.561E-01
Left temporal pole	-0.047	0.068	[-0.179 - 0.086]	4.902E-01	8.310E-01
Right temporal pole	-0.030	0.068	[-0.162 - 0.103]	6.621E-01	8.910E-01
Left isthmus cingulate cortex	0.007	0.068	[-0.126 - 0.139]	9.187E-01	9.664E-01
Right isthmus cingulate cortex	-0.079	0.068	[-0.212 - 0.053]	2.412E-01	7.136E-01

Supplementary Table S111. Cortical thickness differences between individuals with schizophrenia who are unmedicated with antipsychotics and healthy comparison subjects controlling for age and sex

	d	Std. Err.	95% CI	*p*-value	FDR *q*-value
Global mean cortical thickness	-0.157	0.203	[-0.555 - 0.241]	4.386E-01	8.916E-01
Left hemisphere	-0.143	0.195	[-0.526 - 0.240]	4.646E-01	8.916E-01
Right hemisphere	-0.162	0.204	[-0.563 - 0.238]	4.269E-01	8.916E-01
Left fusiform gyrus	-0.094	0.182	[-0.451 - 0.263]	6.043E-01	8.916E-01
Right fusiform gyrus	-0.087	0.155	[-0.391 - 0.217]	5.753E-01	8.916E-01
Left pars opercularis of inferior frontal gyrus	-0.158	0.164	[-0.480 - 0.164]	3.360E-01	8.916E-01
Right pars opercularis of inferior frontal gyrus	-0.135	0.190	[-0.508 - 0.238]	4.784E-01	8.916E-01
Left superior temporal gyrus	-0.279	0.159	[-0.591 - 0.033]	7.994E-02	8.108E-01
Right superior temporal gyrus	-0.333	0.172	[-0.671 - 0.004]	5.296E-02	8.108E-01
Left insula	-0.118	0.142	[-0.396 - 0.161]	4.074E-01	8.916E-01
Right insula	-0.094	0.211	[-0.508 - 0.321]	6.583E-01	8.916E-01
Left lingual gyrus	-0.063	0.119	[-0.296 - 0.169]	5.933E-01	8.916E-01
Right lingual gyrus	0.075	0.149	[-0.217 - 0.366]	6.158E-01	8.916E-01
Left pars triangularis of inferior frontal gyrus	-0.192	0.169	[-0.524 - 0.139]	2.556E-01	8.916E-01
Right pars triangularis of inferior frontal gyrus	-0.148	0.126	[-0.394 - 0.099]	2.418E-01	8.916E-01
Left lateral orbitofrontal cortex	-0.052	0.119	[-0.284 - 0.181]	6.621E-01	8.916E-01
Right lateral orbitofrontal cortex	-0.001	0.190	[-0.374 - 0.371]	9.942E-01	9.942E-01
Left rostral middle frontal gyrus	-0.073	0.182	[-0.430 - 0.283]	6.867E-01	8.916E-01
Right rostral middle frontal gyrus	0.034	0.156	[-0.273 - 0.340]	8.296E-01	9.094E-01
Left middle temporal gyrus	0.025	0.160	[-0.288 - 0.338]	8.759E-01	9.282E-01
Right middle temporal gyrus	-0.235	0.176	[-0.580 - 0.111]	1.833E-01	8.916E-01
Left superior frontal gyrus	-0.057	0.215	[-0.478 - 0.365]	7.930E-01	9.081E-01
Right superior frontal gyrus	-0.076	0.235	[-0.536 - 0.384]	7.457E-01	8.916E-01
Left pars orbitalis of inferior frontal gyrus	-0.092	0.172	[-0.430 - 0.245]	5.919E-01	8.916E-01
Right pars orbitalis of inferior frontal gyrus	-0.241	0.127	[-0.490 - 0.009]	5.880E-02	8.108E-01
Left medial orbitofrontal cortex	-0.109	0.119	[-0.342 - 0.124]	3.583E-01	8.916E-01
Right medial orbitofrontal cortex	0.063	0.119	[-0.170 - 0.295]	5.972E-01	8.916E-01
Left inferior temporal gyrus	0.080	0.119	[-0.152 - 0.313]	4.991E-01	8.916E-01
Right inferior temporal gyrus	0.002	0.172	[-0.334 - 0.339]	9.888E-01	9.942E-01
Left isthmus cingulate cortex	0.094	0.167	[-0.233 - 0.421]	5.729E-01	8.916E-01
Right isthmus cingulate cortex	0.076	0.201	[-0.319 - 0.471]	7.048E-01	8.916E-01
Left banks of superior temporal sulcus	0.036	0.153	[-0.265 - 0.336]	8.163E-01	9.094E-01
Right banks of superior temporal sulcus	-0.064	0.157	[-0.372 - 0.244]	6.840E-01	8.916E-01
Left supramarginal gyrus	-0.204	0.175	[-0.548 - 0.140]	2.448E-01	8.916E-01
Right supramarginal gyrus	-0.320	0.175	[-0.664 - 0.024]	6.790E-02	8.108E-01
Left caudal middle frontal gyrus	-0.050	0.142	[-0.328 - 0.229]	7.260E-01	8.916E-01
Right caudal middle frontal gyrus	-0.074	0.191	[-0.449 - 0.300]	6.973E-01	8.916E-01
Left frontal pole	-0.045	0.119	[-0.278 - 0.187]	7.029E-01	8.916E-01
Right frontal pole	-0.082	0.126	[-0.329 - 0.164]	5.129E-01	8.916E-01
Left posterior cingulate cortex	-0.122	0.142	[-0.399 - 0.156]	3.898E-01	8.916E-01
Right posterior cingulate cortex	0.285	0.187	[-0.082 - 0.652]	1.285E-01	8.916E-01
Left lateral occipital cortex	-0.007	0.137	[-0.275 - 0.262]	9.607E-01	9.886E-01
Right lateral occipital cortex	-0.077	0.158	[-0.386 - 0.233]	6.265E-01	8.916E-01
Left precentral gyrus	-0.197	0.173	[-0.535 - 0.142]	2.546E-01	8.916E-01
Right precentral gyrus	-0.338	0.182	[-0.696 - 0.020]	6.408E-02	8.108E-01
Left parahippocampal gyrus	-0.172	0.119	[-0.405 - 0.060]	1.463E-01	8.916E-01
Right parahippocampal gyrus	-0.194	0.141	[-0.471 - 0.083]	1.694E-01	8.916E-01
Left inferior parietal cortex	-0.142	0.220	[-0.574 - 0.289]	5.178E-01	8.916E-01
Right inferior parietal cortex	-0.141	0.160	[-0.454 - 0.172]	3.761E-01	8.916E-01
Left transverse temporal gyrus	-0.248	0.155	[-0.553 - 0.057]	1.105E-01	8.916E-01
Right transverse temporal gyrus	-0.300	0.129	[-0.553 - -0.048]	1.964E-02	8.108E-01
Left postcentral gyrus	-0.178	0.149	[-0.470 - 0.115]	2.347E-01	8.916E-01
Right postcentral gyrus	-0.188	0.147	[-0.477 - 0.101]	2.017E-01	8.916E-01
Left precuneus	0.062	0.209	[-0.347 - 0.472]	7.660E-01	8.916E-01
Right precuneus	0.111	0.170	[-0.222 - 0.444]	5.131E-01	8.916E-01
Left caudal anterior cingulate cortex	0.117	0.133	[-0.144 - 0.378]	3.801E-01	8.916E-01
Right caudal anterior cingulate cortex	-0.016	0.164	[-0.338 - 0.305]	9.209E-01	9.615E-01
Left cuneus	0.099	0.187	[-0.268 - 0.466]	5.975E-01	8.916E-01
Right cuneus	0.023	0.119	[-0.209 - 0.256]	8.439E-01	9.094E-01
Left rostral anterior cingulate cortex	0.242	0.138	[-0.028 - 0.511]	7.900E-02	8.108E-01
Right rostral anterior cingulate cortex	0.153	0.119	[-0.080 - 0.385]	1.982E-01	8.916E-01
Left pericalcarine cortex	0.106	0.145	[-0.179 - 0.391]	4.660E-01	8.916E-01
Right pericalcarine cortex	0.081	0.122	[-0.159 - 0.320]	5.090E-01	8.916E-01
Left paracentral lobule	-0.143	0.231	[-0.596 - 0.309]	5.351E-01	8.916E-01
Right paracentral lobule	-0.107	0.203	[-0.505 - 0.291]	5.978E-01	8.916E-01
Left superior parietal cortex	-0.174	0.189	[-0.544 - 0.195]	3.554E-01	8.916E-01
Right superior parietal cortex	-0.093	0.145	[-0.377 - 0.192]	5.229E-01	8.916E-01
Left temporal pole	0.085	0.164	[-0.236 - 0.405]	6.049E-01	8.916E-01
Right temporal pole	-0.049	0.158	[-0.359 - 0.261]	7.556E-01	8.916E-01
Left entorhinal cortex	0.027	0.137	[-0.242 - 0.296]	8.454E-01	9.094E-01
Right entorhinal cortex	-0.050	0.158	[-0.360 - 0.259]	7.514E-01	8.916E-01

Supplementary Table S112. Cortical thickness differences between individuals with schizophrenia on second-generation antipsychotic medications and healthy comparison subjects controlling for age and sex

	d	Std. Err.	95% CI	*p*-value	FDR *q*-value
Global mean cortical thickness	-0.403	0.051	[-0.503 - -0.303]	3.057E-15	3.101E-14
Left hemisphere	-0.396	0.055	[-0.504 - -0.288]	5.684E-13	3.363E-12
Right hemisphere	-0.399	0.048	[-0.494 - -0.305]	9.777E-17	2.314E-15
Left fusiform gyrus	-0.485	0.064	[-0.610 - -0.360]	2.781E-14	2.194E-13
Right fusiform gyrus	-0.474	0.060	[-0.592 - -0.356]	3.015E-15	3.101E-14
Left pars opercularis of inferior frontal gyrus	-0.374	0.065	[-0.501 - -0.246]	9.563E-09	3.233E-08
Right pars opercularis of inferior frontal gyrus	-0.414	0.057	[-0.526 - -0.301]	4.901E-13	3.164E-12
Left superior temporal gyrus	-0.488	0.062	[-0.609 - -0.367]	2.952E-15	3.101E-14
Right superior temporal gyrus	-0.453	0.048	[-0.546 - -0.359]	3.370E-21	2.392E-19
Left insula	-0.410	0.048	[-0.503 - -0.316]	1.099E-17	3.901E-16
Right insula	-0.361	0.055	[-0.469 - -0.252]	7.660E-11	4.183E-10
Left lingual gyrus	-0.347	0.048	[-0.441 - -0.253]	3.674E-13	2.608E-12
Right lingual gyrus	-0.387	0.049	[-0.483 - -0.292]	1.680E-15	2.983E-14
Left pars triangularis of inferior frontal gyrus	-0.425	0.090	[-0.602 - -0.248]	2.414E-06	5.713E-06
Right pars triangularis of inferior frontal gyrus	-0.293	0.048	[-0.386 - -0.199]	7.997E-10	3.154E-09
Left lateral orbitofrontal cortex	-0.412	0.086	[-0.580 - -0.244]	1.546E-06	3.786E-06
Right lateral orbitofrontal cortex	-0.363	0.057	[-0.475 - -0.251]	1.912E-10	7.985E-10
Left rostral middle frontal gyrus	-0.399	0.076	[-0.548 - -0.249]	1.768E-07	5.231E-07
Right rostral middle frontal gyrus	-0.323	0.077	[-0.475 - -0.172]	2.950E-05	5.984E-05
Left middle temporal gyrus	-0.407	0.063	[-0.531 - -0.283]	1.244E-10	5.891E-10
Right middle temporal gyrus	-0.419	0.080	[-0.576 - -0.263]	1.531E-07	4.726E-07
Left superior frontal gyrus	-0.354	0.068	[-0.488 - -0.221]	1.911E-07	5.428E-07
Right superior frontal gyrus	-0.364	0.069	[-0.499 - -0.230]	1.158E-07	3.736E-07
Left pars orbitalis of inferior frontal gyrus	-0.361	0.079	[-0.516 - -0.206]	4.974E-06	1.104E-05
Right pars orbitalis of inferior frontal gyrus	-0.322	0.050	[-0.420 - -0.223]	1.448E-10	6.426E-10
Left medial orbitofrontal cortex	-0.362	0.086	[-0.530 - -0.193]	2.672E-05	5.579E-05
Right medial orbitofrontal cortex	-0.375	0.065	[-0.503 - -0.247]	8.827E-09	3.134E-08
Left inferior temporal gyrus	-0.374	0.048	[-0.468 - -0.280]	5.133E-15	4.555E-14
Right inferior temporal gyrus	-0.341	0.053	[-0.445 - -0.237]	1.166E-10	5.891E-10
Left isthmus cingulate cortex	-0.249	0.055	[-0.356 - -0.141]	5.930E-06	1.276E-05
Right isthmus cingulate cortex	-0.283	0.048	[-0.377 - -0.190]	2.814E-09	1.052E-08
Left banks of superior temporal sulcus	-0.292	0.063	[-0.415 - -0.169]	3.382E-06	7.745E-06
Right banks of superior temporal sulcus	-0.300	0.060	[-0.417 - -0.182]	5.453E-07	1.434E-06
Left supramarginal gyrus	-0.287	0.071	[-0.427 - -0.147]	5.839E-05	1.121E-04
Right supramarginal gyrus	-0.203	0.065	[-0.330 - -0.077]	1.670E-03	2.280E-03
Left caudal middle frontal gyrus	-0.233	0.048	[-0.326 - -0.139]	1.027E-06	2.604E-06
Right caudal middle frontal gyrus	-0.245	0.048	[-0.338 - -0.152]	2.726E-07	7.445E-07
Left frontal pole	-0.285	0.085	[-0.450 - -0.119]	7.641E-04	1.107E-03
Right frontal pole	-0.361	0.095	[-0.548 - -0.174]	1.537E-04	2.661E-04
Left posterior cingulate cortex	-0.205	0.051	[-0.305 - -0.104]	6.783E-05	1.267E-04
Right posterior cingulate cortex	-0.264	0.079	[-0.420 - -0.109]	8.461E-04	1.202E-03
Left lateral occipital cortex	-0.165	0.048	[-0.258 - -0.071]	5.432E-04	8.384E-04
Right lateral occipital cortex	-0.198	0.048	[-0.291 - -0.105]	3.151E-05	6.214E-05
Left precentral gyrus	-0.198	0.057	[-0.309 - -0.087]	4.961E-04	7.827E-04
Right precentral gyrus	-0.191	0.051	[-0.291 - -0.091]	1.766E-04	2.915E-04
Left parahippocampal gyrus	-0.243	0.072	[-0.383 - -0.103]	6.807E-04	1.007E-03
Right parahippocampal gyrus	-0.215	0.063	[-0.338 - -0.092]	5.880E-04	8.882E-04
Left inferior parietal cortex	-0.189	0.048	[-0.282 - -0.096]	6.972E-05	1.269E-04
Right inferior parietal cortex	-0.168	0.054	[-0.274 - -0.061]	2.013E-03	2.697E-03
Left transverse temporal gyrus	-0.186	0.048	[-0.279 - -0.093]	9.076E-05	1.611E-04
Right transverse temporal gyrus	-0.220	0.058	[-0.335 - -0.106]	1.606E-04	2.715E-04
Left postcentral gyrus	-0.126	0.052	[-0.229 - -0.024]	1.551E-02	2.039E-02
Right postcentral gyrus	-0.169	0.052	[-0.271 - -0.066]	1.308E-03	1.820E-03
Left precuneus	-0.082	0.048	[-0.176 - 0.011]	8.319E-02	1.018E-01
Right precuneus	-0.168	0.048	[-0.261 - -0.075]	4.102E-04	6.619E-04
Left caudal anterior cingulate cortex	-0.098	0.073	[-0.242 - 0.046]	1.820E-01	2.154E-01
Right caudal anterior cingulate cortex	-0.119	0.050	[-0.216 - -0.022]	1.624E-02	2.089E-02
Left cuneus	-0.114	0.048	[-0.207 - -0.021]	1.648E-02	2.089E-02
Right cuneus	-0.111	0.048	[-0.204 - -0.017]	1.999E-02	2.490E-02
Left rostral anterior cingulate cortex	-0.114	0.092	[-0.295 - 0.066]	2.154E-01	2.467E-01
Right rostral anterior cingulate cortex	-0.074	0.063	[-0.197 - 0.049]	2.394E-01	2.698E-01
Left pericalcarine cortex	-0.077	0.061	[-0.197 - 0.043]	2.080E-01	2.421E-01
Right pericalcarine cortex	-0.020	0.048	[-0.113 - 0.073]	6.687E-01	7.086E-01
Left paracentral lobule	-0.018	0.060	[-0.135 - 0.100]	7.664E-01	7.773E-01
Right paracentral lobule	-0.044	0.057	[-0.156 - 0.069]	4.478E-01	4.891E-01
Left superior parietal cortex	0.003	0.047	[-0.090 - 0.096]	9.488E-01	9.488E-01
Right superior parietal cortex	-0.026	0.047	[-0.119 - 0.067]	5.809E-01	6.249E-01
Left temporal pole	-0.051	0.066	[-0.180 - 0.078]	4.367E-01	4.845E-01
Right temporal pole	0.019	0.059	[-0.097 - 0.135]	7.507E-01	7.724E-01
Left entorhinal cortex	-0.018	0.051	[-0.118 - 0.083]	7.312E-01	7.634E-01
Right entorhinal cortex	-0.121	0.078	[-0.273 - 0.031]	1.175E-01	1.414E-01

Supplementary Table S113. Cortical thickness differences between individuals with schizophrenia on second-generation antipsychotic medications and individuals with schizophrenia who are unmedicated with antipsychotics controlling for age and sex

	d	Std. Err.	95% CI	*p*-value	FDR *q*-value
Global mean cortical thickness	-0.194	0.184	[-0.555 - 0.166]	2.907E-01	6.071E-01
Left hemisphere	-0.188	0.192	[-0.563 - 0.188]	3.270E-01	6.091E-01
Right hemisphere	-0.198	0.174	[-0.539 - 0.142]	2.540E-01	6.071E-01
Left fusiform gyrus	-0.381	0.181	[-0.735 - -0.026]	3.554E-02	2.524E-01
Right fusiform gyrus	-0.333	0.122	[-0.573 - -0.094]	6.419E-03	1.139E-01
Left pars opercularis of inferior frontal gyrus	-0.073	0.122	[-0.311 - 0.166]	5.505E-01	7.230E-01
Right pars opercularis of inferior frontal gyrus	-0.235	0.183	[-0.593 - 0.123]	1.991E-01	5.889E-01
Left superior temporal gyrus	-0.103	0.172	[-0.439 - 0.233]	5.483E-01	7.230E-01
Right superior temporal gyrus	-0.058	0.159	[-0.370 - 0.254]	7.147E-01	8.054E-01
Left insula	-0.219	0.135	[-0.484 - 0.045]	1.034E-01	4.890E-01
Right insula	-0.273	0.184	[-0.634 - 0.088]	1.388E-01	5.121E-01
Left lingual gyrus	-0.284	0.122	[-0.523 - -0.044]	2.015E-02	1.794E-01
Right lingual gyrus	-0.355	0.122	[-0.595 - -0.115]	3.716E-03	8.794E-02
Left pars triangularis of inferior frontal gyrus	-0.057	0.178	[-0.406 - 0.293]	7.505E-01	8.198E-01
Right pars triangularis of inferior frontal gyrus	-0.074	0.122	[-0.313 - 0.164]	5.404E-01	7.230E-01
Left lateral orbitofrontal cortex	-0.277	0.160	[-0.591 - 0.037]	8.369E-02	4.571E-01
Right lateral orbitofrontal cortex	-0.340	0.147	[-0.628 - -0.053]	2.022E-02	1.794E-01
Left rostral middle frontal gyrus	-0.205	0.205	[-0.607 - 0.196]	3.158E-01	6.091E-01
Right rostral middle frontal gyrus	-0.265	0.188	[-0.634 - 0.104]	1.587E-01	5.121E-01
Left middle temporal gyrus	-0.441	0.171	[-0.777 - -0.105]	1.013E-02	1.439E-01
Right middle temporal gyrus	-0.172	0.207	[-0.578 - 0.234]	4.071E-01	6.820E-01
Left superior frontal gyrus	-0.280	0.239	[-0.748 - 0.188]	2.415E-01	6.071E-01
Right superior frontal gyrus	-0.268	0.241	[-0.740 - 0.204]	2.660E-01	6.071E-01
Left pars orbitalis of inferior frontal gyrus	-0.114	0.146	[-0.400 - 0.173]	4.372E-01	6.820E-01
Right pars orbitalis of inferior frontal gyrus	-0.068	0.139	[-0.341 - 0.204]	6.229E-01	7.250E-01
Left medial orbitofrontal cortex	-0.242	0.169	[-0.574 - 0.089]	1.524E-01	5.121E-01
Right medial orbitofrontal cortex	-0.362	0.147	[-0.650 - -0.074]	1.383E-02	1.637E-01
Left inferior temporal gyrus	-0.470	0.137	[-0.739 - -0.201]	6.276E-04	2.530E-02
Right inferior temporal gyrus	-0.310	0.147	[-0.599 - -0.021]	3.533E-02	2.524E-01
Left isthmus cingulate cortex	-0.303	0.180	[-0.655 - 0.048]	9.105E-02	4.618E-01
Right isthmus cingulate cortex	-0.345	0.178	[-0.694 - 0.005]	5.322E-02	3.435E-01
Left banks of superior temporal sulcus	-0.238	0.157	[-0.545 - 0.070]	1.295E-01	5.121E-01
Right banks of superior temporal sulcus	-0.176	0.122	[-0.414 - 0.063]	1.497E-01	5.121E-01
Left supramarginal gyrus	0.022	0.122	[-0.217 - 0.260]	8.576E-01	8.955E-01
Right supramarginal gyrus	0.119	0.154	[-0.183 - 0.421]	4.389E-01	6.820E-01
Left caudal middle frontal gyrus	-0.110	0.127	[-0.358 - 0.138]	3.854E-01	6.674E-01
Right caudal middle frontal gyrus	-0.139	0.193	[-0.516 - 0.239]	4.712E-01	6.969E-01
Left frontal pole	-0.268	0.168	[-0.597 - 0.061]	1.102E-01	4.890E-01
Right frontal pole	-0.220	0.194	[-0.600 - 0.161]	2.575E-01	6.071E-01
Left posterior cingulate cortex	-0.088	0.145	[-0.373 - 0.197]	5.430E-01	7.230E-01
Right posterior cingulate cortex	-0.448	0.132	[-0.707 - -0.188]	7.127E-04	2.530E-02
Left lateral occipital cortex	-0.131	0.122	[-0.370 - 0.107]	2.808E-01	6.071E-01
Right lateral occipital cortex	-0.149	0.122	[-0.388 - 0.090]	2.214E-01	6.071E-01
Left precentral gyrus	0.073	0.138	[-0.198 - 0.345]	5.953E-01	7.230E-01
Right precentral gyrus	0.110	0.143	[-0.171 - 0.391]	4.419E-01	6.820E-01
Left parahippocampal gyrus	0.052	0.161	[-0.264 - 0.368]	7.469E-01	8.198E-01
Right parahippocampal gyrus	0.067	0.162	[-0.250 - 0.384]	6.800E-01	7.787E-01
Left inferior parietal cortex	-0.023	0.198	[-0.410 - 0.364]	9.068E-01	9.331E-01
Right inferior parietal cortex	-0.070	0.141	[-0.346 - 0.207]	6.213E-01	7.250E-01
Left transverse temporal gyrus	0.042	0.175	[-0.300 - 0.385]	8.088E-01	8.700E-01
Right transverse temporal gyrus	0.008	0.126	[-0.240 - 0.255]	9.525E-01	9.525E-01
Left postcentral gyrus	0.157	0.168	[-0.173 - 0.486]	3.508E-01	6.227E-01
Right postcentral gyrus	0.101	0.176	[-0.245 - 0.447]	5.670E-01	7.230E-01
Left precuneus	-0.034	0.166	[-0.360 - 0.292]	8.396E-01	8.897E-01
Right precuneus	-0.121	0.122	[-0.359 - 0.118]	3.209E-01	6.091E-01
Left caudal anterior cingulate cortex	-0.210	0.179	[-0.561 - 0.141]	2.411E-01	6.071E-01
Right caudal anterior cingulate cortex	-0.114	0.176	[-0.460 - 0.232]	5.189E-01	7.230E-01
Left cuneus	-0.215	0.215	[-0.636 - 0.206]	3.162E-01	6.091E-01
Right cuneus	-0.148	0.139	[-0.420 - 0.123]	2.844E-01	6.071E-01
Left rostral anterior cingulate cortex	-0.232	0.130	[-0.487 - 0.022]	7.373E-02	4.362E-01
Right rostral anterior cingulate cortex	-0.159	0.122	[-0.398 - 0.080]	1.912E-01	5.889E-01
Left pericalcarine cortex	-0.169	0.175	[-0.513 - 0.175]	3.346E-01	6.091E-01
Right pericalcarine cortex	-0.100	0.151	[-0.395 - 0.195]	5.063E-01	7.230E-01
Left paracentral lobule	0.107	0.205	[-0.294 - 0.509]	6.008E-01	7.230E-01
Right paracentral lobule	0.134	0.180	[-0.218 - 0.487]	4.551E-01	6.874E-01
Left superior parietal cortex	0.213	0.142	[-0.065 - 0.492]	1.337E-01	5.121E-01
Right superior parietal cortex	0.097	0.122	[-0.141 - 0.336]	4.250E-01	6.820E-01
Left temporal pole	-0.078	0.139	[-0.350 - 0.194]	5.727E-01	7.230E-01
Right temporal pole	0.213	0.194	[-0.166 - 0.593]	2.704E-01	6.071E-01
Left entorhinal cortex	0.067	0.122	[-0.172 - 0.305]	5.833E-01	7.230E-01
Right entorhinal cortex	0.010	0.155	[-0.294 - 0.313]	9.495E-01	9.525E-01

Supplementary Table S114. Cortical thickness differences between individuals with schizophrenia on first-generation antipsychotic medications and healthy comparison subjects controlling for age and sex

	d	Std. Err.	95% CI	*p*-value	FDR *q*-value
Global mean cortical thickness	-0.475	0.170	[-0.807 - -0.142]	5.165E-03	1.358E-02
Left hemisphere	-0.505	0.143	[-0.786 - -0.224]	4.308E-04	1.912E-03
Right hemisphere	-0.425	0.190	[-0.797 - -0.054]	2.489E-02	5.197E-02
Left fusiform gyrus	-0.439	0.101	[-0.636 - -0.241]	1.319E-05	1.561E-04
Right fusiform gyrus	-0.388	0.101	[-0.585 - -0.190]	1.169E-04	8.301E-04
Left pars opercularis of inferior frontal gyrus	-0.588	0.142	[-0.867 - -0.310]	3.440E-05	3.489E-04
Right pars opercularis of inferior frontal gyrus	-0.417	0.219	[-0.846 - 0.012]	5.702E-02	9.639E-02
Left superior temporal gyrus	-0.362	0.101	[-0.560 - -0.165]	3.170E-04	1.875E-03
Right superior temporal gyrus	-0.442	0.129	[-0.696 - -0.189]	6.304E-04	2.238E-03
Left insula	-0.453	0.111	[-0.671 - -0.234]	4.810E-05	4.269E-04
Right insula	-0.415	0.153	[-0.714 - -0.115]	6.735E-03	1.649E-02
Left lingual gyrus	-0.571	0.101	[-0.769 - -0.373]	1.655E-08	1.175E-06
Right lingual gyrus	-0.495	0.101	[-0.693 - -0.296]	9.884E-07	2.339E-05
Left pars triangularis of inferior frontal gyrus	-0.595	0.128	[-0.846 - -0.344]	3.392E-06	6.020E-05
Right pars triangularis of inferior frontal gyrus	-0.458	0.225	[-0.899 - -0.017]	4.178E-02	7.606E-02
Left lateral orbitofrontal cortex	-0.437	0.119	[-0.671 - -0.203]	2.457E-04	1.586E-03
Right lateral orbitofrontal cortex	-0.417	0.194	[-0.797 - -0.038]	3.106E-02	6.126E-02
Left rostral middle frontal gyrus	-0.597	0.168	[-0.927 - -0.268]	3.867E-04	1.912E-03
Right rostral middle frontal gyrus	-0.314	0.217	[-0.740 - 0.112]	1.489E-01	2.034E-01
Left middle temporal gyrus	-0.308	0.101	[-0.505 - -0.111]	2.192E-03	7.039E-03
Right middle temporal gyrus	-0.228	0.161	[-0.544 - 0.088]	1.569E-01	2.090E-01
Left superior frontal gyrus	-0.360	0.102	[-0.560 - -0.160]	4.068E-04	1.912E-03
Right superior frontal gyrus	-0.361	0.143	[-0.641 - -0.081]	1.140E-02	2.699E-02
Left pars orbitalis of inferior frontal gyrus	-0.446	0.101	[-0.644 - -0.248]	9.990E-06	1.419E-04
Right pars orbitalis of inferior frontal gyrus	-0.186	0.171	[-0.522 - 0.151]	2.793E-01	3.542E-01
Left medial orbitofrontal cortex	-0.398	0.165	[-0.722 - -0.074]	1.616E-02	3.700E-02
Right medial orbitofrontal cortex	-0.512	0.101	[-0.710 - -0.314]	4.056E-07	1.440E-05
Left inferior temporal gyrus	-0.359	0.103	[-0.560 - -0.158]	4.708E-04	1.966E-03
Right inferior temporal gyrus	-0.230	0.100	[-0.427 - -0.034]	2.160E-02	4.648E-02
Left isthmus cingulate cortex	-0.384	0.179	[-0.734 - -0.033]	3.204E-02	6.148E-02
Right isthmus cingulate cortex	-0.348	0.100	[-0.545 - -0.151]	5.259E-04	2.075E-03
Left banks of superior temporal sulcus	-0.346	0.101	[-0.543 - -0.149]	5.820E-04	2.175E-03
Right banks of superior temporal sulcus	-0.321	0.100	[-0.518 - -0.124]	1.400E-03	4.733E-03
Left supramarginal gyrus	-0.338	0.188	[-0.707 - 0.031]	7.283E-02	1.100E-01
Right supramarginal gyrus	-0.207	0.147	[-0.496 - 0.081]	1.589E-01	2.090E-01
Left caudal middle frontal gyrus	-0.424	0.156	[-0.730 - -0.119]	6.416E-03	1.627E-02
Right caudal middle frontal gyrus	-0.273	0.176	[-0.618 - 0.071]	1.195E-01	1.696E-01
Left frontal pole	-0.347	0.166	[-0.672 - -0.022]	3.617E-02	6.758E-02
Right frontal pole	-0.295	0.101	[-0.492 - -0.098]	3.382E-03	1.000E-02
Left posterior cingulate cortex	-0.359	0.101	[-0.557 - -0.162]	3.507E-04	1.912E-03
Right posterior cingulate cortex	-0.282	0.100	[-0.479 - -0.086]	4.927E-03	1.345E-02
Left lateral occipital cortex	-0.160	0.100	[-0.356 - 0.036]	1.105E-01	1.601E-01
Right lateral occipital cortex	-0.324	0.161	[-0.640 - -0.009]	4.380E-02	7.775E-02
Left precentral gyrus	-0.165	0.170	[-0.498 - 0.167]	3.302E-01	4.043E-01
Right precentral gyrus	-0.120	0.181	[-0.474 - 0.235]	5.074E-01	5.719E-01
Left parahippocampal gyrus	-0.200	0.100	[-0.396 - -0.003]	4.635E-02	8.026E-02
Right parahippocampal gyrus	-0.307	0.100	[-0.503 - -0.110]	2.280E-03	7.039E-03
Left inferior parietal cortex	-0.273	0.148	[-0.563 - 0.016]	6.444E-02	1.017E-01
Right inferior parietal cortex	-0.276	0.147	[-0.563 - 0.012]	6.015E-02	9.932E-02
Left transverse temporal gyrus	-0.311	0.130	[-0.566 - -0.056]	1.687E-02	3.744E-02
Right transverse temporal gyrus	-0.239	0.143	[-0.520 - 0.042]	9.588E-02	1.418E-01
Left postcentral gyrus	-0.243	0.237	[-0.707 - 0.222]	3.062E-01	3.814E-01
Right postcentral gyrus	-0.179	0.203	[-0.577 - 0.219]	3.786E-01	4.480E-01
Left precuneus	-0.091	0.116	[-0.319 - 0.136]	4.321E-01	4.949E-01
Right precuneus	-0.213	0.185	[-0.575 - 0.150]	2.497E-01	3.223E-01
Left caudal anterior cingulate cortex	-0.123	0.137	[-0.391 - 0.145]	3.694E-01	4.445E-01
Right caudal anterior cingulate cortex	-0.224	0.123	[-0.465 - 0.018]	6.930E-02	1.070E-01
Left cuneus	-0.059	0.100	[-0.255 - 0.137]	5.542E-01	6.149E-01
Right cuneus	-0.185	0.100	[-0.382 - 0.011]	6.434E-02	1.017E-01
Left rostral anterior cingulate cortex	-0.042	0.100	[-0.239 - 0.154]	6.716E-01	7.199E-01
Right rostral anterior cingulate cortex	-0.016	0.100	[-0.212 - 0.181]	8.760E-01	8.760E-01
Left pericalcarine cortex	-0.404	0.101	[-0.601 - -0.206]	6.043E-05	4.767E-04
Right pericalcarine cortex	-0.368	0.129	[-0.621 - -0.116]	4.271E-03	1.213E-02
Left paracentral lobule	0.058	0.100	[-0.138 - 0.254]	5.635E-01	6.155E-01
Right paracentral lobule	0.110	0.128	[-0.140 - 0.361]	3.883E-01	4.520E-01
Left superior parietal cortex	-0.042	0.211	[-0.455 - 0.371]	8.416E-01	8.536E-01
Right superior parietal cortex	0.052	0.224	[-0.387 - 0.490]	8.179E-01	8.416E-01
Left temporal pole	0.145	0.100	[-0.051 - 0.341]	1.475E-01	2.034E-01
Right temporal pole	0.217	0.100	[0.020 - 0.414]	3.067E-02	6.126E-02
Left entorhinal cortex	0.041	0.100	[-0.155 - 0.237]	6.794E-01	7.199E-01
Right entorhinal cortex	0.030	0.100	[-0.166 - 0.226]	7.667E-01	8.005E-01

Supplementary Table S115. Cortical thickness differences between individuals with schizophrenia on first-generation antipsychotic medications and individuals with schizophrenia who are unmedicated with antipsychotics controlling for age and sex

	d	Std. Err.	95% CI	*p*-value	FDR *q*-value
Global mean cortical thickness	-0.468	0.406	[-1.265 - 0.328]	2.488E-01	6.390E-01
Left hemisphere	-0.443	0.367	[-1.161 - 0.276]	2.269E-01	6.390E-01
Right hemisphere	-0.482	0.435	[-1.335 - 0.372]	2.686E-01	6.390E-01
Left fusiform gyrus	-0.193	0.319	[-0.818 - 0.432]	5.451E-01	8.556E-01
Right fusiform gyrus	-0.560	0.402	[-1.348 - 0.228]	1.637E-01	6.390E-01
Left pars opercularis of inferior frontal gyrus	-0.576	0.330	[-1.224 - 0.071]	8.118E-02	6.390E-01
Right pars opercularis of inferior frontal gyrus	-0.741	0.359	[-1.444 - -0.037]	3.904E-02	6.390E-01
Left superior temporal gyrus	-0.279	0.319	[-0.905 - 0.347]	3.827E-01	7.727E-01
Right superior temporal gyrus	-0.426	0.358	[-1.127 - 0.275]	2.335E-01	6.390E-01
Left insula	-0.432	0.380	[-1.178 - 0.314]	2.560E-01	6.390E-01
Right insula	-0.169	0.319	[-0.795 - 0.456]	5.959E-01	8.556E-01
Left lingual gyrus	-0.387	0.339	[-1.051 - 0.277]	2.536E-01	6.390E-01
Right lingual gyrus	-0.544	0.322	[-1.175 - 0.087]	9.121E-02	6.390E-01
Left pars triangularis of inferior frontal gyrus	-0.457	0.322	[-1.087 - 0.174]	1.559E-01	6.390E-01
Right pars triangularis of inferior frontal gyrus	-0.397	0.360	[-1.104 - 0.309]	2.700E-01	6.390E-01
Left lateral orbitofrontal cortex	-0.309	0.321	[-0.937 - 0.319]	3.354E-01	7.215E-01
Right lateral orbitofrontal cortex	-0.273	0.319	[-0.899 - 0.352]	3.918E-01	7.727E-01
Left rostral middle frontal gyrus	-0.193	0.319	[-0.819 - 0.433]	5.459E-01	8.556E-01
Right rostral middle frontal gyrus	-0.378	0.320	[-1.006 - 0.249]	2.376E-01	6.390E-01
Left middle temporal gyrus	-0.301	0.603	[-1.483 - 0.880]	6.171E-01	8.556E-01
Right middle temporal gyrus	0.217	0.564	[-0.888 - 1.322]	7.004E-01	8.556E-01
Left superior frontal gyrus	-0.255	0.319	[-0.881 - 0.370]	4.240E-01	8.136E-01
Right superior frontal gyrus	-0.410	0.321	[-1.039 - 0.218]	2.009E-01	6.390E-01
Left pars orbitalis of inferior frontal gyrus	-0.460	0.321	[-1.089 - 0.169]	1.521E-01	6.390E-01
Right pars orbitalis of inferior frontal gyrus	0.282	0.320	[-0.345 - 0.909]	3.780E-01	7.727E-01
Left medial orbitofrontal cortex	-0.507	0.322	[-1.138 - 0.123]	1.147E-01	6.390E-01
Right medial orbitofrontal cortex	-0.425	0.321	[-1.054 - 0.204]	1.855E-01	6.390E-01
Left inferior temporal gyrus	-0.456	0.324	[-1.090 - 0.179]	1.592E-01	6.390E-01
Right inferior temporal gyrus	-0.574	0.323	[-1.206 - 0.058]	7.525E-02	6.390E-01
Left isthmus cingulate cortex	-0.461	0.321	[-1.091 - 0.169]	1.519E-01	6.390E-01
Right isthmus cingulate cortex	-0.335	0.321	[-0.964 - 0.293]	2.956E-01	6.769E-01
Left banks of superior temporal sulcus	-0.527	0.323	[-1.161 - 0.107]	1.031E-01	6.390E-01
Right banks of superior temporal sulcus	-0.387	0.400	[-1.170 - 0.397]	3.335E-01	7.215E-01
Left supramarginal gyrus	-0.216	0.469	[-1.135 - 0.702]	6.443E-01	8.556E-01
Right supramarginal gyrus	0.021	0.363	[-0.691 - 0.732]	9.548E-01	9.766E-01
Left caudal middle frontal gyrus	-0.354	0.321	[-0.983 - 0.274]	2.695E-01	6.390E-01
Right caudal middle frontal gyrus	-0.819	0.327	[-1.461 - -0.178]	1.228E-02	4.361E-01
Left frontal pole	0.023	0.406	[-0.773 - 0.819]	9.540E-01	9.766E-01
Right frontal pole	-0.408	0.320	[-1.036 - 0.220]	2.029E-01	6.390E-01
Left posterior cingulate cortex	-0.200	0.320	[-0.827 - 0.427]	5.318E-01	8.556E-01
Right posterior cingulate cortex	-0.794	0.401	[-1.579 - -0.008]	4.768E-02	6.390E-01
Left lateral occipital cortex	-0.567	0.323	[-1.200 - 0.066]	7.901E-02	6.390E-01
Right lateral occipital cortex	-1.099	0.334	[-1.753 - -0.446]	9.812E-04	6.967E-02
Left precentral gyrus	-0.122	0.319	[-0.746 - 0.503]	7.021E-01	8.556E-01
Right precentral gyrus	-0.254	0.719	[-1.663 - 1.154]	7.236E-01	8.563E-01
Left parahippocampal gyrus	0.108	0.320	[-0.519 - 0.734]	7.363E-01	8.570E-01
Right parahippocampal gyrus	-0.165	0.320	[-0.792 - 0.462]	6.065E-01	8.556E-01
Left inferior parietal cortex	-0.442	0.695	[-1.804 - 0.920]	5.245E-01	8.556E-01
Right inferior parietal cortex	-0.174	0.391	[-0.941 - 0.593]	6.559E-01	8.556E-01
Left transverse temporal gyrus	-0.238	0.577	[-1.369 - 0.893]	6.802E-01	8.556E-01
Right transverse temporal gyrus	0.027	0.318	[-0.597 - 0.651]	9.320E-01	9.766E-01
Left postcentral gyrus	-0.311	0.788	[-1.856 - 1.234]	6.932E-01	8.556E-01
Right postcentral gyrus	-0.213	0.319	[-0.839 - 0.413]	5.050E-01	8.556E-01
Left precuneus	-0.291	0.622	[-1.510 - 0.928]	6.400E-01	8.556E-01
Right precuneus	-0.333	0.651	[-1.608 - 0.943]	6.091E-01	8.556E-01
Left caudal anterior cingulate cortex	0.118	0.319	[-0.507 - 0.743]	7.110E-01	8.556E-01
Right caudal anterior cingulate cortex	-0.062	0.795	[-1.621 - 1.497]	9.377E-01	9.766E-01
Left cuneus	0.288	0.640	[-0.966 - 1.543]	6.525E-01	8.556E-01
Right cuneus	-0.054	0.319	[-0.679 - 0.571]	8.655E-01	9.766E-01
Left rostral anterior cingulate cortex	0.002	0.319	[-0.622 - 0.627]	9.938E-01	9.938E-01
Right rostral anterior cingulate cortex	-0.030	0.436	[-0.885 - 0.824]	9.444E-01	9.766E-01
Left pericalcarine cortex	-0.027	0.318	[-0.651 - 0.597]	9.314E-01	9.766E-01
Right pericalcarine cortex	-0.234	0.319	[-0.860 - 0.392]	4.646E-01	8.457E-01
Left paracentral lobule	0.242	0.319	[-0.384 - 0.868]	4.482E-01	8.374E-01
Right paracentral lobule	0.197	0.405	[-0.596 - 0.990]	6.267E-01	8.556E-01
Left superior parietal cortex	-0.085	0.608	[-1.276 - 1.107]	8.893E-01	9.766E-01
Right superior parietal cortex	0.044	0.943	[-1.804 - 1.892]	9.629E-01	9.766E-01
Left temporal pole	0.157	0.319	[-0.468 - 0.782]	6.227E-01	8.556E-01
Right temporal pole	0.401	0.321	[-0.228 - 1.031]	2.115E-01	6.390E-01
Left entorhinal cortex	0.393	0.344	[-0.282 - 1.068]	2.541E-01	6.390E-01
Right entorhinal cortex	0.420	0.321	[-0.208 - 1.049]	1.901E-01	6.390E-01

Supplementary Table S116. Cortical thickness differences between individuals with schizophrenia on first-generation antipsychotic medications and individuals with schizophrenia on second-generation antipsychotic medications controlling for age and sex

	d	Std. Err.	95% CI	*p*-value	FDR *q*-value
Global mean cortical thickness	-0.311	0.159	[-0.623 - 0.002]	5.117E-02	2.860E-01
Left hemisphere	-0.318	0.148	[-0.608 - -0.028]	3.142E-02	2.837E-01
Right hemisphere	-0.293	0.168	[-0.623 - 0.037]	8.161E-02	3.219E-01
Left fusiform gyrus	0.043	0.141	[-0.233 - 0.319]	7.601E-01	8.356E-01
Right fusiform gyrus	-0.027	0.141	[-0.304 - 0.250]	8.488E-01	8.734E-01
Left pars opercularis of inferior frontal gyrus	-0.335	0.280	[-0.884 - 0.214]	2.322E-01	5.355E-01
Right pars opercularis of inferior frontal gyrus	-0.298	0.244	[-0.776 - 0.180]	2.211E-01	5.355E-01
Left superior temporal gyrus	0.074	0.215	[-0.347 - 0.495]	7.301E-01	8.228E-01
Right superior temporal gyrus	-0.155	0.146	[-0.442 - 0.131]	2.879E-01	5.355E-01
Left insula	-0.212	0.218	[-0.639 - 0.215]	3.301E-01	5.716E-01
Right insula	-0.346	0.171	[-0.680 - -0.012]	4.246E-02	2.860E-01
Left lingual gyrus	-0.182	0.141	[-0.459 - 0.094]	1.966E-01	5.355E-01
Right lingual gyrus	-0.108	0.184	[-0.468 - 0.252]	5.561E-01	7.450E-01
Left pars triangularis of inferior frontal gyrus	-0.247	0.199	[-0.637 - 0.142]	2.129E-01	5.355E-01
Right pars triangularis of inferior frontal gyrus	-0.373	0.232	[-0.828 - 0.081]	1.074E-01	3.743E-01
Left lateral orbitofrontal cortex	-0.211	0.189	[-0.582 - 0.160]	2.658E-01	5.355E-01
Right lateral orbitofrontal cortex	-0.144	0.173	[-0.482 - 0.195]	4.054E-01	6.465E-01
Left rostral middle frontal gyrus	-0.284	0.221	[-0.718 - 0.150]	2.002E-01	5.355E-01
Right rostral middle frontal gyrus	-0.156	0.205	[-0.558 - 0.246]	4.463E-01	6.807E-01
Left middle temporal gyrus	-0.058	0.141	[-0.336 - 0.219]	6.790E-01	7.883E-01
Right middle temporal gyrus	-0.043	0.244	[-0.520 - 0.435]	8.611E-01	8.734E-01
Left superior frontal gyrus	-0.149	0.141	[-0.426 - 0.128]	2.915E-01	5.355E-01
Right superior frontal gyrus	-0.138	0.141	[-0.415 - 0.139]	3.299E-01	5.716E-01
Left pars orbitalis of inferior frontal gyrus	-0.067	0.141	[-0.343 - 0.210]	6.367E-01	7.883E-01
Right pars orbitalis of inferior frontal gyrus	-0.119	0.199	[-0.509 - 0.272]	5.510E-01	7.450E-01
Left medial orbitofrontal cortex	-0.211	0.192	[-0.587 - 0.166]	2.725E-01	5.355E-01
Right medial orbitofrontal cortex	-0.241	0.142	[-0.518 - 0.037]	8.896E-02	3.324E-01
Left inferior temporal gyrus	-0.119	0.163	[-0.438 - 0.200]	4.641E-01	6.807E-01
Right inferior temporal gyrus	-0.057	0.141	[-0.333 - 0.220]	6.884E-01	7.883E-01
Left isthmus cingulate cortex	-0.255	0.142	[-0.533 - 0.022]	7.160E-02	3.174E-01
Right isthmus cingulate cortex	-0.075	0.141	[-0.351 - 0.202]	5.963E-01	7.698E-01
Left banks of superior temporal sulcus	-0.159	0.142	[-0.436 - 0.119]	2.620E-01	5.355E-01
Right banks of superior temporal sulcus	-0.064	0.142	[-0.343 - 0.215]	6.534E-01	7.883E-01
Left supramarginal gyrus	-0.267	0.218	[-0.694 - 0.160]	2.208E-01	5.355E-01
Right supramarginal gyrus	-0.261	0.142	[-0.539 - 0.017]	6.591E-02	3.120E-01
Left caudal middle frontal gyrus	-0.185	0.176	[-0.530 - 0.161]	2.941E-01	5.355E-01
Right caudal middle frontal gyrus	-0.276	0.142	[-0.554 - 0.003]	5.236E-02	2.860E-01
Left frontal pole	-0.304	0.270	[-0.832 - 0.224]	2.595E-01	5.355E-01
Right frontal pole	0.075	0.141	[-0.202 - 0.352]	5.941E-01	7.698E-01
Left posterior cingulate cortex	-0.339	0.212	[-0.755 - 0.077]	1.107E-01	3.743E-01
Right posterior cingulate cortex	-0.252	0.142	[-0.530 - 0.026]	7.601E-02	3.174E-01
Left lateral occipital cortex	-0.199	0.141	[-0.476 - 0.077]	1.575E-01	4.661E-01
Right lateral occipital cortex	-0.384	0.154	[-0.686 - -0.082]	1.270E-02	2.254E-01
Left precentral gyrus	-0.208	0.142	[-0.485 - 0.070]	1.420E-01	4.583E-01
Right precentral gyrus	-0.061	0.141	[-0.337 - 0.216]	6.673E-01	7.883E-01
Left parahippocampal gyrus	-0.037	0.141	[-0.314 - 0.240]	7.909E-01	8.381E-01
Right parahippocampal gyrus	-0.130	0.180	[-0.484 - 0.223]	4.699E-01	6.807E-01
Left inferior parietal cortex	-0.298	0.142	[-0.576 - -0.019]	3.597E-02	2.837E-01
Right inferior parietal cortex	-0.298	0.142	[-0.576 - -0.020]	3.575E-02	2.837E-01
Left transverse temporal gyrus	-0.266	0.187	[-0.633 - 0.101]	1.552E-01	4.661E-01
Right transverse temporal gyrus	-0.280	0.143	[-0.561 - 0.001]	5.094E-02	2.860E-01
Left postcentral gyrus	-0.338	0.146	[-0.624 - -0.051]	2.081E-02	2.462E-01
Right postcentral gyrus	-0.378	0.150	[-0.672 - -0.085]	1.145E-02	2.254E-01
Left precuneus	-0.098	0.141	[-0.375 - 0.179]	4.890E-01	6.807E-01
Right precuneus	-0.081	0.195	[-0.463 - 0.301]	6.784E-01	7.883E-01
Left caudal anterior cingulate cortex	-0.282	0.150	[-0.576 - 0.012]	6.005E-02	3.045E-01
Right caudal anterior cingulate cortex	-0.160	0.141	[-0.437 - 0.117]	2.572E-01	5.355E-01
Left cuneus	-0.042	0.141	[-0.319 - 0.235]	7.650E-01	8.356E-01
Right cuneus	-0.122	0.177	[-0.469 - 0.224]	4.883E-01	6.807E-01
Left rostral anterior cingulate cortex	-0.210	0.190	[-0.582 - 0.162]	2.684E-01	5.355E-01
Right rostral anterior cingulate cortex	-0.104	0.141	[-0.381 - 0.172]	4.592E-01	6.807E-01
Left pericalcarine cortex	-0.430	0.142	[-0.709 - -0.151]	2.537E-03	1.802E-01
Right pericalcarine cortex	-0.405	0.168	[-0.734 - -0.075]	1.604E-02	2.278E-01
Left paracentral lobule	-0.120	0.141	[-0.396 - 0.157]	3.954E-01	6.465E-01
Right paracentral lobule	-0.034	0.141	[-0.310 - 0.242]	8.119E-01	8.477E-01
Left superior parietal cortex	-0.399	0.143	[-0.679 - -0.119]	5.164E-03	1.833E-01
Right superior parietal cortex	-0.201	0.244	[-0.678 - 0.277]	4.097E-01	6.465E-01
Left temporal pole	0.065	0.141	[-0.212 - 0.342]	6.445E-01	7.883E-01
Right temporal pole	0.007	0.141	[-0.270 - 0.285]	9.580E-01	9.580E-01
Left entorhinal cortex	0.135	0.142	[-0.143 - 0.412]	3.419E-01	5.779E-01
Right entorhinal cortex	0.046	0.166	[-0.280 - 0.372]	7.835E-01	8.381E-01

Supplementary Table S117. Cortical thickness differences between individuals with schizophrenia on both first-generation and second-generation antipsychotic medications and healthy comparison subjects controlling for age and sex

	d	Std. Err.	95% CI	*p*-value	FDR *q*-value
Global mean cortical thickness	-0.784	0.105	[-0.991 - -0.578]	9.390E-14	6.061E-13
Left hemisphere	-0.786	0.100	[-0.981 - -0.590]	3.221E-15	4.574E-14
Right hemisphere	-0.756	0.109	[-0.970 - -0.542]	4.639E-12	2.196E-11
Left fusiform gyrus	-0.660	0.084	[-0.825 - -0.495]	5.025E-15	5.946E-14
Right fusiform gyrus	-0.734	0.097	[-0.924 - -0.545]	3.154E-14	2.799E-13
Left pars opercularis of inferior frontal gyrus	-0.745	0.104	[-0.949 - -0.541]	8.635E-13	4.379E-12
Right pars opercularis of inferior frontal gyrus	-0.662	0.088	[-0.835 - -0.490]	4.895E-14	3.476E-13
Left superior temporal gyrus	-0.512	0.077	[-0.662 - -0.362]	2.239E-11	9.352E-11
Right superior temporal gyrus	-0.527	0.092	[-0.708 - -0.346]	1.103E-08	3.132E-08
Left insula	-0.648	0.073	[-0.791 - -0.506]	4.833E-19	1.144E-17
Right insula	-0.582	0.073	[-0.725 - -0.440]	9.847E-16	1.748E-14
Left lingual gyrus	-0.711	0.073	[-0.854 - -0.568]	1.582E-22	1.123E-20
Right lingual gyrus	-0.680	0.073	[-0.822 - -0.537]	9.790E-21	3.475E-19
Left pars triangularis of inferior frontal gyrus	-0.618	0.095	[-0.804 - -0.432]	6.858E-11	2.319E-10
Right pars triangularis of inferior frontal gyrus	-0.615	0.080	[-0.772 - -0.458]	1.580E-14	1.603E-13
Left lateral orbitofrontal cortex	-0.636	0.123	[-0.877 - -0.396]	2.149E-07	4.923E-07
Right lateral orbitofrontal cortex	-0.651	0.090	[-0.827 - -0.474]	4.833E-13	2.639E-12
Left rostral middle frontal gyrus	-0.738	0.124	[-0.981 - -0.495]	2.779E-09	8.222E-09
Right rostral middle frontal gyrus	-0.669	0.124	[-0.913 - -0.425]	7.578E-08	1.921E-07
Left middle temporal gyrus	-0.595	0.089	[-0.769 - -0.420]	2.468E-11	9.735E-11
Right middle temporal gyrus	-0.585	0.121	[-0.822 - -0.348]	1.301E-06	2.792E-06
Left superior frontal gyrus	-0.753	0.112	[-0.972 - -0.533]	1.658E-11	7.357E-11
Right superior frontal gyrus	-0.685	0.104	[-0.888 - -0.482]	4.045E-11	1.512E-10
Left pars orbitalis of inferior frontal gyrus	-0.531	0.099	[-0.725 - -0.337]	8.228E-08	2.014E-07
Right pars orbitalis of inferior frontal gyrus	-0.572	0.102	[-0.772 - -0.373]	1.793E-08	4.896E-08
Left medial orbitofrontal cortex	-0.465	0.107	[-0.675 - -0.255]	1.382E-05	2.180E-05
Right medial orbitofrontal cortex	-0.512	0.113	[-0.732 - -0.291]	5.451E-06	9.676E-06
Left inferior temporal gyrus	-0.533	0.072	[-0.675 - -0.391]	1.876E-13	1.110E-12
Right inferior temporal gyrus	-0.434	0.104	[-0.638 - -0.230]	2.971E-05	4.488E-05
Left isthmus cingulate cortex	-0.473	0.072	[-0.614 - -0.331]	6.125E-11	2.174E-10
Right isthmus cingulate cortex	-0.452	0.085	[-0.619 - -0.286]	9.266E-08	2.193E-07
Left banks of superior temporal sulcus	-0.548	0.073	[-0.690 - -0.406]	4.208E-14	3.320E-13
Right banks of superior temporal sulcus	-0.442	0.101	[-0.641 - -0.243]	1.338E-05	2.158E-05
Left supramarginal gyrus	-0.489	0.103	[-0.690 - -0.287]	2.016E-06	3.868E-06
Right supramarginal gyrus	-0.438	0.094	[-0.622 - -0.255]	2.779E-06	5.192E-06
Left caudal middle frontal gyrus	-0.507	0.093	[-0.689 - -0.324]	5.024E-08	1.321E-07
Right caudal middle frontal gyrus	-0.580	0.089	[-0.754 - -0.405]	7.826E-11	2.526E-10
Left frontal pole	-0.342	0.083	[-0.505 - -0.178]	4.224E-05	6.248E-05
Right frontal pole	-0.399	0.091	[-0.578 - -0.220]	1.287E-05	2.126E-05
Left posterior cingulate cortex	-0.317	0.073	[-0.459 - -0.175]	1.258E-05	2.126E-05
Right posterior cingulate cortex	-0.514	0.108	[-0.726 - -0.303]	1.890E-06	3.727E-06
Left lateral occipital cortex	-0.495	0.100	[-0.690 - -0.299]	6.835E-07	1.517E-06
Right lateral occipital cortex	-0.502	0.080	[-0.659 - -0.344]	4.339E-10	1.339E-09
Left precentral gyrus	-0.383	0.080	[-0.540 - -0.226]	1.763E-06	3.576E-06
Right precentral gyrus	-0.379	0.083	[-0.542 - -0.216]	5.284E-06	9.619E-06
Left parahippocampal gyrus	-0.340	0.092	[-0.520 - -0.161]	2.012E-04	2.747E-04
Right parahippocampal gyrus	-0.244	0.094	[-0.427 - -0.061]	9.138E-03	1.030E-02
Left inferior parietal cortex	-0.449	0.103	[-0.652 - -0.246]	1.425E-05	2.199E-05
Right inferior parietal cortex	-0.331	0.092	[-0.511 - -0.152]	3.007E-04	3.954E-04
Left transverse temporal gyrus	-0.302	0.076	[-0.450 - -0.153]	6.923E-05	9.831E-05
Right transverse temporal gyrus	-0.329	0.073	[-0.472 - -0.185]	6.752E-06	1.169E-05
Left postcentral gyrus	-0.323	0.120	[-0.559 - -0.088]	7.068E-03	8.226E-03
Right postcentral gyrus	-0.363	0.100	[-0.558 - -0.168]	2.700E-04	3.617E-04
Left precuneus	-0.305	0.090	[-0.482 - -0.128]	7.240E-04	9.179E-04
Right precuneus	-0.298	0.091	[-0.476 - -0.121]	9.852E-04	1.227E-03
Left caudal anterior cingulate cortex	-0.230	0.086	[-0.400 - -0.061]	7.729E-03	8.851E-03
Right caudal anterior cingulate cortex	-0.292	0.102	[-0.491 - -0.093]	4.092E-03	4.843E-03
Left cuneus	-0.349	0.072	[-0.491 - -0.208]	1.337E-06	2.792E-06
Right cuneus	-0.318	0.079	[-0.472 - -0.163]	5.713E-05	8.278E-05
Left rostral anterior cingulate cortex	-0.313	0.095	[-0.499 - -0.126]	1.019E-03	1.247E-03
Right rostral anterior cingulate cortex	-0.149	0.082	[-0.310 - 0.011]	6.874E-02	7.284E-02
Left pericalcarine cortex	-0.227	0.072	[-0.368 - -0.085]	1.654E-03	1.990E-03
Right pericalcarine cortex	-0.252	0.072	[-0.393 - -0.111]	4.734E-04	6.111E-04
Left paracentral lobule	-0.164	0.090	[-0.340 - 0.012]	6.819E-02	7.284E-02
Right paracentral lobule	-0.275	0.072	[-0.416 - -0.134]	1.365E-04	1.901E-04
Left superior parietal cortex	-0.246	0.101	[-0.445 - -0.048]	1.479E-02	1.615E-02
Right superior parietal cortex	-0.256	0.103	[-0.459 - -0.054]	1.324E-02	1.469E-02
Left temporal pole	-0.116	0.117	[-0.345 - 0.114]	3.236E-01	3.282E-01
Right temporal pole	-0.077	0.075	[-0.225 - 0.071]	3.076E-01	3.165E-01
Left entorhinal cortex	-0.144	0.082	[-0.306 - 0.017]	7.923E-02	8.272E-02
Right entorhinal cortex	-0.007	0.093	[-0.190 - 0.175]	9.365E-01	9.365E-01

Supplementary Table S118. Cortical thickness differences between individuals with schizophrenia on both first-generation and second-generation antipsychotic medications and individuals with schizophrenia who are unmedicated with antipsychotics controlling for age and sex

	d	Std. Err.	95% CI	*p*-value	FDR *q*-value
Global mean cortical thickness	-0.468	0.187	[-0.834 - -0.102]	1.218E-02	4.322E-02
Left hemisphere	-0.484	0.207	[-0.889 - -0.079]	1.919E-02	5.212E-02
Right hemisphere	-0.445	0.165	[-0.769 - -0.121]	7.160E-03	3.177E-02
Left fusiform gyrus	-0.440	0.206	[-0.844 - -0.037]	3.246E-02	6.778E-02
Right fusiform gyrus	-0.559	0.156	[-0.865 - -0.254]	3.327E-04	7.874E-03
Left pars opercularis of inferior frontal gyrus	-0.485	0.154	[-0.787 - -0.183]	1.641E-03	1.295E-02
Right pars opercularis of inferior frontal gyrus	-0.476	0.172	[-0.814 - -0.138]	5.743E-03	2.718E-02
Left superior temporal gyrus	-0.266	0.171	[-0.601 - 0.070]	1.204E-01	2.009E-01
Right superior temporal gyrus	-0.211	0.145	[-0.495 - 0.073]	1.452E-01	2.343E-01
Left insula	-0.462	0.147	[-0.750 - -0.175]	1.621E-03	1.295E-02
Right insula	-0.467	0.146	[-0.753 - -0.180]	1.422E-03	1.295E-02
Left lingual gyrus	-0.602	0.147	[-0.890 - -0.313]	4.378E-05	1.554E-03
Right lingual gyrus	-0.676	0.149	[-0.967 - -0.385]	5.284E-06	3.751E-04
Left pars triangularis of inferior frontal gyrus	-0.439	0.238	[-0.906 - 0.029]	6.574E-02	1.228E-01
Right pars triangularis of inferior frontal gyrus	-0.519	0.180	[-0.873 - -0.166]	3.980E-03	2.569E-02
Left lateral orbitofrontal cortex	-0.570	0.205	[-0.970 - -0.169]	5.355E-03	2.718E-02
Right lateral orbitofrontal cortex	-0.537	0.166	[-0.862 - -0.212]	1.195E-03	1.295E-02
Left rostral middle frontal gyrus	-0.469	0.205	[-0.871 - -0.068]	2.202E-02	5.212E-02
Right rostral middle frontal gyrus	-0.461	0.197	[-0.847 - -0.076]	1.906E-02	5.212E-02
Left middle temporal gyrus	-0.631	0.224	[-1.069 - -0.192]	4.812E-03	2.718E-02
Right middle temporal gyrus	-0.290	0.214	[-0.708 - 0.129]	1.754E-01	2.767E-01
Left superior frontal gyrus	-0.514	0.208	[-0.921 - -0.107]	1.341E-02	4.493E-02
Right superior frontal gyrus	-0.493	0.185	[-0.856 - -0.130]	7.776E-03	3.195E-02
Left pars orbitalis of inferior frontal gyrus	-0.371	0.168	[-0.701 - -0.042]	2.731E-02	6.097E-02
Right pars orbitalis of inferior frontal gyrus	-0.380	0.173	[-0.720 - -0.041]	2.797E-02	6.097E-02
Left medial orbitofrontal cortex	-0.410	0.169	[-0.742 - -0.079]	1.518E-02	4.687E-02
Right medial orbitofrontal cortex	-0.657	0.222	[-1.092 - -0.223]	3.013E-03	2.139E-02
Left inferior temporal gyrus	-0.682	0.203	[-1.079 - -0.284]	7.757E-04	1.102E-02
Right inferior temporal gyrus	-0.472	0.178	[-0.821 - -0.123]	8.099E-03	3.195E-02
Left isthmus cingulate cortex	-0.502	0.219	[-0.931 - -0.073]	2.176E-02	5.212E-02
Right isthmus cingulate cortex	-0.336	0.252	[-0.830 - 0.159]	1.834E-01	2.831E-01
Left banks of superior temporal sulcus	-0.498	0.146	[-0.785 - -0.211]	6.737E-04	1.102E-02
Right banks of superior temporal sulcus	-0.339	0.155	[-0.642 - -0.036]	2.834E-02	6.097E-02
Left supramarginal gyrus	-0.208	0.173	[-0.548 - 0.131]	2.289E-01	3.317E-01
Right supramarginal gyrus	-0.103	0.177	[-0.449 - 0.243]	5.605E-01	6.861E-01
Left caudal middle frontal gyrus	-0.359	0.146	[-0.646 - -0.073]	1.392E-02	4.493E-02
Right caudal middle frontal gyrus	-0.404	0.157	[-0.712 - -0.097]	1.002E-02	3.746E-02
Left frontal pole	-0.280	0.145	[-0.564 - 0.005]	5.419E-02	1.040E-01
Right frontal pole	-0.282	0.145	[-0.567 - 0.002]	5.201E-02	1.026E-01
Left posterior cingulate cortex	-0.264	0.228	[-0.711 - 0.184]	2.481E-01	3.524E-01
Right posterior cingulate cortex	-0.502	0.181	[-0.857 - -0.147]	5.540E-03	2.718E-02
Left lateral occipital cortex	-0.336	0.146	[-0.622 - -0.051]	2.084E-02	5.212E-02
Right lateral occipital cortex	-0.355	0.148	[-0.645 - -0.065]	1.641E-02	4.853E-02
Left precentral gyrus	-0.089	0.145	[-0.373 - 0.194]	5.367E-01	6.685E-01
Right precentral gyrus	-0.017	0.146	[-0.303 - 0.269]	9.059E-01	9.372E-01
Left parahippocampal gyrus	-0.068	0.217	[-0.493 - 0.357]	7.543E-01	8.638E-01
Right parahippocampal gyrus	-0.038	0.192	[-0.415 - 0.339]	8.444E-01	9.293E-01
Left inferior parietal cortex	-0.286	0.224	[-0.724 - 0.153]	2.016E-01	3.046E-01
Right inferior parietal cortex	-0.162	0.188	[-0.530 - 0.206]	3.888E-01	5.112E-01
Left transverse temporal gyrus	-0.096	0.144	[-0.378 - 0.186]	5.041E-01	6.391E-01
Right transverse temporal gyrus	-0.016	0.144	[-0.298 - 0.266]	9.108E-01	9.372E-01
Left postcentral gyrus	0.058	0.144	[-0.225 - 0.341]	6.895E-01	8.026E-01
Right postcentral gyrus	-0.115	0.144	[-0.398 - 0.167]	4.239E-01	5.472E-01
Left precuneus	-0.294	0.164	[-0.616 - 0.028]	7.369E-02	1.308E-01
Right precuneus	-0.265	0.146	[-0.550 - 0.021]	6.900E-02	1.256E-01
Left caudal anterior cingulate cortex	-0.335	0.146	[-0.620 - -0.049]	2.166E-02	5.212E-02
Right caudal anterior cingulate cortex	-0.130	0.144	[-0.413 - 0.152]	3.664E-01	5.003E-01
Left cuneus	-0.238	0.197	[-0.624 - 0.149]	2.282E-01	3.317E-01
Right cuneus	-0.225	0.145	[-0.510 - 0.060]	1.217E-01	2.009E-01
Left rostral anterior cingulate cortex	-0.431	0.209	[-0.840 - -0.022]	3.906E-02	7.923E-02
Right rostral anterior cingulate cortex	-0.247	0.145	[-0.532 - 0.038]	8.962E-02	1.552E-01
Left pericalcarine cortex	-0.157	0.150	[-0.451 - 0.138]	2.967E-01	4.131E-01
Right pericalcarine cortex	-0.126	0.145	[-0.410 - 0.158]	3.837E-01	5.112E-01
Left paracentral lobule	0.037	0.239	[-0.431 - 0.505]	8.768E-01	9.293E-01
Right paracentral lobule	-0.057	0.199	[-0.447 - 0.333]	7.741E-01	8.724E-01
Left superior parietal cortex	0.025	0.158	[-0.284 - 0.334]	8.752E-01	9.293E-01
Right superior parietal cortex	0.022	0.144	[-0.261 - 0.306]	8.769E-01	9.293E-01
Left temporal pole	-0.087	0.188	[-0.454 - 0.281]	6.441E-01	7.622E-01
Right temporal pole	0.015	0.198	[-0.373 - 0.403]	9.392E-01	9.392E-01
Left entorhinal cortex	-0.021	0.222	[-0.456 - 0.414]	9.244E-01	9.376E-01
Right entorhinal cortex	0.121	0.224	[-0.318 - 0.559]	5.901E-01	7.101E-01

Supplementary Table S119. Cortical thickness differences between individuals with schizophrenia on both first-generation and second-generation antipsychotic medications and individuals with schizophrenia on second-generation antipsychotic medications controlling for age and sex

	d	Std. Err.	95% CI	*p*-value	FDR *q*-value
Global mean cortical thickness	-0.355	0.082	[-0.517 - -0.194]	1.576E-05	1.926E-04
Left hemisphere	-0.368	0.082	[-0.529 - -0.206]	7.749E-06	1.375E-04
Right hemisphere	-0.343	0.094	[-0.527 - -0.160]	2.429E-04	1.314E-03
Left fusiform gyrus	-0.134	0.082	[-0.294 - 0.026]	1.013E-01	1.352E-01
Right fusiform gyrus	-0.244	0.082	[-0.404 - -0.083]	2.959E-03	7.827E-03
Left pars opercularis of inferior frontal gyrus	-0.410	0.082	[-0.571 - -0.249]	6.173E-07	2.191E-05
Right pars opercularis of inferior frontal gyrus	-0.261	0.082	[-0.422 - -0.101]	1.439E-03	4.645E-03
Left superior temporal gyrus	-0.112	0.082	[-0.272 - 0.049]	1.725E-01	2.076E-01
Right superior temporal gyrus	-0.088	0.082	[-0.248 - 0.073]	2.841E-01	3.104E-01
Left insula	-0.272	0.085	[-0.438 - -0.105]	1.395E-03	4.645E-03
Right insula	-0.124	0.110	[-0.340 - 0.091]	2.575E-01	2.857E-01
Left lingual gyrus	-0.344	0.082	[-0.505 - -0.183]	2.796E-05	2.482E-04
Right lingual gyrus	-0.317	0.082	[-0.478 - -0.156]	1.130E-04	8.022E-04
Left pars triangularis of inferior frontal gyrus	-0.291	0.082	[-0.451 - -0.130]	3.930E-04	1.550E-03
Right pars triangularis of inferior frontal gyrus	-0.328	0.082	[-0.489 - -0.167]	6.526E-05	5.149E-04
Left lateral orbitofrontal cortex	-0.287	0.097	[-0.476 - -0.098]	2.977E-03	7.827E-03
Right lateral orbitofrontal cortex	-0.306	0.082	[-0.467 - -0.146]	1.867E-04	1.105E-03
Left rostral middle frontal gyrus	-0.354	0.082	[-0.515 - -0.193]	1.627E-05	1.926E-04
Right rostral middle frontal gyrus	-0.300	0.082	[-0.461 - -0.139]	2.592E-04	1.314E-03
Left middle temporal gyrus	-0.245	0.082	[-0.406 - -0.085]	2.776E-03	7.827E-03
Right middle temporal gyrus	-0.174	0.099	[-0.368 - 0.021]	8.026E-02	1.163E-01
Left superior frontal gyrus	-0.414	0.082	[-0.576 - -0.253]	4.901E-07	2.191E-05
Right superior frontal gyrus	-0.349	0.082	[-0.510 - -0.188]	2.119E-05	2.149E-04
Left pars orbitalis of inferior frontal gyrus	-0.253	0.082	[-0.413 - -0.092]	2.052E-03	6.070E-03
Right pars orbitalis of inferior frontal gyrus	-0.306	0.093	[-0.488 - -0.124]	9.843E-04	3.678E-03
Left medial orbitofrontal cortex	-0.144	0.096	[-0.332 - 0.043]	1.318E-01	1.641E-01
Right medial orbitofrontal cortex	-0.193	0.082	[-0.353 - -0.033]	1.825E-02	3.411E-02
Left inferior temporal gyrus	-0.182	0.082	[-0.342 - -0.021]	2.671E-02	4.515E-02
Right inferior temporal gyrus	-0.108	0.082	[-0.269 - 0.052]	1.847E-01	2.186E-01
Left isthmus cingulate cortex	-0.188	0.082	[-0.348 - -0.027]	2.185E-02	3.879E-02
Right isthmus cingulate cortex	-0.068	0.125	[-0.313 - 0.178]	5.891E-01	5.891E-01
Left banks of superior temporal sulcus	-0.309	0.082	[-0.470 - -0.148]	1.652E-04	1.067E-03
Right banks of superior temporal sulcus	-0.134	0.104	[-0.337 - 0.069]	1.967E-01	2.283E-01
Left supramarginal gyrus	-0.204	0.082	[-0.365 - -0.044]	1.264E-02	2.590E-02
Right supramarginal gyrus	-0.270	0.110	[-0.487 - -0.054]	1.427E-02	2.799E-02
Left caudal middle frontal gyrus	-0.295	0.082	[-0.456 - -0.134]	3.269E-04	1.372E-03
Right caudal middle frontal gyrus	-0.379	0.083	[-0.542 - -0.216]	5.090E-06	1.205E-04
Left frontal pole	-0.142	0.085	[-0.308 - 0.024]	9.353E-02	1.277E-01
Right frontal pole	-0.081	0.082	[-0.242 - 0.079]	3.201E-01	3.443E-01
Left posterior cingulate cortex	-0.167	0.082	[-0.327 - -0.006]	4.155E-02	6.555E-02
Right posterior cingulate cortex	-0.241	0.116	[-0.468 - -0.015]	3.700E-02	5.970E-02
Left lateral occipital cortex	-0.297	0.082	[-0.458 - -0.136]	2.982E-04	1.372E-03
Right lateral occipital cortex	-0.295	0.082	[-0.456 - -0.134]	3.286E-04	1.372E-03
Left precentral gyrus	-0.226	0.082	[-0.387 - -0.066]	5.765E-03	1.412E-02
Right precentral gyrus	-0.213	0.117	[-0.443 - 0.016]	6.816E-02	1.008E-01
Left parahippocampal gyrus	-0.090	0.133	[-0.351 - 0.171]	4.995E-01	5.216E-01
Right parahippocampal gyrus	-0.054	0.098	[-0.246 - 0.138]	5.832E-01	5.891E-01
Left inferior parietal cortex	-0.271	0.093	[-0.453 - -0.090]	3.429E-03	8.695E-03
Right inferior parietal cortex	-0.181	0.093	[-0.363 - 0.001]	5.155E-02	7.956E-02
Left transverse temporal gyrus	-0.129	0.085	[-0.296 - 0.038]	1.300E-01	1.641E-01
Right transverse temporal gyrus	-0.156	0.092	[-0.336 - 0.024]	8.970E-02	1.249E-01
Left postcentral gyrus	-0.190	0.102	[-0.391 - 0.010]	6.310E-02	9.533E-02
Right postcentral gyrus	-0.241	0.148	[-0.530 - 0.049]	1.029E-01	1.352E-01
Left precuneus	-0.189	0.082	[-0.349 - -0.028]	2.107E-02	3.836E-02
Right precuneus	-0.119	0.082	[-0.279 - 0.041]	1.445E-01	1.769E-01
Left caudal anterior cingulate cortex	-0.182	0.082	[-0.343 - -0.022]	2.597E-02	4.497E-02
Right caudal anterior cingulate cortex	-0.140	0.109	[-0.353 - 0.074]	1.994E-01	2.283E-01
Left cuneus	-0.204	0.082	[-0.365 - -0.043]	1.277E-02	2.590E-02
Right cuneus	-0.240	0.092	[-0.420 - -0.060]	8.883E-03	2.035E-02
Left rostral anterior cingulate cortex	-0.257	0.082	[-0.418 - -0.097]	1.688E-03	5.211E-03
Right rostral anterior cingulate cortex	-0.126	0.082	[-0.287 - 0.034]	1.229E-01	1.587E-01
Left pericalcarine cortex	-0.171	0.082	[-0.331 - -0.010]	3.683E-02	5.970E-02
Right pericalcarine cortex	-0.217	0.084	[-0.382 - -0.052]	1.009E-02	2.240E-02
Left paracentral lobule	-0.220	0.082	[-0.381 - -0.060]	7.125E-03	1.686E-02
Right paracentral lobule	-0.268	0.082	[-0.429 - -0.107]	1.082E-03	3.842E-03
Left superior parietal cortex	-0.257	0.102	[-0.457 - -0.058]	1.132E-02	2.435E-02
Right superior parietal cortex	-0.233	0.095	[-0.419 - -0.046]	1.459E-02	2.799E-02
Left temporal pole	-0.050	0.082	[-0.210 - 0.110]	5.392E-01	5.548E-01
Right temporal pole	-0.140	0.082	[-0.301 - 0.020]	8.555E-02	1.215E-01
Left entorhinal cortex	-0.068	0.082	[-0.228 - 0.093]	4.076E-01	4.319E-01
Right entorhinal cortex	0.100	0.082	[-0.060 - 0.260]	2.189E-01	2.467E-01

Supplementary Table S120. Cortical thickness differences between individuals with schizophrenia on both first-generation and second-generation antipsychotic medications and individuals with schizophrenia on first-generation antipsychotic medications controlling for age and sex

	d	Std. Err.	95% CI	*p*-value	FDR *q*-value
Global mean cortical thickness	-0.055	0.248	[-0.541 - 0.431]	8.249E-01	9.876E-01
Left hemisphere	-0.053	0.224	[-0.493 - 0.387]	8.131E-01	9.876E-01
Right hemisphere	-0.062	0.268	[-0.588 - 0.465]	8.187E-01	9.876E-01
Left fusiform gyrus	-0.192	0.144	[-0.474 - 0.089]	1.808E-01	9.876E-01
Right fusiform gyrus	-0.368	0.144	[-0.651 - -0.086]	1.067E-02	7.578E-01
Left pars opercularis of inferior frontal gyrus	-0.024	0.287	[-0.586 - 0.538]	9.329E-01	9.876E-01
Right pars opercularis of inferior frontal gyrus	-0.081	0.301	[-0.670 - 0.509]	7.888E-01	9.876E-01
Left superior temporal gyrus	0.008	0.168	[-0.321 - 0.336]	9.631E-01	9.876E-01
Right superior temporal gyrus	0.201	0.211	[-0.213 - 0.615]	3.409E-01	9.876E-01
Left insula	-0.149	0.222	[-0.585 - 0.287]	5.032E-01	9.876E-01
Right insula	-0.085	0.144	[-0.367 - 0.196]	5.530E-01	9.876E-01
Left lingual gyrus	-0.141	0.144	[-0.423 - 0.141]	3.267E-01	9.876E-01
Right lingual gyrus	-0.275	0.145	[-0.558 - 0.008]	5.702E-02	9.876E-01
Left pars triangularis of inferior frontal gyrus	0.028	0.221	[-0.404 - 0.460]	8.992E-01	9.876E-01
Right pars triangularis of inferior frontal gyrus	0.066	0.219	[-0.363 - 0.496]	7.622E-01	9.876E-01
Left lateral orbitofrontal cortex	0.060	0.217	[-0.365 - 0.485]	7.826E-01	9.876E-01
Right lateral orbitofrontal cortex	-0.045	0.274	[-0.583 - 0.492]	8.683E-01	9.876E-01
Left rostral middle frontal gyrus	-0.035	0.215	[-0.456 - 0.386]	8.707E-01	9.876E-01
Right rostral middle frontal gyrus	-0.117	0.187	[-0.484 - 0.251]	5.336E-01	9.876E-01
Left middle temporal gyrus	-0.203	0.156	[-0.508 - 0.102]	1.915E-01	9.876E-01
Right middle temporal gyrus	-0.183	0.222	[-0.618 - 0.251]	4.085E-01	9.876E-01
Left superior frontal gyrus	-0.188	0.165	[-0.511 - 0.135]	2.544E-01	9.876E-01
Right superior frontal gyrus	-0.028	0.270	[-0.557 - 0.501]	9.172E-01	9.876E-01
Left pars orbitalis of inferior frontal gyrus	-0.007	0.157	[-0.316 - 0.301]	9.635E-01	9.876E-01
Right pars orbitalis of inferior frontal gyrus	-0.267	0.224	[-0.705 - 0.172]	2.337E-01	9.876E-01
Left medial orbitofrontal cortex	-0.030	0.154	[-0.332 - 0.273]	8.479E-01	9.876E-01
Right medial orbitofrontal cortex	-0.046	0.144	[-0.327 - 0.236]	7.515E-01	9.876E-01
Left inferior temporal gyrus	0.006	0.167	[-0.321 - 0.333]	9.690E-01	9.876E-01
Right inferior temporal gyrus	0.025	0.144	[-0.257 - 0.307]	8.643E-01	9.876E-01
Left isthmus cingulate cortex	0.060	0.143	[-0.221 - 0.341]	6.765E-01	9.876E-01
Right isthmus cingulate cortex	-0.082	0.160	[-0.395 - 0.231]	6.080E-01	9.876E-01
Left banks of superior temporal sulcus	-0.249	0.144	[-0.531 - 0.032]	8.285E-02	9.876E-01
Right banks of superior temporal sulcus	-0.077	0.144	[-0.359 - 0.205]	5.938E-01	9.876E-01
Left supramarginal gyrus	-0.096	0.281	[-0.647 - 0.456]	7.336E-01	9.876E-01
Right supramarginal gyrus	-0.098	0.206	[-0.501 - 0.305]	6.327E-01	9.876E-01
Left caudal middle frontal gyrus	-0.050	0.208	[-0.457 - 0.357]	8.094E-01	9.876E-01
Right caudal middle frontal gyrus	-0.074	0.183	[-0.433 - 0.285]	6.860E-01	9.876E-01
Left frontal pole	0.080	0.207	[-0.326 - 0.485]	6.997E-01	9.876E-01
Right frontal pole	0.078	0.211	[-0.335 - 0.492]	7.107E-01	9.876E-01
Left posterior cingulate cortex	0.194	0.148	[-0.097 - 0.484]	1.919E-01	9.876E-01
Right posterior cingulate cortex	-0.142	0.204	[-0.542 - 0.258]	4.861E-01	9.876E-01
Left lateral occipital cortex	-0.038	0.159	[-0.349 - 0.274]	8.120E-01	9.876E-01
Right lateral occipital cortex	0.073	0.299	[-0.514 - 0.660]	8.075E-01	9.876E-01
Left precentral gyrus	-0.195	0.201	[-0.588 - 0.198]	3.318E-01	9.876E-01
Right precentral gyrus	-0.187	0.223	[-0.623 - 0.249]	4.016E-01	9.876E-01
Left parahippocampal gyrus	-0.056	0.143	[-0.337 - 0.225]	6.973E-01	9.876E-01
Right parahippocampal gyrus	-0.070	0.144	[-0.352 - 0.212]	6.263E-01	9.876E-01
Left inferior parietal cortex	-0.042	0.208	[-0.449 - 0.365]	8.404E-01	9.876E-01
Right inferior parietal cortex	0.094	0.187	[-0.272 - 0.460]	6.152E-01	9.876E-01
Left transverse temporal gyrus	0.202	0.251	[-0.291 - 0.695]	4.218E-01	9.876E-01
Right transverse temporal gyrus	0.110	0.227	[-0.336 - 0.555]	6.292E-01	9.876E-01
Left postcentral gyrus	-0.026	0.337	[-0.685 - 0.634]	9.393E-01	9.876E-01
Right postcentral gyrus	-0.132	0.280	[-0.681 - 0.417]	6.373E-01	9.876E-01
Left precuneus	-0.118	0.144	[-0.399 - 0.164]	4.120E-01	9.876E-01
Right precuneus	-0.009	0.263	[-0.525 - 0.507]	9.737E-01	9.876E-01
Left caudal anterior cingulate cortex	0.001	0.189	[-0.369 - 0.372]	9.945E-01	9.945E-01
Right caudal anterior cingulate cortex	-0.156	0.144	[-0.438 - 0.126]	2.782E-01	9.876E-01
Left cuneus	-0.255	0.144	[-0.538 - 0.027]	7.656E-02	9.876E-01
Right cuneus	-0.063	0.169	[-0.394 - 0.268]	7.092E-01	9.876E-01
Left rostral anterior cingulate cortex	-0.212	0.144	[-0.494 - 0.071]	1.417E-01	9.876E-01
Right rostral anterior cingulate cortex	-0.024	0.144	[-0.306 - 0.257]	8.657E-01	9.876E-01
Left pericalcarine cortex	0.079	0.143	[-0.202 - 0.360]	5.802E-01	9.876E-01
Right pericalcarine cortex	0.169	0.219	[-0.261 - 0.599]	4.420E-01	9.876E-01
Left paracentral lobule	-0.030	0.180	[-0.384 - 0.323]	8.665E-01	9.876E-01
Right paracentral lobule	-0.243	0.144	[-0.526 - 0.039]	9.156E-02	9.876E-01
Left superior parietal cortex	-0.031	0.278	[-0.575 - 0.514]	9.122E-01	9.876E-01
Right superior parietal cortex	-0.157	0.367	[-0.876 - 0.561]	6.675E-01	9.876E-01
Left temporal pole	-0.045	0.144	[-0.327 - 0.237]	7.529E-01	9.876E-01
Right temporal pole	-0.113	0.144	[-0.395 - 0.169]	4.322E-01	9.876E-01
Left entorhinal cortex	-0.197	0.144	[-0.480 - 0.085]	1.713E-01	9.876E-01
Right entorhinal cortex	0.074	0.212	[-0.341 - 0.489]	7.270E-01	9.876E-01

Supplementary Table S121. Partial correlations between cortical thickness and chlorpromazine equivalents controlling for age and sex in individuals with schizophrenia who are medicated with antipsychotics

	Partial *R*	Std. Err.	95% CI	*p*-value	FDR *q*-value
Global mean cortical thickness	-0.165	0.034	[-0.233 - -0.098]	1.574E-06	2.793E-05
Left hemisphere	-0.160	0.033	[-0.226 - -0.095]	1.422E-06	2.793E-05
Right hemisphere	-0.166	0.034	[-0.234 - -0.099]	1.344E-06	2.793E-05
Left fusiform gyrus	-0.147	0.032	[-0.210 - -0.084]	4.459E-06	6.060E-05
Right fusiform gyrus	-0.165	0.032	[-0.227 - -0.102]	2.657E-07	1.887E-05
Left pars opercularis of inferior frontal gyrus	-0.125	0.038	[-0.199 - -0.051]	9.580E-04	2.194E-03
Right pars opercularis of inferior frontal gyrus	-0.116	0.032	[-0.179 - -0.053]	2.984E-04	1.009E-03
Left superior temporal gyrus	-0.124	0.032	[-0.187 - -0.062]	1.018E-04	5.165E-04
Right superior temporal gyrus	-0.089	0.038	[-0.163 - -0.014]	2.001E-02	3.088E-02
Left insula	-0.146	0.032	[-0.209 - -0.083]	5.121E-06	6.060E-05
Right insula	-0.140	0.039	[-0.216 - -0.064]	3.179E-04	1.026E-03
Left lingual gyrus	-0.097	0.032	[-0.160 - -0.035]	2.321E-03	4.708E-03
Right lingual gyrus	-0.098	0.033	[-0.162 - -0.034]	2.605E-03	4.998E-03
Left pars triangularis of inferior frontal gyrus	-0.119	0.032	[-0.182 - -0.057]	1.952E-04	7.700E-04
Right pars triangularis of inferior frontal gyrus	-0.155	0.037	[-0.227 - -0.083]	2.640E-05	1.874E-04
Left lateral orbitofrontal cortex	-0.135	0.034	[-0.202 - -0.068]	8.240E-05	4.500E-04
Right lateral orbitofrontal cortex	-0.151	0.035	[-0.219 - -0.083]	1.438E-05	1.294E-04
Left rostral middle frontal gyrus	-0.115	0.032	[-0.179 - -0.052]	3.705E-04	1.052E-03
Right rostral middle frontal gyrus	-0.124	0.037	[-0.197 - -0.051]	9.398E-04	2.194E-03
Left middle temporal gyrus	-0.084	0.044	[-0.171 - 0.003]	5.946E-02	7.406E-02
Right middle temporal gyrus	-0.119	0.032	[-0.182 - -0.057]	1.927E-04	7.700E-04
Left superior frontal gyrus	-0.165	0.039	[-0.241 - -0.090]	1.828E-05	1.442E-04
Right superior frontal gyrus	-0.162	0.042	[-0.245 - -0.079]	1.361E-04	6.041E-04
Left pars orbitalis of inferior frontal gyrus	-0.114	0.032	[-0.177 - -0.051]	3.630E-04	1.052E-03
Right pars orbitalis of inferior frontal gyrus	-0.109	0.037	[-0.180 - -0.037]	2.903E-03	5.285E-03
Left medial orbitofrontal cortex	-0.123	0.032	[-0.186 - -0.060]	1.200E-04	5.682E-04
Right medial orbitofrontal cortex	-0.070	0.032	[-0.133 - -0.008]	2.770E-02	4.184E-02
Left inferior temporal gyrus	-0.103	0.043	[-0.187 - -0.019]	1.636E-02	2.581E-02
Right inferior temporal gyrus	-0.127	0.032	[-0.190 - -0.064]	7.086E-05	4.192E-04
Left isthmus cingulate cortex	-0.100	0.034	[-0.166 - -0.033]	3.222E-03	5.720E-03
Right isthmus cingulate cortex	-0.094	0.043	[-0.179 - -0.010]	2.919E-02	4.230E-02
Left banks of superior temporal sulcus	-0.101	0.038	[-0.175 - -0.027]	7.673E-03	1.267E-02
Right banks of superior temporal sulcus	-0.097	0.032	[-0.160 - -0.034]	2.468E-03	4.867E-03
Left supramarginal gyrus	-0.090	0.032	[-0.153 - -0.028]	4.798E-03	8.309E-03
Right supramarginal gyrus	-0.093	0.042	[-0.176 - -0.010]	2.841E-02	4.202E-02
Left caudal middle frontal gyrus	-0.117	0.032	[-0.180 - -0.054]	2.563E-04	9.097E-04
Right caudal middle frontal gyrus	-0.144	0.036	[-0.213 - -0.074]	5.204E-05	3.359E-04
Left frontal pole	-0.062	0.032	[-0.125 - 0.001]	5.354E-02	6.911E-02
Right frontal pole	-0.086	0.053	[-0.190 - 0.019]	1.089E-01	1.268E-01
Left posterior cingulate cortex	-0.115	0.032	[-0.177 - -0.052]	3.461E-04	1.052E-03
Right posterior cingulate cortex	-0.107	0.039	[-0.184 - -0.031]	6.187E-03	1.046E-02
Left lateral occipital cortex	-0.139	0.032	[-0.201 - -0.076]	1.458E-05	1.294E-04
Right lateral occipital cortex	-0.123	0.033	[-0.188 - -0.058]	2.064E-04	7.712E-04
Left precentral gyrus	-0.069	0.038	[-0.143 - 0.005]	6.935E-02	8.490E-02
Right precentral gyrus	-0.109	0.032	[-0.172 - -0.046]	6.756E-04	1.777E-03
Left parahippocampal gyrus	-0.053	0.045	[-0.142 - 0.035]	2.385E-01	2.605E-01
Right parahippocampal gyrus	-0.086	0.043	[-0.169 - -0.002]	4.383E-02	5.762E-02
Left inferior parietal cortex	-0.074	0.049	[-0.170 - 0.021]	1.277E-01	1.462E-01
Right inferior parietal cortex	-0.095	0.032	[-0.158 - -0.033]	2.864E-03	5.285E-03
Left transverse temporal gyrus	-0.084	0.032	[-0.147 - -0.022]	8.480E-03	1.368E-02
Right transverse temporal gyrus	-0.055	0.044	[-0.142 - 0.031]	2.095E-01	2.324E-01
Left postcentral gyrus	-0.061	0.032	[-0.124 - 0.002]	5.623E-02	7.129E-02
Right postcentral gyrus	-0.069	0.032	[-0.131 - -0.006]	3.180E-02	4.490E-02
Left precuneus	-0.109	0.032	[-0.172 - -0.047]	6.425E-04	1.754E-03
Right precuneus	-0.094	0.046	[-0.184 - -0.003]	4.262E-02	5.710E-02
Left caudal anterior cingulate cortex	-0.107	0.032	[-0.170 - -0.044]	8.263E-04	2.023E-03
Right caudal anterior cingulate cortex	-0.086	0.048	[-0.179 - 0.008]	7.205E-02	8.671E-02
Left cuneus	-0.108	0.035	[-0.177 - -0.039]	2.176E-03	4.544E-03
Right cuneus	-0.142	0.043	[-0.227 - -0.057]	1.028E-03	2.280E-03
Left rostral anterior cingulate cortex	-0.103	0.032	[-0.166 - -0.040]	1.263E-03	2.717E-03
Right rostral anterior cingulate cortex	-0.058	0.039	[-0.135 - 0.018]	1.358E-01	1.531E-01
Left pericalcarine cortex	-0.036	0.048	[-0.130 - 0.058]	4.489E-01	4.757E-01
Right pericalcarine cortex	-0.023	0.032	[-0.086 - 0.040]	4.729E-01	4.938E-01
Left paracentral lobule	-0.082	0.039	[-0.158 - -0.007]	3.225E-02	4.490E-02
Right paracentral lobule	-0.108	0.032	[-0.171 - -0.045]	7.532E-04	1.910E-03
Left superior parietal cortex	-0.062	0.039	[-0.138 - 0.013]	1.067E-01	1.263E-01
Right superior parietal cortex	-0.067	0.032	[-0.130 - -0.004]	3.645E-02	4.978E-02
Left temporal pole	-0.031	0.038	[-0.105 - 0.044]	4.178E-01	4.494E-01
Right temporal pole	-0.021	0.043	[-0.105 - 0.064]	6.298E-01	6.388E-01
Left entorhinal cortex	-0.007	0.042	[-0.089 - 0.075]	8.717E-01	8.717E-01
Right entorhinal cortex	0.019	0.032	[-0.043 - 0.082]	5.432E-01	5.590E-01

Supplementary Table S122. Cortical thickness differences between individuals with bipolar disorder taking lithium carbonate and individuals with bipolar disorder not taking lithium carbonate controlling for age, sex, and other medications

	d	Std. Err.	95% CI	*p*-value	FDR *q*-value
Global mean cortical thickness	-0.071	0.162	[-0.388 - 0.246]	6.597E-01	9.492E-01
Left hemisphere	-0.071	0.170	[-0.403 - 0.262]	6.772E-01	9.492E-01
Right hemisphere	-0.069	0.157	[-0.377 - 0.240]	6.629E-01	9.492E-01
Left fusiform gyrus	-0.246	0.230	[-0.697 - 0.205]	2.847E-01	9.492E-01
Right fusiform gyrus	-0.100	0.222	[-0.535 - 0.335]	6.527E-01	9.492E-01
Left pars opercularis of inferior frontal gyrus	-0.272	0.151	[-0.568 - 0.024]	7.199E-02	5.783E-01
Right pars opercularis of inferior frontal gyrus	-0.121	0.142	[-0.400 - 0.157]	3.931E-01	9.492E-01
Left superior temporal gyrus	-0.085	0.165	[-0.408 - 0.237]	6.033E-01	9.492E-01
Right superior temporal gyrus	-0.139	0.251	[-0.631 - 0.352]	5.782E-01	9.492E-01
Left insula	-0.031	0.238	[-0.498 - 0.435]	8.957E-01	9.492E-01
Right insula	0.189	0.283	[-0.365 - 0.742]	5.044E-01	9.492E-01
Left lingual gyrus	0.021	0.277	[-0.521 - 0.563]	9.395E-01	9.529E-01
Right lingual gyrus	0.140	0.227	[-0.305 - 0.584]	5.383E-01	9.492E-01
Left pars triangularis of inferior frontal gyrus	-0.413	0.143	[-0.694 - -0.132]	3.951E-03	1.946E-01
Right pars triangularis of inferior frontal gyrus	-0.397	0.211	[-0.810 - 0.016]	5.980E-02	5.783E-01
Left lateral orbitofrontal cortex	0.072	0.223	[-0.366 - 0.510]	7.484E-01	9.492E-01
Right lateral orbitofrontal cortex	0.146	0.273	[-0.390 - 0.682]	5.922E-01	9.492E-01
Left rostral middle frontal gyrus	0.041	0.142	[-0.237 - 0.320]	7.709E-01	9.492E-01
Right rostral middle frontal gyrus	-0.119	0.151	[-0.416 - 0.177]	4.304E-01	9.492E-01
Left middle temporal gyrus	0.033	0.142	[-0.244 - 0.310]	8.154E-01	9.492E-01
Right middle temporal gyrus	-0.216	0.259	[-0.723 - 0.292]	4.047E-01	9.492E-01
Left superior frontal gyrus	-0.130	0.236	[-0.592 - 0.333]	5.828E-01	9.492E-01
Right superior frontal gyrus	-0.152	0.142	[-0.431 - 0.127]	2.858E-01	9.492E-01
Left pars orbitalis of inferior frontal gyrus	-0.062	0.141	[-0.339 - 0.215]	6.621E-01	9.492E-01
Right pars orbitalis of inferior frontal gyrus	-0.399	0.144	[-0.680 - -0.117]	5.482E-03	1.946E-01
Left medial orbitofrontal cortex	-0.085	0.175	[-0.427 - 0.257]	6.262E-01	9.492E-01
Right medial orbitofrontal cortex	-0.022	0.142	[-0.301 - 0.257]	8.751E-01	9.492E-01
Left inferior temporal gyrus	0.039	0.142	[-0.239 - 0.316]	7.847E-01	9.492E-01
Right inferior temporal gyrus	-0.040	0.223	[-0.476 - 0.397]	8.585E-01	9.492E-01
Left isthmus cingulate cortex	0.235	0.143	[-0.044 - 0.515]	9.904E-02	7.032E-01
Right isthmus cingulate cortex	0.009	0.172	[-0.329 - 0.347]	9.599E-01	9.599E-01
Left banks of superior temporal sulcus	-0.256	0.143	[-0.535 - 0.024]	7.330E-02	5.783E-01
Right banks of superior temporal sulcus	-0.273	0.142	[-0.553 - 0.006]	5.490E-02	5.783E-01
Left supramarginal gyrus	-0.410	0.182	[-0.766 - -0.053]	2.419E-02	4.294E-01
Right supramarginal gyrus	-0.047	0.181	[-0.403 - 0.308]	7.949E-01	9.492E-01
Left caudal middle frontal gyrus	-0.138	0.211	[-0.552 - 0.276]	5.150E-01	9.492E-01
Right caudal middle frontal gyrus	-0.066	0.142	[-0.344 - 0.212]	6.418E-01	9.492E-01
Left frontal pole	-0.194	0.265	[-0.713 - 0.326]	4.649E-01	9.492E-01
Right frontal pole	-0.345	0.143	[-0.626 - -0.064]	1.599E-02	3.784E-01
Left posterior cingulate cortex	0.291	0.305	[-0.307 - 0.888]	3.402E-01	9.492E-01
Right posterior cingulate cortex	0.187	0.201	[-0.207 - 0.580]	3.525E-01	9.492E-01
Left lateral occipital cortex	0.013	0.157	[-0.295 - 0.321]	9.347E-01	9.529E-01
Right lateral occipital cortex	0.054	0.142	[-0.225 - 0.332]	7.053E-01	9.492E-01
Left precentral gyrus	0.124	0.144	[-0.158 - 0.406]	3.900E-01	9.492E-01
Right precentral gyrus	0.025	0.142	[-0.254 - 0.304]	8.596E-01	9.492E-01
Left parahippocampal gyrus	-0.118	0.142	[-0.396 - 0.161]	4.078E-01	9.492E-01
Right parahippocampal gyrus	-0.125	0.182	[-0.482 - 0.231]	4.911E-01	9.492E-01
Left inferior parietal cortex	-0.171	0.142	[-0.449 - 0.107]	2.279E-01	9.492E-01
Right inferior parietal cortex	-0.081	0.142	[-0.359 - 0.198]	5.688E-01	9.492E-01
Left transverse temporal gyrus	0.213	0.142	[-0.066 - 0.491]	1.354E-01	8.009E-01
Right transverse temporal gyrus	-0.100	0.234	[-0.559 - 0.359]	6.688E-01	9.492E-01
Left postcentral gyrus	-0.017	0.176	[-0.361 - 0.327]	9.239E-01	9.529E-01
Right postcentral gyrus	0.057	0.234	[-0.403 - 0.516]	8.081E-01	9.492E-01
Left precuneus	-0.051	0.142	[-0.329 - 0.228]	7.214E-01	9.492E-01
Right precuneus	-0.116	0.142	[-0.394 - 0.162]	4.119E-01	9.492E-01
Left caudal anterior cingulate cortex	-0.039	0.141	[-0.316 - 0.238]	7.825E-01	9.492E-01
Right caudal anterior cingulate cortex	0.168	0.142	[-0.110 - 0.447]	2.357E-01	9.492E-01
Left cuneus	-0.165	0.219	[-0.594 - 0.264]	4.498E-01	9.492E-01
Right cuneus	-0.453	0.217	[-0.879 - -0.027]	3.717E-02	5.278E-01
Left rostral anterior cingulate cortex	0.144	0.168	[-0.185 - 0.473]	3.910E-01	9.492E-01
Right rostral anterior cingulate cortex	0.363	0.237	[-0.102 - 0.827]	1.260E-01	8.009E-01
Left pericalcarine cortex	0.024	0.172	[-0.314 - 0.361]	8.903E-01	9.492E-01
Right pericalcarine cortex	-0.190	0.155	[-0.493 - 0.113]	2.195E-01	9.492E-01
Left paracentral lobule	0.033	0.142	[-0.246 - 0.312]	8.169E-01	9.492E-01
Right paracentral lobule	0.074	0.142	[-0.205 - 0.353]	6.019E-01	9.492E-01
Left superior parietal cortex	-0.059	0.150	[-0.353 - 0.236]	6.969E-01	9.492E-01
Right superior parietal cortex	-0.104	0.142	[-0.382 - 0.174]	4.622E-01	9.492E-01
Left temporal pole	-0.267	0.256	[-0.770 - 0.235]	2.966E-01	9.492E-01
Right temporal pole	-0.185	0.345	[-0.860 - 0.491]	5.919E-01	9.492E-01
Left entorhinal cortex	-0.050	0.142	[-0.328 - 0.227]	7.216E-01	9.492E-01
Right entorhinal cortex	0.024	0.142	[-0.255 - 0.303]	8.662E-01	9.492E-01

Supplementary Table S123. Cortical thickness differences between individuals with bipolar disorder taking lithium carbonate and individuals with bipolar disorder not taking lithium carbonate controlling for age, sex, and other medications at 25 years of age or older

	d	Std. Err.	95% CI	*p*-value	FDR *q*-value
Global mean cortical thickness	-0.128	0.185	[-0.491 - 0.234]	4.869E-01	8.363E-01
Left hemisphere	-0.133	0.201	[-0.528 - 0.261]	5.076E-01	8.363E-01
Right hemisphere	-0.119	0.170	[-0.451 - 0.213]	4.832E-01	8.363E-01
Left fusiform gyrus	-0.196	0.230	[-0.646 - 0.254]	3.922E-01	8.363E-01
Right fusiform gyrus	-0.115	0.208	[-0.522 - 0.293]	5.811E-01	8.363E-01
Left pars opercularis of inferior frontal gyrus	-0.234	0.166	[-0.558 - 0.091]	1.583E-01	7.054E-01
Right pars opercularis of inferior frontal gyrus	-0.021	0.189	[-0.392 - 0.350]	9.118E-01	9.952E-01
Left superior temporal gyrus	0.051	0.203	[-0.348 - 0.449]	8.034E-01	9.507E-01
Right superior temporal gyrus	-0.151	0.278	[-0.696 - 0.393]	5.861E-01	8.363E-01
Left insula	-0.033	0.250	[-0.524 - 0.458]	8.953E-01	9.932E-01
Right insula	0.169	0.312	[-0.443 - 0.780]	5.889E-01	8.363E-01
Left lingual gyrus	-0.010	0.296	[-0.589 - 0.570]	9.739E-01	9.952E-01
Right lingual gyrus	0.142	0.219	[-0.287 - 0.571]	5.177E-01	8.363E-01
Left pars triangularis of inferior frontal gyrus	-0.427	0.159	[-0.739 - -0.115]	7.270E-03	2.988E-01
Right pars triangularis of inferior frontal gyrus	-0.436	0.248	[-0.922 - 0.050]	7.847E-02	7.054E-01
Left lateral orbitofrontal cortex	0.072	0.216	[-0.351 - 0.495]	7.394E-01	9.378E-01
Right lateral orbitofrontal cortex	0.213	0.316	[-0.406 - 0.833]	4.992E-01	8.363E-01
Left rostral middle frontal gyrus	0.047	0.149	[-0.244 - 0.338]	7.529E-01	9.378E-01
Right rostral middle frontal gyrus	-0.135	0.149	[-0.426 - 0.156]	3.622E-01	8.363E-01
Left middle temporal gyrus	0.008	0.148	[-0.282 - 0.298]	9.571E-01	9.952E-01
Right middle temporal gyrus	-0.289	0.271	[-0.819 - 0.242]	2.859E-01	8.363E-01
Left superior frontal gyrus	-0.178	0.279	[-0.725 - 0.369]	5.233E-01	8.363E-01
Right superior frontal gyrus	-0.179	0.149	[-0.471 - 0.112]	2.276E-01	7.345E-01
Left pars orbitalis of inferior frontal gyrus	-0.049	0.148	[-0.338 - 0.240]	7.414E-01	9.378E-01
Right pars orbitalis of inferior frontal gyrus	-0.351	0.248	[-0.837 - 0.136]	1.581E-01	7.054E-01
Left medial orbitofrontal cortex	-0.102	0.169	[-0.433 - 0.229]	5.451E-01	8.363E-01
Right medial orbitofrontal cortex	-0.099	0.154	[-0.402 - 0.203]	5.197E-01	8.363E-01
Left inferior temporal gyrus	0.082	0.148	[-0.209 - 0.372]	5.819E-01	8.363E-01
Right inferior temporal gyrus	-0.074	0.230	[-0.524 - 0.377]	7.487E-01	9.378E-01
Left isthmus cingulate cortex	0.223	0.149	[-0.069 - 0.514]	1.344E-01	7.054E-01
Right isthmus cingulate cortex	0.013	0.221	[-0.421 - 0.447]	9.547E-01	9.952E-01
Left banks of superior temporal sulcus	-0.201	0.152	[-0.500 - 0.098]	1.873E-01	7.100E-01
Right banks of superior temporal sulcus	-0.394	0.150	[-0.687 - -0.101]	8.416E-03	2.988E-01
Left supramarginal gyrus	-0.601	0.243	[-1.076 - -0.125]	1.325E-02	3.135E-01
Right supramarginal gyrus	-0.058	0.179	[-0.410 - 0.293]	7.452E-01	9.378E-01
Left caudal middle frontal gyrus	-0.218	0.234	[-0.677 - 0.241]	3.513E-01	8.363E-01
Right caudal middle frontal gyrus	-0.189	0.148	[-0.479 - 0.101]	2.019E-01	7.100E-01
Left frontal pole	-0.196	0.273	[-0.732 - 0.340]	4.736E-01	8.363E-01
Right frontal pole	-0.341	0.149	[-0.634 - -0.049]	2.231E-02	3.960E-01
Left posterior cingulate cortex	0.307	0.349	[-0.378 - 0.992]	3.800E-01	8.363E-01
Right posterior cingulate cortex	0.124	0.189	[-0.247 - 0.495]	5.114E-01	8.363E-01
Left lateral occipital cortex	-0.168	0.229	[-0.616 - 0.281]	4.637E-01	8.363E-01
Right lateral occipital cortex	-0.009	0.148	[-0.300 - 0.282]	9.495E-01	9.952E-01
Left precentral gyrus	0.095	0.148	[-0.195 - 0.386]	5.195E-01	8.363E-01
Right precentral gyrus	0.034	0.157	[-0.274 - 0.342]	8.294E-01	9.519E-01
Left parahippocampal gyrus	-0.209	0.149	[-0.500 - 0.082]	1.590E-01	7.054E-01
Right parahippocampal gyrus	-0.243	0.185	[-0.606 - 0.120]	1.888E-01	7.100E-01
Left inferior parietal cortex	-0.281	0.149	[-0.572 - 0.011]	5.925E-02	7.054E-01
Right inferior parietal cortex	-0.251	0.166	[-0.577 - 0.075]	1.320E-01	7.054E-01
Left transverse temporal gyrus	0.228	0.149	[-0.064 - 0.519]	1.259E-01	7.054E-01
Right transverse temporal gyrus	-0.208	0.263	[-0.724 - 0.307]	4.285E-01	8.363E-01
Left postcentral gyrus	-0.001	0.177	[-0.347 - 0.345]	9.952E-01	9.952E-01
Right postcentral gyrus	0.055	0.200	[-0.336 - 0.447]	7.818E-01	9.499E-01
Left precuneus	-0.032	0.151	[-0.328 - 0.264]	8.313E-01	9.519E-01
Right precuneus	-0.132	0.148	[-0.422 - 0.158]	3.736E-01	8.363E-01
Left caudal anterior cingulate cortex	-0.027	0.148	[-0.317 - 0.263]	8.543E-01	9.628E-01
Right caudal anterior cingulate cortex	0.157	0.148	[-0.134 - 0.447]	2.912E-01	8.363E-01
Left cuneus	-0.133	0.222	[-0.568 - 0.302]	5.495E-01	8.363E-01
Right cuneus	-0.432	0.243	[-0.908 - 0.044]	7.510E-02	7.054E-01
Left rostral anterior cingulate cortex	0.136	0.180	[-0.217 - 0.489]	4.497E-01	8.363E-01
Right rostral anterior cingulate cortex	0.382	0.266	[-0.139 - 0.903]	1.504E-01	7.054E-01
Left pericalcarine cortex	-0.072	0.149	[-0.363 - 0.219]	6.288E-01	8.754E-01
Right pericalcarine cortex	-0.228	0.150	[-0.522 - 0.067]	1.298E-01	7.054E-01
Left paracentral lobule	-0.001	0.148	[-0.292 - 0.289]	9.931E-01	9.952E-01
Right paracentral lobule	0.058	0.148	[-0.233 - 0.348]	6.980E-01	9.378E-01
Left superior parietal cortex	-0.240	0.185	[-0.603 - 0.123]	1.942E-01	7.100E-01
Right superior parietal cortex	-0.186	0.148	[-0.476 - 0.105]	2.100E-01	7.100E-01
Left temporal pole	-0.418	0.251	[-0.911 - 0.074]	9.606E-02	7.054E-01
Right temporal pole	-0.245	0.341	[-0.913 - 0.423]	4.727E-01	8.363E-01
Left entorhinal cortex	-0.151	0.148	[-0.442 - 0.139]	3.070E-01	8.363E-01
Right entorhinal cortex	-0.049	0.183	[-0.407 - 0.309]	7.893E-01	9.499E-01

Supplementary Table S124. Cortical thickness differences between individuals with bipolar disorder taking sodium valproate and individuals with bipolar disorder not taking sodium valproate controlling for age, sex, and other medications

	d	Std. Err.	95% CI	*p*-value	FDR *q*-value
Global mean cortical thickness	-0.755	0.190	[-1.127 - -0.383]	6.880E-05	8.141E-04
Left hemisphere	-0.698	0.189	[-1.069 - -0.327]	2.283E-04	2.026E-03
Right hemisphere	-0.786	0.190	[-1.159 - -0.414]	3.500E-05	5.832E-04
Left fusiform gyrus	-0.756	0.287	[-1.320 - -0.193]	8.491E-03	2.444E-02
Right fusiform gyrus	-0.170	0.310	[-0.778 - 0.438]	5.838E-01	6.579E-01
Left pars opercularis of inferior frontal gyrus	-0.349	0.221	[-0.783 - 0.085]	1.150E-01	1.737E-01
Right pars opercularis of inferior frontal gyrus	-0.327	0.185	[-0.690 - 0.036]	7.714E-02	1.336E-01
Left superior temporal gyrus	0.003	0.277	[-0.540 - 0.547]	9.904E-01	9.904E-01
Right superior temporal gyrus	-0.093	0.482	[-1.038 - 0.851]	8.464E-01	8.883E-01
Left insula	-0.565	0.336	[-1.224 - 0.093]	9.249E-02	1.563E-01
Right insula	-0.734	0.256	[-1.237 - -0.232]	4.159E-03	1.554E-02
Left lingual gyrus	-0.647	0.189	[-1.016 - -0.277]	6.034E-04	4.284E-03
Right lingual gyrus	-0.572	0.187	[-0.938 - -0.205]	2.261E-03	1.147E-02
Left pars triangularis of inferior frontal gyrus	-0.299	0.188	[-0.667 - 0.069]	1.110E-01	1.737E-01
Right pars triangularis of inferior frontal gyrus	-0.477	0.186	[-0.842 - -0.112]	1.042E-02	2.643E-02
Left lateral orbitofrontal cortex	-0.459	0.186	[-0.824 - -0.094]	1.372E-02	3.358E-02
Right lateral orbitofrontal cortex	-0.359	0.248	[-0.844 - 0.126]	1.472E-01	2.105E-01
Left rostral middle frontal gyrus	-0.216	0.311	[-0.825 - 0.393]	4.875E-01	5.582E-01
Right rostral middle frontal gyrus	-0.480	0.187	[-0.847 - -0.113]	1.028E-02	2.643E-02
Left middle temporal gyrus	-0.444	0.186	[-0.808 - -0.079]	1.716E-02	3.996E-02
Right middle temporal gyrus	-0.540	0.187	[-0.907 - -0.174]	3.865E-03	1.524E-02
Left superior frontal gyrus	-0.541	0.187	[-0.908 - -0.174]	3.858E-03	1.524E-02
Right superior frontal gyrus	-0.538	0.207	[-0.943 - -0.133]	9.269E-03	2.531E-02
Left pars orbitalis of inferior frontal gyrus	-0.354	0.191	[-0.729 - 0.020]	6.379E-02	1.192E-01
Right pars orbitalis of inferior frontal gyrus	-0.285	0.287	[-0.847 - 0.277]	3.210E-01	4.144E-01
Left medial orbitofrontal cortex	-0.409	0.219	[-0.838 - 0.019]	6.109E-02	1.172E-01
Right medial orbitofrontal cortex	-0.422	0.186	[-0.786 - -0.058]	2.306E-02	4.961E-02
Left inferior temporal gyrus	-0.507	0.187	[-0.873 - -0.140]	6.756E-03	2.180E-02
Right inferior temporal gyrus	-0.627	0.208	[-1.035 - -0.220]	2.567E-03	1.215E-02
Left isthmus cingulate cortex	-0.626	0.238	[-1.093 - -0.159]	8.605E-03	2.444E-02
Right isthmus cingulate cortex	-0.607	0.256	[-1.108 - -0.107]	1.745E-02	3.996E-02
Left banks of superior temporal sulcus	-0.651	0.189	[-1.021 - -0.281]	5.601E-04	4.284E-03
Right banks of superior temporal sulcus	-0.544	0.331	[-1.192 - 0.104]	9.965E-02	1.645E-01
Left supramarginal gyrus	-0.403	0.285	[-0.961 - 0.155]	1.569E-01	2.184E-01
Right supramarginal gyrus	-0.275	0.306	[-0.876 - 0.325]	3.688E-01	4.501E-01
Left caudal middle frontal gyrus	-0.178	0.214	[-0.598 - 0.241]	4.052E-01	4.795E-01
Right caudal middle frontal gyrus	-0.419	0.186	[-0.783 - -0.054]	2.444E-02	5.103E-02
Left frontal pole	0.140	0.439	[-0.721 - 1.001]	7.502E-01	8.195E-01
Right frontal pole	0.047	0.434	[-0.803 - 0.897]	9.136E-01	9.266E-01
Left posterior cingulate cortex	-0.316	0.195	[-0.699 - 0.067]	1.055E-01	1.702E-01
Right posterior cingulate cortex	-0.547	0.187	[-0.914 - -0.180]	3.480E-03	1.524E-02
Left lateral occipital cortex	-0.576	0.187	[-0.944 - -0.209]	2.103E-03	1.147E-02
Right lateral occipital cortex	-0.614	0.188	[-0.982 - -0.246]	1.071E-03	6.915E-03
Left precentral gyrus	-0.079	0.268	[-0.603 - 0.445]	7.680E-01	8.262E-01
Right precentral gyrus	-0.171	0.185	[-0.533 - 0.191]	3.542E-01	4.491E-01
Left parahippocampal gyrus	-0.413	0.186	[-0.776 - -0.049]	2.622E-02	5.319E-02
Right parahippocampal gyrus	-0.268	0.185	[-0.631 - 0.095]	1.482E-01	2.105E-01
Left inferior parietal cortex	-0.835	0.191	[-1.210 - -0.461]	1.242E-05	4.293E-04
Right inferior parietal cortex	-0.907	0.205	[-1.309 - -0.505]	9.818E-06	4.293E-04
Left transverse temporal gyrus	0.045	0.259	[-0.463 - 0.553]	8.632E-01	8.883E-01
Right transverse temporal gyrus	-0.288	0.279	[-0.835 - 0.259]	3.020E-01	3.971E-01
Left postcentral gyrus	-0.466	0.220	[-0.897 - -0.035]	3.395E-02	6.696E-02
Right postcentral gyrus	-0.509	0.322	[-1.141 - 0.123]	1.147E-01	1.737E-01
Left precuneus	-0.528	0.187	[-0.894 - -0.161]	4.793E-03	1.701E-02
Right precuneus	-0.736	0.189	[-1.106 - -0.366]	9.717E-05	9.856E-04
Left caudal anterior cingulate cortex	-0.033	0.184	[-0.394 - 0.327]	8.561E-01	8.883E-01
Right caudal anterior cingulate cortex	-0.404	0.341	[-1.072 - 0.264]	2.355E-01	3.215E-01
Left cuneus	-0.290	0.264	[-0.809 - 0.228]	2.722E-01	3.647E-01
Right cuneus	-0.523	0.186	[-0.888 - -0.157]	5.054E-03	1.709E-02
Left rostral anterior cingulate cortex	-0.164	0.185	[-0.526 - 0.198]	3.740E-01	4.501E-01
Right rostral anterior cingulate cortex	-0.073	0.198	[-0.461 - 0.316]	7.140E-01	7.921E-01
Left pericalcarine cortex	-0.603	0.188	[-0.972 - -0.234]	1.367E-03	8.087E-03
Right pericalcarine cortex	-0.492	0.186	[-0.858 - -0.127]	8.224E-03	2.444E-02
Left paracentral lobule	-0.333	0.185	[-0.695 - 0.029]	7.167E-02	1.305E-01
Right paracentral lobule	-0.268	0.185	[-0.630 - 0.093]	1.458E-01	2.105E-01
Left superior parietal cortex	-0.816	0.190	[-1.190 - -0.443]	1.814E-05	4.293E-04
Right superior parietal cortex	-0.778	0.190	[-1.149 - -0.406]	4.107E-05	5.832E-04
Left temporal pole	-0.458	0.258	[-0.963 - 0.048]	7.620E-02	1.336E-01
Right temporal pole	-0.302	0.395	[-1.076 - 0.471]	4.435E-01	5.162E-01
Left entorhinal cortex	-0.432	0.186	[-0.797 - -0.068]	2.017E-02	4.475E-02
Right entorhinal cortex	-0.314	0.344	[-0.988 - 0.361]	3.621E-01	4.501E-01

Supplementary Table S125. Cortical thickness differences between individuals with bipolar disorder taking sodium valproate and individuals with bipolar disorder not taking sodium valproate controlling for age, sex, and other medications at 25 years of age or older

	d	Std. Err.	95% CI	*p*-value	FDR *q*-value
Global mean cortical thickness	-0.780	0.200	[-1.172 - -0.387]	9.763E-05	1.733E-03
Left hemisphere	-0.692	0.213	[-1.110 - -0.274]	1.180E-03	5.985E-03
Right hemisphere	-0.846	0.221	[-1.279 - -0.413]	1.271E-04	1.805E-03
Left fusiform gyrus	-0.745	0.286	[-1.307 - -0.184]	9.252E-03	2.856E-02
Right fusiform gyrus	-0.189	0.313	[-0.803 - 0.424]	5.450E-01	6.343E-01
Left pars opercularis of inferior frontal gyrus	-0.314	0.259	[-0.821 - 0.193]	2.247E-01	3.069E-01
Right pars opercularis of inferior frontal gyrus	-0.366	0.195	[-0.749 - 0.017]	6.123E-02	1.087E-01
Left superior temporal gyrus	-0.003	0.287	[-0.566 - 0.559]	9.905E-01	9.920E-01
Right superior temporal gyrus	-0.077	0.496	[-1.050 - 0.896]	8.766E-01	9.290E-01
Left insula	-0.635	0.337	[-1.295 - 0.026]	5.991E-02	1.087E-01
Right insula	-0.753	0.249	[-1.241 - -0.266]	2.463E-03	1.029E-02
Left lingual gyrus	-0.664	0.199	[-1.053 - -0.274]	8.404E-04	5.450E-03
Right lingual gyrus	-0.656	0.198	[-1.045 - -0.268]	9.212E-04	5.450E-03
Left pars triangularis of inferior frontal gyrus	-0.103	0.315	[-0.720 - 0.515]	7.448E-01	8.136E-01
Right pars triangularis of inferior frontal gyrus	-0.531	0.196	[-0.915 - -0.146]	6.895E-03	2.225E-02
Left lateral orbitofrontal cortex	-0.460	0.196	[-0.845 - -0.076]	1.897E-02	4.415E-02
Right lateral orbitofrontal cortex	-0.369	0.252	[-0.862 - 0.124]	1.427E-01	2.202E-01
Left rostral middle frontal gyrus	-0.164	0.346	[-0.843 - 0.514]	6.350E-01	7.139E-01
Right rostral middle frontal gyrus	-0.476	0.197	[-0.861 - -0.091]	1.549E-02	3.928E-02
Left middle temporal gyrus	-0.384	0.195	[-0.766 - -0.001]	4.967E-02	9.531E-02
Right middle temporal gyrus	-0.508	0.197	[-0.894 - -0.123]	9.713E-03	2.874E-02
Left superior frontal gyrus	-0.573	0.212	[-0.987 - -0.158]	6.811E-03	2.225E-02
Right superior frontal gyrus	-0.578	0.247	[-1.061 - -0.094]	1.928E-02	4.415E-02
Left pars orbitalis of inferior frontal gyrus	-0.284	0.210	[-0.695 - 0.127]	1.752E-01	2.539E-01
Right pars orbitalis of inferior frontal gyrus	-0.236	0.270	[-0.765 - 0.293]	3.818E-01	4.841E-01
Left medial orbitofrontal cortex	-0.444	0.196	[-0.829 - -0.059]	2.368E-02	4.945E-02
Right medial orbitofrontal cortex	-0.367	0.195	[-0.750 - 0.015]	5.959E-02	1.087E-01
Left inferior temporal gyrus	-0.498	0.197	[-0.883 - -0.112]	1.137E-02	3.229E-02
Right inferior temporal gyrus	-0.569	0.234	[-1.028 - -0.111]	1.496E-02	3.928E-02
Left isthmus cingulate cortex	-0.630	0.227	[-1.076 - -0.184]	5.610E-03	1.992E-02
Right isthmus cingulate cortex	-0.735	0.202	[-1.130 - -0.340]	2.656E-04	3.143E-03
Left banks of superior temporal sulcus	-0.628	0.198	[-1.017 - -0.240]	1.528E-03	7.233E-03
Right banks of superior temporal sulcus	-0.540	0.349	[-1.225 - 0.145]	1.223E-01	1.949E-01
Left supramarginal gyrus	-0.397	0.318	[-1.021 - 0.227]	2.120E-01	2.951E-01
Right supramarginal gyrus	-0.305	0.322	[-0.937 - 0.327]	3.435E-01	4.434E-01
Left caudal middle frontal gyrus	-0.134	0.213	[-0.551 - 0.283]	5.287E-01	6.303E-01
Right caudal middle frontal gyrus	-0.491	0.197	[-0.876 - -0.105]	1.263E-02	3.450E-02
Left frontal pole	0.123	0.488	[-0.832 - 1.079]	8.004E-01	8.610E-01
Right frontal pole	-0.046	0.429	[-0.887 - 0.796]	9.150E-01	9.554E-01
Left posterior cingulate cortex	-0.337	0.219	[-0.766 - 0.092]	1.235E-01	1.949E-01
Right posterior cingulate cortex	-0.659	0.198	[-1.047 - -0.270]	8.951E-04	5.450E-03
Left lateral occipital cortex	-0.556	0.197	[-0.942 - -0.170]	4.787E-03	1.789E-02
Right lateral occipital cortex	-0.667	0.198	[-1.055 - -0.278]	7.720E-04	5.450E-03
Left precentral gyrus	-0.167	0.261	[-0.679 - 0.345]	5.228E-01	6.303E-01
Right precentral gyrus	-0.306	0.194	[-0.687 - 0.075]	1.153E-01	1.904E-01
Left parahippocampal gyrus	-0.435	0.196	[-0.818 - -0.051]	2.623E-02	5.321E-02
Right parahippocampal gyrus	-0.339	0.195	[-0.722 - 0.044]	8.259E-02	1.396E-01
Left inferior parietal cortex	-0.839	0.201	[-1.233 - -0.445]	3.061E-05	1.240E-03
Right inferior parietal cortex	-0.864	0.278	[-1.408 - -0.320]	1.862E-03	8.263E-03
Left transverse temporal gyrus	-0.017	0.252	[-0.511 - 0.477]	9.459E-01	9.733E-01
Right transverse temporal gyrus	-0.316	0.246	[-0.798 - 0.166]	1.987E-01	2.821E-01
Left postcentral gyrus	-0.493	0.214	[-0.913 - -0.073]	2.145E-02	4.614E-02
Right postcentral gyrus	-0.596	0.341	[-1.265 - 0.073]	8.086E-02	1.396E-01
Left precuneus	-0.500	0.208	[-0.908 - -0.091]	1.646E-02	4.029E-02
Right precuneus	-0.710	0.199	[-1.099 - -0.321]	3.496E-04	3.546E-03
Left caudal anterior cingulate cortex	-0.002	0.193	[-0.381 - 0.377]	9.920E-01	9.920E-01
Right caudal anterior cingulate cortex	-0.487	0.350	[-1.172 - 0.198]	1.636E-01	2.419E-01
Left cuneus	-0.325	0.233	[-0.781 - 0.131]	1.625E-01	2.419E-01
Right cuneus	-0.562	0.197	[-0.948 - -0.177]	4.253E-03	1.678E-02
Left rostral anterior cingulate cortex	-0.159	0.194	[-0.540 - 0.221]	4.120E-01	5.132E-01
Right rostral anterior cingulate cortex	-0.090	0.194	[-0.471 - 0.291]	6.435E-01	7.139E-01
Left pericalcarine cortex	-0.651	0.199	[-1.040 - -0.262]	1.049E-03	5.729E-03
Right pericalcarine cortex	-0.664	0.199	[-1.054 - -0.274]	8.357E-04	5.450E-03
Left paracentral lobule	-0.410	0.195	[-0.792 - -0.028]	3.552E-02	7.005E-02
Right paracentral lobule	-0.220	0.194	[-0.600 - 0.159]	2.554E-01	3.421E-01
Left superior parietal cortex	-0.830	0.200	[-1.223 - -0.437]	3.494E-05	1.240E-03
Right superior parietal cortex	-0.787	0.200	[-1.178 - -0.395]	8.160E-05	1.733E-03
Left temporal pole	-0.352	0.333	[-1.006 - 0.301]	2.905E-01	3.819E-01
Right temporal pole	-0.259	0.441	[-1.124 - 0.606]	5.576E-01	6.385E-01
Left entorhinal cortex	-0.455	0.196	[-0.840 - -0.071]	2.020E-02	4.483E-02
Right entorhinal cortex	-0.247	0.396	[-1.024 - 0.530]	5.327E-01	6.303E-01

Supplementary Table S126. Cortical thickness differences between individuals with bipolar disorder taking second-generation antipsychotics and individuals with bipolar disorder not taking second-generation antipsychotics controlling for age, sex, and other medications

	d	Std. Err.	95% CI	*p*-value	FDR *q*-value
Global mean cortical thickness	0.244	0.268	[-0.280 - 0.768]	3.614E-01	8.877E-01
Left hemisphere	0.224	0.239	[-0.244 - 0.692]	3.475E-01	8.877E-01
Right hemisphere	0.252	0.290	[-0.317 - 0.820]	3.855E-01	8.877E-01
Left fusiform gyrus	-0.045	0.205	[-0.447 - 0.357]	8.257E-01	9.888E-01
Right fusiform gyrus	-0.199	0.304	[-0.795 - 0.397]	5.126E-01	8.877E-01
Left pars opercularis of inferior frontal gyrus	0.017	0.187	[-0.349 - 0.383]	9.264E-01	9.888E-01
Right pars opercularis of inferior frontal gyrus	0.079	0.142	[-0.200 - 0.358]	5.788E-01	9.040E-01
Left superior temporal gyrus	0.185	0.170	[-0.148 - 0.518]	2.763E-01	8.877E-01
Right superior temporal gyrus	0.139	0.269	[-0.389 - 0.666]	6.069E-01	9.040E-01
Left insula	0.225	0.215	[-0.197 - 0.647]	2.968E-01	8.877E-01
Right insula	0.373	0.219	[-0.056 - 0.802]	8.816E-02	6.926E-01
Left lingual gyrus	0.112	0.297	[-0.470 - 0.695]	7.057E-01	9.399E-01
Right lingual gyrus	-0.110	0.297	[-0.692 - 0.473]	7.122E-01	9.399E-01
Left pars triangularis of inferior frontal gyrus	0.030	0.143	[-0.250 - 0.311]	8.314E-01	9.888E-01
Right pars triangularis of inferior frontal gyrus	-0.022	0.175	[-0.364 - 0.320]	8.999E-01	9.888E-01
Left lateral orbitofrontal cortex	0.035	0.149	[-0.257 - 0.328]	8.133E-01	9.888E-01
Right lateral orbitofrontal cortex	0.207	0.258	[-0.299 - 0.714]	4.227E-01	8.877E-01
Left rostral middle frontal gyrus	-0.009	0.192	[-0.385 - 0.366]	9.615E-01	9.893E-01
Right rostral middle frontal gyrus	0.058	0.163	[-0.261 - 0.377]	7.210E-01	9.399E-01
Left middle temporal gyrus	0.172	0.151	[-0.124 - 0.468]	2.547E-01	8.877E-01
Right middle temporal gyrus	0.146	0.187	[-0.221 - 0.512]	4.357E-01	8.877E-01
Left superior frontal gyrus	0.004	0.193	[-0.375 - 0.382]	9.846E-01	9.912E-01
Right superior frontal gyrus	0.108	0.212	[-0.308 - 0.524]	6.112E-01	9.040E-01
Left pars orbitalis of inferior frontal gyrus	0.021	0.199	[-0.369 - 0.412]	9.146E-01	9.888E-01
Right pars orbitalis of inferior frontal gyrus	0.084	0.236	[-0.379 - 0.547]	7.215E-01	9.399E-01
Left medial orbitofrontal cortex	-0.040	0.216	[-0.463 - 0.383]	8.525E-01	9.888E-01
Right medial orbitofrontal cortex	0.002	0.209	[-0.408 - 0.413]	9.912E-01	9.912E-01
Left inferior temporal gyrus	0.128	0.187	[-0.238 - 0.495]	4.932E-01	8.877E-01
Right inferior temporal gyrus	0.208	0.229	[-0.240 - 0.657]	3.628E-01	8.877E-01
Left isthmus cingulate cortex	-0.147	0.214	[-0.566 - 0.271]	4.898E-01	8.877E-01
Right isthmus cingulate cortex	-0.063	0.145	[-0.348 - 0.221]	6.635E-01	9.399E-01
Left banks of superior temporal sulcus	0.012	0.143	[-0.267 - 0.291]	9.331E-01	9.888E-01
Right banks of superior temporal sulcus	-0.031	0.173	[-0.370 - 0.309]	8.592E-01	9.888E-01
Left supramarginal gyrus	0.276	0.166	[-0.049 - 0.602]	9.630E-02	6.926E-01
Right supramarginal gyrus	0.243	0.143	[-0.038 - 0.523]	9.036E-02	6.926E-01
Left caudal middle frontal gyrus	0.212	0.202	[-0.185 - 0.608]	2.958E-01	8.877E-01
Right caudal middle frontal gyrus	0.068	0.168	[-0.261 - 0.397]	6.837E-01	9.399E-01
Left frontal pole	0.150	0.264	[-0.368 - 0.668]	5.702E-01	9.040E-01
Right frontal pole	-0.118	0.177	[-0.465 - 0.229]	5.046E-01	8.877E-01
Left posterior cingulate cortex	-0.020	0.172	[-0.357 - 0.317]	9.062E-01	9.888E-01
Right posterior cingulate cortex	0.194	0.290	[-0.375 - 0.762]	5.040E-01	8.877E-01
Left lateral occipital cortex	0.190	0.143	[-0.090 - 0.470]	1.835E-01	8.877E-01
Right lateral occipital cortex	0.180	0.194	[-0.199 - 0.560]	3.522E-01	8.877E-01
Left precentral gyrus	0.109	0.150	[-0.184 - 0.402]	4.653E-01	8.877E-01
Right precentral gyrus	0.145	0.203	[-0.253 - 0.543]	4.756E-01	8.877E-01
Left parahippocampal gyrus	0.172	0.161	[-0.143 - 0.488]	2.839E-01	8.877E-01
Right parahippocampal gyrus	-0.154	0.195	[-0.537 - 0.228]	4.289E-01	8.877E-01
Left inferior parietal cortex	0.321	0.156	[0.015 - 0.627]	3.975E-02	6.926E-01
Right inferior parietal cortex	0.025	0.208	[-0.383 - 0.432]	9.060E-01	9.888E-01
Left transverse temporal gyrus	0.147	0.175	[-0.197 - 0.490]	4.018E-01	8.877E-01
Right transverse temporal gyrus	-0.047	0.143	[-0.327 - 0.233]	7.413E-01	9.399E-01
Left postcentral gyrus	0.336	0.193	[-0.041 - 0.714]	8.093E-02	6.926E-01
Right postcentral gyrus	0.238	0.143	[-0.043 - 0.519]	9.755E-02	6.926E-01
Left precuneus	0.229	0.309	[-0.376 - 0.833]	4.589E-01	8.877E-01
Right precuneus	0.188	0.267	[-0.336 - 0.712]	4.820E-01	8.877E-01
Left caudal anterior cingulate cortex	0.401	0.162	[0.083 - 0.718]	1.345E-02	4.773E-01
Right caudal anterior cingulate cortex	0.121	0.176	[-0.224 - 0.467]	4.924E-01	8.877E-01
Left cuneus	0.117	0.217	[-0.309 - 0.543]	5.906E-01	9.040E-01
Right cuneus	0.336	0.240	[-0.134 - 0.806]	1.608E-01	8.877E-01
Left rostral anterior cingulate cortex	0.085	0.142	[-0.194 - 0.364]	5.513E-01	9.040E-01
Right rostral anterior cingulate cortex	0.195	0.143	[-0.085 - 0.474]	1.731E-01	8.877E-01
Left pericalcarine cortex	0.202	0.269	[-0.324 - 0.728]	4.514E-01	8.877E-01
Right pericalcarine cortex	0.093	0.150	[-0.202 - 0.388]	5.365E-01	9.040E-01
Left paracentral lobule	0.050	0.148	[-0.240 - 0.340]	7.354E-01	9.399E-01
Right paracentral lobule	0.253	0.226	[-0.191 - 0.696]	2.639E-01	8.877E-01
Left superior parietal cortex	-0.008	0.167	[-0.336 - 0.319]	9.598E-01	9.893E-01
Right superior parietal cortex	0.262	0.144	[-0.020 - 0.544]	6.817E-02	6.926E-01
Left temporal pole	0.255	0.143	[-0.026 - 0.536]	7.483E-02	6.926E-01
Right temporal pole	0.702	0.171	[0.368 - 1.036]	3.889E-05	2.761E-03
Left entorhinal cortex	0.102	0.143	[-0.178 - 0.382]	4.749E-01	8.877E-01
Right entorhinal cortex	0.141	0.182	[-0.215 - 0.498]	4.377E-01	8.877E-01

Supplementary Table S127. Cortical thickness differences between individuals with bipolar disorder taking second-generation antipsychotics and individuals with bipolar disorder not taking second-generation antipsychotics controlling for age, sex, and other medications at 25 years of age or older

	d	Std. Err.	95% CI	*p*-value	FDR *q*-value
Global mean cortical thickness	0.182	0.271	[-0.350 - 0.713]	5.027E-01	9.352E-01
Left hemisphere	0.151	0.242	[-0.323 - 0.626]	5.327E-01	9.352E-01
Right hemisphere	0.203	0.295	[-0.374 - 0.781]	4.899E-01	9.352E-01
Left fusiform gyrus	-0.129	0.199	[-0.520 - 0.261]	5.163E-01	9.352E-01
Right fusiform gyrus	-0.165	0.323	[-0.798 - 0.468]	6.096E-01	9.352E-01
Left pars opercularis of inferior frontal gyrus	-0.071	0.161	[-0.386 - 0.245]	6.605E-01	9.352E-01
Right pars opercularis of inferior frontal gyrus	0.046	0.149	[-0.246 - 0.338]	7.576E-01	9.352E-01
Left superior temporal gyrus	0.026	0.226	[-0.417 - 0.469]	9.073E-01	9.492E-01
Right superior temporal gyrus	0.021	0.257	[-0.484 - 0.525]	9.358E-01	9.492E-01
Left insula	0.210	0.198	[-0.178 - 0.597]	2.885E-01	9.352E-01
Right insula	0.413	0.193	[0.035 - 0.792]	3.231E-02	5.735E-01
Left lingual gyrus	0.147	0.307	[-0.456 - 0.749]	6.335E-01	9.352E-01
Right lingual gyrus	-0.098	0.322	[-0.729 - 0.534]	7.615E-01	9.352E-01
Left pars triangularis of inferior frontal gyrus	-0.061	0.149	[-0.354 - 0.232]	6.837E-01	9.352E-01
Right pars triangularis of inferior frontal gyrus	-0.079	0.170	[-0.412 - 0.254]	6.423E-01	9.352E-01
Left lateral orbitofrontal cortex	0.032	0.150	[-0.261 - 0.325]	8.325E-01	9.352E-01
Right lateral orbitofrontal cortex	0.145	0.324	[-0.491 - 0.780]	6.557E-01	9.352E-01
Left rostral middle frontal gyrus	-0.072	0.195	[-0.453 - 0.310]	7.130E-01	9.352E-01
Right rostral middle frontal gyrus	0.043	0.174	[-0.299 - 0.384]	8.062E-01	9.352E-01
Left middle temporal gyrus	0.081	0.150	[-0.212 - 0.374]	5.884E-01	9.352E-01
Right middle temporal gyrus	0.176	0.150	[-0.117 - 0.469]	2.391E-01	9.352E-01
Left superior frontal gyrus	-0.069	0.205	[-0.471 - 0.333]	7.372E-01	9.352E-01
Right superior frontal gyrus	0.064	0.213	[-0.354 - 0.481]	7.643E-01	9.352E-01
Left pars orbitalis of inferior frontal gyrus	-0.090	0.213	[-0.509 - 0.328]	6.724E-01	9.352E-01
Right pars orbitalis of inferior frontal gyrus	0.015	0.320	[-0.612 - 0.643]	9.617E-01	9.617E-01
Left medial orbitofrontal cortex	0.021	0.224	[-0.419 - 0.460]	9.260E-01	9.492E-01
Right medial orbitofrontal cortex	0.050	0.231	[-0.402 - 0.502]	8.286E-01	9.352E-01
Left inferior temporal gyrus	0.047	0.218	[-0.380 - 0.474]	8.282E-01	9.352E-01
Right inferior temporal gyrus	0.163	0.212	[-0.253 - 0.579]	4.426E-01	9.352E-01
Left isthmus cingulate cortex	-0.023	0.243	[-0.499 - 0.453]	9.241E-01	9.492E-01
Right isthmus cingulate cortex	0.133	0.168	[-0.195 - 0.462]	4.272E-01	9.352E-01
Left banks of superior temporal sulcus	-0.133	0.188	[-0.500 - 0.235]	4.793E-01	9.352E-01
Right banks of superior temporal sulcus	-0.072	0.179	[-0.423 - 0.278]	6.855E-01	9.352E-01
Left supramarginal gyrus	0.262	0.187	[-0.104 - 0.628]	1.607E-01	9.352E-01
Right supramarginal gyrus	0.186	0.150	[-0.107 - 0.480]	2.138E-01	9.352E-01
Left caudal middle frontal gyrus	0.134	0.216	[-0.290 - 0.557]	5.354E-01	9.352E-01
Right caudal middle frontal gyrus	0.085	0.205	[-0.316 - 0.487]	6.762E-01	9.352E-01
Left frontal pole	0.249	0.256	[-0.253 - 0.751]	3.313E-01	9.352E-01
Right frontal pole	-0.052	0.217	[-0.476 - 0.373]	8.111E-01	9.352E-01
Left posterior cingulate cortex	0.037	0.186	[-0.328 - 0.402]	8.430E-01	9.352E-01
Right posterior cingulate cortex	0.246	0.302	[-0.346 - 0.838]	4.160E-01	9.352E-01
Left lateral occipital cortex	0.222	0.161	[-0.094 - 0.538]	1.687E-01	9.352E-01
Right lateral occipital cortex	0.128	0.184	[-0.233 - 0.490]	4.867E-01	9.352E-01
Left precentral gyrus	0.020	0.150	[-0.273 - 0.314]	8.914E-01	9.492E-01
Right precentral gyrus	0.112	0.214	[-0.308 - 0.532]	6.009E-01	9.352E-01
Left parahippocampal gyrus	0.183	0.206	[-0.221 - 0.587]	3.739E-01	9.352E-01
Right parahippocampal gyrus	-0.155	0.232	[-0.610 - 0.301]	5.051E-01	9.352E-01
Left inferior parietal cortex	0.252	0.208	[-0.155 - 0.659]	2.256E-01	9.352E-01
Right inferior parietal cortex	0.054	0.261	[-0.457 - 0.565]	8.366E-01	9.352E-01
Left transverse temporal gyrus	0.160	0.180	[-0.194 - 0.513]	3.755E-01	9.352E-01
Right transverse temporal gyrus	0.034	0.153	[-0.266 - 0.334]	8.255E-01	9.352E-01
Left postcentral gyrus	0.316	0.180	[-0.036 - 0.668]	7.849E-02	9.352E-01
Right postcentral gyrus	0.170	0.150	[-0.124 - 0.464]	2.565E-01	9.352E-01
Left precuneus	0.103	0.297	[-0.480 - 0.685]	7.299E-01	9.352E-01
Right precuneus	0.082	0.265	[-0.438 - 0.601]	7.578E-01	9.352E-01
Left caudal anterior cingulate cortex	0.422	0.188	[0.054 - 0.790]	2.446E-02	5.735E-01
Right caudal anterior cingulate cortex	0.136	0.195	[-0.247 - 0.519]	4.861E-01	9.352E-01
Left cuneus	0.146	0.257	[-0.357 - 0.650]	5.687E-01	9.352E-01
Right cuneus	0.396	0.232	[-0.060 - 0.851]	8.863E-02	9.352E-01
Left rostral anterior cingulate cortex	0.087	0.149	[-0.205 - 0.379]	5.591E-01	9.352E-01
Right rostral anterior cingulate cortex	0.097	0.149	[-0.196 - 0.389]	5.172E-01	9.352E-01
Left pericalcarine cortex	0.212	0.304	[-0.383 - 0.808]	4.852E-01	9.352E-01
Right pericalcarine cortex	0.131	0.150	[-0.163 - 0.424]	3.826E-01	9.352E-01
Left paracentral lobule	0.033	0.152	[-0.266 - 0.331]	8.298E-01	9.352E-01
Right paracentral lobule	0.145	0.215	[-0.276 - 0.565]	5.003E-01	9.352E-01
Left superior parietal cortex	0.030	0.213	[-0.387 - 0.447]	8.873E-01	9.492E-01
Right superior parietal cortex	0.239	0.150	[-0.055 - 0.534]	1.110E-01	9.352E-01
Left temporal pole	0.336	0.150	[0.042 - 0.631]	2.520E-02	5.735E-01
Right temporal pole	0.696	0.154	[0.395 - 0.997]	5.930E-06	4.210E-04
Left entorhinal cortex	0.165	0.150	[-0.129 - 0.458]	2.712E-01	9.352E-01
Right entorhinal cortex	0.235	0.161	[-0.081 - 0.551]	1.453E-01	9.352E-01

Supplementary Table S128. Cortical thickness differences between individuals with bipolar disorder taking first-generation antipsychotics and individuals with bipolar disorder not taking first-generation antipsychotics controlling for age, sex, and other medications

	d	Std. Err.	95% CI	*p*-value	FDR *q*-value
Global mean cortical thickness	-0.363	0.309	[-0.968 - 0.243]	2.405E-01	7.693E-01
Left hemisphere	-0.340	0.309	[-0.944 - 0.265]	2.709E-01	7.693E-01
Right hemisphere	-0.384	0.309	[-0.991 - 0.222]	2.141E-01	7.693E-01
Left fusiform gyrus	-0.411	0.533	[-1.456 - 0.634]	4.411E-01	7.933E-01
Right fusiform gyrus	-0.690	0.314	[-1.305 - -0.075]	2.788E-02	5.909E-01
Left pars opercularis of inferior frontal gyrus	-0.379	0.449	[-1.258 - 0.501]	3.986E-01	7.933E-01
Right pars opercularis of inferior frontal gyrus	-0.624	0.507	[-1.617 - 0.370]	2.185E-01	7.693E-01
Left superior temporal gyrus	-0.636	0.313	[-1.251 - -0.022]	4.235E-02	5.909E-01
Right superior temporal gyrus	-0.565	0.326	[-1.204 - 0.074]	8.323E-02	6.386E-01
Left insula	-0.230	0.309	[-0.835 - 0.375]	4.567E-01	7.933E-01
Right insula	-0.575	0.313	[-1.189 - 0.039]	6.658E-02	5.909E-01
Left lingual gyrus	-0.286	0.376	[-1.023 - 0.450]	4.462E-01	7.933E-01
Right lingual gyrus	-0.217	0.308	[-0.820 - 0.386]	4.804E-01	7.933E-01
Left pars triangularis of inferior frontal gyrus	-0.316	0.311	[-0.924 - 0.293]	3.096E-01	7.933E-01
Right pars triangularis of inferior frontal gyrus	-0.250	0.309	[-0.855 - 0.355]	4.177E-01	7.933E-01
Left lateral orbitofrontal cortex	-0.579	0.314	[-1.195 - 0.037]	6.542E-02	5.909E-01
Right lateral orbitofrontal cortex	-0.932	0.658	[-2.222 - 0.357]	1.564E-01	6.942E-01
Left rostral middle frontal gyrus	-0.356	0.309	[-0.961 - 0.249]	2.487E-01	7.693E-01
Right rostral middle frontal gyrus	-0.221	0.309	[-0.826 - 0.384]	4.738E-01	7.933E-01
Left middle temporal gyrus	-0.320	0.308	[-0.924 - 0.285]	2.998E-01	7.933E-01
Right middle temporal gyrus	-0.655	0.425	[-1.487 - 0.178]	1.235E-01	6.386E-01
Left superior frontal gyrus	-0.124	0.308	[-0.727 - 0.480]	6.883E-01	8.885E-01
Right superior frontal gyrus	-0.202	0.308	[-0.806 - 0.403]	5.132E-01	8.281E-01
Left pars orbitalis of inferior frontal gyrus	-0.343	0.309	[-0.948 - 0.263]	2.675E-01	7.693E-01
Right pars orbitalis of inferior frontal gyrus	-0.367	0.308	[-0.971 - 0.238]	2.347E-01	7.693E-01
Left medial orbitofrontal cortex	-0.495	0.310	[-1.102 - 0.112]	1.103E-01	6.386E-01
Right medial orbitofrontal cortex	-0.610	0.313	[-1.223 - 0.003]	5.122E-02	5.909E-01
Left inferior temporal gyrus	-0.225	0.309	[-0.830 - 0.381]	4.669E-01	7.933E-01
Right inferior temporal gyrus	-0.237	0.308	[-0.840 - 0.366]	4.411E-01	7.933E-01
Left isthmus cingulate cortex	-0.484	0.310	[-1.091 - 0.124]	1.188E-01	6.386E-01
Right isthmus cingulate cortex	-0.044	0.308	[-0.647 - 0.560]	8.875E-01	9.547E-01
Left banks of superior temporal sulcus	0.790	0.318	[0.166 - 1.413]	1.301E-02	5.909E-01
Right banks of superior temporal sulcus	-0.414	0.312	[-1.025 - 0.198]	1.849E-01	7.693E-01
Left supramarginal gyrus	-0.107	0.307	[-0.709 - 0.495]	7.281E-01	9.070E-01
Right supramarginal gyrus	-0.127	0.307	[-0.730 - 0.475]	6.792E-01	8.885E-01
Left caudal middle frontal gyrus	-0.281	0.309	[-0.887 - 0.324]	3.627E-01	7.933E-01
Right caudal middle frontal gyrus	-0.287	0.310	[-0.895 - 0.321]	3.541E-01	7.933E-01
Left frontal pole	-0.780	0.510	[-1.779 - 0.219]	1.259E-01	6.386E-01
Right frontal pole	-0.126	0.307	[-0.728 - 0.476]	6.820E-01	8.885E-01
Left posterior cingulate cortex	-0.350	0.309	[-0.956 - 0.256]	2.575E-01	7.693E-01
Right posterior cingulate cortex	-0.131	0.308	[-0.734 - 0.472]	6.703E-01	8.885E-01
Left lateral occipital cortex	0.034	0.395	[-0.740 - 0.808]	9.315E-01	9.585E-01
Right lateral occipital cortex	-0.622	0.314	[-1.238 - -0.007]	4.747E-02	5.909E-01
Left precentral gyrus	-0.274	0.308	[-0.878 - 0.329]	3.726E-01	7.933E-01
Right precentral gyrus	-0.475	0.310	[-1.083 - 0.133]	1.259E-01	6.386E-01
Left parahippocampal gyrus	-0.007	0.308	[-0.611 - 0.597]	9.818E-01	9.878E-01
Right parahippocampal gyrus	-0.114	0.307	[-0.716 - 0.488]	7.106E-01	9.010E-01
Left inferior parietal cortex	0.081	0.307	[-0.520 - 0.683]	7.908E-01	9.523E-01
Right inferior parietal cortex	0.127	0.308	[-0.476 - 0.730]	6.803E-01	8.885E-01
Left transverse temporal gyrus	-0.239	0.308	[-0.843 - 0.365]	4.383E-01	7.933E-01
Right transverse temporal gyrus	0.005	0.307	[-0.597 - 0.607]	9.878E-01	9.878E-01
Left postcentral gyrus	-0.045	0.308	[-0.648 - 0.558]	8.840E-01	9.547E-01
Right postcentral gyrus	0.512	0.662	[-0.785 - 1.809]	4.392E-01	7.933E-01
Left precuneus	0.289	1.168	[-2.001 - 2.579]	8.047E-01	9.523E-01
Right precuneus	0.079	0.309	[-0.526 - 0.684]	7.986E-01	9.523E-01
Left caudal anterior cingulate cortex	-0.047	0.309	[-0.652 - 0.557]	8.779E-01	9.547E-01
Right caudal anterior cingulate cortex	-0.058	0.308	[-0.661 - 0.545]	8.501E-01	9.547E-01
Left cuneus	-0.737	0.371	[-1.464 - -0.009]	4.715E-02	5.909E-01
Right cuneus	0.144	0.308	[-0.459 - 0.747]	6.398E-01	8.885E-01
Left rostral anterior cingulate cortex	-0.182	0.309	[-0.789 - 0.424]	5.556E-01	8.582E-01
Right rostral anterior cingulate cortex	-0.169	0.308	[-0.773 - 0.434]	5.822E-01	8.612E-01
Left pericalcarine cortex	0.139	0.308	[-0.465 - 0.743]	6.523E-01	8.885E-01
Right pericalcarine cortex	0.059	0.307	[-0.543 - 0.662]	8.472E-01	9.547E-01
Left paracentral lobule	-0.175	0.307	[-0.778 - 0.427]	5.681E-01	8.582E-01
Right paracentral lobule	0.065	0.402	[-0.722 - 0.853]	8.708E-01	9.547E-01
Left superior parietal cortex	-0.218	0.308	[-0.821 - 0.385]	4.779E-01	7.933E-01
Right superior parietal cortex	-0.179	0.308	[-0.783 - 0.424]	5.600E-01	8.582E-01
Left temporal pole	-0.432	0.470	[-1.353 - 0.490]	3.583E-01	7.933E-01
Right temporal pole	0.033	0.338	[-0.629 - 0.694]	9.231E-01	9.585E-01
Left entorhinal cortex	0.064	0.725	[-1.357 - 1.485]	9.297E-01	9.585E-01
Right entorhinal cortex	-0.673	0.450	[-1.556 - 0.210]	1.351E-01	6.397E-01

Supplementary Table S129. Cortical thickness differences between individuals with bipolar disorder taking first-generation antipsychotics and individuals with bipolar disorder not taking first-generation antipsychotics controlling for age, sex, and other medications at 25 years of age or older

	d	Std. Err.	95% CI	*p*-value	FDR *q*-value
Global mean cortical thickness	-0.349	0.315	[-0.967 - 0.270]	2.690E-01	7.110E-01
Left hemisphere	-0.336	0.315	[-0.954 - 0.282]	2.864E-01	7.110E-01
Right hemisphere	-0.357	0.316	[-0.976 - 0.261]	2.579E-01	7.110E-01
Left fusiform gyrus	-0.589	0.691	[-1.944 - 0.766]	3.940E-01	7.770E-01
Right fusiform gyrus	-0.629	0.319	[-1.255 - -0.003]	4.894E-02	7.110E-01
Left pars opercularis of inferior frontal gyrus	-0.393	0.383	[-1.144 - 0.357]	3.041E-01	7.110E-01
Right pars opercularis of inferior frontal gyrus	-0.609	0.518	[-1.624 - 0.405]	2.392E-01	7.110E-01
Left superior temporal gyrus	-1.207	0.774	[-2.723 - 0.309]	1.188E-01	7.110E-01
Right superior temporal gyrus	-0.600	0.409	[-1.401 - 0.202]	1.424E-01	7.110E-01
Left insula	-0.209	0.316	[-0.828 - 0.410]	5.078E-01	8.094E-01
Right insula	-0.332	0.315	[-0.948 - 0.285]	2.921E-01	7.110E-01
Left lingual gyrus	-0.357	0.315	[-0.975 - 0.260]	2.570E-01	7.110E-01
Right lingual gyrus	-0.365	0.316	[-0.985 - 0.255]	2.483E-01	7.110E-01
Left pars triangularis of inferior frontal gyrus	-0.364	0.318	[-0.987 - 0.259]	2.517E-01	7.110E-01
Right pars triangularis of inferior frontal gyrus	-0.104	0.314	[-0.719 - 0.510]	7.392E-01	8.930E-01
Left lateral orbitofrontal cortex	-0.481	0.320	[-1.108 - 0.146]	1.329E-01	7.110E-01
Right lateral orbitofrontal cortex	-1.262	1.031	[-3.284 - 0.759]	2.210E-01	7.110E-01
Left rostral middle frontal gyrus	-0.367	0.315	[-0.985 - 0.250]	2.438E-01	7.110E-01
Right rostral middle frontal gyrus	-0.203	0.315	[-0.820 - 0.414]	5.186E-01	8.094E-01
Left middle temporal gyrus	-0.531	0.318	[-1.154 - 0.091]	9.437E-02	7.110E-01
Right middle temporal gyrus	-0.284	0.314	[-0.901 - 0.332]	3.662E-01	7.503E-01
Left superior frontal gyrus	-0.119	0.315	[-0.737 - 0.498]	7.047E-01	8.833E-01
Right superior frontal gyrus	-0.245	0.315	[-0.863 - 0.373]	4.368E-01	8.034E-01
Left pars orbitalis of inferior frontal gyrus	-0.510	0.320	[-1.137 - 0.117]	1.109E-01	7.110E-01
Right pars orbitalis of inferior frontal gyrus	-0.689	0.324	[-1.324 - -0.054]	3.340E-02	7.110E-01
Left medial orbitofrontal cortex	-0.420	0.315	[-1.038 - 0.198]	1.831E-01	7.110E-01
Right medial orbitofrontal cortex	-0.484	0.317	[-1.106 - 0.137]	1.269E-01	7.110E-01
Left inferior temporal gyrus	-0.415	0.406	[-1.210 - 0.381]	3.070E-01	7.110E-01
Right inferior temporal gyrus	-0.189	0.314	[-0.805 - 0.427]	5.472E-01	8.094E-01
Left isthmus cingulate cortex	-0.359	0.316	[-0.978 - 0.259]	2.547E-01	7.110E-01
Right isthmus cingulate cortex	0.245	0.447	[-0.631 - 1.120]	5.841E-01	8.294E-01
Left banks of superior temporal sulcus	0.375	0.315	[-0.243 - 0.994]	2.338E-01	7.110E-01
Right banks of superior temporal sulcus	-0.296	0.316	[-0.915 - 0.323]	3.482E-01	7.492E-01
Left supramarginal gyrus	-0.043	0.314	[-0.658 - 0.571]	8.900E-01	9.575E-01
Right supramarginal gyrus	-0.123	0.314	[-0.738 - 0.492]	6.951E-01	8.833E-01
Left caudal middle frontal gyrus	-0.340	0.317	[-0.960 - 0.281]	2.838E-01	7.110E-01
Right caudal middle frontal gyrus	-0.046	0.314	[-0.661 - 0.568]	8.823E-01	9.575E-01
Left frontal pole	-0.424	0.318	[-1.047 - 0.199]	1.821E-01	7.110E-01
Right frontal pole	0.117	0.314	[-0.499 - 0.733]	7.092E-01	8.833E-01
Left posterior cingulate cortex	-0.198	0.314	[-0.813 - 0.418]	5.286E-01	8.094E-01
Right posterior cingulate cortex	0.123	0.314	[-0.492 - 0.739]	6.950E-01	8.833E-01
Left lateral occipital cortex	0.456	0.792	[-1.096 - 2.008]	5.646E-01	8.181E-01
Right lateral occipital cortex	-0.567	0.320	[-1.194 - 0.059]	7.606E-02	7.110E-01
Left precentral gyrus	-0.315	0.315	[-0.932 - 0.301]	3.162E-01	7.110E-01
Right precentral gyrus	-0.530	0.319	[-1.156 - 0.095]	9.645E-02	7.110E-01
Left parahippocampal gyrus	0.008	0.315	[-0.610 - 0.625]	9.810E-01	9.810E-01
Right parahippocampal gyrus	-0.103	0.314	[-0.719 - 0.512]	7.421E-01	8.930E-01
Left inferior parietal cortex	0.152	0.314	[-0.464 - 0.767]	6.290E-01	8.588E-01
Right inferior parietal cortex	0.400	0.534	[-0.647 - 1.448]	4.538E-01	8.055E-01
Left transverse temporal gyrus	-0.254	0.315	[-0.871 - 0.362]	4.188E-01	8.034E-01
Right transverse temporal gyrus	0.243	0.316	[-0.376 - 0.863]	4.413E-01	8.034E-01
Left postcentral gyrus	0.060	0.314	[-0.555 - 0.676]	8.474E-01	9.550E-01
Right postcentral gyrus	0.325	0.467	[-0.590 - 1.240]	4.866E-01	8.094E-01
Left precuneus	0.048	0.824	[-1.568 - 1.663]	9.540E-01	9.810E-01
Right precuneus	-0.020	0.314	[-0.635 - 0.596]	9.500E-01	9.810E-01
Left caudal anterior cingulate cortex	0.052	0.315	[-0.564 - 0.669]	8.679E-01	9.575E-01
Right caudal anterior cingulate cortex	0.091	0.314	[-0.524 - 0.706]	7.719E-01	9.134E-01
Left cuneus	-0.838	0.321	[-1.467 - -0.208]	9.099E-03	6.460E-01
Right cuneus	0.398	0.315	[-0.220 - 1.016]	2.067E-01	7.110E-01
Left rostral anterior cingulate cortex	-0.161	0.316	[-0.781 - 0.459]	6.105E-01	8.500E-01
Right rostral anterior cingulate cortex	-0.283	0.316	[-0.902 - 0.336]	3.699E-01	7.503E-01
Left pericalcarine cortex	0.062	0.314	[-0.554 - 0.677]	8.446E-01	9.550E-01
Right pericalcarine cortex	0.072	0.314	[-0.544 - 0.688]	8.197E-01	9.541E-01
Left paracentral lobule	-0.133	0.314	[-0.748 - 0.482]	6.718E-01	8.833E-01
Right paracentral lobule	-0.195	0.314	[-0.810 - 0.420]	5.346E-01	8.094E-01
Left superior parietal cortex	0.008	0.319	[-0.618 - 0.634]	9.797E-01	9.810E-01
Right superior parietal cortex	-0.201	0.314	[-0.817 - 0.415]	5.219E-01	8.094E-01
Left temporal pole	-0.193	0.316	[-0.813 - 0.427]	5.424E-01	8.094E-01
Right temporal pole	-0.016	0.470	[-0.937 - 0.905]	9.721E-01	9.810E-01
Left entorhinal cortex	0.440	0.443	[-0.428 - 1.309]	3.205E-01	7.110E-01
Right entorhinal cortex	-0.454	0.317	[-1.075 - 0.167]	1.522E-01	7.110E-01

Supplementary Table S130. Cortical thickness differences between individuals with bipolar disorder taking antidepressants and individuals with bipolar disorder not taking antidepressants controlling for age, sex, and other medications

	d	Std. Err.	95% CI	*p*-value	FDR *q*-value
Global mean cortical thickness	-0.141	0.242	[-0.614 - 0.332]	5.593E-01	8.758E-01
Left hemisphere	-0.091	0.256	[-0.593 - 0.411]	7.229E-01	9.371E-01
Right hemisphere	-0.192	0.235	[-0.653 - 0.270]	4.152E-01	8.758E-01
Left fusiform gyrus	-0.237	0.157	[-0.544 - 0.070]	1.308E-01	7.438E-01
Right fusiform gyrus	0.012	0.150	[-0.281 - 0.305]	9.355E-01	9.910E-01
Left pars opercularis of inferior frontal gyrus	-0.296	0.150	[-0.590 - -0.001]	4.904E-02	6.964E-01
Right pars opercularis of inferior frontal gyrus	-0.237	0.150	[-0.531 - 0.057]	1.145E-01	7.438E-01
Left superior temporal gyrus	0.023	0.233	[-0.434 - 0.480]	9.225E-01	9.910E-01
Right superior temporal gyrus	-0.214	0.189	[-0.584 - 0.156]	2.573E-01	8.758E-01
Left insula	-0.161	0.291	[-0.731 - 0.409]	5.798E-01	8.758E-01
Right insula	-0.185	0.253	[-0.680 - 0.310]	4.642E-01	8.758E-01
Left lingual gyrus	0.056	0.150	[-0.237 - 0.349]	7.091E-01	9.371E-01
Right lingual gyrus	-0.001	0.149	[-0.293 - 0.292]	9.957E-01	9.957E-01
Left pars triangularis of inferior frontal gyrus	-0.148	0.237	[-0.612 - 0.316]	5.311E-01	8.758E-01
Right pars triangularis of inferior frontal gyrus	-0.222	0.217	[-0.648 - 0.204]	3.077E-01	8.758E-01
Left lateral orbitofrontal cortex	0.130	0.293	[-0.445 - 0.705]	6.575E-01	9.371E-01
Right lateral orbitofrontal cortex	-0.211	0.242	[-0.685 - 0.262]	3.821E-01	8.758E-01
Left rostral middle frontal gyrus	-0.186	0.223	[-0.624 - 0.252]	4.041E-01	8.758E-01
Right rostral middle frontal gyrus	-0.287	0.189	[-0.657 - 0.084]	1.298E-01	7.438E-01
Left middle temporal gyrus	0.127	0.150	[-0.167 - 0.421]	3.966E-01	8.758E-01
Right middle temporal gyrus	-0.064	0.177	[-0.411 - 0.283]	7.177E-01	9.371E-01
Left superior frontal gyrus	-0.302	0.293	[-0.876 - 0.272]	3.029E-01	8.758E-01
Right superior frontal gyrus	-0.362	0.175	[-0.704 - -0.019]	3.835E-02	6.964E-01
Left pars orbitalis of inferior frontal gyrus	-0.095	0.150	[-0.389 - 0.198]	5.245E-01	8.758E-01
Right pars orbitalis of inferior frontal gyrus	-0.176	0.339	[-0.840 - 0.488]	6.029E-01	8.918E-01
Left medial orbitofrontal cortex	0.063	0.307	[-0.540 - 0.665]	8.383E-01	9.582E-01
Right medial orbitofrontal cortex	-0.329	0.232	[-0.784 - 0.126]	1.567E-01	7.438E-01
Left inferior temporal gyrus	-0.117	0.169	[-0.448 - 0.214]	4.879E-01	8.758E-01
Right inferior temporal gyrus	0.213	0.285	[-0.345 - 0.772]	4.534E-01	8.758E-01
Left isthmus cingulate cortex	0.161	0.150	[-0.133 - 0.455]	2.822E-01	8.758E-01
Right isthmus cingulate cortex	-0.169	0.296	[-0.749 - 0.411]	5.679E-01	8.758E-01
Left banks of superior temporal sulcus	-0.018	0.277	[-0.562 - 0.526]	9.491E-01	9.910E-01
Right banks of superior temporal sulcus	-0.213	0.156	[-0.520 - 0.094]	1.733E-01	7.452E-01
Left supramarginal gyrus	-0.103	0.258	[-0.608 - 0.402]	6.899E-01	9.371E-01
Right supramarginal gyrus	-0.379	0.150	[-0.673 - -0.084]	1.173E-02	6.964E-01
Left caudal middle frontal gyrus	-0.207	0.187	[-0.574 - 0.160]	2.686E-01	8.758E-01
Right caudal middle frontal gyrus	-0.319	0.150	[-0.614 - -0.025]	3.360E-02	6.964E-01
Left frontal pole	0.202	0.294	[-0.374 - 0.778]	4.917E-01	8.758E-01
Right frontal pole	-0.257	0.191	[-0.631 - 0.117]	1.784E-01	7.452E-01
Left posterior cingulate cortex	-0.262	0.184	[-0.622 - 0.098]	1.537E-01	7.438E-01
Right posterior cingulate cortex	-0.223	0.374	[-0.956 - 0.509]	5.505E-01	8.758E-01
Left lateral occipital cortex	-0.026	0.150	[-0.319 - 0.267]	8.608E-01	9.582E-01
Right lateral occipital cortex	0.056	0.150	[-0.237 - 0.349]	7.074E-01	9.371E-01
Left precentral gyrus	-0.098	0.149	[-0.391 - 0.195]	5.130E-01	8.758E-01
Right precentral gyrus	-0.255	0.178	[-0.604 - 0.095]	1.530E-01	7.438E-01
Left parahippocampal gyrus	-0.218	0.321	[-0.847 - 0.411]	4.969E-01	8.758E-01
Right parahippocampal gyrus	-0.211	0.178	[-0.559 - 0.137]	2.349E-01	8.758E-01
Left inferior parietal cortex	-0.041	0.150	[-0.334 - 0.253]	7.856E-01	9.454E-01
Right inferior parietal cortex	-0.077	0.236	[-0.541 - 0.386]	7.432E-01	9.371E-01
Left transverse temporal gyrus	-0.045	0.150	[-0.338 - 0.249]	7.655E-01	9.371E-01
Right transverse temporal gyrus	-0.179	0.150	[-0.473 - 0.115]	2.327E-01	8.758E-01
Left postcentral gyrus	-0.142	0.168	[-0.471 - 0.187]	3.969E-01	8.758E-01
Right postcentral gyrus	-0.310	0.153	[-0.610 - -0.010]	4.273E-02	6.964E-01
Left precuneus	-0.105	0.150	[-0.399 - 0.188]	4.822E-01	8.758E-01
Right precuneus	-0.047	0.150	[-0.341 - 0.247]	7.537E-01	9.371E-01
Left caudal anterior cingulate cortex	0.281	0.186	[-0.084 - 0.646]	1.312E-01	7.438E-01
Right caudal anterior cingulate cortex	0.062	0.306	[-0.537 - 0.662]	8.382E-01	9.582E-01
Left cuneus	-0.006	0.149	[-0.298 - 0.286]	9.662E-01	9.942E-01
Right cuneus	0.047	0.149	[-0.246 - 0.340]	7.551E-01	9.371E-01
Left rostral anterior cingulate cortex	-0.150	0.202	[-0.545 - 0.246]	4.592E-01	8.758E-01
Right rostral anterior cingulate cortex	0.013	0.150	[-0.280 - 0.306]	9.310E-01	9.910E-01
Left pericalcarine cortex	-0.026	0.150	[-0.319 - 0.268]	8.638E-01	9.582E-01
Right pericalcarine cortex	-0.094	0.149	[-0.387 - 0.199]	5.293E-01	8.758E-01
Left paracentral lobule	-0.073	0.317	[-0.694 - 0.548]	8.179E-01	9.582E-01
Right paracentral lobule	-0.346	0.244	[-0.825 - 0.133]	1.571E-01	7.438E-01
Left superior parietal cortex	-0.004	0.262	[-0.516 - 0.509]	9.880E-01	9.957E-01
Right superior parietal cortex	-0.092	0.150	[-0.386 - 0.202]	5.398E-01	8.758E-01
Left temporal pole	-0.202	0.221	[-0.636 - 0.231]	3.603E-01	8.758E-01
Right temporal pole	-0.313	0.209	[-0.723 - 0.097]	1.342E-01	7.438E-01
Left entorhinal cortex	-0.315	0.214	[-0.735 - 0.104]	1.409E-01	7.438E-01
Right entorhinal cortex	-0.241	0.271	[-0.773 - 0.291]	3.741E-01	8.758E-01

Supplementary Table S131. Cortical thickness differences between individuals with bipolar disorder taking antidepressants and individuals with bipolar disorder not taking antidepressants controlling for age, sex, and other medications at 25 years of age or older

	d	Std. Err.	95% CI	*p*-value	FDR *q*-value
Global mean cortical thickness	-0.070	0.263	[-0.586 - 0.445]	7.891E-01	9.620E-01
Left hemisphere	-0.031	0.284	[-0.587 - 0.526]	9.131E-01	9.620E-01
Right hemisphere	-0.117	0.252	[-0.610 - 0.377]	6.429E-01	9.620E-01
Left fusiform gyrus	-0.200	0.207	[-0.605 - 0.206]	3.346E-01	9.620E-01
Right fusiform gyrus	-0.091	0.207	[-0.496 - 0.315]	6.604E-01	9.620E-01
Left pars opercularis of inferior frontal gyrus	-0.261	0.157	[-0.568 - 0.046]	9.562E-02	9.620E-01
Right pars opercularis of inferior frontal gyrus	-0.060	0.234	[-0.519 - 0.398]	7.963E-01	9.620E-01
Left superior temporal gyrus	-0.133	0.315	[-0.750 - 0.484]	6.718E-01	9.620E-01
Right superior temporal gyrus	-0.122	0.209	[-0.533 - 0.288]	5.593E-01	9.620E-01
Left insula	-0.194	0.340	[-0.859 - 0.472]	5.688E-01	9.620E-01
Right insula	-0.139	0.279	[-0.686 - 0.407]	6.175E-01	9.620E-01
Left lingual gyrus	-0.061	0.228	[-0.508 - 0.386]	7.892E-01	9.620E-01
Right lingual gyrus	-0.263	0.244	[-0.742 - 0.215]	2.809E-01	9.620E-01
Left pars triangularis of inferior frontal gyrus	-0.086	0.215	[-0.506 - 0.335]	6.900E-01	9.620E-01
Right pars triangularis of inferior frontal gyrus	-0.134	0.183	[-0.492 - 0.225]	4.659E-01	9.620E-01
Left lateral orbitofrontal cortex	0.167	0.285	[-0.390 - 0.725]	5.563E-01	9.620E-01
Right lateral orbitofrontal cortex	-0.029	0.276	[-0.570 - 0.511]	9.153E-01	9.620E-01
Left rostral middle frontal gyrus	-0.066	0.216	[-0.490 - 0.358]	7.605E-01	9.620E-01
Right rostral middle frontal gyrus	-0.117	0.176	[-0.461 - 0.227]	5.050E-01	9.620E-01
Left middle temporal gyrus	0.212	0.250	[-0.278 - 0.703]	3.964E-01	9.620E-01
Right middle temporal gyrus	0.085	0.157	[-0.222 - 0.393]	5.860E-01	9.620E-01
Left superior frontal gyrus	-0.104	0.283	[-0.658 - 0.450]	7.125E-01	9.620E-01
Right superior frontal gyrus	-0.271	0.188	[-0.639 - 0.097]	1.489E-01	9.620E-01
Left pars orbitalis of inferior frontal gyrus	-0.091	0.194	[-0.472 - 0.290]	6.394E-01	9.620E-01
Right pars orbitalis of inferior frontal gyrus	0.108	0.371	[-0.619 - 0.834]	7.714E-01	9.620E-01
Left medial orbitofrontal cortex	0.073	0.312	[-0.539 - 0.686]	8.141E-01	9.620E-01
Right medial orbitofrontal cortex	-0.256	0.233	[-0.713 - 0.200]	2.712E-01	9.620E-01
Left inferior temporal gyrus	-0.019	0.162	[-0.337 - 0.298]	9.052E-01	9.620E-01
Right inferior temporal gyrus	0.218	0.264	[-0.299 - 0.734]	4.089E-01	9.620E-01
Left isthmus cingulate cortex	0.102	0.156	[-0.205 - 0.408]	5.150E-01	9.620E-01
Right isthmus cingulate cortex	-0.258	0.328	[-0.901 - 0.384]	4.301E-01	9.620E-01
Left banks of superior temporal sulcus	0.046	0.248	[-0.439 - 0.532]	8.521E-01	9.620E-01
Right banks of superior temporal sulcus	-0.209	0.156	[-0.515 - 0.097]	1.804E-01	9.620E-01
Left supramarginal gyrus	-0.078	0.326	[-0.718 - 0.561]	8.103E-01	9.620E-01
Right supramarginal gyrus	-0.328	0.157	[-0.635 - -0.021]	3.625E-02	9.620E-01
Left caudal middle frontal gyrus	-0.124	0.188	[-0.493 - 0.245]	5.098E-01	9.620E-01
Right caudal middle frontal gyrus	-0.219	0.156	[-0.526 - 0.088]	1.616E-01	9.620E-01
Left frontal pole	0.116	0.334	[-0.539 - 0.772]	7.276E-01	9.620E-01
Right frontal pole	-0.053	0.222	[-0.488 - 0.382]	8.116E-01	9.620E-01
Left posterior cingulate cortex	-0.291	0.206	[-0.696 - 0.113]	1.582E-01	9.620E-01
Right posterior cingulate cortex	-0.156	0.349	[-0.841 - 0.529]	6.559E-01	9.620E-01
Left lateral occipital cortex	-0.028	0.202	[-0.423 - 0.368]	8.911E-01	9.620E-01
Right lateral occipital cortex	-0.046	0.156	[-0.352 - 0.259]	7.659E-01	9.620E-01
Left precentral gyrus	-0.038	0.157	[-0.345 - 0.269]	8.075E-01	9.620E-01
Right precentral gyrus	-0.345	0.249	[-0.833 - 0.143]	1.653E-01	9.620E-01
Left parahippocampal gyrus	-0.193	0.349	[-0.877 - 0.490]	5.793E-01	9.620E-01
Right parahippocampal gyrus	-0.222	0.223	[-0.658 - 0.215]	3.196E-01	9.620E-01
Left inferior parietal cortex	0.068	0.181	[-0.287 - 0.422]	7.078E-01	9.620E-01
Right inferior parietal cortex	0.030	0.289	[-0.537 - 0.597]	9.179E-01	9.620E-01
Left transverse temporal gyrus	-0.068	0.194	[-0.449 - 0.313]	7.280E-01	9.620E-01
Right transverse temporal gyrus	-0.210	0.204	[-0.609 - 0.190]	3.032E-01	9.620E-01
Left postcentral gyrus	-0.179	0.207	[-0.586 - 0.227]	3.873E-01	9.620E-01
Right postcentral gyrus	-0.246	0.162	[-0.563 - 0.070]	1.274E-01	9.620E-01
Left precuneus	0.012	0.156	[-0.293 - 0.318]	9.373E-01	9.620E-01
Right precuneus	0.010	0.156	[-0.297 - 0.316]	9.502E-01	9.620E-01
Left caudal anterior cingulate cortex	0.432	0.221	[-0.001 - 0.866]	5.073E-02	9.620E-01
Right caudal anterior cingulate cortex	0.132	0.310	[-0.476 - 0.740]	6.703E-01	9.620E-01
Left cuneus	0.017	0.156	[-0.288 - 0.322]	9.136E-01	9.620E-01
Right cuneus	-0.010	0.156	[-0.316 - 0.296]	9.508E-01	9.620E-01
Left rostral anterior cingulate cortex	-0.195	0.263	[-0.709 - 0.320]	4.588E-01	9.620E-01
Right rostral anterior cingulate cortex	0.075	0.157	[-0.232 - 0.382]	6.304E-01	9.620E-01
Left pericalcarine cortex	-0.032	0.157	[-0.339 - 0.275]	8.365E-01	9.620E-01
Right pericalcarine cortex	-0.165	0.157	[-0.472 - 0.142]	2.925E-01	9.620E-01
Left paracentral lobule	-0.128	0.376	[-0.866 - 0.609]	7.327E-01	9.620E-01
Right paracentral lobule	-0.287	0.245	[-0.767 - 0.192]	2.400E-01	9.620E-01
Left superior parietal cortex	-0.016	0.333	[-0.668 - 0.636]	9.620E-01	9.620E-01
Right superior parietal cortex	-0.170	0.228	[-0.616 - 0.276]	4.557E-01	9.620E-01
Left temporal pole	-0.256	0.225	[-0.697 - 0.184]	2.537E-01	9.620E-01
Right temporal pole	-0.317	0.231	[-0.771 - 0.136]	1.702E-01	9.620E-01
Left entorhinal cortex	-0.181	0.214	[-0.600 - 0.239]	3.981E-01	9.620E-01
Right entorhinal cortex	-0.019	0.294	[-0.594 - 0.556]	9.483E-01	9.620E-01

Supplementary Table S132. Cortical thickness differences between individuals with major depressive disorder not taking antidepressants and healthy comparison subjects controlling for age and sex

	d	Std. Err.	95% CI	*p*-value	FDR *q*-value
Global mean cortical thickness	-0.359	0.210	[-0.771 - 0.053]	8.736E-02	2.215E-01
Left hemisphere	-0.422	0.186	[-0.786 - -0.057]	2.334E-02	1.105E-01
Right hemisphere	-0.288	0.236	[-0.750 - 0.174]	2.220E-01	4.148E-01
Left fusiform gyrus	-0.458	0.147	[-0.747 - -0.169]	1.879E-03	3.257E-02
Right fusiform gyrus	-0.092	0.209	[-0.501 - 0.316]	6.584E-01	7.104E-01
Left pars opercularis of inferior frontal gyrus	-0.291	0.146	[-0.577 - -0.005]	4.583E-02	1.627E-01
Right pars opercularis of inferior frontal gyrus	-0.213	0.204	[-0.613 - 0.187]	2.964E-01	4.529E-01
Left superior temporal gyrus	-0.504	0.147	[-0.792 - -0.217]	5.886E-04	1.791E-02
Right superior temporal gyrus	-0.395	0.147	[-0.683 - -0.107]	7.247E-03	7.351E-02
Left insula	-0.553	0.148	[-0.843 - -0.264]	1.781E-04	1.264E-02
Right insula	-0.466	0.200	[-0.858 - -0.073]	2.003E-02	1.094E-01
Left lingual gyrus	-0.387	0.160	[-0.700 - -0.074]	1.531E-02	1.001E-01
Right lingual gyrus	-0.175	0.196	[-0.560 - 0.210]	3.739E-01	5.205E-01
Left pars triangularis of inferior frontal gyrus	-0.031	0.145	[-0.316 - 0.254]	8.313E-01	8.432E-01
Right pars triangularis of inferior frontal gyrus	-0.282	0.200	[-0.674 - 0.109]	1.573E-01	3.460E-01
Left lateral orbitofrontal cortex	-0.498	0.148	[-0.787 - -0.208]	7.568E-04	1.791E-02
Right lateral orbitofrontal cortex	-0.157	0.146	[-0.443 - 0.130]	2.832E-01	4.529E-01
Left rostral middle frontal gyrus	-0.205	0.186	[-0.569 - 0.159]	2.706E-01	4.529E-01
Right rostral middle frontal gyrus	-0.237	0.216	[-0.661 - 0.187]	2.725E-01	4.529E-01
Left middle temporal gyrus	-0.367	0.171	[-0.703 - -0.031]	3.228E-02	1.432E-01
Right middle temporal gyrus	-0.139	0.252	[-0.633 - 0.355]	5.805E-01	6.699E-01
Left superior frontal gyrus	-0.382	0.160	[-0.696 - -0.069]	1.692E-02	1.001E-01
Right superior frontal gyrus	-0.306	0.146	[-0.593 - -0.019]	3.663E-02	1.529E-01
Left pars orbitalis of inferior frontal gyrus	-0.475	0.190	[-0.846 - -0.103]	1.235E-02	1.001E-01
Right pars orbitalis of inferior frontal gyrus	-0.449	0.147	[-0.737 - -0.160]	2.293E-03	3.257E-02
Left medial orbitofrontal cortex	-0.293	0.235	[-0.754 - 0.168]	2.123E-01	4.118E-01
Right medial orbitofrontal cortex	-0.250	0.200	[-0.642 - 0.143]	2.124E-01	4.118E-01
Left inferior temporal gyrus	-0.285	0.146	[-0.572 - 0.002]	5.124E-02	1.654E-01
Right inferior temporal gyrus	-0.162	0.146	[-0.448 - 0.124]	2.672E-01	4.529E-01
Left isthmus cingulate cortex	-0.090	0.145	[-0.375 - 0.196]	5.384E-01	6.479E-01
Right isthmus cingulate cortex	-0.138	0.215	[-0.560 - 0.283]	5.207E-01	6.479E-01
Left banks of superior temporal sulcus	-0.191	0.177	[-0.539 - 0.156]	2.805E-01	4.529E-01
Right banks of superior temporal sulcus	-0.092	0.213	[-0.509 - 0.326]	6.670E-01	7.104E-01
Left supramarginal gyrus	-0.244	0.174	[-0.585 - 0.097]	1.608E-01	3.460E-01
Right supramarginal gyrus	-0.157	0.264	[-0.674 - 0.360]	5.515E-01	6.526E-01
Left caudal middle frontal gyrus	-0.086	0.190	[-0.458 - 0.286]	6.492E-01	7.104E-01
Right caudal middle frontal gyrus	-0.412	0.149	[-0.705 - -0.119]	5.842E-03	6.913E-02
Left frontal pole	-0.261	0.146	[-0.548 - 0.026]	7.429E-02	2.110E-01
Right frontal pole	-0.265	0.219	[-0.695 - 0.165]	2.278E-01	4.148E-01
Left posterior cingulate cortex	-0.170	0.182	[-0.528 - 0.187]	3.504E-01	4.976E-01
Right posterior cingulate cortex	-0.143	0.173	[-0.482 - 0.197]	4.101E-01	5.494E-01
Left lateral occipital cortex	-0.393	0.191	[-0.767 - -0.019]	3.931E-02	1.529E-01
Right lateral occipital cortex	-0.126	0.204	[-0.525 - 0.272]	5.345E-01	6.479E-01
Left precentral gyrus	-0.356	0.146	[-0.642 - -0.069]	1.503E-02	1.001E-01
Right precentral gyrus	-0.306	0.165	[-0.630 - 0.018]	6.406E-02	1.895E-01
Left parahippocampal gyrus	-0.290	0.280	[-0.839 - 0.259]	2.998E-01	4.529E-01
Right parahippocampal gyrus	-0.164	0.161	[-0.481 - 0.152]	3.092E-01	4.574E-01
Left inferior parietal cortex	-0.280	0.163	[-0.599 - 0.039]	8.583E-02	2.215E-01
Right inferior parietal cortex	-0.133	0.207	[-0.539 - 0.273]	5.222E-01	6.479E-01
Left transverse temporal gyrus	-0.184	0.146	[-0.470 - 0.102]	2.075E-01	4.118E-01
Right transverse temporal gyrus	-0.205	0.246	[-0.686 - 0.277]	4.050E-01	5.494E-01
Left postcentral gyrus	-0.279	0.146	[-0.566 - 0.008]	5.684E-02	1.755E-01
Right postcentral gyrus	-0.311	0.195	[-0.694 - 0.071]	1.103E-01	2.587E-01
Left precuneus	-0.060	0.215	[-0.481 - 0.361]	7.792E-01	8.018E-01
Right precuneus	-0.316	0.160	[-0.629 - -0.003]	4.813E-02	1.627E-01
Left caudal anterior cingulate cortex	-0.151	0.145	[-0.436 - 0.134]	2.998E-01	4.529E-01
Right caudal anterior cingulate cortex	-0.080	0.175	[-0.423 - 0.264]	6.504E-01	7.104E-01
Left cuneus	-0.299	0.146	[-0.585 - -0.012]	4.091E-02	1.529E-01
Right cuneus	-0.247	0.152	[-0.544 - 0.050]	1.037E-01	2.538E-01
Left rostral anterior cingulate cortex	-0.334	0.147	[-0.622 - -0.047]	2.254E-02	1.105E-01
Right rostral anterior cingulate cortex	-0.052	0.152	[-0.351 - 0.247]	7.327E-01	7.650E-01
Left pericalcarine cortex	-0.354	0.146	[-0.641 - -0.067]	1.557E-02	1.001E-01
Right pericalcarine cortex	-0.143	0.149	[-0.435 - 0.149]	3.360E-01	4.868E-01
Left paracentral lobule	-0.127	0.233	[-0.585 - 0.330]	5.849E-01	6.699E-01
Right paracentral lobule	-0.354	0.223	[-0.791 - 0.084]	1.130E-01	2.587E-01
Left superior parietal cortex	-0.256	0.207	[-0.661 - 0.149]	2.146E-01	4.118E-01
Right superior parietal cortex	-0.165	0.246	[-0.648 - 0.318]	5.036E-01	6.479E-01
Left temporal pole	-0.341	0.193	[-0.720 - 0.038]	7.806E-02	2.132E-01
Right temporal pole	-0.062	0.145	[-0.347 - 0.223]	6.704E-01	7.104E-01
Left entorhinal cortex	-0.002	0.284	[-0.558 - 0.553]	9.937E-01	9.937E-01
Right entorhinal cortex	0.229	0.341	[-0.440 - 0.898]	5.024E-01	6.479E-01

Supplementary Table S133. Cortical thickness differences between individuals with major depressive disorder not taking antidepressants and healthy comparison subjects controlling for age and sex over 21 years of old

	d	Std. Err.	95% CI	*p*-value	FDR *q*-value
Global mean cortical thickness	-0.358	0.262	[-0.872 - 0.156]	1.720E-01	3.701E-01
Left hemisphere	-0.427	0.238	[-0.893 - 0.039]	7.233E-02	2.193E-01
Right hemisphere	-0.279	0.287	[-0.841 - 0.283]	3.304E-01	5.117E-01
Left fusiform gyrus	-0.435	0.160	[-0.748 - -0.121]	6.531E-03	5.796E-02
Right fusiform gyrus	-0.153	0.248	[-0.638 - 0.333]	5.383E-01	6.705E-01
Left pars opercularis of inferior frontal gyrus	-0.317	0.158	[-0.626 - -0.007]	4.513E-02	2.136E-01
Right pars opercularis of inferior frontal gyrus	-0.154	0.244	[-0.633 - 0.325]	5.291E-01	6.705E-01
Left superior temporal gyrus	-0.510	0.159	[-0.822 - -0.197]	1.376E-03	1.954E-02
Right superior temporal gyrus	-0.400	0.178	[-0.749 - -0.051]	2.448E-02	1.337E-01
Left insula	-0.558	0.160	[-0.872 - -0.244]	5.017E-04	1.954E-02
Right insula	-0.389	0.222	[-0.824 - 0.046]	7.972E-02	2.193E-01
Left lingual gyrus	-0.306	0.169	[-0.637 - 0.024]	6.931E-02	2.193E-01
Right lingual gyrus	-0.059	0.225	[-0.500 - 0.382]	7.940E-01	8.029E-01
Left pars triangularis of inferior frontal gyrus	-0.048	0.157	[-0.357 - 0.260]	7.584E-01	8.029E-01
Right pars triangularis of inferior frontal gyrus	-0.331	0.246	[-0.812 - 0.151]	1.788E-01	3.733E-01
Left lateral orbitofrontal cortex	-0.561	0.198	[-0.948 - -0.174]	4.537E-03	4.602E-02
Right lateral orbitofrontal cortex	-0.250	0.158	[-0.560 - 0.061]	1.153E-01	2.729E-01
Left rostral middle frontal gyrus	-0.403	0.159	[-0.715 - -0.091]	1.125E-02	8.873E-02
Right rostral middle frontal gyrus	-0.297	0.262	[-0.810 - 0.217]	2.574E-01	4.351E-01
Left middle temporal gyrus	-0.484	0.159	[-0.796 - -0.171]	2.396E-03	2.835E-02
Right middle temporal gyrus	-0.188	0.294	[-0.763 - 0.388]	5.232E-01	6.705E-01
Left superior frontal gyrus	-0.388	0.203	[-0.785 - 0.009]	5.548E-02	2.193E-01
Right superior frontal gyrus	-0.314	0.167	[-0.642 - 0.014]	6.037E-02	2.193E-01
Left pars orbitalis of inferior frontal gyrus	-0.402	0.232	[-0.857 - 0.052]	8.264E-02	2.193E-01
Right pars orbitalis of inferior frontal gyrus	-0.517	0.160	[-0.831 - -0.204]	1.234E-03	1.954E-02
Left medial orbitofrontal cortex	-0.277	0.292	[-0.849 - 0.295]	3.423E-01	5.117E-01
Right medial orbitofrontal cortex	-0.187	0.249	[-0.675 - 0.300]	4.510E-01	6.157E-01
Left inferior temporal gyrus	-0.245	0.200	[-0.637 - 0.146]	2.193E-01	3.901E-01
Right inferior temporal gyrus	-0.199	0.162	[-0.516 - 0.119]	2.198E-01	3.901E-01
Left isthmus cingulate cortex	-0.104	0.157	[-0.413 - 0.204]	5.073E-01	6.670E-01
Right isthmus cingulate cortex	-0.296	0.158	[-0.605 - 0.013]	6.035E-02	2.193E-01
Left banks of superior temporal sulcus	-0.190	0.222	[-0.626 - 0.246]	3.939E-01	5.593E-01
Right banks of superior temporal sulcus	-0.082	0.250	[-0.571 - 0.408]	7.438E-01	8.029E-01
Left supramarginal gyrus	-0.250	0.218	[-0.678 - 0.178]	2.520E-01	4.351E-01
Right supramarginal gyrus	-0.078	0.297	[-0.660 - 0.505]	7.941E-01	8.029E-01
Left caudal middle frontal gyrus	-0.224	0.178	[-0.572 - 0.125]	2.084E-01	3.901E-01
Right caudal middle frontal gyrus	-0.531	0.160	[-0.845 - -0.218]	9.025E-04	1.954E-02
Left frontal pole	-0.301	0.172	[-0.638 - 0.036]	8.011E-02	2.193E-01
Right frontal pole	-0.256	0.272	[-0.788 - 0.276]	3.459E-01	5.117E-01
Left posterior cingulate cortex	-0.315	0.184	[-0.675 - 0.045]	8.649E-02	2.193E-01
Right posterior cingulate cortex	-0.271	0.158	[-0.580 - 0.038]	8.597E-02	2.193E-01
Left lateral occipital cortex	-0.393	0.224	[-0.832 - 0.047]	7.976E-02	2.193E-01
Right lateral occipital cortex	-0.059	0.227	[-0.504 - 0.386]	7.946E-01	8.029E-01
Left precentral gyrus	-0.354	0.169	[-0.685 - -0.024]	3.552E-02	1.801E-01
Right precentral gyrus	-0.335	0.191	[-0.709 - 0.039]	7.955E-02	2.193E-01
Left parahippocampal gyrus	-0.367	0.349	[-1.051 - 0.318]	2.936E-01	4.738E-01
Right parahippocampal gyrus	-0.200	0.210	[-0.612 - 0.212]	3.413E-01	5.117E-01
Left inferior parietal cortex	-0.241	0.194	[-0.621 - 0.139]	2.130E-01	3.901E-01
Right inferior parietal cortex	-0.169	0.246	[-0.651 - 0.314]	4.931E-01	6.606E-01
Left transverse temporal gyrus	-0.195	0.158	[-0.505 - 0.114]	2.162E-01	3.901E-01
Right transverse temporal gyrus	-0.084	0.267	[-0.609 - 0.440]	7.525E-01	8.029E-01
Left postcentral gyrus	-0.340	0.180	[-0.693 - 0.013]	5.926E-02	2.193E-01
Right postcentral gyrus	-0.326	0.212	[-0.742 - 0.089]	1.235E-01	2.829E-01
Left precuneus	-0.128	0.247	[-0.612 - 0.357]	6.050E-01	7.281E-01
Right precuneus	-0.326	0.199	[-0.717 - 0.064]	1.014E-01	2.482E-01
Left caudal anterior cingulate cortex	-0.201	0.157	[-0.509 - 0.107]	2.013E-01	3.901E-01
Right caudal anterior cingulate cortex	-0.114	0.193	[-0.493 - 0.266]	5.568E-01	6.816E-01
Left cuneus	-0.365	0.158	[-0.675 - -0.055]	2.121E-02	1.295E-01
Right cuneus	-0.171	0.162	[-0.488 - 0.146]	2.914E-01	4.738E-01
Left rostral anterior cingulate cortex	-0.515	0.159	[-0.827 - -0.202]	1.240E-03	1.954E-02
Right rostral anterior cingulate cortex	-0.060	0.174	[-0.401 - 0.281]	7.320E-01	8.029E-01
Left pericalcarine cortex	-0.388	0.158	[-0.699 - -0.077]	1.432E-02	1.017E-01
Right pericalcarine cortex	-0.055	0.158	[-0.364 - 0.254]	7.286E-01	8.029E-01
Left paracentral lobule	-0.069	0.278	[-0.614 - 0.476]	8.029E-01	8.029E-01
Right paracentral lobule	-0.375	0.264	[-0.892 - 0.141]	1.545E-01	3.429E-01
Left superior parietal cortex	-0.214	0.246	[-0.695 - 0.268]	3.847E-01	5.575E-01
Right superior parietal cortex	-0.144	0.297	[-0.725 - 0.438]	6.277E-01	7.427E-01
Left temporal pole	-0.460	0.201	[-0.853 - -0.067]	2.189E-02	1.295E-01
Right temporal pole	-0.057	0.157	[-0.366 - 0.251]	7.153E-01	8.029E-01
Left entorhinal cortex	0.152	0.341	[-0.516 - 0.821]	6.555E-01	7.630E-01
Right entorhinal cortex	0.312	0.410	[-0.491 - 1.114]	4.467E-01	6.157E-01

Supplementary Table S134. Cortical thickness differences between individuals with major depressive disorder taking antidepressants and healthy comparison subjects controlling for age and sex

	d	Std. Err.	95% CI	*p*-value	FDR *q*-value
Global mean cortical thickness	-0.305	0.124	[-0.548 - -0.063]	1.366E-02	4.620E-02
Left hemisphere	-0.330	0.125	[-0.576 - -0.084]	8.454E-03	3.335E-02
Right hemisphere	-0.266	0.119	[-0.499 - -0.032]	2.584E-02	6.552E-02
Left fusiform gyrus	-0.255	0.086	[-0.423 - -0.087]	2.991E-03	2.093E-02
Right fusiform gyrus	-0.226	0.093	[-0.408 - -0.045]	1.443E-02	4.658E-02
Left pars opercularis of inferior frontal gyrus	-0.286	0.085	[-0.452 - -0.120]	7.554E-04	7.662E-03
Right pars opercularis of inferior frontal gyrus	-0.171	0.122	[-0.409 - 0.068]	1.607E-01	2.487E-01
Left superior temporal gyrus	-0.286	0.080	[-0.443 - -0.128]	3.697E-04	7.475E-03
Right superior temporal gyrus	-0.202	0.087	[-0.373 - -0.031]	2.068E-02	5.646E-02
Left insula	-0.192	0.087	[-0.364 - -0.021]	2.753E-02	6.739E-02
Right insula	-0.190	0.112	[-0.409 - 0.028]	8.779E-02	1.602E-01
Left lingual gyrus	-0.169	0.089	[-0.344 - 0.005]	5.719E-02	1.194E-01
Right lingual gyrus	-0.164	0.094	[-0.349 - 0.020]	8.101E-02	1.598E-01
Left pars triangularis of inferior frontal gyrus	-0.210	0.088	[-0.382 - -0.038]	1.647E-02	4.871E-02
Right pars triangularis of inferior frontal gyrus	-0.185	0.108	[-0.396 - 0.026]	8.573E-02	1.602E-01
Left lateral orbitofrontal cortex	-0.322	0.093	[-0.505 - -0.139]	5.723E-04	7.535E-03
Right lateral orbitofrontal cortex	-0.261	0.091	[-0.440 - -0.083]	4.179E-03	2.119E-02
Left rostral middle frontal gyrus	-0.355	0.125	[-0.600 - -0.110]	4.565E-03	2.161E-02
Right rostral middle frontal gyrus	-0.206	0.106	[-0.414 - 0.003]	5.290E-02	1.138E-01
Left middle temporal gyrus	-0.375	0.102	[-0.575 - -0.176]	2.275E-04	7.475E-03
Right middle temporal gyrus	-0.298	0.103	[-0.501 - -0.096]	3.833E-03	2.093E-02
Left superior frontal gyrus	-0.271	0.105	[-0.478 - -0.065]	1.011E-02	3.592E-02
Right superior frontal gyrus	-0.273	0.086	[-0.442 - -0.104]	1.559E-03	1.384E-02
Left pars orbitalis of inferior frontal gyrus	-0.275	0.095	[-0.460 - -0.090]	3.653E-03	2.093E-02
Right pars orbitalis of inferior frontal gyrus	-0.314	0.080	[-0.471 - -0.157]	8.955E-05	6.358E-03
Left medial orbitofrontal cortex	-0.281	0.109	[-0.496 - -0.067]	1.012E-02	3.592E-02
Right medial orbitofrontal cortex	-0.248	0.085	[-0.415 - -0.081]	3.653E-03	2.093E-02
Left inferior temporal gyrus	-0.225	0.076	[-0.374 - -0.075]	3.256E-03	2.093E-02
Right inferior temporal gyrus	-0.247	0.070	[-0.385 - -0.110]	4.211E-04	7.475E-03
Left isthmus cingulate cortex	-0.175	0.088	[-0.348 - -0.002]	4.740E-02	1.086E-01
Right isthmus cingulate cortex	-0.120	0.086	[-0.288 - 0.048]	1.611E-01	2.487E-01
Left banks of superior temporal sulcus	-0.200	0.083	[-0.364 - -0.037]	1.605E-02	4.871E-02
Right banks of superior temporal sulcus	-0.108	0.101	[-0.306 - 0.091]	2.882E-01	3.590E-01
Left supramarginal gyrus	-0.234	0.100	[-0.430 - -0.038]	1.929E-02	5.477E-02
Right supramarginal gyrus	-0.168	0.096	[-0.356 - 0.019]	7.896E-02	1.598E-01
Left caudal middle frontal gyrus	-0.194	0.114	[-0.419 - 0.030]	8.961E-02	1.602E-01
Right caudal middle frontal gyrus	-0.210	0.093	[-0.392 - -0.028]	2.353E-02	6.187E-02
Left frontal pole	-0.155	0.080	[-0.311 - 0.001]	5.128E-02	1.138E-01
Right frontal pole	-0.152	0.113	[-0.373 - 0.069]	1.770E-01	2.618E-01
Left posterior cingulate cortex	-0.220	0.065	[-0.347 - -0.094]	6.368E-04	7.535E-03
Right posterior cingulate cortex	-0.150	0.071	[-0.290 - -0.010]	3.529E-02	8.352E-02
Left lateral occipital cortex	-0.156	0.094	[-0.341 - 0.029]	9.828E-02	1.702E-01
Right lateral occipital cortex	-0.132	0.106	[-0.340 - 0.076]	2.135E-01	2.860E-01
Left precentral gyrus	-0.271	0.100	[-0.468 - -0.074]	6.915E-03	3.068E-02
Right precentral gyrus	-0.210	0.079	[-0.364 - -0.056]	7.528E-03	3.144E-02
Left parahippocampal gyrus	-0.110	0.086	[-0.279 - 0.059]	2.019E-01	2.811E-01
Right parahippocampal gyrus	-0.033	0.074	[-0.178 - 0.112]	6.578E-01	6.868E-01
Left inferior parietal cortex	-0.160	0.102	[-0.360 - 0.040]	1.171E-01	1.933E-01
Right inferior parietal cortex	-0.158	0.114	[-0.380 - 0.065]	1.647E-01	2.487E-01
Left transverse temporal gyrus	-0.107	0.096	[-0.295 - 0.082]	2.684E-01	3.525E-01
Right transverse temporal gyrus	-0.059	0.096	[-0.248 - 0.129]	5.371E-01	6.002E-01
Left postcentral gyrus	-0.137	0.109	[-0.350 - 0.076]	2.081E-01	2.842E-01
Right postcentral gyrus	-0.153	0.097	[-0.343 - 0.037]	1.138E-01	1.924E-01
Left precuneus	-0.099	0.098	[-0.292 - 0.094]	3.134E-01	3.837E-01
Right precuneus	-0.132	0.078	[-0.285 - 0.021]	9.025E-02	1.602E-01
Left caudal anterior cingulate cortex	-0.084	0.064	[-0.210 - 0.042]	1.919E-01	2.725E-01
Right caudal anterior cingulate cortex	-0.069	0.064	[-0.196 - 0.057]	2.822E-01	3.578E-01
Left cuneus	-0.068	0.081	[-0.227 - 0.091]	4.029E-01	4.849E-01
Right cuneus	-0.052	0.067	[-0.183 - 0.078]	4.347E-01	5.144E-01
Left rostral anterior cingulate cortex	-0.121	0.078	[-0.275 - 0.032]	1.212E-01	1.956E-01
Right rostral anterior cingulate cortex	-0.004	0.079	[-0.159 - 0.152]	9.629E-01	9.629E-01
Left pericalcarine cortex	0.026	0.064	[-0.100 - 0.152]	6.896E-01	7.096E-01
Right pericalcarine cortex	0.048	0.093	[-0.134 - 0.230]	6.030E-01	6.487E-01
Left paracentral lobule	-0.119	0.090	[-0.295 - 0.056]	1.819E-01	2.636E-01
Right paracentral lobule	-0.106	0.097	[-0.295 - 0.083]	2.731E-01	3.525E-01
Left superior parietal cortex	-0.068	0.104	[-0.272 - 0.137]	5.157E-01	5.913E-01
Right superior parietal cortex	-0.044	0.094	[-0.228 - 0.141]	6.421E-01	6.804E-01
Left temporal pole	-0.043	0.070	[-0.179 - 0.094]	5.410E-01	6.002E-01
Right temporal pole	0.055	0.095	[-0.132 - 0.242]	5.649E-01	6.170E-01
Left entorhinal cortex	-0.062	0.095	[-0.248 - 0.125]	5.163E-01	5.913E-01
Right entorhinal cortex	-0.038	0.106	[-0.245 - 0.169]	7.168E-01	7.270E-01

Supplementary Table S135. Cortical thickness differences between individuals with major depressive disorder taking antidepressants and healthy comparison subjects controlling for age and sex over 21 years of old

	d	Std. Err.	95% CI	*p*-value	FDR *q*-value
Global mean cortical thickness	-0.307	0.127	[-0.557 - -0.058]	1.574E-02	4.725E-02
Left hemisphere	-0.332	0.129	[-0.585 - -0.079]	1.020E-02	4.023E-02
Right hemisphere	-0.269	0.122	[-0.508 - -0.029]	2.807E-02	6.873E-02
Left fusiform gyrus	-0.267	0.086	[-0.436 - -0.098]	1.947E-03	1.728E-02
Right fusiform gyrus	-0.225	0.093	[-0.407 - -0.043]	1.515E-02	4.725E-02
Left pars opercularis of inferior frontal gyrus	-0.285	0.087	[-0.455 - -0.114]	1.085E-03	1.531E-02
Right pars opercularis of inferior frontal gyrus	-0.181	0.121	[-0.419 - 0.057]	1.357E-01	2.141E-01
Left superior temporal gyrus	-0.289	0.085	[-0.455 - -0.123]	6.283E-04	1.396E-02
Right superior temporal gyrus	-0.209	0.090	[-0.385 - -0.032]	2.065E-02	5.640E-02
Left insula	-0.204	0.091	[-0.383 - -0.026]	2.446E-02	6.325E-02
Right insula	-0.188	0.112	[-0.408 - 0.032]	9.361E-02	1.704E-01
Left lingual gyrus	-0.152	0.091	[-0.330 - 0.026]	9.343E-02	1.704E-01
Right lingual gyrus	-0.149	0.097	[-0.338 - 0.041]	1.243E-01	2.006E-01
Left pars triangularis of inferior frontal gyrus	-0.202	0.090	[-0.378 - -0.025]	2.494E-02	6.325E-02
Right pars triangularis of inferior frontal gyrus	-0.193	0.112	[-0.413 - 0.027]	8.502E-02	1.677E-01
Left lateral orbitofrontal cortex	-0.316	0.100	[-0.511 - -0.121]	1.510E-03	1.531E-02
Right lateral orbitofrontal cortex	-0.274	0.095	[-0.459 - -0.088]	3.909E-03	2.343E-02
Left rostral middle frontal gyrus	-0.371	0.132	[-0.630 - -0.111]	5.069E-03	2.399E-02
Right rostral middle frontal gyrus	-0.213	0.113	[-0.435 - 0.010]	6.099E-02	1.312E-01
Left middle temporal gyrus	-0.387	0.105	[-0.593 - -0.182]	2.237E-04	8.871E-03
Right middle temporal gyrus	-0.311	0.107	[-0.521 - -0.100]	3.790E-03	2.343E-02
Left superior frontal gyrus	-0.270	0.112	[-0.490 - -0.050]	1.597E-02	4.725E-02
Right superior frontal gyrus	-0.274	0.093	[-0.457 - -0.092]	3.210E-03	2.343E-02
Left pars orbitalis of inferior frontal gyrus	-0.272	0.096	[-0.461 - -0.084]	4.620E-03	2.343E-02
Right pars orbitalis of inferior frontal gyrus	-0.314	0.086	[-0.482 - -0.146]	2.499E-04	8.871E-03
Left medial orbitofrontal cortex	-0.283	0.115	[-0.508 - -0.058]	1.354E-02	4.725E-02
Right medial orbitofrontal cortex	-0.253	0.089	[-0.428 - -0.079]	4.461E-03	2.343E-02
Left inferior temporal gyrus	-0.225	0.078	[-0.379 - -0.072]	4.008E-03	2.343E-02
Right inferior temporal gyrus	-0.247	0.073	[-0.391 - -0.103]	7.864E-04	1.396E-02
Left isthmus cingulate cortex	-0.170	0.087	[-0.340 - 0.000]	4.991E-02	1.107E-01
Right isthmus cingulate cortex	-0.117	0.087	[-0.287 - 0.053]	1.777E-01	2.574E-01
Left banks of superior temporal sulcus	-0.208	0.088	[-0.381 - -0.036]	1.806E-02	5.130E-02
Right banks of superior temporal sulcus	-0.113	0.099	[-0.306 - 0.081]	2.551E-01	3.293E-01
Left supramarginal gyrus	-0.248	0.103	[-0.449 - -0.047]	1.566E-02	4.725E-02
Right supramarginal gyrus	-0.171	0.095	[-0.357 - 0.015]	7.202E-02	1.475E-01
Left caudal middle frontal gyrus	-0.197	0.117	[-0.426 - 0.031]	9.100E-02	1.704E-01
Right caudal middle frontal gyrus	-0.222	0.092	[-0.403 - -0.042]	1.578E-02	4.725E-02
Left frontal pole	-0.172	0.085	[-0.339 - -0.006]	4.206E-02	9.634E-02
Right frontal pole	-0.171	0.118	[-0.402 - 0.060]	1.476E-01	2.265E-01
Left posterior cingulate cortex	-0.209	0.065	[-0.337 - -0.081]	1.395E-03	1.531E-02
Right posterior cingulate cortex	-0.157	0.073	[-0.300 - -0.013]	3.210E-02	7.597E-02
Left lateral occipital cortex	-0.155	0.096	[-0.343 - 0.034]	1.074E-01	1.816E-01
Right lateral occipital cortex	-0.127	0.106	[-0.335 - 0.081]	2.313E-01	3.042E-01
Left precentral gyrus	-0.276	0.100	[-0.472 - -0.079]	5.956E-03	2.643E-02
Right precentral gyrus	-0.213	0.079	[-0.368 - -0.058]	7.198E-03	3.006E-02
Left parahippocampal gyrus	-0.101	0.084	[-0.265 - 0.064]	2.291E-01	3.042E-01
Right parahippocampal gyrus	-0.024	0.073	[-0.167 - 0.119]	7.384E-01	7.598E-01
Left inferior parietal cortex	-0.162	0.100	[-0.359 - 0.035]	1.071E-01	1.816E-01
Right inferior parietal cortex	-0.155	0.109	[-0.369 - 0.058]	1.537E-01	2.274E-01
Left transverse temporal gyrus	-0.100	0.098	[-0.292 - 0.092]	3.082E-01	3.908E-01
Right transverse temporal gyrus	-0.060	0.097	[-0.250 - 0.130]	5.348E-01	6.266E-01
Left postcentral gyrus	-0.133	0.111	[-0.350 - 0.084]	2.287E-01	3.042E-01
Right postcentral gyrus	-0.159	0.101	[-0.357 - 0.038]	1.132E-01	1.870E-01
Left precuneus	-0.093	0.097	[-0.282 - 0.097]	3.377E-01	4.134E-01
Right precuneus	-0.136	0.082	[-0.297 - 0.025]	9.872E-02	1.752E-01
Left caudal anterior cingulate cortex	-0.094	0.065	[-0.222 - 0.034]	1.499E-01	2.265E-01
Right caudal anterior cingulate cortex	-0.080	0.066	[-0.210 - 0.050]	2.263E-01	3.042E-01
Left cuneus	-0.051	0.081	[-0.209 - 0.107]	5.251E-01	6.266E-01
Right cuneus	-0.041	0.071	[-0.181 - 0.099]	5.650E-01	6.304E-01
Left rostral anterior cingulate cortex	-0.144	0.080	[-0.301 - 0.013]	7.269E-02	1.475E-01
Right rostral anterior cingulate cortex	-0.019	0.079	[-0.175 - 0.136]	8.059E-01	8.059E-01
Left pericalcarine cortex	0.029	0.065	[-0.099 - 0.157]	6.554E-01	6.862E-01
Right pericalcarine cortex	0.051	0.094	[-0.132 - 0.235]	5.836E-01	6.355E-01
Left paracentral lobule	-0.110	0.088	[-0.282 - 0.063]	2.116E-01	3.005E-01
Right paracentral lobule	-0.093	0.096	[-0.281 - 0.094]	3.295E-01	4.104E-01
Left superior parietal cortex	-0.059	0.103	[-0.260 - 0.143]	5.682E-01	6.304E-01
Right superior parietal cortex	-0.041	0.093	[-0.223 - 0.141]	6.572E-01	6.862E-01
Left temporal pole	-0.044	0.073	[-0.188 - 0.100]	5.467E-01	6.266E-01
Right temporal pole	0.052	0.096	[-0.137 - 0.241]	5.907E-01	6.355E-01
Left entorhinal cortex	-0.058	0.096	[-0.245 - 0.130]	5.472E-01	6.266E-01
Right entorhinal cortex	-0.033	0.107	[-0.242 - 0.176]	7.558E-01	7.666E-01

Supplementary Table S136. Cortical thickness differences between individuals with major depressive disorder taking antidepressants and individuals with major depressive disorder not taking antidepressants controlling for age and sex

	d	Std. Err.	95% CI	*p*-value	FDR *q*-value
Global mean cortical thickness	-0.183	0.266	[-0.704 - 0.337]	4.899E-01	8.828E-01
Left hemisphere	-0.173	0.280	[-0.722 - 0.377]	5.377E-01	8.828E-01
Right hemisphere	-0.185	0.249	[-0.672 - 0.302]	4.574E-01	8.828E-01
Left fusiform gyrus	0.001	0.133	[-0.260 - 0.262]	9.951E-01	9.951E-01
Right fusiform gyrus	-0.219	0.231	[-0.671 - 0.233]	3.423E-01	8.828E-01
Left pars opercularis of inferior frontal gyrus	-0.067	0.189	[-0.436 - 0.303]	7.238E-01	9.517E-01
Right pars opercularis of inferior frontal gyrus	0.116	0.133	[-0.146 - 0.377]	3.850E-01	8.828E-01
Left superior temporal gyrus	0.053	0.157	[-0.255 - 0.361]	7.382E-01	9.529E-01
Right superior temporal gyrus	0.156	0.133	[-0.105 - 0.417]	2.421E-01	8.828E-01
Left insula	0.139	0.133	[-0.122 - 0.400]	2.968E-01	8.828E-01
Right insula	0.143	0.133	[-0.118 - 0.405]	2.830E-01	8.828E-01
Left lingual gyrus	0.119	0.133	[-0.142 - 0.380]	3.711E-01	8.828E-01
Right lingual gyrus	-0.080	0.133	[-0.342 - 0.181]	5.463E-01	8.828E-01
Left pars triangularis of inferior frontal gyrus	-0.200	0.134	[-0.462 - 0.062]	1.349E-01	8.828E-01
Right pars triangularis of inferior frontal gyrus	0.125	0.133	[-0.136 - 0.387]	3.478E-01	8.828E-01
Left lateral orbitofrontal cortex	-0.075	0.133	[-0.336 - 0.186]	5.719E-01	8.828E-01
Right lateral orbitofrontal cortex	-0.180	0.133	[-0.441 - 0.081]	1.764E-01	8.828E-01
Left rostral middle frontal gyrus	-0.213	0.208	[-0.621 - 0.195]	3.058E-01	8.828E-01
Right rostral middle frontal gyrus	-0.136	0.185	[-0.499 - 0.227]	4.632E-01	8.828E-01
Left middle temporal gyrus	-0.030	0.189	[-0.402 - 0.341]	8.725E-01	9.612E-01
Right middle temporal gyrus	-0.085	0.146	[-0.371 - 0.202]	5.627E-01	8.828E-01
Left superior frontal gyrus	-0.010	0.164	[-0.331 - 0.311]	9.504E-01	9.640E-01
Right superior frontal gyrus	-0.127	0.163	[-0.446 - 0.192]	4.340E-01	8.828E-01
Left pars orbitalis of inferior frontal gyrus	0.020	0.191	[-0.354 - 0.393]	9.183E-01	9.612E-01
Right pars orbitalis of inferior frontal gyrus	0.015	0.133	[-0.246 - 0.275]	9.121E-01	9.612E-01
Left medial orbitofrontal cortex	-0.096	0.147	[-0.384 - 0.193]	5.156E-01	8.828E-01
Right medial orbitofrontal cortex	-0.143	0.133	[-0.403 - 0.118]	2.840E-01	8.828E-01
Left inferior temporal gyrus	-0.094	0.133	[-0.355 - 0.167]	4.811E-01	8.828E-01
Right inferior temporal gyrus	-0.115	0.186	[-0.480 - 0.250]	5.376E-01	8.828E-01
Left isthmus cingulate cortex	-0.187	0.133	[-0.449 - 0.074]	1.609E-01	8.828E-01
Right isthmus cingulate cortex	-0.022	0.202	[-0.419 - 0.374]	9.115E-01	9.612E-01
Left banks of superior temporal sulcus	0.009	0.133	[-0.253 - 0.270]	9.484E-01	9.640E-01
Right banks of superior temporal sulcus	0.026	0.133	[-0.235 - 0.287]	8.440E-01	9.612E-01
Left supramarginal gyrus	-0.310	0.273	[-0.846 - 0.226]	2.569E-01	8.828E-01
Right supramarginal gyrus	-0.239	0.276	[-0.780 - 0.302]	3.868E-01	8.828E-01
Left caudal middle frontal gyrus	-0.138	0.232	[-0.593 - 0.317]	5.533E-01	8.828E-01
Right caudal middle frontal gyrus	0.147	0.133	[-0.114 - 0.408]	2.700E-01	8.828E-01
Left frontal pole	-0.183	0.270	[-0.712 - 0.345]	4.965E-01	8.828E-01
Right frontal pole	-0.081	0.136	[-0.348 - 0.186]	5.518E-01	8.828E-01
Left posterior cingulate cortex	-0.040	0.210	[-0.451 - 0.371]	8.476E-01	9.612E-01
Right posterior cingulate cortex	0.063	0.166	[-0.264 - 0.389]	7.071E-01	9.472E-01
Left lateral occipital cortex	0.037	0.268	[-0.488 - 0.562]	8.909E-01	9.612E-01
Right lateral occipital cortex	-0.267	0.268	[-0.792 - 0.258]	3.188E-01	8.828E-01
Left precentral gyrus	-0.040	0.169	[-0.373 - 0.292]	8.113E-01	9.612E-01
Right precentral gyrus	-0.130	0.159	[-0.443 - 0.182]	4.132E-01	8.828E-01
Left parahippocampal gyrus	0.263	0.207	[-0.143 - 0.669]	2.037E-01	8.828E-01
Right parahippocampal gyrus	0.119	0.133	[-0.142 - 0.380]	3.711E-01	8.828E-01
Left inferior parietal cortex	-0.129	0.250	[-0.619 - 0.361]	6.050E-01	9.139E-01
Right inferior parietal cortex	-0.245	0.283	[-0.800 - 0.309]	3.864E-01	8.828E-01
Left transverse temporal gyrus	-0.155	0.133	[-0.417 - 0.107]	2.457E-01	8.828E-01
Right transverse temporal gyrus	-0.090	0.226	[-0.533 - 0.353]	6.910E-01	9.472E-01
Left postcentral gyrus	-0.066	0.171	[-0.401 - 0.270]	7.010E-01	9.472E-01
Right postcentral gyrus	-0.092	0.199	[-0.483 - 0.299]	6.442E-01	9.257E-01
Left precuneus	-0.125	0.277	[-0.669 - 0.419]	6.519E-01	9.257E-01
Right precuneus	0.158	0.185	[-0.205 - 0.521]	3.943E-01	8.828E-01
Left caudal anterior cingulate cortex	0.079	0.133	[-0.182 - 0.340]	5.509E-01	8.828E-01
Right caudal anterior cingulate cortex	-0.112	0.133	[-0.373 - 0.150]	4.016E-01	8.828E-01
Left cuneus	0.061	0.134	[-0.201 - 0.323]	6.468E-01	9.257E-01
Right cuneus	0.034	0.133	[-0.227 - 0.295]	7.992E-01	9.612E-01
Left rostral anterior cingulate cortex	0.135	0.133	[-0.127 - 0.396]	3.129E-01	8.828E-01
Right rostral anterior cingulate cortex	0.021	0.133	[-0.240 - 0.281]	8.754E-01	9.612E-01
Left pericalcarine cortex	0.182	0.185	[-0.181 - 0.545]	3.253E-01	8.828E-01
Right pericalcarine cortex	-0.017	0.174	[-0.357 - 0.323]	9.206E-01	9.612E-01
Left paracentral lobule	-0.205	0.296	[-0.786 - 0.376]	4.886E-01	8.828E-01
Right paracentral lobule	0.205	0.265	[-0.314 - 0.724]	4.389E-01	8.828E-01
Left superior parietal cortex	-0.070	0.291	[-0.641 - 0.501]	8.105E-01	9.612E-01
Right superior parietal cortex	-0.049	0.282	[-0.602 - 0.503]	8.607E-01	9.612E-01
Left temporal pole	0.119	0.170	[-0.215 - 0.452]	4.855E-01	8.828E-01
Right temporal pole	-0.173	0.133	[-0.434 - 0.089]	1.957E-01	8.828E-01
Left entorhinal cortex	-0.257	0.205	[-0.659 - 0.146]	2.113E-01	8.828E-01
Right entorhinal cortex	-0.463	0.212	[-0.879 - -0.048]	2.881E-02	8.828E-01

Supplementary Table S137. Cortical thickness differences between individuals with major depressive disorder taking antidepressants and individuals with major depressive disorder not taking antidepressants controlling for age and sex over 21 years of old

	d	Std. Err.	95% CI	*p*-value	FDR *q*-value
Global mean cortical thickness	-0.009	0.248	[-0.495 - 0.477]	9.722E-01	9.950E-01
Left hemisphere	0.017	0.268	[-0.508 - 0.543]	9.488E-01	9.950E-01
Right hemisphere	-0.033	0.231	[-0.486 - 0.419]	8.848E-01	9.950E-01
Left fusiform gyrus	-0.001	0.141	[-0.278 - 0.276]	9.950E-01	9.950E-01
Right fusiform gyrus	-0.088	0.207	[-0.494 - 0.319]	6.716E-01	9.950E-01
Left pars opercularis of inferior frontal gyrus	-0.037	0.255	[-0.538 - 0.464]	8.851E-01	9.950E-01
Right pars opercularis of inferior frontal gyrus	0.056	0.185	[-0.307 - 0.418]	7.636E-01	9.950E-01
Left superior temporal gyrus	0.149	0.218	[-0.278 - 0.576]	4.936E-01	9.950E-01
Right superior temporal gyrus	0.187	0.142	[-0.091 - 0.464]	1.877E-01	9.950E-01
Left insula	0.134	0.142	[-0.144 - 0.412]	3.440E-01	9.950E-01
Right insula	0.156	0.157	[-0.151 - 0.462]	3.206E-01	9.950E-01
Left lingual gyrus	0.113	0.142	[-0.165 - 0.391]	4.247E-01	9.950E-01
Right lingual gyrus	-0.039	0.142	[-0.316 - 0.239]	7.850E-01	9.950E-01
Left pars triangularis of inferior frontal gyrus	-0.199	0.142	[-0.478 - 0.079]	1.600E-01	9.950E-01
Right pars triangularis of inferior frontal gyrus	0.152	0.142	[-0.127 - 0.430]	2.852E-01	9.950E-01
Left lateral orbitofrontal cortex	-0.019	0.159	[-0.330 - 0.292]	9.061E-01	9.950E-01
Right lateral orbitofrontal cortex	-0.131	0.142	[-0.408 - 0.147]	3.559E-01	9.950E-01
Left rostral middle frontal gyrus	-0.121	0.150	[-0.415 - 0.174]	4.219E-01	9.950E-01
Right rostral middle frontal gyrus	-0.042	0.151	[-0.339 - 0.254]	7.786E-01	9.950E-01
Left middle temporal gyrus	0.069	0.170	[-0.265 - 0.403]	6.855E-01	9.950E-01
Right middle temporal gyrus	-0.100	0.182	[-0.456 - 0.257]	5.834E-01	9.950E-01
Left superior frontal gyrus	0.026	0.174	[-0.315 - 0.367]	8.820E-01	9.950E-01
Right superior frontal gyrus	-0.106	0.203	[-0.504 - 0.291]	6.000E-01	9.950E-01
Left pars orbitalis of inferior frontal gyrus	0.063	0.149	[-0.229 - 0.356]	6.714E-01	9.950E-01
Right pars orbitalis of inferior frontal gyrus	0.047	0.142	[-0.230 - 0.325]	7.387E-01	9.950E-01
Left medial orbitofrontal cortex	-0.095	0.178	[-0.443 - 0.253]	5.912E-01	9.950E-01
Right medial orbitofrontal cortex	-0.148	0.142	[-0.425 - 0.130]	2.968E-01	9.950E-01
Left inferior temporal gyrus	-0.051	0.142	[-0.329 - 0.226]	7.170E-01	9.950E-01
Right inferior temporal gyrus	-0.002	0.142	[-0.280 - 0.275]	9.879E-01	9.950E-01
Left isthmus cingulate cortex	-0.172	0.182	[-0.529 - 0.185]	3.458E-01	9.950E-01
Right isthmus cingulate cortex	0.045	0.166	[-0.280 - 0.370]	7.852E-01	9.950E-01
Left banks of superior temporal sulcus	0.005	0.142	[-0.272 - 0.283]	9.706E-01	9.950E-01
Right banks of superior temporal sulcus	-0.015	0.142	[-0.293 - 0.262]	9.145E-01	9.950E-01
Left supramarginal gyrus	-0.156	0.248	[-0.642 - 0.331]	5.306E-01	9.950E-01
Right supramarginal gyrus	-0.230	0.326	[-0.869 - 0.409]	4.810E-01	9.950E-01
Left caudal middle frontal gyrus	-0.013	0.223	[-0.450 - 0.425]	9.551E-01	9.950E-01
Right caudal middle frontal gyrus	0.232	0.142	[-0.046 - 0.510]	1.025E-01	9.950E-01
Left frontal pole	-0.135	0.305	[-0.733 - 0.463]	6.583E-01	9.950E-01
Right frontal pole	-0.140	0.156	[-0.446 - 0.166]	3.709E-01	9.950E-01
Left posterior cingulate cortex	0.115	0.180	[-0.237 - 0.468]	5.207E-01	9.950E-01
Right posterior cingulate cortex	0.088	0.151	[-0.208 - 0.384]	5.598E-01	9.950E-01
Left lateral occipital cortex	0.231	0.282	[-0.321 - 0.784]	4.113E-01	9.950E-01
Right lateral occipital cortex	-0.012	0.142	[-0.290 - 0.265]	9.305E-01	9.950E-01
Left precentral gyrus	-0.007	0.207	[-0.413 - 0.399]	9.739E-01	9.950E-01
Right precentral gyrus	-0.074	0.166	[-0.399 - 0.251]	6.538E-01	9.950E-01
Left parahippocampal gyrus	0.287	0.256	[-0.214 - 0.788]	2.609E-01	9.950E-01
Right parahippocampal gyrus	0.169	0.157	[-0.139 - 0.477]	2.814E-01	9.950E-01
Left inferior parietal cortex	0.009	0.202	[-0.386 - 0.404]	9.638E-01	9.950E-01
Right inferior parietal cortex	-0.047	0.262	[-0.560 - 0.466]	8.578E-01	9.950E-01
Left transverse temporal gyrus	-0.091	0.142	[-0.369 - 0.186]	5.192E-01	9.950E-01
Right transverse temporal gyrus	-0.139	0.258	[-0.645 - 0.366]	5.884E-01	9.950E-01
Left postcentral gyrus	0.040	0.178	[-0.309 - 0.390]	8.210E-01	9.950E-01
Right postcentral gyrus	0.042	0.182	[-0.314 - 0.399]	8.158E-01	9.950E-01
Left precuneus	0.077	0.227	[-0.367 - 0.522]	7.337E-01	9.950E-01
Right precuneus	0.211	0.216	[-0.214 - 0.635]	3.303E-01	9.950E-01
Left caudal anterior cingulate cortex	0.052	0.142	[-0.225 - 0.329]	7.133E-01	9.950E-01
Right caudal anterior cingulate cortex	-0.085	0.142	[-0.363 - 0.192]	5.474E-01	9.950E-01
Left cuneus	0.137	0.142	[-0.141 - 0.416]	3.331E-01	9.950E-01
Right cuneus	0.009	0.142	[-0.269 - 0.286]	9.511E-01	9.950E-01
Left rostral anterior cingulate cortex	0.187	0.142	[-0.091 - 0.466]	1.875E-01	9.950E-01
Right rostral anterior cingulate cortex	0.021	0.141	[-0.256 - 0.298]	8.829E-01	9.950E-01
Left pericalcarine cortex	0.252	0.142	[-0.026 - 0.531]	7.606E-02	9.950E-01
Right pericalcarine cortex	-0.030	0.193	[-0.408 - 0.347]	8.757E-01	9.950E-01
Left paracentral lobule	-0.026	0.264	[-0.544 - 0.491]	9.207E-01	9.950E-01
Right paracentral lobule	0.336	0.261	[-0.176 - 0.848]	1.987E-01	9.950E-01
Left superior parietal cortex	0.085	0.326	[-0.553 - 0.723]	7.941E-01	9.950E-01
Right superior parietal cortex	0.106	0.274	[-0.431 - 0.643]	6.978E-01	9.950E-01
Left temporal pole	0.159	0.204	[-0.241 - 0.558]	4.373E-01	9.950E-01
Right temporal pole	-0.121	0.142	[-0.398 - 0.157]	3.950E-01	9.950E-01
Left entorhinal cortex	-0.344	0.251	[-0.835 - 0.147]	1.692E-01	9.950E-01
Right entorhinal cortex	-0.547	0.250	[-1.037 - -0.056]	2.884E-02	9.950E-01

Supplementary Table S138. Cortical thickness differences between individuals with major depressive disorder not taking antipsychotics and healthy comparison subjects controlling for age and sex

	d	Std. Err.	95% CI	*p*-value	FDR *q*-value
Global mean cortical thickness	-0.344	0.129	[-0.597 - -0.090]	7.931E-03	3.312E-02
Left hemisphere	-0.365	0.132	[-0.624 - -0.106]	5.702E-03	3.312E-02
Right hemisphere	-0.301	0.123	[-0.541 - -0.061]	1.410E-02	4.264E-02
Left fusiform gyrus	-0.245	0.101	[-0.443 - -0.046]	1.571E-02	4.461E-02
Right fusiform gyrus	-0.192	0.101	[-0.391 - 0.007]	5.838E-02	1.256E-01
Left pars opercularis of inferior frontal gyrus	-0.254	0.080	[-0.411 - -0.097]	1.536E-03	1.396E-02
Right pars opercularis of inferior frontal gyrus	-0.152	0.129	[-0.404 - 0.100]	2.372E-01	2.955E-01
Left superior temporal gyrus	-0.306	0.077	[-0.457 - -0.154]	7.664E-05	5.442E-03
Right superior temporal gyrus	-0.176	0.086	[-0.345 - -0.007]	4.074E-02	9.642E-02
Left insula	-0.233	0.095	[-0.419 - -0.046]	1.441E-02	4.264E-02
Right insula	-0.201	0.111	[-0.418 - 0.017]	7.056E-02	1.354E-01
Left lingual gyrus	-0.199	0.112	[-0.419 - 0.020]	7.511E-02	1.366E-01
Right lingual gyrus	-0.166	0.124	[-0.409 - 0.076]	1.789E-01	2.442E-01
Left pars triangularis of inferior frontal gyrus	-0.161	0.074	[-0.307 - -0.016]	2.937E-02	7.446E-02
Right pars triangularis of inferior frontal gyrus	-0.182	0.106	[-0.389 - 0.025]	8.427E-02	1.392E-01
Left lateral orbitofrontal cortex	-0.268	0.100	[-0.464 - -0.071]	7.639E-03	3.312E-02
Right lateral orbitofrontal cortex	-0.181	0.103	[-0.382 - 0.020]	7.743E-02	1.366E-01
Left rostral middle frontal gyrus	-0.304	0.099	[-0.498 - -0.109]	2.208E-03	1.568E-02
Right rostral middle frontal gyrus	-0.192	0.105	[-0.397 - 0.013]	6.663E-02	1.352E-01
Left middle temporal gyrus	-0.378	0.106	[-0.586 - -0.171]	3.504E-04	1.244E-02
Right middle temporal gyrus	-0.285	0.107	[-0.496 - -0.075]	7.751E-03	3.312E-02
Left superior frontal gyrus	-0.256	0.102	[-0.455 - -0.057]	1.173E-02	3.964E-02
Right superior frontal gyrus	-0.238	0.075	[-0.385 - -0.091]	1.551E-03	1.396E-02
Left pars orbitalis of inferior frontal gyrus	-0.287	0.090	[-0.464 - -0.110]	1.446E-03	1.396E-02
Right pars orbitalis of inferior frontal gyrus	-0.254	0.097	[-0.445 - -0.064]	8.931E-03	3.523E-02
Left medial orbitofrontal cortex	-0.256	0.129	[-0.510 - -0.003]	4.724E-02	1.082E-01
Right medial orbitofrontal cortex	-0.201	0.075	[-0.349 - -0.053]	7.646E-03	3.312E-02
Left inferior temporal gyrus	-0.206	0.096	[-0.393 - -0.018]	3.131E-02	7.665E-02
Right inferior temporal gyrus	-0.201	0.075	[-0.347 - -0.054]	7.273E-03	3.312E-02
Left isthmus cingulate cortex	-0.108	0.078	[-0.260 - 0.045]	1.665E-01	2.322E-01
Right isthmus cingulate cortex	-0.100	0.072	[-0.242 - 0.042]	1.668E-01	2.322E-01
Left banks of superior temporal sulcus	-0.267	0.084	[-0.433 - -0.102]	1.539E-03	1.396E-02
Right banks of superior temporal sulcus	-0.159	0.092	[-0.339 - 0.021]	8.304E-02	1.392E-01
Left supramarginal gyrus	-0.232	0.090	[-0.408 - -0.056]	9.891E-03	3.696E-02
Right supramarginal gyrus	-0.250	0.101	[-0.447 - -0.053]	1.297E-02	4.187E-02
Left caudal middle frontal gyrus	-0.178	0.108	[-0.390 - 0.034]	1.002E-01	1.581E-01
Right caudal middle frontal gyrus	-0.282	0.097	[-0.472 - -0.093]	3.463E-03	2.236E-02
Left frontal pole	-0.150	0.067	[-0.282 - -0.019]	2.541E-02	6.681E-02
Right frontal pole	-0.143	0.103	[-0.345 - 0.059]	1.646E-01	2.322E-01
Left posterior cingulate cortex	-0.210	0.067	[-0.342 - -0.078]	1.770E-03	1.396E-02
Right posterior cingulate cortex	-0.156	0.083	[-0.319 - 0.007]	6.107E-02	1.275E-01
Left lateral occipital cortex	-0.179	0.102	[-0.379 - 0.021]	7.889E-02	1.366E-01
Right lateral occipital cortex	-0.146	0.113	[-0.368 - 0.076]	1.985E-01	2.563E-01
Left precentral gyrus	-0.330	0.105	[-0.535 - -0.125]	1.603E-03	1.396E-02
Right precentral gyrus	-0.272	0.085	[-0.438 - -0.106]	1.308E-03	1.396E-02
Left parahippocampal gyrus	-0.093	0.101	[-0.291 - 0.105]	3.589E-01	4.110E-01
Right parahippocampal gyrus	-0.018	0.071	[-0.157 - 0.122]	8.018E-01	8.251E-01
Left inferior parietal cortex	-0.226	0.098	[-0.418 - -0.034]	2.121E-02	5.792E-02
Right inferior parietal cortex	-0.168	0.098	[-0.361 - 0.024]	8.692E-02	1.403E-01
Left transverse temporal gyrus	-0.108	0.101	[-0.305 - 0.089]	2.838E-01	3.303E-01
Right transverse temporal gyrus	-0.062	0.108	[-0.273 - 0.149]	5.654E-01	5.991E-01
Left postcentral gyrus	-0.205	0.113	[-0.426 - 0.016]	6.876E-02	1.354E-01
Right postcentral gyrus	-0.195	0.102	[-0.396 - 0.005]	5.645E-02	1.253E-01
Left precuneus	-0.123	0.094	[-0.308 - 0.061]	1.898E-01	2.495E-01
Right precuneus	-0.172	0.067	[-0.304 - -0.040]	1.049E-02	3.724E-02
Left caudal anterior cingulate cortex	-0.041	0.067	[-0.172 - 0.091]	5.442E-01	5.975E-01
Right caudal anterior cingulate cortex	-0.118	0.078	[-0.271 - 0.035]	1.293E-01	1.954E-01
Left cuneus	-0.105	0.075	[-0.252 - 0.043]	1.643E-01	2.322E-01
Right cuneus	-0.075	0.067	[-0.206 - 0.057]	2.655E-01	3.142E-01
Left rostral anterior cingulate cortex	-0.093	0.077	[-0.244 - 0.059]	2.314E-01	2.934E-01
Right rostral anterior cingulate cortex	-0.019	0.081	[-0.178 - 0.140]	8.153E-01	8.270E-01
Left pericalcarine cortex	-0.035	0.080	[-0.192 - 0.121]	6.569E-01	6.858E-01
Right pericalcarine cortex	-0.052	0.087	[-0.223 - 0.119]	5.496E-01	5.975E-01
Left paracentral lobule	-0.163	0.104	[-0.367 - 0.041]	1.168E-01	1.803E-01
Right paracentral lobule	-0.176	0.100	[-0.372 - 0.019]	7.751E-02	1.366E-01
Left superior parietal cortex	-0.154	0.117	[-0.384 - 0.076]	1.897E-01	2.495E-01
Right superior parietal cortex	-0.119	0.104	[-0.323 - 0.085]	2.535E-01	3.051E-01
Left temporal pole	-0.082	0.072	[-0.222 - 0.059]	2.533E-01	3.051E-01
Right temporal pole	0.065	0.094	[-0.119 - 0.250]	4.867E-01	5.486E-01
Left entorhinal cortex	-0.071	0.121	[-0.307 - 0.165]	5.554E-01	5.975E-01
Right entorhinal cortex	-0.011	0.103	[-0.212 - 0.190]	9.140E-01	9.140E-01

Supplementary Table S139. Cortical thickness differences between individuals with major depressive disorder not taking antipsychotics and healthy comparison subjects controlling for age and sex over 21 years of old

	d	Std. Err.	95% CI	*p*-value	FDR *q*-value
Global mean cortical thickness	-0.307	0.130	[-0.563 - -0.052]	1.821E-02	6.496E-02
Left hemisphere	-0.325	0.132	[-0.584 - -0.065]	1.419E-02	6.496E-02
Right hemisphere	-0.274	0.125	[-0.519 - -0.030]	2.806E-02	7.968E-02
Left fusiform gyrus	-0.211	0.095	[-0.397 - -0.026]	2.544E-02	7.661E-02
Right fusiform gyrus	-0.171	0.105	[-0.377 - 0.035]	1.034E-01	1.892E-01
Left pars opercularis of inferior frontal gyrus	-0.260	0.084	[-0.425 - -0.095]	2.059E-03	2.838E-02
Right pars opercularis of inferior frontal gyrus	-0.155	0.131	[-0.413 - 0.102]	2.372E-01	3.239E-01
Left superior temporal gyrus	-0.293	0.082	[-0.454 - -0.132]	3.604E-04	1.853E-02
Right superior temporal gyrus	-0.171	0.093	[-0.353 - 0.011]	6.591E-02	1.462E-01
Left insula	-0.210	0.090	[-0.387 - -0.032]	2.039E-02	6.581E-02
Right insula	-0.179	0.107	[-0.388 - 0.029]	9.219E-02	1.769E-01
Left lingual gyrus	-0.136	0.107	[-0.344 - 0.073]	2.031E-01	2.883E-01
Right lingual gyrus	-0.095	0.116	[-0.321 - 0.132]	4.134E-01	4.974E-01
Left pars triangularis of inferior frontal gyrus	-0.168	0.078	[-0.322 - -0.015]	3.160E-02	8.308E-02
Right pars triangularis of inferior frontal gyrus	-0.210	0.114	[-0.433 - 0.013]	6.479E-02	1.462E-01
Left lateral orbitofrontal cortex	-0.265	0.111	[-0.484 - -0.047]	1.705E-02	6.496E-02
Right lateral orbitofrontal cortex	-0.203	0.109	[-0.417 - 0.011]	6.244E-02	1.462E-01
Left rostral middle frontal gyrus	-0.331	0.115	[-0.557 - -0.105]	4.062E-03	2.884E-02
Right rostral middle frontal gyrus	-0.184	0.115	[-0.409 - 0.040]	1.077E-01	1.902E-01
Left middle temporal gyrus	-0.408	0.118	[-0.639 - -0.178]	5.220E-04	1.853E-02
Right middle temporal gyrus	-0.289	0.115	[-0.515 - -0.063]	1.218E-02	6.496E-02
Left superior frontal gyrus	-0.249	0.112	[-0.468 - -0.030]	2.590E-02	7.661E-02
Right superior frontal gyrus	-0.244	0.086	[-0.414 - -0.075]	4.714E-03	3.043E-02
Left pars orbitalis of inferior frontal gyrus	-0.286	0.098	[-0.478 - -0.095]	3.391E-03	2.838E-02
Right pars orbitalis of inferior frontal gyrus	-0.279	0.106	[-0.486 - -0.072]	8.232E-03	4.870E-02
Left medial orbitofrontal cortex	-0.212	0.130	[-0.468 - 0.044]	1.039E-01	1.892E-01
Right medial orbitofrontal cortex	-0.199	0.084	[-0.364 - -0.034]	1.812E-02	6.496E-02
Left inferior temporal gyrus	-0.166	0.086	[-0.334 - 0.002]	5.330E-02	1.305E-01
Right inferior temporal gyrus	-0.189	0.079	[-0.344 - -0.033]	1.743E-02	6.496E-02
Left isthmus cingulate cortex	-0.097	0.074	[-0.242 - 0.047]	1.857E-01	2.746E-01
Right isthmus cingulate cortex	-0.109	0.085	[-0.275 - 0.057]	1.979E-01	2.867E-01
Left banks of superior temporal sulcus	-0.286	0.096	[-0.474 - -0.098]	2.917E-03	2.838E-02
Right banks of superior temporal sulcus	-0.142	0.082	[-0.303 - 0.018]	8.181E-02	1.707E-01
Left supramarginal gyrus	-0.239	0.099	[-0.432 - -0.046]	1.544E-02	6.496E-02
Right supramarginal gyrus	-0.222	0.094	[-0.406 - -0.038]	1.830E-02	6.496E-02
Left caudal middle frontal gyrus	-0.171	0.114	[-0.395 - 0.053]	1.345E-01	2.093E-01
Right caudal middle frontal gyrus	-0.273	0.087	[-0.443 - -0.104]	1.572E-03	2.838E-02
Left frontal pole	-0.168	0.077	[-0.320 - -0.016]	3.000E-02	8.193E-02
Right frontal pole	-0.167	0.110	[-0.383 - 0.049]	1.293E-01	2.093E-01
Left posterior cingulate cortex	-0.201	0.069	[-0.337 - -0.066]	3.598E-03	2.838E-02
Right posterior cingulate cortex	-0.143	0.084	[-0.307 - 0.022]	8.927E-02	1.761E-01
Left lateral occipital cortex	-0.136	0.091	[-0.314 - 0.042]	1.356E-01	2.093E-01
Right lateral occipital cortex	-0.107	0.113	[-0.329 - 0.114]	3.425E-01	4.347E-01
Left precentral gyrus	-0.285	0.095	[-0.472 - -0.098]	2.807E-03	2.838E-02
Right precentral gyrus	-0.262	0.088	[-0.434 - -0.091]	2.743E-03	2.838E-02
Left parahippocampal gyrus	-0.058	0.091	[-0.235 - 0.120]	5.243E-01	6.005E-01
Right parahippocampal gyrus	0.006	0.069	[-0.130 - 0.141]	9.351E-01	9.622E-01
Left inferior parietal cortex	-0.205	0.099	[-0.400 - -0.011]	3.806E-02	9.651E-02
Right inferior parietal cortex	-0.181	0.102	[-0.380 - 0.019]	7.574E-02	1.630E-01
Left transverse temporal gyrus	-0.079	0.101	[-0.277 - 0.119]	4.355E-01	5.154E-01
Right transverse temporal gyrus	-0.038	0.113	[-0.259 - 0.183]	7.348E-01	8.026E-01
Left postcentral gyrus	-0.169	0.106	[-0.376 - 0.038]	1.098E-01	1.902E-01
Right postcentral gyrus	-0.176	0.102	[-0.377 - 0.024]	8.415E-02	1.707E-01
Left precuneus	-0.109	0.097	[-0.300 - 0.081]	2.597E-01	3.479E-01
Right precuneus	-0.161	0.069	[-0.296 - -0.025]	1.987E-02	6.581E-02
Left caudal anterior cingulate cortex	-0.040	0.069	[-0.176 - 0.095]	5.605E-01	6.218E-01
Right caudal anterior cingulate cortex	-0.122	0.082	[-0.282 - 0.038]	1.350E-01	2.093E-01
Left cuneus	-0.065	0.070	[-0.202 - 0.072]	3.517E-01	4.347E-01
Right cuneus	-0.052	0.069	[-0.187 - 0.084]	4.546E-01	5.291E-01
Left rostral anterior cingulate cortex	-0.115	0.078	[-0.268 - 0.037]	1.387E-01	2.095E-01
Right rostral anterior cingulate cortex	-0.020	0.083	[-0.182 - 0.143]	8.131E-01	8.616E-01
Left pericalcarine cortex	-0.004	0.069	[-0.140 - 0.131]	9.509E-01	9.644E-01
Right pericalcarine cortex	-0.026	0.091	[-0.204 - 0.153]	7.795E-01	8.385E-01
Left paracentral lobule	-0.124	0.102	[-0.325 - 0.076]	2.239E-01	3.117E-01
Right paracentral lobule	-0.155	0.104	[-0.359 - 0.048]	1.336E-01	2.093E-01
Left superior parietal cortex	-0.097	0.103	[-0.298 - 0.104]	3.456E-01	4.347E-01
Right superior parietal cortex	-0.094	0.101	[-0.292 - 0.105]	3.551E-01	4.347E-01
Left temporal pole	-0.080	0.081	[-0.239 - 0.079]	3.263E-01	4.290E-01
Right temporal pole	0.061	0.103	[-0.141 - 0.262]	5.555E-01	6.218E-01
Left entorhinal cortex	-0.019	0.121	[-0.257 - 0.219]	8.767E-01	9.154E-01
Right entorhinal cortex	0.002	0.109	[-0.211 - 0.215]	9.861E-01	9.861E-01

Supplementary Table S140. Cortical thickness differences between individuals with major depressive disorder taking second-generation antipsychotics and healthy comparison subjects controlling for age and sex

	d	Std. Err.	95% CI	*p*-value	FDR *q*-value
Global mean cortical thickness	-0.563	0.238	[-1.029 - -0.097]	1.789E-02	4.886E-02
Left hemisphere	-0.606	0.232	[-1.061 - -0.152]	8.901E-03	3.717E-02
Right hemisphere	-0.506	0.240	[-0.977 - -0.035]	3.540E-02	8.667E-02
Left fusiform gyrus	-0.820	0.146	[-1.105 - -0.534]	1.814E-08	9.709E-07
Right fusiform gyrus	-0.587	0.186	[-0.952 - -0.221]	1.655E-03	1.068E-02
Left pars opercularis of inferior frontal gyrus	-0.491	0.143	[-0.772 - -0.211]	5.987E-04	6.073E-03
Right pars opercularis of inferior frontal gyrus	-0.296	0.142	[-0.574 - -0.018]	3.716E-02	8.795E-02
Left superior temporal gyrus	-0.511	0.213	[-0.928 - -0.094]	1.641E-02	4.839E-02
Right superior temporal gyrus	-0.503	0.144	[-0.784 - -0.221]	4.730E-04	5.597E-03
Left insula	-0.433	0.222	[-0.869 - 0.002]	5.109E-02	9.620E-02
Right insula	-0.354	0.218	[-0.781 - 0.073]	1.046E-01	1.603E-01
Left lingual gyrus	-0.310	0.197	[-0.697 - 0.077]	1.163E-01	1.721E-01
Right lingual gyrus	-0.429	0.143	[-0.710 - -0.149]	2.681E-03	1.464E-02
Left pars triangularis of inferior frontal gyrus	-0.354	0.142	[-0.632 - -0.075]	1.296E-02	4.601E-02
Right pars triangularis of inferior frontal gyrus	-0.409	0.170	[-0.743 - -0.076]	1.620E-02	4.839E-02
Left lateral orbitofrontal cortex	-0.812	0.146	[-1.098 - -0.525]	2.735E-08	9.709E-07
Right lateral orbitofrontal cortex	-0.537	0.222	[-0.972 - -0.103]	1.537E-02	4.839E-02
Left rostral middle frontal gyrus	-0.661	0.209	[-1.071 - -0.250]	1.592E-03	1.068E-02
Right rostral middle frontal gyrus	-0.575	0.241	[-1.047 - -0.103]	1.704E-02	4.839E-02
Left middle temporal gyrus	-0.409	0.176	[-0.753 - -0.065]	1.969E-02	5.178E-02
Right middle temporal gyrus	-0.493	0.242	[-0.967 - -0.020]	4.123E-02	9.147E-02
Left superior frontal gyrus	-0.481	0.270	[-1.011 - 0.049]	7.539E-02	1.287E-01
Right superior frontal gyrus	-0.410	0.265	[-0.929 - 0.109]	1.219E-01	1.766E-01
Left pars orbitalis of inferior frontal gyrus	-0.667	0.187	[-1.034 - -0.301]	3.632E-04	5.157E-03
Right pars orbitalis of inferior frontal gyrus	-0.613	0.193	[-0.993 - -0.234]	1.521E-03	1.068E-02
Left medial orbitofrontal cortex	-0.589	0.231	[-1.042 - -0.135]	1.100E-02	4.228E-02
Right medial orbitofrontal cortex	-0.751	0.227	[-1.195 - -0.306]	9.306E-04	8.259E-03
Left inferior temporal gyrus	-0.595	0.155	[-0.900 - -0.290]	1.288E-04	2.287E-03
Right inferior temporal gyrus	-0.553	0.144	[-0.835 - -0.272]	1.177E-04	2.287E-03
Left isthmus cingulate cortex	-0.429	0.143	[-0.708 - -0.149]	2.639E-03	1.464E-02
Right isthmus cingulate cortex	-0.391	0.154	[-0.694 - -0.088]	1.132E-02	4.228E-02
Left banks of superior temporal sulcus	-0.225	0.194	[-0.606 - 0.156]	2.464E-01	3.019E-01
Right banks of superior temporal sulcus	-0.418	0.215	[-0.838 - 0.003]	5.149E-02	9.620E-02
Left supramarginal gyrus	-0.420	0.251	[-0.913 - 0.072]	9.463E-02	1.500E-01
Right supramarginal gyrus	-0.277	0.243	[-0.753 - 0.199]	2.542E-01	3.058E-01
Left caudal middle frontal gyrus	-0.455	0.164	[-0.776 - -0.133]	5.626E-03	2.663E-02
Right caudal middle frontal gyrus	-0.272	0.266	[-0.794 - 0.250]	3.079E-01	3.584E-01
Left frontal pole	-0.423	0.176	[-0.769 - -0.078]	1.641E-02	4.839E-02
Right frontal pole	-0.502	0.249	[-0.990 - -0.014]	4.378E-02	9.420E-02
Left posterior cingulate cortex	-0.435	0.221	[-0.868 - -0.003]	4.851E-02	9.567E-02
Right posterior cingulate cortex	-0.210	0.266	[-0.731 - 0.311]	4.299E-01	4.769E-01
Left lateral occipital cortex	-0.362	0.273	[-0.898 - 0.173]	1.851E-01	2.479E-01
Right lateral occipital cortex	-0.460	0.232	[-0.915 - -0.005]	4.747E-02	9.567E-02
Left precentral gyrus	-0.403	0.227	[-0.848 - 0.042]	7.614E-02	1.287E-01
Right precentral gyrus	-0.227	0.204	[-0.627 - 0.172]	2.639E-01	3.123E-01
Left parahippocampal gyrus	-0.375	0.142	[-0.654 - -0.096]	8.518E-03	3.717E-02
Right parahippocampal gyrus	-0.292	0.142	[-0.570 - -0.014]	3.987E-02	9.131E-02
Left inferior parietal cortex	-0.225	0.191	[-0.598 - 0.149]	2.380E-01	3.019E-01
Right inferior parietal cortex	-0.136	0.241	[-0.609 - 0.336]	5.717E-01	6.058E-01
Left transverse temporal gyrus	-0.418	0.143	[-0.699 - -0.138]	3.413E-03	1.731E-02
Right transverse temporal gyrus	-0.290	0.154	[-0.592 - 0.011]	5.879E-02	1.070E-01
Left postcentral gyrus	-0.328	0.245	[-0.807 - 0.152]	1.807E-01	2.467E-01
Right postcentral gyrus	-0.475	0.238	[-0.942 - -0.008]	4.611E-02	9.567E-02
Left precuneus	-0.290	0.174	[-0.630 - 0.050]	9.508E-02	1.500E-01
Right precuneus	-0.302	0.165	[-0.625 - 0.021]	6.690E-02	1.188E-01
Left caudal anterior cingulate cortex	-0.206	0.141	[-0.484 - 0.071]	1.445E-01	2.012E-01
Right caudal anterior cingulate cortex	-0.003	0.199	[-0.393 - 0.387]	9.891E-01	9.891E-01
Left cuneus	-0.129	0.202	[-0.525 - 0.267]	5.246E-01	5.644E-01
Right cuneus	-0.198	0.170	[-0.531 - 0.135]	2.434E-01	3.019E-01
Left rostral anterior cingulate cortex	-0.412	0.192	[-0.788 - -0.036]	3.159E-02	8.009E-02
Right rostral anterior cingulate cortex	-0.264	0.221	[-0.697 - 0.168]	2.306E-01	3.019E-01
Left pericalcarine cortex	0.013	0.202	[-0.382 - 0.408]	9.472E-01	9.608E-01
Right pericalcarine cortex	0.256	0.335	[-0.400 - 0.912]	4.451E-01	4.862E-01
Left paracentral lobule	-0.218	0.142	[-0.497 - 0.061]	1.249E-01	1.774E-01
Right paracentral lobule	-0.103	0.241	[-0.576 - 0.370]	6.706E-01	7.001E-01
Left superior parietal cortex	-0.222	0.192	[-0.598 - 0.154]	2.466E-01	3.019E-01
Right superior parietal cortex	-0.115	0.142	[-0.393 - 0.162]	4.159E-01	4.688E-01
Left temporal pole	-0.277	0.161	[-0.592 - 0.038]	8.450E-02	1.395E-01
Right temporal pole	-0.072	0.183	[-0.430 - 0.287]	6.952E-01	7.153E-01
Left entorhinal cortex	-0.236	0.146	[-0.522 - 0.050]	1.061E-01	1.603E-01
Right entorhinal cortex	-0.236	0.251	[-0.727 - 0.255]	3.467E-01	3.970E-01

Supplementary Table S141. Cortical thickness differences between individuals with major depressive disorder taking second-generation antipsychotics and healthy comparison subjects controlling for age and sex over 21 years of old

	d	Std. Err.	95% CI	*p*-value	FDR *q*-value
Global mean cortical thickness	-0.623	0.251	[-1.115 - -0.132]	1.299E-02	3.521E-02
Left hemisphere	-0.672	0.245	[-1.152 - -0.192]	6.076E-03	2.270E-02
Right hemisphere	-0.560	0.253	[-1.056 - -0.064]	2.684E-02	5.445E-02
Left fusiform gyrus	-0.877	0.149	[-1.169 - -0.584]	4.215E-09	2.993E-07
Right fusiform gyrus	-0.637	0.191	[-1.011 - -0.263]	8.417E-04	7.338E-03
Left pars opercularis of inferior frontal gyrus	-0.589	0.147	[-0.877 - -0.301]	6.197E-05	8.799E-04
Right pars opercularis of inferior frontal gyrus	-0.370	0.145	[-0.655 - -0.085]	1.097E-02	3.287E-02
Left superior temporal gyrus	-0.571	0.225	[-1.012 - -0.130]	1.111E-02	3.287E-02
Right superior temporal gyrus	-0.553	0.158	[-0.863 - -0.243]	4.775E-04	5.650E-03
Left insula	-0.460	0.234	[-0.919 - 0.000]	4.978E-02	9.301E-02
Right insula	-0.351	0.233	[-0.807 - 0.106]	1.321E-01	1.752E-01
Left lingual gyrus	-0.301	0.203	[-0.699 - 0.097]	1.384E-01	1.755E-01
Right lingual gyrus	-0.424	0.146	[-0.711 - -0.137]	3.776E-03	1.676E-02
Left pars triangularis of inferior frontal gyrus	-0.372	0.146	[-0.657 - -0.086]	1.070E-02	3.287E-02
Right pars triangularis of inferior frontal gyrus	-0.401	0.220	[-0.831 - 0.030]	6.803E-02	1.098E-01
Left lateral orbitofrontal cortex	-0.861	0.158	[-1.170 - -0.552]	4.924E-08	1.748E-06
Right lateral orbitofrontal cortex	-0.576	0.226	[-1.018 - -0.134]	1.071E-02	3.287E-02
Left rostral middle frontal gyrus	-0.729	0.223	[-1.165 - -0.292]	1.075E-03	7.636E-03
Right rostral middle frontal gyrus	-0.615	0.268	[-1.140 - -0.089]	2.180E-02	4.837E-02
Left middle temporal gyrus	-0.435	0.181	[-0.790 - -0.080]	1.628E-02	3.986E-02
Right middle temporal gyrus	-0.556	0.230	[-1.008 - -0.105]	1.568E-02	3.975E-02
Left superior frontal gyrus	-0.533	0.283	[-1.088 - 0.023]	6.016E-02	1.042E-01
Right superior frontal gyrus	-0.444	0.277	[-0.988 - 0.100]	1.097E-01	1.558E-01
Left pars orbitalis of inferior frontal gyrus	-0.666	0.193	[-1.045 - -0.288]	5.625E-04	5.706E-03
Right pars orbitalis of inferior frontal gyrus	-0.649	0.223	[-1.086 - -0.212]	3.580E-03	1.676E-02
Left medial orbitofrontal cortex	-0.630	0.237	[-1.094 - -0.166]	7.830E-03	2.780E-02
Right medial orbitofrontal cortex	-0.769	0.240	[-1.240 - -0.298]	1.383E-03	8.180E-03
Left inferior temporal gyrus	-0.630	0.150	[-0.923 - -0.337]	2.530E-05	5.987E-04
Right inferior temporal gyrus	-0.605	0.147	[-0.893 - -0.316]	3.974E-05	7.053E-04
Left isthmus cingulate cortex	-0.442	0.146	[-0.728 - -0.157]	2.401E-03	1.218E-02
Right isthmus cingulate cortex	-0.465	0.209	[-0.875 - -0.055]	2.631E-02	5.445E-02
Left banks of superior temporal sulcus	-0.264	0.192	[-0.640 - 0.112]	1.694E-01	2.110E-01
Right banks of superior temporal sulcus	-0.480	0.194	[-0.860 - -0.100]	1.339E-02	3.521E-02
Left supramarginal gyrus	-0.483	0.264	[-0.999 - 0.034]	6.719E-02	1.098E-01
Right supramarginal gyrus	-0.370	0.214	[-0.790 - 0.049]	8.352E-02	1.235E-01
Left caudal middle frontal gyrus	-0.543	0.164	[-0.865 - -0.222]	9.302E-04	7.338E-03
Right caudal middle frontal gyrus	-0.345	0.278	[-0.890 - 0.199]	2.140E-01	2.620E-01
Left frontal pole	-0.484	0.158	[-0.793 - -0.174]	2.196E-03	1.200E-02
Right frontal pole	-0.486	0.273	[-1.021 - 0.050]	7.553E-02	1.192E-01
Left posterior cingulate cortex	-0.476	0.207	[-0.881 - -0.071]	2.111E-02	4.836E-02
Right posterior cingulate cortex	-0.276	0.238	[-0.743 - 0.191]	2.472E-01	2.975E-01
Left lateral occipital cortex	-0.413	0.275	[-0.953 - 0.127]	1.339E-01	1.752E-01
Right lateral occipital cortex	-0.472	0.237	[-0.936 - -0.008]	4.635E-02	8.894E-02
Left precentral gyrus	-0.446	0.232	[-0.900 - 0.008]	5.441E-02	9.892E-02
Right precentral gyrus	-0.221	0.207	[-0.627 - 0.185]	2.852E-01	3.319E-01
Left parahippocampal gyrus	-0.417	0.146	[-0.703 - -0.132]	4.175E-03	1.744E-02
Right parahippocampal gyrus	-0.342	0.145	[-0.626 - -0.057]	1.859E-02	4.400E-02
Left inferior parietal cortex	-0.277	0.182	[-0.634 - 0.079]	1.273E-01	1.739E-01
Right inferior parietal cortex	-0.215	0.232	[-0.669 - 0.239]	3.528E-01	3.976E-01
Left transverse temporal gyrus	-0.469	0.146	[-0.755 - -0.182]	1.351E-03	8.180E-03
Right transverse temporal gyrus	-0.282	0.153	[-0.581 - 0.017]	6.462E-02	1.092E-01
Left postcentral gyrus	-0.401	0.269	[-0.928 - 0.126]	1.357E-01	1.752E-01
Right postcentral gyrus	-0.574	0.205	[-0.976 - -0.172]	5.153E-03	2.033E-02
Left precuneus	-0.356	0.163	[-0.675 - -0.037]	2.872E-02	5.664E-02
Right precuneus	-0.316	0.182	[-0.672 - 0.041]	8.245E-02	1.235E-01
Left caudal anterior cingulate cortex	-0.254	0.145	[-0.537 - 0.030]	7.926E-02	1.223E-01
Right caudal anterior cingulate cortex	-0.105	0.184	[-0.466 - 0.256]	5.687E-01	5.852E-01
Left cuneus	-0.142	0.220	[-0.574 - 0.290]	5.182E-01	5.492E-01
Right cuneus	-0.157	0.202	[-0.552 - 0.239]	4.379E-01	4.710E-01
Left rostral anterior cingulate cortex	-0.483	0.214	[-0.902 - -0.065]	2.361E-02	5.079E-02
Right rostral anterior cingulate cortex	-0.369	0.193	[-0.746 - 0.009]	5.573E-02	9.892E-02
Left pericalcarine cortex	-0.013	0.212	[-0.429 - 0.402]	9.494E-01	9.494E-01
Right pericalcarine cortex	0.257	0.326	[-0.381 - 0.895]	4.300E-01	4.697E-01
Left paracentral lobule	-0.230	0.145	[-0.515 - 0.055]	1.135E-01	1.581E-01
Right paracentral lobule	-0.110	0.243	[-0.586 - 0.365]	6.499E-01	6.591E-01
Left superior parietal cortex	-0.229	0.208	[-0.637 - 0.179]	2.716E-01	3.214E-01
Right superior parietal cortex	-0.131	0.145	[-0.415 - 0.153]	3.652E-01	4.051E-01
Left temporal pole	-0.364	0.146	[-0.650 - -0.078]	1.256E-02	3.521E-02
Right temporal pole	-0.115	0.182	[-0.472 - 0.242]	5.272E-01	5.504E-01
Left entorhinal cortex	-0.241	0.149	[-0.532 - 0.051]	1.058E-01	1.533E-01
Right entorhinal cortex	-0.267	0.258	[-0.773 - 0.239]	3.015E-01	3.453E-01

Supplementary Table S142. Cortical thickness differences between individuals with major depressive disorder taking second-generation antipsychotics and individuals with major depressive disorder not taking antipsychotics controlling for age and sex

	d	Std. Err.	95% CI	*p*-value	FDR *q*-value
Global mean cortical thickness	-0.275	0.259	[-0.782 - 0.232]	2.870E-01	6.524E-01
Left hemisphere	-0.282	0.253	[-0.779 - 0.215]	2.657E-01	6.425E-01
Right hemisphere	-0.260	0.257	[-0.764 - 0.245]	3.128E-01	6.533E-01
Left fusiform gyrus	-0.546	0.165	[-0.868 - -0.223]	9.308E-04	6.609E-02
Right fusiform gyrus	-0.393	0.271	[-0.925 - 0.139]	1.475E-01	4.986E-01
Left pars opercularis of inferior frontal gyrus	-0.238	0.164	[-0.560 - 0.083]	1.457E-01	4.986E-01
Right pars opercularis of inferior frontal gyrus	-0.042	0.235	[-0.503 - 0.419]	8.593E-01	9.293E-01
Left superior temporal gyrus	-0.228	0.196	[-0.613 - 0.157]	2.449E-01	6.425E-01
Right superior temporal gyrus	-0.297	0.177	[-0.643 - 0.049]	9.280E-02	4.986E-01
Left insula	-0.141	0.191	[-0.515 - 0.232]	4.589E-01	7.717E-01
Right insula	-0.194	0.132	[-0.452 - 0.064]	1.406E-01	4.986E-01
Left lingual gyrus	-0.057	0.222	[-0.493 - 0.379]	7.964E-01	9.200E-01
Right lingual gyrus	-0.195	0.131	[-0.452 - 0.063]	1.387E-01	4.986E-01
Left pars triangularis of inferior frontal gyrus	-0.208	0.132	[-0.466 - 0.050]	1.141E-01	4.986E-01
Right pars triangularis of inferior frontal gyrus	-0.086	0.183	[-0.445 - 0.274]	6.405E-01	8.421E-01
Left lateral orbitofrontal cortex	-0.300	0.198	[-0.689 - 0.088]	1.297E-01	4.986E-01
Right lateral orbitofrontal cortex	-0.186	0.299	[-0.771 - 0.400]	5.344E-01	7.717E-01
Left rostral middle frontal gyrus	-0.270	0.132	[-0.529 - -0.012]	4.036E-02	4.986E-01
Right rostral middle frontal gyrus	-0.299	0.199	[-0.689 - 0.090]	1.321E-01	4.986E-01
Left middle temporal gyrus	0.015	0.257	[-0.488 - 0.519]	9.521E-01	9.710E-01
Right middle temporal gyrus	-0.219	0.159	[-0.531 - 0.094]	1.699E-01	5.244E-01
Left superior frontal gyrus	-0.055	0.320	[-0.681 - 0.572]	8.638E-01	9.293E-01
Right superior frontal gyrus	-0.076	0.253	[-0.572 - 0.420]	7.650E-01	9.052E-01
Left pars orbitalis of inferior frontal gyrus	-0.212	0.132	[-0.470 - 0.046]	1.079E-01	4.986E-01
Right pars orbitalis of inferior frontal gyrus	-0.155	0.262	[-0.669 - 0.359]	5.543E-01	7.717E-01
Left medial orbitofrontal cortex	-0.231	0.157	[-0.540 - 0.078]	1.425E-01	4.986E-01
Right medial orbitofrontal cortex	-0.360	0.176	[-0.704 - -0.015]	4.068E-02	4.986E-01
Left inferior temporal gyrus	-0.414	0.215	[-0.835 - 0.007]	5.422E-02	4.986E-01
Right inferior temporal gyrus	-0.395	0.198	[-0.784 - -0.006]	4.660E-02	4.986E-01
Left isthmus cingulate cortex	-0.172	0.132	[-0.430 - 0.086]	1.906E-01	5.638E-01
Right isthmus cingulate cortex	-0.079	0.131	[-0.336 - 0.179]	5.491E-01	7.717E-01
Left banks of superior temporal sulcus	0.085	0.232	[-0.369 - 0.539]	7.145E-01	9.012E-01
Right banks of superior temporal sulcus	-0.242	0.132	[-0.500 - 0.016]	6.595E-02	4.986E-01
Left supramarginal gyrus	-0.166	0.257	[-0.670 - 0.339]	5.197E-01	7.717E-01
Right supramarginal gyrus	-0.067	0.209	[-0.476 - 0.342]	7.477E-01	9.052E-01
Left caudal middle frontal gyrus	-0.303	0.195	[-0.685 - 0.079]	1.203E-01	4.986E-01
Right caudal middle frontal gyrus	0.014	0.271	[-0.516 - 0.545]	9.573E-01	9.710E-01
Left frontal pole	-0.128	0.132	[-0.386 - 0.130]	3.312E-01	6.533E-01
Right frontal pole	-0.095	0.131	[-0.352 - 0.163]	4.717E-01	7.717E-01
Left posterior cingulate cortex	-0.238	0.232	[-0.692 - 0.215]	3.032E-01	6.524E-01
Right posterior cingulate cortex	0.000	0.280	[-0.549 - 0.549]	9.997E-01	9.997E-01
Left lateral occipital cortex	-0.263	0.236	[-0.726 - 0.200]	2.659E-01	6.425E-01
Right lateral occipital cortex	-0.387	0.215	[-0.809 - 0.034]	7.182E-02	4.986E-01
Left precentral gyrus	-0.161	0.198	[-0.549 - 0.226]	4.146E-01	7.359E-01
Right precentral gyrus	0.117	0.191	[-0.258 - 0.492]	5.415E-01	7.717E-01
Left parahippocampal gyrus	-0.089	0.131	[-0.346 - 0.169]	5.003E-01	7.717E-01
Right parahippocampal gyrus	-0.227	0.188	[-0.595 - 0.142]	2.280E-01	6.227E-01
Left inferior parietal cortex	-0.060	0.193	[-0.439 - 0.319]	7.572E-01	9.052E-01
Right inferior parietal cortex	-0.062	0.131	[-0.320 - 0.195]	6.351E-01	8.421E-01
Left transverse temporal gyrus	-0.180	0.131	[-0.438 - 0.077]	1.697E-01	5.244E-01
Right transverse temporal gyrus	-0.213	0.227	[-0.657 - 0.231]	3.473E-01	6.663E-01
Left postcentral gyrus	-0.232	0.186	[-0.596 - 0.132]	2.118E-01	6.015E-01
Right postcentral gyrus	-0.284	0.149	[-0.577 - 0.009]	5.704E-02	4.986E-01
Left precuneus	-0.145	0.173	[-0.484 - 0.195]	4.032E-01	7.341E-01
Right precuneus	-0.194	0.132	[-0.452 - 0.064]	1.408E-01	4.986E-01
Left caudal anterior cingulate cortex	-0.117	0.131	[-0.375 - 0.141]	3.733E-01	6.974E-01
Right caudal anterior cingulate cortex	0.052	0.210	[-0.359 - 0.463]	8.034E-01	9.200E-01
Left cuneus	0.036	0.198	[-0.353 - 0.424]	8.575E-01	9.293E-01
Right cuneus	-0.144	0.131	[-0.402 - 0.113]	2.715E-01	6.425E-01
Left rostral anterior cingulate cortex	-0.193	0.198	[-0.581 - 0.195]	3.302E-01	6.533E-01
Right rostral anterior cingulate cortex	-0.149	0.204	[-0.549 - 0.251]	4.655E-01	7.717E-01
Left pericalcarine cortex	0.026	0.224	[-0.413 - 0.465]	9.071E-01	9.612E-01
Right pericalcarine cortex	0.199	0.313	[-0.415 - 0.813]	5.257E-01	7.717E-01
Left paracentral lobule	-0.113	0.164	[-0.434 - 0.209]	4.921E-01	7.717E-01
Right paracentral lobule	-0.037	0.194	[-0.418 - 0.344]	8.477E-01	9.293E-01
Left superior parietal cortex	-0.198	0.132	[-0.456 - 0.060]	1.328E-01	4.986E-01
Right superior parietal cortex	-0.046	0.131	[-0.304 - 0.211]	7.235E-01	9.012E-01
Left temporal pole	-0.074	0.131	[-0.332 - 0.184]	5.735E-01	7.830E-01
Right temporal pole	-0.143	0.138	[-0.414 - 0.128]	3.023E-01	6.524E-01
Left entorhinal cortex	-0.016	0.229	[-0.464 - 0.432]	9.428E-01	9.710E-01
Right entorhinal cortex	-0.053	0.131	[-0.310 - 0.204]	6.867E-01	8.865E-01

Supplementary Table S143. Cortical thickness differences between individuals with major depressive disorder taking second-generation antipsychotics and individuals with major depressive disorder not taking antipsychotics controlling for age and sex over 21 years of old

	d	Std. Err.	95% CI	*p*-value	FDR *q*-value
Global mean cortical thickness	-0.331	0.280	[-0.880 - 0.218]	2.376E-01	5.366E-01
Left hemisphere	-0.340	0.270	[-0.870 - 0.189]	2.081E-01	4.924E-01
Right hemisphere	-0.311	0.284	[-0.868 - 0.245]	2.725E-01	5.547E-01
Left fusiform gyrus	-0.582	0.181	[-0.938 - -0.226]	1.342E-03	9.529E-02
Right fusiform gyrus	-0.444	0.295	[-1.023 - 0.135]	1.332E-01	4.607E-01
Left pars opercularis of inferior frontal gyrus	-0.311	0.207	[-0.716 - 0.095]	1.331E-01	4.607E-01
Right pars opercularis of inferior frontal gyrus	-0.063	0.241	[-0.535 - 0.409]	7.937E-01	8.945E-01
Left superior temporal gyrus	-0.320	0.215	[-0.743 - 0.102]	1.369E-01	4.607E-01
Right superior temporal gyrus	-0.396	0.228	[-0.843 - 0.052]	8.335E-02	4.607E-01
Left insula	-0.152	0.193	[-0.530 - 0.225]	4.292E-01	6.772E-01
Right insula	-0.194	0.135	[-0.459 - 0.071]	1.508E-01	4.607E-01
Left lingual gyrus	-0.064	0.220	[-0.495 - 0.367]	7.708E-01	8.826E-01
Right lingual gyrus	-0.214	0.135	[-0.479 - 0.051]	1.130E-01	4.607E-01
Left pars triangularis of inferior frontal gyrus	-0.212	0.135	[-0.476 - 0.053]	1.164E-01	4.607E-01
Right pars triangularis of inferior frontal gyrus	-0.080	0.215	[-0.501 - 0.341]	7.107E-01	8.553E-01
Left lateral orbitofrontal cortex	-0.326	0.200	[-0.719 - 0.067]	1.042E-01	4.607E-01
Right lateral orbitofrontal cortex	-0.195	0.301	[-0.784 - 0.394]	5.154E-01	7.272E-01
Left rostral middle frontal gyrus	-0.304	0.135	[-0.570 - -0.039]	2.482E-02	4.607E-01
Right rostral middle frontal gyrus	-0.323	0.230	[-0.773 - 0.128]	1.604E-01	4.607E-01
Left middle temporal gyrus	0.016	0.260	[-0.494 - 0.526]	9.513E-01	9.807E-01
Right middle temporal gyrus	-0.238	0.170	[-0.571 - 0.095]	1.617E-01	4.607E-01
Left superior frontal gyrus	-0.071	0.332	[-0.722 - 0.581]	8.316E-01	8.946E-01
Right superior frontal gyrus	-0.112	0.276	[-0.653 - 0.430]	6.864E-01	8.403E-01
Left pars orbitalis of inferior frontal gyrus	-0.227	0.135	[-0.492 - 0.038]	9.280E-02	4.607E-01
Right pars orbitalis of inferior frontal gyrus	-0.183	0.278	[-0.729 - 0.362]	5.095E-01	7.272E-01
Left medial orbitofrontal cortex	-0.228	0.166	[-0.553 - 0.097]	1.687E-01	4.607E-01
Right medial orbitofrontal cortex	-0.376	0.197	[-0.762 - 0.011]	5.676E-02	4.607E-01
Left inferior temporal gyrus	-0.441	0.219	[-0.871 - -0.011]	4.450E-02	4.607E-01
Right inferior temporal gyrus	-0.420	0.207	[-0.825 - -0.015]	4.204E-02	4.607E-01
Left isthmus cingulate cortex	-0.158	0.135	[-0.422 - 0.106]	2.418E-01	5.366E-01
Right isthmus cingulate cortex	-0.064	0.151	[-0.359 - 0.232]	6.729E-01	8.382E-01
Left banks of superior temporal sulcus	0.084	0.239	[-0.385 - 0.552]	7.265E-01	8.596E-01
Right banks of superior temporal sulcus	-0.242	0.135	[-0.507 - 0.023]	7.328E-02	4.607E-01
Left supramarginal gyrus	-0.190	0.265	[-0.709 - 0.329]	4.735E-01	7.153E-01
Right supramarginal gyrus	-0.126	0.193	[-0.506 - 0.253]	5.135E-01	7.272E-01
Left caudal middle frontal gyrus	-0.365	0.199	[-0.756 - 0.026]	6.707E-02	4.607E-01
Right caudal middle frontal gyrus	-0.073	0.302	[-0.664 - 0.518]	8.083E-01	8.946E-01
Left frontal pole	-0.123	0.135	[-0.388 - 0.141]	3.611E-01	5.826E-01
Right frontal pole	-0.057	0.135	[-0.322 - 0.207]	6.699E-01	8.382E-01
Left posterior cingulate cortex	-0.276	0.212	[-0.692 - 0.141]	1.943E-01	4.819E-01
Right posterior cingulate cortex	-0.076	0.251	[-0.568 - 0.416]	7.629E-01	8.826E-01
Left lateral occipital cortex	-0.338	0.244	[-0.817 - 0.141]	1.668E-01	4.607E-01
Right lateral occipital cortex	-0.416	0.234	[-0.874 - 0.041]	7.452E-02	4.607E-01
Left precentral gyrus	-0.216	0.204	[-0.617 - 0.184]	2.897E-01	5.559E-01
Right precentral gyrus	0.106	0.196	[-0.277 - 0.490]	5.862E-01	7.707E-01
Left parahippocampal gyrus	-0.134	0.135	[-0.398 - 0.130]	3.199E-01	5.717E-01
Right parahippocampal gyrus	-0.297	0.194	[-0.678 - 0.083]	1.257E-01	4.607E-01
Left inferior parietal cortex	-0.113	0.217	[-0.538 - 0.313]	6.045E-01	7.804E-01
Right inferior parietal cortex	-0.124	0.135	[-0.388 - 0.140]	3.575E-01	5.826E-01
Left transverse temporal gyrus	-0.213	0.135	[-0.477 - 0.052]	1.151E-01	4.607E-01
Right transverse temporal gyrus	-0.234	0.236	[-0.697 - 0.229]	3.221E-01	5.717E-01
Left postcentral gyrus	-0.268	0.205	[-0.669 - 0.133]	1.899E-01	4.819E-01
Right postcentral gyrus	-0.318	0.146	[-0.604 - -0.032]	2.913E-02	4.607E-01
Left precuneus	-0.196	0.179	[-0.547 - 0.155]	2.734E-01	5.547E-01
Right precuneus	-0.239	0.135	[-0.504 - 0.026]	7.695E-02	4.607E-01
Left caudal anterior cingulate cortex	-0.145	0.135	[-0.409 - 0.119]	2.826E-01	5.559E-01
Right caudal anterior cingulate cortex	-0.010	0.195	[-0.392 - 0.373]	9.603E-01	9.807E-01
Left cuneus	0.009	0.205	[-0.394 - 0.411]	9.664E-01	9.807E-01
Right cuneus	-0.138	0.135	[-0.403 - 0.126]	3.055E-01	5.709E-01
Left rostral anterior cingulate cortex	-0.195	0.207	[-0.601 - 0.211]	3.460E-01	5.826E-01
Right rostral anterior cingulate cortex	-0.234	0.203	[-0.632 - 0.165]	2.502E-01	5.384E-01
Left pericalcarine cortex	-0.001	0.222	[-0.436 - 0.433]	9.948E-01	9.948E-01
Right pericalcarine cortex	0.192	0.300	[-0.396 - 0.779]	5.224E-01	7.272E-01
Left paracentral lobule	-0.145	0.191	[-0.520 - 0.230]	4.481E-01	6.916E-01
Right paracentral lobule	-0.044	0.198	[-0.432 - 0.345]	8.255E-01	8.946E-01
Left superior parietal cortex	-0.236	0.135	[-0.501 - 0.029]	8.132E-02	4.607E-01
Right superior parietal cortex	-0.077	0.135	[-0.341 - 0.187]	5.679E-01	7.608E-01
Left temporal pole	-0.129	0.135	[-0.393 - 0.135]	3.386E-01	5.826E-01
Right temporal pole	-0.194	0.151	[-0.490 - 0.101]	1.968E-01	4.819E-01
Left entorhinal cortex	-0.010	0.236	[-0.472 - 0.452]	9.669E-01	9.807E-01
Right entorhinal cortex	-0.078	0.135	[-0.342 - 0.186]	5.610E-01	7.608E-01

Supplementary Table S144. Cortical thickness differences between individuals with major depressive disorder taking first-generation antipsychotics and healthy comparison subjects controlling for age and sex

	d	Std. Err.	95% CI	*p*-value	FDR *q*-value
Global mean cortical thickness	-0.510	0.708	[-1.896 - 0.877]	4.715E-01	7.122E-01
Left hemisphere	-0.528	0.712	[-1.923 - 0.867]	4.579E-01	7.068E-01
Right hemisphere	-0.482	0.684	[-1.823 - 0.860]	4.816E-01	7.124E-01
Left fusiform gyrus	-0.509	0.319	[-1.133 - 0.116]	1.105E-01	4.357E-01
Right fusiform gyrus	-0.700	0.265	[-1.219 - -0.182]	8.106E-03	6.395E-02
Left pars opercularis of inferior frontal gyrus	-0.438	0.527	[-1.471 - 0.595]	4.065E-01	6.559E-01
Right pars opercularis of inferior frontal gyrus	-0.311	0.408	[-1.111 - 0.490]	4.465E-01	7.045E-01
Left superior temporal gyrus	-0.755	0.260	[-1.264 - -0.246]	3.652E-03	4.321E-02
Right superior temporal gyrus	-0.748	0.406	[-1.544 - 0.049]	6.586E-02	3.118E-01
Left insula	-0.714	0.470	[-1.634 - 0.207]	1.287E-01	4.767E-01
Right insula	-0.906	0.260	[-1.416 - -0.395]	5.088E-04	1.491E-02
Left lingual gyrus	-0.719	0.257	[-1.223 - -0.215]	5.196E-03	5.270E-02
Right lingual gyrus	-0.589	0.302	[-1.181 - 0.004]	5.146E-02	3.044E-01
Left pars triangularis of inferior frontal gyrus	-0.407	0.473	[-1.333 - 0.520]	3.894E-01	6.438E-01
Right pars triangularis of inferior frontal gyrus	-0.898	0.263	[-1.412 - -0.383]	6.302E-04	1.491E-02
Left lateral orbitofrontal cortex	-0.747	0.559	[-1.842 - 0.348]	1.812E-01	5.046E-01
Right lateral orbitofrontal cortex	-0.444	0.517	[-1.457 - 0.569]	3.899E-01	6.438E-01
Left rostral middle frontal gyrus	-0.793	0.546	[-1.863 - 0.277]	1.461E-01	4.767E-01
Right rostral middle frontal gyrus	-0.772	0.755	[-2.251 - 0.707]	3.061E-01	6.203E-01
Left middle temporal gyrus	-0.424	0.467	[-1.340 - 0.493]	3.648E-01	6.438E-01
Right middle temporal gyrus	-0.643	0.742	[-2.096 - 0.810]	3.859E-01	6.438E-01
Left superior frontal gyrus	-0.594	0.908	[-2.374 - 1.187]	5.134E-01	7.147E-01
Right superior frontal gyrus	-0.340	0.808	[-1.923 - 1.243]	6.740E-01	8.395E-01
Left pars orbitalis of inferior frontal gyrus	-0.686	0.607	[-1.875 - 0.504]	2.587E-01	5.566E-01
Right pars orbitalis of inferior frontal gyrus	-0.602	0.368	[-1.324 - 0.119]	1.016E-01	4.244E-01
Left medial orbitofrontal cortex	-0.938	0.772	[-2.450 - 0.574]	2.242E-01	5.390E-01
Right medial orbitofrontal cortex	-0.748	0.297	[-1.330 - -0.165]	1.196E-02	7.721E-02
Left inferior temporal gyrus	-0.455	0.343	[-1.128 - 0.218]	1.848E-01	5.046E-01
Right inferior temporal gyrus	-0.472	0.256	[-0.973 - 0.029]	6.461E-02	3.118E-01
Left isthmus cingulate cortex	-0.356	0.253	[-0.852 - 0.140]	1.591E-01	4.767E-01
Right isthmus cingulate cortex	-0.557	0.572	[-1.678 - 0.564]	3.301E-01	6.334E-01
Left banks of superior temporal sulcus	0.059	0.800	[-1.509 - 1.627]	9.409E-01	9.840E-01
Right banks of superior temporal sulcus	-0.665	0.258	[-1.172 - -0.159]	1.002E-02	7.115E-02
Left supramarginal gyrus	0.159	0.543	[-0.905 - 1.224]	7.695E-01	8.740E-01
Right supramarginal gyrus	-0.301	0.254	[-0.799 - 0.196]	2.353E-01	5.390E-01
Left caudal middle frontal gyrus	-0.434	0.355	[-1.130 - 0.261]	2.212E-01	5.390E-01
Right caudal middle frontal gyrus	-0.197	0.607	[-1.386 - 0.992]	7.449E-01	8.740E-01
Left frontal pole	-0.812	0.261	[-1.324 - -0.300]	1.870E-03	2.656E-02
Right frontal pole	-0.752	0.629	[-1.984 - 0.481]	2.321E-01	5.390E-01
Left posterior cingulate cortex	-0.330	0.254	[-0.827 - 0.168]	1.937E-01	5.094E-01
Right posterior cingulate cortex	-0.005	0.252	[-0.499 - 0.489]	9.840E-01	9.840E-01
Left lateral occipital cortex	-0.208	0.493	[-1.174 - 0.758]	6.734E-01	8.395E-01
Right lateral occipital cortex	-0.188	0.673	[-1.506 - 1.130]	7.798E-01	8.740E-01
Left precentral gyrus	-0.253	0.372	[-0.982 - 0.476]	4.960E-01	7.147E-01
Right precentral gyrus	-0.470	0.325	[-1.107 - 0.167]	1.485E-01	4.767E-01
Left parahippocampal gyrus	-0.354	0.253	[-0.850 - 0.141]	1.611E-01	4.767E-01
Right parahippocampal gyrus	-0.358	0.254	[-0.855 - 0.139]	1.575E-01	4.767E-01
Left inferior parietal cortex	-0.301	0.299	[-0.887 - 0.285]	3.145E-01	6.203E-01
Right inferior parietal cortex	-0.014	0.521	[-1.035 - 1.006]	9.779E-01	9.840E-01
Left transverse temporal gyrus	-0.814	0.259	[-1.322 - -0.306]	1.673E-03	2.656E-02
Right transverse temporal gyrus	-0.916	0.262	[-1.429 - -0.403]	4.707E-04	1.491E-02
Left postcentral gyrus	-0.164	0.598	[-1.336 - 1.007]	7.832E-01	8.740E-01
Right postcentral gyrus	-0.280	0.543	[-1.344 - 0.785]	6.065E-01	7.975E-01
Left precuneus	-0.031	0.599	[-1.206 - 1.144]	9.591E-01	9.840E-01
Right precuneus	0.218	0.638	[-1.032 - 1.469]	7.321E-01	8.740E-01
Left caudal anterior cingulate cortex	-0.229	0.252	[-0.724 - 0.266]	3.647E-01	6.438E-01
Right caudal anterior cingulate cortex	-0.075	0.489	[-1.033 - 0.884]	8.783E-01	9.593E-01
Left cuneus	-0.165	0.253	[-0.661 - 0.330]	5.124E-01	7.147E-01
Right cuneus	-0.705	0.257	[-1.209 - -0.201]	6.123E-03	5.434E-02
Left rostral anterior cingulate cortex	-0.232	0.252	[-0.727 - 0.263]	3.581E-01	6.438E-01
Right rostral anterior cingulate cortex	-0.064	0.755	[-1.544 - 1.415]	9.321E-01	9.840E-01
Left pericalcarine cortex	-0.478	0.256	[-0.980 - 0.024]	6.213E-02	3.118E-01
Right pericalcarine cortex	-0.253	0.469	[-1.173 - 0.667]	5.894E-01	7.896E-01
Left paracentral lobule	-0.453	0.255	[-0.952 - 0.046]	7.530E-02	3.341E-01
Right paracentral lobule	-0.016	0.485	[-0.968 - 0.935]	9.730E-01	9.840E-01
Left superior parietal cortex	0.336	0.770	[-1.174 - 1.846]	6.624E-01	8.395E-01
Right superior parietal cortex	0.369	0.605	[-0.817 - 1.555]	5.419E-01	7.400E-01
Left temporal pole	-0.543	0.498	[-1.520 - 0.434]	2.764E-01	5.771E-01
Right temporal pole	-0.299	0.260	[-0.810 - 0.211]	2.505E-01	5.558E-01
Left entorhinal cortex	-0.186	0.690	[-1.538 - 1.166]	7.878E-01	8.740E-01
Right entorhinal cortex	-0.228	0.733	[-1.666 - 1.209]	7.554E-01	8.740E-01

Supplementary Table S145. Cortical thickness differences between individuals with major depressive disorder taking first-generation antipsychotics and healthy comparison subjects controlling for age and sex over 21 years of old

	d	Std. Err.	95% CI	*p*-value	FDR *q*-value
Global mean cortical thickness	-0.471	0.745	[-1.931 - 0.988]	5.269E-01	7.583E-01
Left hemisphere	-0.500	0.739	[-1.948 - 0.948]	4.982E-01	7.583E-01
Right hemisphere	-0.434	0.730	[-1.865 - 0.998]	5.526E-01	7.681E-01
Left fusiform gyrus	-0.495	0.331	[-1.144 - 0.154]	1.349E-01	5.162E-01
Right fusiform gyrus	-0.663	0.298	[-1.248 - -0.078]	2.636E-02	1.702E-01
Left pars opercularis of inferior frontal gyrus	-0.394	0.568	[-1.507 - 0.719]	4.878E-01	7.583E-01
Right pars opercularis of inferior frontal gyrus	-0.292	0.426	[-1.127 - 0.542]	4.925E-01	7.583E-01
Left superior temporal gyrus	-0.742	0.260	[-1.252 - -0.233]	4.310E-03	4.931E-02
Right superior temporal gyrus	-0.702	0.448	[-1.580 - 0.176]	1.172E-01	4.894E-01
Left insula	-0.707	0.477	[-1.641 - 0.227]	1.381E-01	5.162E-01
Right insula	-0.874	0.261	[-1.385 - -0.363]	8.063E-04	2.511E-02
Left lingual gyrus	-0.725	0.258	[-1.230 - -0.221]	4.862E-03	4.931E-02
Right lingual gyrus	-0.584	0.307	[-1.186 - 0.017]	5.701E-02	3.114E-01
Left pars triangularis of inferior frontal gyrus	-0.366	0.511	[-1.367 - 0.636]	4.741E-01	7.583E-01
Right pars triangularis of inferior frontal gyrus	-0.860	0.263	[-1.376 - -0.345]	1.061E-03	2.511E-02
Left lateral orbitofrontal cortex	-0.716	0.589	[-1.869 - 0.438]	2.241E-01	6.018E-01
Right lateral orbitofrontal cortex	-0.406	0.553	[-1.490 - 0.677]	4.622E-01	7.583E-01
Left rostral middle frontal gyrus	-0.764	0.574	[-1.888 - 0.360]	1.827E-01	5.688E-01
Right rostral middle frontal gyrus	-0.734	0.792	[-2.286 - 0.817]	3.536E-01	6.785E-01
Left middle temporal gyrus	-0.430	0.462	[-1.336 - 0.476]	3.523E-01	6.785E-01
Right middle temporal gyrus	-0.643	0.742	[-2.097 - 0.810]	3.858E-01	7.208E-01
Left superior frontal gyrus	-0.576	0.926	[-2.391 - 1.239]	5.340E-01	7.583E-01
Right superior frontal gyrus	-0.306	0.841	[-1.954 - 1.342]	7.160E-01	8.833E-01
Left pars orbitalis of inferior frontal gyrus	-0.651	0.640	[-1.906 - 0.605]	3.096E-01	6.785E-01
Right pars orbitalis of inferior frontal gyrus	-0.604	0.367	[-1.324 - 0.115]	9.951E-02	4.416E-01
Left medial orbitofrontal cortex	-0.931	0.779	[-2.458 - 0.596]	2.323E-01	6.018E-01
Right medial orbitofrontal cortex	-0.735	0.309	[-1.341 - -0.130]	1.732E-02	1.230E-01
Left inferior temporal gyrus	-0.457	0.342	[-1.128 - 0.214]	1.818E-01	5.688E-01
Right inferior temporal gyrus	-0.453	0.256	[-0.955 - 0.048]	7.657E-02	3.883E-01
Left isthmus cingulate cortex	-0.342	0.253	[-0.838 - 0.154]	1.768E-01	5.688E-01
Right isthmus cingulate cortex	-0.514	0.532	[-1.557 - 0.530]	3.348E-01	6.785E-01
Left banks of superior temporal sulcus	0.055	0.796	[-1.506 - 1.615]	9.453E-01	9.730E-01
Right banks of superior temporal sulcus	-0.647	0.259	[-1.154 - -0.140]	1.231E-02	9.711E-02
Left supramarginal gyrus	0.204	0.586	[-0.944 - 1.352]	7.275E-01	8.833E-01
Right supramarginal gyrus	-0.283	0.254	[-0.781 - 0.216]	2.664E-01	6.306E-01
Left caudal middle frontal gyrus	-0.386	0.399	[-1.167 - 0.396]	3.334E-01	6.785E-01
Right caudal middle frontal gyrus	-0.166	0.637	[-1.414 - 1.082]	7.941E-01	9.191E-01
Left frontal pole	-0.808	0.261	[-1.321 - -0.296]	1.995E-03	3.376E-02
Right frontal pole	-0.748	0.633	[-1.988 - 0.493]	2.373E-01	6.018E-01
Left posterior cingulate cortex	-0.303	0.254	[-0.801 - 0.195]	2.327E-01	6.018E-01
Right posterior cingulate cortex	0.011	0.252	[-0.484 - 0.505]	9.660E-01	9.730E-01
Left lateral occipital cortex	-0.193	0.507	[-1.187 - 0.800]	7.029E-01	8.833E-01
Right lateral occipital cortex	-0.166	0.694	[-1.527 - 1.195]	8.111E-01	9.191E-01
Left precentral gyrus	-0.220	0.402	[-1.008 - 0.568]	5.842E-01	7.681E-01
Right precentral gyrus	-0.418	0.372	[-1.147 - 0.311]	2.616E-01	6.306E-01
Left parahippocampal gyrus	-0.330	0.253	[-0.826 - 0.166]	1.923E-01	5.688E-01
Right parahippocampal gyrus	-0.333	0.254	[-0.831 - 0.164]	1.894E-01	5.688E-01
Left inferior parietal cortex	-0.263	0.333	[-0.916 - 0.389]	4.284E-01	7.419E-01
Right inferior parietal cortex	0.019	0.552	[-1.063 - 1.101]	9.730E-01	9.730E-01
Left transverse temporal gyrus	-0.788	0.259	[-1.296 - -0.280]	2.378E-03	3.376E-02
Right transverse temporal gyrus	-0.897	0.262	[-1.411 - -0.383]	6.221E-04	2.511E-02
Left postcentral gyrus	-0.132	0.629	[-1.365 - 1.101]	8.340E-01	9.253E-01
Right postcentral gyrus	-0.292	0.532	[-1.335 - 0.751]	5.832E-01	7.681E-01
Left precuneus	-0.045	0.587	[-1.195 - 1.106]	9.396E-01	9.730E-01
Right precuneus	0.216	0.637	[-1.032 - 1.464]	7.340E-01	8.833E-01
Left caudal anterior cingulate cortex	-0.211	0.253	[-0.706 - 0.284]	4.037E-01	7.349E-01
Right caudal anterior cingulate cortex	-0.029	0.532	[-1.072 - 1.014]	9.568E-01	9.730E-01
Left cuneus	-0.165	0.253	[-0.661 - 0.331]	5.143E-01	7.583E-01
Right cuneus	-0.698	0.258	[-1.203 - -0.193]	6.715E-03	5.960E-02
Left rostral anterior cingulate cortex	-0.237	0.253	[-0.732 - 0.259]	3.490E-01	6.785E-01
Right rostral anterior cingulate cortex	-0.048	0.771	[-1.559 - 1.462]	9.500E-01	9.730E-01
Left pericalcarine cortex	-0.491	0.256	[-0.994 - 0.012]	5.558E-02	3.114E-01
Right pericalcarine cortex	-0.258	0.466	[-1.171 - 0.655]	5.798E-01	7.681E-01
Left paracentral lobule	-0.421	0.255	[-0.921 - 0.078]	9.836E-02	4.416E-01
Right paracentral lobule	0.019	0.519	[-0.998 - 1.036]	9.707E-01	9.730E-01
Left superior parietal cortex	0.361	0.795	[-1.197 - 1.920]	6.495E-01	8.385E-01
Right superior parietal cortex	0.402	0.637	[-0.846 - 1.649]	5.281E-01	7.583E-01
Left temporal pole	-0.532	0.508	[-1.529 - 0.464]	2.950E-01	6.757E-01
Right temporal pole	-0.249	0.305	[-0.848 - 0.350]	4.150E-01	7.366E-01
Left entorhinal cortex	-0.166	0.710	[-1.556 - 1.225]	8.155E-01	9.191E-01
Right entorhinal cortex	-0.234	0.728	[-1.662 - 1.193]	7.477E-01	8.848E-01

Supplementary Table S146. Cortical thickness differences between individuals with major depressive disorder taking first-generation antipsychotics and individuals with major depressive disorder not taking antipsychotics controlling for age and sex

	d	Std. Err.	95% CI	*p*-value	FDR *q*-value
Global mean cortical thickness	0.151	0.407	[-0.648 - 0.949]	7.116E-01	9.990E-01
Left hemisphere	0.150	0.407	[-0.646 - 0.947]	7.115E-01	9.990E-01
Right hemisphere	0.143	0.401	[-0.643 - 0.930]	7.208E-01	9.990E-01
Left fusiform gyrus	0.038	0.208	[-0.369 - 0.446]	8.537E-01	9.990E-01
Right fusiform gyrus	0.058	0.208	[-0.350 - 0.466]	7.807E-01	9.990E-01
Left pars opercularis of inferior frontal gyrus	-0.041	0.461	[-0.945 - 0.862]	9.283E-01	9.990E-01
Right pars opercularis of inferior frontal gyrus	0.255	0.558	[-0.839 - 1.349]	6.482E-01	9.990E-01
Left superior temporal gyrus	-0.100	0.208	[-0.507 - 0.308]	6.314E-01	9.990E-01
Right superior temporal gyrus	-0.252	0.208	[-0.661 - 0.156]	2.261E-01	9.990E-01
Left insula	-0.243	0.208	[-0.651 - 0.166]	2.444E-01	9.990E-01
Right insula	-0.294	0.208	[-0.702 - 0.114]	1.579E-01	9.990E-01
Left lingual gyrus	-0.496	0.335	[-1.153 - 0.160]	1.386E-01	9.990E-01
Right lingual gyrus	0.066	0.264	[-0.450 - 0.583]	8.014E-01	9.990E-01
Left pars triangularis of inferior frontal gyrus	0.034	0.421	[-0.791 - 0.859]	9.358E-01	9.990E-01
Right pars triangularis of inferior frontal gyrus	-0.044	0.243	[-0.521 - 0.432]	8.549E-01	9.990E-01
Left lateral orbitofrontal cortex	0.315	0.549	[-0.762 - 1.392]	5.663E-01	9.990E-01
Right lateral orbitofrontal cortex	0.200	0.507	[-0.793 - 1.193]	6.927E-01	9.990E-01
Left rostral middle frontal gyrus	-0.105	0.366	[-0.822 - 0.612]	7.742E-01	9.990E-01
Right rostral middle frontal gyrus	-0.014	0.369	[-0.737 - 0.710]	9.705E-01	9.990E-01
Left middle temporal gyrus	0.255	0.401	[-0.530 - 1.041]	5.240E-01	9.990E-01
Right middle temporal gyrus	-0.253	0.297	[-0.836 - 0.329]	3.939E-01	9.990E-01
Left superior frontal gyrus	0.195	0.622	[-1.023 - 1.414]	7.533E-01	9.990E-01
Right superior frontal gyrus	0.320	0.537	[-0.734 - 1.373]	5.520E-01	9.990E-01
Left pars orbitalis of inferior frontal gyrus	0.295	0.446	[-0.579 - 1.169]	5.082E-01	9.990E-01
Right pars orbitalis of inferior frontal gyrus	0.018	0.285	[-0.541 - 0.577]	9.487E-01	9.990E-01
Left medial orbitofrontal cortex	-0.134	0.478	[-1.070 - 0.802]	7.797E-01	9.990E-01
Right medial orbitofrontal cortex	-0.211	0.306	[-0.811 - 0.389]	4.912E-01	9.990E-01
Left inferior temporal gyrus	0.023	0.230	[-0.428 - 0.474]	9.200E-01	9.990E-01
Right inferior temporal gyrus	0.103	0.211	[-0.311 - 0.518]	6.253E-01	9.990E-01
Left isthmus cingulate cortex	0.106	0.208	[-0.301 - 0.513]	6.094E-01	9.990E-01
Right isthmus cingulate cortex	-0.101	0.253	[-0.597 - 0.396]	6.914E-01	9.990E-01
Left banks of superior temporal sulcus	0.414	0.528	[-0.621 - 1.450]	4.327E-01	9.990E-01
Right banks of superior temporal sulcus	-0.399	0.243	[-0.875 - 0.077]	1.005E-01	9.990E-01
Left supramarginal gyrus	0.307	0.426	[-0.527 - 1.142]	4.704E-01	9.990E-01
Right supramarginal gyrus	0.101	0.352	[-0.590 - 0.792]	7.741E-01	9.990E-01
Left caudal middle frontal gyrus	0.005	0.316	[-0.614 - 0.625]	9.872E-01	9.990E-01
Right caudal middle frontal gyrus	0.173	0.414	[-0.640 - 0.985]	6.769E-01	9.990E-01
Left frontal pole	-0.158	0.208	[-0.565 - 0.250]	4.480E-01	9.990E-01
Right frontal pole	-0.098	0.233	[-0.555 - 0.358]	6.734E-01	9.990E-01
Left posterior cingulate cortex	-0.131	0.331	[-0.779 - 0.517]	6.913E-01	9.990E-01
Right posterior cingulate cortex	0.011	0.286	[-0.549 - 0.571]	9.683E-01	9.990E-01
Left lateral occipital cortex	-0.030	0.208	[-0.438 - 0.377]	8.843E-01	9.990E-01
Right lateral occipital cortex	0.153	0.394	[-0.620 - 0.925]	6.986E-01	9.990E-01
Left precentral gyrus	-0.056	0.221	[-0.489 - 0.377]	7.996E-01	9.990E-01
Right precentral gyrus	-0.002	0.208	[-0.410 - 0.405]	9.908E-01	9.990E-01
Left parahippocampal gyrus	0.066	0.208	[-0.342 - 0.473]	7.526E-01	9.990E-01
Right parahippocampal gyrus	-0.062	0.208	[-0.469 - 0.345]	7.649E-01	9.990E-01
Left inferior parietal cortex	0.122	0.208	[-0.286 - 0.530]	5.567E-01	9.990E-01
Right inferior parietal cortex	0.329	0.208	[-0.079 - 0.737]	1.144E-01	9.990E-01
Left transverse temporal gyrus	-0.072	0.406	[-0.868 - 0.724]	8.597E-01	9.990E-01
Right transverse temporal gyrus	-0.554	0.210	[-0.965 - -0.144]	8.161E-03	5.795E-01
Left postcentral gyrus	0.077	0.242	[-0.398 - 0.552]	7.500E-01	9.990E-01
Right postcentral gyrus	-0.026	0.384	[-0.780 - 0.727]	9.452E-01	9.990E-01
Left precuneus	0.310	0.248	[-0.176 - 0.795]	2.109E-01	9.990E-01
Right precuneus	0.101	0.275	[-0.438 - 0.640]	7.133E-01	9.990E-01
Left caudal anterior cingulate cortex	-0.173	0.208	[-0.580 - 0.234]	4.046E-01	9.990E-01
Right caudal anterior cingulate cortex	-0.038	0.332	[-0.688 - 0.611]	9.078E-01	9.990E-01
Left cuneus	0.000	0.208	[-0.407 - 0.407]	9.990E-01	9.990E-01
Right cuneus	-0.248	0.208	[-0.656 - 0.160]	2.341E-01	9.990E-01
Left rostral anterior cingulate cortex	0.106	0.208	[-0.301 - 0.514]	6.092E-01	9.990E-01
Right rostral anterior cingulate cortex	0.187	0.561	[-0.912 - 1.286]	7.387E-01	9.990E-01
Left pericalcarine cortex	-0.334	0.208	[-0.742 - 0.075]	1.092E-01	9.990E-01
Right pericalcarine cortex	-0.222	0.208	[-0.630 - 0.185]	2.851E-01	9.990E-01
Left paracentral lobule	-0.121	0.208	[-0.529 - 0.286]	5.595E-01	9.990E-01
Right paracentral lobule	0.069	0.387	[-0.690 - 0.828]	8.585E-01	9.990E-01
Left superior parietal cortex	0.294	0.307	[-0.307 - 0.895]	3.381E-01	9.990E-01
Right superior parietal cortex	0.285	0.283	[-0.270 - 0.839]	3.144E-01	9.990E-01
Left temporal pole	0.020	0.340	[-0.647 - 0.687]	9.536E-01	9.990E-01
Right temporal pole	-0.091	0.235	[-0.551 - 0.369]	6.976E-01	9.990E-01
Left entorhinal cortex	0.022	0.208	[-0.385 - 0.429]	9.158E-01	9.990E-01
Right entorhinal cortex	0.220	0.236	[-0.242 - 0.683]	3.505E-01	9.990E-01

Supplementary Table S147. Cortical thickness differences between individuals with major depressive disorder taking first-generation antipsychotics and individuals with major depressive disorder not taking antipsychotics controlling for age and sex over 21 years of old

	d	Std. Err.	95% CI	*p*-value	FDR *q*-value
Global mean cortical thickness	0.145	0.413	[-0.664 - 0.954]	7.260E-01	9.757E-01
Left hemisphere	0.144	0.413	[-0.664 - 0.953]	7.262E-01	9.757E-01
Right hemisphere	0.137	0.406	[-0.659 - 0.934]	7.352E-01	9.757E-01
Left fusiform gyrus	0.038	0.208	[-0.370 - 0.447]	8.539E-01	9.757E-01
Right fusiform gyrus	0.055	0.209	[-0.354 - 0.464]	7.915E-01	9.757E-01
Left pars opercularis of inferior frontal gyrus	-0.041	0.460	[-0.943 - 0.861]	9.291E-01	9.757E-01
Right pars opercularis of inferior frontal gyrus	0.265	0.551	[-0.815 - 1.344]	6.307E-01	9.757E-01
Left superior temporal gyrus	-0.108	0.209	[-0.516 - 0.301]	6.057E-01	9.757E-01
Right superior temporal gyrus	-0.257	0.209	[-0.667 - 0.153]	2.190E-01	9.757E-01
Left insula	-0.230	0.209	[-0.640 - 0.180]	2.715E-01	9.757E-01
Right insula	-0.299	0.209	[-0.708 - 0.111]	1.528E-01	9.757E-01
Left lingual gyrus	-0.501	0.331	[-1.150 - 0.148]	1.305E-01	9.757E-01
Right lingual gyrus	0.063	0.278	[-0.481 - 0.607]	8.210E-01	9.757E-01
Left pars triangularis of inferior frontal gyrus	0.034	0.421	[-0.792 - 0.860]	9.357E-01	9.757E-01
Right pars triangularis of inferior frontal gyrus	-0.044	0.238	[-0.510 - 0.422]	8.534E-01	9.757E-01
Left lateral orbitofrontal cortex	0.314	0.550	[-0.764 - 1.393]	5.678E-01	9.757E-01
Right lateral orbitofrontal cortex	0.201	0.506	[-0.792 - 1.193]	6.920E-01	9.757E-01
Left rostral middle frontal gyrus	-0.104	0.365	[-0.820 - 0.612]	7.755E-01	9.757E-01
Right rostral middle frontal gyrus	-0.014	0.369	[-0.737 - 0.710]	9.706E-01	9.757E-01
Left middle temporal gyrus	0.262	0.392	[-0.507 - 1.032]	5.037E-01	9.757E-01
Right middle temporal gyrus	-0.248	0.296	[-0.828 - 0.331]	4.013E-01	9.757E-01
Left superior frontal gyrus	0.193	0.624	[-1.030 - 1.416]	7.569E-01	9.757E-01
Right superior frontal gyrus	0.312	0.546	[-0.757 - 1.382]	5.670E-01	9.757E-01
Left pars orbitalis of inferior frontal gyrus	0.289	0.456	[-0.605 - 1.182]	5.270E-01	9.757E-01
Right pars orbitalis of inferior frontal gyrus	0.025	0.276	[-0.517 - 0.567]	9.290E-01	9.757E-01
Left medial orbitofrontal cortex	-0.134	0.478	[-1.072 - 0.804]	7.792E-01	9.757E-01
Right medial orbitofrontal cortex	-0.221	0.310	[-0.828 - 0.386]	4.759E-01	9.757E-01
Left inferior temporal gyrus	0.024	0.236	[-0.439 - 0.486]	9.198E-01	9.757E-01
Right inferior temporal gyrus	0.105	0.209	[-0.305 - 0.515]	6.148E-01	9.757E-01
Left isthmus cingulate cortex	0.101	0.208	[-0.307 - 0.510]	6.269E-01	9.757E-01
Right isthmus cingulate cortex	-0.100	0.262	[-0.614 - 0.413]	7.018E-01	9.757E-01
Left banks of superior temporal sulcus	0.426	0.518	[-0.590 - 1.441]	4.112E-01	9.757E-01
Right banks of superior temporal sulcus	-0.400	0.242	[-0.875 - 0.074]	9.818E-02	9.757E-01
Left supramarginal gyrus	0.311	0.425	[-0.522 - 1.145]	4.642E-01	9.757E-01
Right supramarginal gyrus	0.103	0.353	[-0.588 - 0.795]	7.695E-01	9.757E-01
Left caudal middle frontal gyrus	0.009	0.309	[-0.596 - 0.614]	9.757E-01	9.757E-01
Right caudal middle frontal gyrus	0.170	0.417	[-0.647 - 0.988]	6.831E-01	9.757E-01
Left frontal pole	-0.153	0.208	[-0.562 - 0.255]	4.617E-01	9.757E-01
Right frontal pole	-0.097	0.233	[-0.554 - 0.359]	6.764E-01	9.757E-01
Left posterior cingulate cortex	-0.130	0.331	[-0.779 - 0.520]	6.957E-01	9.757E-01
Right posterior cingulate cortex	0.009	0.284	[-0.547 - 0.565]	9.742E-01	9.757E-01
Left lateral occipital cortex	-0.065	0.209	[-0.474 - 0.344]	7.565E-01	9.757E-01
Right lateral occipital cortex	0.129	0.419	[-0.692 - 0.950]	7.585E-01	9.757E-01
Left precentral gyrus	-0.061	0.228	[-0.508 - 0.387]	7.902E-01	9.757E-01
Right precentral gyrus	-0.015	0.209	[-0.423 - 0.394]	9.437E-01	9.757E-01
Left parahippocampal gyrus	0.062	0.209	[-0.347 - 0.471]	7.660E-01	9.757E-01
Right parahippocampal gyrus	-0.075	0.208	[-0.483 - 0.333]	7.192E-01	9.757E-01
Left inferior parietal cortex	0.127	0.209	[-0.282 - 0.537]	5.418E-01	9.757E-01
Right inferior parietal cortex	0.314	0.209	[-0.095 - 0.724]	1.323E-01	9.757E-01
Left transverse temporal gyrus	-0.065	0.404	[-0.857 - 0.728]	8.730E-01	9.757E-01
Right transverse temporal gyrus	-0.585	0.210	[-0.997 - -0.172]	5.458E-03	3.875E-01
Left postcentral gyrus	0.073	0.248	[-0.412 - 0.558]	7.687E-01	9.757E-01
Right postcentral gyrus	-0.028	0.385	[-0.783 - 0.726]	9.411E-01	9.757E-01
Left precuneus	0.301	0.252	[-0.193 - 0.795]	2.319E-01	9.757E-01
Right precuneus	0.079	0.281	[-0.471 - 0.629]	7.783E-01	9.757E-01
Left caudal anterior cingulate cortex	-0.174	0.208	[-0.582 - 0.235]	4.050E-01	9.757E-01
Right caudal anterior cingulate cortex	-0.045	0.336	[-0.703 - 0.613]	8.938E-01	9.757E-01
Left cuneus	-0.021	0.208	[-0.429 - 0.387]	9.206E-01	9.757E-01
Right cuneus	-0.280	0.209	[-0.689 - 0.129]	1.798E-01	9.757E-01
Left rostral anterior cingulate cortex	0.135	0.208	[-0.274 - 0.544]	5.173E-01	9.757E-01
Right rostral anterior cingulate cortex	0.169	0.576	[-0.961 - 1.299]	7.693E-01	9.757E-01
Left pericalcarine cortex	-0.344	0.209	[-0.754 - 0.065]	9.947E-02	9.757E-01
Right pericalcarine cortex	-0.247	0.209	[-0.656 - 0.162]	2.369E-01	9.757E-01
Left paracentral lobule	-0.140	0.209	[-0.549 - 0.269]	5.021E-01	9.757E-01
Right paracentral lobule	0.063	0.388	[-0.697 - 0.823]	8.709E-01	9.757E-01
Left superior parietal cortex	0.291	0.309	[-0.314 - 0.896]	3.462E-01	9.757E-01
Right superior parietal cortex	0.272	0.287	[-0.290 - 0.835]	3.428E-01	9.757E-01
Left temporal pole	0.020	0.341	[-0.648 - 0.688]	9.534E-01	9.757E-01
Right temporal pole	-0.090	0.234	[-0.549 - 0.370]	7.023E-01	9.757E-01
Left entorhinal cortex	0.026	0.208	[-0.382 - 0.434]	9.005E-01	9.757E-01
Right entorhinal cortex	0.221	0.246	[-0.262 - 0.704]	3.702E-01	9.757E-01

Supplementary Table S148. Cortical surface area differences between individuals with schizophrenia who are unmedicated with antipsychotics and healthy comparison subjects controlling for age and sex

	d	Std. Err.	95% CI	*p*-value	FDR *q*-value
Total cortical surface area	-0.164	0.168	[-0.494 - 0.166]	3.301E-01	7.729E-01
Left hemisphere	-0.163	0.164	[-0.484 - 0.158]	3.190E-01	7.729E-01
Right hemisphere	-0.164	0.172	[-0.501 - 0.173]	3.401E-01	7.729E-01
Left superior frontal gyrus	-0.177	0.160	[-0.491 - 0.137]	2.693E-01	7.384E-01
Right superior frontal gyrus	-0.064	0.181	[-0.418 - 0.291]	7.248E-01	9.190E-01
Left pars orbitalis of inferior frontal gyrus	-0.239	0.119	[-0.472 - -0.006]	4.395E-02	6.241E-01
Right pars orbitalis of inferior frontal gyrus	-0.071	0.160	[-0.384 - 0.242]	6.557E-01	9.065E-01
Left precentral gyrus	-0.255	0.177	[-0.602 - 0.091]	1.483E-01	7.384E-01
Right precentral gyrus	-0.100	0.230	[-0.550 - 0.351]	6.639E-01	9.065E-01
Left lingual gyrus	-0.141	0.123	[-0.383 - 0.101]	2.527E-01	7.384E-01
Right lingual gyrus	-0.139	0.182	[-0.495 - 0.217]	4.444E-01	8.718E-01
Left superior temporal gyrus	-0.006	0.191	[-0.380 - 0.368]	9.737E-01	9.876E-01
Right superior temporal gyrus	-0.191	0.172	[-0.529 - 0.147]	2.684E-01	7.384E-01
Left fusiform gyrus	-0.190	0.119	[-0.423 - 0.043]	1.095E-01	7.384E-01
Right fusiform gyrus	-0.045	0.119	[-0.277 - 0.188]	7.071E-01	9.128E-01
Left inferior parietal cortex	-0.178	0.119	[-0.410 - 0.055]	1.347E-01	7.384E-01
Right inferior parietal cortex	-0.085	0.148	[-0.376 - 0.205]	5.649E-01	8.718E-01
Left lateral occipital cortex	0.126	0.210	[-0.285 - 0.537]	5.481E-01	8.718E-01
Right lateral occipital cortex	0.027	0.119	[-0.205 - 0.260]	8.180E-01	9.857E-01
Left rostral middle frontal gyrus	-0.054	0.174	[-0.394 - 0.286]	7.557E-01	9.413E-01
Right rostral middle frontal gyrus	-0.088	0.176	[-0.434 - 0.258]	6.175E-01	9.065E-01
Left precuneus	-0.180	0.136	[-0.446 - 0.087]	1.869E-01	7.384E-01
Right precuneus	-0.062	0.127	[-0.310 - 0.186]	6.264E-01	9.065E-01
Left inferior temporal gyrus	-0.427	0.119	[-0.660 - -0.194]	3.257E-04	2.313E-02
Right inferior temporal gyrus	-0.368	0.119	[-0.601 - -0.135]	1.981E-03	6.101E-02
Left lateral orbitofrontal cortex	-0.199	0.136	[-0.465 - 0.067]	1.420E-01	7.384E-01
Right lateral orbitofrontal cortex	-0.199	0.166	[-0.524 - 0.127]	2.314E-01	7.384E-01
Left middle temporal gyrus	-0.215	0.119	[-0.447 - 0.018]	7.043E-02	7.384E-01
Right middle temporal gyrus	-0.201	0.151	[-0.497 - 0.095]	1.822E-01	7.384E-01
Left postcentral gyrus	-0.122	0.135	[-0.388 - 0.143]	3.666E-01	7.887E-01
Right postcentral gyrus	-0.171	0.138	[-0.441 - 0.099]	2.142E-01	7.384E-01
Left medial orbitofrontal cortex	0.112	0.238	[-0.355 - 0.579]	6.387E-01	9.065E-01
Right medial orbitofrontal cortex	-0.294	0.179	[-0.644 - 0.056]	9.965E-02	7.384E-01
Left cuneus	-0.096	0.119	[-0.330 - 0.139]	4.236E-01	8.718E-01
Right cuneus	-0.064	0.119	[-0.297 - 0.168]	5.874E-01	8.873E-01
Left pars triangularis of inferior frontal gyrus	-0.016	0.148	[-0.306 - 0.274]	9.126E-01	9.876E-01
Right pars triangularis of inferior frontal gyrus	-0.040	0.178	[-0.389 - 0.309]	8.214E-01	9.857E-01
Left superior parietal cortex	-0.171	0.152	[-0.470 - 0.127]	2.606E-01	7.384E-01
Right superior parietal cortex	-0.154	0.134	[-0.415 - 0.108]	2.497E-01	7.384E-01
Left pars opercularis of inferior frontal gyrus	-0.070	0.119	[-0.302 - 0.163]	5.562E-01	8.718E-01
Right pars opercularis of inferior frontal gyrus	-0.023	0.119	[-0.255 - 0.209]	8.462E-01	9.857E-01
Left supramarginal gyrus	-0.007	0.152	[-0.304 - 0.291]	9.642E-01	9.876E-01
Right supramarginal gyrus	-0.162	0.147	[-0.450 - 0.126]	2.704E-01	7.384E-01
Left pericalcarine cortex	-0.079	0.124	[-0.322 - 0.164]	5.232E-01	8.718E-01
Right pericalcarine cortex	-0.077	0.119	[-0.310 - 0.155]	5.154E-01	8.718E-01
Left parahippocampal gyrus	0.000	0.152	[-0.298 - 0.298]	9.994E-01	9.994E-01
Right parahippocampal gyrus	-0.008	0.152	[-0.305 - 0.290]	9.603E-01	9.876E-01
Left caudal middle frontal gyrus	-0.074	0.119	[-0.306 - 0.159]	5.340E-01	8.718E-01
Right caudal middle frontal gyrus	-0.034	0.196	[-0.419 - 0.351]	8.608E-01	9.857E-01
Left transverse temporal gyrus	-0.164	0.137	[-0.433 - 0.105]	2.328E-01	7.384E-01
Right transverse temporal gyrus	0.084	0.142	[-0.194 - 0.362]	5.548E-01	8.718E-01
Left banks of superior temporal sulcus	-0.157	0.122	[-0.397 - 0.082]	1.977E-01	7.384E-01
Right banks of superior temporal sulcus	-0.251	0.159	[-0.562 - 0.061]	1.151E-01	7.384E-01
Left caudal anterior cingulate cortex	0.026	0.140	[-0.249 - 0.301]	8.516E-01	9.857E-01
Right caudal anterior cingulate cortex	-0.270	0.119	[-0.503 - -0.037]	2.305E-02	4.091E-01
Left rostral anterior cingulate cortex	-0.114	0.119	[-0.347 - 0.119]	3.375E-01	7.729E-01
Right rostral anterior cingulate cortex	-0.121	0.119	[-0.353 - 0.111]	3.076E-01	7.729E-01
Left posterior cingulate cortex	-0.074	0.119	[-0.307 - 0.159]	5.339E-01	8.718E-01
Right posterior cingulate cortex	-0.358	0.119	[-0.591 - -0.125]	2.578E-03	6.101E-02
Left frontal pole	-0.093	0.151	[-0.388 - 0.203]	5.388E-01	8.718E-01
Right frontal pole	0.140	0.119	[-0.092 - 0.373]	2.365E-01	7.384E-01
Left paracentral lobule	-0.054	0.130	[-0.309 - 0.202]	6.811E-01	9.124E-01
Right paracentral lobule	-0.191	0.136	[-0.458 - 0.076]	1.601E-01	7.384E-01
Left insula	-0.069	0.119	[-0.301 - 0.164]	5.627E-01	8.718E-01
Right insula	-0.133	0.142	[-0.411 - 0.145]	3.483E-01	7.729E-01
Left entorhinal cortex	-0.045	0.119	[-0.278 - 0.187]	7.036E-01	9.128E-01
Right entorhinal cortex	0.015	0.119	[-0.217 - 0.248]	8.968E-01	9.876E-01
Left temporal pole	-0.004	0.119	[-0.237 - 0.228]	9.704E-01	9.876E-01
Right temporal pole	-0.007	0.132	[-0.266 - 0.252]	9.580E-01	9.876E-01
Left isthmus cingulate cortex	0.005	0.119	[-0.227 - 0.238]	9.646E-01	9.876E-01
Right isthmus cingulate cortex	0.087	0.134	[-0.176 - 0.351]	5.157E-01	8.718E-01

Supplementary Table S149. Cortical surface area differences between individuals with schizophrenia on second-generation antipsychotic medications and healthy comparison subjects controlling for age and sex

	d	Std. Err.	95% CI	*p*-value	FDR *q*-value
Total cortical surface area	-0.382	0.064	[-0.507 - -0.258]	1.811E-09	1.607E-08
Left hemisphere	-0.378	0.064	[-0.502 - -0.253]	3.003E-09	2.369E-08
Right hemisphere	-0.384	0.063	[-0.508 - -0.260]	1.206E-09	1.224E-08
Left superior frontal gyrus	-0.365	0.060	[-0.482 - -0.247]	1.107E-09	1.224E-08
Right superior frontal gyrus	-0.348	0.048	[-0.441 - -0.254]	3.294E-13	2.338E-11
Left pars orbitalis of inferior frontal gyrus	-0.332	0.048	[-0.425 - -0.239]	3.395E-12	1.205E-10
Right pars orbitalis of inferior frontal gyrus	-0.327	0.048	[-0.420 - -0.233]	7.282E-12	1.641E-10
Left precentral gyrus	-0.261	0.048	[-0.355 - -0.168]	4.200E-08	1.988E-07
Right precentral gyrus	-0.237	0.059	[-0.353 - -0.121]	6.039E-05	1.021E-04
Left lingual gyrus	-0.325	0.048	[-0.419 - -0.231]	1.062E-11	1.641E-10
Right lingual gyrus	-0.324	0.048	[-0.417 - -0.230]	1.155E-11	1.641E-10
Left superior temporal gyrus	-0.206	0.051	[-0.305 - -0.106]	5.116E-05	9.082E-05
Right superior temporal gyrus	-0.272	0.073	[-0.415 - -0.129]	1.971E-04	2.978E-04
Left fusiform gyrus	-0.288	0.068	[-0.422 - -0.154]	2.521E-05	5.272E-05
Right fusiform gyrus	-0.300	0.066	[-0.429 - -0.171]	5.456E-06	1.614E-05
Left inferior parietal cortex	-0.232	0.051	[-0.333 - -0.132]	5.683E-06	1.614E-05
Right inferior parietal cortex	-0.282	0.066	[-0.410 - -0.153]	1.802E-05	4.127E-05
Left lateral occipital cortex	-0.233	0.062	[-0.354 - -0.112]	1.670E-04	2.578E-04
Right lateral occipital cortex	-0.293	0.056	[-0.403 - -0.184]	1.384E-07	6.143E-07
Left rostral middle frontal gyrus	-0.294	0.057	[-0.406 - -0.181]	3.002E-07	1.254E-06
Right rostral middle frontal gyrus	-0.220	0.066	[-0.350 - -0.091]	8.219E-04	1.144E-03
Left precuneus	-0.301	0.051	[-0.401 - -0.201]	3.921E-09	2.784E-08
Right precuneus	-0.238	0.048	[-0.332 - -0.145]	5.819E-07	2.295E-06
Left inferior temporal gyrus	-0.280	0.062	[-0.403 - -0.158]	6.959E-06	1.830E-05
Right inferior temporal gyrus	-0.226	0.058	[-0.340 - -0.112]	1.038E-04	1.694E-04
Left lateral orbitofrontal cortex	-0.285	0.050	[-0.383 - -0.187]	1.227E-08	7.257E-08
Right lateral orbitofrontal cortex	-0.228	0.048	[-0.321 - -0.135]	1.664E-06	5.626E-06
Left middle temporal gyrus	-0.193	0.048	[-0.287 - -0.100]	4.795E-05	8.730E-05
Right middle temporal gyrus	-0.254	0.060	[-0.371 - -0.136]	2.393E-05	5.272E-05
Left postcentral gyrus	-0.246	0.059	[-0.362 - -0.130]	3.346E-05	6.421E-05
Right postcentral gyrus	-0.217	0.057	[-0.327 - -0.106]	1.290E-04	2.036E-04
Left medial orbitofrontal cortex	-0.165	0.048	[-0.258 - -0.072]	5.287E-04	7.508E-04
Right medial orbitofrontal cortex	-0.231	0.073	[-0.374 - -0.089]	1.472E-03	1.936E-03
Left cuneus	-0.241	0.053	[-0.345 - -0.137]	5.664E-06	1.614E-05
Right cuneus	-0.268	0.048	[-0.361 - -0.174]	1.928E-08	1.053E-07
Left pars triangularis of inferior frontal gyrus	-0.206	0.050	[-0.304 - -0.108]	3.705E-05	6.922E-05
Right pars triangularis of inferior frontal gyrus	-0.265	0.048	[-0.358 - -0.171]	2.741E-08	1.390E-07
Left superior parietal cortex	-0.235	0.048	[-0.329 - -0.142]	7.607E-07	2.843E-06
Right superior parietal cortex	-0.247	0.056	[-0.357 - -0.137]	1.083E-05	2.651E-05
Left pars opercularis of inferior frontal gyrus	-0.209	0.065	[-0.337 - -0.082]	1.259E-03	1.687E-03
Right pars opercularis of inferior frontal gyrus	-0.224	0.076	[-0.373 - -0.075]	3.130E-03	3.968E-03
Left supramarginal gyrus	-0.217	0.048	[-0.311 - -0.124]	5.039E-06	1.614E-05
Right supramarginal gyrus	-0.210	0.048	[-0.303 - -0.116]	1.047E-05	2.651E-05
Left pericalcarine cortex	-0.223	0.050	[-0.320 - -0.126]	6.663E-06	1.819E-05
Right pericalcarine cortex	-0.279	0.048	[-0.372 - -0.185]	4.863E-09	3.139E-08
Left parahippocampal gyrus	-0.162	0.062	[-0.283 - -0.042]	8.359E-03	9.572E-03
Right parahippocampal gyrus	-0.236	0.056	[-0.346 - -0.126]	2.525E-05	5.272E-05
Left caudal middle frontal gyrus	-0.205	0.070	[-0.341 - -0.069]	3.196E-03	3.981E-03
Right caudal middle frontal gyrus	-0.182	0.066	[-0.310 - -0.053]	5.634E-03	6.667E-03
Left transverse temporal gyrus	-0.171	0.048	[-0.265 - -0.076]	3.961E-04	5.739E-04
Right transverse temporal gyrus	-0.199	0.048	[-0.293 - -0.106]	2.794E-05	5.668E-05
Left banks of superior temporal sulcus	-0.173	0.048	[-0.266 - -0.080]	2.733E-04	4.043E-04
Right banks of superior temporal sulcus	-0.208	0.074	[-0.354 - -0.062]	5.107E-03	6.145E-03
Left caudal anterior cingulate cortex	-0.192	0.048	[-0.285 - -0.099]	5.279E-05	9.142E-05
Right caudal anterior cingulate cortex	-0.135	0.048	[-0.228 - -0.042]	4.533E-03	5.549E-03
Left rostral anterior cingulate cortex	-0.223	0.051	[-0.323 - -0.123]	1.241E-05	2.938E-05
Right rostral anterior cingulate cortex	-0.185	0.048	[-0.278 - -0.091]	1.050E-04	1.694E-04
Left posterior cingulate cortex	-0.231	0.048	[-0.324 - -0.137]	1.279E-06	4.542E-06
Right posterior cingulate cortex	-0.156	0.066	[-0.285 - -0.027]	1.773E-02	1.967E-02
Left frontal pole	-0.126	0.058	[-0.239 - -0.013]	2.884E-02	3.102E-02
Right frontal pole	-0.190	0.062	[-0.312 - -0.069]	2.188E-03	2.824E-03
Left paracentral lobule	-0.146	0.065	[-0.273 - -0.020]	2.335E-02	2.550E-02
Right paracentral lobule	-0.197	0.048	[-0.291 - -0.104]	3.316E-05	6.421E-05
Left insula	-0.162	0.059	[-0.279 - -0.046]	6.254E-03	7.279E-03
Right insula	-0.210	0.063	[-0.334 - -0.086]	9.412E-04	1.285E-03
Left entorhinal cortex	-0.158	0.062	[-0.278 - -0.037]	1.038E-02	1.170E-02
Right entorhinal cortex	-0.048	0.053	[-0.151 - 0.055]	3.637E-01	3.743E-01
Left temporal pole	-0.078	0.071	[-0.218 - 0.061]	2.719E-01	2.839E-01
Right temporal pole	-0.056	0.071	[-0.196 - 0.084]	4.312E-01	4.373E-01
Left isthmus cingulate cortex	-0.032	0.052	[-0.134 - 0.071]	5.475E-01	5.475E-01
Right isthmus cingulate cortex	-0.062	0.048	[-0.155 - 0.031]	1.924E-01	2.039E-01

Supplementary Table S150. Cortical surface area differences between individuals with schizophrenia on second-generation antipsychotic medications and individuals with schizophrenia who are unmedicated with antipsychotics controlling for age and sex

	d	Std. Err.	95% CI	*p*-value	FDR *q*-value
Total cortical surface area	-0.151	0.140	[-0.426 - 0.123]	2.798E-01	6.500E-01
Left hemisphere	-0.147	0.135	[-0.413 - 0.118]	2.768E-01	6.500E-01
Right hemisphere	-0.155	0.144	[-0.437 - 0.128]	2.836E-01	6.500E-01
Left superior frontal gyrus	-0.125	0.135	[-0.389 - 0.138]	3.510E-01	6.500E-01
Right superior frontal gyrus	-0.260	0.161	[-0.576 - 0.056]	1.067E-01	6.500E-01
Left pars orbitalis of inferior frontal gyrus	-0.005	0.122	[-0.243 - 0.233]	9.674E-01	9.994E-01
Right pars orbitalis of inferior frontal gyrus	-0.147	0.147	[-0.435 - 0.141]	3.171E-01	6.500E-01
Left precentral gyrus	0.025	0.151	[-0.271 - 0.321]	8.667E-01	9.994E-01
Right precentral gyrus	-0.047	0.202	[-0.442 - 0.348]	8.139E-01	9.794E-01
Left lingual gyrus	-0.192	0.122	[-0.431 - 0.046]	1.145E-01	6.500E-01
Right lingual gyrus	-0.227	0.158	[-0.535 - 0.082]	1.505E-01	6.500E-01
Left superior temporal gyrus	-0.189	0.168	[-0.518 - 0.141]	2.616E-01	6.500E-01
Right superior temporal gyrus	-0.066	0.198	[-0.454 - 0.322]	7.397E-01	9.379E-01
Left fusiform gyrus	-0.115	0.122	[-0.353 - 0.124]	3.454E-01	6.500E-01
Right fusiform gyrus	-0.180	0.149	[-0.472 - 0.112]	2.270E-01	6.500E-01
Left inferior parietal cortex	-0.018	0.122	[-0.257 - 0.220]	8.804E-01	9.994E-01
Right inferior parietal cortex	-0.130	0.146	[-0.416 - 0.157]	3.754E-01	6.500E-01
Left lateral occipital cortex	-0.229	0.207	[-0.635 - 0.176]	2.679E-01	6.500E-01
Right lateral occipital cortex	-0.205	0.122	[-0.444 - 0.034]	9.223E-02	6.500E-01
Left rostral middle frontal gyrus	-0.216	0.142	[-0.493 - 0.061]	1.270E-01	6.500E-01
Right rostral middle frontal gyrus	-0.115	0.122	[-0.353 - 0.124]	3.457E-01	6.500E-01
Left precuneus	-0.061	0.158	[-0.371 - 0.249]	6.992E-01	9.367E-01
Right precuneus	-0.146	0.143	[-0.427 - 0.135]	3.082E-01	6.500E-01
Left inferior temporal gyrus	0.101	0.122	[-0.138 - 0.339]	4.083E-01	6.617E-01
Right inferior temporal gyrus	0.060	0.122	[-0.178 - 0.299]	6.190E-01	8.618E-01
Left lateral orbitofrontal cortex	0.002	0.122	[-0.237 - 0.240]	9.886E-01	9.994E-01
Right lateral orbitofrontal cortex	-0.005	0.160	[-0.318 - 0.309]	9.762E-01	9.994E-01
Left middle temporal gyrus	0.088	0.122	[-0.151 - 0.326]	4.709E-01	7.430E-01
Right middle temporal gyrus	-0.024	0.122	[-0.263 - 0.214]	8.417E-01	9.960E-01
Left postcentral gyrus	-0.141	0.122	[-0.380 - 0.097]	2.462E-01	6.500E-01
Right postcentral gyrus	0.000	0.164	[-0.321 - 0.321]	9.994E-01	9.994E-01
Left medial orbitofrontal cortex	-0.260	0.210	[-0.672 - 0.152]	2.164E-01	6.500E-01
Right medial orbitofrontal cortex	0.019	0.188	[-0.350 - 0.388]	9.209E-01	9.994E-01
Left cuneus	-0.175	0.122	[-0.413 - 0.064]	1.512E-01	6.500E-01
Right cuneus	-0.188	0.122	[-0.426 - 0.051]	1.224E-01	6.500E-01
Left pars triangularis of inferior frontal gyrus	-0.120	0.197	[-0.506 - 0.266]	5.416E-01	7.847E-01
Right pars triangularis of inferior frontal gyrus	-0.221	0.184	[-0.581 - 0.138]	2.279E-01	6.500E-01
Left superior parietal cortex	-0.007	0.176	[-0.352 - 0.338]	9.678E-01	9.994E-01
Right superior parietal cortex	-0.122	0.134	[-0.384 - 0.141]	3.637E-01	6.500E-01
Left pars opercularis of inferior frontal gyrus	0.007	0.122	[-0.232 - 0.245]	9.563E-01	9.994E-01
Right pars opercularis of inferior frontal gyrus	-0.077	0.122	[-0.316 - 0.161]	5.252E-01	7.769E-01
Left supramarginal gyrus	-0.205	0.181	[-0.560 - 0.149]	2.557E-01	6.500E-01
Right supramarginal gyrus	-0.086	0.158	[-0.397 - 0.224]	5.868E-01	8.333E-01
Left pericalcarine cortex	-0.161	0.122	[-0.399 - 0.077]	1.854E-01	6.500E-01
Right pericalcarine cortex	-0.261	0.122	[-0.500 - -0.022]	3.211E-02	6.500E-01
Left parahippocampal gyrus	-0.182	0.122	[-0.421 - 0.056]	1.337E-01	6.500E-01
Right parahippocampal gyrus	-0.268	0.184	[-0.629 - 0.093]	1.461E-01	6.500E-01
Left caudal middle frontal gyrus	-0.134	0.122	[-0.373 - 0.104]	2.689E-01	6.500E-01
Right caudal middle frontal gyrus	-0.122	0.184	[-0.483 - 0.240]	5.099E-01	7.703E-01
Left transverse temporal gyrus	-0.061	0.184	[-0.421 - 0.299]	7.382E-01	9.379E-01
Right transverse temporal gyrus	-0.285	0.154	[-0.587 - 0.018]	6.505E-02	6.500E-01
Left banks of superior temporal sulcus	0.005	0.122	[-0.234 - 0.243]	9.693E-01	9.994E-01
Right banks of superior temporal sulcus	0.173	0.147	[-0.116 - 0.462]	2.409E-01	6.500E-01
Left caudal anterior cingulate cortex	-0.243	0.163	[-0.562 - 0.076]	1.348E-01	6.500E-01
Right caudal anterior cingulate cortex	0.109	0.122	[-0.129 - 0.347]	3.702E-01	6.500E-01
Left rostral anterior cingulate cortex	-0.138	0.122	[-0.377 - 0.100]	2.559E-01	6.500E-01
Right rostral anterior cingulate cortex	-0.083	0.122	[-0.322 - 0.155]	4.926E-01	7.603E-01
Left posterior cingulate cortex	-0.142	0.122	[-0.380 - 0.097]	2.440E-01	6.500E-01
Right posterior cingulate cortex	0.156	0.122	[-0.083 - 0.395]	2.003E-01	6.500E-01
Left frontal pole	-0.066	0.152	[-0.364 - 0.231]	6.621E-01	9.040E-01
Right frontal pole	-0.394	0.122	[-0.634 - -0.154]	1.270E-03	9.014E-02
Left paracentral lobule	-0.141	0.139	[-0.414 - 0.132]	3.119E-01	6.500E-01
Right paracentral lobule	0.037	0.122	[-0.201 - 0.276]	7.607E-01	9.451E-01
Left insula	0.003	0.122	[-0.235 - 0.242]	9.772E-01	9.994E-01
Right insula	-0.050	0.142	[-0.328 - 0.227]	7.228E-01	9.379E-01
Left entorhinal cortex	-0.126	0.151	[-0.422 - 0.171]	4.055E-01	6.617E-01
Right entorhinal cortex	-0.035	0.122	[-0.274 - 0.203]	7.721E-01	9.451E-01
Left temporal pole	-0.119	0.122	[-0.357 - 0.119]	3.280E-01	6.500E-01
Right temporal pole	-0.124	0.122	[-0.363 - 0.114]	3.079E-01	6.500E-01
Left isthmus cingulate cortex	-0.100	0.122	[-0.339 - 0.138]	4.101E-01	6.617E-01
Right isthmus cingulate cortex	-0.119	0.122	[-0.358 - 0.119]	3.265E-01	6.500E-01

Supplementary Table S151. Cortical surface area differences between individuals with schizophrenia on first-generation antipsychotic medications and healthy comparison subjects controlling for age and sex

	d	Std. Err.	95% CI	*p*-value	FDR *q*-value
Total cortical surface area	-0.404	0.101	[-0.601 - -0.206]	6.102E-05	1.133E-03
Left hemisphere	-0.397	0.101	[-0.594 - -0.199]	8.293E-05	1.178E-03
Right hemisphere	-0.408	0.101	[-0.605 - -0.210]	5.148E-05	1.133E-03
Left superior frontal gyrus	-0.410	0.101	[-0.608 - -0.213]	4.632E-05	1.133E-03
Right superior frontal gyrus	-0.402	0.101	[-0.599 - -0.205]	6.385E-05	1.133E-03
Left pars orbitalis of inferior frontal gyrus	-0.283	0.157	[-0.589 - 0.024]	7.112E-02	1.202E-01
Right pars orbitalis of inferior frontal gyrus	-0.266	0.100	[-0.463 - -0.069]	7.995E-03	2.580E-02
Left precentral gyrus	-0.197	0.153	[-0.497 - 0.103]	1.981E-01	2.468E-01
Right precentral gyrus	-0.272	0.100	[-0.469 - -0.075]	6.812E-03	2.444E-02
Left lingual gyrus	-0.176	0.100	[-0.373 - 0.020]	7.833E-02	1.236E-01
Right lingual gyrus	-0.284	0.100	[-0.481 - -0.087]	4.682E-03	2.183E-02
Left superior temporal gyrus	-0.222	0.100	[-0.419 - -0.026]	2.676E-02	5.821E-02
Right superior temporal gyrus	-0.284	0.126	[-0.532 - -0.036]	2.483E-02	5.705E-02
Left fusiform gyrus	-0.369	0.101	[-0.566 - -0.172]	2.413E-04	2.855E-03
Right fusiform gyrus	-0.182	0.100	[-0.378 - 0.015]	6.976E-02	1.202E-01
Left inferior parietal cortex	-0.287	0.100	[-0.483 - -0.090]	4.318E-03	2.183E-02
Right inferior parietal cortex	-0.285	0.100	[-0.482 - -0.088]	4.528E-03	2.183E-02
Left lateral occipital cortex	-0.376	0.106	[-0.583 - -0.169]	3.779E-04	3.397E-03
Right lateral occipital cortex	-0.287	0.100	[-0.483 - -0.090]	4.267E-03	2.183E-02
Left rostral middle frontal gyrus	-0.330	0.100	[-0.527 - -0.133]	1.033E-03	8.150E-03
Right rostral middle frontal gyrus	-0.316	0.100	[-0.513 - -0.119]	1.660E-03	1.179E-02
Left precuneus	-0.280	0.110	[-0.495 - -0.065]	1.080E-02	3.335E-02
Right precuneus	-0.262	0.127	[-0.512 - -0.012]	3.964E-02	8.041E-02
Left inferior temporal gyrus	-0.280	0.100	[-0.477 - -0.084]	5.223E-03	2.183E-02
Right inferior temporal gyrus	-0.357	0.101	[-0.554 - -0.160]	3.827E-04	3.397E-03
Left lateral orbitofrontal cortex	-0.221	0.153	[-0.521 - 0.078]	1.467E-01	2.003E-01
Right lateral orbitofrontal cortex	-0.249	0.100	[-0.446 - -0.052]	1.308E-02	3.869E-02
Left middle temporal gyrus	-0.086	0.121	[-0.323 - 0.151]	4.776E-01	5.469E-01
Right middle temporal gyrus	-0.153	0.116	[-0.380 - 0.073]	1.849E-01	2.344E-01
Left postcentral gyrus	-0.268	0.150	[-0.563 - 0.027]	7.465E-02	1.205E-01
Right postcentral gyrus	-0.141	0.100	[-0.338 - 0.055]	1.587E-01	2.086E-01
Left medial orbitofrontal cortex	-0.118	0.152	[-0.415 - 0.179]	4.356E-01	5.070E-01
Right medial orbitofrontal cortex	-0.225	0.100	[-0.422 - -0.028]	2.491E-02	5.705E-02
Left cuneus	-0.066	0.100	[-0.262 - 0.130]	5.110E-01	5.759E-01
Right cuneus	-0.273	0.100	[-0.470 - -0.077]	6.446E-03	2.444E-02
Left pars triangularis of inferior frontal gyrus	-0.277	0.114	[-0.500 - -0.054]	1.483E-02	4.212E-02
Right pars triangularis of inferior frontal gyrus	-0.239	0.131	[-0.496 - 0.017]	6.716E-02	1.202E-01
Left superior parietal cortex	-0.059	0.145	[-0.344 - 0.226]	6.838E-01	7.586E-01
Right superior parietal cortex	-0.285	0.125	[-0.531 - -0.040]	2.267E-02	5.551E-02
Left pars opercularis of inferior frontal gyrus	-0.165	0.119	[-0.398 - 0.069]	1.664E-01	2.148E-01
Right pars opercularis of inferior frontal gyrus	-0.297	0.100	[-0.494 - -0.100]	3.117E-03	2.012E-02
Left supramarginal gyrus	-0.197	0.100	[-0.394 - -0.001]	4.917E-02	9.697E-02
Right supramarginal gyrus	-0.182	0.100	[-0.378 - 0.015]	7.009E-02	1.202E-01
Left pericalcarine cortex	-0.180	0.100	[-0.376 - 0.017]	7.312E-02	1.205E-01
Right pericalcarine cortex	-0.241	0.100	[-0.437 - -0.044]	1.638E-02	4.473E-02
Left parahippocampal gyrus	-0.175	0.100	[-0.371 - 0.022]	8.096E-02	1.244E-01
Right parahippocampal gyrus	-0.280	0.100	[-0.477 - -0.084]	5.228E-03	2.183E-02
Left caudal middle frontal gyrus	-0.221	0.132	[-0.480 - 0.038]	9.474E-02	1.401E-01
Right caudal middle frontal gyrus	-0.202	0.142	[-0.481 - 0.076]	1.549E-01	2.075E-01
Left transverse temporal gyrus	-0.220	0.100	[-0.416 - -0.024]	2.808E-02	5.864E-02
Right transverse temporal gyrus	-0.237	0.100	[-0.434 - -0.041]	1.792E-02	4.712E-02
Left banks of superior temporal sulcus	-0.271	0.100	[-0.468 - -0.075]	6.886E-03	2.444E-02
Right banks of superior temporal sulcus	-0.195	0.100	[-0.392 - 0.001]	5.168E-02	9.918E-02
Left caudal anterior cingulate cortex	-0.268	0.100	[-0.465 - -0.071]	7.627E-03	2.579E-02
Right caudal anterior cingulate cortex	-0.231	0.100	[-0.428 - -0.035]	2.121E-02	5.378E-02
Left rostral anterior cingulate cortex	-0.187	0.115	[-0.412 - 0.038]	1.032E-01	1.495E-01
Right rostral anterior cingulate cortex	-0.245	0.111	[-0.462 - -0.028]	2.705E-02	5.821E-02
Left posterior cingulate cortex	-0.128	0.100	[-0.324 - 0.069]	2.025E-01	2.479E-01
Right posterior cingulate cortex	-0.154	0.100	[-0.350 - 0.043]	1.250E-01	1.775E-01
Left frontal pole	-0.029	0.100	[-0.225 - 0.167]	7.723E-01	8.308E-01
Right frontal pole	-0.011	0.100	[-0.207 - 0.185]	9.122E-01	9.315E-01
Left paracentral lobule	-0.056	0.168	[-0.386 - 0.273]	7.376E-01	8.057E-01
Right paracentral lobule	-0.225	0.118	[-0.457 - 0.007]	5.761E-02	1.076E-01
Left insula	-0.204	0.138	[-0.475 - 0.067]	1.392E-01	1.938E-01
Right insula	-0.199	0.115	[-0.424 - 0.026]	8.235E-02	1.244E-01
Left entorhinal cortex	-0.107	0.100	[-0.303 - 0.089]	2.847E-01	3.368E-01
Right entorhinal cortex	-0.026	0.117	[-0.254 - 0.203]	8.264E-01	8.757E-01
Left temporal pole	-0.114	0.100	[-0.310 - 0.082]	2.553E-01	3.073E-01
Right temporal pole	0.019	0.122	[-0.220 - 0.257]	8.773E-01	9.160E-01
Left isthmus cingulate cortex	-0.011	0.114	[-0.234 - 0.211]	9.198E-01	9.315E-01
Right isthmus cingulate cortex	-0.009	0.100	[-0.205 - 0.188]	9.315E-01	9.315E-01

Supplementary Table S152. Cortical surface area differences between individuals with schizophrenia on first-generation antipsychotic medications and individuals with schizophrenia who are unmedicated with antipsychotics controlling for age and sex

	d	Std. Err.	95% CI	*p*-value	FDR *q*-value
Total cortical surface area	-0.104	0.319	[-0.730 - 0.522]	7.455E-01	9.821E-01
Left hemisphere	-0.069	0.320	[-0.695 - 0.557]	8.296E-01	9.821E-01
Right hemisphere	-0.137	0.319	[-0.763 - 0.489]	6.687E-01	9.821E-01
Left superior frontal gyrus	-0.027	0.319	[-0.651 - 0.597]	9.322E-01	9.821E-01
Right superior frontal gyrus	0.086	0.319	[-0.539 - 0.710]	7.877E-01	9.821E-01
Left pars orbitalis of inferior frontal gyrus	0.210	0.325	[-0.427 - 0.848]	5.177E-01	9.821E-01
Right pars orbitalis of inferior frontal gyrus	0.175	0.320	[-0.452 - 0.802]	5.844E-01	9.821E-01
Left precentral gyrus	0.792	0.328	[0.148 - 1.435]	1.587E-02	9.421E-01
Right precentral gyrus	0.034	0.318	[-0.590 - 0.658]	9.143E-01	9.821E-01
Left lingual gyrus	-0.056	0.549	[-1.132 - 1.021]	9.192E-01	9.821E-01
Right lingual gyrus	0.182	0.320	[-0.446 - 0.809]	5.708E-01	9.821E-01
Left superior temporal gyrus	0.128	0.320	[-0.499 - 0.754]	6.897E-01	9.821E-01
Right superior temporal gyrus	0.122	0.319	[-0.503 - 0.748]	7.015E-01	9.821E-01
Left fusiform gyrus	-0.568	0.462	[-1.473 - 0.337]	2.186E-01	9.821E-01
Right fusiform gyrus	-0.561	0.322	[-1.193 - 0.071]	8.188E-02	9.821E-01
Left inferior parietal cortex	-0.487	0.321	[-1.117 - 0.143]	1.298E-01	9.821E-01
Right inferior parietal cortex	-0.494	0.322	[-1.124 - 0.136]	1.246E-01	9.821E-01
Left lateral occipital cortex	-0.039	0.339	[-0.704 - 0.626]	9.078E-01	9.821E-01
Right lateral occipital cortex	-0.199	0.320	[-0.826 - 0.428]	5.335E-01	9.821E-01
Left rostral middle frontal gyrus	-0.129	0.477	[-1.064 - 0.807]	7.878E-01	9.821E-01
Right rostral middle frontal gyrus	-0.132	0.442	[-0.998 - 0.735]	7.660E-01	9.821E-01
Left precuneus	-0.160	0.402	[-0.947 - 0.627]	6.903E-01	9.821E-01
Right precuneus	-0.548	0.322	[-1.180 - 0.083]	8.887E-02	9.821E-01
Left inferior temporal gyrus	-0.059	0.318	[-0.683 - 0.565]	8.535E-01	9.821E-01
Right inferior temporal gyrus	-0.111	0.319	[-0.735 - 0.514]	7.286E-01	9.821E-01
Left lateral orbitofrontal cortex	0.372	0.762	[-1.122 - 1.866]	6.253E-01	9.821E-01
Right lateral orbitofrontal cortex	-0.227	0.427	[-1.063 - 0.610]	5.952E-01	9.821E-01
Left middle temporal gyrus	0.197	0.319	[-0.428 - 0.822]	5.362E-01	9.821E-01
Right middle temporal gyrus	0.051	0.319	[-0.573 - 0.676]	8.724E-01	9.821E-01
Left postcentral gyrus	0.048	0.452	[-0.838 - 0.934]	9.157E-01	9.821E-01
Right postcentral gyrus	-0.291	0.494	[-1.259 - 0.676]	5.552E-01	9.821E-01
Left medial orbitofrontal cortex	-0.166	0.502	[-1.150 - 0.817]	7.402E-01	9.821E-01
Right medial orbitofrontal cortex	0.374	0.320	[-0.254 - 1.002]	2.427E-01	9.821E-01
Left cuneus	-0.214	0.346	[-0.892 - 0.463]	5.352E-01	9.821E-01
Right cuneus	0.002	0.319	[-0.623 - 0.628]	9.940E-01	9.940E-01
Left pars triangularis of inferior frontal gyrus	0.201	0.322	[-0.430 - 0.831]	5.325E-01	9.821E-01
Right pars triangularis of inferior frontal gyrus	0.039	0.448	[-0.840 - 0.918]	9.306E-01	9.821E-01
Left superior parietal cortex	0.522	0.322	[-0.109 - 1.153]	1.049E-01	9.821E-01
Right superior parietal cortex	0.346	0.321	[-0.282 - 0.974]	2.803E-01	9.821E-01
Left pars opercularis of inferior frontal gyrus	0.080	0.319	[-0.546 - 0.706]	8.015E-01	9.821E-01
Right pars opercularis of inferior frontal gyrus	0.126	0.319	[-0.499 - 0.751]	6.917E-01	9.821E-01
Left supramarginal gyrus	-0.303	0.320	[-0.930 - 0.323]	3.429E-01	9.821E-01
Right supramarginal gyrus	0.222	0.320	[-0.404 - 0.849]	4.866E-01	9.821E-01
Left pericalcarine cortex	-0.233	0.319	[-0.859 - 0.392]	4.651E-01	9.821E-01
Right pericalcarine cortex	-0.345	0.320	[-0.973 - 0.282]	2.806E-01	9.821E-01
Left parahippocampal gyrus	-0.117	0.319	[-0.741 - 0.507]	7.135E-01	9.821E-01
Right parahippocampal gyrus	-0.309	0.320	[-0.936 - 0.317]	3.330E-01	9.821E-01
Left caudal middle frontal gyrus	-0.243	0.725	[-1.664 - 1.179]	7.378E-01	9.821E-01
Right caudal middle frontal gyrus	0.320	0.321	[-0.309 - 0.949]	3.184E-01	9.821E-01
Left transverse temporal gyrus	0.438	0.322	[-0.193 - 1.070]	1.738E-01	9.821E-01
Right transverse temporal gyrus	-0.099	0.319	[-0.724 - 0.525]	7.552E-01	9.821E-01
Left banks of superior temporal sulcus	0.014	0.319	[-0.610 - 0.638]	9.650E-01	9.821E-01
Right banks of superior temporal sulcus	-0.375	0.321	[-1.004 - 0.253]	2.420E-01	9.821E-01
Left caudal anterior cingulate cortex	-0.441	0.520	[-1.460 - 0.578]	3.964E-01	9.821E-01
Right caudal anterior cingulate cortex	-0.062	0.319	[-0.687 - 0.563]	8.453E-01	9.821E-01
Left rostral anterior cingulate cortex	-0.289	0.438	[-1.147 - 0.568]	5.082E-01	9.821E-01
Right rostral anterior cingulate cortex	-0.634	0.324	[-1.268 - 0.000]	5.013E-02	9.821E-01
Left posterior cingulate cortex	0.077	0.319	[-0.547 - 0.702]	8.086E-01	9.821E-01
Right posterior cingulate cortex	0.311	0.320	[-0.316 - 0.937]	3.311E-01	9.821E-01
Left frontal pole	-0.150	0.319	[-0.775 - 0.474]	6.370E-01	9.821E-01
Right frontal pole	-0.192	0.350	[-0.879 - 0.494]	5.828E-01	9.821E-01
Left paracentral lobule	0.235	0.800	[-1.333 - 1.804]	7.686E-01	9.821E-01
Right paracentral lobule	-0.300	0.320	[-0.926 - 0.327]	3.485E-01	9.821E-01
Left insula	-0.167	0.338	[-0.829 - 0.495]	6.211E-01	9.821E-01
Right insula	-0.157	0.319	[-0.782 - 0.468]	6.229E-01	9.821E-01
Left entorhinal cortex	-0.382	0.320	[-1.009 - 0.246]	2.334E-01	9.821E-01
Right entorhinal cortex	-0.721	0.325	[-1.358 - -0.084]	2.654E-02	9.421E-01
Left temporal pole	0.016	0.318	[-0.608 - 0.640]	9.611E-01	9.821E-01
Right temporal pole	0.043	0.319	[-0.581 - 0.667]	8.920E-01	9.821E-01
Left isthmus cingulate cortex	0.215	0.319	[-0.411 - 0.840]	5.015E-01	9.821E-01
Right isthmus cingulate cortex	0.013	0.318	[-0.611 - 0.637]	9.682E-01	9.821E-01

Supplementary Table S153. Cortical surface area differences between individuals with schizophrenia on first-generation antipsychotic medications and individuals with schizophrenia on second-generation antipsychotic medications controlling for age and sex

	d	Std. Err.	95% CI	*p*-value	FDR *q*-value
Total cortical surface area	0.051	0.141	[-0.226 - 0.327]	7.197E-01	9.766E-01
Left hemisphere	0.053	0.141	[-0.224 - 0.330]	7.080E-01	9.766E-01
Right hemisphere	0.049	0.141	[-0.228 - 0.326]	7.300E-01	9.766E-01
Left superior frontal gyrus	0.017	0.143	[-0.263 - 0.297]	9.057E-01	9.960E-01
Right superior frontal gyrus	-0.001	0.141	[-0.277 - 0.275]	9.944E-01	9.960E-01
Left pars orbitalis of inferior frontal gyrus	0.056	0.170	[-0.277 - 0.388]	7.419E-01	9.766E-01
Right pars orbitalis of inferior frontal gyrus	0.070	0.141	[-0.206 - 0.347]	6.177E-01	9.766E-01
Left precentral gyrus	0.192	0.196	[-0.192 - 0.577]	3.271E-01	9.766E-01
Right precentral gyrus	-0.079	0.145	[-0.363 - 0.205]	5.864E-01	9.766E-01
Left lingual gyrus	0.256	0.141	[-0.021 - 0.533]	6.979E-02	9.766E-01
Right lingual gyrus	0.189	0.141	[-0.087 - 0.466]	1.801E-01	9.766E-01
Left superior temporal gyrus	-0.001	0.141	[-0.277 - 0.276]	9.960E-01	9.960E-01
Right superior temporal gyrus	0.002	0.154	[-0.299 - 0.304]	9.876E-01	9.960E-01
Left fusiform gyrus	-0.127	0.194	[-0.507 - 0.253]	5.120E-01	9.766E-01
Right fusiform gyrus	-0.010	0.145	[-0.293 - 0.273]	9.450E-01	9.960E-01
Left inferior parietal cortex	0.100	0.141	[-0.176 - 0.377]	4.767E-01	9.766E-01
Right inferior parietal cortex	0.201	0.217	[-0.225 - 0.627]	3.551E-01	9.766E-01
Left lateral occipital cortex	0.128	0.141	[-0.148 - 0.405]	3.634E-01	9.766E-01
Right lateral occipital cortex	0.122	0.141	[-0.155 - 0.399]	3.876E-01	9.766E-01
Left rostral middle frontal gyrus	-0.085	0.141	[-0.362 - 0.191]	5.448E-01	9.766E-01
Right rostral middle frontal gyrus	-0.130	0.141	[-0.407 - 0.147]	3.566E-01	9.766E-01
Left precuneus	-0.053	0.223	[-0.490 - 0.384]	8.130E-01	9.960E-01
Right precuneus	-0.109	0.204	[-0.510 - 0.291]	5.931E-01	9.766E-01
Left inferior temporal gyrus	0.079	0.141	[-0.197 - 0.356]	5.736E-01	9.766E-01
Right inferior temporal gyrus	0.064	0.141	[-0.212 - 0.341]	6.485E-01	9.766E-01
Left lateral orbitofrontal cortex	0.123	0.141	[-0.154 - 0.400]	3.840E-01	9.766E-01
Right lateral orbitofrontal cortex	0.134	0.141	[-0.143 - 0.411]	3.420E-01	9.766E-01
Left middle temporal gyrus	0.175	0.141	[-0.102 - 0.452]	2.156E-01	9.766E-01
Right middle temporal gyrus	0.244	0.170	[-0.089 - 0.577]	1.510E-01	9.766E-01
Left postcentral gyrus	0.182	0.191	[-0.191 - 0.555]	3.395E-01	9.766E-01
Right postcentral gyrus	0.201	0.199	[-0.189 - 0.590]	3.125E-01	9.766E-01
Left medial orbitofrontal cortex	0.140	0.141	[-0.137 - 0.417]	3.208E-01	9.766E-01
Right medial orbitofrontal cortex	0.082	0.141	[-0.195 - 0.359]	5.610E-01	9.766E-01
Left cuneus	0.094	0.141	[-0.183 - 0.370]	5.057E-01	9.766E-01
Right cuneus	0.011	0.141	[-0.265 - 0.287]	9.364E-01	9.960E-01
Left pars triangularis of inferior frontal gyrus	-0.017	0.176	[-0.362 - 0.327]	9.212E-01	9.960E-01
Right pars triangularis of inferior frontal gyrus	0.072	0.141	[-0.204 - 0.349]	6.076E-01	9.766E-01
Left superior parietal cortex	0.048	0.155	[-0.255 - 0.351]	7.565E-01	9.766E-01
Right superior parietal cortex	0.053	0.151	[-0.243 - 0.349]	7.267E-01	9.766E-01
Left pars opercularis of inferior frontal gyrus	0.248	0.187	[-0.118 - 0.614]	1.847E-01	9.766E-01
Right pars opercularis of inferior frontal gyrus	0.094	0.201	[-0.300 - 0.488]	6.408E-01	9.766E-01
Left supramarginal gyrus	-0.089	0.176	[-0.435 - 0.256]	6.129E-01	9.766E-01
Right supramarginal gyrus	0.061	0.141	[-0.215 - 0.338]	6.646E-01	9.766E-01
Left pericalcarine cortex	-0.022	0.141	[-0.299 - 0.254]	8.734E-01	9.960E-01
Right pericalcarine cortex	0.094	0.141	[-0.182 - 0.370]	5.044E-01	9.766E-01
Left parahippocampal gyrus	-0.017	0.141	[-0.294 - 0.259]	9.017E-01	9.960E-01
Right parahippocampal gyrus	0.003	0.169	[-0.328 - 0.334]	9.859E-01	9.960E-01
Left caudal middle frontal gyrus	0.116	0.187	[-0.250 - 0.482]	5.342E-01	9.766E-01
Right caudal middle frontal gyrus	0.041	0.223	[-0.396 - 0.478]	8.548E-01	9.960E-01
Left transverse temporal gyrus	-0.220	0.168	[-0.549 - 0.109]	1.908E-01	9.766E-01
Right transverse temporal gyrus	-0.102	0.174	[-0.442 - 0.238]	5.574E-01	9.766E-01
Left banks of superior temporal sulcus	-0.075	0.141	[-0.351 - 0.202]	5.953E-01	9.766E-01
Right banks of superior temporal sulcus	0.231	0.224	[-0.208 - 0.671]	3.025E-01	9.766E-01
Left caudal anterior cingulate cortex	-0.136	0.214	[-0.554 - 0.283]	5.257E-01	9.766E-01
Right caudal anterior cingulate cortex	0.070	0.141	[-0.207 - 0.346]	6.223E-01	9.766E-01
Left rostral anterior cingulate cortex	-0.107	0.177	[-0.453 - 0.240]	5.462E-01	9.766E-01
Right rostral anterior cingulate cortex	0.004	0.141	[-0.273 - 0.280]	9.794E-01	9.960E-01
Left posterior cingulate cortex	0.274	0.141	[-0.003 - 0.551]	5.215E-02	9.766E-01
Right posterior cingulate cortex	0.155	0.142	[-0.123 - 0.432]	2.746E-01	9.766E-01
Left frontal pole	0.152	0.210	[-0.260 - 0.564]	4.703E-01	9.766E-01
Right frontal pole	-0.006	0.187	[-0.373 - 0.362]	9.759E-01	9.960E-01
Left paracentral lobule	0.255	0.213	[-0.163 - 0.672]	2.317E-01	9.766E-01
Right paracentral lobule	0.059	0.182	[-0.298 - 0.415]	7.461E-01	9.766E-01
Left insula	-0.059	0.141	[-0.336 - 0.218]	6.744E-01	9.766E-01
Right insula	-0.136	0.144	[-0.417 - 0.146]	3.444E-01	9.766E-01
Left entorhinal cortex	0.093	0.156	[-0.213 - 0.399]	5.500E-01	9.766E-01
Right entorhinal cortex	0.100	0.194	[-0.280 - 0.480]	6.058E-01	9.766E-01
Left temporal pole	0.007	0.150	[-0.287 - 0.300]	9.652E-01	9.960E-01
Right temporal pole	0.131	0.181	[-0.224 - 0.486]	4.697E-01	9.766E-01
Left isthmus cingulate cortex	-0.067	0.141	[-0.344 - 0.209]	6.335E-01	9.766E-01
Right isthmus cingulate cortex	-0.010	0.141	[-0.286 - 0.267]	9.460E-01	9.960E-01

Supplementary Table S154. Cortical surface area differences between individuals with schizophrenia on both first-generation and second-generation antipsychotic medications and healthy comparison subjects controlling for age and sex

	d	Std. Err.	95% CI	*p*-value	FDR *q*-value
Total cortical surface area	-0.258	0.073	[-0.401 - -0.114]	4.325E-04	1.024E-02
Left hemisphere	-0.257	0.072	[-0.399 - -0.116]	3.578E-04	1.024E-02
Right hemisphere	-0.258	0.076	[-0.408 - -0.109]	7.139E-04	1.222E-02
Left superior frontal gyrus	-0.325	0.083	[-0.488 - -0.162]	9.608E-05	6.822E-03
Right superior frontal gyrus	-0.243	0.080	[-0.400 - -0.087]	2.339E-03	1.873E-02
Left pars orbitalis of inferior frontal gyrus	-0.221	0.073	[-0.363 - -0.078]	2.374E-03	1.873E-02
Right pars orbitalis of inferior frontal gyrus	-0.184	0.108	[-0.395 - 0.028]	8.921E-02	1.348E-01
Left precentral gyrus	-0.233	0.078	[-0.385 - -0.081]	2.715E-03	1.928E-02
Right precentral gyrus	-0.220	0.072	[-0.361 - -0.079]	2.281E-03	1.873E-02
Left lingual gyrus	-0.155	0.072	[-0.296 - -0.014]	3.110E-02	7.124E-02
Right lingual gyrus	-0.194	0.072	[-0.335 - -0.052]	7.180E-03	3.670E-02
Left superior temporal gyrus	-0.189	0.076	[-0.337 - -0.041]	1.226E-02	4.144E-02
Right superior temporal gyrus	-0.181	0.072	[-0.322 - -0.039]	1.214E-02	4.144E-02
Left fusiform gyrus	-0.261	0.114	[-0.484 - -0.037]	2.240E-02	5.720E-02
Right fusiform gyrus	-0.182	0.108	[-0.394 - 0.031]	9.351E-02	1.383E-01
Left inferior parietal cortex	-0.178	0.096	[-0.366 - 0.011]	6.465E-02	1.067E-01
Right inferior parietal cortex	-0.211	0.102	[-0.411 - -0.011]	3.887E-02	7.947E-02
Left lateral occipital cortex	-0.157	0.072	[-0.298 - -0.016]	2.916E-02	6.901E-02
Right lateral occipital cortex	-0.172	0.072	[-0.313 - -0.031]	1.690E-02	4.810E-02
Left rostral middle frontal gyrus	-0.148	0.072	[-0.290 - -0.007]	3.918E-02	7.947E-02
Right rostral middle frontal gyrus	-0.191	0.085	[-0.357 - -0.024]	2.505E-02	6.134E-02
Left precuneus	-0.200	0.074	[-0.345 - -0.054]	7.102E-03	3.670E-02
Right precuneus	-0.244	0.074	[-0.390 - -0.098]	1.033E-03	1.222E-02
Left inferior temporal gyrus	-0.183	0.072	[-0.324 - -0.041]	1.123E-02	4.144E-02
Right inferior temporal gyrus	-0.114	0.072	[-0.255 - 0.027]	1.139E-01	1.586E-01
Left lateral orbitofrontal cortex	-0.137	0.072	[-0.278 - 0.005]	5.779E-02	1.005E-01
Right lateral orbitofrontal cortex	-0.039	0.075	[-0.185 - 0.107]	5.998E-01	6.262E-01
Left middle temporal gyrus	-0.128	0.090	[-0.304 - 0.047]	1.518E-01	1.996E-01
Right middle temporal gyrus	-0.208	0.072	[-0.349 - -0.066]	3.974E-03	2.565E-02
Left postcentral gyrus	-0.184	0.072	[-0.325 - -0.043]	1.059E-02	4.144E-02
Right postcentral gyrus	-0.133	0.072	[-0.275 - 0.008]	6.395E-02	1.067E-01
Left medial orbitofrontal cortex	-0.046	0.072	[-0.187 - 0.095]	5.210E-01	5.604E-01
Right medial orbitofrontal cortex	-0.091	0.073	[-0.235 - 0.053]	2.163E-01	2.743E-01
Left cuneus	-0.172	0.072	[-0.313 - -0.031]	1.694E-02	4.810E-02
Right cuneus	-0.181	0.072	[-0.322 - -0.040]	1.206E-02	4.144E-02
Left pars triangularis of inferior frontal gyrus	-0.193	0.072	[-0.334 - -0.052]	7.459E-03	3.670E-02
Right pars triangularis of inferior frontal gyrus	-0.104	0.075	[-0.250 - 0.042]	1.642E-01	2.119E-01
Left superior parietal cortex	-0.156	0.075	[-0.303 - -0.008]	3.824E-02	7.947E-02
Right superior parietal cortex	-0.141	0.072	[-0.282 - 0.000]	5.018E-02	9.375E-02
Left pars opercularis of inferior frontal gyrus	-0.203	0.089	[-0.378 - -0.029]	2.256E-02	5.720E-02
Right pars opercularis of inferior frontal gyrus	-0.179	0.098	[-0.372 - 0.013]	6.831E-02	1.102E-01
Left supramarginal gyrus	-0.173	0.072	[-0.314 - -0.031]	1.653E-02	4.810E-02
Right supramarginal gyrus	-0.152	0.089	[-0.326 - 0.022]	8.733E-02	1.348E-01
Left pericalcarine cortex	-0.139	0.072	[-0.281 - 0.003]	5.508E-02	1.003E-01
Right pericalcarine cortex	-0.175	0.072	[-0.316 - -0.034]	1.485E-02	4.794E-02
Left parahippocampal gyrus	-0.088	0.124	[-0.331 - 0.155]	4.793E-01	5.235E-01
Right parahippocampal gyrus	-0.184	0.086	[-0.352 - -0.015]	3.262E-02	7.238E-02
Left caudal middle frontal gyrus	-0.159	0.084	[-0.324 - 0.005]	5.805E-02	1.005E-01
Right caudal middle frontal gyrus	-0.063	0.072	[-0.204 - 0.078]	3.815E-01	4.245E-01
Left transverse temporal gyrus	-0.165	0.072	[-0.306 - -0.024]	2.201E-02	5.720E-02
Right transverse temporal gyrus	-0.163	0.090	[-0.340 - 0.013]	7.014E-02	1.107E-01
Left banks of superior temporal sulcus	-0.174	0.108	[-0.385 - 0.037]	1.062E-01	1.509E-01
Right banks of superior temporal sulcus	-0.136	0.090	[-0.313 - 0.041]	1.310E-01	1.789E-01
Left caudal anterior cingulate cortex	0.063	0.072	[-0.078 - 0.204]	3.826E-01	4.245E-01
Right caudal anterior cingulate cortex	-0.192	0.072	[-0.333 - -0.051]	7.753E-03	3.670E-02
Left rostral anterior cingulate cortex	0.000	0.072	[-0.141 - 0.141]	9.979E-01	9.979E-01
Right rostral anterior cingulate cortex	-0.143	0.072	[-0.284 - -0.002]	4.686E-02	9.241E-02
Left posterior cingulate cortex	-0.079	0.072	[-0.220 - 0.062]	2.732E-01	3.288E-01
Right posterior cingulate cortex	-0.153	0.078	[-0.305 - 0.000]	5.011E-02	9.375E-02
Left frontal pole	-0.180	0.072	[-0.322 - -0.039]	1.220E-02	4.144E-02
Right frontal pole	-0.268	0.081	[-0.427 - -0.109]	9.759E-04	1.222E-02
Left paracentral lobule	-0.067	0.072	[-0.208 - 0.074]	3.496E-01	4.051E-01
Right paracentral lobule	-0.022	0.072	[-0.163 - 0.119]	7.603E-01	7.712E-01
Left insula	-0.088	0.072	[-0.229 - 0.053]	2.214E-01	2.757E-01
Right insula	-0.171	0.105	[-0.376 - 0.034]	1.015E-01	1.471E-01
Left entorhinal cortex	-0.049	0.089	[-0.223 - 0.125]	5.799E-01	6.146E-01
Right entorhinal cortex	-0.105	0.072	[-0.246 - 0.036]	1.455E-01	1.949E-01
Left temporal pole	-0.081	0.072	[-0.222 - 0.060]	2.584E-01	3.164E-01
Right temporal pole	-0.071	0.072	[-0.212 - 0.070]	3.240E-01	3.834E-01
Left isthmus cingulate cortex	-0.067	0.072	[-0.208 - 0.074]	3.537E-01	4.051E-01
Right isthmus cingulate cortex	-0.042	0.088	[-0.215 - 0.132]	6.362E-01	6.547E-01

Supplementary Table S155. Cortical surface area differences between individuals with schizophrenia on both first-generation and second-generation antipsychotic medications and individuals with schizophrenia who are unmedicated with antipsychotics controlling for age and sex

	d	Std. Err.	95% CI	*p*-value	FDR *q*-value
Total cortical surface area	-0.038	0.144	[-0.320 - 0.244]	7.918E-01	9.734E-01
Left hemisphere	-0.041	0.144	[-0.324 - 0.241]	7.746E-01	9.734E-01
Right hemisphere	-0.035	0.144	[-0.317 - 0.247]	8.089E-01	9.734E-01
Left superior frontal gyrus	-0.167	0.145	[-0.450 - 0.116]	2.474E-01	9.734E-01
Right superior frontal gyrus	-0.175	0.145	[-0.459 - 0.109]	2.276E-01	9.734E-01
Left pars orbitalis of inferior frontal gyrus	0.234	0.144	[-0.049 - 0.517]	1.050E-01	9.734E-01
Right pars orbitalis of inferior frontal gyrus	0.187	0.145	[-0.097 - 0.471]	1.957E-01	9.734E-01
Left precentral gyrus	-0.040	0.155	[-0.344 - 0.264]	7.977E-01	9.734E-01
Right precentral gyrus	-0.077	0.225	[-0.518 - 0.363]	7.302E-01	9.734E-01
Left lingual gyrus	-0.097	0.145	[-0.380 - 0.187]	5.047E-01	9.734E-01
Right lingual gyrus	-0.100	0.180	[-0.451 - 0.252]	5.790E-01	9.734E-01
Left superior temporal gyrus	-0.042	0.164	[-0.364 - 0.280]	7.970E-01	9.734E-01
Right superior temporal gyrus	0.131	0.144	[-0.152 - 0.414]	3.656E-01	9.734E-01
Left fusiform gyrus	-0.145	0.144	[-0.428 - 0.138]	3.148E-01	9.734E-01
Right fusiform gyrus	-0.042	0.144	[-0.324 - 0.241]	7.723E-01	9.734E-01
Left inferior parietal cortex	-0.081	0.145	[-0.365 - 0.203]	5.755E-01	9.734E-01
Right inferior parietal cortex	-0.128	0.144	[-0.411 - 0.155]	3.741E-01	9.734E-01
Left lateral occipital cortex	-0.308	0.258	[-0.813 - 0.197]	2.319E-01	9.734E-01
Right lateral occipital cortex	-0.178	0.151	[-0.474 - 0.118]	2.393E-01	9.734E-01
Left rostral middle frontal gyrus	-0.039	0.145	[-0.322 - 0.244]	7.870E-01	9.734E-01
Right rostral middle frontal gyrus	-0.068	0.144	[-0.351 - 0.215]	6.366E-01	9.734E-01
Left precuneus	0.041	0.144	[-0.241 - 0.324]	7.751E-01	9.734E-01
Right precuneus	-0.107	0.144	[-0.389 - 0.175]	4.572E-01	9.734E-01
Left inferior temporal gyrus	0.130	0.145	[-0.153 - 0.414]	3.674E-01	9.734E-01
Right inferior temporal gyrus	0.166	0.145	[-0.117 - 0.450]	2.504E-01	9.734E-01
Left lateral orbitofrontal cortex	0.070	0.144	[-0.212 - 0.352]	6.279E-01	9.734E-01
Right lateral orbitofrontal cortex	0.183	0.144	[-0.100 - 0.466]	2.044E-01	9.734E-01
Left middle temporal gyrus	0.184	0.146	[-0.103 - 0.471]	2.082E-01	9.734E-01
Right middle temporal gyrus	0.018	0.145	[-0.265 - 0.302]	8.993E-01	9.824E-01
Left postcentral gyrus	-0.087	0.144	[-0.370 - 0.196]	5.477E-01	9.734E-01
Right postcentral gyrus	-0.011	0.144	[-0.294 - 0.272]	9.391E-01	9.824E-01
Left medial orbitofrontal cortex	-0.107	0.153	[-0.406 - 0.193]	4.851E-01	9.734E-01
Right medial orbitofrontal cortex	0.059	0.154	[-0.243 - 0.361]	7.033E-01	9.734E-01
Left cuneus	-0.163	0.144	[-0.446 - 0.120]	2.599E-01	9.734E-01
Right cuneus	-0.084	0.144	[-0.367 - 0.199]	5.596E-01	9.734E-01
Left pars triangularis of inferior frontal gyrus	-0.074	0.144	[-0.357 - 0.209]	6.083E-01	9.734E-01
Right pars triangularis of inferior frontal gyrus	-0.007	0.150	[-0.301 - 0.286]	9.604E-01	9.878E-01
Left superior parietal cortex	0.095	0.144	[-0.188 - 0.378]	5.091E-01	9.734E-01
Right superior parietal cortex	0.093	0.144	[-0.190 - 0.375]	5.189E-01	9.734E-01
Left pars opercularis of inferior frontal gyrus	0.011	0.144	[-0.272 - 0.293]	9.409E-01	9.824E-01
Right pars opercularis of inferior frontal gyrus	-0.159	0.144	[-0.442 - 0.124]	2.702E-01	9.734E-01
Left supramarginal gyrus	-0.012	0.144	[-0.295 - 0.271]	9.353E-01	9.824E-01
Right supramarginal gyrus	-0.029	0.145	[-0.312 - 0.255]	8.432E-01	9.814E-01
Left pericalcarine cortex	-0.140	0.197	[-0.527 - 0.246]	4.771E-01	9.734E-01
Right pericalcarine cortex	-0.168	0.145	[-0.451 - 0.116]	2.458E-01	9.734E-01
Left parahippocampal gyrus	-0.229	0.145	[-0.513 - 0.054]	1.129E-01	9.734E-01
Right parahippocampal gyrus	-0.258	0.145	[-0.543 - 0.026]	7.531E-02	9.734E-01
Left caudal middle frontal gyrus	-0.016	0.144	[-0.299 - 0.267]	9.108E-01	9.824E-01
Right caudal middle frontal gyrus	0.021	0.170	[-0.314 - 0.355]	9.039E-01	9.824E-01
Left transverse temporal gyrus	0.156	0.144	[-0.127 - 0.439]	2.810E-01	9.734E-01
Right transverse temporal gyrus	-0.172	0.145	[-0.455 - 0.112]	2.362E-01	9.734E-01
Left banks of superior temporal sulcus	0.030	0.145	[-0.253 - 0.313]	8.354E-01	9.814E-01
Right banks of superior temporal sulcus	0.105	0.145	[-0.179 - 0.388]	4.696E-01	9.734E-01
Left caudal anterior cingulate cortex	0.083	0.181	[-0.273 - 0.438]	6.483E-01	9.734E-01
Right caudal anterior cingulate cortex	0.159	0.144	[-0.124 - 0.442]	2.718E-01	9.734E-01
Left rostral anterior cingulate cortex	0.005	0.144	[-0.278 - 0.287]	9.739E-01	9.878E-01
Right rostral anterior cingulate cortex	0.014	0.145	[-0.269 - 0.298]	9.202E-01	9.824E-01
Left posterior cingulate cortex	0.051	0.144	[-0.231 - 0.334]	7.227E-01	9.734E-01
Right posterior cingulate cortex	0.202	0.145	[-0.081 - 0.485]	1.621E-01	9.734E-01
Left frontal pole	-0.124	0.174	[-0.464 - 0.216]	4.741E-01	9.734E-01
Right frontal pole	-0.538	0.146	[-0.824 - -0.251]	2.365E-04	1.679E-02
Left paracentral lobule	-0.072	0.194	[-0.453 - 0.308]	7.092E-01	9.734E-01
Right paracentral lobule	0.142	0.242	[-0.333 - 0.617]	5.581E-01	9.734E-01
Left insula	0.120	0.144	[-0.162 - 0.403]	4.048E-01	9.734E-01
Right insula	0.124	0.144	[-0.160 - 0.407]	3.922E-01	9.734E-01
Left entorhinal cortex	-0.094	0.145	[-0.377 - 0.189]	5.160E-01	9.734E-01
Right entorhinal cortex	-0.133	0.162	[-0.451 - 0.186]	4.135E-01	9.734E-01
Left temporal pole	-0.061	0.144	[-0.343 - 0.221]	6.734E-01	9.734E-01
Right temporal pole	-0.136	0.168	[-0.466 - 0.193]	4.171E-01	9.734E-01
Left isthmus cingulate cortex	0.001	0.145	[-0.283 - 0.284]	9.965E-01	9.965E-01
Right isthmus cingulate cortex	-0.100	0.145	[-0.383 - 0.184]	4.918E-01	9.734E-01

Supplementary Table S156. Cortical surface area differences between individuals with schizophrenia on both first-generation and second-generation antipsychotic medications and individuals with schizophrenia on second-generation antipsychotic medications controlling for age and sex

	d	Std. Err.	95% CI	*p*-value	FDR *q*-value
Total cortical surface area	0.089	0.082	[-0.071 - 0.250]	2.754E-01	7.723E-01
Left hemisphere	0.085	0.082	[-0.075 - 0.246]	2.968E-01	7.723E-01
Right hemisphere	0.093	0.082	[-0.068 - 0.253]	2.570E-01	7.723E-01
Left superior frontal gyrus	0.026	0.091	[-0.153 - 0.205]	7.756E-01	9.330E-01
Right superior frontal gyrus	0.071	0.101	[-0.127 - 0.268]	4.839E-01	9.026E-01
Left pars orbitalis of inferior frontal gyrus	0.114	0.082	[-0.046 - 0.274]	1.629E-01	7.723E-01
Right pars orbitalis of inferior frontal gyrus	0.170	0.087	[-0.001 - 0.342]	5.140E-02	7.299E-01
Left precentral gyrus	-0.019	0.088	[-0.192 - 0.154]	8.278E-01	9.330E-01
Right precentral gyrus	-0.037	0.082	[-0.197 - 0.123]	6.495E-01	9.330E-01
Left lingual gyrus	0.108	0.082	[-0.053 - 0.268]	1.877E-01	7.723E-01
Right lingual gyrus	0.127	0.082	[-0.033 - 0.287]	1.190E-01	7.723E-01
Left superior temporal gyrus	0.052	0.082	[-0.108 - 0.212]	5.263E-01	9.026E-01
Right superior temporal gyrus	0.107	0.102	[-0.092 - 0.307]	2.922E-01	7.723E-01
Left fusiform gyrus	0.013	0.122	[-0.227 - 0.252]	9.175E-01	9.593E-01
Right fusiform gyrus	0.057	0.128	[-0.194 - 0.309]	6.564E-01	9.330E-01
Left inferior parietal cortex	0.041	0.107	[-0.168 - 0.251]	6.989E-01	9.330E-01
Right inferior parietal cortex	0.024	0.105	[-0.180 - 0.229]	8.148E-01	9.330E-01
Left lateral occipital cortex	0.039	0.082	[-0.122 - 0.199]	6.372E-01	9.330E-01
Right lateral occipital cortex	0.082	0.082	[-0.078 - 0.242]	3.129E-01	7.723E-01
Left rostral middle frontal gyrus	0.124	0.082	[-0.036 - 0.284]	1.293E-01	7.723E-01
Right rostral middle frontal gyrus	0.010	0.082	[-0.150 - 0.170]	9.038E-01	9.593E-01
Left precuneus	0.049	0.084	[-0.114 - 0.213]	5.558E-01	9.026E-01
Right precuneus	-0.035	0.091	[-0.214 - 0.144]	7.052E-01	9.330E-01
Left inferior temporal gyrus	0.052	0.089	[-0.122 - 0.226]	5.594E-01	9.026E-01
Right inferior temporal gyrus	0.116	0.082	[-0.044 - 0.276]	1.556E-01	7.723E-01
Left lateral orbitofrontal cortex	0.107	0.082	[-0.053 - 0.268]	1.895E-01	7.723E-01
Right lateral orbitofrontal cortex	0.184	0.082	[0.023 - 0.345]	2.467E-02	6.936E-01
Left middle temporal gyrus	0.052	0.087	[-0.120 - 0.223]	5.527E-01	9.026E-01
Right middle temporal gyrus	0.013	0.082	[-0.147 - 0.174]	8.704E-01	9.593E-01
Left postcentral gyrus	0.054	0.084	[-0.111 - 0.219]	5.202E-01	9.026E-01
Right postcentral gyrus	0.054	0.082	[-0.106 - 0.214]	5.080E-01	9.026E-01
Left medial orbitofrontal cortex	0.134	0.082	[-0.026 - 0.294]	1.003E-01	7.723E-01
Right medial orbitofrontal cortex	0.131	0.087	[-0.041 - 0.302]	1.353E-01	7.723E-01
Left cuneus	0.021	0.082	[-0.139 - 0.181]	7.935E-01	9.330E-01
Right cuneus	0.083	0.082	[-0.077 - 0.243]	3.084E-01	7.723E-01
Left pars triangularis of inferior frontal gyrus	-0.005	0.088	[-0.177 - 0.167]	9.552E-01	9.617E-01
Right pars triangularis of inferior frontal gyrus	0.186	0.083	[0.023 - 0.349]	2.566E-02	6.936E-01
Left superior parietal cortex	0.090	0.082	[-0.070 - 0.250]	2.716E-01	7.723E-01
Right superior parietal cortex	0.142	0.082	[-0.018 - 0.303]	8.220E-02	7.676E-01
Left pars opercularis of inferior frontal gyrus	0.056	0.082	[-0.105 - 0.216]	4.962E-01	9.026E-01
Right pars opercularis of inferior frontal gyrus	0.052	0.175	[-0.290 - 0.394]	7.671E-01	9.330E-01
Left supramarginal gyrus	0.059	0.088	[-0.113 - 0.231]	4.989E-01	9.026E-01
Right supramarginal gyrus	0.087	0.091	[-0.091 - 0.265]	3.388E-01	7.984E-01
Left pericalcarine cortex	0.032	0.088	[-0.139 - 0.204]	7.120E-01	9.330E-01
Right pericalcarine cortex	0.095	0.082	[-0.065 - 0.255]	2.425E-01	7.723E-01
Left parahippocampal gyrus	0.061	0.149	[-0.231 - 0.353]	6.808E-01	9.330E-01
Right parahippocampal gyrus	0.027	0.096	[-0.161 - 0.214]	7.814E-01	9.330E-01
Left caudal middle frontal gyrus	0.076	0.116	[-0.151 - 0.303]	5.099E-01	9.026E-01
Right caudal middle frontal gyrus	0.113	0.092	[-0.067 - 0.293]	2.203E-01	7.723E-01
Left transverse temporal gyrus	0.030	0.106	[-0.178 - 0.237]	7.781E-01	9.330E-01
Right transverse temporal gyrus	0.024	0.107	[-0.186 - 0.234]	8.246E-01	9.330E-01
Left banks of superior temporal sulcus	-0.024	0.082	[-0.184 - 0.136]	7.694E-01	9.330E-01
Right banks of superior temporal sulcus	-0.008	0.092	[-0.188 - 0.172]	9.323E-01	9.593E-01
Left caudal anterior cingulate cortex	0.199	0.092	[0.020 - 0.379]	2.931E-02	6.936E-01
Right caudal anterior cingulate cortex	-0.009	0.082	[-0.169 - 0.151]	9.146E-01	9.593E-01
Left rostral anterior cingulate cortex	0.162	0.082	[0.002 - 0.323]	4.662E-02	7.299E-01
Right rostral anterior cingulate cortex	0.070	0.089	[-0.104 - 0.243]	4.321E-01	9.026E-01
Left posterior cingulate cortex	0.122	0.089	[-0.052 - 0.296]	1.697E-01	7.723E-01
Right posterior cingulate cortex	0.008	0.082	[-0.152 - 0.168]	9.231E-01	9.593E-01
Left frontal pole	-0.027	0.082	[-0.188 - 0.133]	7.374E-01	9.330E-01
Right frontal pole	-0.141	0.082	[-0.301 - 0.019]	8.497E-02	7.676E-01
Left paracentral lobule	0.091	0.091	[-0.087 - 0.268]	3.154E-01	7.723E-01
Right paracentral lobule	0.140	0.082	[-0.020 - 0.301]	8.649E-02	7.676E-01
Left insula	0.077	0.082	[-0.084 - 0.237]	3.486E-01	7.984E-01
Right insula	0.040	0.101	[-0.158 - 0.239]	6.901E-01	9.330E-01
Left entorhinal cortex	0.114	0.095	[-0.072 - 0.300]	2.311E-01	7.723E-01
Right entorhinal cortex	-0.071	0.082	[-0.231 - 0.089]	3.825E-01	8.487E-01
Left temporal pole	0.031	0.100	[-0.166 - 0.228]	7.561E-01	9.330E-01
Right temporal pole	-0.055	0.082	[-0.215 - 0.105]	4.989E-01	9.026E-01
Left isthmus cingulate cortex	-0.116	0.111	[-0.333 - 0.102]	2.974E-01	7.723E-01
Right isthmus cingulate cortex	-0.005	0.102	[-0.205 - 0.195]	9.617E-01	9.617E-01

Supplementary Table S157. Cortical surface area differences between individuals with schizophrenia on both first-generation and second-generation antipsychotic medications and individuals with schizophrenia on first-generation antipsychotic medications controlling for age and sex

	d	Std. Err.	95% CI	*p*-value	FDR *q*-value
Total cortical surface area	0.023	0.144	[-0.259 - 0.304]	8.744E-01	9.666E-01
Left hemisphere	0.027	0.144	[-0.254 - 0.309]	8.503E-01	9.652E-01
Right hemisphere	0.018	0.144	[-0.263 - 0.300]	8.977E-01	9.666E-01
Left superior frontal gyrus	0.052	0.153	[-0.247 - 0.352]	7.329E-01	9.652E-01
Right superior frontal gyrus	0.035	0.144	[-0.246 - 0.317]	8.057E-01	9.652E-01
Left pars orbitalis of inferior frontal gyrus	-0.046	0.143	[-0.327 - 0.235]	7.472E-01	9.652E-01
Right pars orbitalis of inferior frontal gyrus	-0.063	0.144	[-0.345 - 0.219]	6.616E-01	9.652E-01
Left precentral gyrus	-0.212	0.226	[-0.655 - 0.230]	3.465E-01	9.652E-01
Right precentral gyrus	0.026	0.144	[-0.255 - 0.308]	8.540E-01	9.652E-01
Left lingual gyrus	0.075	0.144	[-0.206 - 0.357]	6.000E-01	9.652E-01
Right lingual gyrus	0.009	0.143	[-0.271 - 0.290]	9.474E-01	9.666E-01
Left superior temporal gyrus	-0.077	0.144	[-0.359 - 0.204]	5.909E-01	9.652E-01
Right superior temporal gyrus	0.033	0.144	[-0.249 - 0.314]	8.210E-01	9.652E-01
Left fusiform gyrus	0.012	0.144	[-0.270 - 0.293]	9.354E-01	9.666E-01
Right fusiform gyrus	-0.166	0.220	[-0.597 - 0.265]	4.509E-01	9.652E-01
Left inferior parietal cortex	0.142	0.144	[-0.140 - 0.424]	3.243E-01	9.652E-01
Right inferior parietal cortex	-0.242	0.273	[-0.778 - 0.294]	3.758E-01	9.652E-01
Left lateral occipital cortex	0.121	0.143	[-0.160 - 0.402]	3.992E-01	9.652E-01
Right lateral occipital cortex	0.146	0.144	[-0.137 - 0.428]	3.119E-01	9.652E-01
Left rostral middle frontal gyrus	0.053	0.143	[-0.228 - 0.334]	7.105E-01	9.652E-01
Right rostral middle frontal gyrus	-0.026	0.143	[-0.306 - 0.255]	8.565E-01	9.652E-01
Left precuneus	-0.057	0.234	[-0.517 - 0.402]	8.076E-01	9.652E-01
Right precuneus	-0.055	0.165	[-0.379 - 0.269]	7.378E-01	9.652E-01
Left inferior temporal gyrus	-0.033	0.144	[-0.315 - 0.248]	8.170E-01	9.652E-01
Right inferior temporal gyrus	0.191	0.144	[-0.091 - 0.473]	1.836E-01	9.652E-01
Left lateral orbitofrontal cortex	-0.039	0.181	[-0.392 - 0.315]	8.309E-01	9.652E-01
Right lateral orbitofrontal cortex	0.094	0.144	[-0.188 - 0.376]	5.127E-01	9.652E-01
Left middle temporal gyrus	-0.116	0.209	[-0.527 - 0.294]	5.789E-01	9.652E-01
Right middle temporal gyrus	-0.202	0.210	[-0.614 - 0.210]	3.371E-01	9.652E-01
Left postcentral gyrus	0.070	0.144	[-0.212 - 0.352]	6.262E-01	9.652E-01
Right postcentral gyrus	-0.145	0.188	[-0.513 - 0.224]	4.419E-01	9.652E-01
Left medial orbitofrontal cortex	0.092	0.201	[-0.302 - 0.486]	6.478E-01	9.652E-01
Right medial orbitofrontal cortex	0.152	0.144	[-0.129 - 0.433]	2.898E-01	9.652E-01
Left cuneus	-0.166	0.144	[-0.448 - 0.116]	2.483E-01	9.652E-01
Right cuneus	0.057	0.143	[-0.224 - 0.337]	6.931E-01	9.652E-01
Left pars triangularis of inferior frontal gyrus	0.064	0.144	[-0.218 - 0.345]	6.574E-01	9.652E-01
Right pars triangularis of inferior frontal gyrus	0.224	0.144	[-0.059 - 0.506]	1.203E-01	9.652E-01
Left superior parietal cortex	-0.110	0.144	[-0.391 - 0.172]	4.448E-01	9.652E-01
Right superior parietal cortex	0.087	0.144	[-0.195 - 0.368]	5.464E-01	9.652E-01
Left pars opercularis of inferior frontal gyrus	-0.058	0.216	[-0.481 - 0.365]	7.880E-01	9.652E-01
Right pars opercularis of inferior frontal gyrus	0.164	0.227	[-0.280 - 0.609]	4.695E-01	9.652E-01
Left supramarginal gyrus	-0.005	0.144	[-0.287 - 0.276]	9.696E-01	9.696E-01
Right supramarginal gyrus	-0.146	0.143	[-0.427 - 0.135]	3.094E-01	9.652E-01
Left pericalcarine cortex	0.123	0.144	[-0.158 - 0.405]	3.898E-01	9.652E-01
Right pericalcarine cortex	0.151	0.143	[-0.130 - 0.432]	2.929E-01	9.652E-01
Left parahippocampal gyrus	-0.087	0.144	[-0.369 - 0.194]	5.433E-01	9.652E-01
Right parahippocampal gyrus	-0.012	0.143	[-0.293 - 0.269]	9.312E-01	9.666E-01
Left caudal middle frontal gyrus	0.170	0.174	[-0.172 - 0.511]	3.301E-01	9.652E-01
Right caudal middle frontal gyrus	0.017	0.175	[-0.326 - 0.360]	9.232E-01	9.666E-01
Left transverse temporal gyrus	-0.027	0.143	[-0.308 - 0.254]	8.503E-01	9.652E-01
Right transverse temporal gyrus	-0.052	0.189	[-0.422 - 0.318]	7.819E-01	9.652E-01
Left banks of superior temporal sulcus	-0.190	0.268	[-0.715 - 0.335]	4.786E-01	9.652E-01
Right banks of superior temporal sulcus	-0.120	0.247	[-0.605 - 0.365]	6.281E-01	9.652E-01
Left caudal anterior cingulate cortex	0.297	0.144	[0.014 - 0.580]	3.964E-02	9.652E-01
Right caudal anterior cingulate cortex	0.008	0.143	[-0.273 - 0.290]	9.529E-01	9.666E-01
Left rostral anterior cingulate cortex	0.259	0.144	[-0.023 - 0.541]	7.165E-02	9.652E-01
Right rostral anterior cingulate cortex	0.215	0.144	[-0.067 - 0.498]	1.354E-01	9.652E-01
Left posterior cingulate cortex	-0.110	0.143	[-0.391 - 0.171]	4.414E-01	9.652E-01
Right posterior cingulate cortex	-0.125	0.207	[-0.531 - 0.281]	5.454E-01	9.652E-01
Left frontal pole	-0.097	0.144	[-0.379 - 0.184]	4.981E-01	9.652E-01
Right frontal pole	-0.278	0.170	[-0.611 - 0.054]	1.013E-01	9.652E-01
Left paracentral lobule	-0.081	0.254	[-0.580 - 0.418]	7.499E-01	9.652E-01
Right paracentral lobule	0.225	0.144	[-0.057 - 0.507]	1.180E-01	9.652E-01
Left insula	0.085	0.143	[-0.196 - 0.366]	5.525E-01	9.652E-01
Right insula	-0.064	0.224	[-0.503 - 0.375]	7.750E-01	9.652E-01
Left entorhinal cortex	-0.072	0.143	[-0.354 - 0.209]	6.138E-01	9.652E-01
Right entorhinal cortex	-0.145	0.144	[-0.427 - 0.137]	3.131E-01	9.652E-01
Left temporal pole	-0.062	0.144	[-0.344 - 0.219]	6.637E-01	9.652E-01
Right temporal pole	-0.149	0.211	[-0.562 - 0.264]	4.804E-01	9.652E-01
Left isthmus cingulate cortex	-0.148	0.188	[-0.517 - 0.221]	4.328E-01	9.652E-01
Right isthmus cingulate cortex	-0.133	0.143	[-0.414 - 0.148]	3.540E-01	9.652E-01

Supplementary Table S158. Partial correlations between cortical surface area and chlorpromazine equivalents controlling for age and sex in individuals with schizophrenia who are medicated with antipsychotics

	Partial *R*	Std. Err.	95% CI	*p*-value	FDR *q*-value
Total cortical surface area	-0.009	0.037	[-0.081 - 0.064]	8.152E-01	9.797E-01
Left hemisphere	-0.005	0.034	[-0.072 - 0.062]	8.899E-01	9.797E-01
Right hemisphere	-0.013	0.040	[-0.091 - 0.065]	7.407E-01	9.797E-01
Left superior frontal gyrus	-0.021	0.033	[-0.085 - 0.043]	5.149E-01	9.797E-01
Right superior frontal gyrus	0.011	0.036	[-0.060 - 0.081]	7.672E-01	9.797E-01
Left pars orbitalis of inferior frontal gyrus	-0.024	0.040	[-0.103 - 0.055]	5.491E-01	9.797E-01
Right pars orbitalis of inferior frontal gyrus	0.044	0.032	[-0.019 - 0.106]	1.731E-01	9.797E-01
Left precentral gyrus	-0.046	0.032	[-0.109 - 0.017]	1.505E-01	9.797E-01
Right precentral gyrus	0.014	0.032	[-0.049 - 0.077]	6.618E-01	9.797E-01
Left lingual gyrus	-0.002	0.038	[-0.077 - 0.072]	9.521E-01	9.797E-01
Right lingual gyrus	0.060	0.032	[-0.003 - 0.123]	6.037E-02	9.797E-01
Left superior temporal gyrus	-0.021	0.033	[-0.085 - 0.043]	5.252E-01	9.797E-01
Right superior temporal gyrus	-0.017	0.041	[-0.097 - 0.063]	6.760E-01	9.797E-01
Left fusiform gyrus	0.048	0.036	[-0.022 - 0.118]	1.764E-01	9.797E-01
Right fusiform gyrus	0.013	0.032	[-0.050 - 0.076]	6.858E-01	9.797E-01
Left inferior parietal cortex	-0.032	0.047	[-0.124 - 0.060]	4.904E-01	9.797E-01
Right inferior parietal cortex	-0.093	0.048	[-0.186 - 0.001]	5.264E-02	9.797E-01
Left lateral occipital cortex	-0.019	0.034	[-0.086 - 0.047]	5.701E-01	9.797E-01
Right lateral occipital cortex	-0.015	0.032	[-0.078 - 0.049]	6.484E-01	9.797E-01
Left rostral middle frontal gyrus	0.007	0.032	[-0.056 - 0.069]	8.348E-01	9.797E-01
Right rostral middle frontal gyrus	-0.040	0.034	[-0.106 - 0.027]	2.422E-01	9.797E-01
Left precuneus	0.031	0.040	[-0.047 - 0.109]	4.358E-01	9.797E-01
Right precuneus	-0.005	0.039	[-0.081 - 0.071]	8.942E-01	9.797E-01
Left inferior temporal gyrus	0.005	0.032	[-0.058 - 0.068]	8.798E-01	9.797E-01
Right inferior temporal gyrus	0.008	0.033	[-0.056 - 0.072]	8.021E-01	9.797E-01
Left lateral orbitofrontal cortex	-0.013	0.032	[-0.076 - 0.050]	6.791E-01	9.797E-01
Right lateral orbitofrontal cortex	0.026	0.032	[-0.037 - 0.088]	4.224E-01	9.797E-01
Left middle temporal gyrus	-0.007	0.045	[-0.095 - 0.080]	8.716E-01	9.797E-01
Right middle temporal gyrus	-0.035	0.051	[-0.135 - 0.064]	4.887E-01	9.797E-01
Left postcentral gyrus	-0.027	0.044	[-0.113 - 0.059]	5.359E-01	9.797E-01
Right postcentral gyrus	-0.002	0.032	[-0.065 - 0.060]	9.413E-01	9.797E-01
Left medial orbitofrontal cortex	0.013	0.042	[-0.070 - 0.095]	7.612E-01	9.797E-01
Right medial orbitofrontal cortex	-0.024	0.039	[-0.101 - 0.052]	5.327E-01	9.797E-01
Left cuneus	-0.001	0.032	[-0.064 - 0.062]	9.790E-01	9.930E-01
Right cuneus	-0.010	0.041	[-0.091 - 0.070]	8.002E-01	9.797E-01
Left pars triangularis of inferior frontal gyrus	-0.049	0.041	[-0.129 - 0.032]	2.358E-01	9.797E-01
Right pars triangularis of inferior frontal gyrus	-0.025	0.032	[-0.087 - 0.038]	4.395E-01	9.797E-01
Left superior parietal cortex	0.040	0.035	[-0.028 - 0.109]	2.486E-01	9.797E-01
Right superior parietal cortex	0.011	0.043	[-0.074 - 0.095]	8.065E-01	9.797E-01
Left pars opercularis of inferior frontal gyrus	-0.004	0.032	[-0.066 - 0.059]	9.127E-01	9.797E-01
Right pars opercularis of inferior frontal gyrus	-0.041	0.037	[-0.114 - 0.032]	2.677E-01	9.797E-01
Left supramarginal gyrus	0.000	0.032	[-0.063 - 0.063]	9.982E-01	9.982E-01
Right supramarginal gyrus	0.037	0.042	[-0.046 - 0.120]	3.855E-01	9.797E-01
Left pericalcarine cortex	0.016	0.032	[-0.047 - 0.079]	6.173E-01	9.797E-01
Right pericalcarine cortex	0.022	0.032	[-0.041 - 0.085]	4.937E-01	9.797E-01
Left parahippocampal gyrus	0.007	0.045	[-0.080 - 0.094]	8.739E-01	9.797E-01
Right parahippocampal gyrus	-0.005	0.038	[-0.079 - 0.068]	8.893E-01	9.797E-01
Left caudal middle frontal gyrus	-0.030	0.032	[-0.093 - 0.033]	3.464E-01	9.797E-01
Right caudal middle frontal gyrus	-0.033	0.033	[-0.098 - 0.032]	3.218E-01	9.797E-01
Left transverse temporal gyrus	-0.049	0.039	[-0.125 - 0.027]	2.091E-01	9.797E-01
Right transverse temporal gyrus	-0.003	0.043	[-0.087 - 0.081]	9.404E-01	9.797E-01
Left banks of superior temporal sulcus	-0.006	0.044	[-0.092 - 0.080]	8.980E-01	9.797E-01
Right banks of superior temporal sulcus	0.007	0.034	[-0.061 - 0.074]	8.411E-01	9.797E-01
Left caudal anterior cingulate cortex	0.005	0.043	[-0.079 - 0.088]	9.157E-01	9.797E-01
Right caudal anterior cingulate cortex	-0.057	0.034	[-0.124 - 0.010]	9.360E-02	9.797E-01
Left rostral anterior cingulate cortex	0.007	0.033	[-0.059 - 0.073]	8.338E-01	9.797E-01
Right rostral anterior cingulate cortex	0.057	0.032	[-0.005 - 0.120]	7.269E-02	9.797E-01
Left posterior cingulate cortex	0.017	0.033	[-0.047 - 0.081]	6.117E-01	9.797E-01
Right posterior cingulate cortex	-0.041	0.049	[-0.137 - 0.054]	3.958E-01	9.797E-01
Left frontal pole	-0.084	0.053	[-0.189 - 0.020]	1.137E-01	9.797E-01
Right frontal pole	-0.073	0.040	[-0.152 - 0.006]	7.092E-02	9.797E-01
Left paracentral lobule	-0.072	0.040	[-0.150 - 0.006]	7.007E-02	9.797E-01
Right paracentral lobule	-0.016	0.043	[-0.099 - 0.068]	7.120E-01	9.797E-01
Left insula	-0.007	0.039	[-0.083 - 0.068]	8.513E-01	9.797E-01
Right insula	0.012	0.053	[-0.092 - 0.117]	8.158E-01	9.797E-01
Left entorhinal cortex	-0.011	0.038	[-0.085 - 0.063]	7.711E-01	9.797E-01
Right entorhinal cortex	-0.040	0.040	[-0.119 - 0.039]	3.259E-01	9.797E-01
Left temporal pole	-0.035	0.032	[-0.098 - 0.028]	2.757E-01	9.797E-01
Right temporal pole	-0.033	0.032	[-0.096 - 0.029]	2.985E-01	9.797E-01
Left isthmus cingulate cortex	0.006	0.048	[-0.088 - 0.099]	9.077E-01	9.797E-01
Right isthmus cingulate cortex	0.031	0.037	[-0.042 - 0.103]	4.074E-01	9.797E-01

Supplementary Table S159. Cortical surface area differences between individuals with bipolar disorder taking lithium carbonate and individuals with bipolar disorder not taking lithium carbonate controlling for age, sex, and other medications

	d	Std. Err.	95% CI	*p*-value	FDR *q*-value
Total cortical surface area	-0.240	0.142	[-0.519 - 0.039]	9.156E-02	3.824E-01
Left hemisphere	-0.252	0.142	[-0.531 - 0.027]	7.673E-02	3.824E-01
Right hemisphere	-0.227	0.142	[-0.506 - 0.052]	1.105E-01	4.130E-01
Left superior frontal gyrus	-0.268	0.142	[-0.546 - 0.011]	5.943E-02	3.824E-01
Right superior frontal gyrus	0.141	0.156	[-0.165 - 0.447]	3.664E-01	6.915E-01
Left pars orbitalis of inferior frontal gyrus	-0.109	0.142	[-0.388 - 0.169]	4.407E-01	7.131E-01
Right pars orbitalis of inferior frontal gyrus	-0.068	0.142	[-0.346 - 0.211]	6.344E-01	9.009E-01
Left precentral gyrus	-0.143	0.206	[-0.546 - 0.261]	4.879E-01	7.513E-01
Right precentral gyrus	-0.308	0.187	[-0.674 - 0.057]	9.830E-02	3.877E-01
Left lingual gyrus	-0.009	0.184	[-0.369 - 0.351]	9.620E-01	9.757E-01
Right lingual gyrus	-0.186	0.142	[-0.464 - 0.093]	1.910E-01	5.929E-01
Left superior temporal gyrus	-0.539	0.203	[-0.937 - -0.141]	7.882E-03	2.202E-01
Right superior temporal gyrus	-0.226	0.240	[-0.697 - 0.245]	3.473E-01	6.850E-01
Left fusiform gyrus	-0.009	0.142	[-0.287 - 0.268]	9.477E-01	9.757E-01
Right fusiform gyrus	-0.249	0.204	[-0.649 - 0.152]	2.237E-01	6.354E-01
Left inferior parietal cortex	0.007	0.142	[-0.270 - 0.285]	9.581E-01	9.757E-01
Right inferior parietal cortex	-0.056	0.142	[-0.334 - 0.223]	6.960E-01	9.369E-01
Left lateral occipital cortex	-0.097	0.142	[-0.375 - 0.182]	4.969E-01	7.513E-01
Right lateral occipital cortex	-0.010	0.142	[-0.288 - 0.268]	9.424E-01	9.757E-01
Left rostral middle frontal gyrus	-0.189	0.151	[-0.486 - 0.108]	2.115E-01	6.258E-01
Right rostral middle frontal gyrus	-0.141	0.183	[-0.499 - 0.218]	4.419E-01	7.131E-01
Left precuneus	-0.112	0.207	[-0.518 - 0.294]	5.883E-01	8.702E-01
Right precuneus	-0.026	0.185	[-0.388 - 0.336]	8.868E-01	9.686E-01
Left inferior temporal gyrus	-0.074	0.171	[-0.409 - 0.260]	6.628E-01	9.227E-01
Right inferior temporal gyrus	-0.165	0.142	[-0.444 - 0.113]	2.445E-01	6.375E-01
Left lateral orbitofrontal cortex	-0.292	0.158	[-0.601 - 0.018]	6.459E-02	3.824E-01
Right lateral orbitofrontal cortex	-0.163	0.142	[-0.441 - 0.115]	2.514E-01	6.375E-01
Left middle temporal gyrus	0.047	0.142	[-0.232 - 0.326]	7.411E-01	9.369E-01
Right middle temporal gyrus	-0.185	0.142	[-0.464 - 0.093]	1.921E-01	5.929E-01
Left postcentral gyrus	-0.259	0.150	[-0.552 - 0.034]	8.362E-02	3.824E-01
Right postcentral gyrus	0.109	0.160	[-0.205 - 0.422]	4.973E-01	7.513E-01
Left medial orbitofrontal cortex	-0.250	0.142	[-0.529 - 0.028]	7.830E-02	3.824E-01
Right medial orbitofrontal cortex	-0.292	0.143	[-0.572 - -0.012]	4.095E-02	3.664E-01
Left cuneus	-0.056	0.189	[-0.427 - 0.314]	7.658E-01	9.369E-01
Right cuneus	-0.308	0.143	[-0.587 - -0.028]	3.122E-02	3.664E-01
Left pars triangularis of inferior frontal gyrus	0.123	0.142	[-0.155 - 0.401]	3.861E-01	6.915E-01
Right pars triangularis of inferior frontal gyrus	0.292	0.143	[0.012 - 0.572]	4.129E-02	3.664E-01
Left superior parietal cortex	-0.397	0.144	[-0.679 - -0.116]	5.653E-03	2.202E-01
Right superior parietal cortex	-0.578	0.222	[-1.013 - -0.142]	9.306E-03	2.202E-01
Left pars opercularis of inferior frontal gyrus	0.135	0.142	[-0.144 - 0.413]	3.429E-01	6.850E-01
Right pars opercularis of inferior frontal gyrus	-0.070	0.185	[-0.434 - 0.293]	7.040E-01	9.369E-01
Left supramarginal gyrus	-0.119	0.142	[-0.396 - 0.159]	4.029E-01	6.915E-01
Right supramarginal gyrus	-0.019	0.263	[-0.534 - 0.495]	9.409E-01	9.757E-01
Left pericalcarine cortex	-0.184	0.181	[-0.540 - 0.171]	3.100E-01	6.846E-01
Right pericalcarine cortex	-0.318	0.143	[-0.598 - -0.038]	2.591E-02	3.664E-01
Left parahippocampal gyrus	0.073	0.142	[-0.205 - 0.350]	6.083E-01	8.814E-01
Right parahippocampal gyrus	-0.003	0.142	[-0.282 - 0.275]	9.810E-01	9.810E-01
Left caudal middle frontal gyrus	-0.169	0.142	[-0.447 - 0.110]	2.351E-01	6.375E-01
Right caudal middle frontal gyrus	-0.159	0.192	[-0.535 - 0.217]	4.080E-01	6.915E-01
Left transverse temporal gyrus	-0.461	0.195	[-0.843 - -0.079]	1.790E-02	3.178E-01
Right transverse temporal gyrus	-0.033	0.175	[-0.375 - 0.310]	8.518E-01	9.450E-01
Left banks of superior temporal sulcus	0.183	0.174	[-0.158 - 0.525]	2.927E-01	6.846E-01
Right banks of superior temporal sulcus	-0.035	0.142	[-0.313 - 0.243]	8.050E-01	9.369E-01
Left caudal anterior cingulate cortex	-0.124	0.142	[-0.403 - 0.154]	3.819E-01	6.915E-01
Right caudal anterior cingulate cortex	-0.240	0.142	[-0.519 - 0.039]	9.121E-02	3.824E-01
Left rostral anterior cingulate cortex	-0.082	0.257	[-0.586 - 0.421]	7.480E-01	9.369E-01
Right rostral anterior cingulate cortex	-0.117	0.142	[-0.395 - 0.161]	4.090E-01	6.915E-01
Left posterior cingulate cortex	-0.028	0.142	[-0.306 - 0.250]	8.427E-01	9.450E-01
Right posterior cingulate cortex	-0.159	0.142	[-0.437 - 0.120]	2.646E-01	6.479E-01
Left frontal pole	0.154	0.152	[-0.144 - 0.452]	3.121E-01	6.846E-01
Right frontal pole	-0.042	0.185	[-0.404 - 0.320]	8.198E-01	9.388E-01
Left paracentral lobule	-0.313	0.161	[-0.629 - 0.003]	5.245E-02	3.824E-01
Right paracentral lobule	-0.151	0.152	[-0.449 - 0.146]	3.182E-01	6.846E-01
Left insula	-0.250	0.172	[-0.587 - 0.087]	1.461E-01	4.938E-01
Right insula	-0.247	0.143	[-0.527 - 0.032]	8.290E-02	3.824E-01
Left entorhinal cortex	-0.041	0.148	[-0.331 - 0.249]	7.805E-01	9.369E-01
Right entorhinal cortex	0.051	0.204	[-0.349 - 0.451]	8.031E-01	9.369E-01
Left temporal pole	-0.086	0.303	[-0.681 - 0.509]	7.767E-01	9.369E-01
Right temporal pole	-0.172	0.181	[-0.528 - 0.183]	3.410E-01	6.850E-01
Left isthmus cingulate cortex	-0.084	0.231	[-0.537 - 0.368]	7.150E-01	9.369E-01
Right isthmus cingulate cortex	0.228	0.157	[-0.079 - 0.535]	1.458E-01	4.938E-01

Supplementary Table S160. Cortical surface area differences between individuals with bipolar disorder taking lithium carbonate and individuals with bipolar disorder not taking lithium carbonate controlling for age, sex, age × sex, age^2, age^2 × sex, ICV, and other medications at 25 years of age or older

	d	Std. Err.	95% CI	*p*-value	FDR *q*-value
Total cortical surface area	0.092	0.344	[-0.583 - 0.767]	7.895E-01	9.292E-01
Left hemisphere	-0.026	0.276	[-0.567 - 0.515]	9.247E-01	9.799E-01
Right hemisphere	0.153	0.402	[-0.634 - 0.940]	7.024E-01	9.292E-01
Left superior frontal gyrus	-0.004	0.285	[-0.562 - 0.555]	9.897E-01	9.897E-01
Right superior frontal gyrus	0.119	0.243	[-0.358 - 0.596]	6.235E-01	9.292E-01
Left pars orbitalis of inferior frontal gyrus	0.088	0.375	[-0.647 - 0.823]	8.146E-01	9.292E-01
Right pars orbitalis of inferior frontal gyrus	0.101	0.390	[-0.663 - 0.864]	7.963E-01	9.292E-01
Left precentral gyrus	0.070	0.148	[-0.220 - 0.361]	6.351E-01	9.292E-01
Right precentral gyrus	-0.110	0.173	[-0.449 - 0.229]	5.239E-01	9.292E-01
Left lingual gyrus	-0.070	0.215	[-0.491 - 0.351]	7.439E-01	9.292E-01
Right lingual gyrus	-0.230	0.228	[-0.676 - 0.217]	3.138E-01	8.720E-01
Left superior temporal gyrus	-0.346	0.234	[-0.804 - 0.113]	1.393E-01	7.033E-01
Right superior temporal gyrus	0.075	0.277	[-0.467 - 0.618]	7.850E-01	9.292E-01
Left fusiform gyrus	0.144	0.208	[-0.263 - 0.551]	4.881E-01	9.292E-01
Right fusiform gyrus	-0.105	0.149	[-0.397 - 0.188]	4.825E-01	9.292E-01
Left inferior parietal cortex	0.049	0.203	[-0.348 - 0.447]	8.073E-01	9.292E-01
Right inferior parietal cortex	0.452	0.251	[-0.041 - 0.944]	7.211E-02	5.732E-01
Left lateral occipital cortex	-0.246	0.199	[-0.637 - 0.145]	2.173E-01	7.714E-01
Right lateral occipital cortex	-0.336	0.238	[-0.802 - 0.131]	1.585E-01	7.033E-01
Left rostral middle frontal gyrus	-0.124	0.276	[-0.665 - 0.418]	6.549E-01	9.292E-01
Right rostral middle frontal gyrus	-0.481	0.193	[-0.859 - -0.103]	1.262E-02	4.479E-01
Left precuneus	-0.478	0.434	[-1.328 - 0.373]	2.708E-01	8.429E-01
Right precuneus	0.073	0.322	[-0.558 - 0.704]	8.201E-01	9.292E-01
Left inferior temporal gyrus	-0.026	0.446	[-0.899 - 0.848]	9.539E-01	9.897E-01
Right inferior temporal gyrus	-0.264	0.241	[-0.737 - 0.208]	2.730E-01	8.429E-01
Left lateral orbitofrontal cortex	0.117	0.368	[-0.604 - 0.838]	7.508E-01	9.292E-01
Right lateral orbitofrontal cortex	0.051	0.228	[-0.396 - 0.498]	8.245E-01	9.292E-01
Left middle temporal gyrus	0.058	0.154	[-0.244 - 0.360]	7.078E-01	9.292E-01
Right middle temporal gyrus	-0.005	0.314	[-0.619 - 0.610]	9.878E-01	9.897E-01
Left postcentral gyrus	-0.469	0.263	[-0.984 - 0.046]	7.419E-02	5.732E-01
Right postcentral gyrus	0.609	0.332	[-0.042 - 1.259]	6.653E-02	5.732E-01
Left medial orbitofrontal cortex	0.102	0.248	[-0.383 - 0.587]	6.797E-01	9.292E-01
Right medial orbitofrontal cortex	-0.177	0.266	[-0.699 - 0.344]	5.051E-01	9.292E-01
Left cuneus	-0.432	0.445	[-1.303 - 0.440]	3.316E-01	8.720E-01
Right cuneus	-0.575	0.312	[-1.186 - 0.036]	6.497E-02	5.732E-01
Left pars triangularis of inferior frontal gyrus	0.089	0.285	[-0.469 - 0.647]	7.535E-01	9.292E-01
Right pars triangularis of inferior frontal gyrus	0.449	0.361	[-0.259 - 1.158]	2.138E-01	7.714E-01
Left superior parietal cortex	0.251	0.454	[-0.638 - 1.141]	5.799E-01	9.292E-01
Right superior parietal cortex	-0.615	0.283	[-1.169 - -0.060]	2.973E-02	5.732E-01
Left pars opercularis of inferior frontal gyrus	0.231	0.204	[-0.169 - 0.631]	2.577E-01	8.429E-01
Right pars opercularis of inferior frontal gyrus	0.401	0.319	[-0.223 - 1.026]	2.080E-01	7.714E-01
Left supramarginal gyrus	0.078	0.204	[-0.320 - 0.477]	7.000E-01	9.292E-01
Right supramarginal gyrus	-0.072	0.266	[-0.593 - 0.450]	7.879E-01	9.292E-01
Left pericalcarine cortex	-0.318	0.319	[-0.943 - 0.306]	3.175E-01	8.720E-01
Right pericalcarine cortex	-0.499	0.151	[-0.795 - -0.204]	9.108E-04	6.467E-02
Left parahippocampal gyrus	0.155	0.216	[-0.268 - 0.578]	4.721E-01	9.292E-01
Right parahippocampal gyrus	0.065	0.216	[-0.357 - 0.488]	7.614E-01	9.292E-01
Left caudal middle frontal gyrus	-0.142	0.269	[-0.668 - 0.385]	5.982E-01	9.292E-01
Right caudal middle frontal gyrus	0.126	0.269	[-0.400 - 0.653]	6.381E-01	9.292E-01
Left transverse temporal gyrus	-0.048	0.361	[-0.756 - 0.660]	8.943E-01	9.620E-01
Right transverse temporal gyrus	0.438	0.294	[-0.138 - 1.013]	1.362E-01	7.033E-01
Left banks of superior temporal sulcus	0.537	0.322	[-0.094 - 1.169]	9.512E-02	6.139E-01
Right banks of superior temporal sulcus	0.460	0.244	[-0.019 - 0.938]	5.955E-02	5.732E-01
Left caudal anterior cingulate cortex	-0.132	0.236	[-0.594 - 0.330]	5.751E-01	9.292E-01
Right caudal anterior cingulate cortex	-0.171	0.172	[-0.508 - 0.166]	3.204E-01	8.720E-01
Left rostral anterior cingulate cortex	-0.122	0.301	[-0.711 - 0.468]	6.851E-01	9.292E-01
Right rostral anterior cingulate cortex	0.108	0.154	[-0.194 - 0.410]	4.843E-01	9.292E-01
Left posterior cingulate cortex	-0.034	0.210	[-0.446 - 0.379]	8.726E-01	9.617E-01
Right posterior cingulate cortex	-0.101	0.160	[-0.414 - 0.212]	5.277E-01	9.292E-01
Left frontal pole	0.537	0.307	[-0.066 - 1.139]	8.073E-02	5.732E-01
Right frontal pole	-0.169	0.333	[-0.822 - 0.484]	6.127E-01	9.292E-01
Left paracentral lobule	-0.143	0.435	[-0.996 - 0.709]	7.417E-01	9.292E-01
Right paracentral lobule	0.411	0.318	[-0.212 - 1.034]	1.955E-01	7.714E-01
Left insula	-0.197	0.442	[-1.063 - 0.669]	6.559E-01	9.292E-01
Right insula	0.491	0.345	[-0.184 - 1.167]	1.541E-01	7.033E-01
Left entorhinal cortex	0.188	0.229	[-0.262 - 0.637]	4.128E-01	9.292E-01
Right entorhinal cortex	-0.051	0.339	[-0.715 - 0.613]	8.804E-01	9.617E-01
Left temporal pole	-0.134	0.399	[-0.916 - 0.648]	7.367E-01	9.292E-01
Right temporal pole	0.004	0.232	[-0.451 - 0.459]	9.860E-01	9.897E-01
Left isthmus cingulate cortex	-0.741	0.364	[-1.454 - -0.027]	4.180E-02	5.732E-01
Right isthmus cingulate cortex	0.630	0.409	[-0.172 - 1.432]	1.234E-01	7.033E-01

Supplementary Table S161. Cortical surface area differences between individuals with bipolar disorder taking lithium carbonate and individuals with bipolar disorder not taking lithium carbonate controlling for age, sex, and other medications at 25 years of age or older

	d	Std. Err.	95% CI	*p*-value	FDR *q*-value
Total cortical surface area	-0.203	0.148	[-0.493 - 0.087]	1.697E-01	5.476E-01
Left hemisphere	-0.219	0.148	[-0.509 - 0.071]	1.389E-01	5.201E-01
Right hemisphere	-0.187	0.148	[-0.477 - 0.103]	2.072E-01	6.129E-01
Left superior frontal gyrus	-0.274	0.171	[-0.609 - 0.060]	1.077E-01	5.201E-01
Right superior frontal gyrus	0.017	0.221	[-0.416 - 0.451]	9.377E-01	9.696E-01
Left pars orbitalis of inferior frontal gyrus	-0.073	0.150	[-0.368 - 0.222]	6.271E-01	8.096E-01
Right pars orbitalis of inferior frontal gyrus	0.007	0.185	[-0.355 - 0.370]	9.696E-01	9.696E-01
Left precentral gyrus	-0.073	0.148	[-0.364 - 0.217]	6.215E-01	8.096E-01
Right precentral gyrus	-0.248	0.148	[-0.538 - 0.042]	9.421E-02	5.201E-01
Left lingual gyrus	-0.037	0.171	[-0.372 - 0.297]	8.268E-01	9.468E-01
Right lingual gyrus	-0.214	0.148	[-0.504 - 0.077]	1.492E-01	5.201E-01
Left superior temporal gyrus	-0.630	0.267	[-1.153 - -0.108]	1.799E-02	4.258E-01
Right superior temporal gyrus	-0.293	0.228	[-0.740 - 0.154]	1.992E-01	6.129E-01
Left fusiform gyrus	-0.006	0.148	[-0.296 - 0.285]	9.689E-01	9.696E-01
Right fusiform gyrus	-0.247	0.204	[-0.646 - 0.153]	2.264E-01	6.182E-01
Left inferior parietal cortex	0.080	0.148	[-0.210 - 0.371]	5.887E-01	8.096E-01
Right inferior parietal cortex	0.077	0.148	[-0.214 - 0.368]	6.047E-01	8.096E-01
Left lateral occipital cortex	-0.203	0.212	[-0.619 - 0.212]	3.374E-01	7.597E-01
Right lateral occipital cortex	-0.083	0.148	[-0.374 - 0.208]	5.749E-01	8.096E-01
Left rostral middle frontal gyrus	-0.103	0.204	[-0.502 - 0.296]	6.138E-01	8.096E-01
Right rostral middle frontal gyrus	-0.171	0.169	[-0.503 - 0.161]	3.120E-01	7.383E-01
Left precuneus	-0.206	0.166	[-0.531 - 0.120]	2.162E-01	6.140E-01
Right precuneus	-0.032	0.178	[-0.380 - 0.316]	8.561E-01	9.497E-01
Left inferior temporal gyrus	0.022	0.194	[-0.359 - 0.402]	9.115E-01	9.696E-01
Right inferior temporal gyrus	-0.129	0.177	[-0.477 - 0.218]	4.659E-01	8.096E-01
Left lateral orbitofrontal cortex	-0.265	0.149	[-0.556 - 0.027]	7.561E-02	5.201E-01
Right lateral orbitofrontal cortex	-0.137	0.148	[-0.427 - 0.152]	3.528E-01	7.597E-01
Left middle temporal gyrus	0.101	0.148	[-0.190 - 0.391]	4.979E-01	8.096E-01
Right middle temporal gyrus	-0.093	0.177	[-0.441 - 0.255]	5.996E-01	8.096E-01
Left postcentral gyrus	-0.306	0.187	[-0.672 - 0.060]	1.017E-01	5.201E-01
Right postcentral gyrus	0.166	0.219	[-0.264 - 0.596]	4.497E-01	8.096E-01
Left medial orbitofrontal cortex	-0.235	0.148	[-0.526 - 0.056]	1.139E-01	5.201E-01
Right medial orbitofrontal cortex	-0.279	0.196	[-0.663 - 0.104]	1.538E-01	5.201E-01
Left cuneus	-0.137	0.181	[-0.492 - 0.218]	4.499E-01	8.096E-01
Right cuneus	-0.487	0.151	[-0.782 - -0.192]	1.235E-03	8.770E-02
Left pars triangularis of inferior frontal gyrus	0.156	0.148	[-0.135 - 0.447]	2.940E-01	7.199E-01
Right pars triangularis of inferior frontal gyrus	0.187	0.202	[-0.208 - 0.583]	3.531E-01	7.597E-01
Left superior parietal cortex	-0.207	0.278	[-0.752 - 0.339]	4.582E-01	8.096E-01
Right superior parietal cortex	-0.501	0.223	[-0.938 - -0.064]	2.461E-02	4.368E-01
Left pars opercularis of inferior frontal gyrus	0.127	0.148	[-0.163 - 0.417]	3.916E-01	8.096E-01
Right pars opercularis of inferior frontal gyrus	-0.154	0.191	[-0.528 - 0.220]	4.193E-01	8.096E-01
Left supramarginal gyrus	-0.061	0.148	[-0.351 - 0.228]	6.778E-01	8.443E-01
Right supramarginal gyrus	-0.013	0.247	[-0.498 - 0.472]	9.589E-01	9.696E-01
Left pericalcarine cortex	-0.288	0.149	[-0.580 - 0.005]	5.377E-02	5.201E-01
Right pericalcarine cortex	-0.410	0.149	[-0.702 - -0.117]	6.025E-03	2.139E-01
Left parahippocampal gyrus	0.222	0.149	[-0.070 - 0.514]	1.361E-01	5.201E-01
Right parahippocampal gyrus	0.111	0.161	[-0.204 - 0.426]	4.894E-01	8.096E-01
Left caudal middle frontal gyrus	-0.157	0.148	[-0.447 - 0.133]	2.895E-01	7.199E-01
Right caudal middle frontal gyrus	-0.066	0.149	[-0.357 - 0.225]	6.553E-01	8.309E-01
Left transverse temporal gyrus	-0.360	0.191	[-0.735 - 0.015]	5.967E-02	5.201E-01
Right transverse temporal gyrus	-0.042	0.192	[-0.419 - 0.334]	8.265E-01	9.468E-01
Left banks of superior temporal sulcus	0.241	0.148	[-0.050 - 0.532]	1.047E-01	5.201E-01
Right banks of superior temporal sulcus	0.089	0.148	[-0.201 - 0.378]	5.486E-01	8.096E-01
Left caudal anterior cingulate cortex	-0.123	0.156	[-0.427 - 0.182]	4.304E-01	8.096E-01
Right caudal anterior cingulate cortex	-0.093	0.185	[-0.456 - 0.271]	6.177E-01	8.096E-01
Left rostral anterior cingulate cortex	-0.189	0.308	[-0.792 - 0.414]	5.395E-01	8.096E-01
Right rostral anterior cingulate cortex	-0.022	0.148	[-0.312 - 0.269]	8.844E-01	9.660E-01
Left posterior cingulate cortex	-0.014	0.157	[-0.321 - 0.293]	9.289E-01	9.696E-01
Right posterior cingulate cortex	0.056	0.212	[-0.360 - 0.473]	7.910E-01	9.360E-01
Left frontal pole	0.249	0.167	[-0.079 - 0.577]	1.367E-01	5.201E-01
Right frontal pole	0.046	0.148	[-0.244 - 0.337]	7.549E-01	9.084E-01
Left paracentral lobule	-0.287	0.186	[-0.651 - 0.078]	1.229E-01	5.201E-01
Right paracentral lobule	-0.090	0.149	[-0.381 - 0.201]	5.456E-01	8.096E-01
Left insula	-0.245	0.162	[-0.564 - 0.073]	1.310E-01	5.201E-01
Right insula	-0.218	0.148	[-0.509 - 0.073]	1.414E-01	5.201E-01
Left entorhinal cortex	0.119	0.194	[-0.262 - 0.500]	5.405E-01	8.096E-01
Right entorhinal cortex	0.165	0.276	[-0.376 - 0.706]	5.509E-01	8.096E-01
Left temporal pole	-0.101	0.293	[-0.675 - 0.473]	7.304E-01	8.941E-01
Right temporal pole	-0.034	0.181	[-0.388 - 0.321]	8.529E-01	9.497E-01
Left isthmus cingulate cortex	-0.262	0.235	[-0.723 - 0.199]	2.651E-01	6.970E-01
Right isthmus cingulate cortex	0.228	0.149	[-0.064 - 0.521]	1.252E-01	5.201E-01

Supplementary Table S162. Cortical surface area differences between individuals with bipolar disorder taking sodium valproate and individuals with bipolar disorder not taking sodium valproate controlling for age, sex, and other medications

	d	Std. Err.	95% CI	*p*-value	FDR *q*-value
Total cortical surface area	0.154	0.465	[-0.758 - 1.065]	7.406E-01	9.834E-01
Left hemisphere	0.131	0.466	[-0.783 - 1.044]	7.792E-01	9.834E-01
Right hemisphere	0.174	0.461	[-0.729 - 1.078]	7.053E-01	9.834E-01
Left superior frontal gyrus	0.276	0.425	[-0.557 - 1.109]	5.158E-01	9.834E-01
Right superior frontal gyrus	0.315	0.407	[-0.483 - 1.114]	4.387E-01	9.834E-01
Left pars orbitalis of inferior frontal gyrus	-0.283	0.185	[-0.646 - 0.079]	1.251E-01	9.834E-01
Right pars orbitalis of inferior frontal gyrus	-0.036	0.305	[-0.634 - 0.562]	9.058E-01	9.834E-01
Left precentral gyrus	-0.142	0.184	[-0.503 - 0.219]	4.406E-01	9.834E-01
Right precentral gyrus	-0.105	0.254	[-0.603 - 0.394]	6.810E-01	9.834E-01
Left lingual gyrus	-0.114	0.435	[-0.967 - 0.738]	7.924E-01	9.834E-01
Right lingual gyrus	0.210	0.489	[-0.748 - 1.169]	6.669E-01	9.834E-01
Left superior temporal gyrus	0.250	0.360	[-0.456 - 0.956]	4.878E-01	9.834E-01
Right superior temporal gyrus	-0.126	0.259	[-0.634 - 0.381]	6.260E-01	9.834E-01
Left fusiform gyrus	-0.005	0.252	[-0.498 - 0.489]	9.845E-01	9.845E-01
Right fusiform gyrus	-0.268	0.346	[-0.947 - 0.410]	4.387E-01	9.834E-01
Left inferior parietal cortex	-0.095	0.389	[-0.858 - 0.669]	8.078E-01	9.834E-01
Right inferior parietal cortex	0.299	0.234	[-0.160 - 0.757]	2.015E-01	9.834E-01
Left lateral occipital cortex	0.124	0.360	[-0.580 - 0.829]	7.295E-01	9.834E-01
Right lateral occipital cortex	-0.034	0.432	[-0.882 - 0.813]	9.370E-01	9.834E-01
Left rostral middle frontal gyrus	-0.124	0.350	[-0.810 - 0.563]	7.242E-01	9.834E-01
Right rostral middle frontal gyrus	-0.052	0.355	[-0.748 - 0.643]	8.827E-01	9.834E-01
Left precuneus	0.098	0.290	[-0.470 - 0.666]	7.354E-01	9.834E-01
Right precuneus	0.247	0.273	[-0.287 - 0.782]	3.646E-01	9.834E-01
Left inferior temporal gyrus	-0.032	0.300	[-0.620 - 0.556]	9.147E-01	9.834E-01
Right inferior temporal gyrus	-0.080	0.410	[-0.883 - 0.722]	8.443E-01	9.834E-01
Left lateral orbitofrontal cortex	-0.028	0.399	[-0.809 - 0.754]	9.445E-01	9.834E-01
Right lateral orbitofrontal cortex	-0.041	0.424	[-0.872 - 0.789]	9.221E-01	9.834E-01
Left middle temporal gyrus	0.209	0.312	[-0.402 - 0.821]	5.024E-01	9.834E-01
Right middle temporal gyrus	-0.022	0.390	[-0.786 - 0.742]	9.557E-01	9.834E-01
Left postcentral gyrus	0.112	0.426	[-0.722 - 0.947]	7.919E-01	9.834E-01
Right postcentral gyrus	0.266	0.353	[-0.427 - 0.958]	4.519E-01	9.834E-01
Left medial orbitofrontal cortex	0.139	0.412	[-0.669 - 0.947]	7.359E-01	9.834E-01
Right medial orbitofrontal cortex	0.154	0.384	[-0.599 - 0.906]	6.892E-01	9.834E-01
Left cuneus	-0.087	0.346	[-0.765 - 0.591]	8.010E-01	9.834E-01
Right cuneus	-0.353	0.445	[-1.227 - 0.520]	4.276E-01	9.834E-01
Left pars triangularis of inferior frontal gyrus	-0.171	0.213	[-0.588 - 0.246]	4.207E-01	9.834E-01
Right pars triangularis of inferior frontal gyrus	-0.125	0.243	[-0.602 - 0.352]	6.084E-01	9.834E-01
Left superior parietal cortex	0.307	0.417	[-0.510 - 1.124]	4.614E-01	9.834E-01
Right superior parietal cortex	0.300	0.427	[-0.537 - 1.138]	4.817E-01	9.834E-01
Left pars opercularis of inferior frontal gyrus	0.116	0.317	[-0.506 - 0.738]	7.151E-01	9.834E-01
Right pars opercularis of inferior frontal gyrus	-0.060	0.184	[-0.420 - 0.301]	7.448E-01	9.834E-01
Left supramarginal gyrus	-0.040	0.436	[-0.894 - 0.815]	9.277E-01	9.834E-01
Right supramarginal gyrus	-0.224	0.367	[-0.942 - 0.495]	5.419E-01	9.834E-01
Left pericalcarine cortex	0.026	0.465	[-0.885 - 0.937]	9.554E-01	9.834E-01
Right pericalcarine cortex	0.197	0.494	[-0.771 - 1.165]	6.897E-01	9.834E-01
Left parahippocampal gyrus	0.069	0.184	[-0.292 - 0.430]	7.082E-01	9.834E-01
Right parahippocampal gyrus	0.212	0.294	[-0.365 - 0.789]	4.716E-01	9.834E-01
Left caudal middle frontal gyrus	0.062	0.275	[-0.476 - 0.601]	8.207E-01	9.834E-01
Right caudal middle frontal gyrus	0.081	0.184	[-0.280 - 0.442]	6.587E-01	9.834E-01
Left transverse temporal gyrus	-0.052	0.312	[-0.663 - 0.559]	8.677E-01	9.834E-01
Right transverse temporal gyrus	-0.219	0.264	[-0.736 - 0.298]	4.066E-01	9.834E-01
Left banks of superior temporal sulcus	0.338	0.245	[-0.143 - 0.819]	1.683E-01	9.834E-01
Right banks of superior temporal sulcus	0.365	0.186	[0.002 - 0.729]	4.894E-02	9.834E-01
Left caudal anterior cingulate cortex	-0.115	0.192	[-0.492 - 0.262]	5.496E-01	9.834E-01
Right caudal anterior cingulate cortex	0.035	0.384	[-0.718 - 0.788]	9.276E-01	9.834E-01
Left rostral anterior cingulate cortex	-0.217	0.242	[-0.692 - 0.258]	3.698E-01	9.834E-01
Right rostral anterior cingulate cortex	0.137	0.185	[-0.225 - 0.499]	4.577E-01	9.834E-01
Left posterior cingulate cortex	-0.057	0.184	[-0.418 - 0.304]	7.574E-01	9.834E-01
Right posterior cingulate cortex	-0.164	0.306	[-0.763 - 0.436]	5.928E-01	9.834E-01
Left frontal pole	-0.189	0.319	[-0.814 - 0.437]	5.542E-01	9.834E-01
Right frontal pole	0.009	0.269	[-0.519 - 0.537]	9.737E-01	9.845E-01
Left paracentral lobule	0.146	0.259	[-0.361 - 0.654]	5.713E-01	9.834E-01
Right paracentral lobule	-0.027	0.390	[-0.791 - 0.736]	9.440E-01	9.834E-01
Left insula	0.298	0.363	[-0.413 - 1.009]	4.113E-01	9.834E-01
Right insula	0.455	0.441	[-0.410 - 1.319]	3.026E-01	9.834E-01
Left entorhinal cortex	0.449	0.316	[-0.170 - 1.067]	1.554E-01	9.834E-01
Right entorhinal cortex	0.134	0.185	[-0.228 - 0.496]	4.679E-01	9.834E-01
Left temporal pole	0.045	0.252	[-0.448 - 0.538]	8.581E-01	9.834E-01
Right temporal pole	0.281	0.326	[-0.357 - 0.920]	3.879E-01	9.834E-01
Left isthmus cingulate cortex	0.123	0.304	[-0.473 - 0.720]	6.851E-01	9.834E-01
Right isthmus cingulate cortex	0.136	0.185	[-0.226 - 0.497]	4.627E-01	9.834E-01

Supplementary Table S163. Cortical surface area differences between individuals with bipolar disorder taking sodium valproate and individuals with bipolar disorder not taking sodium valproate controlling for age, sex, age × sex, age^2, age^2 × sex, ICV, and other medications at 25 years of age or older

	d	Std. Err.	95% CI	*p*-value	FDR *q*-value
Total cortical surface area	-0.089	0.280	[-0.637 - 0.459]	7.509E-01	9.605E-01
Left hemisphere	-0.128	0.277	[-0.671 - 0.414]	6.431E-01	9.605E-01
Right hemisphere	-0.043	0.279	[-0.591 - 0.504]	8.763E-01	9.605E-01
Left superior frontal gyrus	0.007	0.194	[-0.373 - 0.387]	9.723E-01	9.862E-01
Right superior frontal gyrus	0.234	0.195	[-0.148 - 0.616]	2.299E-01	9.605E-01
Left pars orbitalis of inferior frontal gyrus	-0.120	0.194	[-0.500 - 0.260]	5.361E-01	9.605E-01
Right pars orbitalis of inferior frontal gyrus	-0.047	0.260	[-0.557 - 0.464]	8.574E-01	9.605E-01
Left precentral gyrus	-0.091	0.302	[-0.683 - 0.500]	7.620E-01	9.605E-01
Right precentral gyrus	-0.054	0.194	[-0.433 - 0.326]	7.815E-01	9.605E-01
Left lingual gyrus	-0.087	0.253	[-0.583 - 0.410]	7.327E-01	9.605E-01
Right lingual gyrus	0.093	0.324	[-0.542 - 0.727]	7.747E-01	9.605E-01
Left superior temporal gyrus	0.282	0.264	[-0.235 - 0.799]	2.848E-01	9.605E-01
Right superior temporal gyrus	0.002	0.234	[-0.456 - 0.460]	9.938E-01	9.938E-01
Left fusiform gyrus	-0.194	0.289	[-0.760 - 0.372]	5.015E-01	9.605E-01
Right fusiform gyrus	0.050	0.194	[-0.332 - 0.431]	7.987E-01	9.605E-01
Left inferior parietal cortex	-0.388	0.474	[-1.317 - 0.541]	4.130E-01	9.605E-01
Right inferior parietal cortex	0.085	0.350	[-0.600 - 0.770]	8.080E-01	9.605E-01
Left lateral occipital cortex	0.037	0.312	[-0.575 - 0.648]	9.064E-01	9.605E-01
Right lateral occipital cortex	-0.158	0.347	[-0.837 - 0.522]	6.490E-01	9.605E-01
Left rostral middle frontal gyrus	-0.341	0.240	[-0.812 - 0.129]	1.552E-01	9.605E-01
Right rostral middle frontal gyrus	-0.379	0.438	[-1.238 - 0.480]	3.868E-01	9.605E-01
Left precuneus	-0.078	0.212	[-0.492 - 0.337]	7.131E-01	9.605E-01
Right precuneus	-0.093	0.257	[-0.597 - 0.411]	7.173E-01	9.605E-01
Left inferior temporal gyrus	-0.432	0.416	[-1.247 - 0.383]	2.988E-01	9.605E-01
Right inferior temporal gyrus	-0.277	0.371	[-1.005 - 0.450]	4.551E-01	9.605E-01
Left lateral orbitofrontal cortex	0.050	0.354	[-0.645 - 0.745]	8.882E-01	9.605E-01
Right lateral orbitofrontal cortex	-0.091	0.337	[-0.751 - 0.568]	7.860E-01	9.605E-01
Left middle temporal gyrus	0.130	0.283	[-0.424 - 0.685]	6.453E-01	9.605E-01
Right middle temporal gyrus	-0.065	0.194	[-0.445 - 0.316]	7.391E-01	9.605E-01
Left postcentral gyrus	0.114	0.280	[-0.434 - 0.662]	6.838E-01	9.605E-01
Right postcentral gyrus	0.070	0.195	[-0.312 - 0.452]	7.206E-01	9.605E-01
Left medial orbitofrontal cortex	0.041	0.298	[-0.543 - 0.625]	8.904E-01	9.605E-01
Right medial orbitofrontal cortex	0.190	0.422	[-0.638 - 1.017]	6.533E-01	9.605E-01
Left cuneus	-0.199	0.362	[-0.908 - 0.511]	5.829E-01	9.605E-01
Right cuneus	-0.518	0.366	[-1.235 - 0.199]	1.569E-01	9.605E-01
Left pars triangularis of inferior frontal gyrus	-0.195	0.195	[-0.576 - 0.187]	3.170E-01	9.605E-01
Right pars triangularis of inferior frontal gyrus	-0.025	0.316	[-0.645 - 0.595]	9.367E-01	9.780E-01
Left superior parietal cortex	-0.144	0.194	[-0.524 - 0.236]	4.564E-01	9.605E-01
Right superior parietal cortex	0.109	0.194	[-0.272 - 0.489]	5.754E-01	9.605E-01
Left pars opercularis of inferior frontal gyrus	0.218	0.366	[-0.498 - 0.935]	5.504E-01	9.605E-01
Right pars opercularis of inferior frontal gyrus	-0.171	0.304	[-0.766 - 0.425]	5.745E-01	9.605E-01
Left supramarginal gyrus	-0.219	0.363	[-0.931 - 0.492]	5.456E-01	9.605E-01
Right supramarginal gyrus	-0.293	0.440	[-1.155 - 0.569]	5.049E-01	9.605E-01
Left pericalcarine cortex	-0.249	0.200	[-0.641 - 0.144]	2.139E-01	9.605E-01
Right pericalcarine cortex	-0.069	0.319	[-0.695 - 0.557]	8.298E-01	9.605E-01
Left parahippocampal gyrus	0.042	0.194	[-0.339 - 0.423]	8.284E-01	9.605E-01
Right parahippocampal gyrus	0.067	0.283	[-0.487 - 0.622]	8.115E-01	9.605E-01
Left caudal middle frontal gyrus	-0.065	0.194	[-0.445 - 0.315]	7.369E-01	9.605E-01
Right caudal middle frontal gyrus	-0.028	0.194	[-0.408 - 0.352]	8.839E-01	9.605E-01
Left transverse temporal gyrus	-0.200	0.238	[-0.666 - 0.267]	4.014E-01	9.605E-01
Right transverse temporal gyrus	-0.203	0.387	[-0.962 - 0.557]	6.006E-01	9.605E-01
Left banks of superior temporal sulcus	0.493	0.197	[0.108 - 0.878]	1.214E-02	4.391E-01
Right banks of superior temporal sulcus	0.313	0.233	[-0.142 - 0.769]	1.778E-01	9.605E-01
Left caudal anterior cingulate cortex	0.144	0.195	[-0.237 - 0.525]	4.598E-01	9.605E-01
Right caudal anterior cingulate cortex	-0.101	0.358	[-0.801 - 0.600]	7.786E-01	9.605E-01
Left rostral anterior cingulate cortex	-0.492	0.197	[-0.877 - -0.106]	1.237E-02	4.391E-01
Right rostral anterior cingulate cortex	-0.205	0.318	[-0.829 - 0.418]	5.184E-01	9.605E-01
Left posterior cingulate cortex	-0.263	0.286	[-0.824 - 0.297]	3.573E-01	9.605E-01
Right posterior cingulate cortex	-0.176	0.268	[-0.702 - 0.350]	5.114E-01	9.605E-01
Left frontal pole	0.054	0.299	[-0.533 - 0.641]	8.564E-01	9.605E-01
Right frontal pole	0.319	0.287	[-0.244 - 0.882]	2.665E-01	9.605E-01
Left paracentral lobule	-0.026	0.193	[-0.405 - 0.353]	8.914E-01	9.605E-01
Right paracentral lobule	-0.033	0.256	[-0.536 - 0.470]	8.974E-01	9.605E-01
Left insula	0.012	0.194	[-0.369 - 0.392]	9.520E-01	9.796E-01
Right insula	0.374	0.196	[-0.010 - 0.759]	5.606E-02	9.605E-01
Left entorhinal cortex	0.315	0.233	[-0.142 - 0.772]	1.761E-01	9.605E-01
Right entorhinal cortex	0.063	0.264	[-0.454 - 0.580]	8.108E-01	9.605E-01
Left temporal pole	-0.419	0.498	[-1.394 - 0.556]	3.999E-01	9.605E-01
Right temporal pole	0.286	0.287	[-0.276 - 0.847]	3.192E-01	9.605E-01
Left isthmus cingulate cortex	-0.336	0.415	[-1.148 - 0.477]	4.184E-01	9.605E-01
Right isthmus cingulate cortex	0.170	0.194	[-0.209 - 0.550]	3.789E-01	9.605E-01

Supplementary Table S164. Cortical surface area differences between individuals with bipolar disorder taking sodium valproate and individuals with bipolar disorder not taking sodium valproate controlling for age, sex, and other medications at 25 years of age or older

	d	Std. Err.	95% CI	*p*-value	FDR *q*-value
Total cortical surface area	0.152	0.485	[-0.799 - 1.103]	7.547E-01	9.992E-01
Left hemisphere	0.127	0.485	[-0.824 - 1.078]	7.934E-01	9.992E-01
Right hemisphere	0.174	0.482	[-0.771 - 1.118]	7.187E-01	9.992E-01
Left superior frontal gyrus	0.200	0.416	[-0.615 - 1.015]	6.311E-01	9.992E-01
Right superior frontal gyrus	0.333	0.424	[-0.498 - 1.164]	4.317E-01	9.992E-01
Left pars orbitalis of inferior frontal gyrus	-0.194	0.205	[-0.596 - 0.208]	3.448E-01	9.992E-01
Right pars orbitalis of inferior frontal gyrus	-0.068	0.307	[-0.669 - 0.533]	8.245E-01	9.992E-01
Left precentral gyrus	-0.043	0.194	[-0.423 - 0.337]	8.239E-01	9.992E-01
Right precentral gyrus	0.002	0.306	[-0.597 - 0.601]	9.946E-01	9.992E-01
Left lingual gyrus	-0.135	0.430	[-0.977 - 0.708]	7.543E-01	9.992E-01
Right lingual gyrus	0.234	0.523	[-0.791 - 1.260]	6.543E-01	9.992E-01
Left superior temporal gyrus	0.283	0.347	[-0.397 - 0.963]	4.148E-01	9.992E-01
Right superior temporal gyrus	-0.104	0.249	[-0.592 - 0.385]	6.774E-01	9.992E-01
Left fusiform gyrus	-0.014	0.247	[-0.498 - 0.471]	9.556E-01	9.992E-01
Right fusiform gyrus	-0.275	0.368	[-0.996 - 0.446]	4.553E-01	9.992E-01
Left inferior parietal cortex	-0.061	0.427	[-0.898 - 0.775]	8.858E-01	9.992E-01
Right inferior parietal cortex	0.294	0.237	[-0.172 - 0.759]	2.160E-01	9.992E-01
Left lateral occipital cortex	0.134	0.325	[-0.503 - 0.771]	6.803E-01	9.992E-01
Right lateral occipital cortex	-0.079	0.488	[-1.035 - 0.877]	8.714E-01	9.992E-01
Left rostral middle frontal gyrus	-0.102	0.375	[-0.838 - 0.633]	7.849E-01	9.992E-01
Right rostral middle frontal gyrus	-0.007	0.380	[-0.753 - 0.739]	9.856E-01	9.992E-01
Left precuneus	0.056	0.289	[-0.511 - 0.623]	8.472E-01	9.992E-01
Right precuneus	0.255	0.266	[-0.266 - 0.776]	3.375E-01	9.992E-01
Left inferior temporal gyrus	0.003	0.332	[-0.648 - 0.653]	9.934E-01	9.992E-01
Right inferior temporal gyrus	0.014	0.430	[-0.829 - 0.856]	9.747E-01	9.992E-01
Left lateral orbitofrontal cortex	0.010	0.437	[-0.845 - 0.866]	9.810E-01	9.992E-01
Right lateral orbitofrontal cortex	0.002	0.451	[-0.882 - 0.885]	9.970E-01	9.992E-01
Left middle temporal gyrus	0.218	0.346	[-0.461 - 0.897]	5.294E-01	9.992E-01
Right middle temporal gyrus	0.021	0.401	[-0.765 - 0.808]	9.575E-01	9.992E-01
Left postcentral gyrus	0.047	0.455	[-0.844 - 0.938]	9.177E-01	9.992E-01
Right postcentral gyrus	0.168	0.357	[-0.532 - 0.869]	6.377E-01	9.992E-01
Left medial orbitofrontal cortex	0.148	0.390	[-0.617 - 0.912]	7.050E-01	9.992E-01
Right medial orbitofrontal cortex	0.168	0.351	[-0.519 - 0.855]	6.319E-01	9.992E-01
Left cuneus	-0.096	0.427	[-0.933 - 0.742]	8.224E-01	9.992E-01
Right cuneus	-0.364	0.494	[-1.332 - 0.604]	4.610E-01	9.992E-01
Left pars triangularis of inferior frontal gyrus	-0.074	0.215	[-0.495 - 0.347]	7.298E-01	9.992E-01
Right pars triangularis of inferior frontal gyrus	-0.190	0.296	[-0.769 - 0.390]	5.207E-01	9.992E-01
Left superior parietal cortex	0.359	0.471	[-0.565 - 1.282]	4.465E-01	9.992E-01
Right superior parietal cortex	0.298	0.424	[-0.532 - 1.128]	4.819E-01	9.992E-01
Left pars opercularis of inferior frontal gyrus	0.187	0.328	[-0.456 - 0.831]	5.680E-01	9.992E-01
Right pars opercularis of inferior frontal gyrus	-0.010	0.193	[-0.389 - 0.369]	9.589E-01	9.992E-01
Left supramarginal gyrus	-0.092	0.407	[-0.890 - 0.706]	8.204E-01	9.992E-01
Right supramarginal gyrus	-0.245	0.361	[-0.952 - 0.462]	4.967E-01	9.992E-01
Left pericalcarine cortex	-0.017	0.447	[-0.892 - 0.859]	9.698E-01	9.992E-01
Right pericalcarine cortex	0.243	0.540	[-0.816 - 1.302]	6.530E-01	9.992E-01
Left parahippocampal gyrus	0.039	0.194	[-0.340 - 0.419]	8.400E-01	9.992E-01
Right parahippocampal gyrus	0.196	0.293	[-0.378 - 0.770]	5.030E-01	9.992E-01
Left caudal middle frontal gyrus	0.099	0.275	[-0.440 - 0.639]	7.179E-01	9.992E-01
Right caudal middle frontal gyrus	0.119	0.194	[-0.261 - 0.498]	5.404E-01	9.992E-01
Left transverse temporal gyrus	-0.042	0.262	[-0.555 - 0.472]	8.736E-01	9.992E-01
Right transverse temporal gyrus	-0.149	0.274	[-0.687 - 0.388]	5.855E-01	9.992E-01
Left banks of superior temporal sulcus	0.329	0.226	[-0.113 - 0.771]	1.446E-01	9.992E-01
Right banks of superior temporal sulcus	0.323	0.195	[-0.059 - 0.705]	9.795E-02	9.992E-01
Left caudal anterior cingulate cortex	0.017	0.228	[-0.431 - 0.464]	9.421E-01	9.992E-01
Right caudal anterior cingulate cortex	0.000	0.385	[-0.755 - 0.754]	9.992E-01	9.992E-01
Left rostral anterior cingulate cortex	-0.199	0.266	[-0.721 - 0.323]	4.546E-01	9.992E-01
Right rostral anterior cingulate cortex	0.096	0.194	[-0.285 - 0.477]	6.222E-01	9.992E-01
Left posterior cingulate cortex	0.009	0.194	[-0.371 - 0.390]	9.612E-01	9.992E-01
Right posterior cingulate cortex	-0.208	0.314	[-0.824 - 0.408]	5.081E-01	9.992E-01
Left frontal pole	-0.134	0.362	[-0.842 - 0.575]	7.111E-01	9.992E-01
Right frontal pole	-0.032	0.278	[-0.576 - 0.513]	9.090E-01	9.992E-01
Left paracentral lobule	0.092	0.266	[-0.430 - 0.614]	7.293E-01	9.992E-01
Right paracentral lobule	-0.101	0.424	[-0.932 - 0.731]	8.122E-01	9.992E-01
Left insula	0.350	0.358	[-0.352 - 1.052]	3.286E-01	9.992E-01
Right insula	0.462	0.410	[-0.341 - 1.265]	2.595E-01	9.992E-01
Left entorhinal cortex	0.405	0.265	[-0.115 - 0.925]	1.272E-01	9.992E-01
Right entorhinal cortex	0.094	0.194	[-0.287 - 0.475]	6.282E-01	9.992E-01
Left temporal pole	0.030	0.220	[-0.400 - 0.461]	8.899E-01	9.992E-01
Right temporal pole	0.254	0.311	[-0.354 - 0.863]	4.127E-01	9.992E-01
Left isthmus cingulate cortex	0.131	0.333	[-0.521 - 0.784]	6.931E-01	9.992E-01
Right isthmus cingulate cortex	0.152	0.194	[-0.229 - 0.532]	4.340E-01	9.992E-01

Supplementary Table S165. Cortical surface area differences between individuals with bipolar disorder taking second-generation antipsychotics and individuals with bipolar disorder not taking second-generation antipsychotics controlling for age, sex, and other medications

	d	Std. Err.	95% CI	*p*-value	FDR *q*-value
Total cortical surface area	-0.444	0.286	[-1.005 - 0.118]	1.213E-01	4.759E-01
Left hemisphere	-0.452	0.290	[-1.021 - 0.117]	1.196E-01	4.759E-01
Right hemisphere	-0.432	0.283	[-0.986 - 0.122]	1.266E-01	4.759E-01
Left superior frontal gyrus	-0.126	0.182	[-0.482 - 0.231]	4.887E-01	8.378E-01
Right superior frontal gyrus	-0.094	0.236	[-0.557 - 0.369]	6.907E-01	8.494E-01
Left pars orbitalis of inferior frontal gyrus	0.066	0.143	[-0.213 - 0.346]	6.416E-01	8.494E-01
Right pars orbitalis of inferior frontal gyrus	-0.097	0.142	[-0.376 - 0.182]	4.956E-01	8.378E-01
Left precentral gyrus	-0.082	0.248	[-0.568 - 0.404]	7.405E-01	8.494E-01
Right precentral gyrus	0.010	0.143	[-0.269 - 0.290]	9.417E-01	9.681E-01
Left lingual gyrus	-0.449	0.161	[-0.765 - -0.134]	5.200E-03	3.056E-01
Right lingual gyrus	-0.499	0.199	[-0.890 - -0.108]	1.226E-02	3.056E-01
Left superior temporal gyrus	-0.157	0.209	[-0.567 - 0.253]	4.520E-01	8.378E-01
Right superior temporal gyrus	-0.292	0.210	[-0.704 - 0.120]	1.643E-01	5.069E-01
Left fusiform gyrus	-0.336	0.262	[-0.849 - 0.177]	1.991E-01	5.315E-01
Right fusiform gyrus	-0.368	0.290	[-0.937 - 0.200]	2.042E-01	5.315E-01
Left inferior parietal cortex	-0.179	0.245	[-0.658 - 0.301]	4.659E-01	8.378E-01
Right inferior parietal cortex	-0.026	0.155	[-0.331 - 0.278]	8.656E-01	9.172E-01
Left lateral occipital cortex	-0.261	0.212	[-0.677 - 0.154]	2.171E-01	5.315E-01
Right lateral occipital cortex	-0.320	0.144	[-0.602 - -0.039]	2.583E-02	3.056E-01
Left rostral middle frontal gyrus	-0.236	0.196	[-0.621 - 0.149]	2.296E-01	5.435E-01
Right rostral middle frontal gyrus	-0.064	0.180	[-0.417 - 0.288]	7.203E-01	8.494E-01
Left precuneus	-0.253	0.236	[-0.716 - 0.210]	2.848E-01	6.128E-01
Right precuneus	0.005	0.163	[-0.315 - 0.325]	9.734E-01	9.734E-01
Left inferior temporal gyrus	0.053	0.189	[-0.318 - 0.425]	7.776E-01	8.494E-01
Right inferior temporal gyrus	-0.086	0.217	[-0.512 - 0.340]	6.930E-01	8.494E-01
Left lateral orbitofrontal cortex	0.051	0.143	[-0.228 - 0.330]	7.200E-01	8.494E-01
Right lateral orbitofrontal cortex	0.070	0.163	[-0.250 - 0.389]	6.685E-01	8.494E-01
Left middle temporal gyrus	-0.292	0.209	[-0.701 - 0.118]	1.624E-01	5.069E-01
Right middle temporal gyrus	-0.138	0.251	[-0.630 - 0.354]	5.812E-01	8.494E-01
Left postcentral gyrus	-0.109	0.188	[-0.477 - 0.258]	5.596E-01	8.494E-01
Right postcentral gyrus	-0.165	0.147	[-0.453 - 0.123]	2.606E-01	5.781E-01
Left medial orbitofrontal cortex	-0.144	0.238	[-0.611 - 0.323]	5.454E-01	8.494E-01
Right medial orbitofrontal cortex	-0.415	0.282	[-0.968 - 0.137]	1.408E-01	4.759E-01
Left cuneus	-0.484	0.257	[-0.987 - 0.020]	5.973E-02	4.241E-01
Right cuneus	-0.481	0.319	[-1.107 - 0.144]	1.314E-01	4.759E-01
Left pars triangularis of inferior frontal gyrus	0.107	0.173	[-0.231 - 0.446]	5.342E-01	8.494E-01
Right pars triangularis of inferior frontal gyrus	-0.280	0.144	[-0.562 - 0.002]	5.124E-02	4.241E-01
Left superior parietal cortex	-0.268	0.216	[-0.691 - 0.154]	2.137E-01	5.315E-01
Right superior parietal cortex	-0.296	0.257	[-0.799 - 0.207]	2.493E-01	5.710E-01
Left pars opercularis of inferior frontal gyrus	-0.091	0.211	[-0.504 - 0.323]	6.678E-01	8.494E-01
Right pars opercularis of inferior frontal gyrus	-0.311	0.227	[-0.756 - 0.135]	1.713E-01	5.069E-01
Left supramarginal gyrus	-0.471	0.267	[-0.994 - 0.051]	7.707E-02	4.560E-01
Right supramarginal gyrus	-0.220	0.214	[-0.640 - 0.201]	3.056E-01	6.382E-01
Left pericalcarine cortex	-0.648	0.273	[-1.183 - -0.112]	1.777E-02	3.056E-01
Right pericalcarine cortex	-0.415	0.218	[-0.843 - 0.013]	5.753E-02	4.241E-01
Left parahippocampal gyrus	-0.373	0.206	[-0.777 - 0.031]	7.002E-02	4.519E-01
Right parahippocampal gyrus	-0.517	0.316	[-1.136 - 0.101]	1.013E-01	4.759E-01
Left caudal middle frontal gyrus	0.015	0.266	[-0.507 - 0.537]	9.545E-01	9.681E-01
Right caudal middle frontal gyrus	-0.155	0.296	[-0.736 - 0.426]	6.005E-01	8.494E-01
Left transverse temporal gyrus	-0.038	0.143	[-0.317 - 0.242]	7.924E-01	8.524E-01
Right transverse temporal gyrus	0.048	0.142	[-0.231 - 0.326]	7.378E-01	8.494E-01
Left banks of superior temporal sulcus	-0.396	0.176	[-0.741 - -0.051]	2.442E-02	3.056E-01
Right banks of superior temporal sulcus	-0.016	0.183	[-0.374 - 0.343]	9.305E-01	9.681E-01
Left caudal anterior cingulate cortex	-0.121	0.246	[-0.604 - 0.362]	6.239E-01	8.494E-01
Right caudal anterior cingulate cortex	-0.139	0.196	[-0.524 - 0.245]	4.771E-01	8.378E-01
Left rostral anterior cingulate cortex	-0.381	0.257	[-0.885 - 0.123]	1.381E-01	4.759E-01
Right rostral anterior cingulate cortex	0.126	0.143	[-0.154 - 0.405]	3.793E-01	7.404E-01
Left posterior cingulate cortex	0.273	0.144	[-0.008 - 0.555]	5.680E-02	4.241E-01
Right posterior cingulate cortex	-0.051	0.180	[-0.404 - 0.302]	7.763E-01	8.494E-01
Left frontal pole	0.087	0.215	[-0.335 - 0.509]	6.865E-01	8.494E-01
Right frontal pole	-0.792	0.344	[-1.466 - -0.118]	2.133E-02	3.056E-01
Left paracentral lobule	-0.153	0.176	[-0.498 - 0.192]	3.858E-01	7.404E-01
Right paracentral lobule	-0.180	0.143	[-0.460 - 0.101]	2.095E-01	5.315E-01
Left insula	0.068	0.201	[-0.325 - 0.461]	7.345E-01	8.494E-01
Right insula	-0.062	0.200	[-0.455 - 0.331]	7.571E-01	8.494E-01
Left entorhinal cortex	-0.215	0.225	[-0.657 - 0.227]	3.409E-01	6.916E-01
Right entorhinal cortex	-0.139	0.231	[-0.591 - 0.314]	5.481E-01	8.494E-01
Left temporal pole	0.070	0.222	[-0.365 - 0.505]	7.524E-01	8.494E-01
Right temporal pole	-0.383	0.237	[-0.847 - 0.081]	1.060E-01	4.759E-01
Left isthmus cingulate cortex	0.079	0.144	[-0.204 - 0.362]	5.846E-01	8.494E-01
Right isthmus cingulate cortex	-0.298	0.193	[-0.677 - 0.081]	1.237E-01	4.759E-01

Supplementary Table S166. Cortical surface area differences between individuals with bipolar disorder taking second-generation antipsychotics and individuals with bipolar disorder not taking second-generation antipsychotics controlling for age, sex, age × sex, age^2, age^2 × sex, ICV, and other medications at 25 years of age or older

	d	Std. Err.	95% CI	*p*-value	FDR *q*-value
Total cortical surface area	-0.986	0.442	[-1.853 - -0.119]	2.584E-02	2.545E-01
Left hemisphere	-0.893	0.417	[-1.711 - -0.076]	3.226E-02	2.545E-01
Right hemisphere	-1.034	0.468	[-1.951 - -0.116]	2.722E-02	2.545E-01
Left superior frontal gyrus	-0.332	0.236	[-0.795 - 0.131]	1.596E-01	5.152E-01
Right superior frontal gyrus	0.131	0.202	[-0.264 - 0.526]	5.163E-01	7.187E-01
Left pars orbitalis of inferior frontal gyrus	-0.350	0.365	[-1.064 - 0.365]	3.379E-01	6.407E-01
Right pars orbitalis of inferior frontal gyrus	-0.149	0.283	[-0.703 - 0.405]	5.979E-01	7.851E-01
Left precentral gyrus	-0.293	0.286	[-0.853 - 0.267]	3.055E-01	6.101E-01
Right precentral gyrus	-0.231	0.180	[-0.584 - 0.123]	2.007E-01	5.325E-01
Left lingual gyrus	-0.383	0.192	[-0.760 - -0.006]	4.624E-02	2.864E-01
Right lingual gyrus	-0.700	0.352	[-1.389 - -0.011]	4.647E-02	2.864E-01
Left superior temporal gyrus	-0.379	0.373	[-1.110 - 0.352]	3.094E-01	6.101E-01
Right superior temporal gyrus	-0.460	0.352	[-1.149 - 0.229]	1.911E-01	5.325E-01
Left fusiform gyrus	-0.408	0.236	[-0.871 - 0.054]	8.342E-02	3.949E-01
Right fusiform gyrus	-0.211	0.269	[-0.739 - 0.317]	4.330E-01	7.045E-01
Left inferior parietal cortex	-0.259	0.344	[-0.933 - 0.416]	4.519E-01	7.045E-01
Right inferior parietal cortex	-0.389	0.181	[-0.745 - -0.034]	3.194E-02	2.545E-01
Left lateral occipital cortex	0.037	0.257	[-0.466 - 0.541]	8.842E-01	9.370E-01
Right lateral occipital cortex	-0.280	0.172	[-0.617 - 0.056]	1.028E-01	4.561E-01
Left rostral middle frontal gyrus	-0.092	0.208	[-0.500 - 0.316]	6.576E-01	8.114E-01
Right rostral middle frontal gyrus	0.111	0.155	[-0.193 - 0.414]	4.754E-01	7.045E-01
Left precuneus	0.330	0.409	[-0.472 - 1.132]	4.194E-01	7.045E-01
Right precuneus	0.253	0.239	[-0.216 - 0.721]	2.902E-01	6.101E-01
Left inferior temporal gyrus	-0.227	0.314	[-0.842 - 0.388]	4.701E-01	7.045E-01
Right inferior temporal gyrus	-0.384	0.357	[-1.083 - 0.316]	2.824E-01	6.101E-01
Left lateral orbitofrontal cortex	-0.050	0.203	[-0.448 - 0.348]	8.052E-01	8.826E-01
Right lateral orbitofrontal cortex	-0.058	0.193	[-0.437 - 0.321]	7.641E-01	8.762E-01
Left middle temporal gyrus	-0.268	0.197	[-0.654 - 0.118]	1.734E-01	5.325E-01
Right middle temporal gyrus	-0.292	0.197	[-0.679 - 0.095]	1.388E-01	5.133E-01
Left postcentral gyrus	-0.203	0.286	[-0.763 - 0.356]	4.763E-01	7.045E-01
Right postcentral gyrus	-0.324	0.164	[-0.647 - -0.002]	4.841E-02	2.864E-01
Left medial orbitofrontal cortex	-0.199	0.388	[-0.960 - 0.562]	6.082E-01	7.851E-01
Right medial orbitofrontal cortex	-0.183	0.274	[-0.720 - 0.355]	5.053E-01	7.176E-01
Left cuneus	-0.243	0.340	[-0.909 - 0.424]	4.758E-01	7.045E-01
Right cuneus	-0.626	0.408	[-1.426 - 0.173]	1.244E-01	4.908E-01
Left pars triangularis of inferior frontal gyrus	-0.124	0.296	[-0.704 - 0.455]	6.737E-01	8.114E-01
Right pars triangularis of inferior frontal gyrus	-0.415	0.311	[-1.025 - 0.195]	1.829E-01	5.325E-01
Left superior parietal cortex	-1.048	0.467	[-1.964 - -0.133]	2.481E-02	2.545E-01
Right superior parietal cortex	-0.279	0.274	[-0.815 - 0.258]	3.085E-01	6.101E-01
Left pars opercularis of inferior frontal gyrus	-0.077	0.256	[-0.579 - 0.426]	7.652E-01	8.762E-01
Right pars opercularis of inferior frontal gyrus	-0.956	0.425	[-1.789 - -0.124]	2.426E-02	2.545E-01
Left supramarginal gyrus	-0.413	0.172	[-0.749 - -0.077]	1.604E-02	2.545E-01
Right supramarginal gyrus	-0.433	0.340	[-1.098 - 0.233]	2.025E-01	5.325E-01
Left pericalcarine cortex	-0.316	0.271	[-0.847 - 0.214]	2.421E-01	5.928E-01
Right pericalcarine cortex	-0.263	0.277	[-0.806 - 0.280]	3.429E-01	6.407E-01
Left parahippocampal gyrus	-0.288	0.150	[-0.581 - 0.006]	5.473E-02	2.989E-01
Right parahippocampal gyrus	-0.778	0.325	[-1.414 - -0.141]	1.662E-02	2.545E-01
Left caudal middle frontal gyrus	-0.050	0.261	[-0.562 - 0.462]	8.484E-01	9.127E-01
Right caudal middle frontal gyrus	-0.456	0.287	[-1.019 - 0.108]	1.130E-01	4.718E-01
Left transverse temporal gyrus	-0.154	0.435	[-1.007 - 0.699]	7.240E-01	8.567E-01
Right transverse temporal gyrus	-0.072	0.258	[-0.577 - 0.433]	7.803E-01	8.793E-01
Left banks of superior temporal sulcus	-1.081	0.475	[-2.013 - -0.149]	2.301E-02	2.545E-01
Right banks of superior temporal sulcus	-0.301	0.267	[-0.825 - 0.223]	2.598E-01	6.101E-01
Left caudal anterior cingulate cortex	-0.233	0.300	[-0.821 - 0.355]	4.375E-01	7.045E-01
Right caudal anterior cingulate cortex	-0.094	0.165	[-0.417 - 0.230]	5.703E-01	7.639E-01
Left rostral anterior cingulate cortex	-0.002	0.149	[-0.294 - 0.290]	9.891E-01	9.914E-01
Right rostral anterior cingulate cortex	0.167	0.264	[-0.351 - 0.685]	5.272E-01	7.198E-01
Left posterior cingulate cortex	0.238	0.198	[-0.149 - 0.626]	2.280E-01	5.782E-01
Right posterior cingulate cortex	-0.270	0.343	[-0.943 - 0.403]	4.315E-01	7.045E-01
Left frontal pole	-0.027	0.313	[-0.640 - 0.586]	9.306E-01	9.716E-01
Right frontal pole	-0.567	0.304	[-1.164 - 0.029]	6.238E-02	3.164E-01
Left paracentral lobule	0.011	0.207	[-0.394 - 0.416]	9.590E-01	9.868E-01
Right paracentral lobule	-0.536	0.373	[-1.267 - 0.195]	1.505E-01	5.133E-01
Left insula	0.245	0.557	[-0.847 - 1.338]	6.595E-01	8.114E-01
Right insula	-0.387	0.354	[-1.080 - 0.306]	2.739E-01	6.101E-01
Left entorhinal cortex	-0.067	0.274	[-0.603 - 0.470]	8.080E-01	8.826E-01
Right entorhinal cortex	0.003	0.322	[-0.627 - 0.634]	9.914E-01	9.914E-01
Left temporal pole	0.106	0.252	[-0.387 - 0.599]	6.742E-01	8.114E-01
Right temporal pole	-0.214	0.313	[-0.828 - 0.400]	4.944E-01	7.164E-01
Left isthmus cingulate cortex	0.165	0.224	[-0.273 - 0.604]	4.590E-01	7.045E-01
Right isthmus cingulate cortex	-0.443	0.309	[-1.049 - 0.163]	1.518E-01	5.133E-01

Supplementary Table S167. Cortical surface area differences between individuals with bipolar disorder taking second-generation antipsychotics and individuals with bipolar disorder not taking second-generation antipsychotics controlling for age, sex, and other medications at 25 years of age or older

	d	Std. Err.	95% CI	*p*-value	FDR *q*-value
Total cortical surface area	-0.461	0.275	[-1.001 - 0.078]	9.383E-02	4.375E-01
Left hemisphere	-0.447	0.276	[-0.988 - 0.094]	1.056E-01	4.375E-01
Right hemisphere	-0.472	0.274	[-1.010 - 0.066]	8.532E-02	4.375E-01
Left superior frontal gyrus	-0.044	0.185	[-0.407 - 0.318]	8.107E-01	8.855E-01
Right superior frontal gyrus	0.003	0.298	[-0.580 - 0.586]	9.918E-01	9.918E-01
Left pars orbitalis of inferior frontal gyrus	0.056	0.149	[-0.237 - 0.348]	7.091E-01	8.194E-01
Right pars orbitalis of inferior frontal gyrus	-0.217	0.150	[-0.511 - 0.077]	1.479E-01	4.375E-01
Left precentral gyrus	-0.204	0.239	[-0.672 - 0.264]	3.931E-01	6.202E-01
Right precentral gyrus	-0.118	0.149	[-0.410 - 0.174]	4.291E-01	6.527E-01
Left lingual gyrus	-0.414	0.180	[-0.768 - -0.061]	2.166E-02	3.901E-01
Right lingual gyrus	-0.507	0.244	[-0.986 - -0.028]	3.800E-02	3.901E-01
Left superior temporal gyrus	-0.185	0.210	[-0.597 - 0.227]	3.783E-01	6.202E-01
Right superior temporal gyrus	-0.319	0.240	[-0.790 - 0.152]	1.844E-01	4.999E-01
Left fusiform gyrus	-0.347	0.244	[-0.824 - 0.131]	1.551E-01	4.404E-01
Right fusiform gyrus	-0.326	0.250	[-0.816 - 0.164]	1.926E-01	4.999E-01
Left inferior parietal cortex	-0.294	0.244	[-0.772 - 0.184]	2.276E-01	5.386E-01
Right inferior parietal cortex	-0.175	0.168	[-0.504 - 0.154]	2.977E-01	5.562E-01
Left lateral occipital cortex	-0.098	0.322	[-0.729 - 0.533]	7.605E-01	8.437E-01
Right lateral occipital cortex	-0.266	0.178	[-0.616 - 0.083]	1.348E-01	4.375E-01
Left rostral middle frontal gyrus	-0.295	0.238	[-0.761 - 0.171]	2.148E-01	5.258E-01
Right rostral middle frontal gyrus	-0.063	0.170	[-0.396 - 0.271]	7.126E-01	8.194E-01
Left precuneus	-0.191	0.221	[-0.625 - 0.242]	3.871E-01	6.202E-01
Right precuneus	-0.031	0.149	[-0.324 - 0.262]	8.354E-01	8.883E-01
Left inferior temporal gyrus	-0.108	0.229	[-0.557 - 0.342]	6.390E-01	8.102E-01
Right inferior temporal gyrus	-0.277	0.283	[-0.832 - 0.279]	3.286E-01	5.696E-01
Left lateral orbitofrontal cortex	-0.051	0.149	[-0.342 - 0.241]	7.326E-01	8.257E-01
Right lateral orbitofrontal cortex	0.033	0.161	[-0.283 - 0.349]	8.382E-01	8.883E-01
Left middle temporal gyrus	-0.450	0.222	[-0.886 - -0.014]	4.306E-02	3.901E-01
Right middle temporal gyrus	-0.288	0.197	[-0.674 - 0.097]	1.430E-01	4.375E-01
Left postcentral gyrus	-0.130	0.205	[-0.531 - 0.272]	5.266E-01	7.309E-01
Right postcentral gyrus	-0.233	0.150	[-0.527 - 0.061]	1.201E-01	4.375E-01
Left medial orbitofrontal cortex	-0.110	0.228	[-0.557 - 0.336]	6.278E-01	8.102E-01
Right medial orbitofrontal cortex	-0.254	0.241	[-0.726 - 0.218]	2.912E-01	5.562E-01
Left cuneus	-0.500	0.260	[-1.010 - 0.011]	5.494E-02	3.901E-01
Right cuneus	-0.471	0.405	[-1.265 - 0.323]	2.453E-01	5.444E-01
Left pars triangularis of inferior frontal gyrus	0.133	0.170	[-0.199 - 0.466]	4.320E-01	6.527E-01
Right pars triangularis of inferior frontal gyrus	-0.220	0.149	[-0.513 - 0.072]	1.400E-01	4.375E-01
Left superior parietal cortex	-0.550	0.302	[-1.142 - 0.043]	6.890E-02	4.131E-01
Right superior parietal cortex	-0.343	0.266	[-0.864 - 0.178]	1.971E-01	4.999E-01
Left pars opercularis of inferior frontal gyrus	-0.074	0.203	[-0.472 - 0.324]	7.156E-01	8.194E-01
Right pars opercularis of inferior frontal gyrus	-0.227	0.212	[-0.643 - 0.188]	2.838E-01	5.562E-01
Left supramarginal gyrus	-0.430	0.237	[-0.896 - 0.035]	6.981E-02	4.131E-01
Right supramarginal gyrus	-0.156	0.222	[-0.592 - 0.279]	4.818E-01	7.127E-01
Left pericalcarine cortex	-0.508	0.262	[-1.021 - 0.006]	5.291E-02	3.901E-01
Right pericalcarine cortex	-0.324	0.290	[-0.893 - 0.245]	2.641E-01	5.514E-01
Left parahippocampal gyrus	-0.496	0.224	[-0.935 - -0.058]	2.654E-02	3.901E-01
Right parahippocampal gyrus	-0.667	0.317	[-1.288 - -0.046]	3.533E-02	3.901E-01
Left caudal middle frontal gyrus	-0.031	0.237	[-0.496 - 0.433]	8.949E-01	9.222E-01
Right caudal middle frontal gyrus	-0.272	0.278	[-0.818 - 0.274]	3.289E-01	5.696E-01
Left transverse temporal gyrus	-0.060	0.149	[-0.352 - 0.233]	6.898E-01	8.194E-01
Right transverse temporal gyrus	0.064	0.149	[-0.228 - 0.356]	6.670E-01	8.194E-01
Left banks of superior temporal sulcus	-0.599	0.237	[-1.062 - -0.135]	1.138E-02	3.901E-01
Right banks of superior temporal sulcus	-0.215	0.212	[-0.630 - 0.200]	3.094E-01	5.633E-01
Left caudal anterior cingulate cortex	-0.164	0.281	[-0.715 - 0.387]	5.596E-01	7.358E-01
Right caudal anterior cingulate cortex	-0.394	0.245	[-0.874 - 0.086]	1.076E-01	4.375E-01
Left rostral anterior cingulate cortex	-0.279	0.241	[-0.752 - 0.195]	2.485E-01	5.444E-01
Right rostral anterior cingulate cortex	-0.012	0.156	[-0.317 - 0.293]	9.392E-01	9.527E-01
Left posterior cingulate cortex	0.220	0.150	[-0.073 - 0.514]	1.405E-01	4.375E-01
Right posterior cingulate cortex	-0.317	0.277	[-0.861 - 0.227]	2.530E-01	5.444E-01
Left frontal pole	-0.029	0.224	[-0.468 - 0.410]	8.963E-01	9.222E-01
Right frontal pole	-0.740	0.323	[-1.372 - -0.107]	2.190E-02	3.901E-01
Left paracentral lobule	-0.113	0.185	[-0.475 - 0.249]	5.404E-01	7.309E-01
Right paracentral lobule	-0.322	0.163	[-0.641 - -0.003]	4.790E-02	3.901E-01
Left insula	0.122	0.201	[-0.273 - 0.517]	5.456E-01	7.309E-01
Right insula	-0.082	0.204	[-0.482 - 0.318]	6.871E-01	8.194E-01
Left entorhinal cortex	-0.247	0.235	[-0.708 - 0.215]	2.948E-01	5.562E-01
Right entorhinal cortex	-0.240	0.273	[-0.775 - 0.295]	3.795E-01	6.202E-01
Left temporal pole	0.139	0.223	[-0.298 - 0.576]	5.335E-01	7.309E-01
Right temporal pole	-0.474	0.284	[-1.030 - 0.082]	9.482E-02	4.375E-01
Left isthmus cingulate cortex	0.099	0.149	[-0.194 - 0.391]	5.077E-01	7.309E-01
Right isthmus cingulate cortex	-0.288	0.182	[-0.644 - 0.068]	1.126E-01	4.375E-01

Supplementary Table S168. Cortical surface area differences between individuals with bipolar disorder taking first-generation antipsychotics and individuals with bipolar disorder not taking first-generation antipsychotics controlling for age, sex, and other medications

	d	Std. Err.	95% CI	*p*-value	FDR *q*-value
Total cortical surface area	0.084	0.307	[-0.518 - 0.686]	7.844E-01	9.747E-01
Left hemisphere	0.081	0.307	[-0.521 - 0.683]	7.914E-01	9.747E-01
Right hemisphere	0.087	0.307	[-0.516 - 0.689]	7.782E-01	9.747E-01
Left superior frontal gyrus	0.084	0.416	[-0.731 - 0.899]	8.396E-01	9.747E-01
Right superior frontal gyrus	-0.112	0.308	[-0.715 - 0.491]	7.170E-01	9.747E-01
Left pars orbitalis of inferior frontal gyrus	0.011	0.308	[-0.591 - 0.614]	9.703E-01	9.747E-01
Right pars orbitalis of inferior frontal gyrus	-0.556	0.313	[-1.170 - 0.057]	7.554E-02	9.747E-01
Left precentral gyrus	0.508	0.652	[-0.770 - 1.786]	4.358E-01	9.747E-01
Right precentral gyrus	0.450	0.321	[-0.179 - 1.078]	1.610E-01	9.747E-01
Left lingual gyrus	0.183	0.308	[-0.420 - 0.785]	5.529E-01	9.747E-01
Right lingual gyrus	0.160	0.307	[-0.443 - 0.762]	6.032E-01	9.747E-01
Left superior temporal gyrus	0.041	0.307	[-0.561 - 0.644]	8.928E-01	9.747E-01
Right superior temporal gyrus	-0.063	0.308	[-0.667 - 0.541]	8.372E-01	9.747E-01
Left fusiform gyrus	-0.508	0.312	[-1.118 - 0.103]	1.032E-01	9.747E-01
Right fusiform gyrus	-0.110	0.421	[-0.934 - 0.715]	7.941E-01	9.747E-01
Left inferior parietal cortex	-0.138	0.309	[-0.743 - 0.468]	6.562E-01	9.747E-01
Right inferior parietal cortex	0.164	0.597	[-1.006 - 1.334]	7.834E-01	9.747E-01
Left lateral occipital cortex	0.034	0.308	[-0.568 - 0.637]	9.108E-01	9.747E-01
Right lateral occipital cortex	-0.288	0.362	[-0.998 - 0.421]	4.257E-01	9.747E-01
Left rostral middle frontal gyrus	-0.109	0.307	[-0.711 - 0.493]	7.226E-01	9.747E-01
Right rostral middle frontal gyrus	0.140	0.307	[-0.463 - 0.742]	6.495E-01	9.747E-01
Left precuneus	0.377	0.308	[-0.228 - 0.981]	2.216E-01	9.747E-01
Right precuneus	-0.058	0.307	[-0.660 - 0.544]	8.500E-01	9.747E-01
Left inferior temporal gyrus	0.158	0.333	[-0.494 - 0.810]	6.350E-01	9.747E-01
Right inferior temporal gyrus	0.033	0.307	[-0.569 - 0.634]	9.157E-01	9.747E-01
Left lateral orbitofrontal cortex	0.255	0.309	[-0.350 - 0.860]	4.088E-01	9.747E-01
Right lateral orbitofrontal cortex	0.216	0.427	[-0.621 - 1.053]	6.127E-01	9.747E-01
Left middle temporal gyrus	0.594	0.516	[-0.417 - 1.606]	2.495E-01	9.747E-01
Right middle temporal gyrus	0.492	0.313	[-0.122 - 1.105]	1.161E-01	9.747E-01
Left postcentral gyrus	0.349	0.308	[-0.256 - 0.953]	2.579E-01	9.747E-01
Right postcentral gyrus	0.199	0.308	[-0.404 - 0.802]	5.172E-01	9.747E-01
Left medial orbitofrontal cortex	0.263	0.308	[-0.341 - 0.867]	3.933E-01	9.747E-01
Right medial orbitofrontal cortex	-0.355	0.890	[-2.099 - 1.389]	6.901E-01	9.747E-01
Left cuneus	0.146	0.307	[-0.457 - 0.748]	6.351E-01	9.747E-01
Right cuneus	0.159	0.354	[-0.534 - 0.852]	6.531E-01	9.747E-01
Left pars triangularis of inferior frontal gyrus	-0.333	0.308	[-0.937 - 0.271]	2.801E-01	9.747E-01
Right pars triangularis of inferior frontal gyrus	-0.304	0.308	[-0.908 - 0.300]	3.237E-01	9.747E-01
Left superior parietal cortex	0.097	0.307	[-0.505 - 0.699]	7.519E-01	9.747E-01
Right superior parietal cortex	-0.059	0.571	[-1.178 - 1.060]	9.178E-01	9.747E-01
Left pars opercularis of inferior frontal gyrus	-0.164	0.308	[-0.767 - 0.439]	5.942E-01	9.747E-01
Right pars opercularis of inferior frontal gyrus	-0.386	0.311	[-0.995 - 0.223]	2.144E-01	9.747E-01
Left supramarginal gyrus	0.084	0.422	[-0.743 - 0.911]	8.419E-01	9.747E-01
Right supramarginal gyrus	-0.416	0.462	[-1.321 - 0.489]	3.679E-01	9.747E-01
Left pericalcarine cortex	-0.033	0.841	[-1.681 - 1.615]	9.686E-01	9.747E-01
Right pericalcarine cortex	0.470	0.309	[-0.136 - 1.076]	1.287E-01	9.747E-01
Left parahippocampal gyrus	-0.165	0.309	[-0.770 - 0.440]	5.928E-01	9.747E-01
Right parahippocampal gyrus	-0.216	0.307	[-0.819 - 0.387]	4.822E-01	9.747E-01
Left caudal middle frontal gyrus	-0.171	0.307	[-0.773 - 0.432]	5.782E-01	9.747E-01
Right caudal middle frontal gyrus	0.675	0.720	[-0.736 - 2.085]	3.486E-01	9.747E-01
Left transverse temporal gyrus	-0.032	0.308	[-0.636 - 0.573]	9.185E-01	9.747E-01
Right transverse temporal gyrus	-0.472	0.414	[-1.284 - 0.339]	2.540E-01	9.747E-01
Left banks of superior temporal sulcus	-0.268	0.914	[-2.060 - 1.524]	7.692E-01	9.747E-01
Right banks of superior temporal sulcus	0.645	0.375	[-0.091 - 1.380]	8.585E-02	9.747E-01
Left caudal anterior cingulate cortex	-0.436	0.452	[-1.322 - 0.450]	3.352E-01	9.747E-01
Right caudal anterior cingulate cortex	0.369	0.399	[-0.413 - 1.152]	3.549E-01	9.747E-01
Left rostral anterior cingulate cortex	0.023	0.307	[-0.579 - 0.625]	9.393E-01	9.747E-01
Right rostral anterior cingulate cortex	0.913	0.318	[0.290 - 1.537]	4.095E-03	2.908E-01
Left posterior cingulate cortex	0.328	0.659	[-0.964 - 1.620]	6.189E-01	9.747E-01
Right posterior cingulate cortex	-0.228	0.309	[-0.833 - 0.377]	4.605E-01	9.747E-01
Left frontal pole	0.561	0.459	[-0.337 - 1.460]	2.210E-01	9.747E-01
Right frontal pole	-0.600	0.463	[-1.508 - 0.308]	1.952E-01	9.747E-01
Left paracentral lobule	-1.017	0.998	[-2.973 - 0.939]	3.080E-01	9.747E-01
Right paracentral lobule	-0.268	0.343	[-0.940 - 0.404]	4.339E-01	9.747E-01
Left insula	-0.061	0.307	[-0.663 - 0.541]	8.424E-01	9.747E-01
Right insula	0.059	0.314	[-0.556 - 0.674]	8.503E-01	9.747E-01
Left entorhinal cortex	-0.063	0.307	[-0.665 - 0.539]	8.379E-01	9.747E-01
Right entorhinal cortex	-0.082	0.308	[-0.686 - 0.521]	7.889E-01	9.747E-01
Left temporal pole	0.204	0.980	[-1.717 - 2.125]	8.350E-01	9.747E-01
Right temporal pole	-0.010	0.317	[-0.632 - 0.612]	9.747E-01	9.747E-01
Left isthmus cingulate cortex	-0.109	0.309	[-0.715 - 0.496]	7.232E-01	9.747E-01
Right isthmus cingulate cortex	0.016	0.307	[-0.587 - 0.618]	9.597E-01	9.747E-01

Supplementary Table S169. Cortical surface area differences between individuals with bipolar disorder taking first-generation antipsychotics and individuals with bipolar disorder not taking first-generation antipsychotics controlling for age, sex, age × sex, age^2, age^2 × sex, ICV, and other medications at 25 years of age or older

	d	Std. Err.	95% CI	*p*-value	FDR *q*-value
Total cortical surface area	-0.605	0.414	[-1.417 - 0.207]	1.440E-01	5.611E-01
Left hemisphere	-0.519	0.360	[-1.224 - 0.186]	1.489E-01	5.611E-01
Right hemisphere	-0.681	0.462	[-1.586 - 0.225]	1.408E-01	5.611E-01
Left superior frontal gyrus	0.179	0.314	[-0.436 - 0.795]	5.677E-01	7.987E-01
Right superior frontal gyrus	0.426	1.024	[-1.582 - 2.433]	6.776E-01	8.497E-01
Left pars orbitalis of inferior frontal gyrus	-0.123	0.315	[-0.740 - 0.494]	6.960E-01	8.497E-01
Right pars orbitalis of inferior frontal gyrus	-1.569	0.989	[-3.508 - 0.369]	1.126E-01	5.611E-01
Left precentral gyrus	-0.477	0.316	[-1.097 - 0.143]	1.312E-01	5.611E-01
Right precentral gyrus	-0.353	0.318	[-0.976 - 0.270]	2.672E-01	6.631E-01
Left lingual gyrus	-0.138	0.444	[-1.007 - 0.732]	7.562E-01	8.522E-01
Right lingual gyrus	-0.468	0.730	[-1.899 - 0.963]	5.217E-01	7.987E-01
Left superior temporal gyrus	-0.242	0.314	[-0.858 - 0.374]	4.415E-01	7.975E-01
Right superior temporal gyrus	-0.387	0.315	[-1.005 - 0.231]	2.195E-01	6.617E-01
Left fusiform gyrus	-0.909	0.376	[-1.647 - -0.172]	1.563E-02	1.850E-01
Right fusiform gyrus	-0.120	0.352	[-0.810 - 0.570]	7.334E-01	8.522E-01
Left inferior parietal cortex	-0.637	0.320	[-1.264 - -0.009]	4.666E-02	4.141E-01
Right inferior parietal cortex	-0.835	0.325	[-1.471 - -0.199]	1.011E-02	1.697E-01
Left lateral occipital cortex	0.516	1.036	[-1.515 - 2.546]	6.187E-01	7.987E-01
Right lateral occipital cortex	0.073	1.109	[-2.100 - 2.246]	9.474E-01	9.609E-01
Left rostral middle frontal gyrus	-0.160	0.314	[-0.775 - 0.455]	6.094E-01	7.987E-01
Right rostral middle frontal gyrus	0.212	0.316	[-0.406 - 0.831]	5.010E-01	7.987E-01
Left precuneus	0.158	0.314	[-0.458 - 0.774]	6.156E-01	7.987E-01
Right precuneus	-0.418	0.317	[-1.039 - 0.202]	1.865E-01	6.019E-01
Left inferior temporal gyrus	-0.885	0.757	[-2.369 - 0.599]	2.423E-01	6.617E-01
Right inferior temporal gyrus	-0.766	0.653	[-2.045 - 0.513]	2.407E-01	6.617E-01
Left lateral orbitofrontal cortex	-0.034	0.314	[-0.648 - 0.581]	9.138E-01	9.572E-01
Right lateral orbitofrontal cortex	-0.170	0.455	[-1.062 - 0.722]	7.082E-01	8.497E-01
Left middle temporal gyrus	0.033	0.314	[-0.582 - 0.648]	9.167E-01	9.572E-01
Right middle temporal gyrus	-0.384	0.690	[-1.736 - 0.968]	5.778E-01	7.987E-01
Left postcentral gyrus	0.252	0.314	[-0.364 - 0.868]	4.227E-01	7.975E-01
Right postcentral gyrus	-1.019	1.017	[-3.011 - 0.974]	3.163E-01	7.018E-01
Left medial orbitofrontal cortex	0.426	0.316	[-0.194 - 1.046]	1.776E-01	6.006E-01
Right medial orbitofrontal cortex	0.081	0.452	[-0.805 - 0.967]	8.580E-01	9.372E-01
Left cuneus	-0.158	0.315	[-0.775 - 0.460]	6.166E-01	7.987E-01
Right cuneus	0.421	0.810	[-1.165 - 2.008]	6.027E-01	7.987E-01
Left pars triangularis of inferior frontal gyrus	-0.493	0.316	[-1.112 - 0.127]	1.190E-01	5.611E-01
Right pars triangularis of inferior frontal gyrus	0.042	0.517	[-0.972 - 1.056]	9.352E-01	9.609E-01
Left superior parietal cortex	-2.382	2.655	[-7.586 - 2.822]	3.697E-01	7.954E-01
Right superior parietal cortex	-0.378	1.048	[-2.431 - 1.675]	7.181E-01	8.497E-01
Left pars opercularis of inferior frontal gyrus	-0.232	0.314	[-0.848 - 0.384]	4.601E-01	7.975E-01
Right pars opercularis of inferior frontal gyrus	-1.487	1.054	[-3.552 - 0.578]	1.581E-01	5.611E-01
Left supramarginal gyrus	-0.440	0.638	[-1.692 - 0.811]	4.904E-01	7.987E-01
Right supramarginal gyrus	-1.848	1.678	[-5.136 - 1.441]	2.709E-01	6.631E-01
Left pericalcarine cortex	-0.179	1.105	[-2.345 - 1.988]	8.715E-01	9.375E-01
Right pericalcarine cortex	0.873	0.324	[0.238 - 1.509]	7.044E-03	1.667E-01
Left parahippocampal gyrus	-0.357	0.317	[-0.978 - 0.265]	2.608E-01	6.631E-01
Right parahippocampal gyrus	-0.811	0.323	[-1.444 - -0.179]	1.195E-02	1.697E-01
Left caudal middle frontal gyrus	-0.199	0.314	[-0.814 - 0.417]	5.269E-01	7.987E-01
Right caudal middle frontal gyrus	0.124	0.315	[-0.494 - 0.741]	6.949E-01	8.497E-01
Left transverse temporal gyrus	-0.347	0.434	[-1.196 - 0.503]	4.239E-01	7.975E-01
Right transverse temporal gyrus	-0.653	0.453	[-1.541 - 0.235]	1.495E-01	5.611E-01
Left banks of superior temporal sulcus	-1.360	0.336	[-2.019 - -0.702]	5.176E-05	3.675E-03
Right banks of superior temporal sulcus	0.181	0.314	[-0.435 - 0.797]	5.645E-01	7.987E-01
Left caudal anterior cingulate cortex	-1.133	0.328	[-1.776 - -0.490]	5.513E-04	1.957E-02
Right caudal anterior cingulate cortex	0.019	0.789	[-1.527 - 1.565]	9.812E-01	9.812E-01
Left rostral anterior cingulate cortex	0.250	0.315	[-0.367 - 0.866]	4.278E-01	7.975E-01
Right rostral anterior cingulate cortex	0.539	0.381	[-0.208 - 1.287]	1.573E-01	5.611E-01
Left posterior cingulate cortex	0.194	0.604	[-0.989 - 1.378]	7.476E-01	8.522E-01
Right posterior cingulate cortex	-1.929	1.816	[-5.489 - 1.631]	2.882E-01	6.821E-01
Left frontal pole	0.233	0.316	[-0.386 - 0.852]	4.605E-01	7.975E-01
Right frontal pole	-0.676	0.323	[-1.310 - -0.042]	3.657E-02	3.709E-01
Left paracentral lobule	-0.731	0.471	[-1.655 - 0.192]	1.204E-01	5.611E-01
Right paracentral lobule	-0.805	0.544	[-1.870 - 0.261]	1.388E-01	5.611E-01
Left insula	1.169	1.509	[-1.787 - 4.126]	4.382E-01	7.975E-01
Right insula	-0.767	0.988	[-2.703 - 1.169]	4.376E-01	7.975E-01
Left entorhinal cortex	-0.720	0.455	[-1.611 - 0.171]	1.133E-01	5.611E-01
Right entorhinal cortex	-0.383	0.317	[-1.005 - 0.239]	2.270E-01	6.617E-01
Left temporal pole	0.269	0.930	[-1.553 - 2.091]	7.720E-01	8.565E-01
Right temporal pole	-0.785	1.126	[-2.992 - 1.421]	4.853E-01	7.987E-01
Left isthmus cingulate cortex	-0.173	0.315	[-0.790 - 0.444]	5.835E-01	7.987E-01
Right isthmus cingulate cortex	-1.544	1.524	[-4.531 - 1.443]	3.109E-01	7.018E-01

Supplementary Table S170. Cortical surface area differences between individuals with bipolar disorder taking first-generation antipsychotics and individuals with bipolar disorder not taking first-generation antipsychotics controlling for age, sex, and other medications at 25 years of age or older

	d	Std. Err.	95% CI	*p*-value	FDR *q*-value
Total cortical surface area	-0.023	0.314	[-0.638 - 0.592]	9.416E-01	9.899E-01
Left hemisphere	0.015	0.314	[-0.600 - 0.630]	9.620E-01	9.899E-01
Right hemisphere	-0.060	0.314	[-0.675 - 0.554]	8.474E-01	9.899E-01
Left superior frontal gyrus	0.488	0.318	[-0.135 - 1.110]	1.251E-01	9.899E-01
Right superior frontal gyrus	0.629	1.005	[-1.341 - 2.598]	5.314E-01	9.899E-01
Left pars orbitalis of inferior frontal gyrus	0.170	0.314	[-0.446 - 0.785]	5.891E-01	9.899E-01
Right pars orbitalis of inferior frontal gyrus	-0.998	0.605	[-2.184 - 0.188]	9.918E-02	9.899E-01
Left precentral gyrus	0.001	0.315	[-0.616 - 0.618]	9.981E-01	9.981E-01
Right precentral gyrus	-0.025	0.314	[-0.641 - 0.590]	9.354E-01	9.899E-01
Left lingual gyrus	0.163	0.314	[-0.453 - 0.779]	6.032E-01	9.899E-01
Right lingual gyrus	0.188	0.314	[-0.427 - 0.804]	5.487E-01	9.899E-01
Left superior temporal gyrus	0.016	0.315	[-0.602 - 0.634]	9.595E-01	9.899E-01
Right superior temporal gyrus	-0.083	0.315	[-0.700 - 0.534]	7.928E-01	9.899E-01
Left fusiform gyrus	-0.653	0.322	[-1.284 - -0.022]	4.243E-02	7.531E-01
Right fusiform gyrus	-0.029	0.314	[-0.645 - 0.587]	9.270E-01	9.899E-01
Left inferior parietal cortex	-0.356	0.315	[-0.973 - 0.261]	2.587E-01	9.899E-01
Right inferior parietal cortex	-0.300	0.315	[-0.917 - 0.316]	3.394E-01	9.899E-01
Left lateral occipital cortex	0.821	1.005	[-1.149 - 2.791]	4.143E-01	9.899E-01
Right lateral occipital cortex	0.347	1.064	[-1.738 - 2.432]	7.442E-01	9.899E-01
Left rostral middle frontal gyrus	-0.029	0.314	[-0.645 - 0.587]	9.259E-01	9.899E-01
Right rostral middle frontal gyrus	0.143	0.314	[-0.472 - 0.758]	6.485E-01	9.899E-01
Left precuneus	0.428	0.316	[-0.191 - 1.047]	1.753E-01	9.899E-01
Right precuneus	-0.202	0.314	[-0.818 - 0.414]	5.208E-01	9.899E-01
Left inferior temporal gyrus	-0.057	0.314	[-0.672 - 0.557]	8.546E-01	9.899E-01
Right inferior temporal gyrus	-0.341	0.340	[-1.008 - 0.326]	3.161E-01	9.899E-01
Left lateral orbitofrontal cortex	0.064	0.314	[-0.551 - 0.680]	8.374E-01	9.899E-01
Right lateral orbitofrontal cortex	0.115	0.512	[-0.888 - 1.119]	8.220E-01	9.899E-01
Left middle temporal gyrus	0.150	0.314	[-0.465 - 0.766]	6.318E-01	9.899E-01
Right middle temporal gyrus	0.117	0.314	[-0.497 - 0.732]	7.082E-01	9.899E-01
Left postcentral gyrus	0.467	0.317	[-0.155 - 1.088]	1.410E-01	9.899E-01
Right postcentral gyrus	-0.082	0.314	[-0.698 - 0.534]	7.940E-01	9.899E-01
Left medial orbitofrontal cortex	0.377	0.315	[-0.241 - 0.995]	2.315E-01	9.899E-01
Right medial orbitofrontal cortex	0.153	0.329	[-0.492 - 0.799]	6.413E-01	9.899E-01
Left cuneus	0.176	0.314	[-0.440 - 0.792]	5.760E-01	9.899E-01
Right cuneus	0.808	0.958	[-1.068 - 2.685]	3.986E-01	9.899E-01
Left pars triangularis of inferior frontal gyrus	-0.373	0.315	[-0.991 - 0.244]	2.362E-01	9.899E-01
Right pars triangularis of inferior frontal gyrus	-0.055	0.315	[-0.672 - 0.562]	8.608E-01	9.899E-01
Left superior parietal cortex	-0.620	0.735	[-2.060 - 0.821]	3.990E-01	9.899E-01
Right superior parietal cortex	-0.070	0.601	[-1.248 - 1.107]	9.070E-01	9.899E-01
Left pars opercularis of inferior frontal gyrus	-0.198	0.314	[-0.814 - 0.418]	5.289E-01	9.899E-01
Right pars opercularis of inferior frontal gyrus	-0.187	0.314	[-0.802 - 0.429]	5.522E-01	9.899E-01
Left supramarginal gyrus	-0.002	0.371	[-0.730 - 0.726]	9.957E-01	9.981E-01
Right supramarginal gyrus	-0.241	0.317	[-0.862 - 0.379]	4.465E-01	9.899E-01
Left pericalcarine cortex	0.285	0.689	[-1.066 - 1.635]	6.794E-01	9.899E-01
Right pericalcarine cortex	0.985	0.331	[0.336 - 1.634]	2.942E-03	2.089E-01
Left parahippocampal gyrus	-0.417	0.335	[-1.073 - 0.239]	2.130E-01	9.899E-01
Right parahippocampal gyrus	-0.680	0.321	[-1.310 - -0.050]	3.451E-02	7.531E-01
Left caudal middle frontal gyrus	-0.287	0.315	[-0.905 - 0.331]	3.633E-01	9.899E-01
Right caudal middle frontal gyrus	0.115	0.315	[-0.502 - 0.731]	7.156E-01	9.899E-01
Left transverse temporal gyrus	-0.053	0.314	[-0.669 - 0.562]	8.648E-01	9.899E-01
Right transverse temporal gyrus	-0.273	0.316	[-0.892 - 0.345]	3.865E-01	9.899E-01
Left banks of superior temporal sulcus	-0.676	0.483	[-1.622 - 0.270]	1.613E-01	9.899E-01
Right banks of superior temporal sulcus	0.351	0.316	[-0.268 - 0.970]	2.667E-01	9.899E-01
Left caudal anterior cingulate cortex	-0.784	0.320	[-1.412 - -0.157]	1.424E-02	5.055E-01
Right caudal anterior cingulate cortex	-0.170	0.853	[-1.842 - 1.501]	8.416E-01	9.899E-01
Left rostral anterior cingulate cortex	0.201	0.314	[-0.415 - 0.817]	5.226E-01	9.899E-01
Right rostral anterior cingulate cortex	0.572	0.317	[-0.049 - 1.194]	7.108E-02	9.899E-01
Left posterior cingulate cortex	0.106	0.485	[-0.844 - 1.056]	8.265E-01	9.899E-01
Right posterior cingulate cortex	-1.215	1.114	[-3.398 - 0.968]	2.753E-01	9.899E-01
Left frontal pole	0.235	0.315	[-0.381 - 0.852]	4.545E-01	9.899E-01
Right frontal pole	-0.731	0.511	[-1.733 - 0.270]	1.524E-01	9.899E-01
Left paracentral lobule	-0.532	0.372	[-1.262 - 0.197]	1.525E-01	9.899E-01
Right paracentral lobule	-0.487	0.462	[-1.391 - 0.418]	2.917E-01	9.899E-01
Left insula	0.179	0.469	[-0.740 - 1.097]	7.026E-01	9.899E-01
Right insula	-0.026	0.421	[-0.851 - 0.800]	9.516E-01	9.899E-01
Left entorhinal cortex	-0.237	0.314	[-0.853 - 0.379]	4.508E-01	9.899E-01
Right entorhinal cortex	-0.302	0.316	[-0.920 - 0.317]	3.392E-01	9.899E-01
Left temporal pole	0.606	0.573	[-0.517 - 1.729]	2.904E-01	9.899E-01
Right temporal pole	-0.346	0.681	[-1.681 - 0.988]	6.110E-01	9.899E-01
Left isthmus cingulate cortex	0.224	0.316	[-0.396 - 0.844]	4.785E-01	9.899E-01
Right isthmus cingulate cortex	-0.215	0.316	[-0.835 - 0.405]	4.963E-01	9.899E-01

Supplementary Table S171. Cortical surface area differences between individuals with bipolar disorder taking antidepressants and individuals with bipolar disorder not taking antidepressants controlling for age, sex, and other medications

	d	Std. Err.	95% CI	*p*-value	FDR *q*-value
Total cortical surface area	-0.058	0.184	[-0.419 - 0.303]	7.519E-01	9.825E-01
Left hemisphere	-0.076	0.180	[-0.428 - 0.276]	6.727E-01	9.825E-01
Right hemisphere	-0.039	0.188	[-0.408 - 0.330]	8.357E-01	9.825E-01
Left superior frontal gyrus	-0.255	0.228	[-0.702 - 0.192]	2.630E-01	9.825E-01
Right superior frontal gyrus	0.153	0.175	[-0.191 - 0.497]	3.832E-01	9.825E-01
Left pars orbitalis of inferior frontal gyrus	0.015	0.150	[-0.279 - 0.309]	9.205E-01	9.825E-01
Right pars orbitalis of inferior frontal gyrus	-0.036	0.149	[-0.329 - 0.257]	8.118E-01	9.825E-01
Left precentral gyrus	0.062	0.257	[-0.442 - 0.565]	8.109E-01	9.825E-01
Right precentral gyrus	-0.161	0.214	[-0.581 - 0.258]	4.503E-01	9.825E-01
Left lingual gyrus	0.047	0.150	[-0.246 - 0.340]	7.547E-01	9.825E-01
Right lingual gyrus	-0.016	0.149	[-0.309 - 0.276]	9.126E-01	9.825E-01
Left superior temporal gyrus	0.124	0.149	[-0.169 - 0.417]	4.056E-01	9.825E-01
Right superior temporal gyrus	-0.109	0.223	[-0.547 - 0.328]	6.238E-01	9.825E-01
Left fusiform gyrus	0.200	0.150	[-0.094 - 0.494]	1.819E-01	9.825E-01
Right fusiform gyrus	0.000	0.149	[-0.292 - 0.293]	9.977E-01	9.977E-01
Left inferior parietal cortex	0.028	0.149	[-0.264 - 0.321]	8.492E-01	9.825E-01
Right inferior parietal cortex	0.018	0.236	[-0.445 - 0.482]	9.380E-01	9.825E-01
Left lateral occipital cortex	0.142	0.149	[-0.151 - 0.435]	3.427E-01	9.825E-01
Right lateral occipital cortex	0.034	0.151	[-0.262 - 0.330]	8.224E-01	9.825E-01
Left rostral middle frontal gyrus	-0.149	0.150	[-0.442 - 0.144]	3.191E-01	9.825E-01
Right rostral middle frontal gyrus	-0.156	0.150	[-0.450 - 0.137]	2.960E-01	9.825E-01
Left precuneus	-0.043	0.150	[-0.337 - 0.250]	7.718E-01	9.825E-01
Right precuneus	-0.062	0.154	[-0.363 - 0.239]	6.861E-01	9.825E-01
Left inferior temporal gyrus	-0.111	0.160	[-0.425 - 0.203]	4.897E-01	9.825E-01
Right inferior temporal gyrus	0.125	0.149	[-0.168 - 0.418]	4.040E-01	9.825E-01
Left lateral orbitofrontal cortex	-0.132	0.300	[-0.720 - 0.455]	6.585E-01	9.825E-01
Right lateral orbitofrontal cortex	0.033	0.226	[-0.410 - 0.476]	8.837E-01	9.825E-01
Left middle temporal gyrus	-0.063	0.150	[-0.357 - 0.230]	6.718E-01	9.825E-01
Right middle temporal gyrus	0.073	0.166	[-0.253 - 0.398]	6.623E-01	9.825E-01
Left postcentral gyrus	-0.130	0.208	[-0.538 - 0.278]	5.315E-01	9.825E-01
Right postcentral gyrus	-0.020	0.191	[-0.395 - 0.355]	9.180E-01	9.825E-01
Left medial orbitofrontal cortex	0.175	0.150	[-0.118 - 0.469]	2.418E-01	9.825E-01
Right medial orbitofrontal cortex	0.204	0.150	[-0.090 - 0.498]	1.732E-01	9.825E-01
Left cuneus	0.051	0.150	[-0.243 - 0.345]	7.333E-01	9.825E-01
Right cuneus	-0.077	0.152	[-0.376 - 0.222]	6.128E-01	9.825E-01
Left pars triangularis of inferior frontal gyrus	0.096	0.228	[-0.352 - 0.544]	6.744E-01	9.825E-01
Right pars triangularis of inferior frontal gyrus	0.279	0.150	[-0.015 - 0.572]	6.253E-02	9.825E-01
Left superior parietal cortex	-0.007	0.178	[-0.356 - 0.342]	9.685E-01	9.825E-01
Right superior parietal cortex	0.036	0.173	[-0.304 - 0.376]	8.370E-01	9.825E-01
Left pars opercularis of inferior frontal gyrus	-0.304	0.228	[-0.751 - 0.143]	1.822E-01	9.825E-01
Right pars opercularis of inferior frontal gyrus	0.011	0.179	[-0.339 - 0.361]	9.510E-01	9.825E-01
Left supramarginal gyrus	0.030	0.194	[-0.350 - 0.411]	8.765E-01	9.825E-01
Right supramarginal gyrus	0.127	0.149	[-0.165 - 0.419]	3.951E-01	9.825E-01
Left pericalcarine cortex	-0.165	0.150	[-0.459 - 0.129]	2.715E-01	9.825E-01
Right pericalcarine cortex	-0.161	0.150	[-0.456 - 0.134]	2.837E-01	9.825E-01
Left parahippocampal gyrus	0.047	0.218	[-0.381 - 0.475]	8.311E-01	9.825E-01
Right parahippocampal gyrus	0.079	0.149	[-0.213 - 0.372]	5.951E-01	9.825E-01
Left caudal middle frontal gyrus	-0.224	0.150	[-0.517 - 0.070]	1.354E-01	9.825E-01
Right caudal middle frontal gyrus	-0.257	0.290	[-0.826 - 0.311]	3.750E-01	9.825E-01
Left transverse temporal gyrus	0.139	0.150	[-0.154 - 0.433]	3.528E-01	9.825E-01
Right transverse temporal gyrus	0.339	0.150	[0.045 - 0.634]	2.386E-02	8.469E-01
Left banks of superior temporal sulcus	0.074	0.199	[-0.316 - 0.465]	7.089E-01	9.825E-01
Right banks of superior temporal sulcus	0.253	0.150	[-0.040 - 0.547]	9.022E-02	9.825E-01
Left caudal anterior cingulate cortex	0.106	0.223	[-0.331 - 0.543]	6.349E-01	9.825E-01
Right caudal anterior cingulate cortex	0.020	0.165	[-0.303 - 0.344]	9.024E-01	9.825E-01
Left rostral anterior cingulate cortex	-0.312	0.254	[-0.810 - 0.185]	2.187E-01	9.825E-01
Right rostral anterior cingulate cortex	-0.170	0.156	[-0.477 - 0.136]	2.765E-01	9.825E-01
Left posterior cingulate cortex	0.410	0.151	[0.114 - 0.706]	6.570E-03	4.665E-01
Right posterior cingulate cortex	0.048	0.149	[-0.245 - 0.341]	7.466E-01	9.825E-01
Left frontal pole	0.197	0.242	[-0.277 - 0.672]	4.142E-01	9.825E-01
Right frontal pole	-0.118	0.283	[-0.673 - 0.438]	6.782E-01	9.825E-01
Left paracentral lobule	-0.223	0.150	[-0.517 - 0.070]	1.360E-01	9.825E-01
Right paracentral lobule	-0.115	0.150	[-0.409 - 0.178]	4.420E-01	9.825E-01
Left insula	-0.197	0.280	[-0.746 - 0.352]	4.815E-01	9.825E-01
Right insula	-0.087	0.223	[-0.523 - 0.349]	6.964E-01	9.825E-01
Left entorhinal cortex	0.061	0.233	[-0.396 - 0.518]	7.927E-01	9.825E-01
Right entorhinal cortex	0.013	0.271	[-0.518 - 0.544]	9.619E-01	9.825E-01
Left temporal pole	0.197	0.274	[-0.340 - 0.734]	4.714E-01	9.825E-01
Right temporal pole	0.062	0.268	[-0.464 - 0.587]	8.176E-01	9.825E-01
Left isthmus cingulate cortex	-0.006	0.149	[-0.298 - 0.287]	9.687E-01	9.825E-01
Right isthmus cingulate cortex	0.172	0.149	[-0.121 - 0.465]	2.492E-01	9.825E-01

Supplementary Table S172. Cortical surface area differences between individuals with bipolar disorder taking antidepressants and individuals with bipolar disorder not taking antidepressants controlling for age, sex, age × sex, age^2, age^2 × sex, ICV, and other medications at 25 years of age or older

	d	Std. Err.	95% CI	*p*-value	FDR *q*-value
Total cortical surface area	0.060	0.227	[-0.384 - 0.505]	7.906E-01	9.322E-01
Left hemisphere	0.034	0.203	[-0.363 - 0.432]	8.660E-01	9.322E-01
Right hemisphere	0.109	0.265	[-0.410 - 0.629]	6.797E-01	9.322E-01
Left superior frontal gyrus	-0.506	0.230	[-0.958 - -0.054]	2.817E-02	4.999E-01
Right superior frontal gyrus	0.324	0.165	[0.001 - 0.647]	4.922E-02	5.390E-01
Left pars orbitalis of inferior frontal gyrus	0.247	0.424	[-0.585 - 1.078]	5.610E-01	9.321E-01
Right pars orbitalis of inferior frontal gyrus	0.128	0.210	[-0.284 - 0.539]	5.435E-01	9.321E-01
Left precentral gyrus	0.273	0.285	[-0.286 - 0.832]	3.382E-01	8.210E-01
Right precentral gyrus	-0.022	0.301	[-0.612 - 0.568]	9.410E-01	9.544E-01
Left lingual gyrus	-0.158	0.157	[-0.466 - 0.149]	3.131E-01	8.210E-01
Right lingual gyrus	-0.298	0.314	[-0.915 - 0.318]	3.431E-01	8.210E-01
Left superior temporal gyrus	0.146	0.330	[-0.501 - 0.792]	6.590E-01	9.322E-01
Right superior temporal gyrus	-0.287	0.373	[-1.019 - 0.445]	4.422E-01	8.970E-01
Left fusiform gyrus	0.213	0.243	[-0.264 - 0.689]	3.816E-01	8.210E-01
Right fusiform gyrus	0.273	0.199	[-0.117 - 0.664]	1.703E-01	7.370E-01
Left inferior parietal cortex	-0.023	0.173	[-0.362 - 0.316]	8.928E-01	9.322E-01
Right inferior parietal cortex	0.096	0.254	[-0.402 - 0.593]	7.059E-01	9.322E-01
Left lateral occipital cortex	-0.043	0.296	[-0.624 - 0.538]	8.851E-01	9.322E-01
Right lateral occipital cortex	-0.382	0.329	[-1.027 - 0.263]	2.459E-01	8.210E-01
Left rostral middle frontal gyrus	0.055	0.196	[-0.329 - 0.438]	7.802E-01	9.322E-01
Right rostral middle frontal gyrus	0.053	0.196	[-0.331 - 0.436]	7.873E-01	9.322E-01
Left precuneus	0.141	0.208	[-0.267 - 0.550]	4.969E-01	9.284E-01
Right precuneus	0.057	0.245	[-0.424 - 0.538]	8.160E-01	9.322E-01
Left inferior temporal gyrus	-0.597	0.406	[-1.394 - 0.199]	1.415E-01	7.370E-01
Right inferior temporal gyrus	0.269	0.375	[-0.467 - 1.005]	4.736E-01	9.089E-01
Left lateral orbitofrontal cortex	0.664	0.461	[-0.240 - 1.569]	1.499E-01	7.370E-01
Right lateral orbitofrontal cortex	0.295	0.157	[-0.013 - 0.604]	6.073E-02	5.390E-01
Left middle temporal gyrus	-0.003	0.156	[-0.309 - 0.302]	9.829E-01	9.829E-01
Right middle temporal gyrus	0.203	0.223	[-0.234 - 0.639]	3.626E-01	8.210E-01
Left postcentral gyrus	0.032	0.329	[-0.612 - 0.676]	9.219E-01	9.486E-01
Right postcentral gyrus	0.264	0.310	[-0.344 - 0.871]	3.947E-01	8.241E-01
Left medial orbitofrontal cortex	0.379	0.158	[0.069 - 0.688]	1.642E-02	3.887E-01
Right medial orbitofrontal cortex	0.331	0.267	[-0.193 - 0.854]	2.158E-01	8.063E-01
Left cuneus	-0.183	0.279	[-0.730 - 0.364]	5.124E-01	9.321E-01
Right cuneus	-0.365	0.275	[-0.905 - 0.175]	1.857E-01	7.370E-01
Left pars triangularis of inferior frontal gyrus	0.099	0.309	[-0.506 - 0.704]	7.482E-01	9.322E-01
Right pars triangularis of inferior frontal gyrus	0.149	0.157	[-0.158 - 0.455]	3.427E-01	8.210E-01
Left superior parietal cortex	0.245	0.388	[-0.515 - 1.005]	5.275E-01	9.321E-01
Right superior parietal cortex	-0.206	0.156	[-0.513 - 0.100]	1.868E-01	7.370E-01
Left pars opercularis of inferior frontal gyrus	-0.358	0.245	[-0.839 - 0.122]	1.434E-01	7.370E-01
Right pars opercularis of inferior frontal gyrus	-0.676	0.410	[-1.479 - 0.128]	9.922E-02	7.044E-01
Left supramarginal gyrus	-0.117	0.249	[-0.606 - 0.372]	6.397E-01	9.322E-01
Right supramarginal gyrus	-0.171	0.296	[-0.752 - 0.410]	5.645E-01	9.321E-01
Left pericalcarine cortex	-0.315	0.158	[-0.624 - -0.006]	4.556E-02	5.390E-01
Right pericalcarine cortex	-0.303	0.157	[-0.612 - 0.005]	5.391E-02	5.390E-01
Left parahippocampal gyrus	0.095	0.220	[-0.337 - 0.526]	6.663E-01	9.322E-01
Right parahippocampal gyrus	-0.146	0.156	[-0.453 - 0.160]	3.483E-01	8.210E-01
Left caudal middle frontal gyrus	-0.220	0.157	[-0.527 - 0.087]	1.609E-01	7.370E-01
Right caudal middle frontal gyrus	-0.210	0.235	[-0.671 - 0.251]	3.725E-01	8.210E-01
Left transverse temporal gyrus	0.067	0.260	[-0.443 - 0.576]	7.980E-01	9.322E-01
Right transverse temporal gyrus	0.204	0.210	[-0.209 - 0.616]	3.326E-01	8.210E-01
Left banks of superior temporal sulcus	0.080	0.567	[-1.032 - 1.191]	8.884E-01	9.322E-01
Right banks of superior temporal sulcus	0.149	0.156	[-0.157 - 0.455]	3.410E-01	8.210E-01
Left caudal anterior cingulate cortex	0.448	0.165	[0.124 - 0.771]	6.655E-03	2.363E-01
Right caudal anterior cingulate cortex	0.061	0.312	[-0.551 - 0.673]	8.450E-01	9.322E-01
Left rostral anterior cingulate cortex	-0.132	0.306	[-0.731 - 0.467]	6.655E-01	9.322E-01
Right rostral anterior cingulate cortex	0.060	0.347	[-0.621 - 0.740]	8.633E-01	9.322E-01
Left posterior cingulate cortex	0.822	0.284	[0.266 - 1.378]	3.760E-03	2.363E-01
Right posterior cingulate cortex	0.035	0.228	[-0.411 - 0.481]	8.785E-01	9.322E-01
Left frontal pole	0.722	0.506	[-0.270 - 1.715]	1.539E-01	7.370E-01
Right frontal pole	0.145	0.283	[-0.409 - 0.699]	6.080E-01	9.322E-01
Left paracentral lobule	0.104	0.380	[-0.640 - 0.849]	7.834E-01	9.322E-01
Right paracentral lobule	-0.107	0.213	[-0.523 - 0.310]	6.165E-01	9.322E-01
Left insula	-0.170	0.459	[-1.069 - 0.729]	7.102E-01	9.322E-01
Right insula	-0.239	0.263	[-0.754 - 0.276]	3.628E-01	8.210E-01
Left entorhinal cortex	0.082	0.296	[-0.498 - 0.663]	7.809E-01	9.322E-01
Right entorhinal cortex	0.219	0.247	[-0.266 - 0.704]	3.761E-01	8.210E-01
Left temporal pole	0.229	0.318	[-0.393 - 0.851]	4.709E-01	9.089E-01
Right temporal pole	0.546	0.320	[-0.081 - 1.173]	8.798E-02	6.941E-01
Left isthmus cingulate cortex	-0.277	0.279	[-0.823 - 0.269]	3.207E-01	8.210E-01
Right isthmus cingulate cortex	0.157	0.353	[-0.534 - 0.848]	6.556E-01	9.322E-01

Supplementary Table S173. Cortical surface area differences between individuals with bipolar disorder taking antidepressants and individuals with bipolar disorder not taking antidepressants controlling for age, sex, and other medications at 25 years of age or older

	d	Std. Err.	95% CI	*p*-value	FDR *q*-value
Total cortical surface area	0.029	0.157	[-0.278 - 0.335]	8.550E-01	9.663E-01
Left hemisphere	0.013	0.156	[-0.294 - 0.320]	9.339E-01	9.663E-01
Right hemisphere	0.044	0.157	[-0.263 - 0.351]	7.778E-01	9.663E-01
Left superior frontal gyrus	-0.342	0.242	[-0.817 - 0.133]	1.581E-01	9.544E-01
Right superior frontal gyrus	0.182	0.157	[-0.125 - 0.490]	2.452E-01	9.544E-01
Left pars orbitalis of inferior frontal gyrus	0.123	0.156	[-0.182 - 0.428]	4.292E-01	9.544E-01
Right pars orbitalis of inferior frontal gyrus	-0.053	0.156	[-0.360 - 0.254]	7.351E-01	9.663E-01
Left precentral gyrus	0.244	0.176	[-0.100 - 0.588]	1.647E-01	9.544E-01
Right precentral gyrus	-0.070	0.196	[-0.453 - 0.313]	7.208E-01	9.663E-01
Left lingual gyrus	0.062	0.156	[-0.244 - 0.368]	6.921E-01	9.663E-01
Right lingual gyrus	-0.041	0.156	[-0.347 - 0.264]	7.902E-01	9.663E-01
Left superior temporal gyrus	0.148	0.157	[-0.160 - 0.455]	3.463E-01	9.544E-01
Right superior temporal gyrus	-0.120	0.226	[-0.563 - 0.322]	5.939E-01	9.663E-01
Left fusiform gyrus	0.243	0.157	[-0.065 - 0.551]	1.227E-01	9.544E-01
Right fusiform gyrus	0.056	0.156	[-0.249 - 0.362]	7.172E-01	9.663E-01
Left inferior parietal cortex	0.118	0.174	[-0.223 - 0.460]	4.974E-01	9.544E-01
Right inferior parietal cortex	0.039	0.256	[-0.463 - 0.541]	8.786E-01	9.663E-01
Left lateral occipital cortex	0.079	0.177	[-0.268 - 0.426]	6.553E-01	9.663E-01
Right lateral occipital cortex	0.088	0.156	[-0.218 - 0.394]	5.729E-01	9.663E-01
Left rostral middle frontal gyrus	-0.142	0.169	[-0.472 - 0.189]	4.017E-01	9.544E-01
Right rostral middle frontal gyrus	-0.098	0.156	[-0.403 - 0.207]	5.299E-01	9.663E-01
Left precuneus	-0.111	0.156	[-0.417 - 0.195]	4.780E-01	9.544E-01
Right precuneus	-0.096	0.156	[-0.403 - 0.211]	5.392E-01	9.663E-01
Left inferior temporal gyrus	-0.012	0.198	[-0.399 - 0.376]	9.527E-01	9.663E-01
Right inferior temporal gyrus	0.280	0.157	[-0.027 - 0.587]	7.388E-02	9.544E-01
Left lateral orbitofrontal cortex	-0.007	0.279	[-0.553 - 0.539]	9.800E-01	9.800E-01
Right lateral orbitofrontal cortex	0.112	0.230	[-0.339 - 0.562]	6.267E-01	9.663E-01
Left middle temporal gyrus	-0.026	0.165	[-0.349 - 0.298]	8.752E-01	9.663E-01
Right middle temporal gyrus	0.160	0.221	[-0.273 - 0.594]	4.679E-01	9.544E-01
Left postcentral gyrus	-0.078	0.183	[-0.437 - 0.281]	6.690E-01	9.663E-01
Right postcentral gyrus	0.015	0.219	[-0.414 - 0.443]	9.457E-01	9.663E-01
Left medial orbitofrontal cortex	0.194	0.156	[-0.113 - 0.501]	2.147E-01	9.544E-01
Right medial orbitofrontal cortex	0.151	0.173	[-0.188 - 0.490]	3.822E-01	9.544E-01
Left cuneus	0.145	0.157	[-0.162 - 0.453]	3.535E-01	9.544E-01
Right cuneus	-0.032	0.158	[-0.342 - 0.279]	8.409E-01	9.663E-01
Left pars triangularis of inferior frontal gyrus	0.287	0.157	[-0.021 - 0.595]	6.748E-02	9.544E-01
Right pars triangularis of inferior frontal gyrus	0.132	0.157	[-0.175 - 0.440]	3.985E-01	9.544E-01
Left superior parietal cortex	0.305	0.320	[-0.323 - 0.933]	3.407E-01	9.544E-01
Right superior parietal cortex	0.012	0.172	[-0.327 - 0.350]	9.468E-01	9.663E-01
Left pars opercularis of inferior frontal gyrus	-0.344	0.266	[-0.866 - 0.178]	1.962E-01	9.544E-01
Right pars opercularis of inferior frontal gyrus	-0.057	0.180	[-0.411 - 0.296]	7.501E-01	9.663E-01
Left supramarginal gyrus	-0.013	0.173	[-0.352 - 0.326]	9.400E-01	9.663E-01
Right supramarginal gyrus	0.134	0.156	[-0.171 - 0.439]	3.890E-01	9.544E-01
Left pericalcarine cortex	-0.175	0.157	[-0.482 - 0.131]	2.627E-01	9.544E-01
Right pericalcarine cortex	-0.127	0.156	[-0.433 - 0.179]	4.154E-01	9.544E-01
Left parahippocampal gyrus	0.277	0.203	[-0.120 - 0.675]	1.713E-01	9.544E-01
Right parahippocampal gyrus	0.081	0.156	[-0.225 - 0.386]	6.043E-01	9.663E-01
Left caudal middle frontal gyrus	-0.264	0.157	[-0.571 - 0.044]	9.287E-02	9.544E-01
Right caudal middle frontal gyrus	-0.203	0.275	[-0.742 - 0.337]	4.619E-01	9.544E-01
Left transverse temporal gyrus	0.051	0.183	[-0.308 - 0.410]	7.809E-01	9.663E-01
Right transverse temporal gyrus	0.268	0.157	[-0.040 - 0.576]	8.756E-02	9.544E-01
Left banks of superior temporal sulcus	0.160	0.157	[-0.147 - 0.468]	3.061E-01	9.544E-01
Right banks of superior temporal sulcus	0.157	0.156	[-0.148 - 0.463]	3.130E-01	9.544E-01
Left caudal anterior cingulate cortex	0.155	0.223	[-0.283 - 0.593]	4.869E-01	9.544E-01
Right caudal anterior cingulate cortex	0.029	0.215	[-0.393 - 0.450]	8.936E-01	9.663E-01
Left rostral anterior cingulate cortex	-0.368	0.231	[-0.821 - 0.086]	1.119E-01	9.544E-01
Right rostral anterior cingulate cortex	-0.158	0.157	[-0.466 - 0.149]	3.126E-01	9.544E-01
Left posterior cingulate cortex	0.559	0.159	[0.248 - 0.870]	4.271E-04	3.033E-02
Right posterior cingulate cortex	-0.056	0.156	[-0.362 - 0.251]	7.218E-01	9.663E-01
Left frontal pole	0.205	0.297	[-0.378 - 0.787]	4.913E-01	9.544E-01
Right frontal pole	-0.081	0.256	[-0.583 - 0.421]	7.527E-01	9.663E-01
Left paracentral lobule	-0.208	0.156	[-0.514 - 0.098]	1.831E-01	9.544E-01
Right paracentral lobule	-0.207	0.183	[-0.565 - 0.152]	2.592E-01	9.544E-01
Left insula	-0.099	0.243	[-0.576 - 0.377]	6.832E-01	9.663E-01
Right insula	-0.059	0.157	[-0.366 - 0.248]	7.079E-01	9.663E-01
Left entorhinal cortex	0.192	0.240	[-0.279 - 0.663]	4.243E-01	9.544E-01
Right entorhinal cortex	0.050	0.239	[-0.418 - 0.518]	8.347E-01	9.663E-01
Left temporal pole	0.271	0.223	[-0.167 - 0.709]	2.249E-01	9.544E-01
Right temporal pole	0.122	0.220	[-0.310 - 0.553]	5.812E-01	9.663E-01
Left isthmus cingulate cortex	-0.022	0.156	[-0.329 - 0.285]	8.881E-01	9.663E-01
Right isthmus cingulate cortex	0.146	0.156	[-0.160 - 0.451]	3.501E-01	9.544E-01

Supplementary Table S174. Cortical surface area differences between individuals with major depressive disorder not taking antidepressants and healthy comparison subjects controlling for age and sex

	d	Std. Err.	95% CI	*p*-value	FDR *q*-value
Total cortical surface area	-0.253	0.170	[-0.585 - 0.080]	1.364E-01	4.560E-01
Left hemisphere	-0.255	0.176	[-0.600 - 0.090]	1.470E-01	4.560E-01
Right hemisphere	-0.249	0.162	[-0.567 - 0.070]	1.259E-01	4.560E-01
Left superior frontal gyrus	-0.203	0.148	[-0.492 - 0.087]	1.697E-01	4.587E-01
Right superior frontal gyrus	-0.267	0.146	[-0.553 - 0.019]	6.724E-02	4.560E-01
Left pars orbitalis of inferior frontal gyrus	-0.296	0.177	[-0.643 - 0.051]	9.502E-02	4.560E-01
Right pars orbitalis of inferior frontal gyrus	-0.216	0.146	[-0.502 - 0.069]	1.380E-01	4.560E-01
Left precentral gyrus	-0.150	0.146	[-0.436 - 0.136]	3.030E-01	5.562E-01
Right precentral gyrus	-0.159	0.146	[-0.444 - 0.127]	2.753E-01	5.562E-01
Left lingual gyrus	-0.270	0.292	[-0.842 - 0.303]	3.557E-01	6.013E-01
Right lingual gyrus	-0.245	0.210	[-0.657 - 0.167]	2.434E-01	5.296E-01
Left superior temporal gyrus	-0.191	0.179	[-0.542 - 0.161]	2.877E-01	5.562E-01
Right superior temporal gyrus	-0.092	0.146	[-0.377 - 0.194]	5.298E-01	7.677E-01
Left fusiform gyrus	-0.198	0.146	[-0.484 - 0.088]	1.744E-01	4.587E-01
Right fusiform gyrus	-0.301	0.191	[-0.675 - 0.073]	1.149E-01	4.560E-01
Left inferior parietal cortex	-0.107	0.219	[-0.537 - 0.323]	6.245E-01	8.210E-01
Right inferior parietal cortex	-0.083	0.250	[-0.573 - 0.406]	7.381E-01	9.035E-01
Left lateral occipital cortex	-0.182	0.148	[-0.473 - 0.109]	2.196E-01	5.197E-01
Right lateral occipital cortex	-0.358	0.183	[-0.717 - 0.001]	5.055E-02	4.560E-01
Left rostral middle frontal gyrus	-0.255	0.146	[-0.541 - 0.031]	8.044E-02	4.560E-01
Right rostral middle frontal gyrus	-0.050	0.179	[-0.400 - 0.300]	7.796E-01	9.120E-01
Left precuneus	-0.290	0.146	[-0.577 - -0.004]	4.690E-02	4.560E-01
Right precuneus	-0.113	0.146	[-0.399 - 0.173]	4.391E-01	6.927E-01
Left inferior temporal gyrus	-0.072	0.169	[-0.403 - 0.260]	6.723E-01	8.679E-01
Right inferior temporal gyrus	-0.174	0.169	[-0.506 - 0.158]	3.041E-01	5.562E-01
Left lateral orbitofrontal cortex	-0.305	0.146	[-0.590 - -0.019]	3.636E-02	4.560E-01
Right lateral orbitofrontal cortex	-0.257	0.146	[-0.542 - 0.028]	7.764E-02	4.560E-01
Left middle temporal gyrus	-0.096	0.146	[-0.382 - 0.189]	5.091E-01	7.530E-01
Right middle temporal gyrus	-0.247	0.187	[-0.613 - 0.119]	1.857E-01	4.708E-01
Left postcentral gyrus	-0.130	0.219	[-0.559 - 0.299]	5.522E-01	7.734E-01
Right postcentral gyrus	-0.285	0.146	[-0.571 - 0.002]	5.164E-02	4.560E-01
Left medial orbitofrontal cortex	-0.415	0.173	[-0.754 - -0.076]	1.637E-02	4.560E-01
Right medial orbitofrontal cortex	-0.280	0.146	[-0.567 - 0.006]	5.486E-02	4.560E-01
Left cuneus	-0.260	0.185	[-0.622 - 0.102]	1.599E-01	4.560E-01
Right cuneus	-0.263	0.156	[-0.568 - 0.043]	9.212E-02	4.560E-01
Left pars triangularis of inferior frontal gyrus	-0.266	0.146	[-0.552 - 0.020]	6.854E-02	4.560E-01
Right pars triangularis of inferior frontal gyrus	-0.283	0.154	[-0.585 - 0.019]	6.631E-02	4.560E-01
Left superior parietal cortex	-0.164	0.230	[-0.615 - 0.287]	4.773E-01	7.366E-01
Right superior parietal cortex	-0.104	0.150	[-0.397 - 0.190]	4.877E-01	7.367E-01
Left pars opercularis of inferior frontal gyrus	0.046	0.188	[-0.323 - 0.415]	8.066E-01	9.236E-01
Right pars opercularis of inferior frontal gyrus	0.002	0.145	[-0.283 - 0.287]	9.892E-01	9.978E-01
Left supramarginal gyrus	-0.206	0.146	[-0.492 - 0.080]	1.587E-01	4.560E-01
Right supramarginal gyrus	-0.161	0.146	[-0.447 - 0.125]	2.696E-01	5.562E-01
Left pericalcarine cortex	-0.276	0.197	[-0.662 - 0.110]	1.606E-01	4.560E-01
Right pericalcarine cortex	-0.377	0.203	[-0.774 - 0.021]	6.349E-02	4.560E-01
Left parahippocampal gyrus	0.096	0.191	[-0.277 - 0.470]	6.142E-01	8.210E-01
Right parahippocampal gyrus	-0.310	0.202	[-0.707 - 0.086]	1.246E-01	4.560E-01
Left caudal middle frontal gyrus	-0.234	0.150	[-0.529 - 0.060]	1.189E-01	4.560E-01
Right caudal middle frontal gyrus	0.020	0.156	[-0.286 - 0.326]	8.975E-01	9.716E-01
Left transverse temporal gyrus	-0.216	0.146	[-0.501 - 0.069]	1.375E-01	4.560E-01
Right transverse temporal gyrus	-0.169	0.145	[-0.455 - 0.116]	2.441E-01	5.296E-01
Left banks of superior temporal sulcus	-0.055	0.146	[-0.340 - 0.230]	7.040E-01	8.799E-01
Right banks of superior temporal sulcus	-0.031	0.207	[-0.437 - 0.376]	8.826E-01	9.716E-01
Left caudal anterior cingulate cortex	-0.100	0.175	[-0.442 - 0.242]	5.664E-01	7.734E-01
Right caudal anterior cingulate cortex	-0.184	0.145	[-0.469 - 0.101]	2.060E-01	5.043E-01
Left rostral anterior cingulate cortex	-0.146	0.146	[-0.431 - 0.140]	3.171E-01	5.628E-01
Right rostral anterior cingulate cortex	-0.184	0.158	[-0.494 - 0.127]	2.461E-01	5.296E-01
Left posterior cingulate cortex	-0.059	0.214	[-0.477 - 0.360]	7.836E-01	9.120E-01
Right posterior cingulate cortex	-0.119	0.145	[-0.404 - 0.166]	4.135E-01	6.673E-01
Left frontal pole	-0.142	0.166	[-0.468 - 0.185]	3.947E-01	6.518E-01
Right frontal pole	0.000	0.146	[-0.286 - 0.285]	9.978E-01	9.978E-01
Left paracentral lobule	-0.021	0.145	[-0.306 - 0.264]	8.859E-01	9.716E-01
Right paracentral lobule	-0.044	0.145	[-0.329 - 0.241]	7.616E-01	9.120E-01
Left insula	-0.012	0.206	[-0.417 - 0.393]	9.541E-01	9.978E-01
Right insula	0.018	0.148	[-0.272 - 0.308]	9.031E-01	9.716E-01
Left entorhinal cortex	0.088	0.149	[-0.204 - 0.380]	5.557E-01	7.734E-01
Right entorhinal cortex	0.055	0.146	[-0.231 - 0.341]	7.064E-01	8.799E-01
Left temporal pole	-0.001	0.146	[-0.286 - 0.285]	9.965E-01	9.978E-01
Right temporal pole	-0.008	0.146	[-0.293 - 0.278]	9.572E-01	9.978E-01
Left isthmus cingulate cortex	-0.149	0.145	[-0.434 - 0.136]	3.055E-01	5.562E-01
Right isthmus cingulate cortex	-0.137	0.146	[-0.423 - 0.149]	3.472E-01	6.012E-01

Supplementary Table S175. Cortical surface area differences between individuals with major depressive disorder not taking antidepressants and healthy comparison subjects controlling for age and sex over 21 years of old

	d	Std. Err.	95% CI	*p*-value	FDR *q*-value
Total cortical surface area	-0.189	0.217	[-0.616 - 0.237]	3.835E-01	8.489E-01
Left hemisphere	-0.181	0.224	[-0.620 - 0.259]	4.200E-01	8.489E-01
Right hemisphere	-0.197	0.209	[-0.607 - 0.213]	3.467E-01	8.489E-01
Left superior frontal gyrus	-0.104	0.157	[-0.413 - 0.205]	5.091E-01	8.613E-01
Right superior frontal gyrus	-0.227	0.158	[-0.536 - 0.082]	1.506E-01	8.489E-01
Left pars orbitalis of inferior frontal gyrus	-0.166	0.201	[-0.561 - 0.228]	4.087E-01	8.489E-01
Right pars orbitalis of inferior frontal gyrus	-0.175	0.180	[-0.528 - 0.178]	3.316E-01	8.489E-01
Left precentral gyrus	-0.164	0.179	[-0.514 - 0.186]	3.598E-01	8.489E-01
Right precentral gyrus	-0.181	0.158	[-0.490 - 0.128]	2.511E-01	8.489E-01
Left lingual gyrus	-0.195	0.354	[-0.888 - 0.498]	5.813E-01	8.786E-01
Right lingual gyrus	-0.245	0.251	[-0.737 - 0.247]	3.294E-01	8.489E-01
Left superior temporal gyrus	-0.190	0.245	[-0.671 - 0.290]	4.373E-01	8.489E-01
Right superior temporal gyrus	-0.062	0.207	[-0.468 - 0.344]	7.638E-01	9.331E-01
Left fusiform gyrus	-0.175	0.164	[-0.496 - 0.146]	2.848E-01	8.489E-01
Right fusiform gyrus	-0.150	0.159	[-0.462 - 0.163]	3.473E-01	8.489E-01
Left inferior parietal cortex	-0.065	0.242	[-0.539 - 0.409]	7.885E-01	9.331E-01
Right inferior parietal cortex	0.037	0.281	[-0.514 - 0.589]	8.942E-01	9.438E-01
Left lateral occipital cortex	-0.122	0.163	[-0.442 - 0.197]	4.531E-01	8.489E-01
Right lateral occipital cortex	-0.376	0.235	[-0.836 - 0.084]	1.092E-01	8.489E-01
Left rostral middle frontal gyrus	-0.200	0.158	[-0.509 - 0.109]	2.047E-01	8.489E-01
Right rostral middle frontal gyrus	-0.024	0.225	[-0.464 - 0.417]	9.151E-01	9.438E-01
Left precuneus	-0.241	0.158	[-0.550 - 0.069]	1.272E-01	8.489E-01
Right precuneus	-0.096	0.158	[-0.405 - 0.214]	5.442E-01	8.786E-01
Left inferior temporal gyrus	-0.032	0.171	[-0.367 - 0.302]	8.499E-01	9.438E-01
Right inferior temporal gyrus	-0.136	0.192	[-0.512 - 0.240]	4.782E-01	8.489E-01
Left lateral orbitofrontal cortex	-0.150	0.163	[-0.470 - 0.170]	3.588E-01	8.489E-01
Right lateral orbitofrontal cortex	-0.142	0.157	[-0.450 - 0.167]	3.677E-01	8.489E-01
Left middle temporal gyrus	-0.037	0.158	[-0.346 - 0.272]	8.144E-01	9.438E-01
Right middle temporal gyrus	-0.284	0.221	[-0.717 - 0.149]	1.987E-01	8.489E-01
Left postcentral gyrus	-0.154	0.233	[-0.610 - 0.303]	5.095E-01	8.613E-01
Right postcentral gyrus	-0.307	0.158	[-0.617 - 0.003]	5.255E-02	8.489E-01
Left medial orbitofrontal cortex	-0.379	0.236	[-0.841 - 0.083]	1.075E-01	8.489E-01
Right medial orbitofrontal cortex	-0.229	0.181	[-0.584 - 0.127]	2.078E-01	8.489E-01
Left cuneus	-0.180	0.240	[-0.651 - 0.291]	4.537E-01	8.489E-01
Right cuneus	-0.294	0.188	[-0.661 - 0.074]	1.171E-01	8.489E-01
Left pars triangularis of inferior frontal gyrus	-0.299	0.158	[-0.609 - 0.011]	5.843E-02	8.489E-01
Right pars triangularis of inferior frontal gyrus	-0.183	0.158	[-0.493 - 0.127]	2.473E-01	8.489E-01
Left superior parietal cortex	-0.148	0.294	[-0.724 - 0.429]	6.156E-01	8.786E-01
Right superior parietal cortex	-0.100	0.184	[-0.461 - 0.260]	5.853E-01	8.786E-01
Left pars opercularis of inferior frontal gyrus	0.170	0.227	[-0.275 - 0.615]	4.545E-01	8.489E-01
Right pars opercularis of inferior frontal gyrus	-0.003	0.157	[-0.311 - 0.306]	9.864E-01	9.864E-01
Left supramarginal gyrus	-0.135	0.158	[-0.444 - 0.175]	3.933E-01	8.489E-01
Right supramarginal gyrus	-0.112	0.158	[-0.421 - 0.197]	4.779E-01	8.489E-01
Left pericalcarine cortex	-0.217	0.237	[-0.680 - 0.247]	3.597E-01	8.489E-01
Right pericalcarine cortex	-0.373	0.248	[-0.859 - 0.114]	1.337E-01	8.489E-01
Left parahippocampal gyrus	0.184	0.194	[-0.197 - 0.565]	3.435E-01	8.489E-01
Right parahippocampal gyrus	-0.316	0.254	[-0.814 - 0.183]	2.144E-01	8.489E-01
Left caudal middle frontal gyrus	-0.216	0.202	[-0.612 - 0.180]	2.860E-01	8.489E-01
Right caudal middle frontal gyrus	0.068	0.227	[-0.376 - 0.513]	7.627E-01	9.331E-01
Left transverse temporal gyrus	-0.152	0.158	[-0.461 - 0.157]	3.349E-01	8.489E-01
Right transverse temporal gyrus	-0.134	0.157	[-0.442 - 0.175]	3.957E-01	8.489E-01
Left banks of superior temporal sulcus	-0.024	0.157	[-0.333 - 0.284]	8.782E-01	9.438E-01
Right banks of superior temporal sulcus	0.033	0.262	[-0.480 - 0.547]	8.983E-01	9.438E-01
Left caudal anterior cingulate cortex	-0.087	0.222	[-0.522 - 0.347]	6.930E-01	8.786E-01
Right caudal anterior cingulate cortex	-0.147	0.178	[-0.496 - 0.203]	4.110E-01	8.489E-01
Left rostral anterior cingulate cortex	-0.091	0.158	[-0.400 - 0.218]	5.644E-01	8.786E-01
Right rostral anterior cingulate cortex	-0.106	0.252	[-0.600 - 0.389]	6.754E-01	8.786E-01
Left posterior cingulate cortex	0.101	0.252	[-0.393 - 0.595]	6.888E-01	8.786E-01
Right posterior cingulate cortex	-0.070	0.157	[-0.378 - 0.238]	6.559E-01	8.786E-01
Left frontal pole	-0.180	0.192	[-0.556 - 0.196]	3.480E-01	8.489E-01
Right frontal pole	-0.017	0.168	[-0.346 - 0.311]	9.172E-01	9.438E-01
Left paracentral lobule	0.028	0.157	[-0.280 - 0.336]	8.607E-01	9.438E-01
Right paracentral lobule	0.010	0.157	[-0.298 - 0.319]	9.473E-01	9.608E-01
Left insula	0.109	0.239	[-0.360 - 0.579]	6.475E-01	8.786E-01
Right insula	0.055	0.195	[-0.327 - 0.437]	7.783E-01	9.331E-01
Left entorhinal cortex	0.066	0.158	[-0.243 - 0.375]	6.750E-01	8.786E-01
Right entorhinal cortex	0.087	0.188	[-0.282 - 0.456]	6.442E-01	8.786E-01
Left temporal pole	0.018	0.158	[-0.290 - 0.327]	9.071E-01	9.438E-01
Right temporal pole	-0.070	0.157	[-0.379 - 0.239]	6.564E-01	8.786E-01
Left isthmus cingulate cortex	-0.186	0.157	[-0.495 - 0.122]	2.362E-01	8.489E-01
Right isthmus cingulate cortex	-0.071	0.161	[-0.386 - 0.245]	6.616E-01	8.786E-01

Supplementary Table S176. Cortical surface area differences between individuals with major depressive disorder taking antidepressants and healthy comparison subjects controlling for age and sex

	d	Std. Err.	95% CI	*p*-value	FDR *q*-value
Total cortical surface area	-0.200	0.066	[-0.330 - -0.071]	2.458E-03	3.490E-02
Left hemisphere	-0.201	0.065	[-0.329 - -0.072]	2.143E-03	3.490E-02
Right hemisphere	-0.199	0.067	[-0.330 - -0.067]	3.008E-03	3.559E-02
Left superior frontal gyrus	-0.202	0.076	[-0.351 - -0.054]	7.550E-03	4.591E-02
Right superior frontal gyrus	-0.236	0.086	[-0.404 - -0.067]	6.171E-03	4.382E-02
Left pars orbitalis of inferior frontal gyrus	-0.150	0.075	[-0.298 - -0.003]	4.604E-02	1.099E-01
Right pars orbitalis of inferior frontal gyrus	-0.154	0.064	[-0.280 - -0.027]	1.714E-02	6.533E-02
Left precentral gyrus	-0.135	0.075	[-0.283 - 0.013]	7.315E-02	1.514E-01
Right precentral gyrus	-0.094	0.064	[-0.221 - 0.032]	1.424E-01	2.298E-01
Left lingual gyrus	-0.117	0.076	[-0.266 - 0.033]	1.258E-01	2.178E-01
Right lingual gyrus	-0.146	0.070	[-0.283 - -0.009]	3.720E-02	1.016E-01
Left superior temporal gyrus	-0.123	0.065	[-0.251 - 0.004]	5.836E-02	1.256E-01
Right superior temporal gyrus	-0.169	0.065	[-0.296 - -0.043]	8.738E-03	4.772E-02
Left fusiform gyrus	-0.119	0.074	[-0.265 - 0.026]	1.063E-01	1.935E-01
Right fusiform gyrus	-0.181	0.065	[-0.307 - -0.054]	5.105E-03	4.382E-02
Left inferior parietal cortex	-0.188	0.076	[-0.338 - -0.038]	1.382E-02	6.280E-02
Right inferior parietal cortex	-0.132	0.074	[-0.277 - 0.013]	7.464E-02	1.514E-01
Left lateral occipital cortex	-0.110	0.064	[-0.237 - 0.016]	8.734E-02	1.722E-01
Right lateral occipital cortex	-0.168	0.071	[-0.306 - -0.029]	1.748E-02	6.533E-02
Left rostral middle frontal gyrus	-0.129	0.065	[-0.255 - -0.002]	4.644E-02	1.099E-01
Right rostral middle frontal gyrus	-0.101	0.070	[-0.237 - 0.035]	1.465E-01	2.312E-01
Left precuneus	-0.197	0.065	[-0.324 - -0.070]	2.274E-03	3.490E-02
Right precuneus	-0.058	0.067	[-0.188 - 0.073]	3.888E-01	4.525E-01
Left inferior temporal gyrus	-0.128	0.064	[-0.255 - -0.002]	4.618E-02	1.099E-01
Right inferior temporal gyrus	-0.088	0.069	[-0.223 - 0.047]	2.025E-01	2.819E-01
Left lateral orbitofrontal cortex	-0.132	0.064	[-0.258 - -0.005]	4.119E-02	1.083E-01
Right lateral orbitofrontal cortex	-0.036	0.075	[-0.184 - 0.111]	6.311E-01	6.687E-01
Left middle temporal gyrus	-0.151	0.070	[-0.288 - -0.013]	3.245E-02	9.352E-02
Right middle temporal gyrus	-0.165	0.068	[-0.297 - -0.032]	1.504E-02	6.280E-02
Left postcentral gyrus	-0.152	0.067	[-0.283 - -0.021]	2.336E-02	7.899E-02
Right postcentral gyrus	-0.196	0.074	[-0.340 - -0.052]	7.759E-03	4.591E-02
Left medial orbitofrontal cortex	-0.078	0.079	[-0.232 - 0.077]	3.254E-01	4.053E-01
Right medial orbitofrontal cortex	-0.126	0.065	[-0.253 - 0.000]	5.024E-02	1.151E-01
Left cuneus	-0.203	0.074	[-0.348 - -0.059]	5.715E-03	4.382E-02
Right cuneus	-0.210	0.065	[-0.336 - -0.083]	1.181E-03	3.490E-02
Left pars triangularis of inferior frontal gyrus	-0.158	0.065	[-0.284 - -0.031]	1.456E-02	6.280E-02
Right pars triangularis of inferior frontal gyrus	-0.143	0.064	[-0.270 - -0.017]	2.623E-02	8.466E-02
Left superior parietal cortex	-0.102	0.064	[-0.229 - 0.024]	1.127E-01	2.001E-01
Right superior parietal cortex	-0.088	0.064	[-0.214 - 0.039]	1.744E-01	2.580E-01
Left pars opercularis of inferior frontal gyrus	-0.127	0.086	[-0.296 - 0.041]	1.384E-01	2.285E-01
Right pars opercularis of inferior frontal gyrus	-0.066	0.080	[-0.223 - 0.091]	4.085E-01	4.604E-01
Left supramarginal gyrus	-0.065	0.072	[-0.207 - 0.077]	3.715E-01	4.396E-01
Right supramarginal gyrus	-0.092	0.070	[-0.230 - 0.046]	1.899E-01	2.696E-01
Left pericalcarine cortex	-0.157	0.073	[-0.300 - -0.014]	3.102E-02	9.352E-02
Right pericalcarine cortex	-0.230	0.070	[-0.368 - -0.092]	1.083E-03	3.490E-02
Left parahippocampal gyrus	-0.059	0.064	[-0.185 - 0.067]	3.593E-01	4.323E-01
Right parahippocampal gyrus	-0.090	0.068	[-0.223 - 0.043]	1.870E-01	2.696E-01
Left caudal middle frontal gyrus	-0.083	0.067	[-0.215 - 0.049]	2.183E-01	2.980E-01
Right caudal middle frontal gyrus	-0.034	0.072	[-0.175 - 0.108]	6.429E-01	6.713E-01
Left transverse temporal gyrus	-0.055	0.064	[-0.181 - 0.072]	3.959E-01	4.534E-01
Right transverse temporal gyrus	-0.106	0.064	[-0.233 - 0.020]	9.858E-02	1.842E-01
Left banks of superior temporal sulcus	-0.084	0.073	[-0.228 - 0.059]	2.503E-01	3.231E-01
Right banks of superior temporal sulcus	-0.095	0.103	[-0.298 - 0.107]	3.562E-01	4.323E-01
Left caudal anterior cingulate cortex	-0.165	0.065	[-0.291 - -0.038]	1.072E-02	5.436E-02
Right caudal anterior cingulate cortex	-0.091	0.065	[-0.219 - 0.038]	1.661E-01	2.509E-01
Left rostral anterior cingulate cortex	-0.107	0.064	[-0.234 - 0.019]	9.648E-02	1.842E-01
Right rostral anterior cingulate cortex	-0.045	0.077	[-0.196 - 0.106]	5.589E-01	6.013E-01
Left posterior cingulate cortex	-0.097	0.064	[-0.224 - 0.029]	1.306E-01	2.207E-01
Right posterior cingulate cortex	-0.161	0.076	[-0.309 - -0.013]	3.293E-02	9.352E-02
Left frontal pole	-0.128	0.067	[-0.260 - 0.003]	5.527E-02	1.226E-01
Right frontal pole	-0.180	0.064	[-0.307 - -0.054]	5.144E-03	4.382E-02
Left paracentral lobule	-0.038	0.064	[-0.165 - 0.088]	5.513E-01	6.013E-01
Right paracentral lobule	-0.146	0.064	[-0.273 - -0.020]	2.325E-02	7.899E-02
Left insula	-0.075	0.064	[-0.202 - 0.051]	2.420E-01	3.182E-01
Right insula	-0.093	0.079	[-0.247 - 0.062]	2.393E-01	3.182E-01
Left entorhinal cortex	0.001	0.064	[-0.125 - 0.128]	9.833E-01	9.833E-01
Right entorhinal cortex	0.045	0.064	[-0.082 - 0.171]	4.876E-01	5.410E-01
Left temporal pole	-0.091	0.064	[-0.217 - 0.035]	1.584E-01	2.445E-01
Right temporal pole	-0.066	0.064	[-0.193 - 0.060]	3.025E-01	3.836E-01
Left isthmus cingulate cortex	-0.018	0.064	[-0.144 - 0.108]	7.780E-01	7.891E-01
Right isthmus cingulate cortex	0.029	0.071	[-0.110 - 0.169]	6.803E-01	7.000E-01

Supplementary Table S177. Cortical surface area differences between individuals with major depressive disorder taking antidepressants and healthy comparison subjects controlling for age and sex over 21 years of old

	d	Std. Err.	95% CI	*p*-value	FDR *q*-value
Total cortical surface area	-0.192	0.069	[-0.326 - -0.057]	5.185E-03	4.652E-02
Left hemisphere	-0.192	0.068	[-0.325 - -0.058]	4.973E-03	4.652E-02
Right hemisphere	-0.190	0.069	[-0.325 - -0.055]	5.764E-03	4.652E-02
Left superior frontal gyrus	-0.191	0.077	[-0.341 - -0.041]	1.267E-02	6.462E-02
Right superior frontal gyrus	-0.232	0.088	[-0.405 - -0.059]	8.470E-03	5.517E-02
Left pars orbitalis of inferior frontal gyrus	-0.150	0.080	[-0.307 - 0.007]	6.173E-02	1.487E-01
Right pars orbitalis of inferior frontal gyrus	-0.159	0.065	[-0.287 - -0.031]	1.501E-02	6.671E-02
Left precentral gyrus	-0.123	0.080	[-0.280 - 0.035]	1.266E-01	2.178E-01
Right precentral gyrus	-0.089	0.065	[-0.217 - 0.039]	1.729E-01	2.728E-01
Left lingual gyrus	-0.117	0.077	[-0.269 - 0.034]	1.288E-01	2.178E-01
Right lingual gyrus	-0.140	0.069	[-0.274 - -0.005]	4.143E-02	1.201E-01
Left superior temporal gyrus	-0.127	0.068	[-0.260 - 0.007]	6.284E-02	1.487E-01
Right superior temporal gyrus	-0.169	0.068	[-0.301 - -0.036]	1.274E-02	6.462E-02
Left fusiform gyrus	-0.115	0.075	[-0.262 - 0.033]	1.279E-01	2.178E-01
Right fusiform gyrus	-0.180	0.065	[-0.308 - -0.052]	5.898E-03	4.652E-02
Left inferior parietal cortex	-0.185	0.079	[-0.339 - -0.030]	1.894E-02	7.910E-02
Right inferior parietal cortex	-0.137	0.081	[-0.296 - 0.021]	8.920E-02	1.863E-01
Left lateral occipital cortex	-0.100	0.065	[-0.228 - 0.029]	1.279E-01	2.178E-01
Right lateral occipital cortex	-0.159	0.069	[-0.295 - -0.024]	2.089E-02	8.241E-02
Left rostral middle frontal gyrus	-0.126	0.065	[-0.254 - 0.002]	5.382E-02	1.365E-01
Right rostral middle frontal gyrus	-0.095	0.068	[-0.229 - 0.039]	1.649E-01	2.661E-01
Left precuneus	-0.187	0.065	[-0.315 - -0.059]	4.317E-03	4.652E-02
Right precuneus	-0.041	0.069	[-0.176 - 0.093]	5.461E-01	6.130E-01
Left inferior temporal gyrus	-0.118	0.065	[-0.246 - 0.010]	7.171E-02	1.642E-01
Right inferior temporal gyrus	-0.090	0.072	[-0.231 - 0.052]	2.144E-01	3.044E-01
Left lateral orbitofrontal cortex	-0.133	0.065	[-0.261 - -0.005]	4.230E-02	1.201E-01
Right lateral orbitofrontal cortex	-0.032	0.075	[-0.179 - 0.116]	6.737E-01	7.035E-01
Left middle temporal gyrus	-0.149	0.071	[-0.287 - -0.010]	3.536E-02	1.141E-01
Right middle temporal gyrus	-0.169	0.069	[-0.304 - -0.033]	1.503E-02	6.671E-02
Left postcentral gyrus	-0.151	0.069	[-0.286 - -0.017]	2.750E-02	9.764E-02
Right postcentral gyrus	-0.201	0.077	[-0.353 - -0.049]	9.388E-03	5.554E-02
Left medial orbitofrontal cortex	-0.076	0.082	[-0.238 - 0.086]	3.564E-01	4.148E-01
Right medial orbitofrontal cortex	-0.130	0.065	[-0.259 - -0.002]	4.659E-02	1.225E-01
Left cuneus	-0.202	0.073	[-0.345 - -0.058]	5.842E-03	4.652E-02
Right cuneus	-0.204	0.065	[-0.332 - -0.076]	1.841E-03	4.652E-02
Left pars triangularis of inferior frontal gyrus	-0.146	0.071	[-0.285 - -0.007]	3.977E-02	1.201E-01
Right pars triangularis of inferior frontal gyrus	-0.123	0.071	[-0.263 - 0.016]	8.341E-02	1.795E-01
Left superior parietal cortex	-0.088	0.069	[-0.222 - 0.046]	1.989E-01	3.004E-01
Right superior parietal cortex	-0.069	0.065	[-0.198 - 0.059]	2.885E-01	3.636E-01
Left pars opercularis of inferior frontal gyrus	-0.113	0.088	[-0.285 - 0.059]	1.977E-01	3.004E-01
Right pars opercularis of inferior frontal gyrus	-0.051	0.089	[-0.226 - 0.124]	5.683E-01	6.130E-01
Left supramarginal gyrus	-0.068	0.073	[-0.212 - 0.076]	3.538E-01	4.148E-01
Right supramarginal gyrus	-0.101	0.066	[-0.231 - 0.028]	1.253E-01	2.178E-01
Left pericalcarine cortex	-0.150	0.071	[-0.290 - -0.011]	3.444E-02	1.141E-01
Right pericalcarine cortex	-0.224	0.069	[-0.359 - -0.089]	1.145E-03	4.652E-02
Left parahippocampal gyrus	-0.069	0.065	[-0.197 - 0.059]	2.919E-01	3.636E-01
Right parahippocampal gyrus	-0.097	0.065	[-0.225 - 0.031]	1.386E-01	2.289E-01
Left caudal middle frontal gyrus	-0.072	0.065	[-0.200 - 0.056]	2.729E-01	3.608E-01
Right caudal middle frontal gyrus	-0.023	0.073	[-0.166 - 0.120]	7.505E-01	7.612E-01
Left transverse temporal gyrus	-0.061	0.065	[-0.190 - 0.067]	3.471E-01	4.148E-01
Right transverse temporal gyrus	-0.100	0.065	[-0.228 - 0.028]	1.271E-01	2.178E-01
Left banks of superior temporal sulcus	-0.095	0.076	[-0.244 - 0.053]	2.079E-01	3.044E-01
Right banks of superior temporal sulcus	-0.111	0.104	[-0.314 - 0.092]	2.849E-01	3.636E-01
Left caudal anterior cingulate cortex	-0.172	0.065	[-0.300 - -0.044]	8.548E-03	5.517E-02
Right caudal anterior cingulate cortex	-0.076	0.070	[-0.213 - 0.061]	2.744E-01	3.608E-01
Left rostral anterior cingulate cortex	-0.104	0.065	[-0.232 - 0.024]	1.112E-01	2.178E-01
Right rostral anterior cingulate cortex	-0.042	0.080	[-0.198 - 0.114]	5.993E-01	6.351E-01
Left posterior cingulate cortex	-0.106	0.065	[-0.234 - 0.022]	1.038E-01	2.106E-01
Right posterior cingulate cortex	-0.157	0.078	[-0.310 - -0.004]	4.399E-02	1.201E-01
Left frontal pole	-0.120	0.069	[-0.256 - 0.015]	8.160E-02	1.795E-01
Right frontal pole	-0.181	0.065	[-0.309 - -0.053]	5.669E-03	4.652E-02
Left paracentral lobule	-0.038	0.065	[-0.166 - 0.090]	5.587E-01	6.130E-01
Right paracentral lobule	-0.145	0.065	[-0.273 - -0.017]	2.692E-02	9.764E-02
Left insula	-0.077	0.066	[-0.206 - 0.053]	2.460E-01	3.382E-01
Right insula	-0.099	0.079	[-0.254 - 0.057]	2.144E-01	3.044E-01
Left entorhinal cortex	-0.024	0.066	[-0.154 - 0.106]	7.161E-01	7.368E-01
Right entorhinal cortex	0.049	0.065	[-0.079 - 0.177]	4.547E-01	5.207E-01
Left temporal pole	-0.076	0.065	[-0.204 - 0.053]	2.477E-01	3.382E-01
Right temporal pole	-0.062	0.065	[-0.190 - 0.066]	3.429E-01	4.148E-01
Left isthmus cingulate cortex	-0.012	0.065	[-0.140 - 0.116]	8.520E-01	8.520E-01
Right isthmus cingulate cortex	0.044	0.077	[-0.107 - 0.195]	5.698E-01	6.130E-01

Supplementary Table S178. Cortical surface area differences between individuals with major depressive disorder taking antidepressants and individuals with major depressive disorder not taking antidepressants controlling for age and sex

	d	Std. Err.	95% CI	*p*-value	FDR *q*-value
Total cortical surface area	0.133	0.133	[-0.128 - 0.394]	3.168E-01	8.996E-01
Left hemisphere	0.132	0.133	[-0.129 - 0.393]	3.216E-01	8.996E-01
Right hemisphere	0.133	0.133	[-0.128 - 0.394]	3.174E-01	8.996E-01
Left superior frontal gyrus	0.071	0.133	[-0.190 - 0.332]	5.951E-01	8.996E-01
Right superior frontal gyrus	0.159	0.133	[-0.102 - 0.421]	2.321E-01	8.996E-01
Left pars orbitalis of inferior frontal gyrus	0.032	0.133	[-0.229 - 0.293]	8.124E-01	9.691E-01
Right pars orbitalis of inferior frontal gyrus	0.120	0.133	[-0.141 - 0.381]	3.673E-01	8.996E-01
Left precentral gyrus	0.041	0.133	[-0.220 - 0.302]	7.572E-01	9.601E-01
Right precentral gyrus	0.068	0.133	[-0.193 - 0.329]	6.082E-01	8.996E-01
Left lingual gyrus	0.048	0.203	[-0.349 - 0.445]	8.121E-01	9.691E-01
Right lingual gyrus	0.062	0.194	[-0.319 - 0.443]	7.496E-01	9.601E-01
Left superior temporal gyrus	0.140	0.163	[-0.179 - 0.459]	3.904E-01	8.996E-01
Right superior temporal gyrus	0.046	0.133	[-0.214 - 0.307]	7.278E-01	9.569E-01
Left fusiform gyrus	0.095	0.158	[-0.214 - 0.404]	5.476E-01	8.996E-01
Right fusiform gyrus	0.134	0.133	[-0.127 - 0.395]	3.159E-01	8.996E-01
Left inferior parietal cortex	-0.102	0.133	[-0.363 - 0.160]	4.455E-01	8.996E-01
Right inferior parietal cortex	0.135	0.133	[-0.126 - 0.397]	3.092E-01	8.996E-01
Left lateral occipital cortex	0.133	0.179	[-0.218 - 0.485]	4.581E-01	8.996E-01
Right lateral occipital cortex	0.279	0.143	[-0.001 - 0.560]	5.110E-02	8.996E-01
Left rostral middle frontal gyrus	0.248	0.133	[-0.014 - 0.510]	6.322E-02	8.996E-01
Right rostral middle frontal gyrus	0.056	0.133	[-0.204 - 0.317]	6.715E-01	8.996E-01
Left precuneus	0.160	0.133	[-0.101 - 0.422]	2.293E-01	8.996E-01
Right precuneus	0.144	0.137	[-0.125 - 0.412]	2.939E-01	8.996E-01
Left inferior temporal gyrus	0.009	0.133	[-0.252 - 0.269]	9.489E-01	9.741E-01
Right inferior temporal gyrus	0.002	0.198	[-0.386 - 0.391]	9.906E-01	9.906E-01
Left lateral orbitofrontal cortex	0.129	0.133	[-0.132 - 0.390]	3.323E-01	8.996E-01
Right lateral orbitofrontal cortex	0.263	0.134	[0.001 - 0.525]	4.917E-02	8.996E-01
Left middle temporal gyrus	0.108	0.134	[-0.154 - 0.369]	4.203E-01	8.996E-01
Right middle temporal gyrus	0.156	0.142	[-0.123 - 0.435]	2.738E-01	8.996E-01
Left postcentral gyrus	0.108	0.133	[-0.153 - 0.369]	4.165E-01	8.996E-01
Right postcentral gyrus	0.010	0.133	[-0.251 - 0.271]	9.415E-01	9.741E-01
Left medial orbitofrontal cortex	0.211	0.244	[-0.267 - 0.688]	3.871E-01	8.996E-01
Right medial orbitofrontal cortex	0.169	0.133	[-0.092 - 0.430]	2.041E-01	8.996E-01
Left cuneus	0.122	0.183	[-0.236 - 0.480]	5.034E-01	8.996E-01
Right cuneus	-0.010	0.133	[-0.271 - 0.251]	9.379E-01	9.741E-01
Left pars triangularis of inferior frontal gyrus	0.093	0.133	[-0.168 - 0.354]	4.863E-01	8.996E-01
Right pars triangularis of inferior frontal gyrus	0.117	0.133	[-0.144 - 0.378]	3.787E-01	8.996E-01
Left superior parietal cortex	0.134	0.238	[-0.333 - 0.601]	5.728E-01	8.996E-01
Right superior parietal cortex	0.007	0.133	[-0.254 - 0.268]	9.603E-01	9.741E-01
Left pars opercularis of inferior frontal gyrus	-0.140	0.179	[-0.490 - 0.211]	4.348E-01	8.996E-01
Right pars opercularis of inferior frontal gyrus	0.093	0.133	[-0.168 - 0.354]	4.868E-01	8.996E-01
Left supramarginal gyrus	0.207	0.133	[-0.055 - 0.468]	1.215E-01	8.996E-01
Right supramarginal gyrus	0.089	0.133	[-0.171 - 0.350]	5.013E-01	8.996E-01
Left pericalcarine cortex	0.017	0.133	[-0.245 - 0.278]	9.008E-01	9.691E-01
Right pericalcarine cortex	-0.102	0.142	[-0.380 - 0.177]	4.735E-01	8.996E-01
Left parahippocampal gyrus	-0.128	0.176	[-0.472 - 0.216]	4.653E-01	8.996E-01
Right parahippocampal gyrus	0.065	0.133	[-0.196 - 0.326]	6.275E-01	8.996E-01
Left caudal middle frontal gyrus	0.143	0.134	[-0.119 - 0.405]	2.837E-01	8.996E-01
Right caudal middle frontal gyrus	-0.023	0.167	[-0.350 - 0.303]	8.881E-01	9.691E-01
Left transverse temporal gyrus	0.166	0.133	[-0.095 - 0.428]	2.118E-01	8.996E-01
Right transverse temporal gyrus	0.075	0.133	[-0.186 - 0.336]	5.733E-01	8.996E-01
Left banks of superior temporal sulcus	0.088	0.133	[-0.173 - 0.349]	5.102E-01	8.996E-01
Right banks of superior temporal sulcus	0.017	0.133	[-0.244 - 0.278]	8.981E-01	9.691E-01
Left caudal anterior cingulate cortex	-0.049	0.290	[-0.618 - 0.520]	8.671E-01	9.691E-01
Right caudal anterior cingulate cortex	0.207	0.133	[-0.055 - 0.468]	1.214E-01	8.996E-01
Left rostral anterior cingulate cortex	0.095	0.214	[-0.324 - 0.515]	6.564E-01	8.996E-01
Right rostral anterior cingulate cortex	0.153	0.195	[-0.228 - 0.535]	4.308E-01	8.996E-01
Left posterior cingulate cortex	-0.078	0.140	[-0.353 - 0.197]	5.773E-01	8.996E-01
Right posterior cingulate cortex	0.019	0.133	[-0.242 - 0.280]	8.866E-01	9.691E-01
Left frontal pole	0.147	0.149	[-0.144 - 0.439]	3.221E-01	8.996E-01
Right frontal pole	-0.071	0.160	[-0.385 - 0.242]	6.554E-01	8.996E-01
Left paracentral lobule	-0.058	0.133	[-0.320 - 0.203]	6.632E-01	8.996E-01
Right paracentral lobule	-0.062	0.133	[-0.323 - 0.199]	6.416E-01	8.996E-01
Left insula	-0.035	0.133	[-0.297 - 0.226]	7.905E-01	9.691E-01
Right insula	-0.118	0.192	[-0.495 - 0.259]	5.389E-01	8.996E-01
Left entorhinal cortex	0.069	0.139	[-0.203 - 0.341]	6.190E-01	8.996E-01
Right entorhinal cortex	0.019	0.133	[-0.242 - 0.280]	8.867E-01	9.691E-01
Left temporal pole	-0.185	0.133	[-0.446 - 0.077]	1.661E-01	8.996E-01
Right temporal pole	-0.127	0.190	[-0.499 - 0.245]	5.028E-01	8.996E-01
Left isthmus cingulate cortex	0.041	0.187	[-0.325 - 0.407]	8.261E-01	9.691E-01
Right isthmus cingulate cortex	0.158	0.133	[-0.103 - 0.419]	2.362E-01	8.996E-01

Supplementary Table S179. Cortical surface area differences between individuals with major depressive disorder taking antidepressants and individuals with major depressive disorder not taking antidepressants controlling for age and sex over 21 years of old

	d	Std. Err.	95% CI	*p*-value	FDR *q*-value
Total cortical surface area	0.112	0.142	[-0.166 - 0.390]	4.289E-01	9.591E-01
Left hemisphere	0.103	0.142	[-0.174 - 0.381]	4.663E-01	9.591E-01
Right hemisphere	0.119	0.142	[-0.158 - 0.397]	3.995E-01	9.591E-01
Left superior frontal gyrus	0.081	0.141	[-0.196 - 0.358]	5.661E-01	9.591E-01
Right superior frontal gyrus	0.164	0.142	[-0.113 - 0.442]	2.463E-01	9.591E-01
Left pars orbitalis of inferior frontal gyrus	0.048	0.142	[-0.229 - 0.326]	7.338E-01	9.591E-01
Right pars orbitalis of inferior frontal gyrus	0.123	0.142	[-0.155 - 0.401]	3.870E-01	9.591E-01
Left precentral gyrus	0.043	0.142	[-0.234 - 0.321]	7.589E-01	9.591E-01
Right precentral gyrus	0.098	0.142	[-0.180 - 0.375]	4.902E-01	9.591E-01
Left lingual gyrus	-0.004	0.246	[-0.486 - 0.478]	9.877E-01	9.877E-01
Right lingual gyrus	0.105	0.225	[-0.335 - 0.546]	6.400E-01	9.591E-01
Left superior temporal gyrus	0.174	0.208	[-0.233 - 0.582]	4.013E-01	9.591E-01
Right superior temporal gyrus	0.017	0.142	[-0.260 - 0.295]	9.032E-01	9.591E-01
Left fusiform gyrus	0.130	0.171	[-0.205 - 0.465]	4.463E-01	9.591E-01
Right fusiform gyrus	0.092	0.142	[-0.185 - 0.370]	5.144E-01	9.591E-01
Left inferior parietal cortex	-0.096	0.142	[-0.374 - 0.182]	4.978E-01	9.591E-01
Right inferior parietal cortex	0.107	0.142	[-0.171 - 0.385]	4.501E-01	9.591E-01
Left lateral occipital cortex	0.117	0.200	[-0.275 - 0.509]	5.599E-01	9.591E-01
Right lateral occipital cortex	0.309	0.164	[-0.012 - 0.631]	5.936E-02	9.591E-01
Left rostral middle frontal gyrus	0.158	0.142	[-0.119 - 0.436]	2.632E-01	9.591E-01
Right rostral middle frontal gyrus	0.042	0.141	[-0.235 - 0.319]	7.673E-01	9.591E-01
Left precuneus	0.147	0.142	[-0.131 - 0.424]	3.001E-01	9.591E-01
Right precuneus	0.143	0.142	[-0.135 - 0.420]	3.136E-01	9.591E-01
Left inferior temporal gyrus	-0.031	0.142	[-0.308 - 0.247]	8.291E-01	9.591E-01
Right inferior temporal gyrus	0.040	0.207	[-0.367 - 0.446]	8.484E-01	9.591E-01
Left lateral orbitofrontal cortex	0.078	0.159	[-0.234 - 0.390]	6.249E-01	9.591E-01
Right lateral orbitofrontal cortex	0.165	0.142	[-0.113 - 0.443]	2.457E-01	9.591E-01
Left middle temporal gyrus	0.027	0.142	[-0.251 - 0.305]	8.487E-01	9.591E-01
Right middle temporal gyrus	0.189	0.142	[-0.089 - 0.467]	1.826E-01	9.591E-01
Left postcentral gyrus	0.117	0.142	[-0.161 - 0.394]	4.104E-01	9.591E-01
Right postcentral gyrus	0.022	0.142	[-0.256 - 0.300]	8.777E-01	9.591E-01
Left medial orbitofrontal cortex	0.234	0.273	[-0.301 - 0.768]	3.918E-01	9.591E-01
Right medial orbitofrontal cortex	0.106	0.142	[-0.172 - 0.383]	4.553E-01	9.591E-01
Left cuneus	-0.048	0.142	[-0.326 - 0.230]	7.348E-01	9.591E-01
Right cuneus	-0.030	0.142	[-0.308 - 0.248]	8.340E-01	9.591E-01
Left pars triangularis of inferior frontal gyrus	0.137	0.142	[-0.141 - 0.414]	3.354E-01	9.591E-01
Right pars triangularis of inferior frontal gyrus	0.127	0.142	[-0.151 - 0.404]	3.711E-01	9.591E-01
Left superior parietal cortex	0.123	0.282	[-0.429 - 0.675]	6.625E-01	9.591E-01
Right superior parietal cortex	-0.028	0.142	[-0.306 - 0.250]	8.426E-01	9.591E-01
Left pars opercularis of inferior frontal gyrus	-0.088	0.184	[-0.448 - 0.271]	6.299E-01	9.591E-01
Right pars opercularis of inferior frontal gyrus	0.145	0.142	[-0.133 - 0.423]	3.062E-01	9.591E-01
Left supramarginal gyrus	0.180	0.142	[-0.098 - 0.458]	2.054E-01	9.591E-01
Right supramarginal gyrus	0.033	0.142	[-0.244 - 0.310]	8.155E-01	9.591E-01
Left pericalcarine cortex	-0.048	0.142	[-0.325 - 0.230]	7.368E-01	9.591E-01
Right pericalcarine cortex	-0.114	0.142	[-0.392 - 0.164]	4.220E-01	9.591E-01
Left parahippocampal gyrus	-0.148	0.209	[-0.558 - 0.262]	4.800E-01	9.591E-01
Right parahippocampal gyrus	0.067	0.159	[-0.244 - 0.378]	6.712E-01	9.591E-01
Left caudal middle frontal gyrus	0.193	0.142	[-0.085 - 0.470]	1.740E-01	9.591E-01
Right caudal middle frontal gyrus	-0.031	0.155	[-0.334 - 0.273]	8.432E-01	9.591E-01
Left transverse temporal gyrus	0.154	0.181	[-0.200 - 0.508]	3.935E-01	9.591E-01
Right transverse temporal gyrus	0.016	0.142	[-0.262 - 0.293]	9.127E-01	9.591E-01
Left banks of superior temporal sulcus	0.076	0.142	[-0.202 - 0.353]	5.934E-01	9.591E-01
Right banks of superior temporal sulcus	-0.053	0.142	[-0.330 - 0.225]	7.103E-01	9.591E-01
Left caudal anterior cingulate cortex	-0.015	0.337	[-0.675 - 0.645]	9.650E-01	9.788E-01
Right caudal anterior cingulate cortex	0.225	0.142	[-0.053 - 0.503]	1.133E-01	9.591E-01
Left rostral anterior cingulate cortex	0.118	0.243	[-0.359 - 0.595]	6.286E-01	9.591E-01
Right rostral anterior cingulate cortex	0.230	0.149	[-0.063 - 0.522]	1.235E-01	9.591E-01
Left posterior cingulate cortex	-0.122	0.188	[-0.491 - 0.247]	5.177E-01	9.591E-01
Right posterior cingulate cortex	0.026	0.142	[-0.252 - 0.304]	8.539E-01	9.591E-01
Left frontal pole	0.120	0.184	[-0.241 - 0.481]	5.152E-01	9.591E-01
Right frontal pole	-0.025	0.168	[-0.354 - 0.305]	8.837E-01	9.591E-01
Left paracentral lobule	-0.063	0.142	[-0.340 - 0.215]	6.583E-01	9.591E-01
Right paracentral lobule	-0.086	0.142	[-0.364 - 0.191]	5.418E-01	9.591E-01
Left insula	-0.012	0.142	[-0.290 - 0.266]	9.311E-01	9.591E-01
Right insula	-0.085	0.189	[-0.455 - 0.284]	6.513E-01	9.591E-01
Left entorhinal cortex	0.031	0.142	[-0.246 - 0.309]	8.263E-01	9.591E-01
Right entorhinal cortex	0.012	0.141	[-0.265 - 0.289]	9.321E-01	9.591E-01
Left temporal pole	-0.162	0.142	[-0.439 - 0.116]	2.531E-01	9.591E-01
Right temporal pole	-0.095	0.142	[-0.373 - 0.183]	5.032E-01	9.591E-01
Left isthmus cingulate cortex	0.145	0.142	[-0.133 - 0.423]	3.062E-01	9.591E-01
Right isthmus cingulate cortex	0.156	0.142	[-0.122 - 0.434]	2.716E-01	9.591E-01

Supplementary Table S180. Cortical surface area differences between individuals with major depressive disorder not taking antipsychotics and healthy comparison subjects controlling for age and sex

	d	Std. Err.	95% CI	*p*-value	FDR *q*-value
Total cortical surface area	-0.214	0.067	[-0.346 - -0.082]	1.475E-03	1.833E-02
Left hemisphere	-0.217	0.067	[-0.349 - -0.085]	1.294E-03	1.833E-02
Right hemisphere	-0.210	0.067	[-0.342 - -0.078]	1.848E-03	1.875E-02
Left superior frontal gyrus	-0.238	0.073	[-0.380 - -0.095]	1.073E-03	1.833E-02
Right superior frontal gyrus	-0.238	0.084	[-0.403 - -0.074]	4.561E-03	3.238E-02
Left pars orbitalis of inferior frontal gyrus	-0.181	0.067	[-0.313 - -0.049]	7.154E-03	3.628E-02
Right pars orbitalis of inferior frontal gyrus	-0.201	0.067	[-0.333 - -0.069]	2.793E-03	2.479E-02
Left precentral gyrus	-0.181	0.067	[-0.313 - -0.050]	7.006E-03	3.628E-02
Right precentral gyrus	-0.107	0.067	[-0.238 - 0.025]	1.124E-01	1.813E-01
Left lingual gyrus	-0.127	0.069	[-0.262 - 0.007]	6.391E-02	1.266E-01
Right lingual gyrus	-0.159	0.067	[-0.290 - -0.027]	1.808E-02	6.112E-02
Left superior temporal gyrus	-0.119	0.067	[-0.251 - 0.013]	7.632E-02	1.376E-01
Right superior temporal gyrus	-0.170	0.081	[-0.329 - -0.011]	3.632E-02	9.551E-02
Left fusiform gyrus	-0.137	0.083	[-0.299 - 0.025]	9.723E-02	1.644E-01
Right fusiform gyrus	-0.194	0.067	[-0.326 - -0.062]	4.007E-03	3.161E-02
Left inferior parietal cortex	-0.174	0.076	[-0.323 - -0.025]	2.198E-02	6.785E-02
Right inferior parietal cortex	-0.091	0.080	[-0.249 - 0.067]	2.586E-01	3.166E-01
Left lateral occipital cortex	-0.076	0.067	[-0.208 - 0.055]	2.559E-01	3.166E-01
Right lateral occipital cortex	-0.173	0.067	[-0.305 - -0.041]	1.006E-02	4.676E-02
Left rostral middle frontal gyrus	-0.163	0.082	[-0.324 - -0.002]	4.753E-02	1.125E-01
Right rostral middle frontal gyrus	-0.094	0.085	[-0.261 - 0.073]	2.679E-01	3.223E-01
Left precuneus	-0.220	0.067	[-0.352 - -0.088]	1.062E-03	1.833E-02
Right precuneus	-0.067	0.067	[-0.198 - 0.065]	3.205E-01	3.710E-01
Left inferior temporal gyrus	-0.123	0.067	[-0.255 - 0.008]	6.600E-02	1.266E-01
Right inferior temporal gyrus	-0.125	0.067	[-0.257 - 0.007]	6.248E-02	1.266E-01
Left lateral orbitofrontal cortex	-0.182	0.067	[-0.314 - -0.050]	6.761E-03	3.628E-02
Right lateral orbitofrontal cortex	-0.098	0.075	[-0.245 - 0.049]	1.915E-01	2.719E-01
Left middle temporal gyrus	-0.150	0.067	[-0.282 - -0.018]	2.573E-02	7.613E-02
Right middle temporal gyrus	-0.169	0.067	[-0.300 - -0.037]	1.224E-02	4.830E-02
Left postcentral gyrus	-0.104	0.082	[-0.264 - 0.057]	2.048E-01	2.744E-01
Right postcentral gyrus	-0.182	0.085	[-0.347 - -0.016]	3.193E-02	8.720E-02
Left medial orbitofrontal cortex	-0.152	0.086	[-0.322 - 0.017]	7.750E-02	1.376E-01
Right medial orbitofrontal cortex	-0.163	0.069	[-0.298 - -0.028]	1.807E-02	6.112E-02
Left cuneus	-0.197	0.082	[-0.358 - -0.036]	1.666E-02	6.112E-02
Right cuneus	-0.213	0.067	[-0.345 - -0.081]	1.549E-03	1.833E-02
Left pars triangularis of inferior frontal gyrus	-0.203	0.079	[-0.359 - -0.047]	1.054E-02	4.676E-02
Right pars triangularis of inferior frontal gyrus	-0.182	0.067	[-0.313 - -0.050]	6.893E-03	3.628E-02
Left superior parietal cortex	-0.137	0.067	[-0.268 - -0.005]	4.212E-02	1.031E-01
Right superior parietal cortex	-0.099	0.067	[-0.230 - 0.033]	1.424E-01	2.106E-01
Left pars opercularis of inferior frontal gyrus	-0.127	0.080	[-0.284 - 0.030]	1.121E-01	1.813E-01
Right pars opercularis of inferior frontal gyrus	-0.091	0.070	[-0.228 - 0.047]	1.964E-01	2.735E-01
Left supramarginal gyrus	-0.091	0.067	[-0.223 - 0.041]	1.754E-01	2.542E-01
Right supramarginal gyrus	-0.104	0.070	[-0.240 - 0.033]	1.356E-01	2.093E-01
Left pericalcarine cortex	-0.159	0.068	[-0.293 - -0.025]	2.010E-02	6.487E-02
Right pericalcarine cortex	-0.241	0.067	[-0.373 - -0.109]	3.384E-04	1.833E-02
Left parahippocampal gyrus	-0.014	0.072	[-0.154 - 0.127]	8.467E-01	8.588E-01
Right parahippocampal gyrus	-0.066	0.067	[-0.198 - 0.065]	3.240E-01	3.710E-01
Left caudal middle frontal gyrus	-0.103	0.070	[-0.240 - 0.034]	1.407E-01	2.106E-01
Right caudal middle frontal gyrus	-0.058	0.067	[-0.190 - 0.074]	3.874E-01	4.366E-01
Left transverse temporal gyrus	-0.083	0.067	[-0.214 - 0.049]	2.186E-01	2.874E-01
Right transverse temporal gyrus	-0.125	0.067	[-0.257 - 0.006]	6.220E-02	1.266E-01
Left banks of superior temporal sulcus	-0.131	0.067	[-0.263 - 0.001]	5.093E-02	1.167E-01
Right banks of superior temporal sulcus	-0.063	0.097	[-0.254 - 0.128]	5.190E-01	5.500E-01
Left caudal anterior cingulate cortex	-0.113	0.067	[-0.244 - 0.019]	9.392E-02	1.626E-01
Right caudal anterior cingulate cortex	-0.166	0.081	[-0.325 - -0.006]	4.211E-02	1.031E-01
Left rostral anterior cingulate cortex	-0.121	0.067	[-0.253 - 0.011]	7.139E-02	1.334E-01
Right rostral anterior cingulate cortex	-0.060	0.077	[-0.211 - 0.091]	4.369E-01	4.847E-01
Left posterior cingulate cortex	-0.073	0.067	[-0.204 - 0.059]	2.801E-01	3.315E-01
Right posterior cingulate cortex	-0.180	0.071	[-0.320 - -0.041]	1.138E-02	4.754E-02
Left frontal pole	-0.146	0.067	[-0.278 - -0.015]	2.953E-02	8.387E-02
Right frontal pole	-0.125	0.067	[-0.257 - 0.007]	6.247E-02	1.266E-01
Left paracentral lobule	-0.038	0.067	[-0.170 - 0.093]	5.684E-01	5.935E-01
Right paracentral lobule	-0.124	0.067	[-0.256 - 0.008]	6.466E-02	1.266E-01
Left insula	-0.080	0.067	[-0.212 - 0.052]	2.346E-01	3.028E-01
Right insula	-0.111	0.073	[-0.254 - 0.031]	1.259E-01	1.986E-01
Left entorhinal cortex	-0.042	0.089	[-0.217 - 0.132]	6.354E-01	6.538E-01
Right entorhinal cortex	0.049	0.070	[-0.088 - 0.186]	4.815E-01	5.180E-01
Left temporal pole	-0.085	0.067	[-0.217 - 0.046]	2.040E-01	2.744E-01
Right temporal pole	-0.047	0.067	[-0.179 - 0.084]	4.799E-01	5.180E-01
Left isthmus cingulate cortex	-0.076	0.067	[-0.208 - 0.055]	2.559E-01	3.166E-01
Right isthmus cingulate cortex	0.003	0.067	[-0.129 - 0.134]	9.696E-01	9.696E-01

Supplementary Table S181. Cortical surface area differences between individuals with major depressive disorder not taking antipsychotics and healthy comparison subjects controlling for age and sex over 21 years of old

	d	Std. Err.	95% CI	*p*-value	FDR *q*-value
Total cortical surface area	-0.189	0.069	[-0.324 - -0.053]	6.439E-03	6.130E-02
Left hemisphere	-0.191	0.070	[-0.328 - -0.054]	6.277E-03	6.130E-02
Right hemisphere	-0.185	0.069	[-0.320 - -0.049]	7.678E-03	6.130E-02
Left superior frontal gyrus	-0.197	0.069	[-0.333 - -0.061]	4.421E-03	6.130E-02
Right superior frontal gyrus	-0.213	0.085	[-0.380 - -0.046]	1.261E-02	7.790E-02
Left pars orbitalis of inferior frontal gyrus	-0.163	0.069	[-0.298 - -0.027]	1.870E-02	8.299E-02
Right pars orbitalis of inferior frontal gyrus	-0.197	0.069	[-0.332 - -0.061]	4.379E-03	6.130E-02
Left precentral gyrus	-0.159	0.069	[-0.295 - -0.024]	2.137E-02	8.924E-02
Right precentral gyrus	-0.086	0.069	[-0.221 - 0.049]	2.133E-01	3.292E-01
Left lingual gyrus	-0.121	0.080	[-0.277 - 0.036]	1.300E-01	2.319E-01
Right lingual gyrus	-0.146	0.069	[-0.282 - -0.011]	3.438E-02	1.300E-01
Left superior temporal gyrus	-0.105	0.069	[-0.240 - 0.031]	1.307E-01	2.319E-01
Right superior temporal gyrus	-0.156	0.090	[-0.332 - 0.020]	8.143E-02	1.984E-01
Left fusiform gyrus	-0.142	0.085	[-0.309 - 0.025]	9.584E-02	2.016E-01
Right fusiform gyrus	-0.172	0.069	[-0.307 - -0.036]	1.317E-02	7.790E-02
Left inferior parietal cortex	-0.171	0.088	[-0.345 - 0.002]	5.277E-02	1.784E-01
Right inferior parietal cortex	-0.089	0.091	[-0.268 - 0.089]	3.274E-01	4.078E-01
Left lateral occipital cortex	-0.063	0.069	[-0.199 - 0.072]	3.605E-01	4.272E-01
Right lateral occipital cortex	-0.163	0.069	[-0.299 - -0.028]	1.838E-02	8.299E-02
Left rostral middle frontal gyrus	-0.133	0.086	[-0.302 - 0.036]	1.224E-01	2.319E-01
Right rostral middle frontal gyrus	-0.066	0.082	[-0.226 - 0.095]	4.231E-01	4.845E-01
Left precuneus	-0.210	0.069	[-0.345 - -0.074]	2.429E-03	6.123E-02
Right precuneus	-0.051	0.069	[-0.186 - 0.085]	4.617E-01	5.188E-01
Left inferior temporal gyrus	-0.114	0.069	[-0.250 - 0.021]	9.755E-02	2.016E-01
Right inferior temporal gyrus	-0.124	0.069	[-0.259 - 0.012]	7.357E-02	1.935E-01
Left lateral orbitofrontal cortex	-0.163	0.069	[-0.298 - -0.027]	1.869E-02	8.299E-02
Right lateral orbitofrontal cortex	-0.056	0.069	[-0.191 - 0.080]	4.195E-01	4.845E-01
Left middle temporal gyrus	-0.125	0.069	[-0.261 - 0.010]	6.956E-02	1.900E-01
Right middle temporal gyrus	-0.184	0.069	[-0.320 - -0.049]	7.770E-03	6.130E-02
Left postcentral gyrus	-0.104	0.088	[-0.276 - 0.068]	2.348E-01	3.530E-01
Right postcentral gyrus	-0.176	0.094	[-0.362 - 0.009]	6.199E-02	1.834E-01
Left medial orbitofrontal cortex	-0.122	0.094	[-0.307 - 0.062]	1.945E-01	3.069E-01
Right medial orbitofrontal cortex	-0.144	0.079	[-0.298 - 0.011]	6.884E-02	1.900E-01
Left cuneus	-0.186	0.088	[-0.359 - -0.013]	3.480E-02	1.300E-01
Right cuneus	-0.209	0.069	[-0.344 - -0.073]	2.587E-03	6.123E-02
Left pars triangularis of inferior frontal gyrus	-0.173	0.069	[-0.309 - -0.037]	1.246E-02	7.790E-02
Right pars triangularis of inferior frontal gyrus	-0.133	0.069	[-0.269 - 0.003]	5.570E-02	1.798E-01
Left superior parietal cortex	-0.120	0.069	[-0.256 - 0.015]	8.216E-02	1.984E-01
Right superior parietal cortex	-0.079	0.069	[-0.214 - 0.057]	2.536E-01	3.530E-01
Left pars opercularis of inferior frontal gyrus	-0.099	0.084	[-0.264 - 0.067]	2.428E-01	3.530E-01
Right pars opercularis of inferior frontal gyrus	-0.093	0.084	[-0.258 - 0.071]	2.645E-01	3.611E-01
Left supramarginal gyrus	-0.080	0.069	[-0.215 - 0.056]	2.492E-01	3.530E-01
Right supramarginal gyrus	-0.097	0.069	[-0.233 - 0.038]	1.595E-01	2.697E-01
Left pericalcarine cortex	-0.142	0.075	[-0.290 - 0.006]	6.020E-02	1.834E-01
Right pericalcarine cortex	-0.232	0.069	[-0.368 - -0.097]	7.838E-04	5.565E-02
Left parahippocampal gyrus	-0.029	0.069	[-0.164 - 0.107]	6.789E-01	6.986E-01
Right parahippocampal gyrus	-0.076	0.071	[-0.215 - 0.063]	2.824E-01	3.760E-01
Left caudal middle frontal gyrus	-0.087	0.075	[-0.233 - 0.060]	2.460E-01	3.530E-01
Right caudal middle frontal gyrus	-0.050	0.070	[-0.186 - 0.087]	4.749E-01	5.188E-01
Left transverse temporal gyrus	-0.092	0.069	[-0.227 - 0.043]	1.825E-01	2.945E-01
Right transverse temporal gyrus	-0.118	0.069	[-0.254 - 0.017]	8.662E-02	1.984E-01
Left banks of superior temporal sulcus	-0.139	0.069	[-0.274 - -0.003]	4.492E-02	1.595E-01
Right banks of superior temporal sulcus	-0.076	0.106	[-0.283 - 0.131]	4.700E-01	5.188E-01
Left caudal anterior cingulate cortex	-0.116	0.069	[-0.251 - 0.020]	9.416E-02	2.016E-01
Right caudal anterior cingulate cortex	-0.134	0.089	[-0.308 - 0.039]	1.295E-01	2.319E-01
Left rostral anterior cingulate cortex	-0.113	0.069	[-0.248 - 0.023]	1.022E-01	2.016E-01
Right rostral anterior cingulate cortex	-0.051	0.088	[-0.224 - 0.122]	5.626E-01	5.962E-01
Left posterior cingulate cortex	-0.066	0.069	[-0.202 - 0.069]	3.357E-01	4.110E-01
Right posterior cingulate cortex	-0.186	0.078	[-0.338 - -0.034]	1.670E-02	8.299E-02
Left frontal pole	-0.126	0.073	[-0.270 - 0.017]	8.518E-02	1.984E-01
Right frontal pole	-0.122	0.074	[-0.267 - 0.024]	1.006E-01	2.016E-01
Left paracentral lobule	-0.022	0.069	[-0.157 - 0.113]	7.491E-01	7.491E-01
Right paracentral lobule	-0.099	0.069	[-0.234 - 0.037]	1.537E-01	2.661E-01
Left insula	-0.063	0.069	[-0.199 - 0.072]	3.610E-01	4.272E-01
Right insula	-0.107	0.077	[-0.258 - 0.044]	1.634E-01	2.698E-01
Left entorhinal cortex	-0.049	0.091	[-0.228 - 0.129]	5.869E-01	6.128E-01
Right entorhinal cortex	0.046	0.077	[-0.105 - 0.197]	5.514E-01	5.931E-01
Left temporal pole	-0.074	0.069	[-0.209 - 0.062]	2.879E-01	3.760E-01
Right temporal pole	-0.070	0.069	[-0.205 - 0.065]	3.108E-01	3.941E-01
Left isthmus cingulate cortex	-0.073	0.069	[-0.208 - 0.062]	2.913E-01	3.760E-01
Right isthmus cingulate cortex	0.027	0.073	[-0.117 - 0.170]	7.164E-01	7.267E-01

Supplementary Table S182. Cortical surface area differences between individuals with major depressive disorder taking second-generation antipsychotics and healthy comparison subjects controlling for age and sex

	d	Std. Err.	95% CI	*p*-value	FDR *q*-value
Total cortical surface area	0.060	0.207	[-0.345 - 0.465]	7.703E-01	9.802E-01
Left hemisphere	0.063	0.201	[-0.331 - 0.456]	7.552E-01	9.802E-01
Right hemisphere	0.058	0.212	[-0.359 - 0.474]	7.863E-01	9.802E-01
Left superior frontal gyrus	0.061	0.142	[-0.217 - 0.338]	6.678E-01	9.802E-01
Right superior frontal gyrus	0.029	0.192	[-0.348 - 0.406]	8.817E-01	9.802E-01
Left pars orbitalis of inferior frontal gyrus	0.041	0.280	[-0.508 - 0.590]	8.834E-01	9.802E-01
Right pars orbitalis of inferior frontal gyrus	0.010	0.238	[-0.456 - 0.476]	9.664E-01	9.802E-01
Left precentral gyrus	0.049	0.177	[-0.298 - 0.396]	7.832E-01	9.802E-01
Right precentral gyrus	0.041	0.198	[-0.347 - 0.430]	8.345E-01	9.802E-01
Left lingual gyrus	-0.223	0.143	[-0.503 - 0.058]	1.201E-01	9.802E-01
Right lingual gyrus	-0.113	0.142	[-0.391 - 0.164]	4.232E-01	9.802E-01
Left superior temporal gyrus	0.107	0.142	[-0.171 - 0.385]	4.498E-01	9.802E-01
Right superior temporal gyrus	0.080	0.183	[-0.279 - 0.439]	6.613E-01	9.802E-01
Left fusiform gyrus	0.036	0.238	[-0.430 - 0.503]	8.790E-01	9.802E-01
Right fusiform gyrus	-0.219	0.279	[-0.766 - 0.327]	4.315E-01	9.802E-01
Left inferior parietal cortex	-0.103	0.250	[-0.594 - 0.387]	6.793E-01	9.802E-01
Right inferior parietal cortex	-0.077	0.224	[-0.515 - 0.362]	7.320E-01	9.802E-01
Left lateral occipital cortex	-0.046	0.165	[-0.368 - 0.277]	7.812E-01	9.802E-01
Right lateral occipital cortex	0.137	0.244	[-0.340 - 0.615]	5.735E-01	9.802E-01
Left rostral middle frontal gyrus	0.016	0.184	[-0.344 - 0.376]	9.306E-01	9.802E-01
Right rostral middle frontal gyrus	0.103	0.199	[-0.288 - 0.494]	6.050E-01	9.802E-01
Left precuneus	0.156	0.191	[-0.218 - 0.531]	4.136E-01	9.802E-01
Right precuneus	0.190	0.178	[-0.158 - 0.539]	2.841E-01	9.802E-01
Left inferior temporal gyrus	-0.013	0.159	[-0.324 - 0.298]	9.338E-01	9.802E-01
Right inferior temporal gyrus	0.113	0.223	[-0.324 - 0.550]	6.129E-01	9.802E-01
Left lateral orbitofrontal cortex	0.121	0.142	[-0.156 - 0.399]	3.919E-01	9.802E-01
Right lateral orbitofrontal cortex	0.123	0.142	[-0.155 - 0.400]	3.855E-01	9.802E-01
Left middle temporal gyrus	0.045	0.249	[-0.443 - 0.533]	8.567E-01	9.802E-01
Right middle temporal gyrus	0.136	0.195	[-0.246 - 0.519]	4.848E-01	9.802E-01
Left postcentral gyrus	0.022	0.218	[-0.405 - 0.450]	9.189E-01	9.802E-01
Right postcentral gyrus	-0.201	0.171	[-0.537 - 0.134]	2.391E-01	9.802E-01
Left medial orbitofrontal cortex	0.026	0.182	[-0.332 - 0.383]	8.871E-01	9.802E-01
Right medial orbitofrontal cortex	-0.014	0.141	[-0.291 - 0.263]	9.214E-01	9.802E-01
Left cuneus	-0.289	0.142	[-0.568 - -0.011]	4.166E-02	7.797E-01
Right cuneus	-0.122	0.141	[-0.400 - 0.155]	3.871E-01	9.802E-01
Left pars triangularis of inferior frontal gyrus	0.041	0.233	[-0.416 - 0.498]	8.611E-01	9.802E-01
Right pars triangularis of inferior frontal gyrus	-0.039	0.141	[-0.316 - 0.238]	7.844E-01	9.802E-01
Left superior parietal cortex	0.140	0.179	[-0.211 - 0.492]	4.337E-01	9.802E-01
Right superior parietal cortex	-0.008	0.145	[-0.292 - 0.276]	9.564E-01	9.802E-01
Left pars opercularis of inferior frontal gyrus	0.295	0.166	[-0.029 - 0.620]	7.467E-02	8.835E-01
Right pars opercularis of inferior frontal gyrus	0.305	0.230	[-0.145 - 0.755]	1.837E-01	9.802E-01
Left supramarginal gyrus	0.106	0.142	[-0.171 - 0.384]	4.540E-01	9.802E-01
Right supramarginal gyrus	0.104	0.147	[-0.185 - 0.392]	4.812E-01	9.802E-01
Left pericalcarine cortex	-0.379	0.157	[-0.686 - -0.073]	1.534E-02	7.797E-01
Right pericalcarine cortex	-0.296	0.142	[-0.574 - -0.017]	3.777E-02	7.797E-01
Left parahippocampal gyrus	-0.074	0.168	[-0.403 - 0.255]	6.601E-01	9.802E-01
Right parahippocampal gyrus	-0.267	0.142	[-0.546 - 0.011]	5.981E-02	8.493E-01
Left caudal middle frontal gyrus	0.057	0.230	[-0.394 - 0.507]	8.056E-01	9.802E-01
Right caudal middle frontal gyrus	0.300	0.224	[-0.139 - 0.740]	1.805E-01	9.802E-01
Left transverse temporal gyrus	0.214	0.206	[-0.191 - 0.618]	3.010E-01	9.802E-01
Right transverse temporal gyrus	-0.039	0.142	[-0.317 - 0.239]	7.842E-01	9.802E-01
Left banks of superior temporal sulcus	0.283	0.224	[-0.157 - 0.723]	2.070E-01	9.802E-01
Right banks of superior temporal sulcus	0.028	0.206	[-0.375 - 0.431]	8.912E-01	9.802E-01
Left caudal anterior cingulate cortex	-0.058	0.195	[-0.440 - 0.325]	7.677E-01	9.802E-01
Right caudal anterior cingulate cortex	0.036	0.172	[-0.301 - 0.373]	8.351E-01	9.802E-01
Left rostral anterior cingulate cortex	0.143	0.142	[-0.135 - 0.421]	3.127E-01	9.802E-01
Right rostral anterior cingulate cortex	0.193	0.232	[-0.261 - 0.647]	4.053E-01	9.802E-01
Left posterior cingulate cortex	0.110	0.193	[-0.268 - 0.487]	5.688E-01	9.802E-01
Right posterior cingulate cortex	0.016	0.214	[-0.404 - 0.435]	9.422E-01	9.802E-01
Left frontal pole	-0.104	0.141	[-0.381 - 0.173]	4.611E-01	9.802E-01
Right frontal pole	-0.125	0.141	[-0.402 - 0.152]	3.764E-01	9.802E-01
Left paracentral lobule	-0.034	0.153	[-0.334 - 0.267]	8.263E-01	9.802E-01
Right paracentral lobule	-0.074	0.217	[-0.500 - 0.351]	7.315E-01	9.802E-01
Left insula	0.151	0.142	[-0.127 - 0.429]	2.867E-01	9.802E-01
Right insula	0.002	0.142	[-0.276 - 0.279]	9.889E-01	9.889E-01
Left entorhinal cortex	0.374	0.185	[0.010 - 0.737]	4.393E-02	7.797E-01
Right entorhinal cortex	0.188	0.142	[-0.090 - 0.465]	1.856E-01	9.802E-01
Left temporal pole	-0.019	0.141	[-0.297 - 0.258]	8.916E-01	9.802E-01
Right temporal pole	0.292	0.272	[-0.240 - 0.824]	2.827E-01	9.802E-01
Left isthmus cingulate cortex	0.185	0.142	[-0.093 - 0.463]	1.918E-01	9.802E-01
Right isthmus cingulate cortex	0.266	0.212	[-0.150 - 0.682]	2.102E-01	9.802E-01

Supplementary Table S183. Cortical surface area differences between individuals with major depressive disorder taking second-generation antipsychotics and healthy comparison subjects controlling for age and sex over 21 years of old

	d	Std. Err.	95% CI	*p*-value	FDR *q*-value
Total cortical surface area	0.088	0.224	[-0.351 - 0.526]	6.949E-01	9.551E-01
Left hemisphere	0.093	0.220	[-0.338 - 0.524]	6.724E-01	9.551E-01
Right hemisphere	0.082	0.227	[-0.363 - 0.527]	7.188E-01	9.551E-01
Left superior frontal gyrus	0.080	0.146	[-0.206 - 0.366]	5.845E-01	9.551E-01
Right superior frontal gyrus	0.088	0.231	[-0.365 - 0.541]	7.026E-01	9.551E-01
Left pars orbitalis of inferior frontal gyrus	0.076	0.331	[-0.573 - 0.726]	8.181E-01	9.551E-01
Right pars orbitalis of inferior frontal gyrus	-0.038	0.264	[-0.555 - 0.479]	8.855E-01	9.551E-01
Left precentral gyrus	0.069	0.188	[-0.300 - 0.438]	7.149E-01	9.551E-01
Right precentral gyrus	-0.052	0.145	[-0.336 - 0.231]	7.185E-01	9.551E-01
Left lingual gyrus	-0.264	0.145	[-0.548 - 0.020]	6.820E-02	9.340E-01
Right lingual gyrus	-0.145	0.145	[-0.429 - 0.138]	3.145E-01	9.517E-01
Left superior temporal gyrus	0.165	0.192	[-0.212 - 0.542]	3.913E-01	9.551E-01
Right superior temporal gyrus	0.119	0.207	[-0.286 - 0.524]	5.651E-01	9.551E-01
Left fusiform gyrus	0.058	0.252	[-0.437 - 0.552]	8.196E-01	9.551E-01
Right fusiform gyrus	-0.200	0.288	[-0.763 - 0.364]	4.869E-01	9.551E-01
Left inferior parietal cortex	-0.135	0.233	[-0.592 - 0.322]	5.616E-01	9.551E-01
Right inferior parietal cortex	-0.082	0.243	[-0.558 - 0.395]	7.377E-01	9.551E-01
Left lateral occipital cortex	-0.013	0.161	[-0.329 - 0.302]	9.348E-01	9.599E-01
Right lateral occipital cortex	0.155	0.233	[-0.302 - 0.612]	5.068E-01	9.551E-01
Left rostral middle frontal gyrus	0.044	0.196	[-0.340 - 0.428]	8.239E-01	9.551E-01
Right rostral middle frontal gyrus	0.141	0.209	[-0.267 - 0.550]	4.978E-01	9.551E-01
Left precuneus	0.218	0.204	[-0.182 - 0.618]	2.856E-01	9.517E-01
Right precuneus	0.227	0.178	[-0.122 - 0.577]	2.024E-01	9.340E-01
Left inferior temporal gyrus	-0.026	0.145	[-0.310 - 0.257]	8.567E-01	9.551E-01
Right inferior temporal gyrus	0.129	0.233	[-0.328 - 0.586]	5.799E-01	9.551E-01
Left lateral orbitofrontal cortex	0.216	0.182	[-0.141 - 0.573]	2.350E-01	9.340E-01
Right lateral orbitofrontal cortex	0.174	0.145	[-0.110 - 0.458]	2.294E-01	9.340E-01
Left middle temporal gyrus	0.050	0.263	[-0.466 - 0.566]	8.491E-01	9.551E-01
Right middle temporal gyrus	0.146	0.204	[-0.254 - 0.547]	4.741E-01	9.551E-01
Left postcentral gyrus	-0.039	0.186	[-0.403 - 0.325]	8.337E-01	9.551E-01
Right postcentral gyrus	-0.224	0.160	[-0.538 - 0.090]	1.616E-01	9.340E-01
Left medial orbitofrontal cortex	0.060	0.198	[-0.328 - 0.448]	7.621E-01	9.551E-01
Right medial orbitofrontal cortex	-0.023	0.144	[-0.306 - 0.260]	8.720E-01	9.551E-01
Left cuneus	-0.296	0.145	[-0.580 - -0.011]	4.156E-02	9.340E-01
Right cuneus	-0.109	0.144	[-0.392 - 0.174]	4.501E-01	9.551E-01
Left pars triangularis of inferior frontal gyrus	0.063	0.256	[-0.439 - 0.565]	8.057E-01	9.551E-01
Right pars triangularis of inferior frontal gyrus	-0.028	0.144	[-0.311 - 0.255]	8.449E-01	9.551E-01
Left superior parietal cortex	0.116	0.153	[-0.184 - 0.415]	4.493E-01	9.551E-01
Right superior parietal cortex	0.019	0.159	[-0.293 - 0.331]	9.060E-01	9.551E-01
Left pars opercularis of inferior frontal gyrus	0.355	0.209	[-0.054 - 0.764]	8.895E-02	9.340E-01
Right pars opercularis of inferior frontal gyrus	0.345	0.244	[-0.135 - 0.824]	1.587E-01	9.340E-01
Left supramarginal gyrus	0.143	0.145	[-0.140 - 0.427]	3.217E-01	9.517E-01
Right supramarginal gyrus	0.105	0.145	[-0.180 - 0.390]	4.708E-01	9.551E-01
Left pericalcarine cortex	-0.403	0.173	[-0.743 - -0.064]	1.978E-02	9.340E-01
Right pericalcarine cortex	-0.292	0.145	[-0.577 - -0.007]	4.452E-02	9.340E-01
Left parahippocampal gyrus	-0.078	0.196	[-0.463 - 0.306]	6.898E-01	9.551E-01
Right parahippocampal gyrus	-0.243	0.168	[-0.572 - 0.086]	1.485E-01	9.340E-01
Left caudal middle frontal gyrus	0.095	0.237	[-0.369 - 0.560]	6.873E-01	9.551E-01
Right caudal middle frontal gyrus	0.384	0.232	[-0.072 - 0.839]	9.900E-02	9.340E-01
Left transverse temporal gyrus	0.327	0.263	[-0.189 - 0.842]	2.147E-01	9.340E-01
Right transverse temporal gyrus	-0.015	0.145	[-0.299 - 0.268]	9.148E-01	9.551E-01
Left banks of superior temporal sulcus	0.251	0.242	[-0.223 - 0.726]	2.991E-01	9.517E-01
Right banks of superior temporal sulcus	0.014	0.206	[-0.389 - 0.417]	9.464E-01	9.599E-01
Left caudal anterior cingulate cortex	0.037	0.246	[-0.446 - 0.520]	8.795E-01	9.551E-01
Right caudal anterior cingulate cortex	0.038	0.187	[-0.328 - 0.404]	8.379E-01	9.551E-01
Left rostral anterior cingulate cortex	0.168	0.179	[-0.183 - 0.519]	3.487E-01	9.521E-01
Right rostral anterior cingulate cortex	0.283	0.294	[-0.294 - 0.860]	3.369E-01	9.521E-01
Left posterior cingulate cortex	0.144	0.237	[-0.320 - 0.608]	5.437E-01	9.551E-01
Right posterior cingulate cortex	0.024	0.217	[-0.402 - 0.450]	9.132E-01	9.551E-01
Left frontal pole	-0.127	0.144	[-0.410 - 0.156]	3.806E-01	9.551E-01
Right frontal pole	-0.146	0.144	[-0.430 - 0.137]	3.111E-01	9.517E-01
Left paracentral lobule	-0.022	0.183	[-0.380 - 0.336]	9.036E-01	9.551E-01
Right paracentral lobule	-0.105	0.204	[-0.504 - 0.294]	6.071E-01	9.551E-01
Left insula	0.159	0.145	[-0.125 - 0.443]	2.727E-01	9.517E-01
Right insula	0.032	0.168	[-0.297 - 0.360]	8.495E-01	9.551E-01
Left entorhinal cortex	0.271	0.147	[-0.018 - 0.560]	6.560E-02	9.340E-01
Right entorhinal cortex	0.262	0.178	[-0.088 - 0.611]	1.422E-01	9.340E-01
Left temporal pole	-0.003	0.145	[-0.286 - 0.281]	9.859E-01	9.859E-01
Right temporal pole	0.341	0.288	[-0.224 - 0.906]	2.368E-01	9.340E-01
Left isthmus cingulate cortex	0.205	0.145	[-0.079 - 0.489]	1.577E-01	9.340E-01
Right isthmus cingulate cortex	0.257	0.213	[-0.162 - 0.675]	2.295E-01	9.340E-01

Supplementary Table S184. Cortical surface area differences between individuals with major depressive disorder taking second-generation antipsychotics and individuals with major depressive disorder not taking antipsychotics controlling for age and sex

	d	Std. Err.	95% CI	*p*-value	FDR *q*-value
Total cortical surface area	0.065	0.139	[-0.207 - 0.337]	6.408E-01	9.391E-01
Left hemisphere	0.081	0.146	[-0.205 - 0.366]	5.802E-01	9.391E-01
Right hemisphere	0.048	0.133	[-0.213 - 0.309]	7.189E-01	9.391E-01
Left superior frontal gyrus	0.124	0.131	[-0.134 - 0.381]	3.457E-01	9.391E-01
Right superior frontal gyrus	-0.039	0.149	[-0.331 - 0.252]	7.909E-01	9.391E-01
Left pars orbitalis of inferior frontal gyrus	0.114	0.211	[-0.300 - 0.528]	5.902E-01	9.391E-01
Right pars orbitalis of inferior frontal gyrus	0.121	0.246	[-0.360 - 0.602]	6.215E-01	9.391E-01
Left precentral gyrus	0.069	0.177	[-0.277 - 0.415]	6.963E-01	9.391E-01
Right precentral gyrus	-0.033	0.207	[-0.439 - 0.374]	8.736E-01	9.453E-01
Left lingual gyrus	-0.078	0.173	[-0.416 - 0.260]	6.520E-01	9.391E-01
Right lingual gyrus	-0.051	0.132	[-0.308 - 0.207]	7.008E-01	9.391E-01
Left superior temporal gyrus	0.169	0.131	[-0.088 - 0.427]	1.976E-01	9.391E-01
Right superior temporal gyrus	0.067	0.140	[-0.207 - 0.341]	6.312E-01	9.391E-01
Left fusiform gyrus	0.147	0.225	[-0.293 - 0.587]	5.129E-01	9.391E-01
Right fusiform gyrus	-0.128	0.248	[-0.615 - 0.358]	6.053E-01	9.391E-01
Left inferior parietal cortex	-0.071	0.188	[-0.439 - 0.297]	7.057E-01	9.391E-01
Right inferior parietal cortex	-0.145	0.204	[-0.544 - 0.254]	4.759E-01	9.391E-01
Left lateral occipital cortex	-0.072	0.132	[-0.330 - 0.186]	5.866E-01	9.391E-01
Right lateral occipital cortex	0.193	0.169	[-0.138 - 0.524]	2.531E-01	9.391E-01
Left rostral middle frontal gyrus	-0.051	0.174	[-0.392 - 0.290]	7.691E-01	9.391E-01
Right rostral middle frontal gyrus	0.037	0.141	[-0.240 - 0.313]	7.944E-01	9.391E-01
Left precuneus	0.129	0.150	[-0.164 - 0.422]	3.885E-01	9.391E-01
Right precuneus	0.052	0.131	[-0.205 - 0.310]	6.907E-01	9.391E-01
Left inferior temporal gyrus	0.117	0.131	[-0.141 - 0.374]	3.752E-01	9.391E-01
Right inferior temporal gyrus	0.154	0.154	[-0.147 - 0.455]	3.170E-01	9.391E-01
Left lateral orbitofrontal cortex	0.159	0.131	[-0.099 - 0.417]	2.268E-01	9.391E-01
Right lateral orbitofrontal cortex	0.093	0.131	[-0.165 - 0.350]	4.803E-01	9.391E-01
Left middle temporal gyrus	0.004	0.207	[-0.401 - 0.410]	9.835E-01	9.974E-01
Right middle temporal gyrus	0.177	0.152	[-0.122 - 0.476]	2.456E-01	9.391E-01
Left postcentral gyrus	-0.042	0.203	[-0.439 - 0.355]	8.370E-01	9.453E-01
Right postcentral gyrus	-0.064	0.146	[-0.350 - 0.222]	6.624E-01	9.391E-01
Left medial orbitofrontal cortex	-0.011	0.210	[-0.423 - 0.401]	9.586E-01	9.974E-01
Right medial orbitofrontal cortex	0.092	0.131	[-0.166 - 0.349]	4.860E-01	9.391E-01
Left cuneus	-0.067	0.131	[-0.324 - 0.190]	6.094E-01	9.391E-01
Right cuneus	0.000	0.131	[-0.257 - 0.257]	9.974E-01	9.974E-01
Left pars triangularis of inferior frontal gyrus	0.179	0.182	[-0.178 - 0.536]	3.253E-01	9.391E-01
Right pars triangularis of inferior frontal gyrus	0.134	0.131	[-0.124 - 0.391]	3.083E-01	9.391E-01
Left superior parietal cortex	0.092	0.172	[-0.245 - 0.429]	5.918E-01	9.391E-01
Right superior parietal cortex	-0.022	0.131	[-0.279 - 0.235]	8.669E-01	9.453E-01
Left pars opercularis of inferior frontal gyrus	0.164	0.132	[-0.094 - 0.422]	2.125E-01	9.391E-01
Right pars opercularis of inferior frontal gyrus	0.108	0.172	[-0.229 - 0.445]	5.305E-01	9.391E-01
Left supramarginal gyrus	0.119	0.131	[-0.138 - 0.376]	3.650E-01	9.391E-01
Right supramarginal gyrus	0.134	0.131	[-0.124 - 0.392]	3.079E-01	9.391E-01
Left pericalcarine cortex	-0.211	0.141	[-0.487 - 0.066]	1.352E-01	9.391E-01
Right pericalcarine cortex	-0.047	0.131	[-0.304 - 0.210]	7.195E-01	9.391E-01
Left parahippocampal gyrus	-0.084	0.187	[-0.451 - 0.283]	6.535E-01	9.391E-01
Right parahippocampal gyrus	-0.122	0.172	[-0.459 - 0.215]	4.775E-01	9.391E-01
Left caudal middle frontal gyrus	-0.015	0.218	[-0.442 - 0.411]	9.441E-01	9.974E-01
Right caudal middle frontal gyrus	0.104	0.204	[-0.297 - 0.504]	6.121E-01	9.391E-01
Left transverse temporal gyrus	0.090	0.153	[-0.209 - 0.390]	5.545E-01	9.391E-01
Right transverse temporal gyrus	0.035	0.131	[-0.222 - 0.292]	7.888E-01	9.391E-01
Left banks of superior temporal sulcus	0.195	0.132	[-0.063 - 0.453]	1.382E-01	9.391E-01
Right banks of superior temporal sulcus	-0.038	0.153	[-0.338 - 0.263]	8.065E-01	9.391E-01
Left caudal anterior cingulate cortex	-0.046	0.142	[-0.324 - 0.232]	7.455E-01	9.391E-01
Right caudal anterior cingulate cortex	0.130	0.131	[-0.128 - 0.387]	3.233E-01	9.391E-01
Left rostral anterior cingulate cortex	0.095	0.131	[-0.163 - 0.352]	4.710E-01	9.391E-01
Right rostral anterior cingulate cortex	0.081	0.134	[-0.181 - 0.343]	5.440E-01	9.391E-01
Left posterior cingulate cortex	0.023	0.148	[-0.268 - 0.313]	8.788E-01	9.453E-01
Right posterior cingulate cortex	0.001	0.210	[-0.411 - 0.413]	9.970E-01	9.974E-01
Left frontal pole	-0.040	0.163	[-0.360 - 0.280]	8.069E-01	9.391E-01
Right frontal pole	-0.131	0.160	[-0.445 - 0.183]	4.138E-01	9.391E-01
Left paracentral lobule	-0.030	0.155	[-0.334 - 0.274]	8.481E-01	9.453E-01
Right paracentral lobule	-0.106	0.223	[-0.543 - 0.330]	6.324E-01	9.391E-01
Left insula	0.075	0.131	[-0.182 - 0.333]	5.657E-01	9.391E-01
Right insula	0.113	0.132	[-0.145 - 0.371]	3.911E-01	9.391E-01
Left entorhinal cortex	0.241	0.132	[-0.017 - 0.499]	6.686E-02	9.391E-01
Right entorhinal cortex	0.120	0.131	[-0.137 - 0.378]	3.600E-01	9.391E-01
Left temporal pole	0.188	0.132	[-0.070 - 0.446]	1.527E-01	9.391E-01
Right temporal pole	0.393	0.273	[-0.142 - 0.928]	1.498E-01	9.391E-01
Left isthmus cingulate cortex	0.096	0.131	[-0.162 - 0.354]	4.646E-01	9.391E-01
Right isthmus cingulate cortex	0.037	0.131	[-0.221 - 0.294]	7.797E-01	9.391E-01

Supplementary Table S185. Cortical surface area differences between individuals with major depressive disorder taking second-generation antipsychotics and individuals with major depressive disorder not taking antipsychotics controlling for age and sex over 21 years of old

	d	Std. Err.	95% CI	*p*-value	FDR *q*-value
Total cortical surface area	0.075	0.163	[-0.245 - 0.394]	6.465E-01	9.646E-01
Left hemisphere	0.094	0.170	[-0.239 - 0.428]	5.787E-01	9.646E-01
Right hemisphere	0.055	0.157	[-0.252 - 0.362]	7.264E-01	9.646E-01
Left superior frontal gyrus	0.124	0.135	[-0.140 - 0.388]	3.580E-01	9.646E-01
Right superior frontal gyrus	-0.005	0.178	[-0.353 - 0.344]	9.784E-01	9.923E-01
Left pars orbitalis of inferior frontal gyrus	0.138	0.257	[-0.364 - 0.641]	5.895E-01	9.646E-01
Right pars orbitalis of inferior frontal gyrus	0.054	0.260	[-0.457 - 0.564]	8.365E-01	9.732E-01
Left precentral gyrus	0.047	0.181	[-0.308 - 0.402]	7.954E-01	9.732E-01
Right precentral gyrus	-0.112	0.150	[-0.407 - 0.183]	4.572E-01	9.646E-01
Left lingual gyrus	-0.106	0.207	[-0.511 - 0.300]	6.094E-01	9.646E-01
Right lingual gyrus	-0.080	0.138	[-0.352 - 0.191]	5.611E-01	9.646E-01
Left superior temporal gyrus	0.167	0.135	[-0.098 - 0.431]	2.167E-01	9.646E-01
Right superior temporal gyrus	0.069	0.153	[-0.231 - 0.368]	6.530E-01	9.646E-01
Left fusiform gyrus	0.168	0.231	[-0.286 - 0.622]	4.680E-01	9.646E-01
Right fusiform gyrus	-0.103	0.246	[-0.585 - 0.380]	6.772E-01	9.646E-01
Left inferior parietal cortex	-0.104	0.183	[-0.462 - 0.254]	5.691E-01	9.646E-01
Right inferior parietal cortex	-0.154	0.214	[-0.573 - 0.264]	4.699E-01	9.646E-01
Left lateral occipital cortex	-0.054	0.135	[-0.319 - 0.210]	6.873E-01	9.646E-01
Right lateral occipital cortex	0.170	0.157	[-0.137 - 0.478]	2.769E-01	9.646E-01
Left rostral middle frontal gyrus	-0.028	0.182	[-0.384 - 0.328]	8.781E-01	9.732E-01
Right rostral middle frontal gyrus	0.053	0.147	[-0.236 - 0.342]	7.180E-01	9.646E-01
Left precuneus	0.196	0.173	[-0.143 - 0.535]	2.573E-01	9.646E-01
Right precuneus	0.099	0.135	[-0.165 - 0.363]	4.608E-01	9.646E-01
Left inferior temporal gyrus	0.123	0.135	[-0.142 - 0.387]	3.630E-01	9.646E-01
Right inferior temporal gyrus	0.207	0.178	[-0.141 - 0.556]	2.434E-01	9.646E-01
Left lateral orbitofrontal cortex	0.220	0.135	[-0.045 - 0.485]	1.030E-01	9.646E-01
Right lateral orbitofrontal cortex	0.125	0.135	[-0.139 - 0.390]	3.540E-01	9.646E-01
Left middle temporal gyrus	0.017	0.215	[-0.405 - 0.440]	9.361E-01	9.732E-01
Right middle temporal gyrus	0.191	0.158	[-0.118 - 0.501]	2.252E-01	9.646E-01
Left postcentral gyrus	-0.059	0.203	[-0.457 - 0.338]	7.700E-01	9.732E-01
Right postcentral gyrus	-0.068	0.135	[-0.333 - 0.196]	6.123E-01	9.646E-01
Left medial orbitofrontal cortex	0.014	0.213	[-0.403 - 0.432]	9.458E-01	9.732E-01
Right medial orbitofrontal cortex	0.071	0.135	[-0.193 - 0.335]	5.991E-01	9.646E-01
Left cuneus	-0.055	0.135	[-0.319 - 0.209]	6.810E-01	9.646E-01
Right cuneus	-0.012	0.135	[-0.275 - 0.252]	9.304E-01	9.732E-01
Left pars triangularis of inferior frontal gyrus	0.209	0.195	[-0.173 - 0.592]	2.825E-01	9.646E-01
Right pars triangularis of inferior frontal gyrus	0.124	0.135	[-0.140 - 0.388]	3.587E-01	9.646E-01
Left superior parietal cortex	0.043	0.135	[-0.221 - 0.308]	7.472E-01	9.646E-01
Right superior parietal cortex	0.012	0.135	[-0.252 - 0.276]	9.297E-01	9.732E-01
Left pars opercularis of inferior frontal gyrus	0.219	0.139	[-0.054 - 0.491]	1.153E-01	9.646E-01
Right pars opercularis of inferior frontal gyrus	0.123	0.193	[-0.255 - 0.501]	5.229E-01	9.646E-01
Left supramarginal gyrus	0.161	0.135	[-0.103 - 0.425]	2.315E-01	9.646E-01
Right supramarginal gyrus	0.152	0.135	[-0.113 - 0.416]	2.606E-01	9.646E-01
Left pericalcarine cortex	-0.251	0.176	[-0.596 - 0.095]	1.548E-01	9.646E-01
Right pericalcarine cortex	-0.079	0.135	[-0.343 - 0.185]	5.565E-01	9.646E-01
Left parahippocampal gyrus	-0.064	0.195	[-0.447 - 0.318]	7.413E-01	9.646E-01
Right parahippocampal gyrus	-0.106	0.185	[-0.468 - 0.257]	5.672E-01	9.646E-01
Left caudal middle frontal gyrus	0.000	0.215	[-0.422 - 0.422]	9.991E-01	9.991E-01
Right caudal middle frontal gyrus	0.171	0.206	[-0.233 - 0.574]	4.072E-01	9.646E-01
Left transverse temporal gyrus	0.177	0.208	[-0.231 - 0.586]	3.940E-01	9.646E-01
Right transverse temporal gyrus	0.052	0.135	[-0.212 - 0.316]	6.986E-01	9.646E-01
Left banks of superior temporal sulcus	0.169	0.140	[-0.105 - 0.443]	2.273E-01	9.646E-01
Right banks of superior temporal sulcus	-0.028	0.163	[-0.347 - 0.291]	8.621E-01	9.732E-01
Left caudal anterior cingulate cortex	-0.020	0.175	[-0.364 - 0.324]	9.086E-01	9.732E-01
Right caudal anterior cingulate cortex	0.113	0.135	[-0.152 - 0.377]	4.034E-01	9.646E-01
Left rostral anterior cingulate cortex	0.077	0.159	[-0.234 - 0.388]	6.264E-01	9.646E-01
Right rostral anterior cingulate cortex	0.171	0.193	[-0.207 - 0.550]	3.751E-01	9.646E-01
Left posterior cingulate cortex	0.032	0.172	[-0.305 - 0.368]	8.535E-01	9.732E-01
Right posterior cingulate cortex	0.020	0.219	[-0.409 - 0.449]	9.267E-01	9.732E-01
Left frontal pole	-0.069	0.173	[-0.409 - 0.271]	6.914E-01	9.646E-01
Right frontal pole	-0.150	0.166	[-0.476 - 0.175]	3.654E-01	9.646E-01
Left paracentral lobule	-0.044	0.169	[-0.375 - 0.286]	7.920E-01	9.732E-01
Right paracentral lobule	-0.126	0.208	[-0.533 - 0.282]	5.456E-01	9.646E-01
Left insula	0.091	0.135	[-0.173 - 0.355]	5.006E-01	9.646E-01
Right insula	0.148	0.135	[-0.117 - 0.412]	2.730E-01	9.646E-01
Left entorhinal cortex	0.203	0.135	[-0.062 - 0.467]	1.331E-01	9.646E-01
Right entorhinal cortex	0.141	0.135	[-0.123 - 0.405]	2.966E-01	9.646E-01
Left temporal pole	0.169	0.135	[-0.095 - 0.434]	2.094E-01	9.646E-01
Right temporal pole	0.460	0.302	[-0.133 - 1.052]	1.285E-01	9.646E-01
Left isthmus cingulate cortex	0.115	0.135	[-0.149 - 0.379]	3.946E-01	9.646E-01
Right isthmus cingulate cortex	0.013	0.135	[-0.251 - 0.277]	9.217E-01	9.732E-01

Supplementary Table S186. Cortical surface area differences between individuals with major depressive disorder taking first-generation antipsychotics and healthy comparison subjects controlling for age and sex

	d	Std. Err.	95% CI	*p*-value	FDR *q*-value
Total cortical surface area	-0.552	0.396	[-1.329 - 0.224]	1.635E-01	5.353E-01
Left hemisphere	-0.526	0.407	[-1.323 - 0.271]	1.960E-01	5.353E-01
Right hemisphere	-0.574	0.382	[-1.323 - 0.174]	1.327E-01	5.353E-01
Left superior frontal gyrus	-0.180	0.252	[-0.674 - 0.314]	4.754E-01	7.032E-01
Right superior frontal gyrus	-0.438	0.253	[-0.935 - 0.058]	8.341E-02	5.353E-01
Left pars orbitalis of inferior frontal gyrus	-0.362	0.352	[-1.052 - 0.328]	3.041E-01	5.907E-01
Right pars orbitalis of inferior frontal gyrus	-0.298	0.275	[-0.837 - 0.241]	2.789E-01	5.907E-01
Left precentral gyrus	-0.148	0.252	[-0.642 - 0.346]	5.566E-01	7.467E-01
Right precentral gyrus	-0.110	0.252	[-0.604 - 0.384]	6.637E-01	8.035E-01
Left lingual gyrus	-0.822	0.424	[-1.653 - 0.009]	5.246E-02	4.944E-01
Right lingual gyrus	-0.802	0.573	[-1.926 - 0.322]	1.618E-01	5.353E-01
Left superior temporal gyrus	-0.206	0.370	[-0.931 - 0.520]	5.786E-01	7.608E-01
Right superior temporal gyrus	-0.265	0.389	[-1.027 - 0.497]	4.962E-01	7.045E-01
Left fusiform gyrus	-0.201	0.253	[-0.696 - 0.293]	4.250E-01	6.821E-01
Right fusiform gyrus	-0.244	0.252	[-0.738 - 0.250]	3.335E-01	5.920E-01
Left inferior parietal cortex	-0.409	0.253	[-0.906 - 0.087]	1.063E-01	5.353E-01
Right inferior parietal cortex	-0.303	0.253	[-0.798 - 0.192]	2.305E-01	5.455E-01
Left lateral occipital cortex	-0.088	0.252	[-0.582 - 0.407]	7.284E-01	8.157E-01
Right lateral occipital cortex	-0.532	0.537	[-1.584 - 0.520]	3.217E-01	5.907E-01
Left rostral middle frontal gyrus	-0.495	0.360	[-1.201 - 0.211]	1.695E-01	5.353E-01
Right rostral middle frontal gyrus	-0.299	0.336	[-0.958 - 0.360]	3.739E-01	6.321E-01
Left precuneus	-0.652	0.255	[-1.152 - -0.152]	1.064E-02	2.518E-01
Right precuneus	-0.335	0.253	[-0.830 - 0.160]	1.851E-01	5.353E-01
Left inferior temporal gyrus	-0.289	0.252	[-0.784 - 0.205]	2.516E-01	5.763E-01
Right inferior temporal gyrus	-0.456	0.255	[-0.955 - 0.043]	7.336E-02	5.353E-01
Left lateral orbitofrontal cortex	-0.440	0.418	[-1.261 - 0.380]	2.926E-01	5.907E-01
Right lateral orbitofrontal cortex	-0.293	0.586	[-1.441 - 0.856]	6.175E-01	7.961E-01
Left middle temporal gyrus	-0.311	0.753	[-1.787 - 1.164]	6.790E-01	8.035E-01
Right middle temporal gyrus	-0.495	0.395	[-1.270 - 0.280]	2.105E-01	5.455E-01
Left postcentral gyrus	-0.396	0.297	[-0.978 - 0.186]	1.820E-01	5.353E-01
Right postcentral gyrus	-0.720	0.257	[-1.223 - -0.217]	5.004E-03	2.518E-01
Left medial orbitofrontal cortex	-0.356	0.254	[-0.853 - 0.142]	1.610E-01	5.353E-01
Right medial orbitofrontal cortex	-0.319	0.302	[-0.910 - 0.272]	2.901E-01	5.907E-01
Left cuneus	-0.249	0.252	[-0.743 - 0.246]	3.245E-01	5.907E-01
Right cuneus	-0.306	0.253	[-0.801 - 0.189]	2.253E-01	5.455E-01
Left pars triangularis of inferior frontal gyrus	0.004	0.316	[-0.616 - 0.623]	9.906E-01	9.916E-01
Right pars triangularis of inferior frontal gyrus	-0.488	0.254	[-0.986 - 0.011]	5.505E-02	4.944E-01
Left superior parietal cortex	-0.513	0.268	[-1.038 - 0.013]	5.571E-02	4.944E-01
Right superior parietal cortex	-0.496	0.254	[-0.993 - 0.001]	5.048E-02	4.944E-01
Left pars opercularis of inferior frontal gyrus	-0.377	0.490	[-1.338 - 0.584]	4.419E-01	6.821E-01
Right pars opercularis of inferior frontal gyrus	0.229	0.253	[-0.266 - 0.724]	3.649E-01	6.319E-01
Left supramarginal gyrus	-0.377	0.253	[-0.873 - 0.120]	1.371E-01	5.353E-01
Right supramarginal gyrus	-0.662	0.257	[-1.165 - -0.158]	1.000E-02	2.518E-01
Left pericalcarine cortex	-0.304	0.253	[-0.799 - 0.191]	2.284E-01	5.455E-01
Right pericalcarine cortex	-0.498	0.254	[-0.996 - 0.000]	5.012E-02	4.944E-01
Left parahippocampal gyrus	-0.117	0.252	[-0.611 - 0.377]	6.430E-01	8.010E-01
Right parahippocampal gyrus	-0.419	0.253	[-0.915 - 0.077]	9.792E-02	5.353E-01
Left caudal middle frontal gyrus	-0.057	0.293	[-0.633 - 0.518]	8.447E-01	9.087E-01
Right caudal middle frontal gyrus	0.175	0.253	[-0.321 - 0.670]	4.896E-01	7.045E-01
Left transverse temporal gyrus	-0.395	0.284	[-0.952 - 0.162]	1.644E-01	5.353E-01
Right transverse temporal gyrus	-0.384	0.253	[-0.880 - 0.112]	1.293E-01	5.353E-01
Left banks of superior temporal sulcus	-0.290	0.376	[-1.028 - 0.447]	4.406E-01	6.821E-01
Right banks of superior temporal sulcus	-0.232	0.591	[-1.391 - 0.927]	6.949E-01	8.088E-01
Left caudal anterior cingulate cortex	-0.325	0.519	[-1.341 - 0.692]	5.313E-01	7.397E-01
Right caudal anterior cingulate cortex	-0.015	0.252	[-0.509 - 0.480]	9.541E-01	9.887E-01
Left rostral anterior cingulate cortex	-0.217	0.278	[-0.762 - 0.328]	4.356E-01	6.821E-01
Right rostral anterior cingulate cortex	-0.044	0.252	[-0.538 - 0.449]	8.603E-01	9.117E-01
Left posterior cingulate cortex	-0.101	0.272	[-0.634 - 0.432]	7.109E-01	8.141E-01
Right posterior cingulate cortex	-0.183	0.253	[-0.678 - 0.312]	4.690E-01	7.032E-01
Left frontal pole	-0.418	0.257	[-0.923 - 0.086]	1.042E-01	5.353E-01
Right frontal pole	-0.122	0.252	[-0.616 - 0.372]	6.279E-01	7.961E-01
Left paracentral lobule	-0.003	0.252	[-0.497 - 0.491]	9.916E-01	9.916E-01
Right paracentral lobule	-0.012	0.252	[-0.506 - 0.481]	9.609E-01	9.887E-01
Left insula	-0.148	0.252	[-0.642 - 0.346]	5.574E-01	7.467E-01
Right insula	-0.274	0.253	[-0.770 - 0.222]	2.794E-01	5.907E-01
Left entorhinal cortex	-0.130	0.383	[-0.880 - 0.621]	7.353E-01	8.157E-01
Right entorhinal cortex	-0.107	0.252	[-0.601 - 0.387]	6.715E-01	8.035E-01
Left temporal pole	-0.249	0.253	[-0.745 - 0.247]	3.244E-01	5.907E-01
Right temporal pole	-0.329	0.253	[-0.825 - 0.166]	1.929E-01	5.353E-01
Left isthmus cingulate cortex	0.051	0.252	[-0.444 - 0.545]	8.411E-01	9.087E-01
Right isthmus cingulate cortex	-0.376	0.253	[-0.871 - 0.120]	1.370E-01	5.353E-01

Supplementary Table S187. Cortical surface area differences between individuals with major depressive disorder taking first-generation antipsychotics and healthy comparison subjects controlling for age and sex over 21 years of old

	d	Std. Err.	95% CI	*p*-value	FDR *q*-value
Total cortical surface area	-0.564	0.408	[-1.364 - 0.235]	1.667E-01	5.548E-01
Left hemisphere	-0.541	0.421	[-1.366 - 0.284]	1.990E-01	5.599E-01
Right hemisphere	-0.583	0.391	[-1.349 - 0.183]	1.357E-01	5.548E-01
Left superior frontal gyrus	-0.189	0.252	[-0.684 - 0.306]	4.544E-01	6.984E-01
Right superior frontal gyrus	-0.451	0.254	[-0.948 - 0.046]	7.547E-02	5.381E-01
Left pars orbitalis of inferior frontal gyrus	-0.396	0.383	[-1.147 - 0.355]	3.015E-01	5.693E-01
Right pars orbitalis of inferior frontal gyrus	-0.305	0.283	[-0.859 - 0.248]	2.799E-01	5.693E-01
Left precentral gyrus	-0.143	0.252	[-0.638 - 0.351]	5.699E-01	7.493E-01
Right precentral gyrus	-0.126	0.255	[-0.626 - 0.373]	6.205E-01	7.736E-01
Left lingual gyrus	-0.830	0.432	[-1.677 - 0.017]	5.477E-02	5.381E-01
Right lingual gyrus	-0.807	0.579	[-1.942 - 0.328]	1.634E-01	5.548E-01
Left superior temporal gyrus	-0.216	0.381	[-0.962 - 0.530]	5.698E-01	7.493E-01
Right superior temporal gyrus	-0.256	0.382	[-1.004 - 0.492]	5.029E-01	7.021E-01
Left fusiform gyrus	-0.213	0.253	[-0.708 - 0.283]	3.998E-01	6.601E-01
Right fusiform gyrus	-0.259	0.253	[-0.754 - 0.236]	3.047E-01	5.693E-01
Left inferior parietal cortex	-0.397	0.254	[-0.894 - 0.100]	1.179E-01	5.548E-01
Right inferior parietal cortex	-0.284	0.253	[-0.779 - 0.212]	2.618E-01	5.693E-01
Left lateral occipital cortex	-0.086	0.252	[-0.581 - 0.409]	7.335E-01	8.137E-01
Right lateral occipital cortex	-0.549	0.553	[-1.633 - 0.536]	3.214E-01	5.850E-01
Left rostral middle frontal gyrus	-0.493	0.359	[-1.197 - 0.211]	1.702E-01	5.548E-01
Right rostral middle frontal gyrus	-0.284	0.324	[-0.919 - 0.351]	3.805E-01	6.433E-01
Left precuneus	-0.668	0.270	[-1.198 - -0.139]	1.333E-02	3.155E-01
Right precuneus	-0.321	0.253	[-0.817 - 0.175]	2.050E-01	5.599E-01
Left inferior temporal gyrus	-0.274	0.253	[-0.770 - 0.221]	2.775E-01	5.693E-01
Right inferior temporal gyrus	-0.436	0.255	[-0.936 - 0.063]	8.701E-02	5.548E-01
Left lateral orbitofrontal cortex	-0.446	0.425	[-1.279 - 0.386]	2.932E-01	5.693E-01
Right lateral orbitofrontal cortex	-0.288	0.582	[-1.429 - 0.853]	6.210E-01	7.736E-01
Left middle temporal gyrus	-0.324	0.766	[-1.825 - 1.177]	6.718E-01	8.035E-01
Right middle temporal gyrus	-0.528	0.426	[-1.362 - 0.306]	2.150E-01	5.654E-01
Left postcentral gyrus	-0.380	0.283	[-0.934 - 0.175]	1.798E-01	5.550E-01
Right postcentral gyrus	-0.739	0.257	[-1.243 - -0.235]	4.044E-03	2.871E-01
Left medial orbitofrontal cortex	-0.347	0.254	[-0.845 - 0.151]	1.719E-01	5.548E-01
Right medial orbitofrontal cortex	-0.335	0.317	[-0.956 - 0.285]	2.899E-01	5.693E-01
Left cuneus	-0.236	0.253	[-0.731 - 0.259]	3.504E-01	6.069E-01
Right cuneus	-0.295	0.253	[-0.790 - 0.201]	2.438E-01	5.693E-01
Left pars triangularis of inferior frontal gyrus	-0.041	0.277	[-0.584 - 0.502]	8.823E-01	9.372E-01
Right pars triangularis of inferior frontal gyrus	-0.487	0.255	[-0.987 - 0.012]	5.561E-02	5.381E-01
Left superior parietal cortex	-0.499	0.256	[-1.001 - 0.003]	5.152E-02	5.381E-01
Right superior parietal cortex	-0.472	0.254	[-0.969 - 0.026]	6.320E-02	5.381E-01
Left pars opercularis of inferior frontal gyrus	-0.399	0.512	[-1.402 - 0.604]	4.351E-01	6.984E-01
Right pars opercularis of inferior frontal gyrus	0.260	0.253	[-0.236 - 0.756]	3.047E-01	5.693E-01
Left supramarginal gyrus	-0.393	0.254	[-0.891 - 0.104]	1.211E-01	5.548E-01
Right supramarginal gyrus	-0.666	0.257	[-1.171 - -0.162]	9.593E-03	3.155E-01
Left pericalcarine cortex	-0.301	0.253	[-0.796 - 0.195]	2.343E-01	5.693E-01
Right pericalcarine cortex	-0.509	0.255	[-1.008 - -0.011]	4.537E-02	5.381E-01
Left parahippocampal gyrus	-0.134	0.252	[-0.629 - 0.360]	5.950E-01	7.681E-01
Right parahippocampal gyrus	-0.450	0.254	[-0.948 - 0.047]	7.579E-02	5.381E-01
Left caudal middle frontal gyrus	-0.063	0.299	[-0.649 - 0.523]	8.335E-01	9.105E-01
Right caudal middle frontal gyrus	0.176	0.253	[-0.320 - 0.672]	4.875E-01	7.021E-01
Left transverse temporal gyrus	-0.392	0.283	[-0.946 - 0.162]	1.650E-01	5.548E-01
Right transverse temporal gyrus	-0.406	0.254	[-0.903 - 0.091]	1.095E-01	5.548E-01
Left banks of superior temporal sulcus	-0.288	0.375	[-1.023 - 0.447]	4.429E-01	6.984E-01
Right banks of superior temporal sulcus	-0.224	0.585	[-1.370 - 0.922]	7.016E-01	8.035E-01
Left caudal anterior cingulate cortex	-0.307	0.504	[-1.294 - 0.680]	5.416E-01	7.395E-01
Right caudal anterior cingulate cortex	0.001	0.252	[-0.493 - 0.496]	9.954E-01	9.954E-01
Left rostral anterior cingulate cortex	-0.186	0.252	[-0.680 - 0.309]	4.623E-01	6.984E-01
Right rostral anterior cingulate cortex	-0.020	0.252	[-0.514 - 0.474]	9.371E-01	9.784E-01
Left posterior cingulate cortex	-0.111	0.282	[-0.663 - 0.441]	6.941E-01	8.035E-01
Right posterior cingulate cortex	-0.169	0.253	[-0.664 - 0.327]	5.043E-01	7.021E-01
Left frontal pole	-0.449	0.285	[-1.008 - 0.111]	1.160E-01	5.548E-01
Right frontal pole	-0.101	0.252	[-0.596 - 0.393]	6.878E-01	8.035E-01
Left paracentral lobule	0.013	0.252	[-0.482 - 0.507]	9.598E-01	9.860E-01
Right paracentral lobule	-0.009	0.252	[-0.503 - 0.485]	9.721E-01	9.860E-01
Left insula	-0.179	0.252	[-0.673 - 0.316]	4.784E-01	7.021E-01
Right insula	-0.295	0.254	[-0.792 - 0.202]	2.445E-01	5.693E-01
Left entorhinal cortex	-0.165	0.416	[-0.980 - 0.650]	6.911E-01	8.035E-01
Right entorhinal cortex	-0.089	0.252	[-0.583 - 0.406]	7.248E-01	8.137E-01
Left temporal pole	-0.245	0.253	[-0.741 - 0.251]	3.336E-01	5.921E-01
Right temporal pole	-0.334	0.253	[-0.830 - 0.163]	1.878E-01	5.555E-01
Left isthmus cingulate cortex	0.037	0.253	[-0.458 - 0.532]	8.844E-01	9.372E-01
Right isthmus cingulate cortex	-0.346	0.253	[-0.842 - 0.150]	1.714E-01	5.548E-01

Supplementary Table S188. Cortical surface area differences between individuals with major depressive disorder taking first-generation antipsychotics and individuals with major depressive disorder not taking antipsychotics controlling for age and sex

	d	Std. Err.	95% CI	*p*-value	FDR *q*-value
Total cortical surface area	-0.390	0.356	[-1.088 - 0.308]	2.737E-01	8.842E-01
Left hemisphere	-0.378	0.365	[-1.093 - 0.337]	2.998E-01	8.842E-01
Right hemisphere	-0.399	0.346	[-1.077 - 0.280]	2.498E-01	8.842E-01
Left superior frontal gyrus	-0.121	0.296	[-0.702 - 0.459]	6.824E-01	9.140E-01
Right superior frontal gyrus	-0.248	0.208	[-0.657 - 0.161]	2.343E-01	8.842E-01
Left pars orbitalis of inferior frontal gyrus	-0.062	0.313	[-0.677 - 0.552]	8.419E-01	9.252E-01
Right pars orbitalis of inferior frontal gyrus	-0.147	0.251	[-0.638 - 0.344]	5.580E-01	9.055E-01
Left precentral gyrus	-0.137	0.278	[-0.683 - 0.409]	6.230E-01	9.076E-01
Right precentral gyrus	-0.285	0.302	[-0.877 - 0.307]	3.455E-01	8.842E-01
Left lingual gyrus	-0.330	0.256	[-0.831 - 0.171]	1.970E-01	8.842E-01
Right lingual gyrus	-0.437	0.424	[-1.269 - 0.394]	3.026E-01	8.842E-01
Left superior temporal gyrus	-0.091	0.320	[-0.719 - 0.536]	7.751E-01	9.252E-01
Right superior temporal gyrus	-0.470	0.485	[-1.420 - 0.480]	3.321E-01	8.842E-01
Left fusiform gyrus	-0.207	0.208	[-0.614 - 0.201]	3.203E-01	8.842E-01
Right fusiform gyrus	-0.043	0.208	[-0.450 - 0.365]	8.376E-01	9.252E-01
Left inferior parietal cortex	-0.206	0.248	[-0.693 - 0.281]	4.070E-01	8.842E-01
Right inferior parietal cortex	-0.201	0.208	[-0.609 - 0.207]	3.350E-01	8.842E-01
Left lateral occipital cortex	-0.031	0.208	[-0.438 - 0.377]	8.830E-01	9.252E-01
Right lateral occipital cortex	-0.153	0.363	[-0.863 - 0.558]	6.733E-01	9.140E-01
Left rostral middle frontal gyrus	-0.497	0.258	[-1.003 - 0.008]	5.374E-02	8.842E-01
Right rostral middle frontal gyrus	-0.344	0.209	[-0.752 - 0.065]	9.922E-02	8.842E-01
Left precuneus	-0.440	0.253	[-0.935 - 0.055]	8.152E-02	8.842E-01
Right precuneus	-0.286	0.208	[-0.694 - 0.123]	1.706E-01	8.842E-01
Left inferior temporal gyrus	0.091	0.208	[-0.317 - 0.498]	6.630E-01	9.140E-01
Right inferior temporal gyrus	-0.118	0.208	[-0.525 - 0.289]	5.692E-01	9.055E-01
Left lateral orbitofrontal cortex	-0.501	0.400	[-1.284 - 0.282]	2.102E-01	8.842E-01
Right lateral orbitofrontal cortex	-0.468	0.570	[-1.585 - 0.650]	4.123E-01	8.842E-01
Left middle temporal gyrus	-0.207	0.398	[-0.987 - 0.574]	6.039E-01	9.076E-01
Right middle temporal gyrus	-0.217	0.378	[-0.958 - 0.524]	5.661E-01	9.055E-01
Left postcentral gyrus	-0.170	0.349	[-0.854 - 0.514]	6.264E-01	9.076E-01
Right postcentral gyrus	-0.300	0.310	[-0.908 - 0.308]	3.341E-01	8.842E-01
Left medial orbitofrontal cortex	-0.195	0.208	[-0.603 - 0.213]	3.490E-01	8.842E-01
Right medial orbitofrontal cortex	-0.396	0.382	[-1.145 - 0.354]	3.005E-01	8.842E-01
Left cuneus	0.060	0.329	[-0.585 - 0.705]	8.552E-01	9.252E-01
Right cuneus	-0.036	0.208	[-0.444 - 0.372]	8.632E-01	9.252E-01
Left pars triangularis of inferior frontal gyrus	0.032	0.208	[-0.376 - 0.439]	8.782E-01	9.252E-01
Right pars triangularis of inferior frontal gyrus	-0.156	0.208	[-0.563 - 0.251]	4.527E-01	8.842E-01
Left superior parietal cortex	-0.548	0.269	[-1.074 - -0.021]	4.154E-02	8.842E-01
Right superior parietal cortex	-0.323	0.302	[-0.916 - 0.269]	2.847E-01	8.842E-01
Left pars opercularis of inferior frontal gyrus	-0.312	0.332	[-0.963 - 0.338]	3.467E-01	8.842E-01
Right pars opercularis of inferior frontal gyrus	0.241	0.208	[-0.167 - 0.649]	2.478E-01	8.842E-01
Left supramarginal gyrus	-0.157	0.208	[-0.564 - 0.251]	4.508E-01	8.842E-01
Right supramarginal gyrus	-0.324	0.208	[-0.732 - 0.084]	1.200E-01	8.842E-01
Left pericalcarine cortex	-0.148	0.208	[-0.556 - 0.260]	4.762E-01	8.842E-01
Right pericalcarine cortex	-0.100	0.336	[-0.757 - 0.558]	7.669E-01	9.252E-01
Left parahippocampal gyrus	-0.166	0.208	[-0.575 - 0.242]	4.239E-01	8.842E-01
Right parahippocampal gyrus	-0.444	0.393	[-1.213 - 0.326]	2.584E-01	8.842E-01
Left caudal middle frontal gyrus	-0.091	0.339	[-0.756 - 0.573]	7.874E-01	9.252E-01
Right caudal middle frontal gyrus	-0.064	0.267	[-0.588 - 0.460]	8.108E-01	9.252E-01
Left transverse temporal gyrus	-0.255	0.328	[-0.897 - 0.387]	4.358E-01	8.842E-01
Right transverse temporal gyrus	-0.194	0.208	[-0.601 - 0.214]	3.508E-01	8.842E-01
Left banks of superior temporal sulcus	-0.183	0.208	[-0.591 - 0.225]	3.789E-01	8.842E-01
Right banks of superior temporal sulcus	-0.339	0.473	[-1.265 - 0.588]	4.735E-01	8.842E-01
Left caudal anterior cingulate cortex	0.045	0.353	[-0.646 - 0.736]	8.991E-01	9.252E-01
Right caudal anterior cingulate cortex	0.125	0.305	[-0.472 - 0.723]	6.805E-01	9.140E-01
Left rostral anterior cingulate cortex	-0.225	0.288	[-0.789 - 0.339]	4.346E-01	8.842E-01
Right rostral anterior cingulate cortex	0.053	0.231	[-0.401 - 0.507]	8.186E-01	9.252E-01
Left posterior cingulate cortex	0.003	0.208	[-0.405 - 0.411]	9.886E-01	9.886E-01
Right posterior cingulate cortex	-0.081	0.208	[-0.489 - 0.326]	6.952E-01	9.140E-01
Left frontal pole	-0.231	0.209	[-0.640 - 0.177]	2.673E-01	8.842E-01
Right frontal pole	-0.028	0.208	[-0.436 - 0.380]	8.929E-01	9.252E-01
Left paracentral lobule	-0.277	0.398	[-1.057 - 0.502]	4.857E-01	8.842E-01
Right paracentral lobule	-0.174	0.208	[-0.582 - 0.235]	4.047E-01	8.842E-01
Left insula	-0.130	0.208	[-0.537 - 0.278]	5.331E-01	9.055E-01
Right insula	-0.114	0.208	[-0.522 - 0.293]	5.823E-01	9.055E-01
Left entorhinal cortex	-0.003	0.208	[-0.410 - 0.404]	9.885E-01	9.886E-01
Right entorhinal cortex	-0.117	0.208	[-0.524 - 0.290]	5.739E-01	9.055E-01
Left temporal pole	-0.044	0.208	[-0.451 - 0.364]	8.332E-01	9.252E-01
Right temporal pole	-0.040	0.208	[-0.447 - 0.368]	8.476E-01	9.252E-01
Left isthmus cingulate cortex	-0.133	0.245	[-0.613 - 0.347]	5.867E-01	9.055E-01
Right isthmus cingulate cortex	-0.356	0.261	[-0.866 - 0.155]	1.723E-01	8.842E-01

Supplementary Table S189. Cortical surface differences between individuals with major depressive disorder taking first-generation antipsychotics and individuals with major depressive disorder not taking antipsychotics controlling for age and sex over 21 years of old

	d	Std. Err.	95% CI	*p*-value	FDR *q*-value
Total cortical surface area	-0.391	0.355	[-1.087 - 0.306]	2.718E-01	8.634E-01
Left hemisphere	-0.379	0.364	[-1.092 - 0.333]	2.969E-01	8.634E-01
Right hemisphere	-0.399	0.346	[-1.078 - 0.279]	2.490E-01	8.634E-01
Left superior frontal gyrus	-0.123	0.291	[-0.693 - 0.447]	6.728E-01	9.124E-01
Right superior frontal gyrus	-0.228	0.209	[-0.638 - 0.182]	2.755E-01	8.634E-01
Left pars orbitalis of inferior frontal gyrus	-0.065	0.307	[-0.666 - 0.536]	8.320E-01	9.130E-01
Right pars orbitalis of inferior frontal gyrus	-0.147	0.245	[-0.628 - 0.333]	5.478E-01	9.124E-01
Left precentral gyrus	-0.140	0.270	[-0.669 - 0.389]	6.045E-01	9.124E-01
Right precentral gyrus	-0.287	0.296	[-0.867 - 0.294]	3.329E-01	8.634E-01
Left lingual gyrus	-0.329	0.259	[-0.837 - 0.180]	2.051E-01	8.634E-01
Right lingual gyrus	-0.443	0.421	[-1.269 - 0.382]	2.925E-01	8.634E-01
Left superior temporal gyrus	-0.096	0.315	[-0.713 - 0.521]	7.608E-01	9.130E-01
Right superior temporal gyrus	-0.474	0.480	[-1.414 - 0.467]	3.236E-01	8.634E-01
Left fusiform gyrus	-0.200	0.209	[-0.608 - 0.209]	3.385E-01	8.634E-01
Right fusiform gyrus	-0.056	0.208	[-0.465 - 0.352]	7.873E-01	9.130E-01
Left inferior parietal cortex	-0.212	0.243	[-0.687 - 0.264]	3.831E-01	8.634E-01
Right inferior parietal cortex	-0.188	0.209	[-0.597 - 0.221]	3.681E-01	8.634E-01
Left lateral occipital cortex	-0.028	0.209	[-0.437 - 0.381]	8.948E-01	9.343E-01
Right lateral occipital cortex	-0.168	0.356	[-0.865 - 0.529]	6.369E-01	9.124E-01
Left rostral middle frontal gyrus	-0.501	0.254	[-0.999 - -0.004]	4.823E-02	8.634E-01
Right rostral middle frontal gyrus	-0.348	0.209	[-0.758 - 0.062]	9.592E-02	8.634E-01
Left precuneus	-0.441	0.253	[-0.936 - 0.055]	8.117E-02	8.634E-01
Right precuneus	-0.269	0.209	[-0.678 - 0.141]	1.986E-01	8.634E-01
Left inferior temporal gyrus	0.085	0.208	[-0.323 - 0.494]	6.824E-01	9.124E-01
Right inferior temporal gyrus	-0.115	0.208	[-0.523 - 0.294]	5.817E-01	9.124E-01
Left lateral orbitofrontal cortex	-0.500	0.401	[-1.286 - 0.287]	2.131E-01	8.634E-01
Right lateral orbitofrontal cortex	-0.468	0.570	[-1.585 - 0.648]	4.112E-01	8.634E-01
Left middle temporal gyrus	-0.208	0.396	[-0.985 - 0.569]	5.993E-01	9.124E-01
Right middle temporal gyrus	-0.213	0.382	[-0.962 - 0.536]	5.772E-01	9.124E-01
Left postcentral gyrus	-0.158	0.355	[-0.853 - 0.538]	6.564E-01	9.124E-01
Right postcentral gyrus	-0.316	0.299	[-0.902 - 0.270]	2.908E-01	8.634E-01
Left medial orbitofrontal cortex	-0.197	0.209	[-0.607 - 0.212]	3.453E-01	8.634E-01
Right medial orbitofrontal cortex	-0.401	0.374	[-1.133 - 0.332]	2.840E-01	8.634E-01
Left cuneus	0.067	0.334	[-0.588 - 0.723]	8.405E-01	9.130E-01
Right cuneus	-0.055	0.209	[-0.464 - 0.354]	7.911E-01	9.130E-01
Left pars triangularis of inferior frontal gyrus	0.036	0.208	[-0.372 - 0.445]	8.616E-01	9.130E-01
Right pars triangularis of inferior frontal gyrus	-0.158	0.208	[-0.566 - 0.250]	4.483E-01	8.841E-01
Left superior parietal cortex	-0.546	0.279	[-1.094 - 0.002]	5.070E-02	8.634E-01
Right superior parietal cortex	-0.310	0.314	[-0.925 - 0.305]	3.228E-01	8.634E-01
Left pars opercularis of inferior frontal gyrus	-0.313	0.331	[-0.963 - 0.336]	3.441E-01	8.634E-01
Right pars opercularis of inferior frontal gyrus	0.223	0.209	[-0.186 - 0.632]	2.858E-01	8.634E-01
Left supramarginal gyrus	-0.167	0.208	[-0.576 - 0.242]	4.233E-01	8.634E-01
Right supramarginal gyrus	-0.320	0.209	[-0.730 - 0.089]	1.253E-01	8.634E-01
Left pericalcarine cortex	-0.139	0.209	[-0.549 - 0.270]	5.042E-01	9.124E-01
Right pericalcarine cortex	-0.124	0.339	[-0.788 - 0.540]	7.143E-01	9.124E-01
Left parahippocampal gyrus	-0.180	0.209	[-0.589 - 0.229]	3.892E-01	8.634E-01
Right parahippocampal gyrus	-0.449	0.385	[-1.205 - 0.306]	2.434E-01	8.634E-01
Left caudal middle frontal gyrus	-0.096	0.330	[-0.742 - 0.551]	7.715E-01	9.130E-01
Right caudal middle frontal gyrus	-0.061	0.278	[-0.606 - 0.485]	8.275E-01	9.130E-01
Left transverse temporal gyrus	-0.260	0.326	[-0.900 - 0.380]	4.256E-01	8.634E-01
Right transverse temporal gyrus	-0.203	0.209	[-0.611 - 0.206]	3.310E-01	8.634E-01
Left banks of superior temporal sulcus	-0.192	0.209	[-0.601 - 0.217]	3.583E-01	8.634E-01
Right banks of superior temporal sulcus	-0.336	0.476	[-1.269 - 0.597]	4.804E-01	8.976E-01
Left caudal anterior cingulate cortex	0.040	0.351	[-0.647 - 0.728]	9.082E-01	9.345E-01
Right caudal anterior cingulate cortex	0.114	0.308	[-0.489 - 0.716]	7.117E-01	9.124E-01
Left rostral anterior cingulate cortex	-0.232	0.282	[-0.785 - 0.320]	4.101E-01	8.634E-01
Right rostral anterior cingulate cortex	0.084	0.233	[-0.373 - 0.540]	7.196E-01	9.124E-01
Left posterior cingulate cortex	0.003	0.209	[-0.406 - 0.412]	9.881E-01	9.881E-01
Right posterior cingulate cortex	-0.078	0.208	[-0.487 - 0.330]	7.073E-01	9.124E-01
Left frontal pole	-0.217	0.215	[-0.639 - 0.204]	3.122E-01	8.634E-01
Right frontal pole	-0.038	0.209	[-0.447 - 0.371]	8.554E-01	9.130E-01
Left paracentral lobule	-0.286	0.394	[-1.058 - 0.487]	4.684E-01	8.976E-01
Right paracentral lobule	-0.176	0.209	[-0.585 - 0.234]	4.000E-01	8.634E-01
Left insula	-0.124	0.208	[-0.533 - 0.285]	5.519E-01	9.124E-01
Right insula	-0.095	0.209	[-0.504 - 0.314]	6.490E-01	9.124E-01
Left entorhinal cortex	-0.008	0.208	[-0.417 - 0.401]	9.695E-01	9.833E-01
Right entorhinal cortex	-0.128	0.208	[-0.536 - 0.281]	5.404E-01	9.124E-01
Left temporal pole	-0.068	0.208	[-0.477 - 0.340]	7.430E-01	9.130E-01
Right temporal pole	-0.040	0.209	[-0.449 - 0.368]	8.465E-01	9.130E-01
Left isthmus cingulate cortex	-0.133	0.258	[-0.639 - 0.373]	6.072E-01	9.124E-01
Right isthmus cingulate cortex	-0.357	0.259	[-0.864 - 0.150]	1.678E-01	8.634E-01

Supplementary Table S190. Partial correlations between cortical thickness and PANSS total controlling for age and sex in individuals with schizophrenia

	Partial *R*	Std. Err.	95% CI	*p*-value	FDR *q*-value
Global mean cortical thickness	0.004	0.049	[-0.091 - 0.100]	9.276E-01	9.694E-01
Left hemisphere	0.015	0.055	[-0.092 - 0.122]	7.869E-01	9.694E-01
Right hemisphere	-0.006	0.044	[-0.091 - 0.080]	8.984E-01	9.694E-01
Left fusiform gyrus	-0.020	0.040	[-0.099 - 0.059]	6.146E-01	9.694E-01
Right fusiform gyrus	-0.018	0.041	[-0.098 - 0.062]	6.580E-01	9.694E-01
Left pars opercularis of inferior frontal gyrus	0.034	0.072	[-0.108 - 0.175]	6.420E-01	9.694E-01
Right pars opercularis of inferior frontal gyrus	-0.033	0.056	[-0.142 - 0.076]	5.516E-01	9.694E-01
Left superior temporal gyrus	0.067	0.044	[-0.020 - 0.153]	1.313E-01	9.694E-01
Right superior temporal gyrus	0.047	0.043	[-0.037 - 0.131]	2.766E-01	9.694E-01
Left insula	0.038	0.055	[-0.069 - 0.145]	4.889E-01	9.694E-01
Right insula	0.035	0.040	[-0.044 - 0.113]	3.905E-01	9.694E-01
Left lingual gyrus	-0.013	0.040	[-0.092 - 0.066]	7.425E-01	9.694E-01
Right lingual gyrus	0.061	0.040	[-0.018 - 0.140]	1.298E-01	9.694E-01
Left pars triangularis of inferior frontal gyrus	0.007	0.061	[-0.113 - 0.126]	9.108E-01	9.694E-01
Right pars triangularis of inferior frontal gyrus	0.020	0.042	[-0.062 - 0.102]	6.272E-01	9.694E-01
Left lateral orbitofrontal cortex	-0.056	0.053	[-0.159 - 0.047]	2.846E-01	9.694E-01
Right lateral orbitofrontal cortex	-0.008	0.040	[-0.087 - 0.071]	8.393E-01	9.694E-01
Left rostral middle frontal gyrus	-0.007	0.062	[-0.129 - 0.115]	9.054E-01	9.694E-01
Right rostral middle frontal gyrus	-0.004	0.061	[-0.124 - 0.117]	9.497E-01	9.694E-01
Left middle temporal gyrus	-0.034	0.056	[-0.144 - 0.075]	5.384E-01	9.694E-01
Right middle temporal gyrus	-0.042	0.052	[-0.144 - 0.060]	4.203E-01	9.694E-01
Left superior frontal gyrus	-0.012	0.040	[-0.091 - 0.066]	7.566E-01	9.694E-01
Right superior frontal gyrus	-0.024	0.054	[-0.131 - 0.082]	6.525E-01	9.694E-01
Left pars orbitalis of inferior frontal gyrus	-0.040	0.054	[-0.145 - 0.065]	4.532E-01	9.694E-01
Right pars orbitalis of inferior frontal gyrus	0.004	0.048	[-0.090 - 0.098]	9.329E-01	9.694E-01
Left medial orbitofrontal cortex	0.033	0.044	[-0.054 - 0.120]	4.584E-01	9.694E-01
Right medial orbitofrontal cortex	-0.062	0.040	[-0.141 - 0.017]	1.226E-01	9.694E-01
Left inferior temporal gyrus	-0.048	0.040	[-0.127 - 0.031]	2.358E-01	9.694E-01
Right inferior temporal gyrus	-0.059	0.041	[-0.139 - 0.022]	1.530E-01	9.694E-01
Left isthmus cingulate cortex	0.038	0.062	[-0.084 - 0.161]	5.407E-01	9.694E-01
Right isthmus cingulate cortex	-0.075	0.040	[-0.154 - 0.004]	6.290E-02	9.694E-01
Left banks of superior temporal sulcus	0.010	0.063	[-0.113 - 0.133]	8.707E-01	9.694E-01
Right banks of superior temporal sulcus	-0.006	0.056	[-0.115 - 0.103]	9.141E-01	9.694E-01
Left supramarginal gyrus	0.022	0.058	[-0.091 - 0.135]	7.019E-01	9.694E-01
Right supramarginal gyrus	0.041	0.040	[-0.038 - 0.119]	3.143E-01	9.694E-01
Left caudal middle frontal gyrus	0.008	0.053	[-0.096 - 0.112]	8.805E-01	9.694E-01
Right caudal middle frontal gyrus	-0.076	0.040	[-0.155 - 0.003]	6.004E-02	9.694E-01
Left frontal pole	-0.014	0.049	[-0.111 - 0.083]	7.765E-01	9.694E-01
Right frontal pole	-0.004	0.041	[-0.084 - 0.077]	9.299E-01	9.694E-01
Left posterior cingulate cortex	0.015	0.071	[-0.124 - 0.153]	8.364E-01	9.694E-01
Right posterior cingulate cortex	-0.018	0.060	[-0.136 - 0.100]	7.618E-01	9.694E-01
Left lateral occipital cortex	-0.045	0.059	[-0.160 - 0.071]	4.497E-01	9.694E-01
Right lateral occipital cortex	-0.033	0.040	[-0.112 - 0.046]	4.145E-01	9.694E-01
Left precentral gyrus	0.072	0.071	[-0.067 - 0.211]	3.086E-01	9.694E-01
Right precentral gyrus	0.002	0.040	[-0.077 - 0.081]	9.637E-01	9.694E-01
Left parahippocampal gyrus	0.036	0.040	[-0.042 - 0.115]	3.657E-01	9.694E-01
Right parahippocampal gyrus	0.048	0.040	[-0.031 - 0.127]	2.306E-01	9.694E-01
Left inferior parietal cortex	0.087	0.059	[-0.028 - 0.202]	1.368E-01	9.694E-01
Right inferior parietal cortex	-0.005	0.040	[-0.084 - 0.074]	8.970E-01	9.694E-01
Left transverse temporal gyrus	0.040	0.045	[-0.048 - 0.128]	3.760E-01	9.694E-01
Right transverse temporal gyrus	0.037	0.040	[-0.042 - 0.116]	3.560E-01	9.694E-01
Left postcentral gyrus	0.032	0.055	[-0.075 - 0.140]	5.519E-01	9.694E-01
Right postcentral gyrus	0.021	0.040	[-0.058 - 0.100]	5.968E-01	9.694E-01
Left precuneus	0.002	0.040	[-0.077 - 0.080]	9.694E-01	9.694E-01
Right precuneus	0.097	0.086	[-0.071 - 0.266]	2.586E-01	9.694E-01
Left caudal anterior cingulate cortex	-0.003	0.055	[-0.111 - 0.106]	9.615E-01	9.694E-01
Right caudal anterior cingulate cortex	0.006	0.073	[-0.137 - 0.149]	9.388E-01	9.694E-01
Left cuneus	-0.017	0.058	[-0.130 - 0.096]	7.627E-01	9.694E-01
Right cuneus	0.018	0.040	[-0.061 - 0.097]	6.481E-01	9.694E-01
Left rostral anterior cingulate cortex	0.062	0.054	[-0.045 - 0.168]	2.559E-01	9.694E-01
Right rostral anterior cingulate cortex	0.025	0.065	[-0.102 - 0.152]	6.976E-01	9.694E-01
Left pericalcarine cortex	0.084	0.058	[-0.029 - 0.197]	1.430E-01	9.694E-01
Right pericalcarine cortex	0.081	0.043	[-0.002 - 0.165]	5.637E-02	9.694E-01
Left paracentral lobule	0.034	0.045	[-0.054 - 0.122]	4.481E-01	9.694E-01
Right paracentral lobule	0.012	0.040	[-0.067 - 0.091]	7.680E-01	9.694E-01
Left superior parietal cortex	0.094	0.086	[-0.073 - 0.262]	2.695E-01	9.694E-01
Right superior parietal cortex	0.007	0.040	[-0.072 - 0.086]	8.638E-01	9.694E-01
Left temporal pole	0.017	0.058	[-0.096 - 0.131]	7.662E-01	9.694E-01
Right temporal pole	0.097	0.060	[-0.020 - 0.215]	1.042E-01	9.694E-01
Left entorhinal cortex	-0.007	0.040	[-0.086 - 0.072]	8.543E-01	9.694E-01
Right entorhinal cortex	0.076	0.077	[-0.075 - 0.227]	3.221E-01	9.694E-01

Supplementary Table S191. Partial correlations between cortical thickness and PANSS positive controlling for age and sex in individuals with schizophrenia

	Partial *R*	Std. Err.	95% CI	*p*-value	FDR *q*-value
Global mean cortical thickness	0.035	0.051	[-0.064 - 0.134]	4.873E-01	9.538E-01
Left hemisphere	0.042	0.057	[-0.071 - 0.154]	4.663E-01	9.538E-01
Right hemisphere	0.028	0.046	[-0.062 - 0.118]	5.402E-01	9.538E-01
Left fusiform gyrus	0.017	0.055	[-0.091 - 0.125]	7.537E-01	9.791E-01
Right fusiform gyrus	0.037	0.068	[-0.097 - 0.171]	5.878E-01	9.538E-01
Left pars opercularis of inferior frontal gyrus	0.006	0.050	[-0.093 - 0.105]	9.116E-01	9.791E-01
Right pars opercularis of inferior frontal gyrus	0.015	0.040	[-0.064 - 0.094]	7.139E-01	9.791E-01
Left superior temporal gyrus	0.067	0.040	[-0.011 - 0.146]	9.364E-02	8.004E-01
Right superior temporal gyrus	0.057	0.040	[-0.021 - 0.136]	1.533E-01	8.004E-01
Left insula	0.048	0.058	[-0.067 - 0.162]	4.149E-01	9.538E-01
Right insula	0.070	0.040	[-0.009 - 0.149]	8.155E-02	8.004E-01
Left lingual gyrus	0.059	0.040	[-0.019 - 0.138]	1.400E-01	8.004E-01
Right lingual gyrus	0.116	0.040	[0.037 - 0.195]	3.876E-03	2.752E-01
Left pars triangularis of inferior frontal gyrus	0.044	0.080	[-0.113 - 0.200]	5.855E-01	9.538E-01
Right pars triangularis of inferior frontal gyrus	0.089	0.057	[-0.022 - 0.201]	1.172E-01	8.004E-01
Left lateral orbitofrontal cortex	-0.024	0.058	[-0.138 - 0.089]	6.750E-01	9.791E-01
Right lateral orbitofrontal cortex	0.014	0.040	[-0.065 - 0.093]	7.262E-01	9.791E-01
Left rostral middle frontal gyrus	-0.013	0.058	[-0.127 - 0.102]	8.274E-01	9.791E-01
Right rostral middle frontal gyrus	-0.036	0.045	[-0.123 - 0.052]	4.212E-01	9.538E-01
Left middle temporal gyrus	0.043	0.068	[-0.090 - 0.176]	5.243E-01	9.538E-01
Right middle temporal gyrus	0.037	0.065	[-0.090 - 0.165]	5.665E-01	9.538E-01
Left superior frontal gyrus	-0.036	0.046	[-0.126 - 0.055]	4.423E-01	9.538E-01
Right superior frontal gyrus	-0.037	0.046	[-0.128 - 0.053]	4.147E-01	9.538E-01
Left pars orbitalis of inferior frontal gyrus	0.006	0.041	[-0.074 - 0.086]	8.831E-01	9.791E-01
Right pars orbitalis of inferior frontal gyrus	0.069	0.040	[-0.010 - 0.147]	8.873E-02	8.004E-01
Left medial orbitofrontal cortex	0.035	0.059	[-0.080 - 0.151]	5.487E-01	9.538E-01
Right medial orbitofrontal cortex	-0.029	0.047	[-0.120 - 0.063]	5.409E-01	9.538E-01
Left inferior temporal gyrus	-0.030	0.045	[-0.118 - 0.058]	5.016E-01	9.538E-01
Right inferior temporal gyrus	0.033	0.051	[-0.066 - 0.133]	5.109E-01	9.538E-01
Left isthmus cingulate cortex	0.041	0.055	[-0.068 - 0.149]	4.615E-01	9.538E-01
Right isthmus cingulate cortex	-0.057	0.055	[-0.164 - 0.051]	3.022E-01	9.538E-01
Left banks of superior temporal sulcus	0.003	0.042	[-0.080 - 0.086]	9.371E-01	9.791E-01
Right banks of superior temporal sulcus	0.034	0.046	[-0.057 - 0.125]	4.663E-01	9.538E-01
Left supramarginal gyrus	0.042	0.050	[-0.055 - 0.140]	3.963E-01	9.538E-01
Right supramarginal gyrus	0.062	0.040	[-0.017 - 0.141]	1.220E-01	8.004E-01
Left caudal middle frontal gyrus	-0.003	0.053	[-0.106 - 0.100]	9.515E-01	9.791E-01
Right caudal middle frontal gyrus	-0.061	0.046	[-0.151 - 0.030]	1.871E-01	8.301E-01
Left frontal pole	-0.001	0.040	[-0.080 - 0.078]	9.802E-01	9.917E-01
Right frontal pole	0.036	0.040	[-0.043 - 0.115]	3.757E-01	9.538E-01
Left posterior cingulate cortex	0.020	0.085	[-0.147 - 0.187]	8.156E-01	9.791E-01
Right posterior cingulate cortex	-0.001	0.064	[-0.126 - 0.124]	9.917E-01	9.917E-01
Left lateral occipital cortex	-0.010	0.052	[-0.112 - 0.092]	8.463E-01	9.791E-01
Right lateral occipital cortex	-0.008	0.040	[-0.087 - 0.071]	8.473E-01	9.791E-01
Left precentral gyrus	0.072	0.069	[-0.063 - 0.207]	2.941E-01	9.538E-01
Right precentral gyrus	-0.011	0.052	[-0.113 - 0.090]	8.287E-01	9.791E-01
Left parahippocampal gyrus	0.054	0.049	[-0.043 - 0.150]	2.746E-01	9.538E-01
Right parahippocampal gyrus	0.076	0.052	[-0.026 - 0.177]	1.442E-01	8.004E-01
Left inferior parietal cortex	0.075	0.054	[-0.030 - 0.180]	1.607E-01	8.004E-01
Right inferior parietal cortex	0.010	0.047	[-0.081 - 0.102]	8.249E-01	9.791E-01
Left transverse temporal gyrus	0.051	0.046	[-0.039 - 0.141]	2.643E-01	9.538E-01
Right transverse temporal gyrus	0.052	0.040	[-0.027 - 0.131]	1.994E-01	8.328E-01
Left postcentral gyrus	0.041	0.062	[-0.081 - 0.163]	5.125E-01	9.538E-01
Right postcentral gyrus	0.028	0.043	[-0.056 - 0.113]	5.114E-01	9.538E-01
Left precuneus	0.015	0.046	[-0.076 - 0.106]	7.420E-01	9.791E-01
Right precuneus	0.034	0.042	[-0.049 - 0.117]	4.201E-01	9.538E-01
Left caudal anterior cingulate cortex	0.014	0.061	[-0.106 - 0.133]	8.222E-01	9.791E-01
Right caudal anterior cingulate cortex	0.015	0.073	[-0.129 - 0.159]	8.381E-01	9.791E-01
Left cuneus	0.038	0.076	[-0.111 - 0.187]	6.180E-01	9.538E-01
Right cuneus	0.003	0.040	[-0.076 - 0.081]	9.504E-01	9.791E-01
Left rostral anterior cingulate cortex	0.074	0.052	[-0.028 - 0.176]	1.528E-01	8.004E-01
Right rostral anterior cingulate cortex	-0.006	0.044	[-0.093 - 0.080]	8.846E-01	9.791E-01
Left pericalcarine cortex	0.105	0.058	[-0.009 - 0.219]	7.151E-02	8.004E-01
Right pericalcarine cortex	0.082	0.040	[0.003 - 0.161]	4.261E-02	8.004E-01
Left paracentral lobule	0.024	0.060	[-0.093 - 0.141]	6.859E-01	9.791E-01
Right paracentral lobule	0.003	0.040	[-0.076 - 0.082]	9.348E-01	9.791E-01
Left superior parietal cortex	0.088	0.051	[-0.011 - 0.188]	8.142E-02	8.004E-01
Right superior parietal cortex	0.020	0.040	[-0.059 - 0.099]	6.143E-01	9.538E-01
Left temporal pole	-0.019	0.063	[-0.143 - 0.105]	7.634E-01	9.791E-01
Right temporal pole	0.070	0.051	[-0.030 - 0.169]	1.691E-01	8.004E-01
Left entorhinal cortex	-0.004	0.040	[-0.083 - 0.074]	9.122E-01	9.791E-01
Right entorhinal cortex	0.027	0.051	[-0.074 - 0.127]	5.998E-01	9.538E-01

Supplementary Table S192. Partial correlations between cortical thickness and PANSS negative controlling for age and sex in individuals with schizophrenia

	Partial *R*	Std. Err.	95% CI	*p*-value	FDR *q*-value
Global mean cortical thickness	-0.022	0.064	[-0.147 - 0.103]	7.280E-01	9.981E-01
Left hemisphere	-0.009	0.064	[-0.135 - 0.117]	8.845E-01	9.981E-01
Right hemisphere	-0.033	0.063	[-0.155 - 0.090]	6.023E-01	9.981E-01
Left fusiform gyrus	-0.074	0.040	[-0.153 - 0.005]	6.526E-02	9.981E-01
Right fusiform gyrus	-0.073	0.052	[-0.175 - 0.029]	1.606E-01	9.981E-01
Left pars opercularis of inferior frontal gyrus	0.015	0.069	[-0.121 - 0.151]	8.276E-01	9.981E-01
Right pars opercularis of inferior frontal gyrus	-0.059	0.049	[-0.155 - 0.037]	2.320E-01	9.981E-01
Left superior temporal gyrus	0.002	0.080	[-0.155 - 0.159]	9.790E-01	9.981E-01
Right superior temporal gyrus	-0.026	0.057	[-0.138 - 0.087]	6.557E-01	9.981E-01
Left insula	0.024	0.059	[-0.091 - 0.139]	6.832E-01	9.981E-01
Right insula	0.002	0.040	[-0.077 - 0.081]	9.680E-01	9.981E-01
Left lingual gyrus	-0.030	0.040	[-0.109 - 0.049]	4.606E-01	9.981E-01
Right lingual gyrus	-0.009	0.040	[-0.088 - 0.070]	8.251E-01	9.981E-01
Left pars triangularis of inferior frontal gyrus	-0.003	0.050	[-0.101 - 0.095]	9.496E-01	9.981E-01
Right pars triangularis of inferior frontal gyrus	-0.023	0.044	[-0.111 - 0.064]	5.970E-01	9.981E-01
Left lateral orbitofrontal cortex	-0.081	0.042	[-0.163 - 0.000]	5.073E-02	9.981E-01
Right lateral orbitofrontal cortex	-0.023	0.048	[-0.117 - 0.071]	6.318E-01	9.981E-01
Left rostral middle frontal gyrus	-0.001	0.065	[-0.129 - 0.127]	9.835E-01	9.981E-01
Right rostral middle frontal gyrus	0.020	0.074	[-0.125 - 0.166]	7.841E-01	9.981E-01
Left middle temporal gyrus	-0.072	0.065	[-0.200 - 0.056]	2.687E-01	9.981E-01
Right middle temporal gyrus	-0.055	0.095	[-0.241 - 0.131]	5.621E-01	9.981E-01
Left superior frontal gyrus	-0.008	0.045	[-0.097 - 0.080]	8.547E-01	9.981E-01
Right superior frontal gyrus	-0.005	0.060	[-0.122 - 0.113]	9.360E-01	9.981E-01
Left pars orbitalis of inferior frontal gyrus	-0.044	0.047	[-0.137 - 0.048]	3.481E-01	9.981E-01
Right pars orbitalis of inferior frontal gyrus	-0.056	0.043	[-0.140 - 0.029]	1.947E-01	9.981E-01
Left medial orbitofrontal cortex	-0.004	0.040	[-0.083 - 0.075]	9.192E-01	9.981E-01
Right medial orbitofrontal cortex	-0.051	0.040	[-0.130 - 0.028]	2.021E-01	9.981E-01
Left inferior temporal gyrus	-0.050	0.040	[-0.129 - 0.029]	2.134E-01	9.981E-01
Right inferior temporal gyrus	-0.103	0.056	[-0.212 - 0.006]	6.491E-02	9.981E-01
Left isthmus cingulate cortex	-0.005	0.045	[-0.094 - 0.084]	9.129E-01	9.981E-01
Right isthmus cingulate cortex	-0.093	0.058	[-0.206 - 0.020]	1.081E-01	9.981E-01
Left banks of superior temporal sulcus	0.022	0.083	[-0.140 - 0.184]	7.913E-01	9.981E-01
Right banks of superior temporal sulcus	-0.023	0.075	[-0.169 - 0.124]	7.626E-01	9.981E-01
Left supramarginal gyrus	0.016	0.075	[-0.131 - 0.163]	8.322E-01	9.981E-01
Right supramarginal gyrus	0.013	0.050	[-0.085 - 0.111]	7.985E-01	9.981E-01
Left caudal middle frontal gyrus	0.001	0.054	[-0.106 - 0.108]	9.844E-01	9.981E-01
Right caudal middle frontal gyrus	-0.072	0.040	[-0.151 - 0.007]	7.259E-02	9.981E-01
Left frontal pole	-0.023	0.058	[-0.136 - 0.090]	6.898E-01	9.981E-01
Right frontal pole	-0.054	0.041	[-0.134 - 0.026]	1.865E-01	9.981E-01
Left posterior cingulate cortex	-0.006	0.051	[-0.107 - 0.095]	9.087E-01	9.981E-01
Right posterior cingulate cortex	-0.014	0.066	[-0.143 - 0.115]	8.317E-01	9.981E-01
Left lateral occipital cortex	-0.053	0.063	[-0.177 - 0.071]	4.044E-01	9.981E-01
Right lateral occipital cortex	-0.046	0.054	[-0.152 - 0.060]	3.949E-01	9.981E-01
Left precentral gyrus	0.031	0.061	[-0.088 - 0.150]	6.107E-01	9.981E-01
Right precentral gyrus	0.003	0.043	[-0.081 - 0.087]	9.424E-01	9.981E-01
Left parahippocampal gyrus	0.000	0.040	[-0.079 - 0.079]	9.981E-01	9.981E-01
Right parahippocampal gyrus	0.013	0.040	[-0.066 - 0.092]	7.510E-01	9.981E-01
Left inferior parietal cortex	0.066	0.049	[-0.030 - 0.162]	1.790E-01	9.981E-01
Right inferior parietal cortex	-0.013	0.064	[-0.138 - 0.113]	8.453E-01	9.981E-01
Left transverse temporal gyrus	0.018	0.040	[-0.061 - 0.096]	6.627E-01	9.981E-01
Right transverse temporal gyrus	0.009	0.050	[-0.088 - 0.107]	8.488E-01	9.981E-01
Left postcentral gyrus	0.032	0.052	[-0.069 - 0.134]	5.313E-01	9.981E-01
Right postcentral gyrus	0.023	0.042	[-0.059 - 0.105]	5.805E-01	9.981E-01
Left precuneus	0.009	0.091	[-0.169 - 0.187]	9.229E-01	9.981E-01
Right precuneus	0.031	0.041	[-0.048 - 0.111]	4.400E-01	9.981E-01
Left caudal anterior cingulate cortex	0.004	0.052	[-0.097 - 0.105]	9.375E-01	9.981E-01
Right caudal anterior cingulate cortex	0.019	0.058	[-0.094 - 0.131]	7.451E-01	9.981E-01
Left cuneus	0.000	0.053	[-0.103 - 0.104]	9.940E-01	9.981E-01
Right cuneus	0.042	0.040	[-0.037 - 0.121]	2.949E-01	9.981E-01
Left rostral anterior cingulate cortex	0.062	0.057	[-0.049 - 0.174]	2.715E-01	9.981E-01
Right rostral anterior cingulate cortex	0.045	0.065	[-0.081 - 0.172]	4.825E-01	9.981E-01
Left pericalcarine cortex	0.052	0.040	[-0.027 - 0.131]	1.979E-01	9.981E-01
Right pericalcarine cortex	0.086	0.040	[0.007 - 0.165]	3.222E-02	9.981E-01
Left paracentral lobule	0.050	0.040	[-0.029 - 0.129]	2.163E-01	9.981E-01
Right paracentral lobule	0.013	0.040	[-0.066 - 0.091]	7.549E-01	9.981E-01
Left superior parietal cortex	0.049	0.077	[-0.101 - 0.200]	5.201E-01	9.981E-01
Right superior parietal cortex	-0.034	0.075	[-0.181 - 0.112]	6.443E-01	9.981E-01
Left temporal pole	0.026	0.055	[-0.081 - 0.134]	6.310E-01	9.981E-01
Right temporal pole	0.092	0.054	[-0.013 - 0.197]	8.498E-02	9.981E-01
Left entorhinal cortex	-0.020	0.040	[-0.098 - 0.059]	6.276E-01	9.981E-01
Right entorhinal cortex	0.027	0.051	[-0.072 - 0.127]	5.896E-01	9.981E-01

Supplementary Table S193. Partial correlations between cortical thickness and PANSS general controlling for age and sex in individuals with schizophrenia

	Partial *R*	Std. Err.	95% CI	*p*-value	FDR *q*-value
Global mean cortical thickness	-0.007	0.040	[-0.086 - 0.072]	8.616E-01	9.979E-01
Left hemisphere	0.002	0.041	[-0.079 - 0.083]	9.574E-01	9.979E-01
Right hemisphere	-0.015	0.040	[-0.094 - 0.064]	7.069E-01	9.979E-01
Left fusiform gyrus	-0.011	0.040	[-0.089 - 0.068]	7.927E-01	9.979E-01
Right fusiform gyrus	-0.019	0.040	[-0.097 - 0.060]	6.444E-01	9.979E-01
Left pars opercularis of inferior frontal gyrus	0.024	0.061	[-0.096 - 0.143]	6.954E-01	9.979E-01
Right pars opercularis of inferior frontal gyrus	-0.036	0.054	[-0.141 - 0.069]	5.028E-01	9.979E-01
Left superior temporal gyrus	0.073	0.040	[-0.006 - 0.152]	7.014E-02	9.979E-01
Right superior temporal gyrus	0.056	0.040	[-0.023 - 0.135]	1.613E-01	9.979E-01
Left insula	0.028	0.050	[-0.070 - 0.127]	5.724E-01	9.979E-01
Right insula	0.031	0.040	[-0.048 - 0.110]	4.467E-01	9.979E-01
Left lingual gyrus	-0.035	0.040	[-0.114 - 0.044]	3.888E-01	9.979E-01
Right lingual gyrus	0.068	0.048	[-0.025 - 0.161]	1.528E-01	9.979E-01
Left pars triangularis of inferior frontal gyrus	-0.010	0.050	[-0.108 - 0.089]	8.445E-01	9.979E-01
Right pars triangularis of inferior frontal gyrus	0.007	0.040	[-0.072 - 0.086]	8.571E-01	9.979E-01
Left lateral orbitofrontal cortex	-0.054	0.057	[-0.166 - 0.058]	3.446E-01	9.979E-01
Right lateral orbitofrontal cortex	-0.020	0.040	[-0.099 - 0.059]	6.148E-01	9.979E-01
Left rostral middle frontal gyrus	-0.029	0.054	[-0.134 - 0.076]	5.872E-01	9.979E-01
Right rostral middle frontal gyrus	-0.029	0.051	[-0.129 - 0.071]	5.663E-01	9.979E-01
Left middle temporal gyrus	-0.042	0.041	[-0.122 - 0.037]	2.961E-01	9.979E-01
Right middle temporal gyrus	-0.045	0.040	[-0.124 - 0.034]	2.616E-01	9.979E-01
Left superior frontal gyrus	-0.009	0.040	[-0.088 - 0.070]	8.220E-01	9.979E-01
Right superior frontal gyrus	-0.034	0.043	[-0.118 - 0.050]	4.331E-01	9.979E-01
Left pars orbitalis of inferior frontal gyrus	-0.044	0.045	[-0.132 - 0.043]	3.192E-01	9.979E-01
Right pars orbitalis of inferior frontal gyrus	0.008	0.058	[-0.105 - 0.122]	8.850E-01	9.979E-01
Left medial orbitofrontal cortex	0.043	0.046	[-0.047 - 0.133]	3.516E-01	9.979E-01
Right medial orbitofrontal cortex	-0.079	0.040	[-0.158 - 0.000]	4.869E-02	9.979E-01
Left inferior temporal gyrus	-0.048	0.040	[-0.127 - 0.031]	2.306E-01	9.979E-01
Right inferior temporal gyrus	-0.064	0.040	[-0.143 - 0.015]	1.137E-01	9.979E-01
Left isthmus cingulate cortex	0.056	0.068	[-0.077 - 0.190]	4.083E-01	9.979E-01
Right isthmus cingulate cortex	-0.057	0.040	[-0.136 - 0.022]	1.596E-01	9.979E-01
Left banks of superior temporal sulcus	0.015	0.047	[-0.076 - 0.106]	7.472E-01	9.979E-01
Right banks of superior temporal sulcus	-0.019	0.040	[-0.098 - 0.059]	6.292E-01	9.979E-01
Left supramarginal gyrus	-0.001	0.047	[-0.094 - 0.091]	9.774E-01	9.979E-01
Right supramarginal gyrus	0.024	0.040	[-0.055 - 0.103]	5.545E-01	9.979E-01
Left caudal middle frontal gyrus	0.005	0.050	[-0.092 - 0.103]	9.155E-01	9.979E-01
Right caudal middle frontal gyrus	-0.077	0.040	[-0.156 - 0.002]	5.553E-02	9.979E-01
Left frontal pole	-0.016	0.049	[-0.112 - 0.079]	7.353E-01	9.979E-01
Right frontal pole	0.002	0.043	[-0.081 - 0.086]	9.602E-01	9.979E-01
Left posterior cingulate cortex	0.014	0.064	[-0.111 - 0.139]	8.226E-01	9.979E-01
Right posterior cingulate cortex	-0.031	0.043	[-0.116 - 0.054]	4.706E-01	9.979E-01
Left lateral occipital cortex	-0.040	0.040	[-0.119 - 0.039]	3.210E-01	9.979E-01
Right lateral occipital cortex	-0.040	0.040	[-0.119 - 0.039]	3.160E-01	9.979E-01
Left precentral gyrus	0.057	0.062	[-0.065 - 0.178]	3.618E-01	9.979E-01
Right precentral gyrus	-0.001	0.040	[-0.080 - 0.078]	9.834E-01	9.979E-01
Left parahippocampal gyrus	0.040	0.040	[-0.039 - 0.119]	3.212E-01	9.979E-01
Right parahippocampal gyrus	0.041	0.040	[-0.038 - 0.120]	3.078E-01	9.979E-01
Left inferior parietal cortex	0.136	0.091	[-0.042 - 0.315]	1.343E-01	9.979E-01
Right inferior parietal cortex	-0.005	0.040	[-0.084 - 0.074]	8.981E-01	9.979E-01
Left transverse temporal gyrus	0.039	0.040	[-0.040 - 0.118]	3.297E-01	9.979E-01
Right transverse temporal gyrus	0.035	0.040	[-0.044 - 0.114]	3.884E-01	9.979E-01
Left postcentral gyrus	0.010	0.045	[-0.079 - 0.099]	8.315E-01	9.979E-01
Right postcentral gyrus	0.002	0.040	[-0.077 - 0.081]	9.630E-01	9.979E-01
Left precuneus	0.000	0.040	[-0.079 - 0.079]	9.979E-01	9.979E-01
Right precuneus	0.044	0.055	[-0.064 - 0.152]	4.264E-01	9.979E-01
Left caudal anterior cingulate cortex	-0.018	0.045	[-0.107 - 0.071]	6.941E-01	9.979E-01
Right caudal anterior cingulate cortex	-0.006	0.069	[-0.141 - 0.129]	9.281E-01	9.979E-01
Left cuneus	-0.033	0.041	[-0.113 - 0.048]	4.237E-01	9.979E-01
Right cuneus	0.006	0.040	[-0.073 - 0.085]	8.860E-01	9.979E-01
Left rostral anterior cingulate cortex	0.043	0.058	[-0.072 - 0.157]	4.636E-01	9.979E-01
Right rostral anterior cingulate cortex	0.021	0.066	[-0.109 - 0.150]	7.557E-01	9.979E-01
Left pericalcarine cortex	0.053	0.040	[-0.026 - 0.131]	1.917E-01	9.979E-01
Right pericalcarine cortex	0.074	0.040	[-0.005 - 0.153]	6.682E-02	9.979E-01
Left paracentral lobule	0.016	0.041	[-0.065 - 0.098]	6.924E-01	9.979E-01
Right paracentral lobule	0.007	0.040	[-0.072 - 0.085]	8.715E-01	9.979E-01
Left superior parietal cortex	0.051	0.057	[-0.060 - 0.162]	3.700E-01	9.979E-01
Right superior parietal cortex	0.000	0.040	[-0.079 - 0.079]	9.946E-01	9.979E-01
Left temporal pole	0.021	0.057	[-0.090 - 0.133]	7.084E-01	9.979E-01
Right temporal pole	0.082	0.060	[-0.037 - 0.200]	1.758E-01	9.979E-01
Left entorhinal cortex	-0.007	0.040	[-0.085 - 0.072]	8.700E-01	9.979E-01
Right entorhinal cortex	0.040	0.059	[-0.076 - 0.156]	4.992E-01	9.979E-01

Supplementary Table S194. Partial correlations between cortical thickness and severity of symptoms measured by the HDRS-17 controlling for age and sex in individuals with major depressive disorder

	Partial *R*	Std. Err.	95% CI	*p*-value	FDR *q*-value
Global mean cortical thickness	-0.124	0.051	[-0.224 - -0.024]	1.495E-02	1.096E-01
Left hemisphere	-0.128	0.051	[-0.229 - -0.028]	1.200E-02	1.065E-01
Right hemisphere	-0.113	0.054	[-0.219 - -0.007]	3.589E-02	1.883E-01
Left fusiform gyrus	-0.060	0.051	[-0.161 - 0.040]	2.410E-01	4.388E-01
Right fusiform gyrus	-0.132	0.051	[-0.232 - -0.032]	9.821E-03	9.961E-02
Left pars opercularis of inferior frontal gyrus	-0.075	0.061	[-0.194 - 0.044]	2.164E-01	4.363E-01
Right pars opercularis of inferior frontal gyrus	-0.081	0.059	[-0.197 - 0.036]	1.752E-01	4.218E-01
Left superior temporal gyrus	-0.050	0.052	[-0.151 - 0.052]	3.357E-01	5.017E-01
Right superior temporal gyrus	-0.082	0.068	[-0.214 - 0.051]	2.274E-01	4.363E-01
Left insula	-0.057	0.062	[-0.178 - 0.063]	3.533E-01	5.017E-01
Right insula	-0.065	0.058	[-0.178 - 0.049]	2.631E-01	4.671E-01
Left lingual gyrus	-0.034	0.060	[-0.151 - 0.083]	5.732E-01	7.017E-01
Right lingual gyrus	-0.021	0.070	[-0.159 - 0.116]	7.600E-01	8.175E-01
Left pars triangularis of inferior frontal gyrus	-0.034	0.058	[-0.148 - 0.080]	5.598E-01	6.972E-01
Right pars triangularis of inferior frontal gyrus	-0.077	0.061	[-0.196 - 0.041]	2.020E-01	4.218E-01
Left lateral orbitofrontal cortex	-0.121	0.051	[-0.221 - -0.021]	1.784E-02	1.151E-01
Right lateral orbitofrontal cortex	-0.035	0.088	[-0.208 - 0.139]	6.962E-01	7.847E-01
Left rostral middle frontal gyrus	-0.155	0.056	[-0.266 - -0.045]	5.662E-03	9.961E-02
Right rostral middle frontal gyrus	-0.083	0.064	[-0.207 - 0.042]	1.954E-01	4.218E-01
Left middle temporal gyrus	-0.075	0.053	[-0.180 - 0.029]	1.586E-01	4.171E-01
Right middle temporal gyrus	-0.055	0.059	[-0.171 - 0.061]	3.509E-01	5.017E-01
Left superior frontal gyrus	-0.168	0.051	[-0.268 - -0.068]	1.025E-03	4.281E-02
Right superior frontal gyrus	-0.153	0.051	[-0.253 - -0.053]	2.787E-03	6.596E-02
Left pars orbitalis of inferior frontal gyrus	-0.119	0.059	[-0.235 - -0.003]	4.346E-02	1.883E-01
Right pars orbitalis of inferior frontal gyrus	-0.063	0.067	[-0.194 - 0.069]	3.484E-01	5.017E-01
Left medial orbitofrontal cortex	-0.105	0.051	[-0.206 - -0.005]	3.899E-02	1.883E-01
Right medial orbitofrontal cortex	-0.089	0.059	[-0.206 - 0.027]	1.319E-01	3.955E-01
Left inferior temporal gyrus	-0.019	0.062	[-0.141 - 0.102]	7.555E-01	8.175E-01
Right inferior temporal gyrus	-0.024	0.051	[-0.125 - 0.076]	6.320E-01	7.368E-01
Left isthmus cingulate cortex	-0.037	0.060	[-0.155 - 0.081]	5.416E-01	6.972E-01
Right isthmus cingulate cortex	-0.160	0.078	[-0.314 - -0.007]	4.077E-02	1.883E-01
Left banks of superior temporal sulcus	-0.033	0.051	[-0.134 - 0.067]	5.165E-01	6.791E-01
Right banks of superior temporal sulcus	-0.054	0.051	[-0.154 - 0.046]	2.911E-01	4.697E-01
Left supramarginal gyrus	-0.076	0.062	[-0.198 - 0.047]	2.258E-01	4.363E-01
Right supramarginal gyrus	-0.076	0.051	[-0.176 - 0.025]	1.395E-01	3.955E-01
Left caudal middle frontal gyrus	-0.137	0.053	[-0.240 - -0.033]	9.457E-03	9.961E-02
Right caudal middle frontal gyrus	-0.165	0.085	[-0.331 - 0.001]	5.097E-02	1.903E-01
Left frontal pole	-0.075	0.051	[-0.175 - 0.026]	1.448E-01	3.955E-01
Right frontal pole	-0.165	0.051	[-0.266 - -0.065]	1.206E-03	4.281E-02
Left posterior cingulate cortex	-0.061	0.064	[-0.186 - 0.064]	3.380E-01	5.017E-01
Right posterior cingulate cortex	-0.110	0.052	[-0.212 - -0.008]	3.414E-02	1.883E-01
Left lateral occipital cortex	-0.093	0.051	[-0.193 - 0.007]	6.934E-02	2.345E-01
Right lateral occipital cortex	-0.140	0.071	[-0.280 - 0.000]	4.963E-02	1.903E-01
Left precentral gyrus	-0.152	0.058	[-0.267 - -0.038]	8.873E-03	9.961E-02
Right precentral gyrus	-0.084	0.085	[-0.250 - 0.082]	3.229E-01	5.017E-01
Left parahippocampal gyrus	-0.053	0.069	[-0.189 - 0.084]	4.489E-01	6.013E-01
Right parahippocampal gyrus	0.003	0.057	[-0.108 - 0.115]	9.545E-01	9.545E-01
Left inferior parietal cortex	-0.053	0.067	[-0.184 - 0.078]	4.276E-01	5.839E-01
Right inferior parietal cortex	-0.036	0.071	[-0.175 - 0.104]	6.161E-01	7.368E-01
Left transverse temporal gyrus	-0.067	0.052	[-0.169 - 0.034]	1.936E-01	4.218E-01
Right transverse temporal gyrus	0.012	0.051	[-0.089 - 0.112]	8.210E-01	8.700E-01
Left postcentral gyrus	0.019	0.051	[-0.082 - 0.119]	7.160E-01	7.943E-01
Right postcentral gyrus	-0.074	0.053	[-0.178 - 0.031]	1.656E-01	4.198E-01
Left precuneus	-0.126	0.065	[-0.254 - 0.002]	5.361E-02	1.903E-01
Right precuneus	-0.095	0.074	[-0.241 - 0.051]	2.006E-01	4.218E-01
Left caudal anterior cingulate cortex	-0.075	0.051	[-0.175 - 0.026]	1.444E-01	3.955E-01
Right caudal anterior cingulate cortex	-0.060	0.051	[-0.161 - 0.040]	2.367E-01	4.388E-01
Left cuneus	-0.089	0.082	[-0.250 - 0.072]	2.789E-01	4.697E-01
Right cuneus	-0.013	0.082	[-0.173 - 0.147]	8.734E-01	8.859E-01
Left rostral anterior cingulate cortex	0.020	0.051	[-0.080 - 0.120]	6.908E-01	7.847E-01
Right rostral anterior cingulate cortex	0.041	0.068	[-0.093 - 0.175]	5.516E-01	6.972E-01
Left pericalcarine cortex	-0.086	0.081	[-0.243 - 0.072]	2.883E-01	4.697E-01
Right pericalcarine cortex	-0.013	0.078	[-0.166 - 0.140]	8.676E-01	8.859E-01
Left paracentral lobule	-0.060	0.055	[-0.167 - 0.047]	2.711E-01	4.695E-01
Right paracentral lobule	-0.068	0.051	[-0.168 - 0.032]	1.813E-01	4.218E-01
Left superior parietal cortex	-0.124	0.051	[-0.224 - -0.024]	1.544E-02	1.096E-01
Right superior parietal cortex	-0.102	0.051	[-0.203 - -0.002]	4.508E-02	1.883E-01
Left temporal pole	-0.034	0.071	[-0.172 - 0.105]	6.330E-01	7.368E-01
Right temporal pole	0.011	0.056	[-0.098 - 0.121]	8.422E-01	8.794E-01
Left entorhinal cortex	0.041	0.051	[-0.059 - 0.141]	4.211E-01	5.839E-01
Right entorhinal cortex	0.077	0.051	[-0.023 - 0.177]	1.335E-01	3.955E-01

Supplementary Table S195. Partial correlations between cortical thickness and severity of symptoms measured by the HDRS-17 controlling for age and sex in individuals with major depressive disorder over 21 years of old

	Partial *R*	Std. Err.	95% CI	*p*-value	FDR *q*-value
Global mean cortical thickness	-0.123	0.052	[-0.224 - -0.022]	1.710E-02	1.172E-01
Left hemisphere	-0.128	0.052	[-0.229 - -0.026]	1.342E-02	1.172E-01
Right hemisphere	-0.114	0.052	[-0.215 - -0.012]	2.779E-02	1.644E-01
Left fusiform gyrus	-0.060	0.052	[-0.162 - 0.041]	2.451E-01	4.818E-01
Right fusiform gyrus	-0.123	0.052	[-0.224 - -0.022]	1.708E-02	1.172E-01
Left pars opercularis of inferior frontal gyrus	-0.068	0.065	[-0.196 - 0.060]	2.955E-01	4.961E-01
Right pars opercularis of inferior frontal gyrus	-0.092	0.060	[-0.210 - 0.026]	1.255E-01	3.566E-01
Left superior temporal gyrus	-0.033	0.053	[-0.137 - 0.071]	5.302E-01	6.722E-01
Right superior temporal gyrus	-0.073	0.068	[-0.206 - 0.060]	2.812E-01	4.961E-01
Left insula	-0.056	0.064	[-0.182 - 0.070]	3.856E-01	5.369E-01
Right insula	-0.067	0.064	[-0.193 - 0.059]	2.949E-01	4.961E-01
Left lingual gyrus	-0.036	0.059	[-0.151 - 0.079]	5.414E-01	6.744E-01
Right lingual gyrus	-0.016	0.075	[-0.164 - 0.132]	8.308E-01	8.503E-01
Left pars triangularis of inferior frontal gyrus	-0.038	0.060	[-0.156 - 0.080]	5.276E-01	6.722E-01
Right pars triangularis of inferior frontal gyrus	-0.085	0.054	[-0.190 - 0.020]	1.127E-01	3.334E-01
Left lateral orbitofrontal cortex	-0.127	0.052	[-0.228 - -0.026]	1.377E-02	1.172E-01
Right lateral orbitofrontal cortex	-0.039	0.090	[-0.215 - 0.137]	6.629E-01	7.845E-01
Left rostral middle frontal gyrus	-0.165	0.054	[-0.270 - -0.059]	2.188E-03	3.884E-02
Right rostral middle frontal gyrus	-0.085	0.064	[-0.210 - 0.040]	1.821E-01	4.309E-01
Left middle temporal gyrus	-0.078	0.053	[-0.182 - 0.026]	1.396E-01	3.814E-01
Right middle temporal gyrus	-0.061	0.059	[-0.177 - 0.055]	3.046E-01	4.961E-01
Left superior frontal gyrus	-0.176	0.052	[-0.277 - -0.074]	6.677E-04	2.370E-02
Right superior frontal gyrus	-0.159	0.052	[-0.260 - -0.057]	2.120E-03	3.884E-02
Left pars orbitalis of inferior frontal gyrus	-0.119	0.063	[-0.243 - 0.004]	5.745E-02	2.147E-01
Right pars orbitalis of inferior frontal gyrus	-0.063	0.068	[-0.197 - 0.070]	3.544E-01	5.243E-01
Left medial orbitofrontal cortex	-0.109	0.052	[-0.210 - -0.008]	3.520E-02	1.922E-01
Right medial orbitofrontal cortex	-0.100	0.052	[-0.202 - 0.003]	5.706E-02	2.147E-01
Left inferior temporal gyrus	-0.022	0.063	[-0.145 - 0.101]	7.252E-01	8.001E-01
Right inferior temporal gyrus	-0.024	0.052	[-0.125 - 0.077]	6.435E-01	7.743E-01
Left isthmus cingulate cortex	-0.056	0.059	[-0.172 - 0.059]	3.394E-01	5.127E-01
Right isthmus cingulate cortex	-0.140	0.072	[-0.281 - 0.000]	5.028E-02	2.147E-01
Left banks of superior temporal sulcus	-0.033	0.052	[-0.135 - 0.069]	5.297E-01	6.722E-01
Right banks of superior temporal sulcus	-0.059	0.052	[-0.160 - 0.042]	2.511E-01	4.818E-01
Left supramarginal gyrus	-0.079	0.063	[-0.202 - 0.045]	2.129E-01	4.581E-01
Right supramarginal gyrus	-0.073	0.052	[-0.174 - 0.028]	1.579E-01	4.153E-01
Left caudal middle frontal gyrus	-0.139	0.052	[-0.240 - -0.038]	7.206E-03	8.945E-02
Right caudal middle frontal gyrus	-0.163	0.089	[-0.338 - 0.012]	6.824E-02	2.422E-01
Left frontal pole	-0.070	0.052	[-0.172 - 0.031]	1.727E-01	4.229E-01
Right frontal pole	-0.180	0.052	[-0.281 - -0.079]	4.967E-04	2.370E-02
Left posterior cingulate cortex	-0.079	0.091	[-0.258 - 0.099]	3.830E-01	5.369E-01
Right posterior cingulate cortex	-0.100	0.052	[-0.201 - 0.001]	5.230E-02	2.147E-01
Left lateral occipital cortex	-0.089	0.052	[-0.191 - 0.012]	8.361E-02	2.607E-01
Right lateral occipital cortex	-0.131	0.068	[-0.265 - 0.003]	5.622E-02	2.147E-01
Left precentral gyrus	-0.159	0.060	[-0.276 - -0.042]	7.559E-03	8.945E-02
Right precentral gyrus	-0.089	0.085	[-0.257 - 0.078]	2.963E-01	4.961E-01
Left parahippocampal gyrus	-0.037	0.067	[-0.169 - 0.096]	5.872E-01	7.188E-01
Right parahippocampal gyrus	0.024	0.061	[-0.096 - 0.144]	6.955E-01	7.965E-01
Left inferior parietal cortex	-0.045	0.058	[-0.158 - 0.069]	4.410E-01	5.908E-01
Right inferior parietal cortex	-0.020	0.065	[-0.148 - 0.107]	7.519E-01	8.001E-01
Left transverse temporal gyrus	-0.063	0.055	[-0.170 - 0.044]	2.478E-01	4.818E-01
Right transverse temporal gyrus	0.008	0.052	[-0.093 - 0.109]	8.766E-01	8.766E-01
Left postcentral gyrus	0.021	0.052	[-0.080 - 0.122]	6.870E-01	7.965E-01
Right postcentral gyrus	-0.067	0.052	[-0.168 - 0.035]	1.978E-01	4.497E-01
Left precuneus	-0.116	0.066	[-0.245 - 0.012]	7.671E-02	2.593E-01
Right precuneus	-0.091	0.074	[-0.236 - 0.055]	2.214E-01	4.622E-01
Left caudal anterior cingulate cortex	-0.071	0.052	[-0.172 - 0.030]	1.686E-01	4.229E-01
Right caudal anterior cingulate cortex	-0.052	0.052	[-0.153 - 0.049]	3.163E-01	4.991E-01
Left cuneus	-0.086	0.083	[-0.248 - 0.076]	2.988E-01	4.961E-01
Right cuneus	-0.026	0.081	[-0.185 - 0.133]	7.521E-01	8.001E-01
Left rostral anterior cingulate cortex	0.016	0.052	[-0.085 - 0.118]	7.523E-01	8.001E-01
Right rostral anterior cingulate cortex	0.059	0.066	[-0.070 - 0.188]	3.689E-01	5.345E-01
Left pericalcarine cortex	-0.063	0.079	[-0.217 - 0.091]	4.242E-01	5.793E-01
Right pericalcarine cortex	-0.017	0.083	[-0.180 - 0.146]	8.383E-01	8.503E-01
Left paracentral lobule	-0.058	0.059	[-0.174 - 0.059]	3.309E-01	5.108E-01
Right paracentral lobule	-0.066	0.052	[-0.167 - 0.035]	2.027E-01	4.497E-01
Left superior parietal cortex	-0.122	0.052	[-0.223 - -0.021]	1.816E-02	1.172E-01
Right superior parietal cortex	-0.101	0.052	[-0.202 - 0.001]	5.153E-02	2.147E-01
Left temporal pole	-0.023	0.072	[-0.164 - 0.119]	7.550E-01	8.001E-01
Right temporal pole	0.014	0.056	[-0.095 - 0.124]	7.971E-01	8.323E-01
Left entorhinal cortex	0.053	0.052	[-0.049 - 0.154]	3.074E-01	4.961E-01
Right entorhinal cortex	0.089	0.052	[-0.012 - 0.190]	8.446E-02	2.607E-01

Supplementary Table S196. Partial correlations between cortical thickness and severity of symptoms measured by the BDI-II controlling for age and sex in individuals with major depressive disorder

	Partial *R*	Std. Err.	95% CI	*p*-value	FDR *q*-value
Global mean cortical thickness	-0.064	0.071	[-0.203 - 0.076]	3.722E-01	8.038E-01
Left hemisphere	-0.086	0.061	[-0.205 - 0.034]	1.601E-01	8.038E-01
Right hemisphere	-0.032	0.082	[-0.194 - 0.129]	6.958E-01	8.944E-01
Left fusiform gyrus	-0.093	0.058	[-0.207 - 0.021]	1.096E-01	7.880E-01
Right fusiform gyrus	-0.018	0.058	[-0.133 - 0.096]	7.520E-01	9.039E-01
Left pars opercularis of inferior frontal gyrus	-0.113	0.058	[-0.227 - 0.001]	5.228E-02	7.424E-01
Right pars opercularis of inferior frontal gyrus	-0.037	0.058	[-0.152 - 0.077]	5.206E-01	8.038E-01
Left superior temporal gyrus	-0.118	0.058	[-0.232 - -0.004]	4.240E-02	7.424E-01
Right superior temporal gyrus	-0.101	0.058	[-0.215 - 0.014]	8.401E-02	7.880E-01
Left insula	0.018	0.071	[-0.121 - 0.157]	8.002E-01	9.307E-01
Right insula	-0.017	0.058	[-0.132 - 0.097]	7.638E-01	9.039E-01
Left lingual gyrus	-0.058	0.071	[-0.198 - 0.082]	4.158E-01	8.038E-01
Right lingual gyrus	-0.004	0.071	[-0.142 - 0.135]	9.571E-01	9.705E-01
Left pars triangularis of inferior frontal gyrus	-0.004	0.058	[-0.118 - 0.110]	9.432E-01	9.705E-01
Right pars triangularis of inferior frontal gyrus	-0.108	0.064	[-0.233 - 0.017]	9.069E-02	7.880E-01
Left lateral orbitofrontal cortex	-0.100	0.066	[-0.229 - 0.029]	1.285E-01	7.916E-01
Right lateral orbitofrontal cortex	0.080	0.068	[-0.053 - 0.213]	2.367E-01	8.038E-01
Left rostral middle frontal gyrus	-0.063	0.086	[-0.231 - 0.106]	4.677E-01	8.038E-01
Right rostral middle frontal gyrus	-0.062	0.082	[-0.222 - 0.098]	4.490E-01	8.038E-01
Left middle temporal gyrus	-0.039	0.058	[-0.153 - 0.075]	5.062E-01	8.038E-01
Right middle temporal gyrus	-0.070	0.063	[-0.194 - 0.053]	2.645E-01	8.038E-01
Left superior frontal gyrus	-0.059	0.092	[-0.239 - 0.121]	5.208E-01	8.038E-01
Right superior frontal gyrus	-0.064	0.109	[-0.277 - 0.150]	5.591E-01	8.144E-01
Left pars orbitalis of inferior frontal gyrus	-0.093	0.058	[-0.207 - 0.021]	1.110E-01	7.880E-01
Right pars orbitalis of inferior frontal gyrus	0.059	0.079	[-0.095 - 0.213]	4.506E-01	8.038E-01
Left medial orbitofrontal cortex	-0.062	0.058	[-0.176 - 0.052]	2.889E-01	8.038E-01
Right medial orbitofrontal cortex	-0.048	0.058	[-0.162 - 0.066]	4.118E-01	8.038E-01
Left inferior temporal gyrus	-0.072	0.058	[-0.186 - 0.042]	2.154E-01	8.038E-01
Right inferior temporal gyrus	0.070	0.075	[-0.076 - 0.217]	3.480E-01	8.038E-01
Left isthmus cingulate cortex	-0.120	0.059	[-0.235 - -0.005]	4.146E-02	7.424E-01
Right isthmus cingulate cortex	-0.061	0.058	[-0.175 - 0.053]	2.927E-01	8.038E-01
Left banks of superior temporal sulcus	-0.021	0.058	[-0.135 - 0.093]	7.151E-01	8.944E-01
Right banks of superior temporal sulcus	-0.121	0.058	[-0.235 - -0.007]	3.827E-02	7.424E-01
Left supramarginal gyrus	-0.035	0.060	[-0.152 - 0.083]	5.621E-01	8.144E-01
Right supramarginal gyrus	-0.005	0.069	[-0.140 - 0.129]	9.372E-01	9.705E-01
Left caudal middle frontal gyrus	-0.075	0.066	[-0.205 - 0.054]	2.555E-01	8.038E-01
Right caudal middle frontal gyrus	-0.070	0.100	[-0.266 - 0.126]	4.862E-01	8.038E-01
Left frontal pole	-0.067	0.058	[-0.182 - 0.047]	2.466E-01	8.038E-01
Right frontal pole	-0.017	0.070	[-0.154 - 0.121]	8.127E-01	9.307E-01
Left posterior cingulate cortex	-0.018	0.101	[-0.216 - 0.181]	8.619E-01	9.705E-01
Right posterior cingulate cortex	-0.036	0.058	[-0.150 - 0.078]	5.321E-01	8.038E-01
Left lateral occipital cortex	-0.087	0.058	[-0.201 - 0.028]	1.372E-01	7.916E-01
Right lateral occipital cortex	-0.046	0.062	[-0.167 - 0.076]	4.591E-01	8.038E-01
Left precentral gyrus	-0.060	0.062	[-0.180 - 0.061]	3.333E-01	8.038E-01
Right precentral gyrus	0.073	0.092	[-0.107 - 0.254]	4.258E-01	8.038E-01
Left parahippocampal gyrus	-0.120	0.058	[-0.234 - -0.005]	4.000E-02	7.424E-01
Right parahippocampal gyrus	-0.044	0.058	[-0.158 - 0.070]	4.523E-01	8.038E-01
Left inferior parietal cortex	-0.030	0.060	[-0.147 - 0.087]	6.154E-01	8.402E-01
Right inferior parietal cortex	-0.062	0.058	[-0.176 - 0.052]	2.868E-01	8.038E-01
Left transverse temporal gyrus	-0.041	0.058	[-0.155 - 0.073]	4.853E-01	8.038E-01
Right transverse temporal gyrus	-0.035	0.064	[-0.161 - 0.091]	5.876E-01	8.258E-01
Left postcentral gyrus	0.021	0.062	[-0.100 - 0.142]	7.334E-01	8.977E-01
Right postcentral gyrus	-0.025	0.063	[-0.149 - 0.098]	6.858E-01	8.944E-01
Left precuneus	-0.068	0.064	[-0.193 - 0.057]	2.850E-01	8.038E-01
Right precuneus	-0.030	0.082	[-0.191 - 0.132]	7.180E-01	8.944E-01
Left caudal anterior cingulate cortex	-0.085	0.058	[-0.199 - 0.029]	1.449E-01	7.916E-01
Right caudal anterior cingulate cortex	-0.050	0.058	[-0.164 - 0.064]	3.880E-01	8.038E-01
Left cuneus	0.093	0.074	[-0.052 - 0.238]	2.095E-01	8.038E-01
Right cuneus	0.005	0.086	[-0.164 - 0.175]	9.502E-01	9.705E-01
Left rostral anterior cingulate cortex	-0.037	0.058	[-0.151 - 0.077]	5.248E-01	8.038E-01
Right rostral anterior cingulate cortex	0.047	0.075	[-0.100 - 0.195]	5.307E-01	8.038E-01
Left pericalcarine cortex	0.059	0.058	[-0.055 - 0.173]	3.110E-01	8.038E-01
Right pericalcarine cortex	-0.003	0.062	[-0.123 - 0.118]	9.660E-01	9.705E-01
Left paracentral lobule	-0.034	0.064	[-0.159 - 0.091]	5.932E-01	8.258E-01
Right paracentral lobule	0.006	0.072	[-0.135 - 0.146]	9.371E-01	9.705E-01
Left superior parietal cortex	-0.024	0.058	[-0.138 - 0.090]	6.794E-01	8.944E-01
Right superior parietal cortex	0.002	0.058	[-0.112 - 0.116]	9.705E-01	9.705E-01
Left temporal pole	-0.103	0.058	[-0.217 - 0.011]	7.636E-02	7.880E-01
Right temporal pole	0.004	0.058	[-0.110 - 0.118]	9.434E-01	9.705E-01
Left entorhinal cortex	0.046	0.061	[-0.073 - 0.166]	4.458E-01	8.038E-01
Right entorhinal cortex	0.070	0.061	[-0.049 - 0.188]	2.520E-01	8.038E-01

Supplementary Table S197. Partial correlations between cortical thickness and severity of symptoms measured by the BDI-II controlling for age and sex in individuals with major depressive disorder over 21 years of old

	Partial *R*	Std. Err.	95% CI	*p*-value	FDR *q*-value
Global mean cortical thickness	-0.058	0.072	[-0.198 - 0.083]	4.220E-01	8.028E-01
Left hemisphere	-0.080	0.063	[-0.203 - 0.043]	2.015E-01	8.028E-01
Right hemisphere	-0.027	0.082	[-0.188 - 0.135]	7.473E-01	9.324E-01
Left fusiform gyrus	-0.092	0.058	[-0.207 - 0.022]	1.138E-01	8.028E-01
Right fusiform gyrus	-0.014	0.058	[-0.129 - 0.100]	8.075E-01	9.563E-01
Left pars opercularis of inferior frontal gyrus	-0.095	0.058	[-0.210 - 0.019]	1.028E-01	8.028E-01
Right pars opercularis of inferior frontal gyrus	-0.050	0.058	[-0.165 - 0.064]	3.889E-01	8.028E-01
Left superior temporal gyrus	-0.097	0.058	[-0.211 - 0.018]	9.740E-02	8.028E-01
Right superior temporal gyrus	-0.089	0.058	[-0.204 - 0.025]	1.260E-01	8.028E-01
Left insula	0.025	0.073	[-0.118 - 0.168]	7.346E-01	9.324E-01
Right insula	-0.011	0.058	[-0.125 - 0.104]	8.559E-01	9.605E-01
Left lingual gyrus	-0.063	0.068	[-0.196 - 0.070]	3.533E-01	8.028E-01
Right lingual gyrus	-0.014	0.066	[-0.144 - 0.116]	8.348E-01	9.604E-01
Left pars triangularis of inferior frontal gyrus	-0.002	0.058	[-0.117 - 0.112]	9.678E-01	9.946E-01
Right pars triangularis of inferior frontal gyrus	-0.111	0.063	[-0.234 - 0.012]	7.596E-02	8.028E-01
Left lateral orbitofrontal cortex	-0.090	0.066	[-0.219 - 0.039]	1.703E-01	8.028E-01
Right lateral orbitofrontal cortex	0.085	0.068	[-0.048 - 0.217]	2.114E-01	8.028E-01
Left rostral middle frontal gyrus	-0.072	0.088	[-0.244 - 0.100]	4.124E-01	8.028E-01
Right rostral middle frontal gyrus	-0.067	0.084	[-0.232 - 0.098]	4.251E-01	8.028E-01
Left middle temporal gyrus	-0.043	0.058	[-0.158 - 0.071]	4.603E-01	8.170E-01
Right middle temporal gyrus	-0.071	0.066	[-0.200 - 0.058]	2.797E-01	8.028E-01
Left superior frontal gyrus	-0.066	0.095	[-0.253 - 0.120]	4.841E-01	8.256E-01
Right superior frontal gyrus	-0.072	0.113	[-0.293 - 0.148]	5.202E-01	8.394E-01
Left pars orbitalis of inferior frontal gyrus	-0.093	0.058	[-0.208 - 0.021]	1.107E-01	8.028E-01
Right pars orbitalis of inferior frontal gyrus	0.065	0.078	[-0.088 - 0.217]	4.073E-01	8.028E-01
Left medial orbitofrontal cortex	-0.061	0.058	[-0.175 - 0.054]	2.973E-01	8.028E-01
Right medial orbitofrontal cortex	-0.052	0.058	[-0.166 - 0.063]	3.773E-01	8.028E-01
Left inferior temporal gyrus	-0.076	0.058	[-0.190 - 0.039]	1.954E-01	8.028E-01
Right inferior temporal gyrus	0.067	0.080	[-0.089 - 0.224]	4.008E-01	8.028E-01
Left isthmus cingulate cortex	-0.140	0.058	[-0.255 - -0.026]	1.650E-02	8.028E-01
Right isthmus cingulate cortex	-0.059	0.058	[-0.173 - 0.056]	3.163E-01	8.028E-01
Left banks of superior temporal sulcus	-0.012	0.058	[-0.126 - 0.103]	8.387E-01	9.604E-01
Right banks of superior temporal sulcus	-0.120	0.058	[-0.234 - -0.005]	4.023E-02	8.028E-01
Left supramarginal gyrus	-0.033	0.059	[-0.149 - 0.084]	5.847E-01	8.608E-01
Right supramarginal gyrus	0.009	0.062	[-0.113 - 0.132]	8.794E-01	9.605E-01
Left caudal middle frontal gyrus	-0.078	0.067	[-0.209 - 0.053]	2.436E-01	8.028E-01
Right caudal middle frontal gyrus	-0.054	0.102	[-0.254 - 0.145]	5.940E-01	8.608E-01
Left frontal pole	-0.065	0.058	[-0.179 - 0.050]	2.690E-01	8.028E-01
Right frontal pole	-0.026	0.071	[-0.164 - 0.113]	7.154E-01	9.324E-01
Left posterior cingulate cortex	-0.005	0.099	[-0.198 - 0.189]	9.635E-01	9.946E-01
Right posterior cingulate cortex	-0.020	0.058	[-0.134 - 0.095]	7.365E-01	9.324E-01
Left lateral occipital cortex	-0.082	0.058	[-0.196 - 0.033]	1.609E-01	8.028E-01
Right lateral occipital cortex	-0.043	0.062	[-0.166 - 0.079]	4.884E-01	8.256E-01
Left precentral gyrus	-0.056	0.065	[-0.183 - 0.071]	3.873E-01	8.028E-01
Right precentral gyrus	0.071	0.092	[-0.109 - 0.251]	4.410E-01	8.028E-01
Left parahippocampal gyrus	-0.115	0.058	[-0.229 - 0.000]	4.975E-02	8.028E-01
Right parahippocampal gyrus	-0.034	0.058	[-0.149 - 0.080]	5.579E-01	8.608E-01
Left inferior parietal cortex	-0.016	0.058	[-0.131 - 0.098]	7.791E-01	9.537E-01
Right inferior parietal cortex	-0.049	0.058	[-0.164 - 0.065]	3.991E-01	8.028E-01
Left transverse temporal gyrus	-0.033	0.058	[-0.148 - 0.081]	5.707E-01	8.608E-01
Right transverse temporal gyrus	-0.035	0.065	[-0.162 - 0.092]	5.891E-01	8.608E-01
Left postcentral gyrus	0.024	0.066	[-0.106 - 0.155]	7.143E-01	9.324E-01
Right postcentral gyrus	-0.014	0.058	[-0.129 - 0.100]	8.081E-01	9.563E-01
Left precuneus	-0.053	0.068	[-0.187 - 0.081]	4.390E-01	8.028E-01
Right precuneus	-0.027	0.084	[-0.191 - 0.137]	7.455E-01	9.324E-01
Left caudal anterior cingulate cortex	-0.079	0.058	[-0.193 - 0.036]	1.771E-01	8.028E-01
Right caudal anterior cingulate cortex	-0.046	0.058	[-0.160 - 0.069]	4.360E-01	8.028E-01
Left cuneus	0.086	0.072	[-0.055 - 0.228]	2.323E-01	8.028E-01
Right cuneus	-0.012	0.077	[-0.162 - 0.139]	8.766E-01	9.605E-01
Left rostral anterior cingulate cortex	-0.039	0.058	[-0.153 - 0.076]	5.062E-01	8.359E-01
Right rostral anterior cingulate cortex	0.058	0.065	[-0.069 - 0.186]	3.689E-01	8.028E-01
Left pericalcarine cortex	0.069	0.058	[-0.046 - 0.183]	2.406E-01	8.028E-01
Right pericalcarine cortex	0.002	0.065	[-0.125 - 0.128]	9.805E-01	9.946E-01
Left paracentral lobule	-0.025	0.063	[-0.148 - 0.098]	6.894E-01	9.324E-01
Right paracentral lobule	0.007	0.073	[-0.135 - 0.149]	9.231E-01	9.782E-01
Left superior parietal cortex	-0.019	0.058	[-0.133 - 0.096]	7.485E-01	9.324E-01
Right superior parietal cortex	0.006	0.058	[-0.108 - 0.121]	9.126E-01	9.782E-01
Left temporal pole	-0.093	0.058	[-0.208 - 0.021]	1.106E-01	8.028E-01
Right temporal pole	0.000	0.058	[-0.114 - 0.115]	9.996E-01	9.996E-01
Left entorhinal cortex	0.055	0.058	[-0.060 - 0.169]	3.505E-01	8.028E-01
Right entorhinal cortex	0.081	0.058	[-0.034 - 0.195]	1.676E-01	8.028E-01

Supplementary Table S198. Partial correlations between cortical surface area and PANSS total controlling for age and sex in individuals with schizophrenia

	Partial *R*	Std. Err.	95% CI	*p*-value	FDR *q*-value
Total cortical surface area	0.013	0.053	[-0.090 - 0.116]	8.014E-01	9.834E-01
Left hemisphere	0.006	0.055	[-0.102 - 0.114]	9.151E-01	9.834E-01
Right hemisphere	0.024	0.048	[-0.070 - 0.118]	6.118E-01	9.834E-01
Left superior frontal gyrus	-0.001	0.040	[-0.080 - 0.078]	9.813E-01	9.834E-01
Right superior frontal gyrus	0.037	0.051	[-0.063 - 0.137]	4.654E-01	9.834E-01
Left pars orbitalis of inferior frontal gyrus	0.017	0.049	[-0.079 - 0.113]	7.294E-01	9.834E-01
Right pars orbitalis of inferior frontal gyrus	-0.021	0.040	[-0.100 - 0.058]	5.951E-01	9.834E-01
Left precentral gyrus	0.016	0.043	[-0.067 - 0.099]	7.081E-01	9.834E-01
Right precentral gyrus	0.024	0.060	[-0.094 - 0.142]	6.916E-01	9.834E-01
Left lingual gyrus	-0.011	0.064	[-0.136 - 0.114]	8.645E-01	9.834E-01
Right lingual gyrus	0.006	0.056	[-0.104 - 0.116]	9.133E-01	9.834E-01
Left superior temporal gyrus	-0.014	0.058	[-0.128 - 0.100]	8.132E-01	9.834E-01
Right superior temporal gyrus	0.043	0.053	[-0.061 - 0.147]	4.184E-01	9.834E-01
Left fusiform gyrus	-0.010	0.041	[-0.090 - 0.070]	8.019E-01	9.834E-01
Right fusiform gyrus	-0.056	0.045	[-0.144 - 0.032]	2.110E-01	9.834E-01
Left inferior parietal cortex	0.015	0.040	[-0.064 - 0.093]	7.179E-01	9.834E-01
Right inferior parietal cortex	-0.033	0.043	[-0.117 - 0.051]	4.381E-01	9.834E-01
Left lateral occipital cortex	-0.048	0.054	[-0.154 - 0.058]	3.763E-01	9.834E-01
Right lateral occipital cortex	0.004	0.049	[-0.091 - 0.099]	9.283E-01	9.834E-01
Left rostral middle frontal gyrus	0.025	0.043	[-0.060 - 0.110]	5.694E-01	9.834E-01
Right rostral middle frontal gyrus	0.059	0.042	[-0.024 - 0.142]	1.625E-01	9.834E-01
Left precuneus	0.018	0.040	[-0.060 - 0.097]	6.462E-01	9.834E-01
Right precuneus	0.004	0.052	[-0.099 - 0.106]	9.437E-01	9.834E-01
Left inferior temporal gyrus	0.042	0.040	[-0.037 - 0.121]	2.965E-01	9.834E-01
Right inferior temporal gyrus	0.068	0.040	[-0.010 - 0.147]	8.899E-02	9.834E-01
Left lateral orbitofrontal cortex	-0.029	0.060	[-0.147 - 0.089]	6.332E-01	9.834E-01
Right lateral orbitofrontal cortex	0.028	0.043	[-0.056 - 0.113]	5.080E-01	9.834E-01
Left middle temporal gyrus	0.025	0.046	[-0.066 - 0.115]	5.943E-01	9.834E-01
Right middle temporal gyrus	0.020	0.040	[-0.059 - 0.098]	6.267E-01	9.834E-01
Left postcentral gyrus	0.064	0.044	[-0.022 - 0.149]	1.439E-01	9.834E-01
Right postcentral gyrus	0.032	0.052	[-0.069 - 0.133]	5.350E-01	9.834E-01
Left medial orbitofrontal cortex	0.049	0.040	[-0.030 - 0.128]	2.257E-01	9.834E-01
Right medial orbitofrontal cortex	0.045	0.040	[-0.034 - 0.124]	2.623E-01	9.834E-01
Left cuneus	0.084	0.040	[0.005 - 0.163]	3.652E-02	9.834E-01
Right cuneus	0.024	0.057	[-0.088 - 0.136]	6.734E-01	9.834E-01
Left pars triangularis of inferior frontal gyrus	0.031	0.068	[-0.102 - 0.165]	6.454E-01	9.834E-01
Right pars triangularis of inferior frontal gyrus	-0.001	0.040	[-0.080 - 0.078]	9.831E-01	9.834E-01
Left superior parietal cortex	-0.073	0.073	[-0.216 - 0.071]	3.202E-01	9.834E-01
Right superior parietal cortex	-0.005	0.042	[-0.086 - 0.077]	9.118E-01	9.834E-01
Left pars opercularis of inferior frontal gyrus	-0.025	0.054	[-0.131 - 0.081]	6.401E-01	9.834E-01
Right pars opercularis of inferior frontal gyrus	0.002	0.052	[-0.099 - 0.103]	9.744E-01	9.834E-01
Left supramarginal gyrus	0.020	0.053	[-0.085 - 0.124]	7.118E-01	9.834E-01
Right supramarginal gyrus	0.036	0.052	[-0.066 - 0.138]	4.860E-01	9.834E-01
Left pericalcarine cortex	0.047	0.046	[-0.043 - 0.137]	3.071E-01	9.834E-01
Right pericalcarine cortex	0.022	0.040	[-0.056 - 0.101]	5.775E-01	9.834E-01
Left parahippocampal gyrus	-0.006	0.045	[-0.095 - 0.082]	8.863E-01	9.834E-01
Right parahippocampal gyrus	-0.057	0.040	[-0.136 - 0.022]	1.544E-01	9.834E-01
Left caudal middle frontal gyrus	0.044	0.044	[-0.043 - 0.131]	3.166E-01	9.834E-01
Right caudal middle frontal gyrus	0.020	0.040	[-0.059 - 0.099]	6.168E-01	9.834E-01
Left transverse temporal gyrus	-0.003	0.045	[-0.091 - 0.085]	9.493E-01	9.834E-01
Right transverse temporal gyrus	0.014	0.059	[-0.102 - 0.130]	8.126E-01	9.834E-01
Left banks of superior temporal sulcus	0.066	0.040	[-0.013 - 0.145]	9.949E-02	9.834E-01
Right banks of superior temporal sulcus	-0.031	0.040	[-0.110 - 0.048]	4.450E-01	9.834E-01
Left caudal anterior cingulate cortex	0.046	0.040	[-0.033 - 0.125]	2.571E-01	9.834E-01
Right caudal anterior cingulate cortex	-0.146	0.088	[-0.319 - 0.028]	9.941E-02	9.834E-01
Left rostral anterior cingulate cortex	-0.016	0.047	[-0.108 - 0.077]	7.355E-01	9.834E-01
Right rostral anterior cingulate cortex	0.012	0.051	[-0.089 - 0.113]	8.196E-01	9.834E-01
Left posterior cingulate cortex	0.063	0.046	[-0.027 - 0.152]	1.713E-01	9.834E-01
Right posterior cingulate cortex	0.002	0.042	[-0.081 - 0.085]	9.611E-01	9.834E-01
Left frontal pole	0.010	0.063	[-0.113 - 0.133]	8.751E-01	9.834E-01
Right frontal pole	-0.046	0.063	[-0.169 - 0.077]	4.636E-01	9.834E-01
Left paracentral lobule	0.010	0.052	[-0.092 - 0.111]	8.532E-01	9.834E-01
Right paracentral lobule	0.028	0.063	[-0.096 - 0.151]	6.607E-01	9.834E-01
Left insula	0.023	0.052	[-0.078 - 0.125]	6.517E-01	9.834E-01
Right insula	0.126	0.063	[0.003 - 0.249]	4.410E-02	9.834E-01
Left entorhinal cortex	-0.001	0.043	[-0.085 - 0.083]	9.834E-01	9.834E-01
Right entorhinal cortex	-0.045	0.049	[-0.140 - 0.050]	3.571E-01	9.834E-01
Left temporal pole	0.050	0.057	[-0.061 - 0.162]	3.788E-01	9.834E-01
Right temporal pole	-0.004	0.040	[-0.083 - 0.075]	9.218E-01	9.834E-01
Left isthmus cingulate cortex	0.006	0.051	[-0.095 - 0.106]	9.084E-01	9.834E-01
Right isthmus cingulate cortex	0.114	0.042	[0.032 - 0.195]	6.141E-03	4.360E-01

Supplementary Table S199. Partial correlations between cortical surface area and PANSS positive controlling for age and sex in individuals with schizophrenia

	Partial *R*	Std. Err.	95% CI	*p*-value	FDR *q*-value
Total cortical surface area	-0.024	0.060	[-0.141 - 0.093]	6.855E-01	9.566E-01
Left hemisphere	-0.027	0.062	[-0.149 - 0.094]	6.572E-01	9.566E-01
Right hemisphere	-0.015	0.054	[-0.120 - 0.091]	7.868E-01	9.566E-01
Left superior frontal gyrus	-0.026	0.040	[-0.105 - 0.053]	5.170E-01	9.566E-01
Right superior frontal gyrus	0.037	0.046	[-0.052 - 0.127]	4.169E-01	9.566E-01
Left pars orbitalis of inferior frontal gyrus	-0.011	0.043	[-0.096 - 0.073]	7.892E-01	9.566E-01
Right pars orbitalis of inferior frontal gyrus	-0.027	0.043	[-0.110 - 0.057]	5.301E-01	9.566E-01
Left precentral gyrus	-0.022	0.040	[-0.101 - 0.057]	5.910E-01	9.566E-01
Right precentral gyrus	-0.005	0.072	[-0.147 - 0.137]	9.431E-01	9.566E-01
Left lingual gyrus	-0.027	0.074	[-0.171 - 0.118]	7.182E-01	9.566E-01
Right lingual gyrus	-0.016	0.065	[-0.143 - 0.112]	8.104E-01	9.566E-01
Left superior temporal gyrus	-0.054	0.058	[-0.168 - 0.059]	3.484E-01	9.566E-01
Right superior temporal gyrus	0.008	0.048	[-0.086 - 0.102]	8.680E-01	9.566E-01
Left fusiform gyrus	0.003	0.040	[-0.076 - 0.082]	9.353E-01	9.566E-01
Right fusiform gyrus	-0.044	0.040	[-0.123 - 0.035]	2.727E-01	9.566E-01
Left inferior parietal cortex	0.007	0.045	[-0.080 - 0.095]	8.698E-01	9.566E-01
Right inferior parietal cortex	-0.046	0.045	[-0.133 - 0.041]	2.992E-01	9.566E-01
Left lateral occipital cortex	-0.049	0.066	[-0.178 - 0.080]	4.532E-01	9.566E-01
Right lateral occipital cortex	-0.032	0.054	[-0.137 - 0.074]	5.554E-01	9.566E-01
Left rostral middle frontal gyrus	-0.015	0.051	[-0.116 - 0.086]	7.681E-01	9.566E-01
Right rostral middle frontal gyrus	0.021	0.055	[-0.087 - 0.128]	7.051E-01	9.566E-01
Left precuneus	-0.009	0.040	[-0.088 - 0.070]	8.208E-01	9.566E-01
Right precuneus	-0.035	0.040	[-0.114 - 0.044]	3.828E-01	9.566E-01
Left inferior temporal gyrus	0.033	0.040	[-0.046 - 0.112]	4.110E-01	9.566E-01
Right inferior temporal gyrus	0.040	0.040	[-0.039 - 0.119]	3.171E-01	9.566E-01
Left lateral orbitofrontal cortex	-0.036	0.065	[-0.163 - 0.092]	5.855E-01	9.566E-01
Right lateral orbitofrontal cortex	0.020	0.057	[-0.091 - 0.131]	7.195E-01	9.566E-01
Left middle temporal gyrus	-0.029	0.084	[-0.193 - 0.136]	7.324E-01	9.566E-01
Right middle temporal gyrus	-0.020	0.040	[-0.098 - 0.059]	6.269E-01	9.566E-01
Left postcentral gyrus	0.052	0.040	[-0.027 - 0.131]	1.961E-01	9.566E-01
Right postcentral gyrus	-0.007	0.053	[-0.112 - 0.097]	8.926E-01	9.566E-01
Left medial orbitofrontal cortex	0.001	0.041	[-0.079 - 0.081]	9.790E-01	9.790E-01
Right medial orbitofrontal cortex	0.064	0.052	[-0.037 - 0.166]	2.134E-01	9.566E-01
Left cuneus	0.041	0.041	[-0.039 - 0.121]	3.135E-01	9.566E-01
Right cuneus	-0.099	0.092	[-0.280 - 0.082]	2.818E-01	9.566E-01
Left pars triangularis of inferior frontal gyrus	0.005	0.061	[-0.115 - 0.124]	9.382E-01	9.566E-01
Right pars triangularis of inferior frontal gyrus	-0.029	0.040	[-0.108 - 0.049]	4.638E-01	9.566E-01
Left superior parietal cortex	-0.087	0.063	[-0.210 - 0.036]	1.658E-01	9.566E-01
Right superior parietal cortex	-0.027	0.042	[-0.108 - 0.055]	5.205E-01	9.566E-01
Left pars opercularis of inferior frontal gyrus	-0.027	0.066	[-0.158 - 0.103]	6.795E-01	9.566E-01
Right pars opercularis of inferior frontal gyrus	-0.015	0.040	[-0.094 - 0.064]	7.065E-01	9.566E-01
Left supramarginal gyrus	-0.019	0.046	[-0.110 - 0.072]	6.900E-01	9.566E-01
Right supramarginal gyrus	0.010	0.056	[-0.101 - 0.121]	8.612E-01	9.566E-01
Left pericalcarine cortex	0.005	0.055	[-0.102 - 0.113]	9.238E-01	9.566E-01
Right pericalcarine cortex	-0.046	0.068	[-0.179 - 0.087]	4.977E-01	9.566E-01
Left parahippocampal gyrus	0.067	0.097	[-0.123 - 0.256]	4.908E-01	9.566E-01
Right parahippocampal gyrus	-0.071	0.040	[-0.150 - 0.008]	7.648E-02	9.566E-01
Left caudal middle frontal gyrus	0.028	0.046	[-0.061 - 0.118]	5.363E-01	9.566E-01
Right caudal middle frontal gyrus	-0.025	0.040	[-0.103 - 0.054]	5.414E-01	9.566E-01
Left transverse temporal gyrus	-0.017	0.058	[-0.130 - 0.097]	7.753E-01	9.566E-01
Right transverse temporal gyrus	-0.034	0.054	[-0.140 - 0.071]	5.275E-01	9.566E-01
Left banks of superior temporal sulcus	0.050	0.040	[-0.029 - 0.130]	2.141E-01	9.566E-01
Right banks of superior temporal sulcus	0.014	0.040	[-0.065 - 0.093]	7.233E-01	9.566E-01
Left caudal anterior cingulate cortex	0.053	0.044	[-0.034 - 0.140]	2.350E-01	9.566E-01
Right caudal anterior cingulate cortex	-0.015	0.044	[-0.102 - 0.072]	7.306E-01	9.566E-01
Left rostral anterior cingulate cortex	-0.006	0.052	[-0.109 - 0.096]	9.018E-01	9.566E-01
Right rostral anterior cingulate cortex	-0.015	0.044	[-0.101 - 0.070]	7.277E-01	9.566E-01
Left posterior cingulate cortex	0.033	0.046	[-0.058 - 0.124]	4.799E-01	9.566E-01
Right posterior cingulate cortex	0.020	0.040	[-0.059 - 0.098]	6.278E-01	9.566E-01
Left frontal pole	-0.026	0.053	[-0.130 - 0.079]	6.312E-01	9.566E-01
Right frontal pole	-0.043	0.066	[-0.171 - 0.086]	5.126E-01	9.566E-01
Left paracentral lobule	-0.019	0.044	[-0.105 - 0.067]	6.612E-01	9.566E-01
Right paracentral lobule	0.014	0.055	[-0.094 - 0.121]	8.029E-01	9.566E-01
Left insula	-0.010	0.044	[-0.096 - 0.076]	8.183E-01	9.566E-01
Right insula	0.036	0.052	[-0.065 - 0.138]	4.840E-01	9.566E-01
Left entorhinal cortex	0.032	0.049	[-0.065 - 0.128]	5.183E-01	9.566E-01
Right entorhinal cortex	-0.025	0.042	[-0.108 - 0.057]	5.473E-01	9.566E-01
Left temporal pole	0.085	0.053	[-0.019 - 0.189]	1.076E-01	9.566E-01
Right temporal pole	0.006	0.040	[-0.073 - 0.085]	8.836E-01	9.566E-01
Left isthmus cingulate cortex	-0.012	0.048	[-0.106 - 0.083]	8.040E-01	9.566E-01
Right isthmus cingulate cortex	0.070	0.050	[-0.027 - 0.167]	1.579E-01	9.566E-01

Supplementary Table S200. Partial correlations between cortical surface area and PANSS negative controlling for age and sex in individuals with schizophrenia

	Partial *R*	Std. Err.	95% CI	*p*-value	FDR *q*-value
Total cortical surface area	0.014	0.064	[-0.112 - 0.140]	8.263E-01	9.532E-01
Left hemisphere	0.004	0.066	[-0.126 - 0.134]	9.461E-01	9.735E-01
Right hemisphere	0.026	0.061	[-0.094 - 0.146]	6.682E-01	9.532E-01
Left superior frontal gyrus	-0.016	0.063	[-0.138 - 0.107]	8.016E-01	9.532E-01
Right superior frontal gyrus	0.016	0.051	[-0.084 - 0.117]	7.498E-01	9.532E-01
Left pars orbitalis of inferior frontal gyrus	0.015	0.057	[-0.097 - 0.128]	7.888E-01	9.532E-01
Right pars orbitalis of inferior frontal gyrus	-0.013	0.040	[-0.092 - 0.065]	7.382E-01	9.532E-01
Left precentral gyrus	0.029	0.041	[-0.051 - 0.108]	4.800E-01	9.532E-01
Right precentral gyrus	0.041	0.051	[-0.060 - 0.141]	4.266E-01	9.532E-01
Left lingual gyrus	-0.047	0.063	[-0.170 - 0.076]	4.542E-01	9.532E-01
Right lingual gyrus	-0.010	0.054	[-0.115 - 0.095]	8.495E-01	9.532E-01
Left superior temporal gyrus	-0.017	0.082	[-0.178 - 0.143]	8.319E-01	9.532E-01
Right superior temporal gyrus	0.032	0.067	[-0.099 - 0.162]	6.346E-01	9.532E-01
Left fusiform gyrus	-0.025	0.050	[-0.124 - 0.073]	6.148E-01	9.532E-01
Right fusiform gyrus	-0.070	0.063	[-0.193 - 0.052]	2.612E-01	9.532E-01
Left inferior parietal cortex	0.029	0.040	[-0.050 - 0.108]	4.687E-01	9.532E-01
Right inferior parietal cortex	-0.012	0.043	[-0.096 - 0.072]	7.766E-01	9.532E-01
Left lateral occipital cortex	-0.019	0.041	[-0.099 - 0.062]	6.517E-01	9.532E-01
Right lateral occipital cortex	0.009	0.040	[-0.070 - 0.087]	8.310E-01	9.532E-01
Left rostral middle frontal gyrus	0.027	0.045	[-0.062 - 0.116]	5.490E-01	9.532E-01
Right rostral middle frontal gyrus	0.053	0.047	[-0.040 - 0.145]	2.647E-01	9.532E-01
Left precuneus	0.002	0.054	[-0.104 - 0.109]	9.649E-01	9.786E-01
Right precuneus	0.025	0.070	[-0.112 - 0.163]	7.167E-01	9.532E-01
Left inferior temporal gyrus	0.066	0.040	[-0.013 - 0.145]	1.022E-01	9.532E-01
Right inferior temporal gyrus	0.093	0.042	[0.011 - 0.175]	2.550E-02	9.053E-01
Left lateral orbitofrontal cortex	-0.038	0.079	[-0.193 - 0.116]	6.275E-01	9.532E-01
Right lateral orbitofrontal cortex	0.017	0.068	[-0.117 - 0.150]	8.060E-01	9.532E-01
Left middle temporal gyrus	0.007	0.053	[-0.097 - 0.111]	8.995E-01	9.532E-01
Right middle temporal gyrus	0.052	0.055	[-0.056 - 0.161]	3.450E-01	9.532E-01
Left postcentral gyrus	0.059	0.044	[-0.029 - 0.146]	1.876E-01	9.532E-01
Right postcentral gyrus	0.034	0.057	[-0.077 - 0.146]	5.460E-01	9.532E-01
Left medial orbitofrontal cortex	0.055	0.047	[-0.037 - 0.146]	2.410E-01	9.532E-01
Right medial orbitofrontal cortex	0.044	0.059	[-0.070 - 0.159]	4.497E-01	9.532E-01
Left cuneus	0.083	0.040	[0.004 - 0.162]	4.012E-02	9.496E-01
Right cuneus	0.062	0.052	[-0.040 - 0.164]	2.361E-01	9.532E-01
Left pars triangularis of inferior frontal gyrus	0.026	0.069	[-0.110 - 0.162]	7.045E-01	9.532E-01
Right pars triangularis of inferior frontal gyrus	0.013	0.040	[-0.066 - 0.092]	7.429E-01	9.532E-01
Left superior parietal cortex	-0.050	0.077	[-0.201 - 0.101]	5.152E-01	9.532E-01
Right superior parietal cortex	0.014	0.055	[-0.093 - 0.121]	7.920E-01	9.532E-01
Left pars opercularis of inferior frontal gyrus	-0.024	0.057	[-0.136 - 0.088]	6.782E-01	9.532E-01
Right pars opercularis of inferior frontal gyrus	0.016	0.055	[-0.092 - 0.123]	7.738E-01	9.532E-01
Left supramarginal gyrus	0.023	0.063	[-0.101 - 0.147]	7.147E-01	9.532E-01
Right supramarginal gyrus	0.028	0.059	[-0.089 - 0.144]	6.426E-01	9.532E-01
Left pericalcarine cortex	0.026	0.050	[-0.071 - 0.124]	5.976E-01	9.532E-01
Right pericalcarine cortex	0.006	0.040	[-0.073 - 0.085]	8.805E-01	9.532E-01
Left parahippocampal gyrus	0.012	0.051	[-0.088 - 0.111]	8.164E-01	9.532E-01
Right parahippocampal gyrus	-0.026	0.040	[-0.105 - 0.052]	5.106E-01	9.532E-01
Left caudal middle frontal gyrus	0.032	0.057	[-0.079 - 0.144]	5.690E-01	9.532E-01
Right caudal middle frontal gyrus	0.054	0.041	[-0.027 - 0.134]	1.909E-01	9.532E-01
Left transverse temporal gyrus	-0.007	0.054	[-0.113 - 0.099]	8.982E-01	9.532E-01
Right transverse temporal gyrus	0.031	0.062	[-0.090 - 0.152]	6.168E-01	9.532E-01
Left banks of superior temporal sulcus	0.036	0.040	[-0.043 - 0.115]	3.688E-01	9.532E-01
Right banks of superior temporal sulcus	-0.070	0.069	[-0.205 - 0.066]	3.133E-01	9.532E-01
Left caudal anterior cingulate cortex	0.035	0.048	[-0.060 - 0.129]	4.724E-01	9.532E-01
Right caudal anterior cingulate cortex	-0.025	0.042	[-0.107 - 0.057]	5.472E-01	9.532E-01
Left rostral anterior cingulate cortex	-0.008	0.049	[-0.105 - 0.088]	8.650E-01	9.532E-01
Right rostral anterior cingulate cortex	-0.020	0.080	[-0.178 - 0.138]	8.056E-01	9.532E-01
Left posterior cingulate cortex	0.068	0.040	[-0.011 - 0.147]	9.153E-02	9.532E-01
Right posterior cingulate cortex	0.014	0.040	[-0.065 - 0.093]	7.307E-01	9.532E-01
Left frontal pole	-0.001	0.040	[-0.080 - 0.078]	9.792E-01	9.792E-01
Right frontal pole	-0.049	0.050	[-0.148 - 0.050]	3.282E-01	9.532E-01
Left paracentral lobule	0.006	0.054	[-0.100 - 0.111]	9.142E-01	9.545E-01
Right paracentral lobule	0.047	0.040	[-0.032 - 0.126]	2.463E-01	9.532E-01
Left insula	0.016	0.057	[-0.096 - 0.128]	7.803E-01	9.532E-01
Right insula	0.108	0.046	[0.018 - 0.199]	1.836E-02	9.053E-01
Left entorhinal cortex	0.011	0.047	[-0.080 - 0.103]	8.088E-01	9.532E-01
Right entorhinal cortex	-0.073	0.052	[-0.174 - 0.028]	1.592E-01	9.532E-01
Left temporal pole	0.041	0.055	[-0.067 - 0.150]	4.582E-01	9.532E-01
Right temporal pole	-0.008	0.040	[-0.087 - 0.071]	8.436E-01	9.532E-01
Left isthmus cingulate cortex	0.028	0.056	[-0.083 - 0.138]	6.245E-01	9.532E-01
Right isthmus cingulate cortex	0.108	0.056	[-0.002 - 0.219]	5.493E-02	9.532E-01

Supplementary Table S201. Partial correlations between cortical surface area and PANSS general controlling for age and sex in individuals with schizophrenia

	Partial *R*	Std. Err.	95% CI	*p*-value	FDR *q*-value
Total cortical surface area	0.013	0.043	[-0.071 - 0.097]	7.648E-01	9.525E-01
Left hemisphere	0.008	0.044	[-0.078 - 0.094]	8.504E-01	9.525E-01
Right hemisphere	0.020	0.040	[-0.059 - 0.099]	6.166E-01	9.525E-01
Left superior frontal gyrus	-0.006	0.040	[-0.085 - 0.073]	8.756E-01	9.525E-01
Right superior frontal gyrus	0.025	0.046	[-0.065 - 0.114]	5.907E-01	9.525E-01
Left pars orbitalis of inferior frontal gyrus	0.018	0.048	[-0.075 - 0.112]	7.029E-01	9.525E-01
Right pars orbitalis of inferior frontal gyrus	-0.022	0.040	[-0.101 - 0.057]	5.884E-01	9.525E-01
Left precentral gyrus	0.017	0.044	[-0.070 - 0.104]	7.050E-01	9.525E-01
Right precentral gyrus	0.011	0.053	[-0.093 - 0.115]	8.338E-01	9.525E-01
Left lingual gyrus	0.010	0.054	[-0.096 - 0.116]	8.558E-01	9.525E-01
Right lingual gyrus	0.004	0.059	[-0.112 - 0.119]	9.507E-01	9.525E-01
Left superior temporal gyrus	-0.007	0.040	[-0.086 - 0.072]	8.556E-01	9.525E-01
Right superior temporal gyrus	0.040	0.040	[-0.039 - 0.119]	3.199E-01	9.525E-01
Left fusiform gyrus	-0.024	0.044	[-0.110 - 0.062]	5.894E-01	9.525E-01
Right fusiform gyrus	-0.056	0.040	[-0.135 - 0.023]	1.665E-01	9.525E-01
Left inferior parietal cortex	-0.004	0.040	[-0.083 - 0.075]	9.137E-01	9.525E-01
Right inferior parietal cortex	-0.043	0.040	[-0.122 - 0.036]	2.823E-01	9.525E-01
Left lateral occipital cortex	-0.055	0.052	[-0.157 - 0.046]	2.832E-01	9.525E-01
Right lateral occipital cortex	0.014	0.054	[-0.092 - 0.119]	8.009E-01	9.525E-01
Left rostral middle frontal gyrus	0.022	0.047	[-0.071 - 0.114]	6.455E-01	9.525E-01
Right rostral middle frontal gyrus	0.056	0.040	[-0.023 - 0.135]	1.638E-01	9.525E-01
Left precuneus	0.017	0.040	[-0.062 - 0.096]	6.747E-01	9.525E-01
Right precuneus	-0.003	0.045	[-0.092 - 0.086]	9.525E-01	9.525E-01
Left inferior temporal gyrus	0.021	0.040	[-0.058 - 0.100]	6.080E-01	9.525E-01
Right inferior temporal gyrus	0.047	0.040	[-0.032 - 0.126]	2.423E-01	9.525E-01
Left lateral orbitofrontal cortex	-0.045	0.060	[-0.163 - 0.073]	4.585E-01	9.525E-01
Right lateral orbitofrontal cortex	0.022	0.040	[-0.057 - 0.101]	5.907E-01	9.525E-01
Left middle temporal gyrus	0.031	0.040	[-0.048 - 0.110]	4.421E-01	9.525E-01
Right middle temporal gyrus	0.003	0.040	[-0.076 - 0.082]	9.471E-01	9.525E-01
Left postcentral gyrus	0.041	0.047	[-0.051 - 0.133]	3.840E-01	9.525E-01
Right postcentral gyrus	0.028	0.044	[-0.058 - 0.114]	5.304E-01	9.525E-01
Left medial orbitofrontal cortex	0.048	0.040	[-0.030 - 0.127]	2.289E-01	9.525E-01
Right medial orbitofrontal cortex	0.021	0.040	[-0.058 - 0.100]	6.009E-01	9.525E-01
Left cuneus	0.089	0.040	[0.010 - 0.168]	2.724E-02	9.525E-01
Right cuneus	0.024	0.057	[-0.088 - 0.136]	6.748E-01	9.525E-01
Left pars triangularis of inferior frontal gyrus	0.046	0.060	[-0.071 - 0.164]	4.391E-01	9.525E-01
Right pars triangularis of inferior frontal gyrus	0.004	0.040	[-0.075 - 0.082]	9.295E-01	9.525E-01
Left superior parietal cortex	-0.084	0.073	[-0.228 - 0.059]	2.480E-01	9.525E-01
Right superior parietal cortex	-0.017	0.040	[-0.095 - 0.062]	6.813E-01	9.525E-01
Left pars opercularis of inferior frontal gyrus	-0.024	0.043	[-0.108 - 0.060]	5.715E-01	9.525E-01
Right pars opercularis of inferior frontal gyrus	-0.005	0.051	[-0.106 - 0.096]	9.200E-01	9.525E-01
Left supramarginal gyrus	0.021	0.040	[-0.058 - 0.099]	6.094E-01	9.525E-01
Right supramarginal gyrus	0.022	0.052	[-0.079 - 0.123]	6.713E-01	9.525E-01
Left pericalcarine cortex	0.060	0.040	[-0.019 - 0.138]	1.391E-01	9.525E-01
Right pericalcarine cortex	0.042	0.040	[-0.037 - 0.121]	3.013E-01	9.525E-01
Left parahippocampal gyrus	-0.017	0.040	[-0.096 - 0.062]	6.783E-01	9.525E-01
Right parahippocampal gyrus	-0.059	0.040	[-0.138 - 0.020]	1.432E-01	9.525E-01
Left caudal middle frontal gyrus	0.036	0.040	[-0.043 - 0.115]	3.717E-01	9.525E-01
Right caudal middle frontal gyrus	0.012	0.040	[-0.067 - 0.091]	7.733E-01	9.525E-01
Left transverse temporal gyrus	-0.004	0.040	[-0.083 - 0.075]	9.230E-01	9.525E-01
Right transverse temporal gyrus	0.009	0.054	[-0.097 - 0.114]	8.702E-01	9.525E-01
Left banks of superior temporal sulcus	0.069	0.040	[-0.010 - 0.148]	8.826E-02	9.525E-01
Right banks of superior temporal sulcus	-0.047	0.040	[-0.126 - 0.032]	2.400E-01	9.525E-01
Left caudal anterior cingulate cortex	0.029	0.040	[-0.050 - 0.108]	4.672E-01	9.525E-01
Right caudal anterior cingulate cortex	-0.092	0.066	[-0.222 - 0.038]	1.650E-01	9.525E-01
Left rostral anterior cingulate cortex	-0.031	0.046	[-0.122 - 0.060]	5.073E-01	9.525E-01
Right rostral anterior cingulate cortex	-0.007	0.061	[-0.126 - 0.113]	9.103E-01	9.525E-01
Left posterior cingulate cortex	0.051	0.058	[-0.062 - 0.165]	3.754E-01	9.525E-01
Right posterior cingulate cortex	-0.035	0.059	[-0.151 - 0.081]	5.498E-01	9.525E-01
Left frontal pole	0.049	0.085	[-0.117 - 0.216]	5.633E-01	9.525E-01
Right frontal pole	-0.029	0.061	[-0.149 - 0.090]	6.302E-01	9.525E-01
Left paracentral lobule	0.015	0.040	[-0.064 - 0.093]	7.170E-01	9.525E-01
Right paracentral lobule	0.038	0.059	[-0.077 - 0.154]	5.157E-01	9.525E-01
Left insula	0.023	0.048	[-0.072 - 0.118]	6.338E-01	9.525E-01
Right insula	0.177	0.091	[-0.002 - 0.356]	5.205E-02	9.525E-01
Left entorhinal cortex	-0.027	0.040	[-0.106 - 0.051]	4.960E-01	9.525E-01
Right entorhinal cortex	-0.020	0.040	[-0.099 - 0.059]	6.185E-01	9.525E-01
Left temporal pole	0.015	0.051	[-0.085 - 0.114]	7.753E-01	9.525E-01
Right temporal pole	-0.005	0.040	[-0.084 - 0.074]	8.976E-01	9.525E-01
Left isthmus cingulate cortex	-0.013	0.054	[-0.120 - 0.093]	8.043E-01	9.525E-01
Right isthmus cingulate cortex	0.097	0.041	[0.016 - 0.178]	1.907E-02	9.525E-01

Supplementary Table S202. Partial correlations between cortical surface area and severity of symptoms measured by the HDRS-17 controlling for age and sex in individuals with major depressive disorder

	Partial *R*	Std. Err.	95% CI	*p*-value	FDR *q*-value
Total cortical surface area	0.043	0.055	[-0.065 - 0.152]	4.340E-01	9.111E-01
Left hemisphere	0.046	0.056	[-0.064 - 0.157]	4.098E-01	9.111E-01
Right hemisphere	0.040	0.054	[-0.066 - 0.147]	4.576E-01	9.111E-01
Left superior frontal gyrus	0.104	0.051	[0.003 - 0.204]	4.264E-02	9.111E-01
Right superior frontal gyrus	0.045	0.051	[-0.055 - 0.145]	3.751E-01	9.111E-01
Left pars orbitalis of inferior frontal gyrus	-0.038	0.052	[-0.140 - 0.063]	4.613E-01	9.111E-01
Right pars orbitalis of inferior frontal gyrus	0.040	0.051	[-0.060 - 0.140]	4.365E-01	9.111E-01
Left precentral gyrus	0.076	0.051	[-0.024 - 0.176]	1.352E-01	9.111E-01
Right precentral gyrus	-0.029	0.058	[-0.143 - 0.086]	6.256E-01	9.346E-01
Left lingual gyrus	0.052	0.070	[-0.084 - 0.189]	4.525E-01	9.111E-01
Right lingual gyrus	-0.063	0.064	[-0.189 - 0.063]	3.277E-01	9.111E-01
Left superior temporal gyrus	-0.009	0.056	[-0.119 - 0.102]	8.766E-01	9.346E-01
Right superior temporal gyrus	0.033	0.055	[-0.075 - 0.140]	5.547E-01	9.346E-01
Left fusiform gyrus	0.050	0.051	[-0.050 - 0.151]	3.240E-01	9.111E-01
Right fusiform gyrus	0.065	0.064	[-0.060 - 0.191]	3.069E-01	9.111E-01
Left inferior parietal cortex	0.080	0.051	[-0.020 - 0.180]	1.160E-01	9.111E-01
Right inferior parietal cortex	-0.018	0.069	[-0.153 - 0.116]	7.894E-01	9.346E-01
Left lateral occipital cortex	-0.067	0.082	[-0.227 - 0.094]	4.164E-01	9.111E-01
Right lateral occipital cortex	0.006	0.051	[-0.094 - 0.107]	8.997E-01	9.346E-01
Left rostral middle frontal gyrus	0.100	0.079	[-0.054 - 0.255]	2.032E-01	9.111E-01
Right rostral middle frontal gyrus	0.058	0.051	[-0.042 - 0.158]	2.572E-01	9.111E-01
Left precuneus	0.052	0.076	[-0.098 - 0.202]	4.944E-01	9.111E-01
Right precuneus	0.065	0.060	[-0.053 - 0.182]	2.801E-01	9.111E-01
Left inferior temporal gyrus	0.081	0.051	[-0.019 - 0.181]	1.129E-01	9.111E-01
Right inferior temporal gyrus	0.058	0.052	[-0.045 - 0.160]	2.721E-01	9.111E-01
Left lateral orbitofrontal cortex	0.041	0.063	[-0.082 - 0.165]	5.116E-01	9.111E-01
Right lateral orbitofrontal cortex	0.042	0.052	[-0.060 - 0.145]	4.172E-01	9.111E-01
Left middle temporal gyrus	0.025	0.075	[-0.123 - 0.172]	7.438E-01	9.346E-01
Right middle temporal gyrus	0.045	0.057	[-0.067 - 0.158]	4.302E-01	9.111E-01
Left postcentral gyrus	0.044	0.051	[-0.056 - 0.145]	3.852E-01	9.111E-01
Right postcentral gyrus	0.033	0.051	[-0.067 - 0.134]	5.133E-01	9.111E-01
Left medial orbitofrontal cortex	0.022	0.052	[-0.079 - 0.123]	6.729E-01	9.346E-01
Right medial orbitofrontal cortex	0.056	0.082	[-0.104 - 0.217]	4.911E-01	9.111E-01
Left cuneus	-0.030	0.051	[-0.130 - 0.071]	5.634E-01	9.346E-01
Right cuneus	0.006	0.051	[-0.094 - 0.106]	9.082E-01	9.346E-01
Left pars triangularis of inferior frontal gyrus	0.034	0.062	[-0.087 - 0.155]	5.819E-01	9.346E-01
Right pars triangularis of inferior frontal gyrus	0.010	0.051	[-0.090 - 0.110]	8.453E-01	9.346E-01
Left superior parietal cortex	0.030	0.076	[-0.118 - 0.179]	6.876E-01	9.346E-01
Right superior parietal cortex	0.034	0.064	[-0.091 - 0.159]	5.930E-01	9.346E-01
Left pars opercularis of inferior frontal gyrus	-0.022	0.051	[-0.122 - 0.079]	6.738E-01	9.346E-01
Right pars opercularis of inferior frontal gyrus	0.048	0.086	[-0.121 - 0.217]	5.794E-01	9.346E-01
Left supramarginal gyrus	-0.016	0.051	[-0.116 - 0.084]	7.510E-01	9.346E-01
Right supramarginal gyrus	0.121	0.051	[0.021 - 0.221]	1.812E-02	9.111E-01
Left pericalcarine cortex	-0.046	0.053	[-0.150 - 0.058]	3.877E-01	9.111E-01
Right pericalcarine cortex	-0.099	0.056	[-0.210 - 0.011]	7.827E-02	9.111E-01
Left parahippocampal gyrus	0.068	0.051	[-0.032 - 0.168]	1.850E-01	9.111E-01
Right parahippocampal gyrus	0.066	0.058	[-0.047 - 0.179]	2.539E-01	9.111E-01
Left caudal middle frontal gyrus	0.035	0.051	[-0.065 - 0.135]	4.970E-01	9.111E-01
Right caudal middle frontal gyrus	0.075	0.053	[-0.029 - 0.179]	1.557E-01	9.111E-01
Left transverse temporal gyrus	-0.012	0.051	[-0.112 - 0.088]	8.169E-01	9.346E-01
Right transverse temporal gyrus	0.014	0.051	[-0.086 - 0.114]	7.825E-01	9.346E-01
Left banks of superior temporal sulcus	-0.024	0.066	[-0.153 - 0.106]	7.213E-01	9.346E-01
Right banks of superior temporal sulcus	0.013	0.051	[-0.087 - 0.113]	7.962E-01	9.346E-01
Left caudal anterior cingulate cortex	0.012	0.051	[-0.088 - 0.112]	8.158E-01	9.346E-01
Right caudal anterior cingulate cortex	0.008	0.057	[-0.104 - 0.121]	8.829E-01	9.346E-01
Left rostral anterior cingulate cortex	-0.046	0.055	[-0.154 - 0.062]	4.040E-01	9.111E-01
Right rostral anterior cingulate cortex	-0.114	0.075	[-0.262 - 0.033]	1.295E-01	9.111E-01
Left posterior cingulate cortex	0.018	0.056	[-0.092 - 0.127]	7.508E-01	9.346E-01
Right posterior cingulate cortex	0.038	0.051	[-0.062 - 0.138]	4.552E-01	9.111E-01
Left frontal pole	0.023	0.051	[-0.078 - 0.123]	6.585E-01	9.346E-01
Right frontal pole	0.086	0.084	[-0.078 - 0.250]	3.033E-01	9.111E-01
Left paracentral lobule	-0.031	0.062	[-0.153 - 0.091]	6.169E-01	9.346E-01
Right paracentral lobule	0.000	0.051	[-0.101 - 0.100]	9.943E-01	9.943E-01
Left insula	-0.030	0.077	[-0.182 - 0.122]	7.012E-01	9.346E-01
Right insula	0.008	0.055	[-0.099 - 0.115]	8.843E-01	9.346E-01
Left entorhinal cortex	-0.009	0.051	[-0.109 - 0.091]	8.556E-01	9.346E-01
Right entorhinal cortex	-0.109	0.051	[-0.209 - -0.009]	3.286E-02	9.111E-01
Left temporal pole	0.076	0.053	[-0.027 - 0.180]	1.492E-01	9.111E-01
Right temporal pole	-0.008	0.064	[-0.134 - 0.117]	8.952E-01	9.346E-01
Left isthmus cingulate cortex	0.071	0.075	[-0.075 - 0.217]	3.416E-01	9.111E-01
Right isthmus cingulate cortex	-0.004	0.054	[-0.110 - 0.102]	9.453E-01	9.588E-01

Supplementary Table S203. Partial correlations between cortical surface area and severity of symptoms measured by the HDRS-17 controlling for age and sex in individuals with major depressive disorder over 21 years of old

	Partial *R*	Std. Err.	95% CI	*p*-value	FDR *q*-value
Total cortical surface area	0.038	0.052	[-0.063 - 0.139]	4.604E-01	8.945E-01
Left hemisphere	0.041	0.052	[-0.060 - 0.142]	4.300E-01	8.945E-01
Right hemisphere	0.036	0.052	[-0.065 - 0.137]	4.889E-01	8.945E-01
Left superior frontal gyrus	0.102	0.052	[0.000 - 0.203]	4.905E-02	8.945E-01
Right superior frontal gyrus	0.040	0.052	[-0.061 - 0.141]	4.368E-01	8.945E-01
Left pars orbitalis of inferior frontal gyrus	-0.030	0.054	[-0.137 - 0.076]	5.770E-01	8.945E-01
Right pars orbitalis of inferior frontal gyrus	0.047	0.052	[-0.054 - 0.149]	3.586E-01	8.945E-01
Left precentral gyrus	0.080	0.052	[-0.021 - 0.181]	1.202E-01	8.945E-01
Right precentral gyrus	-0.023	0.052	[-0.124 - 0.078]	6.542E-01	8.945E-01
Left lingual gyrus	0.058	0.063	[-0.065 - 0.182]	3.562E-01	8.945E-01
Right lingual gyrus	-0.056	0.055	[-0.163 - 0.052]	3.107E-01	8.945E-01
Left superior temporal gyrus	-0.012	0.054	[-0.119 - 0.094]	8.219E-01	9.412E-01
Right superior temporal gyrus	0.032	0.052	[-0.069 - 0.134]	5.305E-01	8.945E-01
Left fusiform gyrus	0.040	0.052	[-0.061 - 0.141]	4.411E-01	8.945E-01
Right fusiform gyrus	0.049	0.057	[-0.063 - 0.162]	3.887E-01	8.945E-01
Left inferior parietal cortex	0.080	0.052	[-0.021 - 0.181]	1.213E-01	8.945E-01
Right inferior parietal cortex	-0.025	0.068	[-0.159 - 0.108]	7.091E-01	8.945E-01
Left lateral occipital cortex	-0.074	0.082	[-0.235 - 0.088]	3.704E-01	8.945E-01
Right lateral occipital cortex	0.010	0.052	[-0.091 - 0.111]	8.505E-01	9.435E-01
Left rostral middle frontal gyrus	0.086	0.076	[-0.063 - 0.236]	2.558E-01	8.945E-01
Right rostral middle frontal gyrus	0.053	0.052	[-0.049 - 0.154]	3.077E-01	8.945E-01
Left precuneus	0.041	0.076	[-0.107 - 0.190]	5.874E-01	8.945E-01
Right precuneus	0.069	0.060	[-0.048 - 0.186]	2.489E-01	8.945E-01
Left inferior temporal gyrus	0.084	0.052	[-0.017 - 0.185]	1.030E-01	8.945E-01
Right inferior temporal gyrus	0.055	0.054	[-0.050 - 0.160]	3.037E-01	8.945E-01
Left lateral orbitofrontal cortex	0.032	0.063	[-0.091 - 0.156]	6.078E-01	8.945E-01
Right lateral orbitofrontal cortex	0.041	0.052	[-0.060 - 0.143]	4.221E-01	8.945E-01
Left middle temporal gyrus	0.008	0.069	[-0.127 - 0.142]	9.107E-01	9.609E-01
Right middle temporal gyrus	0.049	0.058	[-0.064 - 0.163]	3.944E-01	8.945E-01
Left postcentral gyrus	0.046	0.052	[-0.055 - 0.147]	3.708E-01	8.945E-01
Right postcentral gyrus	0.034	0.052	[-0.067 - 0.135]	5.086E-01	8.945E-01
Left medial orbitofrontal cortex	0.020	0.052	[-0.082 - 0.121]	7.049E-01	8.945E-01
Right medial orbitofrontal cortex	0.068	0.085	[-0.099 - 0.235]	4.237E-01	8.945E-01
Left cuneus	-0.022	0.052	[-0.123 - 0.079]	6.667E-01	8.945E-01
Right cuneus	0.018	0.052	[-0.083 - 0.119]	7.271E-01	8.945E-01
Left pars triangularis of inferior frontal gyrus	0.041	0.063	[-0.082 - 0.164]	5.141E-01	8.945E-01
Right pars triangularis of inferior frontal gyrus	0.010	0.052	[-0.091 - 0.111]	8.499E-01	9.435E-01
Left superior parietal cortex	0.024	0.071	[-0.115 - 0.164]	7.307E-01	8.945E-01
Right superior parietal cortex	0.019	0.055	[-0.089 - 0.127]	7.285E-01	8.945E-01
Left pars opercularis of inferior frontal gyrus	-0.019	0.052	[-0.120 - 0.082]	7.097E-01	8.945E-01
Right pars opercularis of inferior frontal gyrus	0.047	0.087	[-0.123 - 0.218]	5.856E-01	8.945E-01
Left supramarginal gyrus	-0.028	0.055	[-0.135 - 0.080]	6.150E-01	8.945E-01
Right supramarginal gyrus	0.121	0.052	[0.019 - 0.222]	1.961E-02	6.960E-01
Left pericalcarine cortex	-0.045	0.052	[-0.146 - 0.056]	3.860E-01	8.945E-01
Right pericalcarine cortex	-0.099	0.052	[-0.200 - 0.002]	5.529E-02	8.945E-01
Left parahippocampal gyrus	0.050	0.052	[-0.051 - 0.151]	3.307E-01	8.945E-01
Right parahippocampal gyrus	0.055	0.053	[-0.049 - 0.159]	2.968E-01	8.945E-01
Left caudal middle frontal gyrus	0.023	0.052	[-0.079 - 0.124]	6.605E-01	8.945E-01
Right caudal middle frontal gyrus	0.067	0.052	[-0.034 - 0.168]	1.940E-01	8.945E-01
Left transverse temporal gyrus	-0.014	0.052	[-0.115 - 0.088]	7.931E-01	9.231E-01
Right transverse temporal gyrus	0.018	0.052	[-0.083 - 0.119]	7.253E-01	8.945E-01
Left banks of superior temporal sulcus	-0.020	0.063	[-0.143 - 0.104]	7.559E-01	9.050E-01
Right banks of superior temporal sulcus	0.021	0.052	[-0.081 - 0.122]	6.886E-01	8.945E-01
Left caudal anterior cingulate cortex	0.015	0.052	[-0.086 - 0.117]	7.648E-01	9.050E-01
Right caudal anterior cingulate cortex	0.005	0.056	[-0.105 - 0.116]	9.247E-01	9.609E-01
Left rostral anterior cingulate cortex	-0.047	0.052	[-0.148 - 0.054]	3.632E-01	8.945E-01
Right rostral anterior cingulate cortex	-0.111	0.077	[-0.261 - 0.040]	1.490E-01	8.945E-01
Left posterior cingulate cortex	0.023	0.057	[-0.089 - 0.135]	6.914E-01	8.945E-01
Right posterior cingulate cortex	0.033	0.052	[-0.068 - 0.134]	5.198E-01	8.945E-01
Left frontal pole	0.026	0.052	[-0.075 - 0.127]	6.142E-01	8.945E-01
Right frontal pole	0.099	0.085	[-0.068 - 0.266]	2.461E-01	8.945E-01
Left paracentral lobule	-0.026	0.065	[-0.154 - 0.102]	6.874E-01	8.945E-01
Right paracentral lobule	0.004	0.052	[-0.097 - 0.106]	9.338E-01	9.609E-01
Left insula	-0.038	0.078	[-0.191 - 0.115]	6.241E-01	8.945E-01
Right insula	-0.005	0.055	[-0.113 - 0.103]	9.287E-01	9.609E-01
Left entorhinal cortex	-0.002	0.052	[-0.103 - 0.099]	9.734E-01	9.873E-01
Right entorhinal cortex	-0.128	0.052	[-0.229 - -0.027]	1.315E-02	6.960E-01
Left temporal pole	0.080	0.052	[-0.021 - 0.181]	1.216E-01	8.945E-01
Right temporal pole	-0.006	0.062	[-0.128 - 0.117]	9.261E-01	9.609E-01
Left isthmus cingulate cortex	0.085	0.076	[-0.064 - 0.233]	2.637E-01	8.945E-01
Right isthmus cingulate cortex	0.001	0.052	[-0.101 - 0.102]	9.903E-01	9.903E-01

Supplementary Table S204. Partial correlations between cortical surface area and severity of symptoms measured by the BDI-II controlling for age and sex in individuals with major depressive disorder

	Partial *R*	Std. Err.	95% CI	*p*-value	FDR *q*-value
Total cortical surface area	0.019	0.069	[-0.117 - 0.154]	7.847E-01	9.275E-01
Left hemisphere	0.030	0.070	[-0.108 - 0.167]	6.711E-01	9.228E-01
Right hemisphere	0.009	0.067	[-0.123 - 0.141]	8.984E-01	9.380E-01
Left superior frontal gyrus	0.046	0.073	[-0.097 - 0.189]	5.302E-01	9.034E-01
Right superior frontal gyrus	0.094	0.058	[-0.020 - 0.208]	1.077E-01	9.034E-01
Left pars orbitalis of inferior frontal gyrus	-0.056	0.058	[-0.171 - 0.058]	3.327E-01	9.034E-01
Right pars orbitalis of inferior frontal gyrus	-0.055	0.058	[-0.169 - 0.059]	3.470E-01	9.034E-01
Left precentral gyrus	0.099	0.058	[-0.015 - 0.214]	8.749E-02	9.034E-01
Right precentral gyrus	-0.035	0.077	[-0.185 - 0.116]	6.523E-01	9.228E-01
Left lingual gyrus	-0.038	0.058	[-0.152 - 0.076]	5.136E-01	9.034E-01
Right lingual gyrus	-0.039	0.058	[-0.154 - 0.075]	4.976E-01	9.034E-01
Left superior temporal gyrus	0.039	0.066	[-0.091 - 0.169]	5.555E-01	9.034E-01
Right superior temporal gyrus	0.026	0.058	[-0.088 - 0.140]	6.568E-01	9.228E-01
Left fusiform gyrus	0.030	0.079	[-0.125 - 0.185]	7.040E-01	9.228E-01
Right fusiform gyrus	-0.080	0.058	[-0.194 - 0.034]	1.688E-01	9.034E-01
Left inferior parietal cortex	-0.013	0.058	[-0.127 - 0.101]	8.224E-01	9.275E-01
Right inferior parietal cortex	-0.046	0.058	[-0.160 - 0.068]	4.321E-01	9.034E-01
Left lateral occipital cortex	-0.016	0.092	[-0.196 - 0.164]	8.622E-01	9.275E-01
Right lateral occipital cortex	-0.015	0.058	[-0.129 - 0.099]	7.966E-01	9.275E-01
Left rostral middle frontal gyrus	0.081	0.069	[-0.054 - 0.216]	2.394E-01	9.034E-01
Right rostral middle frontal gyrus	0.041	0.063	[-0.083 - 0.165]	5.160E-01	9.034E-01
Left precuneus	0.036	0.058	[-0.078 - 0.150]	5.324E-01	9.034E-01
Right precuneus	0.014	0.076	[-0.136 - 0.163]	8.579E-01	9.275E-01
Left inferior temporal gyrus	-0.028	0.073	[-0.171 - 0.115]	7.019E-01	9.228E-01
Right inferior temporal gyrus	0.058	0.097	[-0.133 - 0.249]	5.518E-01	9.034E-01
Left lateral orbitofrontal cortex	0.052	0.080	[-0.105 - 0.210]	5.153E-01	9.034E-01
Right lateral orbitofrontal cortex	-0.031	0.058	[-0.145 - 0.083]	5.939E-01	9.034E-01
Left middle temporal gyrus	0.018	0.096	[-0.171 - 0.206]	8.554E-01	9.275E-01
Right middle temporal gyrus	0.036	0.060	[-0.082 - 0.154]	5.468E-01	9.034E-01
Left postcentral gyrus	0.043	0.061	[-0.077 - 0.163]	4.804E-01	9.034E-01
Right postcentral gyrus	0.034	0.061	[-0.086 - 0.155]	5.784E-01	9.034E-01
Left medial orbitofrontal cortex	0.047	0.058	[-0.067 - 0.161]	4.212E-01	9.034E-01
Right medial orbitofrontal cortex	0.044	0.058	[-0.070 - 0.158]	4.490E-01	9.034E-01
Left cuneus	-0.094	0.060	[-0.212 - 0.024]	1.199E-01	9.034E-01
Right cuneus	0.053	0.058	[-0.061 - 0.167]	3.592E-01	9.034E-01
Left pars triangularis of inferior frontal gyrus	0.033	0.063	[-0.091 - 0.156]	6.033E-01	9.034E-01
Right pars triangularis of inferior frontal gyrus	-0.044	0.076	[-0.193 - 0.105]	5.655E-01	9.034E-01
Left superior parietal cortex	-0.036	0.058	[-0.150 - 0.078]	5.373E-01	9.034E-01
Right superior parietal cortex	-0.035	0.092	[-0.215 - 0.145]	7.018E-01	9.228E-01
Left pars opercularis of inferior frontal gyrus	0.016	0.060	[-0.101 - 0.133]	7.866E-01	9.275E-01
Right pars opercularis of inferior frontal gyrus	-0.003	0.066	[-0.133 - 0.127]	9.610E-01	9.747E-01
Left supramarginal gyrus	0.052	0.058	[-0.062 - 0.166]	3.750E-01	9.034E-01
Right supramarginal gyrus	0.072	0.058	[-0.042 - 0.186]	2.160E-01	9.034E-01
Left pericalcarine cortex	-0.122	0.065	[-0.249 - 0.004]	5.868E-02	9.034E-01
Right pericalcarine cortex	-0.094	0.070	[-0.231 - 0.042]	1.754E-01	9.034E-01
Left parahippocampal gyrus	0.040	0.058	[-0.074 - 0.154]	4.924E-01	9.034E-01
Right parahippocampal gyrus	0.017	0.085	[-0.150 - 0.184]	8.410E-01	9.275E-01
Left caudal middle frontal gyrus	0.031	0.058	[-0.083 - 0.145]	5.933E-01	9.034E-01
Right caudal middle frontal gyrus	0.061	0.058	[-0.053 - 0.175]	2.931E-01	9.034E-01
Left transverse temporal gyrus	0.008	0.058	[-0.106 - 0.122]	8.955E-01	9.380E-01
Right transverse temporal gyrus	0.005	0.058	[-0.109 - 0.119]	9.311E-01	9.581E-01
Left banks of superior temporal sulcus	0.072	0.058	[-0.042 - 0.187]	2.136E-01	9.034E-01
Right banks of superior temporal sulcus	-0.072	0.058	[-0.187 - 0.042]	2.131E-01	9.034E-01
Left caudal anterior cingulate cortex	0.061	0.058	[-0.053 - 0.175]	2.972E-01	9.034E-01
Right caudal anterior cingulate cortex	0.044	0.087	[-0.126 - 0.214]	6.087E-01	9.034E-01
Left rostral anterior cingulate cortex	0.049	0.058	[-0.065 - 0.163]	3.972E-01	9.034E-01
Right rostral anterior cingulate cortex	0.024	0.103	[-0.178 - 0.225]	8.173E-01	9.275E-01
Left posterior cingulate cortex	0.029	0.081	[-0.129 - 0.187]	7.186E-01	9.228E-01
Right posterior cingulate cortex	0.028	0.081	[-0.131 - 0.187]	7.278E-01	9.228E-01
Left frontal pole	0.097	0.088	[-0.076 - 0.269]	2.730E-01	9.034E-01
Right frontal pole	0.065	0.103	[-0.137 - 0.267]	5.298E-01	9.034E-01
Left paracentral lobule	0.000	0.080	[-0.157 - 0.156]	9.964E-01	9.964E-01
Right paracentral lobule	0.061	0.068	[-0.073 - 0.194]	3.730E-01	9.034E-01
Left insula	0.014	0.076	[-0.136 - 0.164]	8.561E-01	9.275E-01
Right insula	0.041	0.080	[-0.116 - 0.198]	6.107E-01	9.034E-01
Left entorhinal cortex	-0.062	0.058	[-0.176 - 0.052]	2.846E-01	9.034E-01
Right entorhinal cortex	-0.093	0.059	[-0.208 - 0.022]	1.126E-01	9.034E-01
Left temporal pole	0.063	0.060	[-0.054 - 0.180]	2.917E-01	9.034E-01
Right temporal pole	0.034	0.058	[-0.080 - 0.148]	5.609E-01	9.034E-01
Left isthmus cingulate cortex	0.077	0.090	[-0.100 - 0.253]	3.949E-01	9.034E-01
Right isthmus cingulate cortex	-0.119	0.079	[-0.274 - 0.036]	1.336E-01	9.034E-01

Supplementary Table S205. Partial correlations between cortical surface area and severity of symptoms measured by the BDI-II controlling for age and sex in individuals with major depressive disorder over 21 years of old

	Partial *R*	Std. Err.	95% CI	*p*-value	FDR *q*-value
Total cortical surface area	0.033	0.083	[-0.129 - 0.196]	6.862E-01	9.263E-01
Left hemisphere	0.045	0.084	[-0.119 - 0.208]	5.937E-01	9.263E-01
Right hemisphere	0.023	0.081	[-0.137 - 0.182]	7.786E-01	9.447E-01
Left superior frontal gyrus	0.050	0.078	[-0.103 - 0.202]	5.235E-01	9.263E-01
Right superior frontal gyrus	0.095	0.061	[-0.024 - 0.213]	1.185E-01	9.263E-01
Left pars orbitalis of inferior frontal gyrus	-0.051	0.058	[-0.165 - 0.064]	3.837E-01	9.263E-01
Right pars orbitalis of inferior frontal gyrus	-0.040	0.058	[-0.154 - 0.075]	4.961E-01	9.263E-01
Left precentral gyrus	0.106	0.058	[-0.009 - 0.220]	7.035E-02	9.263E-01
Right precentral gyrus	-0.006	0.065	[-0.135 - 0.122]	9.239E-01	9.583E-01
Left lingual gyrus	-0.023	0.058	[-0.138 - 0.091]	6.930E-01	9.263E-01
Right lingual gyrus	-0.023	0.068	[-0.157 - 0.110]	7.306E-01	9.263E-01
Left superior temporal gyrus	0.064	0.081	[-0.095 - 0.224]	4.296E-01	9.263E-01
Right superior temporal gyrus	0.039	0.071	[-0.101 - 0.178]	5.878E-01	9.263E-01
Left fusiform gyrus	0.029	0.080	[-0.128 - 0.187]	7.138E-01	9.263E-01
Right fusiform gyrus	-0.071	0.064	[-0.196 - 0.054]	2.651E-01	9.263E-01
Left inferior parietal cortex	-0.009	0.058	[-0.123 - 0.106]	8.798E-01	9.583E-01
Right inferior parietal cortex	-0.050	0.058	[-0.164 - 0.065]	3.948E-01	9.263E-01
Left lateral occipital cortex	-0.005	0.099	[-0.199 - 0.188]	9.562E-01	9.583E-01
Right lateral occipital cortex	0.004	0.072	[-0.138 - 0.146]	9.555E-01	9.583E-01
Left rostral middle frontal gyrus	0.093	0.079	[-0.061 - 0.248]	2.361E-01	9.263E-01
Right rostral middle frontal gyrus	0.048	0.069	[-0.087 - 0.184]	4.863E-01	9.263E-01
Left precuneus	0.036	0.058	[-0.078 - 0.151]	5.377E-01	9.263E-01
Right precuneus	0.017	0.079	[-0.137 - 0.171]	8.280E-01	9.482E-01
Left inferior temporal gyrus	-0.021	0.076	[-0.170 - 0.128]	7.788E-01	9.447E-01
Right inferior temporal gyrus	0.062	0.101	[-0.137 - 0.261]	5.390E-01	9.263E-01
Left lateral orbitofrontal cortex	0.040	0.072	[-0.102 - 0.182]	5.781E-01	9.263E-01
Right lateral orbitofrontal cortex	-0.034	0.058	[-0.148 - 0.081]	5.652E-01	9.263E-01
Left middle temporal gyrus	0.022	0.099	[-0.173 - 0.216]	8.273E-01	9.482E-01
Right middle temporal gyrus	0.043	0.065	[-0.085 - 0.171]	5.105E-01	9.263E-01
Left postcentral gyrus	0.058	0.073	[-0.084 - 0.200]	4.233E-01	9.263E-01
Right postcentral gyrus	0.038	0.068	[-0.096 - 0.171]	5.780E-01	9.263E-01
Left medial orbitofrontal cortex	0.051	0.058	[-0.063 - 0.166]	3.805E-01	9.263E-01
Right medial orbitofrontal cortex	0.057	0.058	[-0.058 - 0.171]	3.317E-01	9.263E-01
Left cuneus	-0.074	0.070	[-0.211 - 0.062]	2.859E-01	9.263E-01
Right cuneus	0.061	0.058	[-0.053 - 0.176]	2.936E-01	9.263E-01
Left pars triangularis of inferior frontal gyrus	0.041	0.079	[-0.114 - 0.196]	6.054E-01	9.263E-01
Right pars triangularis of inferior frontal gyrus	-0.031	0.090	[-0.207 - 0.145]	7.273E-01	9.263E-01
Left superior parietal cortex	-0.007	0.069	[-0.142 - 0.128]	9.213E-01	9.583E-01
Right superior parietal cortex	-0.042	0.089	[-0.216 - 0.132]	6.380E-01	9.263E-01
Left pars opercularis of inferior frontal gyrus	0.014	0.059	[-0.101 - 0.129]	8.132E-01	9.482E-01
Right pars opercularis of inferior frontal gyrus	0.004	0.071	[-0.136 - 0.143]	9.583E-01	9.583E-01
Left supramarginal gyrus	0.046	0.058	[-0.069 - 0.160]	4.333E-01	9.263E-01
Right supramarginal gyrus	0.080	0.058	[-0.035 - 0.194]	1.711E-01	9.263E-01
Left pericalcarine cortex	-0.124	0.065	[-0.251 - 0.003]	5.587E-02	9.263E-01
Right pericalcarine cortex	-0.095	0.070	[-0.233 - 0.042]	1.746E-01	9.263E-01
Left parahippocampal gyrus	0.034	0.058	[-0.080 - 0.149]	5.567E-01	9.263E-01
Right parahippocampal gyrus	0.010	0.081	[-0.149 - 0.169]	9.040E-01	9.583E-01
Left caudal middle frontal gyrus	0.025	0.058	[-0.090 - 0.139]	6.729E-01	9.263E-01
Right caudal middle frontal gyrus	0.055	0.058	[-0.060 - 0.169]	3.466E-01	9.263E-01
Left transverse temporal gyrus	0.025	0.067	[-0.107 - 0.158]	7.086E-01	9.263E-01
Right transverse temporal gyrus	0.016	0.058	[-0.099 - 0.130]	7.850E-01	9.447E-01
Left banks of superior temporal sulcus	0.082	0.058	[-0.033 - 0.196]	1.623E-01	9.263E-01
Right banks of superior temporal sulcus	-0.066	0.058	[-0.180 - 0.049]	2.608E-01	9.263E-01
Left caudal anterior cingulate cortex	0.062	0.058	[-0.053 - 0.176]	2.917E-01	9.263E-01
Right caudal anterior cingulate cortex	0.059	0.095	[-0.128 - 0.246]	5.379E-01	9.263E-01
Left rostral anterior cingulate cortex	0.056	0.058	[-0.059 - 0.170]	3.405E-01	9.263E-01
Right rostral anterior cingulate cortex	0.039	0.111	[-0.179 - 0.257]	7.277E-01	9.263E-01
Left posterior cingulate cortex	0.034	0.083	[-0.129 - 0.196]	6.848E-01	9.263E-01
Right posterior cingulate cortex	0.041	0.088	[-0.132 - 0.214]	6.435E-01	9.263E-01
Left frontal pole	0.121	0.101	[-0.077 - 0.319]	2.307E-01	9.263E-01
Right frontal pole	0.077	0.105	[-0.129 - 0.283]	4.623E-01	9.263E-01
Left paracentral lobule	0.009	0.087	[-0.162 - 0.180]	9.139E-01	9.583E-01
Right paracentral lobule	0.077	0.066	[-0.053 - 0.207]	2.450E-01	9.263E-01
Left insula	0.011	0.076	[-0.137 - 0.160]	8.797E-01	9.583E-01
Right insula	0.028	0.071	[-0.111 - 0.168]	6.931E-01	9.263E-01
Left entorhinal cortex	-0.052	0.058	[-0.167 - 0.062]	3.724E-01	9.263E-01
Right entorhinal cortex	-0.112	0.058	[-0.226 - 0.003]	5.582E-02	9.263E-01
Left temporal pole	0.054	0.058	[-0.061 - 0.168]	3.593E-01	9.263E-01
Right temporal pole	0.030	0.058	[-0.085 - 0.144]	6.104E-01	9.263E-01
Left isthmus cingulate cortex	0.086	0.094	[-0.097 - 0.270]	3.571E-01	9.263E-01
Right isthmus cingulate cortex	-0.095	0.081	[-0.255 - 0.064]	2.421E-01	9.263E-01

Supplementary Table S206. Demographic characteristics used in the analyses of individuals with autism spectrum disorder and healthy comparison subjects controlling for IQ as an additional covariate

		Healthy Comparison Subjects							Individuals with autism spectram disorder						
					Age (range: 18-76)		IQ (range: 70-149)					Age (range: 11-55)		IQ (range: 56-137)	
Protocol name	Total *N*	*N*	Male	Female	Mean	s.d.	Mean	s.d.	*N*	Male	Female	Mean	s.d.	Mean	s.d.
Total	1,359	1,215	628	587	34.0	14.5	112.0	12.3	144	108	36	27.8	8.9	103.6	16.2
Osaka1	443	406	190	216	35.5	12.5	110.0	12.1	37	27	10	26.0	10.4	99.2	18.5
Osaka2	252	235	130	105	31.3	13.3	115.4	11.4	17	12	5	24.6	9.0	99.4	14.5
Osaka3	574	546	282	264	34.4	16.4	112.1	12.6	28	16	12	26.1	10.2	101.3	16.1
Showa1	90	28	26	2	27.9	5.0	110.2	11.2	62	53	9	30.4	6.6	108.4	14.1

Supplementary Table S207. Cortical thickness differences between individuals with autism spectrum disorder and healthy comparison subjects controlling for age, sex, and IQ

	d	Std. Err.	95% CI	*p*-value	FDR *p*-value
Global mean cortical thickness	0.055	0.115	[-0.170 - 0.280]	6.322E-01	9.831E-01
Left hemisphere	0.028	0.118	[-0.204 - 0.260]	8.132E-01	9.831E-01
Right hemisphere	0.081	0.109	[-0.133 - 0.294]	4.593E-01	9.831E-01
Left fusiform gyrus	-0.011	0.118	[-0.242 - 0.220]	9.278E-01	9.831E-01
Right fusiform gyrus	-0.215	0.192	[-0.591 - 0.162]	2.640E-01	9.831E-01
Left pars opercularis of inferior frontal gyrus	0.125	0.102	[-0.075 - 0.326]	2.207E-01	9.831E-01
Right pars opercularis of inferior frontal gyrus	0.078	0.102	[-0.123 - 0.278]	4.463E-01	9.831E-01
Left superior temporal gyrus	0.177	0.102	[-0.023 - 0.378]	8.355E-02	9.831E-01
Right superior temporal gyrus	0.161	0.102	[-0.039 - 0.362]	1.144E-01	9.831E-01
Left insula	0.040	0.192	[-0.335 - 0.416]	8.334E-01	9.831E-01
Right insula	0.053	0.139	[-0.220 - 0.325]	7.033E-01	9.831E-01
Left lingual gyrus	-0.146	0.142	[-0.424 - 0.132]	3.034E-01	9.831E-01
Right lingual gyrus	-0.112	0.116	[-0.340 - 0.116]	3.348E-01	9.831E-01
Left pars triangularis of inferior frontal gyrus	0.215	0.114	[-0.008 - 0.438]	5.867E-02	9.831E-01
Right pars triangularis of inferior frontal gyrus	0.104	0.102	[-0.096 - 0.305]	3.072E-01	9.831E-01
Left lateral orbitofrontal cortex	0.098	0.102	[-0.103 - 0.298]	3.383E-01	9.831E-01
Right lateral orbitofrontal cortex	-0.077	0.102	[-0.277 - 0.124]	4.521E-01	9.831E-01
Left rostral middle frontal gyrus	0.118	0.102	[-0.082 - 0.319]	2.471E-01	9.831E-01
Right rostral middle frontal gyrus	0.156	0.113	[-0.065 - 0.376]	1.669E-01	9.831E-01
Left middle temporal gyrus	-0.024	0.183	[-0.382 - 0.334]	8.972E-01	9.831E-01
Right middle temporal gyrus	0.024	0.136	[-0.242 - 0.290]	8.597E-01	9.831E-01
Left superior frontal gyrus	0.036	0.102	[-0.164 - 0.236]	7.248E-01	9.831E-01
Right superior frontal gyrus	0.074	0.108	[-0.137 - 0.284]	4.940E-01	9.831E-01
Left pars orbitalis of inferior frontal gyrus	-0.095	0.108	[-0.307 - 0.118]	3.819E-01	9.831E-01
Right pars orbitalis of inferior frontal gyrus	-0.004	0.117	[-0.234 - 0.226]	9.755E-01	9.895E-01
Left medial orbitofrontal cortex	0.018	0.102	[-0.182 - 0.219]	8.580E-01	9.831E-01
Right medial orbitofrontal cortex	0.149	0.102	[-0.051 - 0.350]	1.446E-01	9.831E-01
Left inferior temporal gyrus	-0.032	0.221	[-0.465 - 0.402]	8.853E-01	9.831E-01
Right inferior temporal gyrus	0.041	0.170	[-0.293 - 0.375]	8.091E-01	9.831E-01
Left isthmus cingulate cortex	0.013	0.132	[-0.245 - 0.271]	9.233E-01	9.831E-01
Right isthmus cingulate cortex	-0.068	0.102	[-0.268 - 0.133]	5.079E-01	9.831E-01
Left banks of superior temporal sulcus	0.046	0.185	[-0.316 - 0.409]	8.026E-01	9.831E-01
Right banks of superior temporal sulcus	0.042	0.205	[-0.360 - 0.443]	8.391E-01	9.831E-01
Left supramarginal gyrus	-0.164	0.156	[-0.470 - 0.141]	2.915E-01	9.831E-01
Right supramarginal gyrus	0.007	0.102	[-0.194 - 0.207]	9.492E-01	9.895E-01
Left caudal middle frontal gyrus	0.122	0.154	[-0.180 - 0.424]	4.288E-01	9.831E-01
Right caudal middle frontal gyrus	0.317	0.177	[-0.029 - 0.663]	7.223E-02	9.831E-01
Left frontal pole	0.032	0.102	[-0.168 - 0.232]	7.549E-01	9.831E-01
Right frontal pole	0.123	0.112	[-0.095 - 0.342]	2.693E-01	9.831E-01
Left posterior cingulate cortex	0.229	0.102	[0.029 - 0.430]	2.498E-02	7.133E-01
Right posterior cingulate cortex	0.004	0.102	[-0.197 - 0.204]	9.697E-01	9.895E-01
Left lateral occipital cortex	-0.105	0.102	[-0.305 - 0.096]	3.054E-01	9.831E-01
Right lateral occipital cortex	-0.028	0.102	[-0.229 - 0.172]	7.837E-01	9.831E-01
Left precentral gyrus	0.055	0.102	[-0.146 - 0.255]	5.920E-01	9.831E-01
Right precentral gyrus	0.013	0.111	[-0.205 - 0.230]	9.087E-01	9.831E-01
Left parahippocampal gyrus	0.030	0.102	[-0.171 - 0.231]	7.690E-01	9.831E-01
Right parahippocampal gyrus	0.187	0.138	[-0.084 - 0.457]	1.760E-01	9.831E-01
Left inferior parietal cortex	-0.084	0.105	[-0.290 - 0.123]	4.279E-01	9.831E-01
Right inferior parietal cortex	0.043	0.102	[-0.158 - 0.243]	6.761E-01	9.831E-01
Left transverse temporal gyrus	0.136	0.142	[-0.142 - 0.414]	3.387E-01	9.831E-01
Right transverse temporal gyrus	-0.050	0.225	[-0.492 - 0.392]	8.257E-01	9.831E-01
Left postcentral gyrus	0.016	0.162	[-0.300 - 0.333]	9.195E-01	9.831E-01
Right postcentral gyrus	-0.028	0.178	[-0.377 - 0.321]	8.762E-01	9.831E-01
Left precuneus	0.023	0.102	[-0.178 - 0.223]	8.255E-01	9.831E-01
Right precuneus	0.069	0.102	[-0.131 - 0.270]	4.992E-01	9.831E-01
Left caudal anterior cingulate cortex	-0.033	0.102	[-0.233 - 0.168]	7.494E-01	9.831E-01
Right caudal anterior cingulate cortex	0.079	0.147	[-0.208 - 0.366]	5.896E-01	9.831E-01
Left cuneus	0.180	0.173	[-0.159 - 0.520]	2.974E-01	9.831E-01
Right cuneus	0.229	0.106	[0.022 - 0.436]	3.014E-02	7.133E-01
Left rostral anterior cingulate cortex	0.016	0.103	[-0.185 - 0.217]	8.771E-01	9.831E-01
Right rostral anterior cingulate cortex	0.157	0.117	[-0.072 - 0.387]	1.783E-01	9.831E-01
Left pericalcarine cortex	-0.041	0.102	[-0.241 - 0.160]	6.918E-01	9.831E-01
Right pericalcarine cortex	-0.001	0.105	[-0.205 - 0.204]	9.957E-01	9.957E-01
Left paracentral lobule	0.078	0.102	[-0.122 - 0.279]	4.429E-01	9.831E-01
Right paracentral lobule	0.044	0.123	[-0.197 - 0.285]	7.217E-01	9.831E-01
Left superior parietal cortex	-0.086	0.102	[-0.286 - 0.115]	4.019E-01	9.831E-01
Right superior parietal cortex	0.106	0.102	[-0.094 - 0.307]	2.978E-01	9.831E-01
Left temporal pole	0.014	0.118	[-0.217 - 0.246]	9.030E-01	9.831E-01
Right temporal pole	0.240	0.102	[0.039 - 0.440]	1.922E-02	7.133E-01
Left entorhinal cortex	0.069	0.126	[-0.178 - 0.316]	5.814E-01	9.831E-01
Right entorhinal cortex	0.090	0.102	[-0.110 - 0.291]	3.772E-01	9.831E-01

Supplementary Table S208. Cortical thickness differences between individuals with autism spectrum disorder and healthy comparison subjects controlling for age, sex, global mean cortical thickness, and IQ

	d	Std. Err.	95% CI	*p*-value	FDR *p*-value
Left fusiform gyrus	-0.061	0.102	[-0.262 - 0.139]	5.499E-01	9.936E-01
Right fusiform gyrus	-0.367	0.219	[-0.795 - 0.062]	9.336E-02	5.291E-01
Left pars opercularis of inferior frontal gyrus	0.122	0.102	[-0.079 - 0.322]	2.344E-01	8.550E-01
Right pars opercularis of inferior frontal gyrus	0.062	0.102	[-0.138 - 0.263]	5.428E-01	9.936E-01
Left superior temporal gyrus	0.151	0.163	[-0.169 - 0.470]	3.553E-01	9.252E-01
Right superior temporal gyrus	0.175	0.102	[-0.025 - 0.376]	8.697E-02	5.291E-01
Left insula	0.001	0.156	[-0.305 - 0.308]	9.936E-01	9.936E-01
Right insula	0.032	0.125	[-0.213 - 0.277]	7.977E-01	9.936E-01
Left lingual gyrus	-0.231	0.102	[-0.432 - -0.030]	2.412E-02	3.491E-01
Right lingual gyrus	-0.174	0.102	[-0.375 - 0.026]	8.857E-02	5.291E-01
Left pars triangularis of inferior frontal gyrus	0.209	0.128	[-0.042 - 0.459]	1.033E-01	5.403E-01
Right pars triangularis of inferior frontal gyrus	0.086	0.102	[-0.114 - 0.286]	4.007E-01	9.252E-01
Left lateral orbitofrontal cortex	0.081	0.102	[-0.119 - 0.281]	4.285E-01	9.399E-01
Right lateral orbitofrontal cortex	-0.125	0.102	[-0.325 - 0.076]	2.227E-01	8.550E-01
Left rostral middle frontal gyrus	0.113	0.102	[-0.088 - 0.313]	2.698E-01	9.173E-01
Right rostral middle frontal gyrus	0.152	0.155	[-0.152 - 0.455]	3.278E-01	9.252E-01
Left middle temporal gyrus	-0.094	0.170	[-0.428 - 0.240]	5.793E-01	9.936E-01
Right middle temporal gyrus	-0.023	0.102	[-0.224 - 0.177]	8.190E-01	9.936E-01
Left superior frontal gyrus	0.005	0.102	[-0.195 - 0.206]	9.583E-01	9.936E-01
Right superior frontal gyrus	0.070	0.135	[-0.195 - 0.335]	6.039E-01	9.936E-01
Left pars orbitalis of inferior frontal gyrus	-0.144	0.109	[-0.357 - 0.068]	1.830E-01	8.295E-01
Right pars orbitalis of inferior frontal gyrus	-0.043	0.123	[-0.284 - 0.198]	7.261E-01	9.936E-01
Left medial orbitofrontal cortex	-0.011	0.102	[-0.211 - 0.190]	9.176E-01	9.936E-01
Right medial orbitofrontal cortex	0.127	0.107	[-0.084 - 0.337]	2.389E-01	8.550E-01
Left inferior temporal gyrus	-0.110	0.215	[-0.532 - 0.312]	6.087E-01	9.936E-01
Right inferior temporal gyrus	-0.005	0.134	[-0.267 - 0.257]	9.702E-01	9.936E-01
Left isthmus cingulate cortex	-0.011	0.123	[-0.251 - 0.230]	9.306E-01	9.936E-01
Right isthmus cingulate cortex	-0.094	0.110	[-0.310 - 0.122]	3.958E-01	9.252E-01
Left banks of superior temporal sulcus	0.005	0.204	[-0.394 - 0.405]	9.787E-01	9.936E-01
Right banks of superior temporal sulcus	0.002	0.187	[-0.364 - 0.369]	9.893E-01	9.936E-01
Left supramarginal gyrus	-0.306	0.136	[-0.573 - -0.039]	2.479E-02	3.491E-01
Right supramarginal gyrus	-0.043	0.102	[-0.243 - 0.158]	6.749E-01	9.936E-01
Left caudal middle frontal gyrus	0.103	0.135	[-0.162 - 0.368]	4.463E-01	9.485E-01
Right caudal middle frontal gyrus	0.358	0.196	[-0.027 - 0.743]	6.837E-02	5.291E-01
Left frontal pole	0.016	0.102	[-0.185 - 0.216]	8.776E-01	9.936E-01
Right frontal pole	0.103	0.102	[-0.097 - 0.304]	3.132E-01	9.252E-01
Left posterior cingulate cortex	0.228	0.102	[0.028 - 0.429]	2.567E-02	3.491E-01
Right posterior cingulate cortex	-0.027	0.102	[-0.228 - 0.173]	7.892E-01	9.936E-01
Left lateral occipital cortex	-0.179	0.102	[-0.380 - 0.021]	7.960E-02	5.291E-01
Right lateral occipital cortex	-0.089	0.102	[-0.289 - 0.112]	3.853E-01	9.252E-01
Left precentral gyrus	0.033	0.102	[-0.168 - 0.234]	7.472E-01	9.936E-01
Right precentral gyrus	-0.019	0.152	[-0.318 - 0.280]	9.015E-01	9.936E-01
Left parahippocampal gyrus	0.023	0.102	[-0.177 - 0.224]	8.203E-01	9.936E-01
Right parahippocampal gyrus	0.174	0.147	[-0.114 - 0.462]	2.375E-01	8.550E-01
Left inferior parietal cortex	-0.181	0.102	[-0.381 - 0.020]	7.704E-02	5.291E-01
Right inferior parietal cortex	-0.003	0.102	[-0.203 - 0.198]	9.784E-01	9.936E-01
Left transverse temporal gyrus	0.108	0.102	[-0.092 - 0.309]	2.905E-01	9.252E-01
Right transverse temporal gyrus	-0.092	0.210	[-0.504 - 0.320]	6.613E-01	9.936E-01
Left postcentral gyrus	-0.038	0.129	[-0.290 - 0.214]	7.667E-01	9.936E-01
Right postcentral gyrus	-0.076	0.203	[-0.474 - 0.322]	7.076E-01	9.936E-01
Left precuneus	-0.059	0.169	[-0.390 - 0.272]	7.260E-01	9.936E-01
Right precuneus	0.040	0.102	[-0.160 - 0.241]	6.939E-01	9.936E-01
Left caudal anterior cingulate cortex	-0.056	0.102	[-0.257 - 0.144]	5.812E-01	9.936E-01
Right caudal anterior cingulate cortex	0.050	0.111	[-0.168 - 0.268]	6.511E-01	9.936E-01
Left cuneus	0.177	0.176	[-0.168 - 0.523]	3.148E-01	9.252E-01
Right cuneus	0.238	0.106	[0.031 - 0.445]	2.445E-02	3.491E-01
Left rostral anterior cingulate cortex	-0.005	0.102	[-0.206 - 0.195]	9.580E-01	9.936E-01
Right rostral anterior cingulate cortex	0.141	0.102	[-0.059 - 0.342]	1.675E-01	8.135E-01
Left pericalcarine cortex	-0.052	0.102	[-0.252 - 0.149]	6.138E-01	9.936E-01
Right pericalcarine cortex	-0.011	0.102	[-0.211 - 0.190]	9.154E-01	9.936E-01
Left paracentral lobule	0.066	0.102	[-0.135 - 0.266]	5.205E-01	9.936E-01
Right paracentral lobule	0.020	0.132	[-0.239 - 0.279]	8.807E-01	9.936E-01
Left superior parietal cortex	-0.177	0.102	[-0.377 - 0.024]	8.440E-02	5.291E-01
Right superior parietal cortex	0.097	0.102	[-0.103 - 0.298]	3.427E-01	9.252E-01
Left temporal pole	-0.016	0.141	[-0.291 - 0.259]	9.094E-01	9.936E-01
Right temporal pole	0.237	0.102	[0.036 - 0.437]	2.066E-02	3.491E-01
Left entorhinal cortex	0.059	0.116	[-0.169 - 0.287]	6.142E-01	9.936E-01
Right entorhinal cortex	0.085	0.102	[-0.116 - 0.285]	4.082E-01	9.252E-01

Supplementary Table S209. Cortical surface area differences between individuals with autism spectrum disorder and healthy comparison subjects controlling for age, sex, and IQ

	d	Std. Err.	95% CI	*p*-value	FDR *p*-value
Total cortical surface area	-0.109	0.130	[-0.364 - 0.146]	4.033E-01	9.363E-01
Left hemisphere	-0.103	0.129	[-0.357 - 0.151]	4.261E-01	9.363E-01
Right hemisphere	-0.113	0.130	[-0.369 - 0.142]	3.842E-01	9.363E-01
Left superior frontal gyrus	-0.070	0.129	[-0.323 - 0.183]	5.881E-01	9.435E-01
Right superior frontal gyrus	-0.117	0.146	[-0.403 - 0.168]	4.212E-01	9.363E-01
Left pars orbitalis of inferior frontal gyrus	-0.127	0.170	[-0.460 - 0.206]	4.541E-01	9.363E-01
Right pars orbitalis of inferior frontal gyrus	-0.156	0.177	[-0.504 - 0.192]	3.785E-01	9.363E-01
Left precentral gyrus	-0.147	0.102	[-0.348 - 0.054]	1.510E-01	9.363E-01
Right precentral gyrus	-0.055	0.127	[-0.305 - 0.194]	6.645E-01	9.435E-01
Left lingual gyrus	-0.044	0.244	[-0.522 - 0.435]	8.585E-01	9.613E-01
Right lingual gyrus	-0.163	0.178	[-0.513 - 0.187]	3.611E-01	9.363E-01
Left superior temporal gyrus	-0.019	0.102	[-0.220 - 0.181]	8.498E-01	9.613E-01
Right superior temporal gyrus	-0.019	0.109	[-0.233 - 0.194]	8.582E-01	9.613E-01
Left fusiform gyrus	-0.024	0.110	[-0.239 - 0.191]	8.262E-01	9.613E-01
Right fusiform gyrus	0.118	0.170	[-0.215 - 0.451]	4.872E-01	9.435E-01
Left inferior parietal cortex	-0.081	0.102	[-0.281 - 0.120]	4.289E-01	9.363E-01
Right inferior parietal cortex	-0.175	0.102	[-0.376 - 0.026]	8.713E-02	9.363E-01
Left lateral occipital cortex	-0.044	0.102	[-0.245 - 0.156]	6.656E-01	9.435E-01
Right lateral occipital cortex	-0.020	0.175	[-0.363 - 0.322]	9.067E-01	9.613E-01
Left rostral middle frontal gyrus	-0.126	0.104	[-0.330 - 0.078]	2.261E-01	9.363E-01
Right rostral middle frontal gyrus	-0.130	0.116	[-0.356 - 0.097]	2.627E-01	9.363E-01
Left precuneus	0.071	0.128	[-0.179 - 0.321]	5.773E-01	9.435E-01
Right precuneus	-0.099	0.107	[-0.308 - 0.109]	3.510E-01	9.363E-01
Left inferior temporal gyrus	-0.012	0.158	[-0.322 - 0.298]	9.396E-01	9.613E-01
Right inferior temporal gyrus	0.025	0.102	[-0.175 - 0.226]	8.050E-01	9.613E-01
Left lateral orbitofrontal cortex	-0.137	0.102	[-0.337 - 0.064]	1.821E-01	9.363E-01
Right lateral orbitofrontal cortex	-0.098	0.115	[-0.323 - 0.127]	3.918E-01	9.363E-01
Left middle temporal gyrus	-0.131	0.102	[-0.331 - 0.070]	2.012E-01	9.363E-01
Right middle temporal gyrus	-0.199	0.102	[-0.400 - 0.001]	5.167E-02	9.363E-01
Left postcentral gyrus	-0.162	0.102	[-0.362 - 0.039]	1.141E-01	9.363E-01
Right postcentral gyrus	-0.132	0.169	[-0.463 - 0.199]	4.352E-01	9.363E-01
Left medial orbitofrontal cortex	-0.085	0.102	[-0.286 - 0.115]	4.043E-01	9.363E-01
Right medial orbitofrontal cortex	-0.096	0.102	[-0.297 - 0.104]	3.455E-01	9.363E-01
Left cuneus	0.021	0.152	[-0.276 - 0.318]	8.912E-01	9.613E-01
Right cuneus	-0.051	0.122	[-0.290 - 0.188]	6.778E-01	9.435E-01
Left pars triangularis of inferior frontal gyrus	0.038	0.142	[-0.241 - 0.316]	7.917E-01	9.613E-01
Right pars triangularis of inferior frontal gyrus	0.143	0.186	[-0.222 - 0.507]	4.430E-01	9.363E-01
Left superior parietal cortex	-0.070	0.161	[-0.386 - 0.246]	6.636E-01	9.435E-01
Right superior parietal cortex	-0.121	0.102	[-0.322 - 0.079]	2.352E-01	9.363E-01
Left pars opercularis of inferior frontal gyrus	-0.084	0.102	[-0.284 - 0.117]	4.135E-01	9.363E-01
Right pars opercularis of inferior frontal gyrus	-0.111	0.102	[-0.312 - 0.089]	2.759E-01	9.363E-01
Left supramarginal gyrus	-0.058	0.111	[-0.276 - 0.160]	6.010E-01	9.435E-01
Right supramarginal gyrus	-0.060	0.126	[-0.306 - 0.186]	6.333E-01	9.435E-01
Left pericalcarine cortex	-0.008	0.203	[-0.406 - 0.389]	9.668E-01	9.668E-01
Right pericalcarine cortex	-0.083	0.195	[-0.464 - 0.299]	6.714E-01	9.435E-01
Left parahippocampal gyrus	-0.189	0.102	[-0.390 - 0.012]	6.536E-02	9.363E-01
Right parahippocampal gyrus	-0.124	0.186	[-0.488 - 0.240]	5.034E-01	9.435E-01
Left caudal middle frontal gyrus	-0.176	0.102	[-0.376 - 0.025]	8.621E-02	9.363E-01
Right caudal middle frontal gyrus	-0.057	0.102	[-0.258 - 0.143]	5.765E-01	9.435E-01
Left transverse temporal gyrus	0.016	0.102	[-0.185 - 0.216]	8.792E-01	9.613E-01
Right transverse temporal gyrus	-0.075	0.102	[-0.276 - 0.125]	4.616E-01	9.363E-01
Left banks of superior temporal sulcus	-0.007	0.102	[-0.208 - 0.193]	9.422E-01	9.613E-01
Right banks of superior temporal sulcus	-0.177	0.102	[-0.378 - 0.023]	8.296E-02	9.363E-01
Left caudal anterior cingulate cortex	0.021	0.118	[-0.210 - 0.252]	8.591E-01	9.613E-01
Right caudal anterior cingulate cortex	-0.050	0.134	[-0.313 - 0.212]	7.071E-01	9.473E-01
Left rostral anterior cingulate cortex	-0.031	0.120	[-0.267 - 0.204]	7.936E-01	9.613E-01
Right rostral anterior cingulate cortex	-0.062	0.102	[-0.263 - 0.138]	5.435E-01	9.435E-01
Left posterior cingulate cortex	-0.120	0.102	[-0.320 - 0.081]	2.422E-01	9.363E-01
Right posterior cingulate cortex	0.059	0.152	[-0.240 - 0.357]	7.008E-01	9.473E-01
Left frontal pole	0.024	0.102	[-0.177 - 0.224]	8.175E-01	9.613E-01
Right frontal pole	0.009	0.137	[-0.259 - 0.277]	9.477E-01	9.613E-01
Left paracentral lobule	-0.115	0.102	[-0.315 - 0.086]	2.615E-01	9.363E-01
Right paracentral lobule	0.010	0.102	[-0.190 - 0.210]	9.226E-01	9.613E-01
Left insula	-0.096	0.102	[-0.296 - 0.105]	3.496E-01	9.363E-01
Right insula	-0.094	0.102	[-0.294 - 0.107]	3.595E-01	9.363E-01
Left entorhinal cortex	-0.184	0.102	[-0.384 - 0.017]	7.235E-02	9.363E-01
Right entorhinal cortex	0.050	0.102	[-0.151 - 0.250]	6.258E-01	9.435E-01
Left temporal pole	0.020	0.110	[-0.195 - 0.234]	8.583E-01	9.613E-01
Right temporal pole	-0.050	0.102	[-0.251 - 0.150]	6.214E-01	9.435E-01
Left isthmus cingulate cortex	-0.125	0.111	[-0.343 - 0.092]	2.581E-01	9.363E-01
Right isthmus cingulate cortex	0.058	0.133	[-0.202 - 0.319]	6.602E-01	9.435E-01

Supplementary Table S210. Cortical surface area differences between individuals with autism spectrum disorder and healthy comparison subjects controlling for age, sex, total cortical surface area, and IQ

	d	Std. Err.	95% CI	*p*-value	FDR *p*-value
Left superior frontal gyrus	0.029	0.139	[-0.244 - 0.302]	8.362E-01	9.847E-01
Right superior frontal gyrus	-0.059	0.151	[-0.355 - 0.237]	6.961E-01	9.847E-01
Left pars orbitalis of inferior frontal gyrus	-0.082	0.169	[-0.413 - 0.248]	6.264E-01	9.847E-01
Right pars orbitalis of inferior frontal gyrus	-0.118	0.175	[-0.461 - 0.225]	5.013E-01	9.847E-01
Left precentral gyrus	-0.069	0.102	[-0.270 - 0.131]	4.991E-01	9.847E-01
Right precentral gyrus	0.011	0.102	[-0.190 - 0.211]	9.170E-01	9.847E-01
Left lingual gyrus	0.013	0.227	[-0.432 - 0.457]	9.558E-01	9.847E-01
Right lingual gyrus	-0.130	0.181	[-0.485 - 0.225]	4.731E-01	9.847E-01
Left superior temporal gyrus	0.103	0.102	[-0.097 - 0.304]	3.127E-01	9.847E-01
Right superior temporal gyrus	0.090	0.206	[-0.314 - 0.494]	6.632E-01	9.847E-01
Left fusiform gyrus	0.077	0.102	[-0.123 - 0.278]	4.503E-01	9.847E-01
Right fusiform gyrus	0.222	0.117	[-0.008 - 0.451]	5.822E-02	9.847E-01
Left inferior parietal cortex	0.016	0.102	[-0.184 - 0.216]	8.755E-01	9.847E-01
Right inferior parietal cortex	-0.123	0.102	[-0.324 - 0.077]	2.276E-01	9.847E-01
Left lateral occipital cortex	0.016	0.134	[-0.246 - 0.278]	9.043E-01	9.847E-01
Right lateral occipital cortex	0.050	0.139	[-0.221 - 0.322]	7.167E-01	9.847E-01
Left rostral middle frontal gyrus	-0.046	0.174	[-0.387 - 0.295]	7.933E-01	9.847E-01
Right rostral middle frontal gyrus	-0.060	0.102	[-0.261 - 0.140]	5.555E-01	9.847E-01
Left precuneus	0.223	0.102	[0.022 - 0.423]	2.946E-02	9.847E-01
Right precuneus	-0.012	0.102	[-0.213 - 0.188]	9.034E-01	9.847E-01
Left inferior temporal gyrus	0.083	0.180	[-0.271 - 0.436]	6.468E-01	9.847E-01
Right inferior temporal gyrus	0.151	0.125	[-0.093 - 0.396]	2.250E-01	9.847E-01
Left lateral orbitofrontal cortex	-0.049	0.112	[-0.268 - 0.170]	6.592E-01	9.847E-01
Right lateral orbitofrontal cortex	-0.002	0.177	[-0.348 - 0.344]	9.909E-01	9.909E-01
Left middle temporal gyrus	-0.055	0.102	[-0.256 - 0.145]	5.883E-01	9.847E-01
Right middle temporal gyrus	-0.173	0.189	[-0.542 - 0.197]	3.599E-01	9.847E-01
Left postcentral gyrus	-0.095	0.109	[-0.309 - 0.119]	3.835E-01	9.847E-01
Right postcentral gyrus	-0.112	0.106	[-0.321 - 0.096]	2.922E-01	9.847E-01
Left medial orbitofrontal cortex	-0.010	0.102	[-0.210 - 0.191]	9.228E-01	9.847E-01
Right medial orbitofrontal cortex	-0.007	0.102	[-0.207 - 0.194]	9.487E-01	9.847E-01
Left cuneus	0.079	0.138	[-0.191 - 0.350]	5.660E-01	9.847E-01
Right cuneus	0.014	0.102	[-0.187 - 0.214]	8.940E-01	9.847E-01
Left pars triangularis of inferior frontal gyrus	0.080	0.102	[-0.121 - 0.280]	4.365E-01	9.847E-01
Right pars triangularis of inferior frontal gyrus	0.210	0.175	[-0.133 - 0.553]	2.308E-01	9.847E-01
Left superior parietal cortex	-0.021	0.102	[-0.222 - 0.179]	8.362E-01	9.847E-01
Right superior parietal cortex	-0.047	0.102	[-0.247 - 0.154]	6.468E-01	9.847E-01
Left pars opercularis of inferior frontal gyrus	-0.027	0.102	[-0.228 - 0.173]	7.901E-01	9.847E-01
Right pars opercularis of inferior frontal gyrus	-0.067	0.122	[-0.306 - 0.172]	5.810E-01	9.847E-01
Left supramarginal gyrus	0.030	0.102	[-0.170 - 0.230]	7.692E-01	9.847E-01
Right supramarginal gyrus	0.006	0.108	[-0.205 - 0.218]	9.533E-01	9.847E-01
Left pericalcarine cortex	0.047	0.196	[-0.337 - 0.431]	8.117E-01	9.847E-01
Right pericalcarine cortex	-0.042	0.183	[-0.400 - 0.316]	8.195E-01	9.847E-01
Left parahippocampal gyrus	-0.131	0.110	[-0.347 - 0.085]	2.355E-01	9.847E-01
Right parahippocampal gyrus	-0.092	0.191	[-0.466 - 0.282]	6.287E-01	9.847E-01
Left caudal middle frontal gyrus	-0.133	0.102	[-0.333 - 0.068]	1.941E-01	9.847E-01
Right caudal middle frontal gyrus	0.008	0.102	[-0.193 - 0.208]	9.385E-01	9.847E-01
Left transverse temporal gyrus	0.092	0.102	[-0.108 - 0.293]	3.667E-01	9.847E-01
Right transverse temporal gyrus	0.006	0.116	[-0.220 - 0.233]	9.554E-01	9.847E-01
Left banks of superior temporal sulcus	0.064	0.102	[-0.136 - 0.265]	5.296E-01	9.847E-01
Right banks of superior temporal sulcus	-0.151	0.135	[-0.415 - 0.113]	2.635E-01	9.847E-01
Left caudal anterior cingulate cortex	0.061	0.102	[-0.140 - 0.261]	5.532E-01	9.847E-01
Right caudal anterior cingulate cortex	-0.021	0.158	[-0.330 - 0.288]	8.935E-01	9.847E-01
Left rostral anterior cingulate cortex	0.044	0.102	[-0.157 - 0.244]	6.695E-01	9.847E-01
Right rostral anterior cingulate cortex	-0.006	0.102	[-0.207 - 0.194]	9.506E-01	9.847E-01
Left posterior cingulate cortex	-0.059	0.102	[-0.259 - 0.142]	5.655E-01	9.847E-01
Right posterior cingulate cortex	0.141	0.149	[-0.152 - 0.433]	3.456E-01	9.847E-01
Left frontal pole	0.069	0.102	[-0.132 - 0.269]	5.010E-01	9.847E-01
Right frontal pole	0.036	0.113	[-0.185 - 0.257]	7.485E-01	9.847E-01
Left paracentral lobule	-0.057	0.102	[-0.258 - 0.143]	5.762E-01	9.847E-01
Right paracentral lobule	0.080	0.105	[-0.126 - 0.287]	4.466E-01	9.847E-01
Left insula	0.001	0.102	[-0.199 - 0.202]	9.906E-01	9.909E-01
Right insula	-0.019	0.102	[-0.219 - 0.181]	8.533E-01	9.847E-01
Left entorhinal cortex	-0.141	0.102	[-0.342 - 0.059]	1.678E-01	9.847E-01
Right entorhinal cortex	0.093	0.102	[-0.108 - 0.293]	3.639E-01	9.847E-01
Left temporal pole	0.078	0.102	[-0.122 - 0.279]	4.434E-01	9.847E-01
Right temporal pole	-0.013	0.102	[-0.214 - 0.187]	8.968E-01	9.847E-01
Left isthmus cingulate cortex	-0.082	0.118	[-0.314 - 0.149]	4.864E-01	9.847E-01
Right isthmus cingulate cortex	0.137	0.102	[-0.063 - 0.338]	1.793E-01	9.847E-01

Supplementary Table S211. Cortical volume differences between individuals with autism spectrum disorder and healthy comparison subjects controlling for age, sex, and IQ

	d	Std. Err.	95% CI	*p*-value	FDR *p*-value
Total cortical volume	-0.091	0.139	[-0.363 - 0.181]	5.116E-01	9.065E-01
Left hemisphere	-0.100	0.134	[-0.363 - 0.162]	4.537E-01	9.065E-01
Right hemisphere	-0.080	0.143	[-0.361 - 0.200]	5.739E-01	9.065E-01
Left superior frontal gyrus	-0.054	0.126	[-0.300 - 0.193]	6.689E-01	9.065E-01
Right superior frontal gyrus	-0.094	0.162	[-0.410 - 0.223]	5.627E-01	9.065E-01
Left lateral orbitofrontal cortex	-0.092	0.102	[-0.293 - 0.108]	3.680E-01	9.065E-01
Right lateral orbitofrontal cortex	-0.147	0.109	[-0.361 - 0.068]	1.801E-01	9.065E-01
Left pars orbitalis of inferior frontal gyrus	-0.171	0.128	[-0.422 - 0.080]	1.821E-01	9.065E-01
Right pars orbitalis of inferior frontal gyrus	-0.154	0.161	[-0.471 - 0.162]	3.391E-01	9.065E-01
Left rostral middle frontal gyrus	-0.057	0.147	[-0.346 - 0.231]	6.974E-01	9.065E-01
Right rostral middle frontal gyrus	-0.062	0.136	[-0.328 - 0.205]	6.508E-01	9.065E-01
Left superior temporal gyrus	0.057	0.102	[-0.143 - 0.257]	5.768E-01	9.065E-01
Right superior temporal gyrus	0.055	0.102	[-0.145 - 0.256]	5.886E-01	9.065E-01
Left lingual gyrus	-0.105	0.251	[-0.597 - 0.386]	6.744E-01	9.065E-01
Right lingual gyrus	-0.207	0.144	[-0.490 - 0.075]	1.498E-01	9.065E-01
Left fusiform gyrus	-0.042	0.102	[-0.243 - 0.158]	6.781E-01	9.065E-01
Right fusiform gyrus	0.021	0.131	[-0.236 - 0.279]	8.721E-01	9.805E-01
Left medial orbitofrontal cortex	-0.089	0.102	[-0.290 - 0.111]	3.834E-01	9.065E-01
Right medial orbitofrontal cortex	-0.008	0.102	[-0.208 - 0.193]	9.404E-01	9.833E-01
Left inferior temporal gyrus	-0.014	0.166	[-0.340 - 0.312]	9.310E-01	9.833E-01
Right inferior temporal gyrus	0.055	0.118	[-0.177 - 0.286]	6.420E-01	9.065E-01
Left precentral gyrus	-0.116	0.102	[-0.317 - 0.084]	2.551E-01	9.065E-01
Right precentral gyrus	-0.034	0.148	[-0.324 - 0.257]	8.201E-01	9.424E-01
Left middle temporal gyrus	-0.137	0.102	[-0.337 - 0.064]	1.809E-01	9.065E-01
Right middle temporal gyrus	-0.177	0.102	[-0.378 - 0.023]	8.323E-02	9.065E-01
Left pars triangularis of inferior frontal gyrus	0.088	0.107	[-0.121 - 0.297]	4.103E-01	9.065E-01
Right pars triangularis of inferior frontal gyrus	0.190	0.206	[-0.214 - 0.593]	3.574E-01	9.065E-01
Left insula	-0.064	0.126	[-0.311 - 0.183]	6.133E-01	9.065E-01
Right insula	-0.093	0.102	[-0.293 - 0.108]	3.662E-01	9.065E-01
Left pars opercularis of inferior frontal gyrus	-0.053	0.102	[-0.254 - 0.147]	6.016E-01	9.065E-01
Right pars opercularis of inferior frontal gyrus	-0.098	0.102	[-0.299 - 0.102]	3.378E-01	9.065E-01
Left lateral occipital cortex	-0.097	0.102	[-0.297 - 0.104]	3.452E-01	9.065E-01
Right lateral occipital cortex	-0.039	0.168	[-0.367 - 0.290]	8.171E-01	9.424E-01
Left parahippocampal gyrus	-0.192	0.102	[-0.392 - 0.009]	6.123E-02	9.065E-01
Right parahippocampal gyrus	-0.006	0.117	[-0.235 - 0.223]	9.602E-01	9.833E-01
Left inferior parietal cortex	-0.121	0.102	[-0.321 - 0.080]	2.377E-01	9.065E-01
Right inferior parietal cortex	-0.167	0.102	[-0.367 - 0.034]	1.036E-01	9.065E-01
Left frontal pole	0.040	0.102	[-0.160 - 0.240]	6.953E-01	9.065E-01
Right frontal pole	0.065	0.133	[-0.196 - 0.325]	6.256E-01	9.065E-01
Left supramarginal gyrus	-0.140	0.102	[-0.340 - 0.061]	1.725E-01	9.065E-01
Right supramarginal gyrus	-0.059	0.131	[-0.317 - 0.198]	6.511E-01	9.065E-01
Left transverse temporal gyrus	0.065	0.102	[-0.135 - 0.265]	5.252E-01	9.065E-01
Right transverse temporal gyrus	-0.118	0.146	[-0.405 - 0.169]	4.187E-01	9.065E-01
Left precuneus	0.080	0.137	[-0.188 - 0.348]	5.577E-01	9.065E-01
Right precuneus	-0.058	0.137	[-0.328 - 0.211]	6.703E-01	9.065E-01
Left caudal middle frontal gyrus	-0.151	0.102	[-0.351 - 0.050]	1.411E-01	9.065E-01
Right caudal middle frontal gyrus	0.026	0.102	[-0.174 - 0.227]	7.963E-01	9.424E-01
Left banks of superior temporal sulcus	0.003	0.102	[-0.198 - 0.203]	9.802E-01	9.833E-01
Right banks of superior temporal sulcus	-0.165	0.102	[-0.366 - 0.035]	1.061E-01	9.065E-01
Left posterior cingulate cortex	-0.039	0.102	[-0.240 - 0.161]	7.022E-01	9.065E-01
Right posterior cingulate cortex	0.081	0.180	[-0.271 - 0.433]	6.529E-01	9.065E-01
Left postcentral gyrus	-0.119	0.157	[-0.426 - 0.189]	4.492E-01	9.065E-01
Right postcentral gyrus	-0.112	0.206	[-0.516 - 0.292]	5.866E-01	9.065E-01
Left cuneus	0.111	0.217	[-0.314 - 0.537]	6.081E-01	9.065E-01
Right cuneus	0.084	0.165	[-0.239 - 0.407]	6.107E-01	9.065E-01
Left caudal anterior cingulate cortex	0.004	0.102	[-0.196 - 0.205]	9.678E-01	9.833E-01
Right caudal anterior cingulate cortex	-0.019	0.179	[-0.370 - 0.333]	9.179E-01	9.833E-01
Left rostral anterior cingulate cortex	-0.039	0.111	[-0.255 - 0.178]	7.270E-01	9.217E-01
Right rostral anterior cingulate cortex	-0.002	0.102	[-0.203 - 0.198]	9.833E-01	9.833E-01
Left superior parietal cortex	-0.104	0.153	[-0.405 - 0.196]	4.966E-01	9.065E-01
Right superior parietal cortex	-0.076	0.102	[-0.277 - 0.124]	4.557E-01	9.065E-01
Left isthmus cingulate cortex	-0.113	0.167	[-0.440 - 0.214]	4.976E-01	9.065E-01
Right isthmus cingulate cortex	0.035	0.155	[-0.268 - 0.338]	8.207E-01	9.424E-01
Left pericalcarine cortex	-0.025	0.169	[-0.356 - 0.306]	8.839E-01	9.805E-01
Right pericalcarine cortex	-0.069	0.180	[-0.421 - 0.283]	7.008E-01	9.065E-01
Left paracentral lobule	-0.062	0.102	[-0.262 - 0.139]	5.451E-01	9.065E-01
Right paracentral lobule	0.023	0.102	[-0.178 - 0.223]	8.229E-01	9.424E-01
Left entorhinal cortex	-0.149	0.102	[-0.350 - 0.051]	1.439E-01	9.065E-01
Right entorhinal cortex	0.109	0.102	[-0.091 - 0.309]	2.866E-01	9.065E-01
Left temporal pole	0.026	0.102	[-0.175 - 0.226]	8.009E-01	9.424E-01
Right temporal pole	0.099	0.102	[-0.101 - 0.300]	3.307E-01	9.065E-01

Supplementary Table S212. Cortical volume differences between individuals with autism spectrum disorder and healthy comparison subjects controlling for age, sex, ICV, and IQ

	d	Std. Err.	95% CI	*p*-value	FDR *p*-value
Left superior frontal gyrus	-0.054	0.103	[-0.255 - 0.147]	5.996E-01	8.698E-01
Right superior frontal gyrus	-0.108	0.149	[-0.400 - 0.184]	4.684E-01	8.698E-01
Left lateral orbitofrontal cortex	-0.073	0.102	[-0.274 - 0.127]	4.735E-01	8.698E-01
Right lateral orbitofrontal cortex	-0.147	0.102	[-0.348 - 0.053]	1.506E-01	8.698E-01
Left pars orbitalis of inferior frontal gyrus	-0.190	0.102	[-0.390 - 0.011]	6.403E-02	8.698E-01
Right pars orbitalis of inferior frontal gyrus	-0.160	0.147	[-0.449 - 0.128]	2.757E-01	8.698E-01
Left rostral middle frontal gyrus	-0.080	0.117	[-0.309 - 0.148]	4.910E-01	8.698E-01
Right rostral middle frontal gyrus	-0.073	0.122	[-0.311 - 0.165]	5.494E-01	8.698E-01
Left superior temporal gyrus	0.092	0.102	[-0.108 - 0.293]	3.677E-01	8.698E-01
Right superior temporal gyrus	0.092	0.117	[-0.138 - 0.321]	4.329E-01	8.698E-01
Left lingual gyrus	-0.112	0.236	[-0.575 - 0.350]	6.334E-01	8.698E-01
Right lingual gyrus	-0.219	0.137	[-0.488 - 0.051]	1.116E-01	8.698E-01
Left fusiform gyrus	-0.031	0.102	[-0.232 - 0.169]	7.595E-01	9.061E-01
Right fusiform gyrus	0.021	0.102	[-0.180 - 0.221]	8.385E-01	9.503E-01
Left medial orbitofrontal cortex	-0.090	0.102	[-0.291 - 0.111]	3.789E-01	8.698E-01
Right medial orbitofrontal cortex	0.018	0.102	[-0.183 - 0.218]	8.612E-01	9.559E-01
Left inferior temporal gyrus	-0.005	0.162	[-0.323 - 0.314]	9.774E-01	9.933E-01
Right inferior temporal gyrus	0.079	0.116	[-0.148 - 0.306]	4.926E-01	8.698E-01
Left precentral gyrus	-0.109	0.102	[-0.309 - 0.092]	2.882E-01	8.698E-01
Right precentral gyrus	-0.055	0.102	[-0.256 - 0.145]	5.887E-01	8.698E-01
Left middle temporal gyrus	-0.134	0.102	[-0.334 - 0.067]	1.912E-01	8.698E-01
Right middle temporal gyrus	-0.193	0.112	[-0.412 - 0.026]	8.375E-02	8.698E-01
Left pars triangularis of inferior frontal gyrus	0.109	0.102	[-0.091 - 0.310]	2.858E-01	8.698E-01
Right pars triangularis of inferior frontal gyrus	0.198	0.185	[-0.164 - 0.561]	2.839E-01	8.698E-01
Left insula	-0.078	0.102	[-0.278 - 0.123]	4.473E-01	8.698E-01
Right insula	-0.092	0.102	[-0.292 - 0.109]	3.690E-01	8.698E-01
Left pars opercularis of inferior frontal gyrus	-0.042	0.102	[-0.242 - 0.159]	6.822E-01	8.698E-01
Right pars opercularis of inferior frontal gyrus	-0.087	0.102	[-0.287 - 0.114]	3.959E-01	8.698E-01
Left lateral occipital cortex	-0.105	0.110	[-0.320 - 0.110]	3.388E-01	8.698E-01
Right lateral occipital cortex	-0.057	0.152	[-0.355 - 0.242]	7.088E-01	8.698E-01
Left parahippocampal gyrus	-0.189	0.102	[-0.390 - 0.012]	6.484E-02	8.698E-01
Right parahippocampal gyrus	-0.002	0.132	[-0.260 - 0.257]	9.897E-01	9.933E-01
Left inferior parietal cortex	-0.115	0.102	[-0.316 - 0.085]	2.595E-01	8.698E-01
Right inferior parietal cortex	-0.182	0.102	[-0.383 - 0.019]	7.524E-02	8.698E-01
Left frontal pole	0.043	0.102	[-0.158 - 0.243]	6.751E-01	8.698E-01
Right frontal pole	0.057	0.114	[-0.166 - 0.280]	6.165E-01	8.698E-01
Left supramarginal gyrus	-0.138	0.102	[-0.339 - 0.062]	1.757E-01	8.698E-01
Right supramarginal gyrus	-0.068	0.102	[-0.268 - 0.133]	5.076E-01	8.698E-01
Left transverse temporal gyrus	0.093	0.102	[-0.108 - 0.293]	3.639E-01	8.698E-01
Right transverse temporal gyrus	-0.130	0.115	[-0.355 - 0.096]	2.595E-01	8.698E-01
Left precuneus	0.092	0.102	[-0.108 - 0.293]	3.675E-01	8.698E-01
Right precuneus	-0.083	0.102	[-0.284 - 0.117]	4.146E-01	8.698E-01
Left caudal middle frontal gyrus	-0.165	0.102	[-0.366 - 0.035]	1.066E-01	8.698E-01
Right caudal middle frontal gyrus	0.043	0.102	[-0.157 - 0.244]	6.725E-01	8.698E-01
Left banks of superior temporal sulcus	0.015	0.102	[-0.186 - 0.215]	8.856E-01	9.559E-01
Right banks of superior temporal sulcus	-0.174	0.102	[-0.375 - 0.026]	8.868E-02	8.698E-01
Left posterior cingulate cortex	-0.025	0.102	[-0.225 - 0.176]	8.094E-01	9.351E-01
Right posterior cingulate cortex	0.086	0.155	[-0.217 - 0.389]	5.786E-01	8.698E-01
Left postcentral gyrus	-0.136	0.122	[-0.376 - 0.103]	2.657E-01	8.698E-01
Right postcentral gyrus	-0.149	0.177	[-0.495 - 0.197]	3.994E-01	8.698E-01
Left cuneus	0.101	0.210	[-0.311 - 0.513]	6.311E-01	8.698E-01
Right cuneus	0.072	0.138	[-0.198 - 0.341]	6.010E-01	8.698E-01
Left caudal anterior cingulate cortex	0.003	0.102	[-0.197 - 0.204]	9.727E-01	9.933E-01
Right caudal anterior cingulate cortex	-0.026	0.175	[-0.370 - 0.317]	8.806E-01	9.559E-01
Left rostral anterior cingulate cortex	-0.037	0.102	[-0.238 - 0.163]	7.163E-01	8.698E-01
Right rostral anterior cingulate cortex	0.001	0.102	[-0.200 - 0.201]	9.933E-01	9.933E-01
Left superior parietal cortex	-0.135	0.114	[-0.358 - 0.089]	2.376E-01	8.698E-01
Right superior parietal cortex	-0.067	0.102	[-0.268 - 0.133]	5.121E-01	8.698E-01
Left isthmus cingulate cortex	-0.156	0.187	[-0.522 - 0.210]	4.042E-01	8.698E-01
Right isthmus cingulate cortex	0.035	0.146	[-0.251 - 0.320]	8.114E-01	9.351E-01
Left pericalcarine cortex	-0.017	0.164	[-0.338 - 0.304]	9.170E-01	9.743E-01
Right pericalcarine cortex	-0.074	0.172	[-0.410 - 0.263]	6.681E-01	8.698E-01
Left paracentral lobule	-0.050	0.102	[-0.251 - 0.150]	6.232E-01	8.698E-01
Right paracentral lobule	0.046	0.102	[-0.154 - 0.247]	6.510E-01	8.698E-01
Left entorhinal cortex	-0.143	0.102	[-0.343 - 0.058]	1.627E-01	8.698E-01
Right entorhinal cortex	0.124	0.102	[-0.077 - 0.324]	2.257E-01	8.698E-01
Left temporal pole	0.039	0.102	[-0.161 - 0.240]	7.013E-01	8.698E-01
Right temporal pole	0.109	0.102	[-0.091 - 0.309]	2.864E-01	8.698E-01
